# Comparative Genomics Reveals the Core Gene Toolbox for the Fungus-Insect Symbiosis

**DOI:** 10.1128/mBio.00636-18

**Published:** 2018-05-15

**Authors:** Yan Wang, Matt Stata, Wei Wang, Jason E. Stajich, Merlin M. White, Jean-Marc Moncalvo

**Affiliations:** a Department of Ecology and Evolutionary Biology, University of Toronto, Toronto, Ontario, Canada; b Department of Natural History, Royal Ontario Museum, Toronto, Ontario, Canada; c Department of Microbiology and Plant Pathology, University of California, Riverside, Riverside, California, USA; d Institute for Integrative Genome Biology, University of California, Riverside, Riverside, California, USA; e Department of Biological Sciences, Boise State University, Boise, Idaho, USA; University of California, Berkeley

**Keywords:** FISCoG, phylogenomics, Trichomycetes, Zoopagomycota, Zygomycota

## Abstract

Modern genomics has shed light on many entomopathogenic fungi and expanded our knowledge widely; however, little is known about the genomic features of the insect-commensal fungi. Harpellales are obligate commensals living in the digestive tracts of disease-bearing insects (black flies, midges, and mosquitoes). In this study, we produced and annotated whole-genome sequences of nine Harpellales taxa and conducted the first comparative analyses to infer the genomic diversity within the members of the Harpellales. The genomes of the insect gut fungi feature low (26% to 37%) GC content and large genome size variations (25 to 102 Mb). Further comparisons with insect-pathogenic fungi (from both Ascomycota and Zoopagomycota), as well as with free-living relatives (as negative controls), helped to identify a gene toolbox that is essential to the fungus-insect symbiosis. The results not only narrow the genomic scope of fungus-insect interactions from several thousands to eight core players but also distinguish host invasion strategies employed by insect pathogens and commensals. The genomic content suggests that insect commensal fungi rely mostly on adhesion protein anchors that target digestive system, while entomopathogenic fungi have higher numbers of transmembrane helices, signal peptides, and pathogen-host interaction (PHI) genes across the whole genome and enrich genes as well as functional domains to inactivate the host inflammation system and suppress the host defense. Phylogenomic analyses have revealed that genome sizes of Harpellales fungi vary among lineages with an integer-multiple pattern, which implies that ancient genome duplications may have occurred within the gut of insects.

## IMPORTANCE

Insect guts harbor various microbes that are important for host digestion, immune response, and disease dispersal in certain cases. Bacteria, which are among the primary endosymbionts, have been studied extensively. However, fungi, which are also frequently encountered, are poorly known with respect to their biology within the insect guts. To understand the genomic features and related biology, we produced the whole-genome sequences of nine gut commensal fungi from disease-bearing insects (black flies, midges, and mosquitoes). The results show that insect gut fungi tend to have low GC content across their genomes. By comparing these commensals with entomopathogenic and free-living fungi that have available genome sequences, we found a universal core gene toolbox that is unique and thus potentially important for the insect-fungus symbiosis. This comparative work also uncovered different host invasion strategies employed by insect pathogens and commensals, as well as a model system to study ancient fungal genome duplication within the gut of insects.

## INTRODUCTION

Many microfungi have obligate symbiotic relationships with other eukaryotes (1–3). Insects, the most species-rich group of animals, are often associated with fungal symbionts (4, 5). Fungus-insect relationships have mostly been documented and studied for the disease-bearing behavior of the insects and effects of transmission to humans and livestock (6, 7). Several fungi are well known to cause insect disease or death (8–10), although there is a suite of complex interactions that range from parasitism to commensalism and mutualism, according to the results of feedback with their hosts (11).

Various symbiotic interactions between insects and fungi have been recorded. For example, Attine ant farm cultivars of fungi (2) and aphids acquire fungal genes to make their own carotenoids (12). On the other hand, fungi are able to consume insects and even control their behaviors by forming peripheral networks encircling the host muscle and using detailed mechanisms to secure their own dispersal success, including examples such as *Entomophthora muscae* found in house flies (13) and Ophiocordyceps unilateralis in arboreal ants (14, 15). Some fungi kill pest insects such as Beauveria bassiana (16), *Metarhizium* spp. (17), and *Smittium morbosum* (18), and in some cases they have inspired the development of multiple biocontrol pesticides that use the fungal products (3, 19, 20). Furthermore, complex interactions between fungi and insects, in the form of bioactive by-products, have long been considered for medicinal usages, such as Ophiocordyceps sinensis (synonym Cordyceps sinensis) (21, 22) and Cordyceps militaris (10).

Recent advances in next-generation sequencing (NGS) and bioinformatics techniques are enabling detailed examinations and searches for genetic elements that are responsible for fungus-insect association in a novel and direct way (23–25). de Bekker et al. have shown that the genetic tool used by O. unilateralis to manipulate the behavior of ant hosts is a compound made of ergot, alkaloids, polyketides, and nonribosomal peptides, which have effects on central nervous systems (26). Xiao et al. found that B. bassiana has active gene sets that work as sensors to detect various environments and host types (27). Comparisons of two closely related *Metarhizium* fungi, the locust-specific pathogen M. acridum and the generalist M. robertsii (synonym M. anisopliae) (28), revealed that both produce strikingly larger proportions of secreted proteins than other fungi but that the genome of M. robertsii includes expanded families of genes encoding proteases, chitinase, polyketide synthases, and nonribosomal peptide synthetases, in response to its wider need for cuticle degradation, detoxification, and toxin biosynthesis for various host types (29). Most of the available whole-genome sequences of insect-associated fungi are from pathogens (27, 29–31). However, to obtain a broader and fundamental understanding of the biology of insect-fungus symbioses, it is important to include fungal commensals as well. By comparing the genome sequences of fungal pathogens and commensals, we aim to identify the universal gene toolbox that is available to various fungal symbionts and that is presumably critical in establishing the symbiotic relationship with the insects.

Harpellales fungi obligately associate with gut linings of aquatic larvae or nymphs of insects (lower Diptera) (5, 32). It has been estimated that their symbiotic relationship has existed since the Permian period (about 270 million years ago) (33). The Harpellales fungi compose a traditional order of Trichomycetes (Zygomycota) (5) and have been recently classified under the new phylum of Zoopagomycota based on genome-scale data (34). Most species within the Harpellales are considered commensals, and they are better known for their harmonious partnerships with the insect hosts, mostly due to their efficient synchronized development (32). One exception would be the species *Smittium morbosum*, which was reported with a unique parasitic lifestyle and which can kill mosquito larvae by penetrating the host gut linings from the inside, anchoring to the exoskeleton, and preventing the host from molting (18). The parasitic stage is also known in the life history of a number of Harpellales genera (i.e., *Genistellospora*, *Harpella*, and *Pennella*) that associate with black flies (35). It has been suggested that certain circumstances (e.g., nutritional stress, pH pressure, etc.) are important for these commensal fungi to shift the relationship with insects between ally and enemy boundaries in both the short and long terms (36–38).

We have sequenced and annotated four new Harpellales genomes in this study. We used these data and five additional genomes to compare a broad range of Harpellales lineages (39, 40). The objectives were to describe the genome features of Harpellales commensals, to conduct analyses of comparative genomics among the nine Harpellales taxa, and to identify the fungus-insect symbiotic core gene (FISCoG) set by further comparing these commensals with the entomopathogenic fungi and free-living relatives. The results of those analyses and the identification of a FISCoG toolbox will help to improve understanding of the basic biology of and the evolutionary relationships between fungi and insects. These resources will be fundamental to efforts to discover the genetic boundaries of symbiosis among parasitism, mutualism, and commensalism.

## RESULTS

### General genome features and comparative genomics of Harpellales*.*

**FIG 1:**
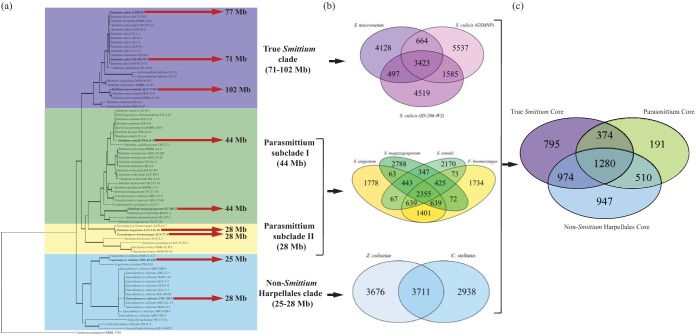
Genome size variation across recognized subclades and Venn diagrams showing homologues across the nine genome-sequenced members of the Harpellales. (a) Harpellales phylogenetic tree based on 5 genes (reconstruced using the data set from reference 41 by adding the strains of S. culicis ID-206-W2 and Capniomyces stellatus MIS-10-108). Branches indicated in bold are considered strongly supported, with Bayesian posterior probability (BPP) values of >95% and maximum-likelihood bootstrap probability (MLBP) values of >0.70. Genome sizes of the recently sequenced 9 taxa were mapped with subclade information (non-*Smittium* Harpellales, true *Smittium*, Parasmittium subclades I and II). (b) Venn diagrams for each subclade derived from analysis of reciprocal best matches of protein-coding genes, showing relatedness and homologous comparisons across the subclades of Harpellales. (c) Identification of the Harpellales feature genes. Clade-specific genes were also identified in comparisons of the three major subclades of Harpellales.

**FIG 2:**
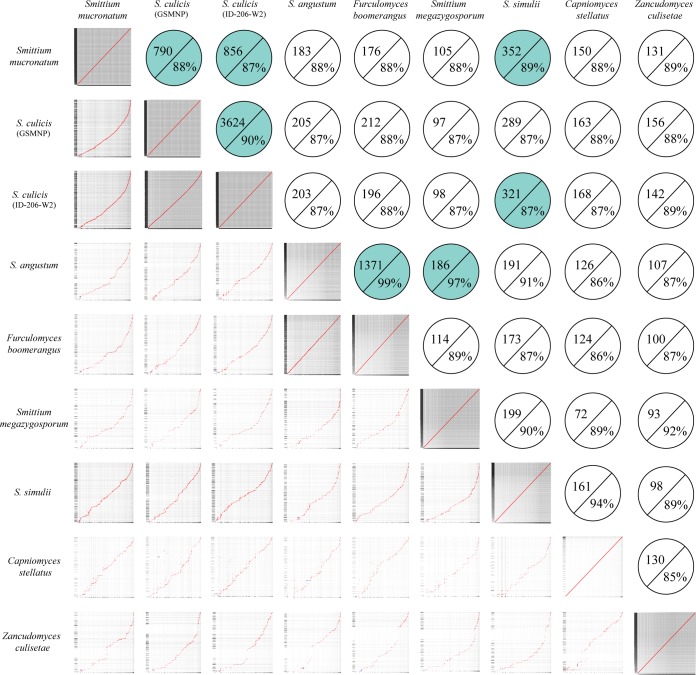
Whole-genome dot plots among the nine Harpellales genome sequences (centered diagonally, from lower left corner; determined using MUMmer plotting). Circles with detailed outputs of comparisons with exact match numbers (left) and the identity level of the matches (right) (centered diagonally, from upper right corner) are shown. Light blue circles indicate the pairs with matched regions longer than 100 kb. A default minimum cluster length of 65 bp was used for the comparison pairs, except for S. mucronatum and S. culicis (GSMNP) (75 bp), S. mucronatum and S. culicis (ID-206-W2) (75 bp), S. culicis (GSMNP) and S. culicis (ID-206-W2) (350 bp), S. angustum and Furculomyces boomerangus (4,000 bp), and S. angustum and S. simulii (70 bp), as well as S. simulii and Capniomyces stellatus (74 bp). Self-comparisons were performed using a minimum cluster length of 500 bp.

Four Harpellales species genomes were sequenced to 165× to 230× coverage and assembled into 1,131 to 3,927 scaffolds (>1 kb). Genome sizes were estimated from the assemblies. There were two classes of genome sizes: one class was 44 Mb (*Smittium simulii* and S. megazygosporum), and one was approximately 28 Mb (S. angustum and Furculomyces boomerangus). The GC (guanine-cytosine) ratios across the whole genomes range from 28% to 33%. The core eukaryotic gene mapping approach (CEGMA) recovered more than 94% of core eukaryotic genes in all four genome assemblies. The *ab initio* protein-coding gene prediction identified 6,519 to 7,385 genes. Detailed genome features and statistics for these four taxa are listed in Table 1 along with those of previously sequenced members of this clade. The genome size variation among the nine Harpellales taxa presented an integer-multiple pattern among lineages of the 5-gene phylogenetic tree (Fig. 1a; reconstructed from reference 41). The names of “true *Smittium*,” “Parasmittium,” and “non-*Smittium* Harpellales” are labels adapted from a previous study (41) as well to refer to the major divergences among the members of this section of the Harpellales tree of life (32, 41, 42) (Fig. 1a). Venn diagrams show the number of shared and uncommon genes for subclades (Fig. 1b) and for Harpellales as a whole group (Fig. 1c). Based on these data, 3,423, 2,355, and 3,711 protein-coding genes are shared within the “true *Smittium*,” “Parasmittium,” and “non-*Smittium* Harpellales” subclades, respectively, and 1,280 of the genes were recovered from all three subclades and thus are proposed to be the genes that are common among the members of Harpellales ("Harpellales common genes") (Fig. 1c). By allowing up to three missing taxa (i.e., by requiring any given gene to be present among six of the nine Harpellales taxa at minimum), the number of Harpellales common genes was found to have increased to 3,094. The genome-level relatedness of the nine Harpellales taxa was shown by aligning and plotting the scaffolds against each other (Fig. 2). The most closely related pairs were suggested by both high-density dot plots and dot alignment proximity to the diagonal curve. The detailed comparison statistics listed in the circles in Fig. 2 indicate both the number and identity level of the matches. Seven pairs were highlighted since their matched bases were longer than 100 kb and are thus suggested to be closely related taxa among the total of 36 comparison pairs (Fig. 2). All three members of the true *Smittium* clade exhibit close relationships with each other, and the two species from the Parasmittium subclade II were revealed to feature the highest identity level (99%). Interestingly, the two individuals from the Parasmittium subclade I presented divergent affinity results; S. megazygosporum shows high identity to one member of the Parasmittium subclade II (S. angustum), and S. simulii is more similar to the two members of the true *Smittium* clade (S. mucronatum and S. culicis ID-206-W2) than to members of the other taxa included here.

**TABLE 1:** Genome features and statistics of the nine Harpellales taxa

Strain	No. of scaffolds (>1 kb)	Genome size by scaffolds (Mb)	% CEGMA (248 in total)	GC ratio (%)	No. of predicted gene models	Repeat ratio (%)	SNP ratio (%)	NCBI accession no.
Smittium culicis GSMNP	6,137	77.12	97.98	28.61	11,209	3.34	0.45	LSSN00000000
Smittium culicis ID-206-W2	7,749	71.05	97.58	29.46	10,024	3.64	0.68	LSSM00000000
Smittium mucronatum	7,797	102.35	93.55	26.05	8,712	2.94	0.75	LSSL00000000
Zancudomyces culisetae	1,954	28.70	92.74	35.52	7,387	4.29	0.64	LSSK00000000
*Smittium megazygosporum*	3,927	43.63	96.77	32.49	7,132	4.60	0.41	MBFS00000000
Smittium angustum	1,283	28.05	99.19	32.40	7,385	1.60	0.43	MBFU00000000
Furculomyces boomerangus	1,312	28.13	99.19	32.37	7,338	1.58	0.43	MBFT00000000
Capniomyces stellatus	72	24.85	97.18	37.82	6,649	4.54	0.06	LUVW00000000
*Smittium simulii*	1,131	43.91	94.76	28.38	6,519	3.38	0.58	MBFR00000000

### Phylogenomics of the Harpellales*.*

**FIG 3:**
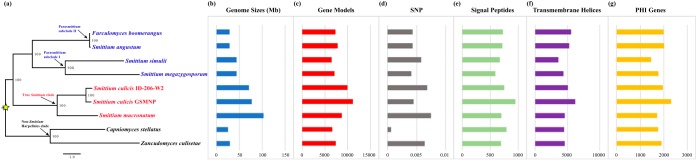
Phylogenomics and genome statistics of Harpellales. (a) The phylogenomic tree was reconstructed based on a concatenated alignment of 1,241 homologues using IQ-TREE v1.5.3 for maximum-likelihood analysis and ultrabootstrap analysis performed with 1,000 replications (true *Smittium* and Parasmittium members are colored in red and blue, respectively, while non-*Smittium* Harpellales taxa are in black). (b to g) Genomic feature of the Harpellales in the order of genome sizes (b), predicted gene models (c), single nucleotide polymorphism sites (d), signal peptide numbers (e), transmembrane helix numbers (f), and numbers of genes that have homologues in the Pathogen-Host Interaction (PHI) database (g).

**FIG 4:**
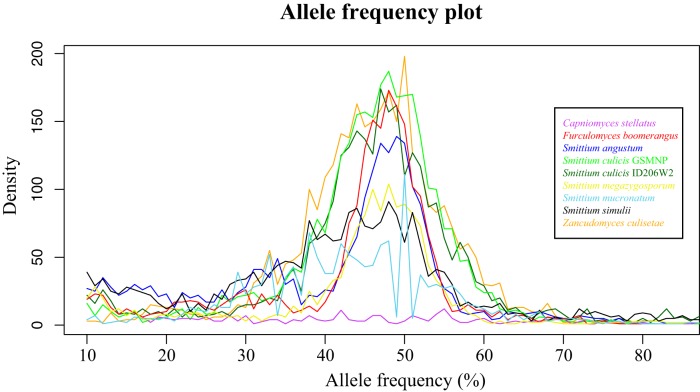
Genome-wide allele frequency distribution among the single-copy orthologs of the nine Harpellales taxa (applied to 460 to 484 transcripts individually, allowing one taxon to be missing from among the nine). Eight of the nine taxa (except C. stellatus) exhibited a cumulative percentage around the 50% position, suggesting a disomic tendency of the genomes. Specifically, 1,398 to 3,235 variable nucleotide positions were analyzed and plotted for the eight Harpellales (allele frequency interval of 10% to 90%) but only 238 for the C. stellatus.

The Harpellales phylogenomic tree was reconstructed based on 1,241 homologous protein sequences (Fig. 3a). The tree provides a robust phylogeny of the Harpellales and supports the topology proposed earlier on the basis of analyses performed using five genes (41). With the genomic information mapped to the phylogenomic tree (Fig. 3b to g), the data clearly show a pattern of lineage-specific genome size variation—the two lineages depicted in Fig. 3a (Parasmittium subclade II and Non-*Smittium* Harpellales clade), including Capniomyces stellatus, Furculomyces boomerangus, Smittium angustum, and Zancudomyces culisetae, have genome sizes between 25 and 28 Mb, whereas the taxa among other lineages show the genome sizes close to intervals in multiples of 25 Mb. This pattern implies that whole-genome duplication (WGD) might have occurred more than once and that the initial event might have been close to the time of divergence between the *Smittium* and non-*Smittium* clades (Fig. 3a; labeled with a star). Regardless of their genome size differences, there is no striking distinction with respect to the numbers of gene models, signal peptide genes, transmembrane helix genes, and pathogen-host interaction genes (Fig. 3b and c and 3e to g). Among the four taxa whose genome sizes are close to 25 Mb, we identified 1,071 single-copy genes. Almost half of them (582 of the 1,071) are found with multiple copies in at least one of the remaining five taxa (genome size, ≥44 Mb) (see Table S1 in the supplemental material). In total, 527 phylogenetic trees were reconstructed based on the well-recovered “single-copy” genes (55 of the 582 homologous groups were disregarded due to short alignment length) to infer their phylogenetic relationships (see Fig. S1 in the supplemental material). Most of the 527 homologous copies from the same taxon group with each other, and none of them exhibits divergence with statistical support. This result implies that the identified genome-size duplications may represent independent events and might be less likely to be due to interspecies hybridizations. The sites with single nucleotide polymorphisms (SNPs) were persistently found by the use of the binary version of the sequence alignment map files of the Harpellales genomes. The percentages of the SNP sites across the eight genomes of Harpellales species (except that of Capniomyces stellatus) range from 0.41% to 0.75%, while that of Capniomyces stellatus has a much lower value (0.06%; Table 1) (Fig. 3d). These ratios (except that corresponding to C. stallatus) are comparable to those of the following diploid organisms from various kingdoms: 0.19% in Pisum sativum L. (43), 0.32% in Homo sapiens (44), 0.35% in Edhazardia aedis (31), and 1.2% in Candida albicans (45). The genome-wide allele frequency plot for the single-copy orthologs additionally supports this peculiar finding and suggests that Capniomyces stellatus is likely haploid whereas the other eight Harpellales may be all diploid, with a noticeable peak at the position of 50% allele frequency (Fig. 4).

10.1128/mBio.00636-18.1 FIG S1  A total of 527 independent phylogenetic trees were reconstructed using the maximum-likelihood method to infer the relatedness of multiple copies found from the same taxa. For each of the 527 trees, the four Harpellales taxa with the smallest genomes (Capniomyces stellatus, Furculomyces boomerangus, Smittium angustum, and Zancudomyces culisetae; 25 to 28 Mb) contain only one copy of the orthologous gene; at least one of the remaining five taxa has two or more copies. Tree tips were labeled with the locus name as follows: Capniomyces stellatus (CSE02), Furculomyces boomerangus (BB559), Smittium angustum (BB558), S. megazygosporum (BB560), S. mucronatum (AYI68), S. simulii (BB561), S. culicis strains GSMNP (AYI70) and ID206W2 (AYI69), and Zancudomyces culisetae (AX774). Download FIG S1, PDF file, 1.3 MB. © Crown copyright 2018. 2018 Crown This content is distributed under the terms of the Creative Commons Attribution 4.0 International license.

10.1128/mBio.00636-18.3 TABLE S1  A total of 1,071 single-copy genes were identified from the four Harpellales taxa with the smallest genomes (Capniomyces stellatus, Furculomyces boomerangus, Smittium angustum, and Zancudomyces culisetae; 25 to 28 Mb). In total, 582 of the 1,071 orthologous groups (ranked at the top) were selected for further phylogenetic analyses (at least one of the remaining five taxa have minimum of two copies). Each taxon is labeled with the locus name individually as follows: Capniomyces stellatus (CSE02), Furculomyces boomerangus (BB559), Smittium angustum (BB558), S. megazygosporum (BB560), S. mucronatum (AYI68), S. simulii (BB561), S. culicis strains GSMNP (AYI70) and ID206W2 (AYI69), and Zancudomyces culisetae (AX774). Download TABLE S1, PDF file, 0.5 MB. © Crown copyright 2018. 2018 Crown This content is distributed under the terms of the Creative Commons Attribution 4.0 International license.

### Fungus-insect symbiotic core gene/domain toolbox.

**FIG 5:**
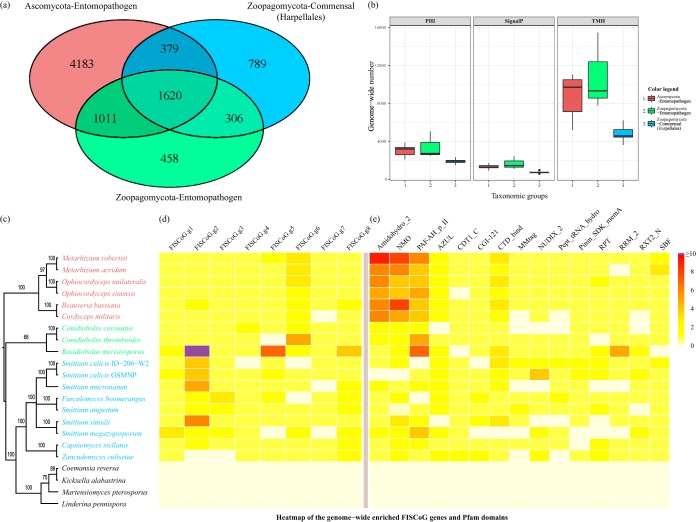
Comparative genomics between the entomopathogenic fungi (Ascomycota in red and Zoopagomycota in green) and insect commensals of the Harpellales (in blue). (a) Venn diagram derived from interphylum homologues with the aim to sort out fungus-insect symbiotic core genes (FISCoGs), using pathogenic representatives both from Ascomycota and Zoopagomycota and commensals from Harpellales. (b) Box plot comparisons of genome-wide PHI genes, signal peptides, and transmembrane helices among the three groups. (c) Cladogram exhibiting the phylogenetic relationship of the included taxa based on 29 shared single-copy genes. (d) Heat map enrichment of the FISCoG toolbox among the insect-associated fungi (analyzed by removing the 1,612 false-positive hits with non-insect-associated Zoopagomycota genomes from those corresponding to the 1,620 shared genes in panel a). (e) Heat map comparison showing the enrichment pattern of genome-wide Pfam domains (detailed information for the fungus-insect symbiotic core domains is listed in Table S3).

Representatives of the Ascomycota entomopathogenic fungi (Beauveria bassiana, Cordyceps militaris, Metarhizium acridum, Metarhizium robertsii, Ophiocordyceps sinensis, and Ophiocordyceps unilateralis) share 7,193 protein-coding genes (with no more than 2 of the 6 taxa allowed to be missing from those sharing the genes) (26, 27, 29, 46–48). Zoopagomycota entomopathogenic representatives (Basidiobolus meristosporus, Conidiobolus coronatus, and Conidiobolus thromboides) share 3,395 protein-coding genes (with no taxa allowed to be missing) (Fig. S2) (49–51). By comparing the 3,094 Harpellales common genes (with no more than 3 of the 9 taxa allowed to be missing) with both the Ascomycota and Zoopagomycota entomopathogenic core genes, we identified 1,620 protein-coding genes that are shared among both the insect commensals and pathogens (Fig. 5a). In screening out universal genes that are not specific to the fungus-insect associations, we found that 1,612 of them (among 1,620 protein-coding genes) had similarity hits (with an E-value of less than 1E^−5^) in at least one of the negative-control proteomes among free-living Zoopagomycota fungi (Coemansia reversa, Kickxella alabastrina, Linderina pennispora, and Martensiomyces pterosporus) (49, 50). Only eight genes were found to be unique to these insect-associated fungi. Here we propose these eight protein-coding genes as candidates for the fungus-insect symbiotic core gene (FISCoG) toolbox. Functional annotation indicates that these FISCoGs are mainly responsible for adhesion, binding, gene transcription, and protein degradation in various subcellular locations (Table 2). Four of the eight genes were found to have homologues in the pathogen-host interaction (PHI) database (PHI:733/PHI:6752, PHI:2524, PHI:4231, and PHI:5571), matching the evidence seen with the invasive pulmonary pathogen Aspergillus fumigatus (52), rice blast fungus Magnaporthe oryzae (53–55), and cereal-devastating pathogens Fusarium verticillioides and F. graminearum (56), as well as Salmonella enterica, the causative agent of a spectrum of diseases (57). In a broader comparison, Harpellales genomes harbor fewer transmembrane helix genes, pathogen-host interaction genes, and signal peptide genes than entomopathogenic species of both Ascomycota and Zoopagomycota (Fig. 5b). Interestingly, O. sinensis, like Harpellales species, possesses small amounts of the products mentioned above, which may reflect their biological distinction in their use of the oral-gut pathway during the fungal-insect infection rather than of the exoskeleton route used by other fungal pathogens (Table S2). The FISCoG enrichment analyses (Fig. 5d) further revealed that Harpellales and Basidiobolus meristosporus have the most genes encoding the cell adhesion proteins (FISCoG.g2), whereas entomopathogenic fungi tend to have more copies of genes encoding “platelet-activating factor acetylhydrolase” (PAF-AH) (FISCoG.g6) than Harpellales commensals. The genome-wide protein family (Pfam) domain enrichment pattern suggests that 15 domains are fungus-insect symbiotic core domains (FISCoD) and that 3 of them—“Amidohydro_2,” nitronate monooxygenase (“NMO"), and “PAF-AH_p_II”—are specifically enriched in entomopathogenic fungi (Fig. 5e).

10.1128/mBio.00636-18.2 FIG S2  Venn diagram derived from reciprocal best match of protein-coding genes among Basidiobolus meristosporus, Conidiobolus coronatus, and Conidiobolus thromboides. Download FIG S2, PDF file, 0.4 MB. © Crown copyright 2018. 2018 Crown This content is distributed under the terms of the Creative Commons Attribution 4.0 International license.

10.1128/mBio.00636-18.4 TABLE S2  Genome-wide comparisons among 18 insect-associated fungi. Download TABLE S2, PDF file, 0.1 MB. © Crown copyright 2018. 2018 Crown This content is distributed under the terms of the Creative Commons Attribution 4.0 International license.

10.1128/mBio.00636-18.5 TABLE S3  Information on the fungus-insect symbiotic core domains. Download TABLE S3, PDF file, 0.1 MB. © Crown copyright 2018. 2018 Crown This content is distributed under the terms of the Creative Commons Attribution 4.0 International license.

**TABLE 2:** Information and detailed annotations of the FISCoG toolbox^a^

FISCoG	Description	GO name(s) (with GO-Slim)	Homologue with demonstrated function(s)	Subcellular location prediction (probability)	PHI hit(s) (1E−3)
FISCoG.g1	Peroxisomal NADH pyrophosphatase NUDT12	F, hydrolase activity	Regulation of concn of peroxisomal nicotinamide nucleotide cofactors required for oxidative metabolism (72)	Peroxisomal (0.65/1.00)	N/A
FISCoG.g2	Fasciclin domain- containing protein (beta-Ig-H3)	C, fungal vacuole membrane, extracellular space, membrane, integral component of membrane; P, macroautophagy	Cell adhesion protein (58–61)	Extracellular (0.80/1.00)	PHI:4231
FISCoG.g3	Acyl-CoA N- acyltransferase	F, N-acetyltransferase activity (transferring acyl groups)	Involvement in intestinal colonization and systemic infection (57)	Cytoplasmic (0.96/1.00)	PHI:5571
FISCoG.g4	Nuclear movement protein NudC	N/A	Nuclear migration and distribution (52)	Cytoplasmic (0.73/1.00)	PHI:2524
FISCoG.g5	F-box/LRR-repeat protein 2	F, protein kinase activity, ATP binding, kinase activity, ligase activity; P, protein phosphorylation, phosphorylation	Ubiquitin ligase complex F-box protein that mediates the ubiquitination and subsequent proteasomal degradation of target proteins (69, 70)	Cytoplasmic (0.81/1.00)	PHI:733; PHI:6752
FISCoG.g6	Platelet-activating factor acetylhydrolase	F, 1-alkyl-2- acetylglycerophosphocholine esterase activity; P, lipid catabolic process	Enzyme that catabolizes platelet-activating factor (65, 66)	Cytoplasmic (0.68/1.00)	N/A
FISCoG.g7	Putative SET-like protein	N/A	Related to growth control, gene transcription, and chromatin structure (73)	Nuclear (0.88/1.00)	N/A
FISCoG.g8	RNA-binding protein Nrd1	F, RNA binding; C, cytoplasm; P, negative regulation of conjugation with cellular fusion by regulation of transcription from RNA polymerase II promoter (reproduction, biosynthetic process, cellular nitrogen compound metabolic process)	Nucleotide binding; nucleic acid binding (71)	Nuclear (0.94/1.00)	N/A

a F, molecular function; C, cellular component; P, biological process; N/A, not available.

## DISCUSSION

### Fungus-insect symbiotic core genes/domains toolbox.

By comparing the insect-symbiotic fungal genomes, including both commensals and pathogens corresponding to the taxa included in this study, we identified an eight-gene toolbox consisting of genes that are unique to the fungus-insect associations and missing from the closely related free-living relatives. The genome-wide enrichment analyses of these eight genes also highlighted several players that are important for the fungi during symbiosis. FISCoG.g2 was found to be specifically enriched in Harpellales and *Basidiobolus*, whereas FISCoG.g6 is instead enriched in entomopathogenic fungi (Fig. 5b). The FISCoG.g2 gene is a novel gene encoding 562 amino acids (aa), and the N-terminal 300 aa contain two repeats of the fasciclin (fas1) domain in close succession, while the C-terminal region (~262 aa) has no known function assigned (according to the results of a BLAST search, with the best hit with the *beta-ig-h3*/*fasciclin* gene; Table 2). Fas1 domain-containing proteins participate in cell adhesion and communication and are present in many eukaryotes (58–61). FISCoG.g2 is thus suggested to have similar adhesion functions at a minimum. Many fungal pathogens utilize proteins to adhere to host cells and in formation of biofilms. Some well-characterized genes in Candida albicans and Metarhizium anisopliae include *Als1* (62), *Flo11* (63), and *Mad1* and *Mad2* (64). A search of these genes found only a few (Flo11 and Mad1) or none (Als1 and Mad2) in the Harpellales genomes. These disparate data with respect to the preference for an adhesion protein reflect the different tools utilized by these symbiotic fungi in establishing the relationship with their animal hosts. In addition, FISCoG.g2 is suggested to be an extracellular protein (Table 2) and Harpellales asexual spores were earlier found to release glue in certain pH environments during the passage through the host gut (38). Give the evidence, it is reasonable to expect that a novel gut-attaching strategy is utilized by the Harpellales fungi. In that regard, FISCoG.g2 is suggested to be an important excreted protein for the gut-dwelling lifestyle of Harpellales. Homologues of the FISCoG.g6 are annotated with the function of being platelet-activating factor acetylhydrolases (PAF-AH). The platelet-activating factor (PAF) is often referred to specifically as a proinflammatory messenger and is widely utilized in both vertebrate and invertebrate animals (65, 66). The biological functions of the PAF-AH include regulation of inflammation through the inactivation or deconstruction of PAF in the animal system and thus play an important role during the initial step of fungal invasion. From the independent view of the comparative Pfam domains (Fig. 5c), the corresponding “PAF-AH_p_II” domain has also been identified as an important functional domain uniquely maintained by the insect-associated fungi and enriched in the entomopathogens. In addition, the Amidohydro_2 and NMO domains present a similar pattern but are enriched only in the Ascomycota entomopathogens (Fig. 5c). Nitronate monooxygenase (NMO) was recently proven to be a novel factor in suppressing the host defense and promoting invasive hyphal growth and development during the pathogenic invasion using the example of the rice pathogen Magnaporthe oryzae (67). As a result, similar biological roles for the NMO-containing proteins during the fungus-insect interactions are anticipated, with the evidence that NMO was uniquely identified in insect-associated fungal proteomes and enriched in the entomopathogenic species. The specific function of the Amidohydro_2 domain is still unknown; however, the amidohydrolase family includes adenine deaminase, which has been found to be important for adenine utilization and for providing a nitrogen source (68). Both FISCoG.g6 and PAF-AH_p_II and additional domains of NMO and Amidohydro_2 are suggested to serve important biological functions for the interaction of entomopathogens (especially Ascomycota representatives) with their insect hosts.

Interestingly, the Basidiobolus meristosporus genome is enriched for FISCoG.g2, FISCoG.g5, and FISCoG.g8. Similarly to the aquatic Harpellales fungi, B. meristosporus seems to favor this novel fasciclin adhesion protein as well to maintain its residency within amphibian and insect hosts. FISCoG.g5 is suggested to have the function of mediating ubiquitination and subsequent proteasomal degradation of targeted proteins (69, 70). FISCoG.g8 is predicted to be a differentiation regulator and has outstanding nucleotide/nucleic acid binding ability (according to the results of a BLAST search, with the best hit with Nrd1) (71). It is implied by the enrichment data that these three FISCoGs play bigger roles in the interaction of B. meristosporus with the animal hosts, emphasizing the abilities of adhesion, protein degradation, and nucleotide binding. The other four FISCoGs present no obvious enrichment patterns, although they have been suggested to contribute to oxidative metabolism, nuclear migration, and gene transcriptions during the fungus-insect interactions (52, 57, 72, 73).

The Harpellales fungal spores enter immature aquatic insect hosts via oral ingestion, and they germinate in the midgut or hindgut, where they finish the rest of the life cycle and release asexual or sexual spores at maturity during the intermolt phases of their insect hosts (5). This route of entry throughout ingestion and in-gut development exemplifies the prominent differences between Harpellales and most entomopathogenic fungi, including B. bassiana, Conidiobolus coronatus, C. thromboides, Cordyceps militaris, M. robertsii, and O. unilateralis, which heavily rely on their ability to degrade and penetrate the chitinous exoskeleton of potential insect hosts (27, 74). Usually, the secreted proteins that allow these penetration processes also play major roles in immune evasion (31). Ophiocordyceps sinensis is special in the way that it infects insect hosts through spiracles or the mouth and thus avoids the cuticle degradation step (75). As a result, the presentation of smaller amounts of signal peptide genes, transmembrane helix genes, and PHI genes in both Harpellales and O. sinensis than in the rest of the entomopathogens studied might be explained by their similar host invasion strategies in the form of taking the route of available openings of the host (see Table S2 in the supplemental material). The recent identification of protein family expansions (30, 75) corroborated the suggestion that O. sinensis has a much smaller number of CYP52 enzymes, subtilisins, trypsins, and aspartyl proteases than B. bassiana, C. militaris, or M. robertsii and that all are utilized for degradation of the insect cuticles.

### Host specificity.

It was suggested previously that a high proportion of secreted proteins positively correlate with the fungal parasitic lifestyle (27). Here we show that PHI genes, signal peptide genes, and transmembrance helix genes are all found in greater numbers in both Ascomycota and Zoopagomycota pathogens than in the Harpellales commensals (Fig. 5b). Genomes of host generalists tend to be equipped with genes that encode expanded numbers of protein families, while specialists encode greater numbers of species-specific proteins but lack the diversity of genes that encode the secretory signal peptides that are used to interact with various hosts (31). Surprisingly, a few host-specialized fungi are found with large genome sizes, although the number of gene models does not increase proportionally (31, 39, 48, 76). One interpretation suggests that the genome size variation could be a consequence of variation in telomeric regions represented by noncoding and small genomic repetitions and, thus, that the larger genome size is accompanied by increased genomic complexity (76).

Ophiocordyceps sinensis requires the ghost moth (Hepialidae) as a host to complete its life cycle, and both the fungus and insect hosts are endemic in the Qinghai-Tibet Plateau in western China (77). Ophiocordyceps sinensis was reported to have potential hosts, including 57 species of the ghost moth (78), although low genetic diversity among these potential hosts was suggested by the results of studies performed using the mitochondrial cytochrome C oxidase subunit I (COI) marker (77). Contemporarily, Zhang et al. (79) revealed higher genetic variation in both the fungus and the ghost moth using 7 fungal and 3 insect markers and further suggested that O. sinensis cospeciated with the ghost moth on the basis of significant cophylogenetic congruence and similar divergence times. In accordance with the aforementioned results, we assume that O. sinensis is highly restricted to the ghost moth hosts; thus, we treated it as a host-specialized fungal pathogen in this study (Table S2). O. unilateralis is a well-known specialized fungal pathogen that manipulates and kills formicine ants (74).

The generalist-specialist pairs in taxonomic clade present comparable patterns—the genome of the generalist M. robertsii includes more signal peptide genes (1,707 versus 1,212), transmembrane helix genes (11,022 versus 9,136), and PHI genes (3,858 versus 3,268) than the specialist M. acridum; similarly, genes of the members of the specialist group consisting of O. sinensis and O. unilateralis encode noticeably fewer signal peptides, transmembrane helices, and PHI proteins than the genes of the other Ascomycota generalist entomopathogens (Table S2). The higher signal peptide number in O. unilateralis than in O. sinensis may contribute to a process described in a recent finding in which O. unilateralis cells invade host muscle fibers and form networks throughout the body to perform the manipulations (15). Similar patterns have been revealed in the Harpellales (Zoopagomycota; Kickxellomycotina). The hosts of Smittium mucronatum were found to be restricted to *Psectrocladius* (midge) (5), and the numbers of its signal peptide genes (700), transmembrane helix genes (4,550), and PHI genes (1,725) were all found to be lower than the corresponding Harpellales averages (733, 4,837, and 1,880) (Table S2). Capniomyces stellatus, as another host-specialized Harpellales, was reported to be present specifically in winter stoneflies (Capniidae and Taeniopterygidae) (80, 81). Similarly to S. mucronatum, Capniomyces stellatus harbors lower numbers of transmembrane helix genes (4,440) and PHI genes (1,763) than the Harpellales averages but a higher number of signal peptide genes (792). The data imply that C. stellatus may maintain a more intensive interaction with the stonefly hosts than we previously thought. The noncoincidental large genome sizes of O. sinensis and S. mucronatum may be the result of host specialization and increased genome complexity, a conjecture that was also strengthened by several independent findings (31, 48, 76).

### Harpellales genome evolution.

We used the newly produced Harpellales genome sequences to reconstruct the first phylogenomic tree of Harpellales using 1,241 orthologous genes (Fig. 3a). Analysis of the phylogenomic tree indicates a pattern implying that multiple genome-level duplications may have occurred (Fig. 3b). One parsimonious possibility is that the genome size of the Harpellales ancestor is similar with that of the members of the basal non-*Smittium* Harpellales clade (*Capniomyces* and *Zancudomyces*). The sizes remain the same in both clades of non-*Smittium* Harpellales and in Parasmittium subclade II. The size nearly doubled (from ~25 Mb to ~44 Mb) when Parasmittium subclade I diverged from the Parasmittium subclade II. The true *Smittium* genome sizes (71 to 102 Mb) approximately tripled in comparison to that of the Harpellales ancestor (~25 Mb), and then the genome size of S. mucronatum enlarged further due to the host specialization. Alternatively, the genome size of the Parasmittium ancestor might have experienced duplication followed by reduction of the levels of Parasmittium subclade II members when their genomes further evolved, rearranged, and got rid of the redundant regions. The availability of genome sequences of *Smittium morbosum* and *Stachylina* from a sister clade would help confirm this explanation (Fig. 1a). Unfortunately, the lack of culture of both strains prevented us from getting their genome information easily. The true *Smittium* clade holds the largest genome sizes in the Harpellales. It is also likely that the genome sizes of the true *Smittium* members underwent more than one whole-genome duplication from the Harpellales ancestor (Table S1).

It is still relatively rare to discover whole-genome duplication (WGD) events in fungal genomes, although studies of the members from both Dikarya and early-diverging groups have resulted in the recent publication of independent reports of the finding of WGD, with increasing number of genome sequences and improving technology (82–86). The yeast (Saccharomyces cerevisiae) genome has been intensively studied since its production (87). Found with many duplicated genes, yeasts were thought to have undergone genome duplication that was followed by massive gene loss events (88), although this was not officially confirmed until the production of the genome of a more basal relative, Kluyveromyces waltii (82). Phylogenetic evidence recently found that the yeast genome doubling was the result of a contemporaneous interspecies hybridization within the baker’s yeast lineage (89). Immediately after that, the genome hybridization model was proposed (90). Multiple Harpellales species have been identified from the same host gut, and in some cases, they may influence their relative locations along the gut (91). It would be worthwhile to further test whether the interspecies hybridization model might explain the Harpellales genome multiplication. In total, 1,071 single-copy genes were identified from the four smaller-genome-size taxa (genome sizes of between 25 and 28 Mb). The finding of various numbers of copies (0 to 4) present in the larger-genome-size taxa (genome sizes of ≥44 Mb) suggests that the duplication events were so ancient that many of the copies have been lost (Table S1). The 527 independent phylogenetic trees were built using the single-copy markers, and none supports the interspecies hybridization hypothesis (Fig. S1). However, we still cannot exclude the possibility of ancient hybridization due to the fact that the signal might be too weak to correspond to the ancient age of the event. In addition, this method also cannot exclude the idea of the possibility of hybridization between closely related species. The *Smittium* and non-*Smittium* clades diverged much earlier (270 Ma) than the yeast hybridization event (100 to 140 Ma), which could be one of the major obstacles to detection of such a signal (33, 89, 92). The draft quality of Harpellales genomes also prevents us from comparing their syntenic data at the chromosome level. With future research efforts designed to refine high-quality genome assemblies, new comparative genomic methods will serve to help unravel the mystery of this symbiotic system.

## MATERIALS AND METHODS

### Fungal strains, DNA extraction, and whole-genome sequencing.

Furculomyces boomerangus (AUS-77-4; ARSEF 9021), Smittium angustum (AUS-126-30; ARSEF 9241), S. megazygosporum (SC-DP-2; ARSEF 9037), and S. simulii (SWE-8-4; ARSEF 9139) were obtained from the USDA-ARS Collection of Entomopathogenic Fungal Cultures (ARSEF). Fungal cultures were grown and DNA was extracted following earlier protocols (39). TruSeq Nano paired-end (PE) libraries with an insertion size of 500 bp were prepared for each of Furculomyces boomerangus, S. angustum, and S. megazygosporum. *Smittium simulii* was prepared using one PCR-free PE library (500-bp insertion size) and two Nextera mate-pair (MP) libraries (3-kb and 5-kb insertion sizes). All were sequenced using an Illumina HiSeq 2500 platform (2 × 125-bp read length) at the Donnelly Sequencing Center, University of Toronto (Canada).

### Genome assembly and annotation.

Raw FASTQ sequence reads were subjected to adapter trimming using Trim Galore v0.4.1 (http://www.bioinformatics.babraham.ac.uk/projects/trim_galore/) and were quality checked using FASTQC v0.11.4 (http://www.bioinformatics.babraham.ac.uk/projects/fastqc/). Genomes were assembled with RAY v2.3.1 (93). The scaffolds of S. simulii were built using SSPACE (94). Satellites, simple repeats, and low-complexity sequences were annotated with RepeatMasker v4.0.5 (http://www.repeatmasker.org) and Tandem Repeat Finder v4.07b (95), corresponding to fungal sequences from RepBase (96). The genomes were annotated with the Funannotate v0.6.2 pipeline (https://github.com/nextgenusfs/funannotate), employing tools of Augustus (97), GeneMark.hmm-ES (98), and EVM (99). Gene function was inferred from matches to the databases of Pfam (100), Merops (101), CAZy (102), InterProScan (103), and Swiss-Prot (104). Product descriptions were assigned with homologues with 60% similarity across 60% of the protein length (105). CEGMA v2.4.010312 was used to identify the presence of core eukaryotic-protein-coding genes and for subsequent evaluation of genome coverage (106). Secreted proteins were predicted using SignalP v4.1 (no truncation to the sequence length) (107), and transmembrane helices were predicted using TMHMM v2.0 (108). Potential pathogenic proteins were identified using BLASTP against pathogen-host interaction (PHI) database v4.4 (with 4,376 entries) (109).

### Homologue identification and phylogenomics of Harpellales.

Putative homologues among the nine Harpellales genomes were identified using two independent methods. The first method employed reciprocal similarity searches using BLASTP v2.2.30 (cutting E value set to 1E^−5^) and a Perl script (“Find_mutual_BestHit.pl”; available from GitHub) and filtering for the reciprocal best hit. The second method employed MCL v14-137 (110) and a Python script (“Ortho_Rep.py”; available from GitHub), selecting the representatives of each clustering group based on a theoretical graph approach—the best sequence of each taxon from each cluster was chosen based on its connectivity (weighted by log_10_-transformed E-value) to all other sequences. Venn diagrams were produced using R v3.1.3 to visualize the results of comparisons. A group of all 1,280 of the shared Harpellales homologues retrieved as described above was used for phylogenomic analyses. Multiple-sequence alignment was performed using MUSCLE v3.6 (111). Gaps and poorly aligned regions were excluded using trimAl v1.4 (112). Aligned protein sequences that were longer than 50 amino acids (1,241 of the 1,280 homologues) were selected using a Python script (“Filter_Len.py”; available from GitHub) and then concatenated using FASconCAT-G v1.02 (113). Data corresponding to appropriate substitutional models, the best partition scheme, phylogenomic tree reconstruction, and ultrafast bootstrap analyses were all inferred and analyses conducted within the IQ-TREE v1.5.3 package (114–116). In order to test whether the revealed integer-multiple pattern of genome sizes was due to interspecies hybridization, we estimated the phylogenetic relationship of the multiple-copy homologous genes within the larger genomes (44 to 102 Mb). We used the comparative genomic pipeline (https://github.com/stajichlab/Comparative_pipeline) to identify 582 single-copy protein-coding genes that were all shared among the four taxa with the smallest genome sizes (25 to 28 Mb) and that also were found to be present with a minimum of two copies in at least one of the remaining five taxa (genome sizes between 44 and 102 Mb). Individual homologous alignments were prepared similarly to those described above. Finally, 527 phylogenetic trees were reconstructed using the maximum-likelihood analysis and the ultrabootstrap method and 1,000 replications with the IQ-TREE tool (55 of the 582 alignments were discarded due to having a length shorter than 50 aa).

### Single nucleotide polymorphisms, allele frequencies, and dot plot comparisons.

The whole-genome-wide SNPs were identified using BWA (117), SAMtools (118), Picard Tools (http://broadinstitute.github.io/picard), and the GATK toolkit (119), according to the previously described pipeline method (120). A total of 460 to 484 single-copy orthologs among the nine Harpellales (with one taxon allowed to be missing from the total of nine taxa) were discovered from the earlier step using the comparative pipeline. The individual allele frequency was calculated using the read counts of the binary alignment map (BAM) files against the single-copy orthologs with the “pileup2snp” function of VarScan v2.3.9 (125) (default parameters were determined using a –min-coverage value of 10 and a *P* value of 0.05). The allele frequency value was then rounded to an integer value before being plotted with the number of occurrences and level of density using R v3.1.3 (http://www.r-project.org). The Harpellales genome dot plot comparisons were generated using the MUMmer (v3.23) package (121). The “nucmer” algorithm and the “delta-filter” function were employed to find the reciprocal best matches between the genome scaffolds. Total numbers of matches and aligned bases were calculated using the “show-coords” function. The percentage of identity was calculated using a Python script (“Calcu_Identity.py”; available from GitHub).

### FISCoG toolbox.

The FISCoG toolbox was identified by comparing the Harpellales core genes (including those allowed to be mostly missing but minimally present in six of the nine taxa) with those corresponding to the representatives of entomopathogenic fungi from the Ascomycota and Zoopagomycota groups (27, 29, 46, 49–51). These included six taxa from Ascomycota—Beauveria bassiana, Cordyceps militaris, Metarhizium acridum, Metarhizium robertsii, Ophiocordyceps sinensis, and Ophiocordyceps unilateralis (allowing each gene to be missing from up to two taxa) and three from Zoopagomycota—*Basidiobolus meristosporus*, Conidiobolus coronatus, and Conidiobolus thromboides (with no genes allowed to be missing from any taxa). The FISCoG candidates were then subjected to a BLAST search (cutting E-value set to 1E^−5^) against the non-insect-associated Zoopagomycota genomes (Coemansia reversa, Kickxella alabastrina, Linderina pennispora, and Martensiomyces pterosporus) to filter out potential false-positive hits. FISCoG enrichment in each fungal taxon was assessed using BLASTP. The enrichment heat map was produced using the “aheat map” function in R package “NMF” (122). The cladogram of the included taxa was reconstructed using IQ-TREE, based on 29 proteins (longer than 50 aa) encoded by single-copy genes. The GO names and subcellular protein locations of the FISCoGs were predicted using Blast2GO v5.0 and the MultiLoc2 webserver against the fungal high-resolution database, respectively (123, 124).

### Data availability.

All four Harpellales taxa and corresponding genome information have been deposited in DDBJ/ENA/GenBank under the following accession numbers: BioProject identifier (ID) PRJNA329411; BioSamples IDs SAMN05412443, SAMN05412446, SAMN05412450, and SAMN05412451; and Whole-Genome Shotgun IDs MBFU00000000, MBFT00000000, MBFS00000000, and MBFR00000000. Multiple-sequence alignment and phylogenetic tree files are archived in TreeBASE (study ID S22516). Script files created for this study are available at https://github.com/YanWangTF.
