# Supplementary figures and images for: Comparative Genomics Reveals the Core Gene Toolbox for the Fungus-Insect Symbiosis
Source: mBio. 2018 May 15;9(3):e00636-18. doi: 10.1128/mBio.00636-18 (PMC5954228; doi:10.1128/mBio.00636-18)

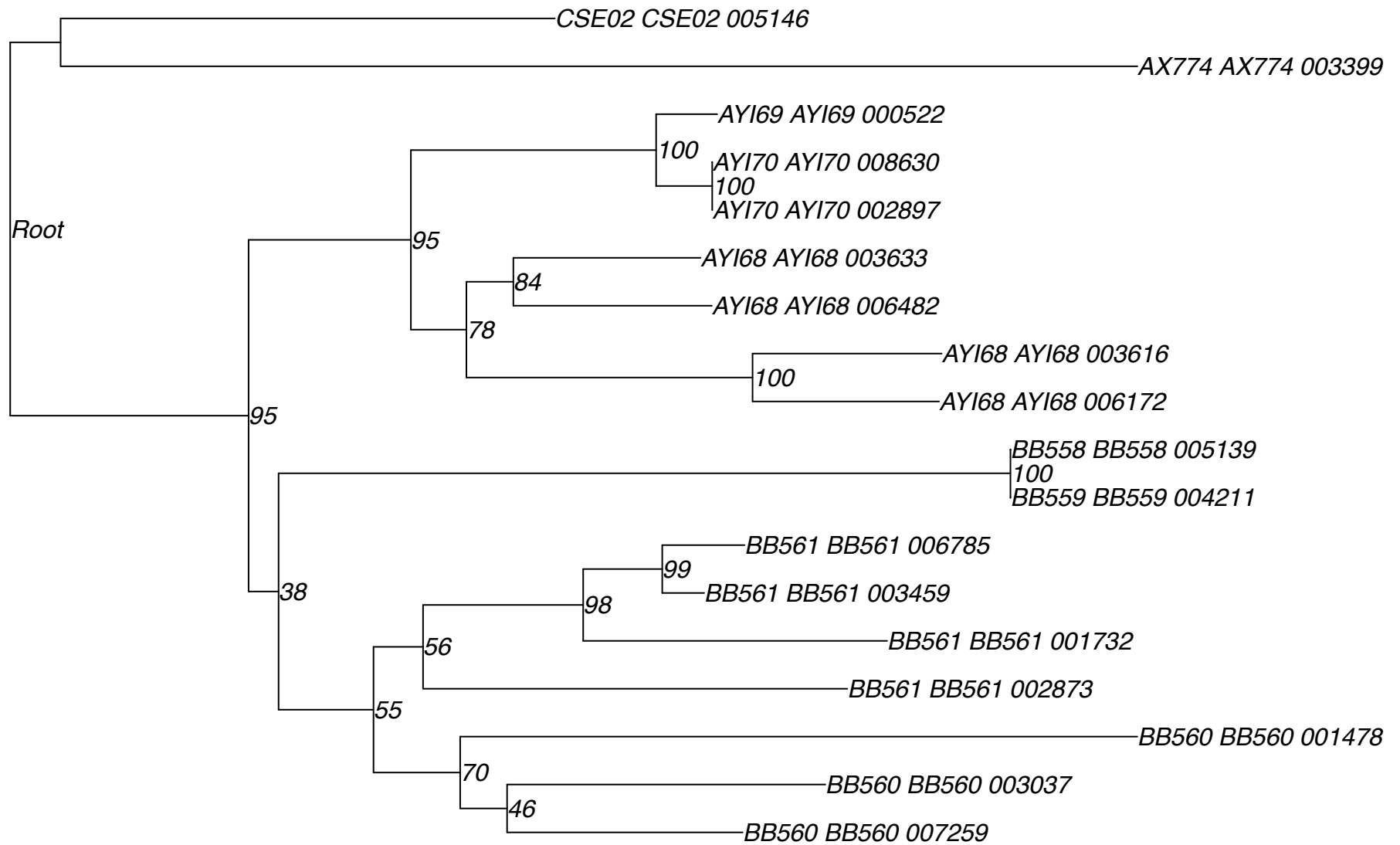

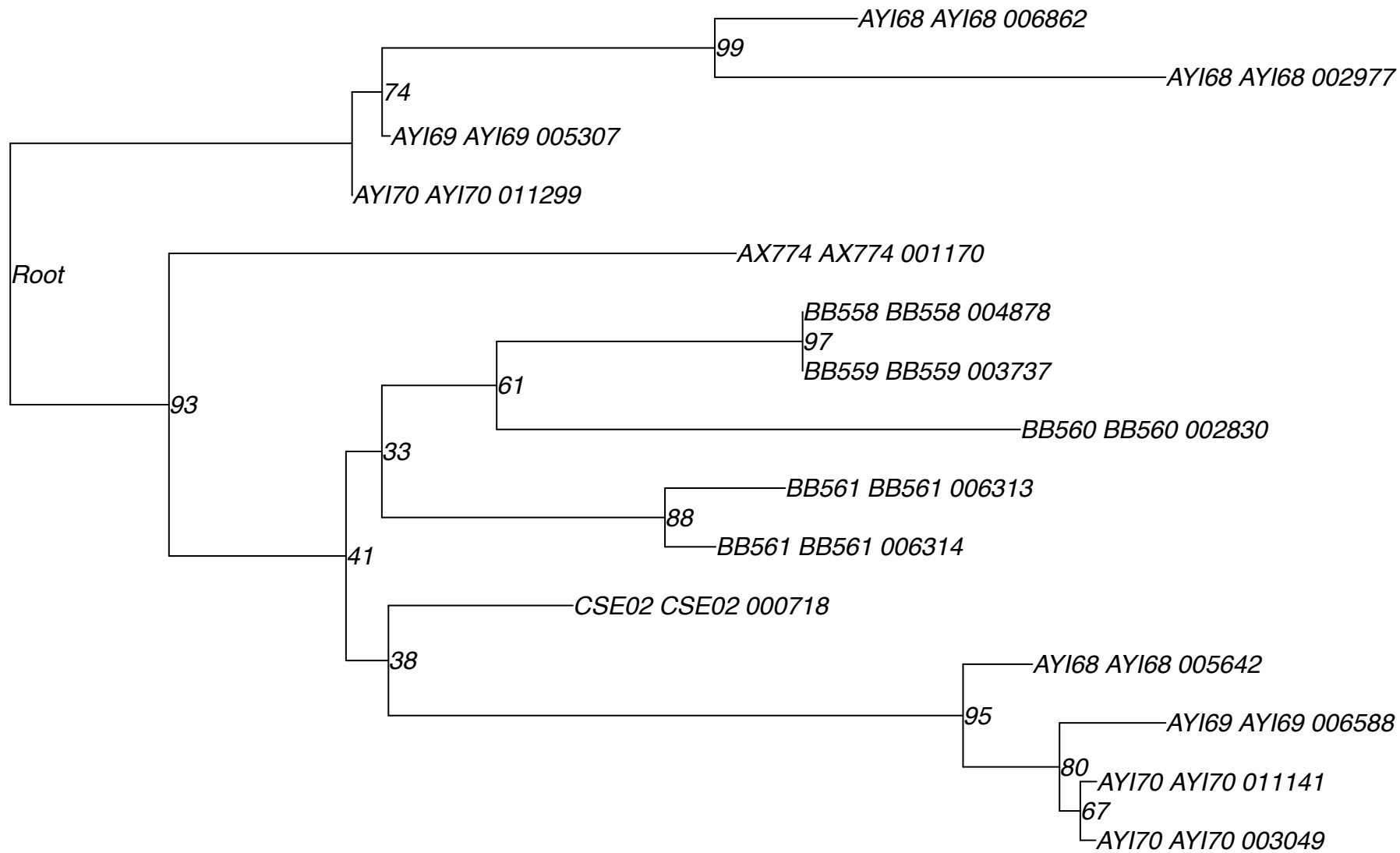

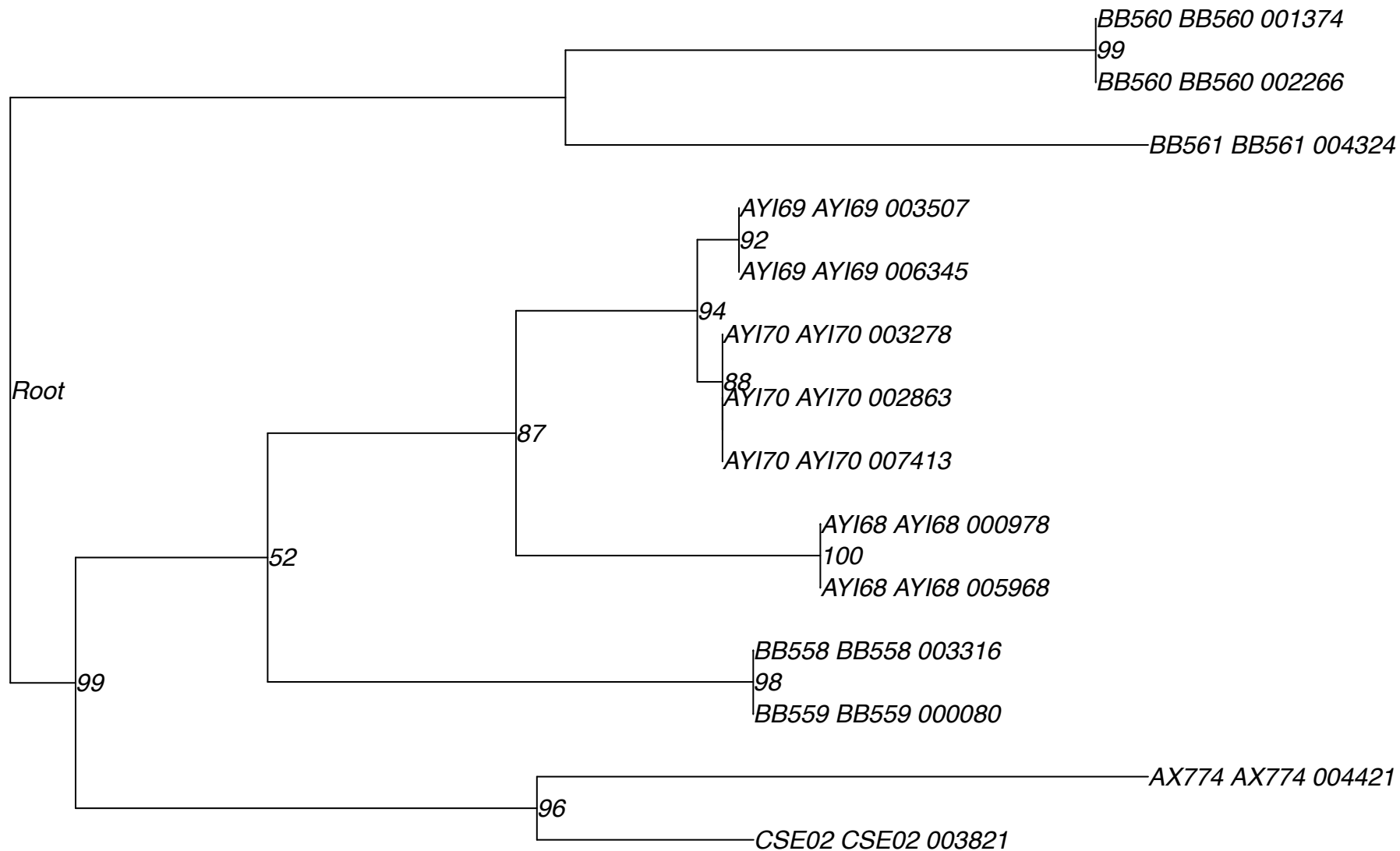

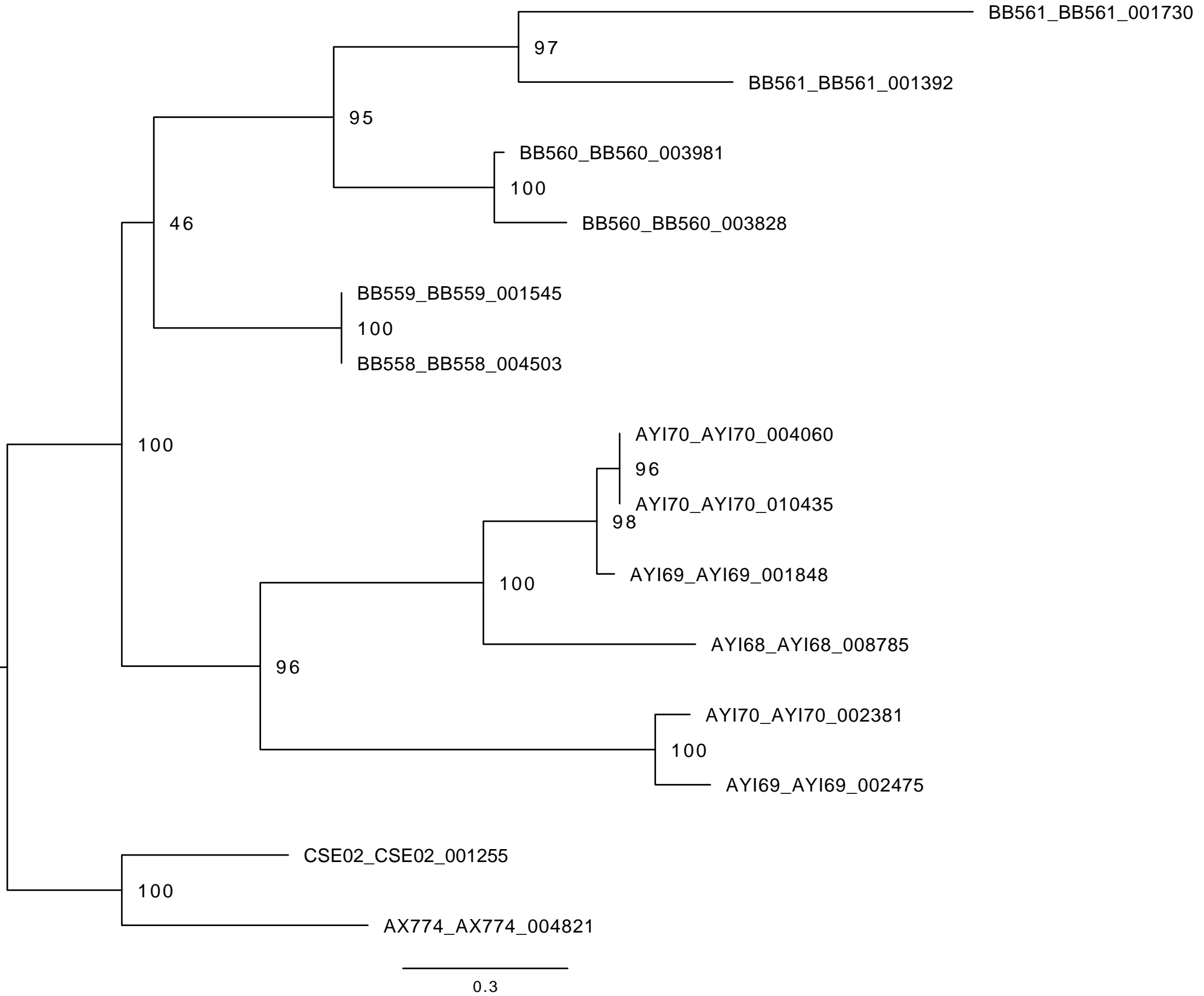

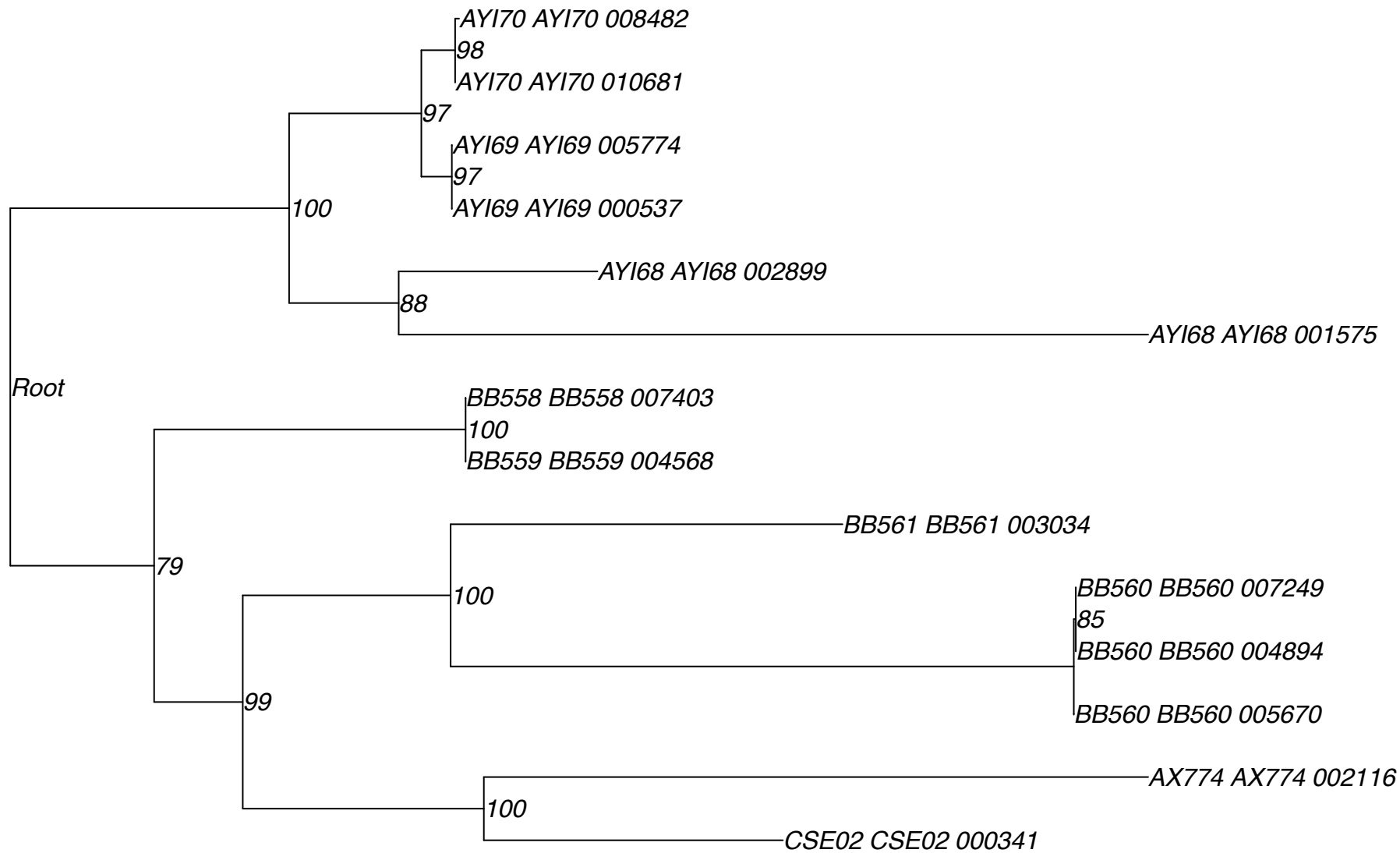

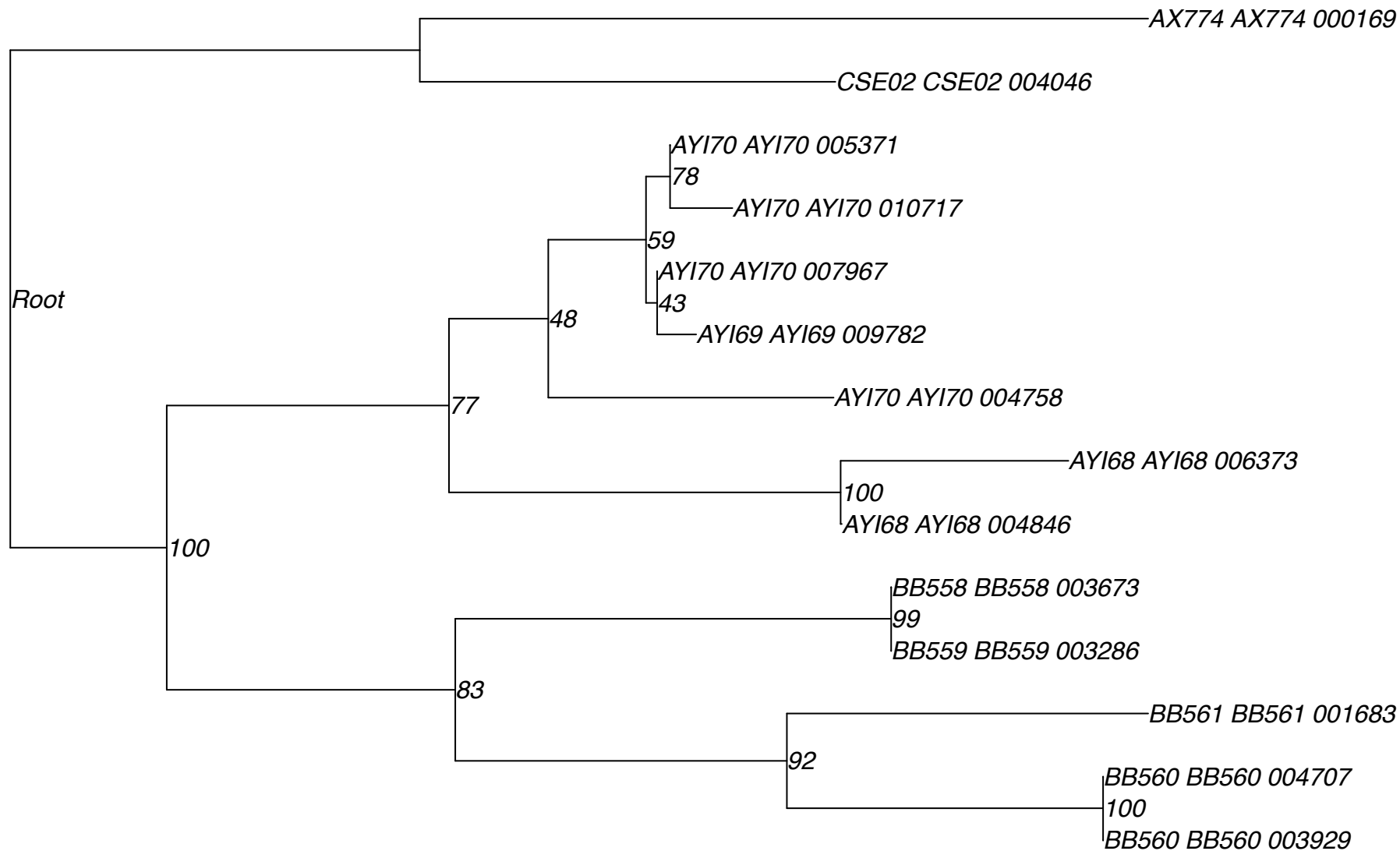

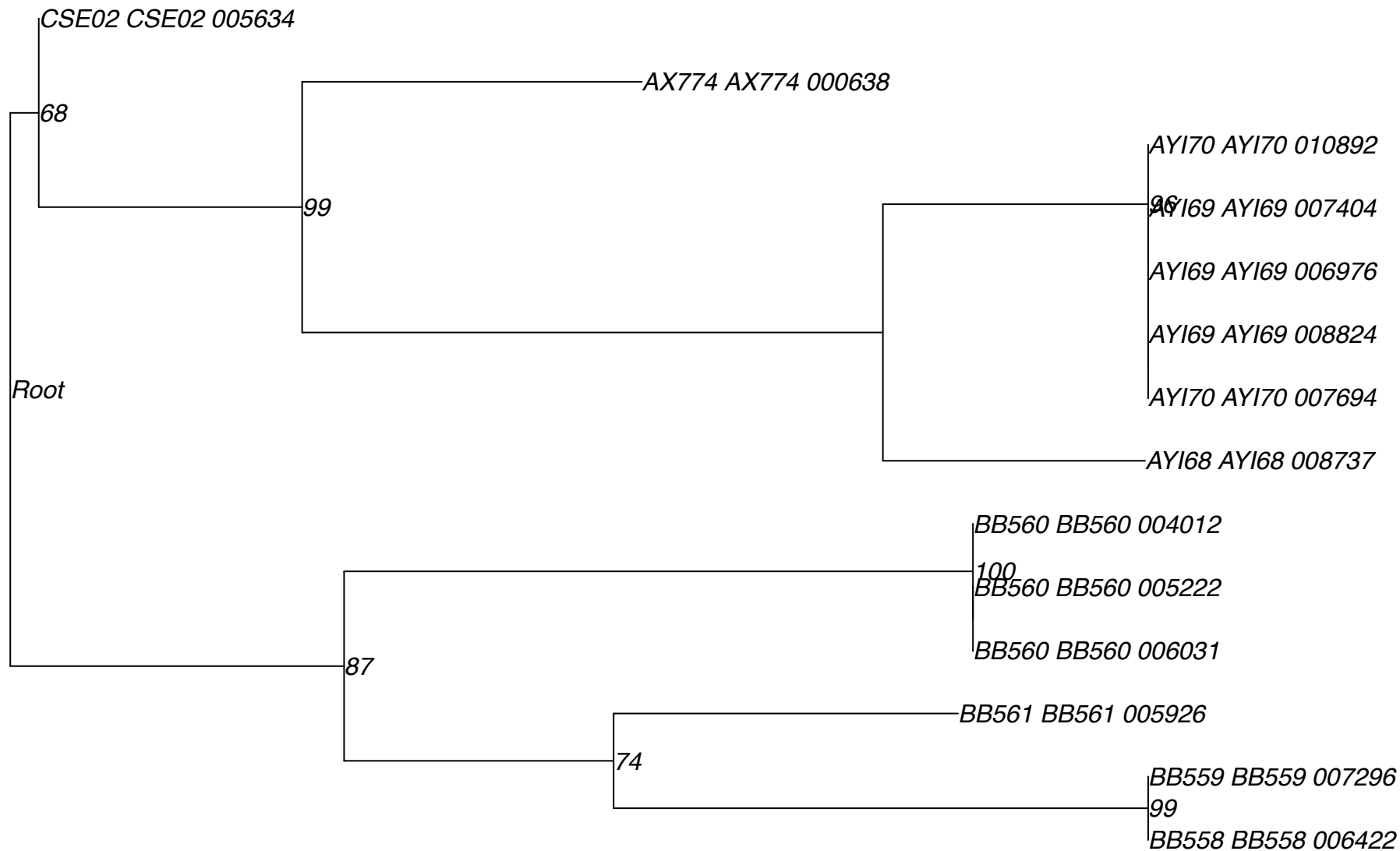

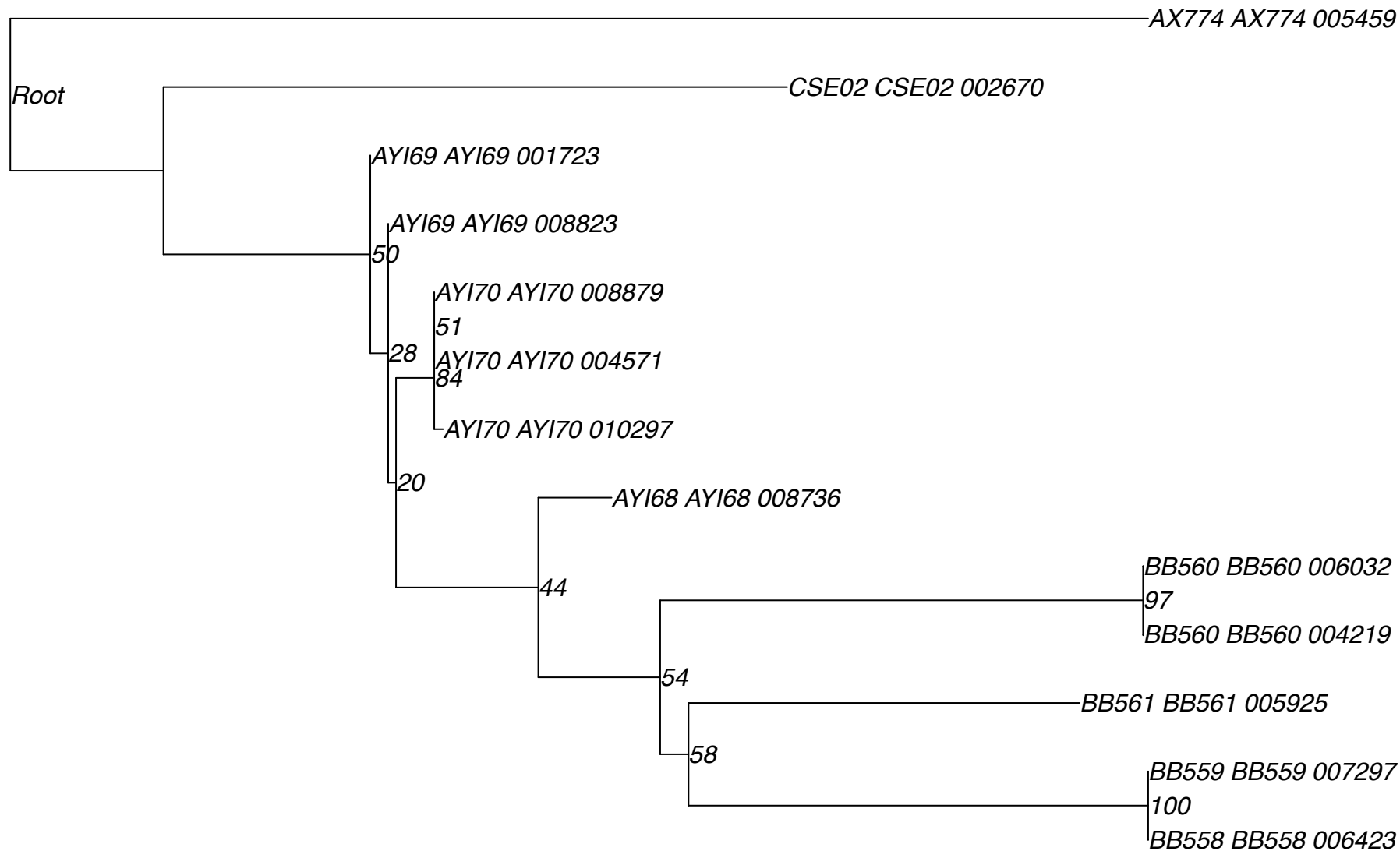

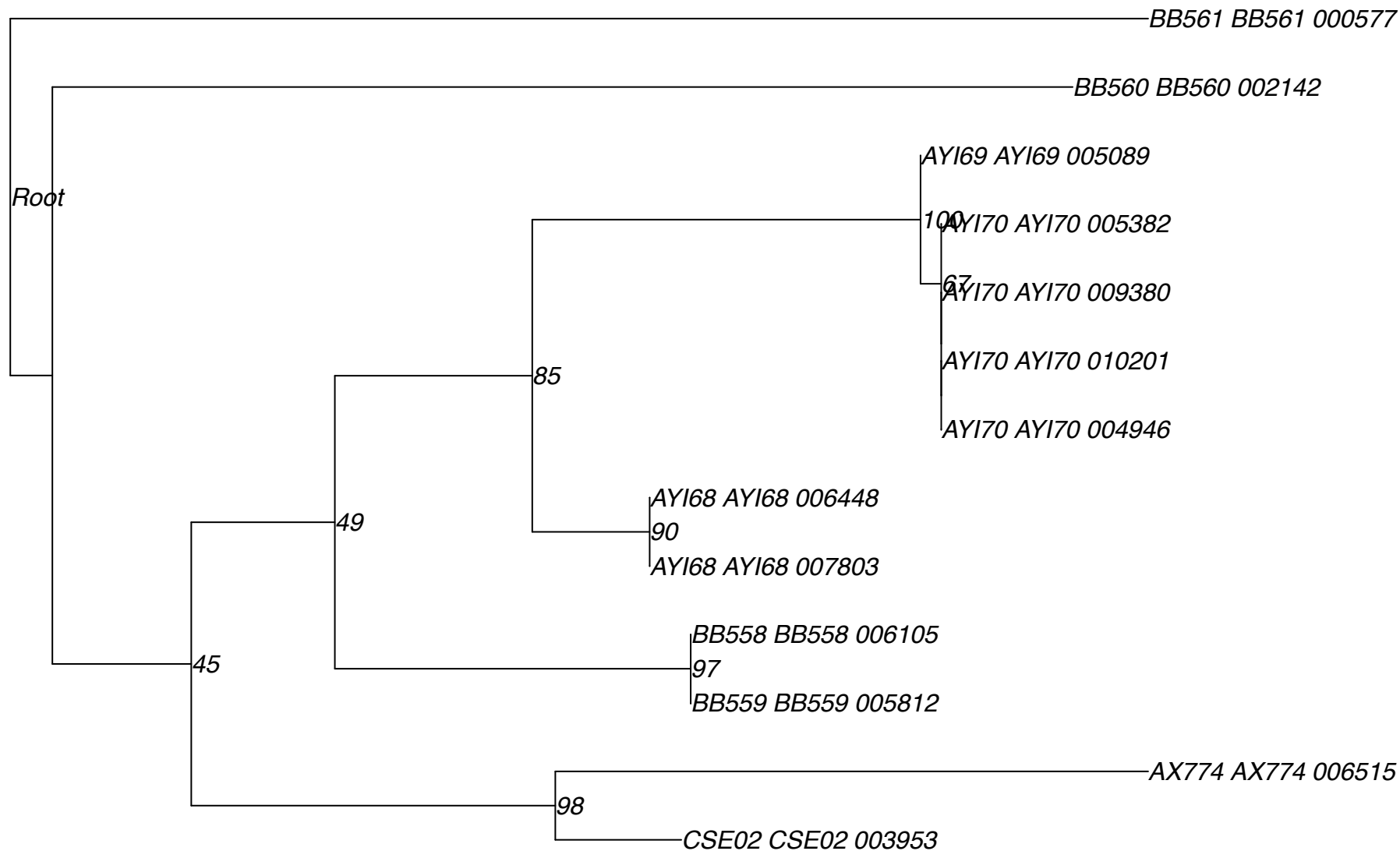

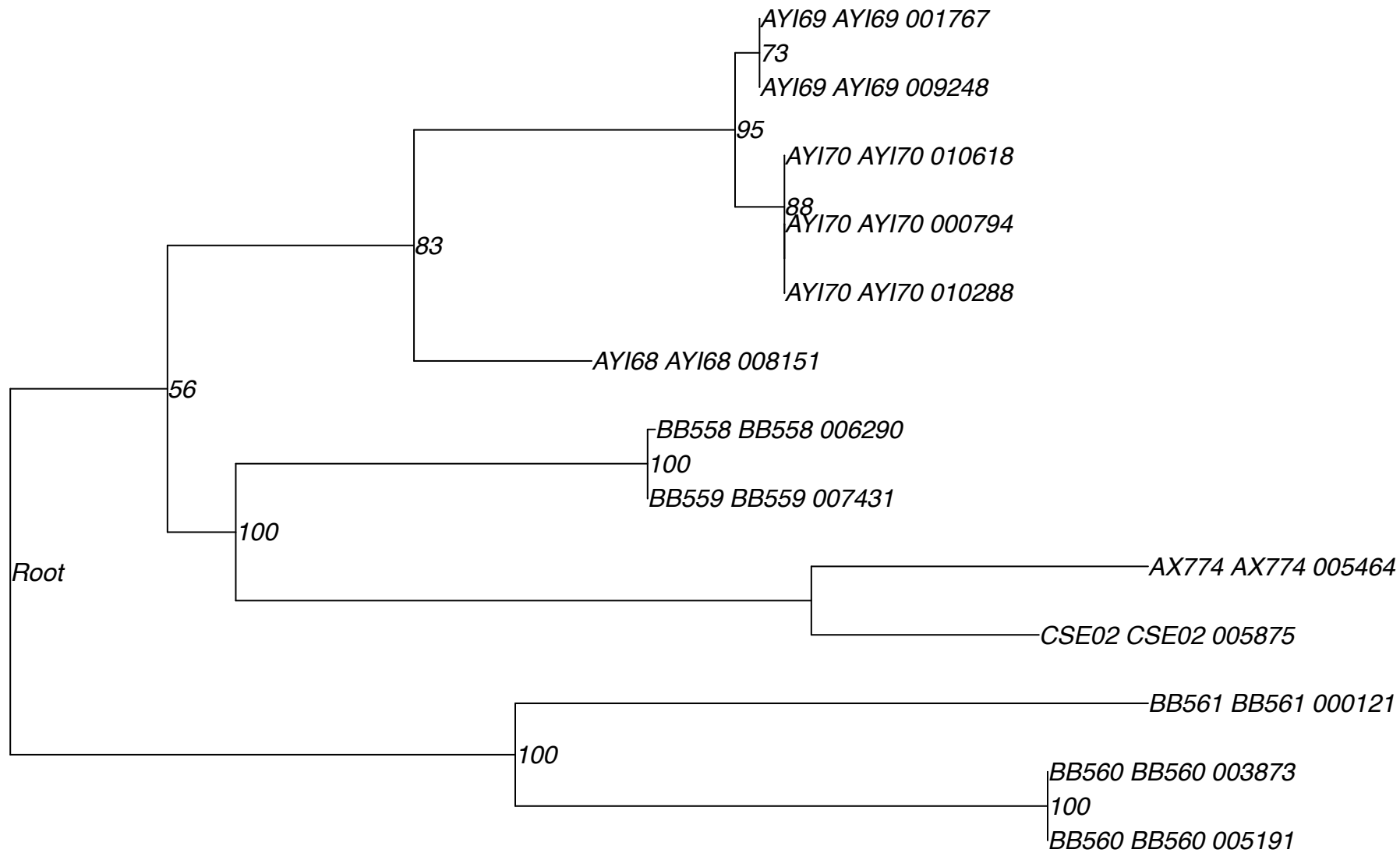

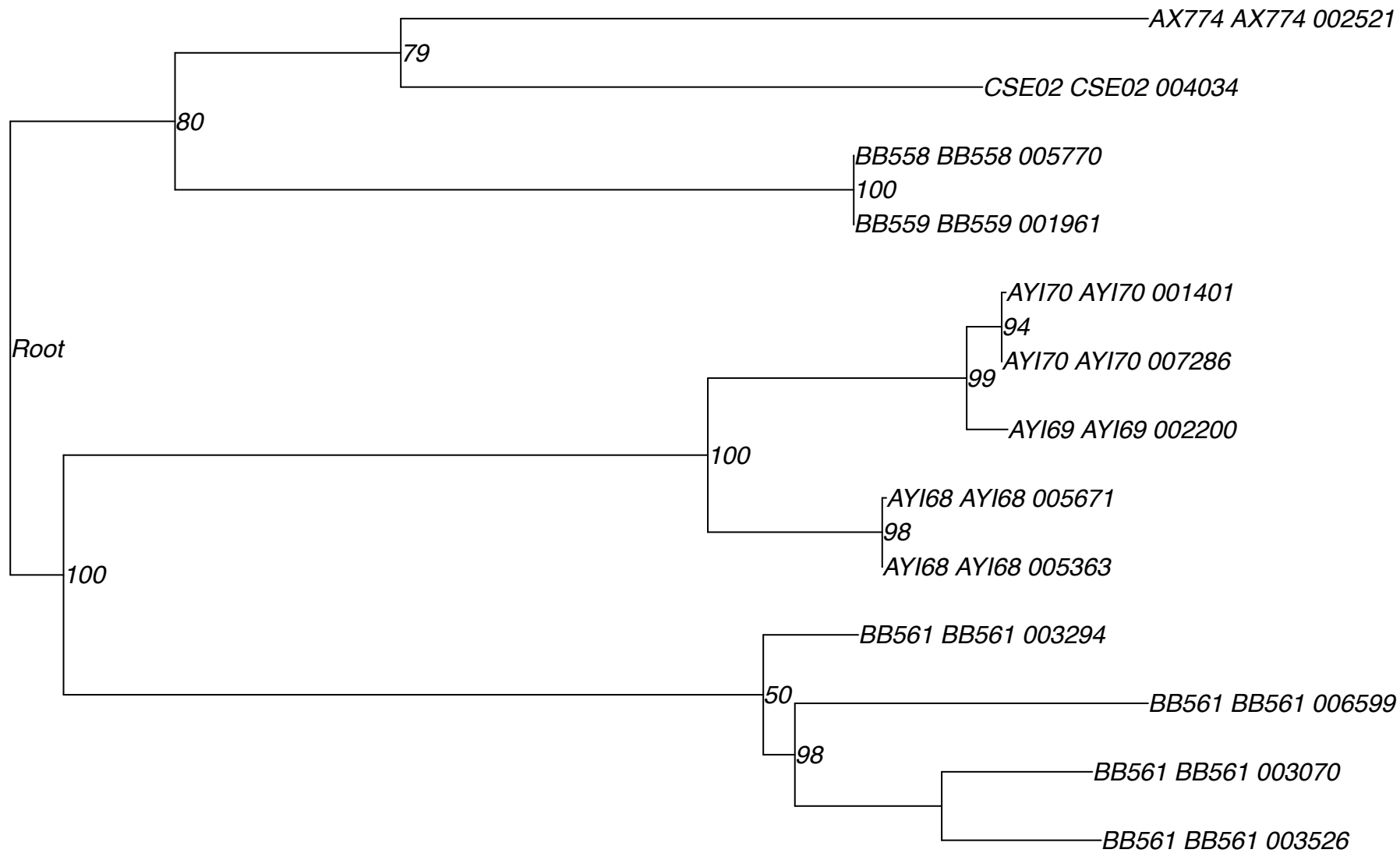

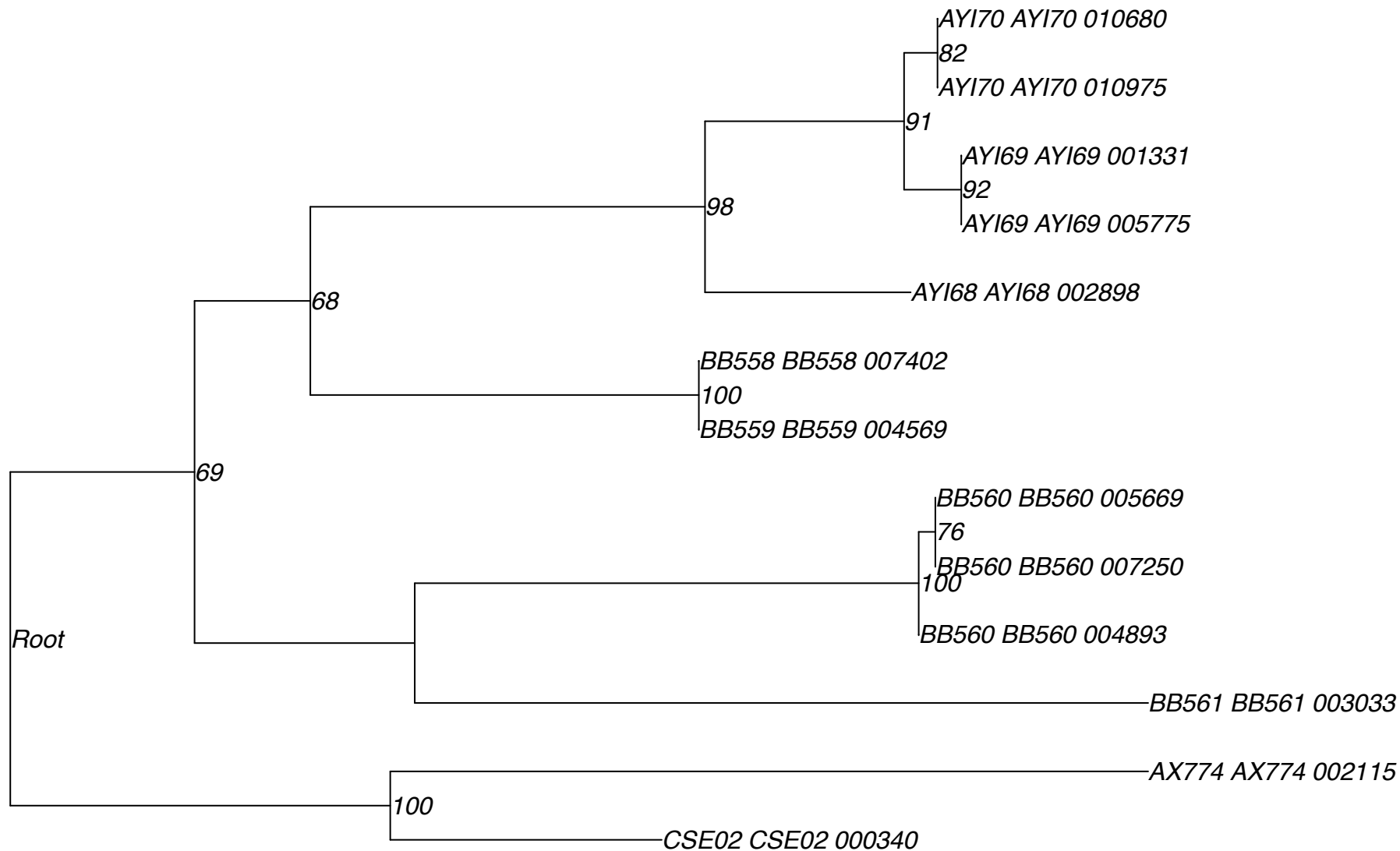

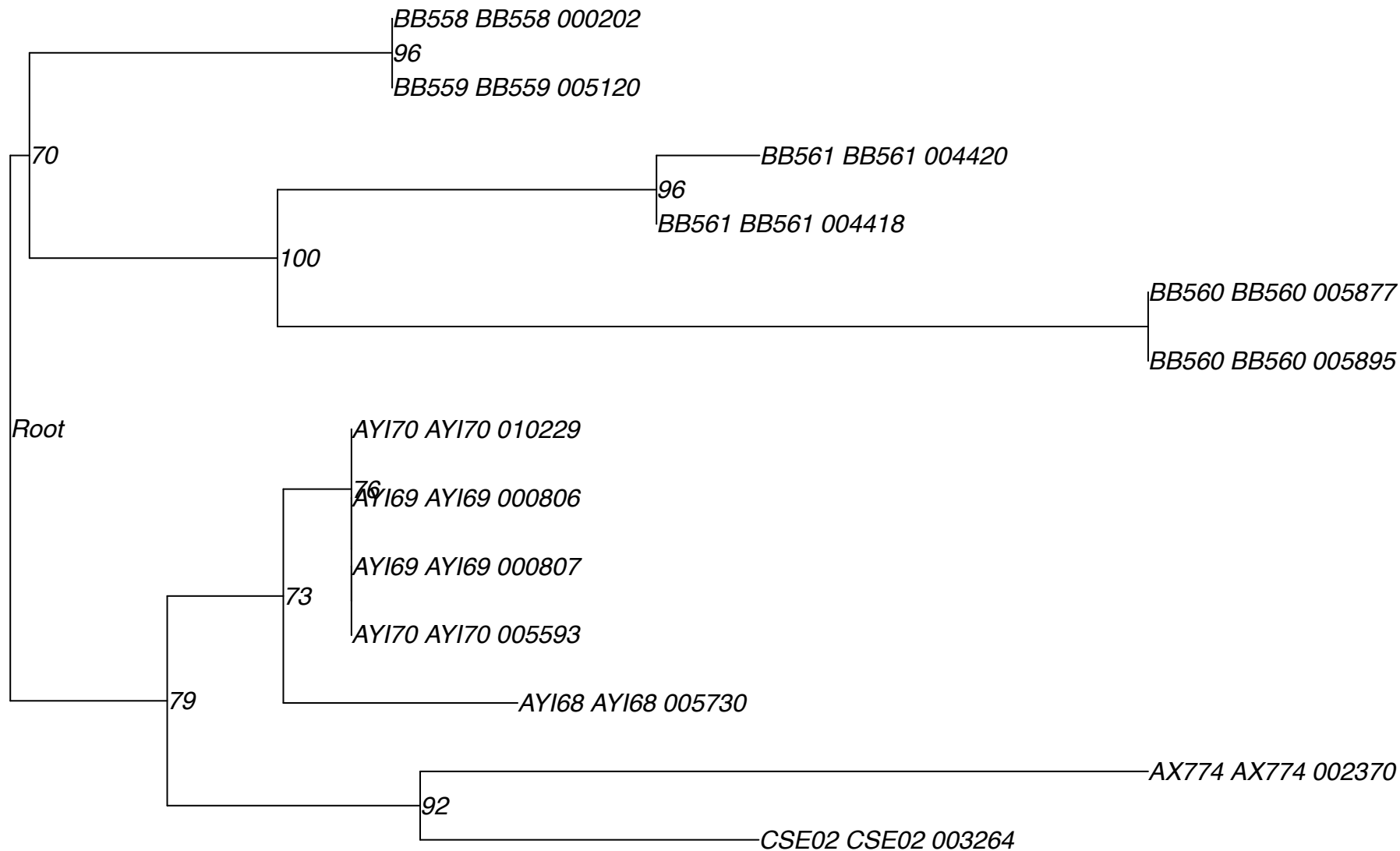

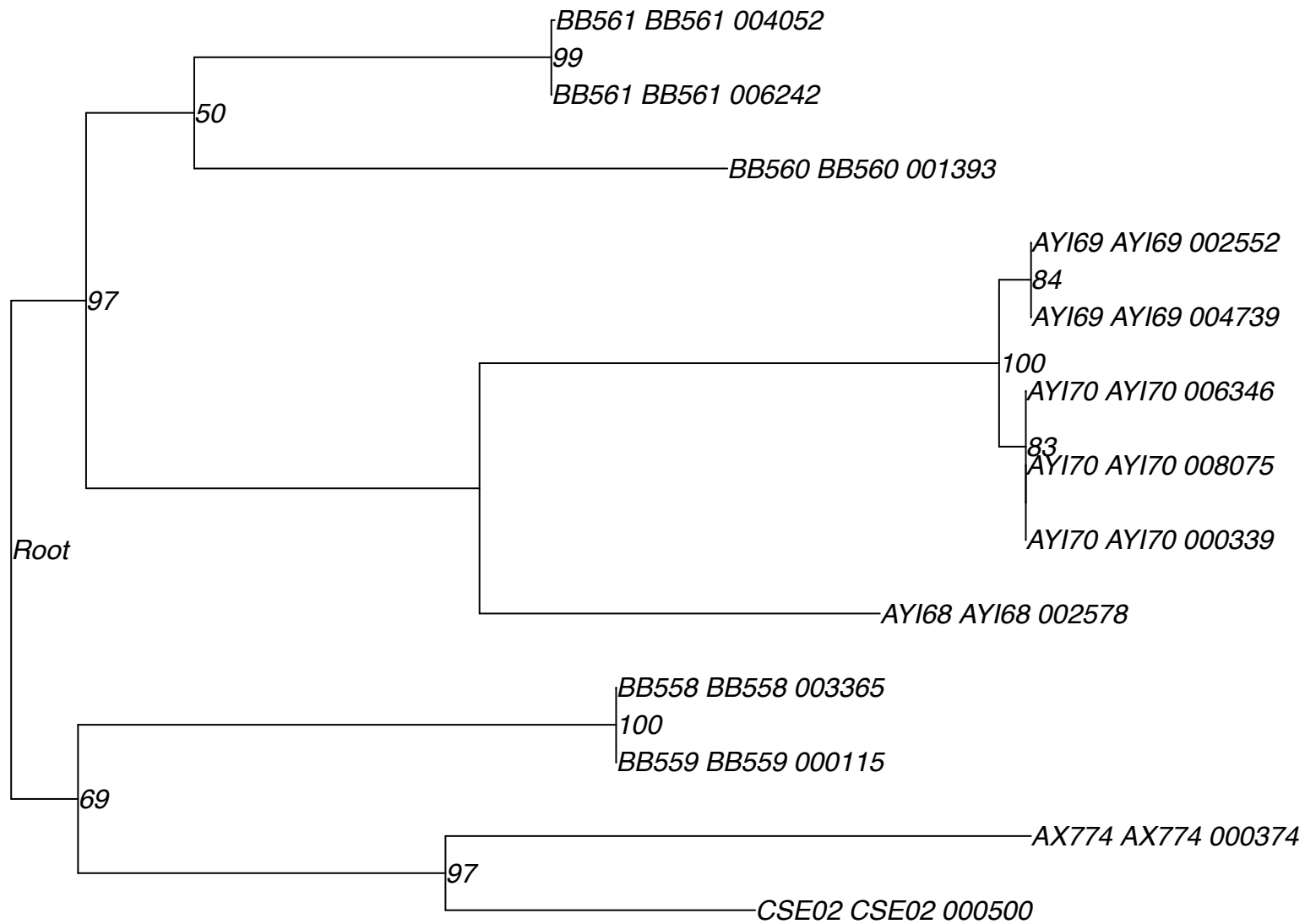

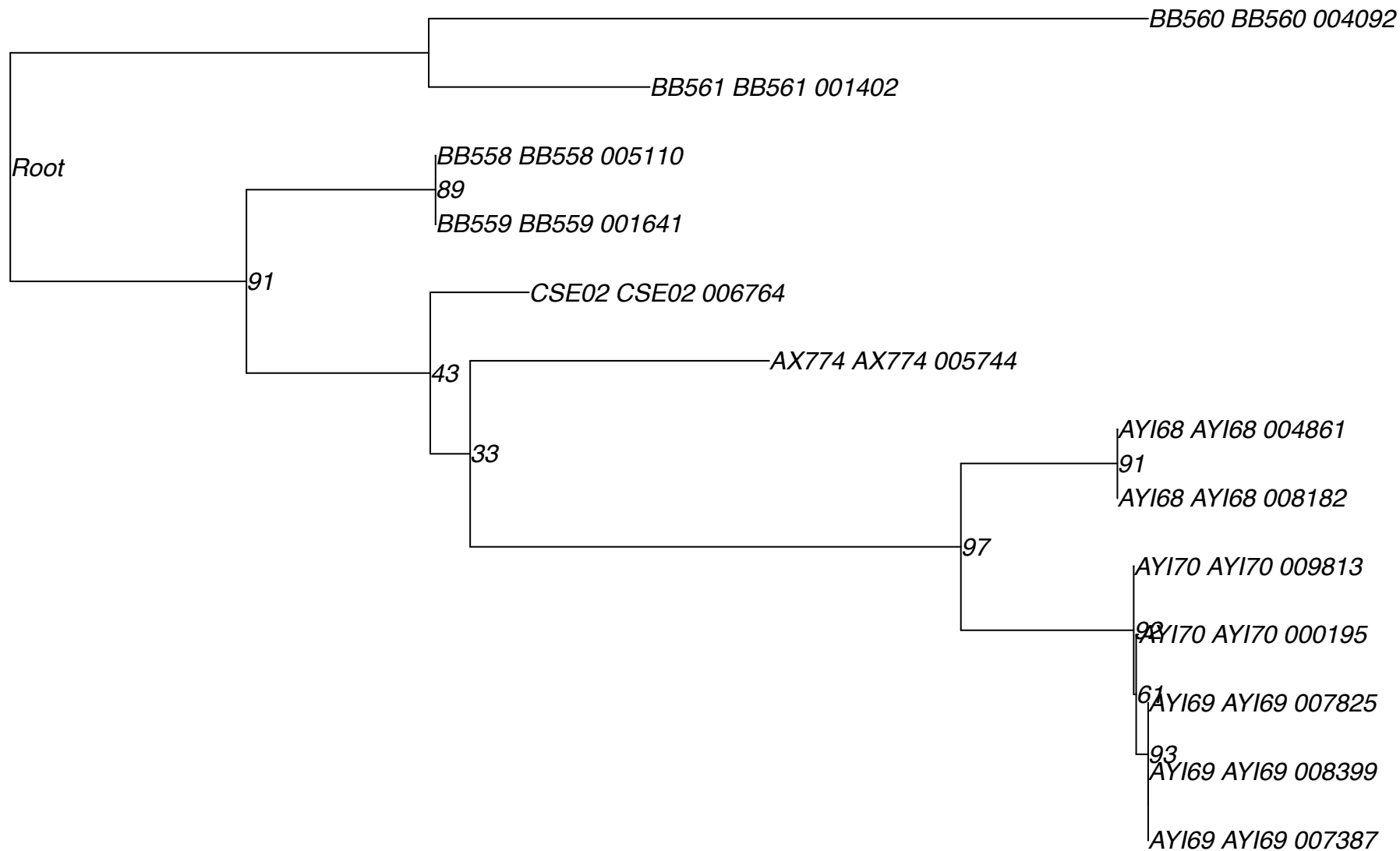

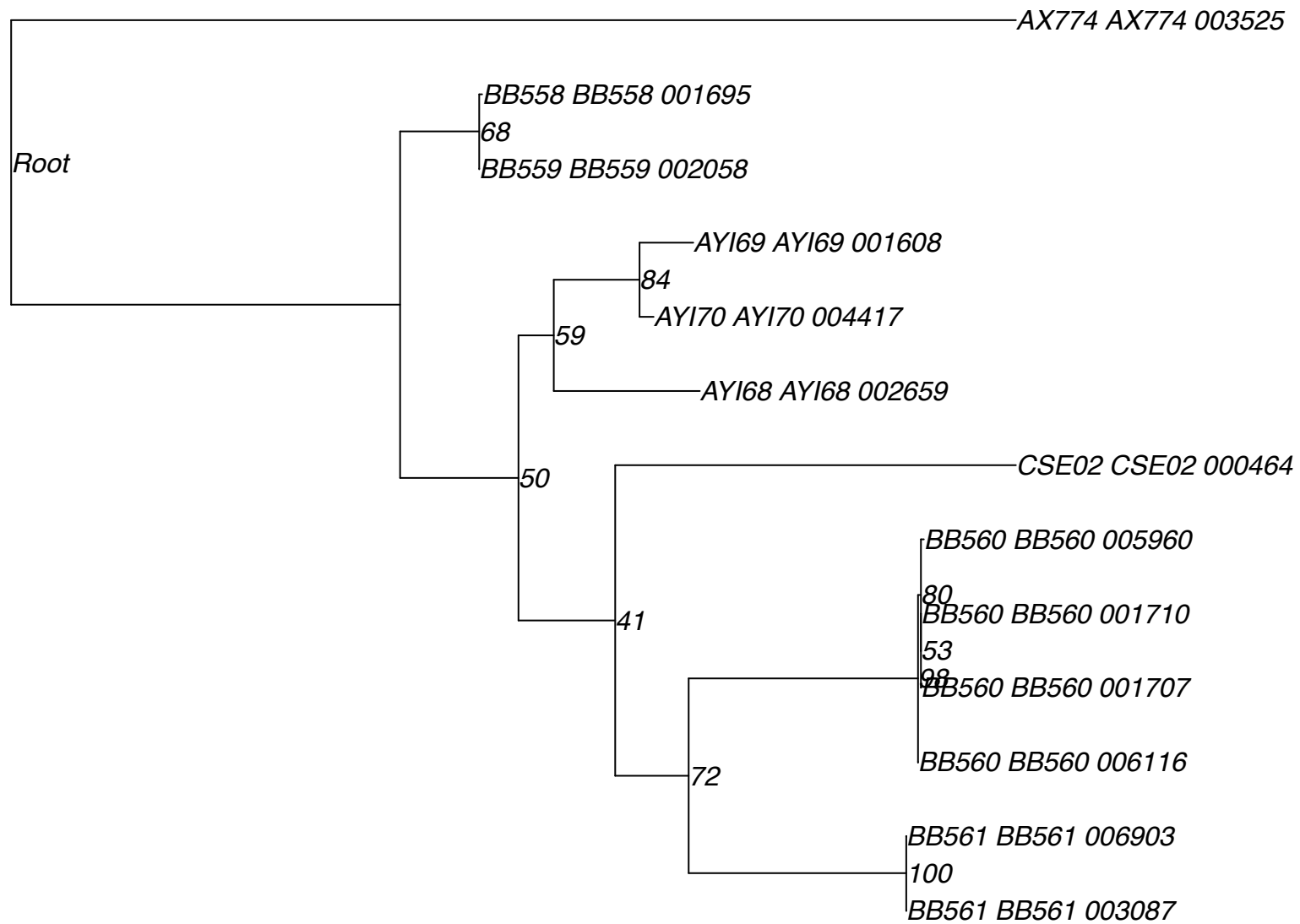

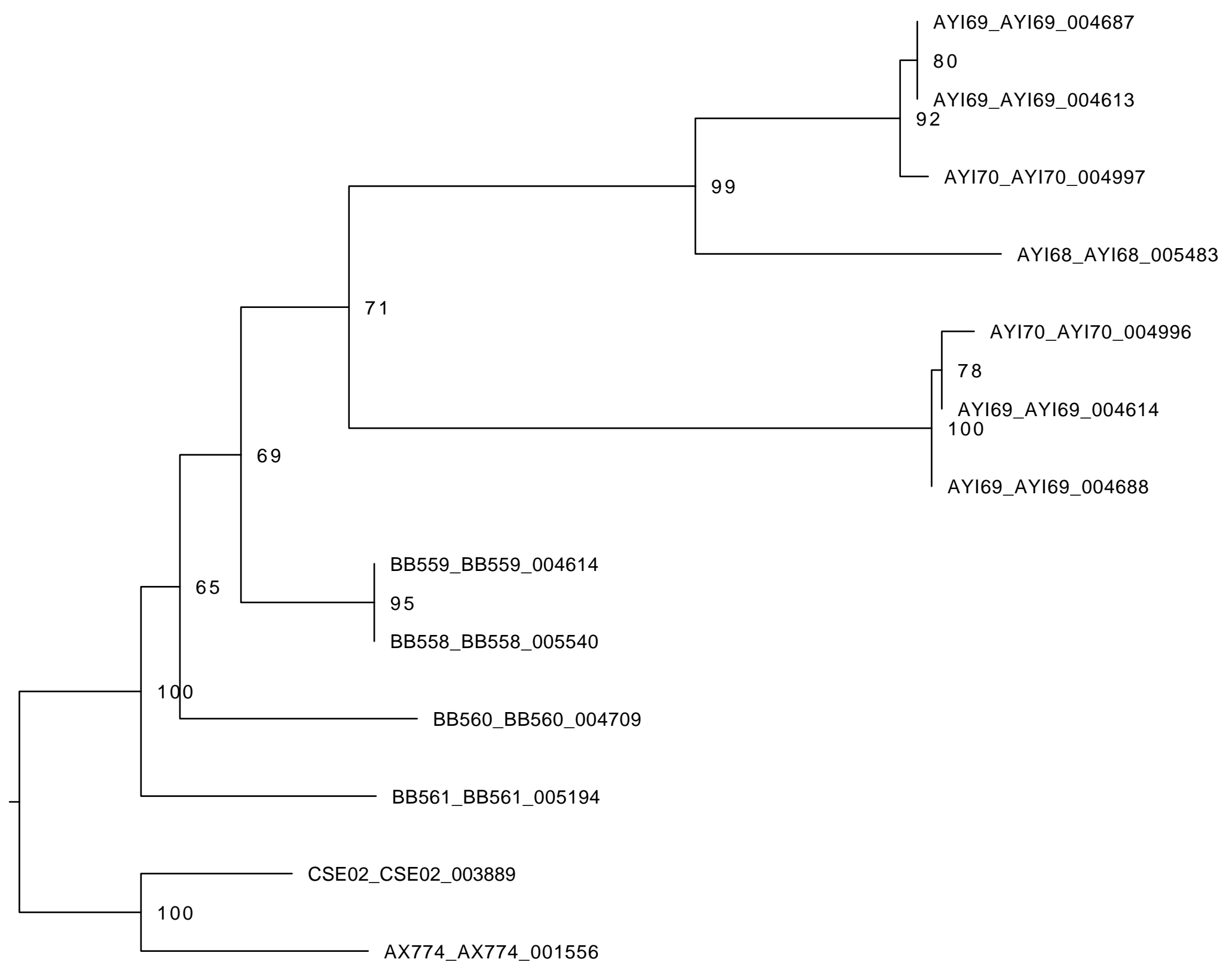

0.2

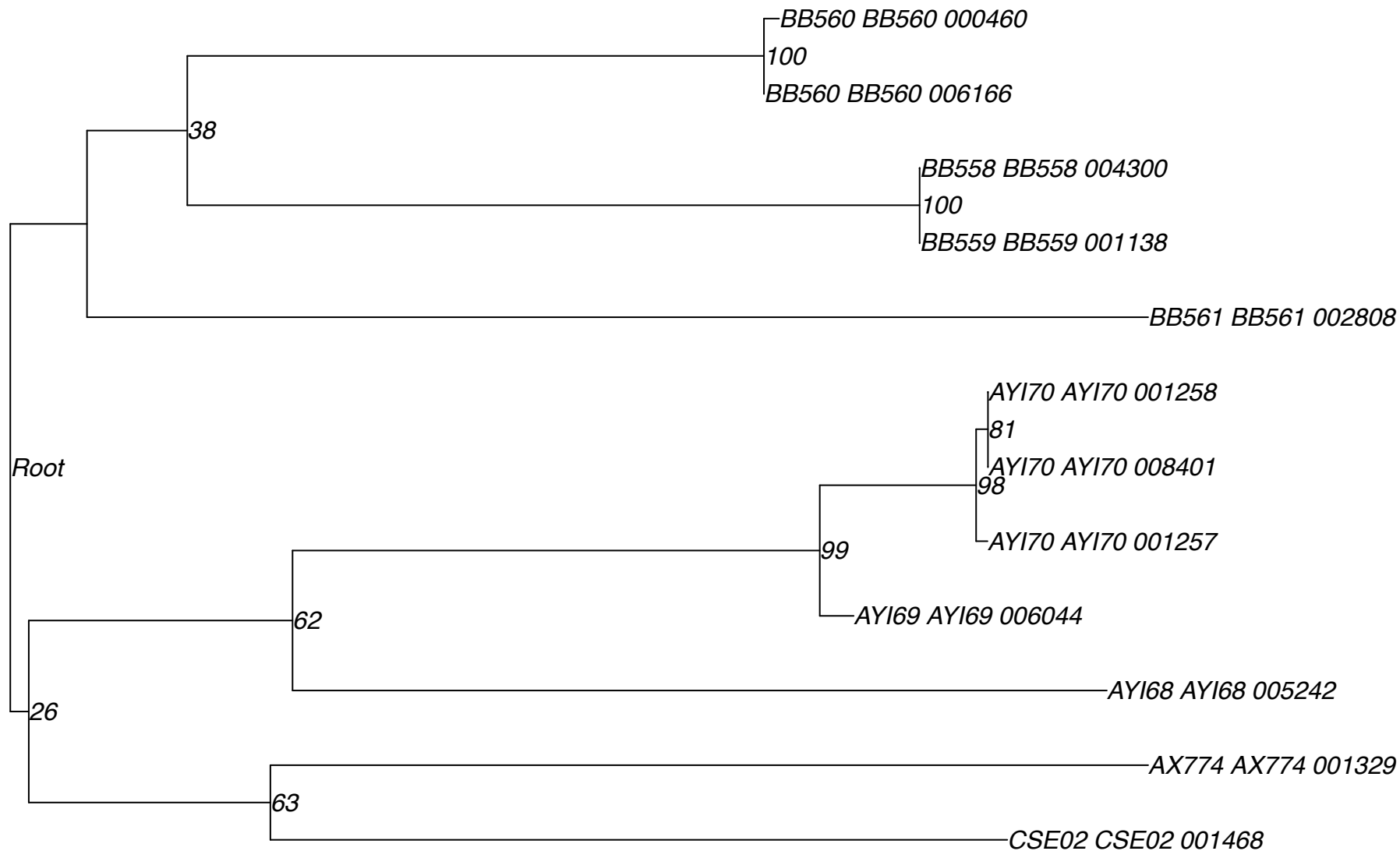

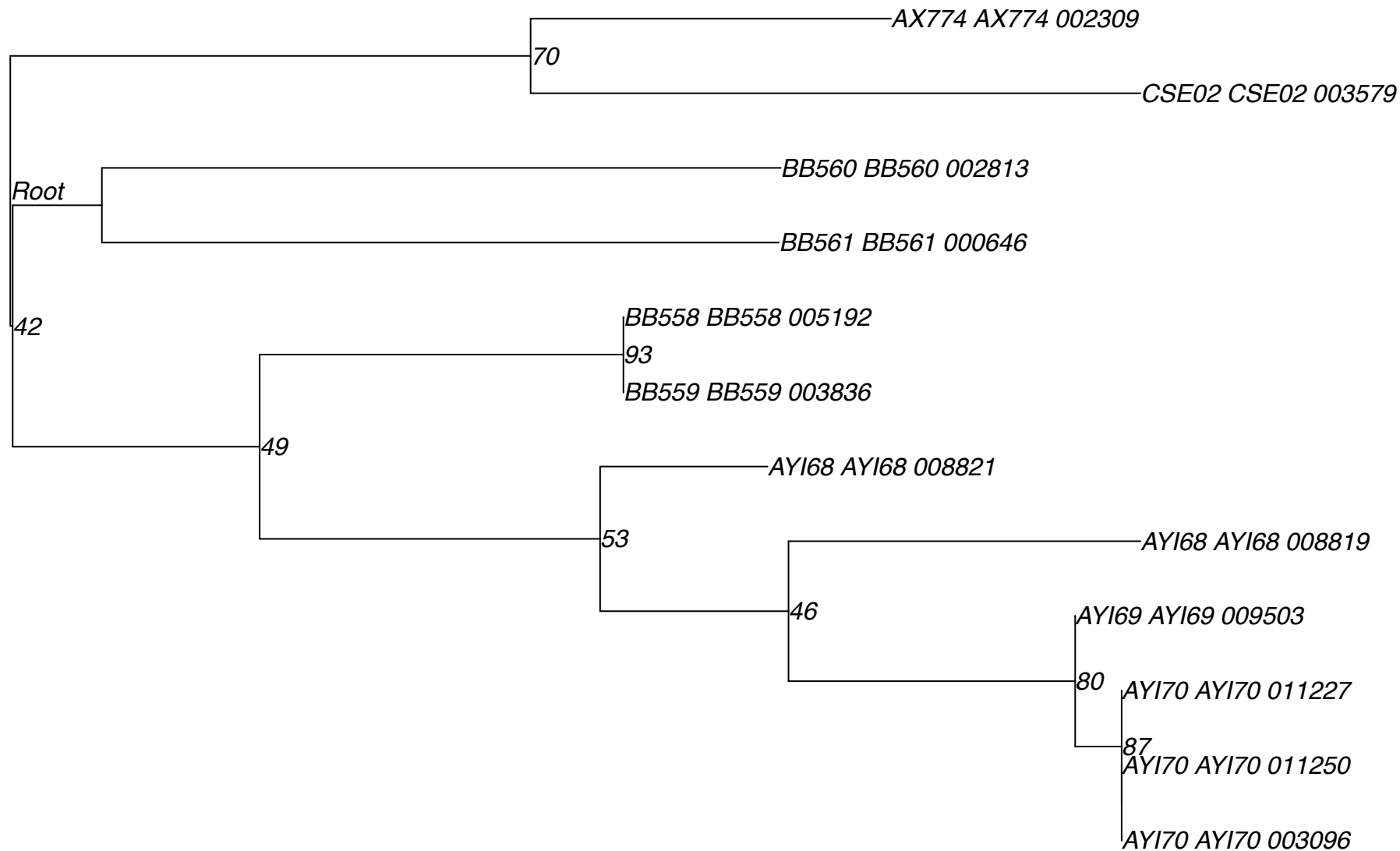

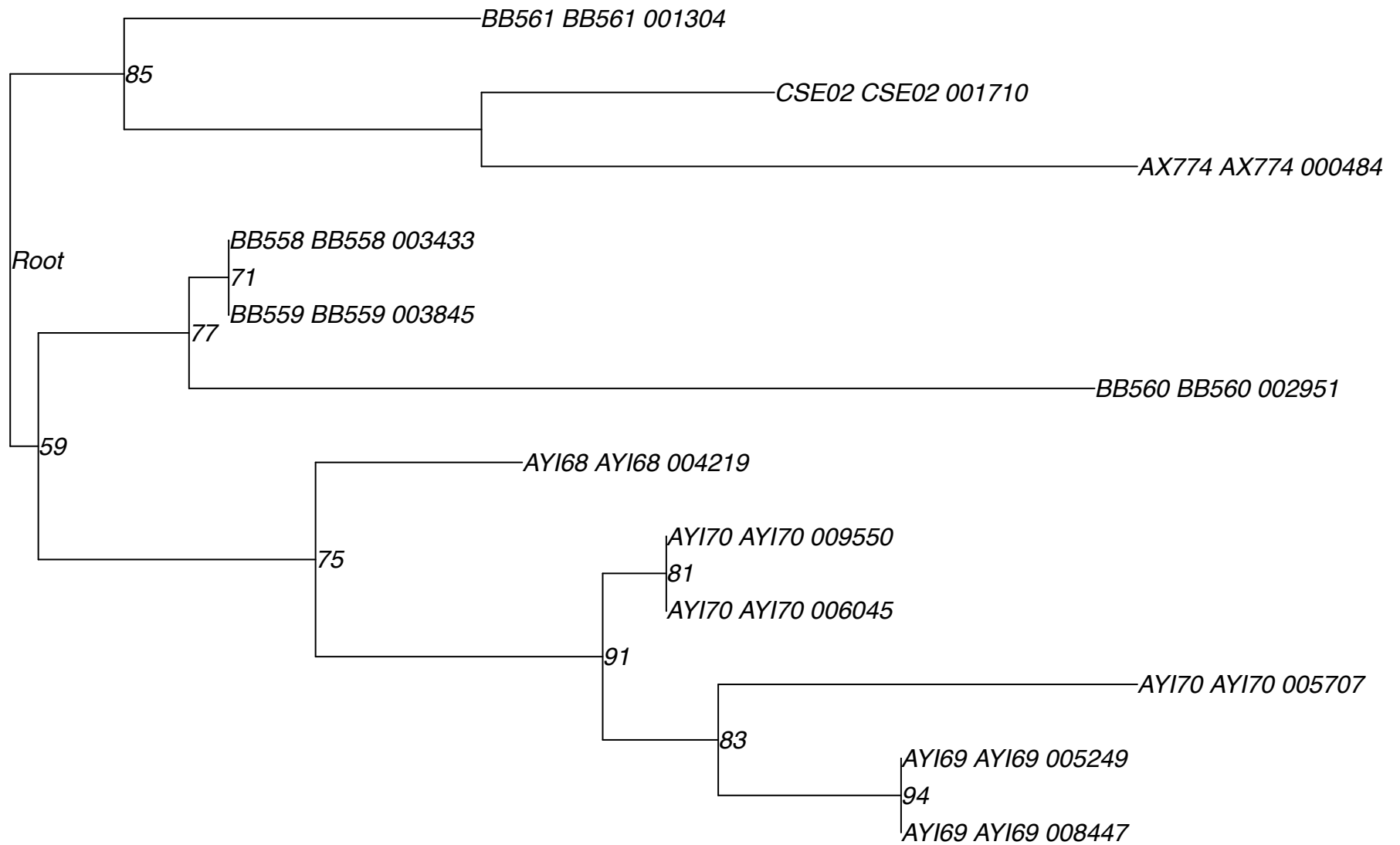

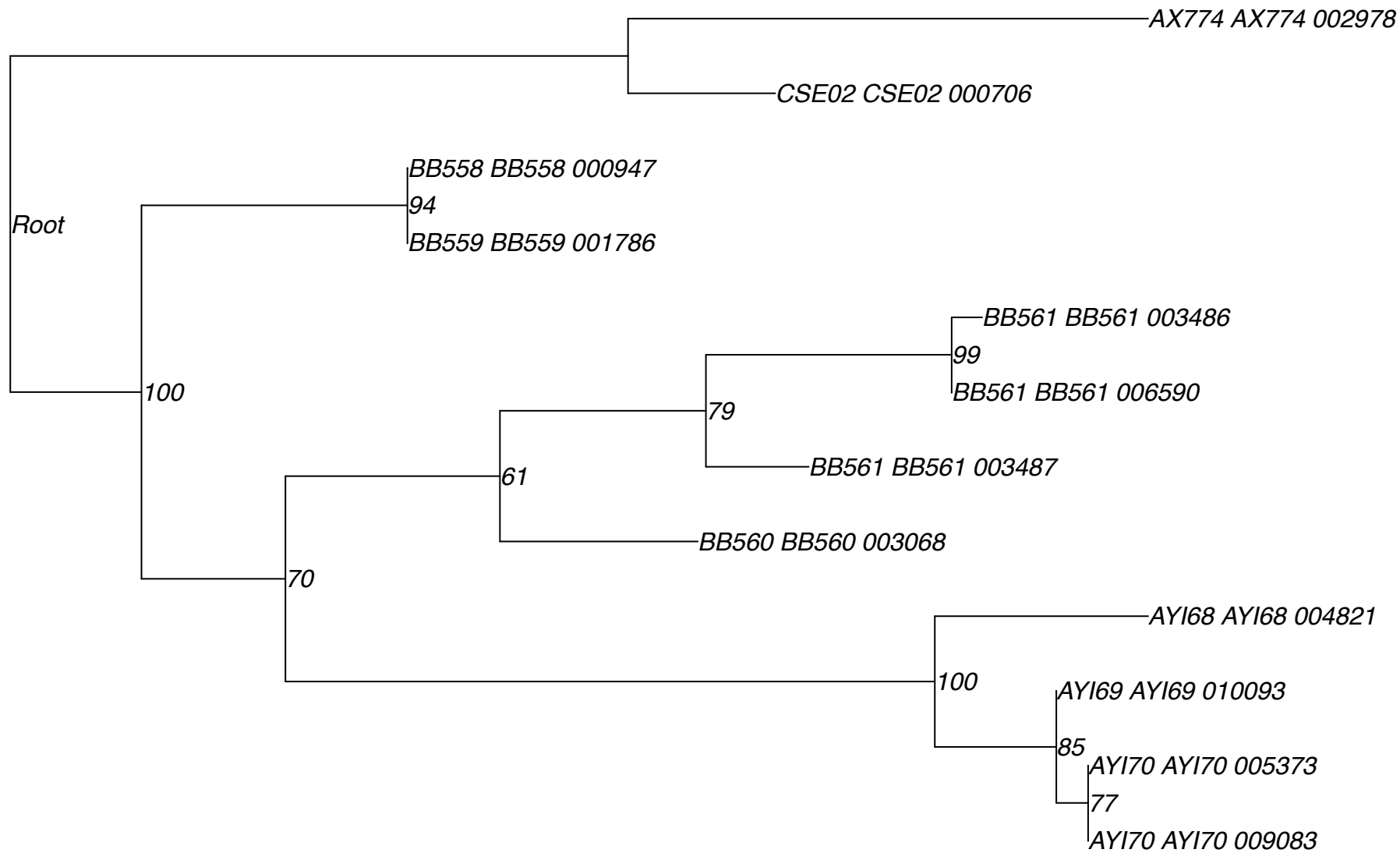

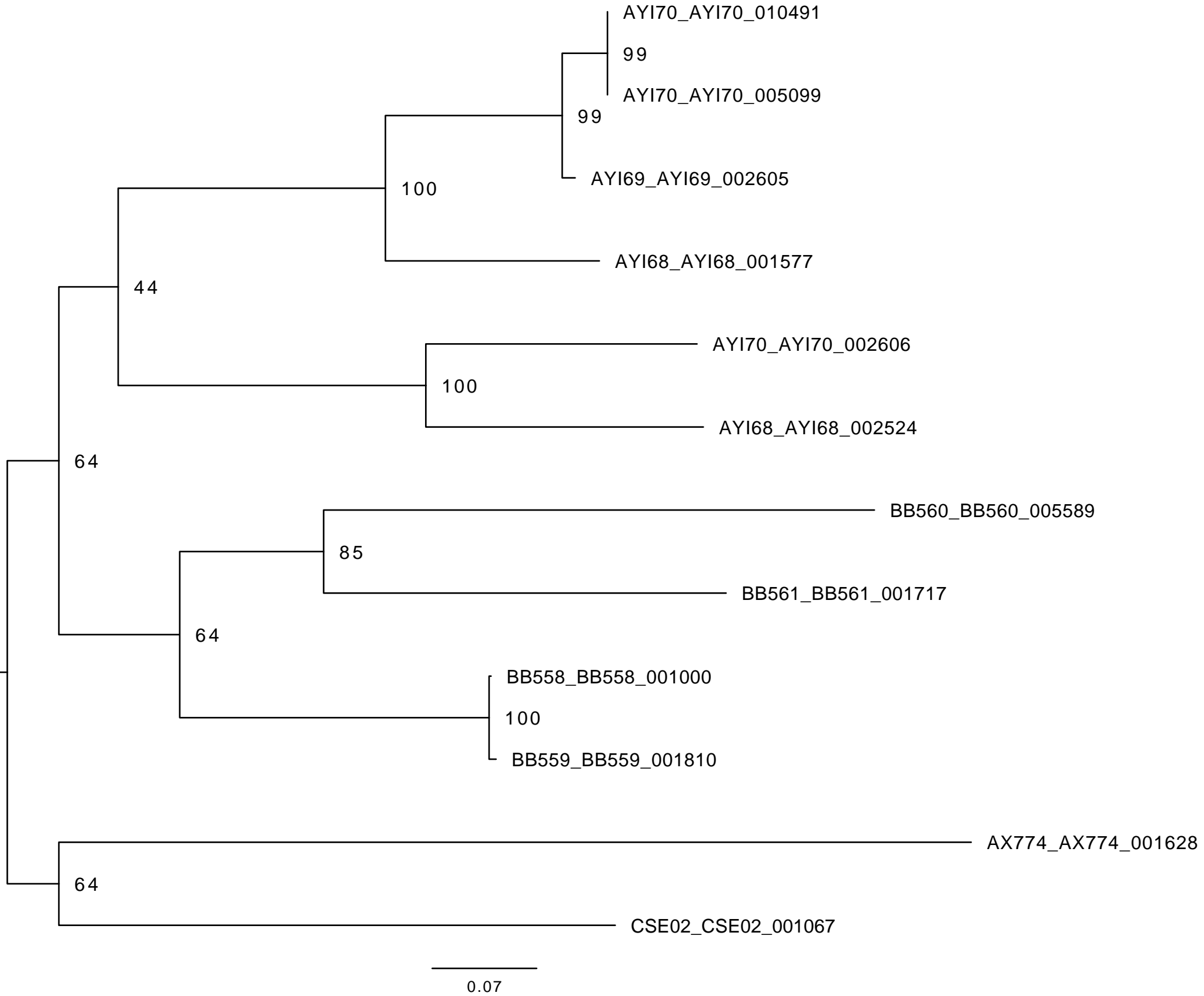

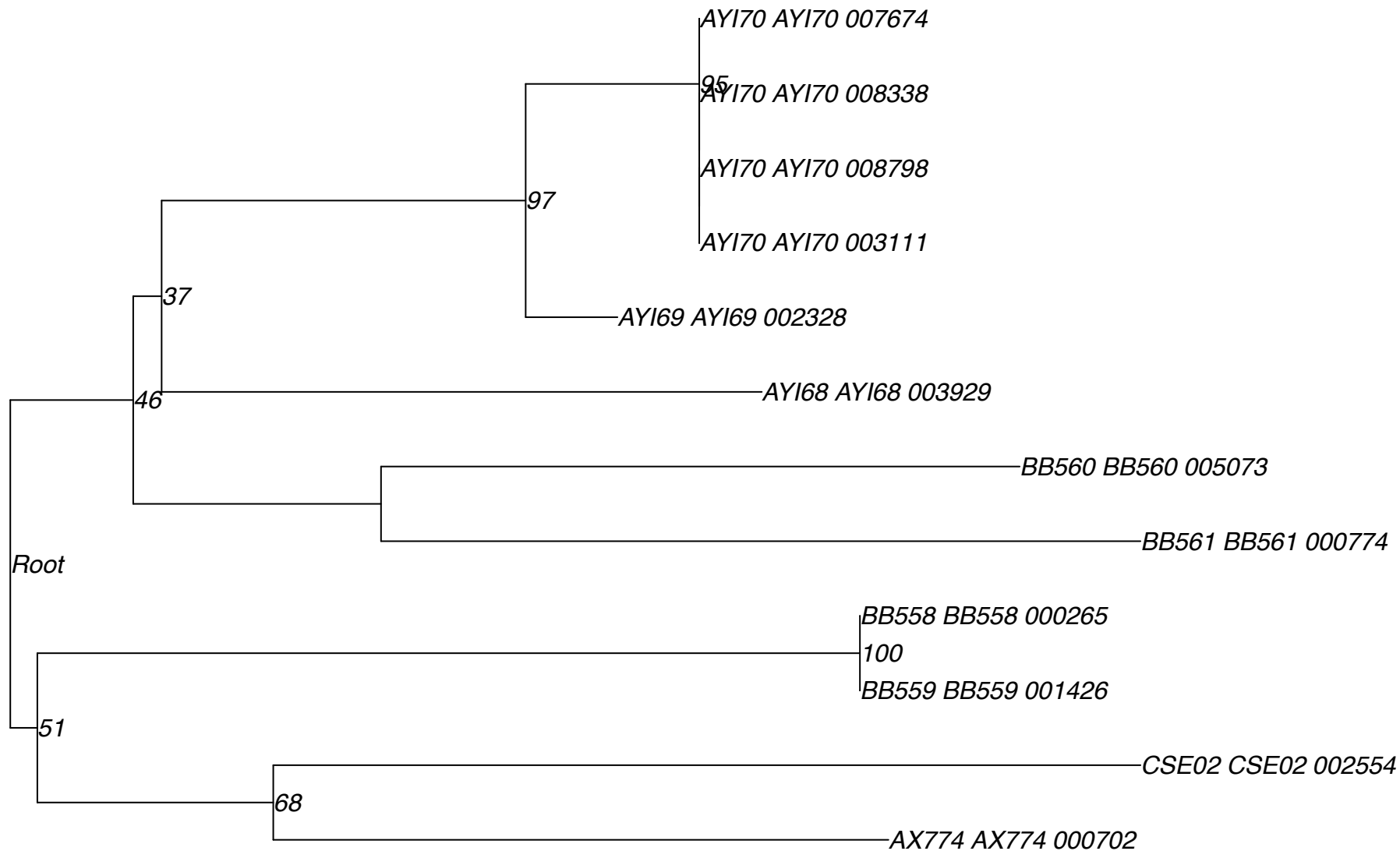

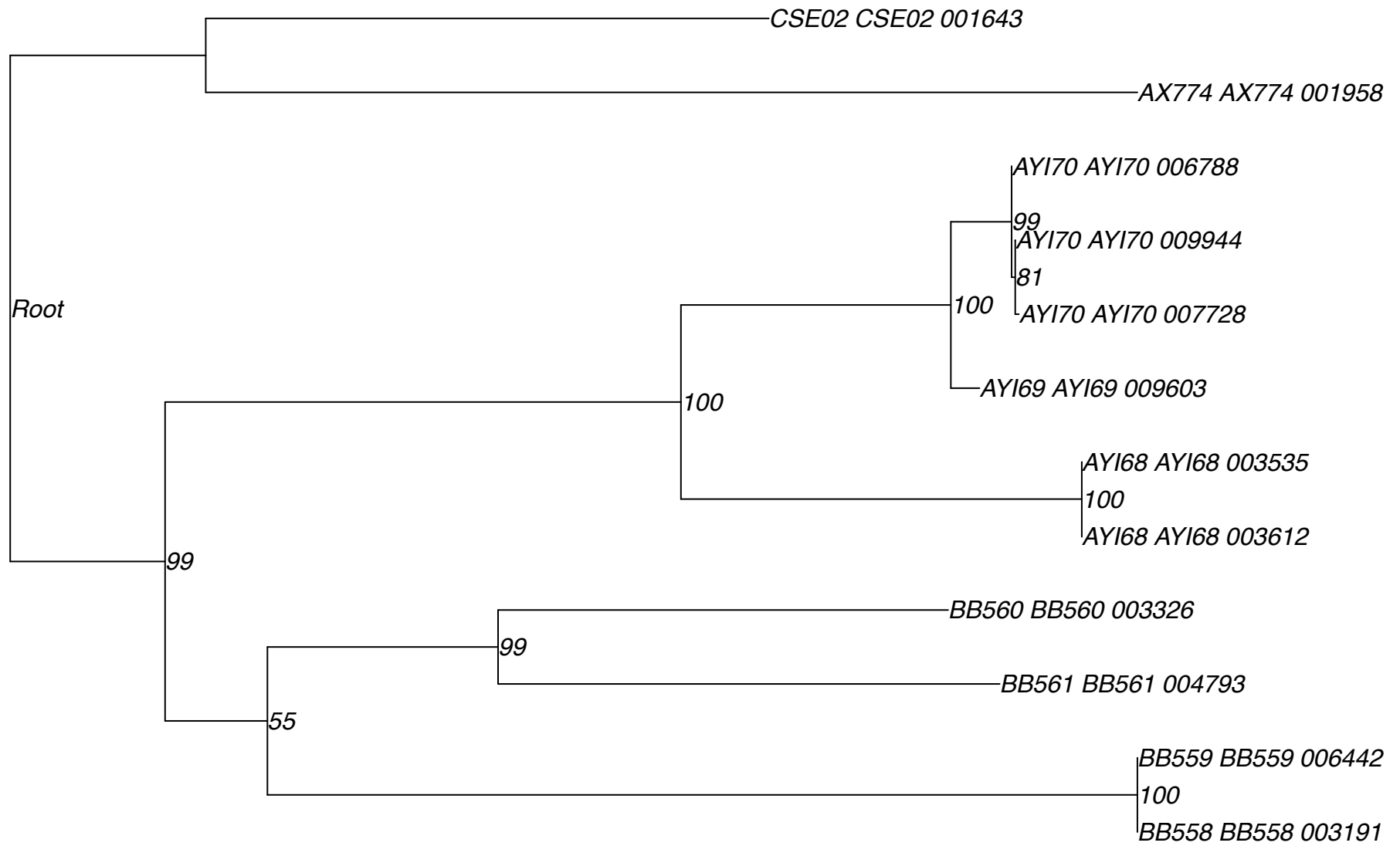

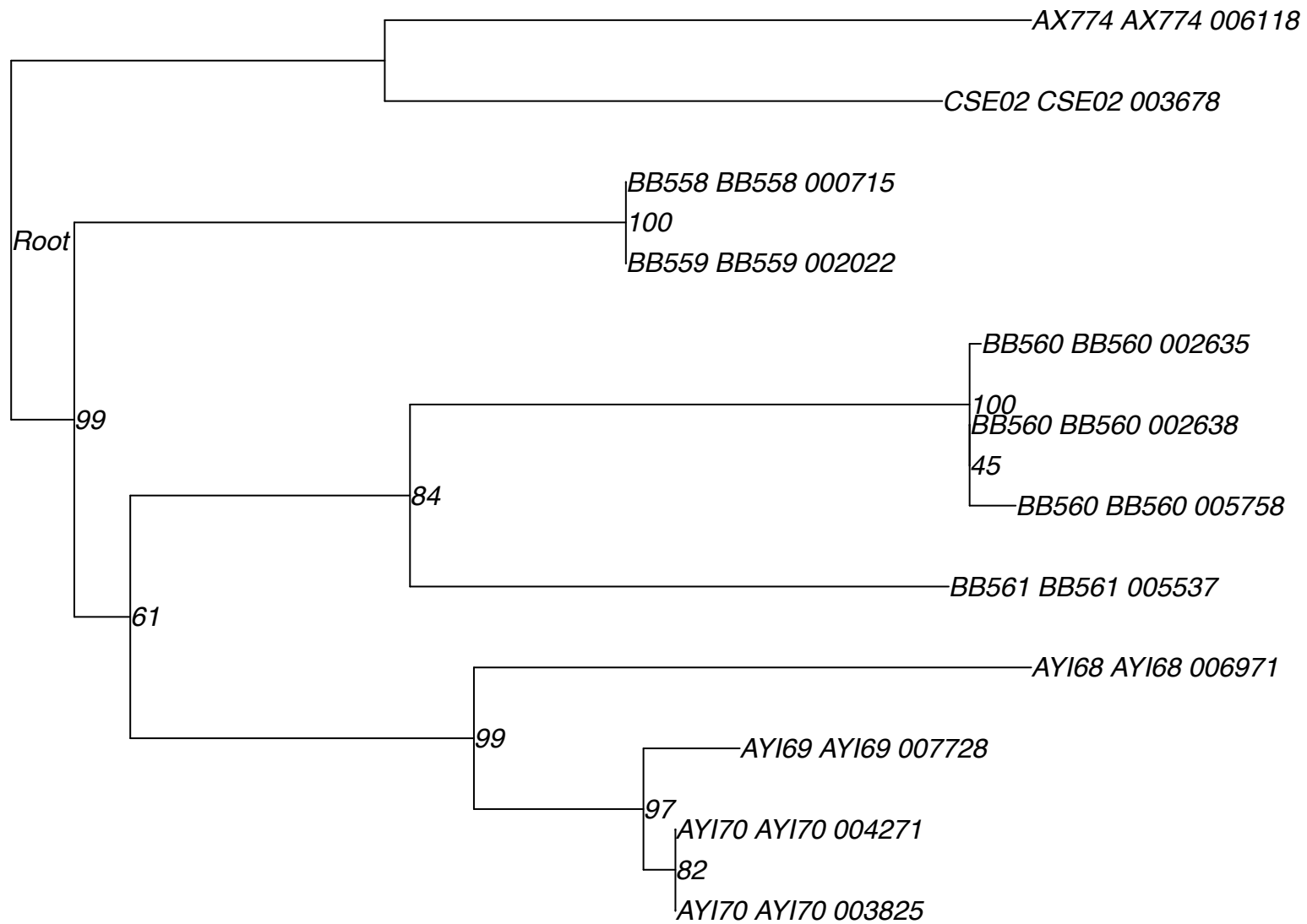

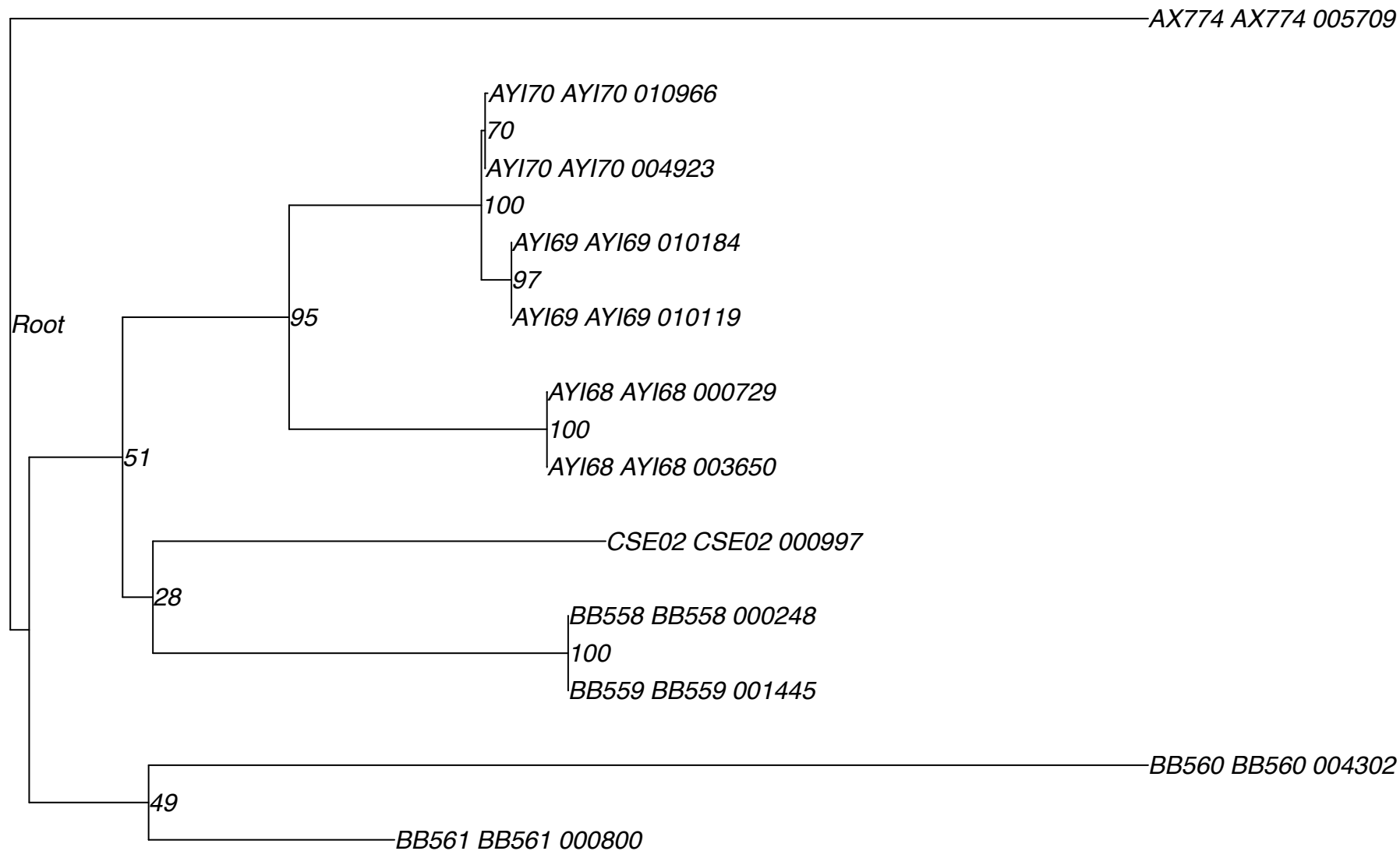

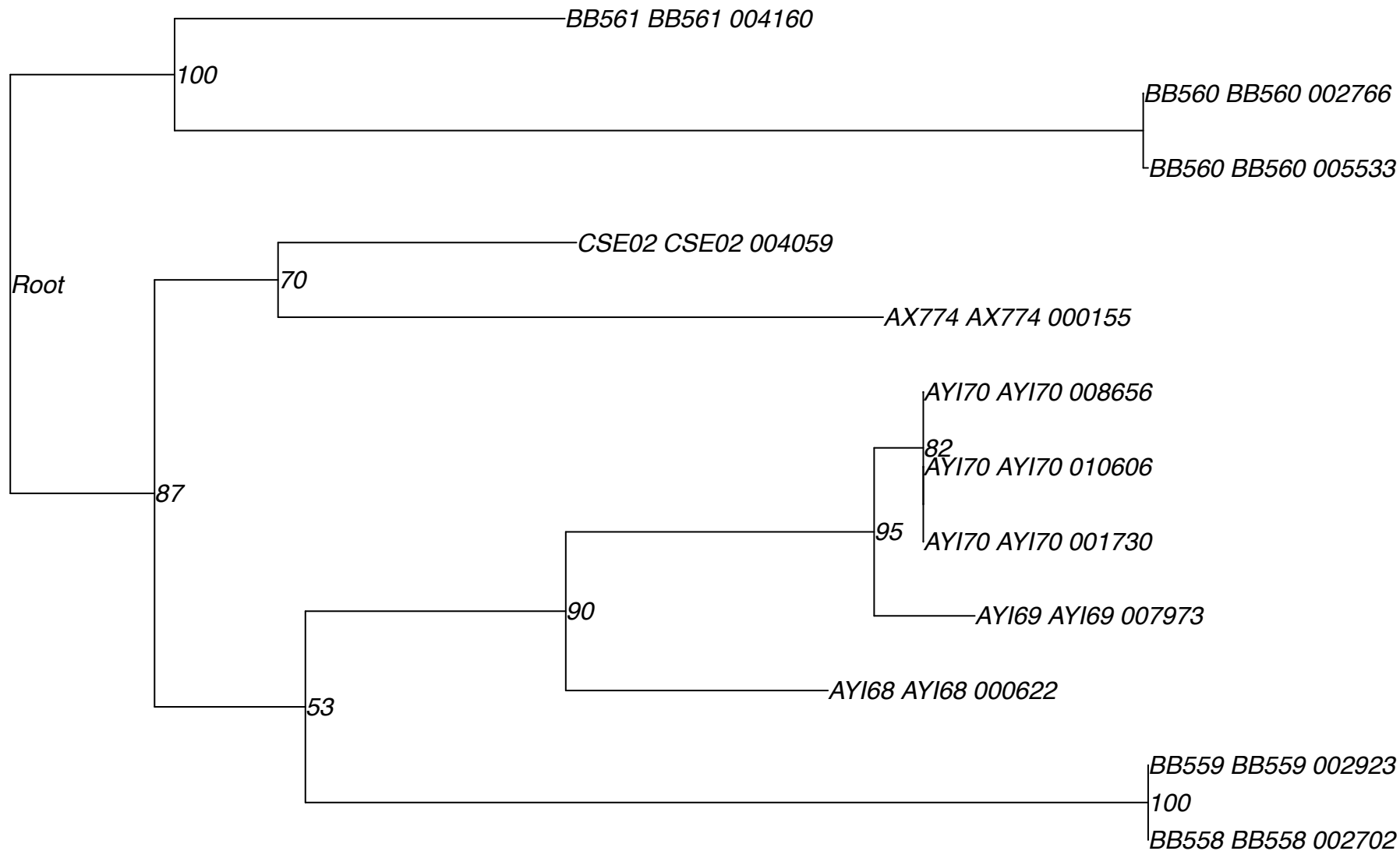

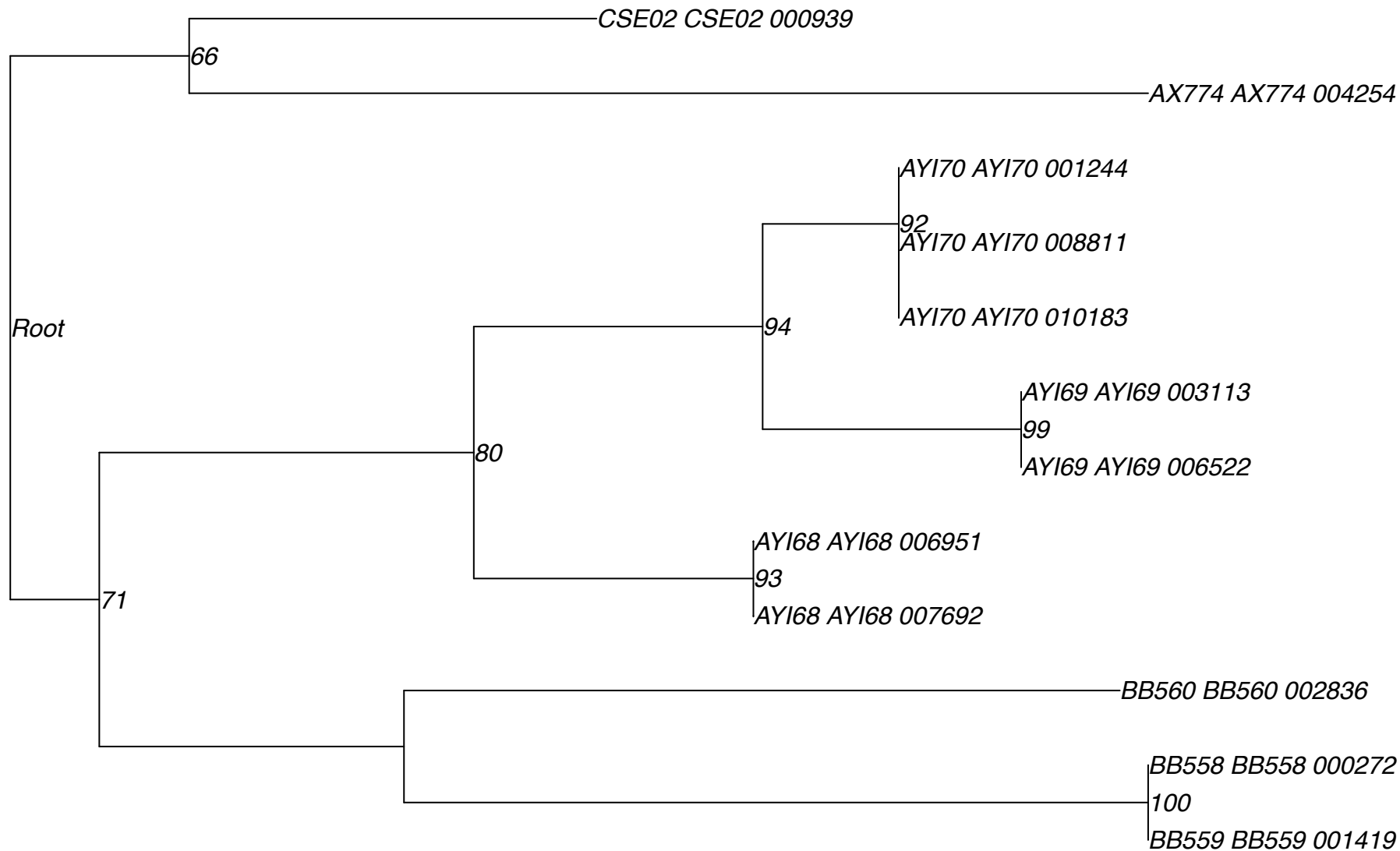

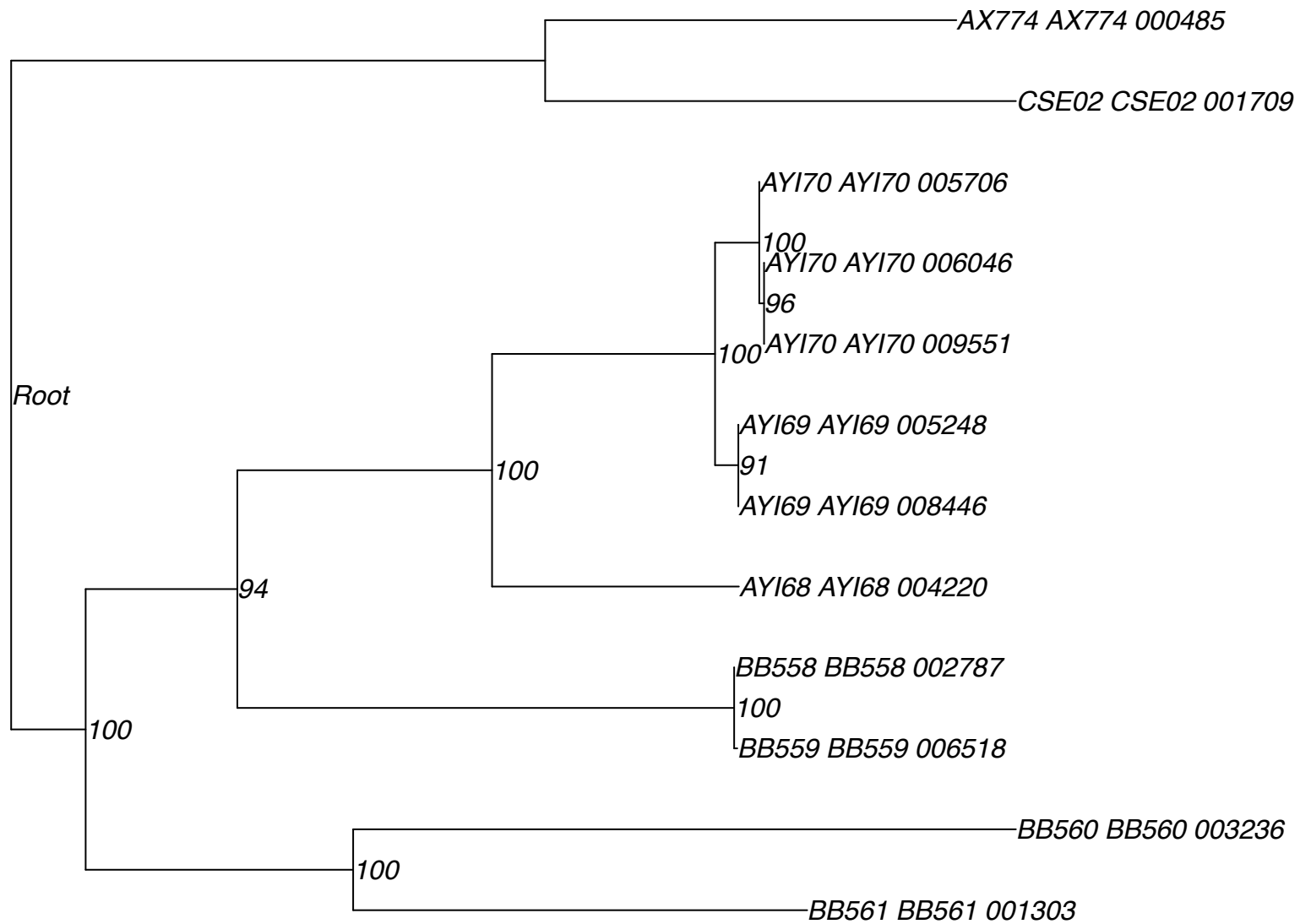

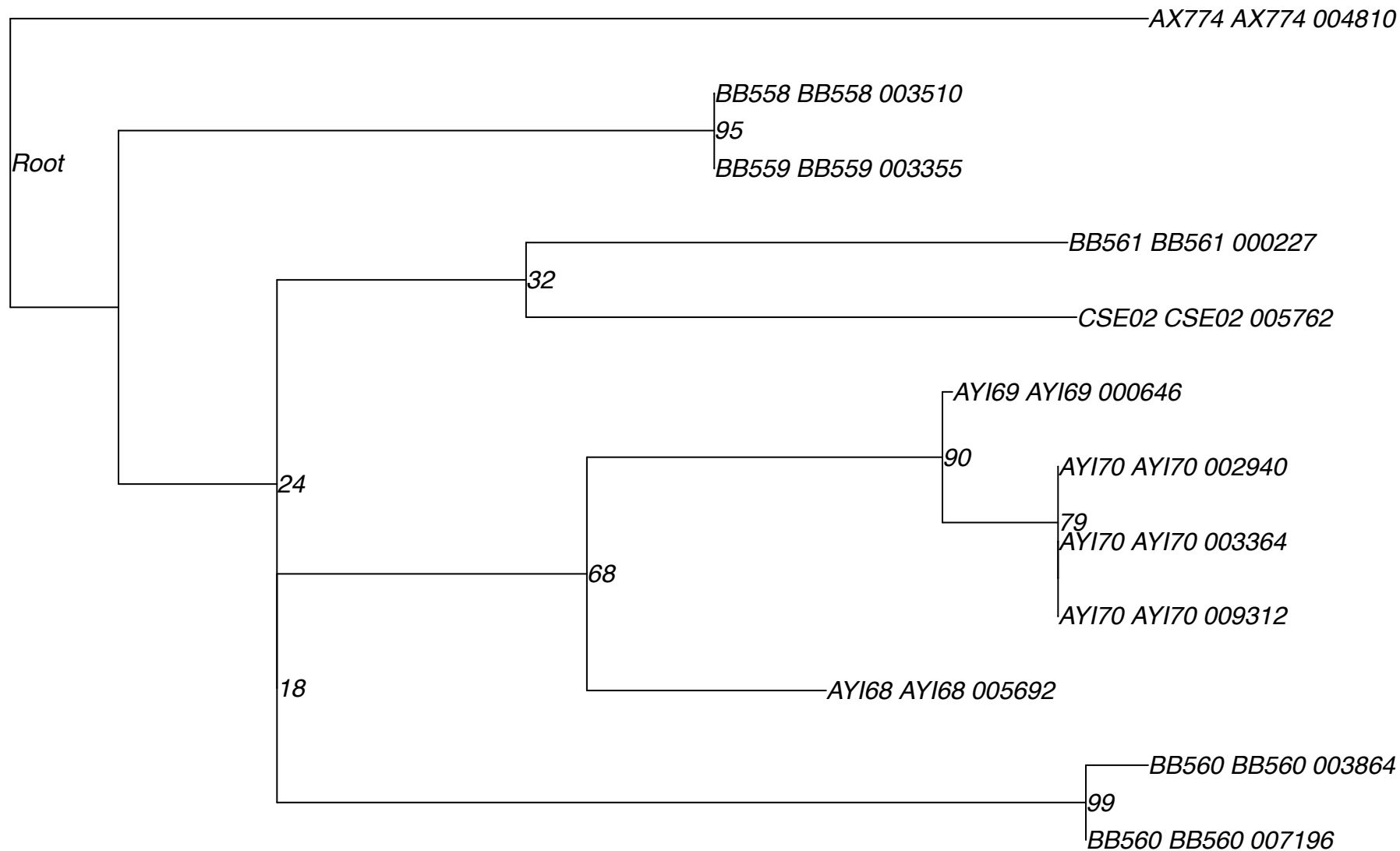

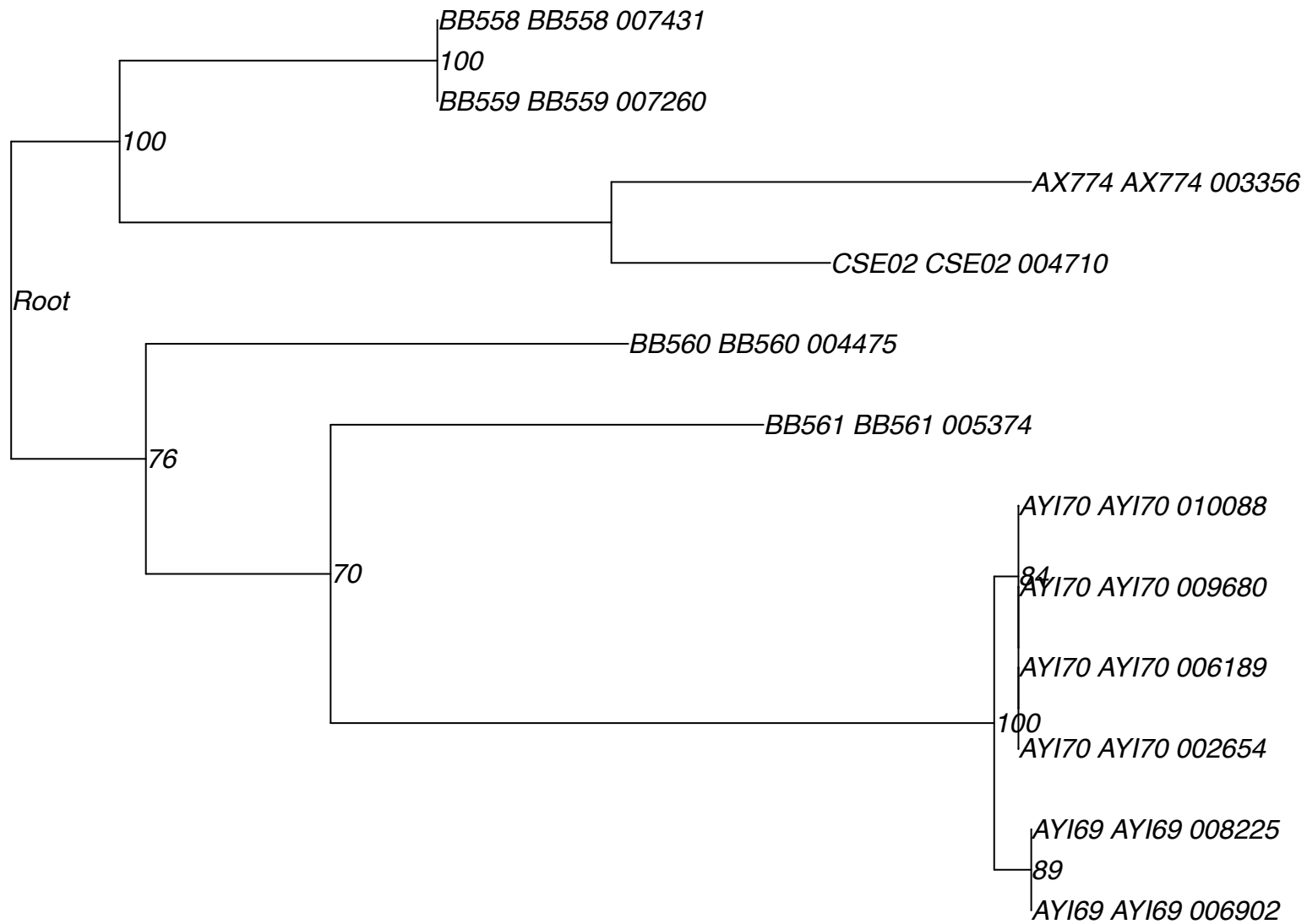

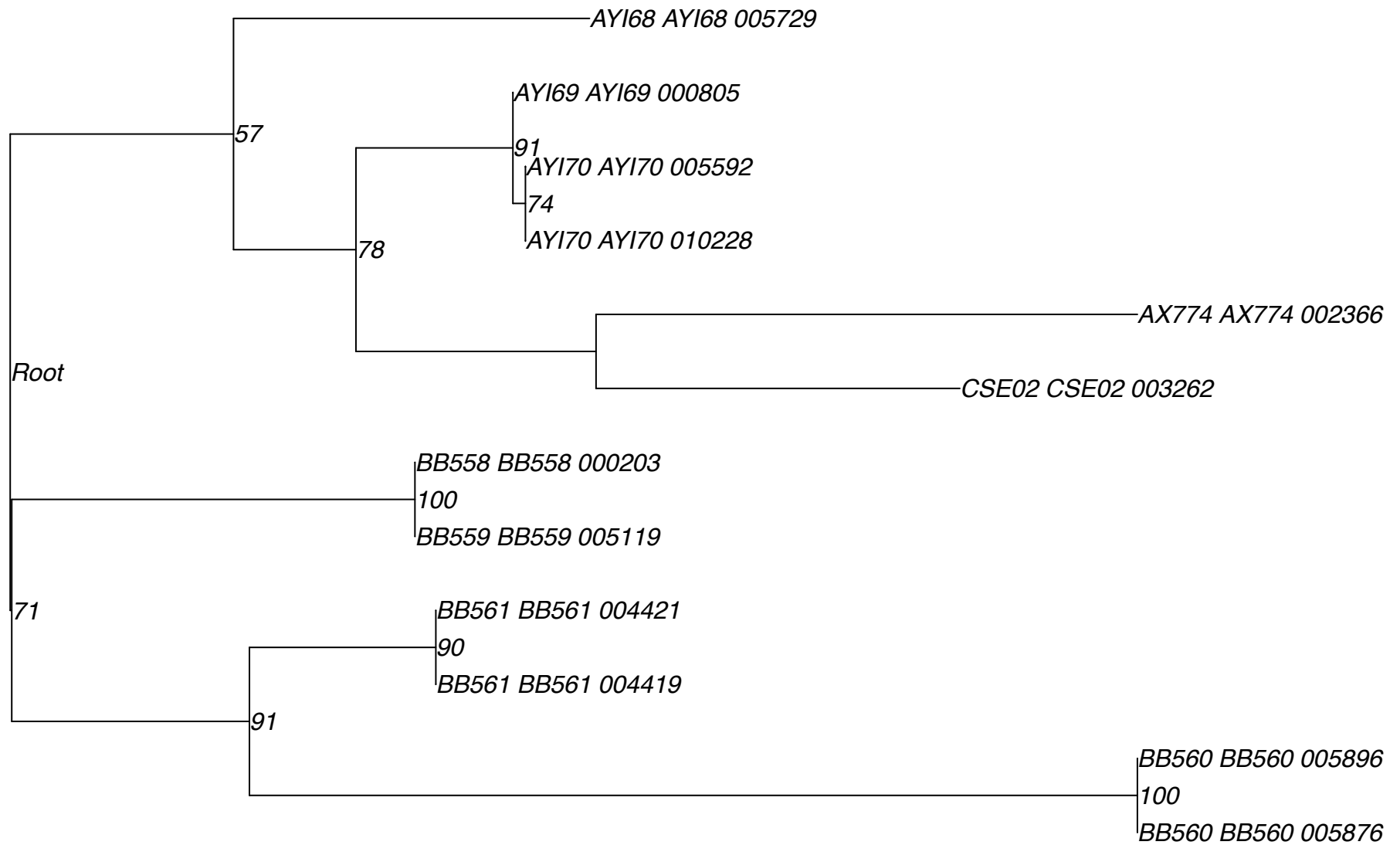

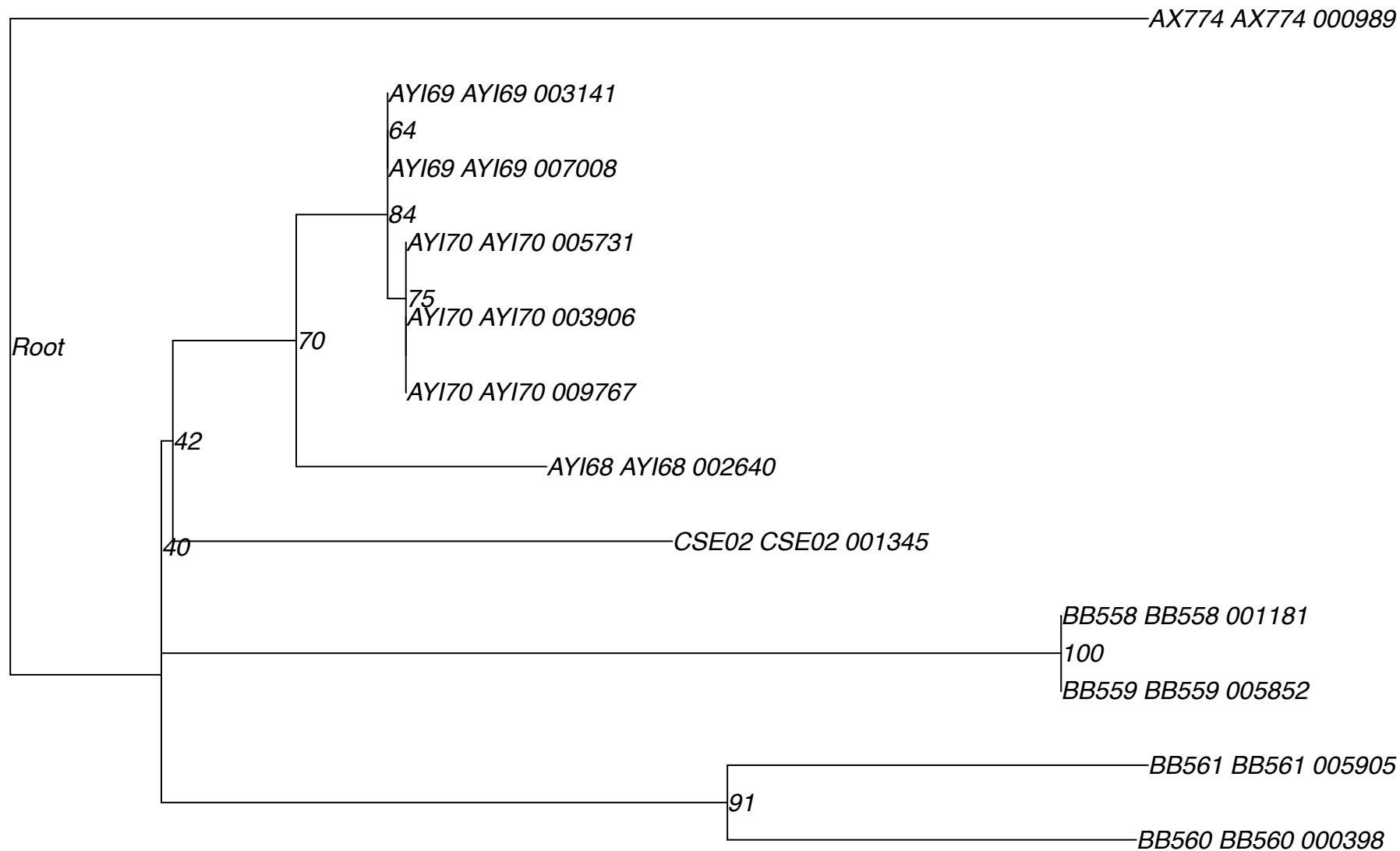

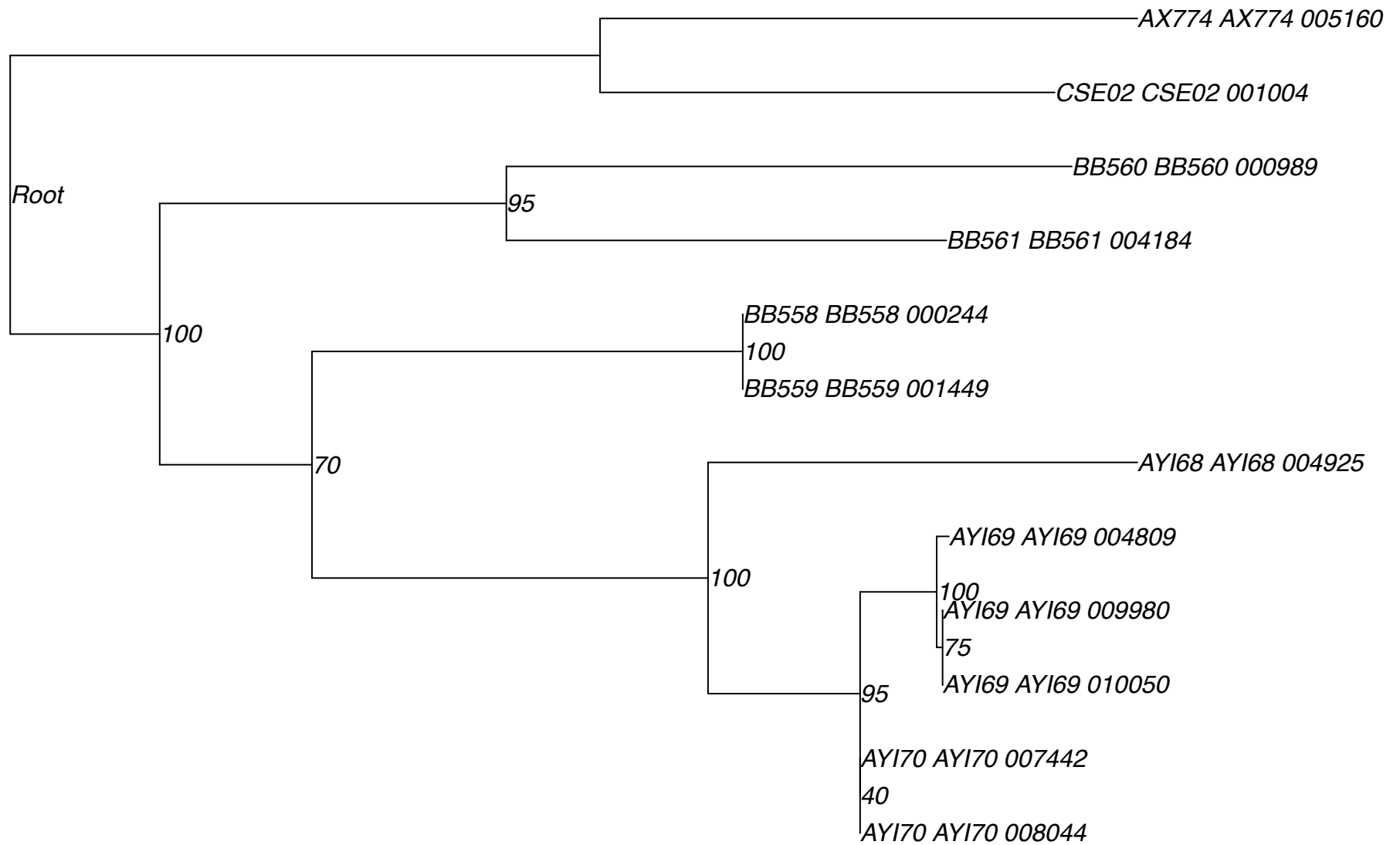

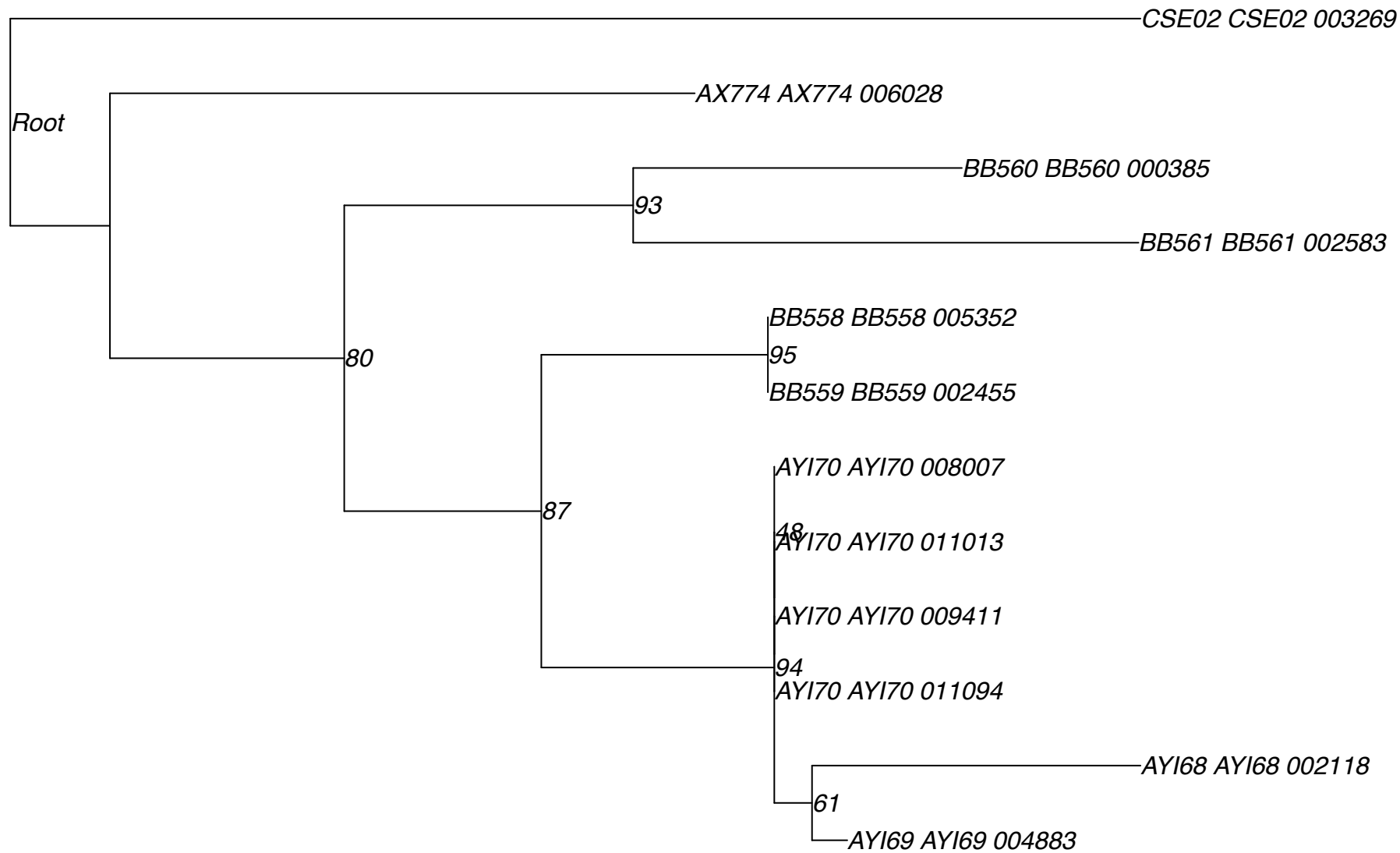

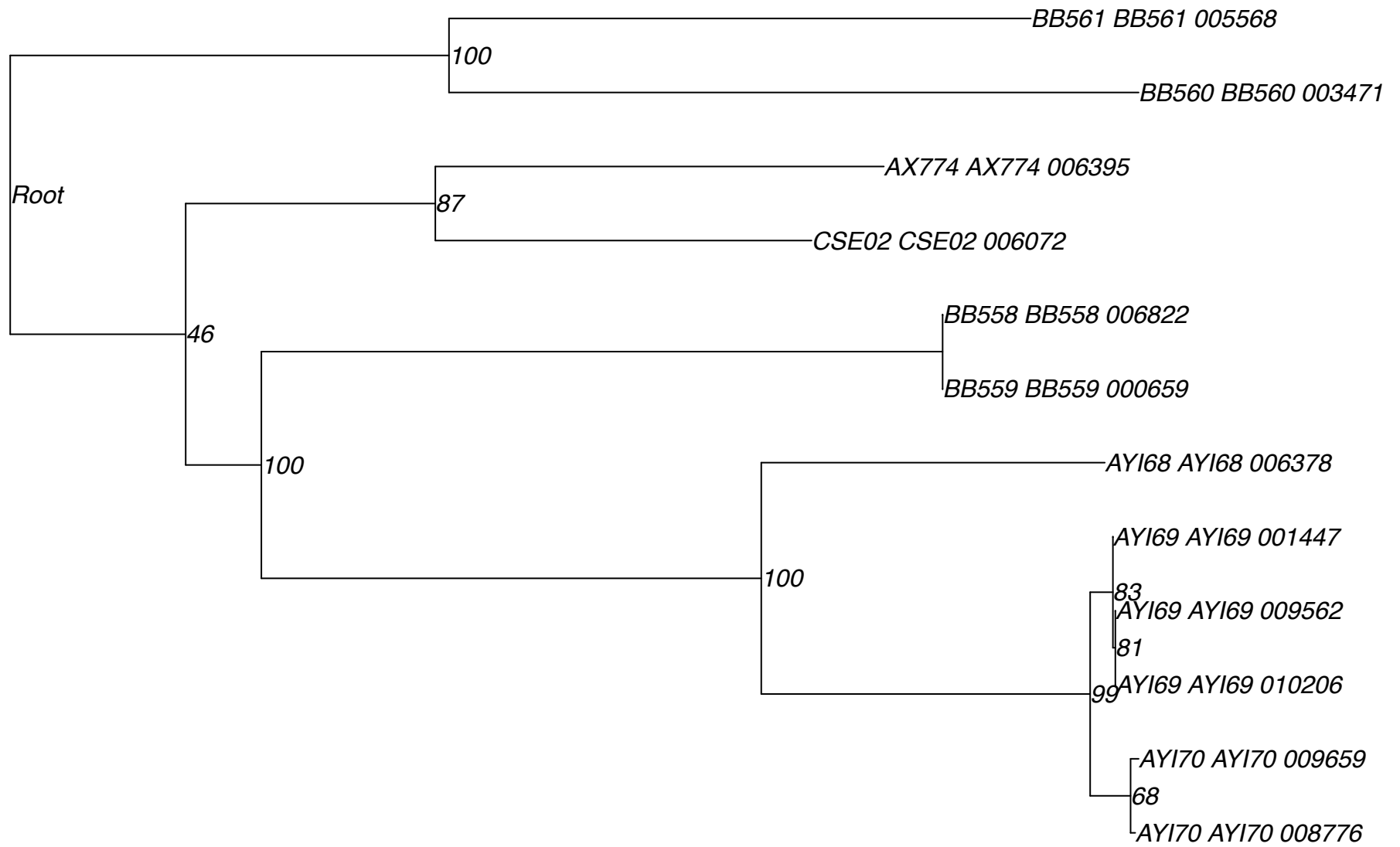

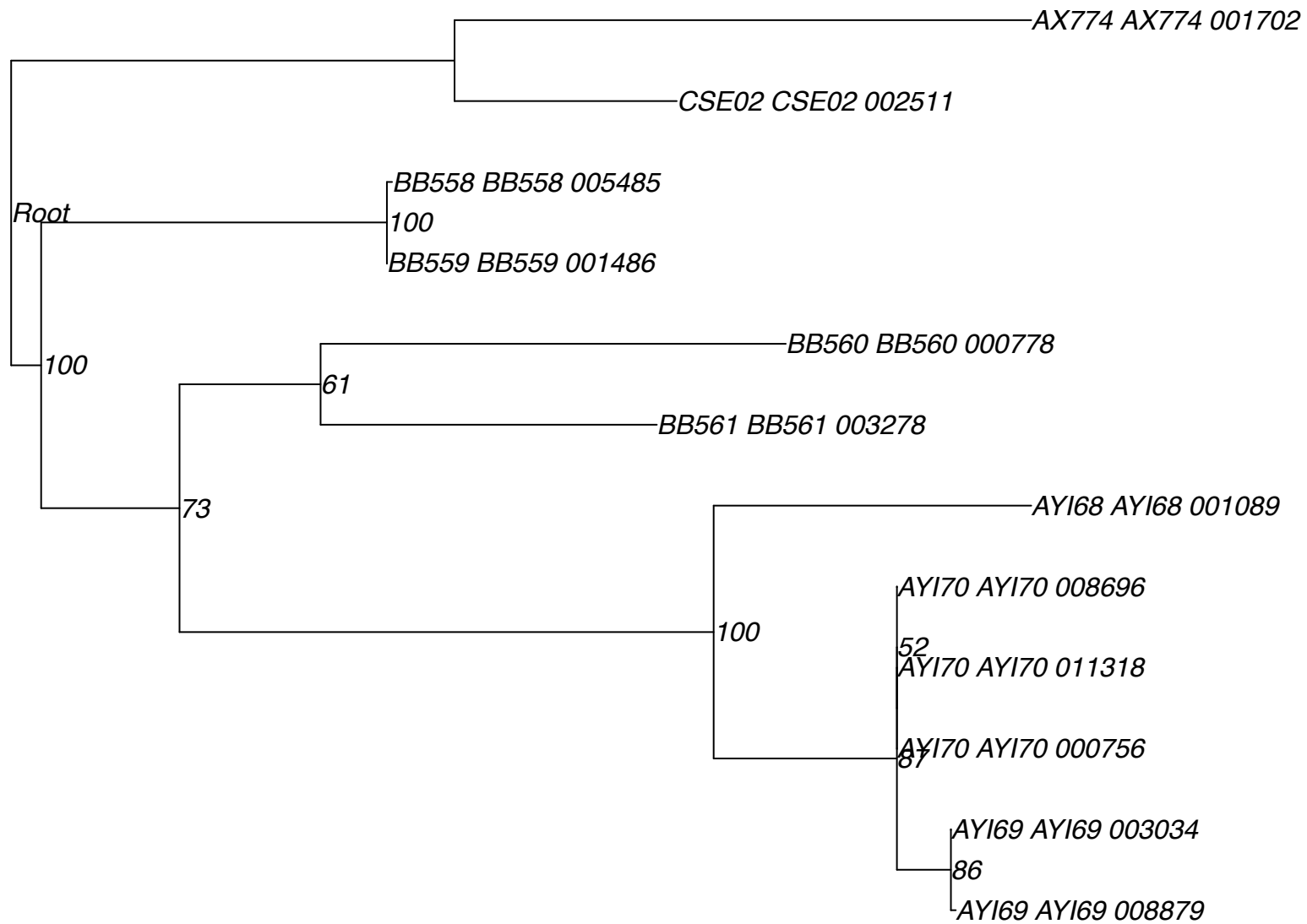

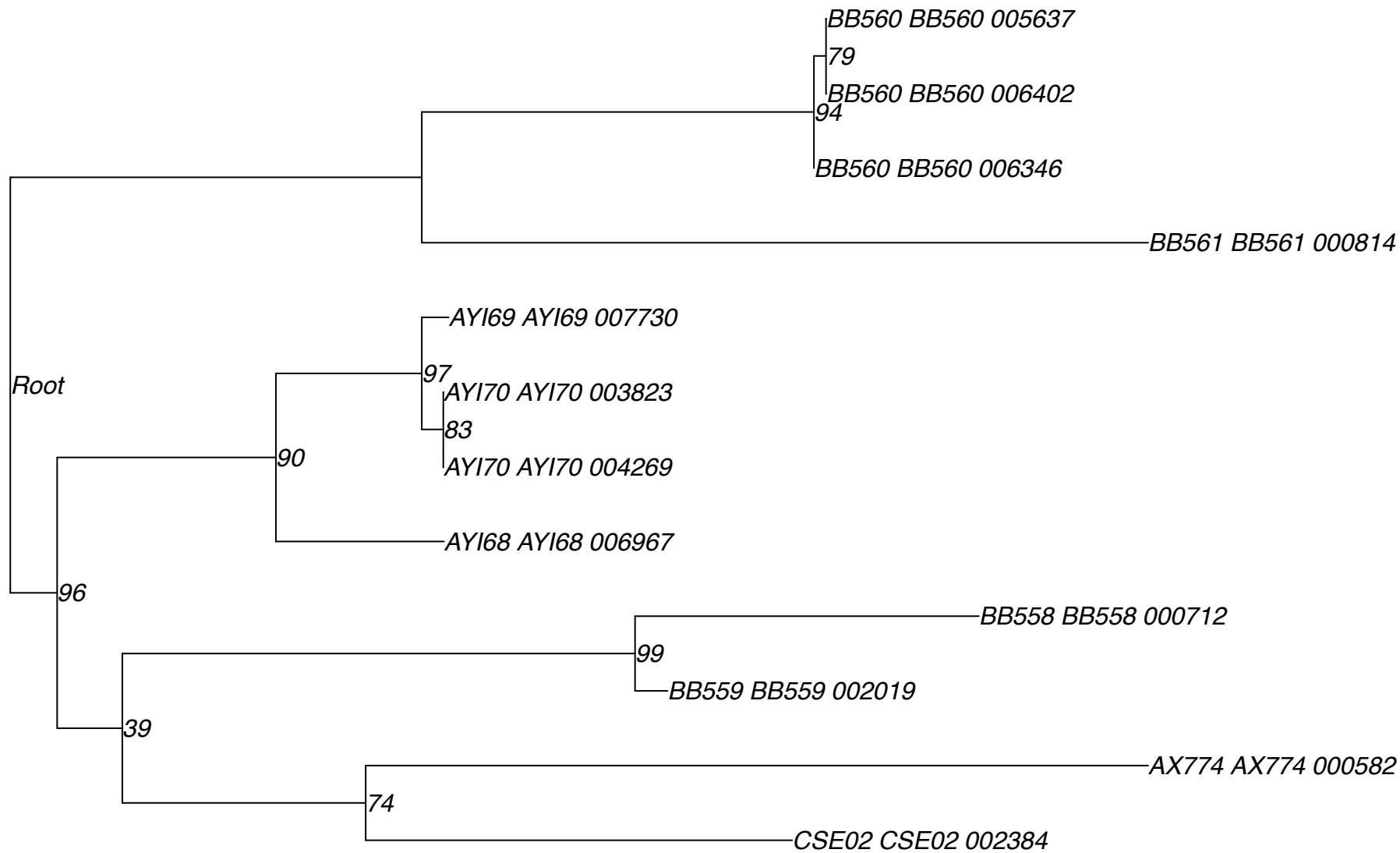

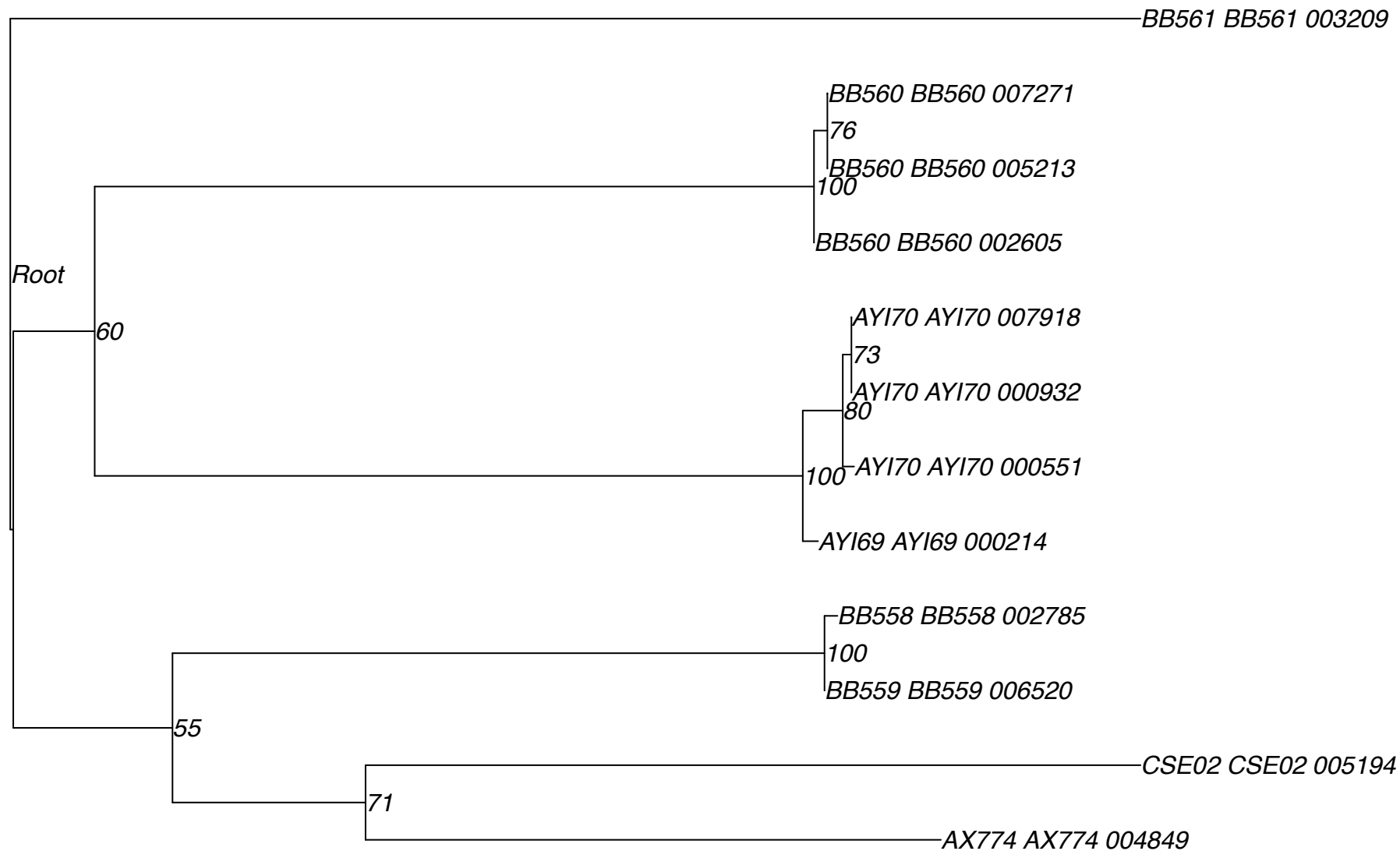

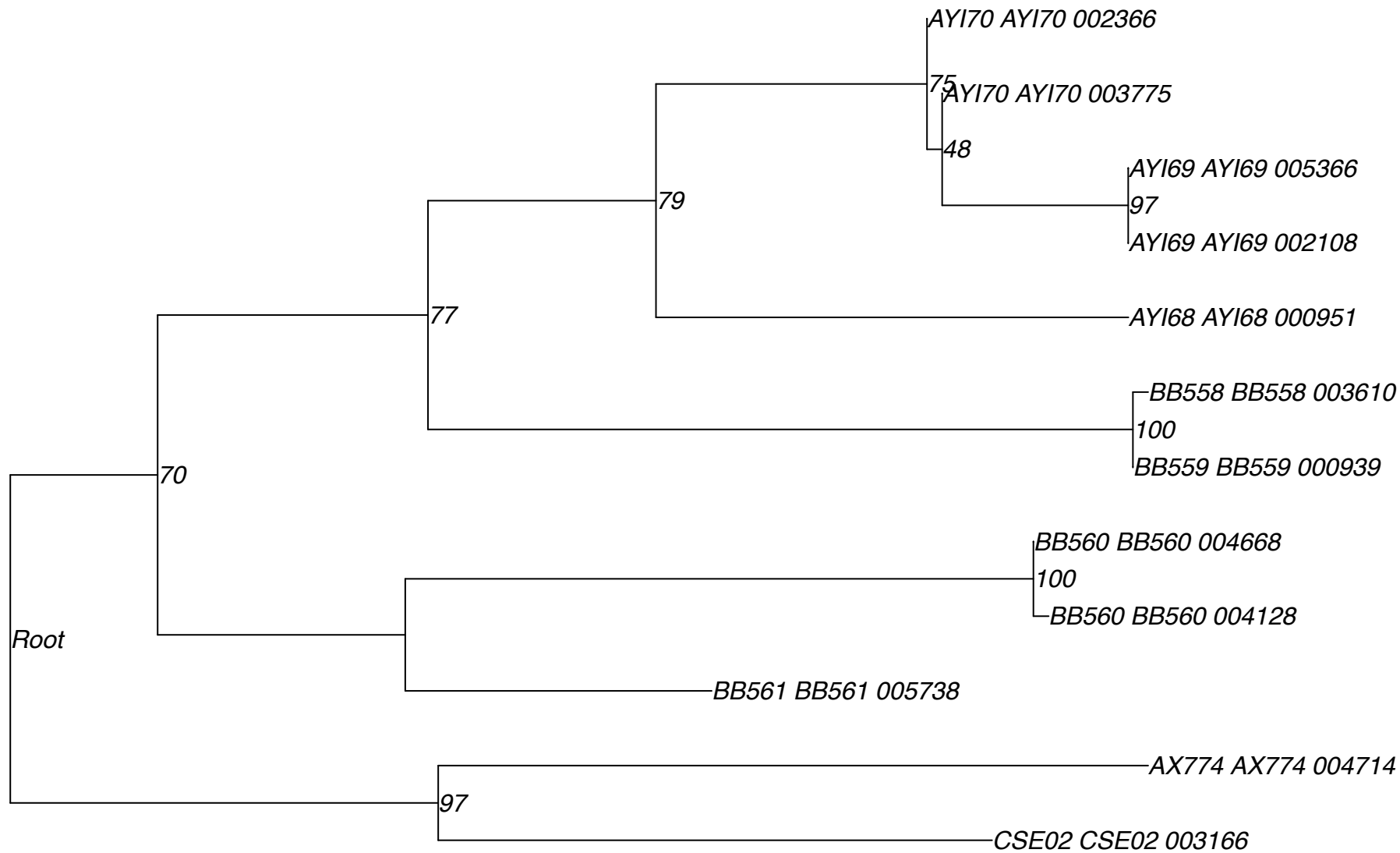

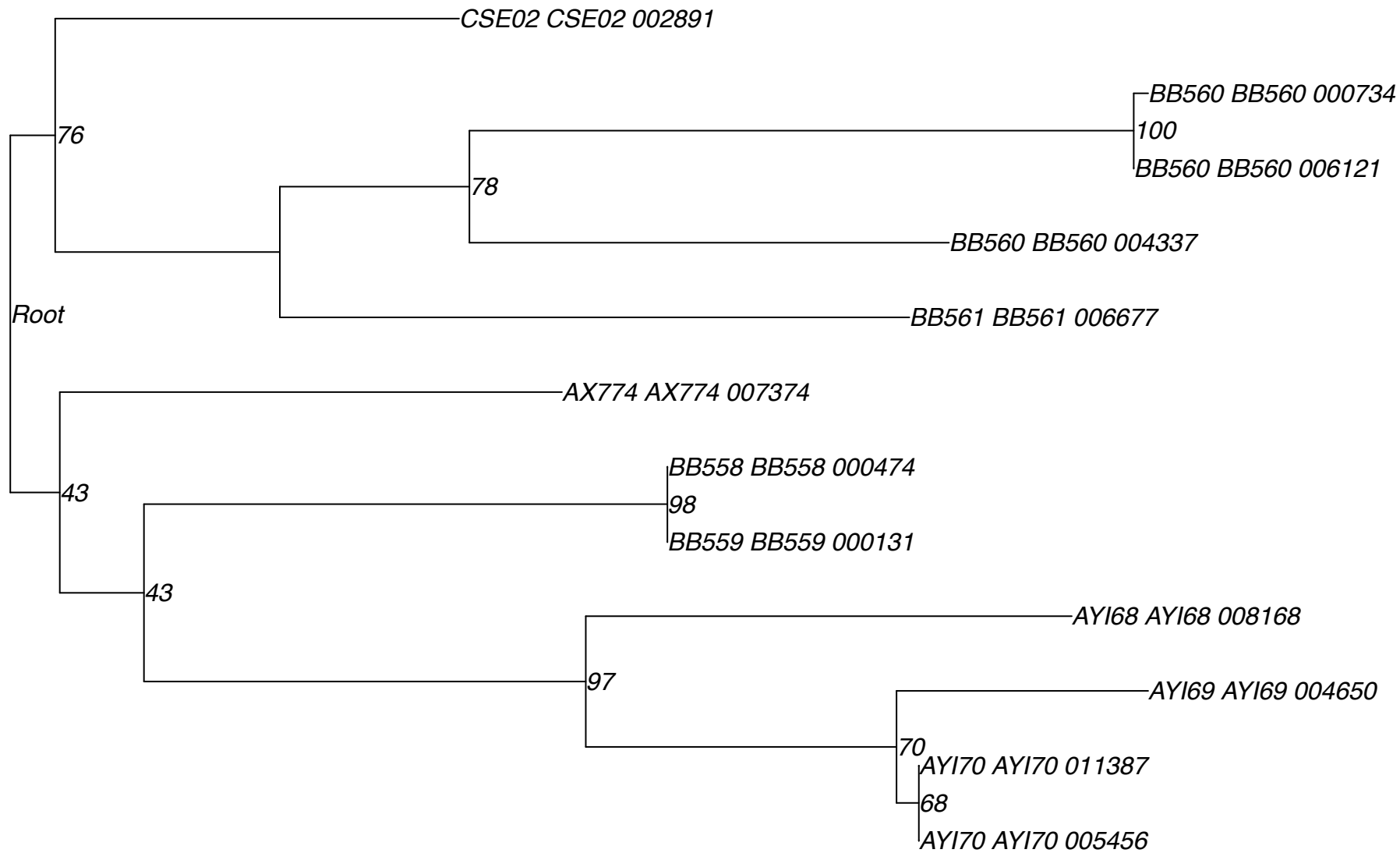

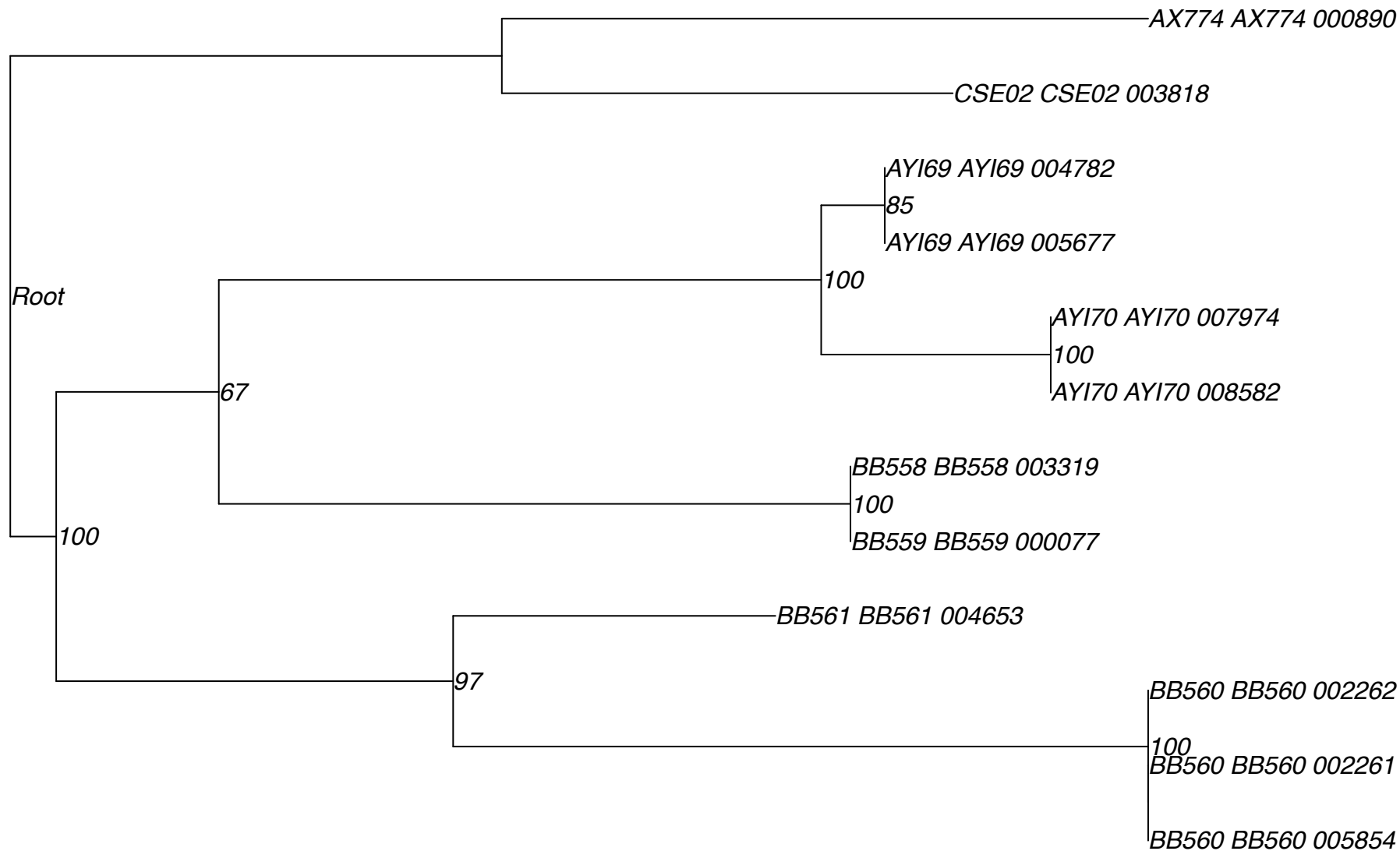

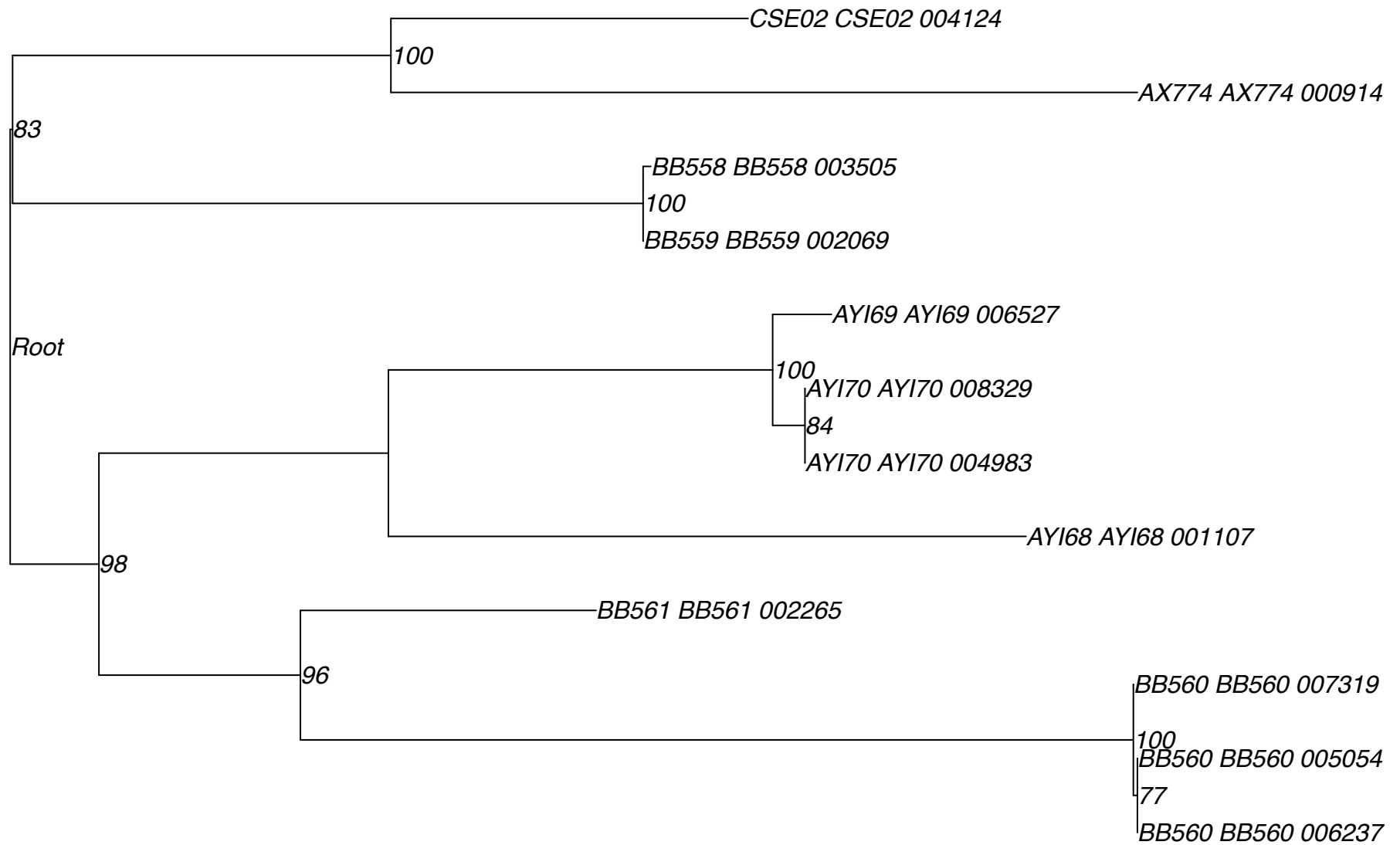

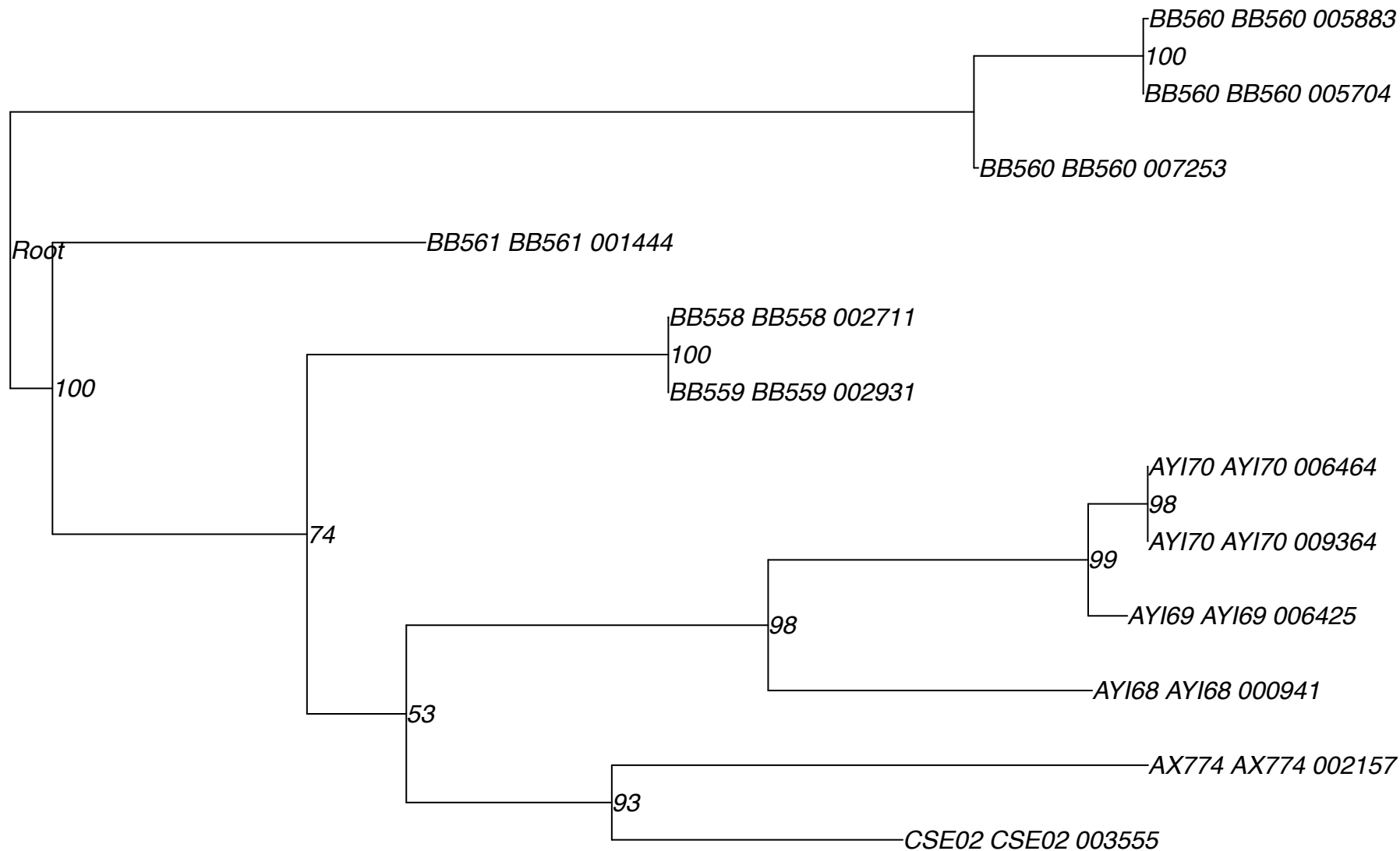

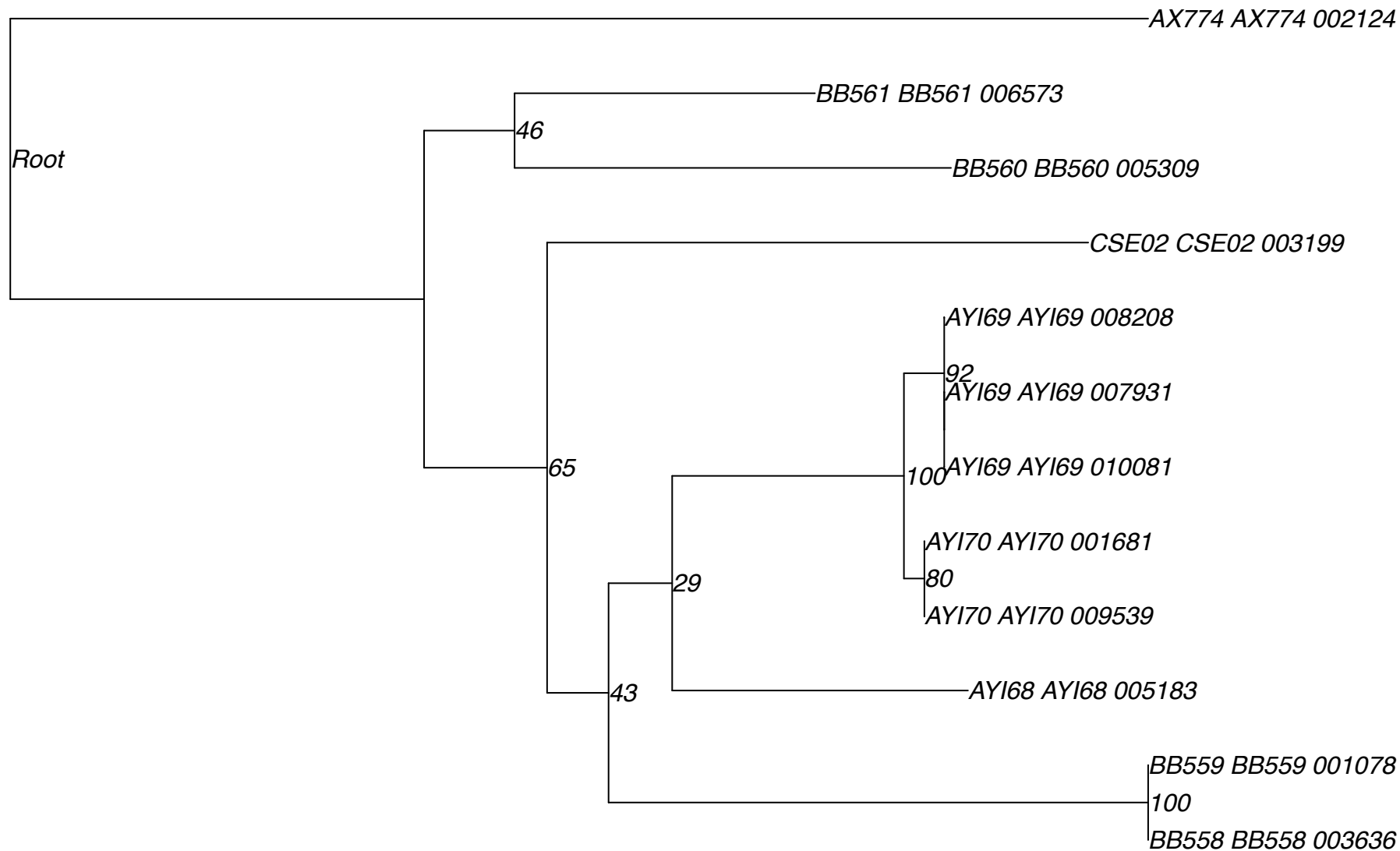

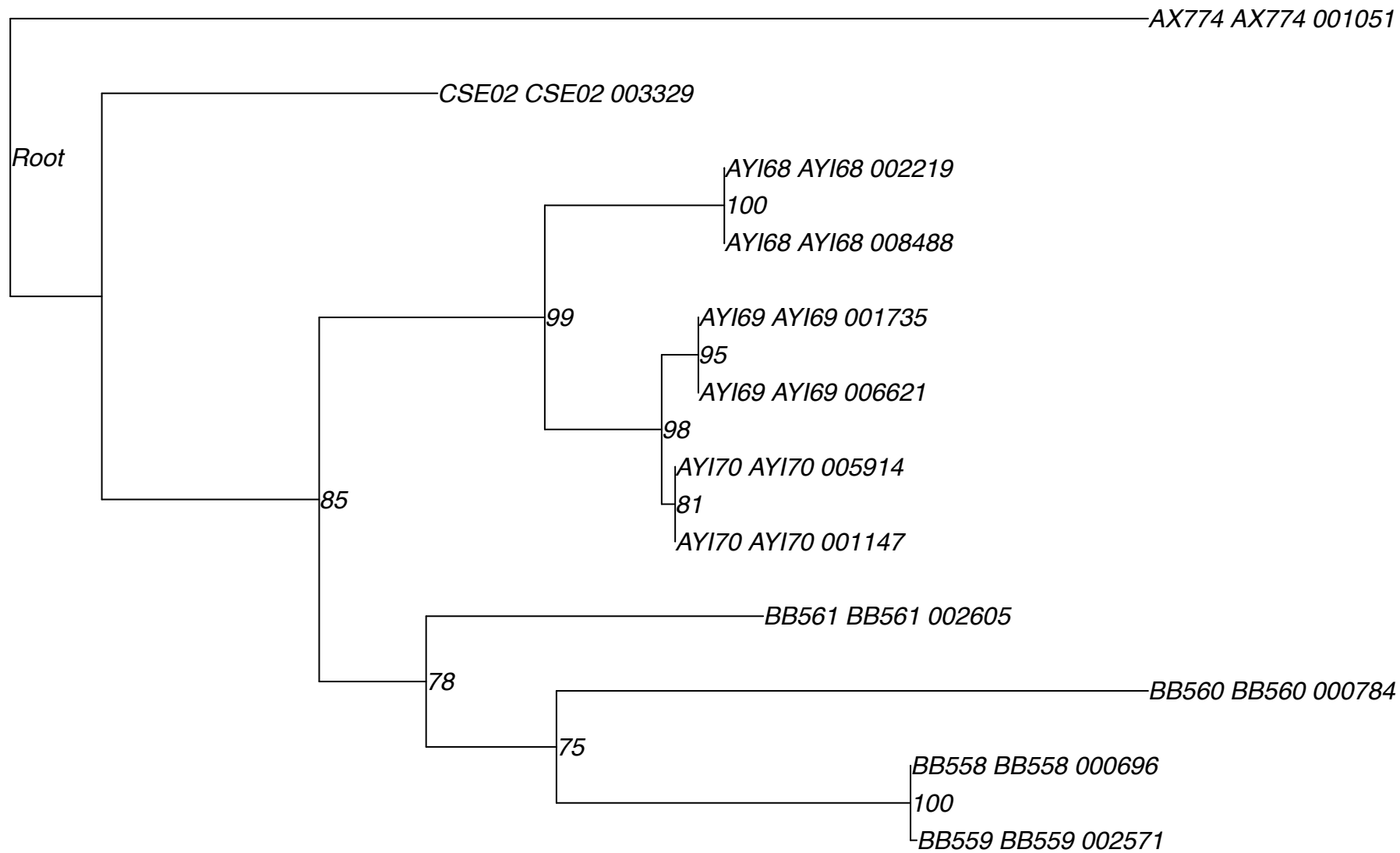

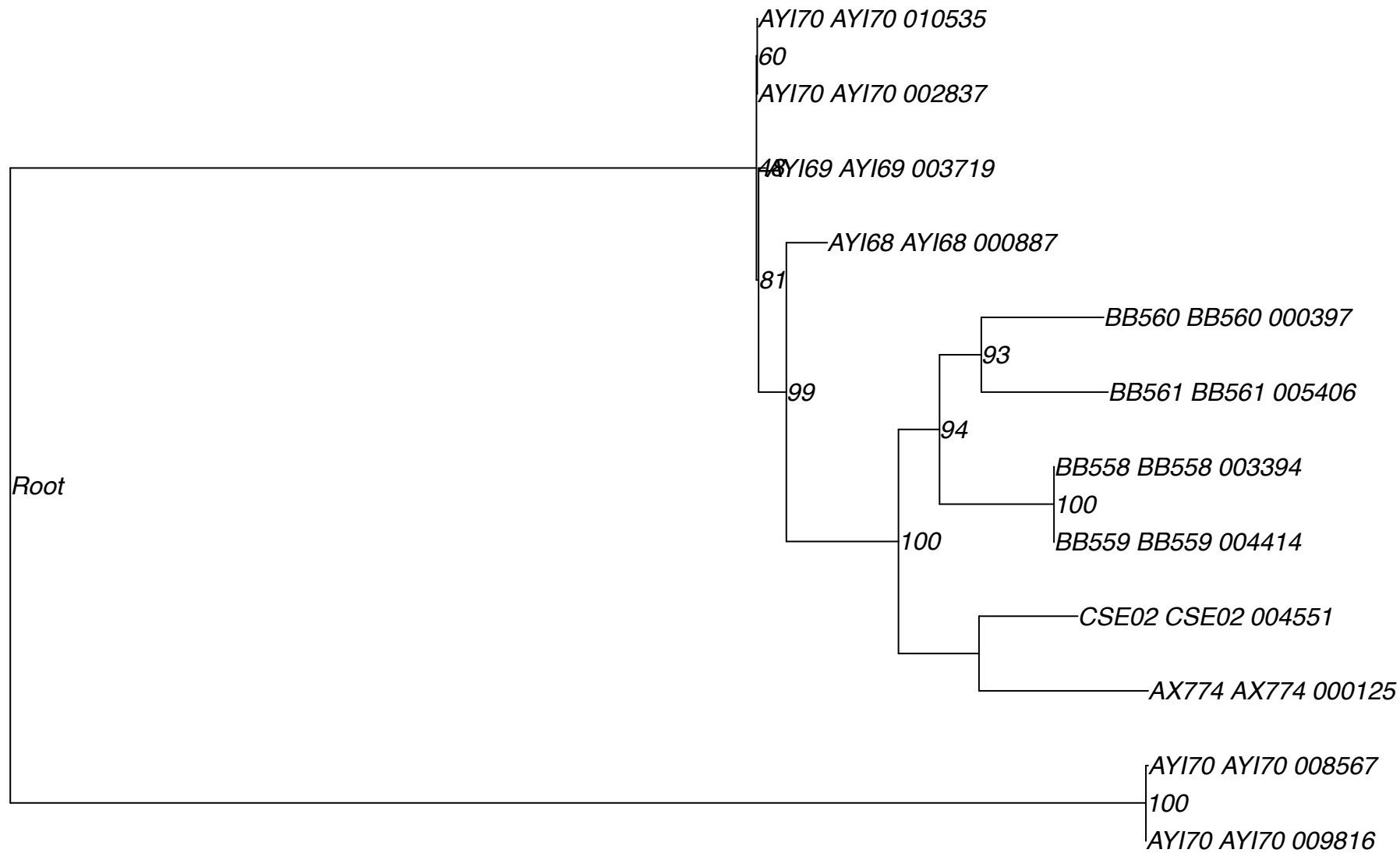

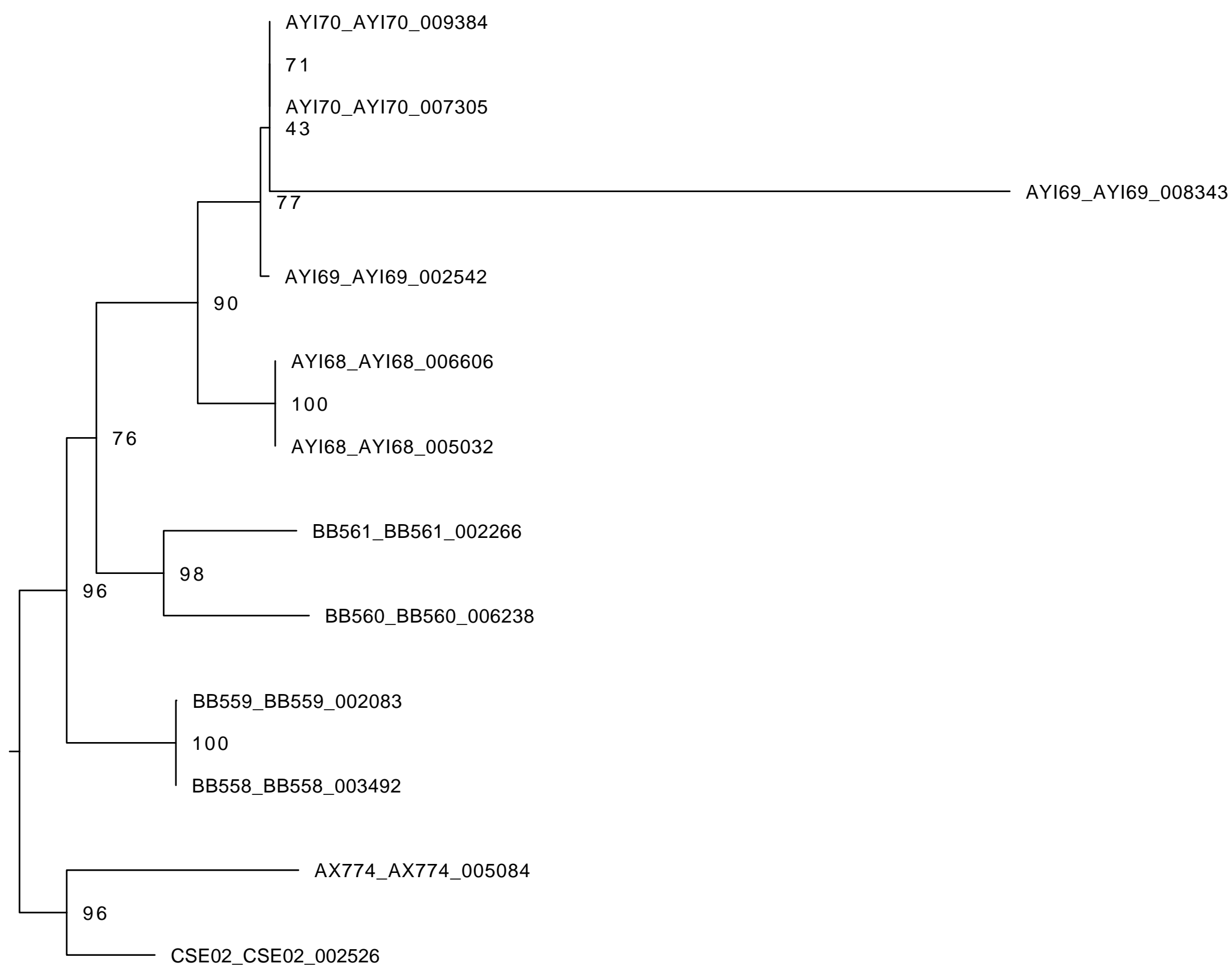

0.5

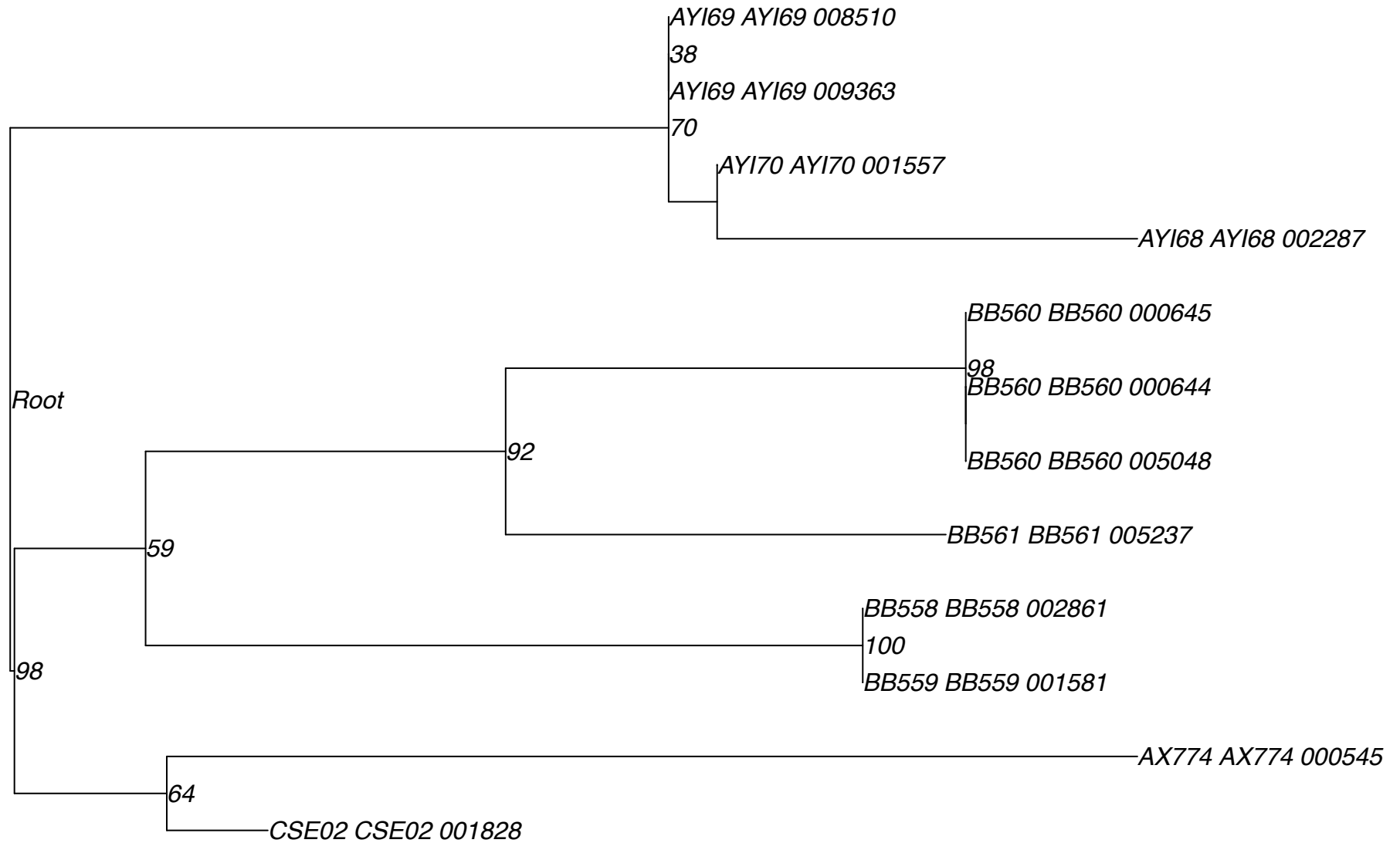

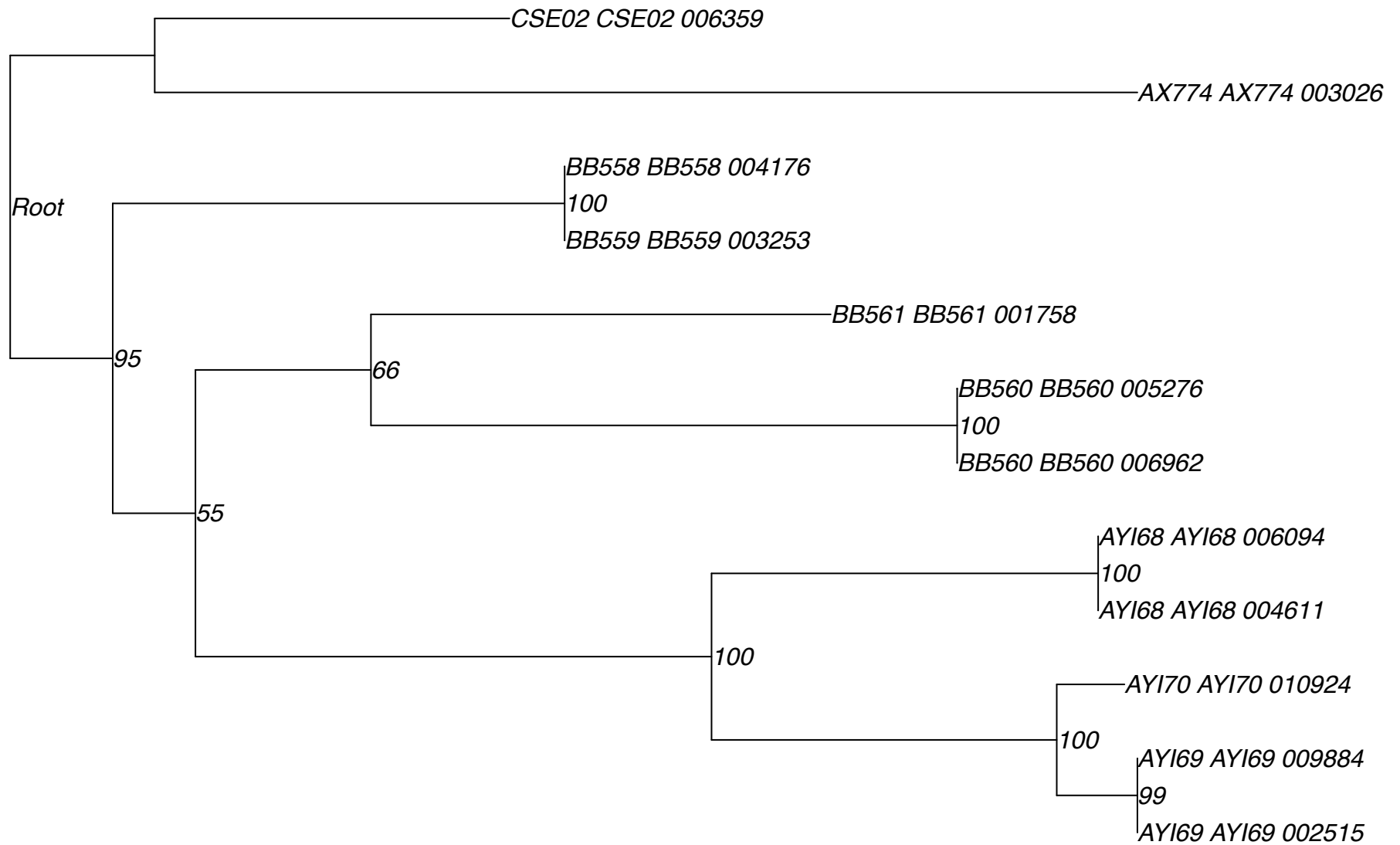

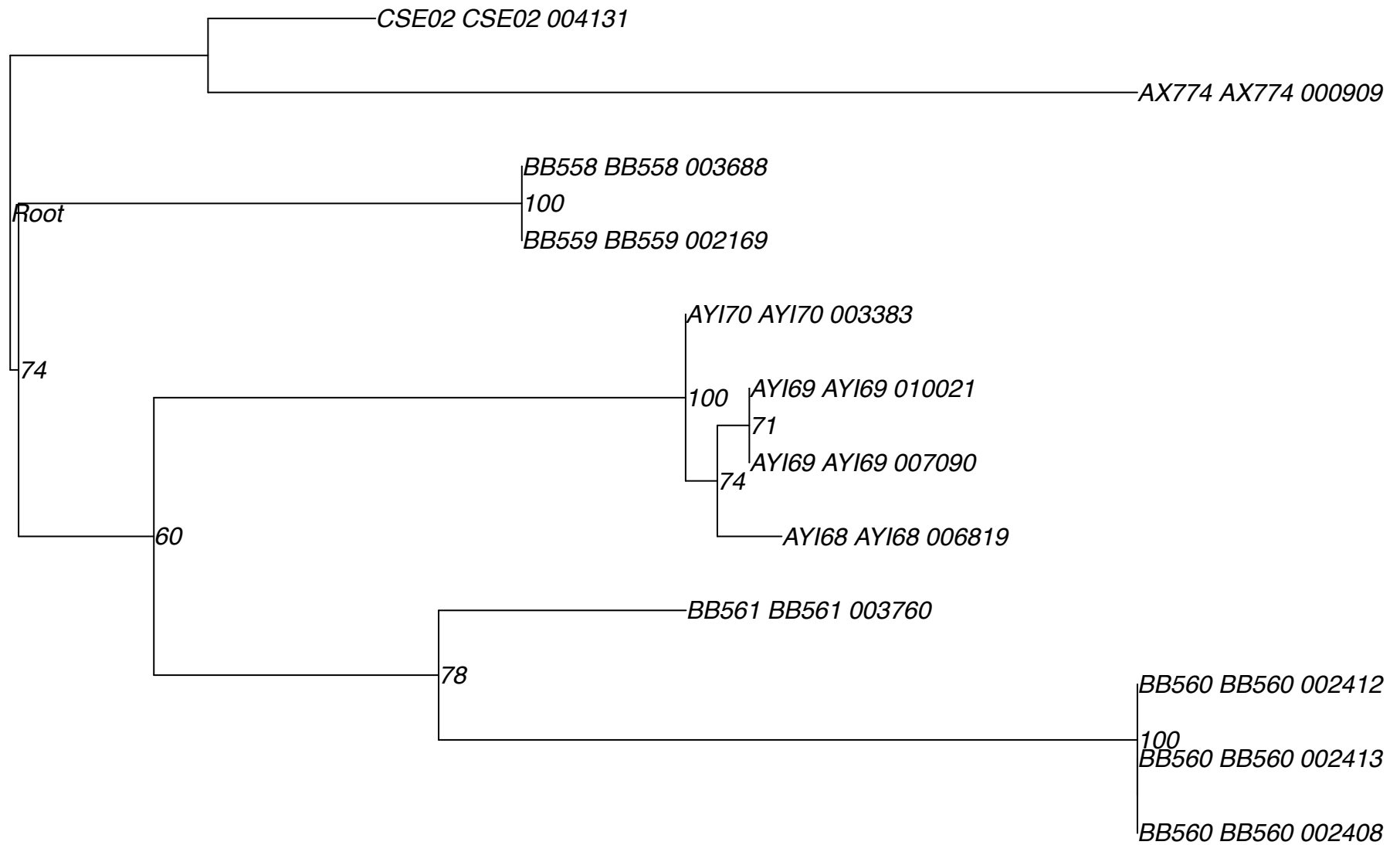

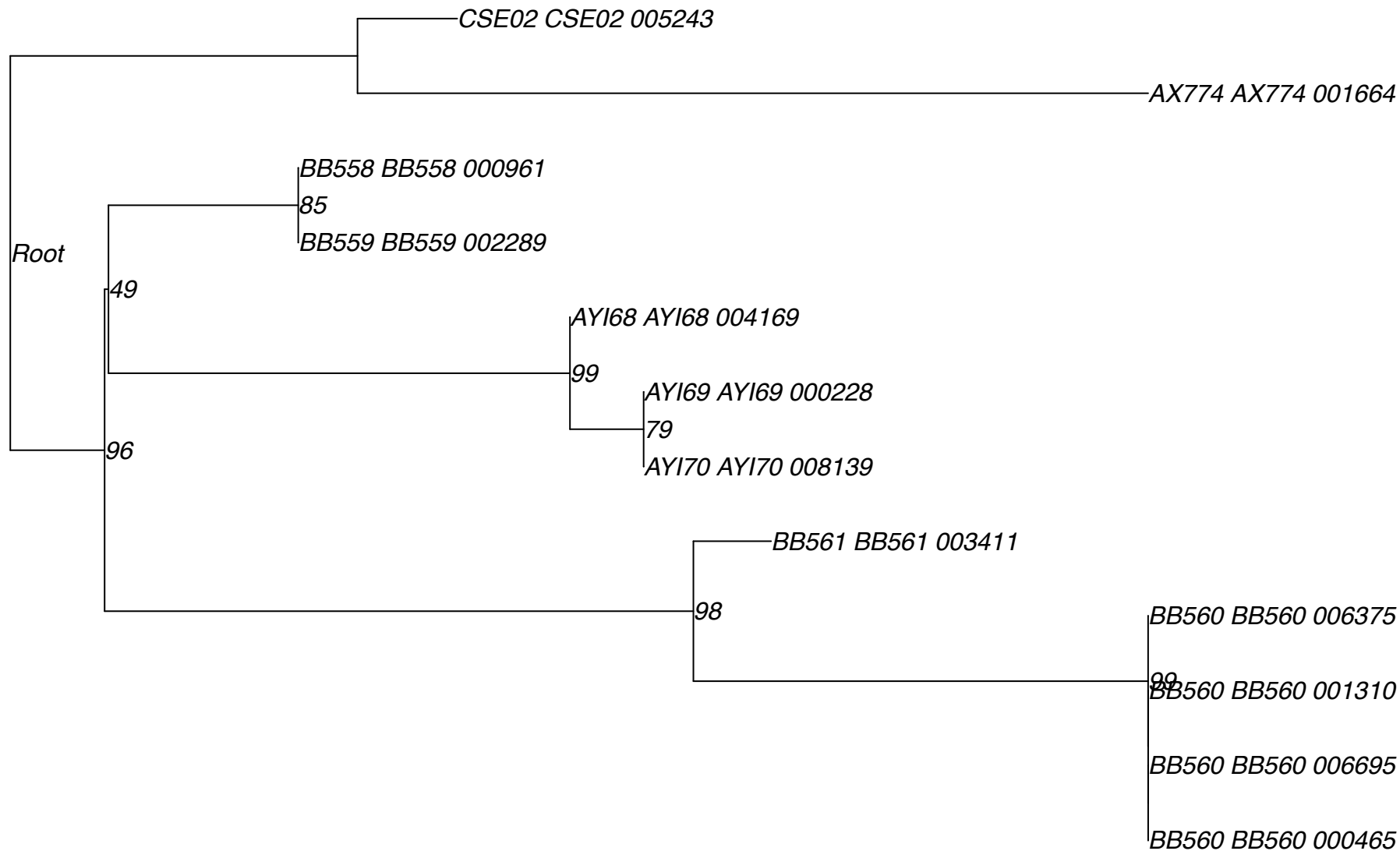

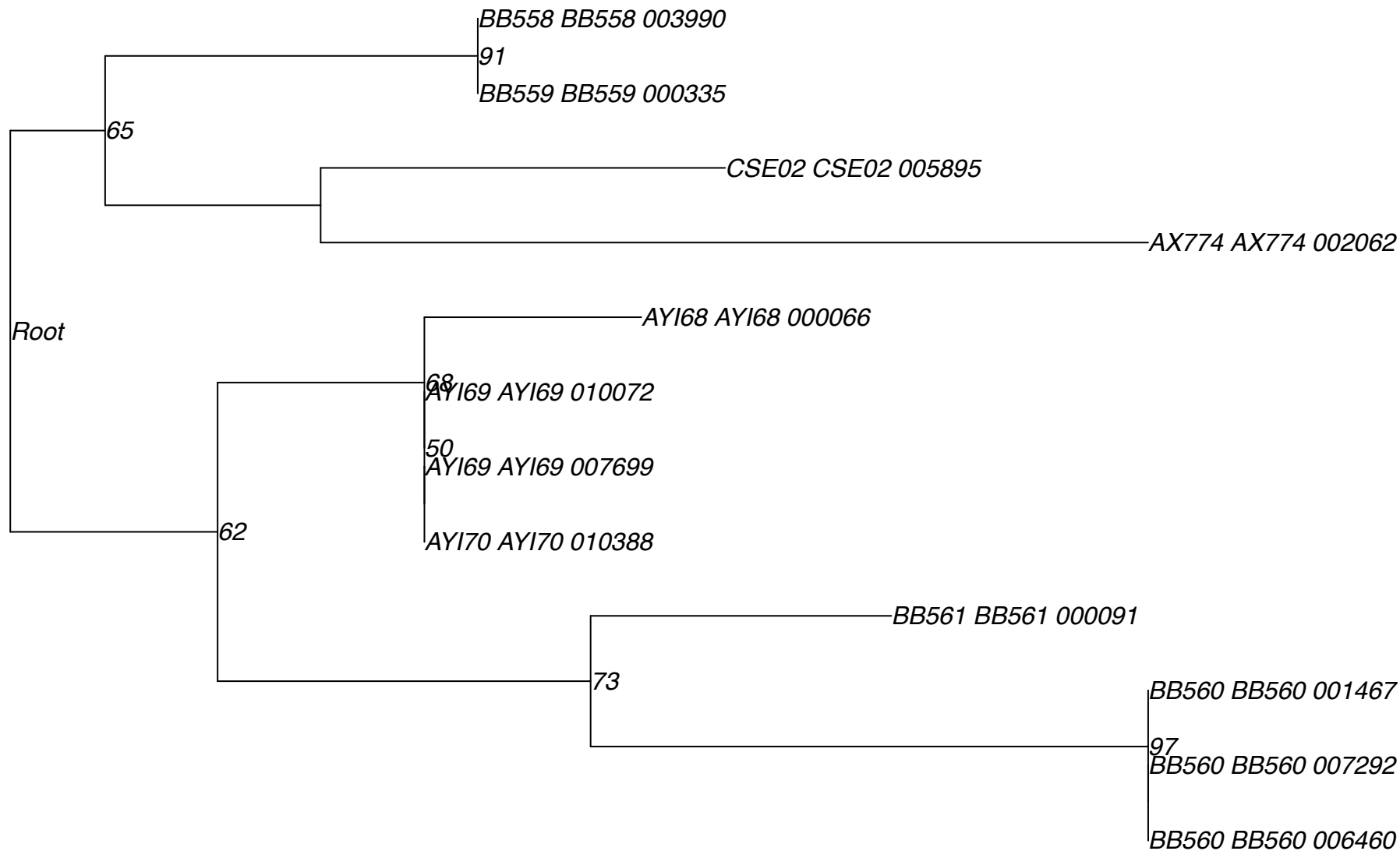

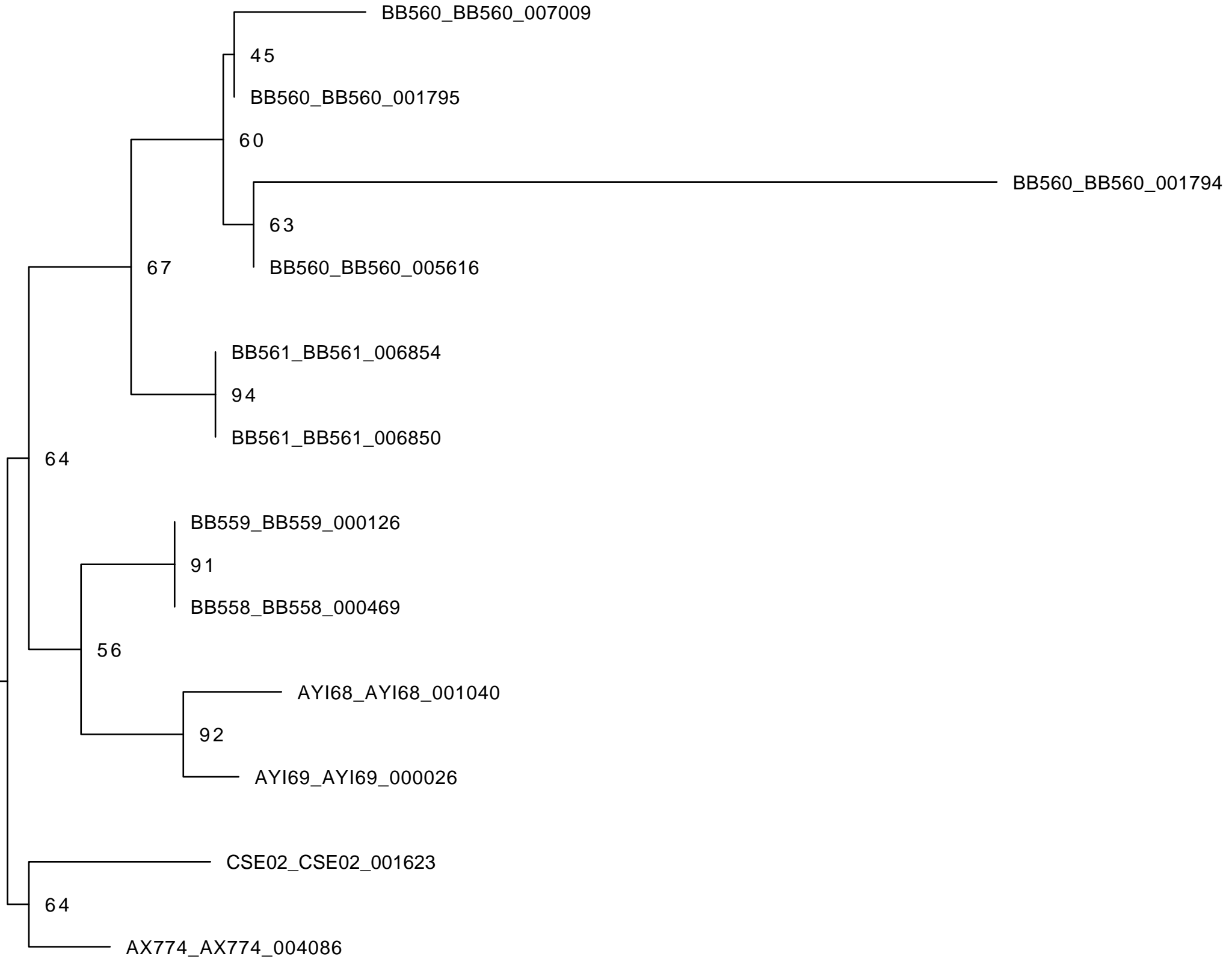

0.3

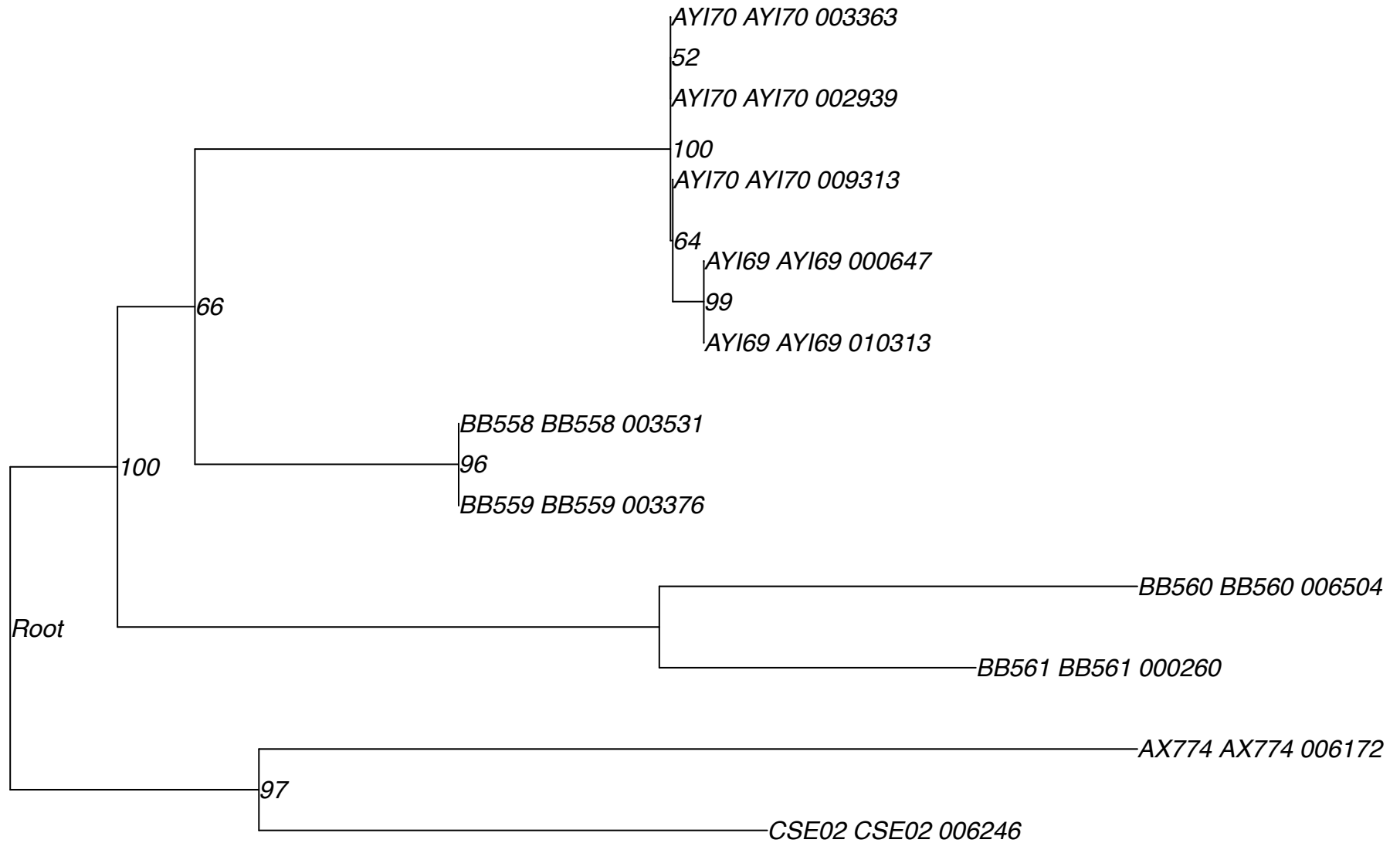

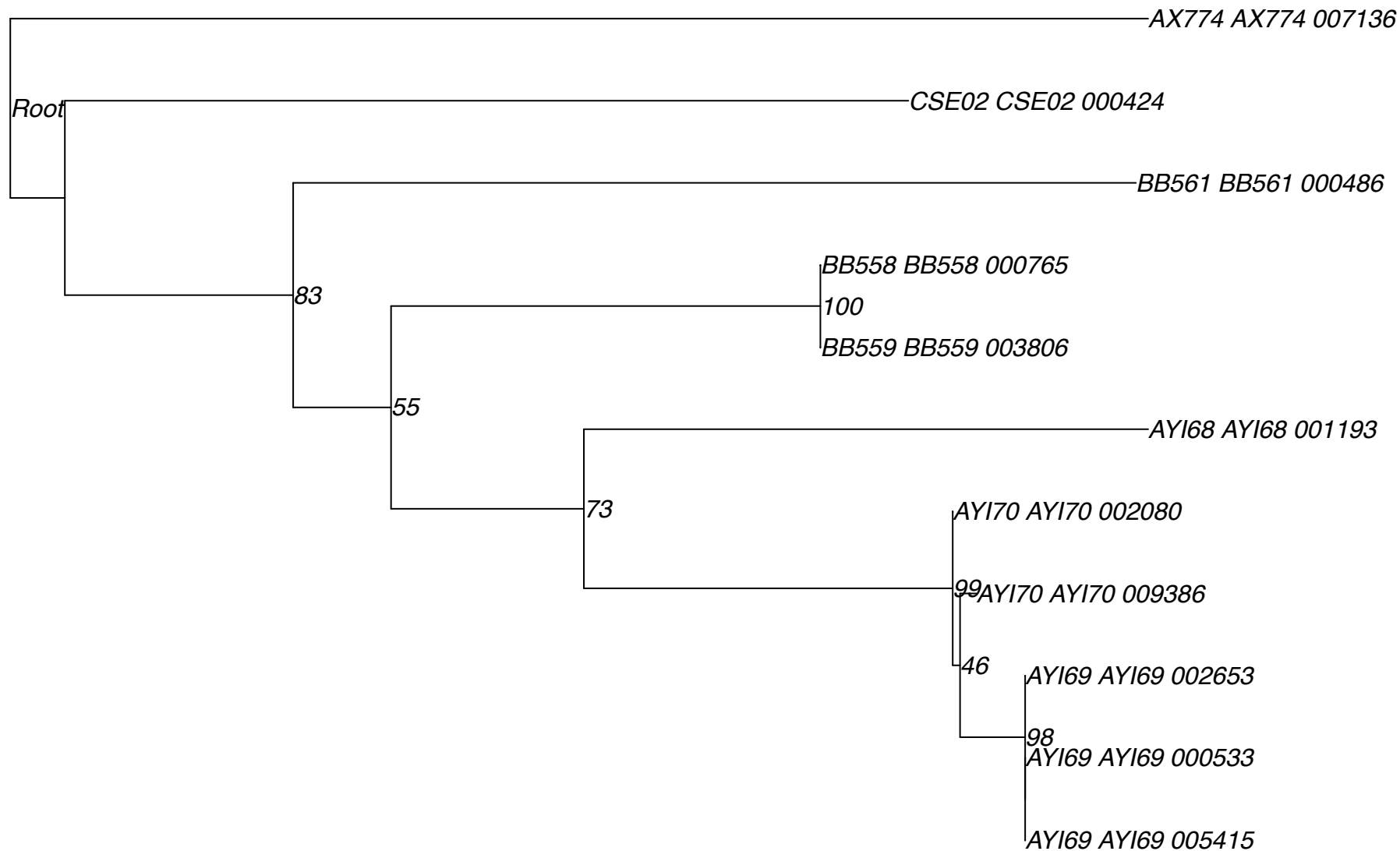

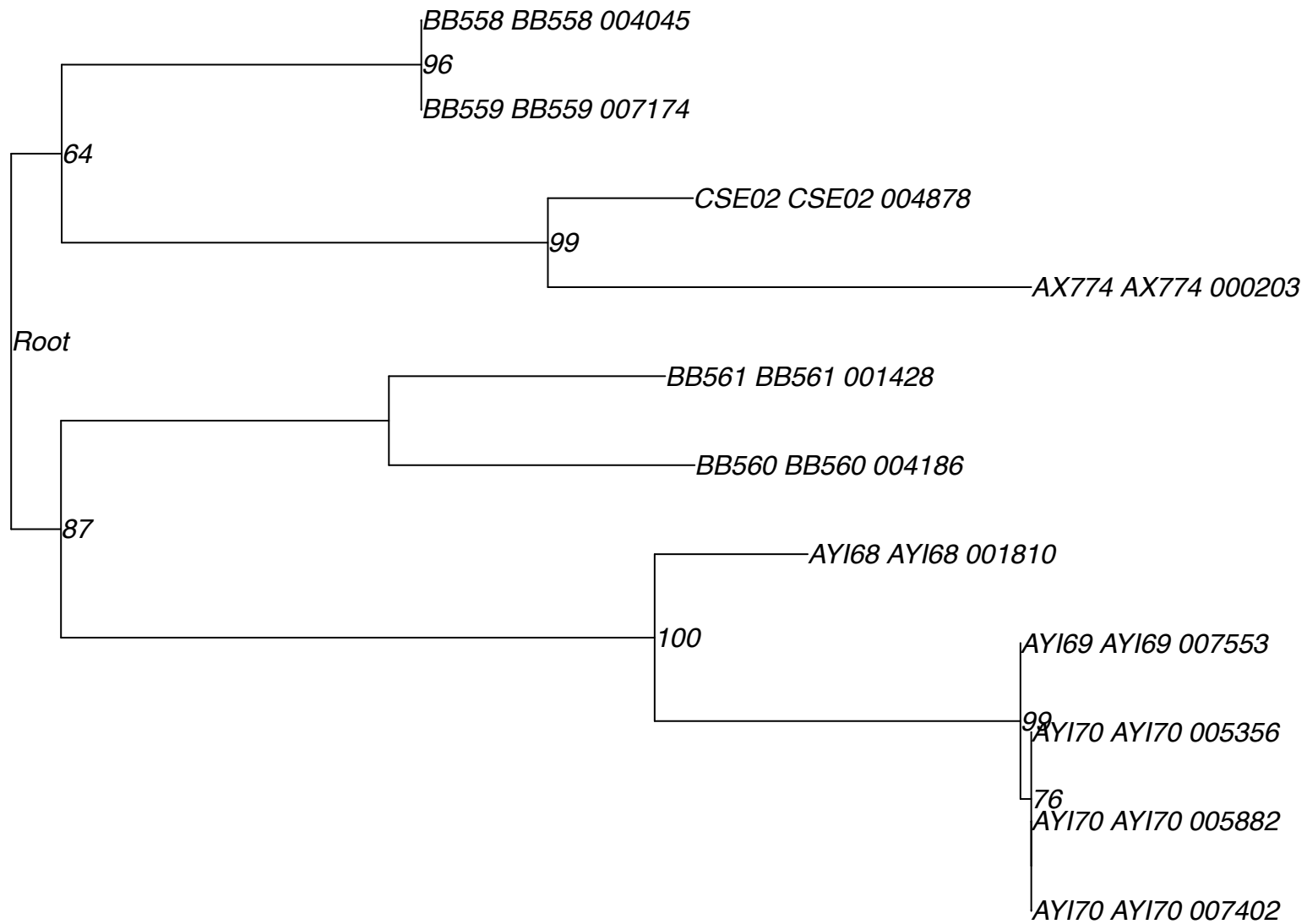

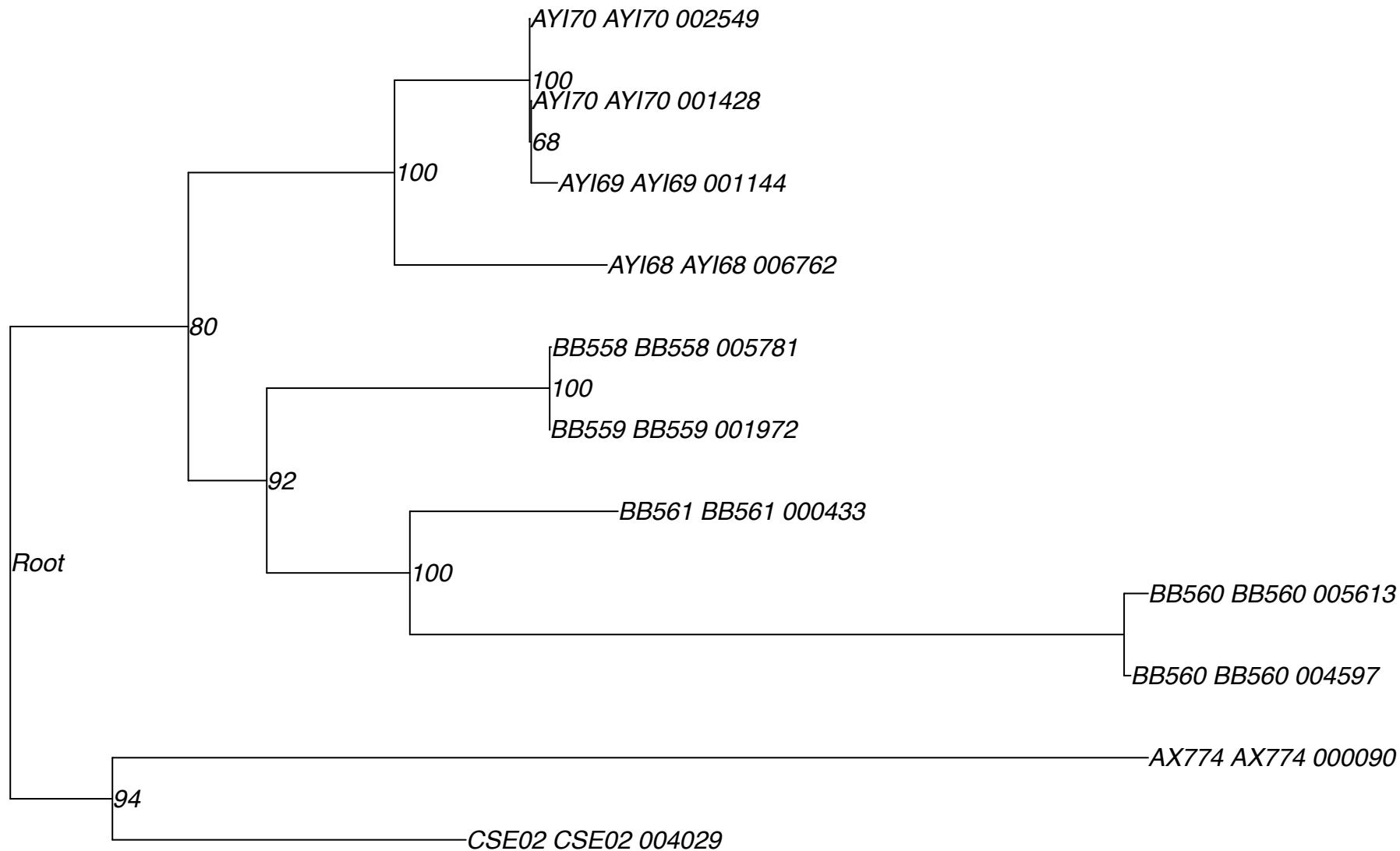

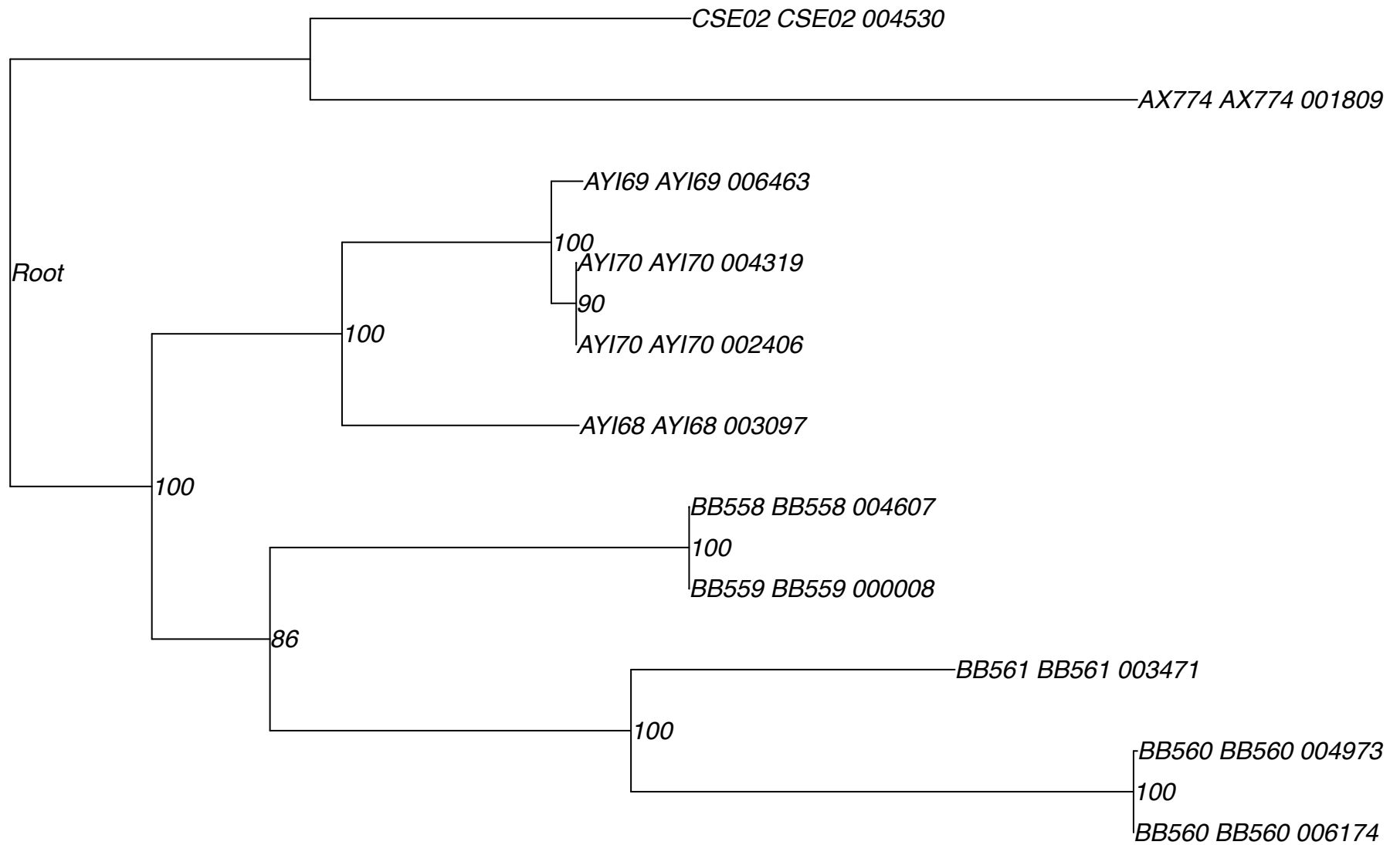

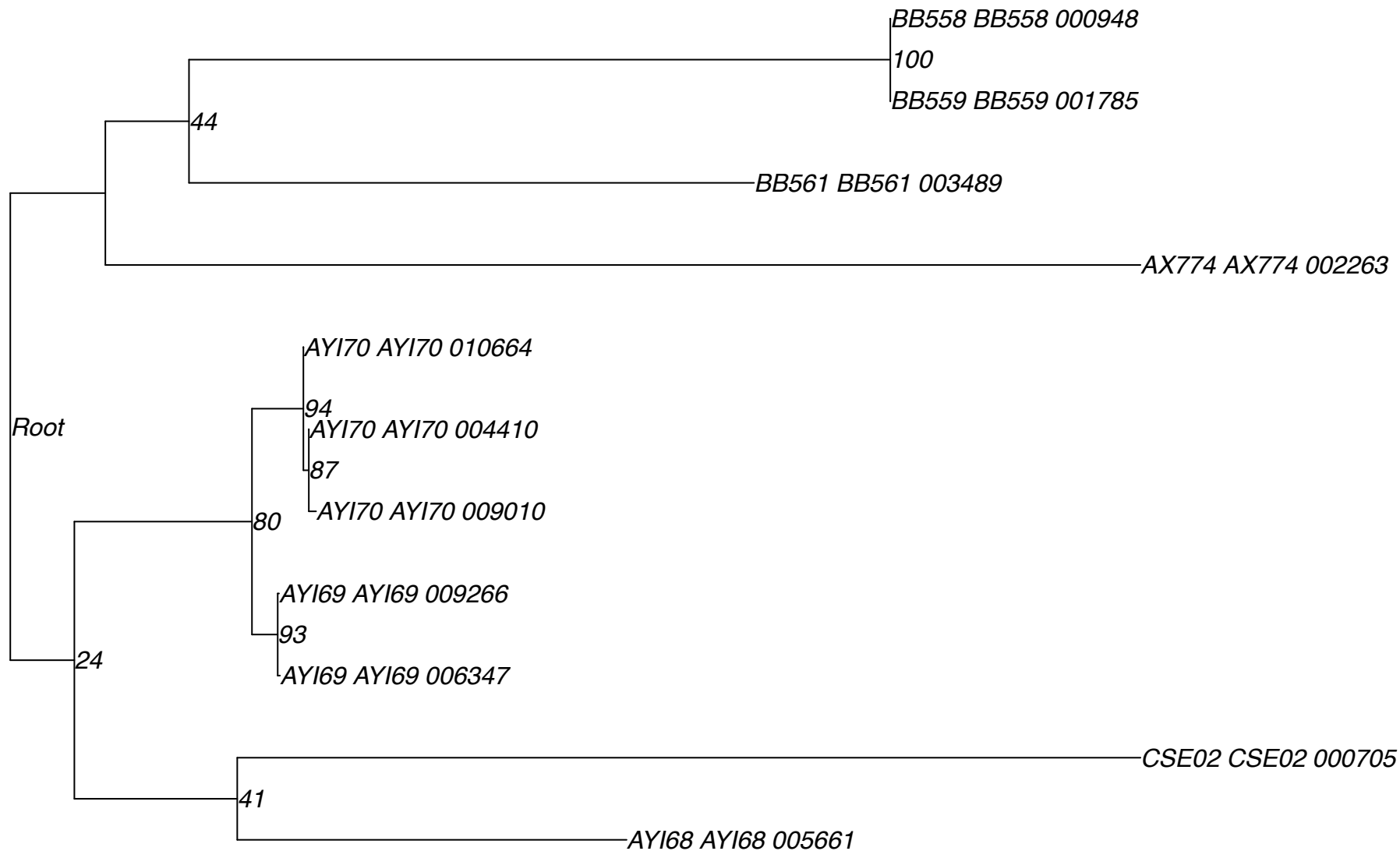

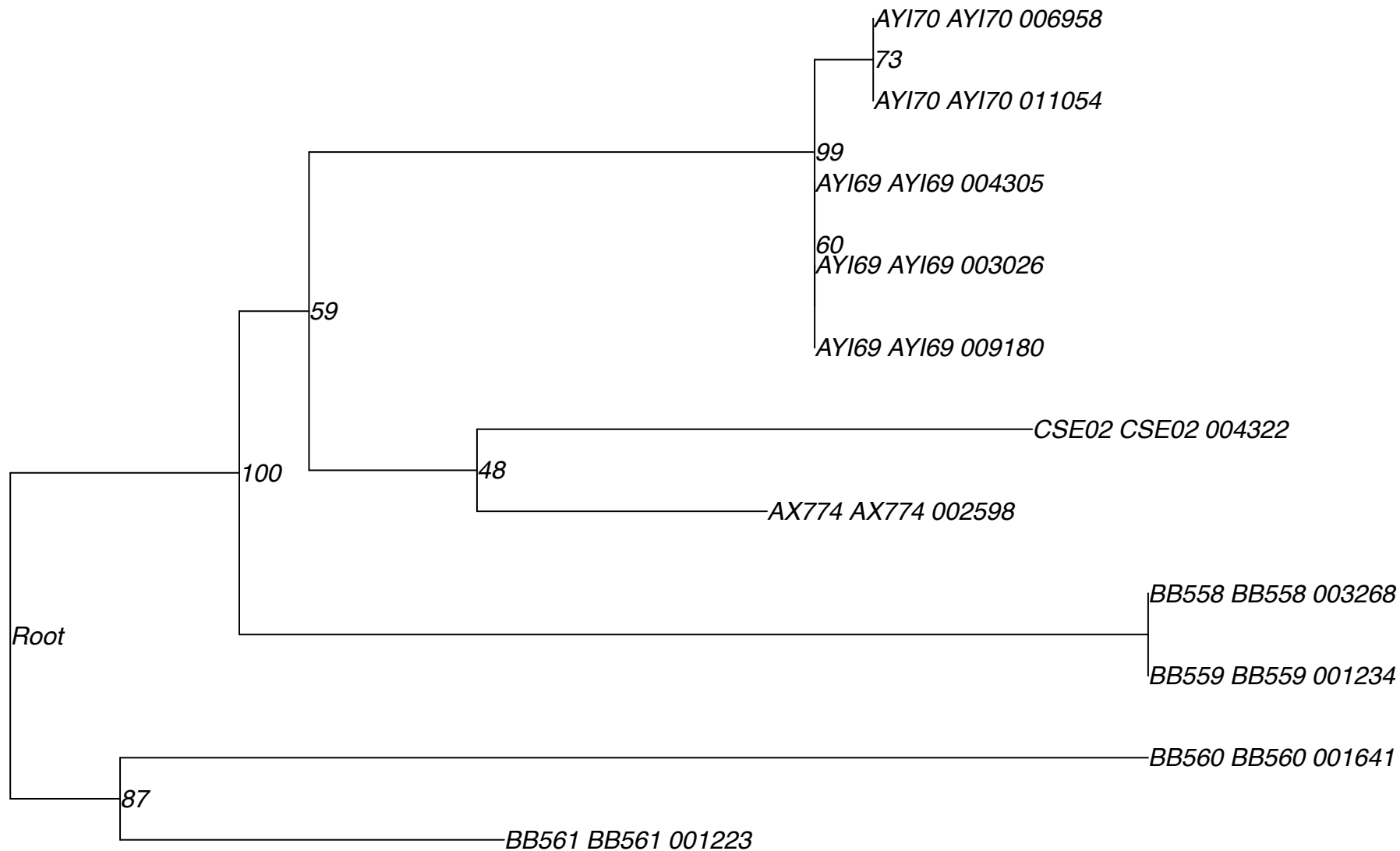

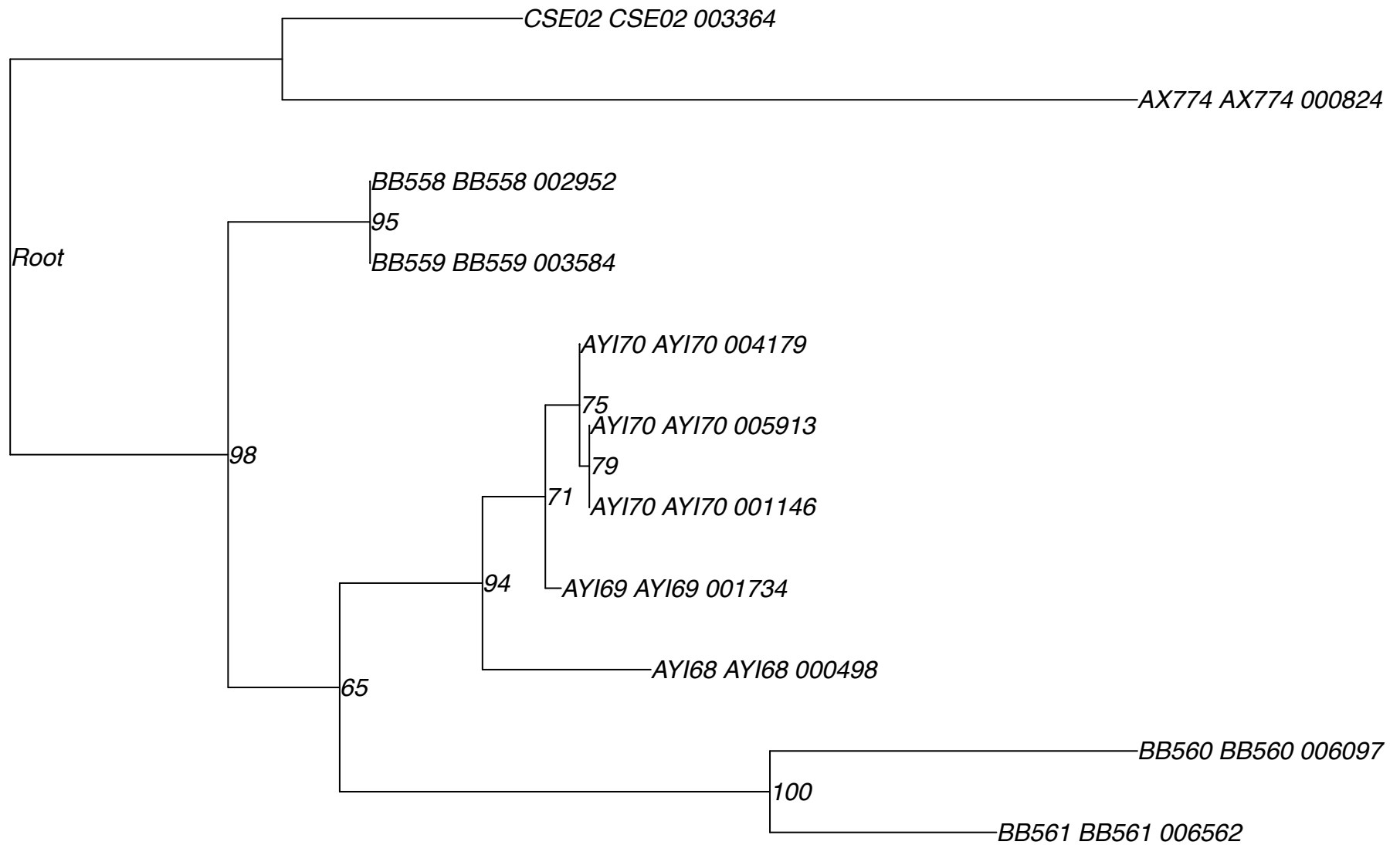

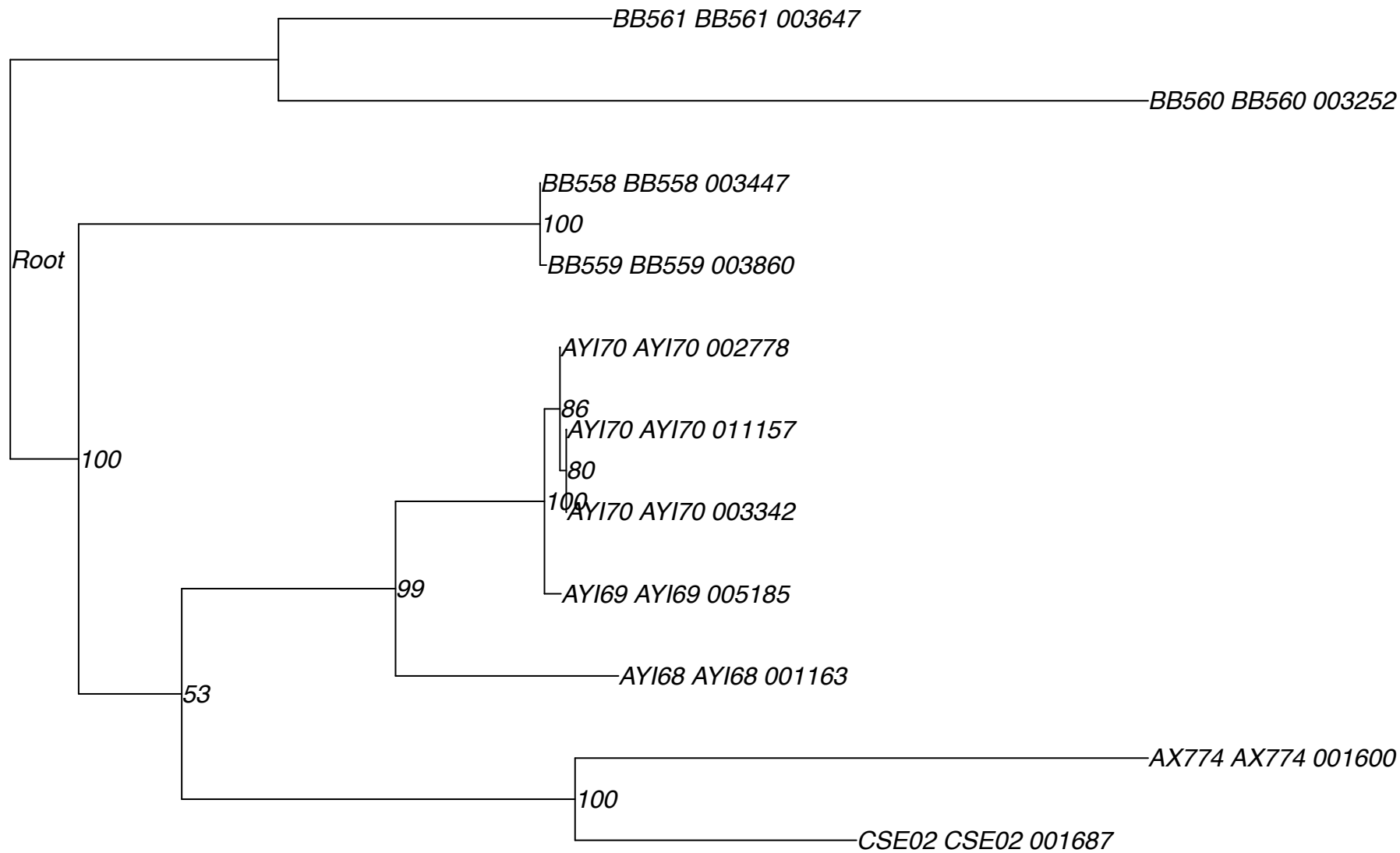

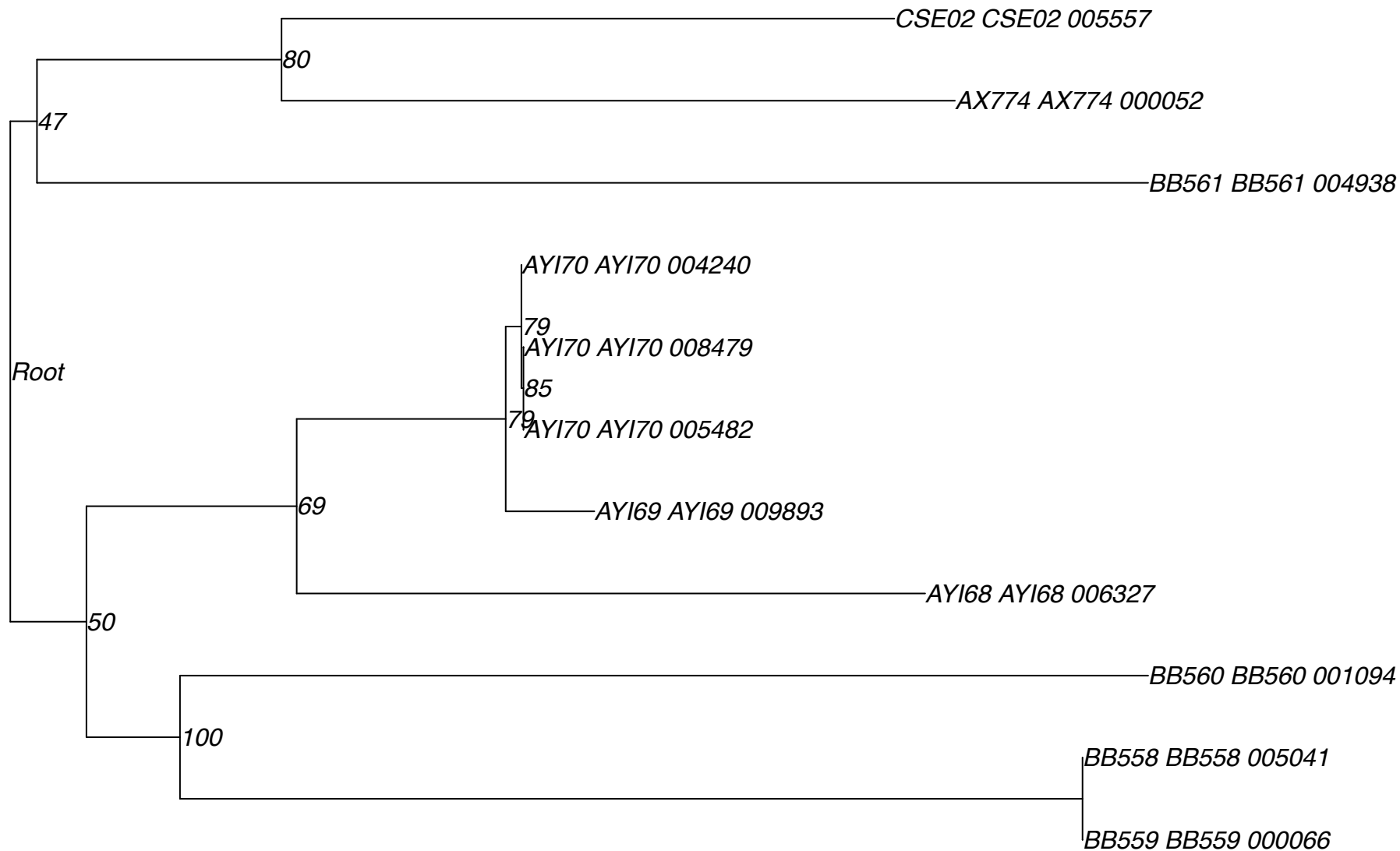

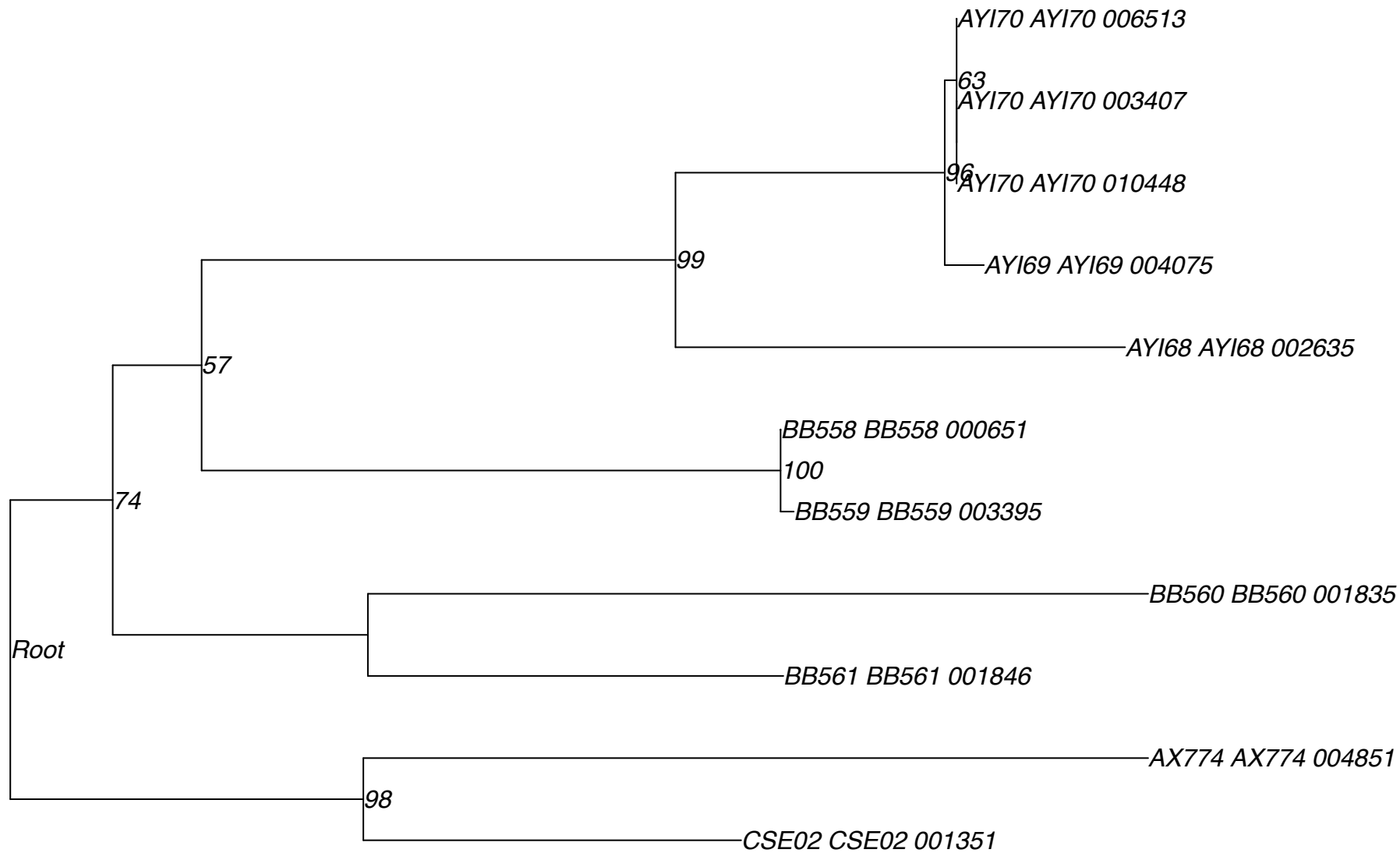

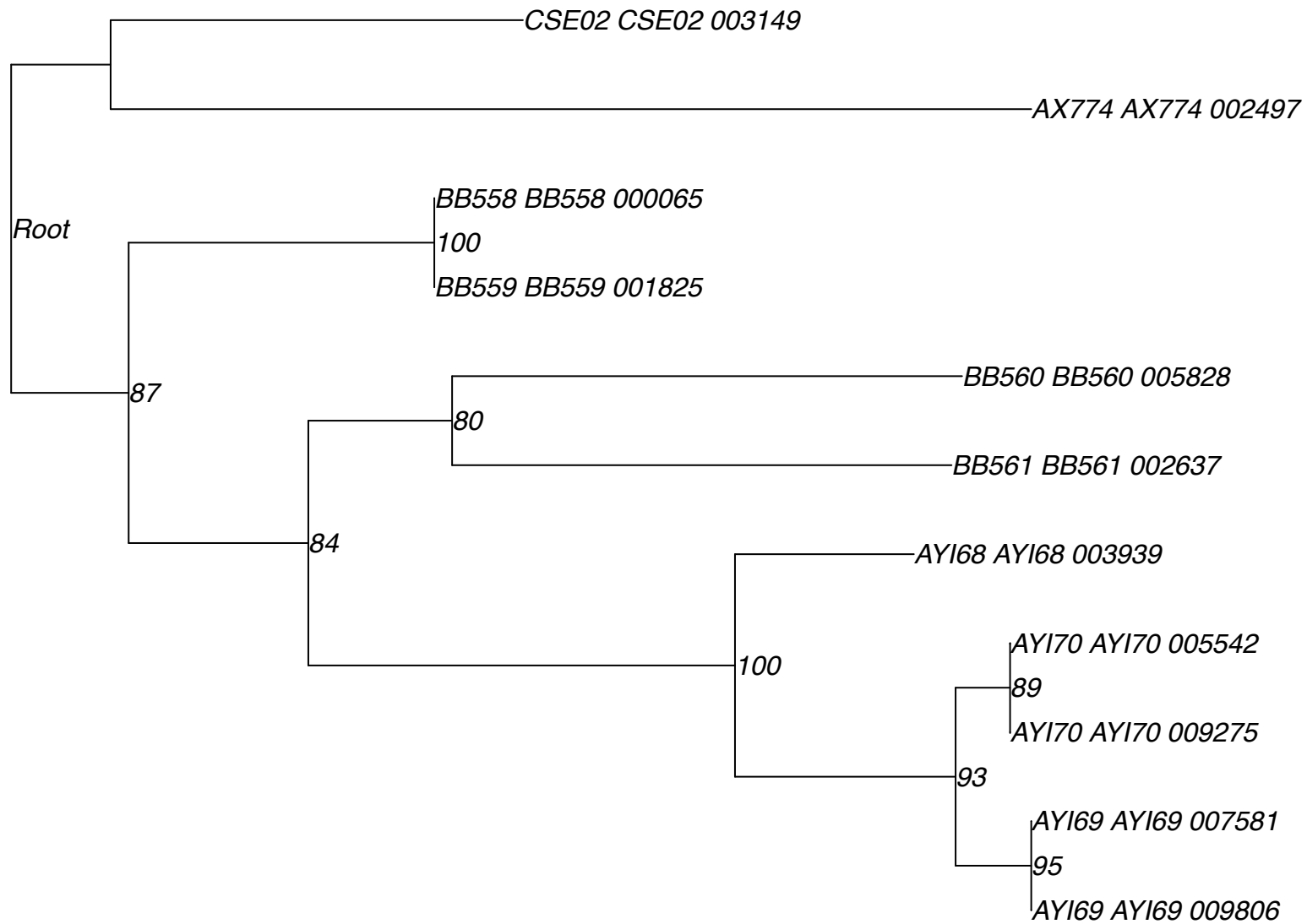

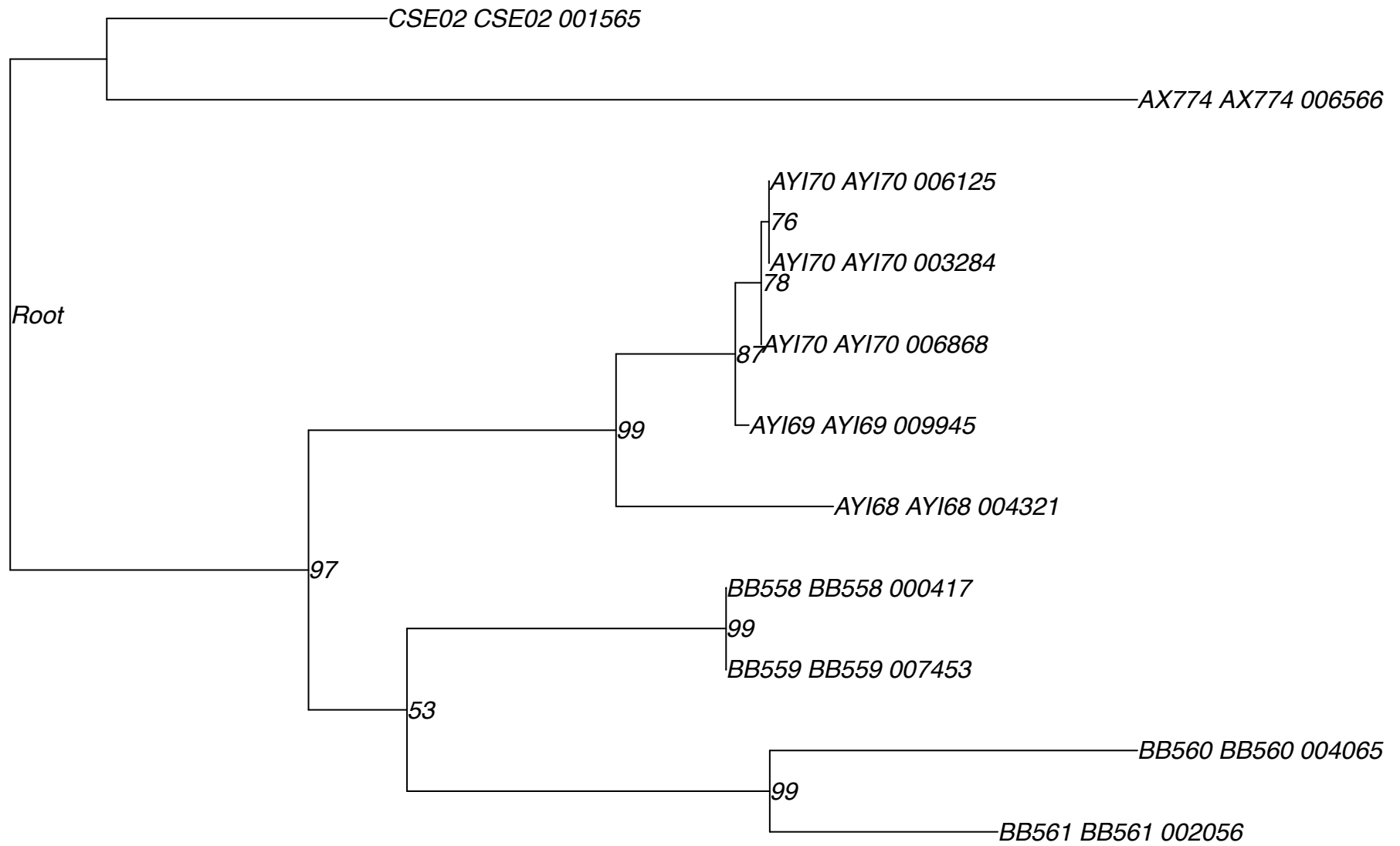

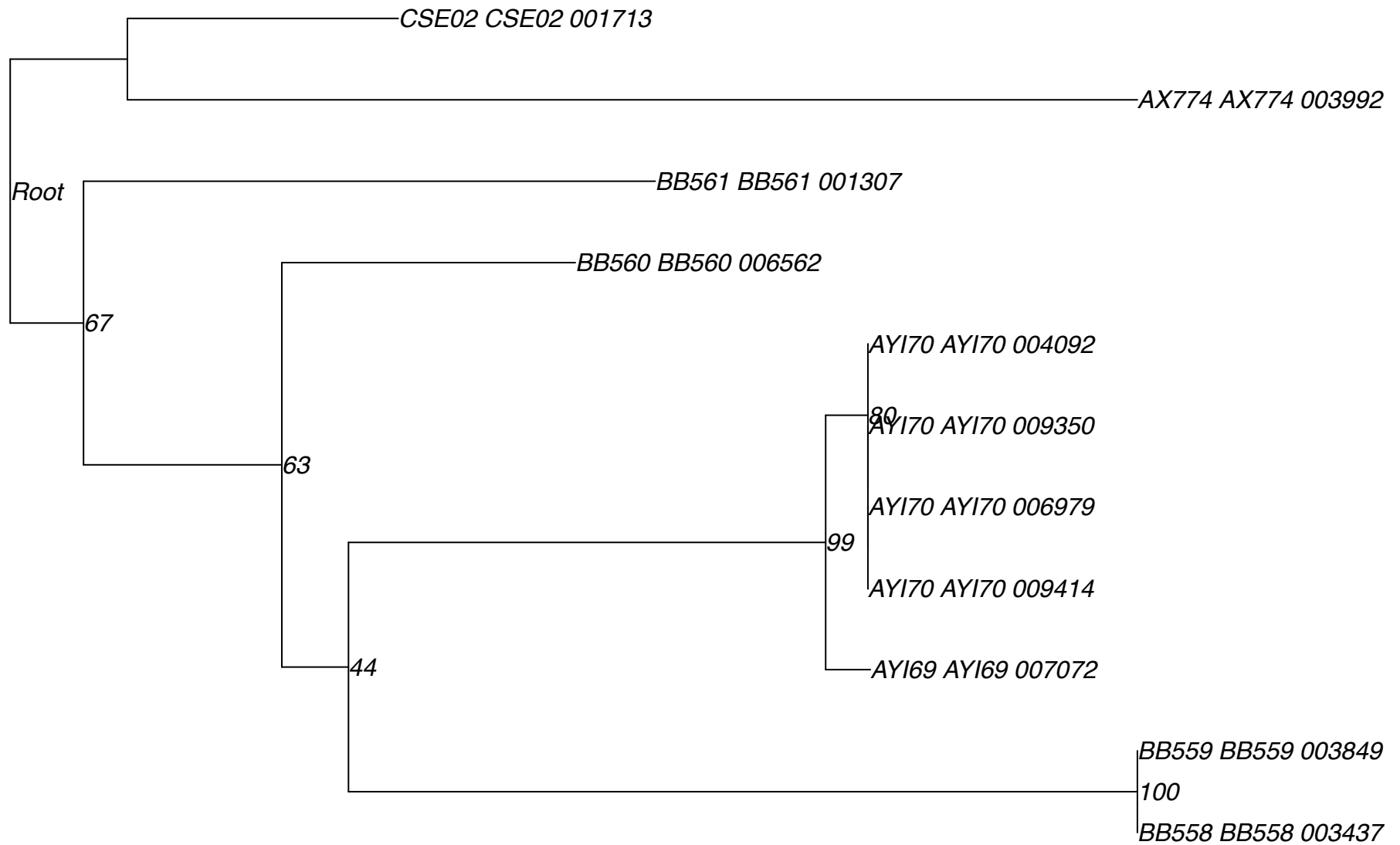

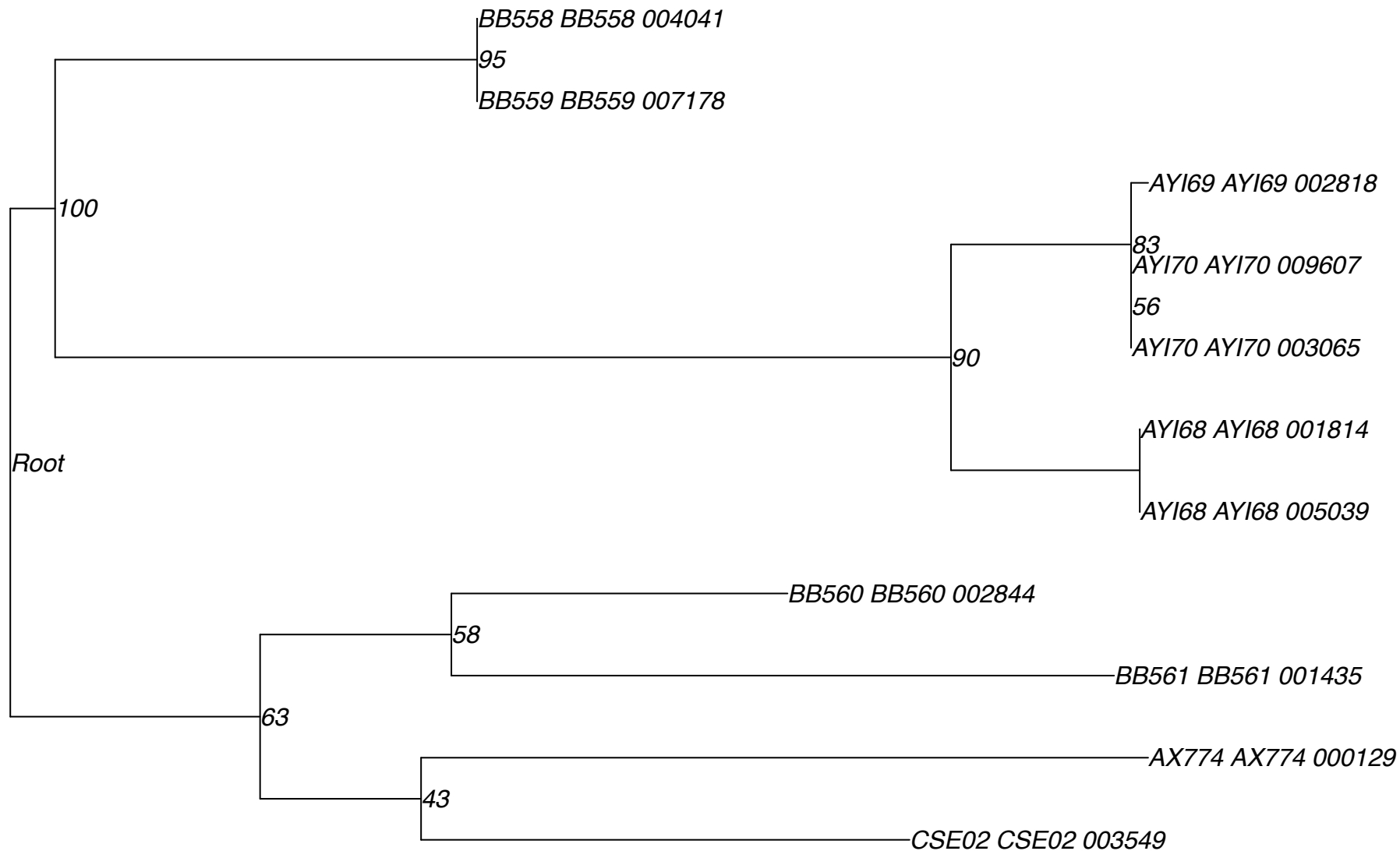

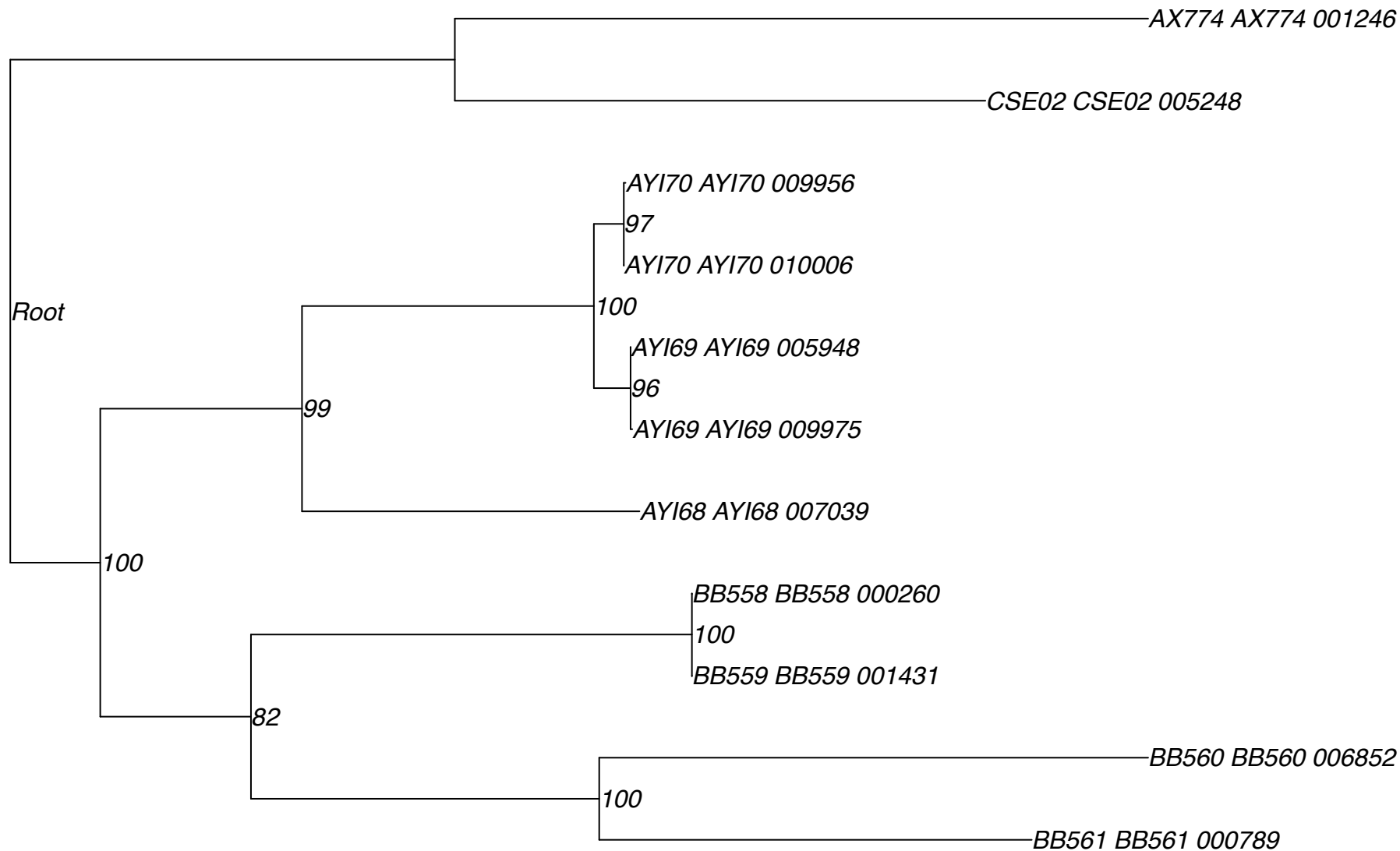

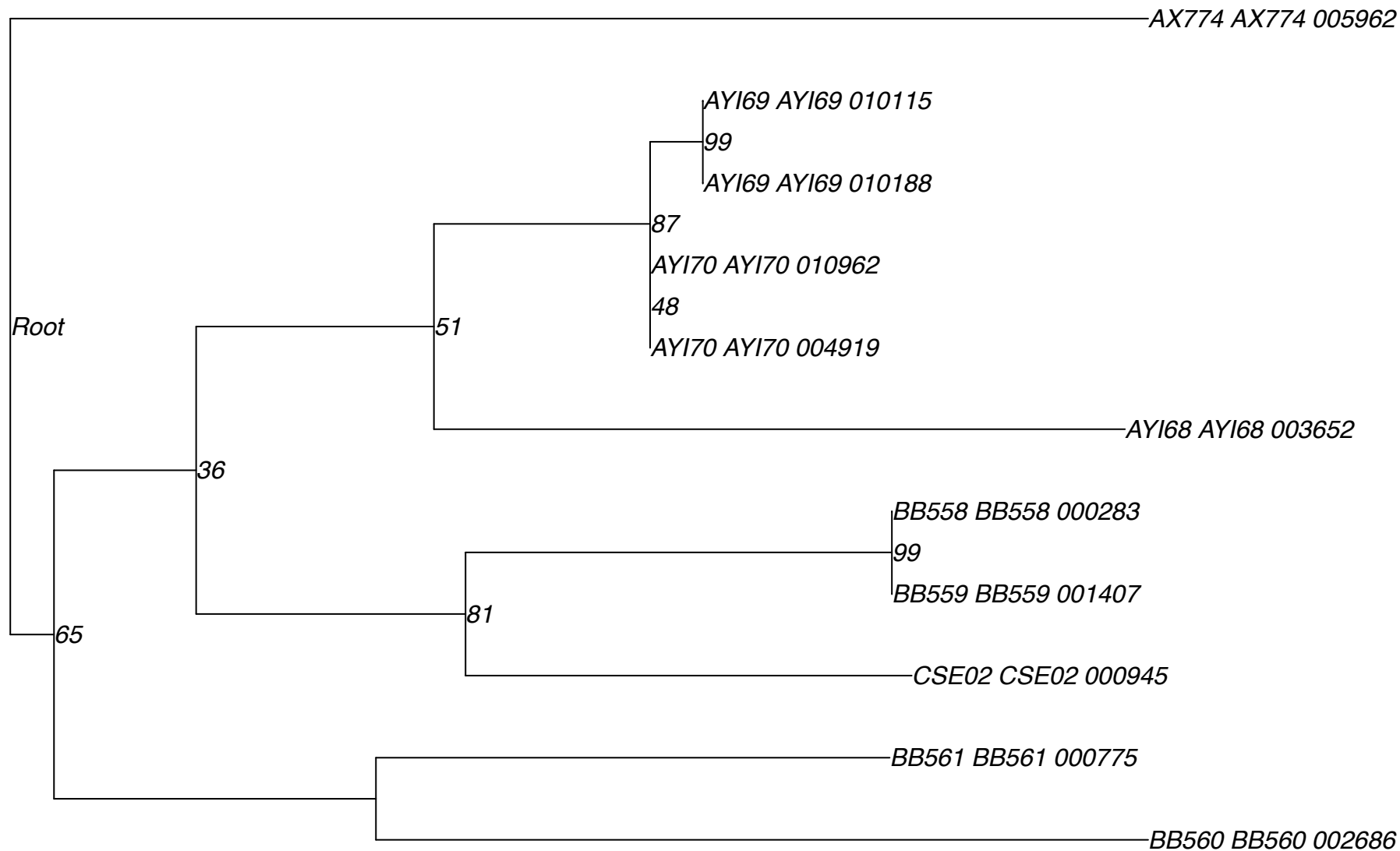

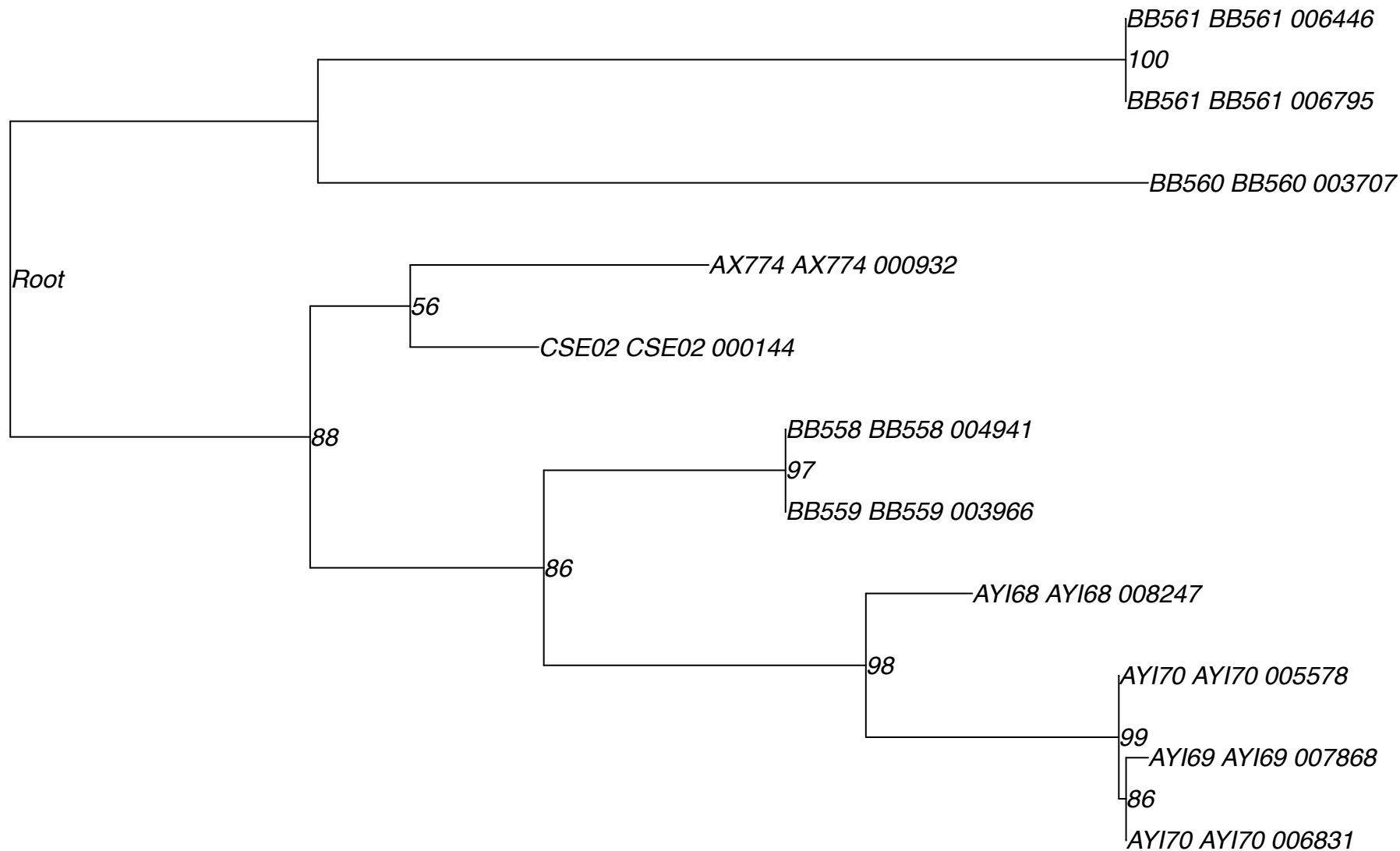

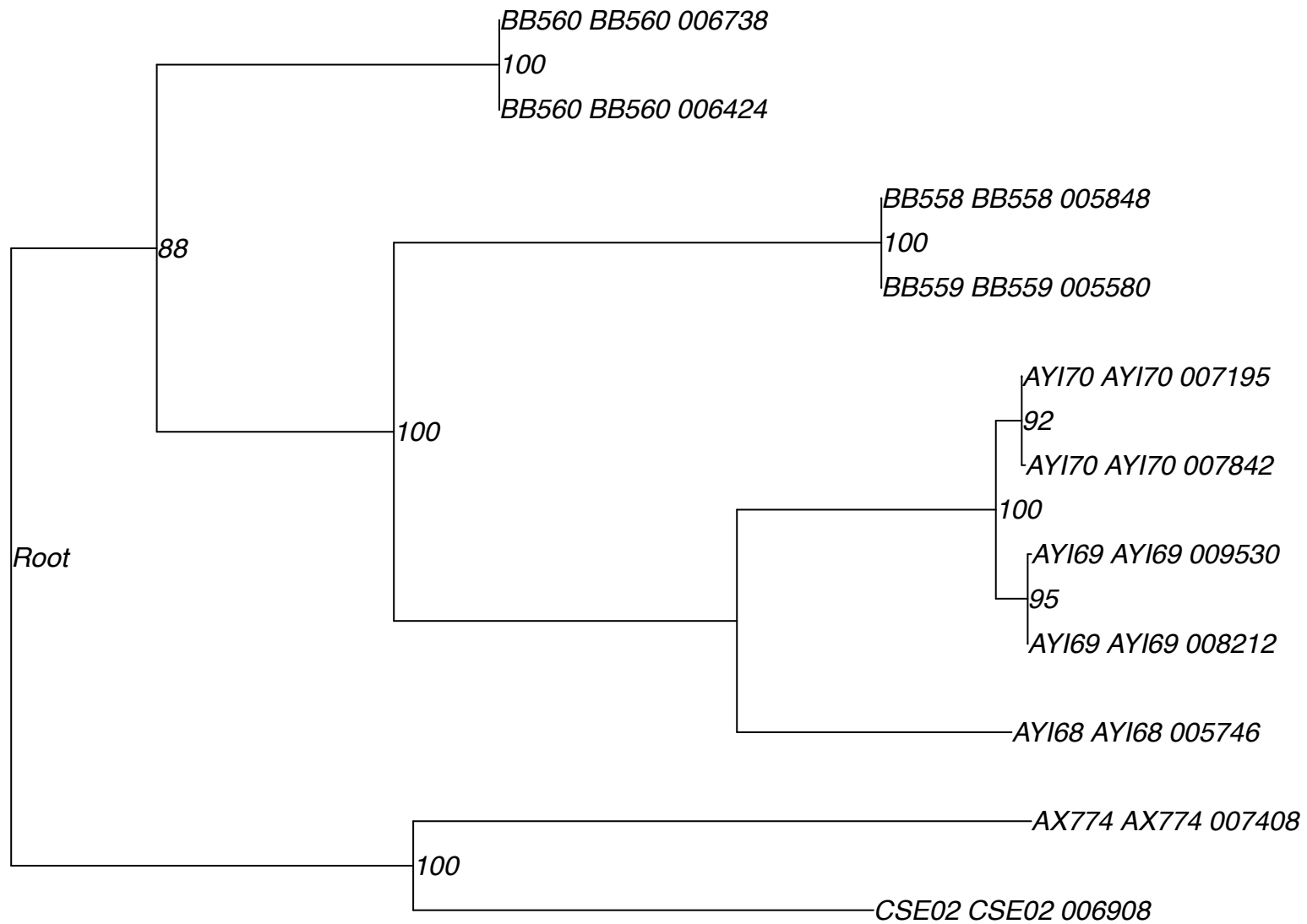

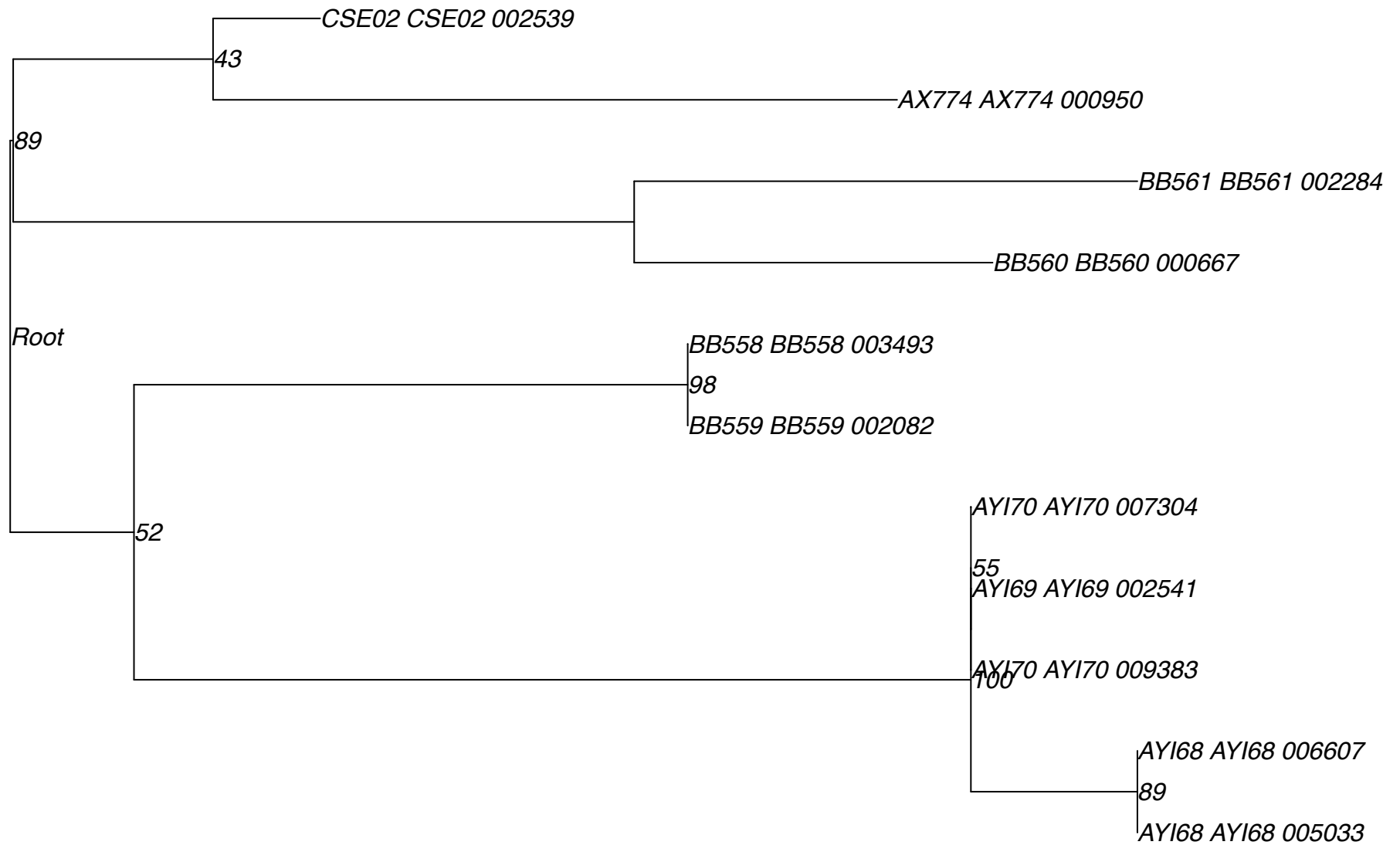

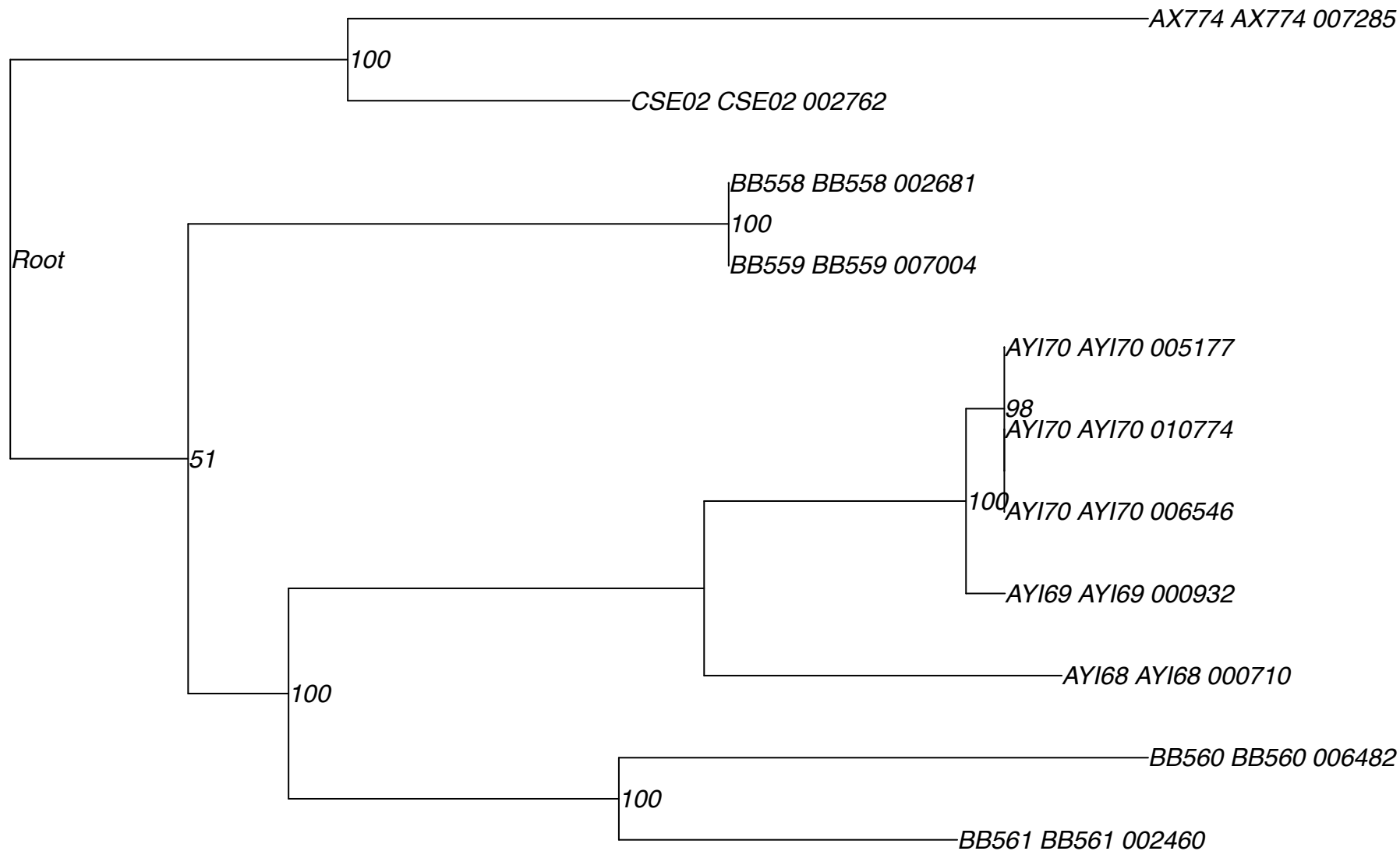

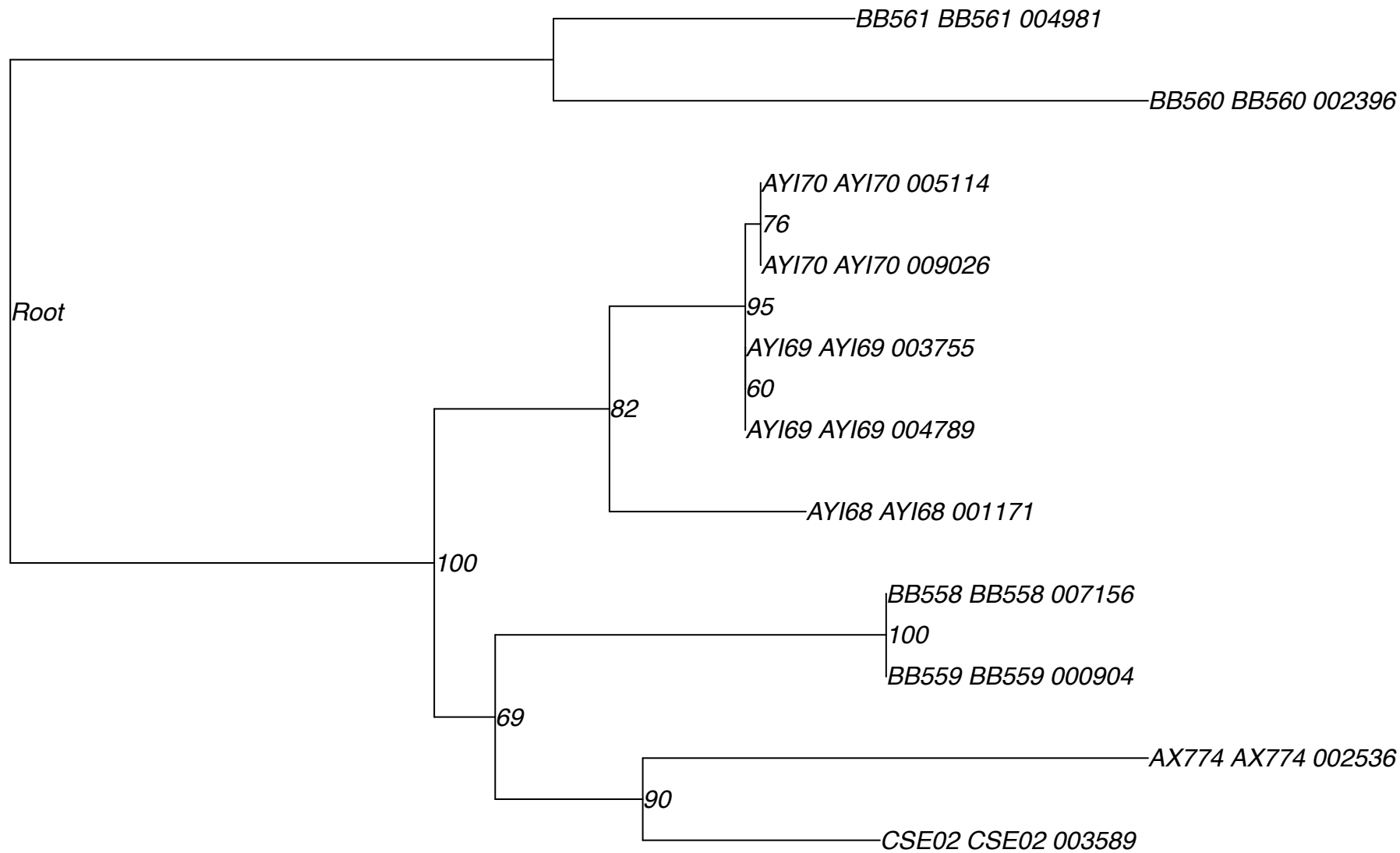

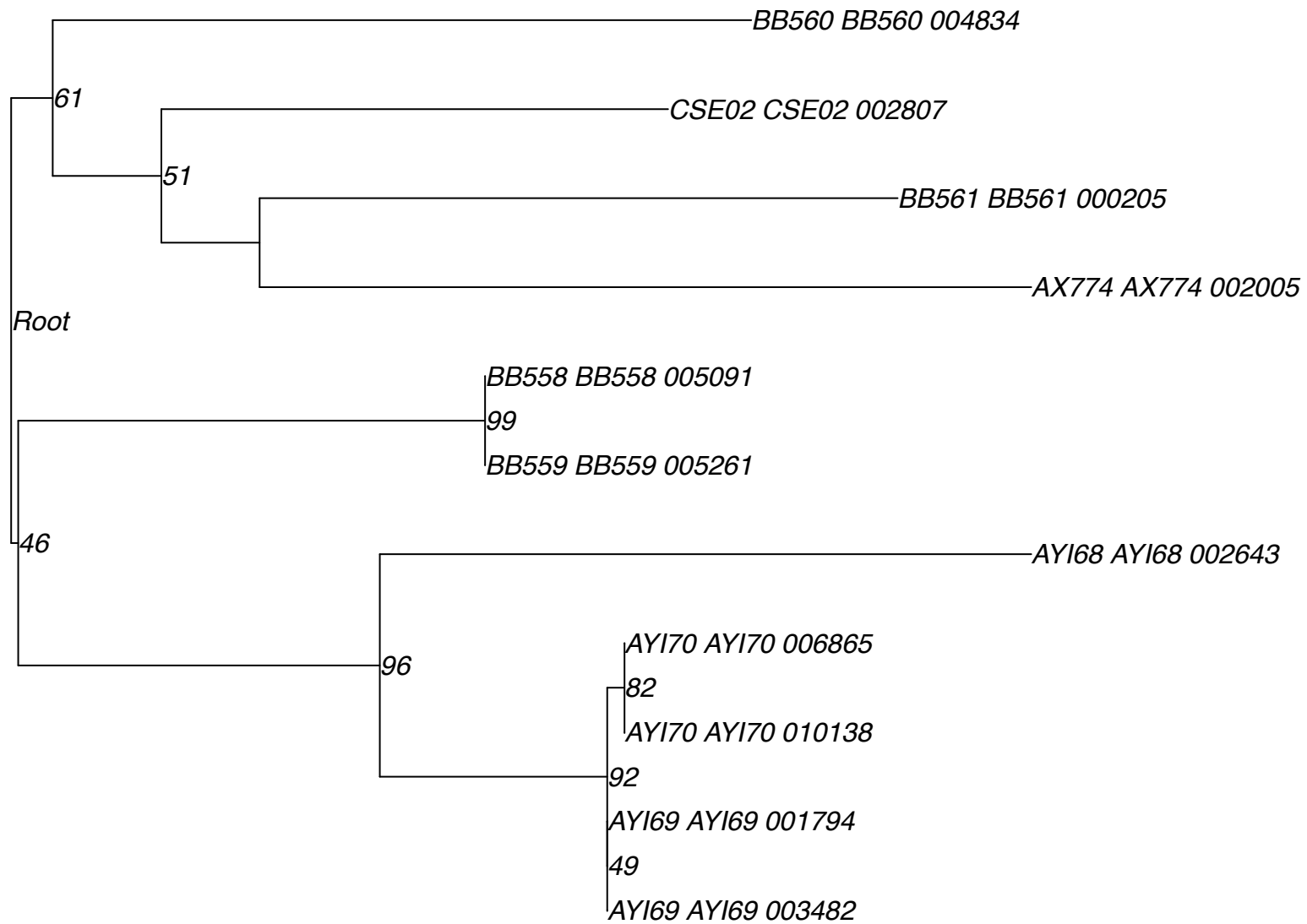

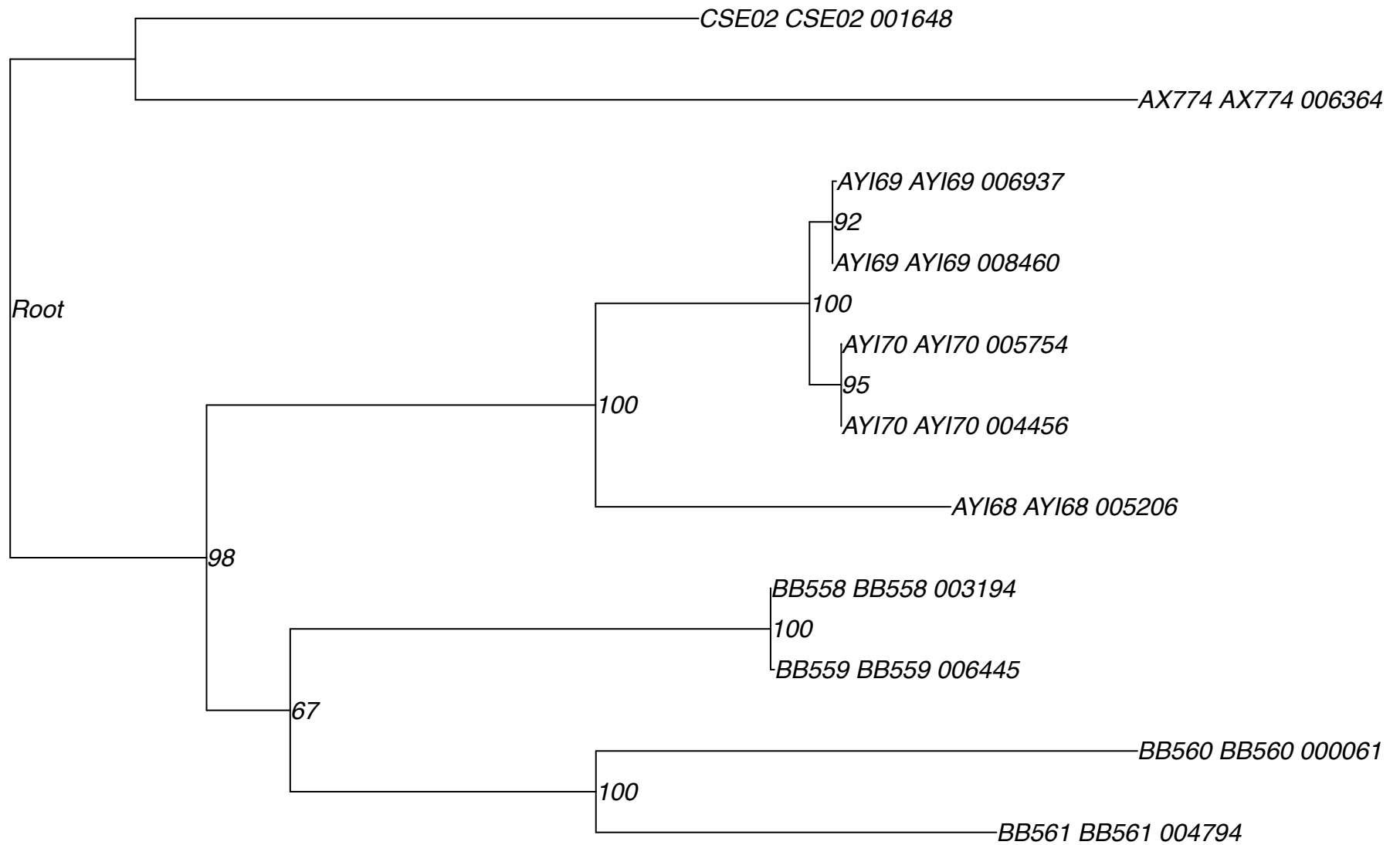

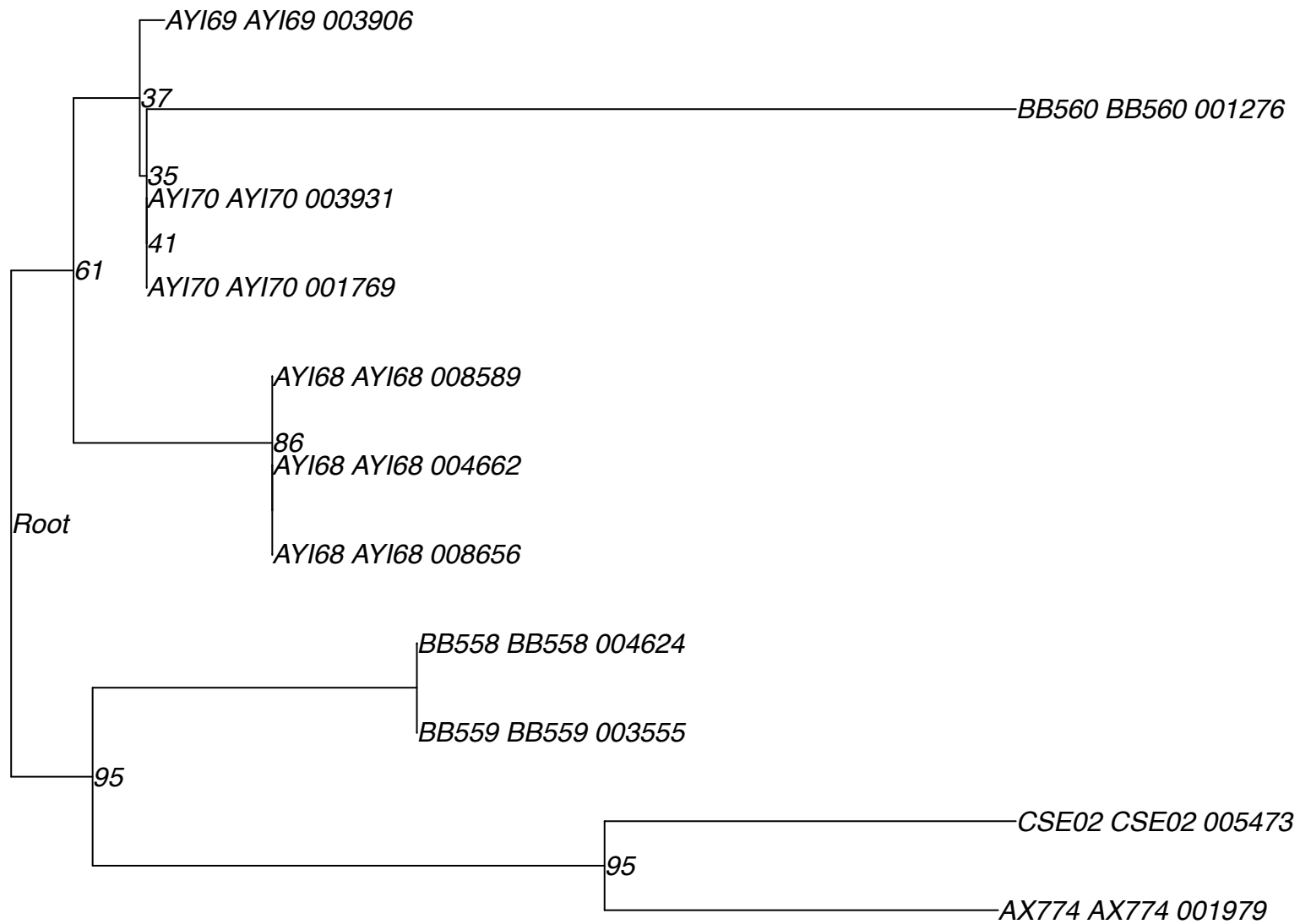

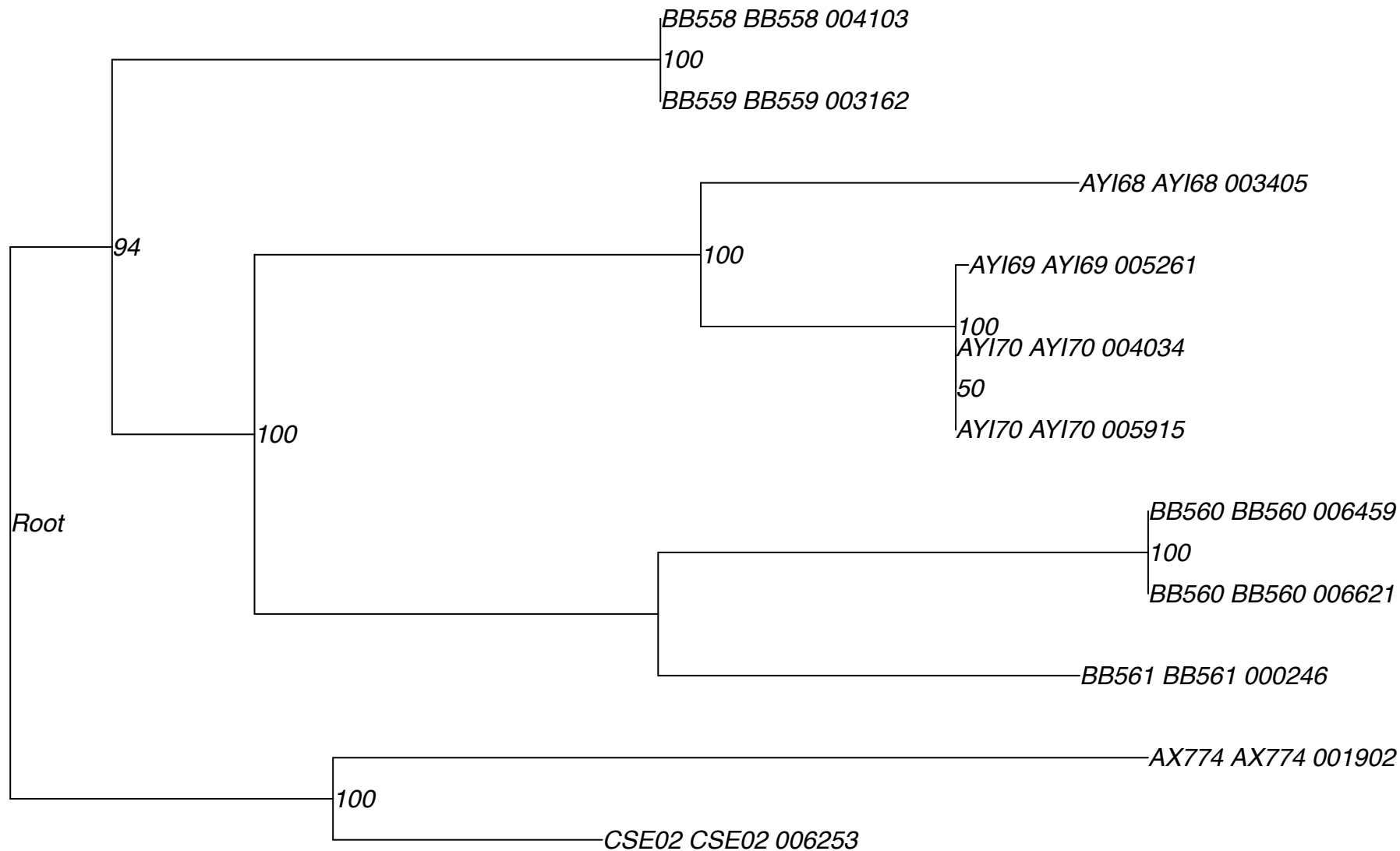

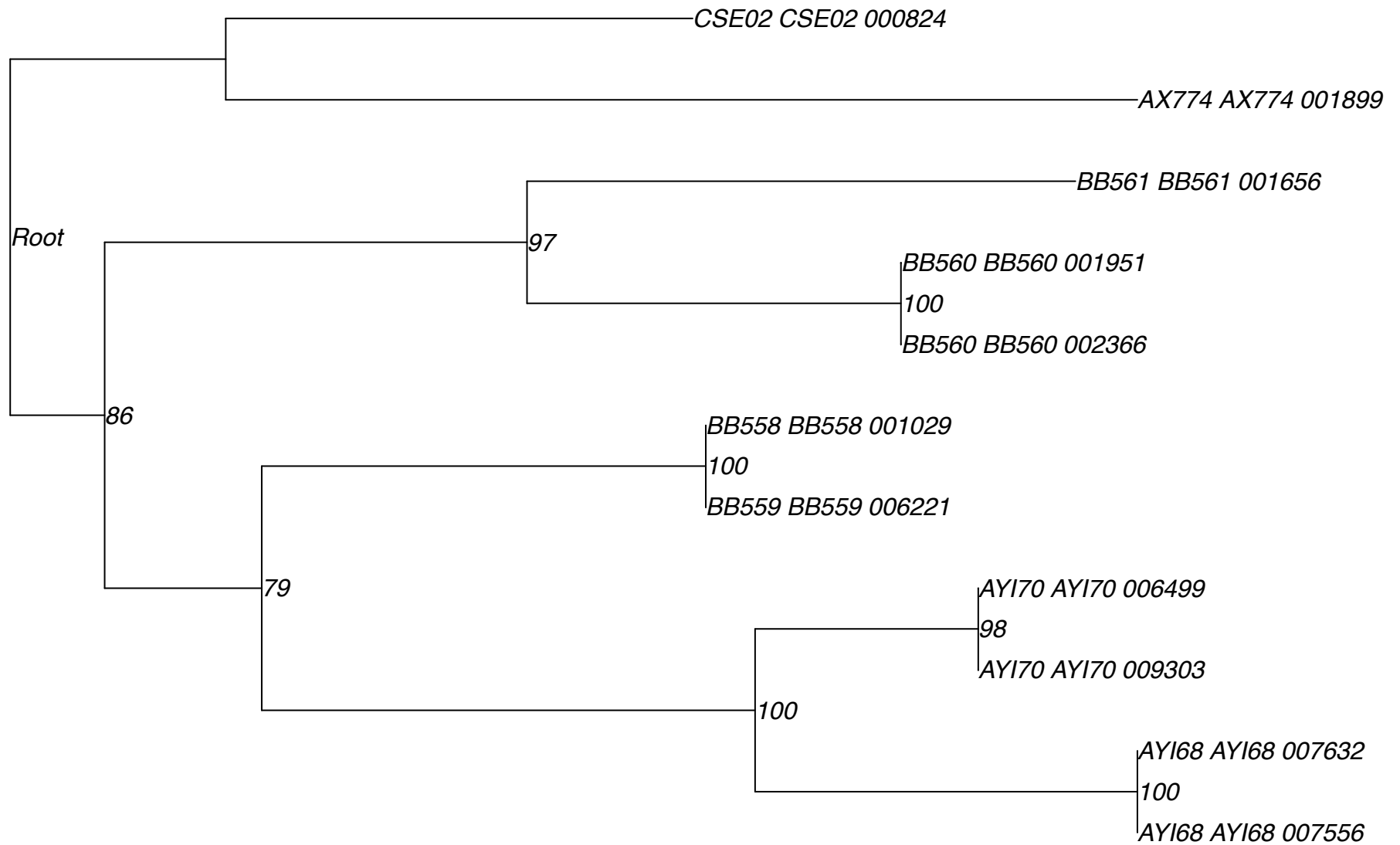

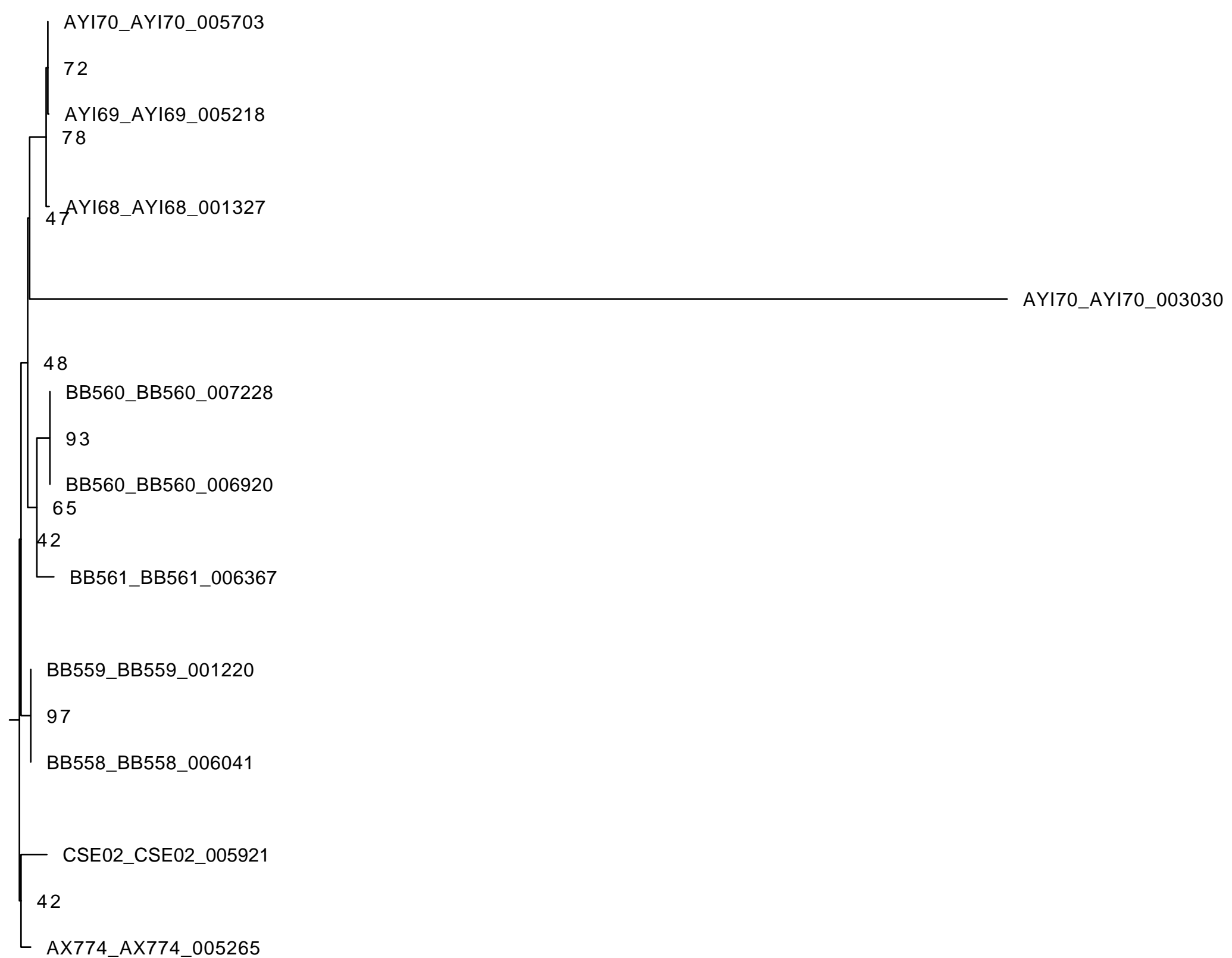

0.8

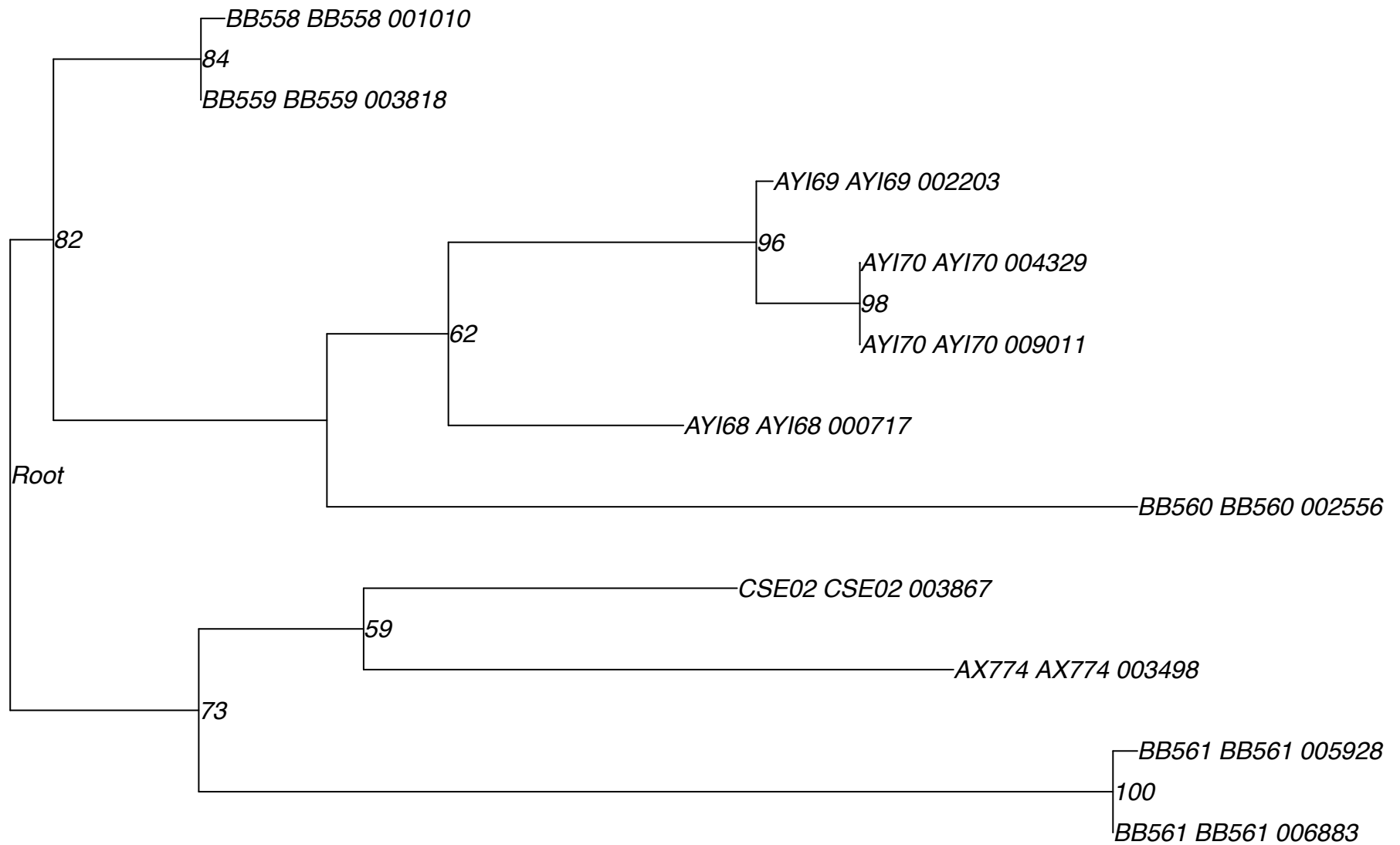

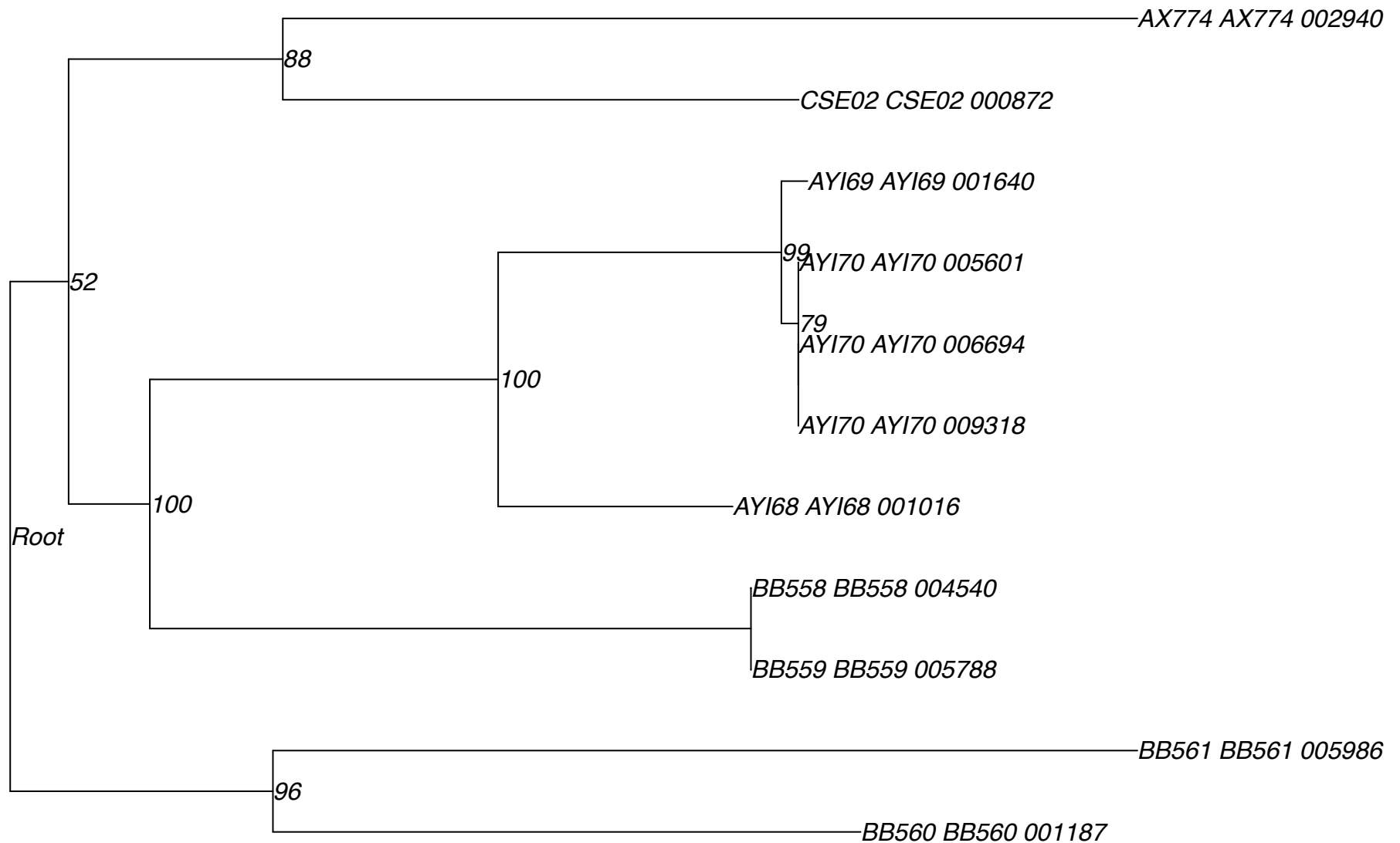

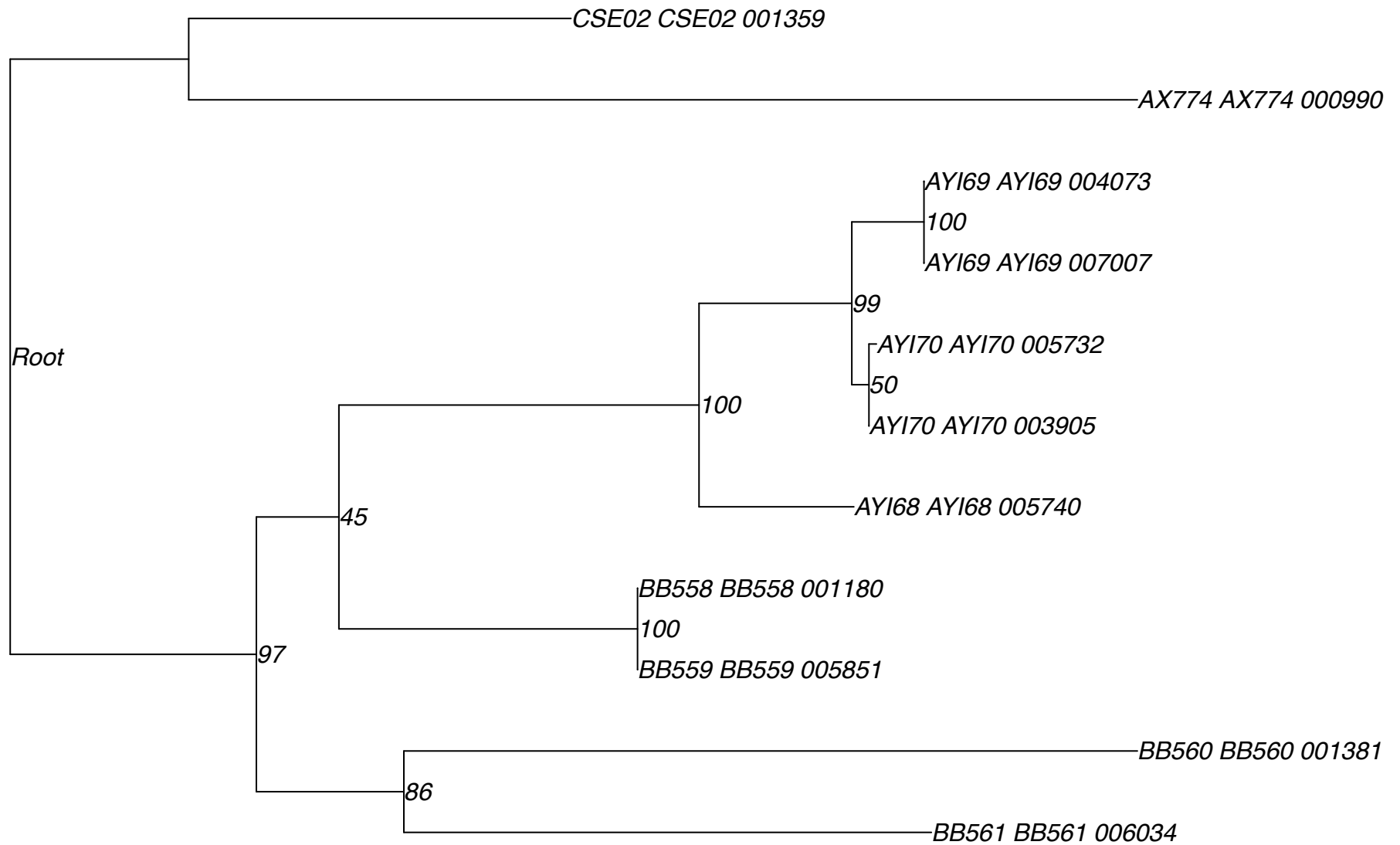

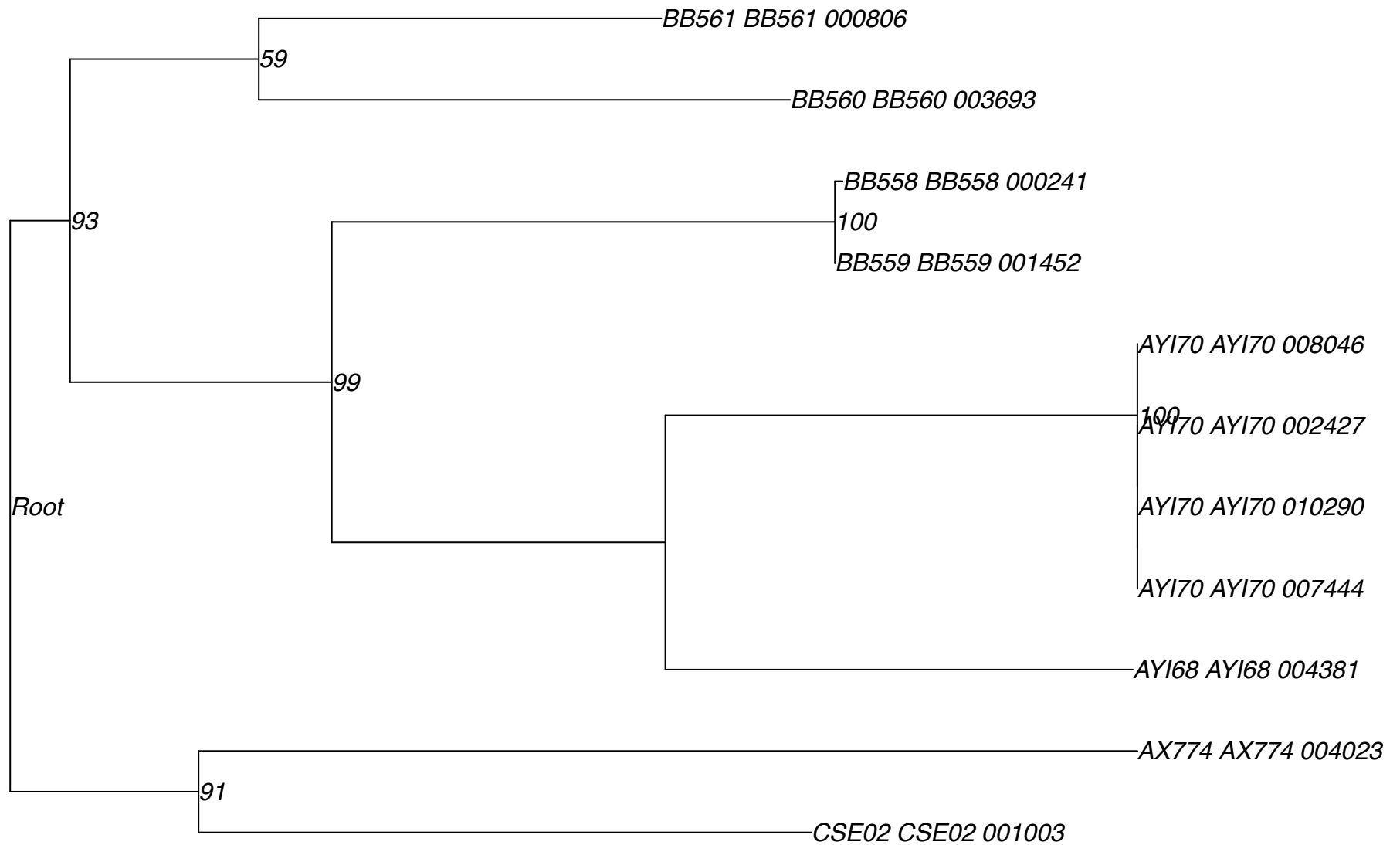

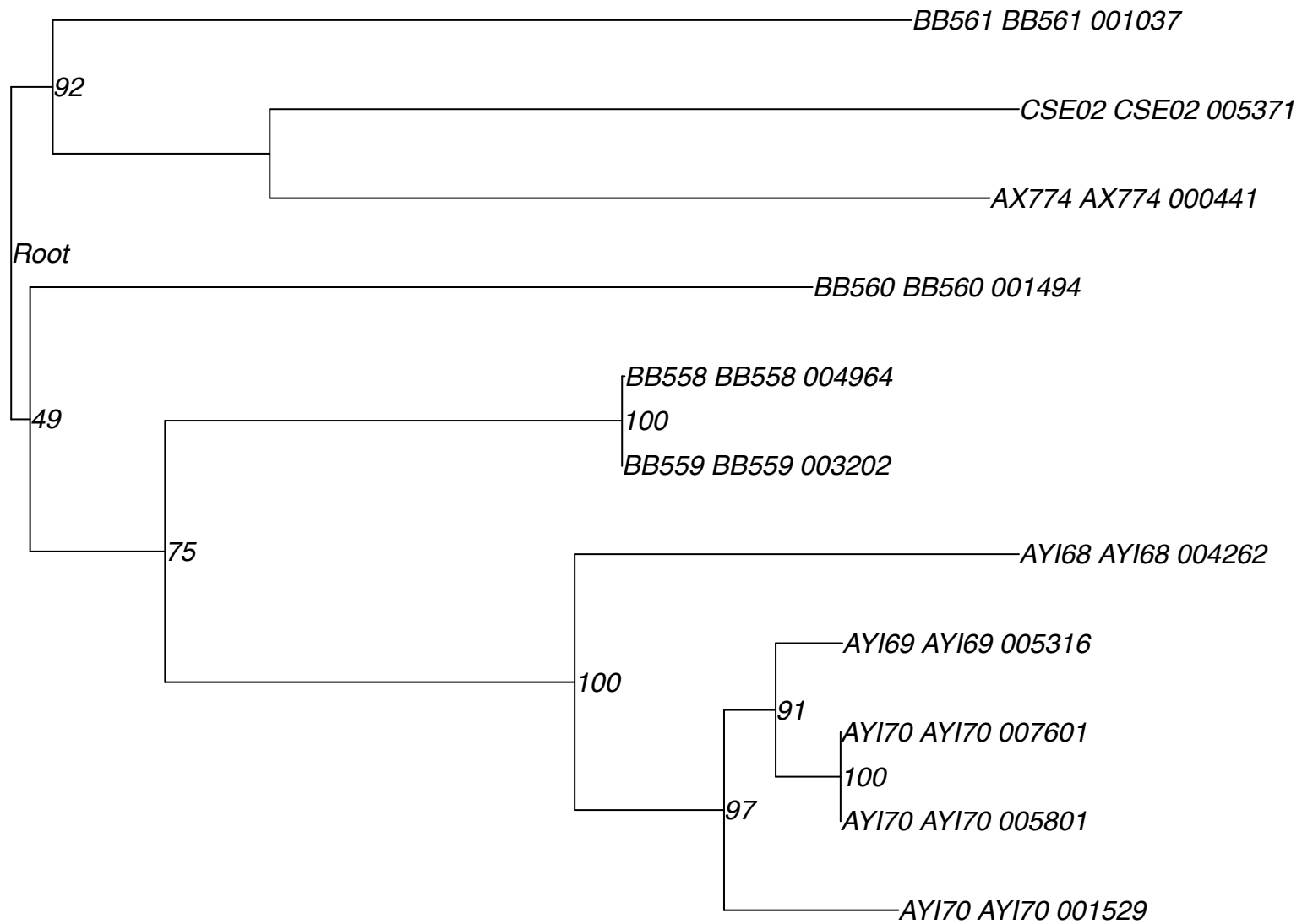

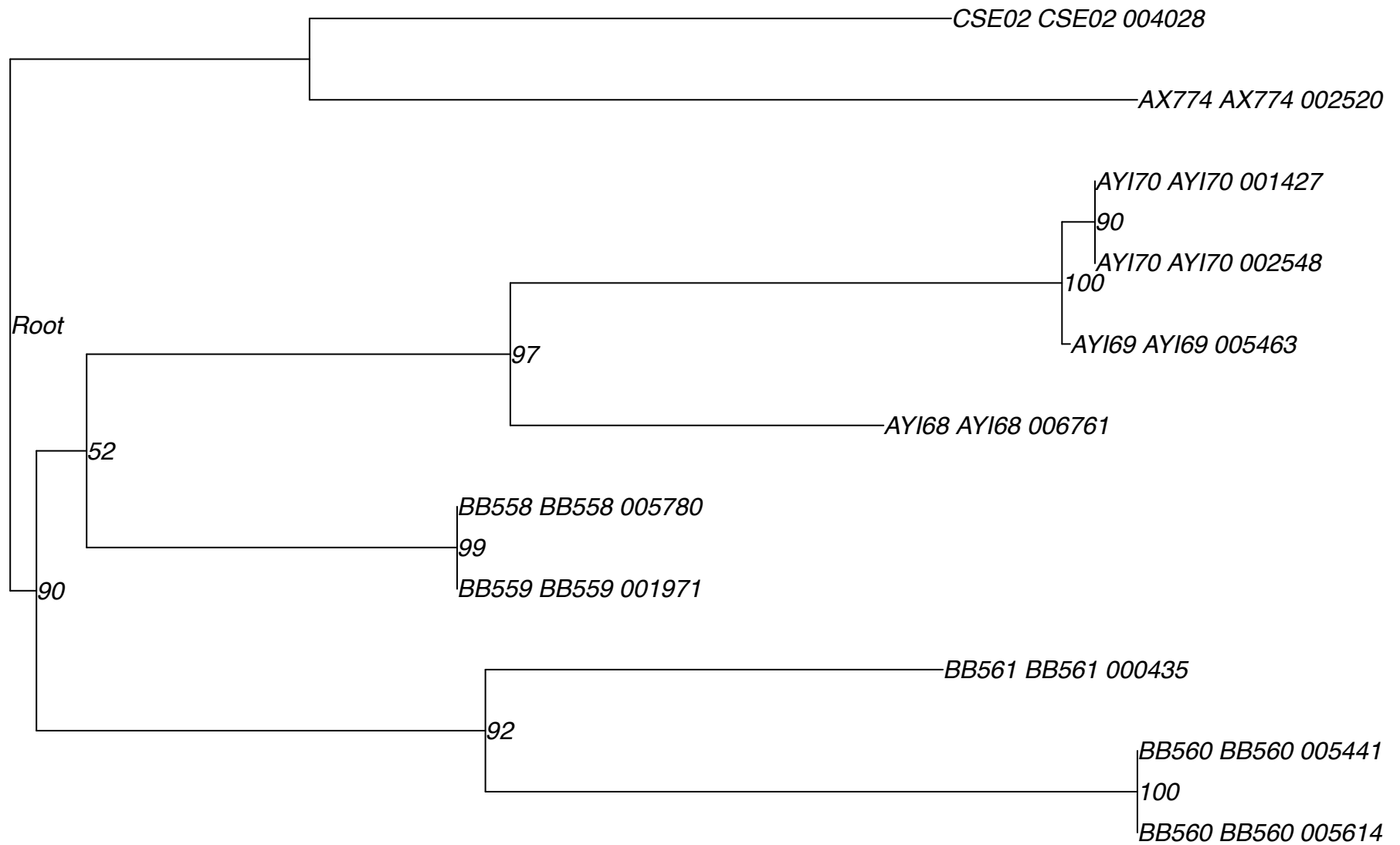

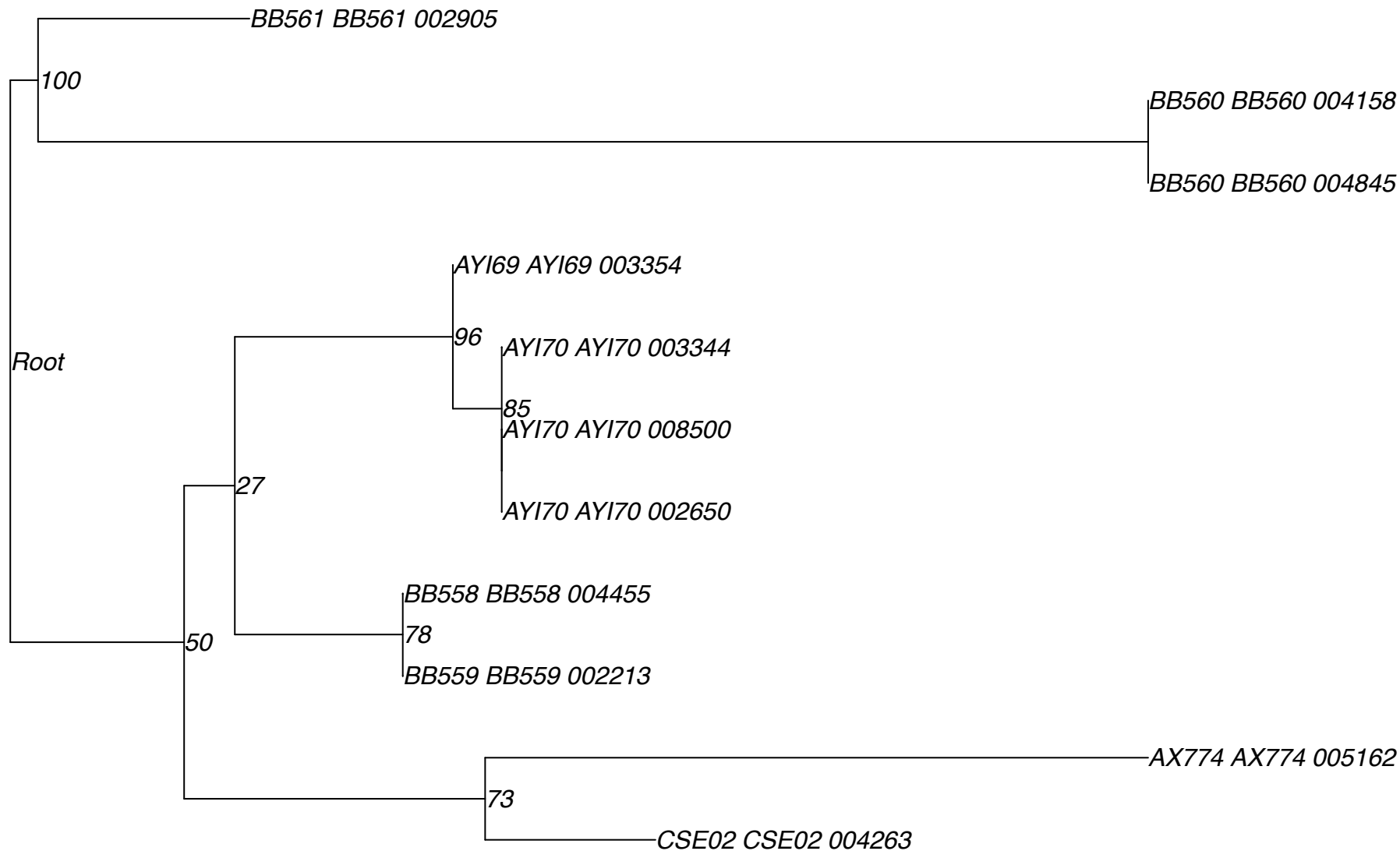

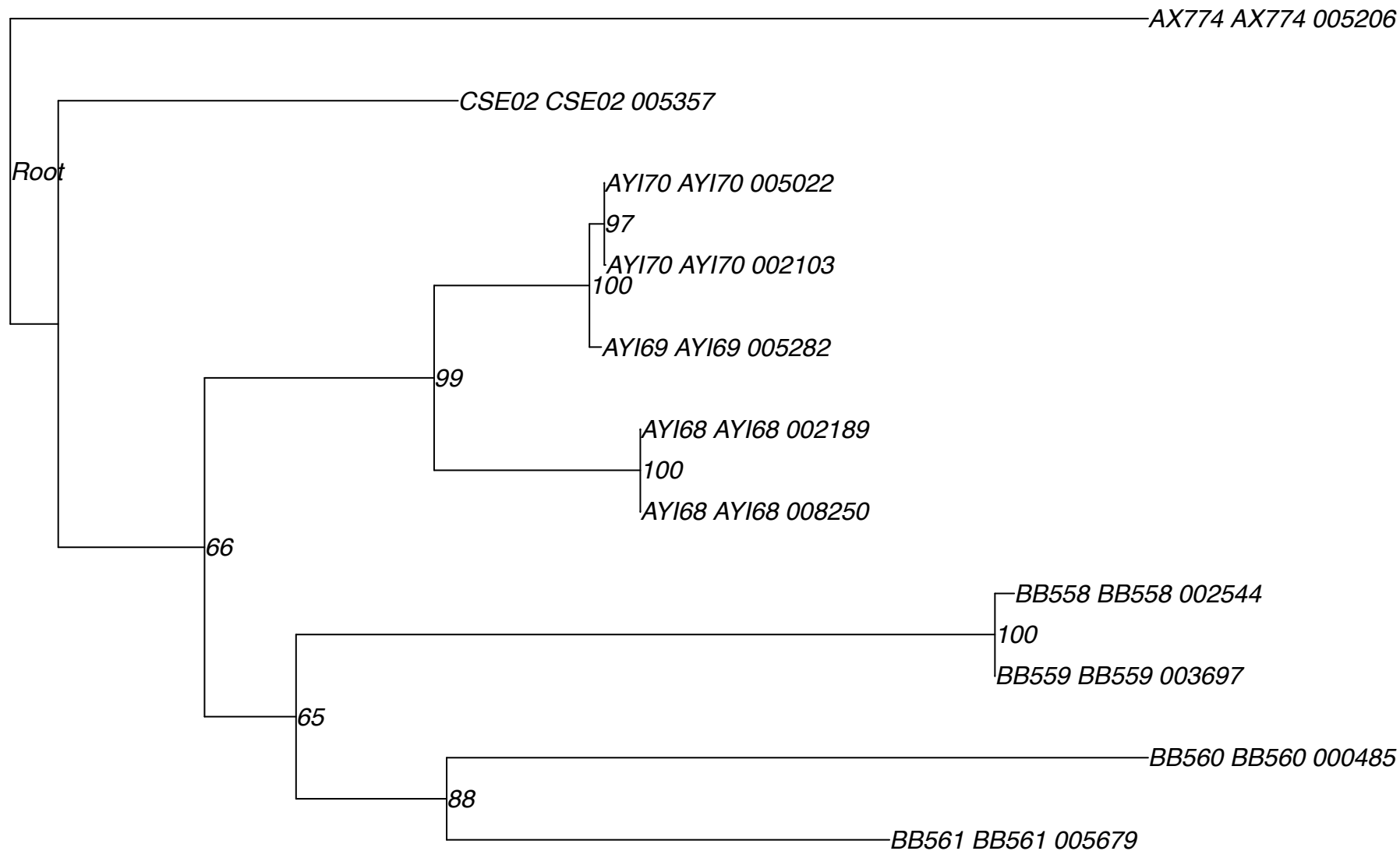

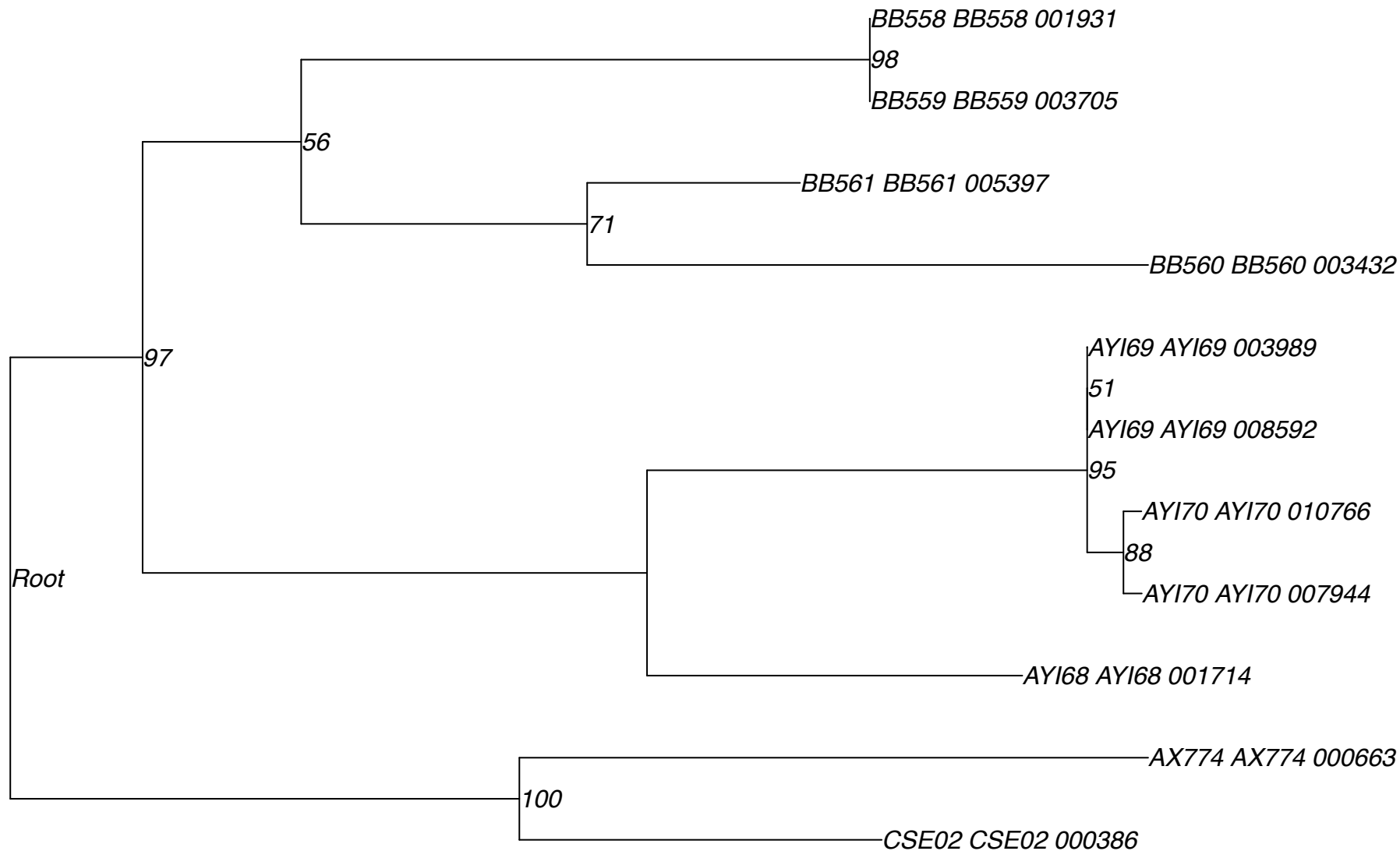

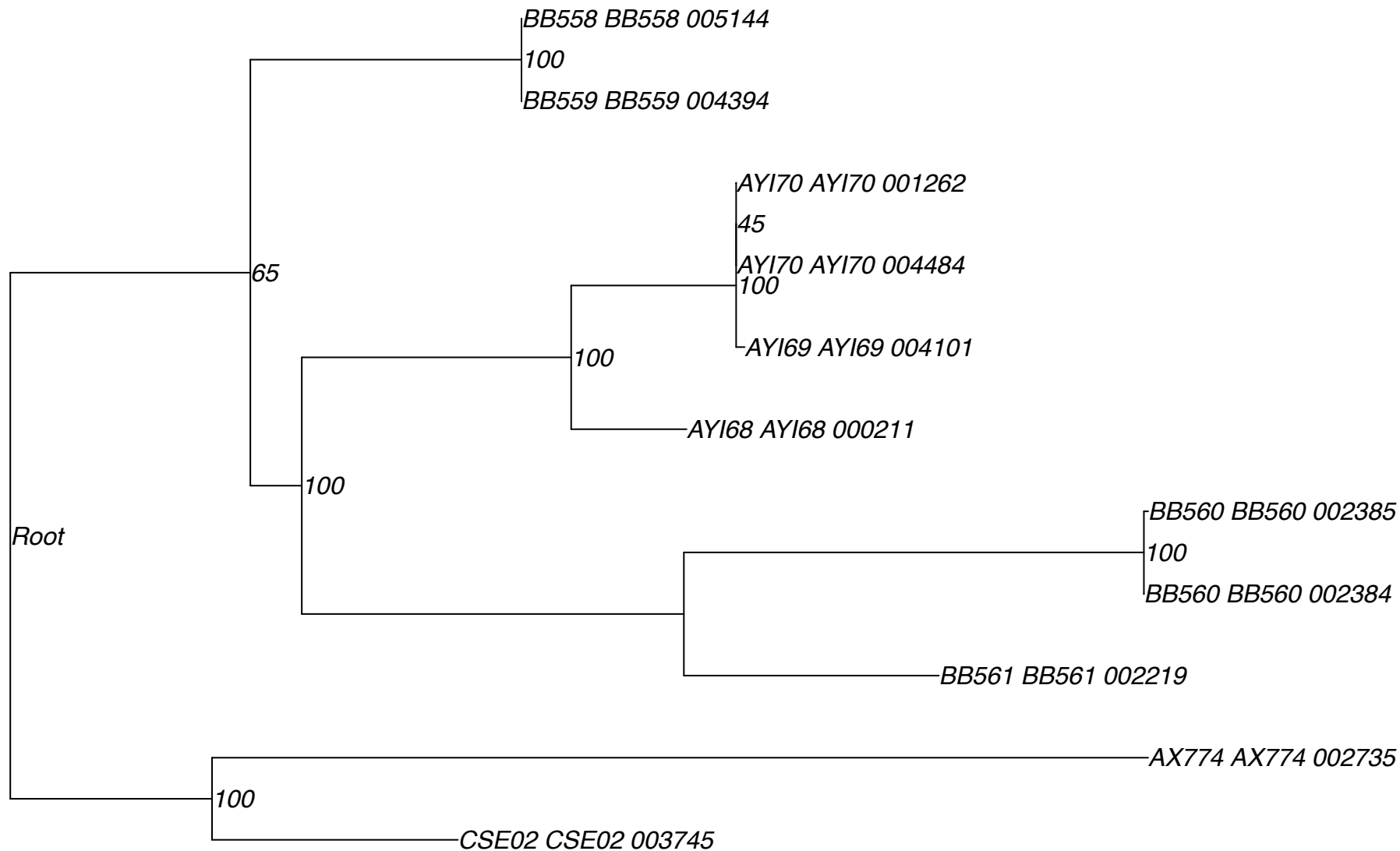

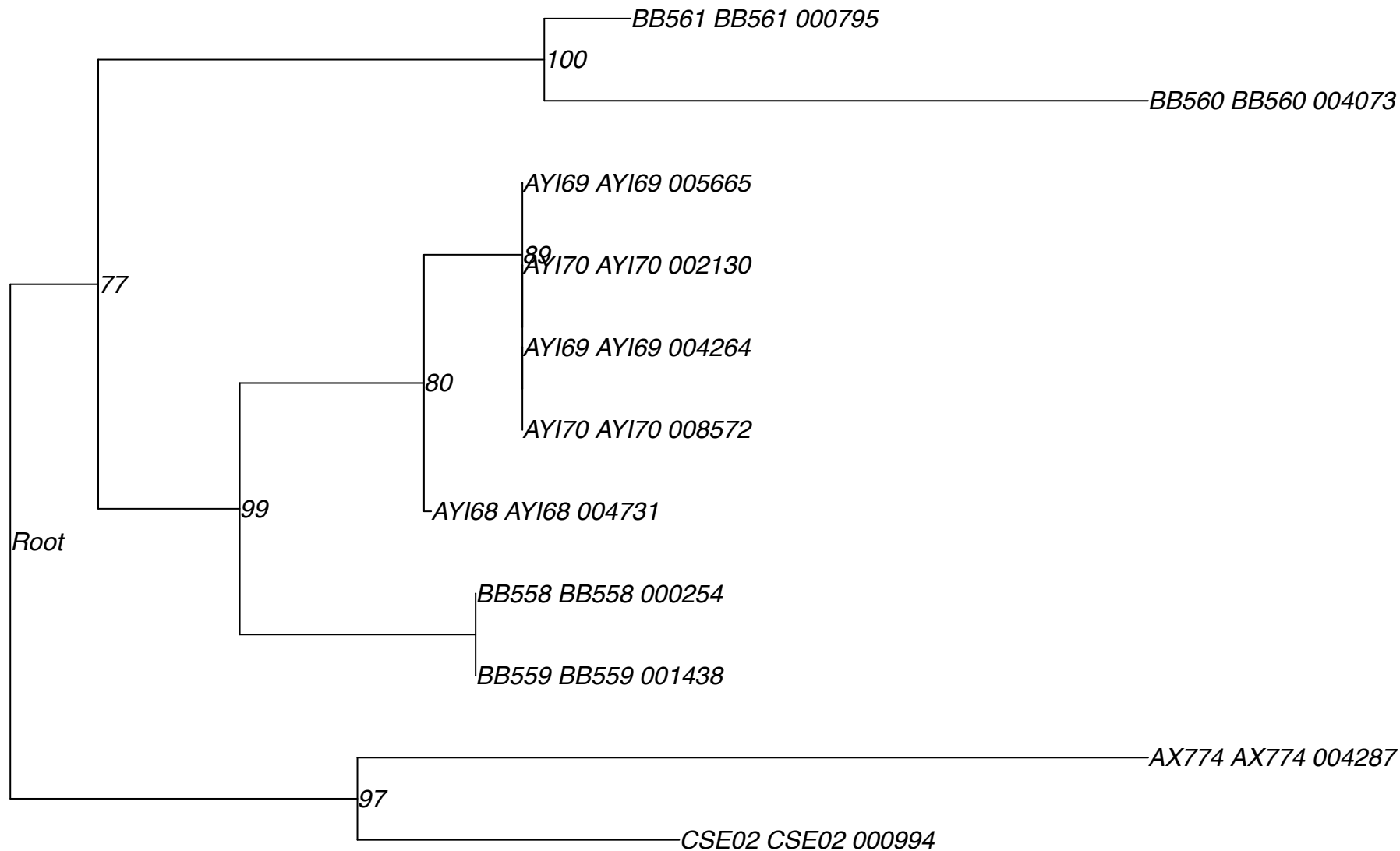

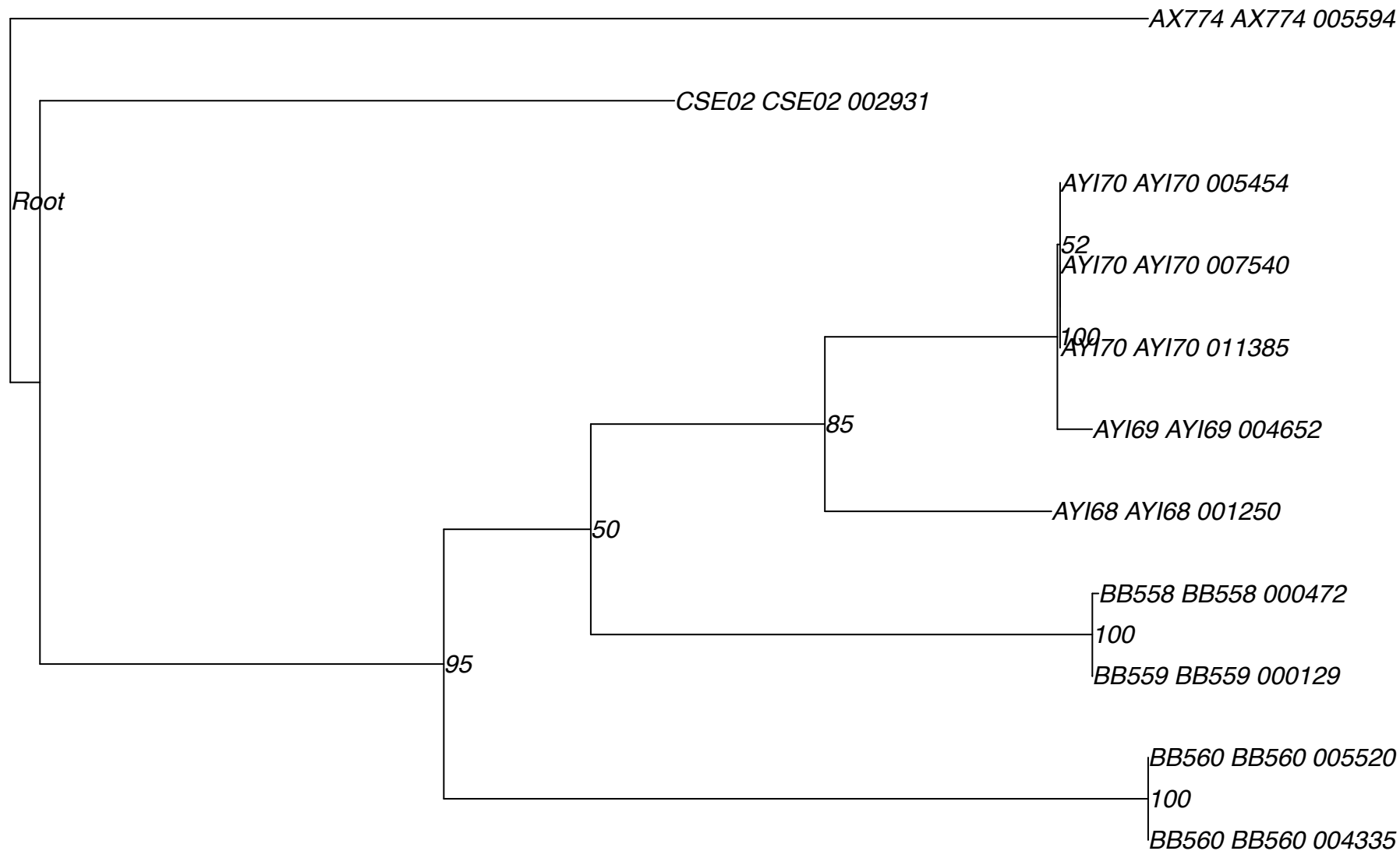

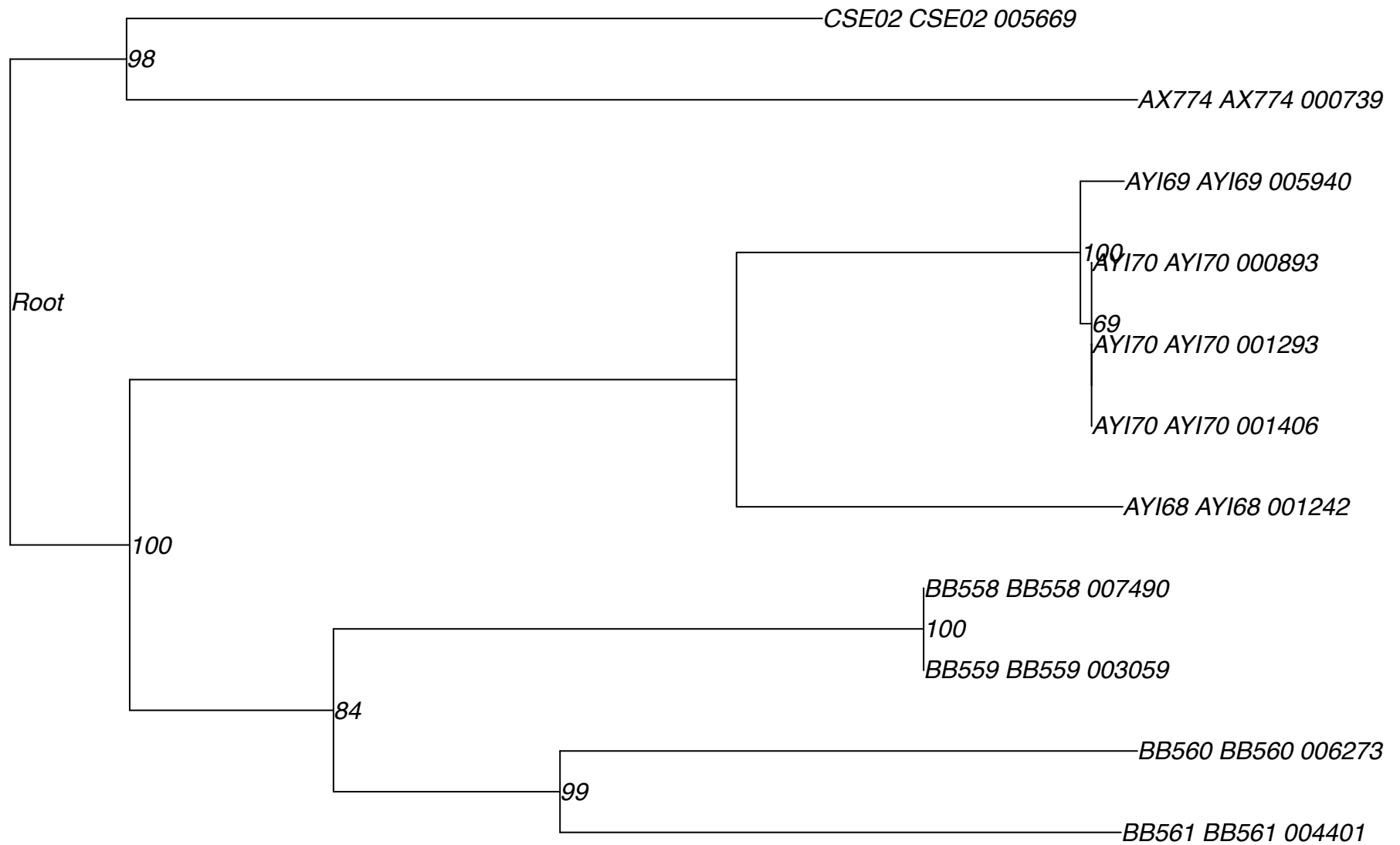

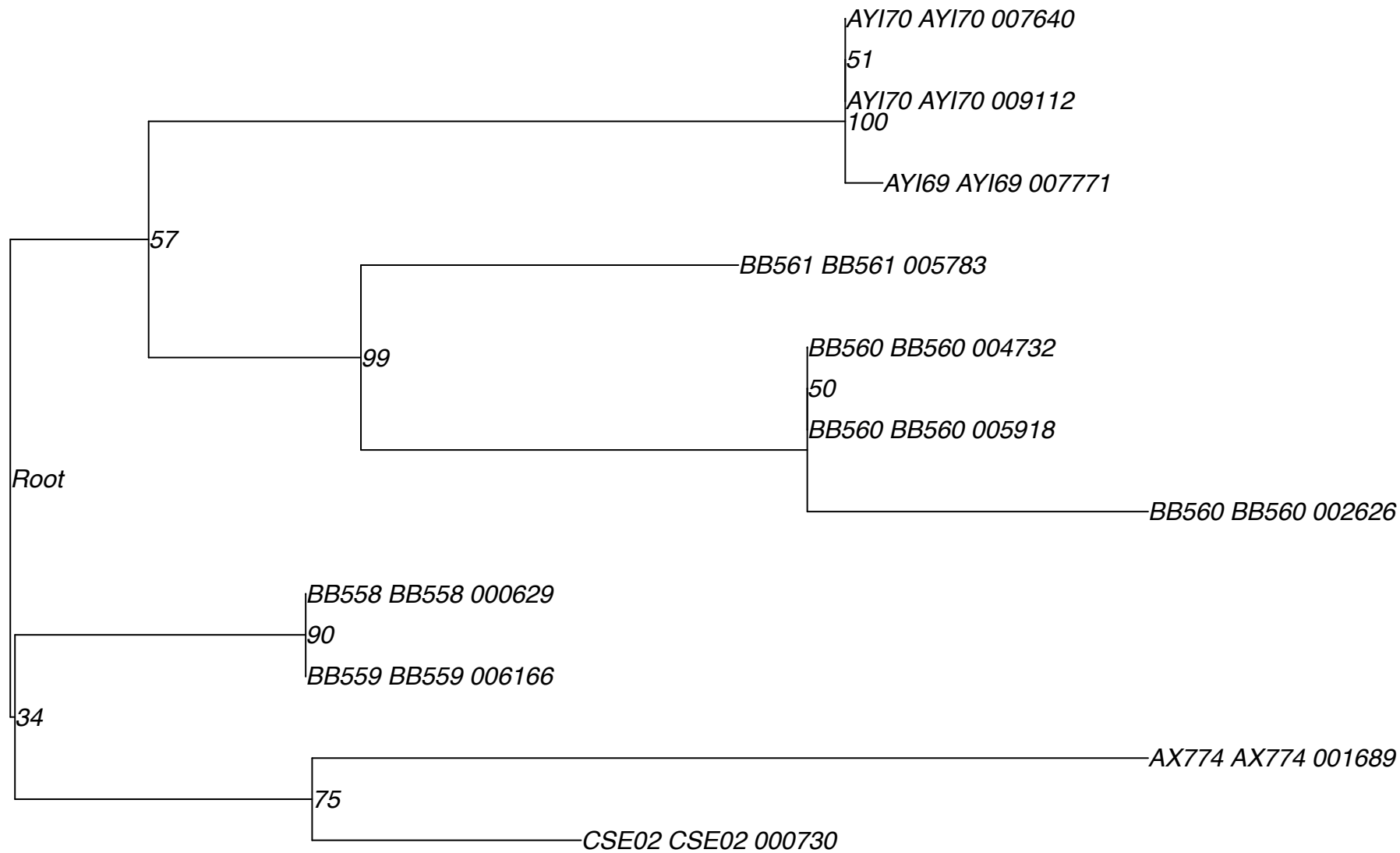

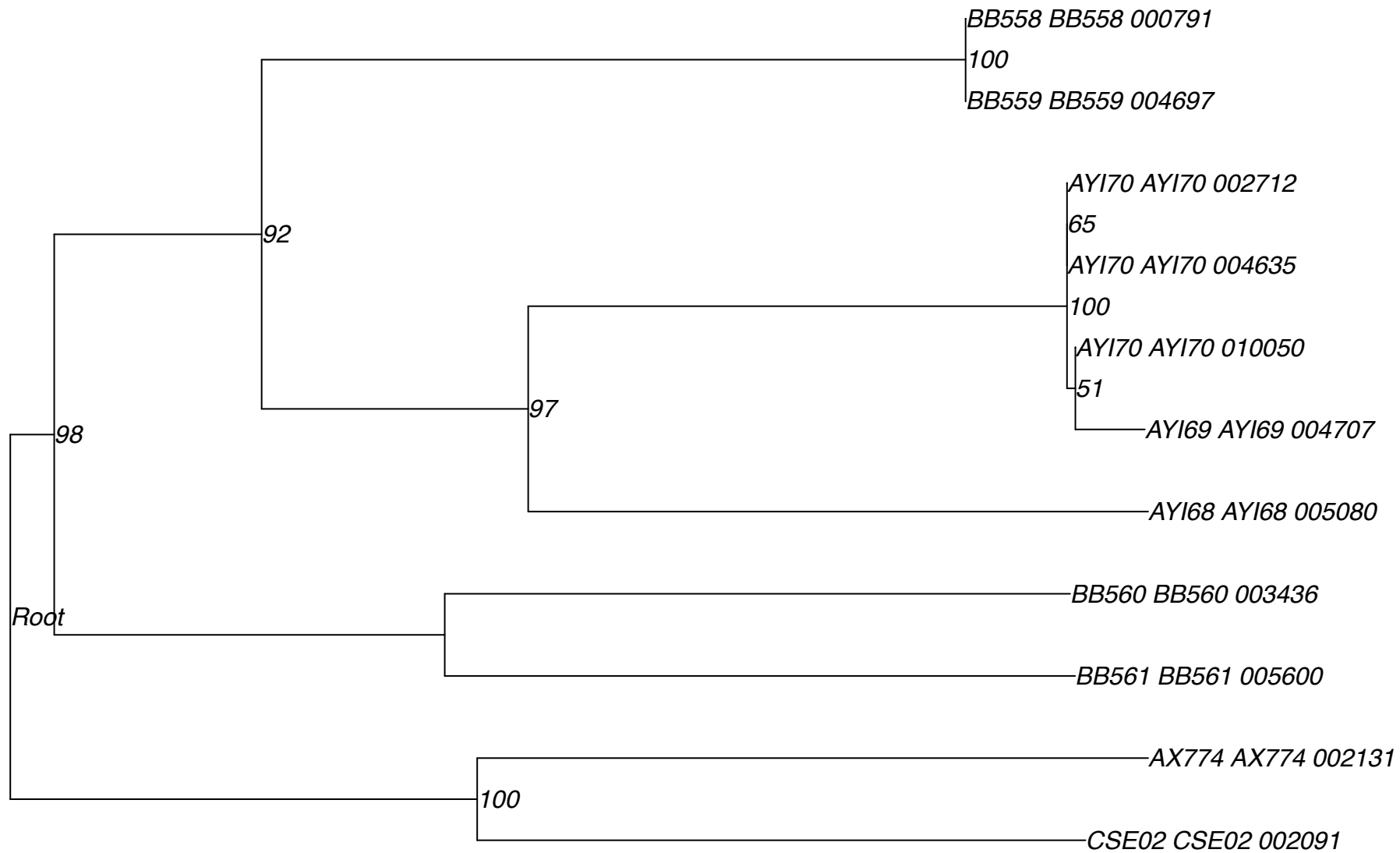

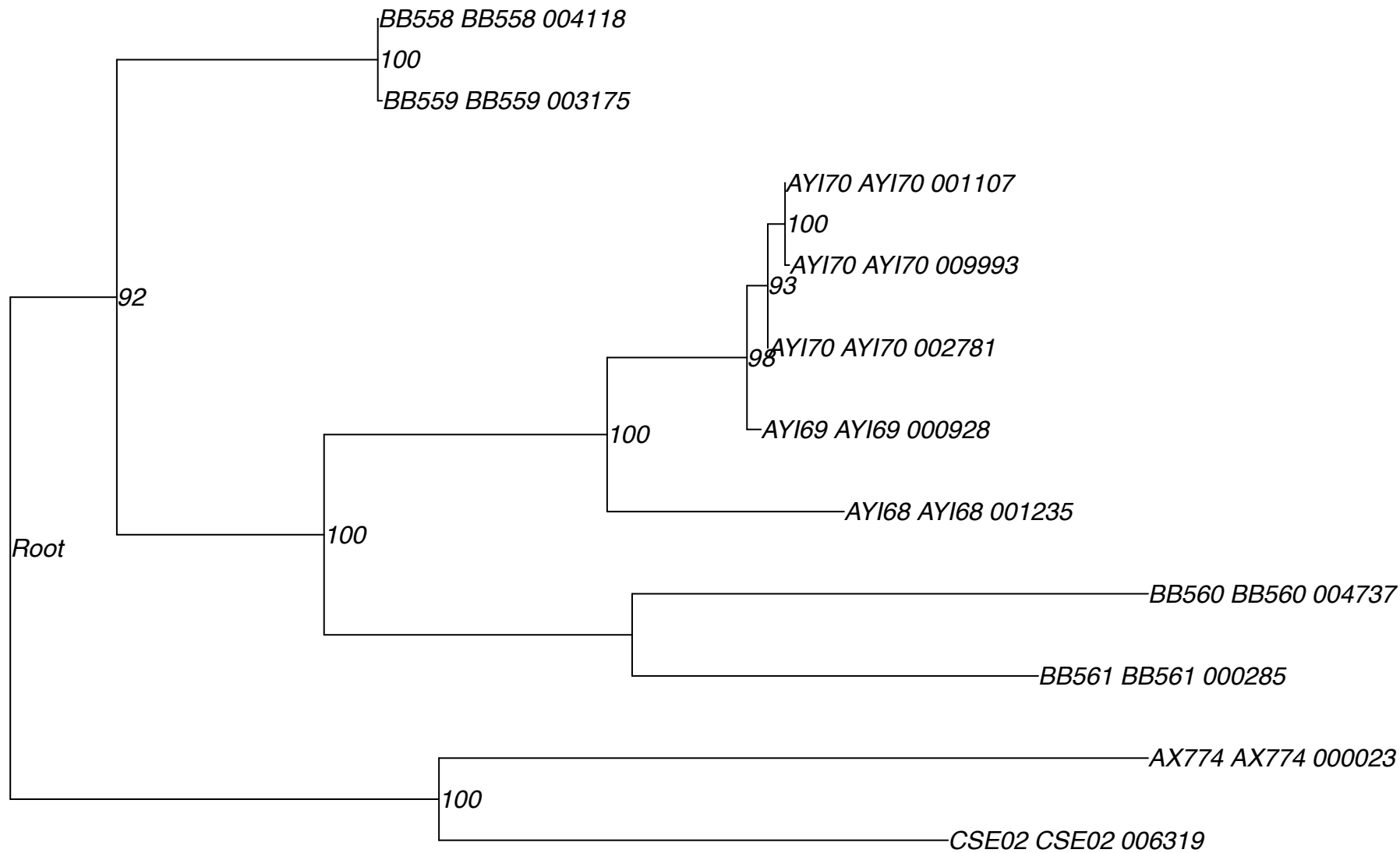

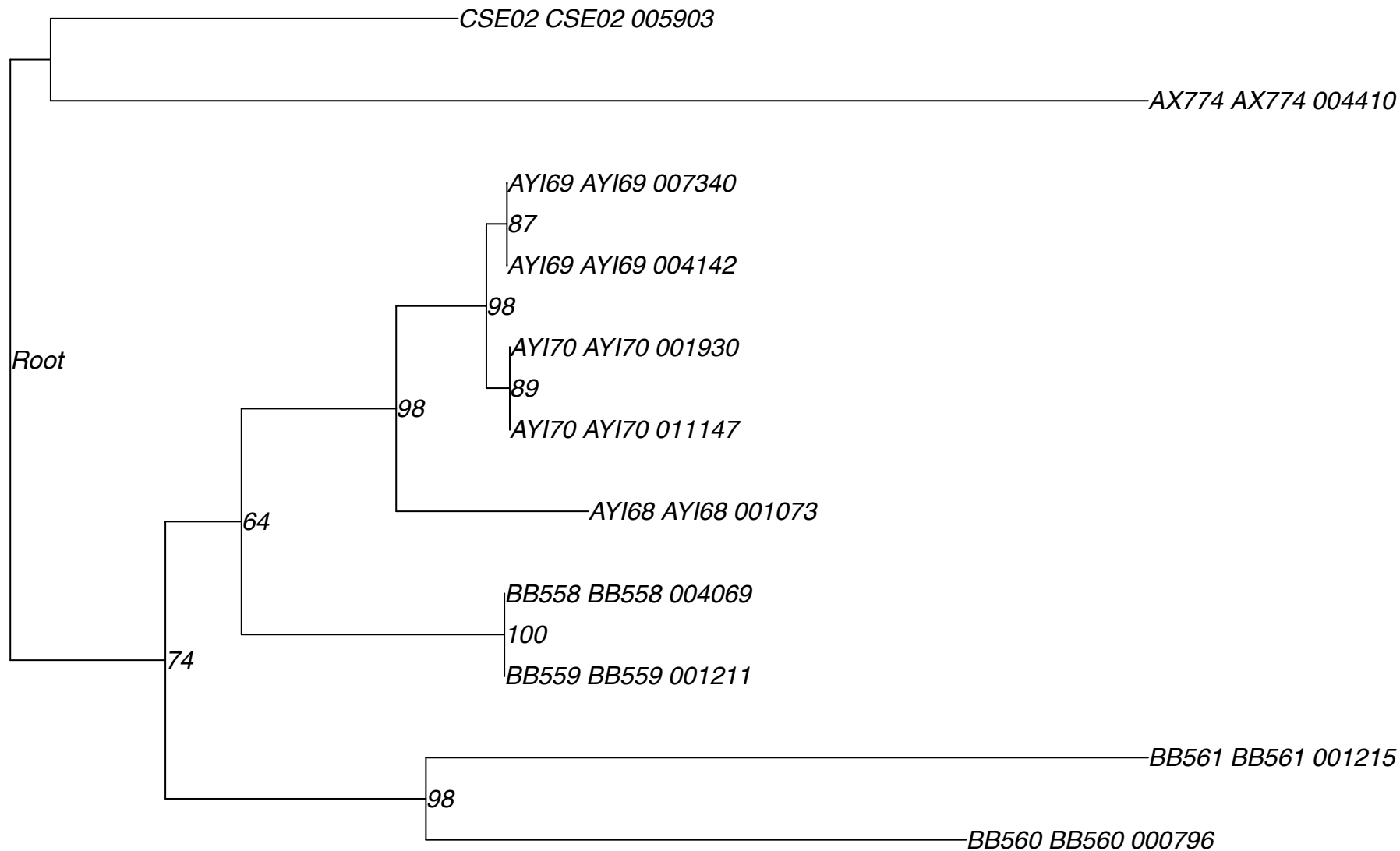

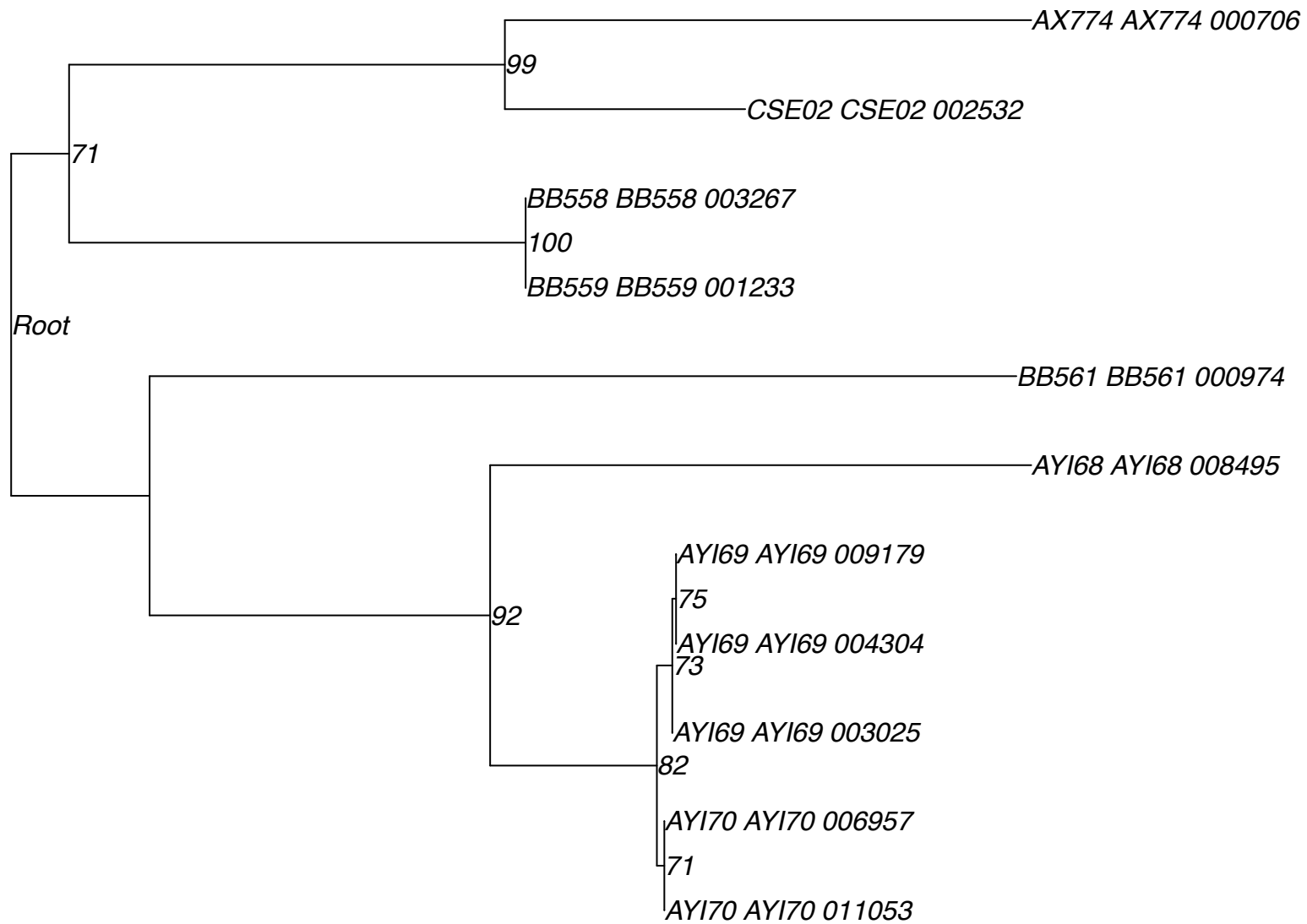

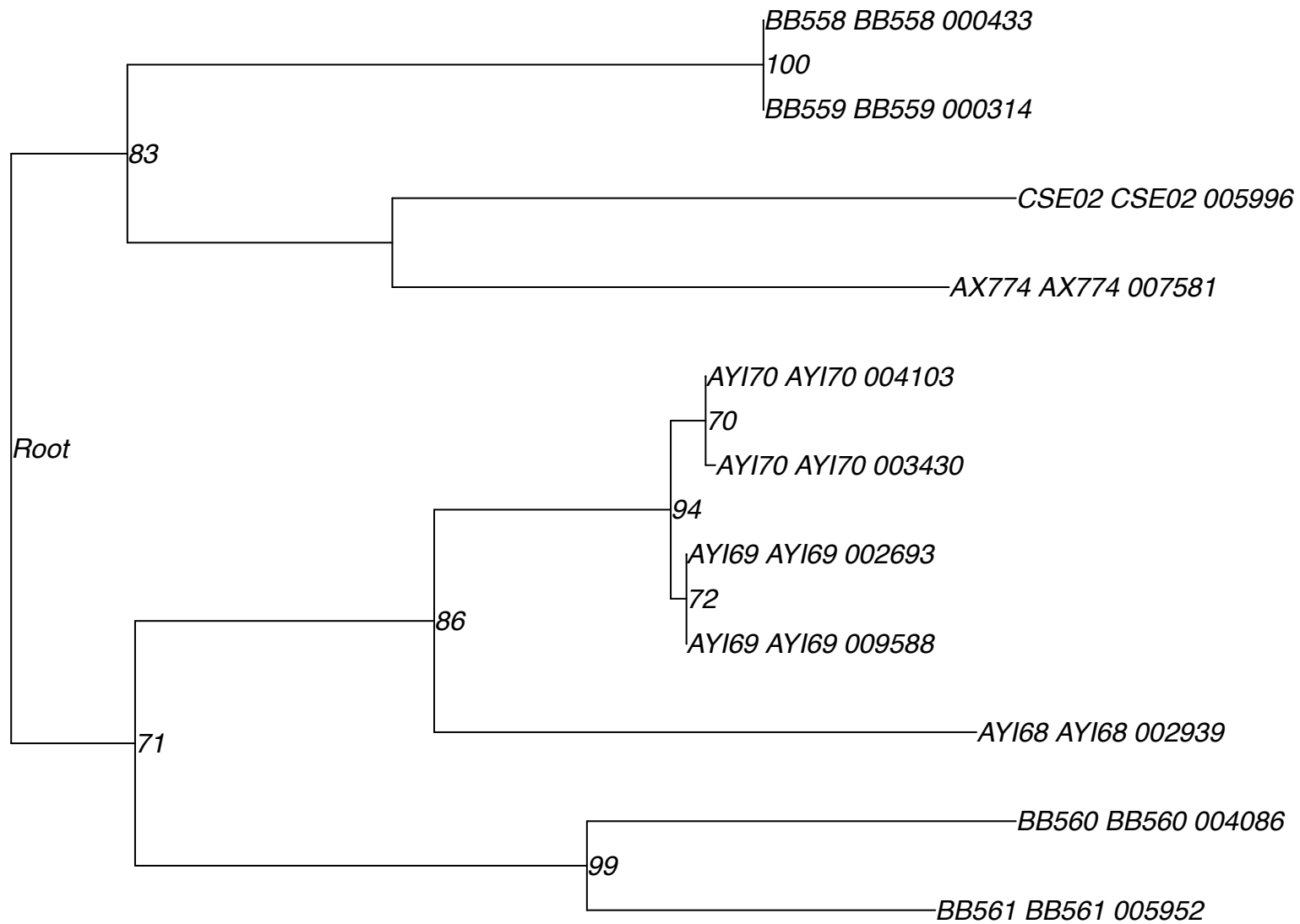

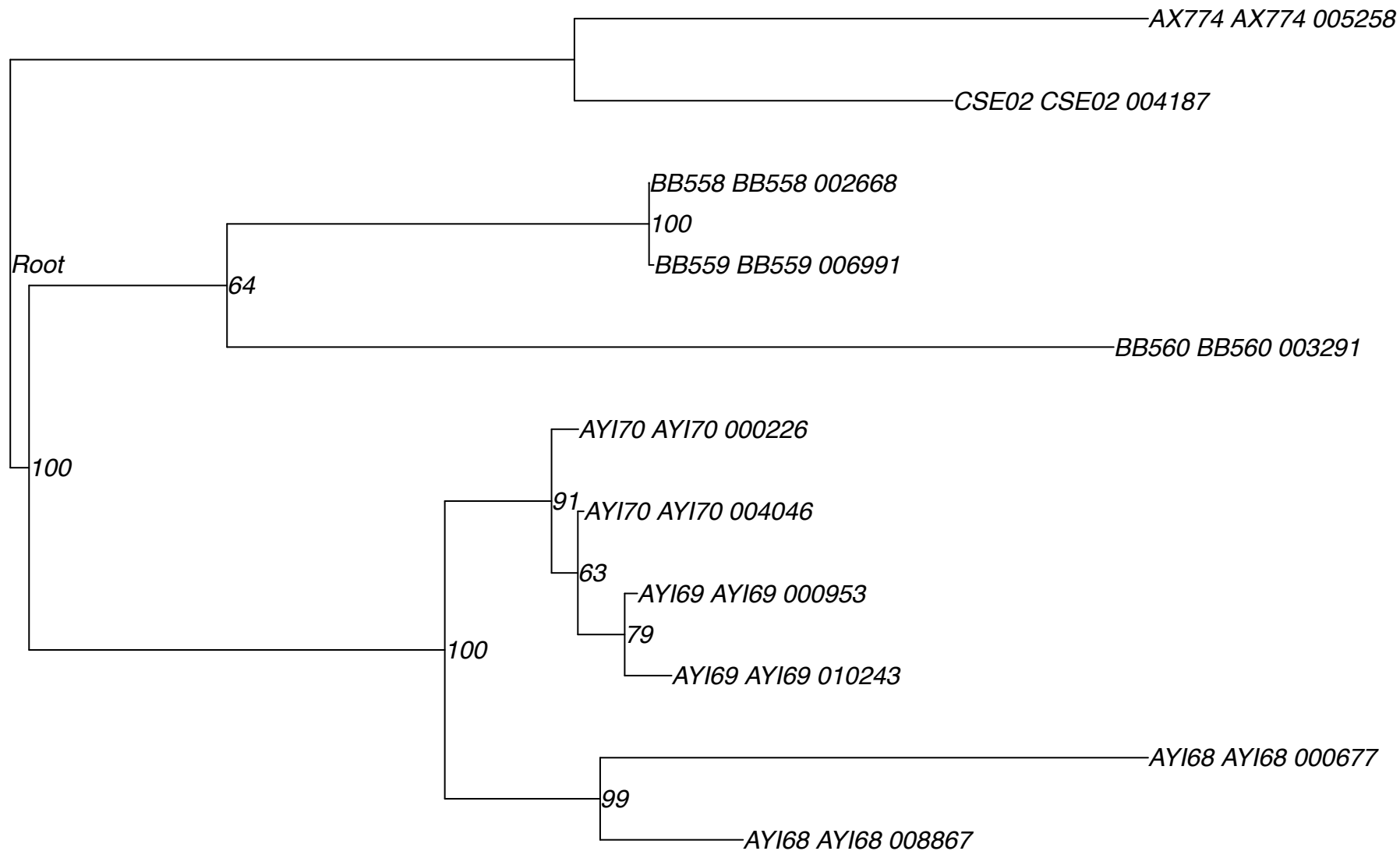

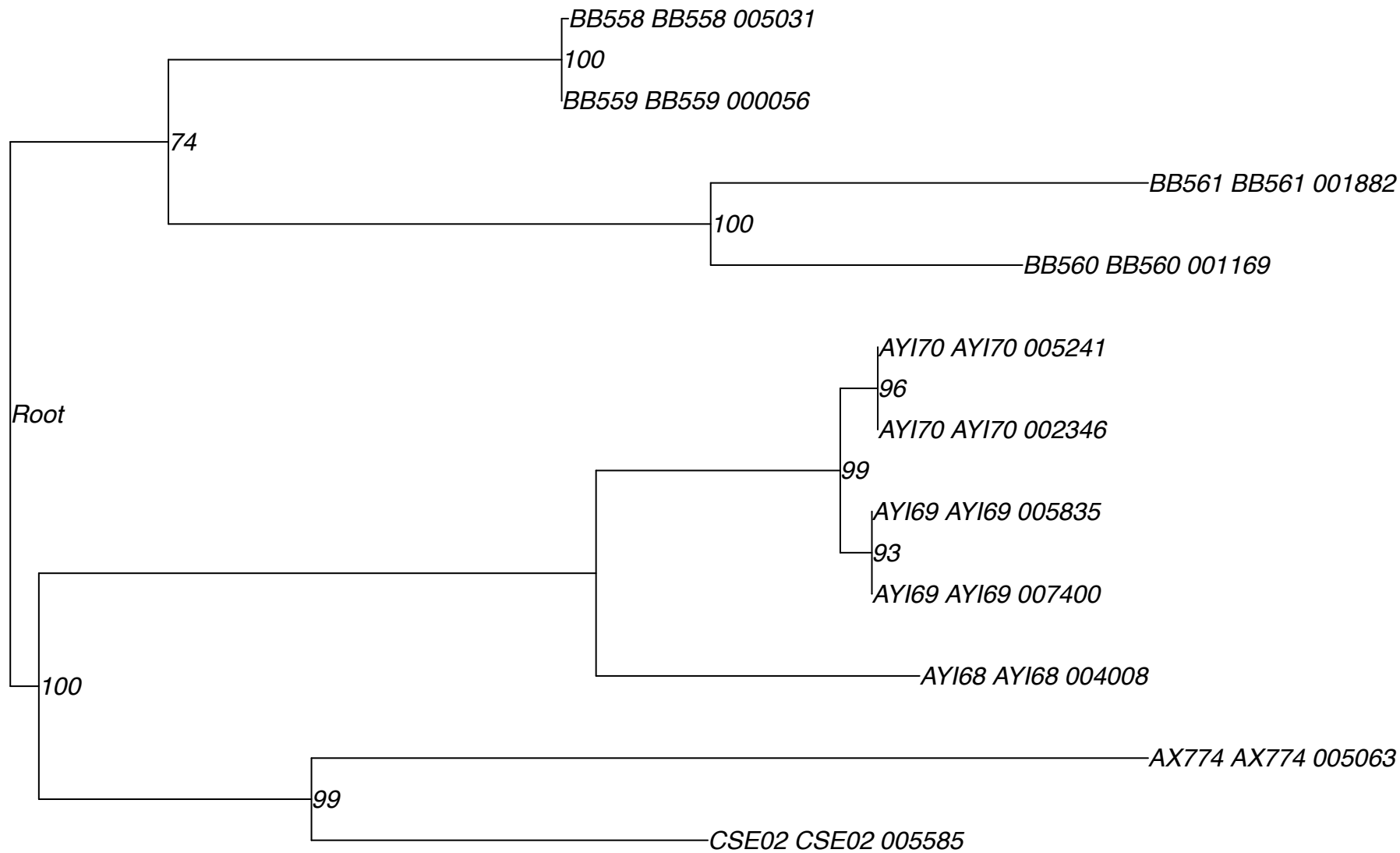

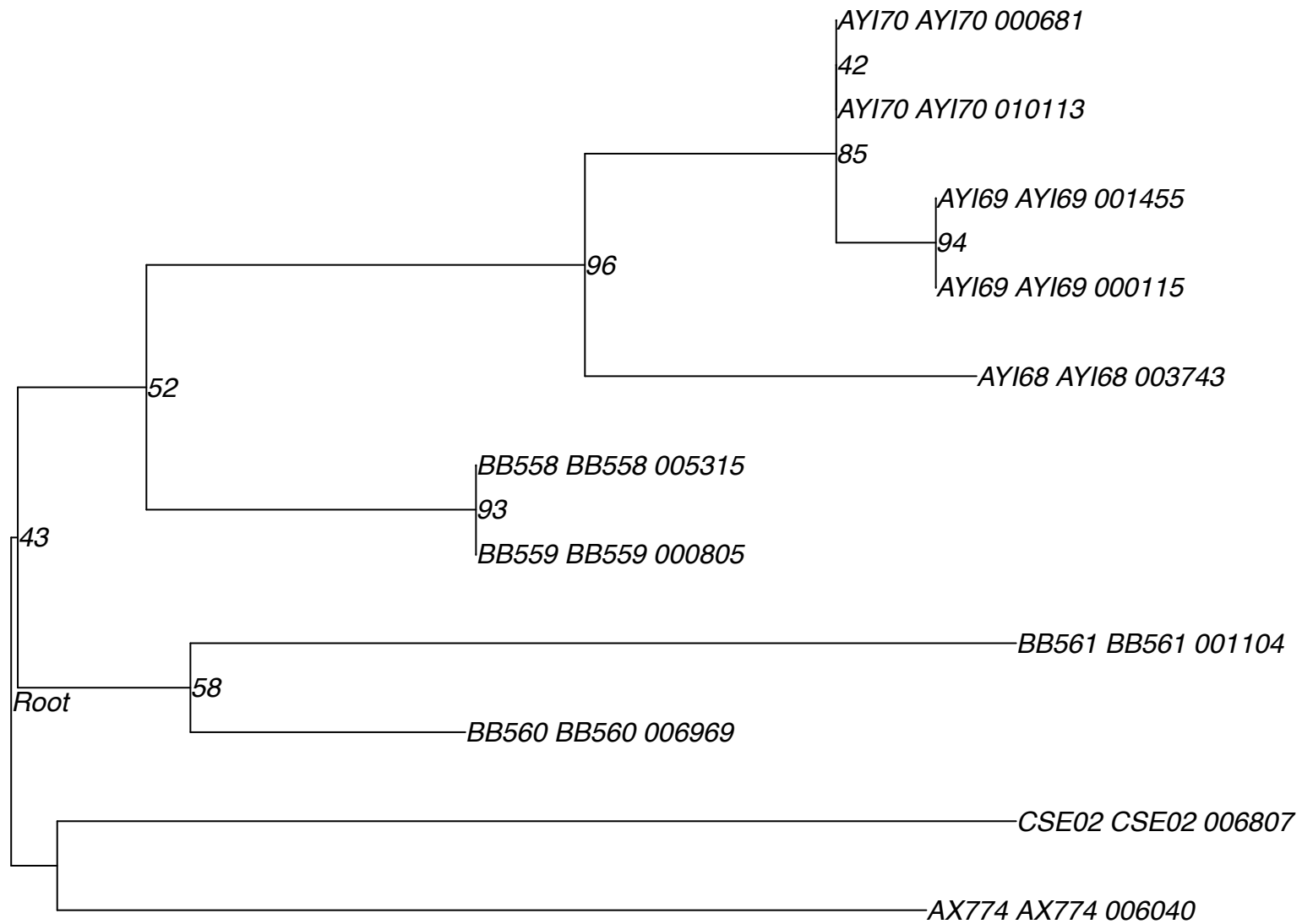

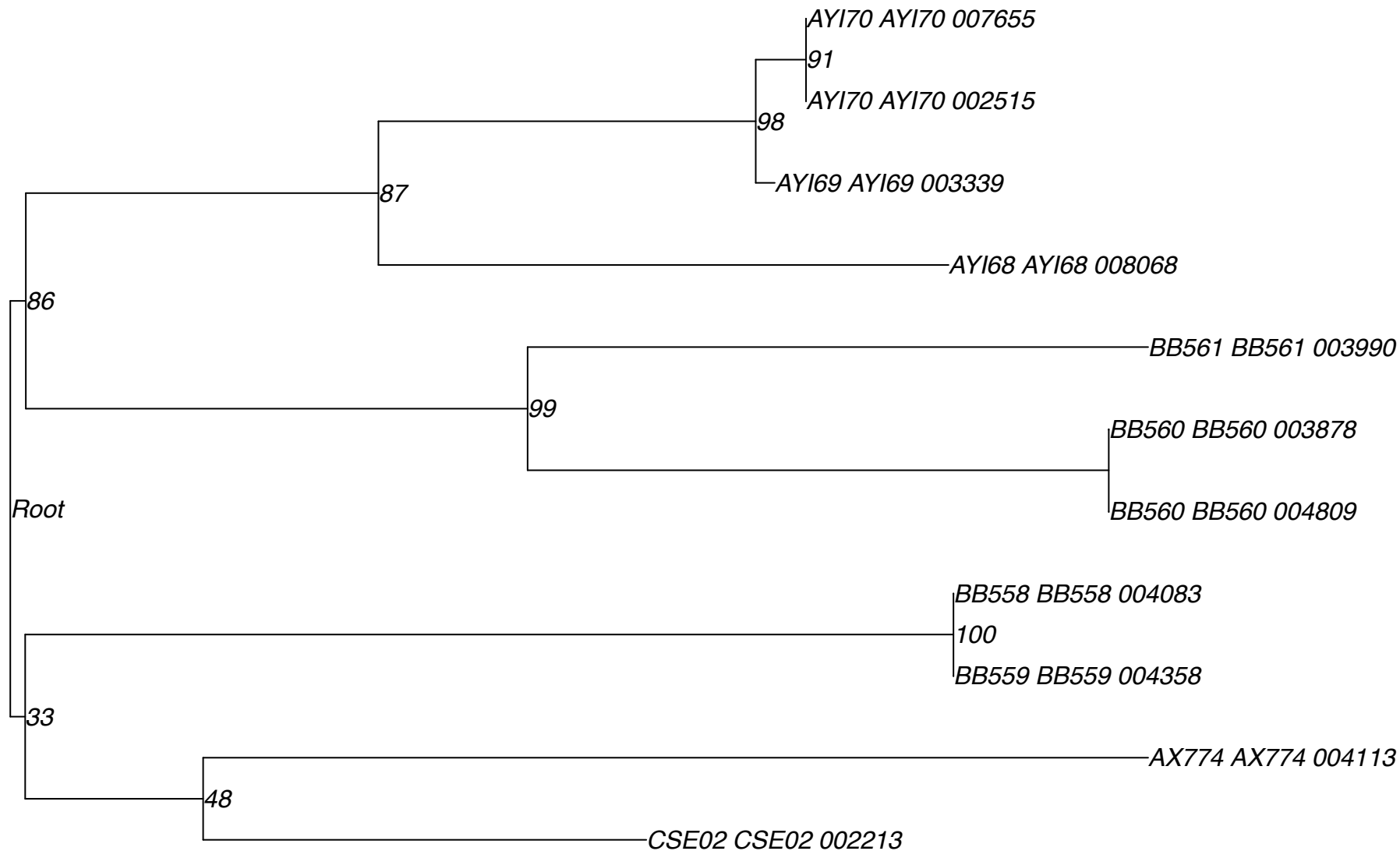

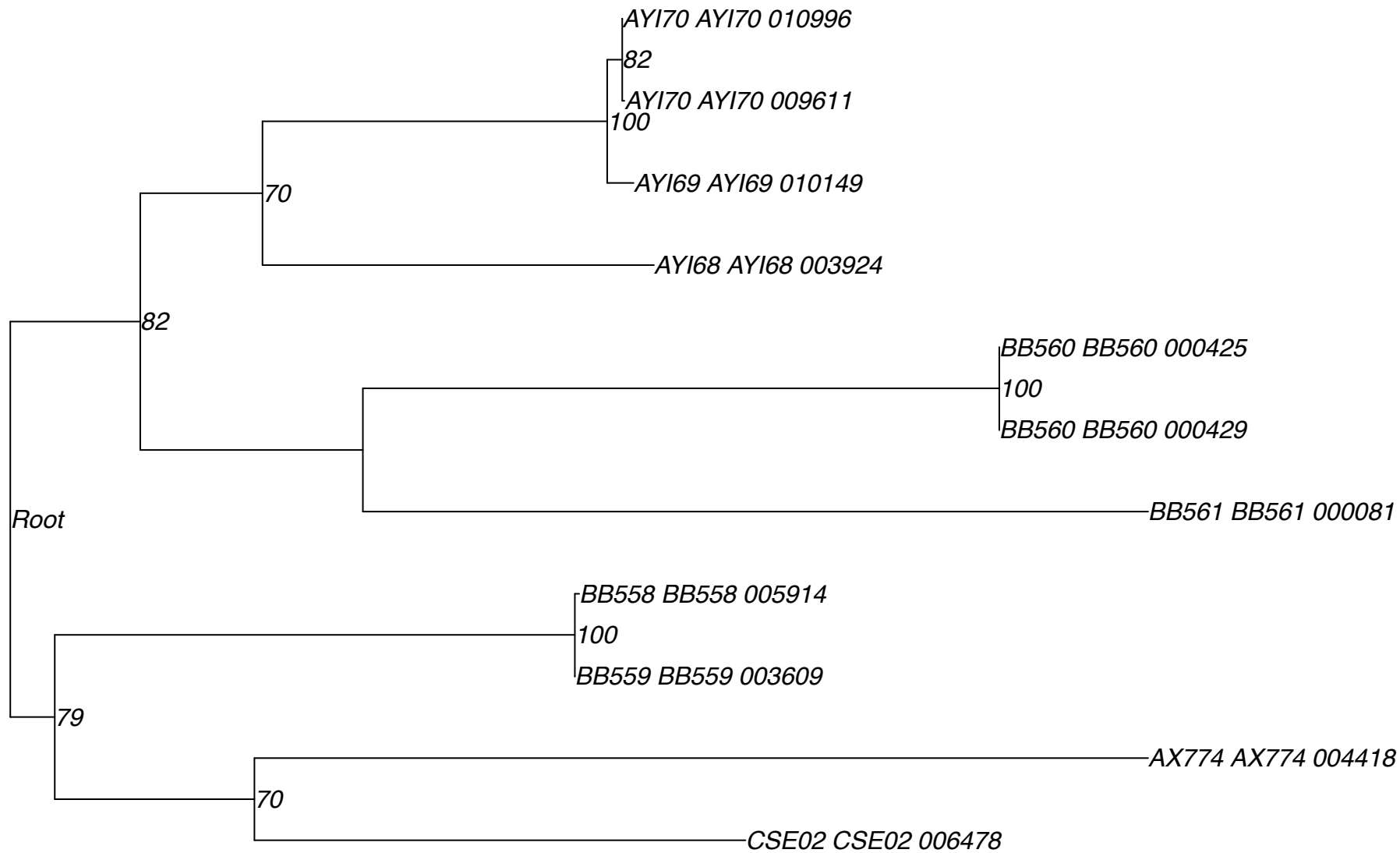

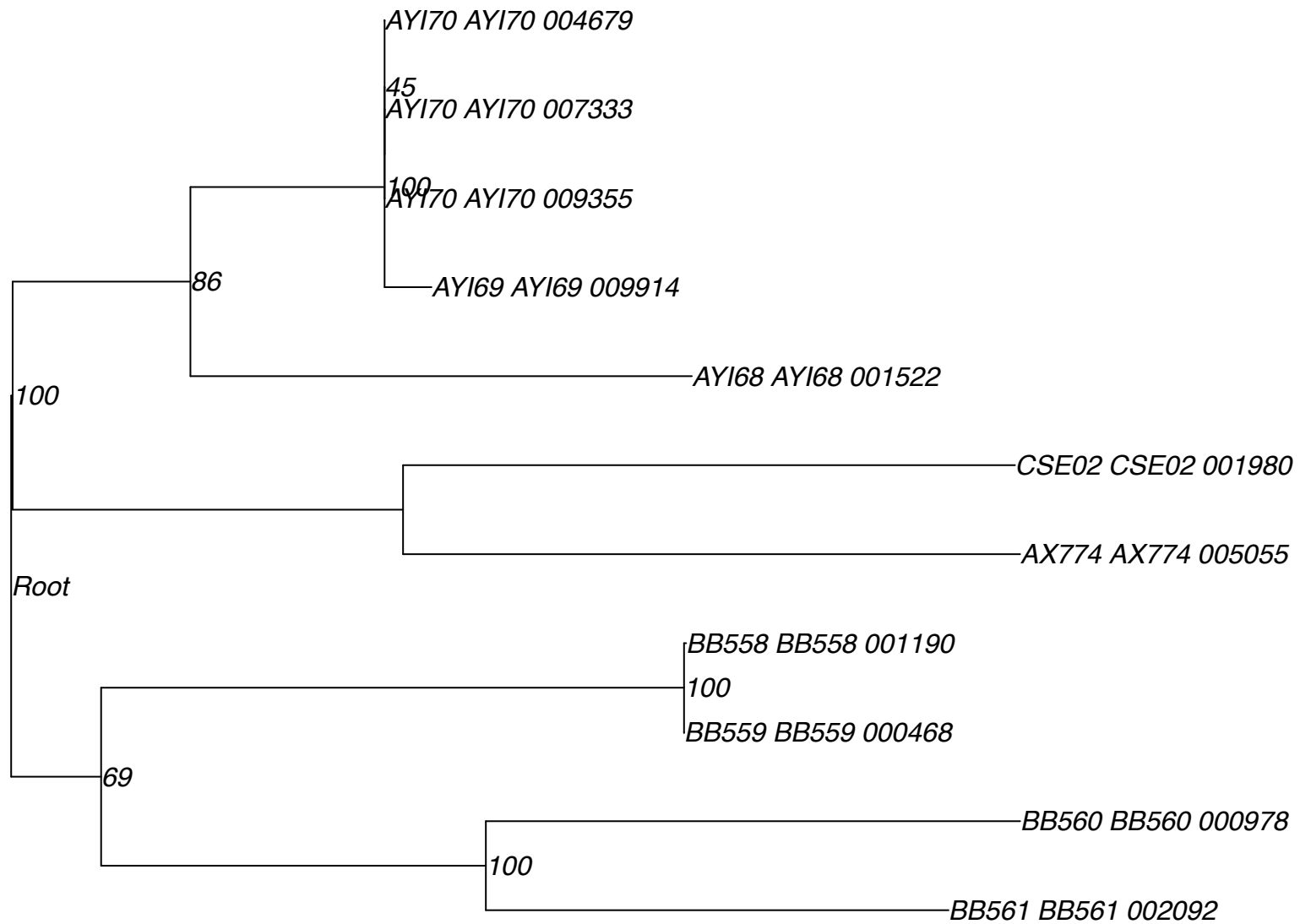

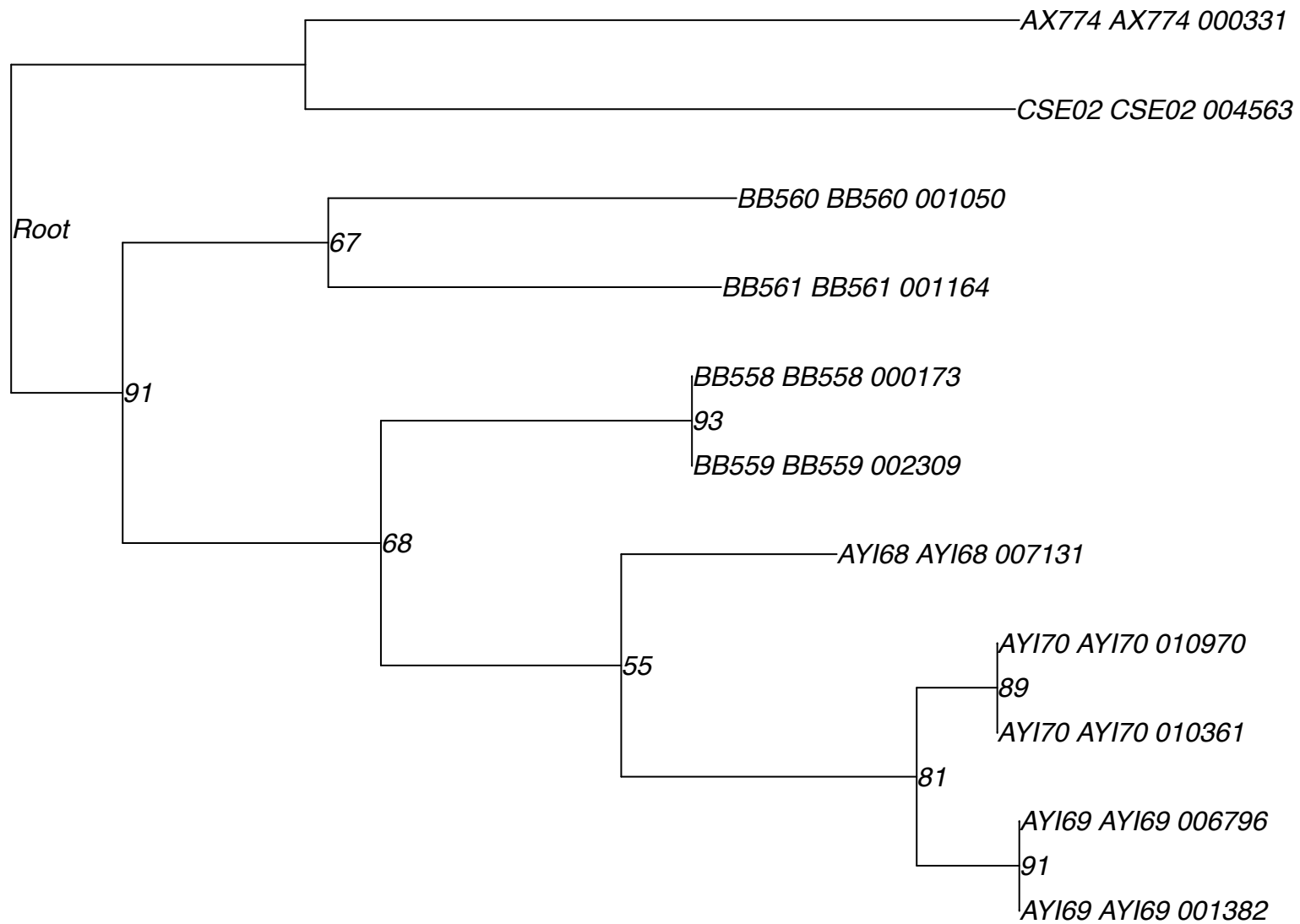

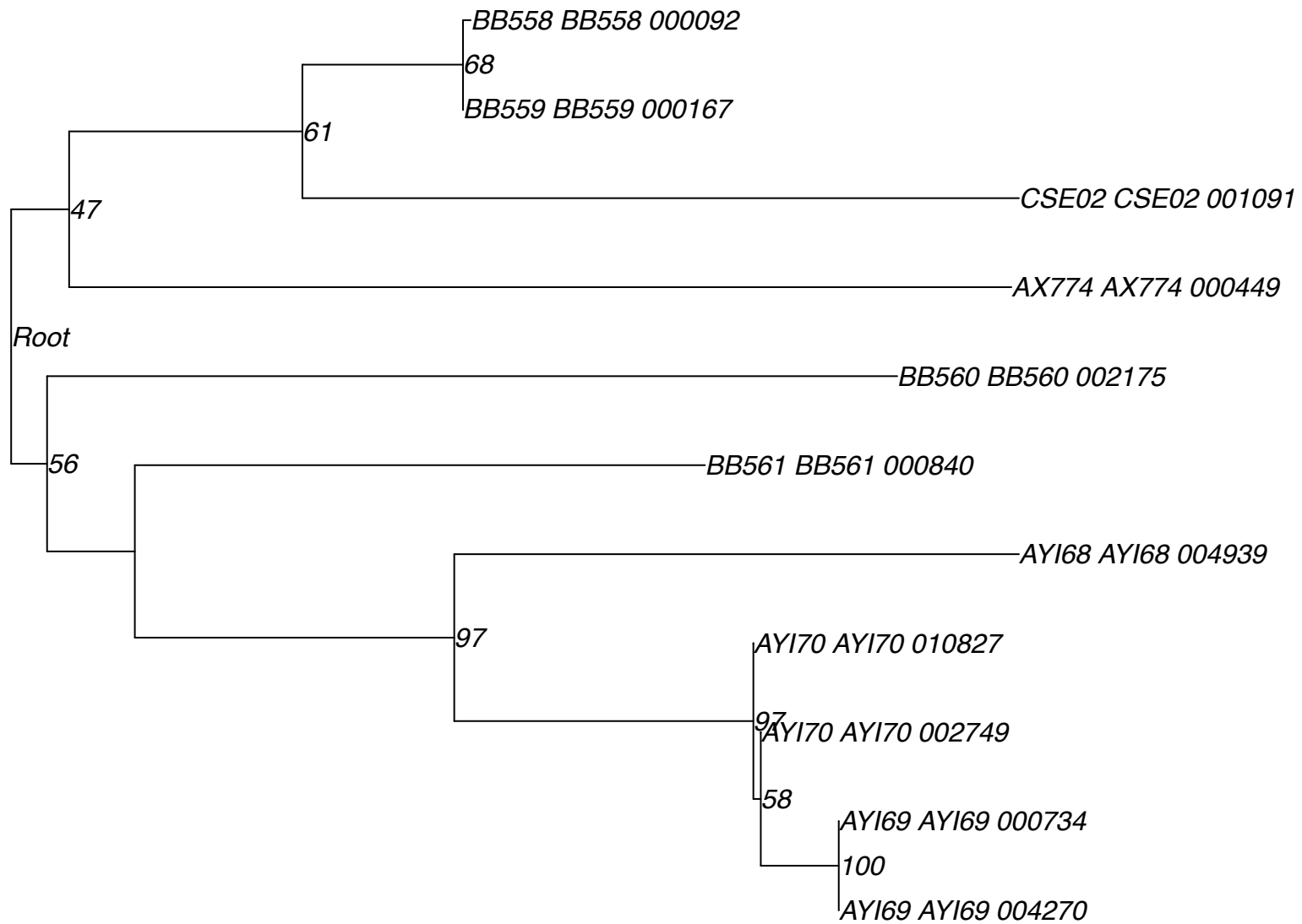

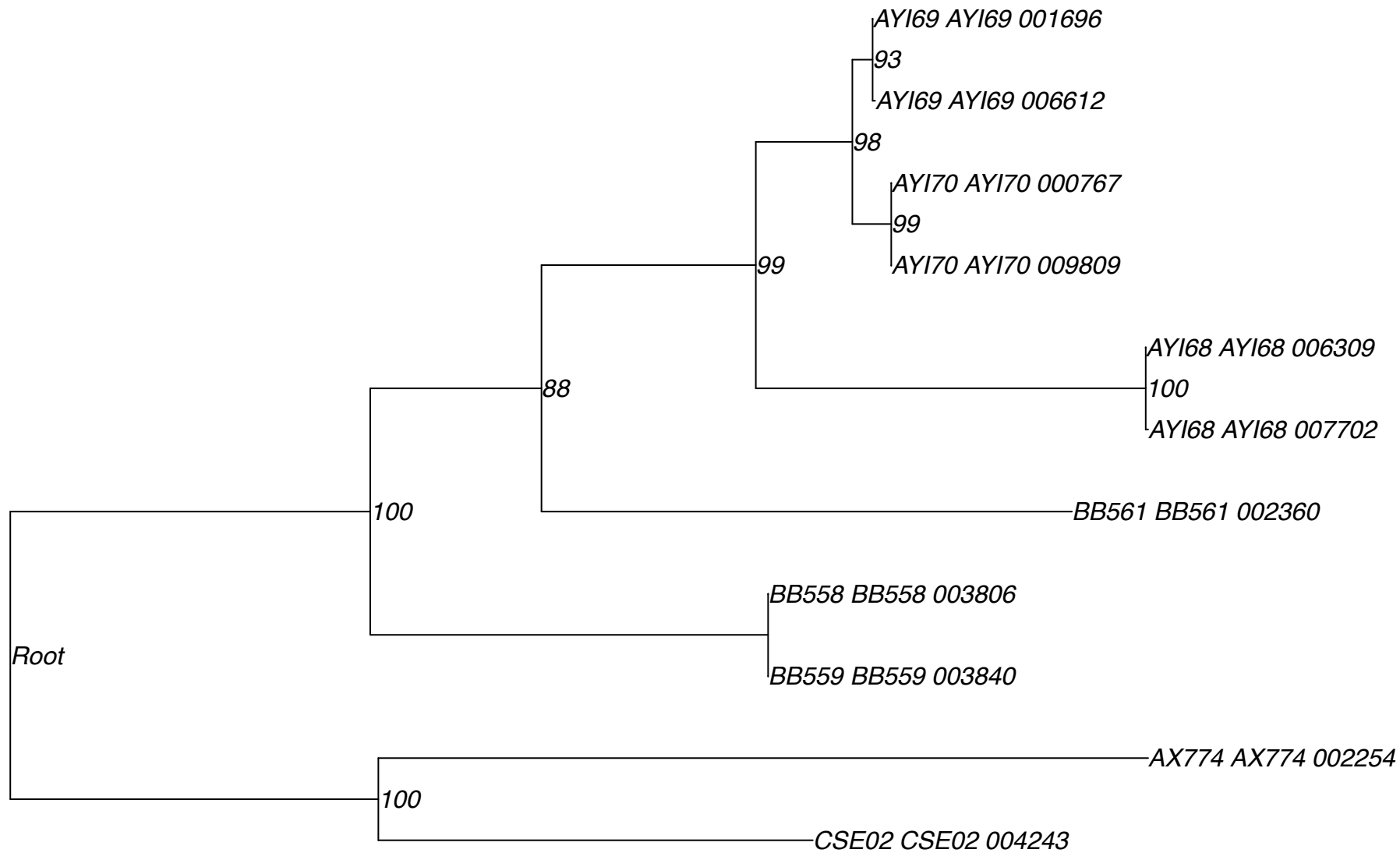

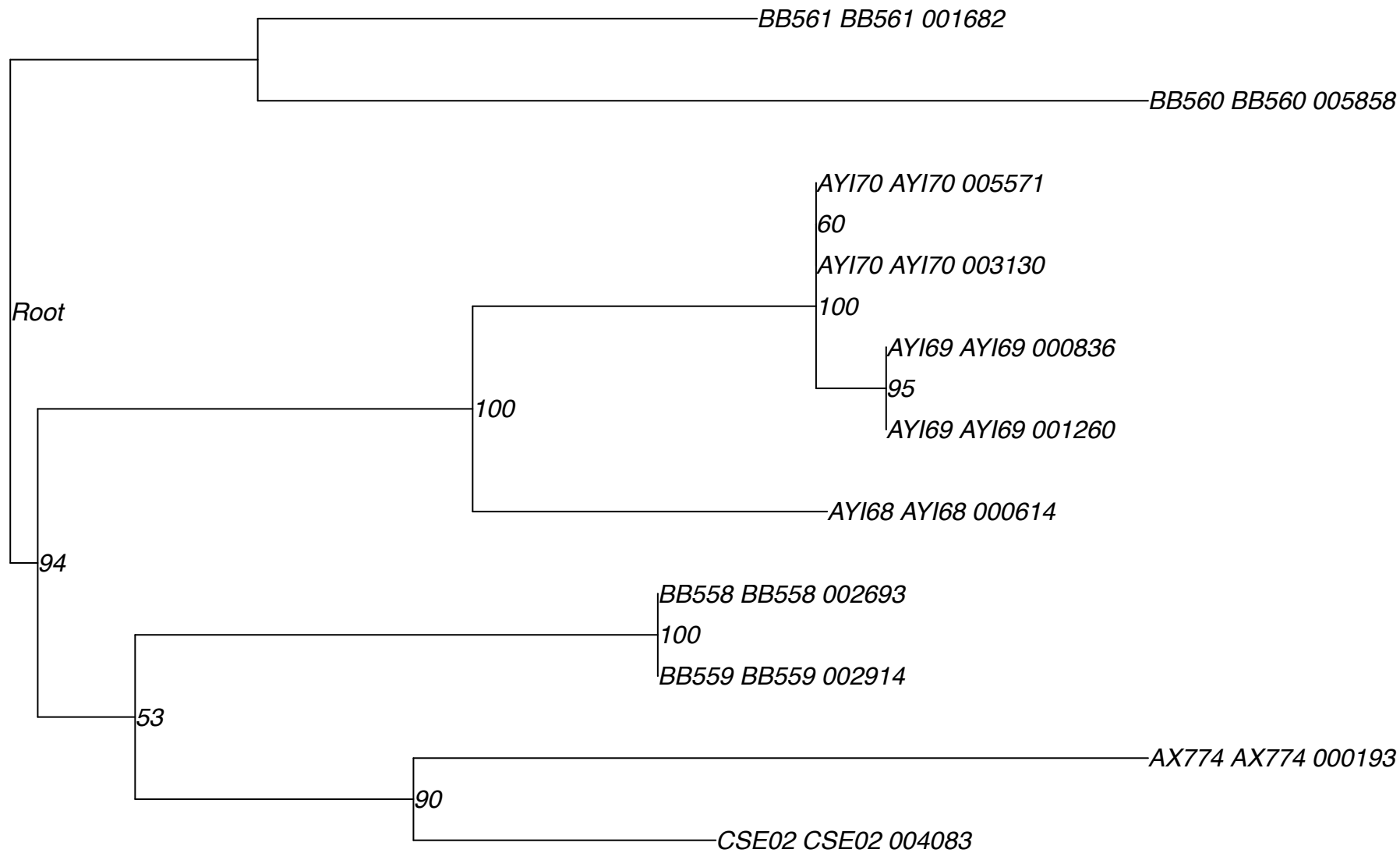

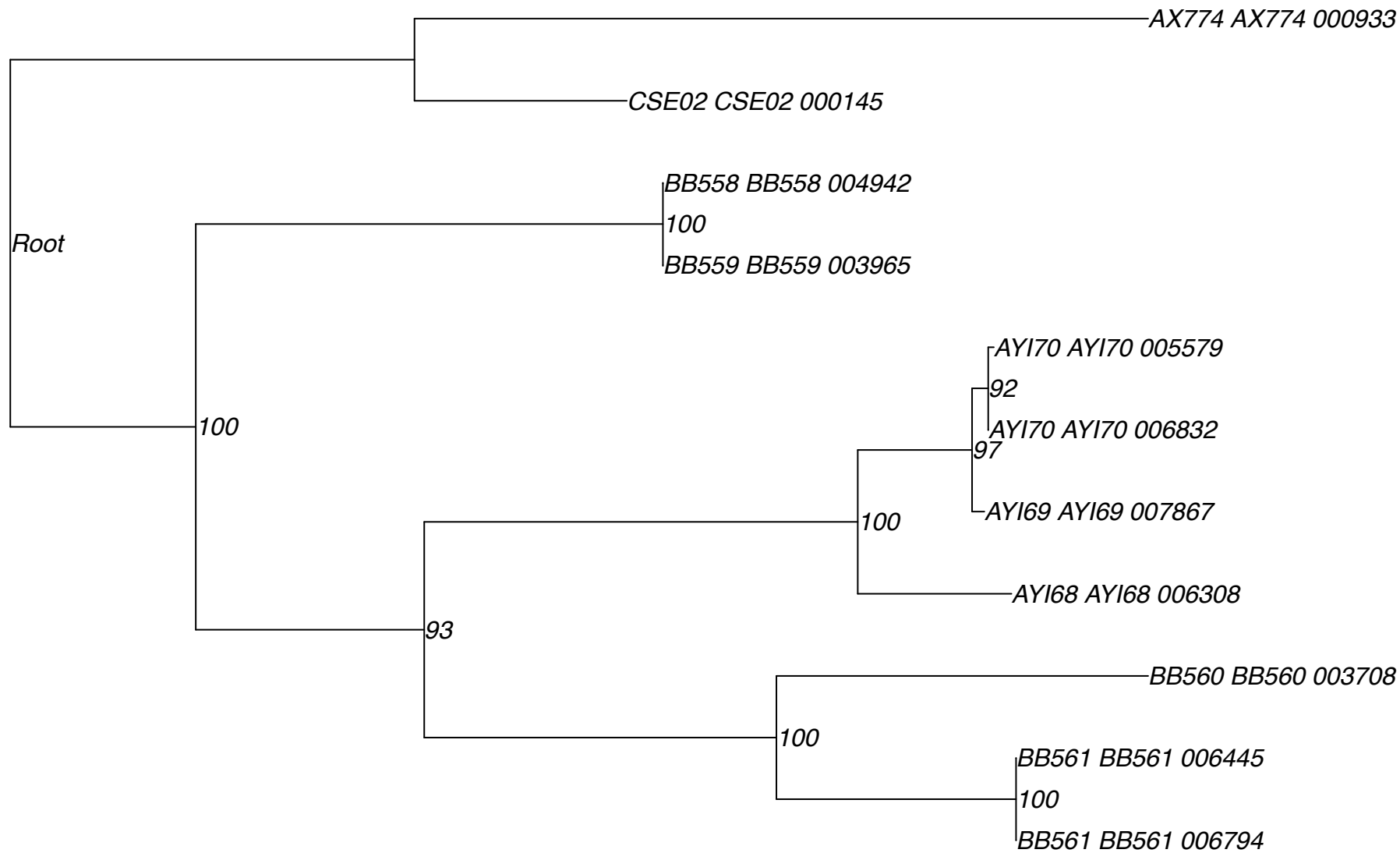

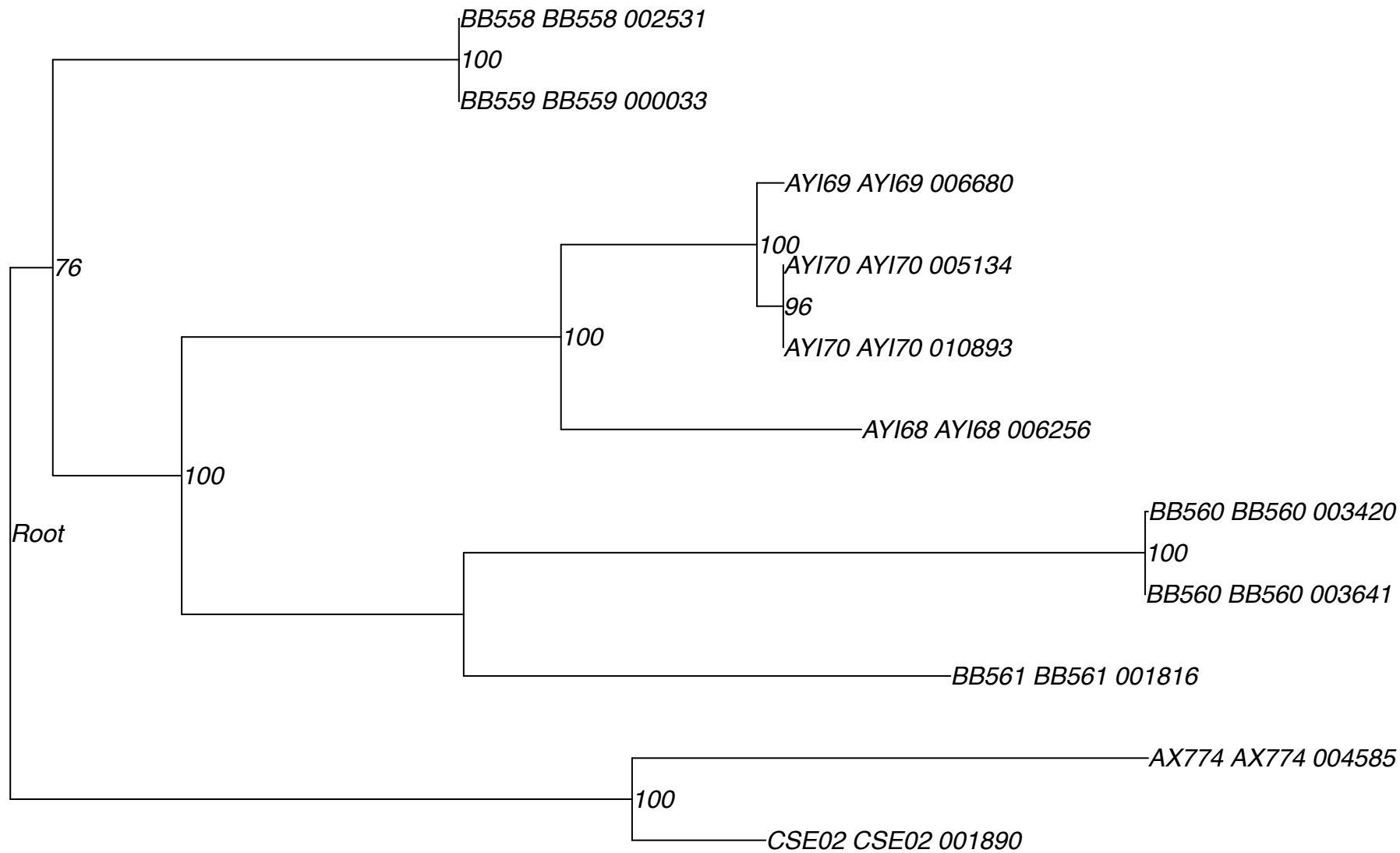

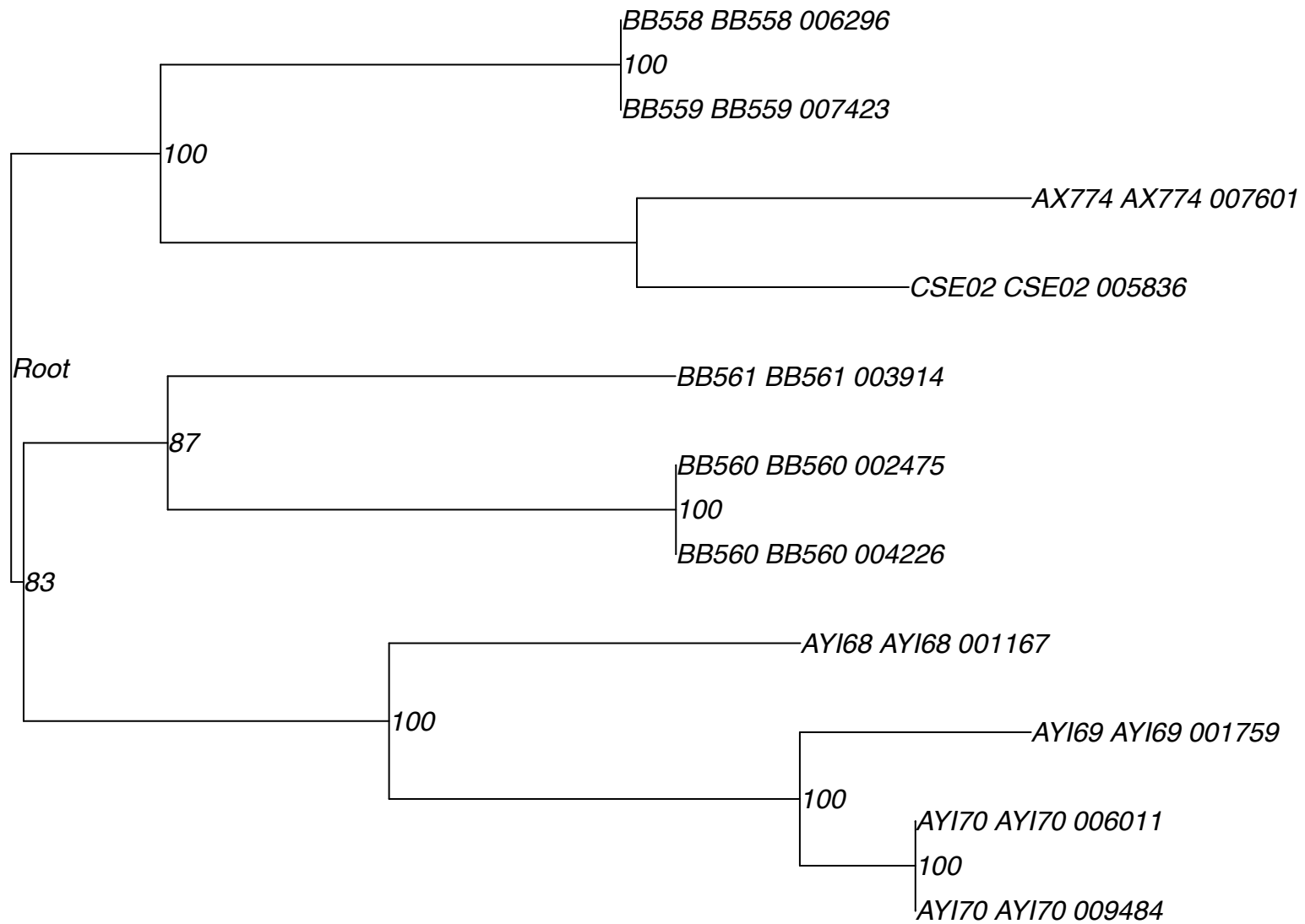

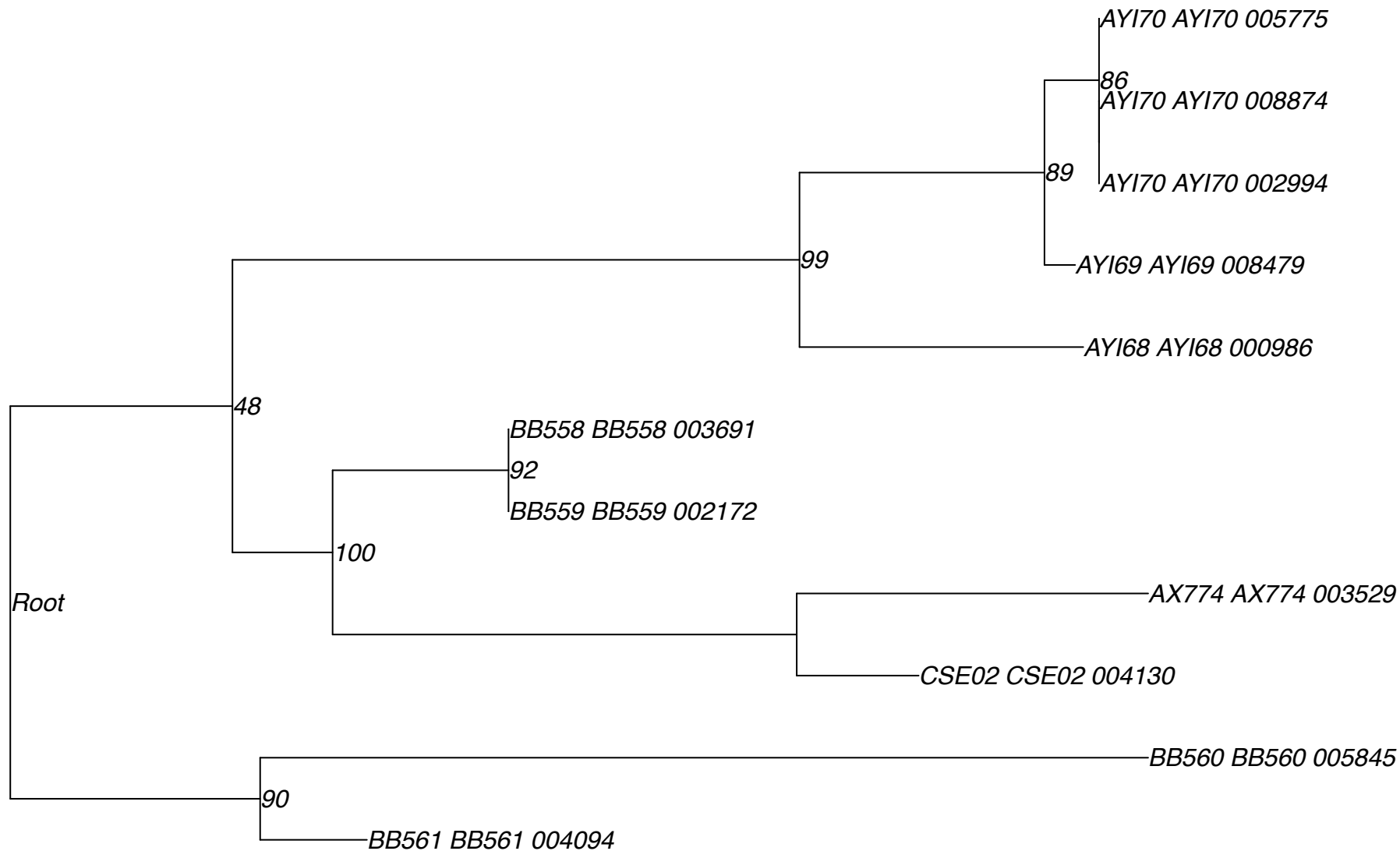

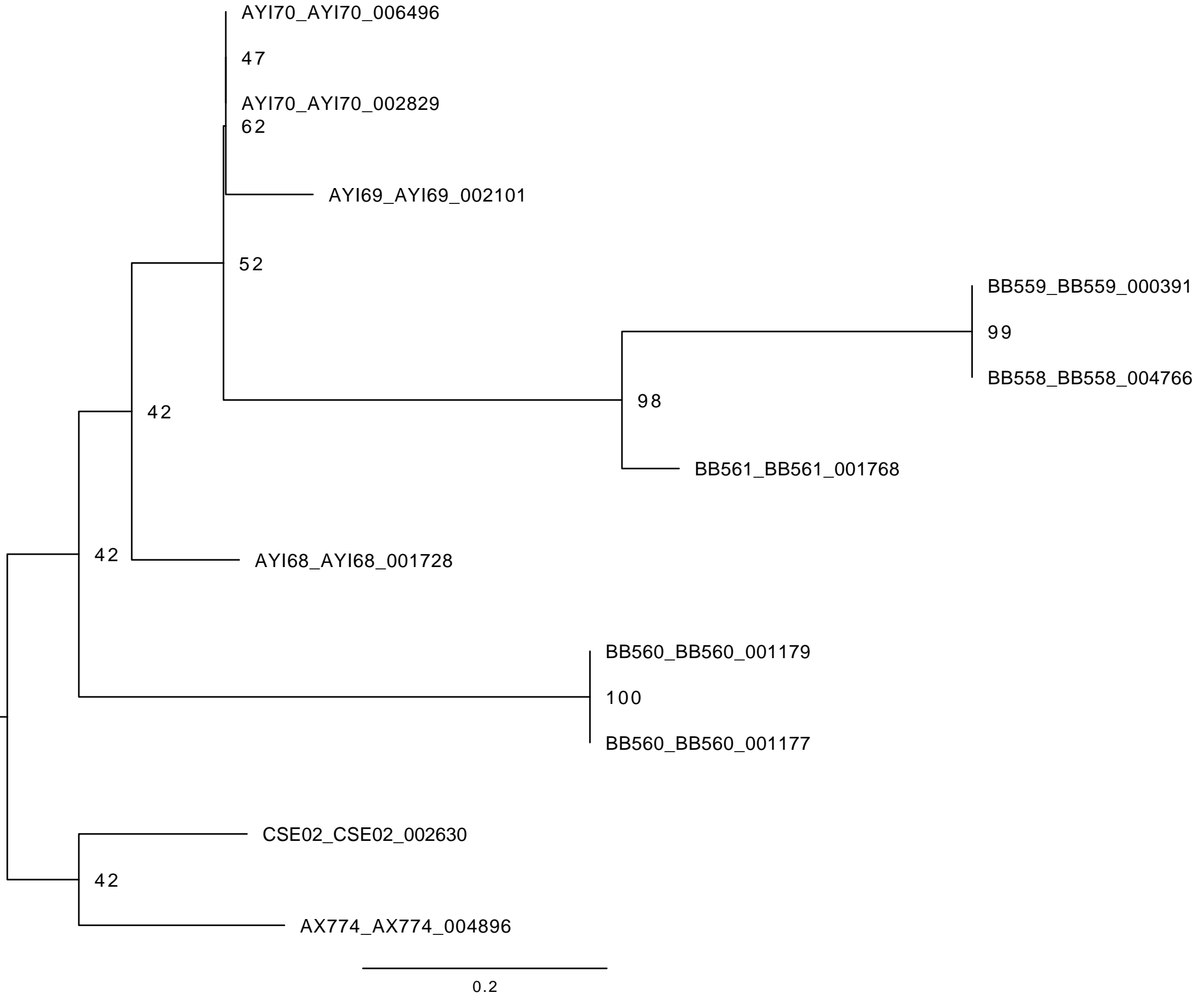

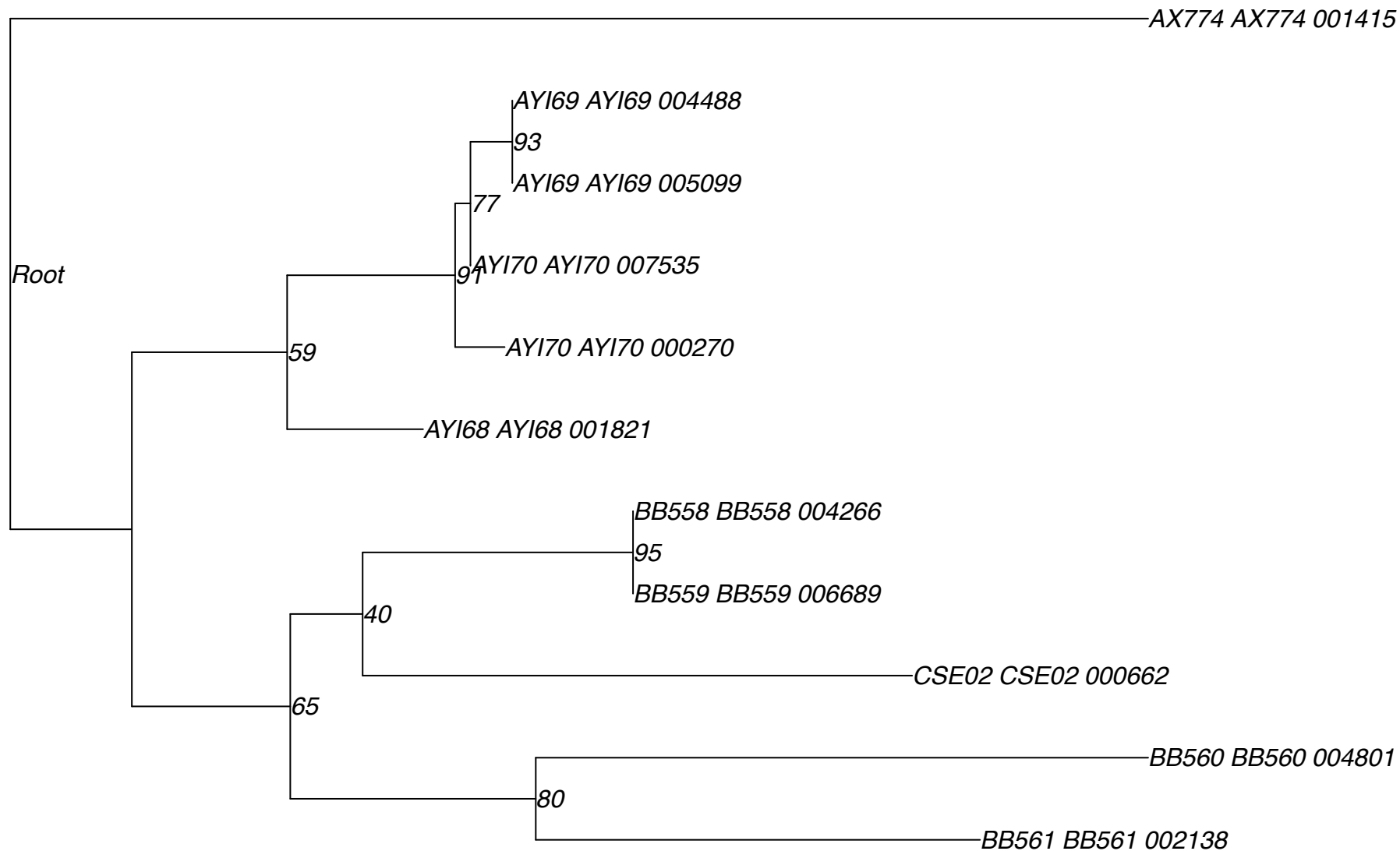

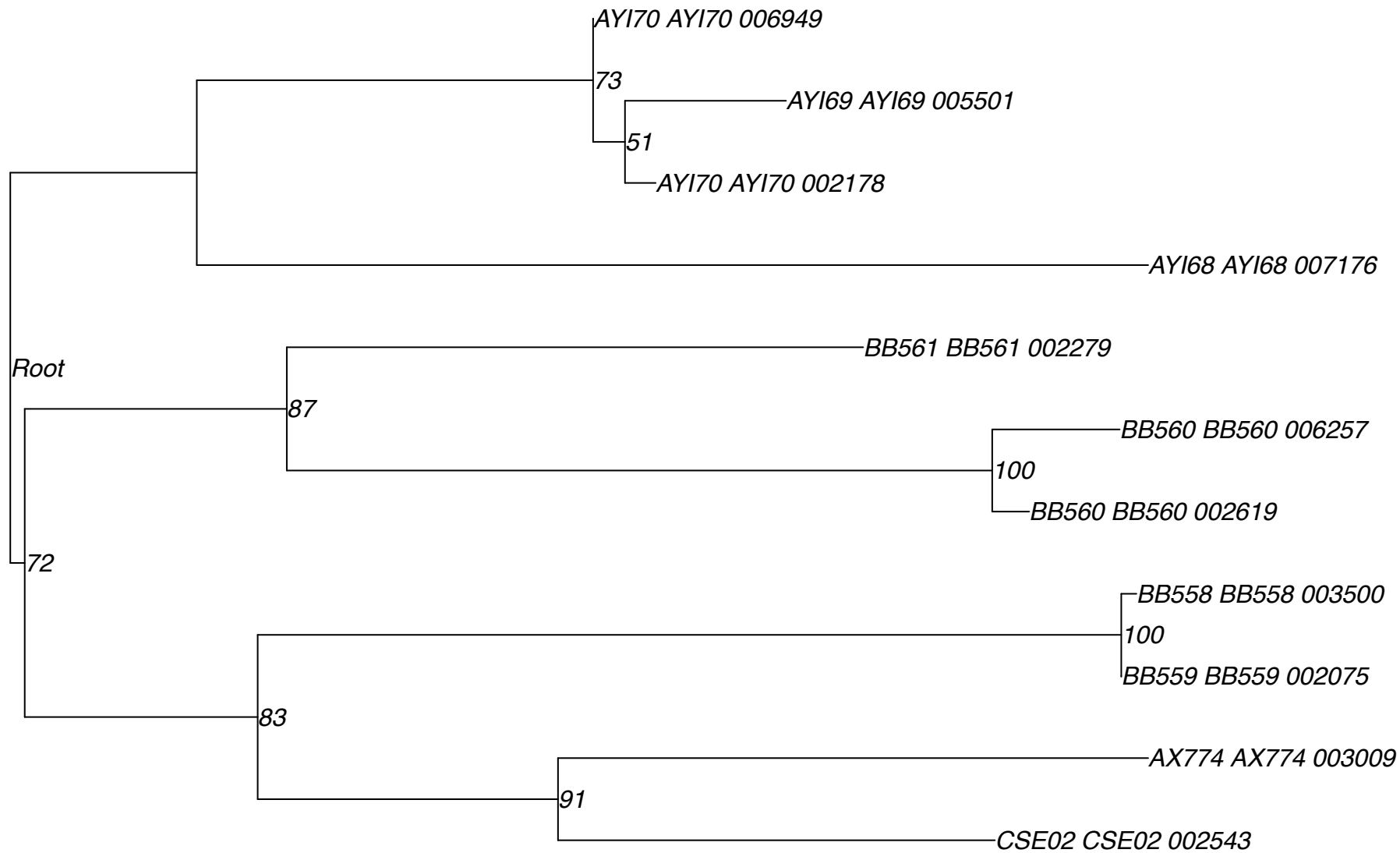

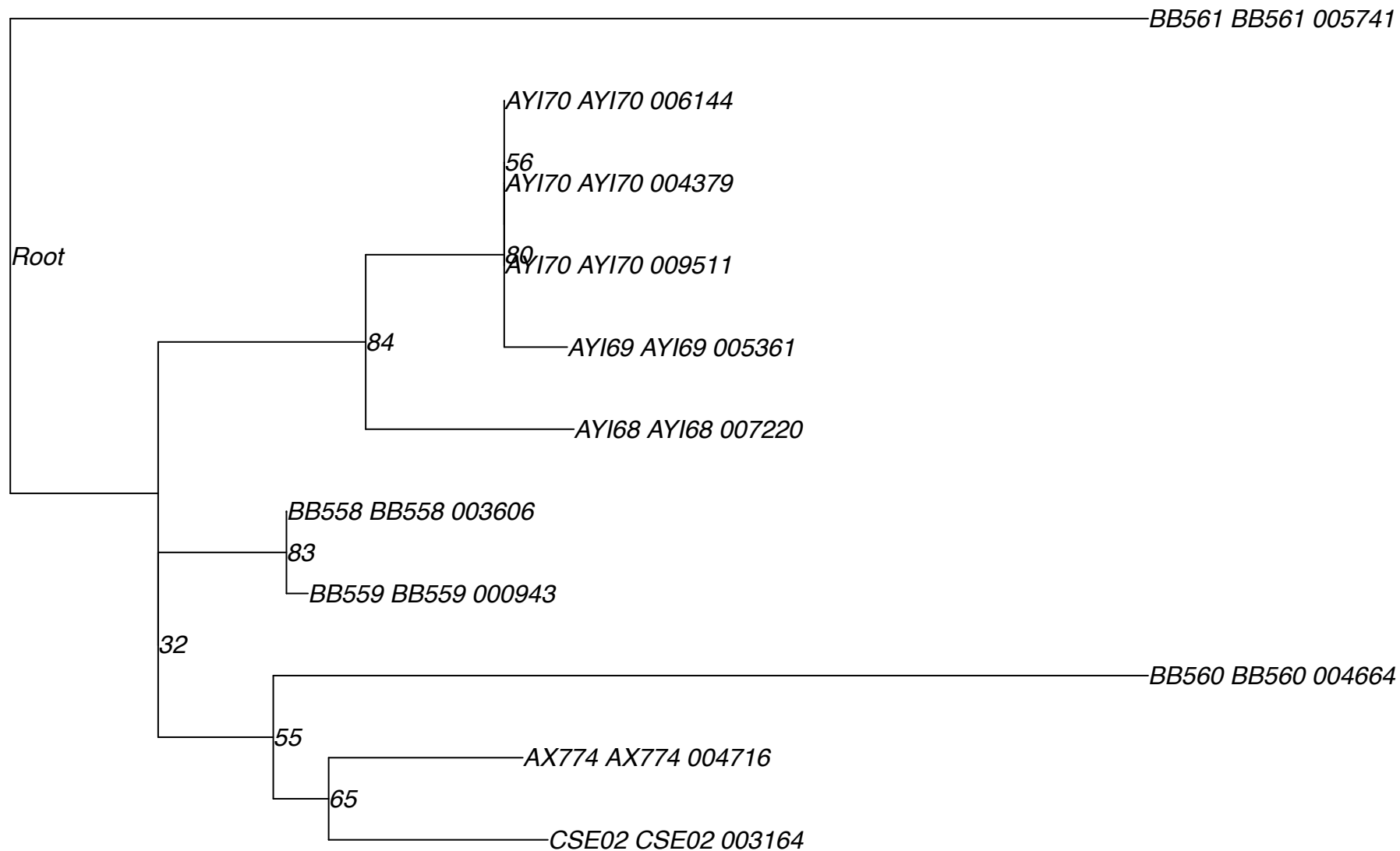

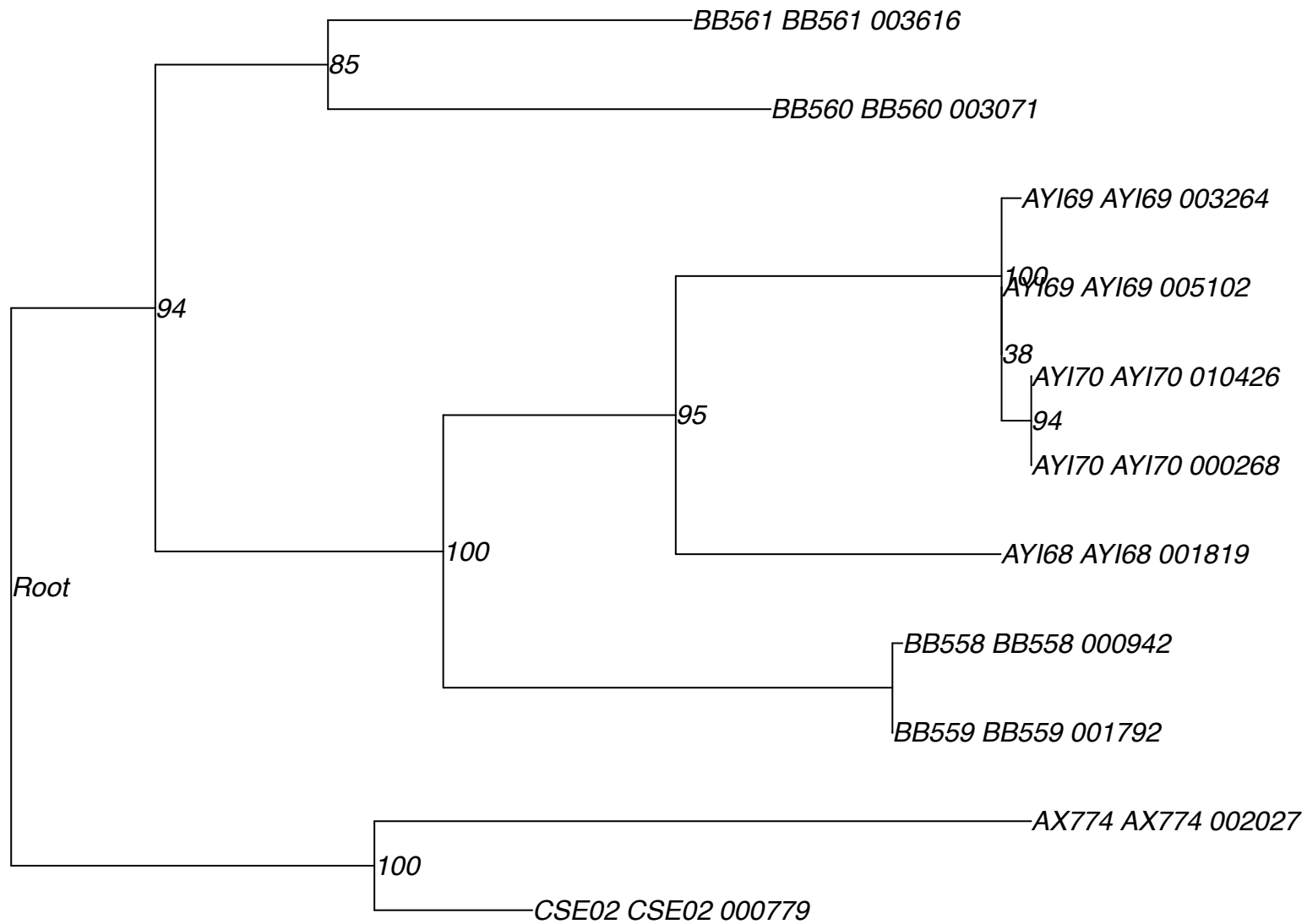

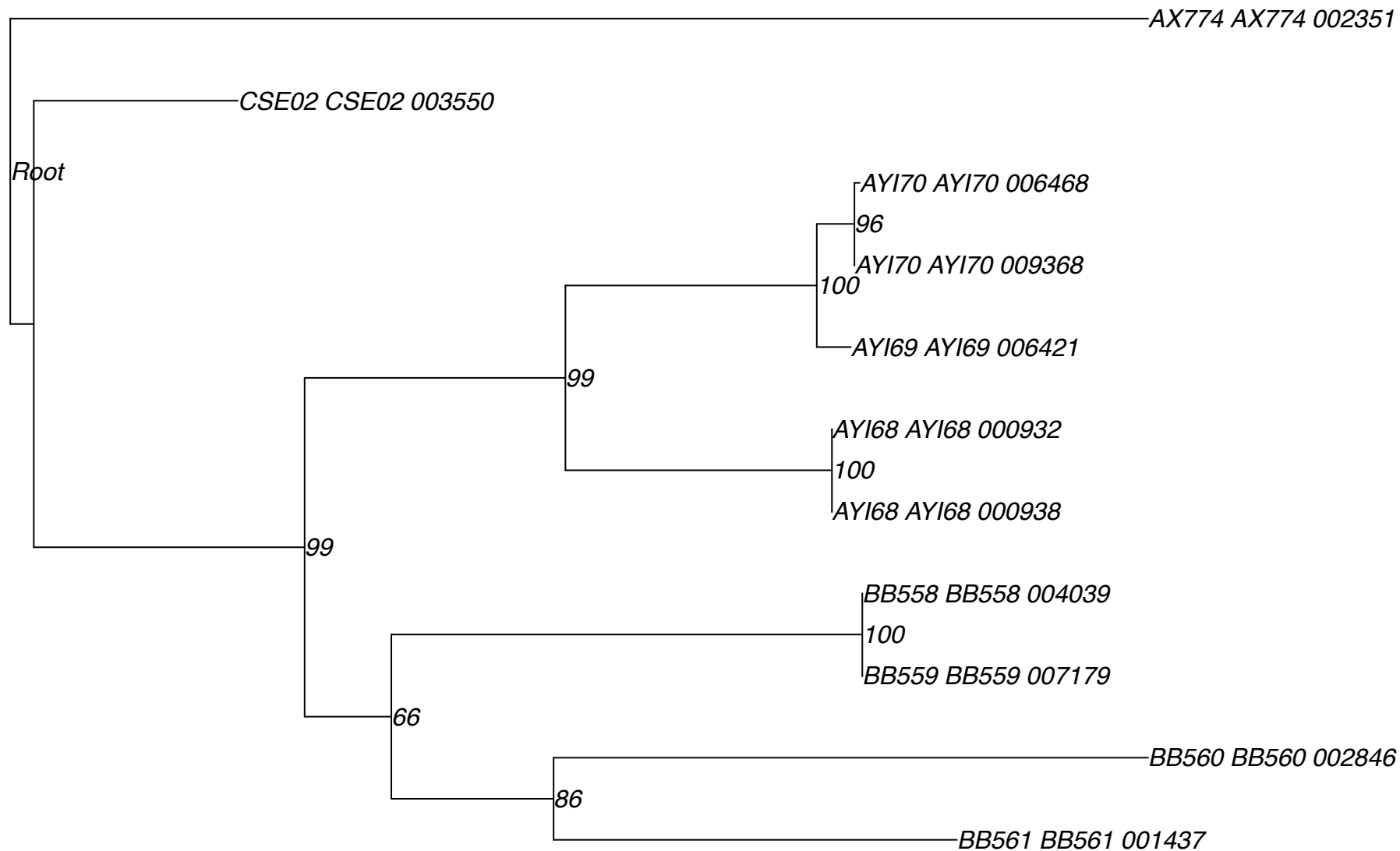

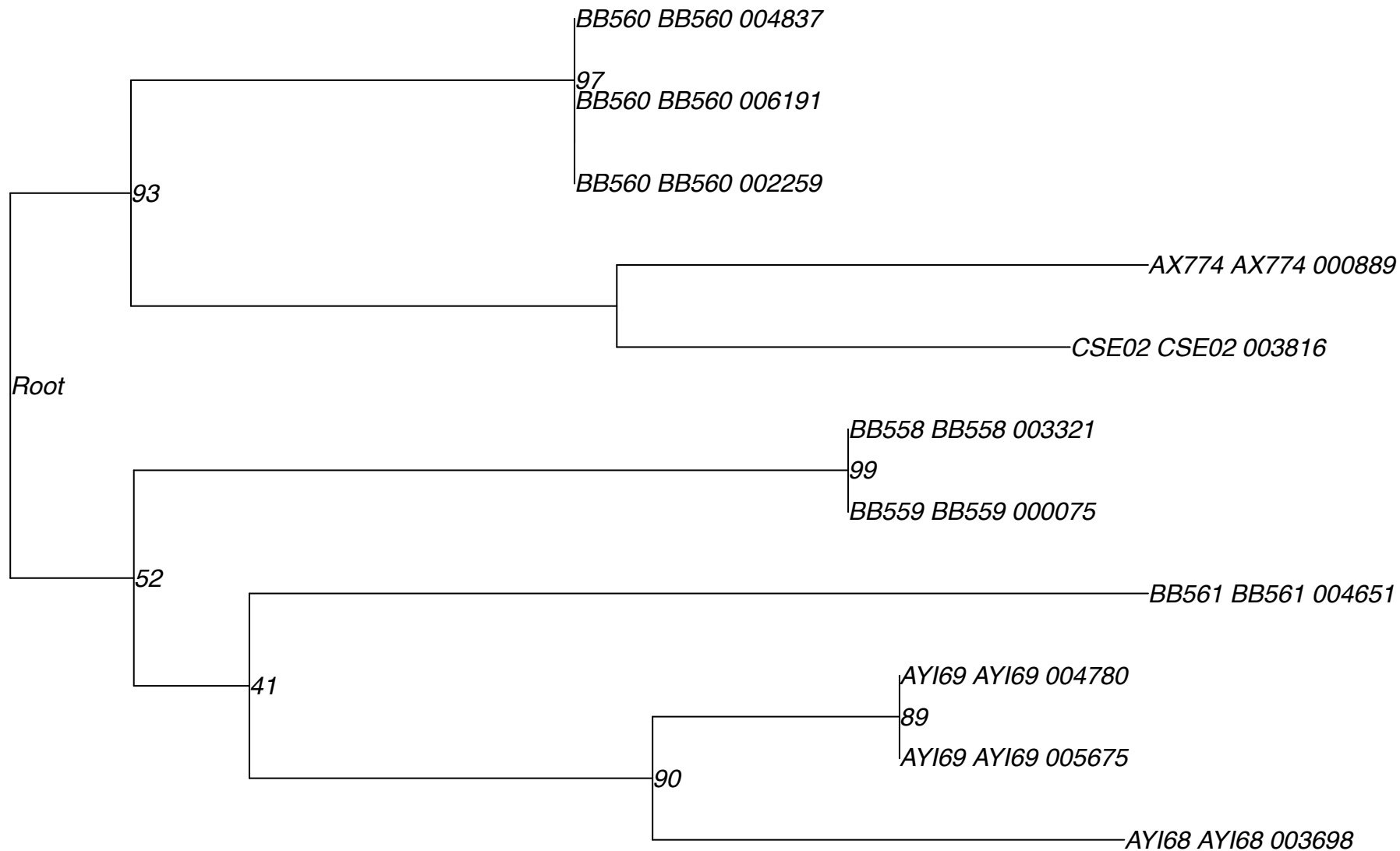

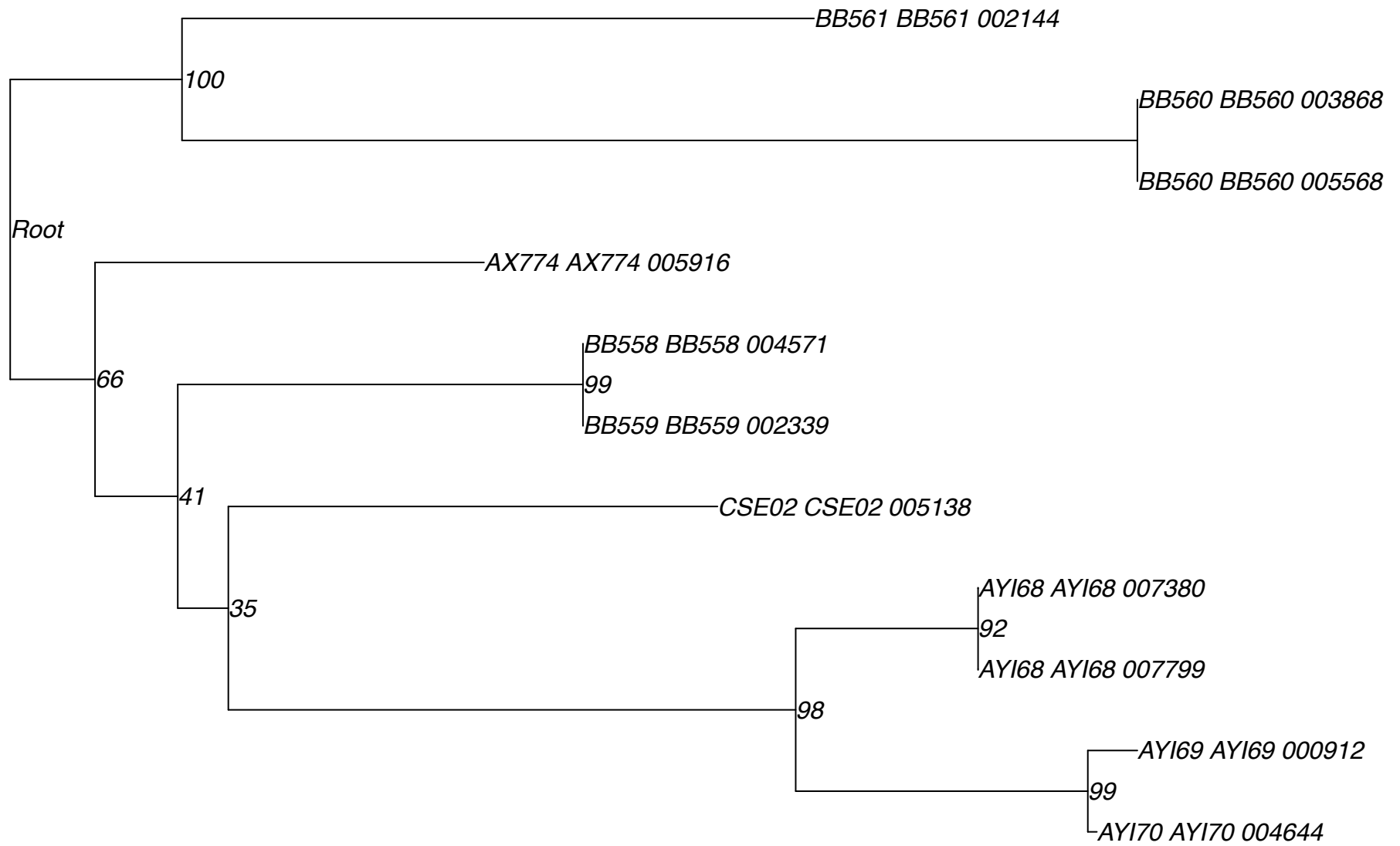

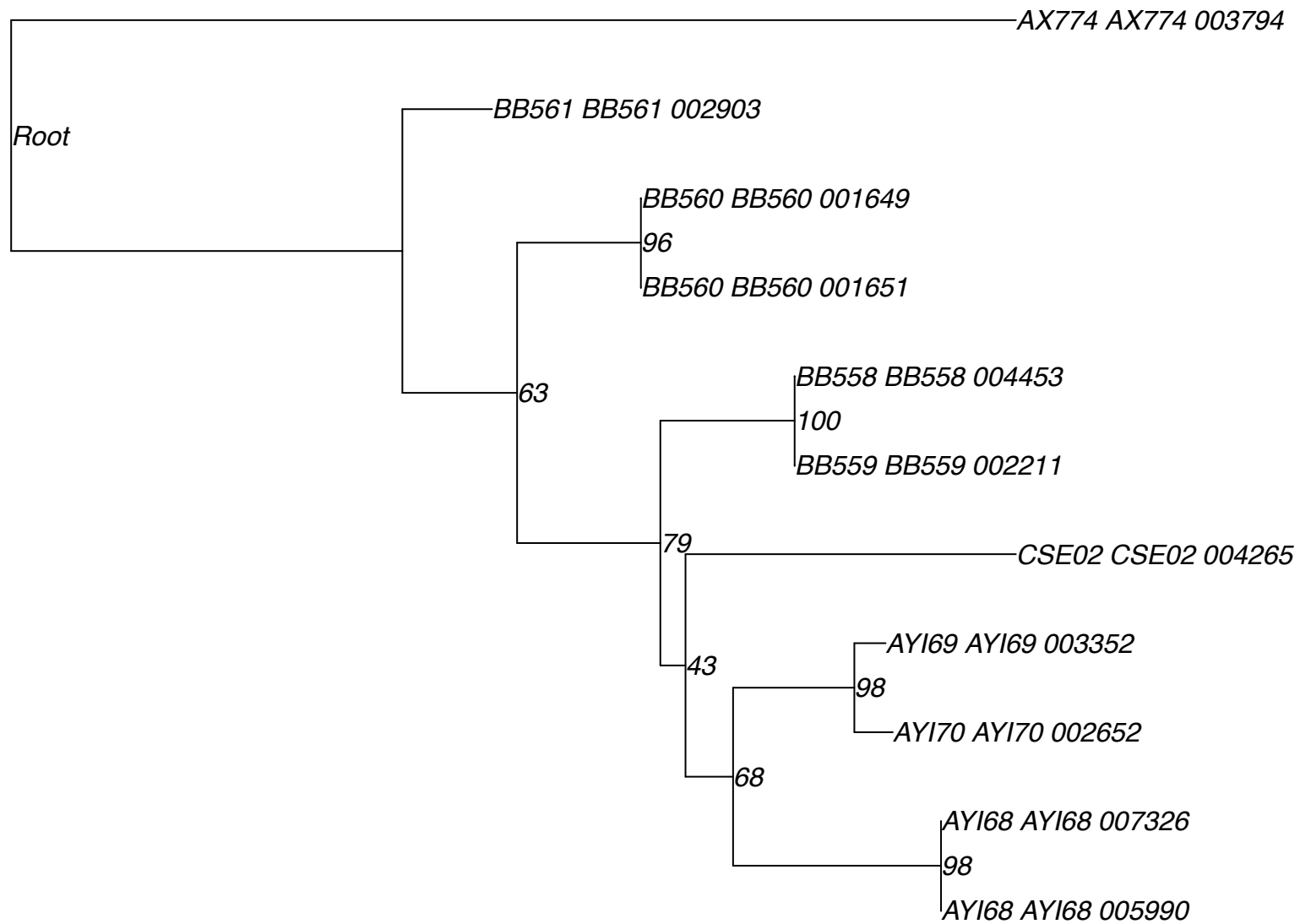

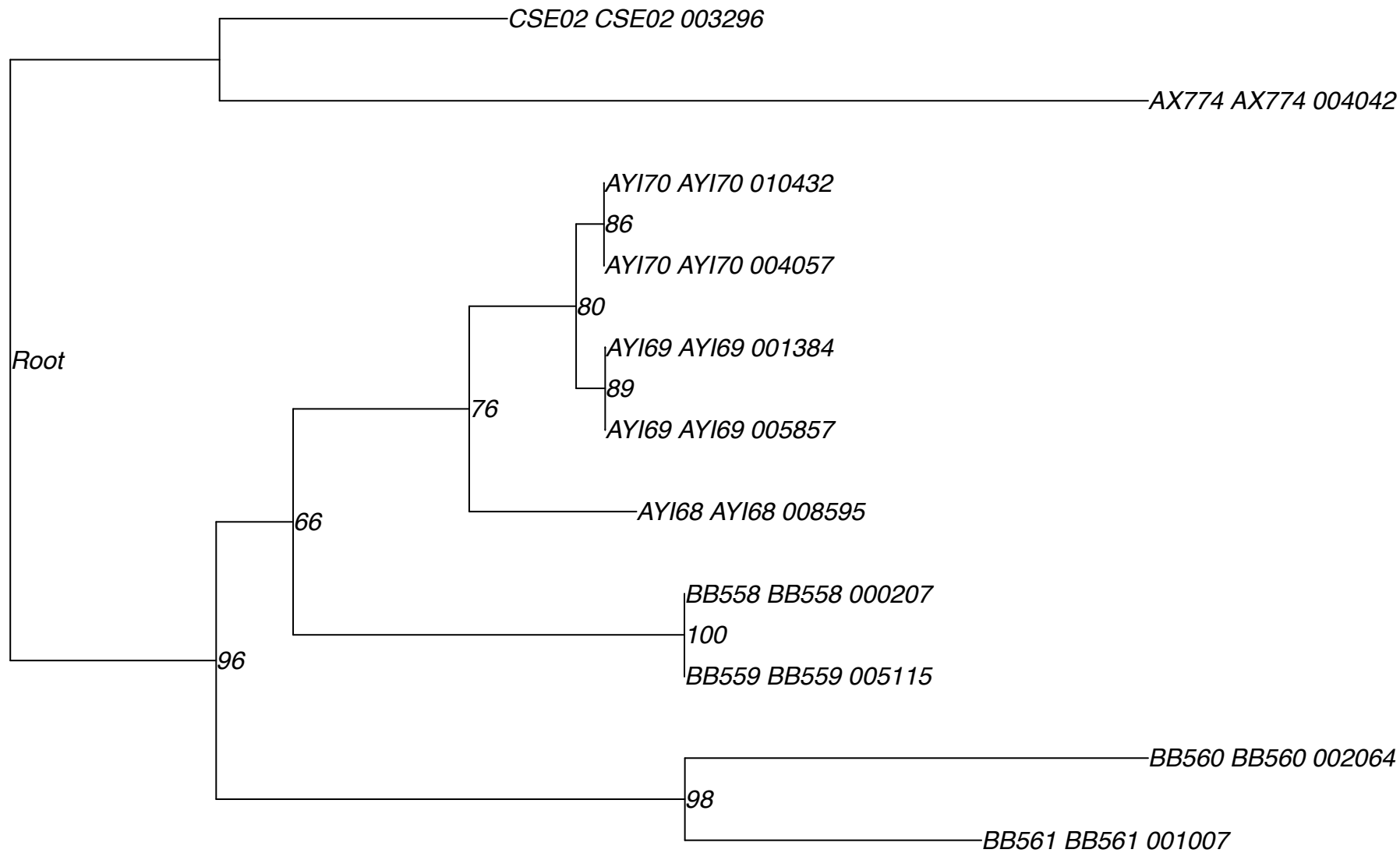

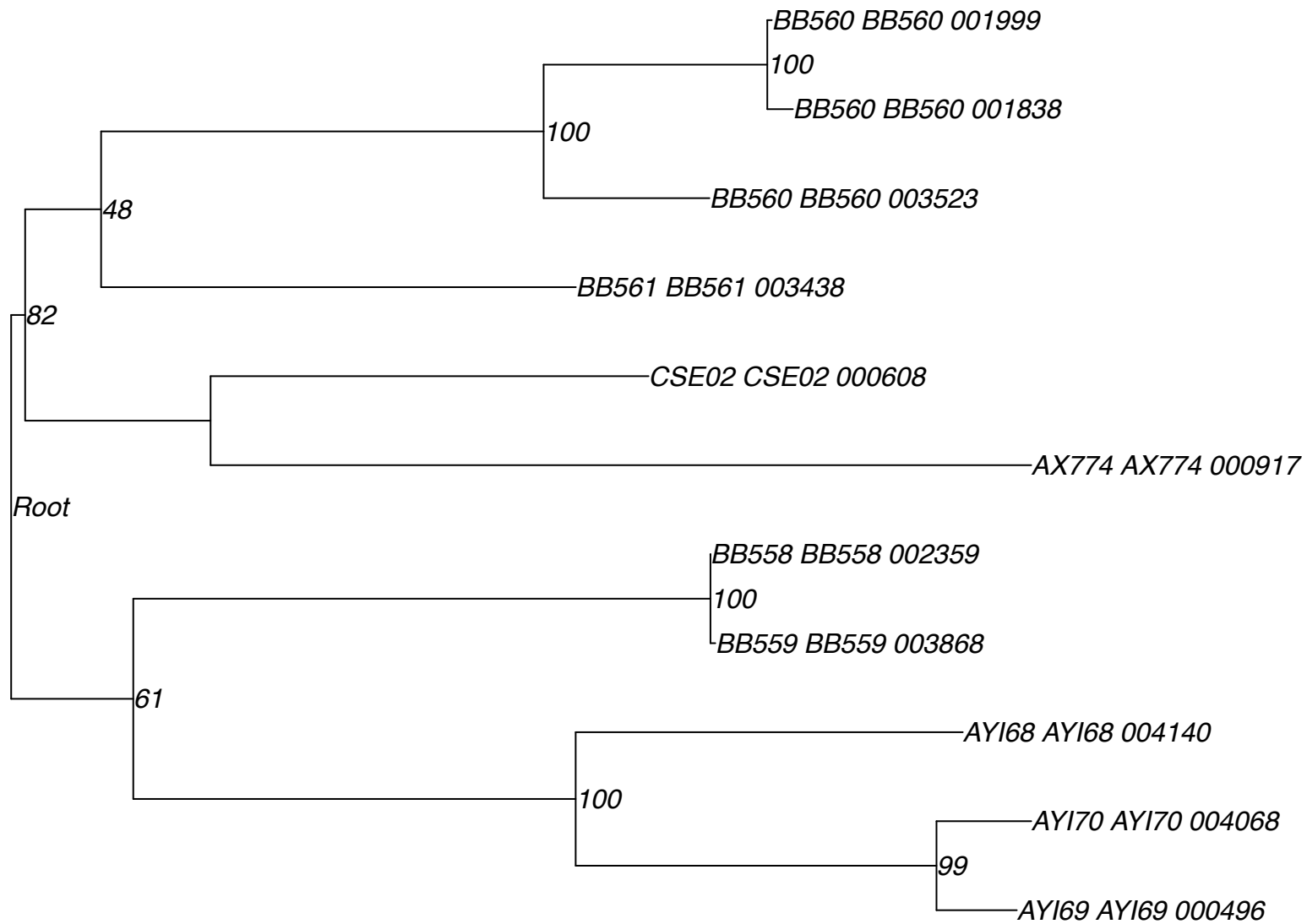

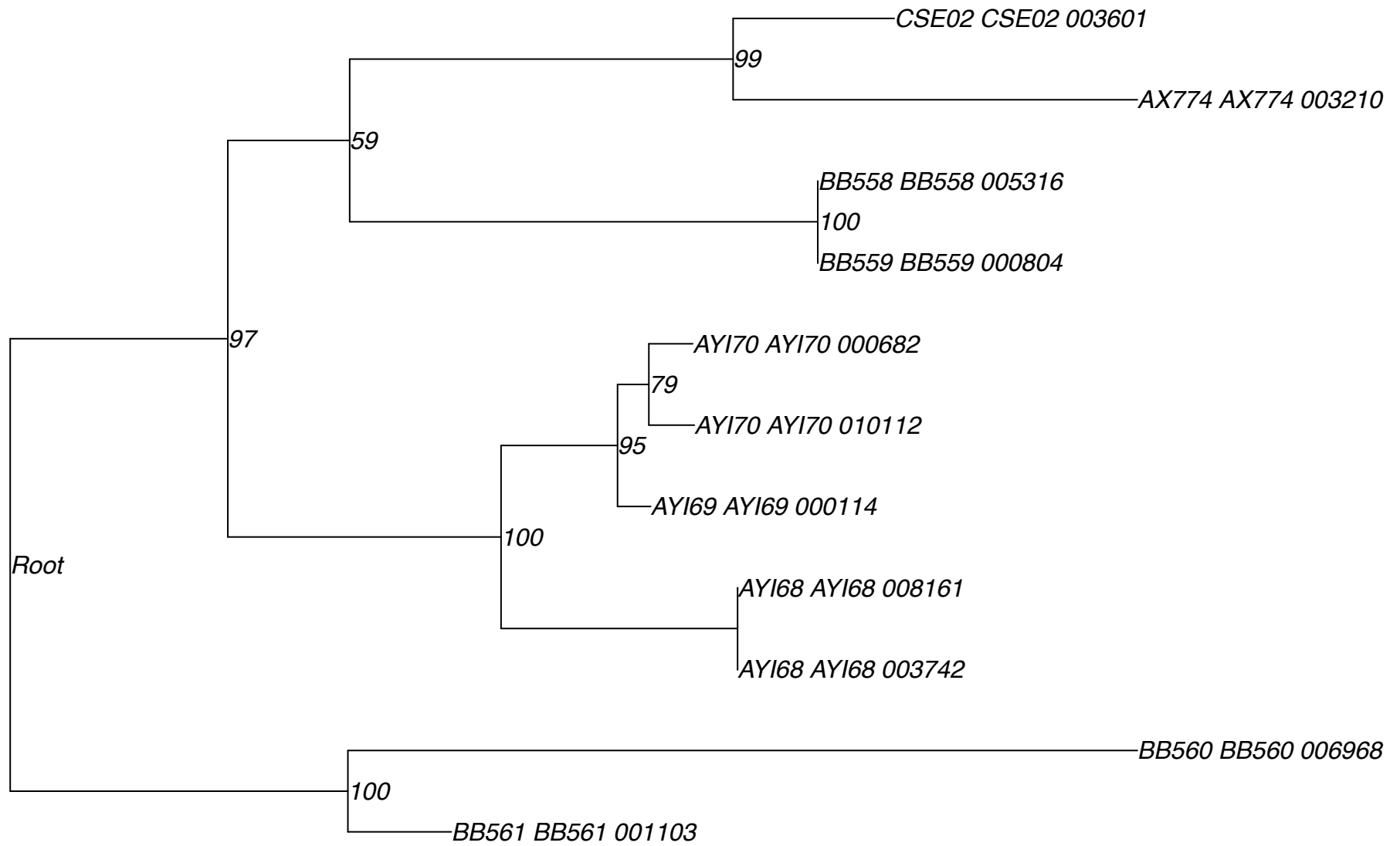

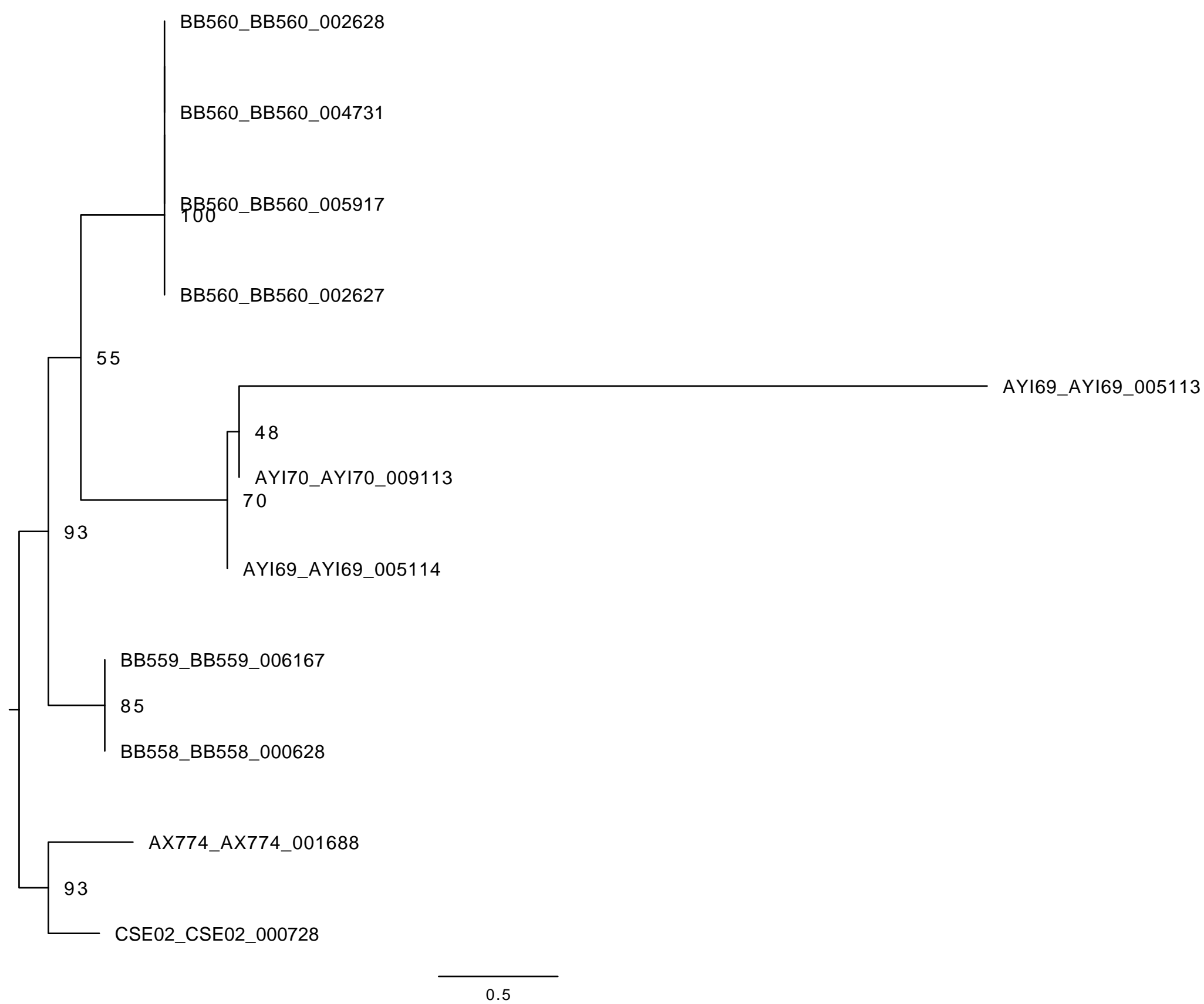

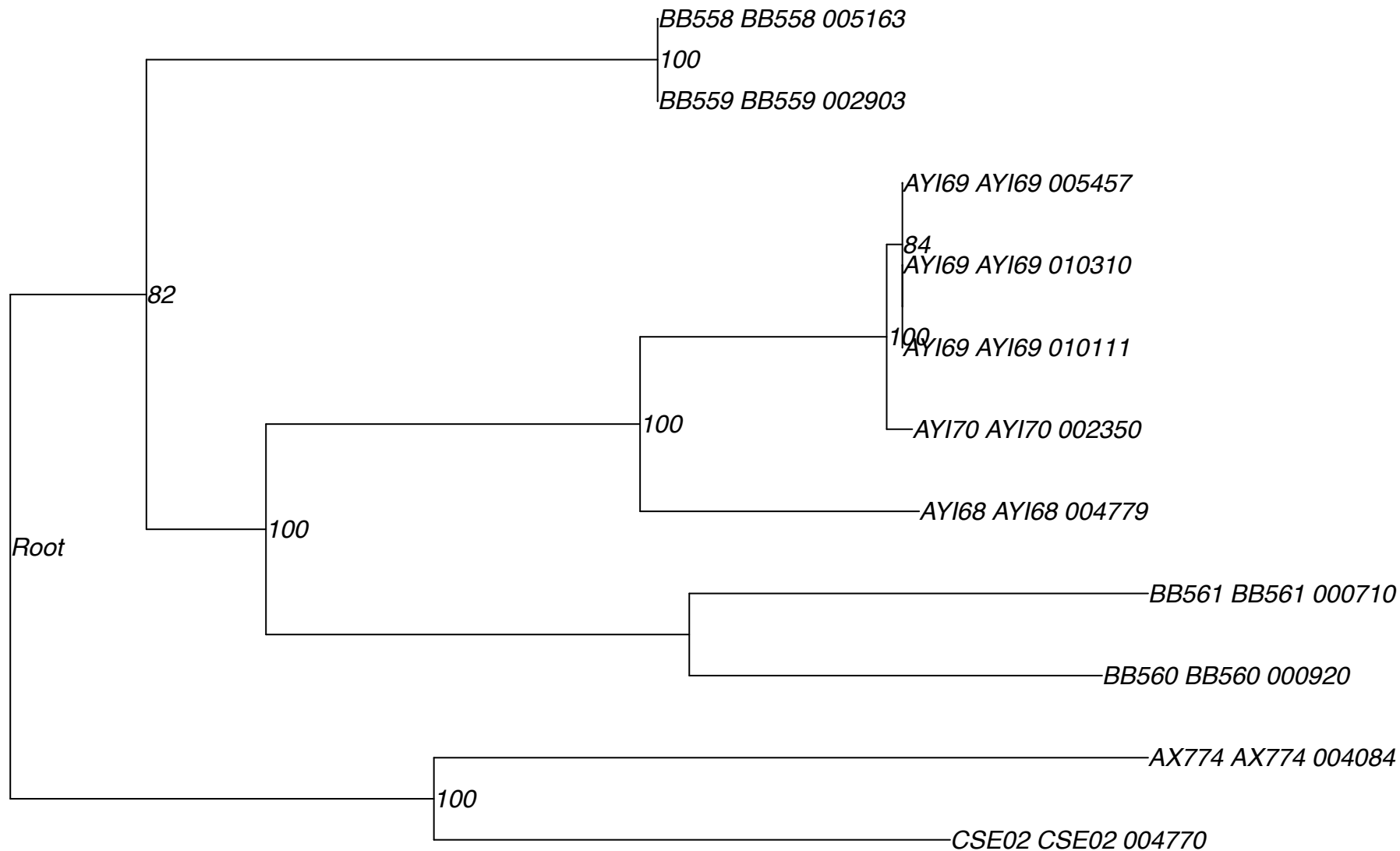

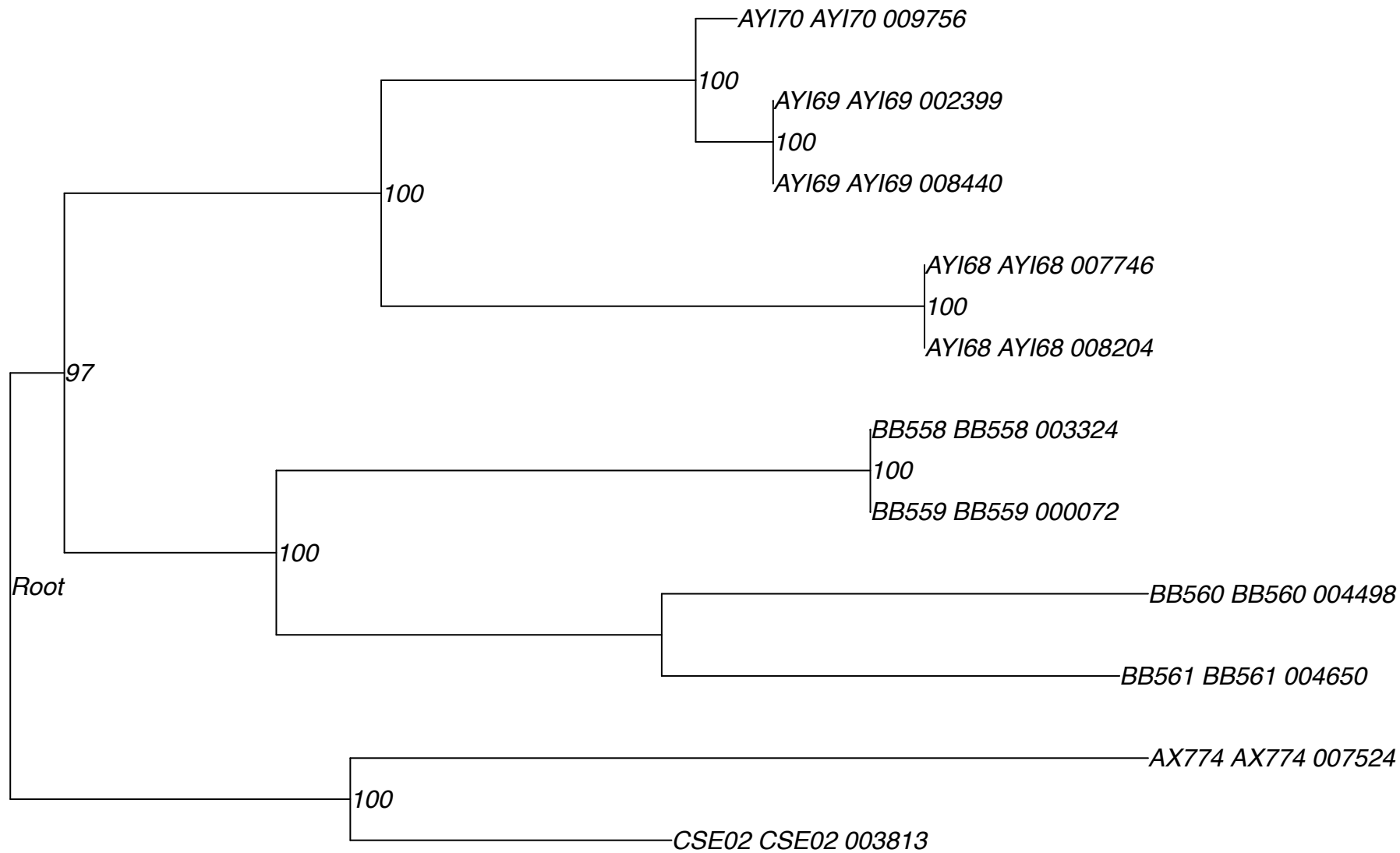

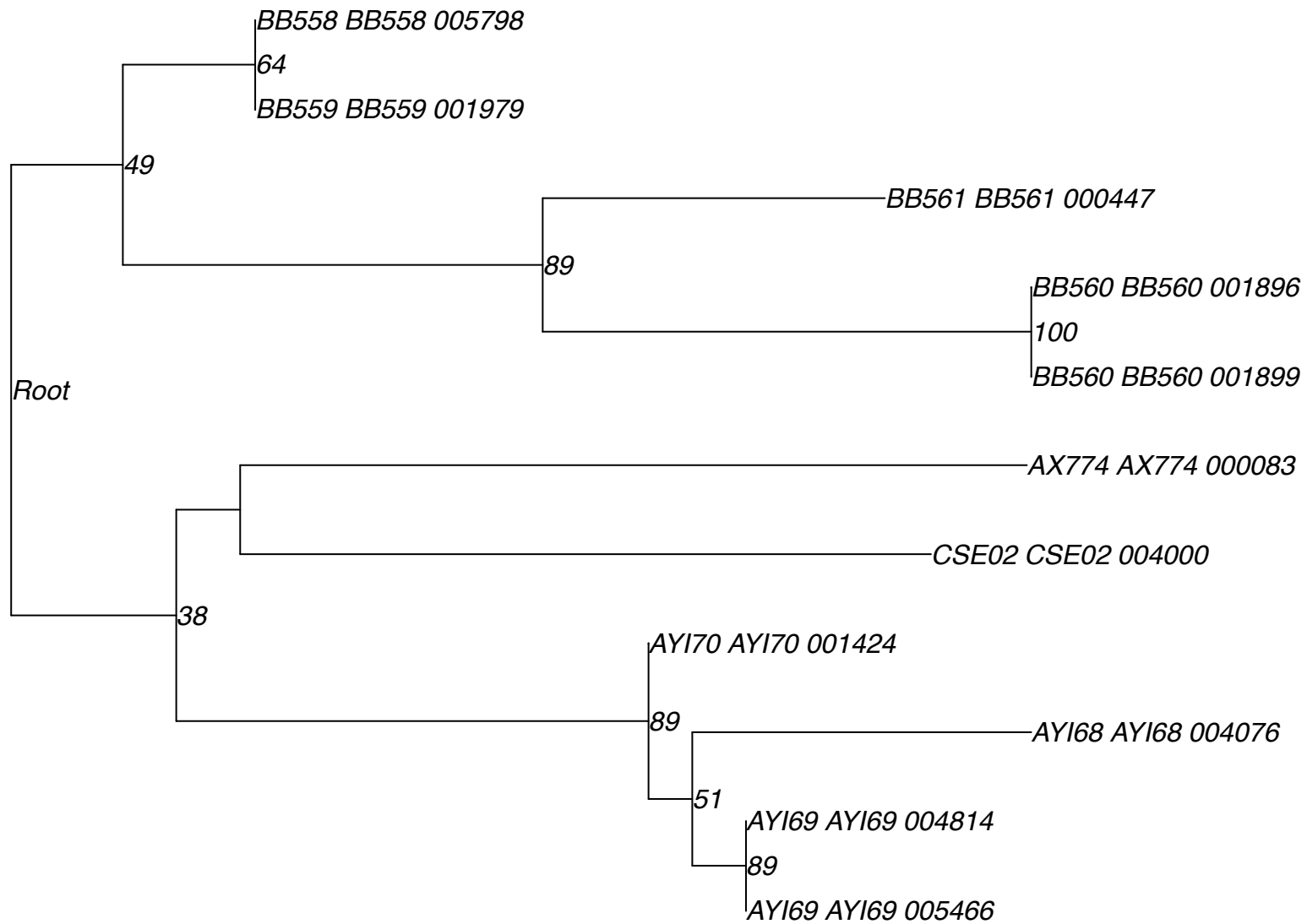

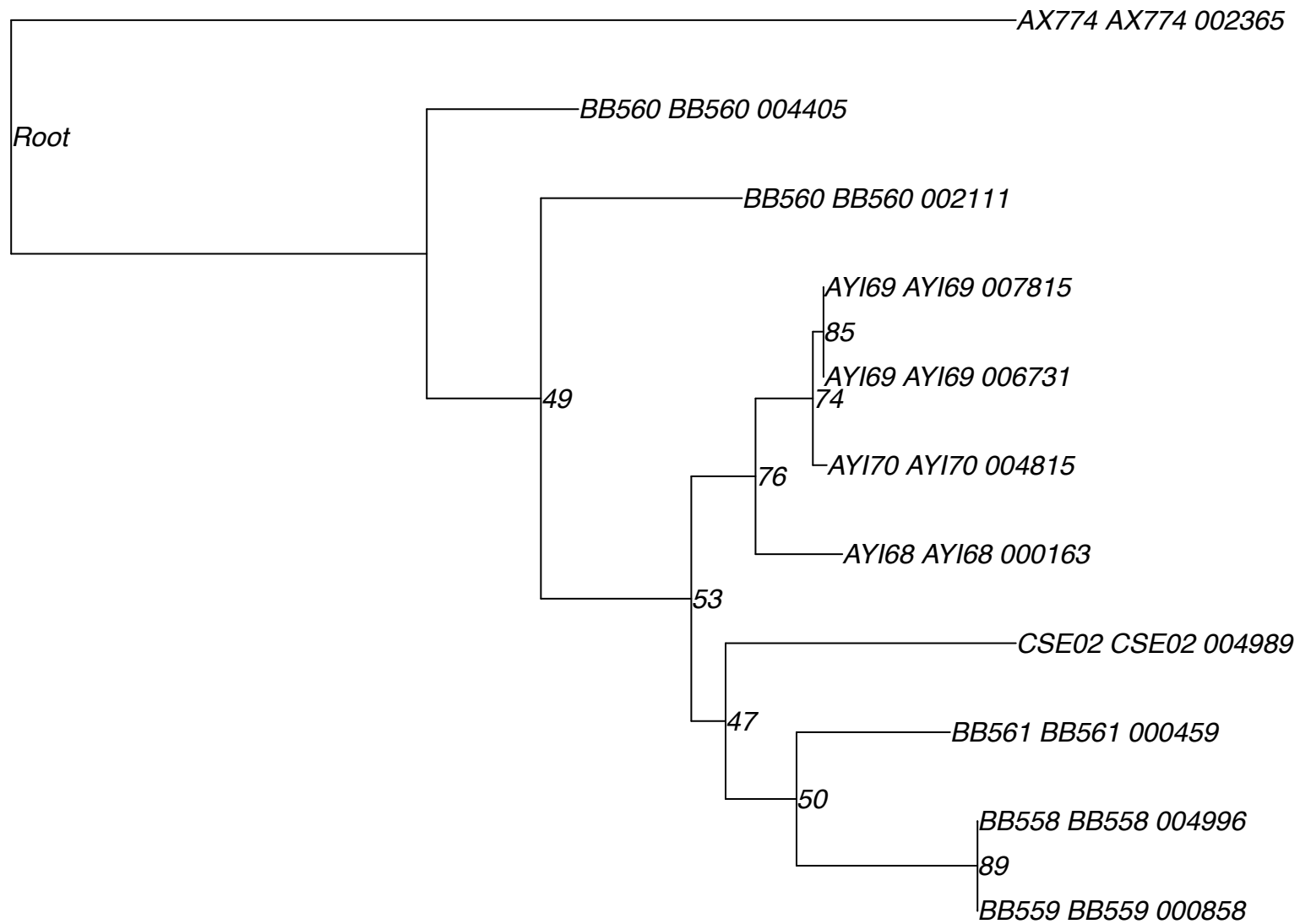

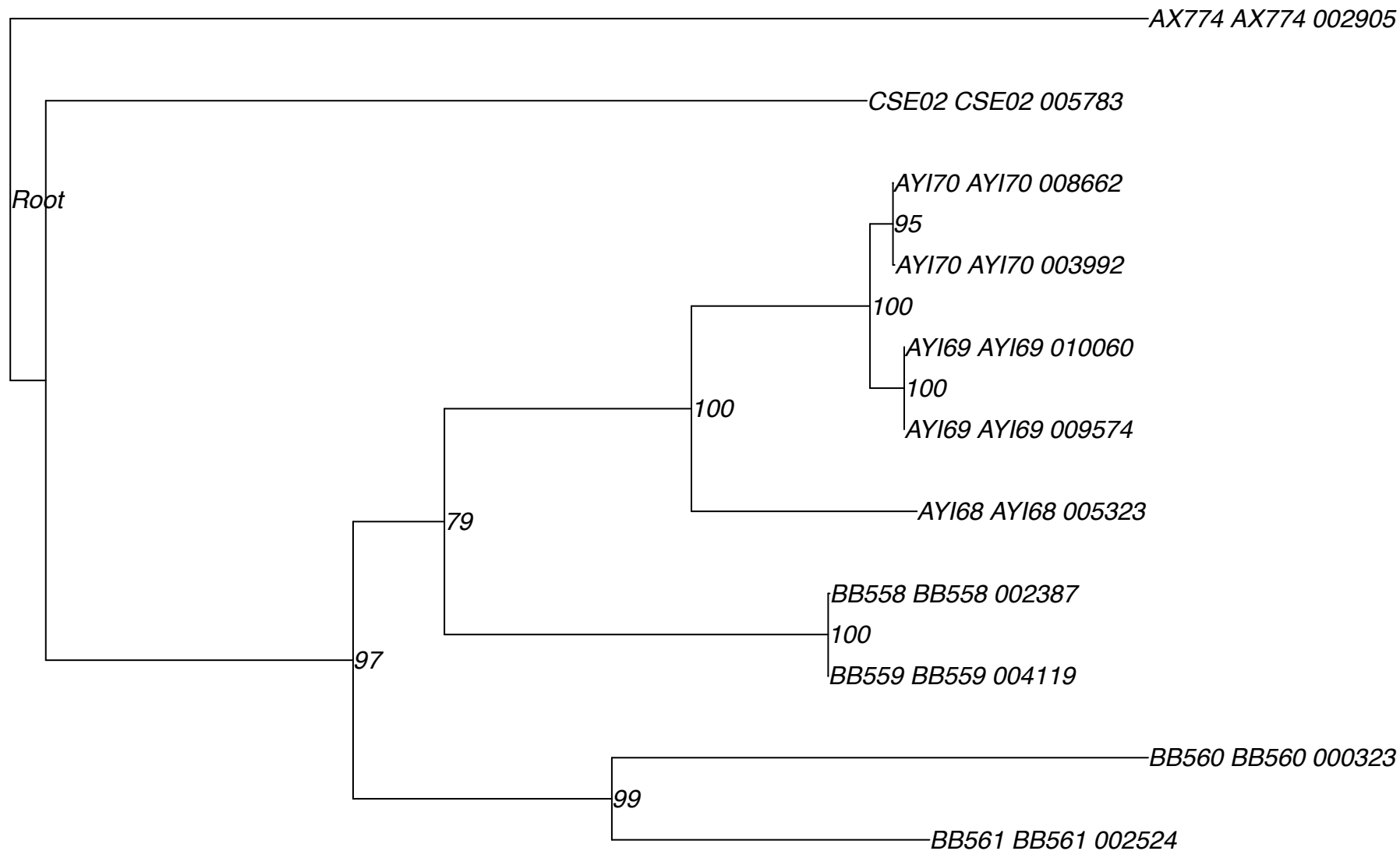

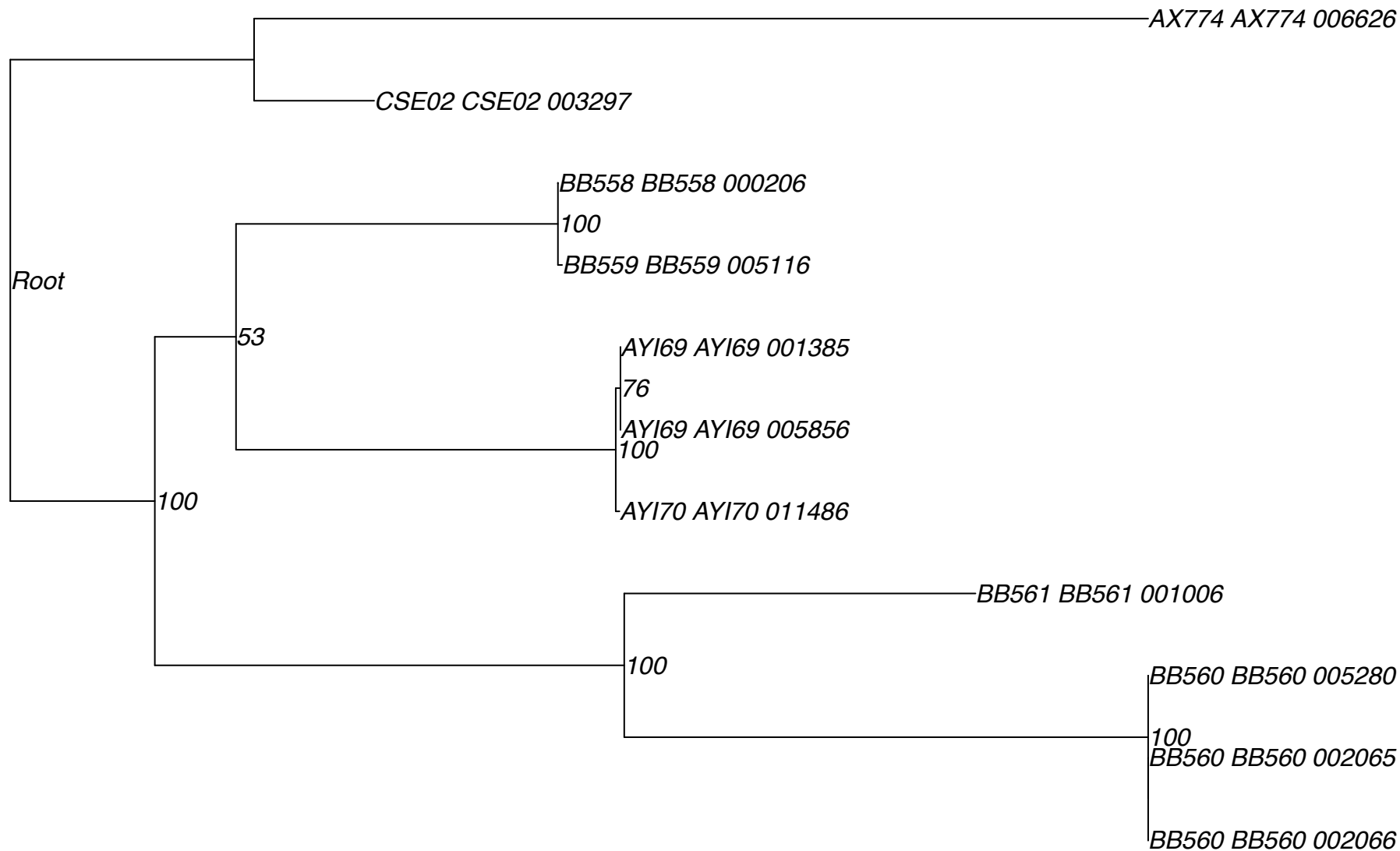

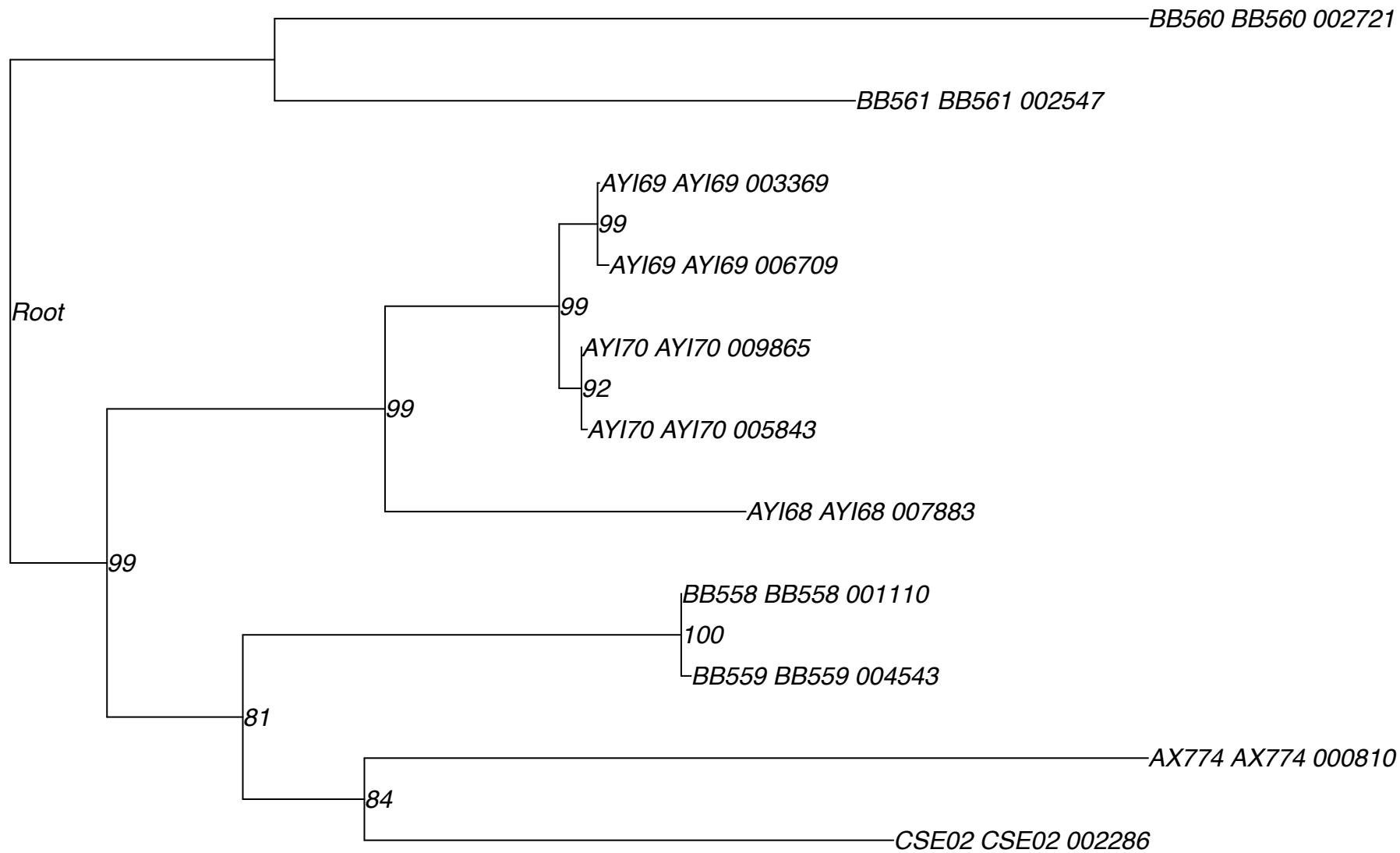

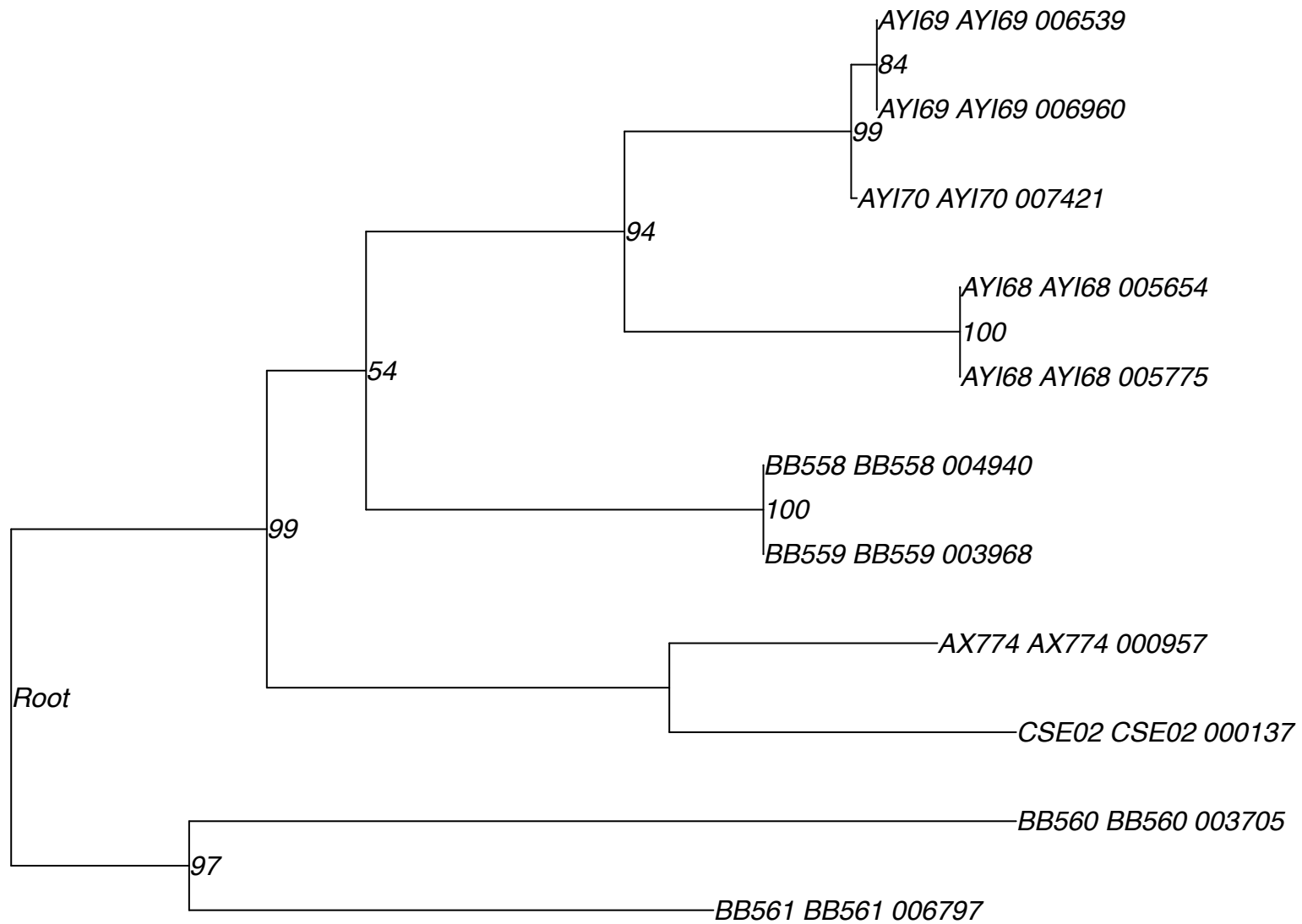

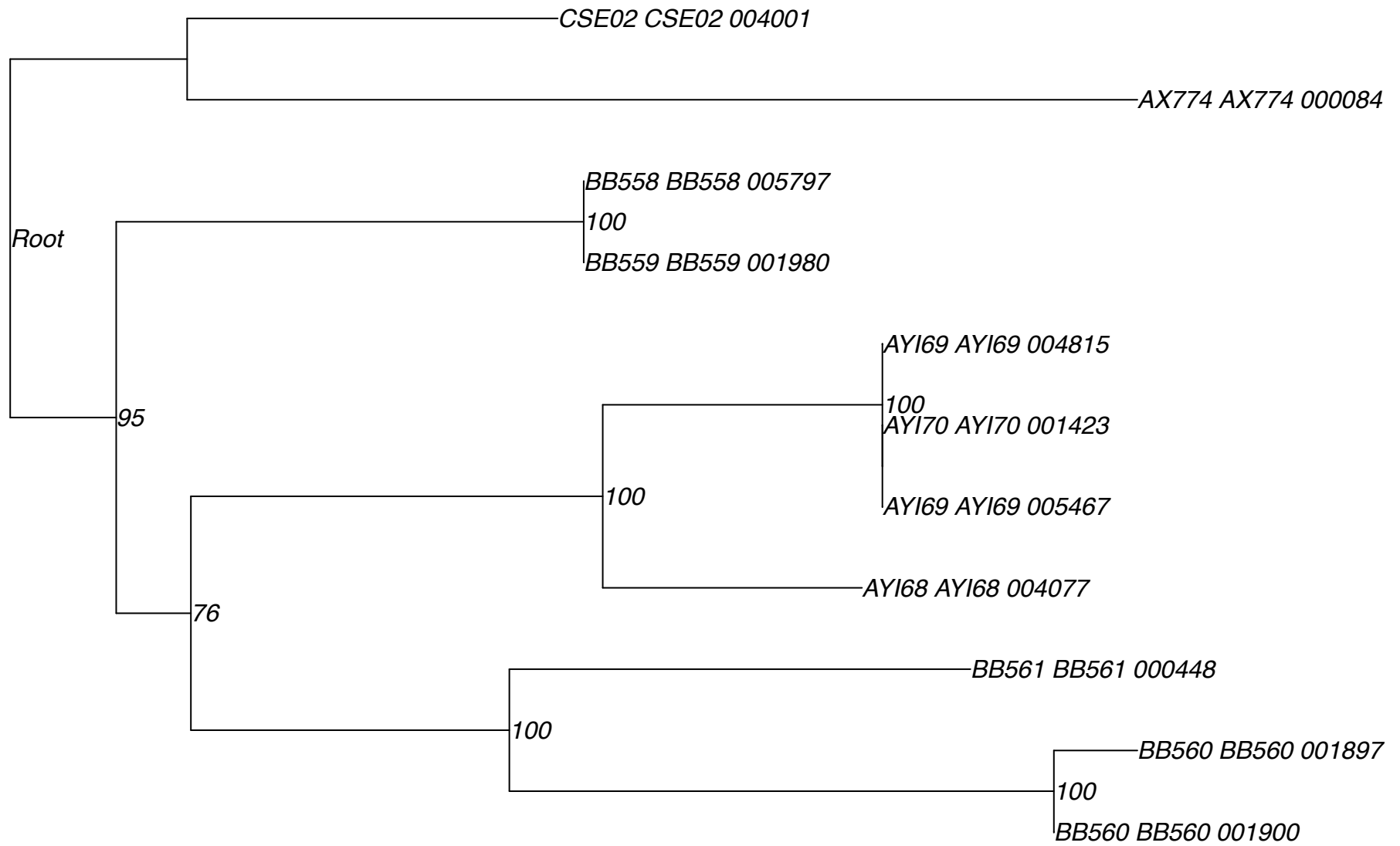

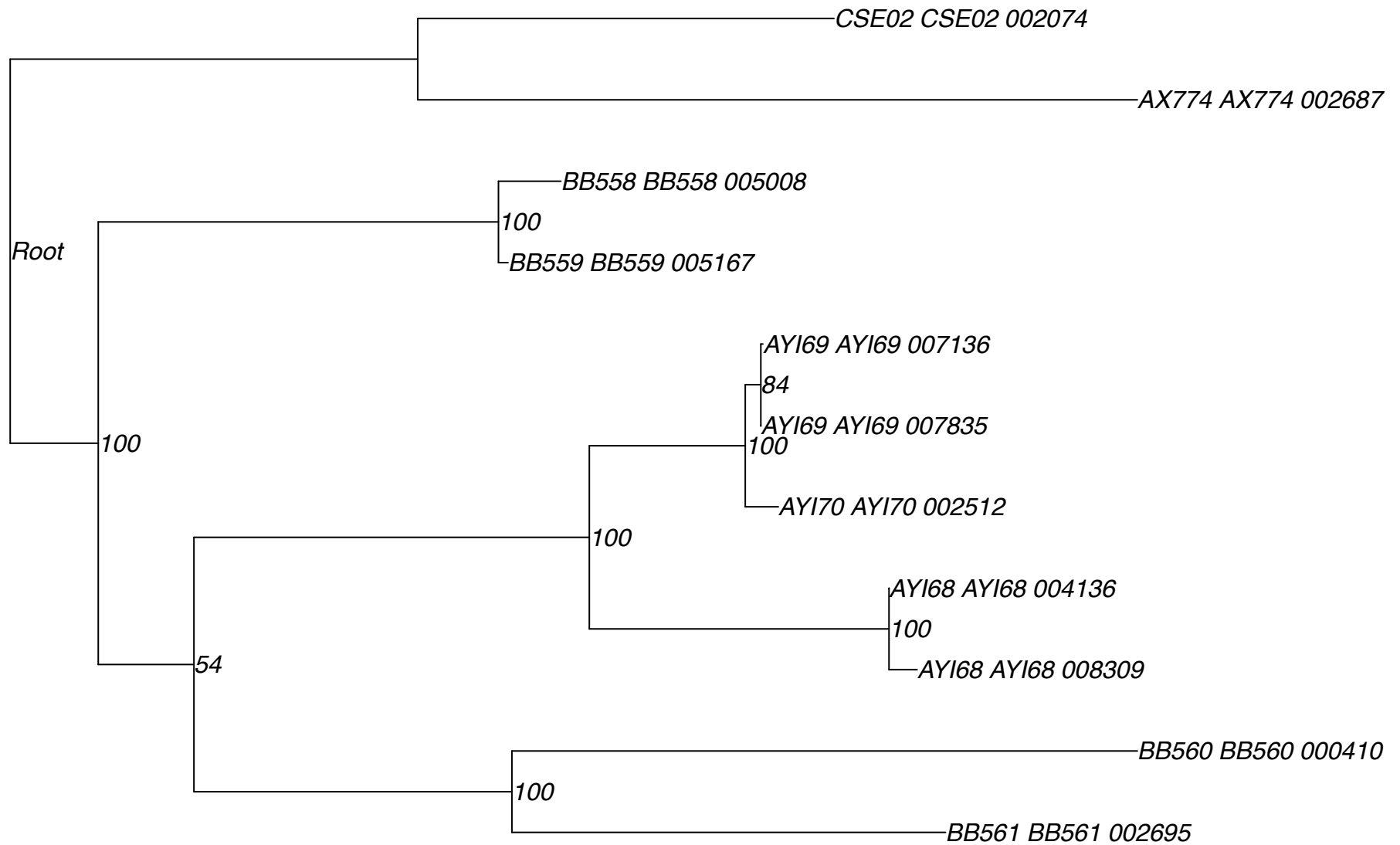

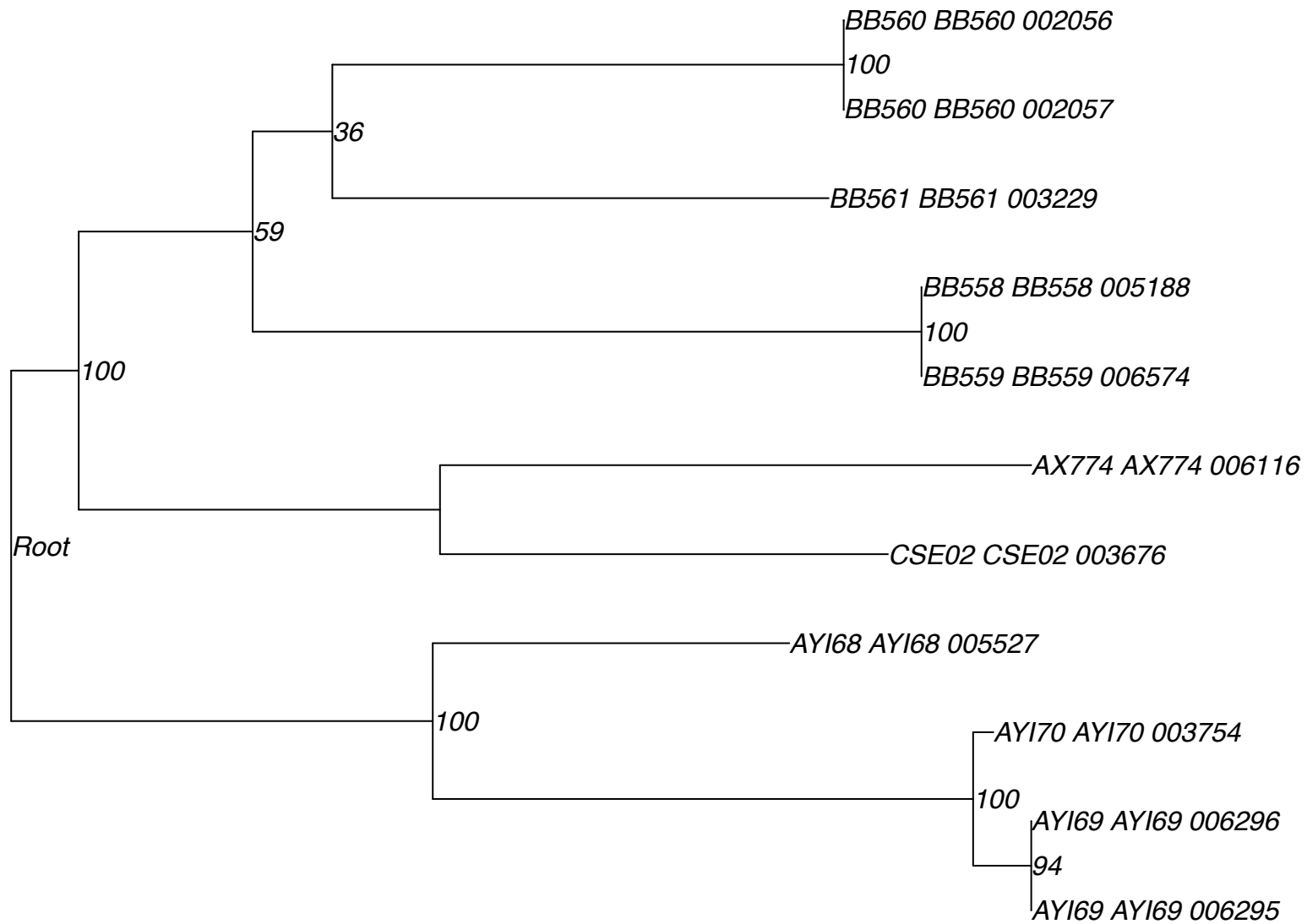

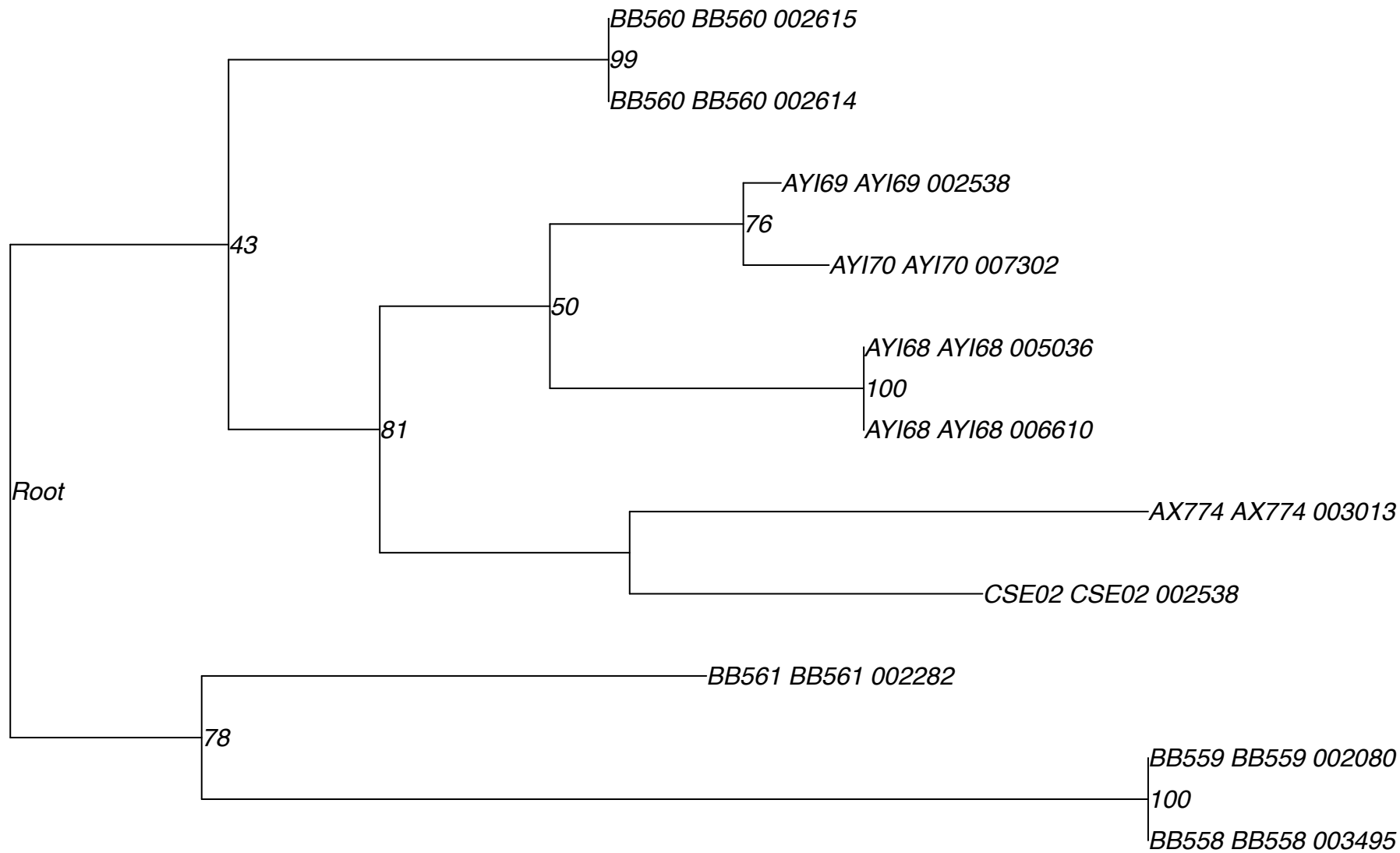

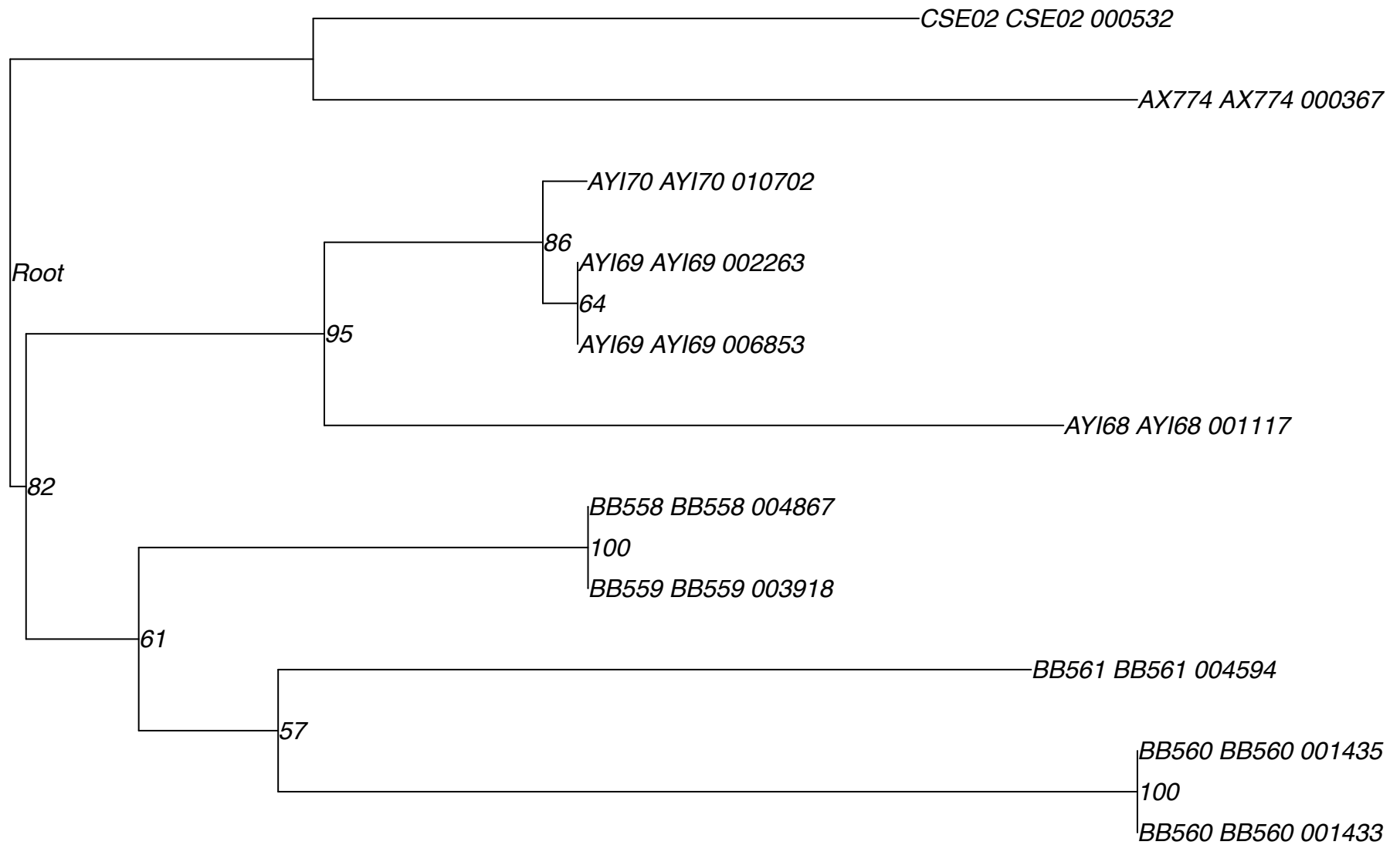

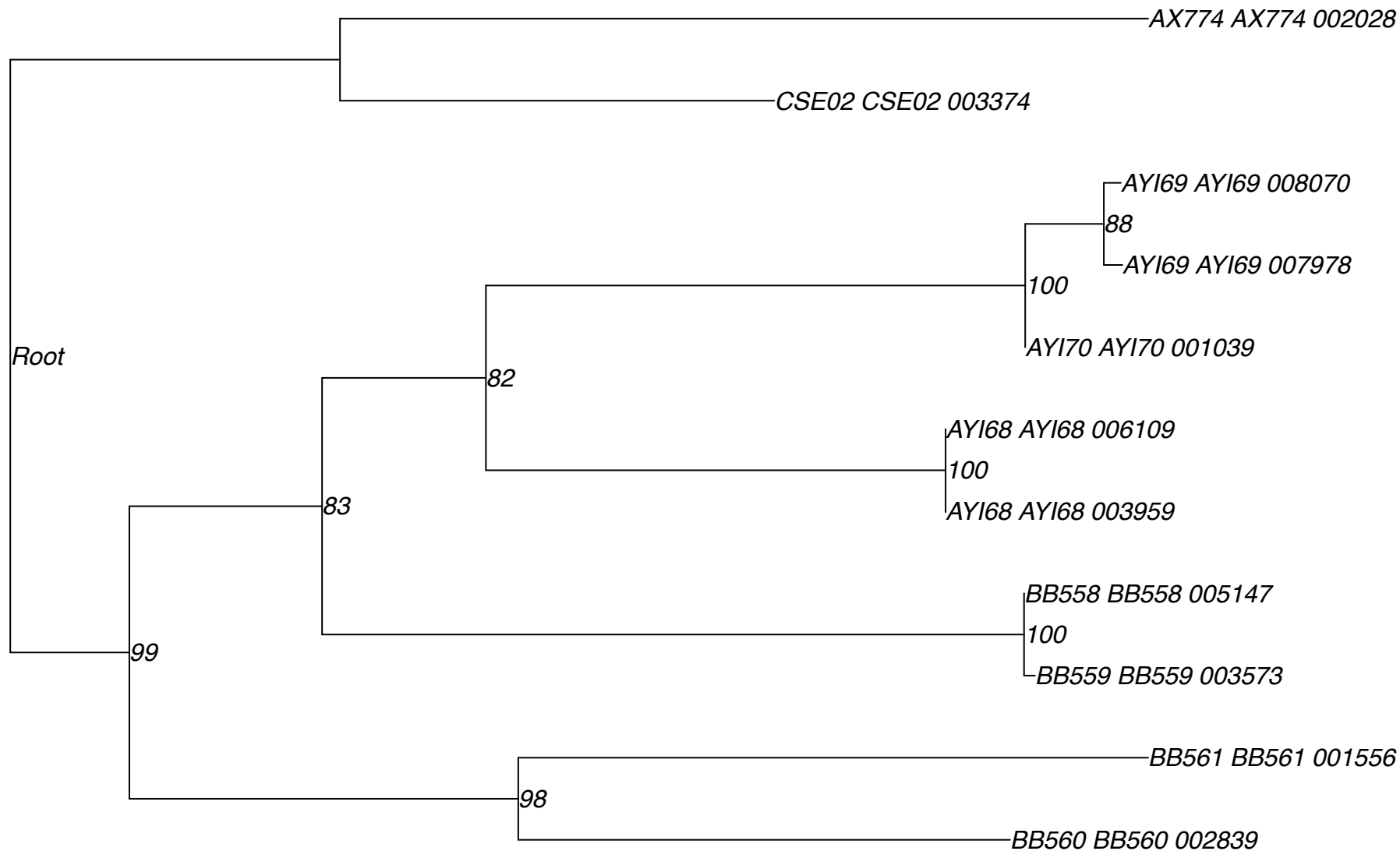

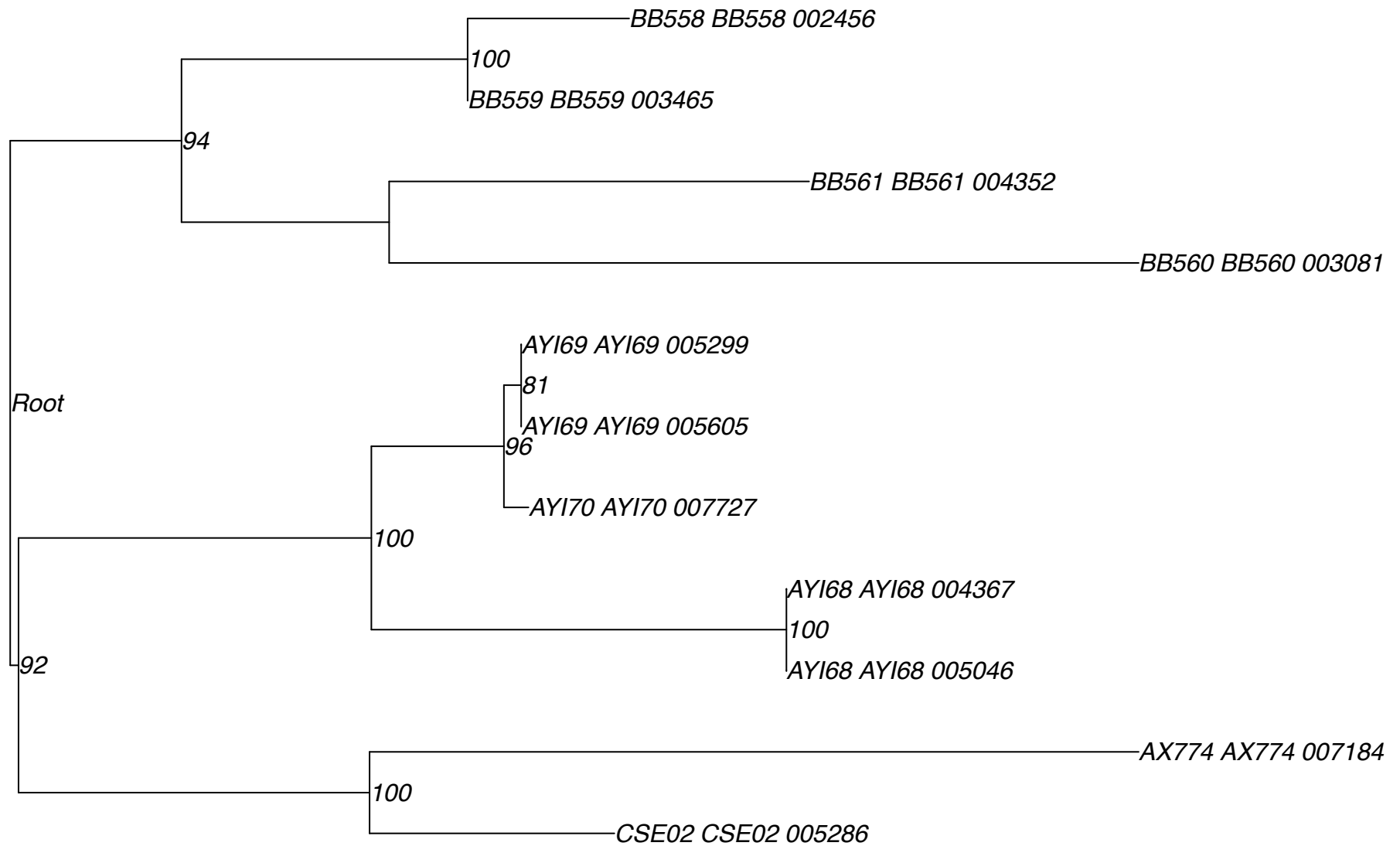

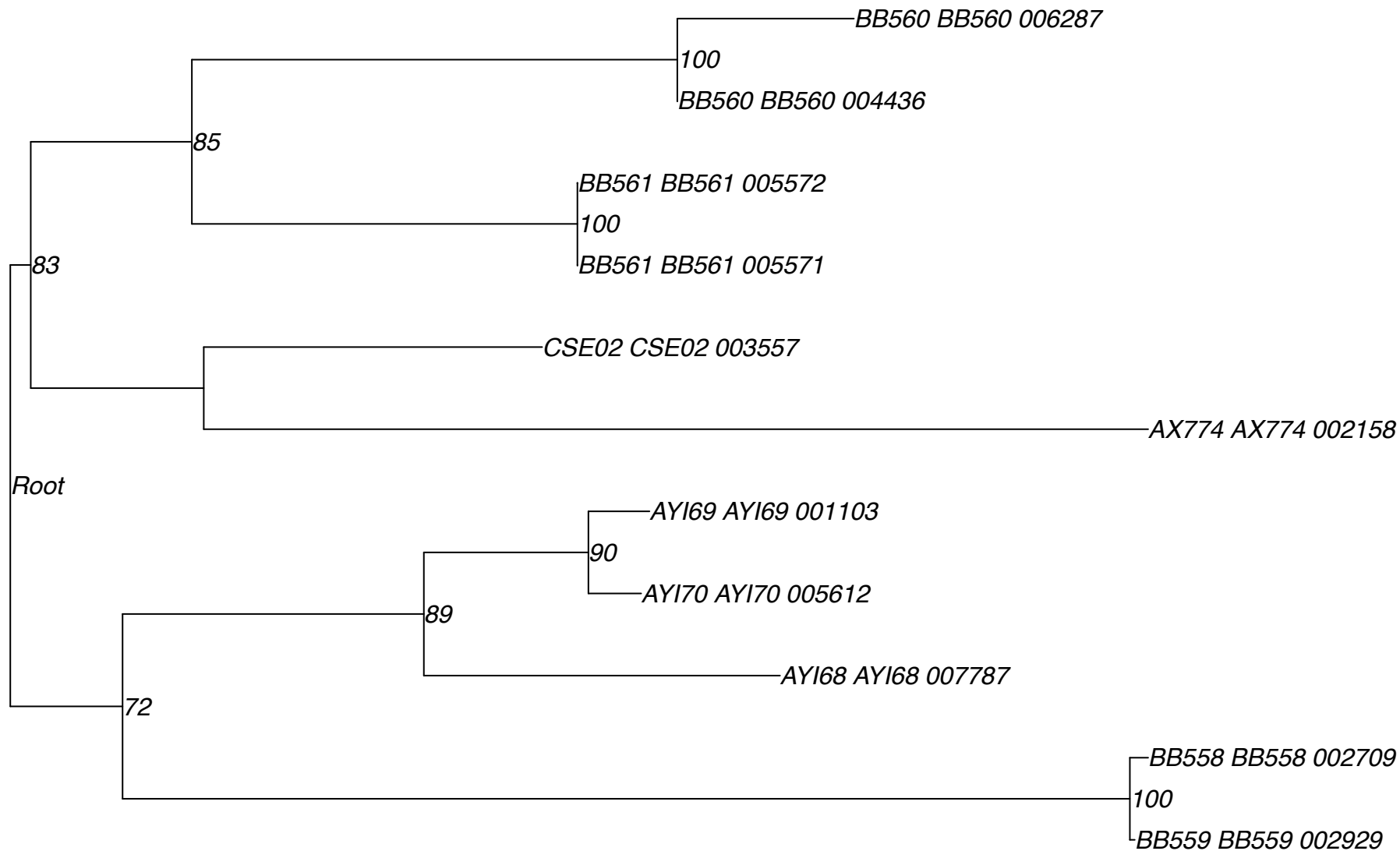

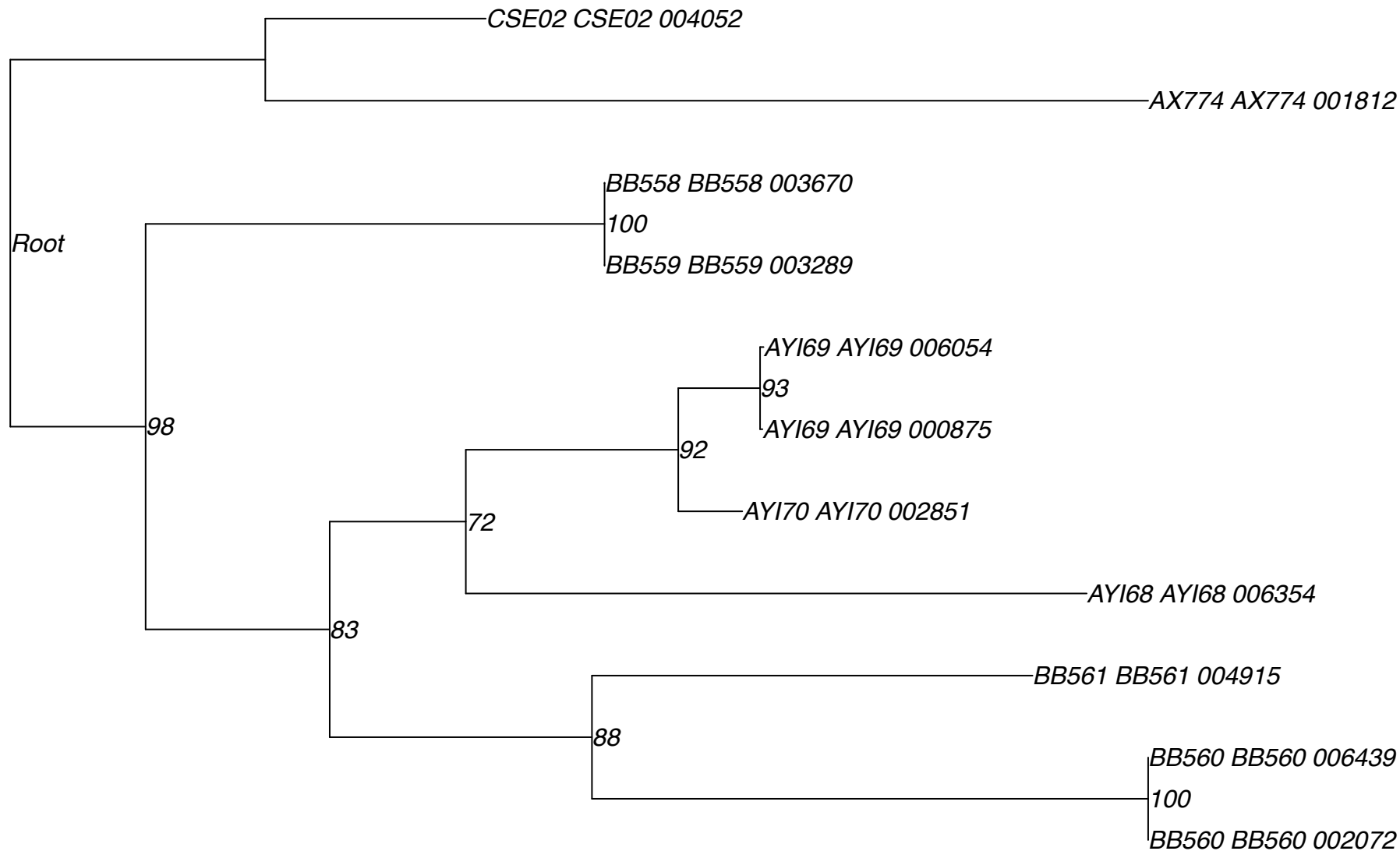

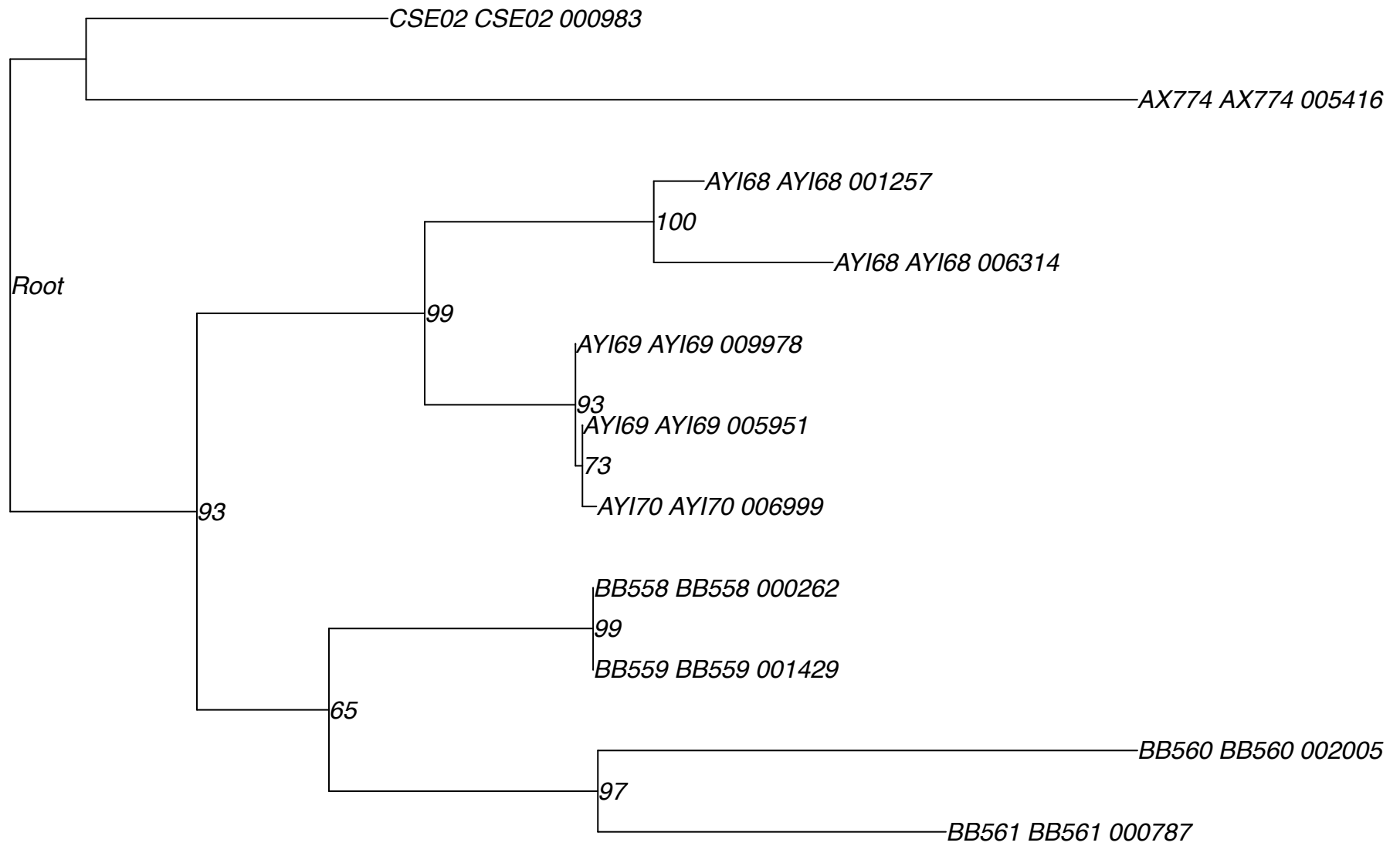

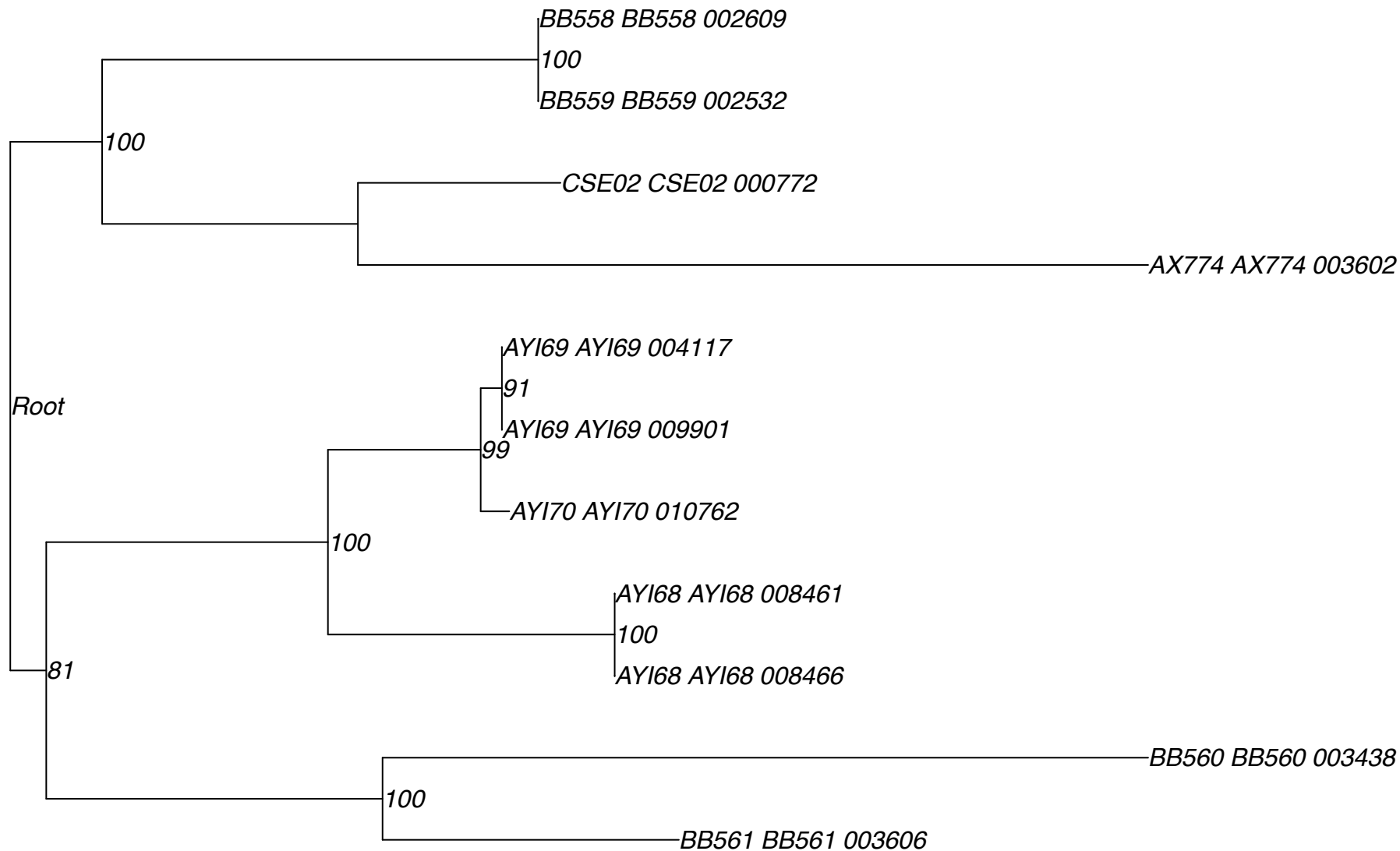

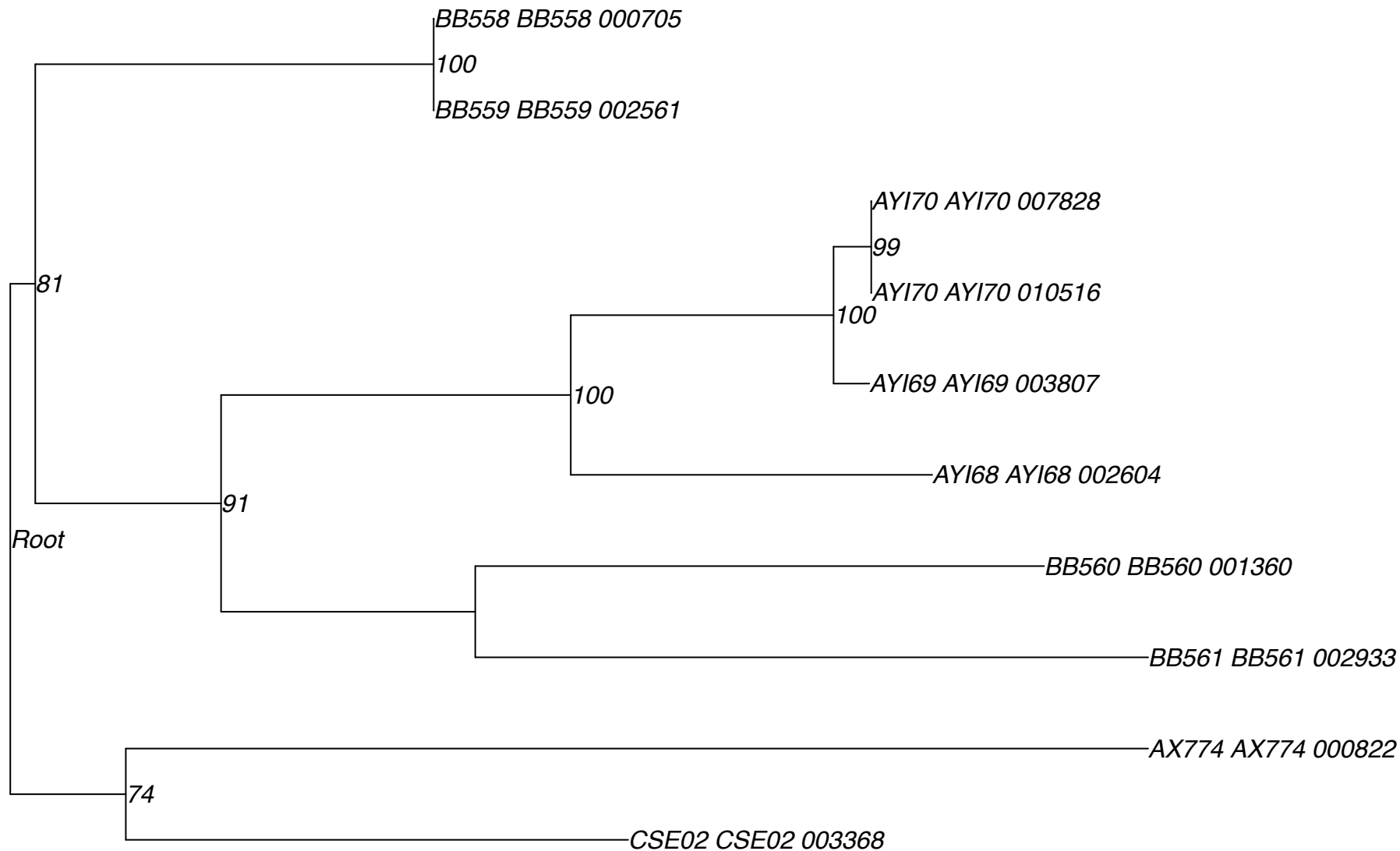

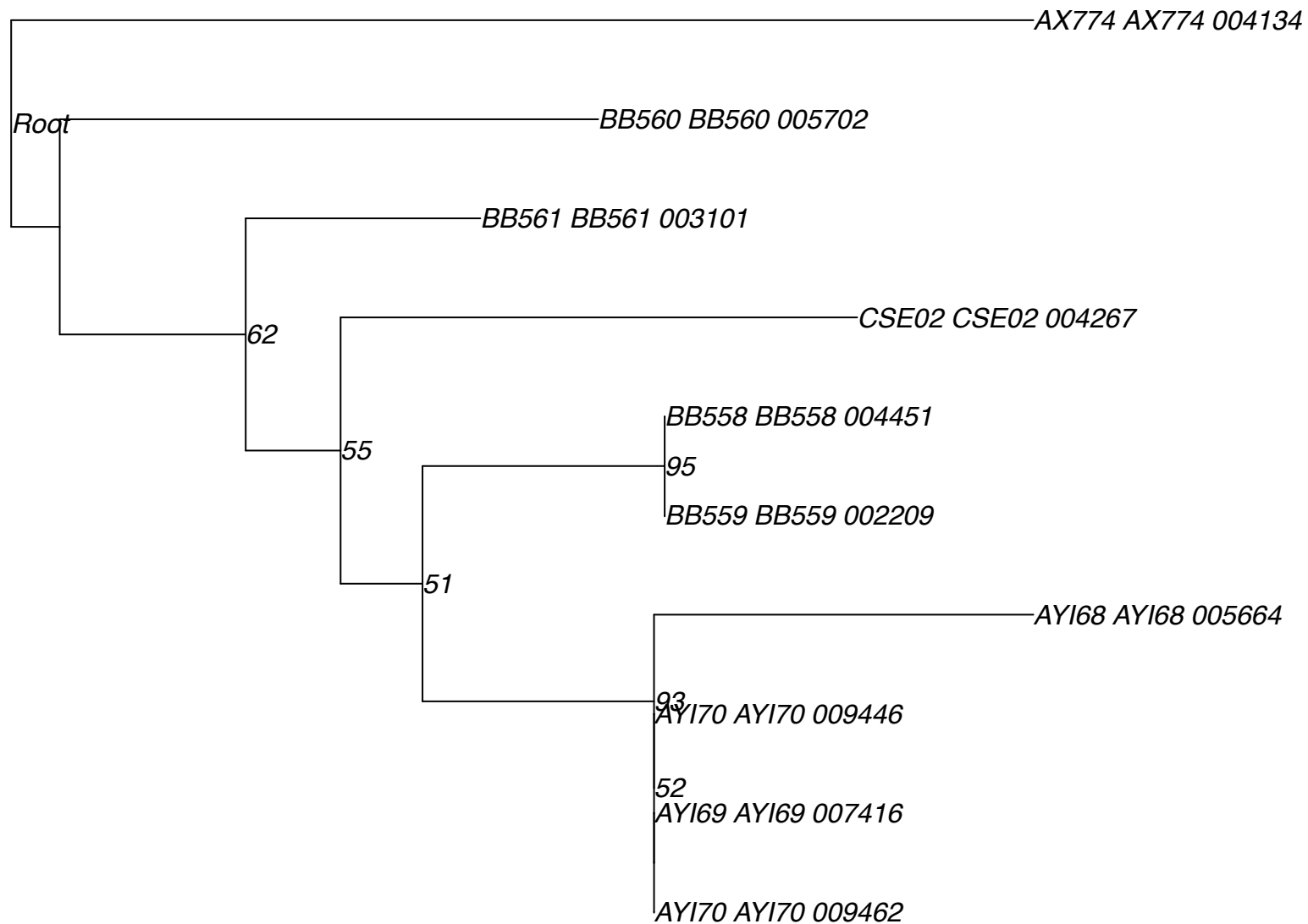

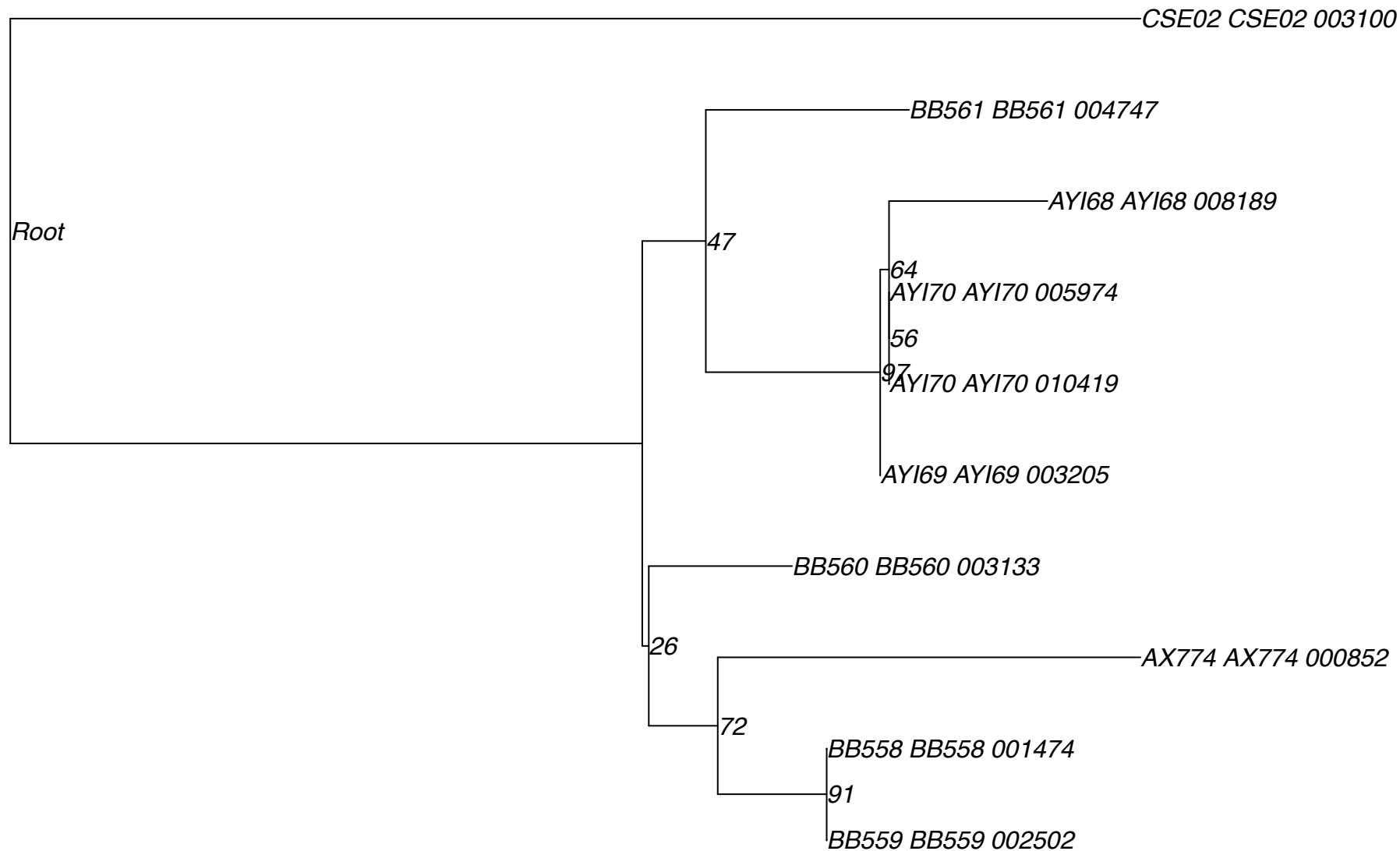

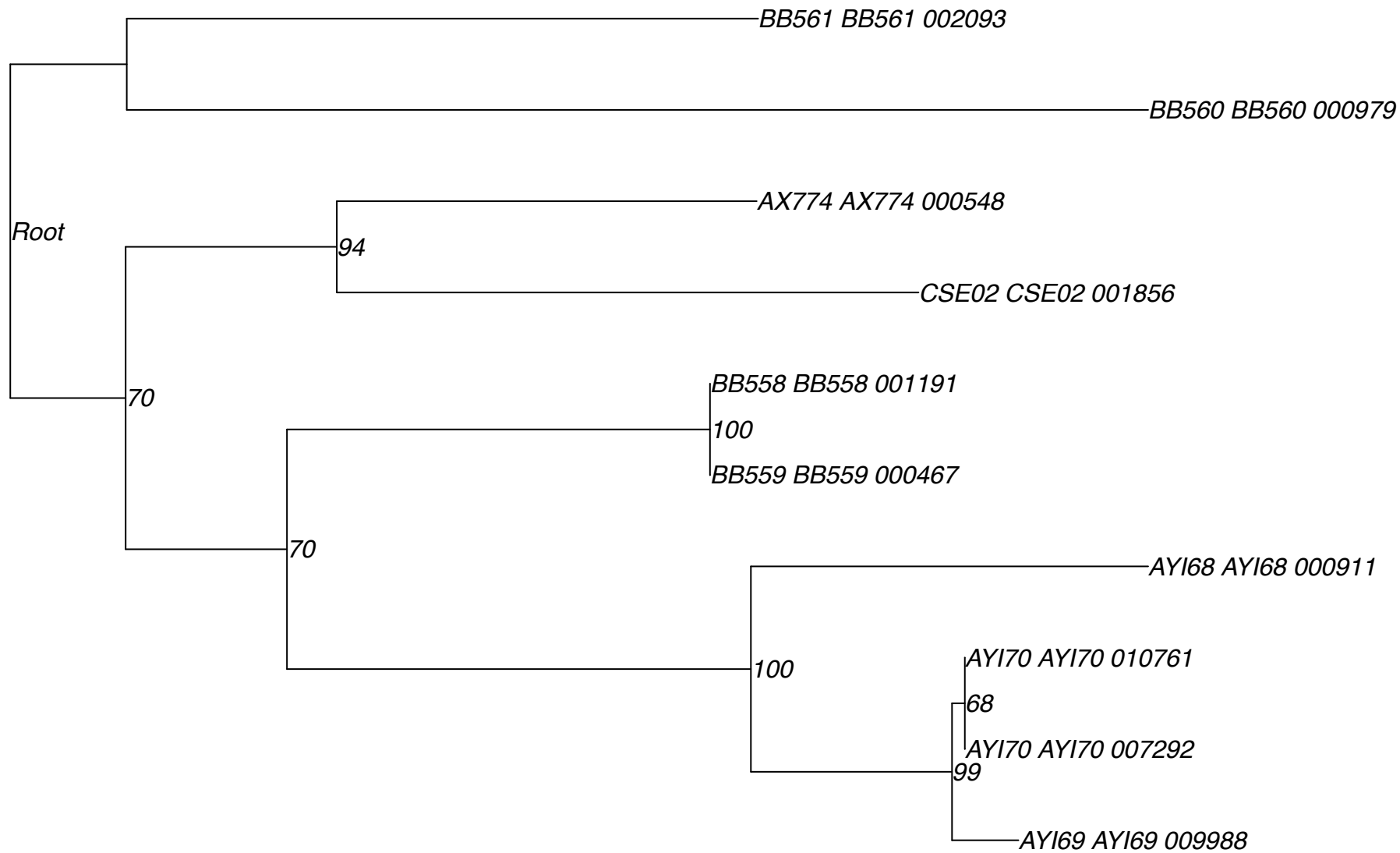

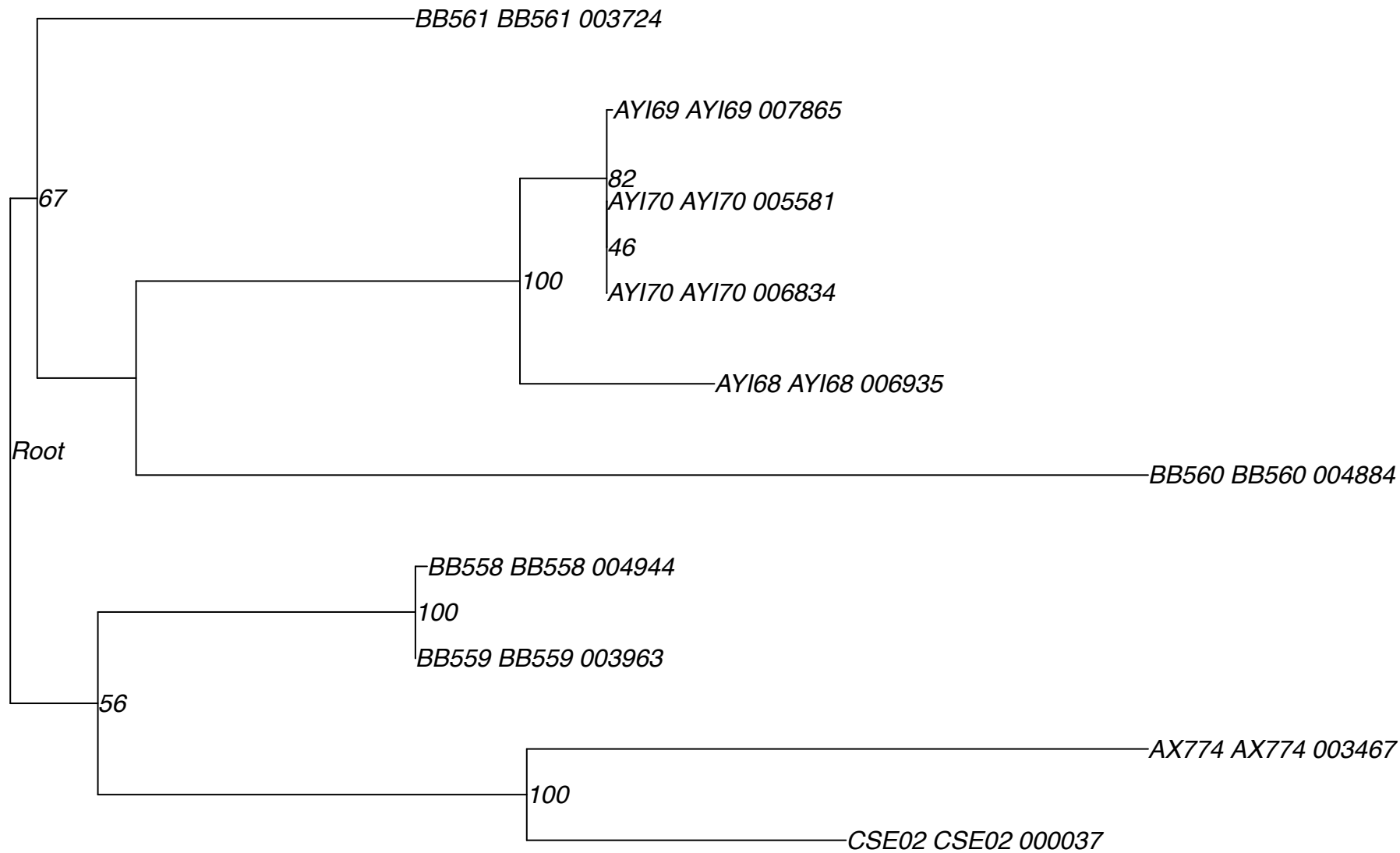

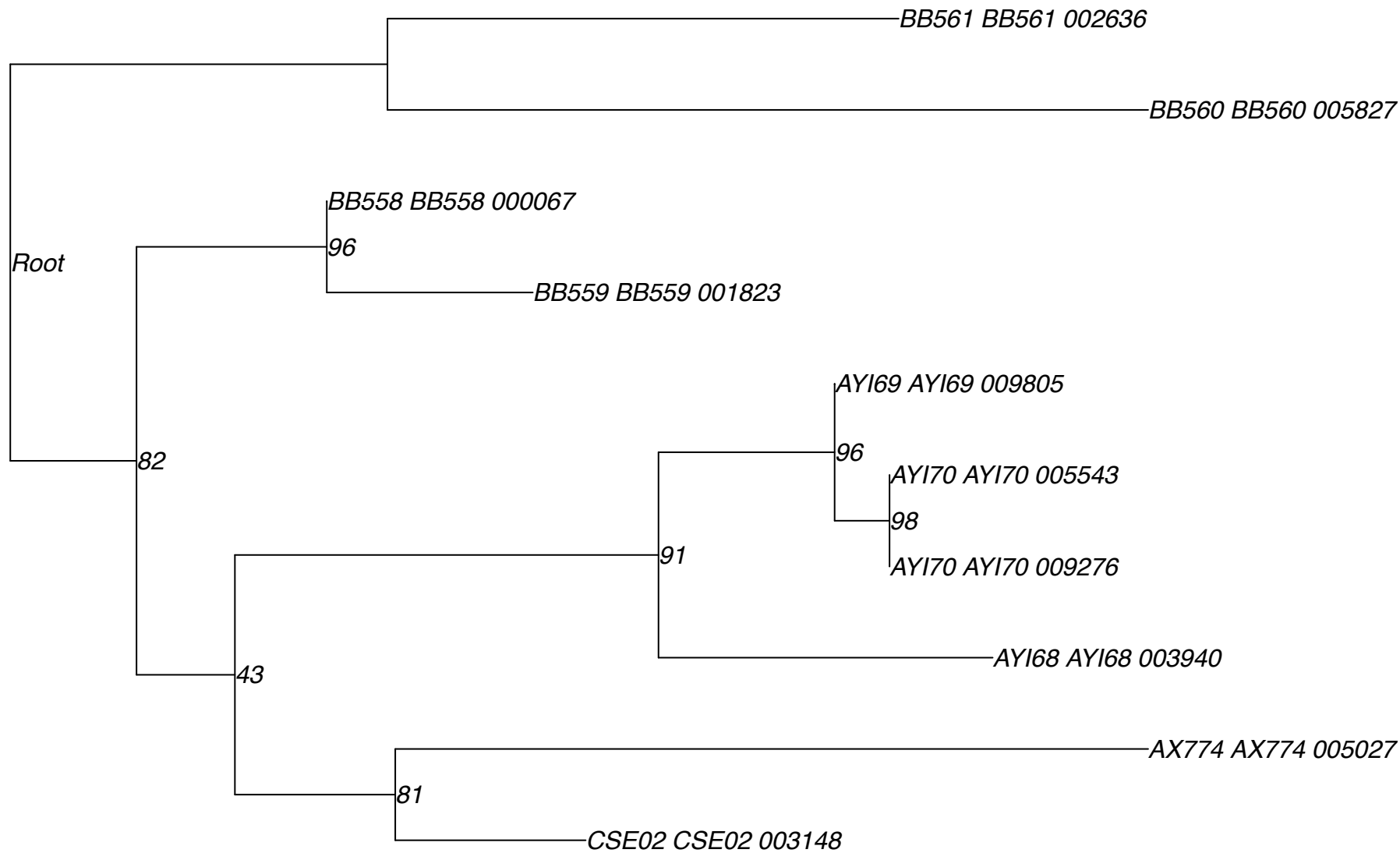

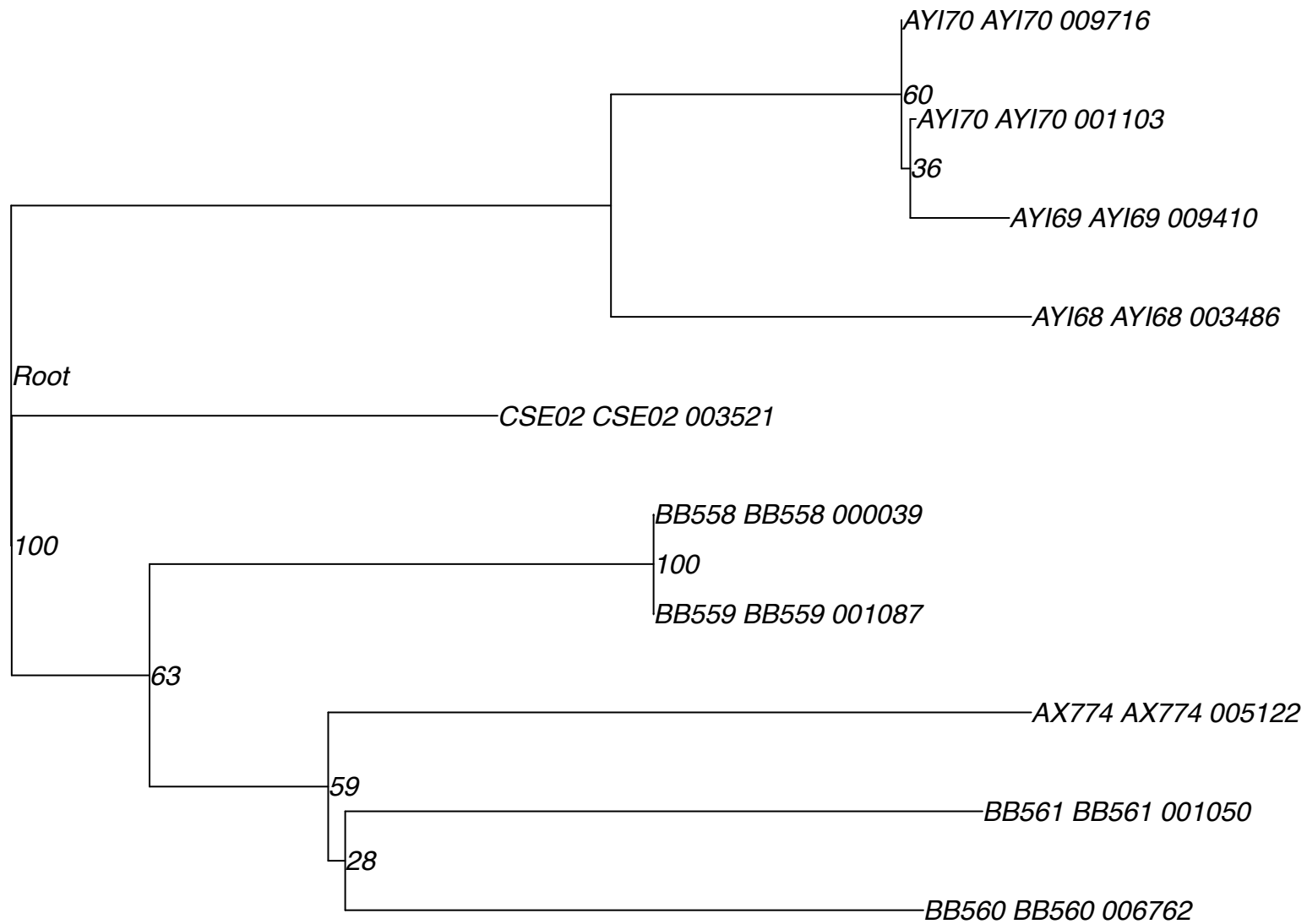

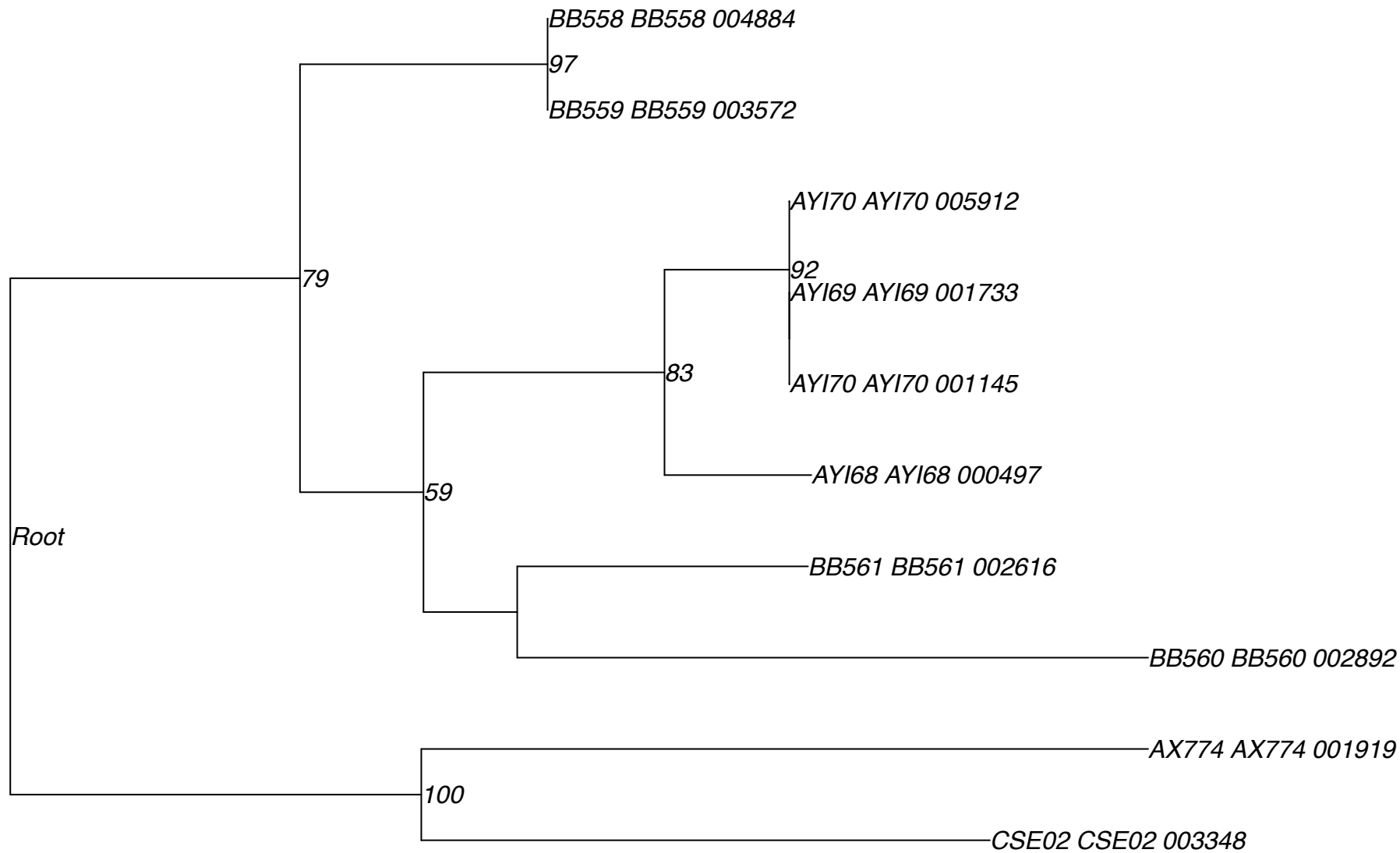

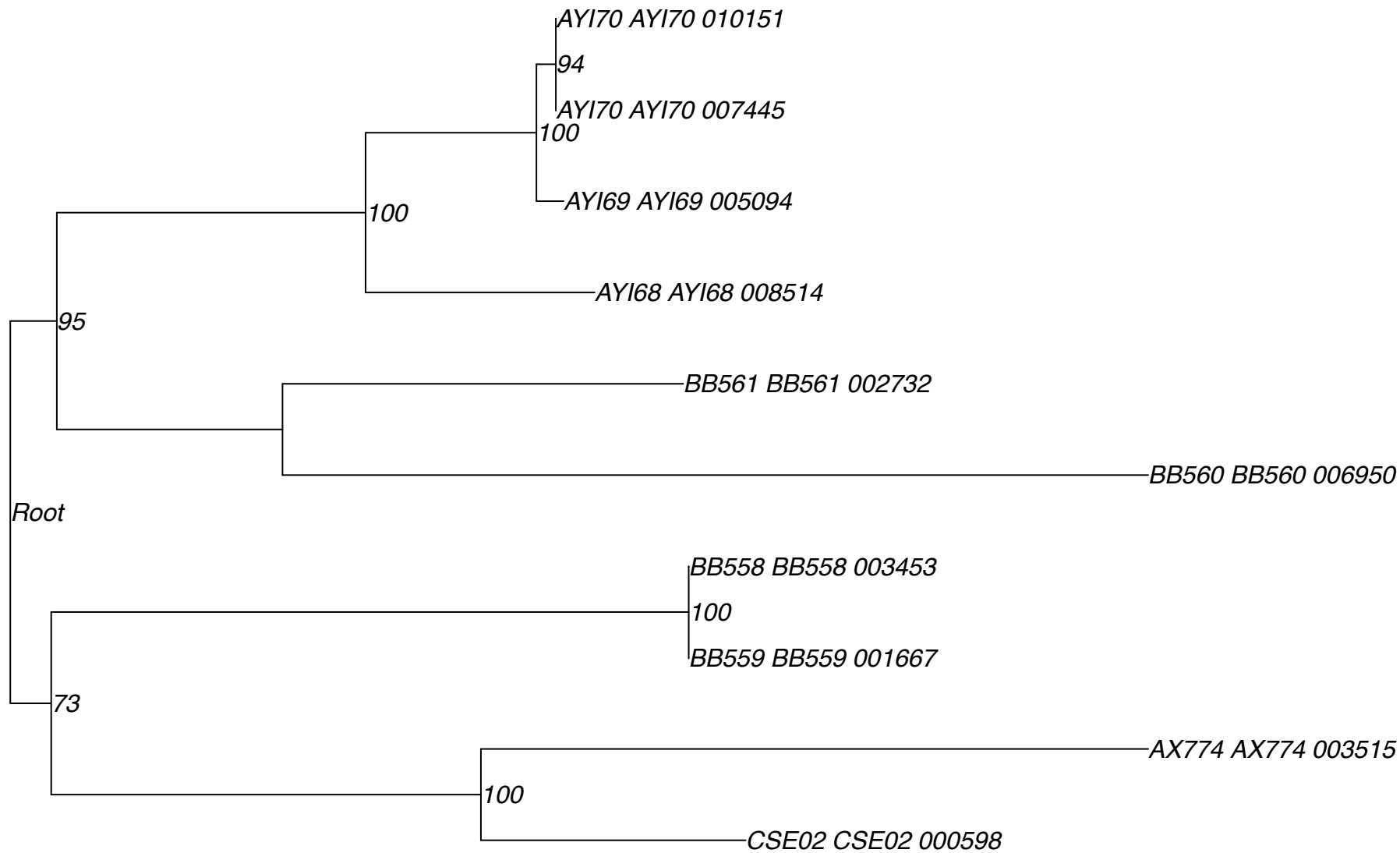

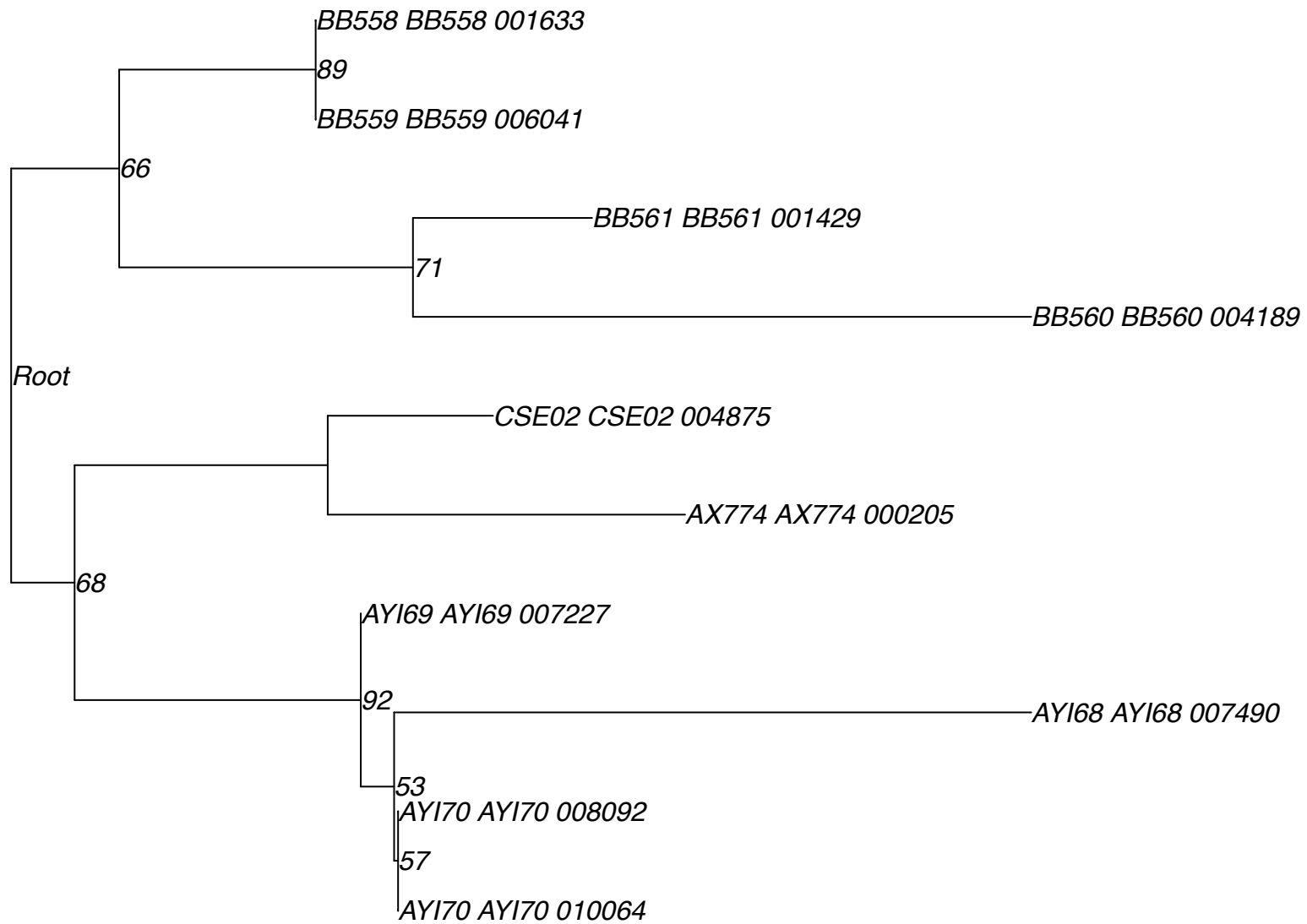

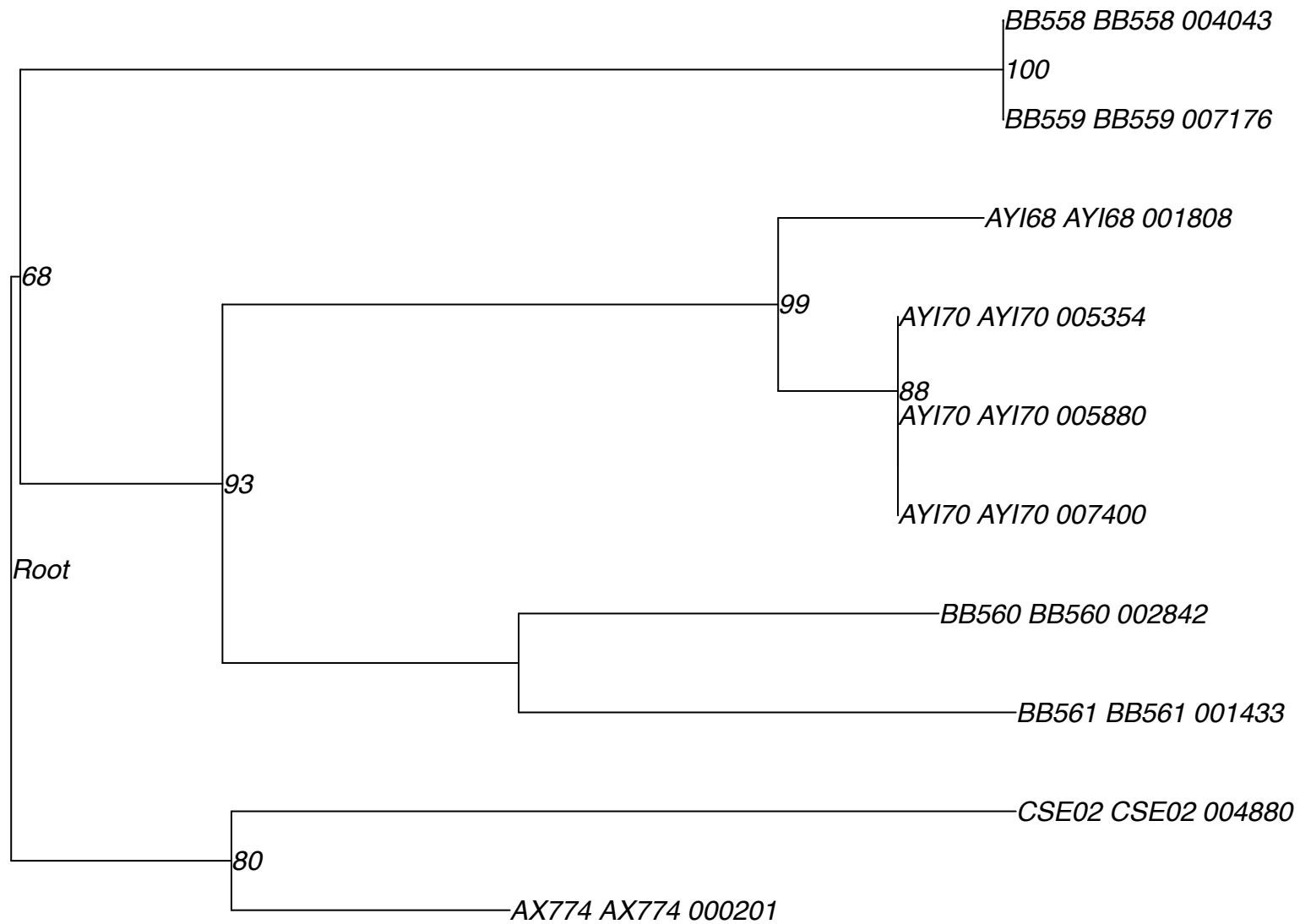

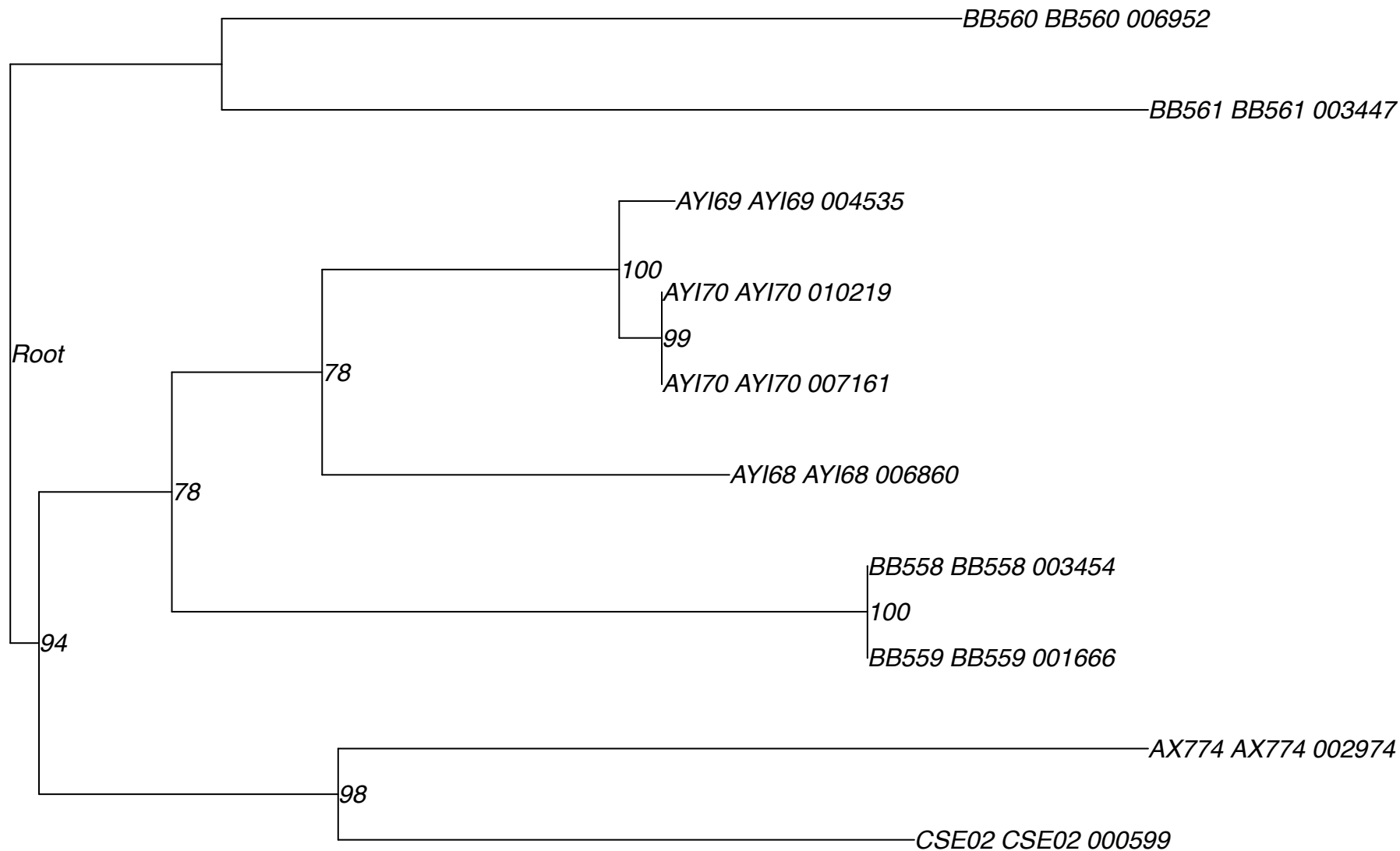

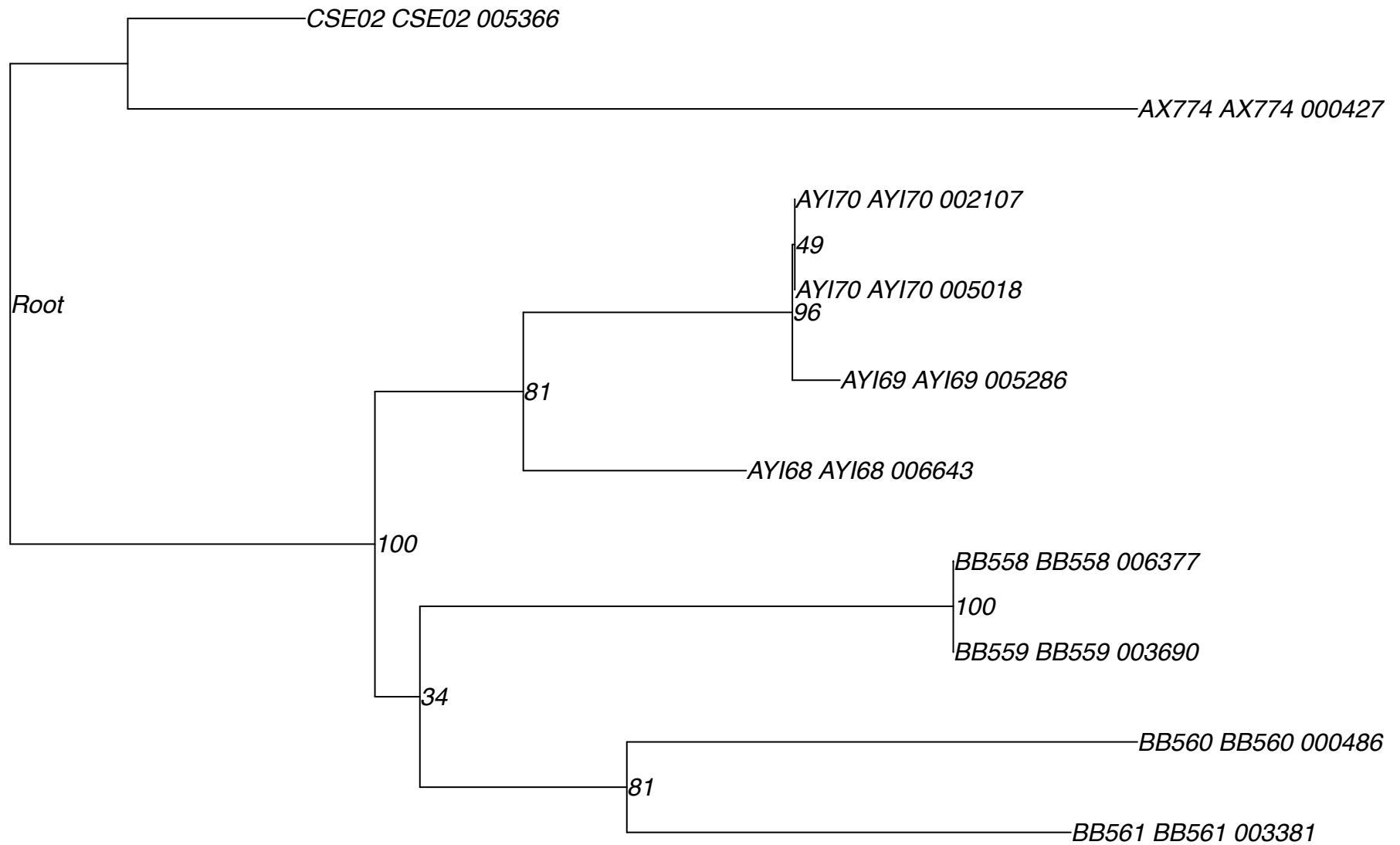

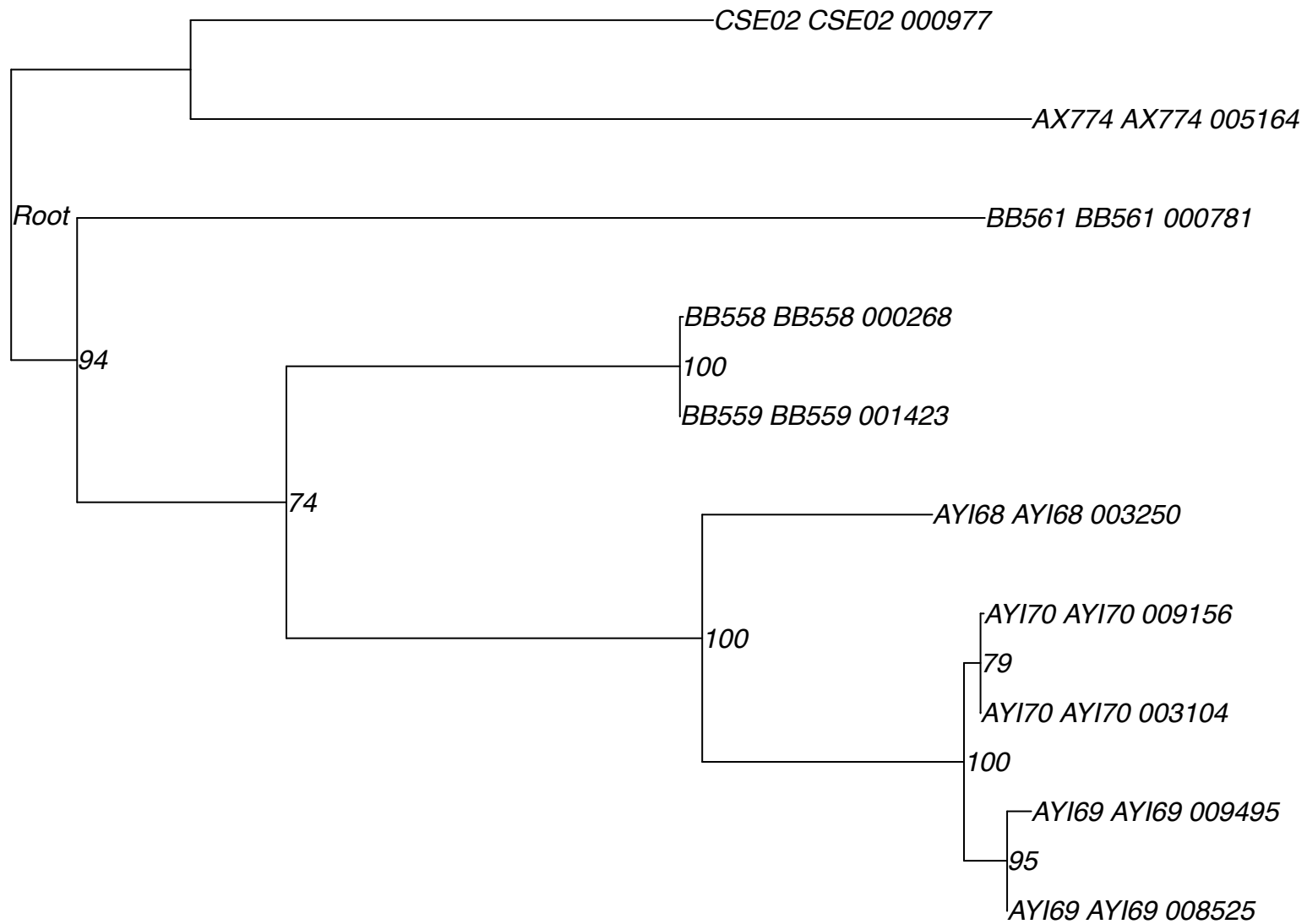

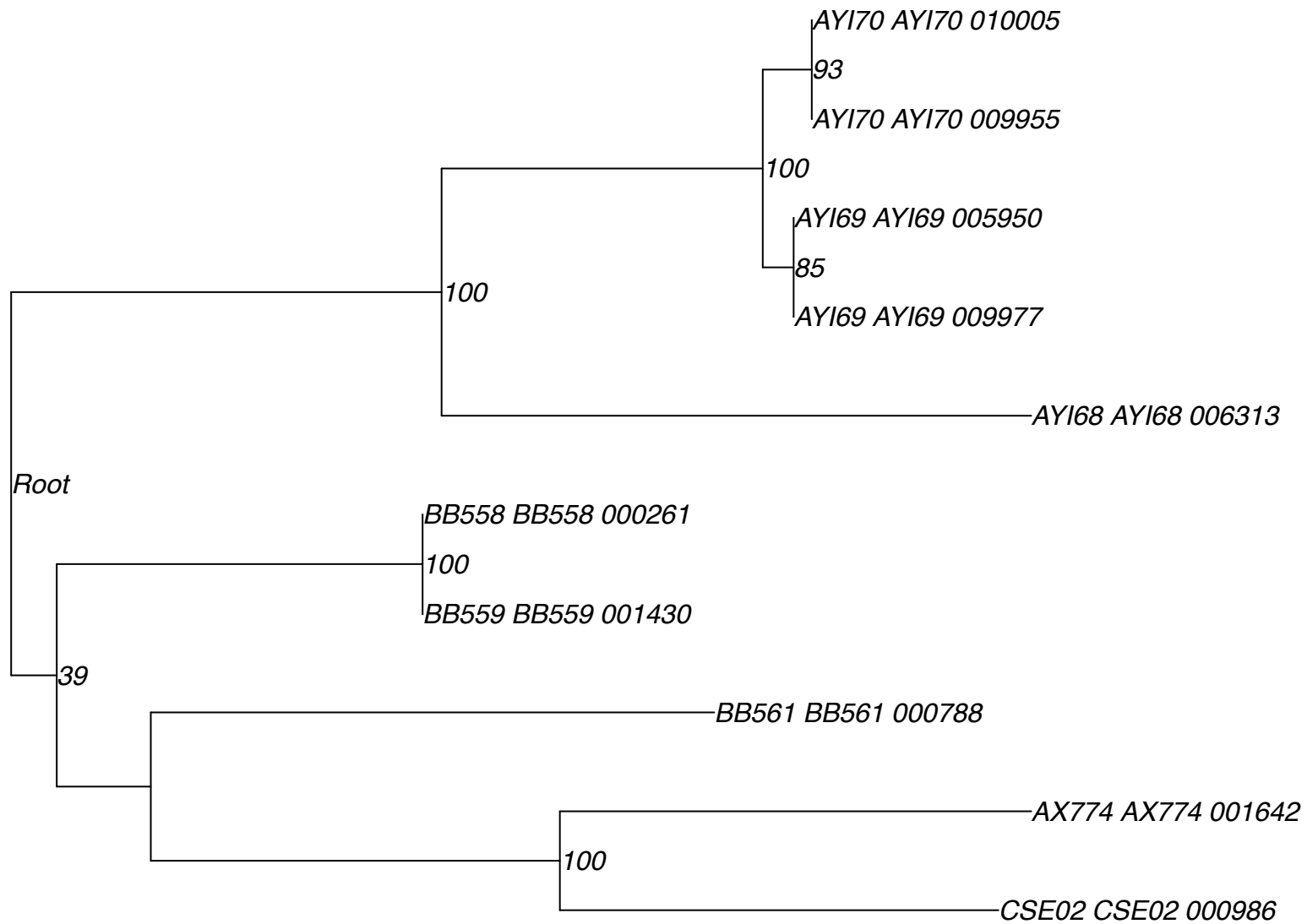

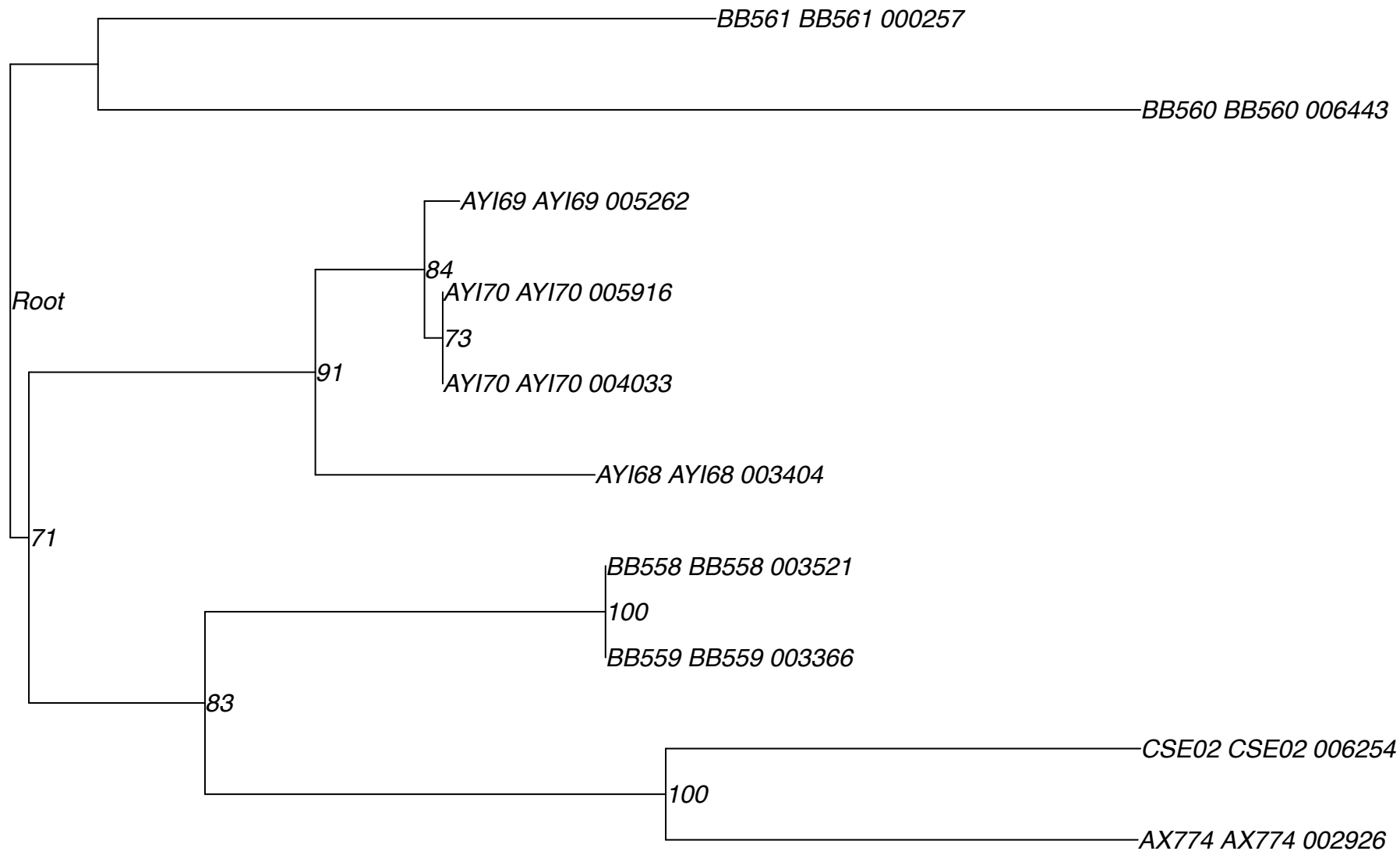

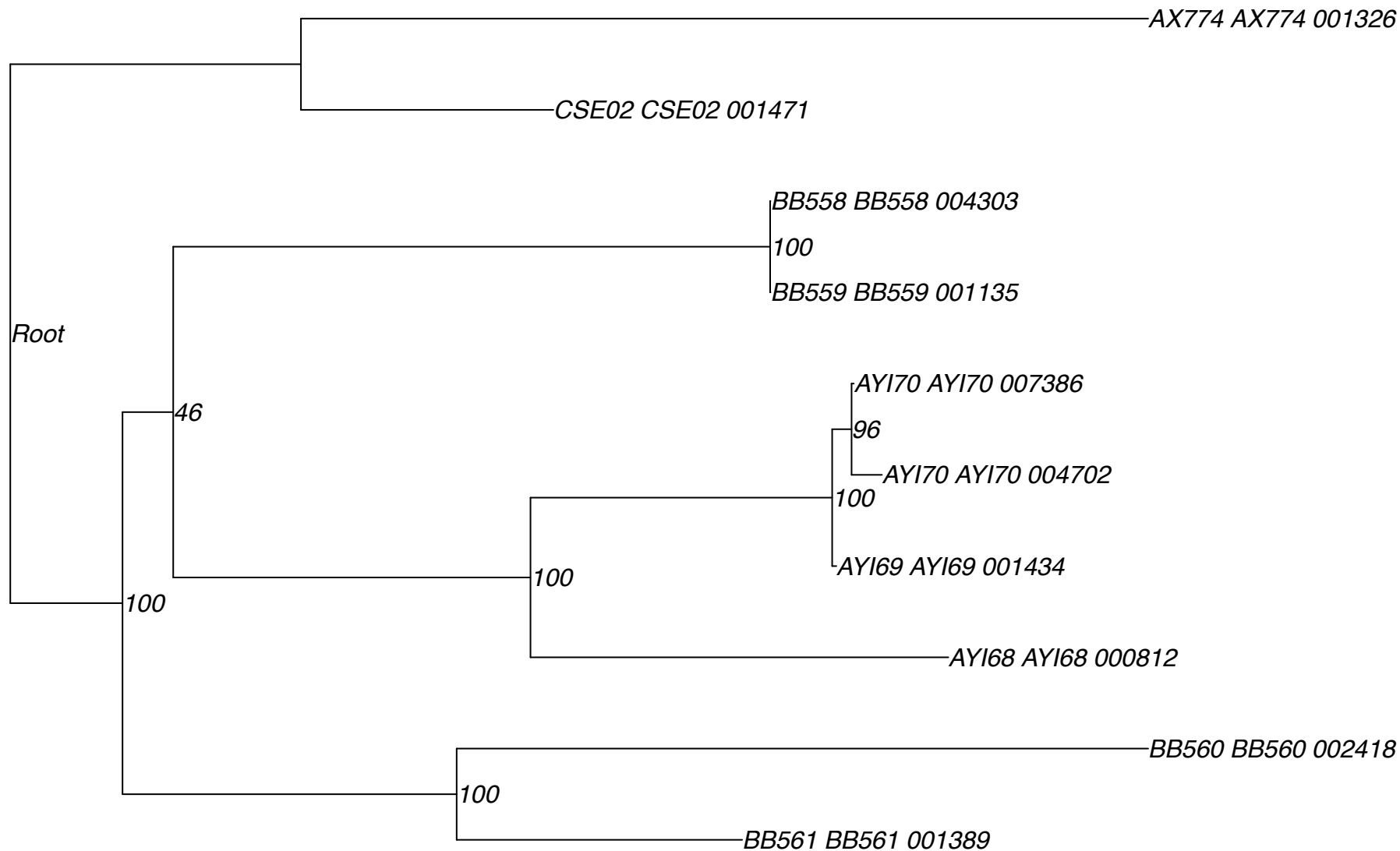

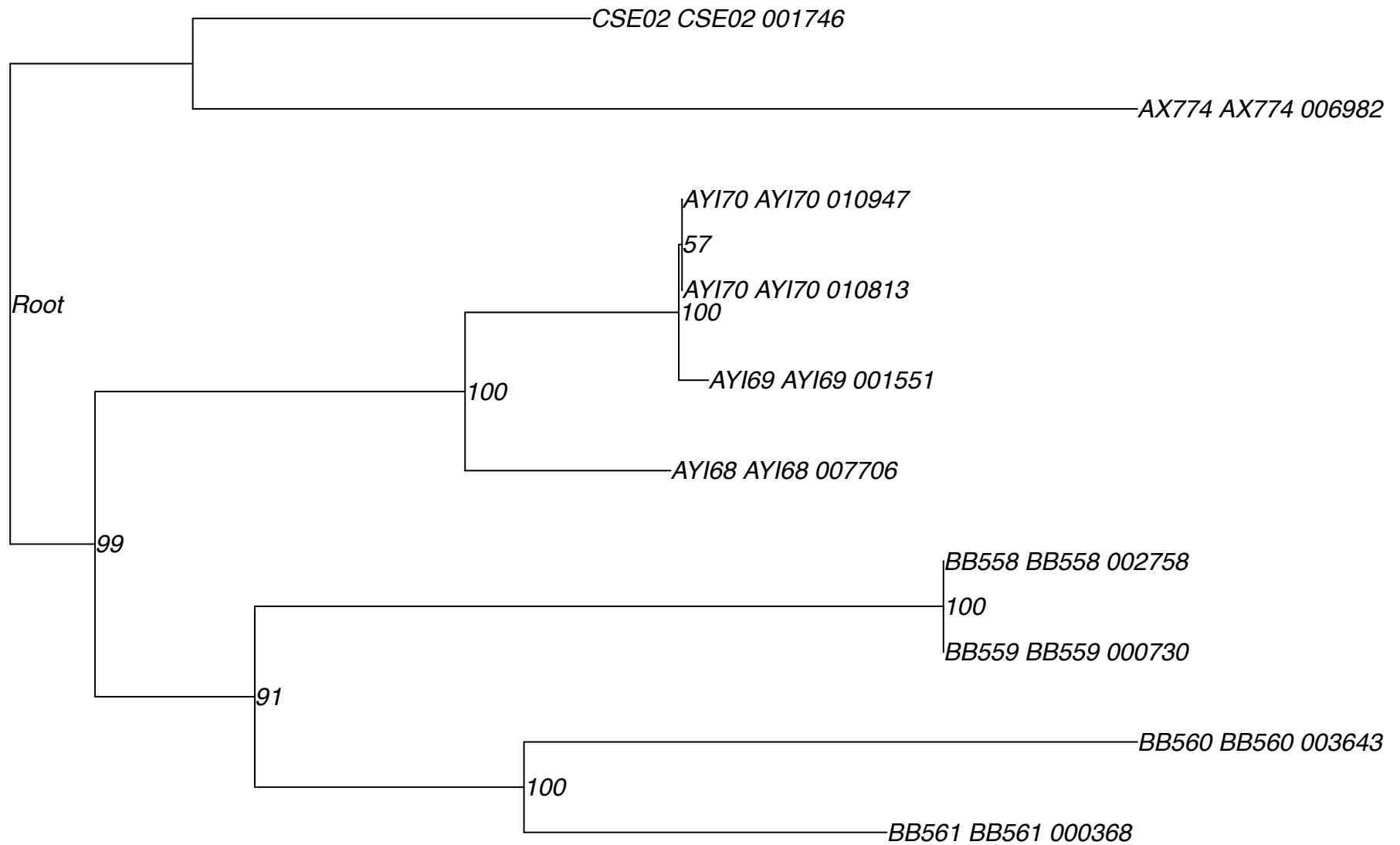

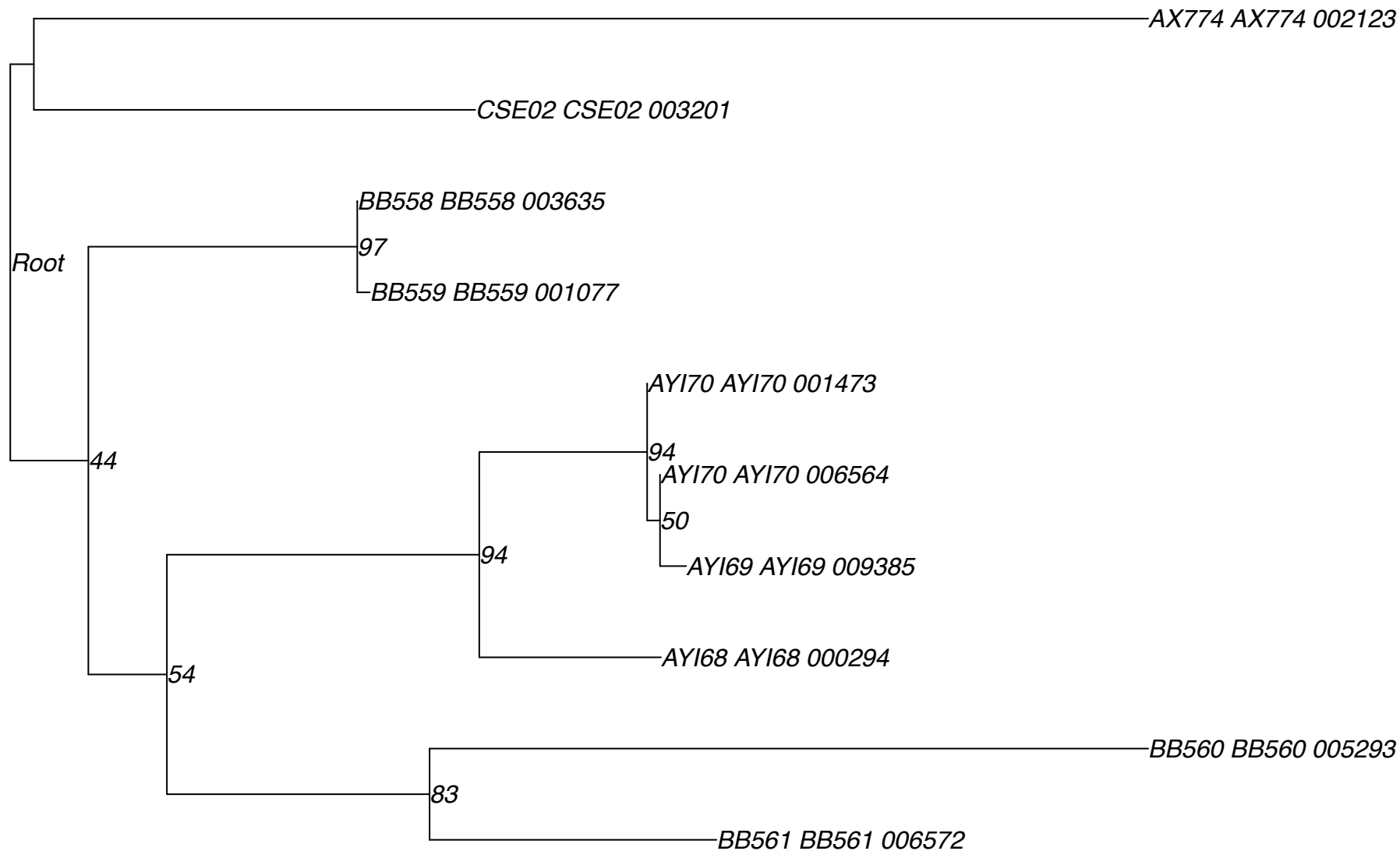

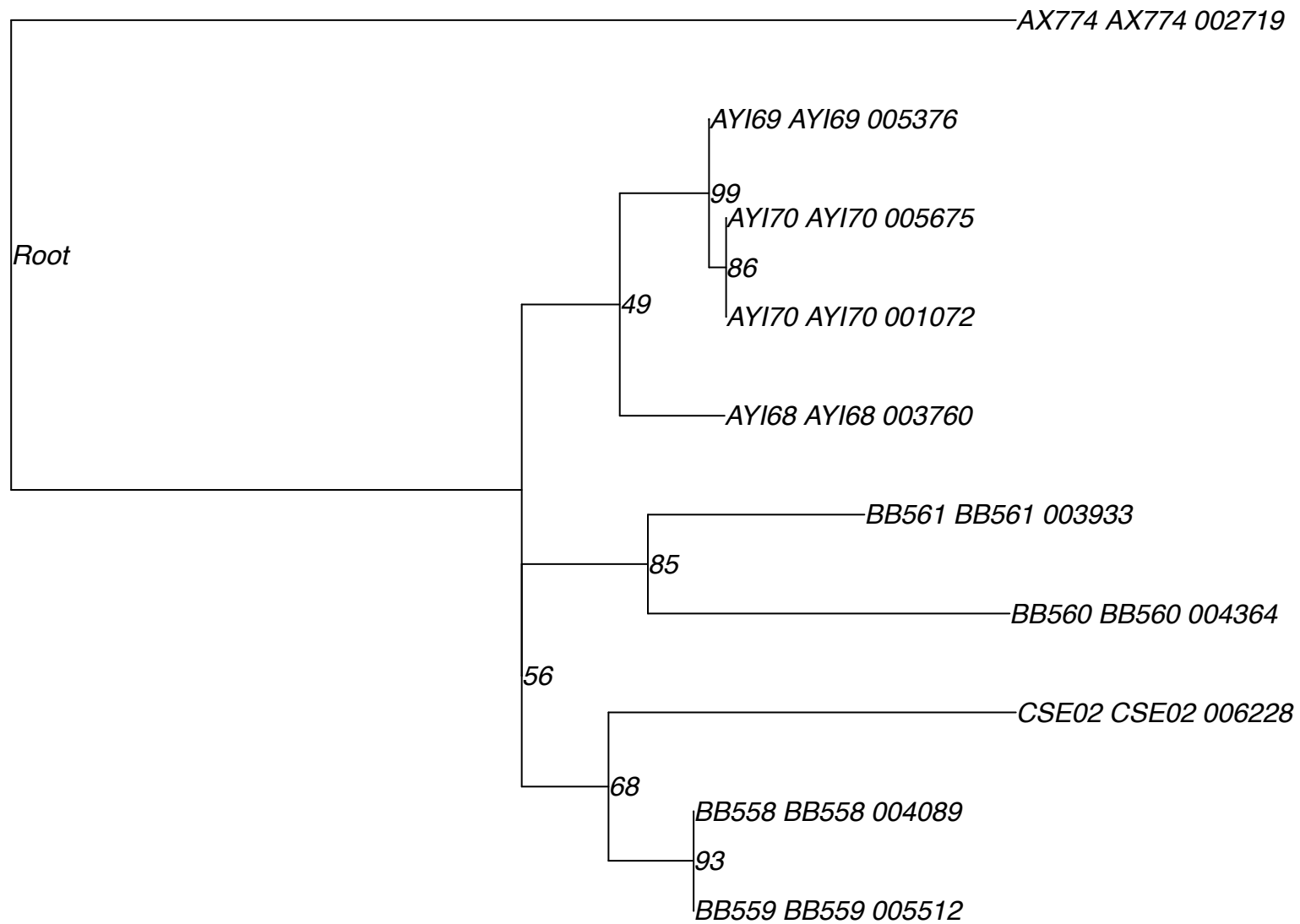

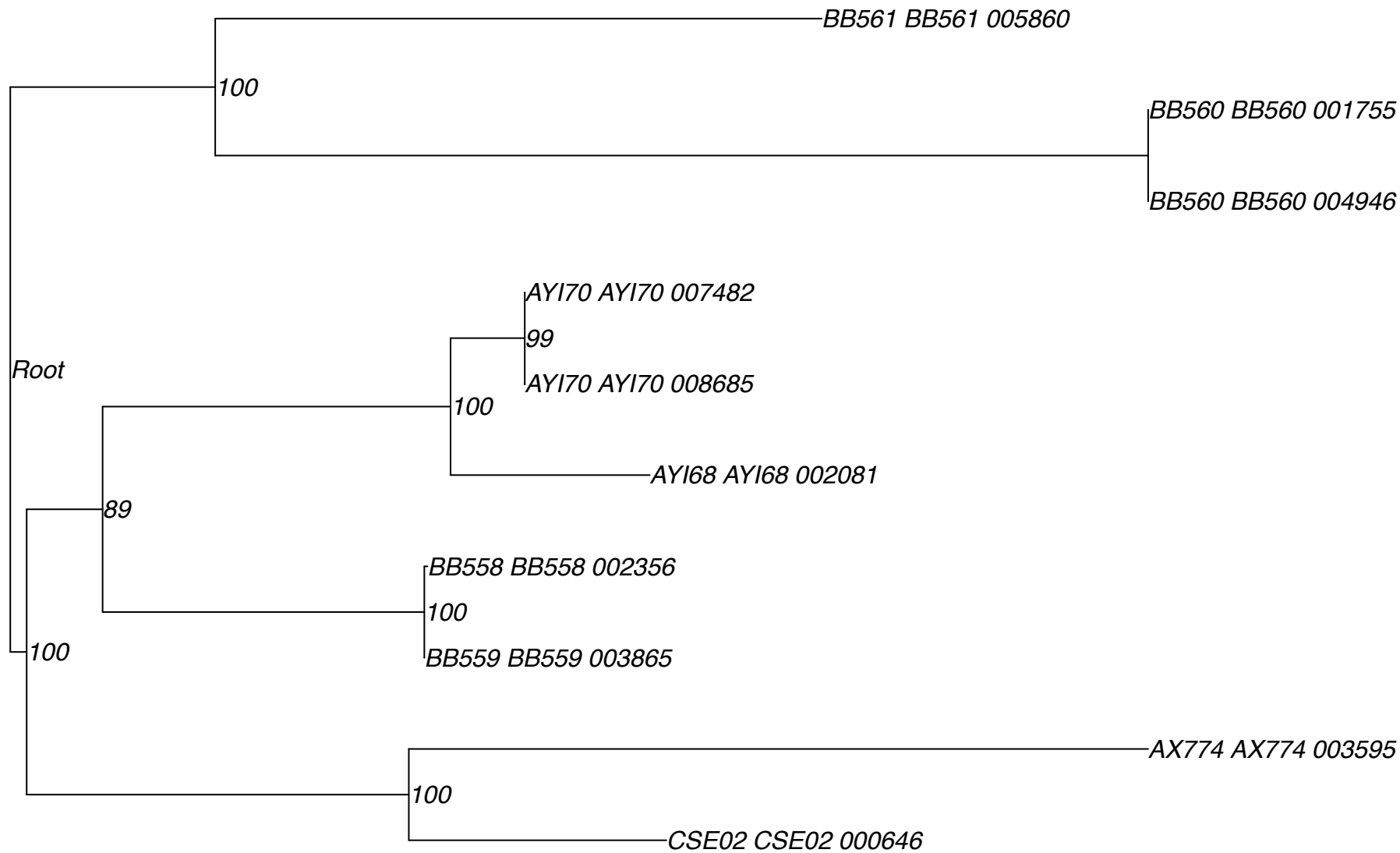

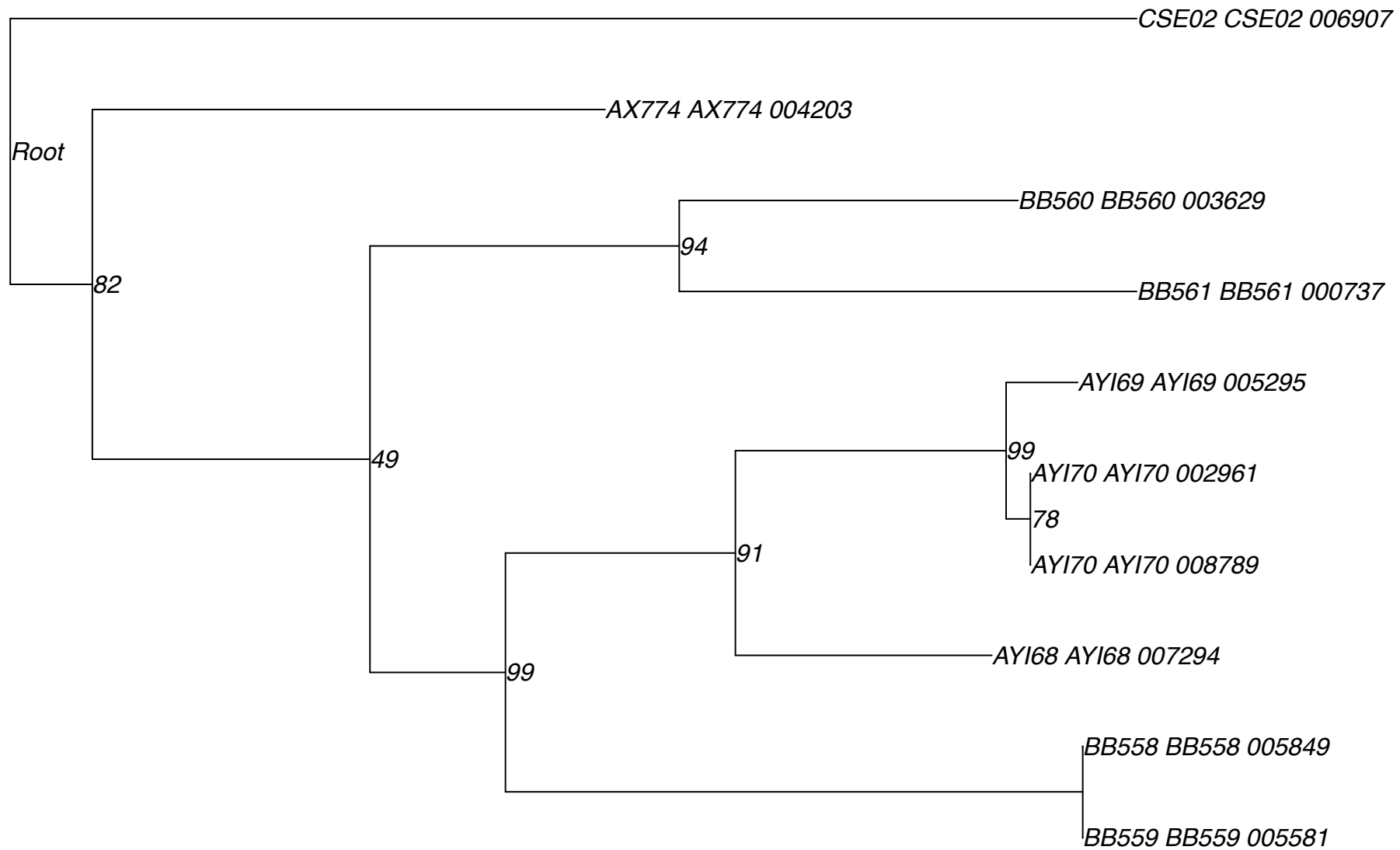

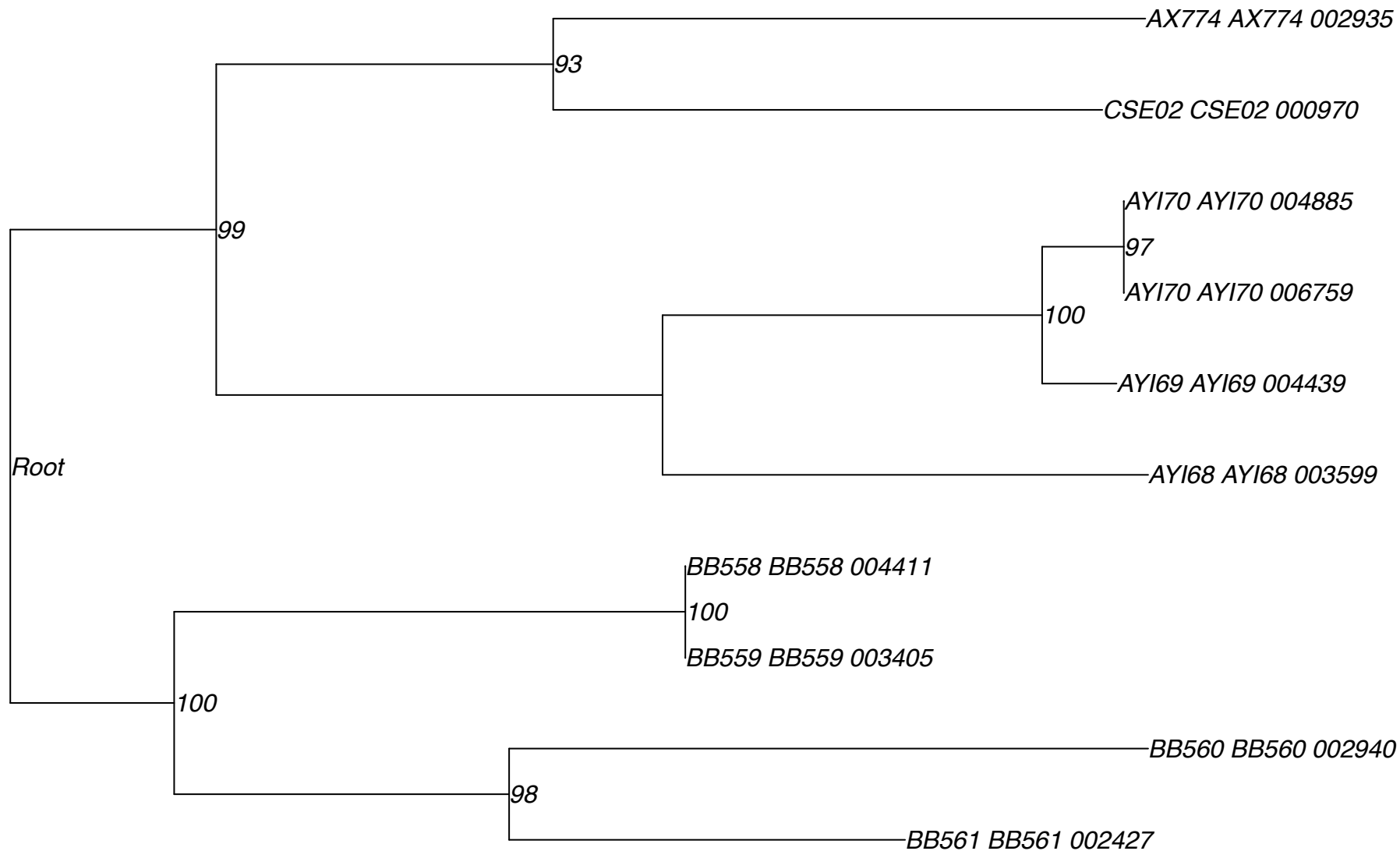

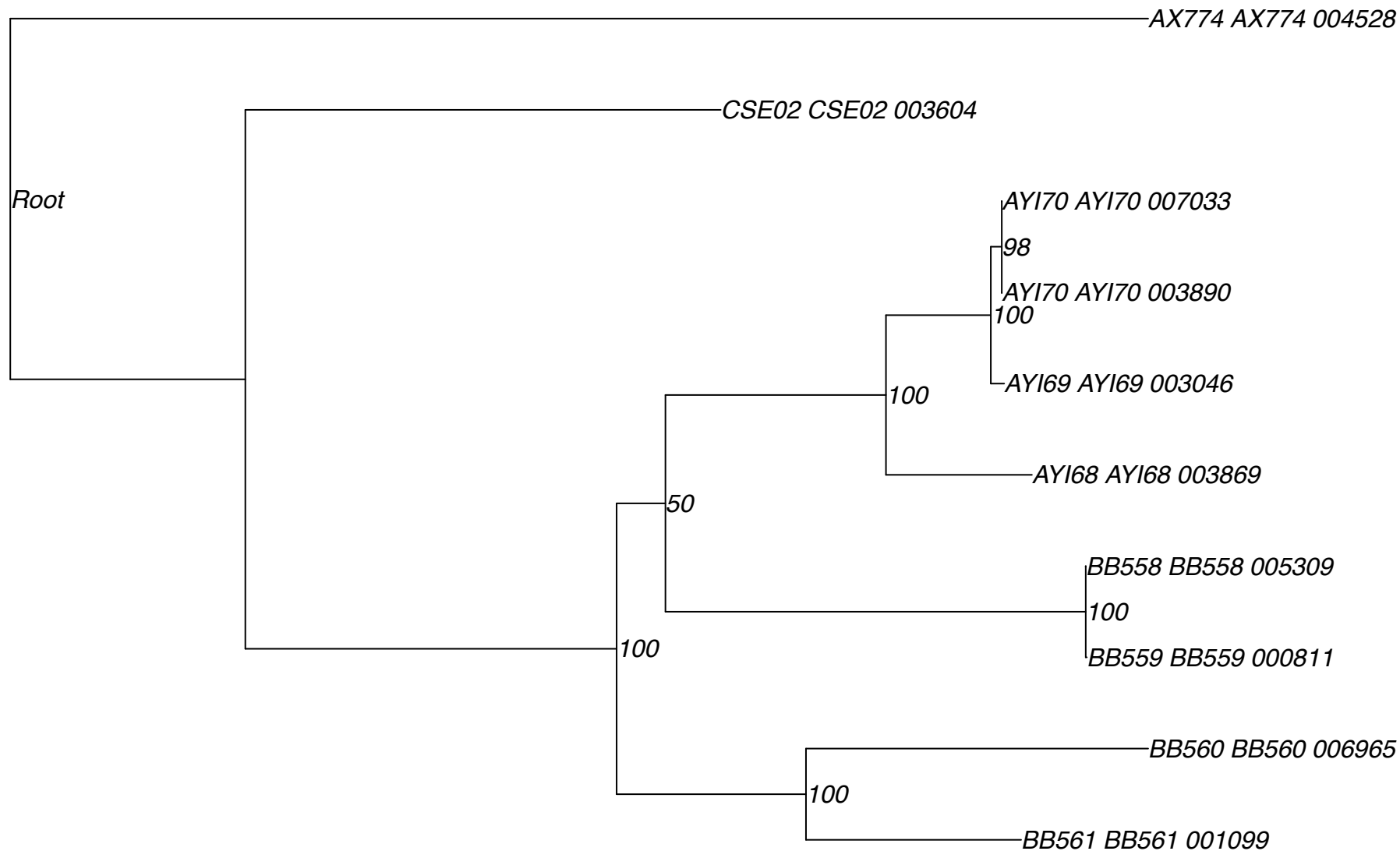

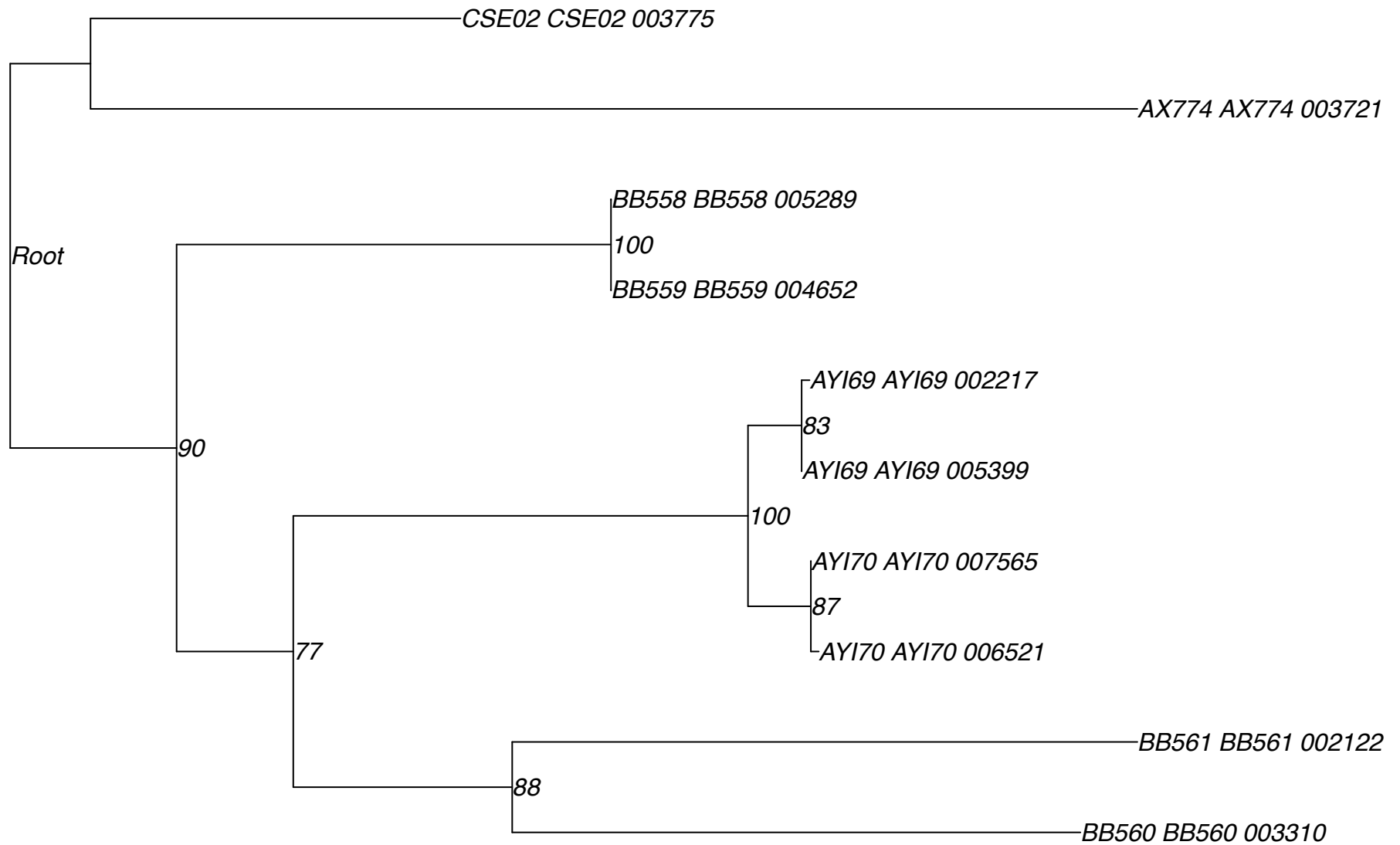

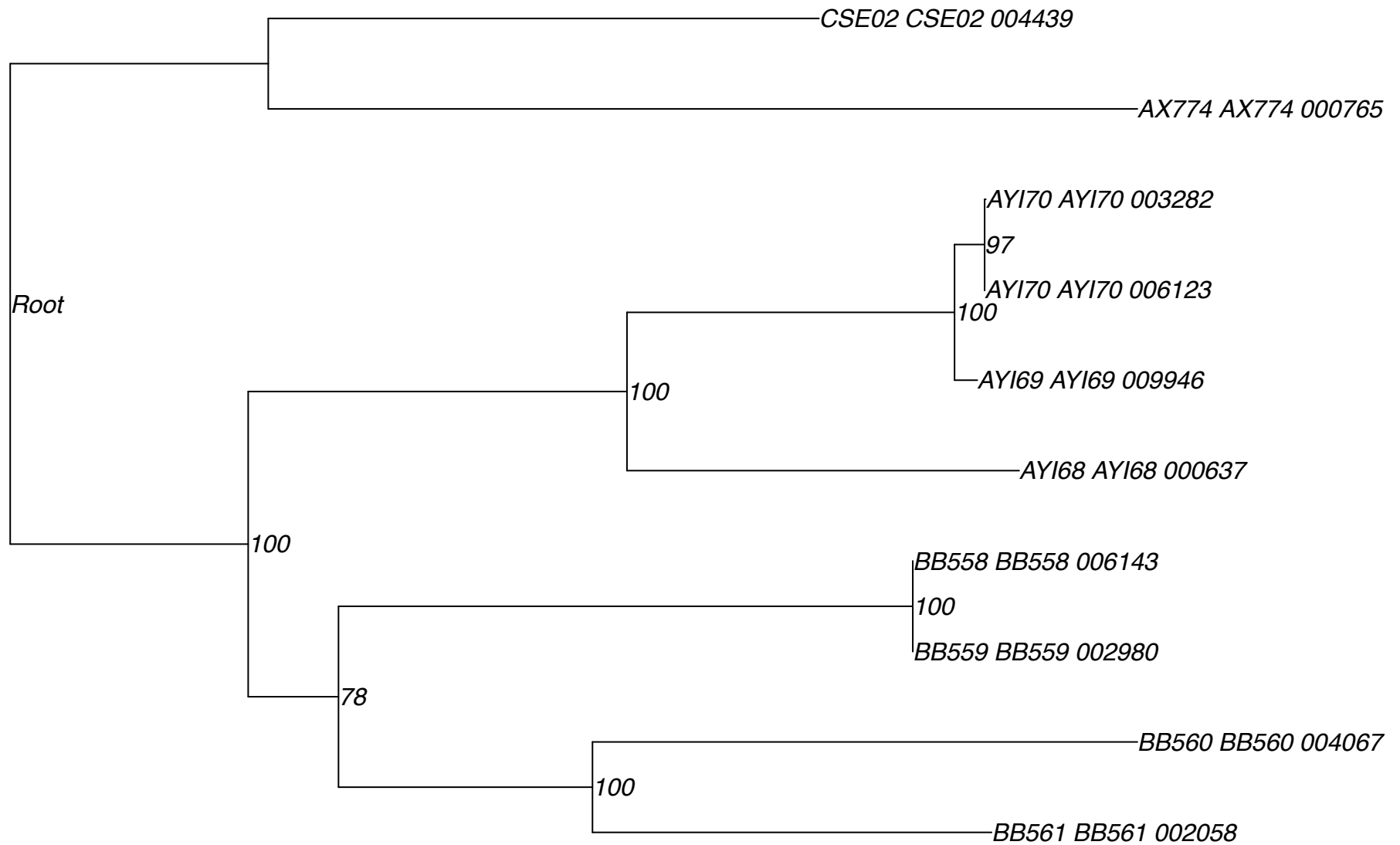

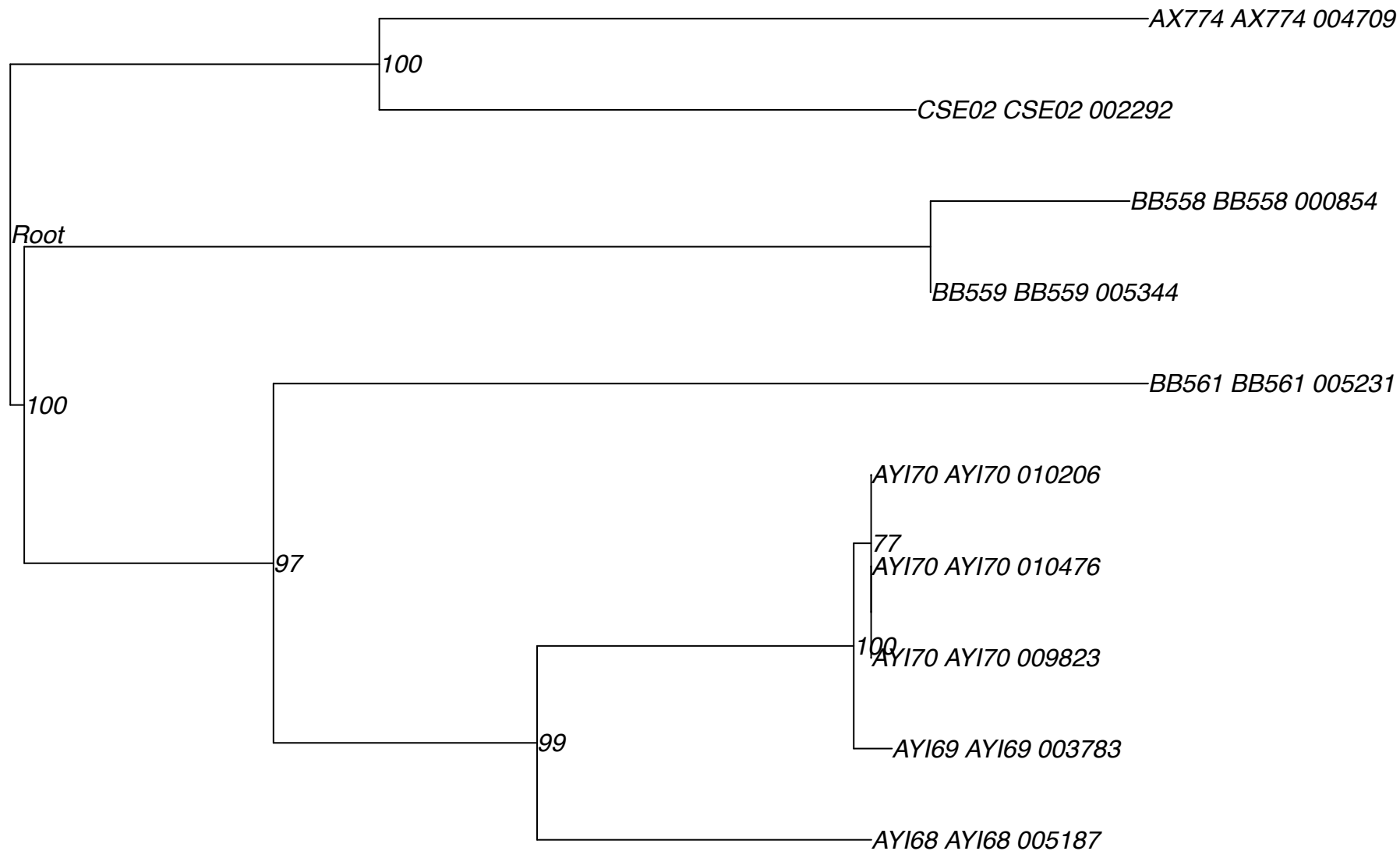

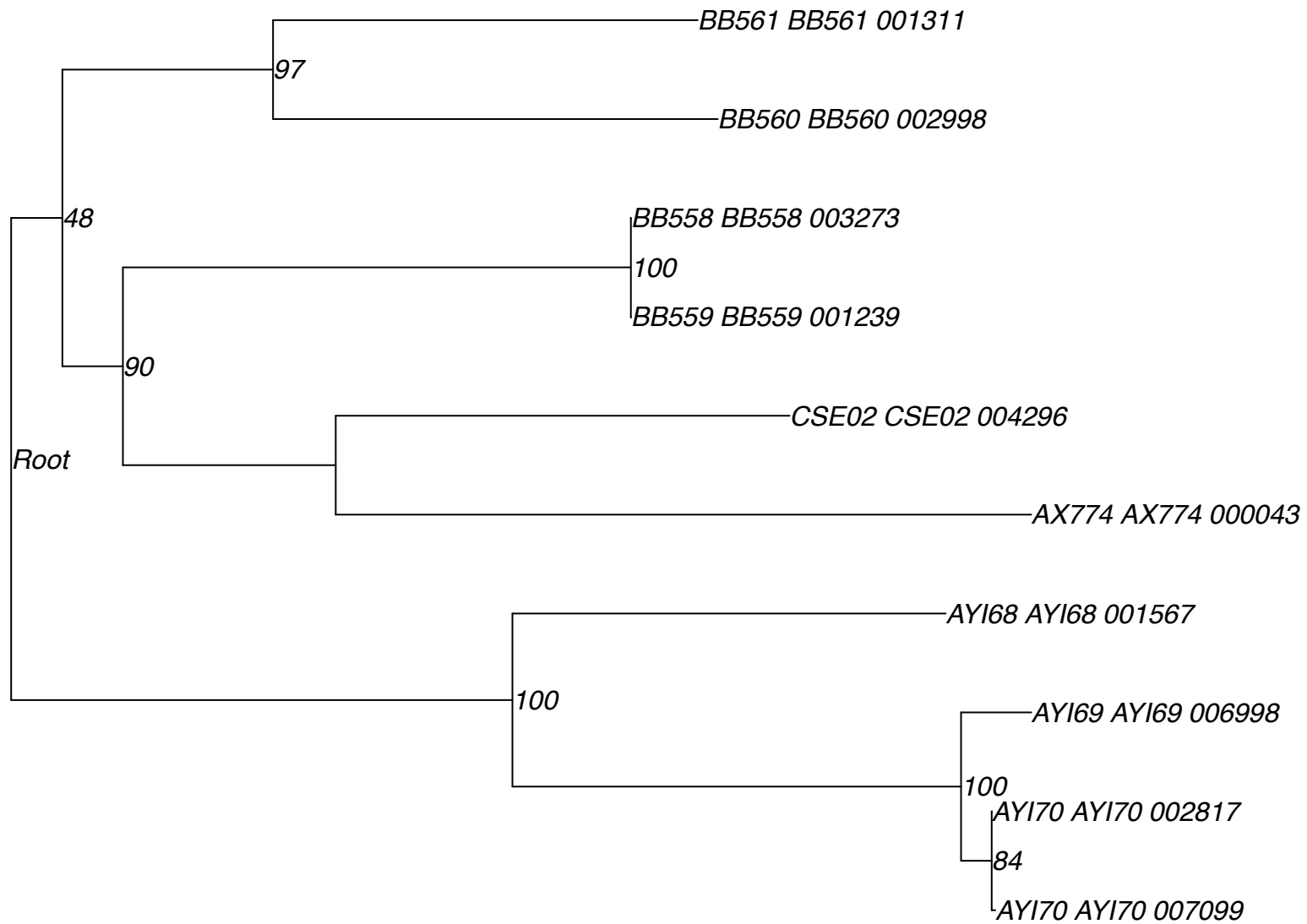

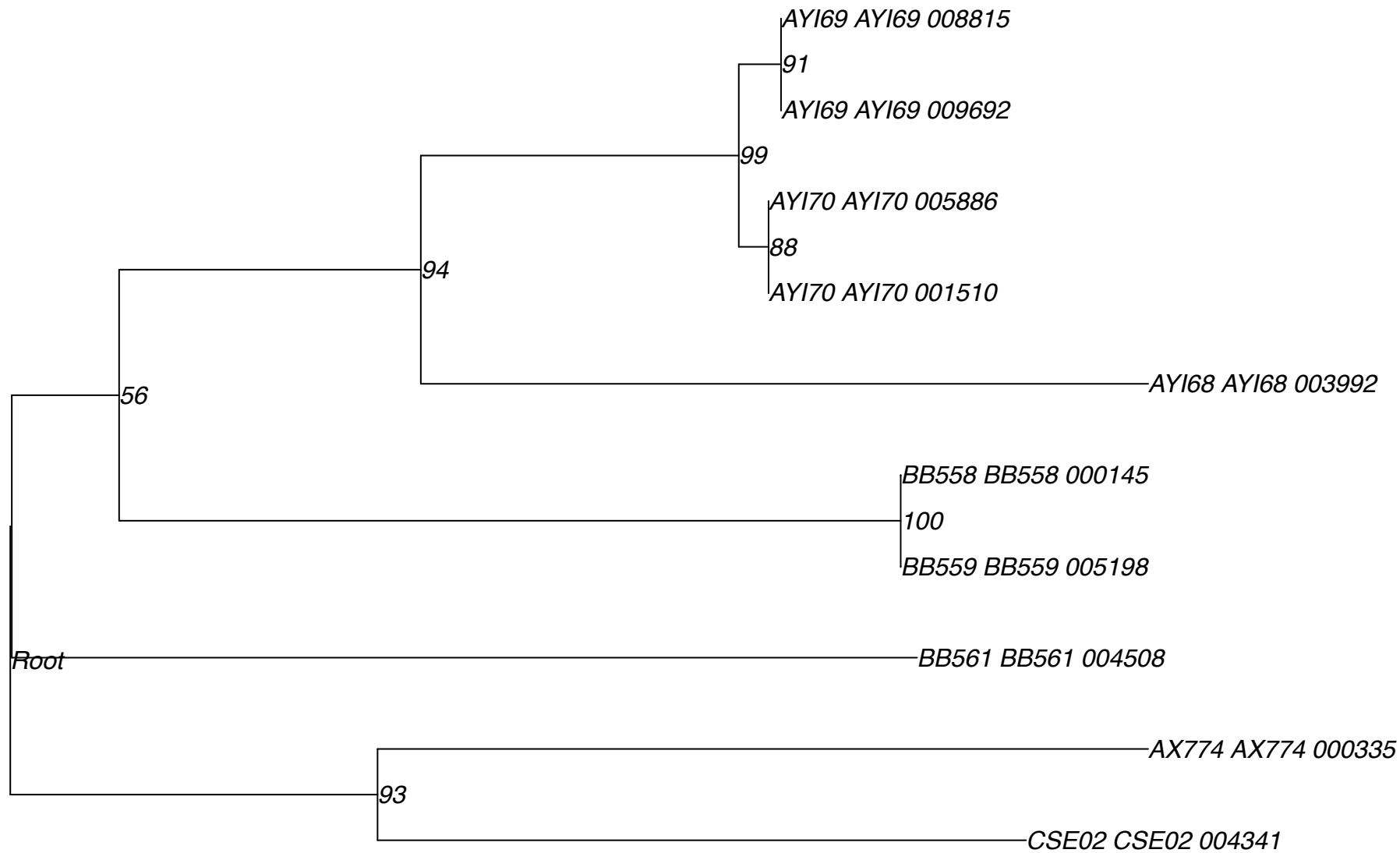

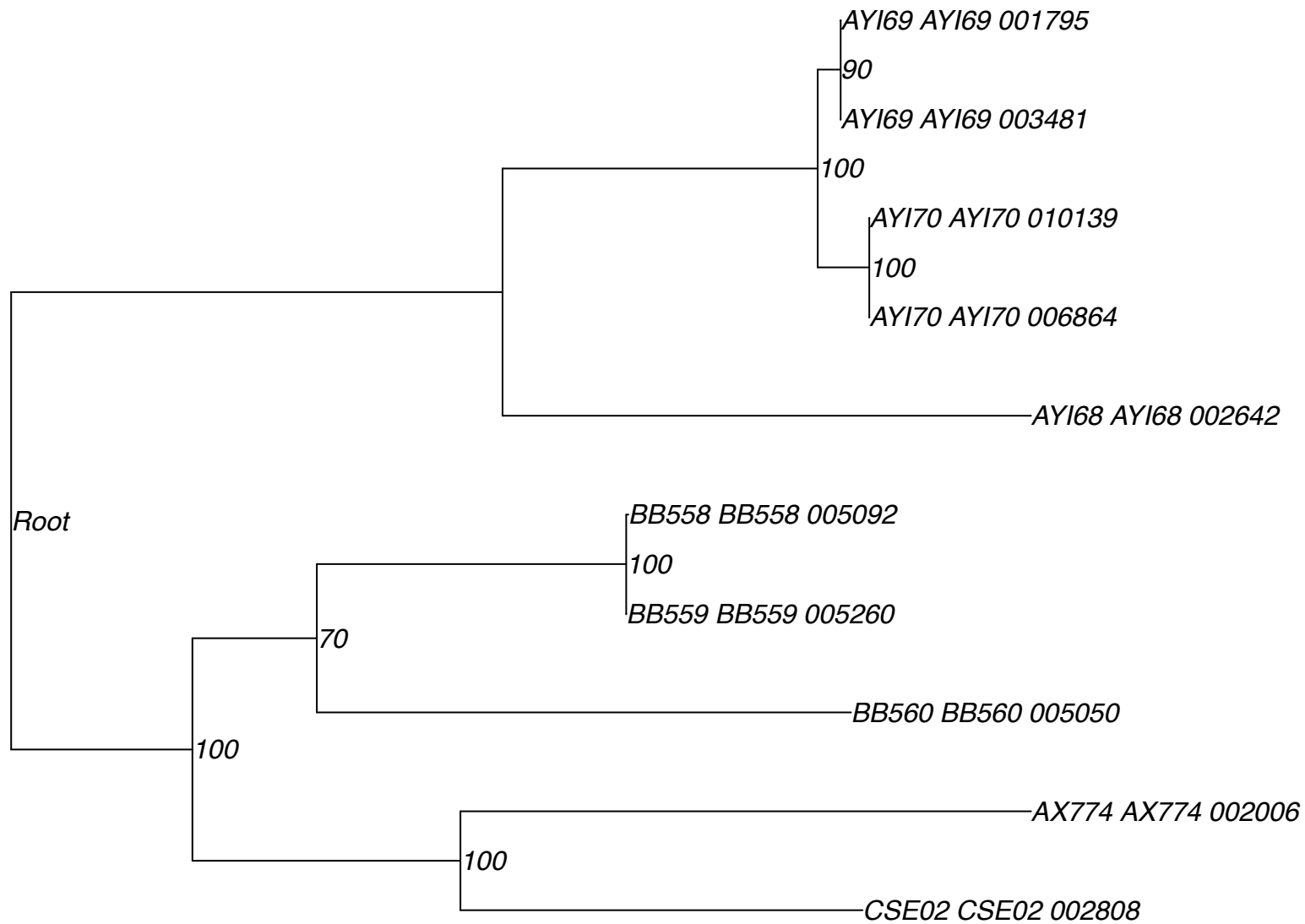

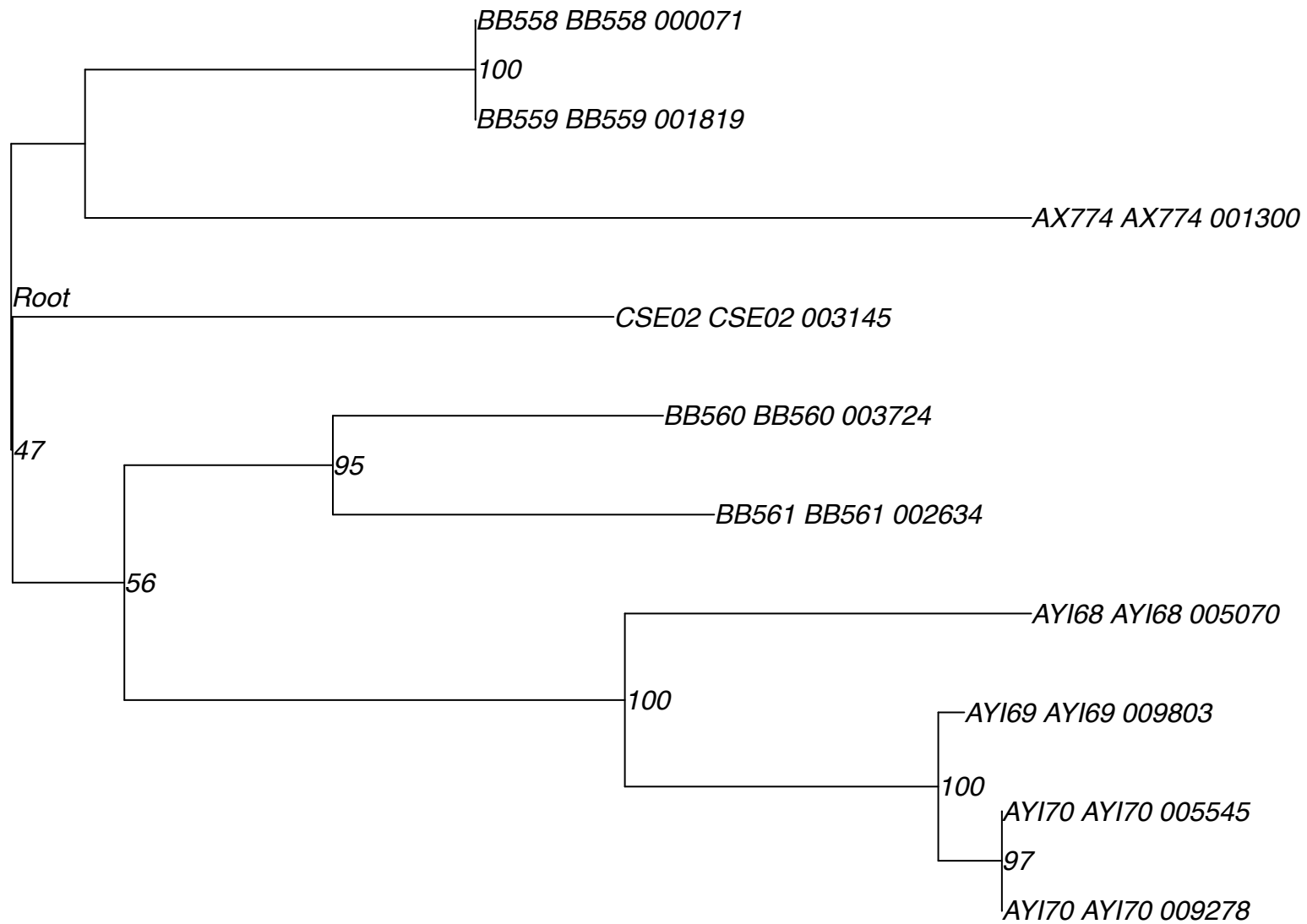

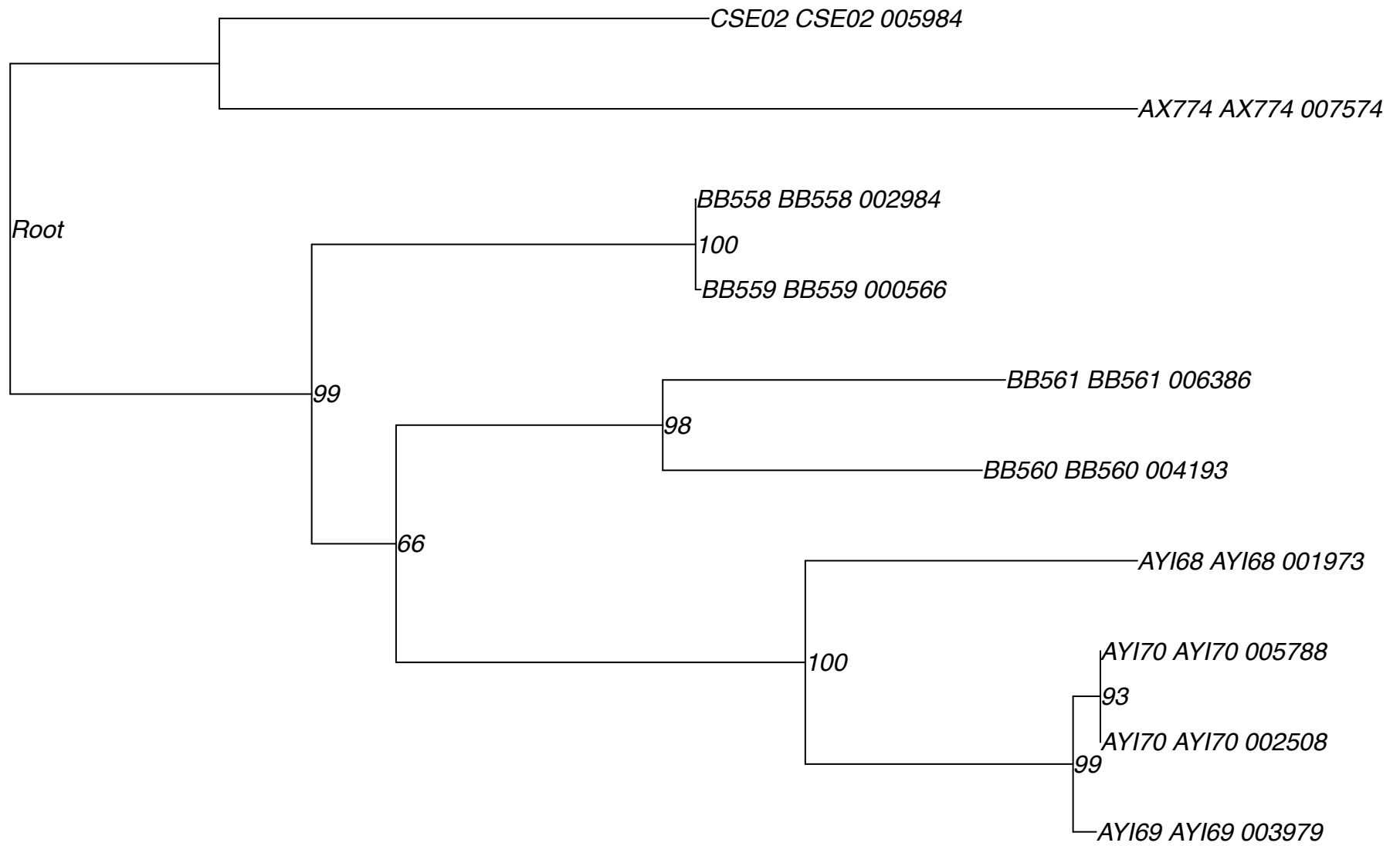

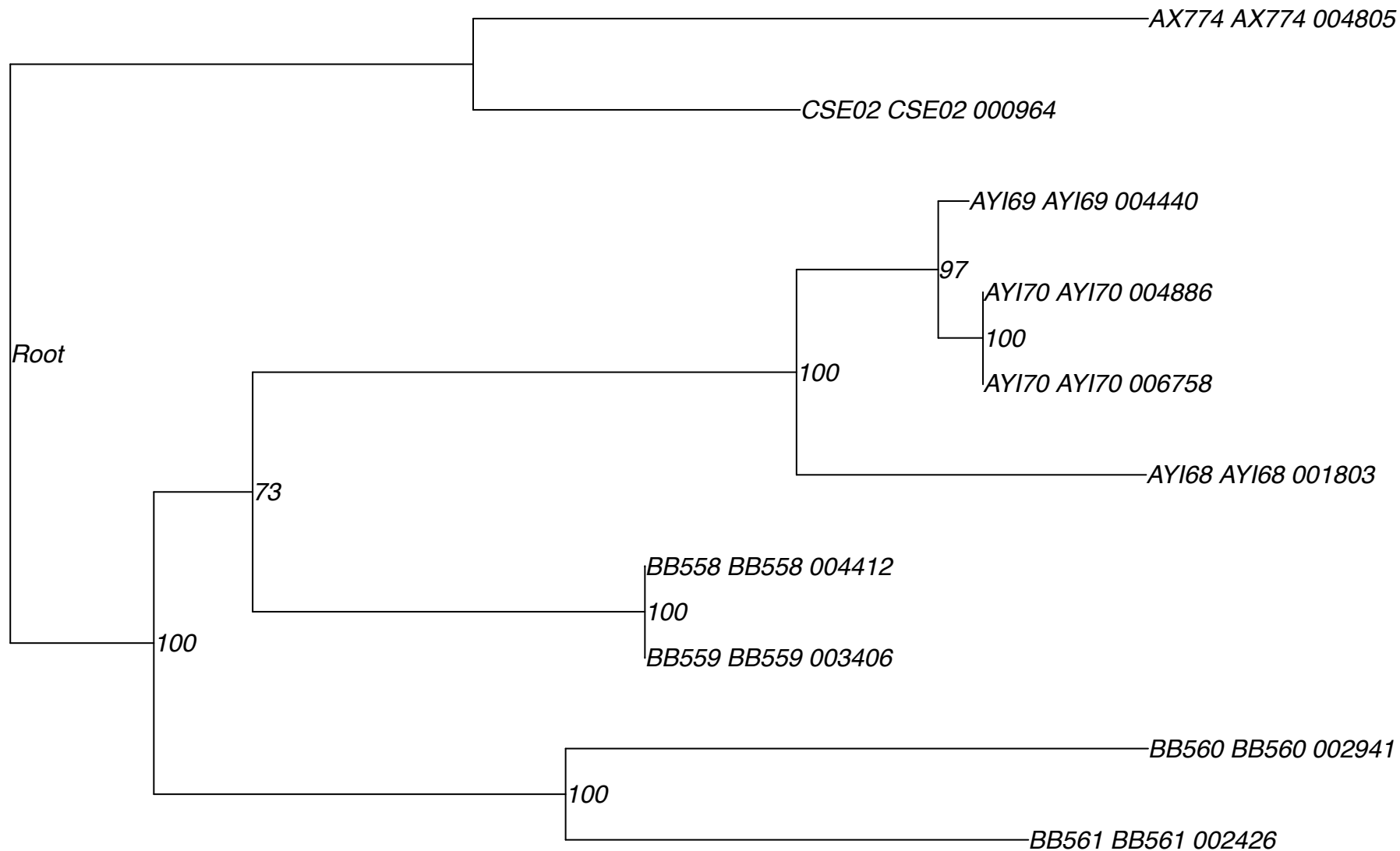

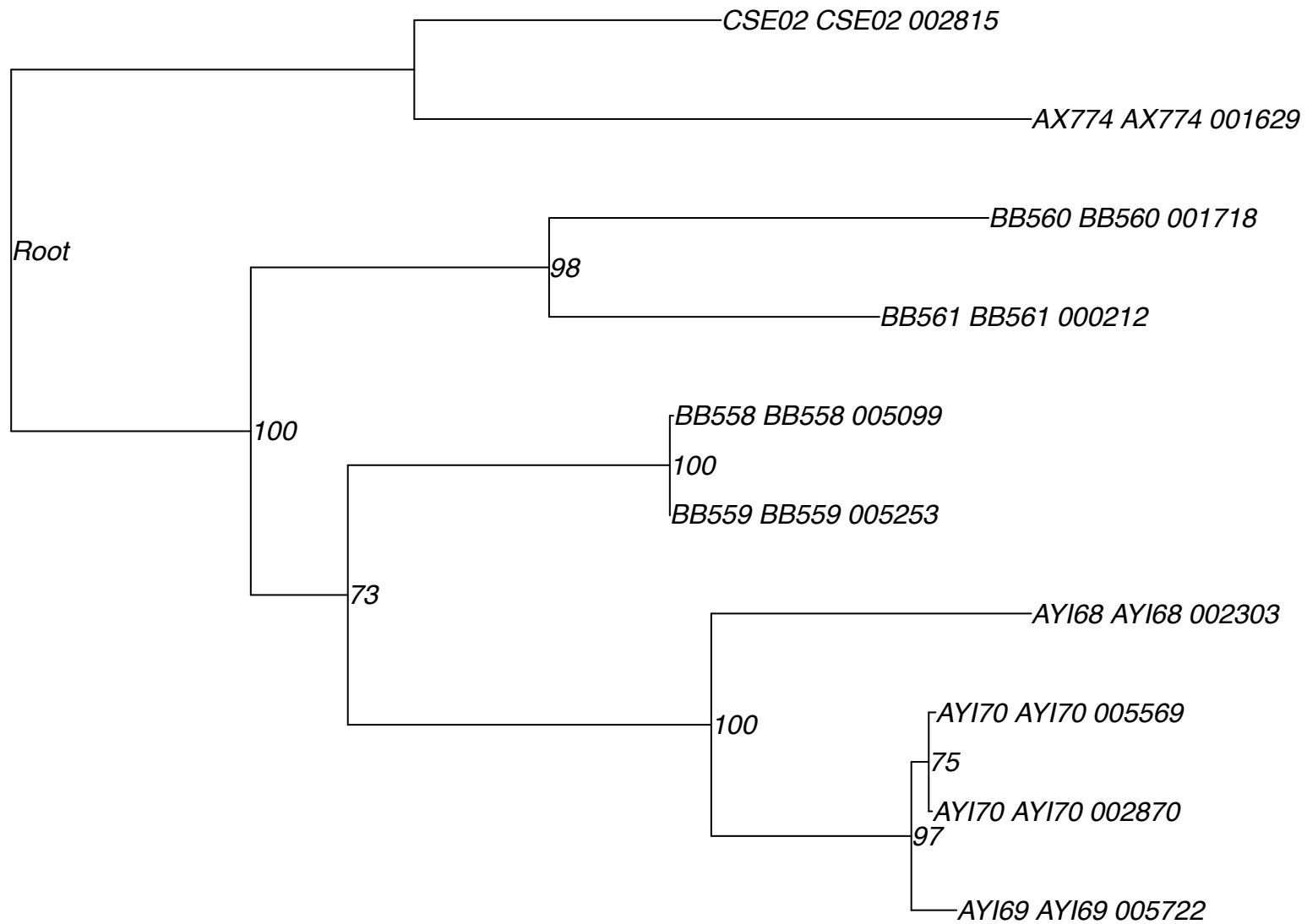

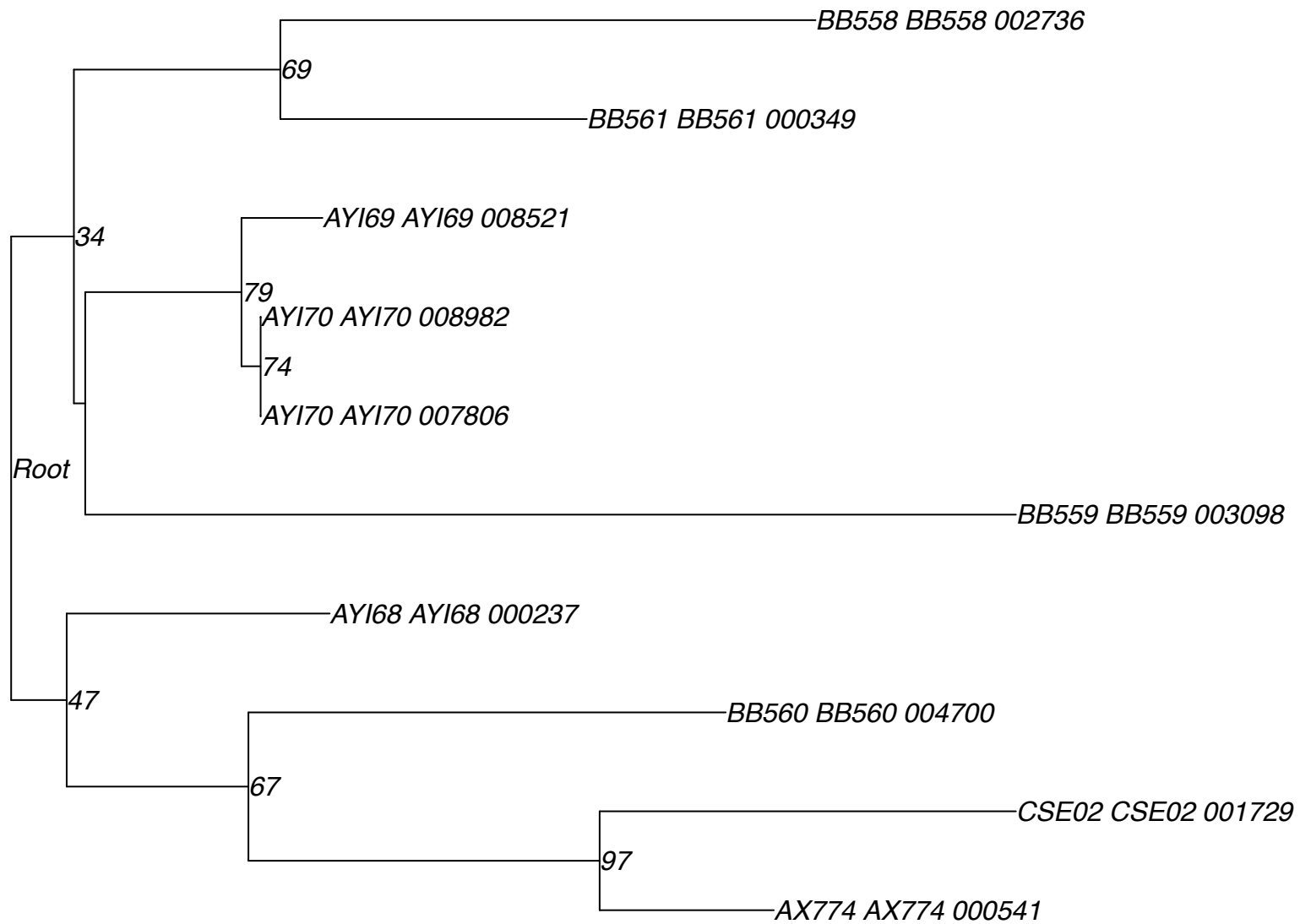

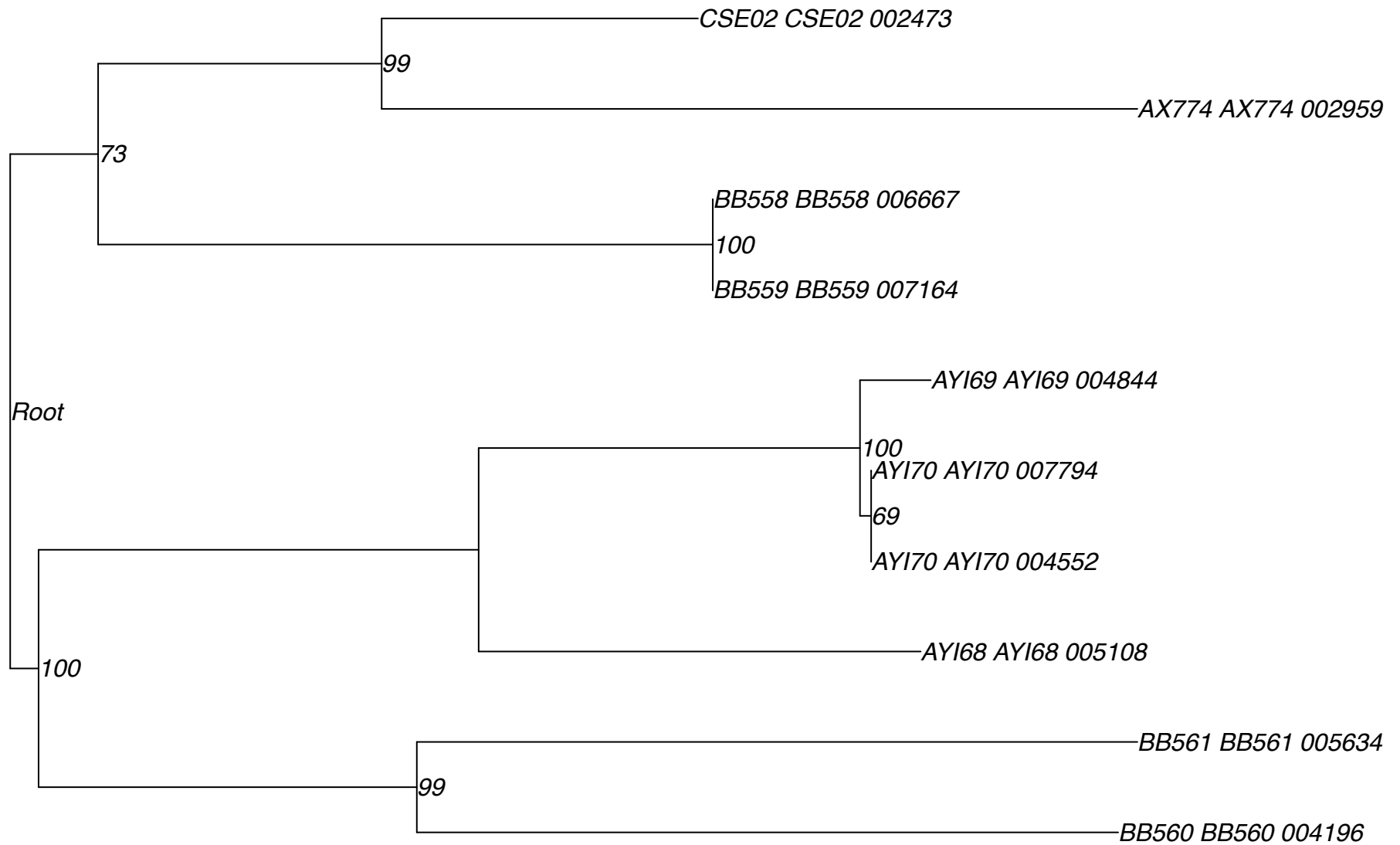

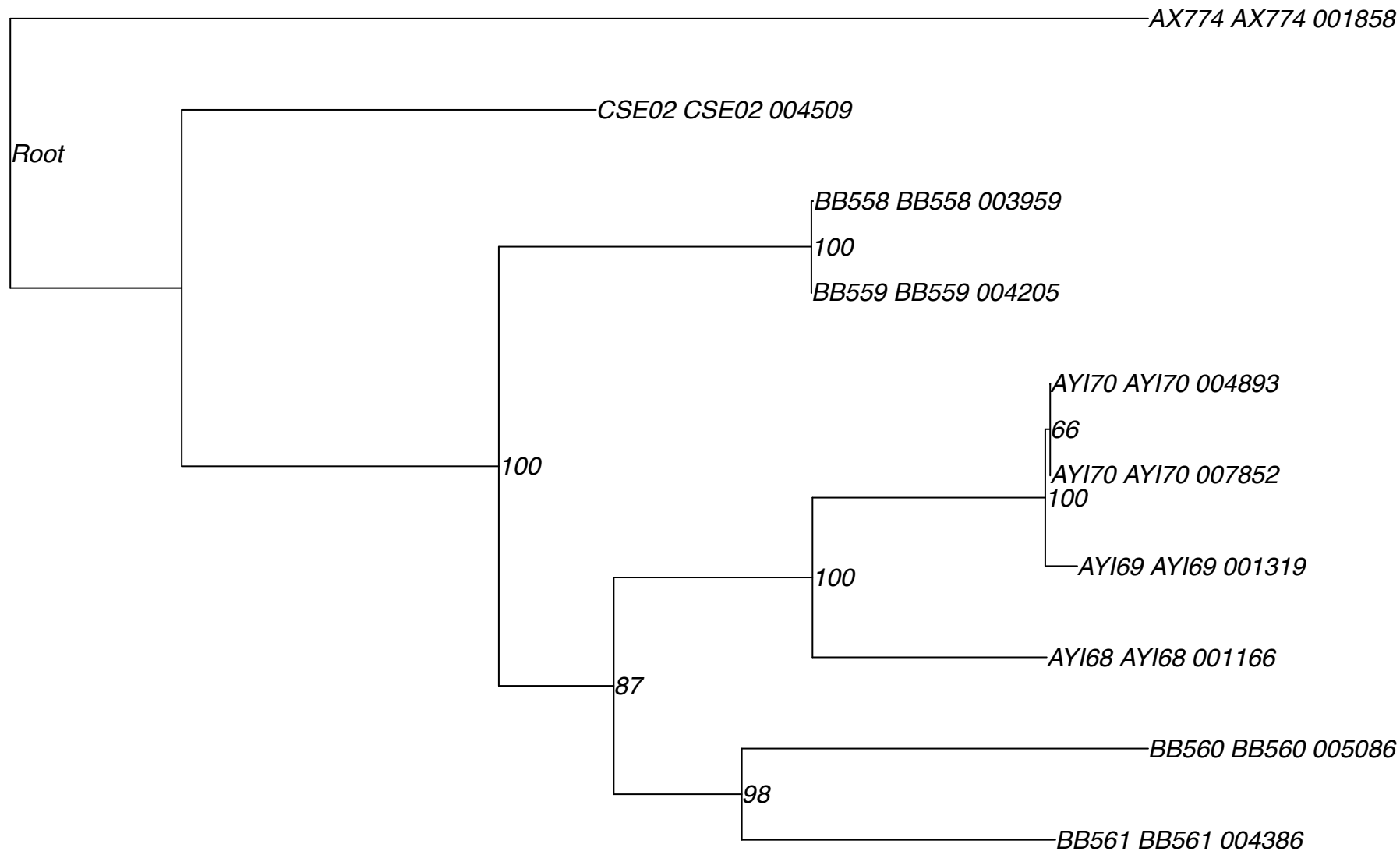

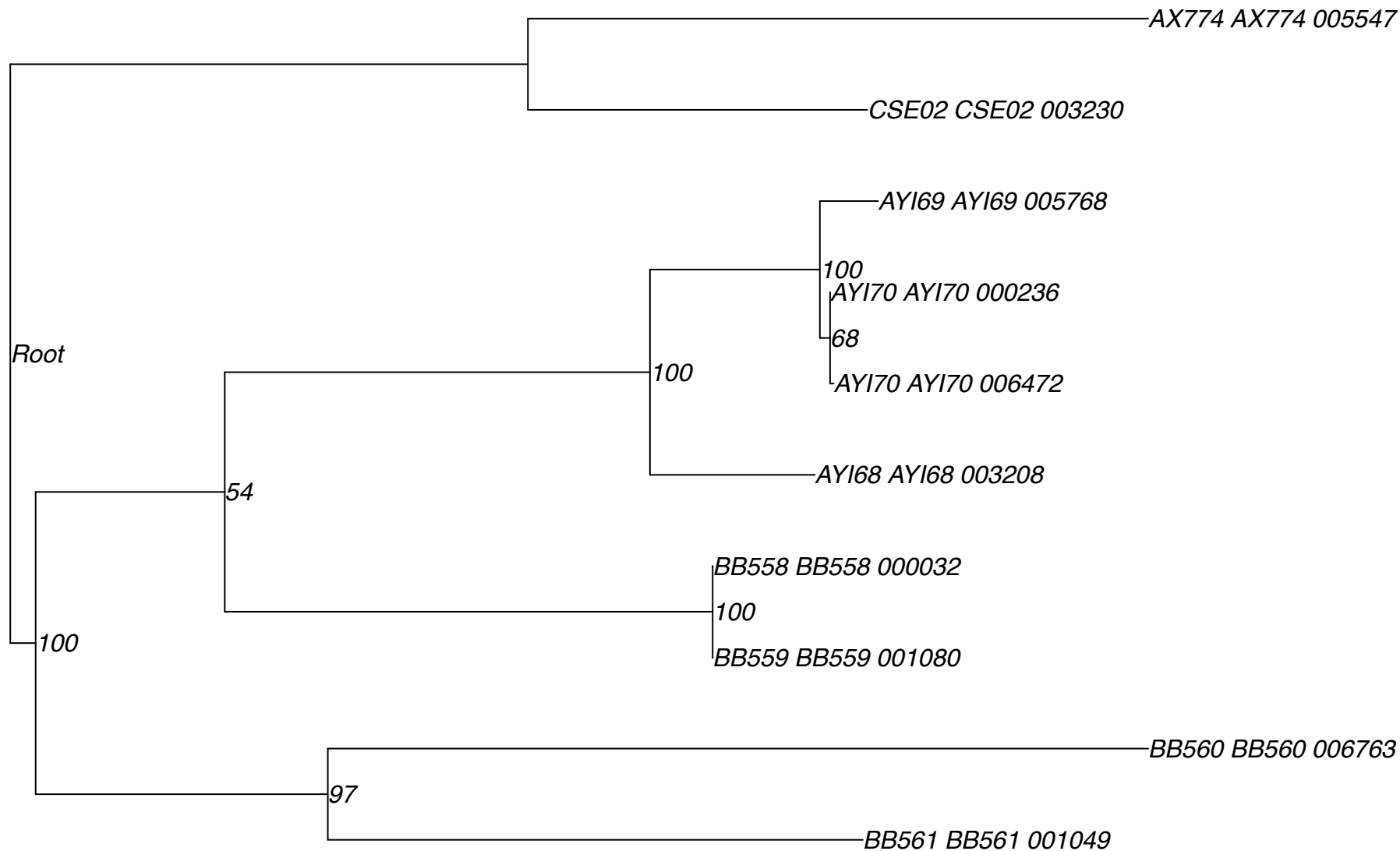

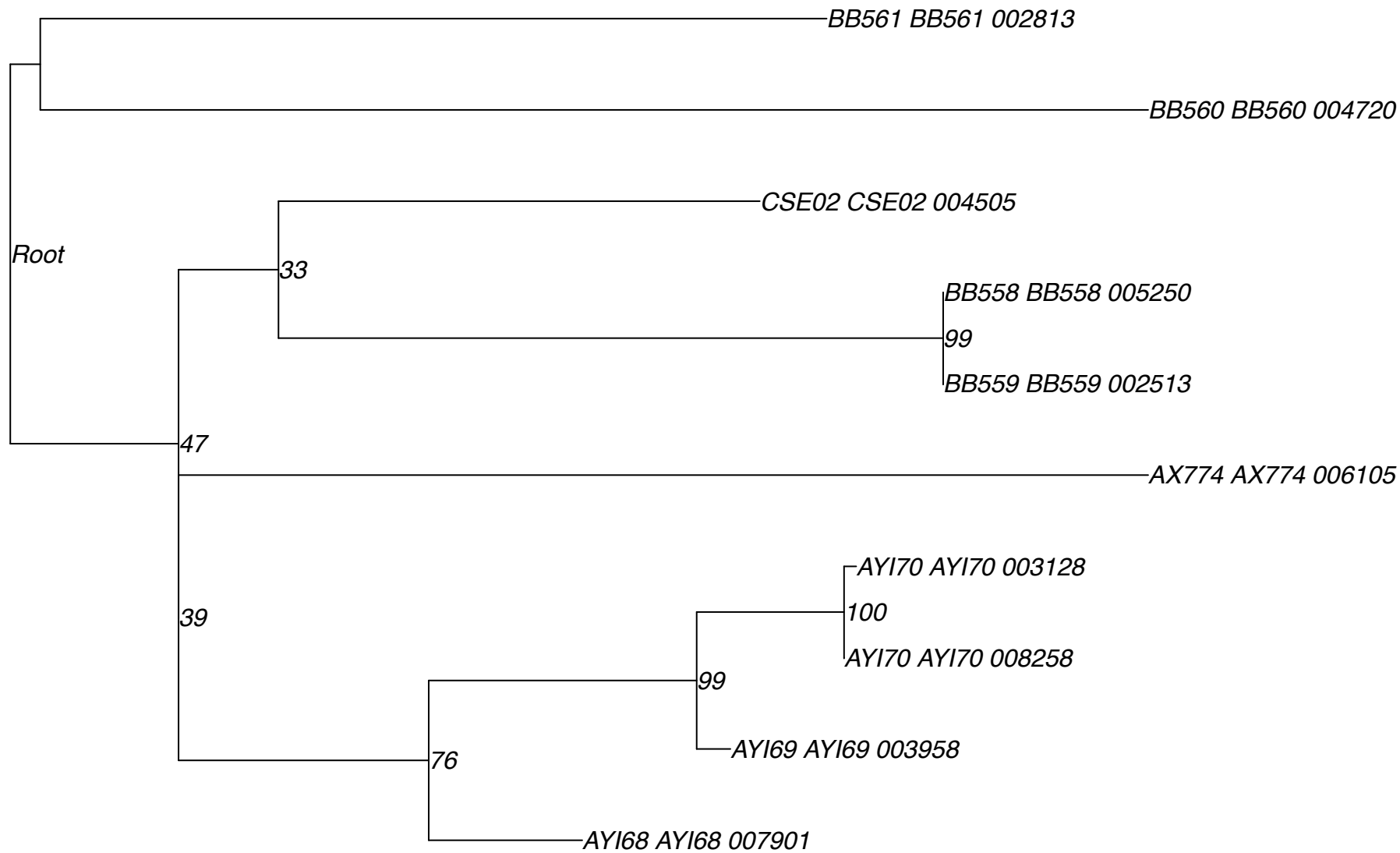

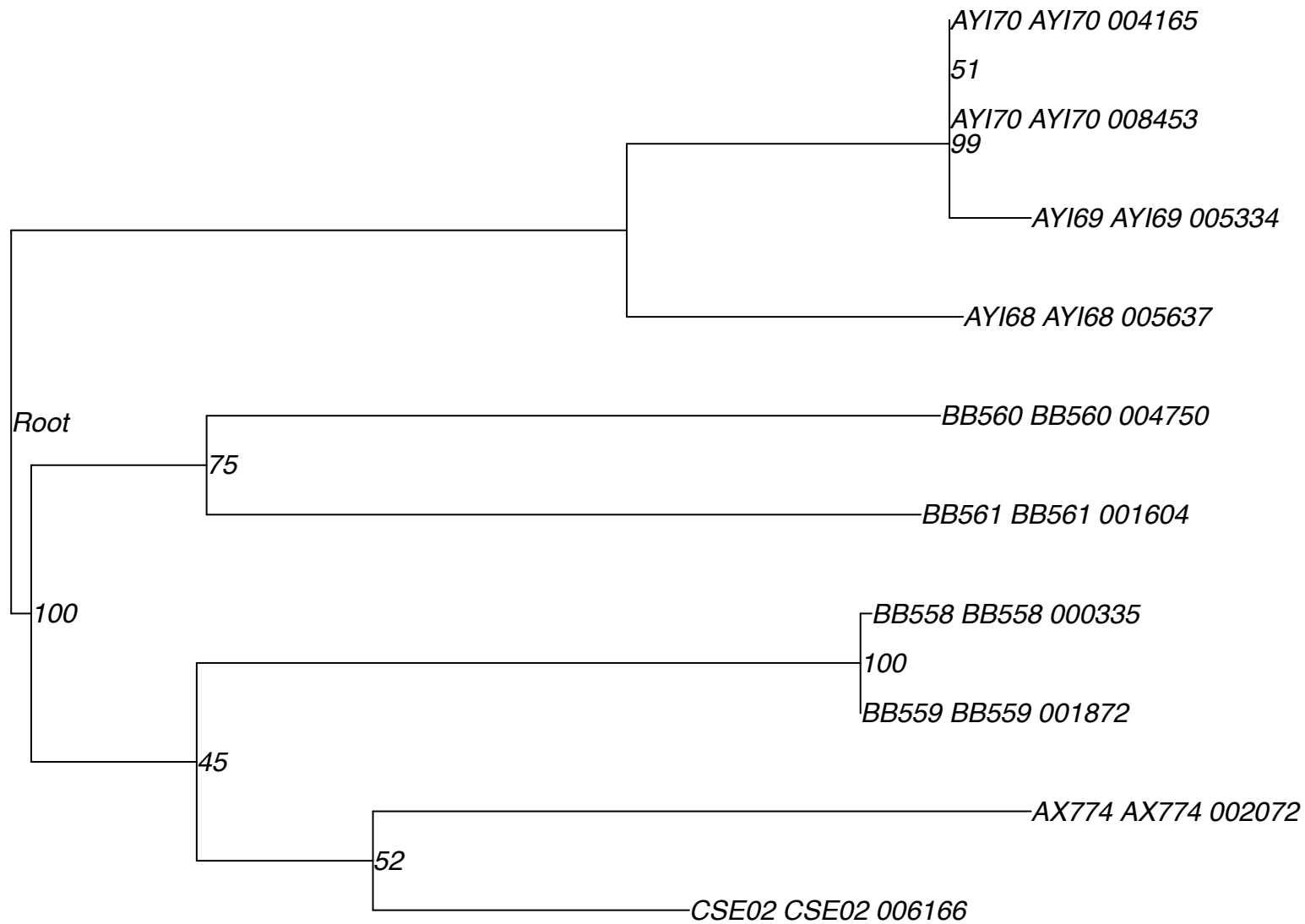

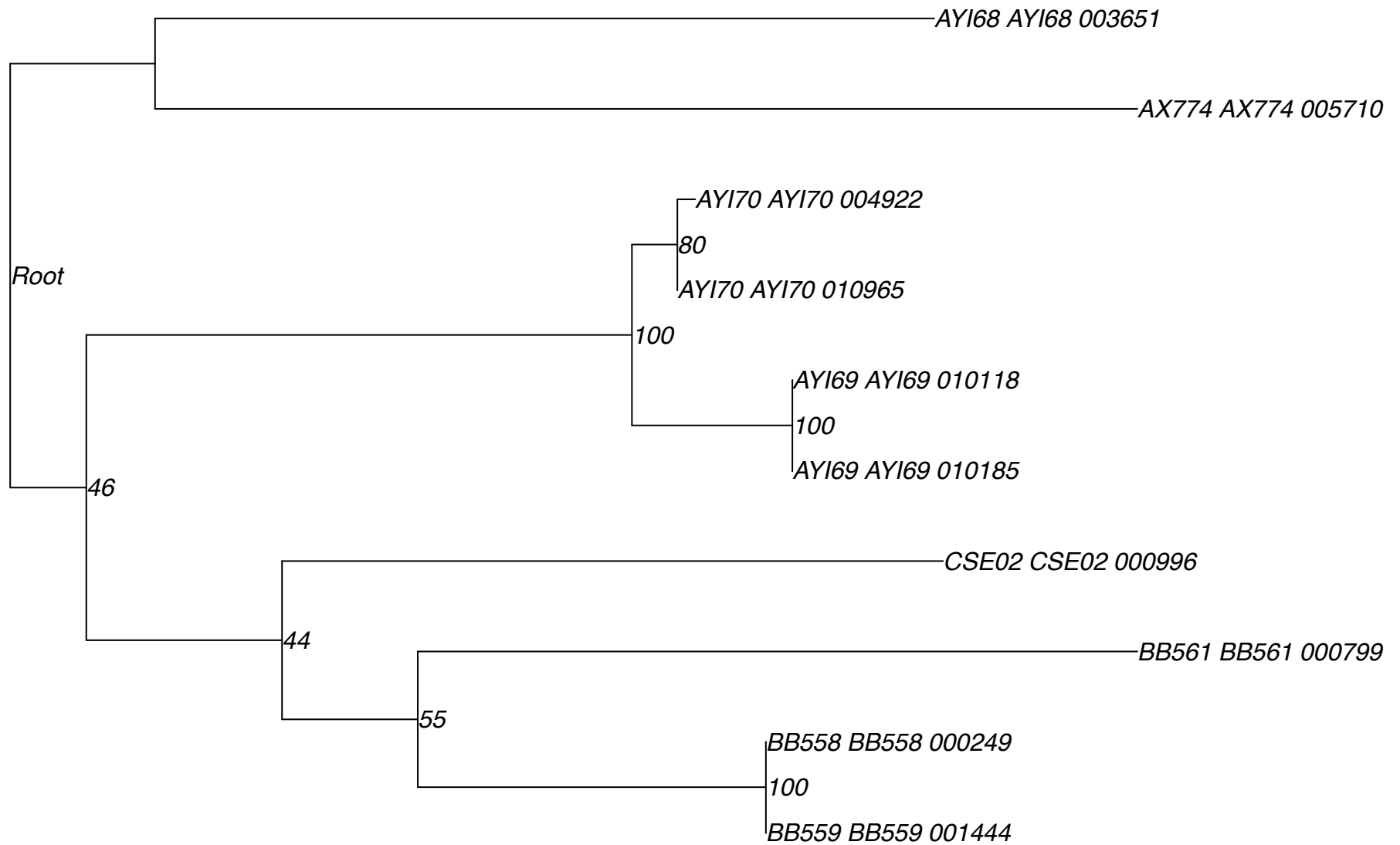

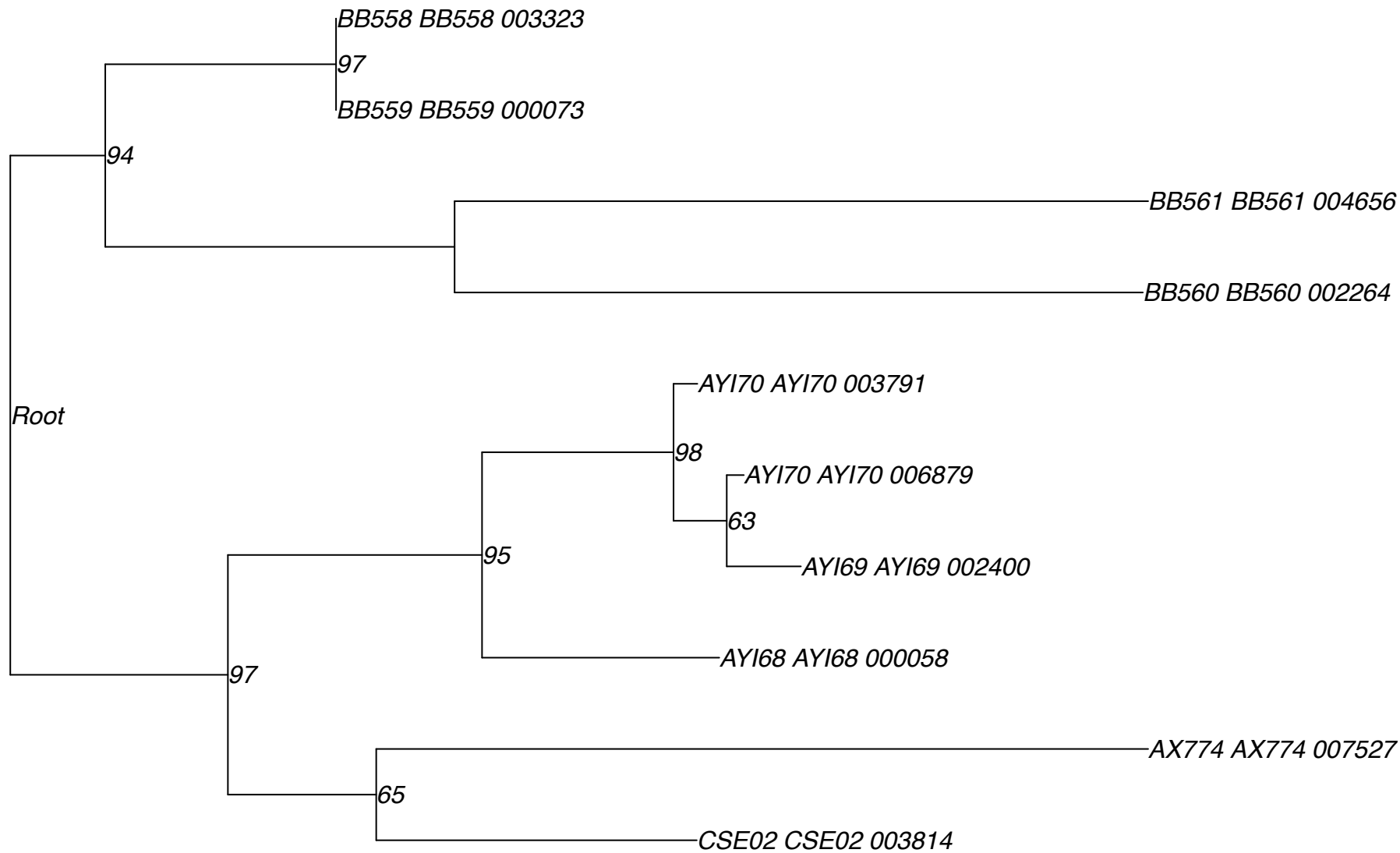

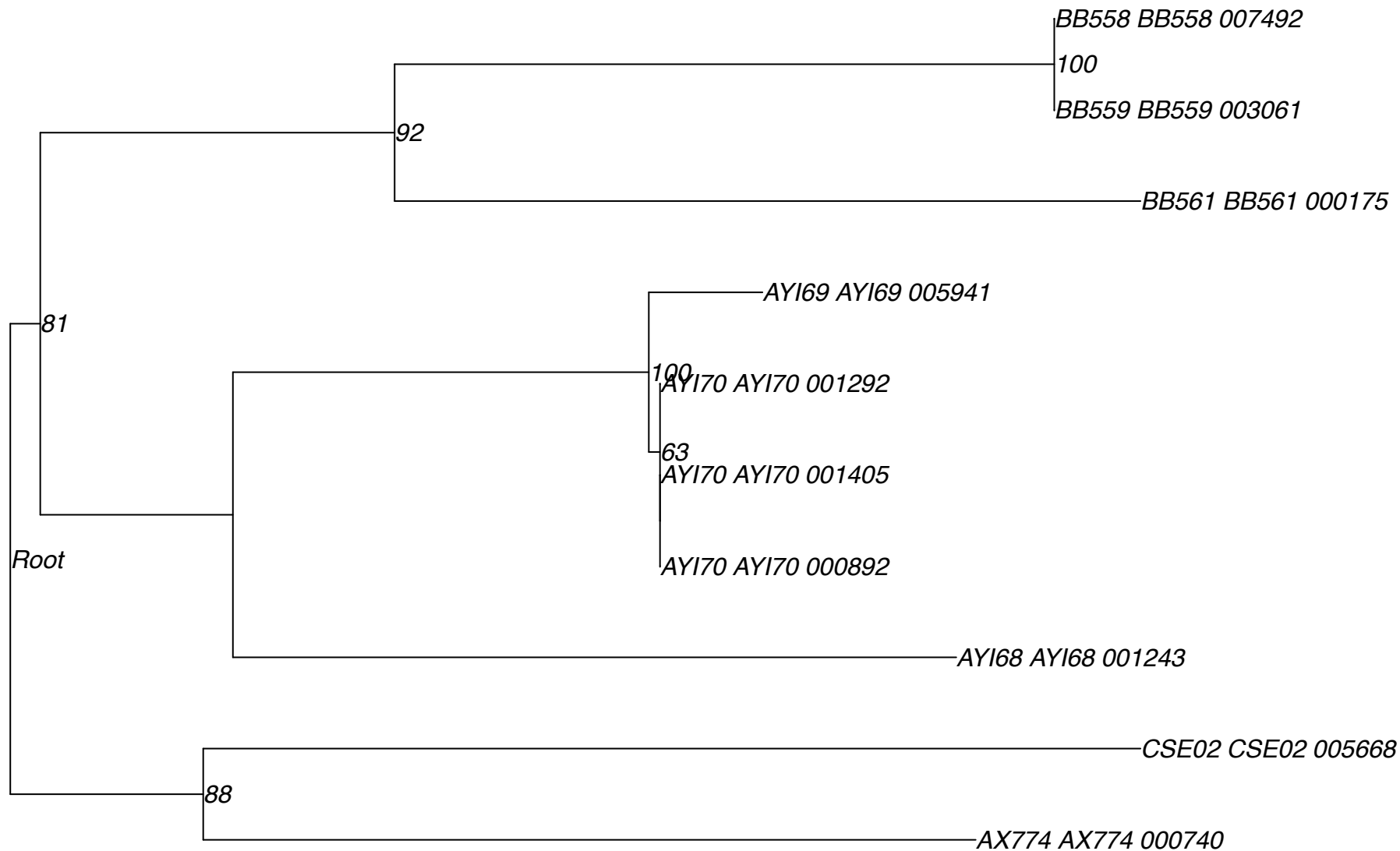

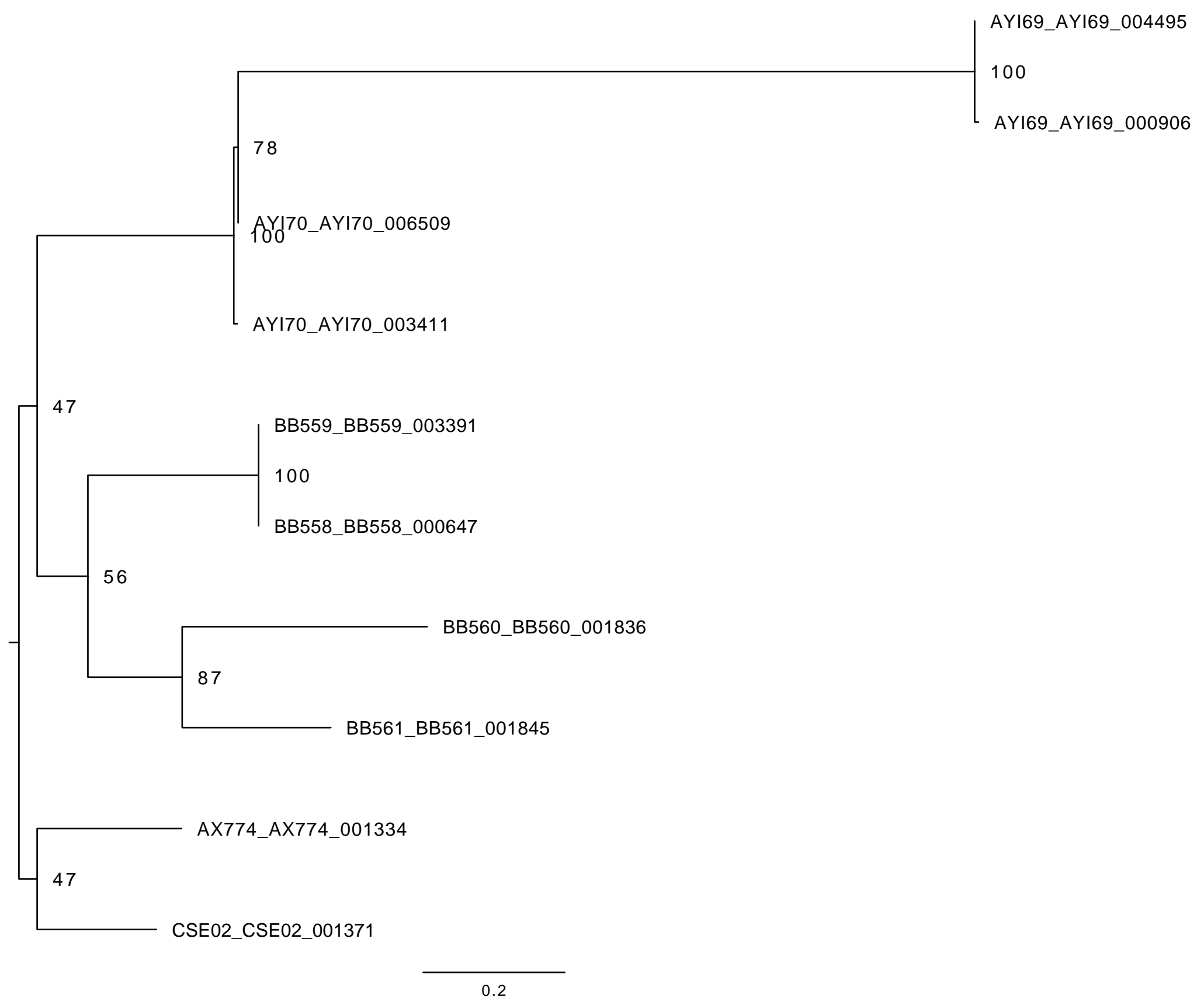

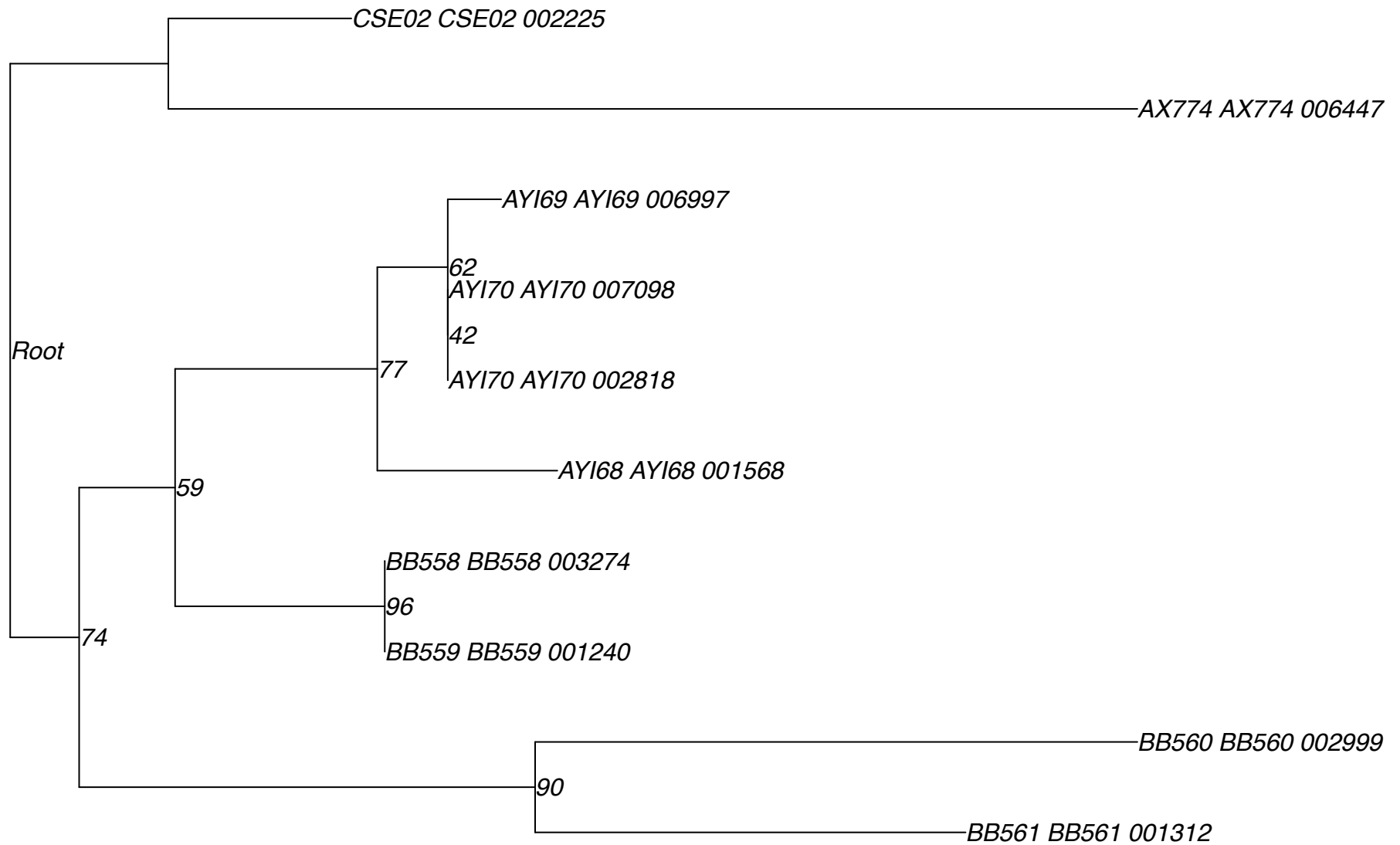

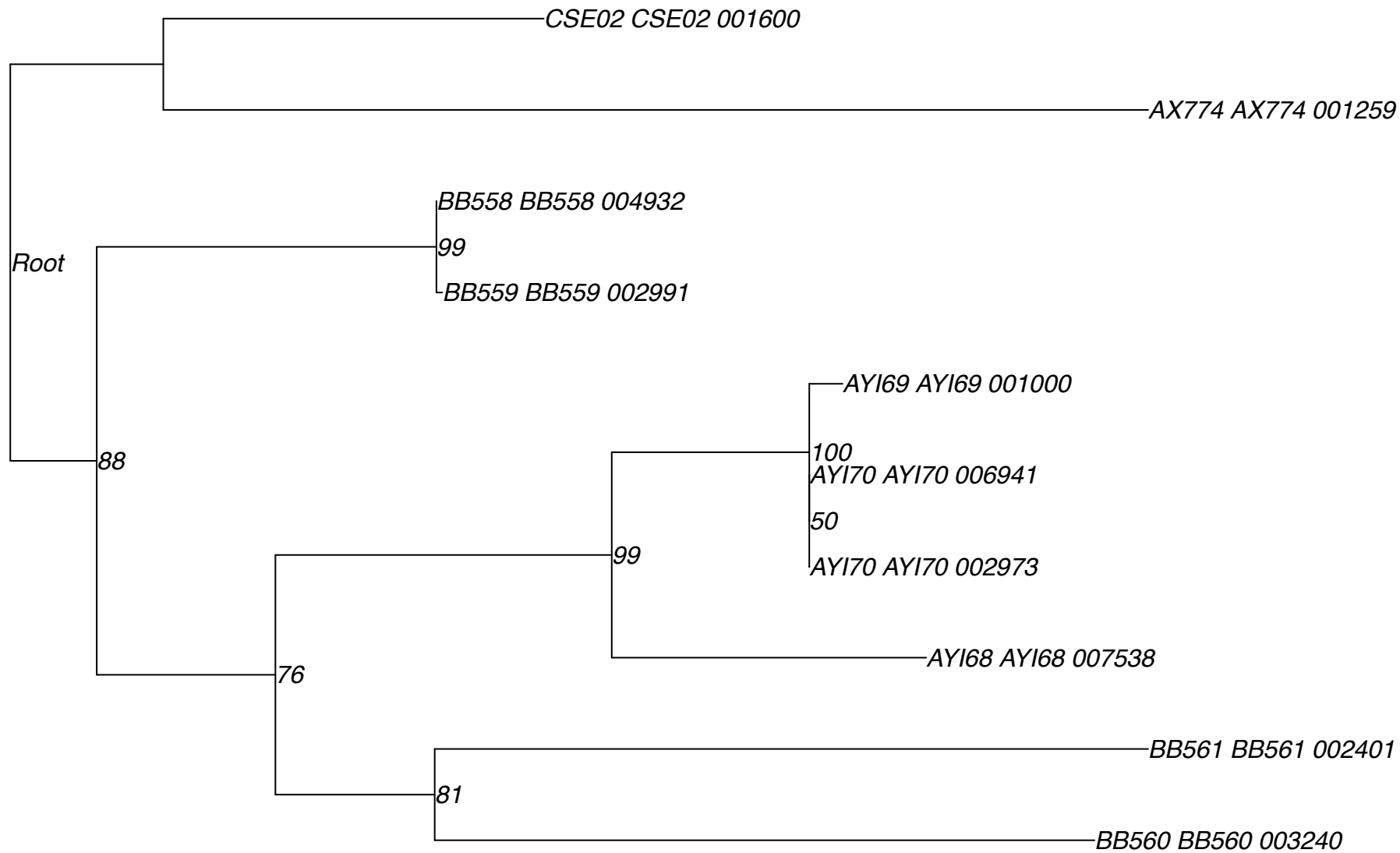

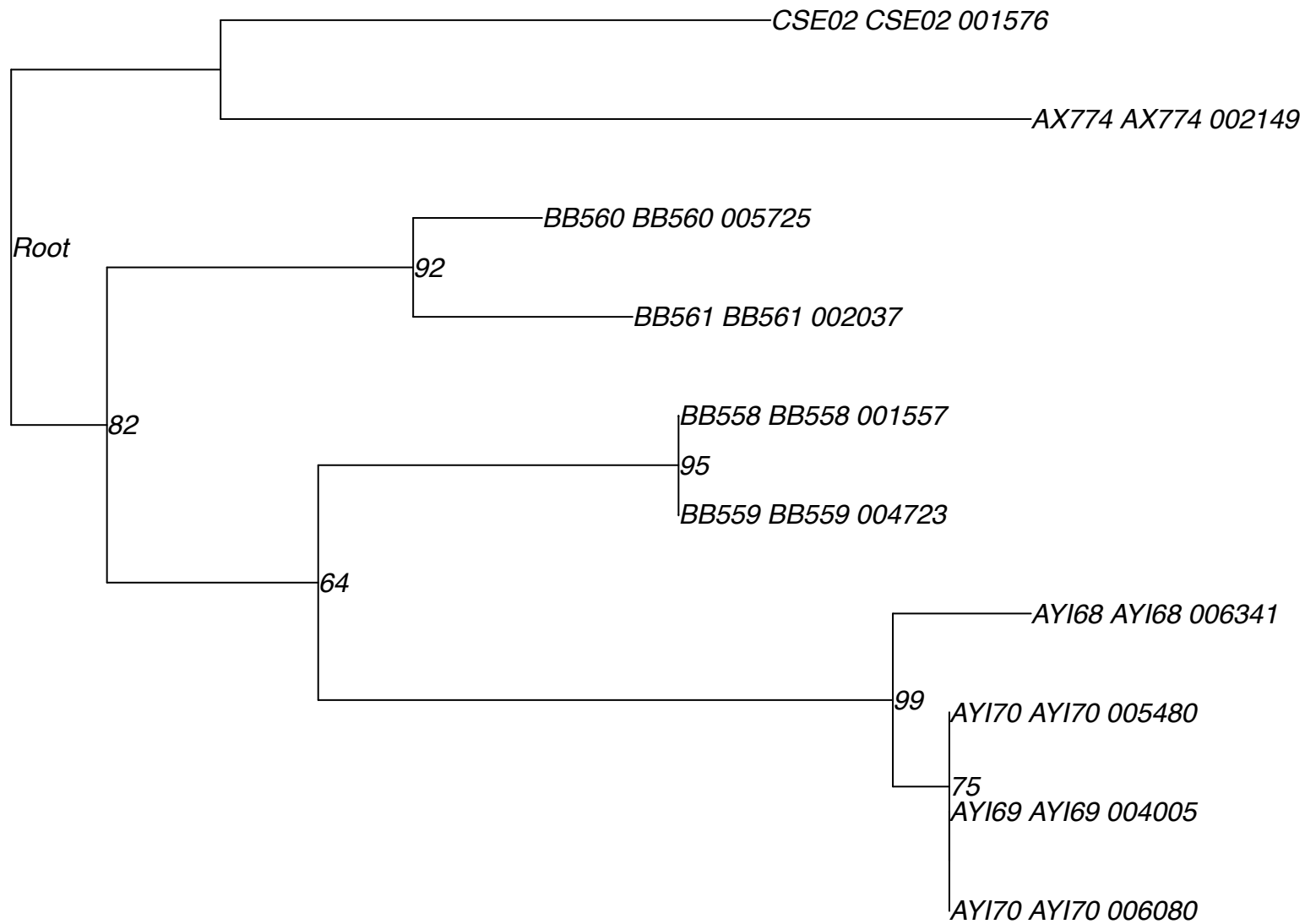

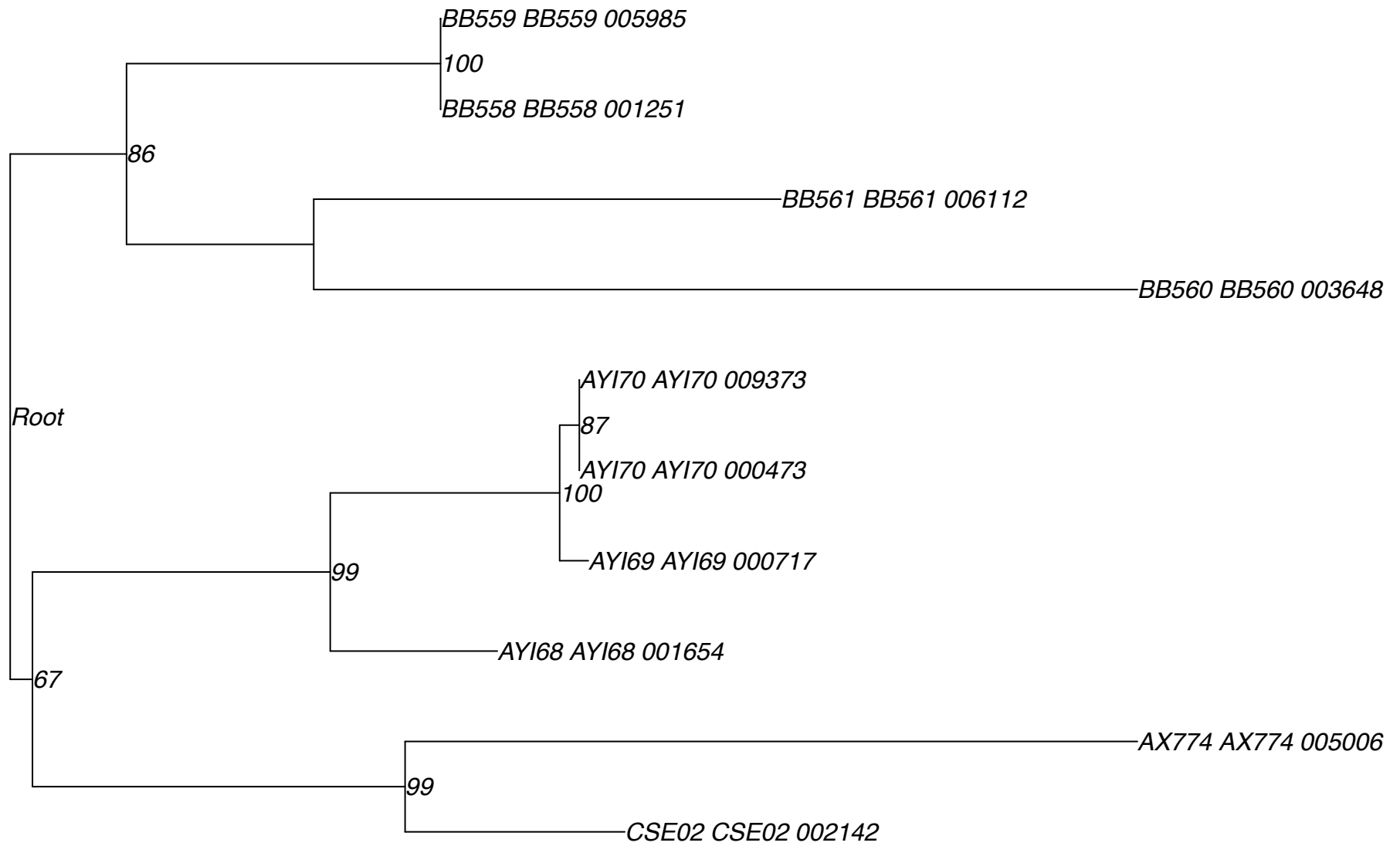

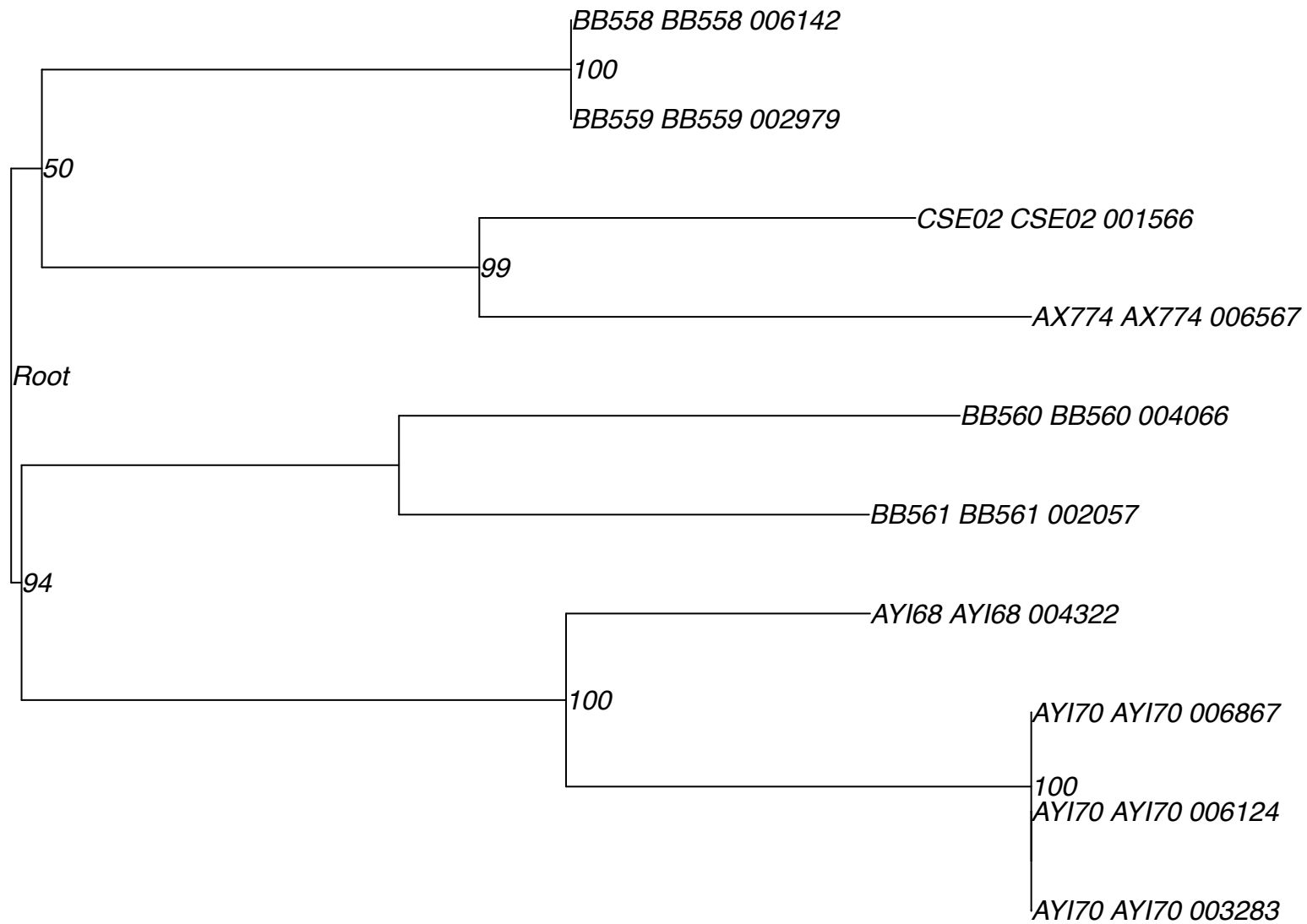

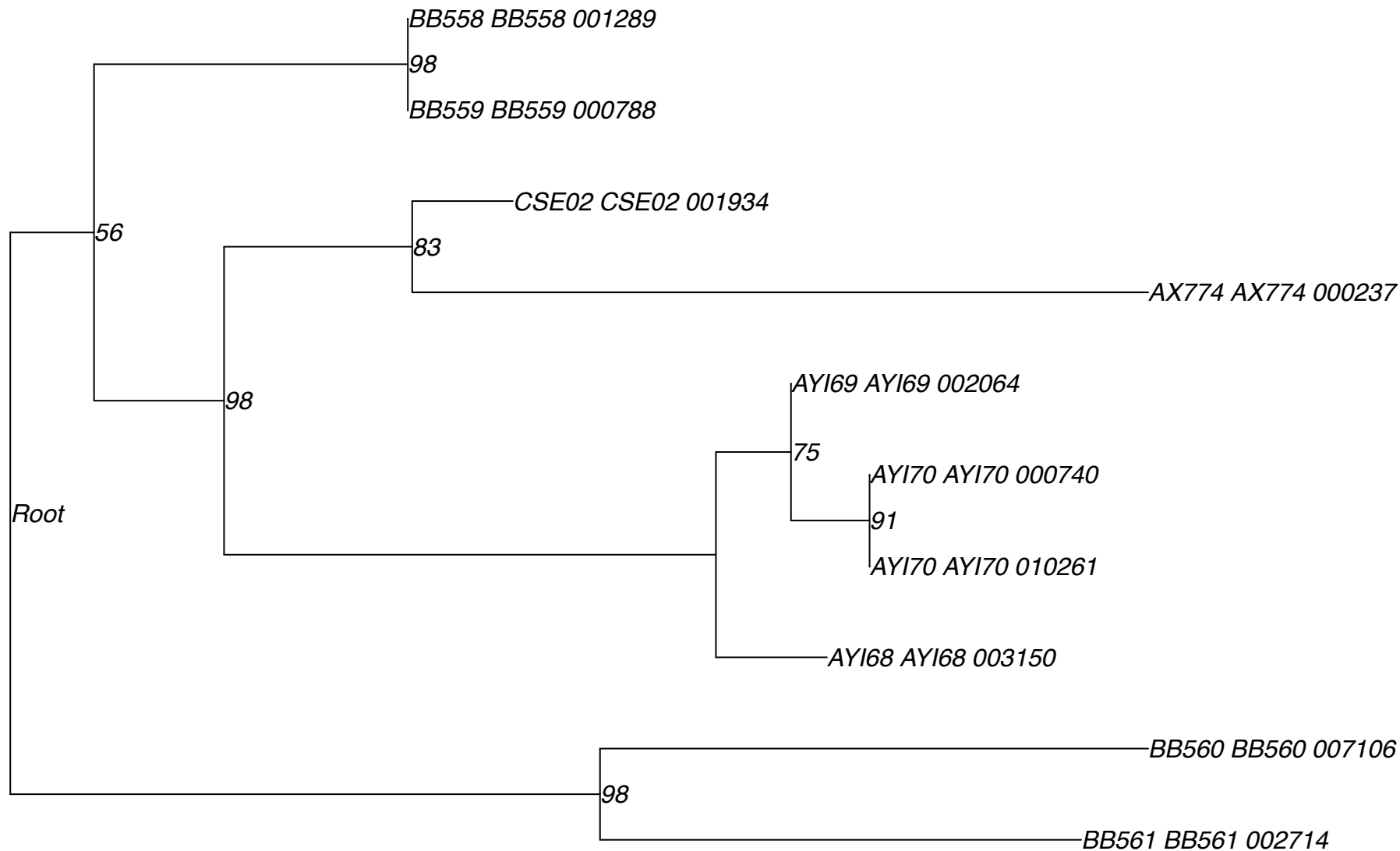

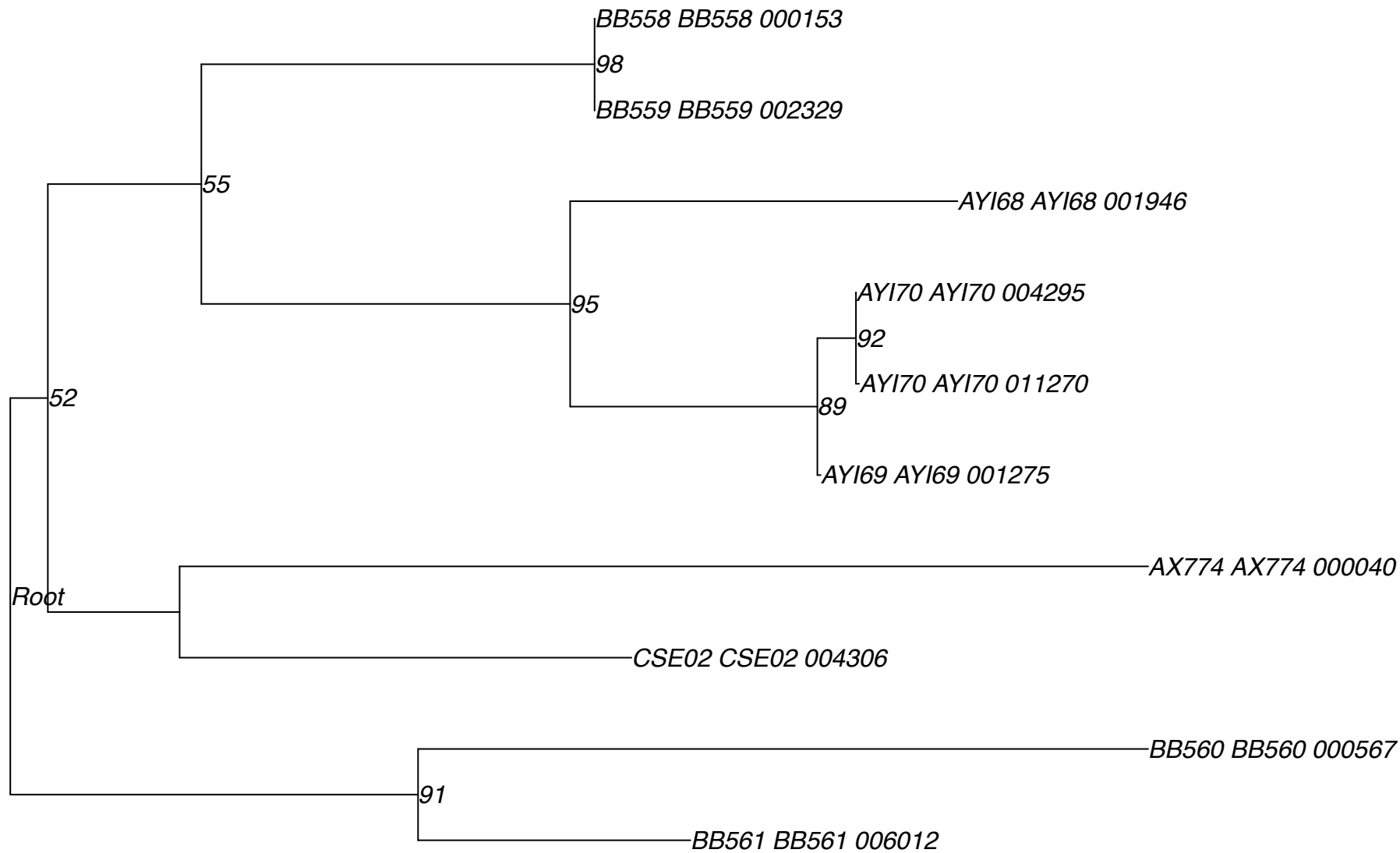

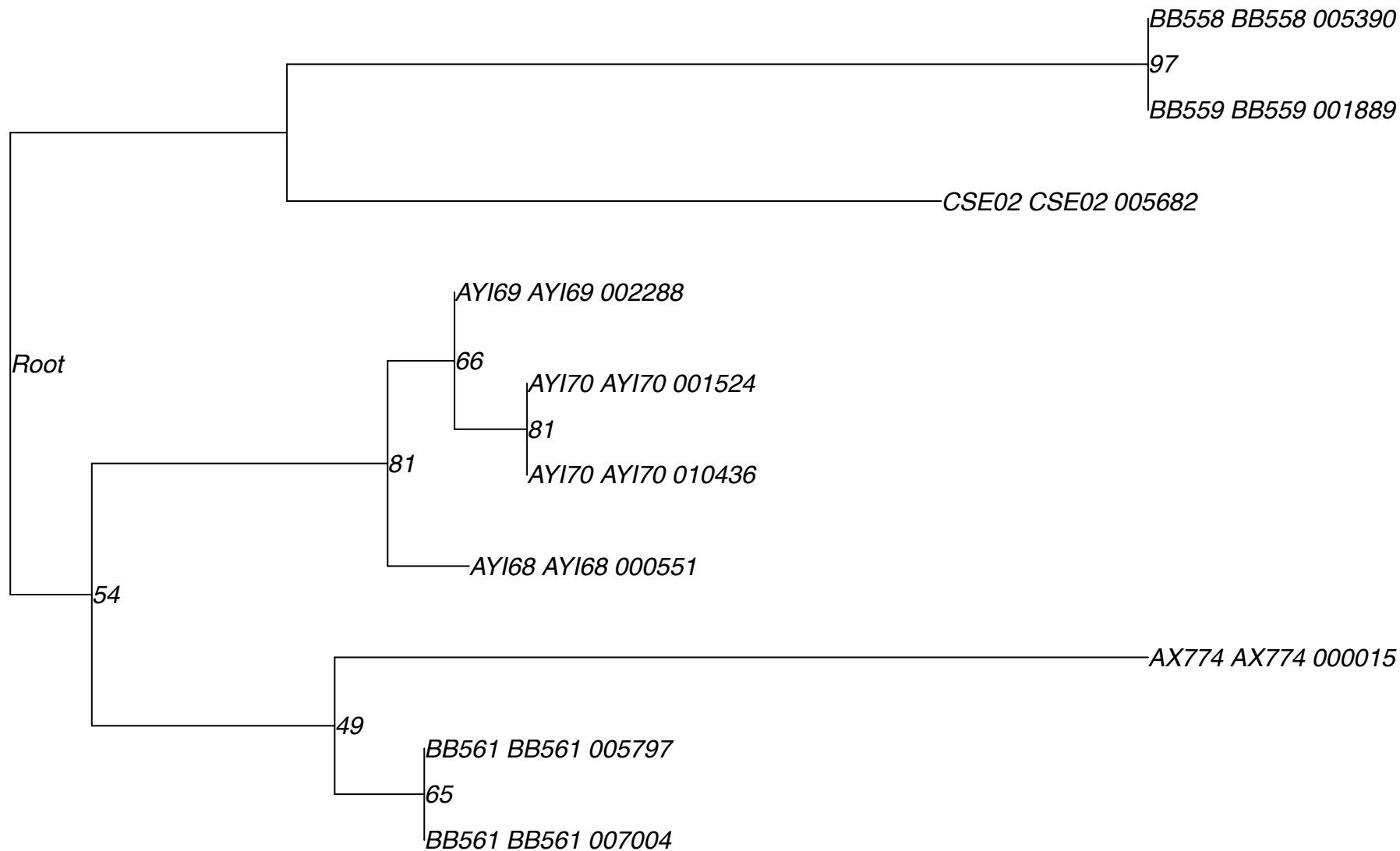

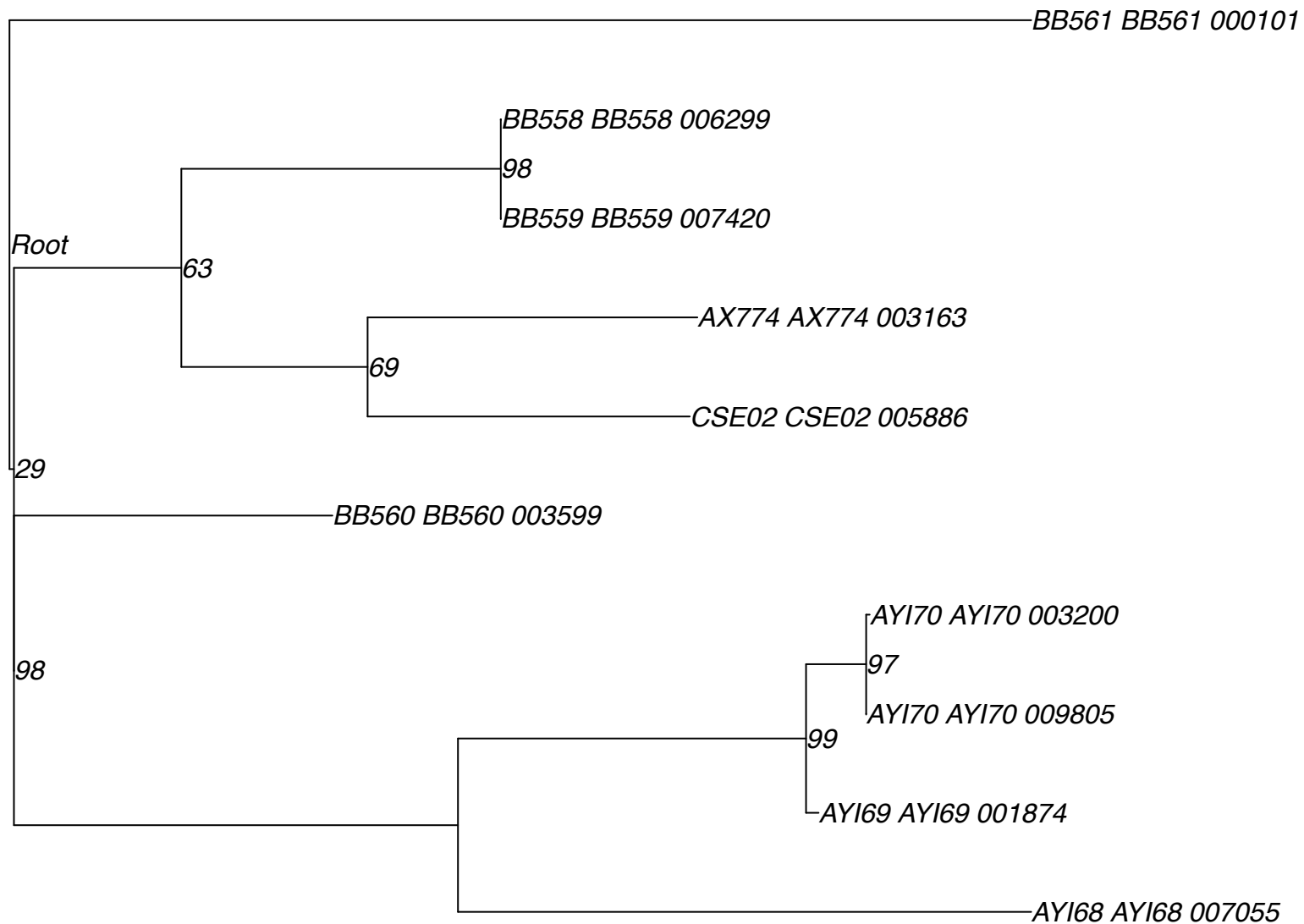

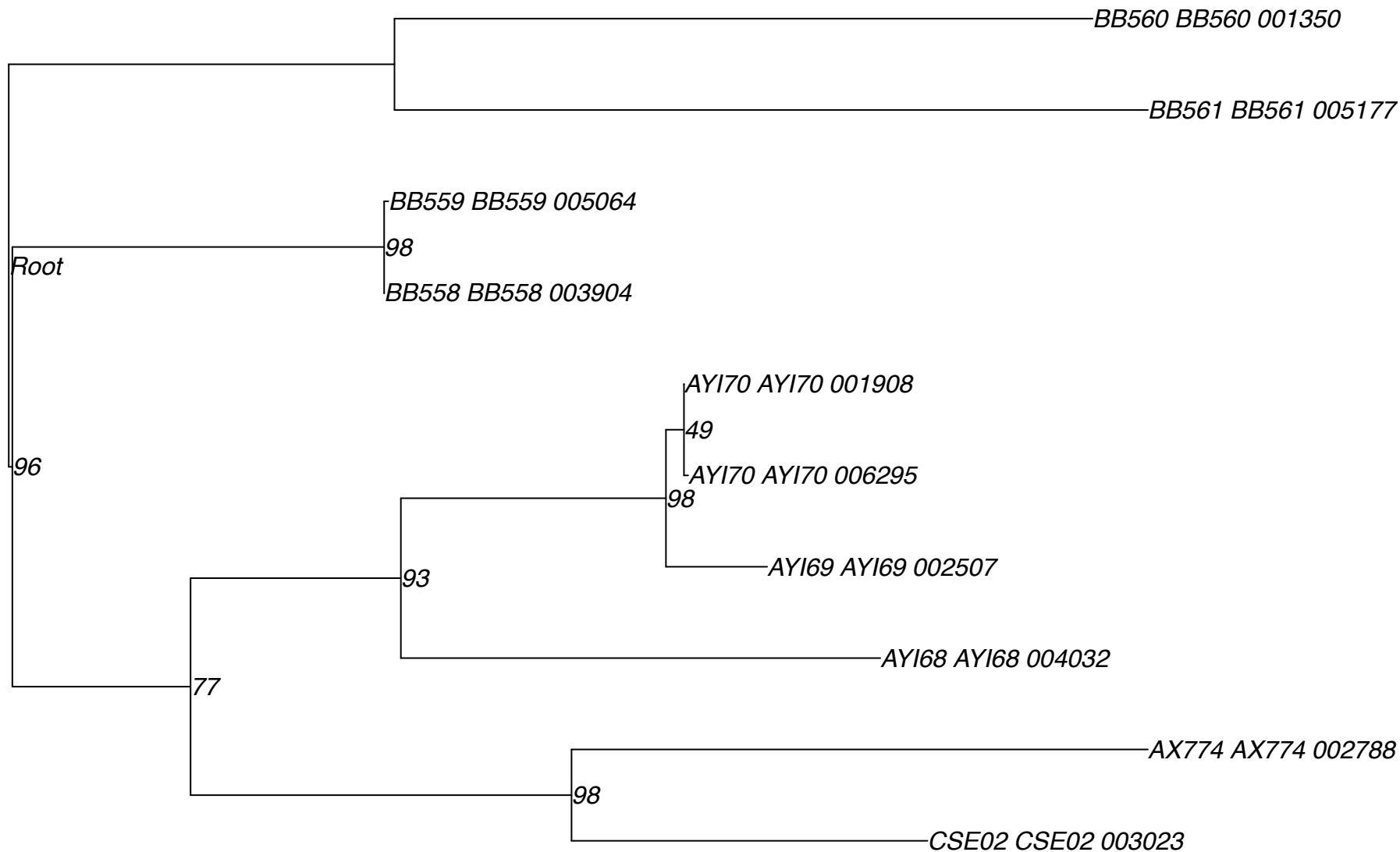

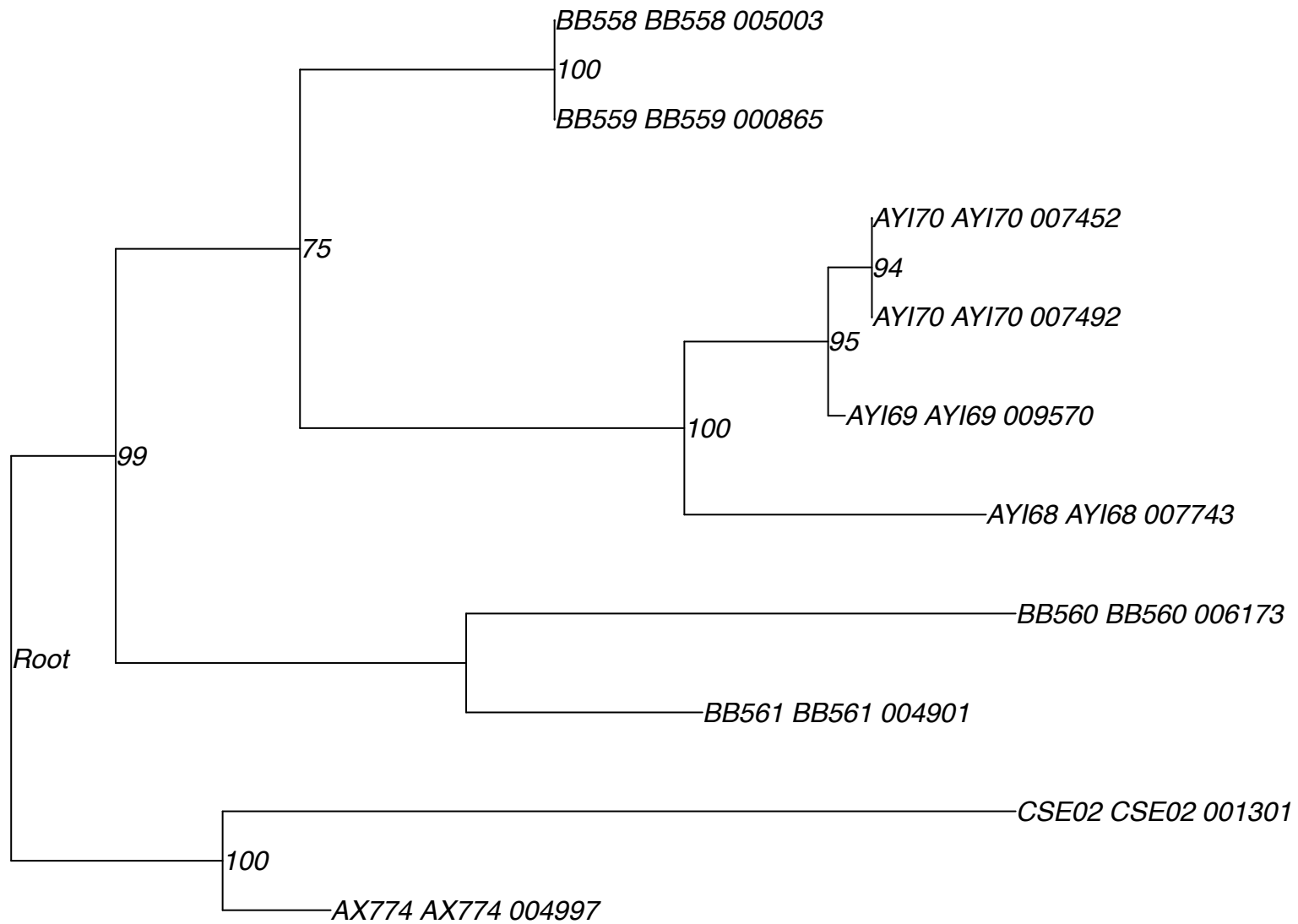

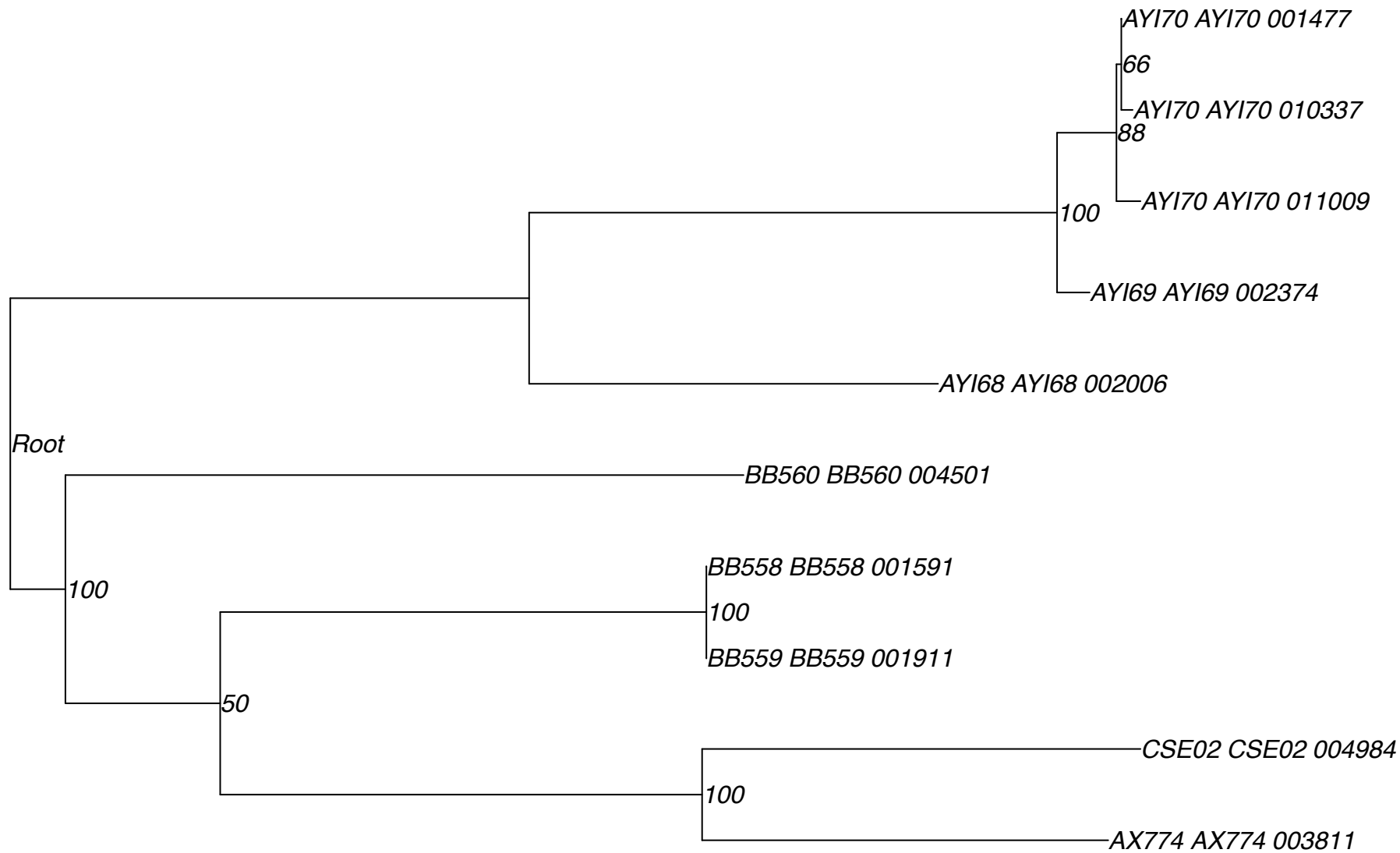

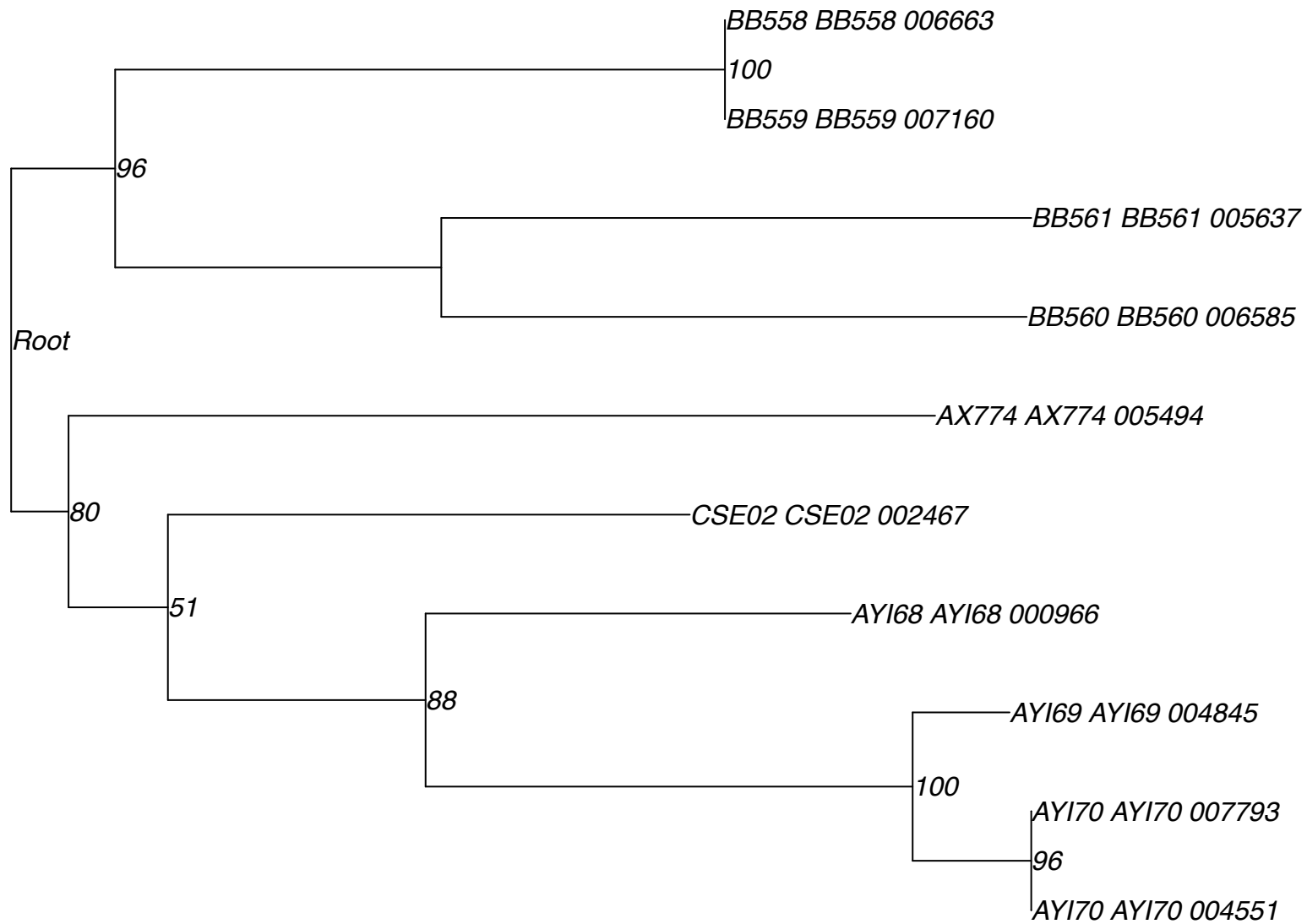

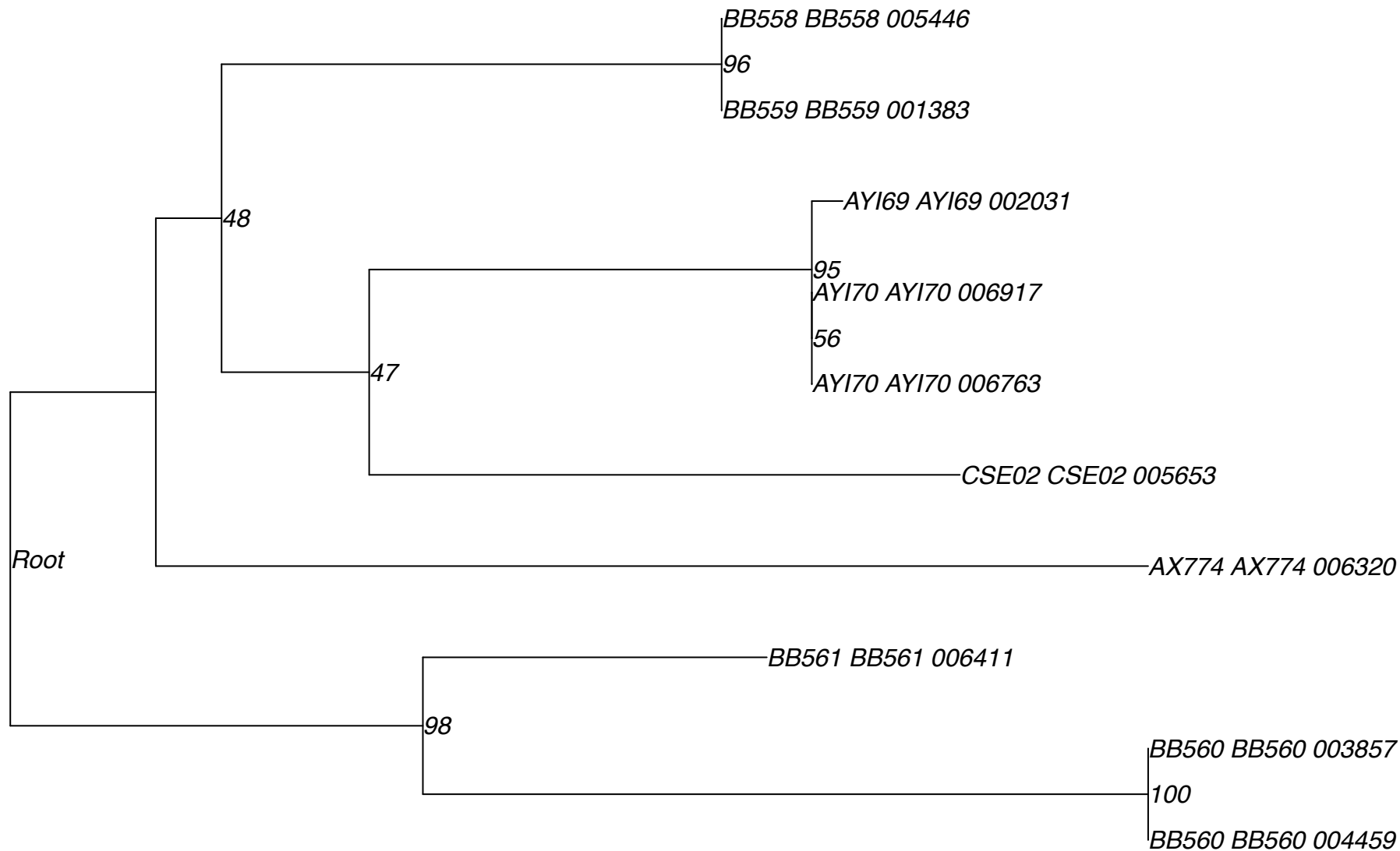

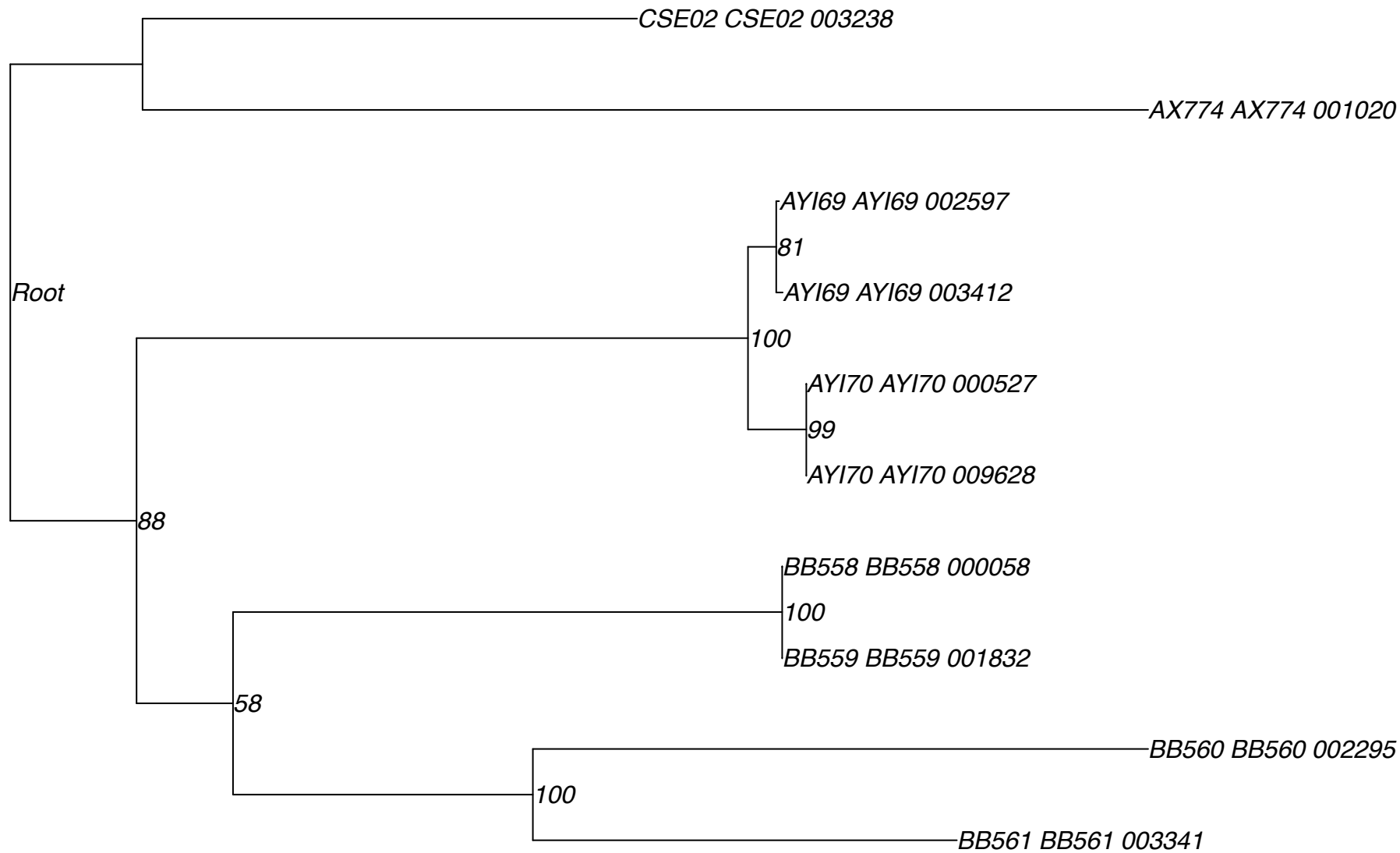

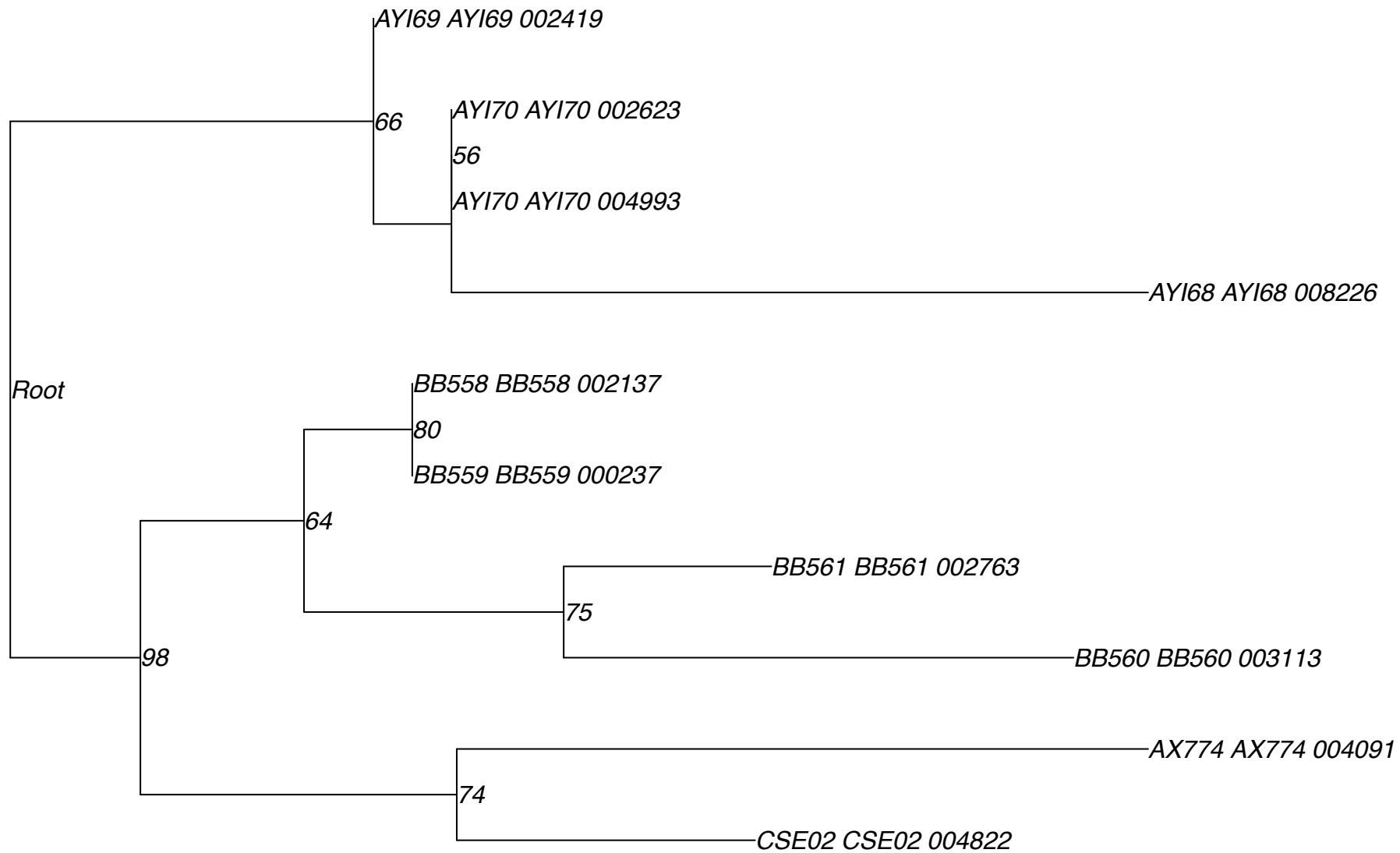

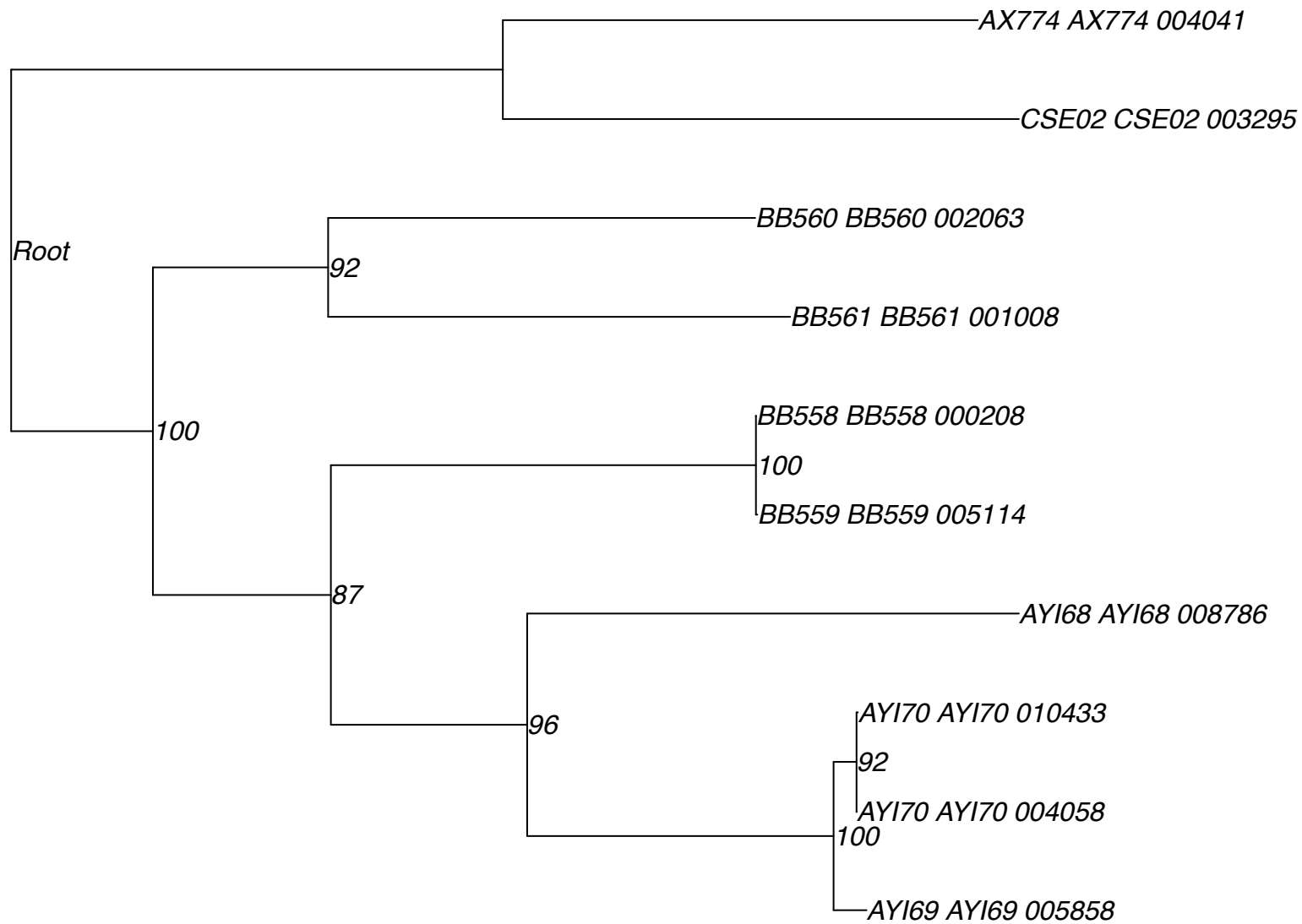

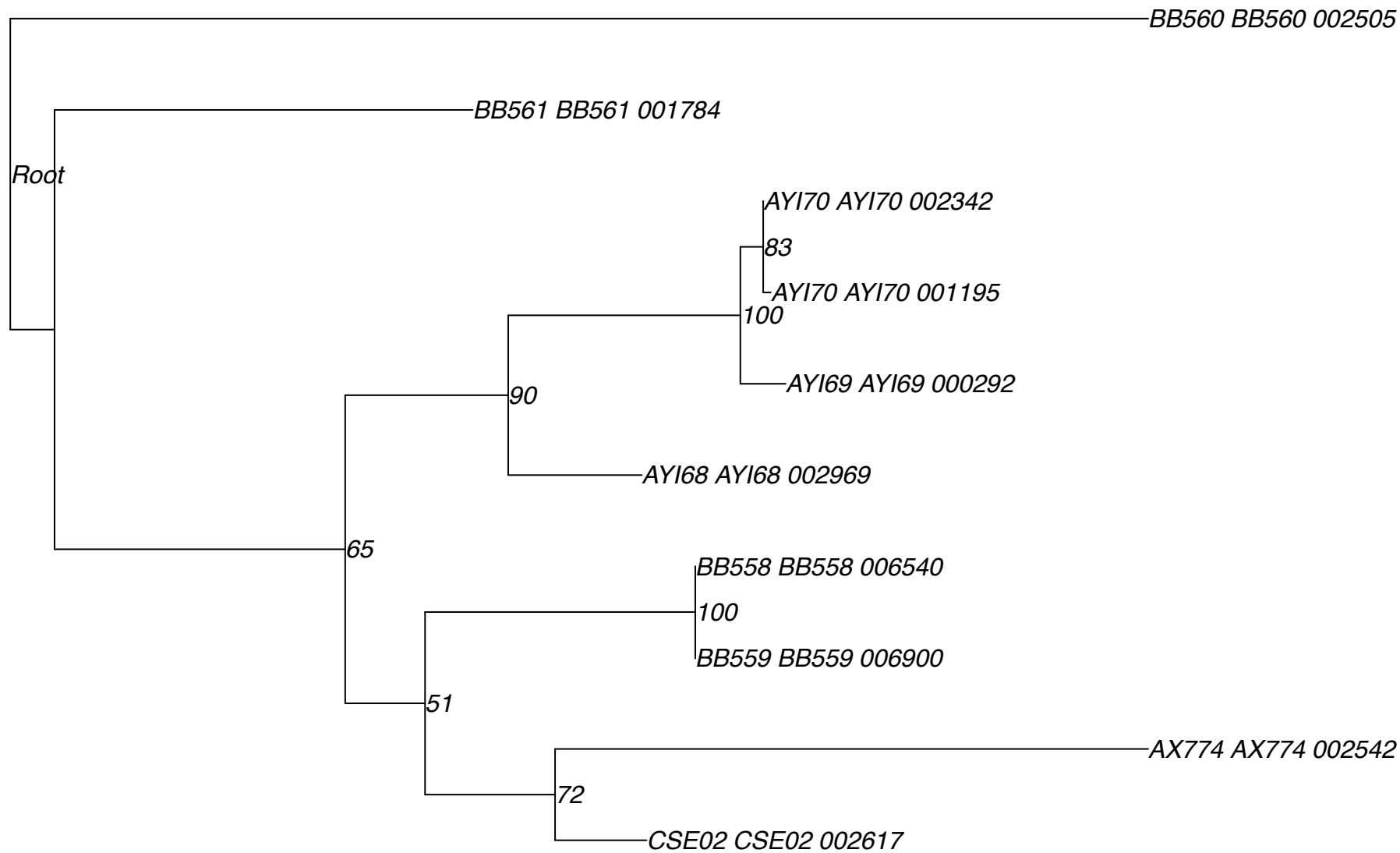

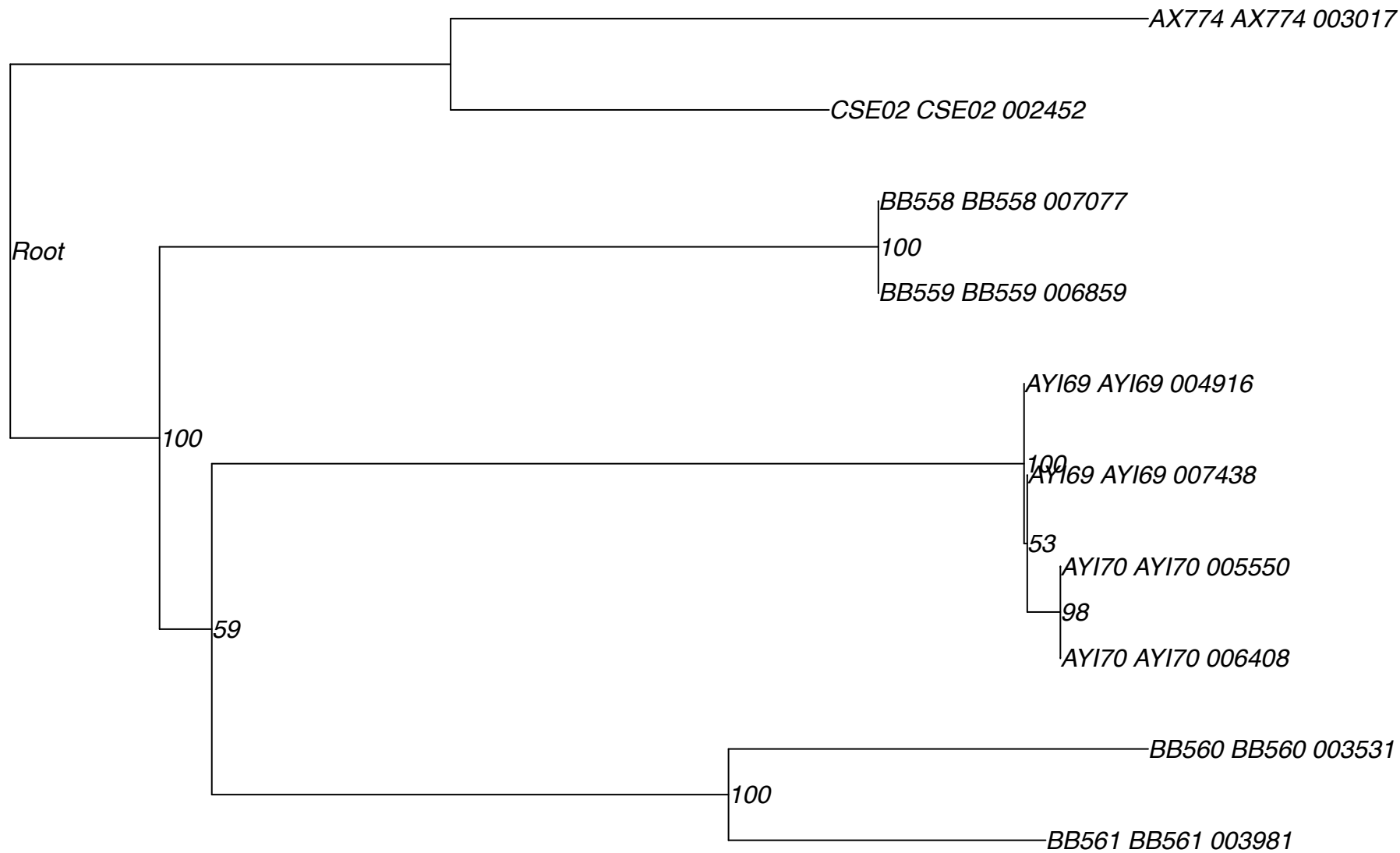

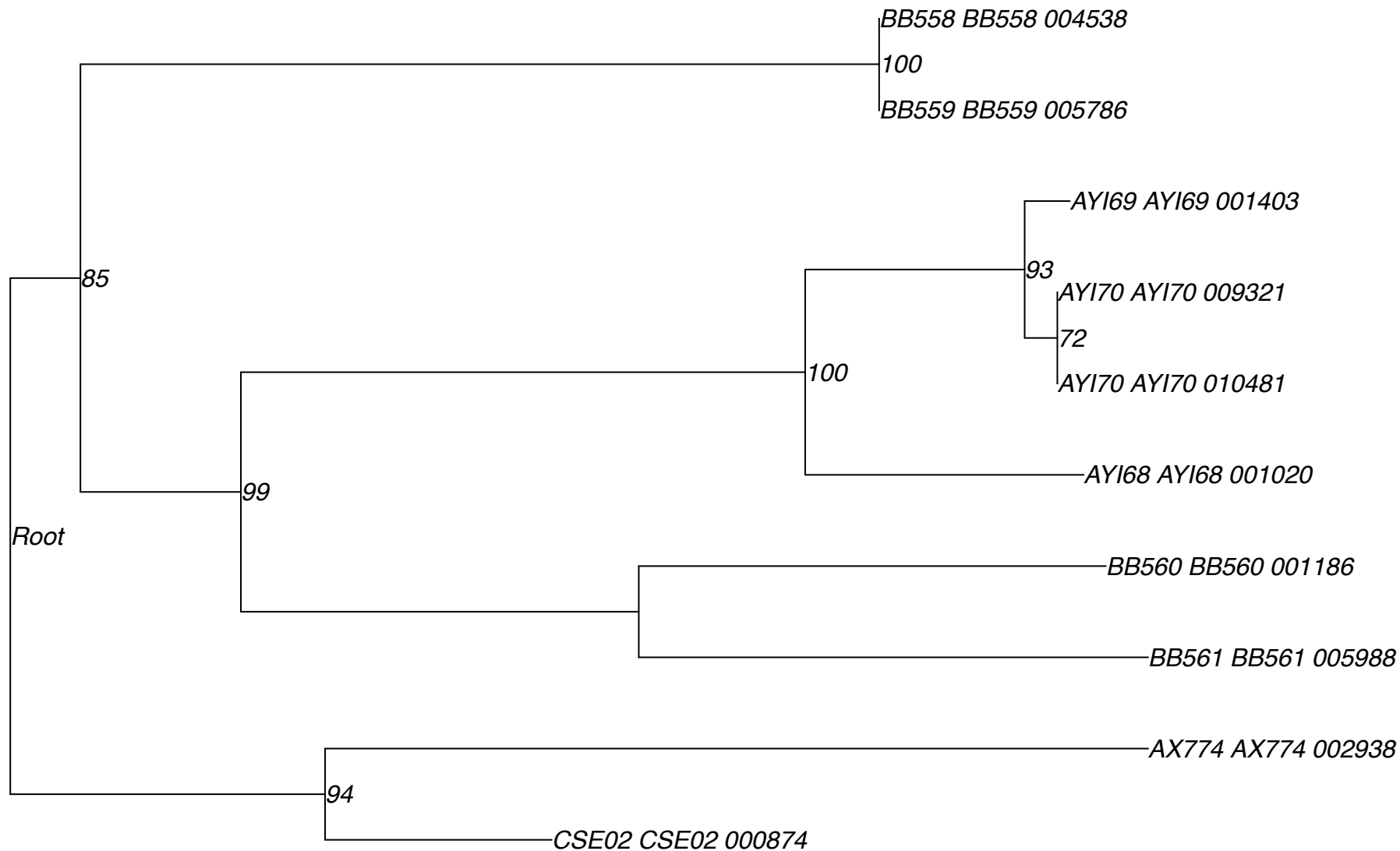

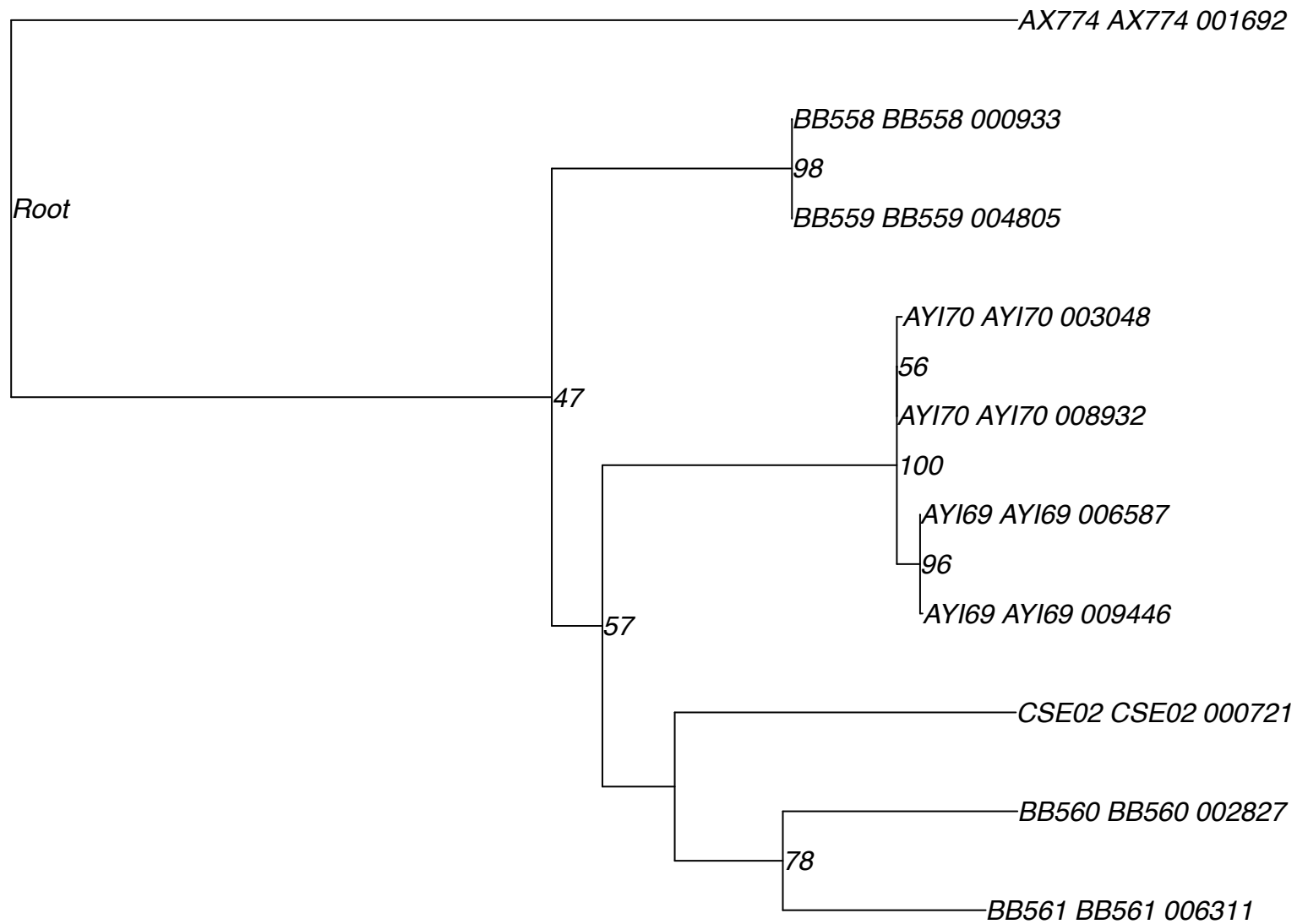

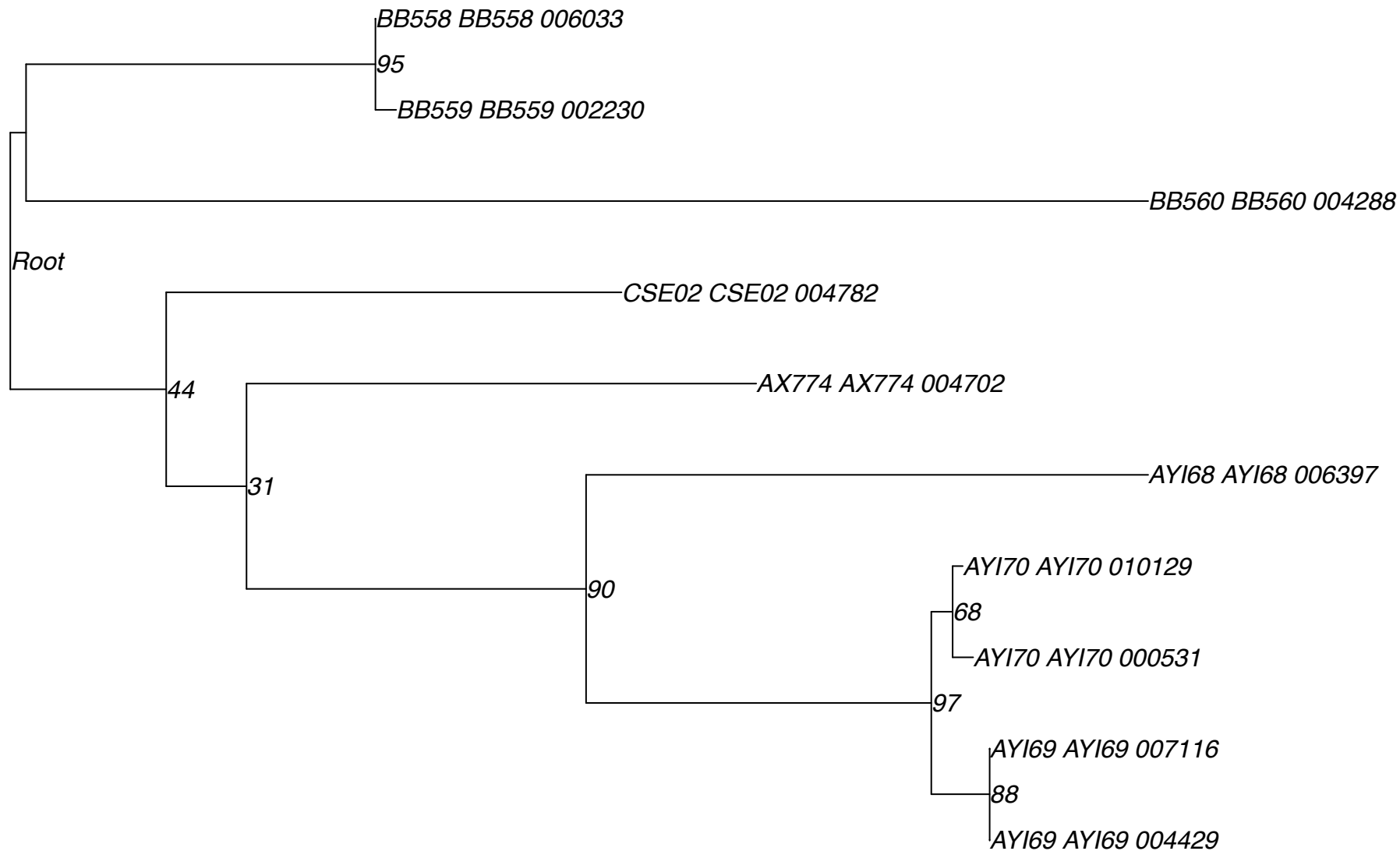

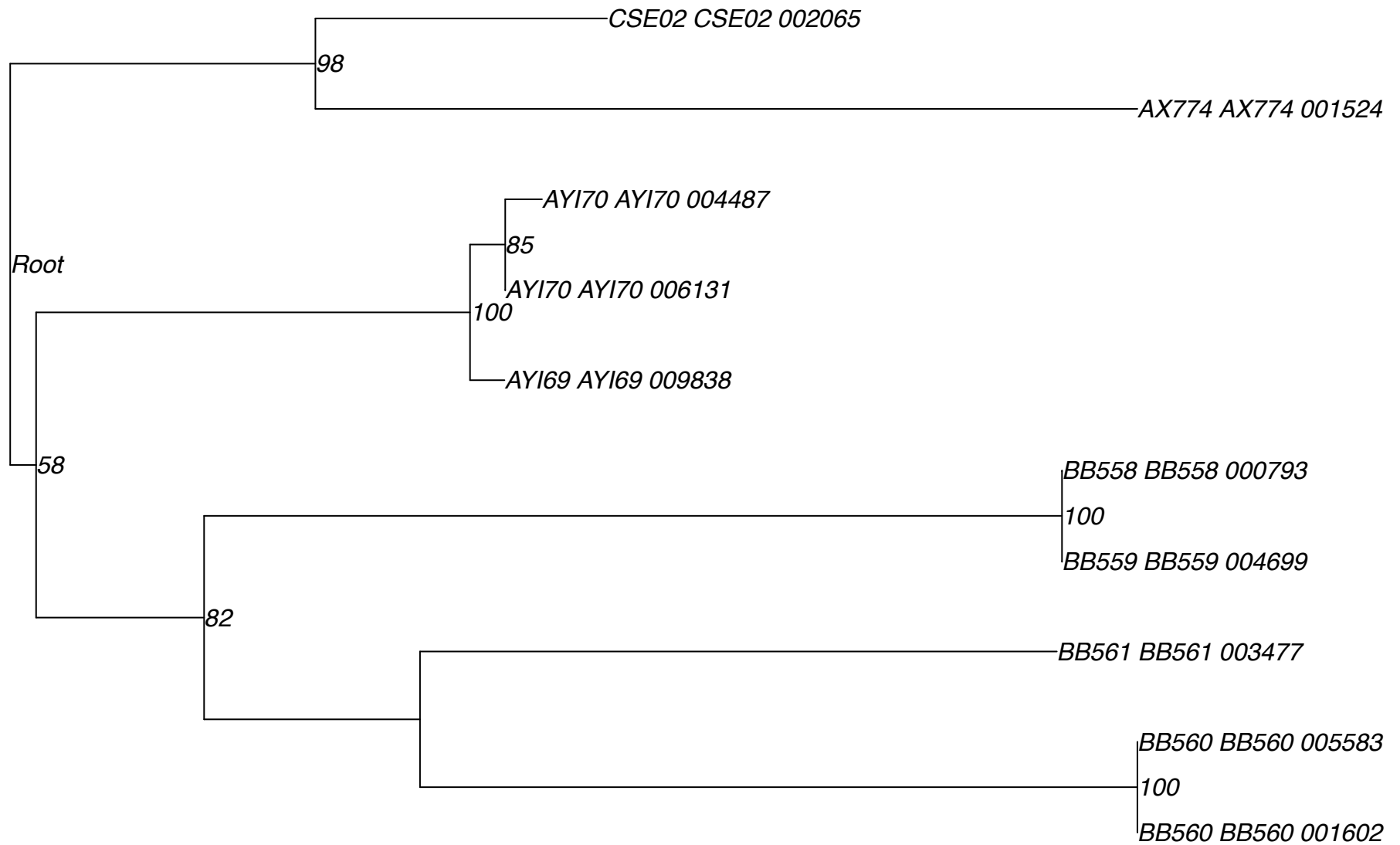

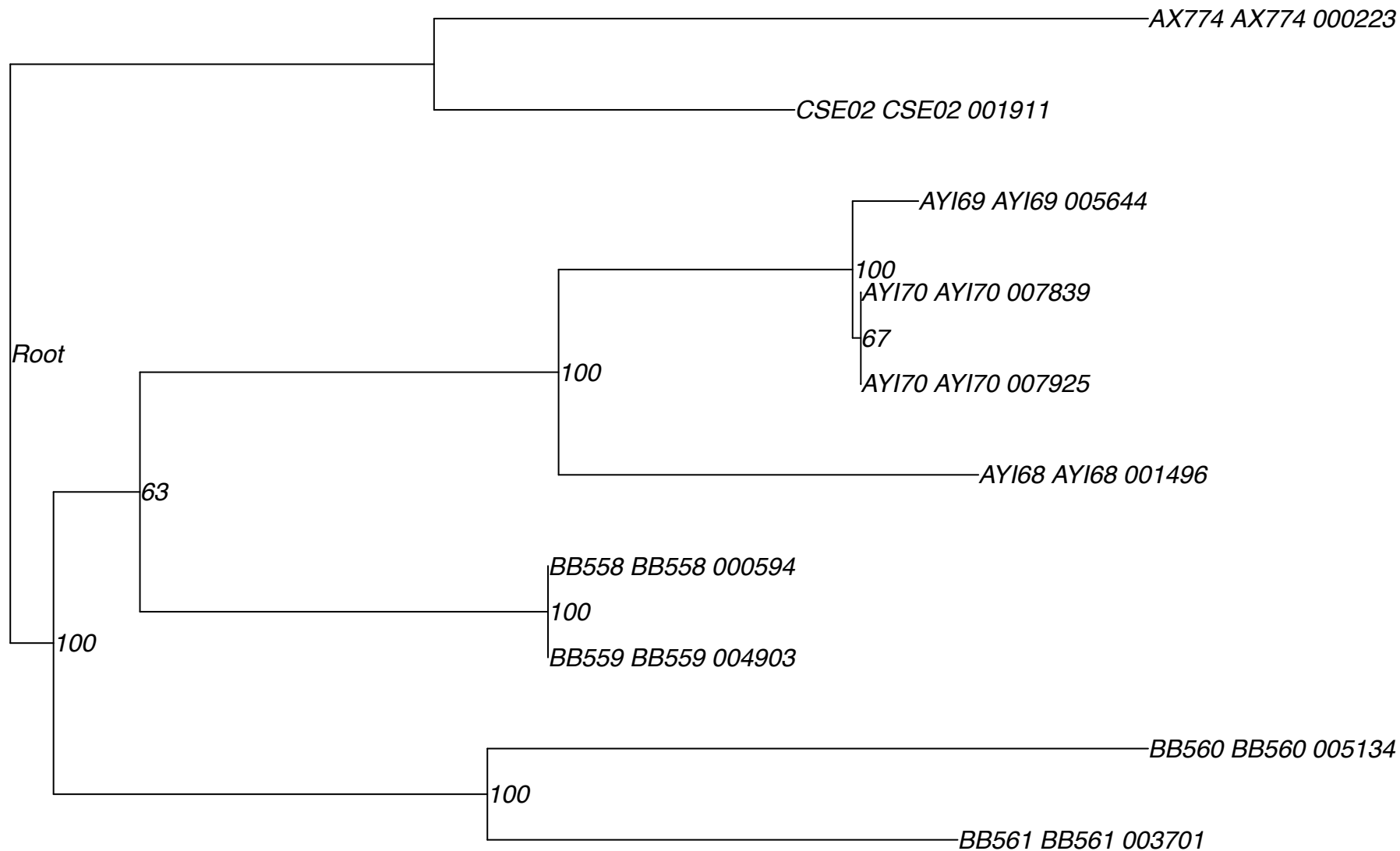

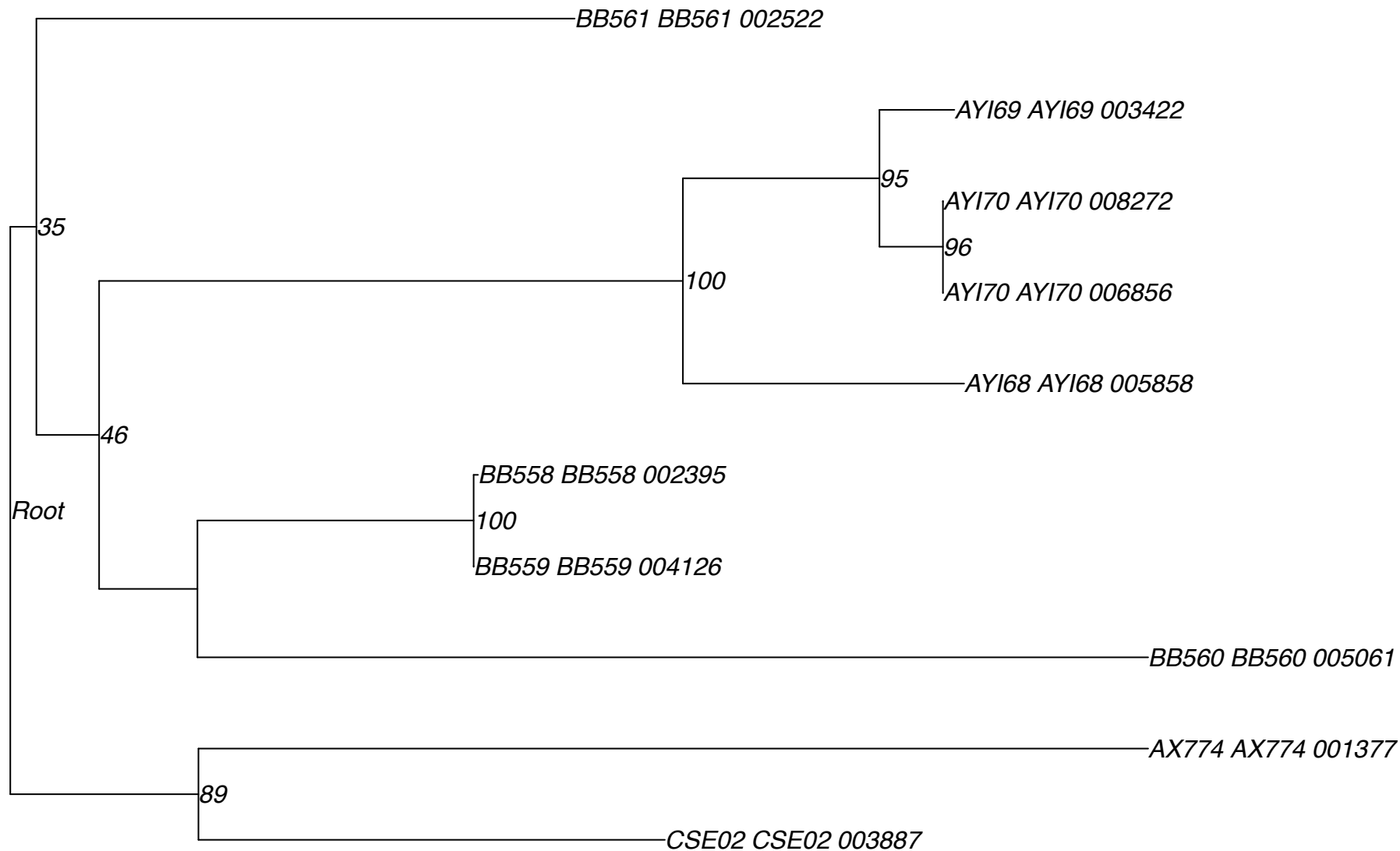

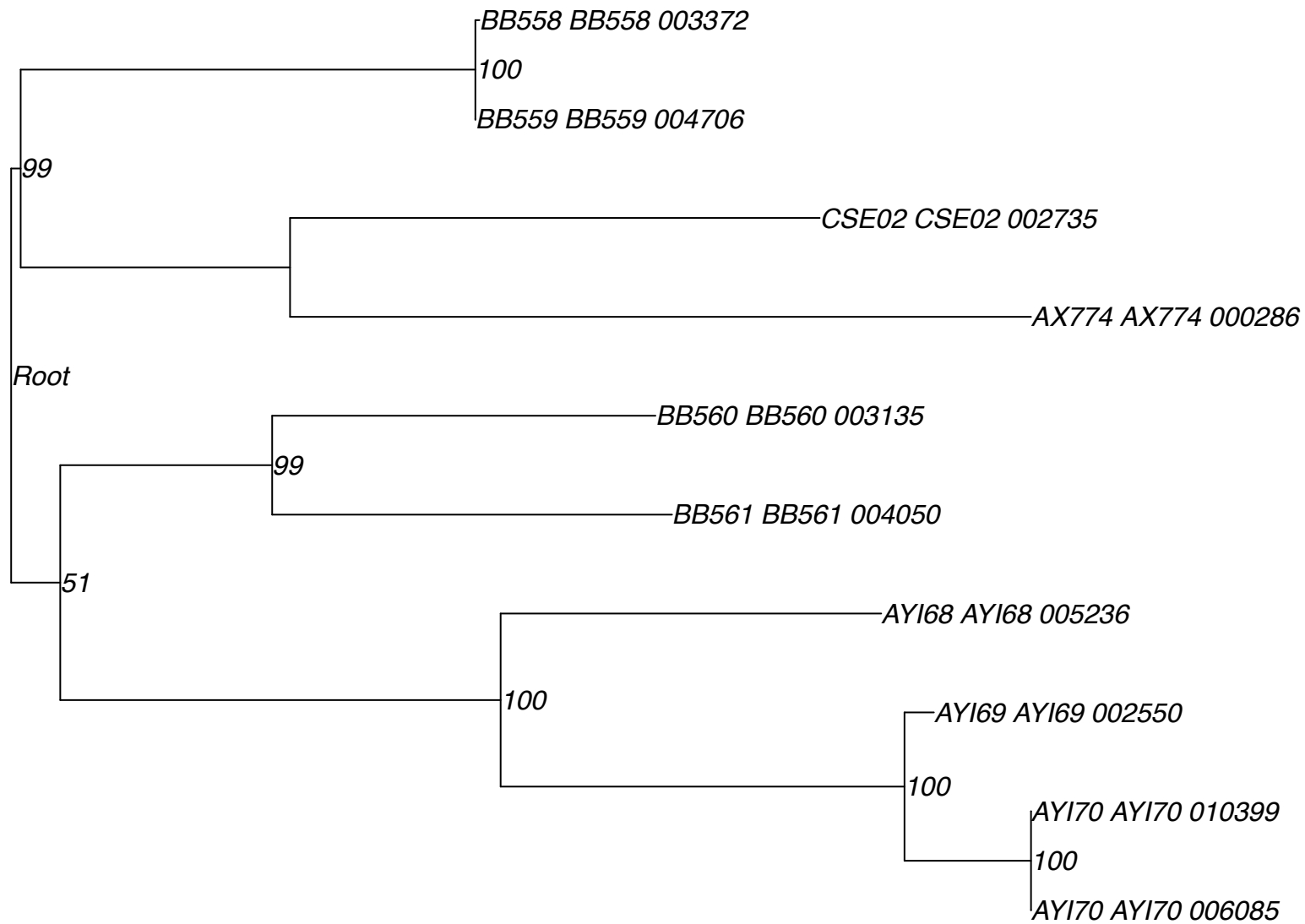

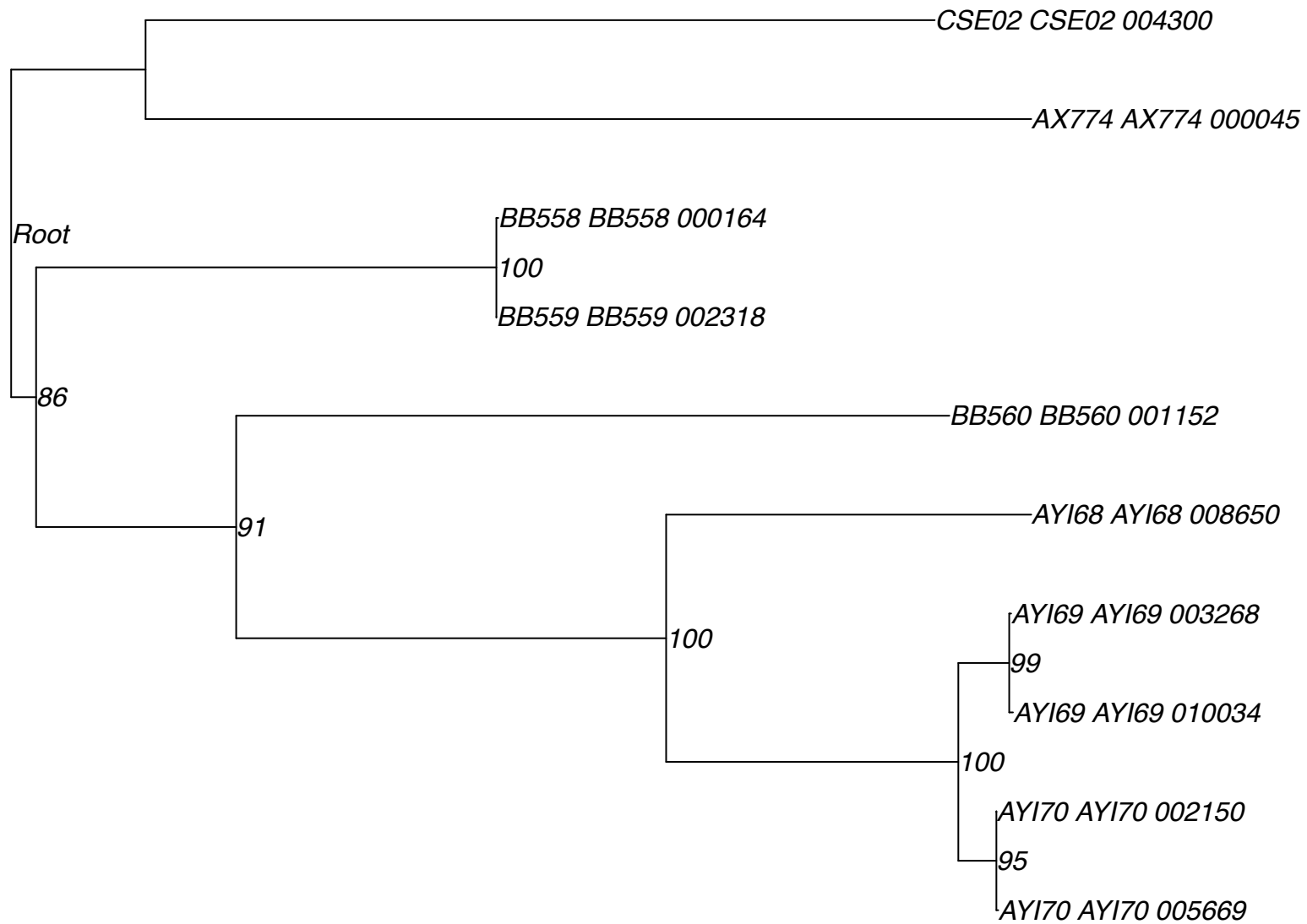

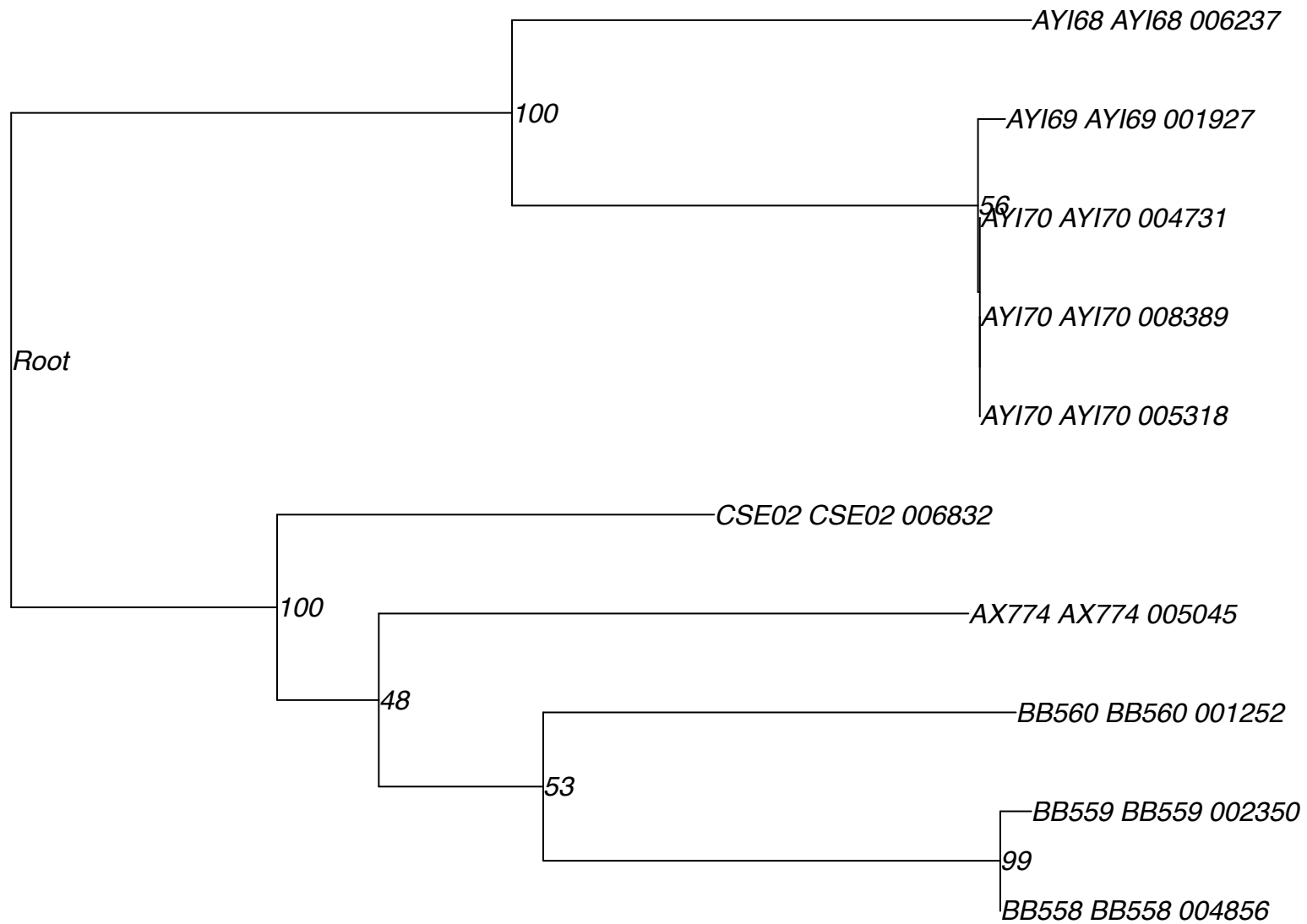

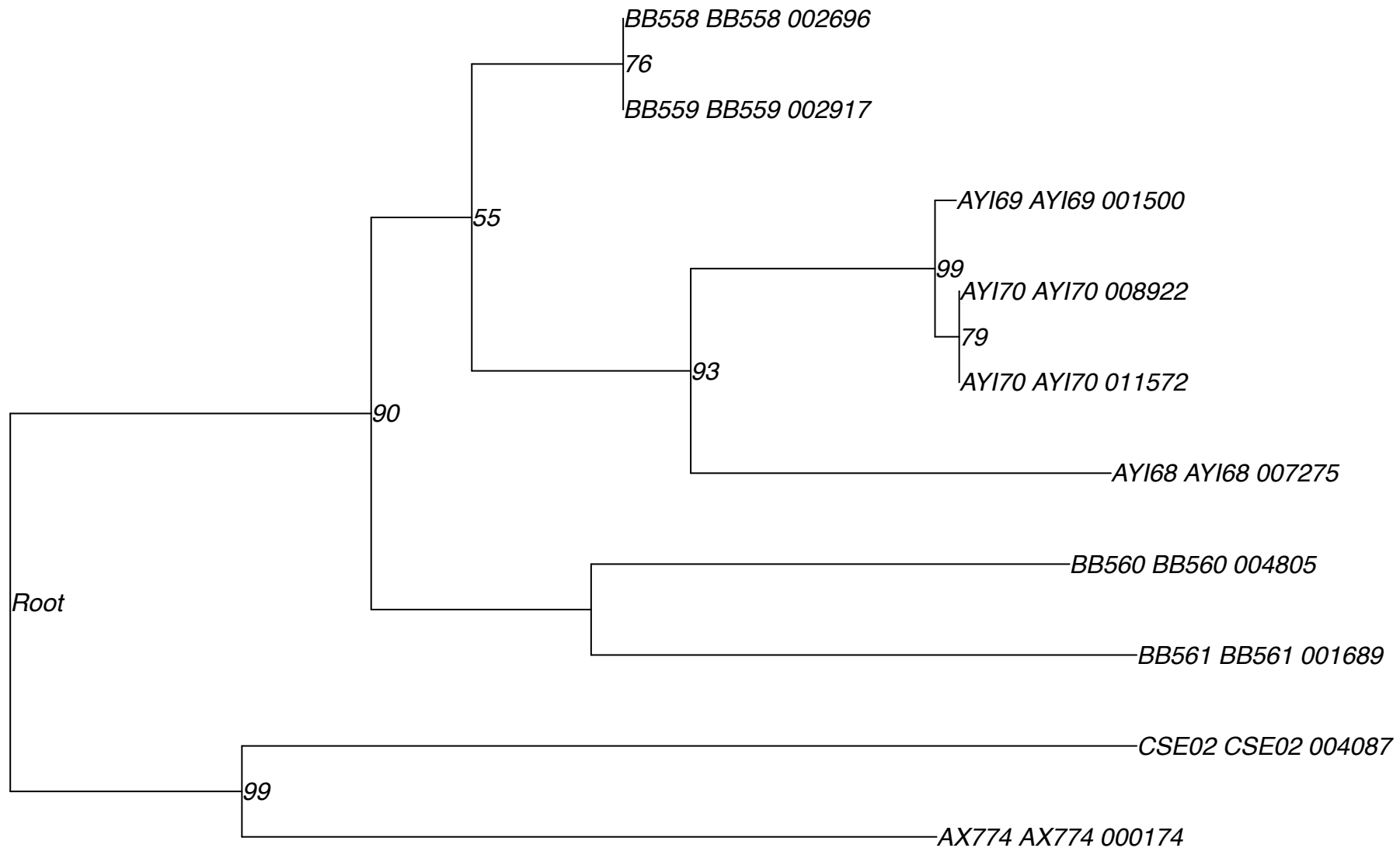

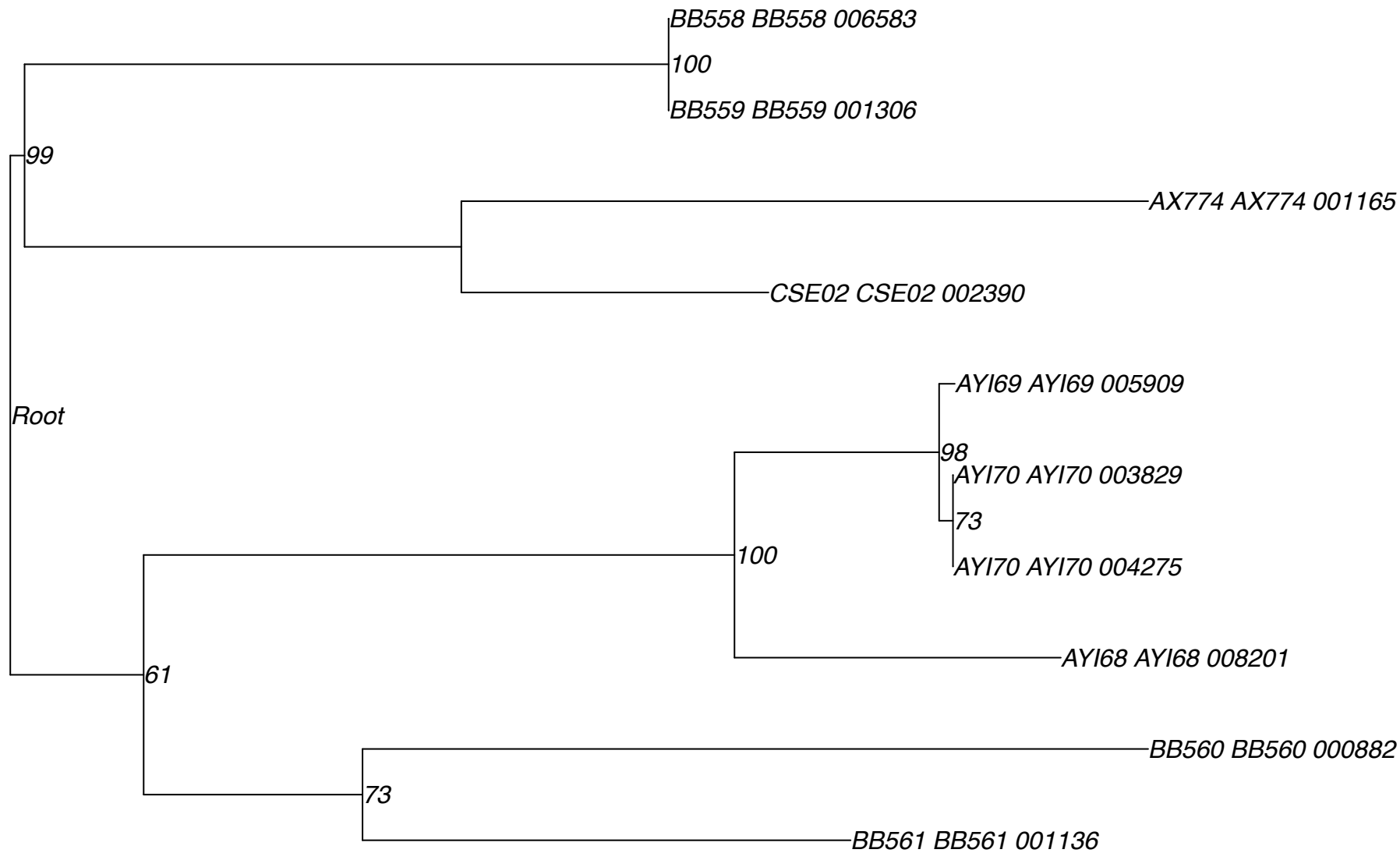

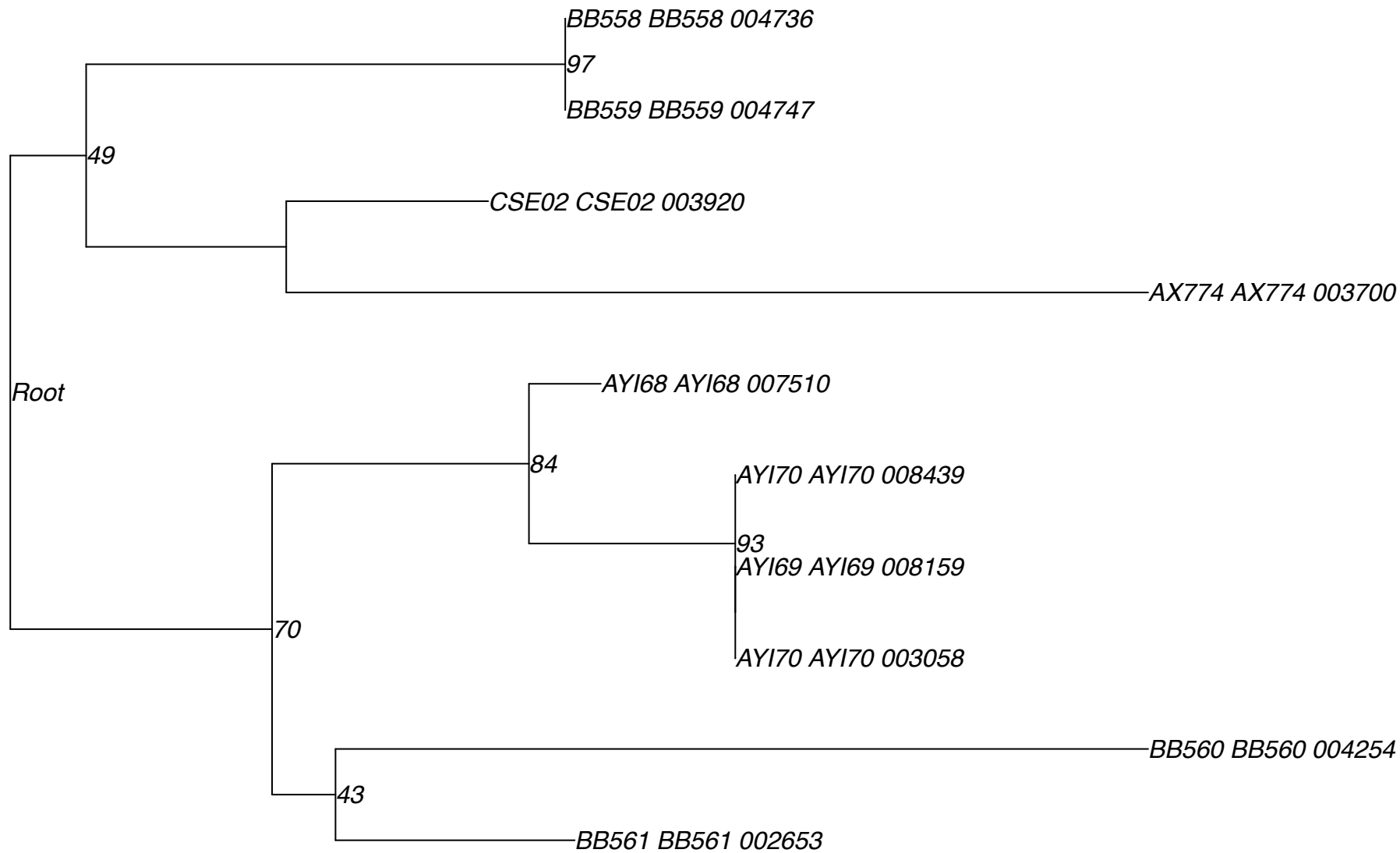

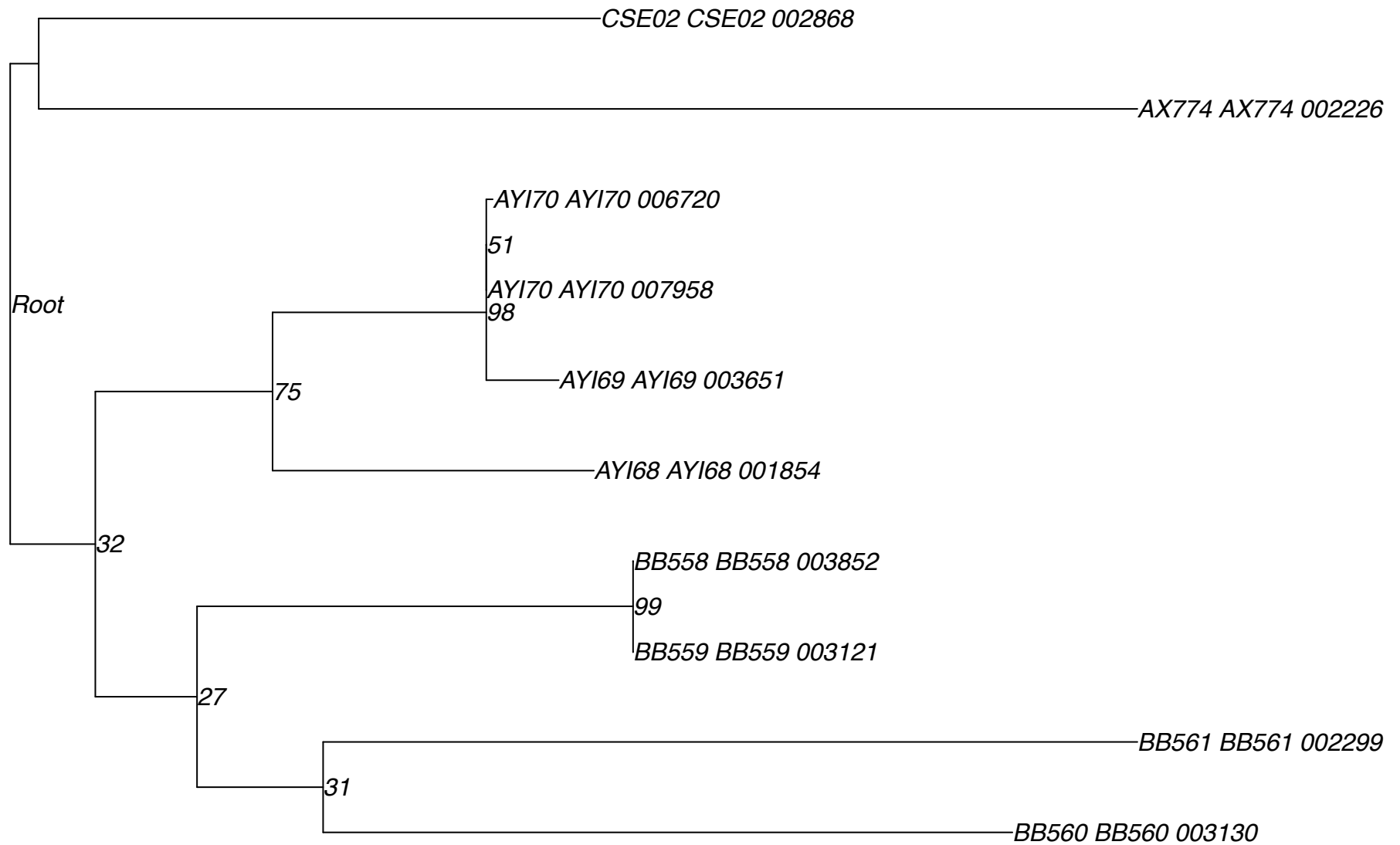

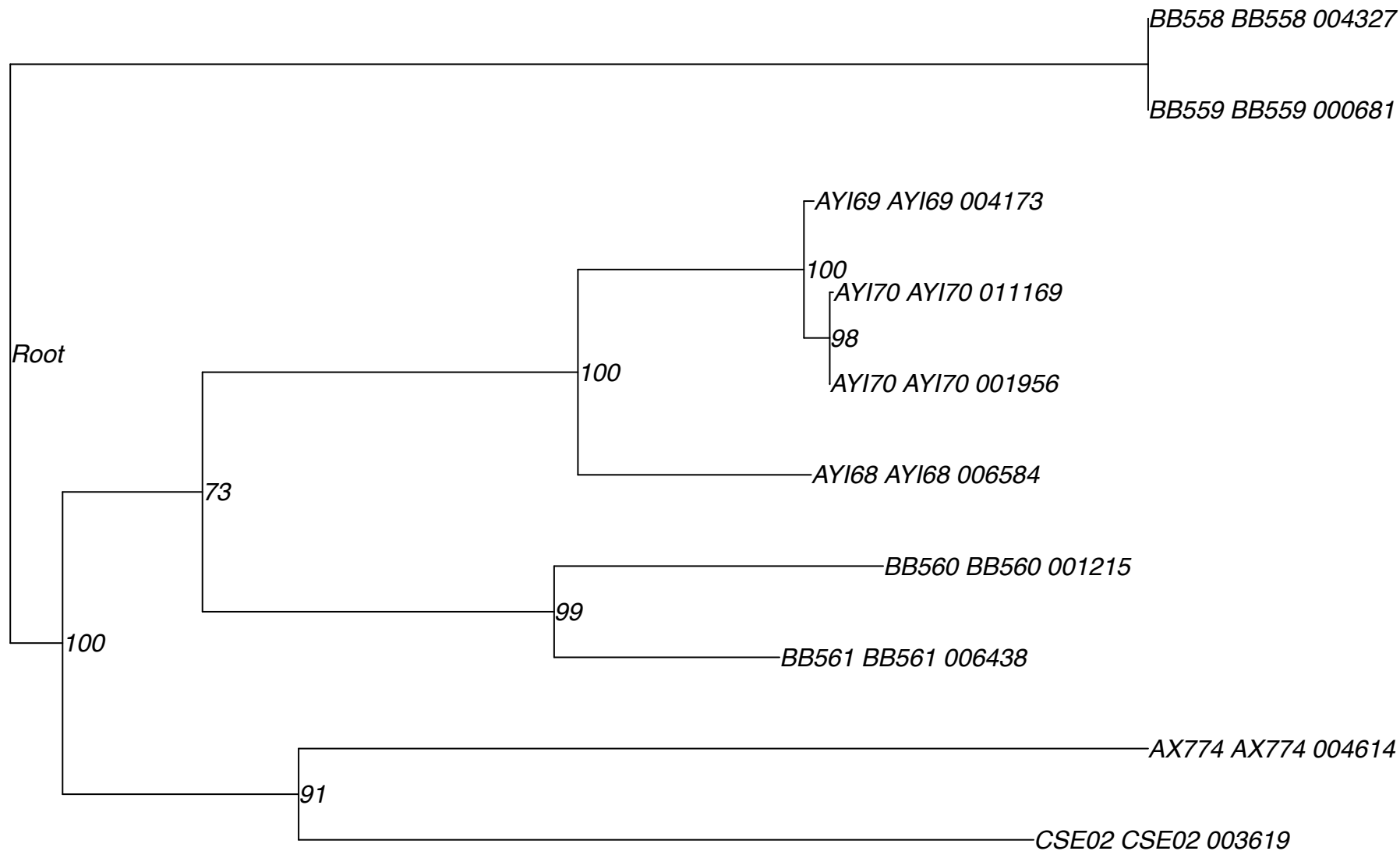

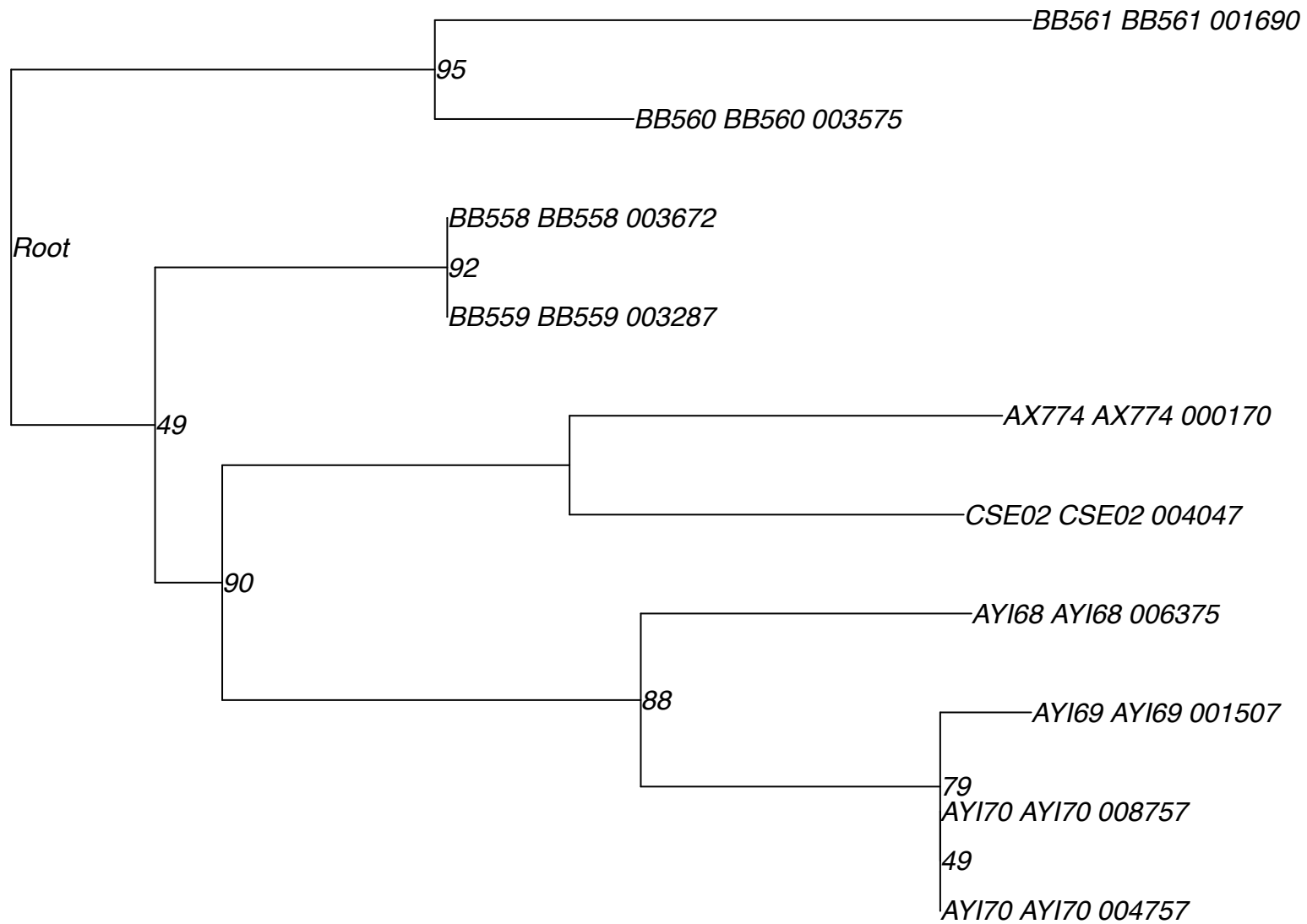

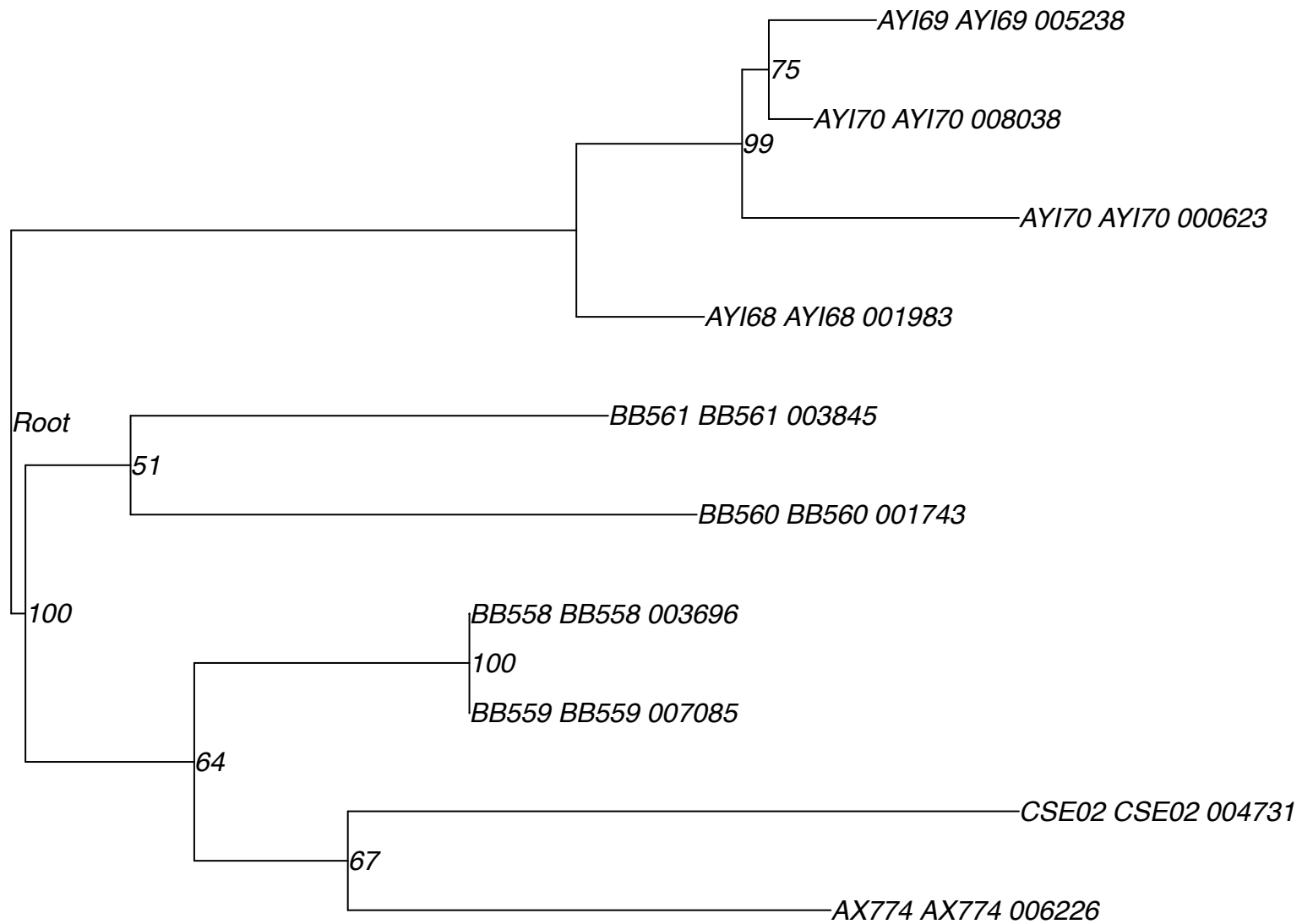

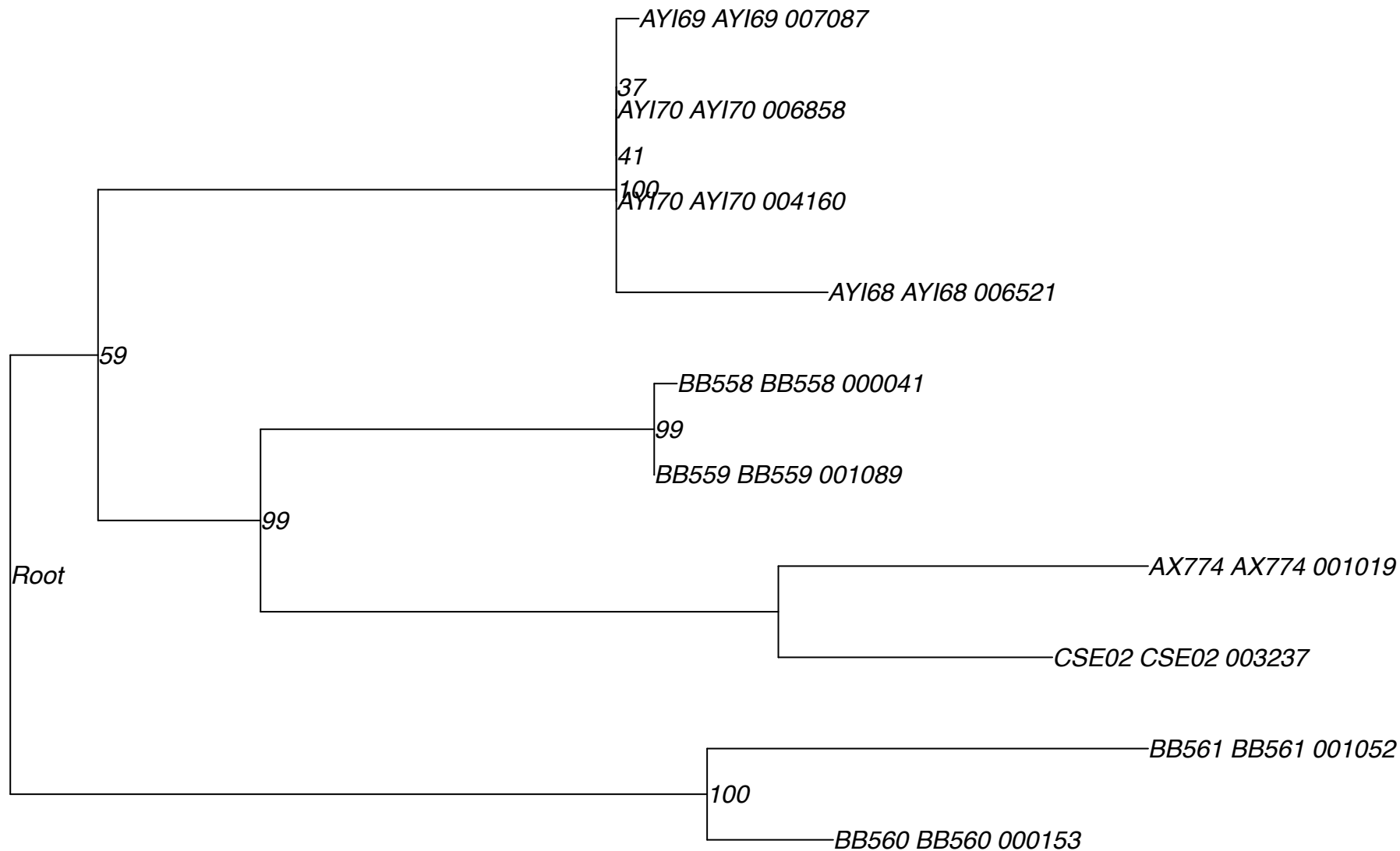

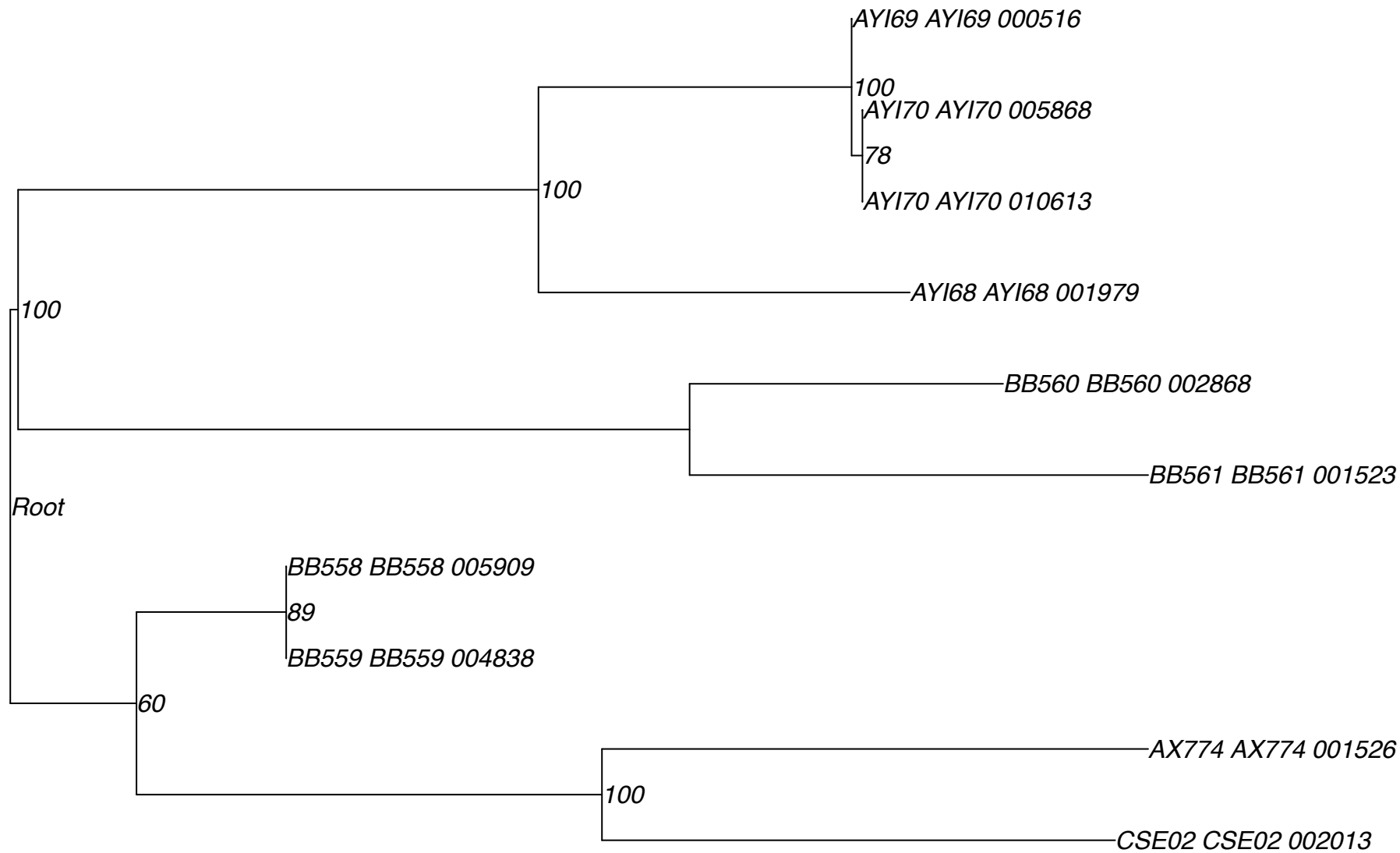

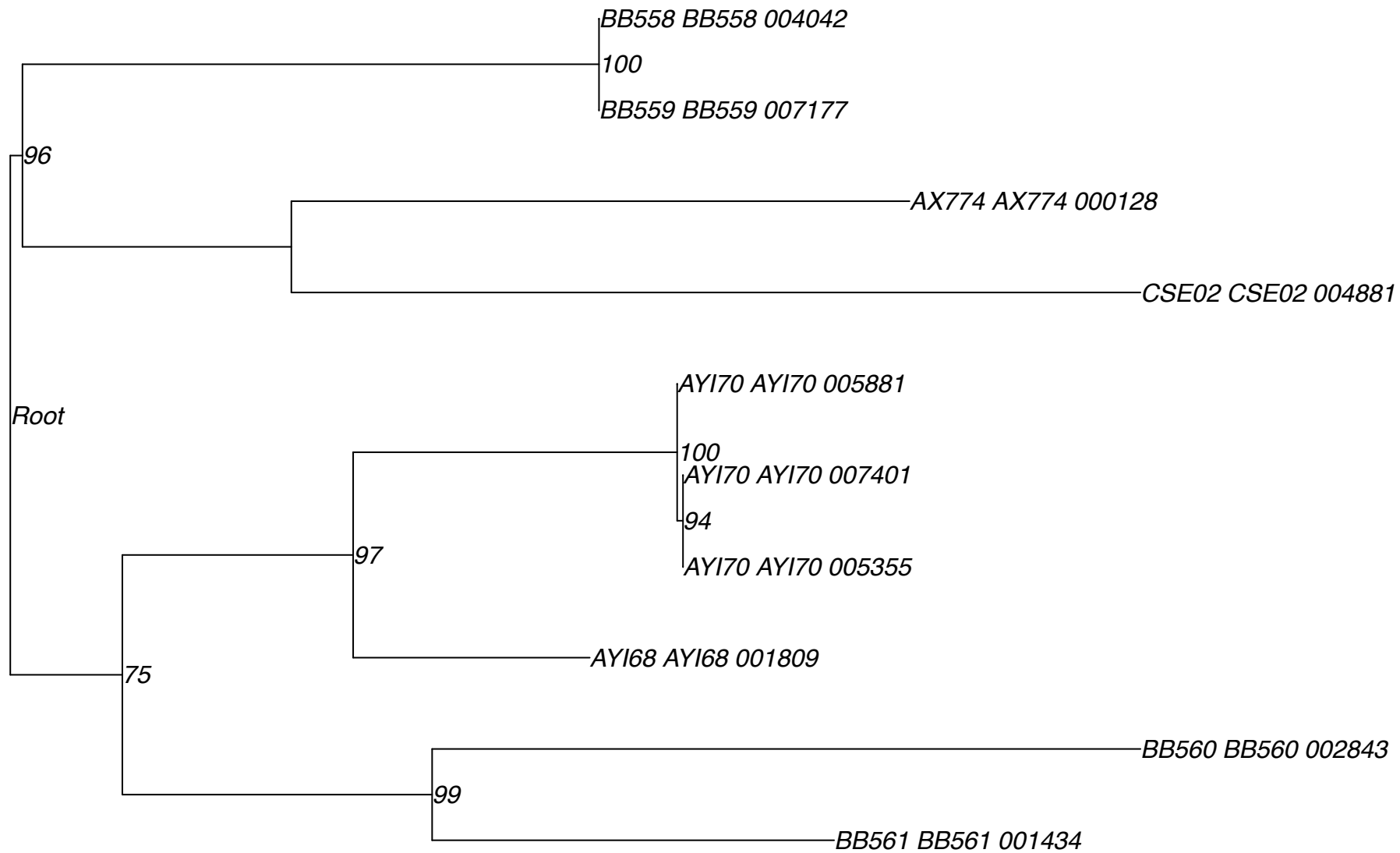

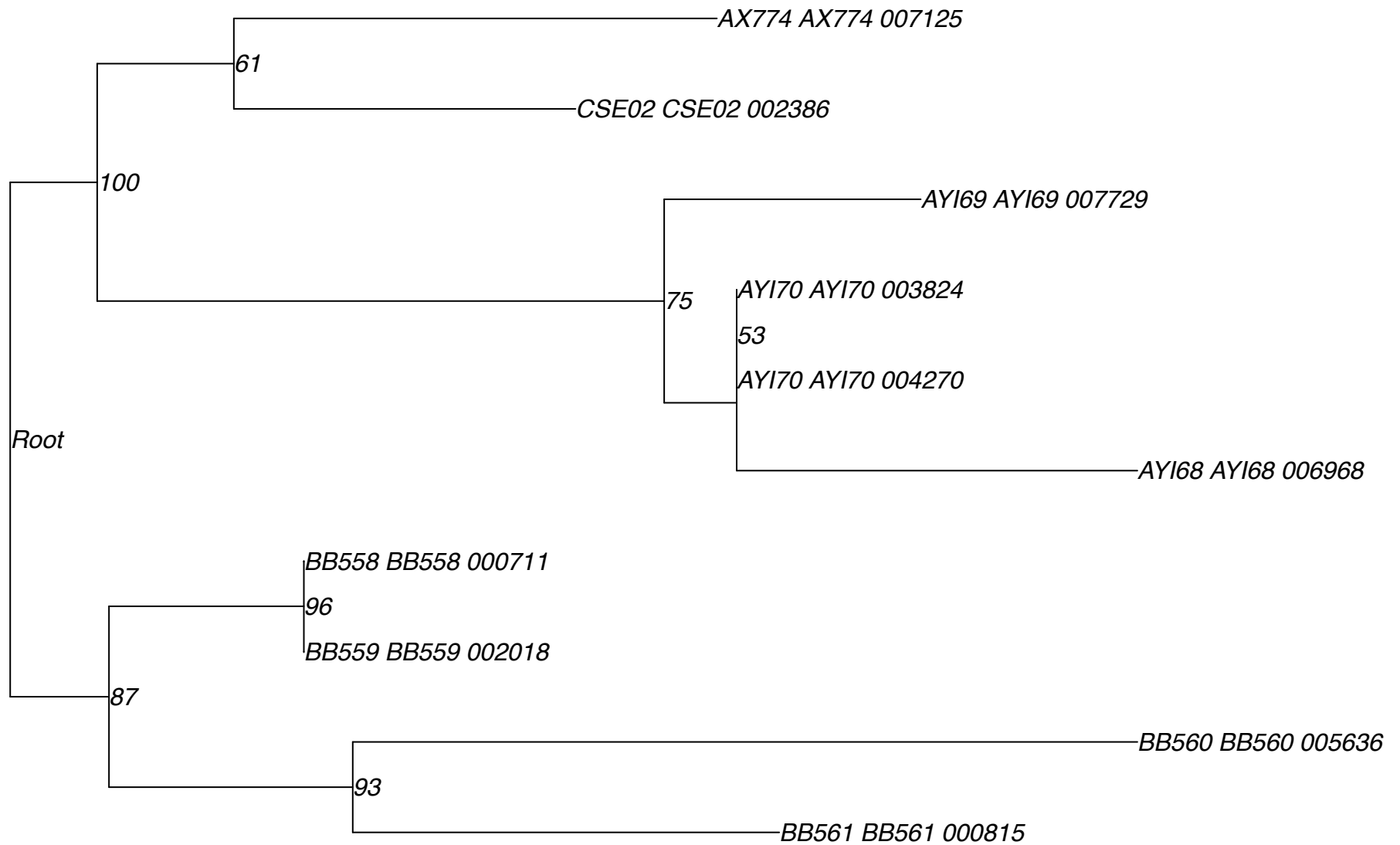

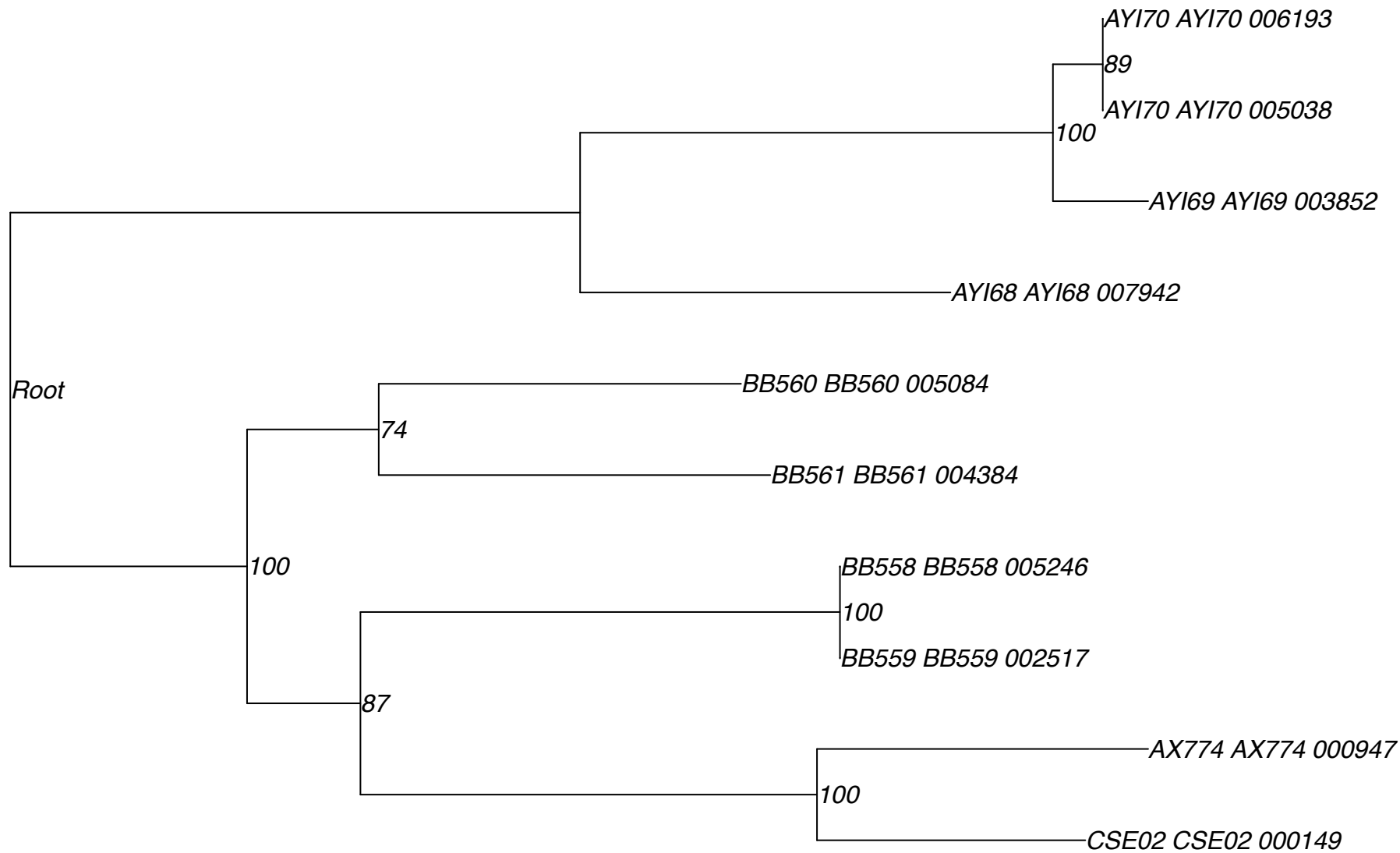

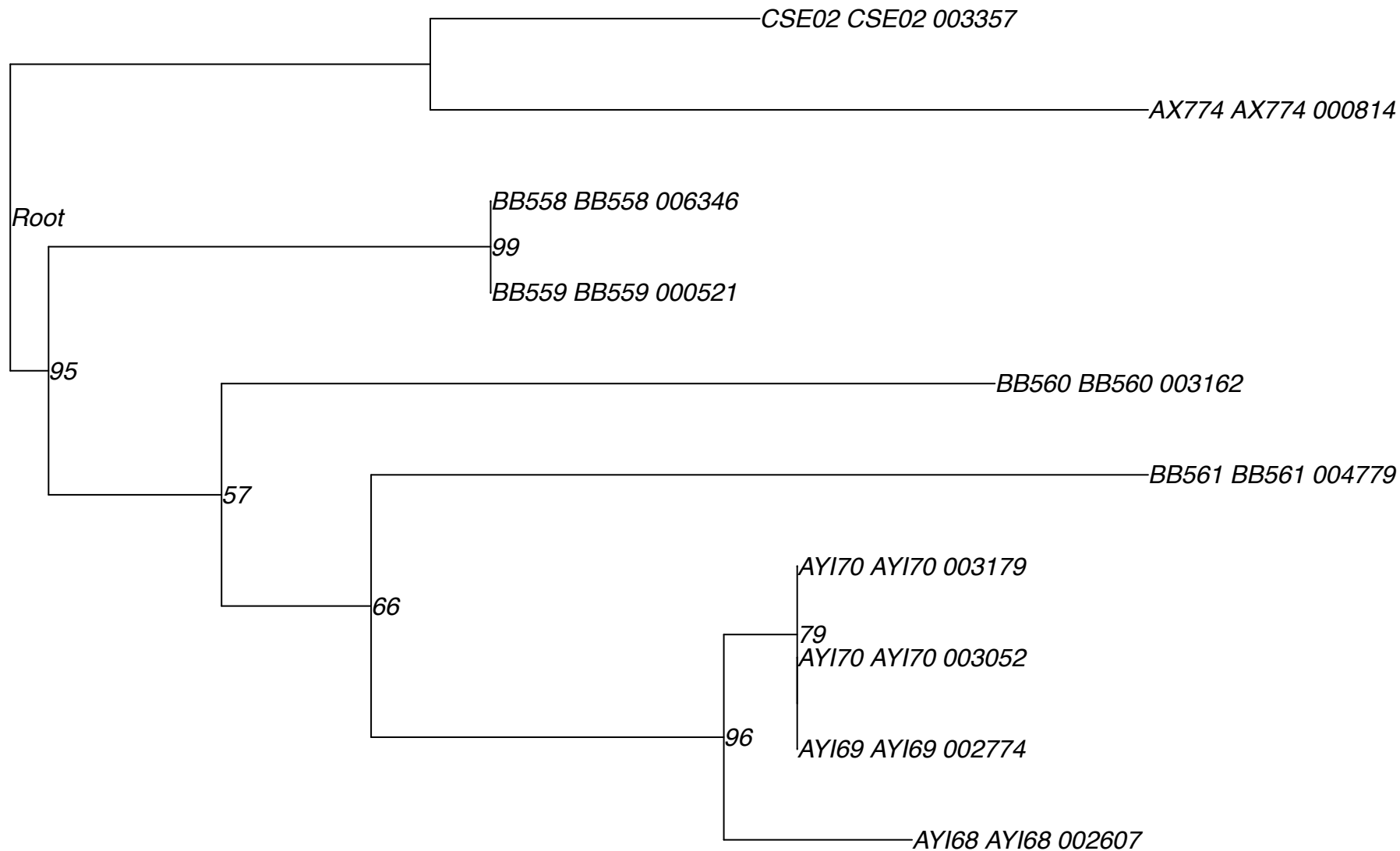

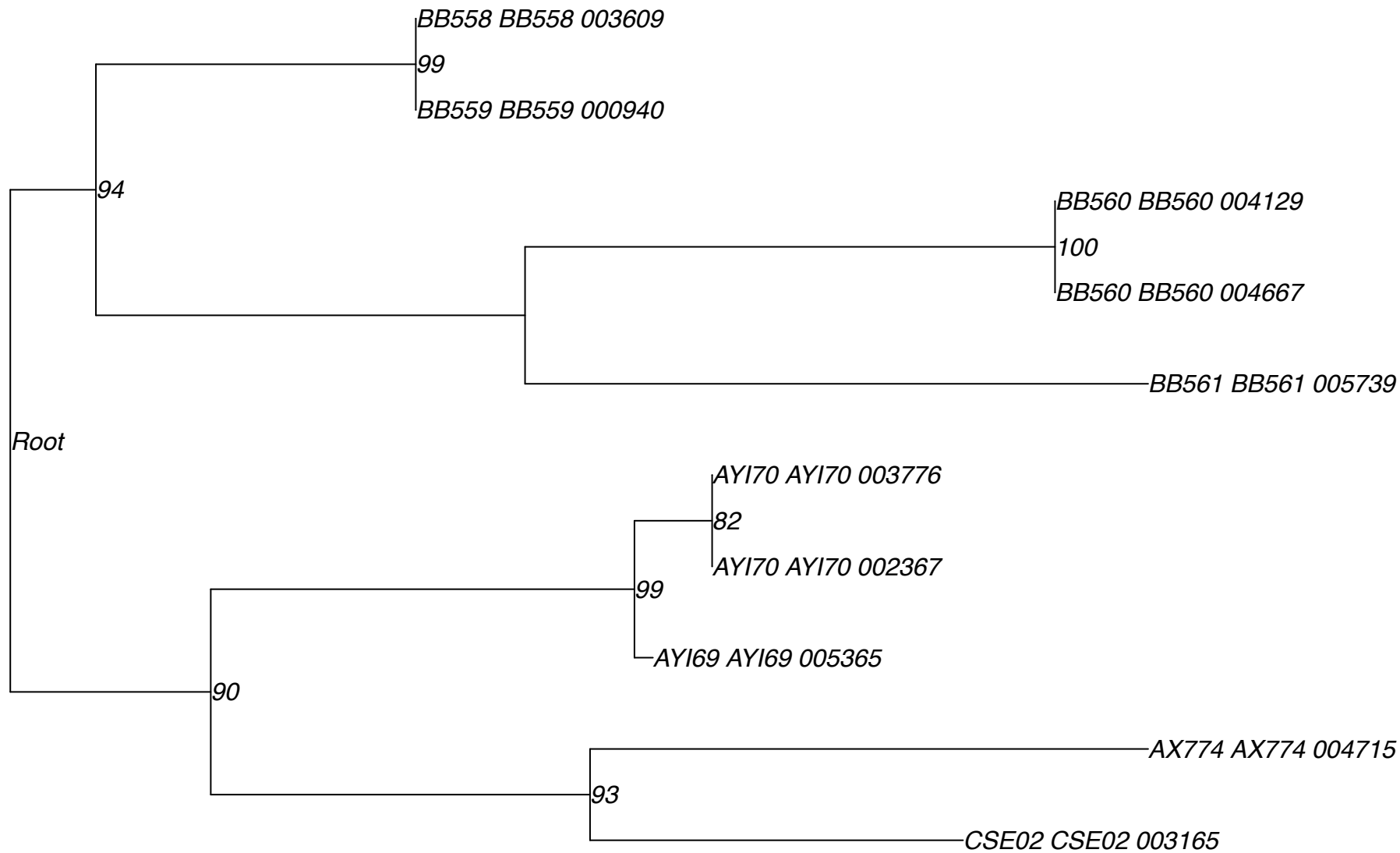

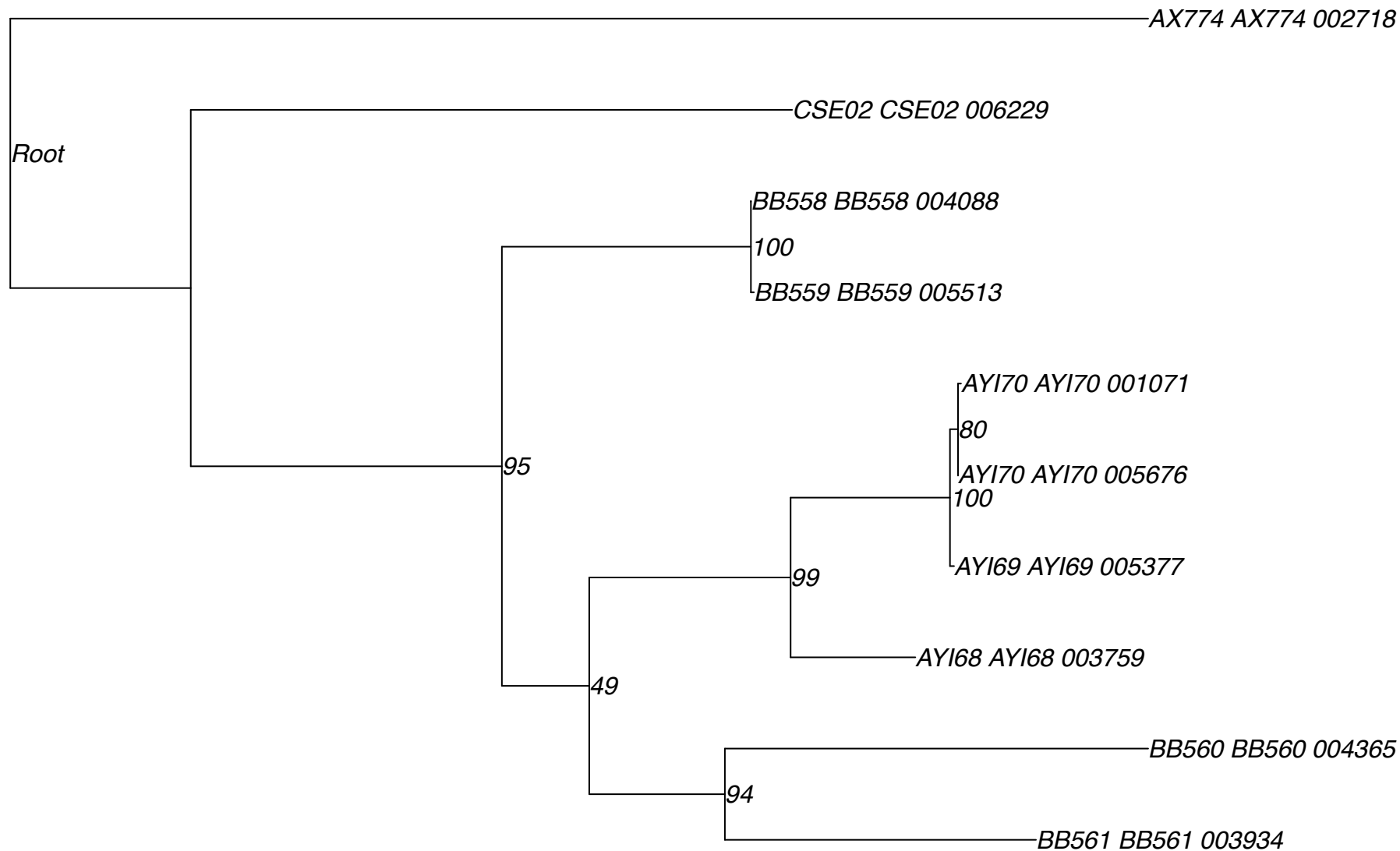

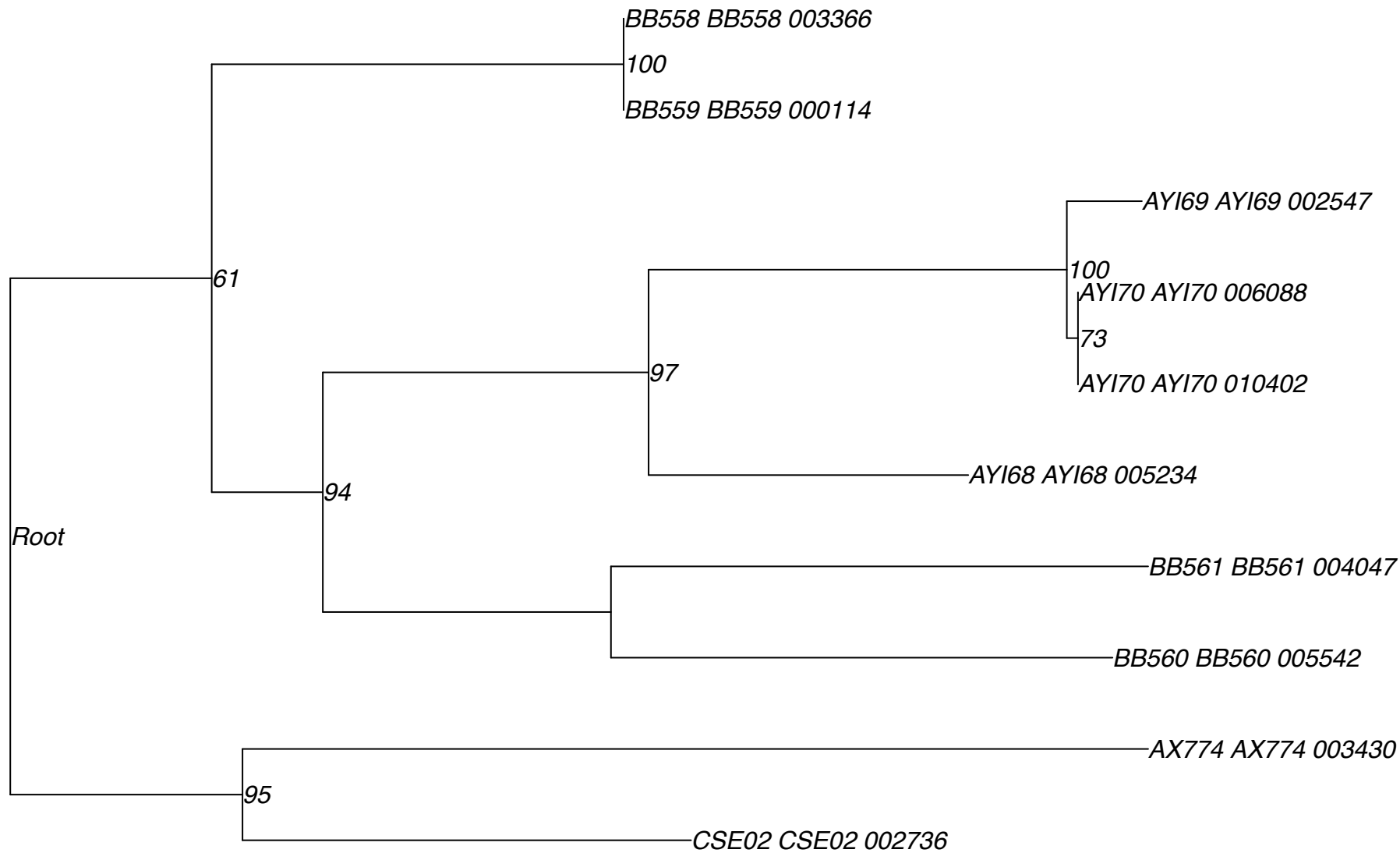

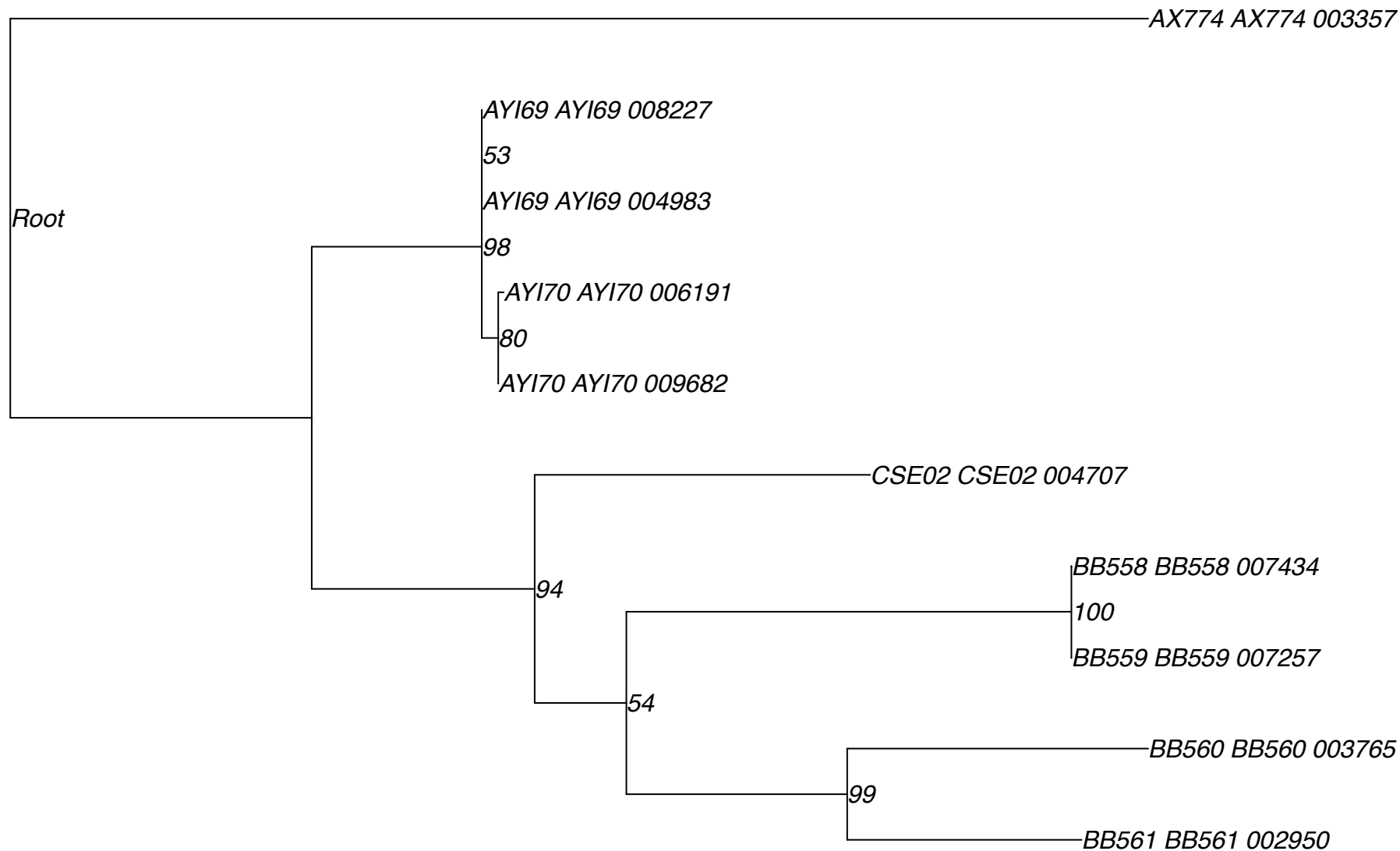

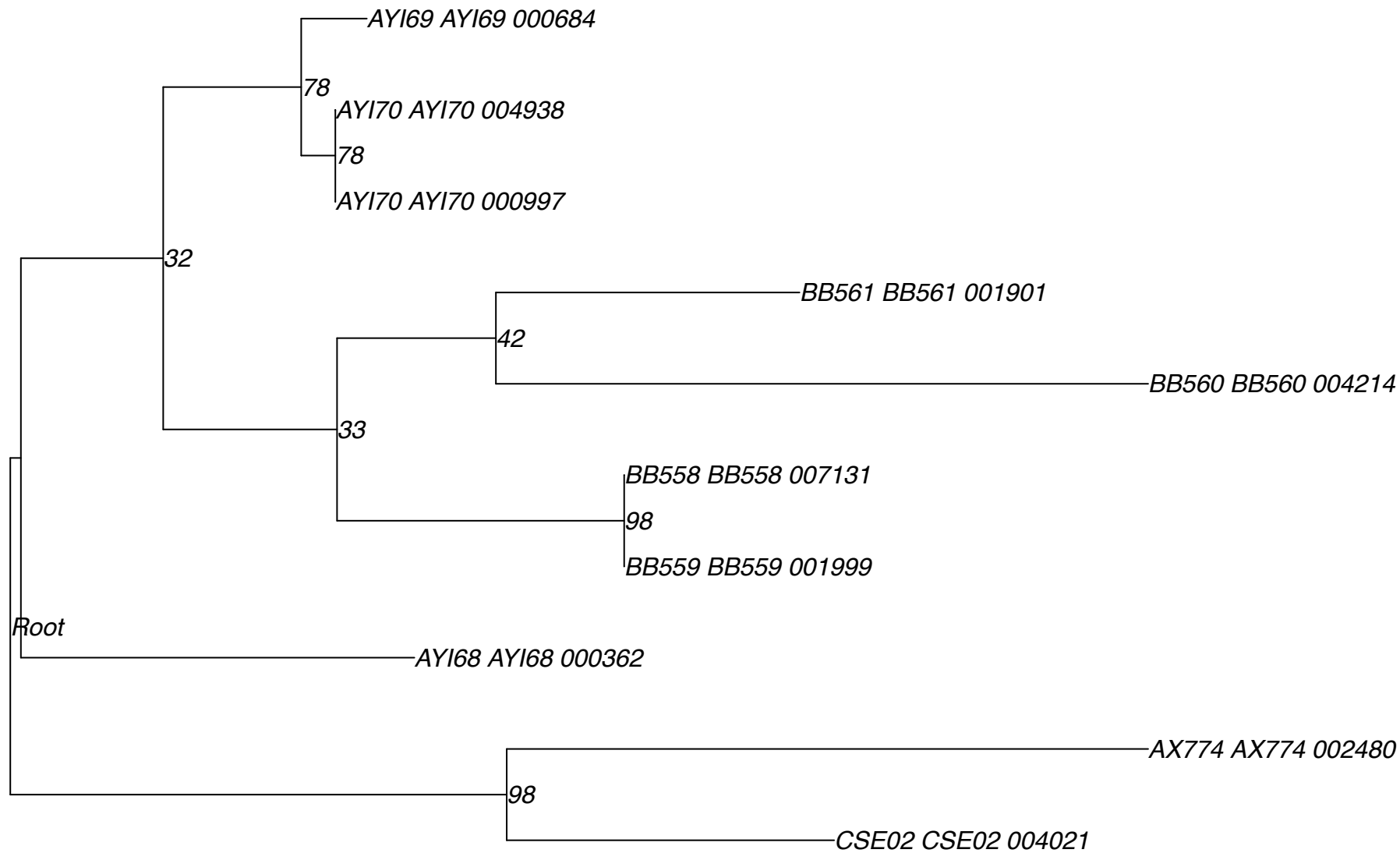

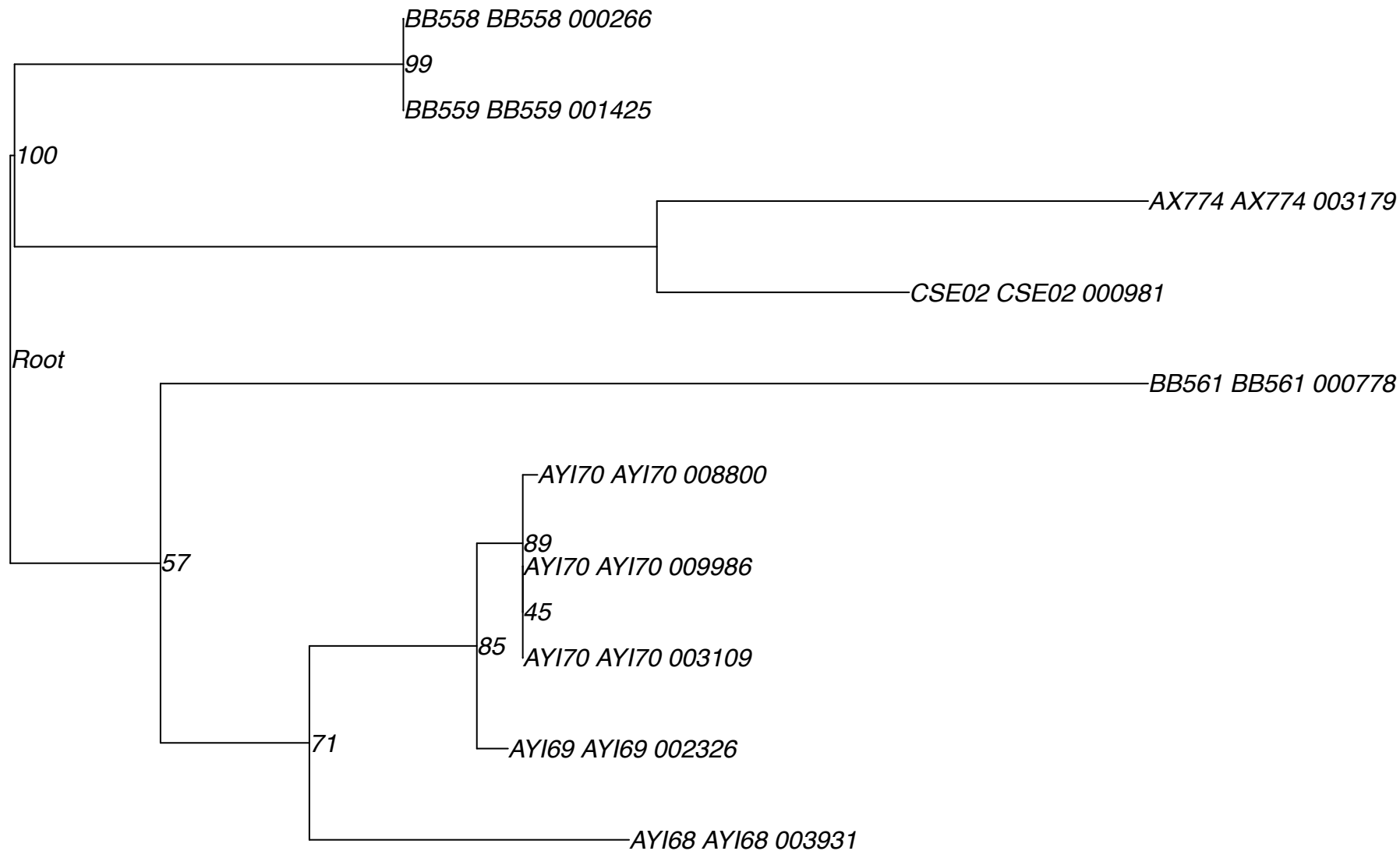

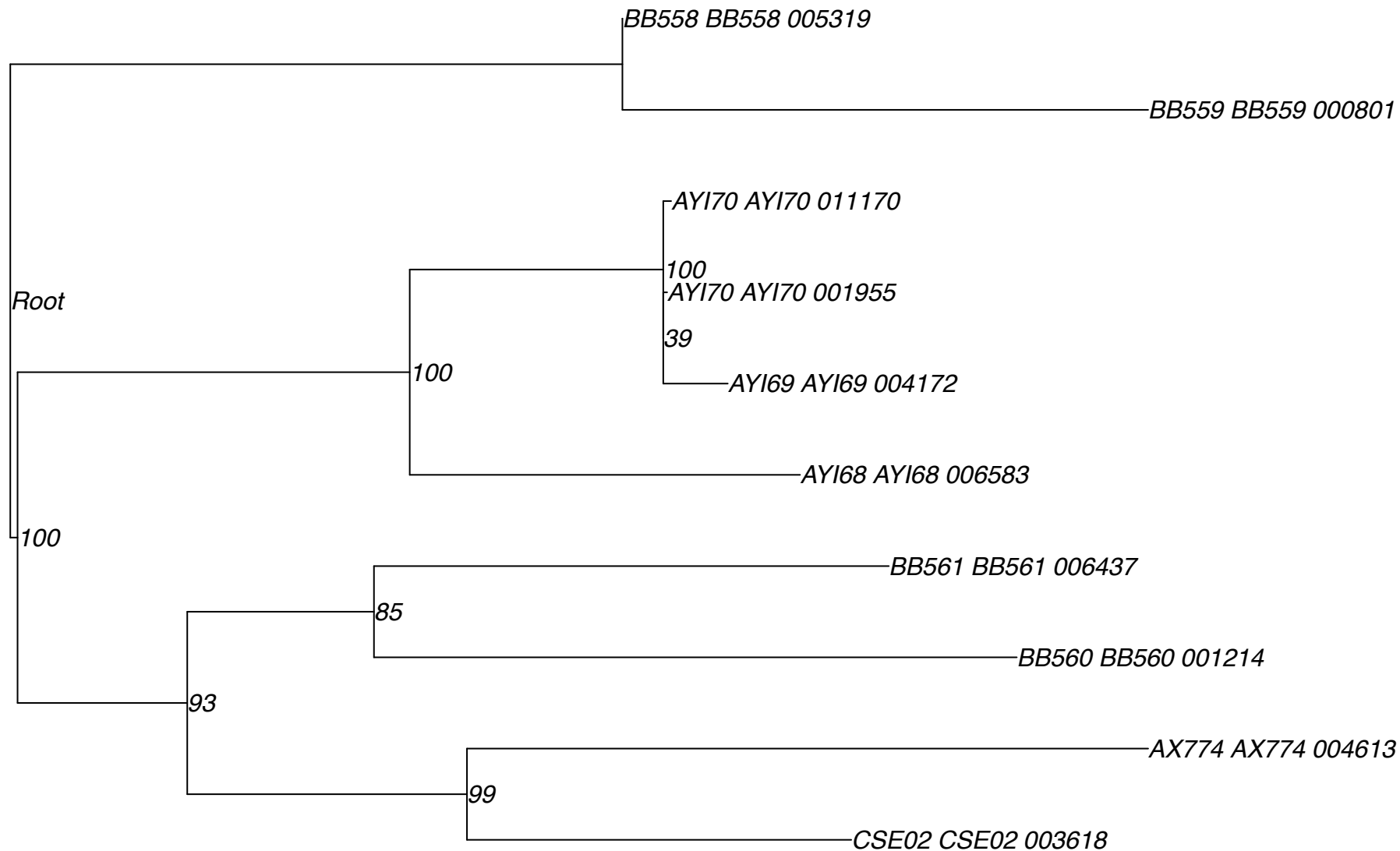

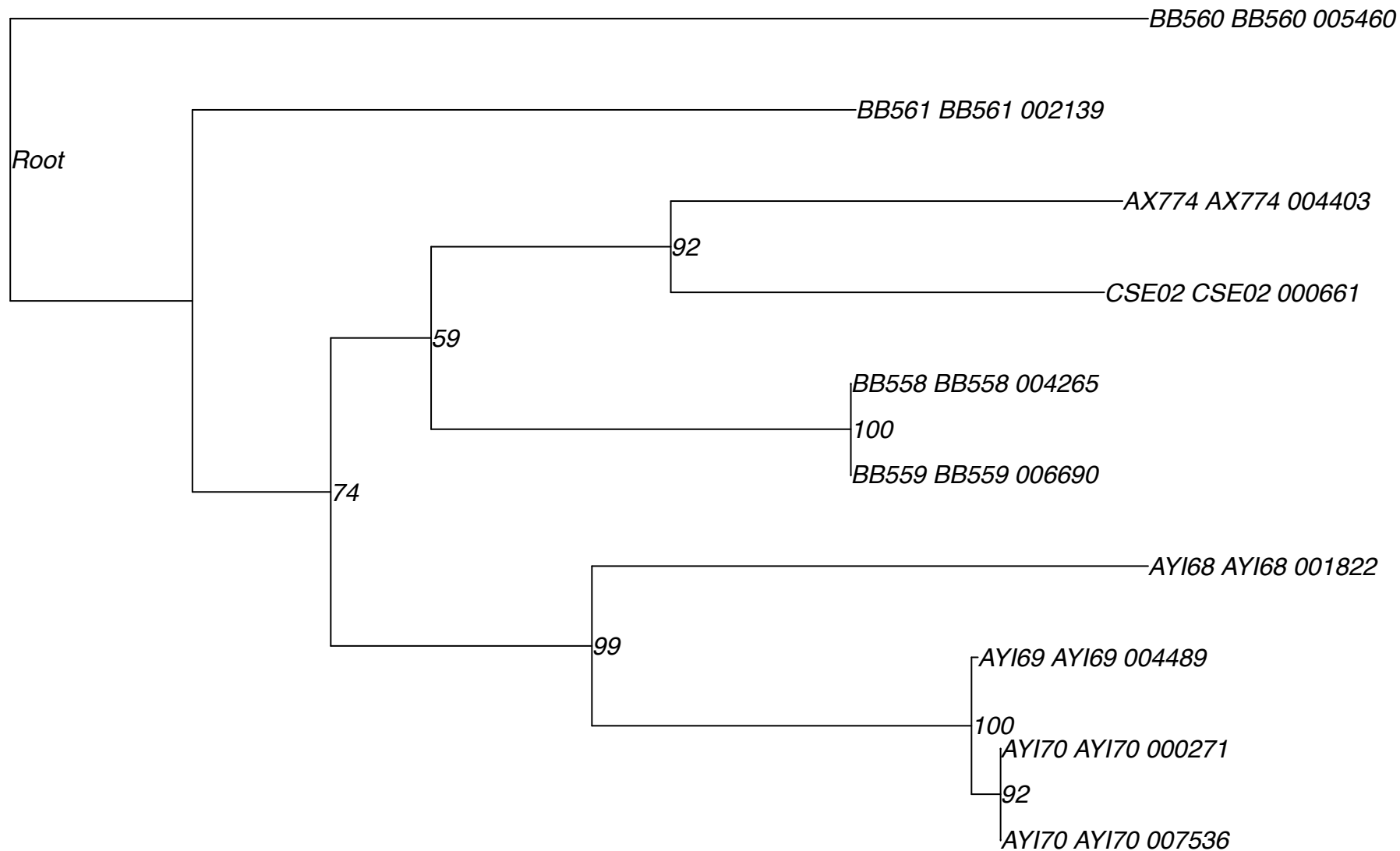

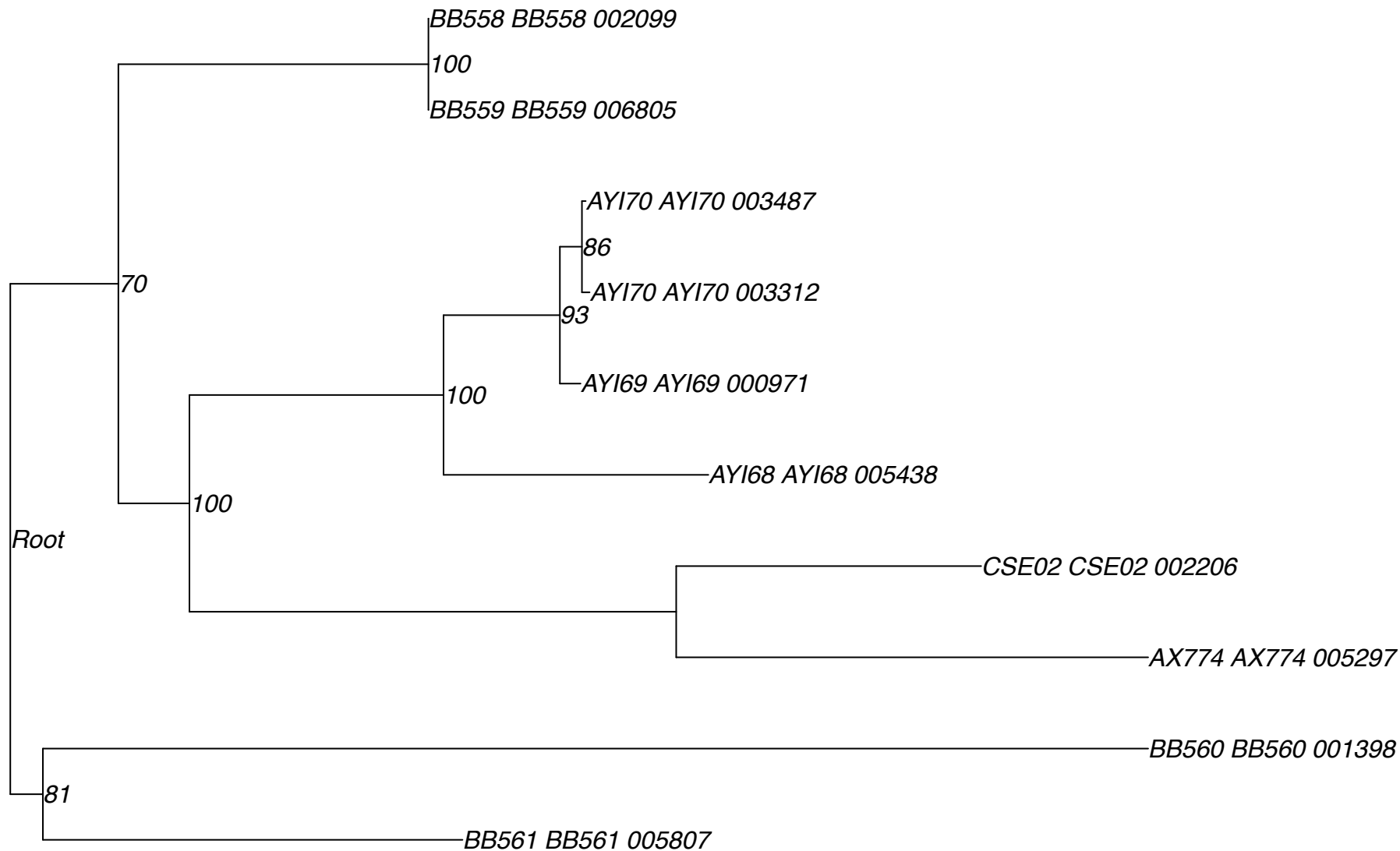

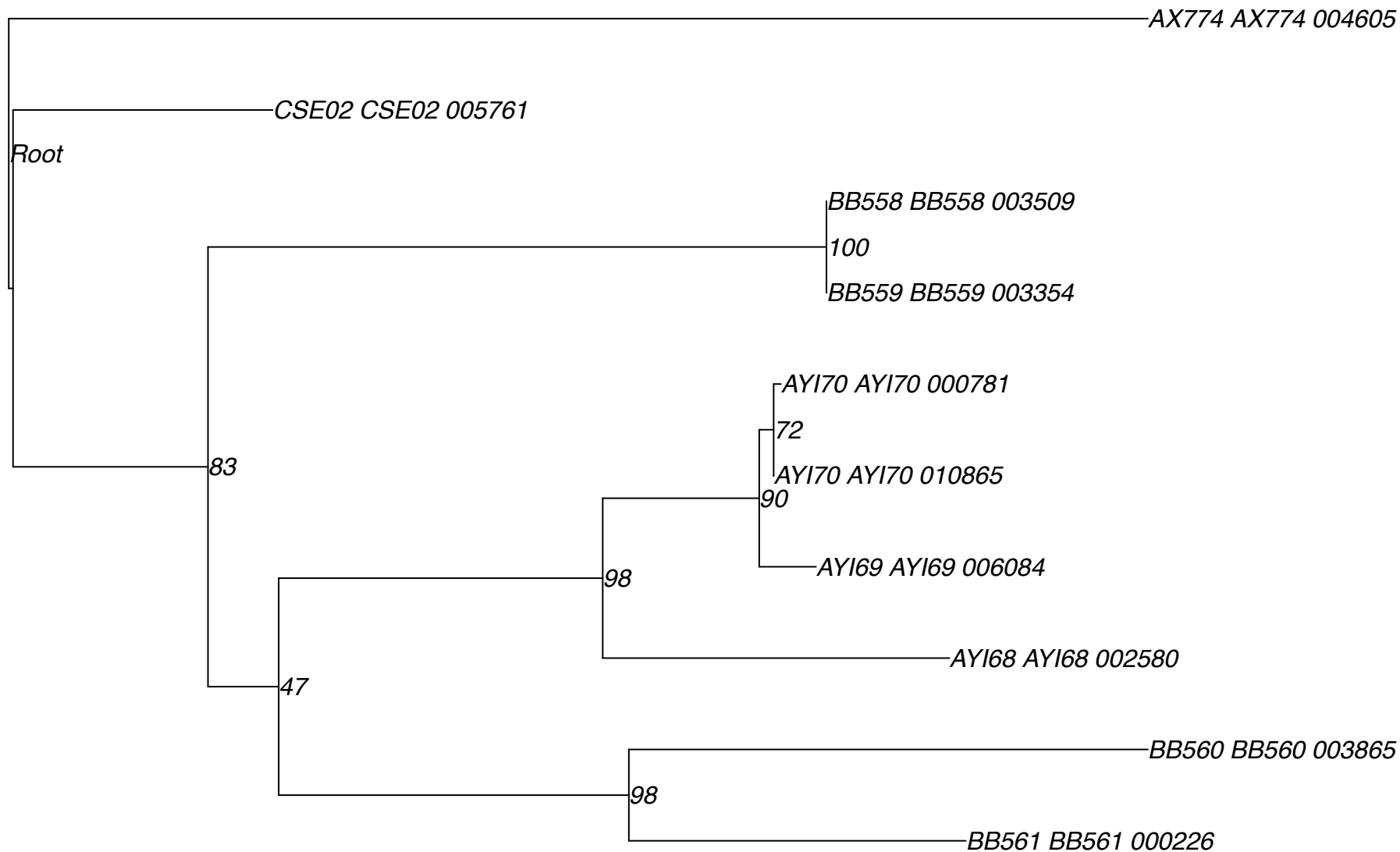

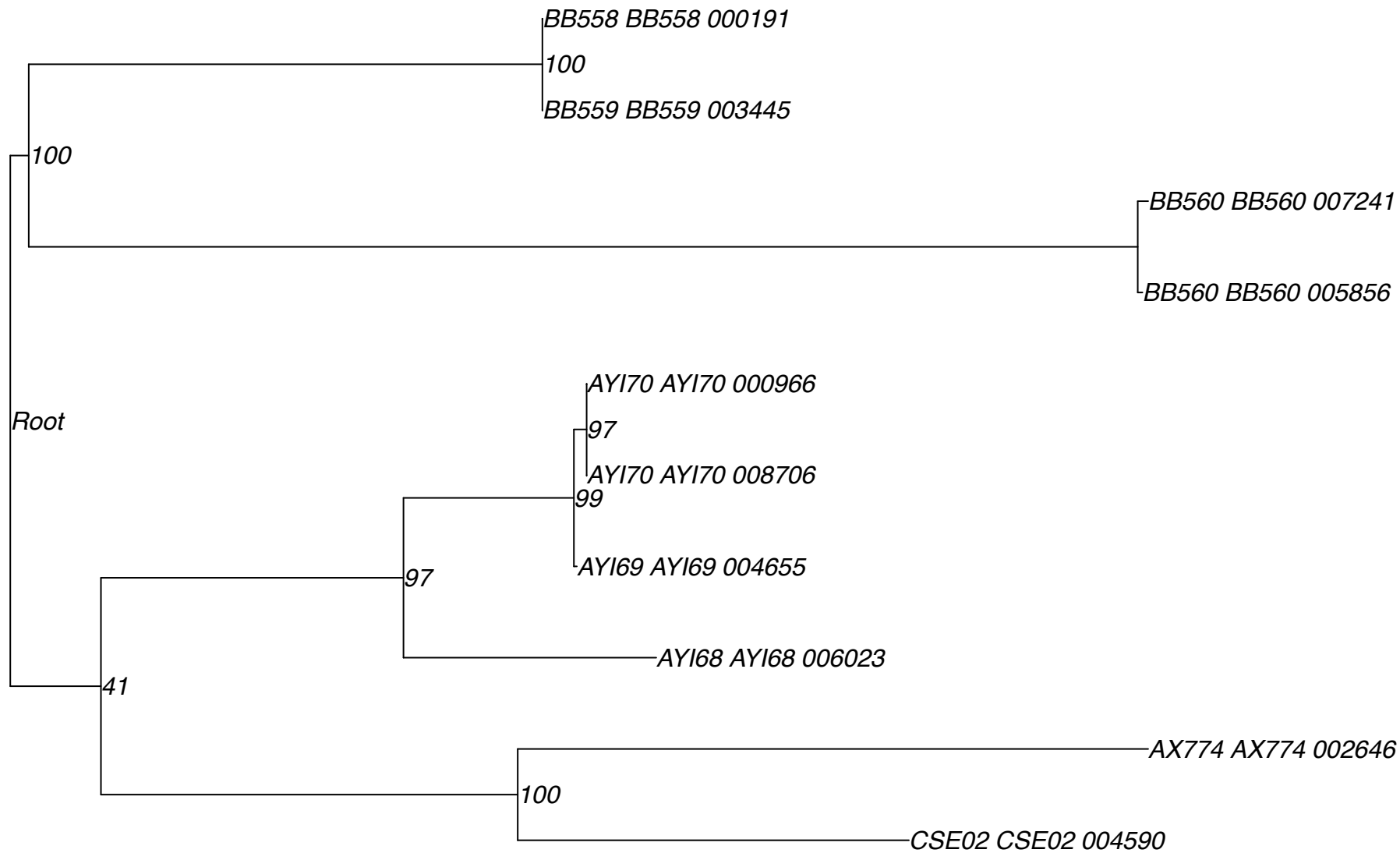

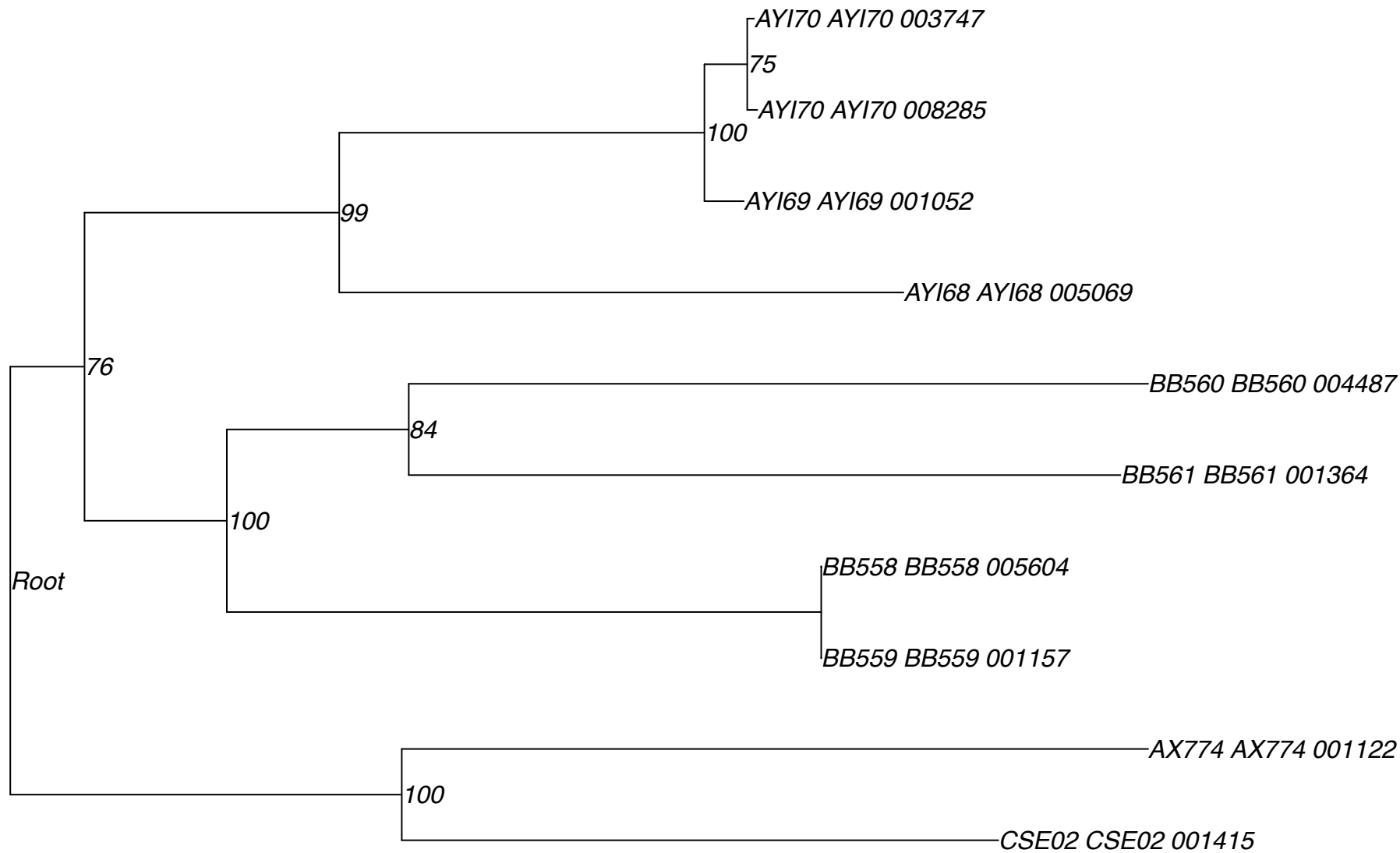

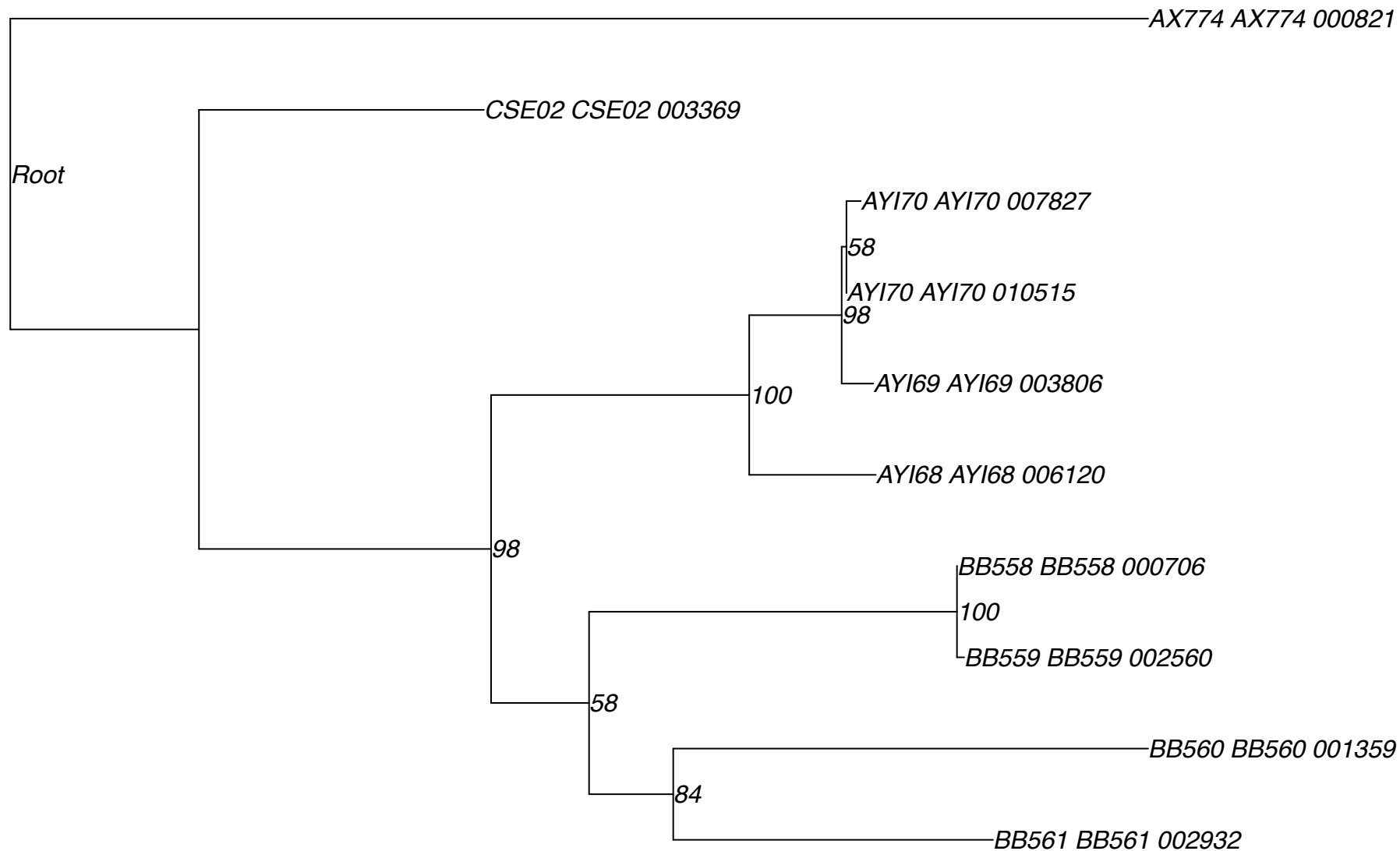

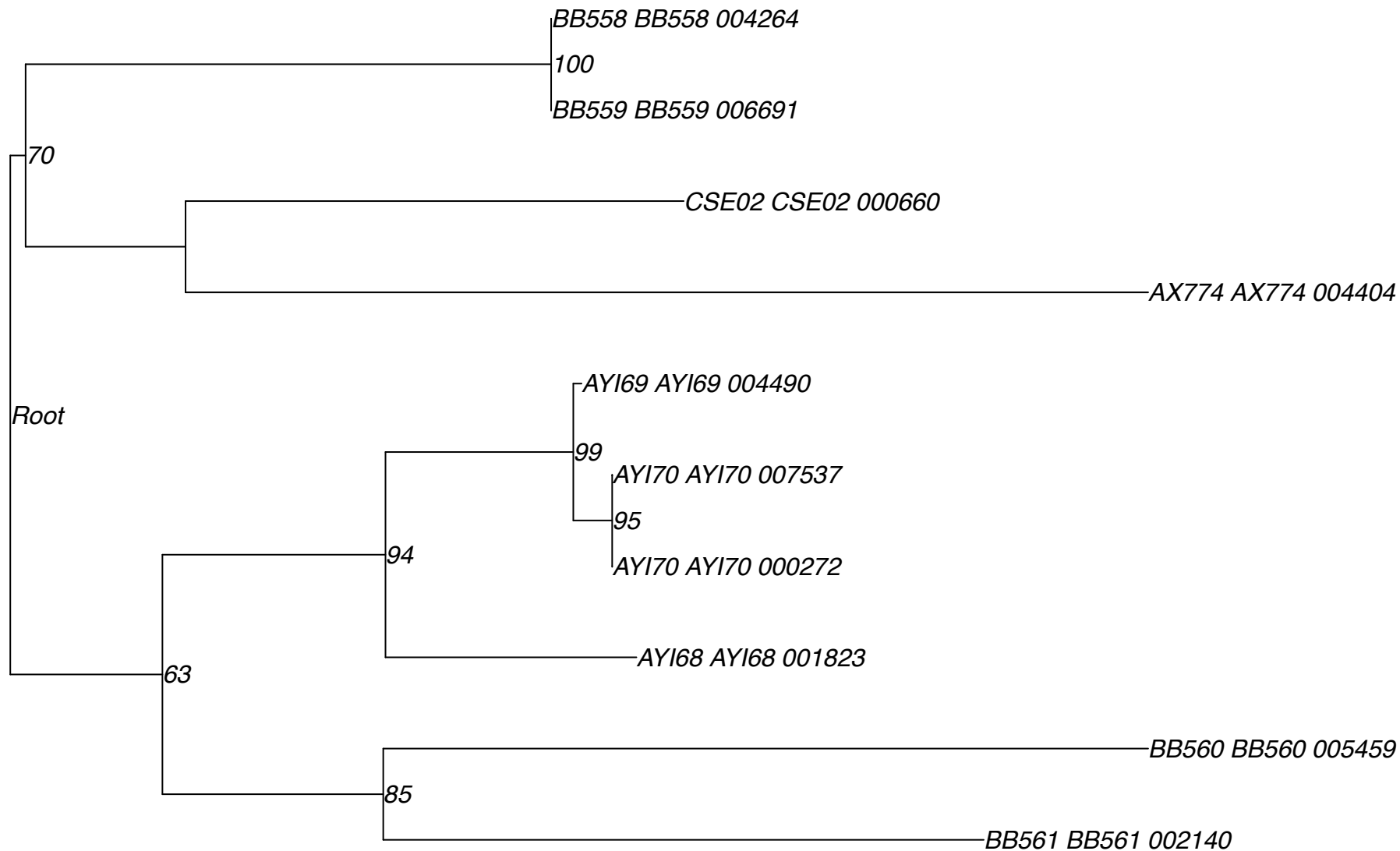

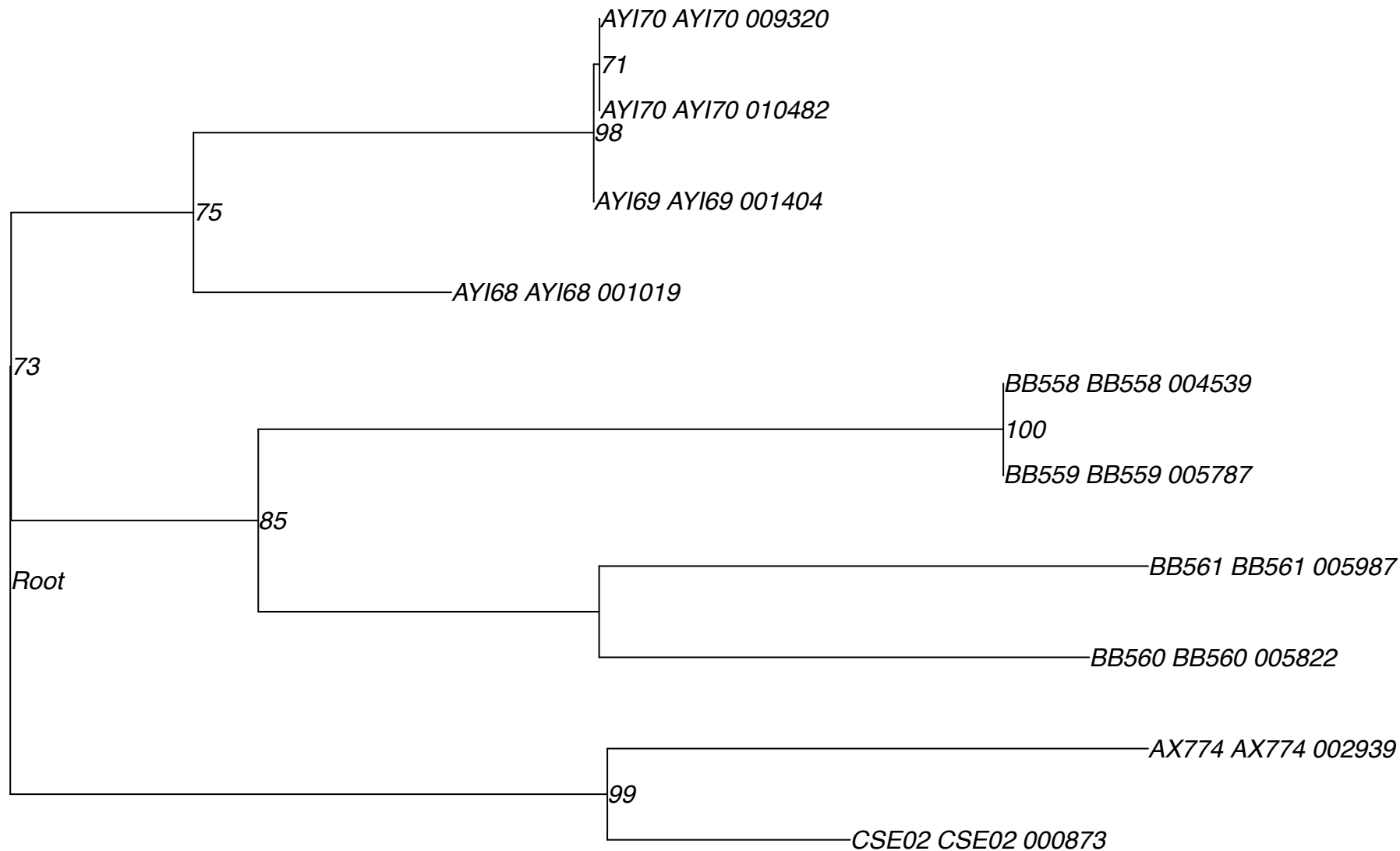

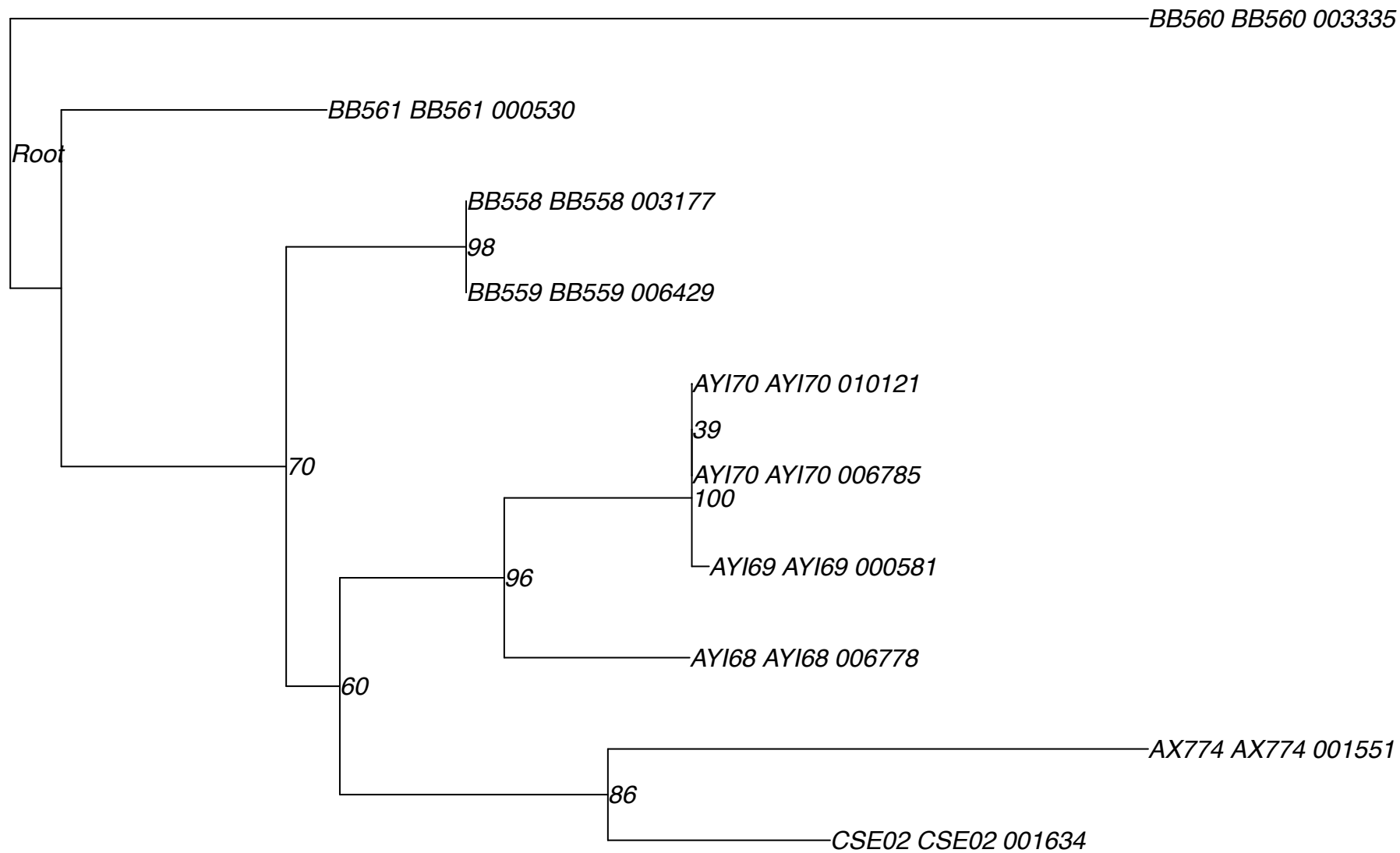

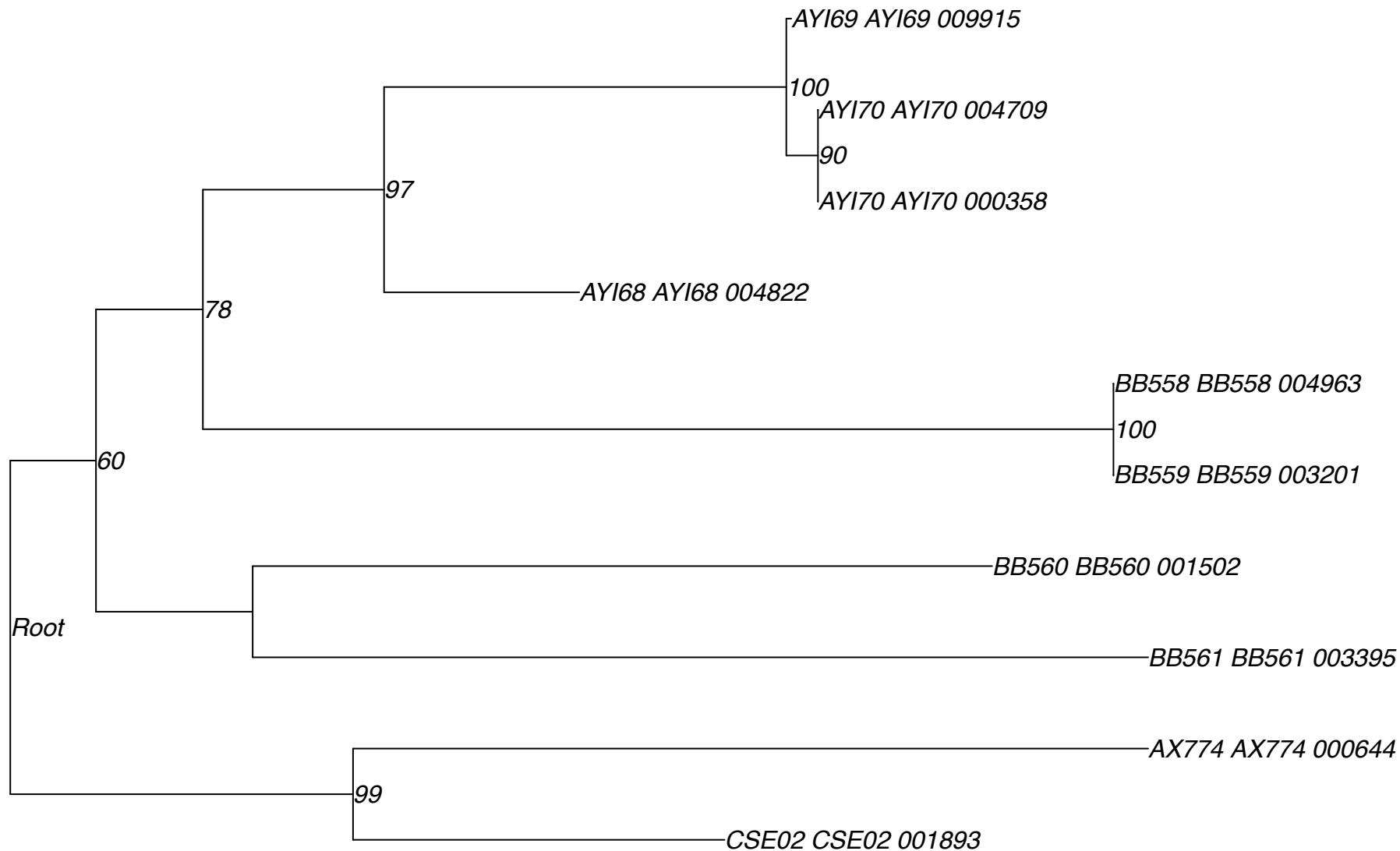

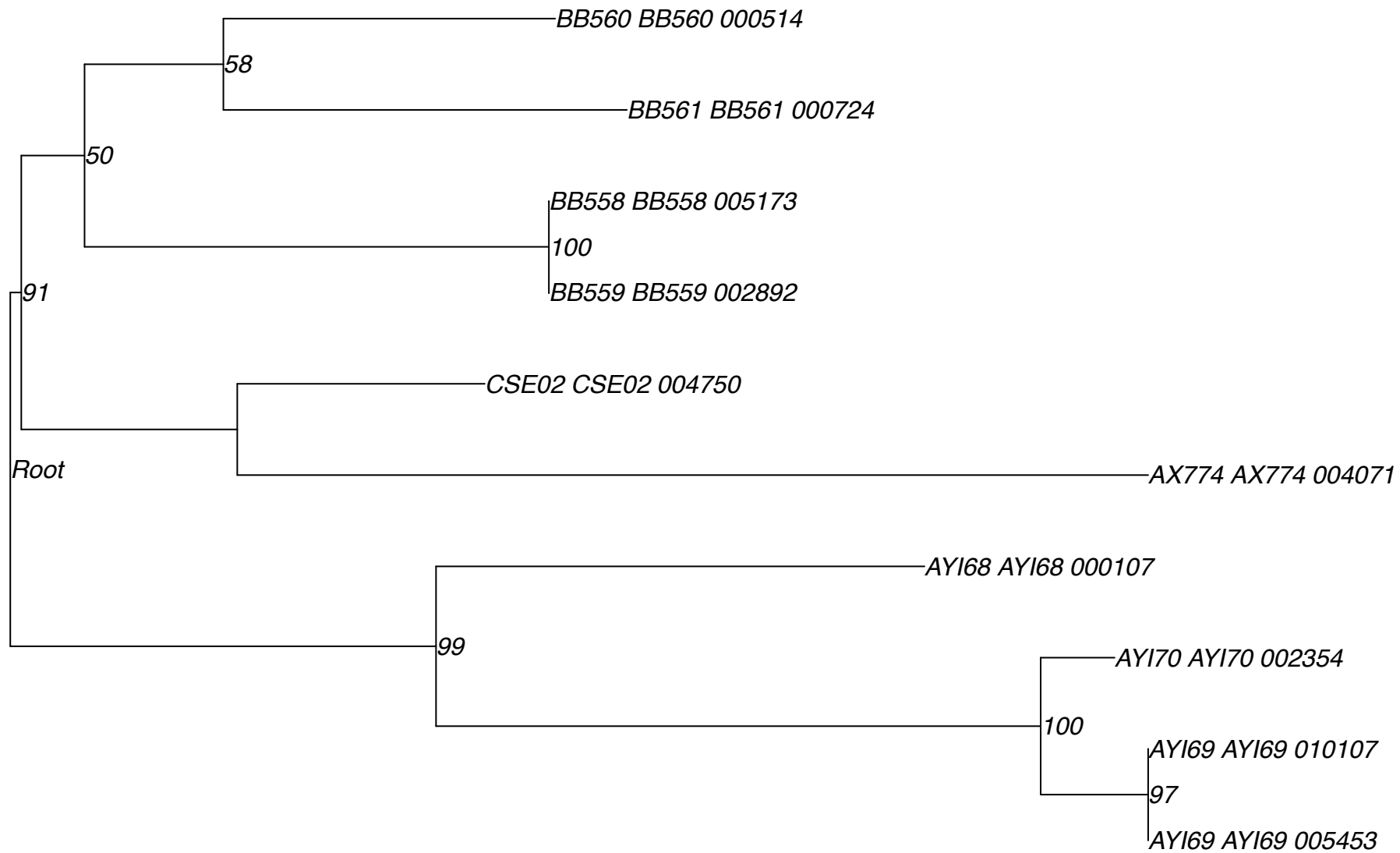

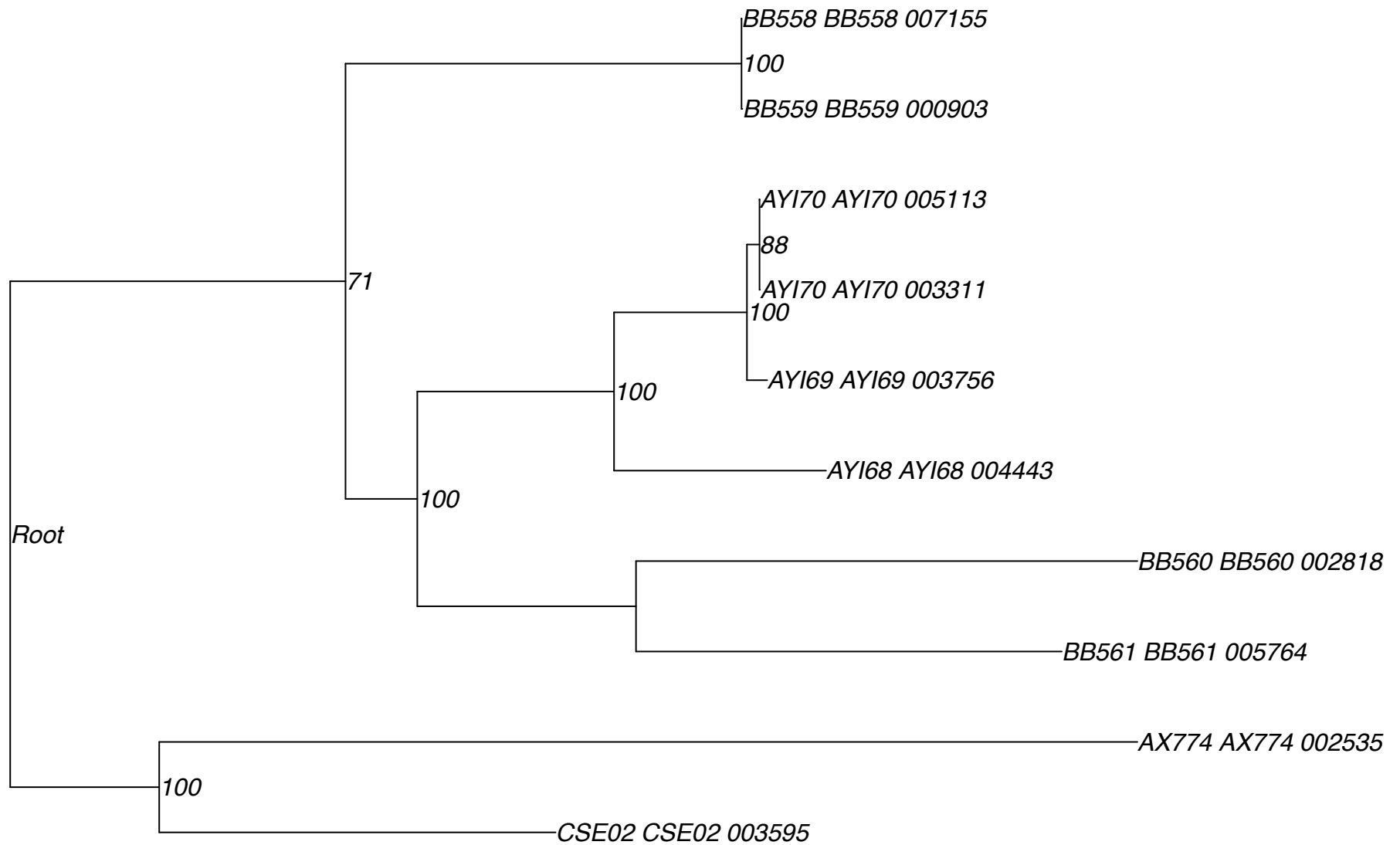

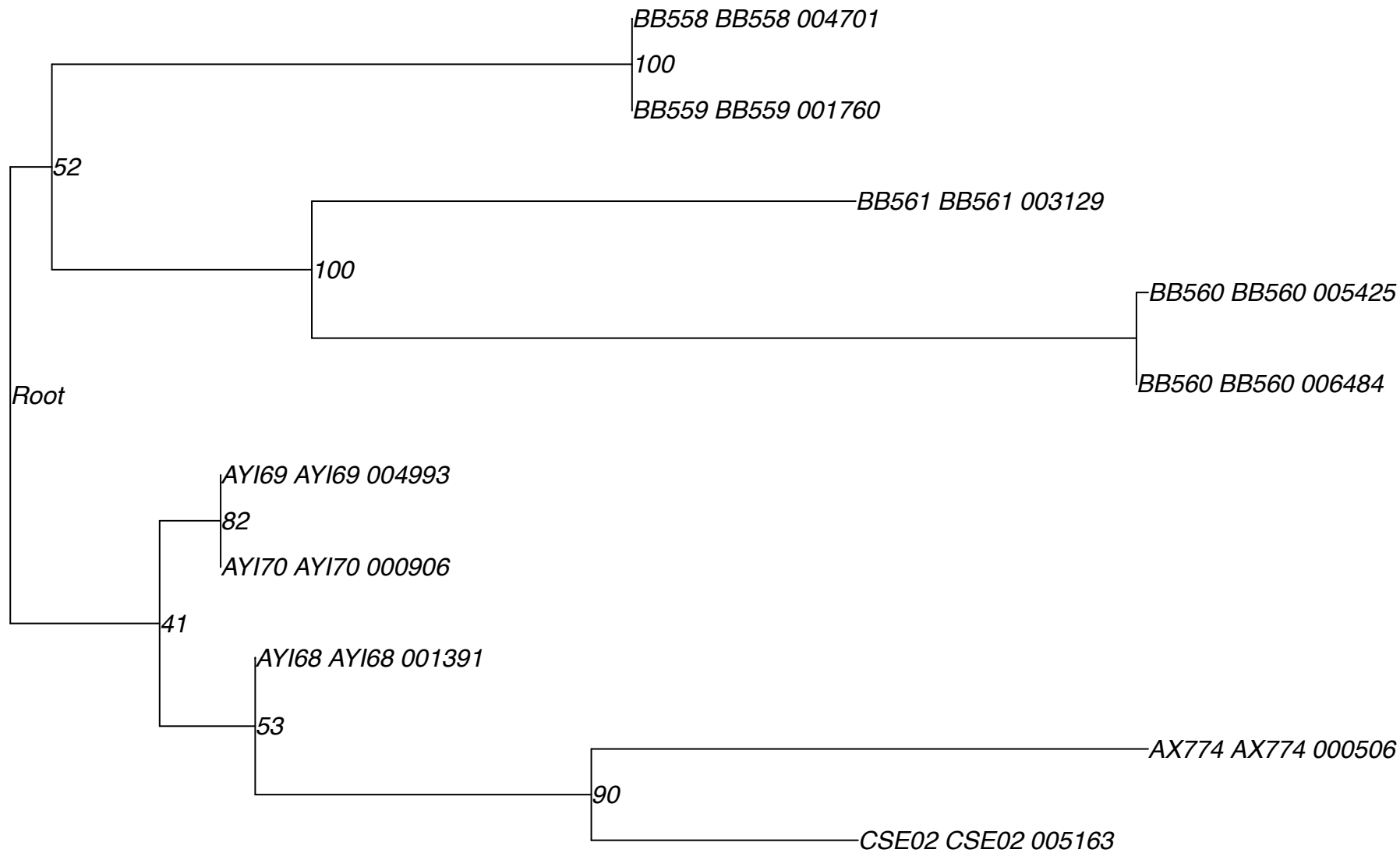

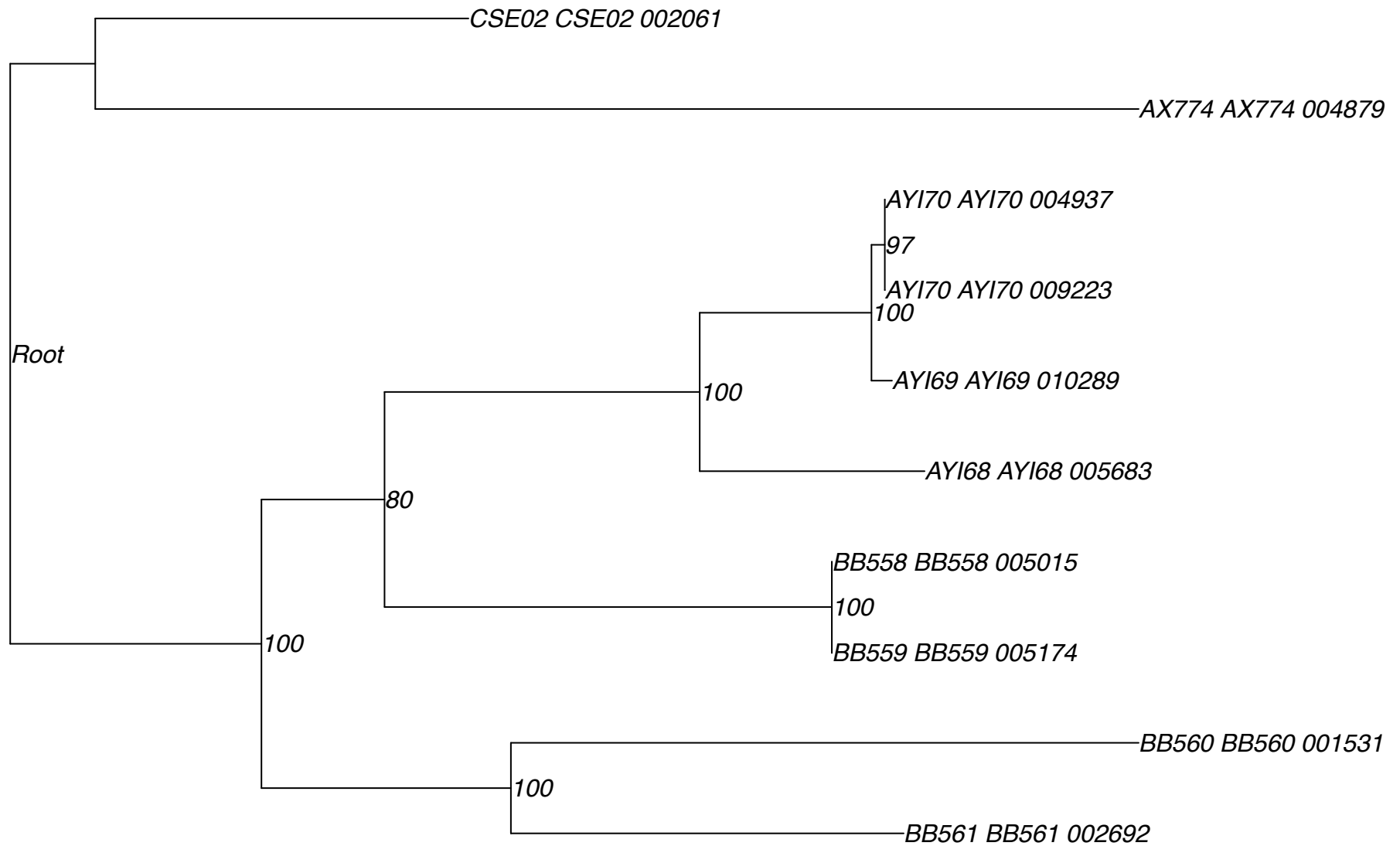

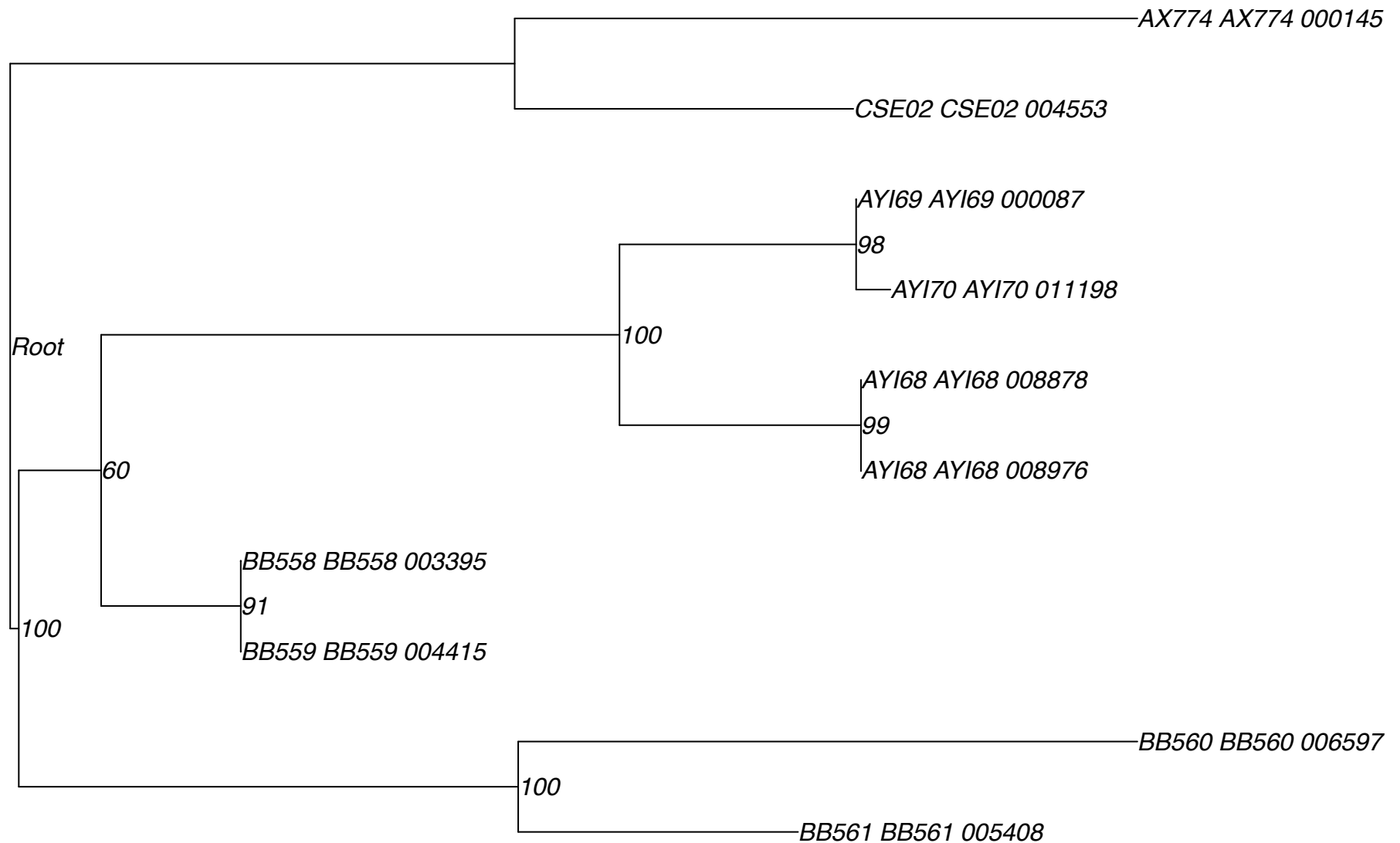

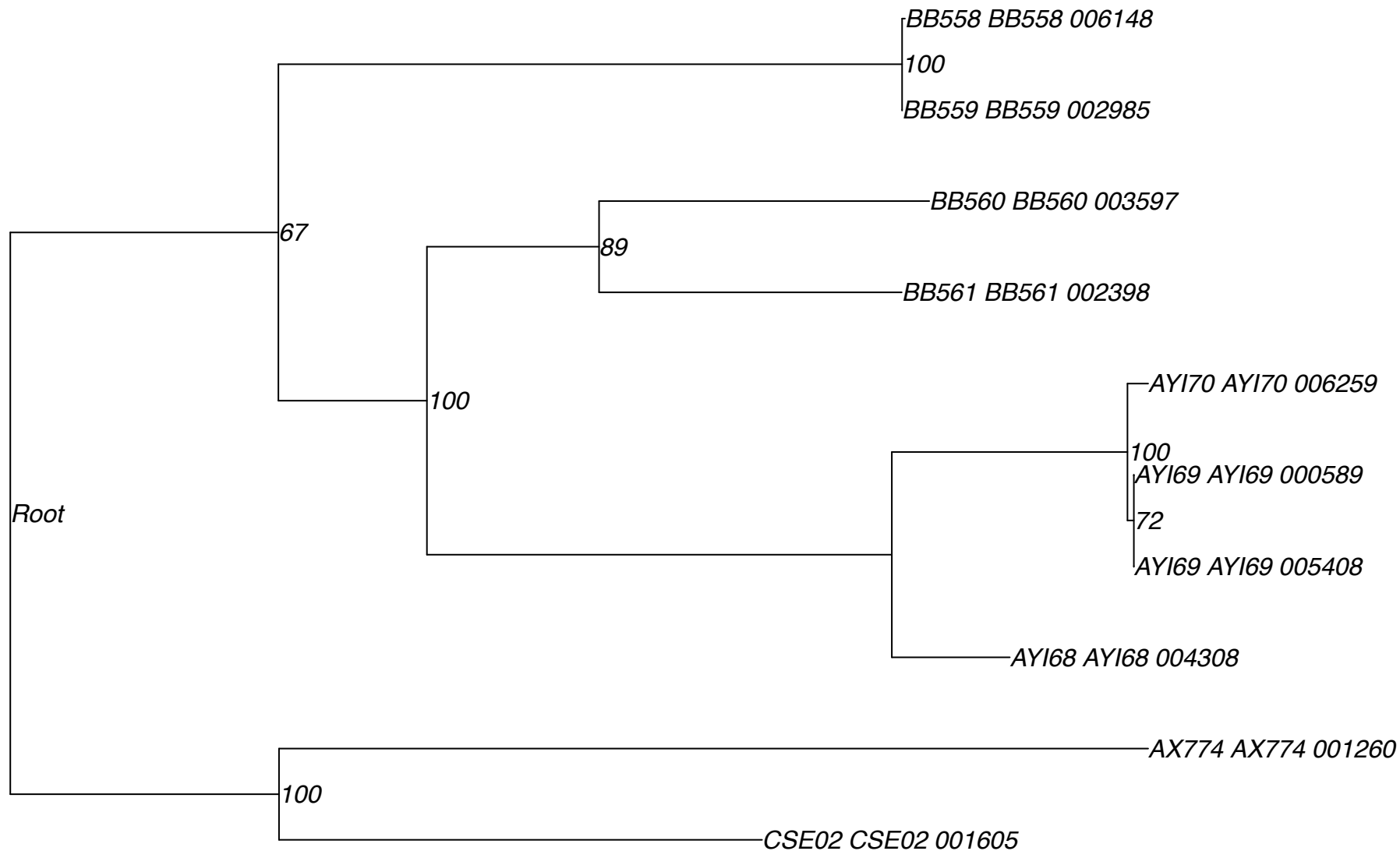

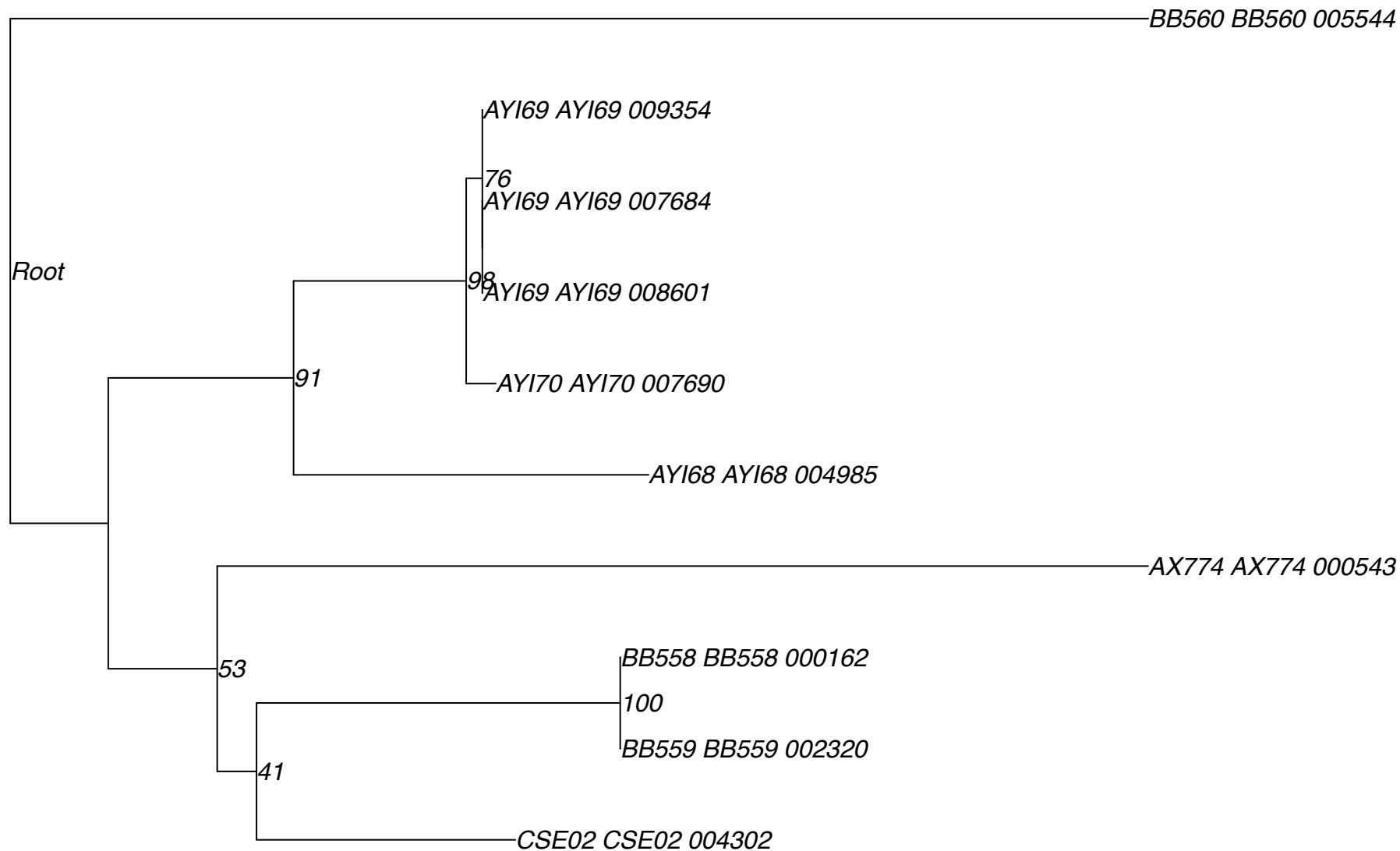

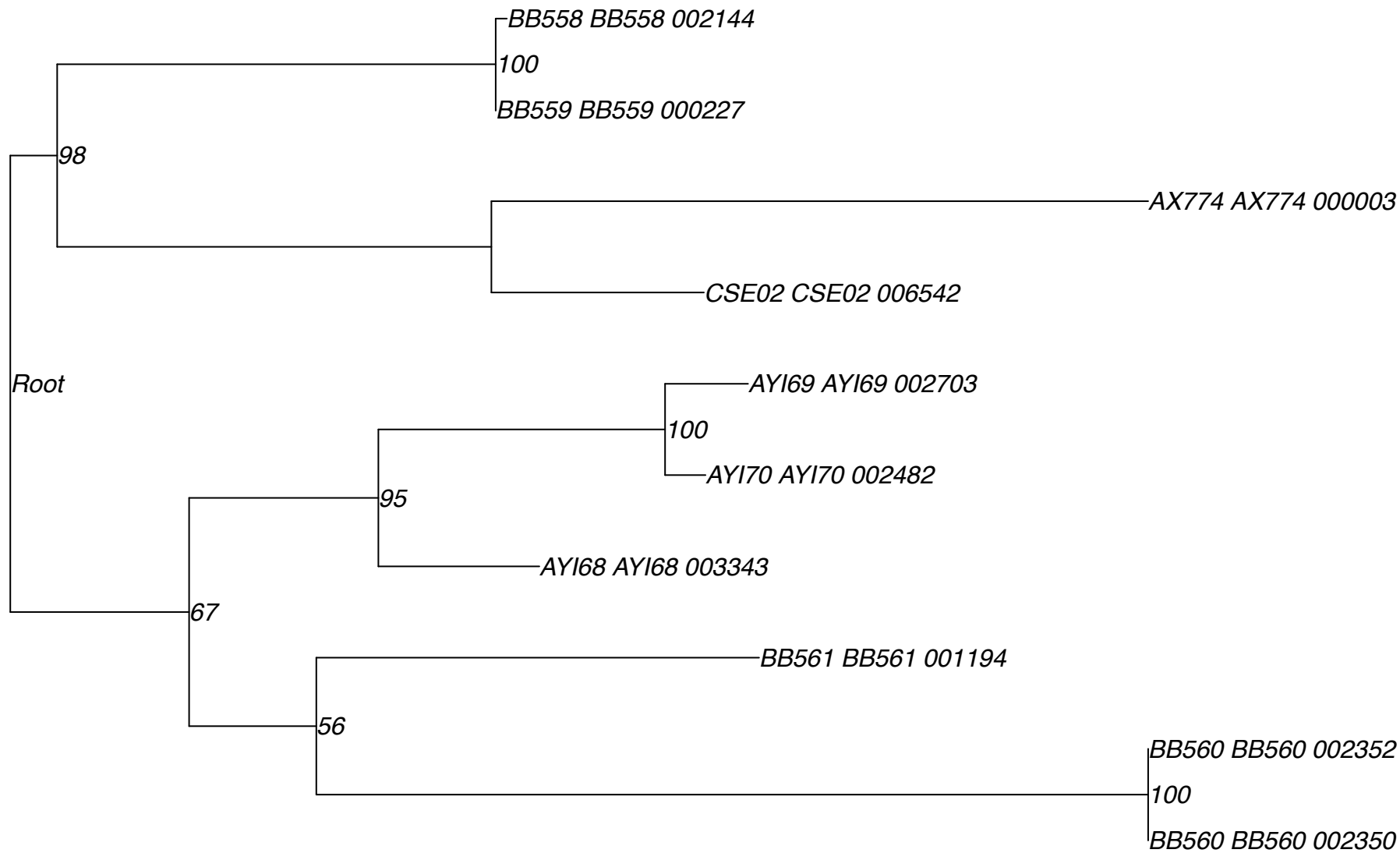

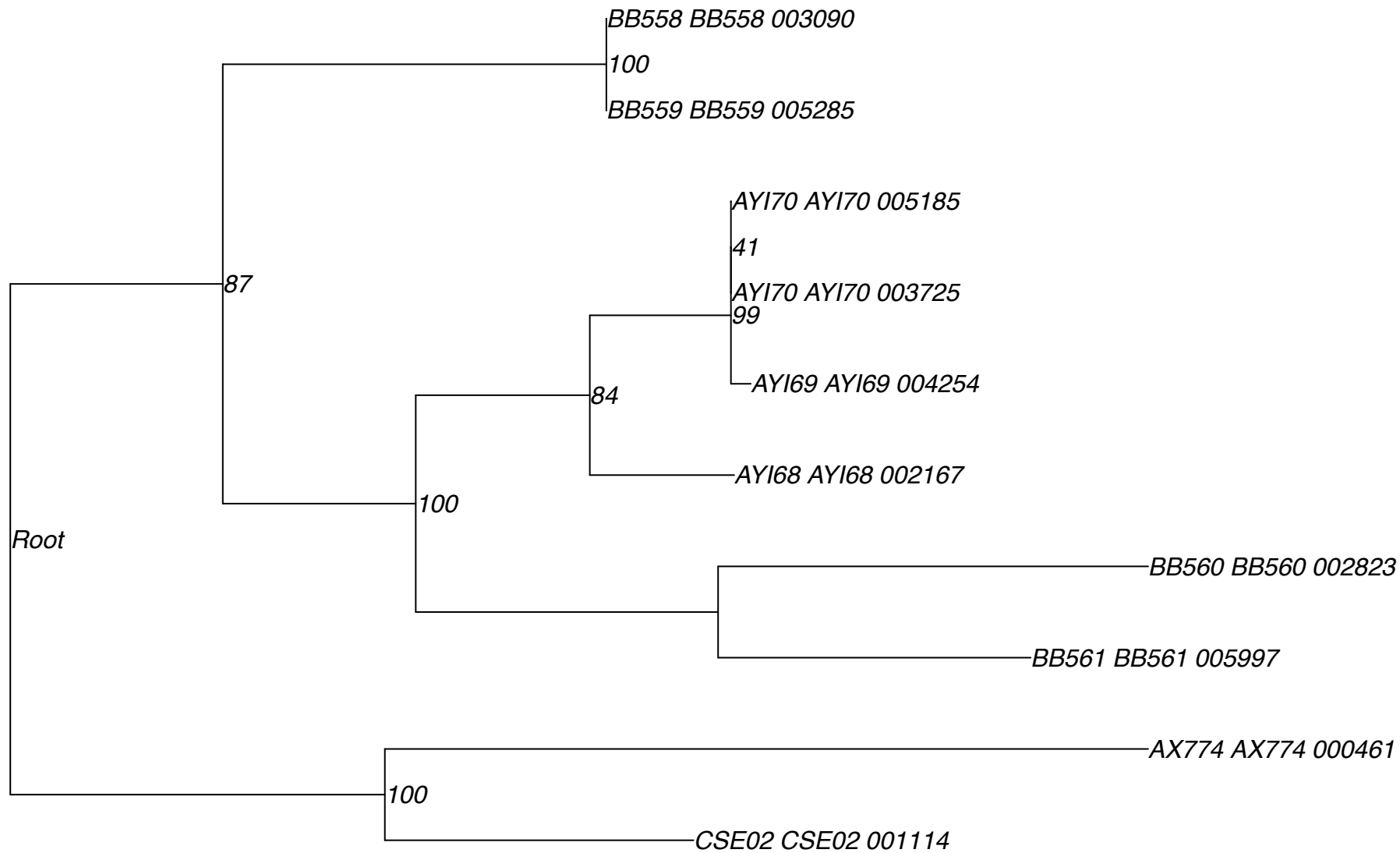

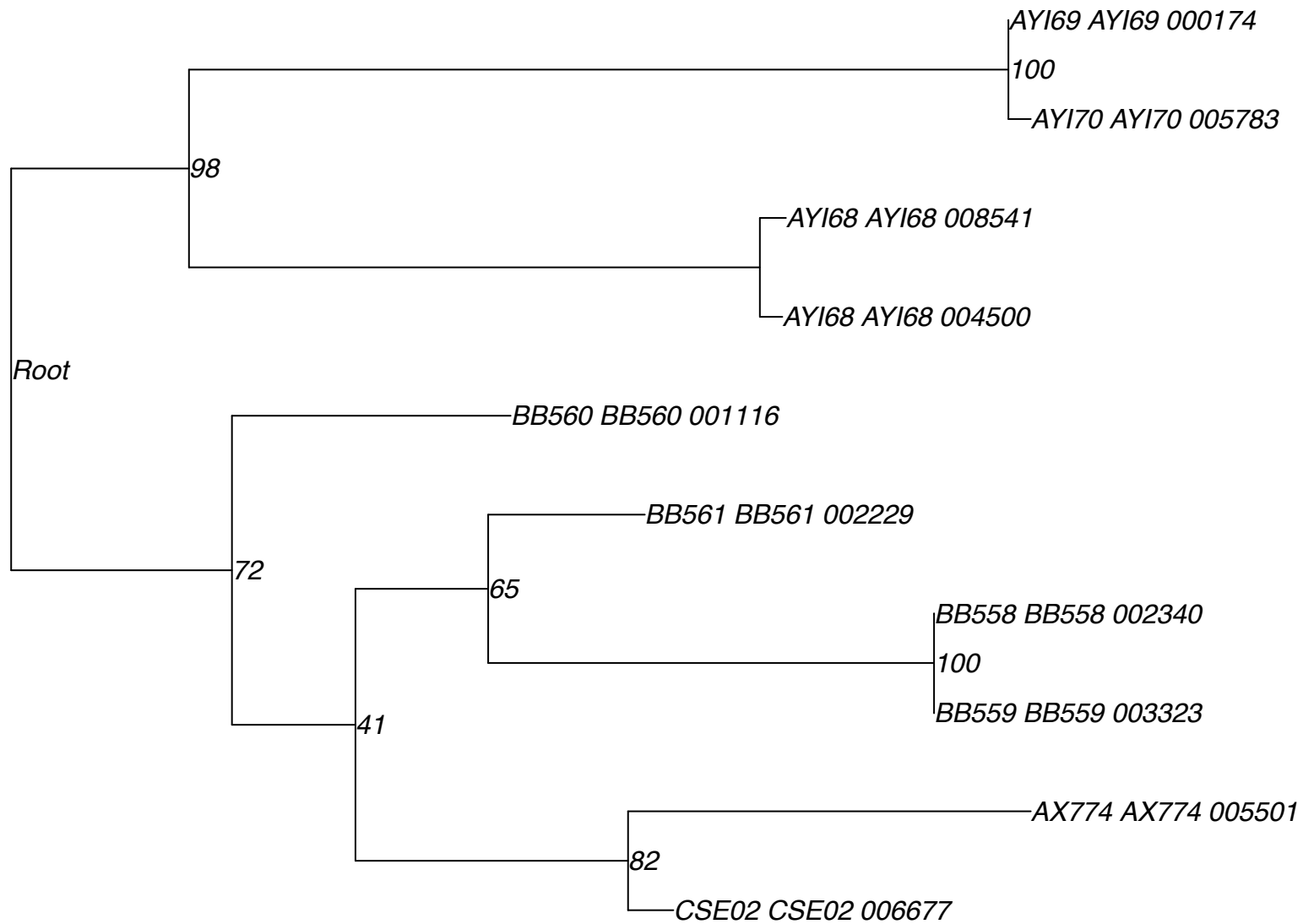

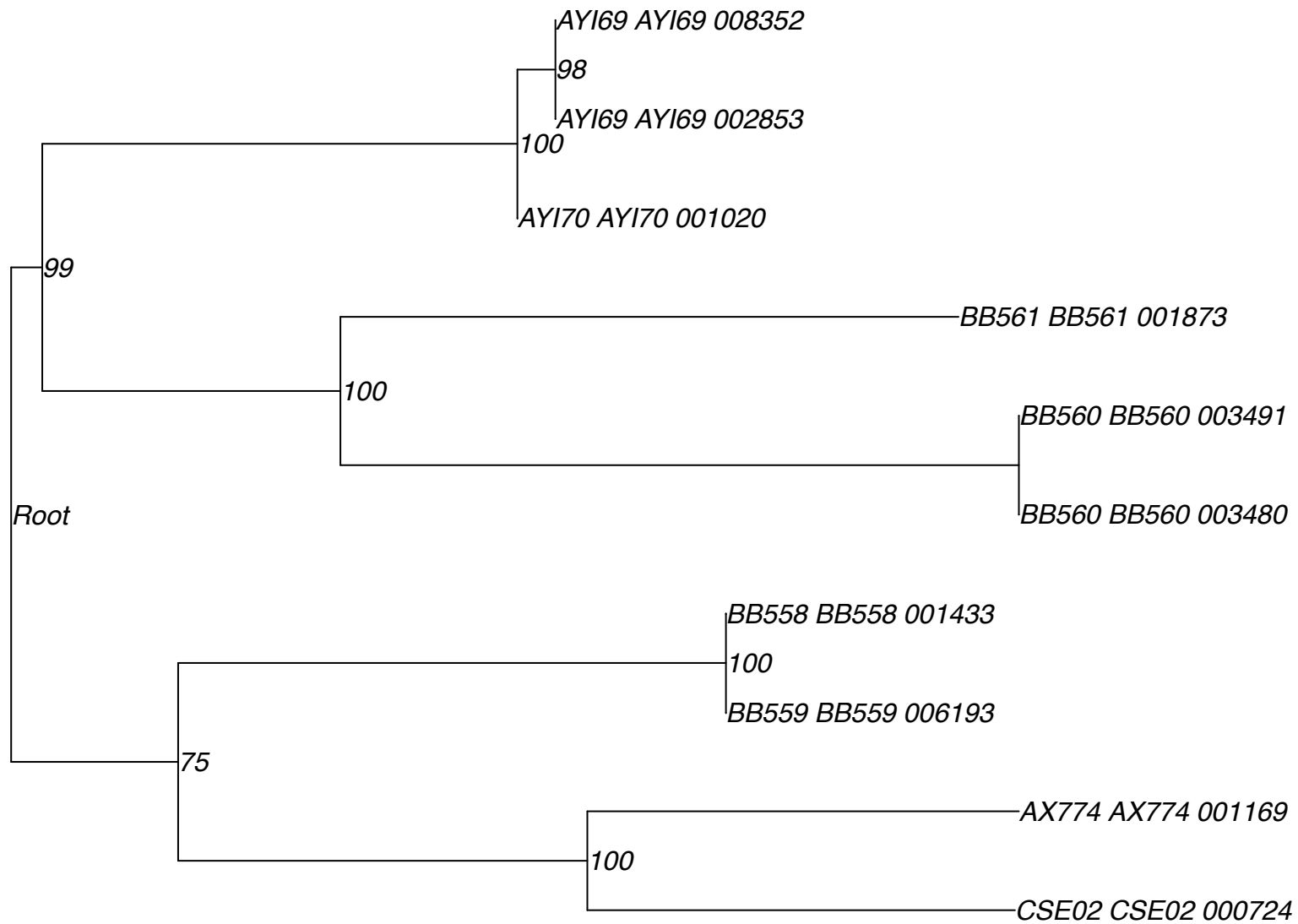

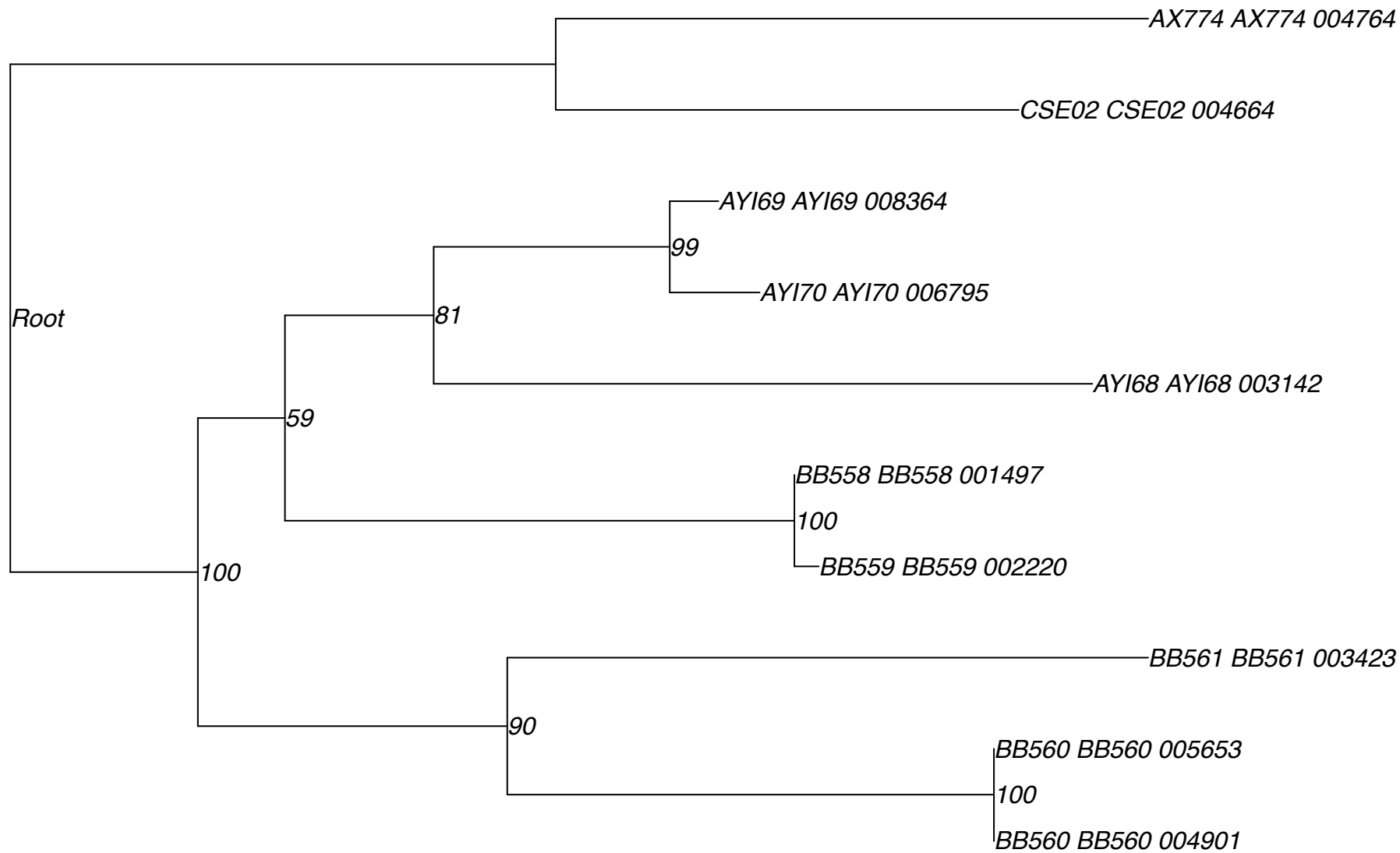

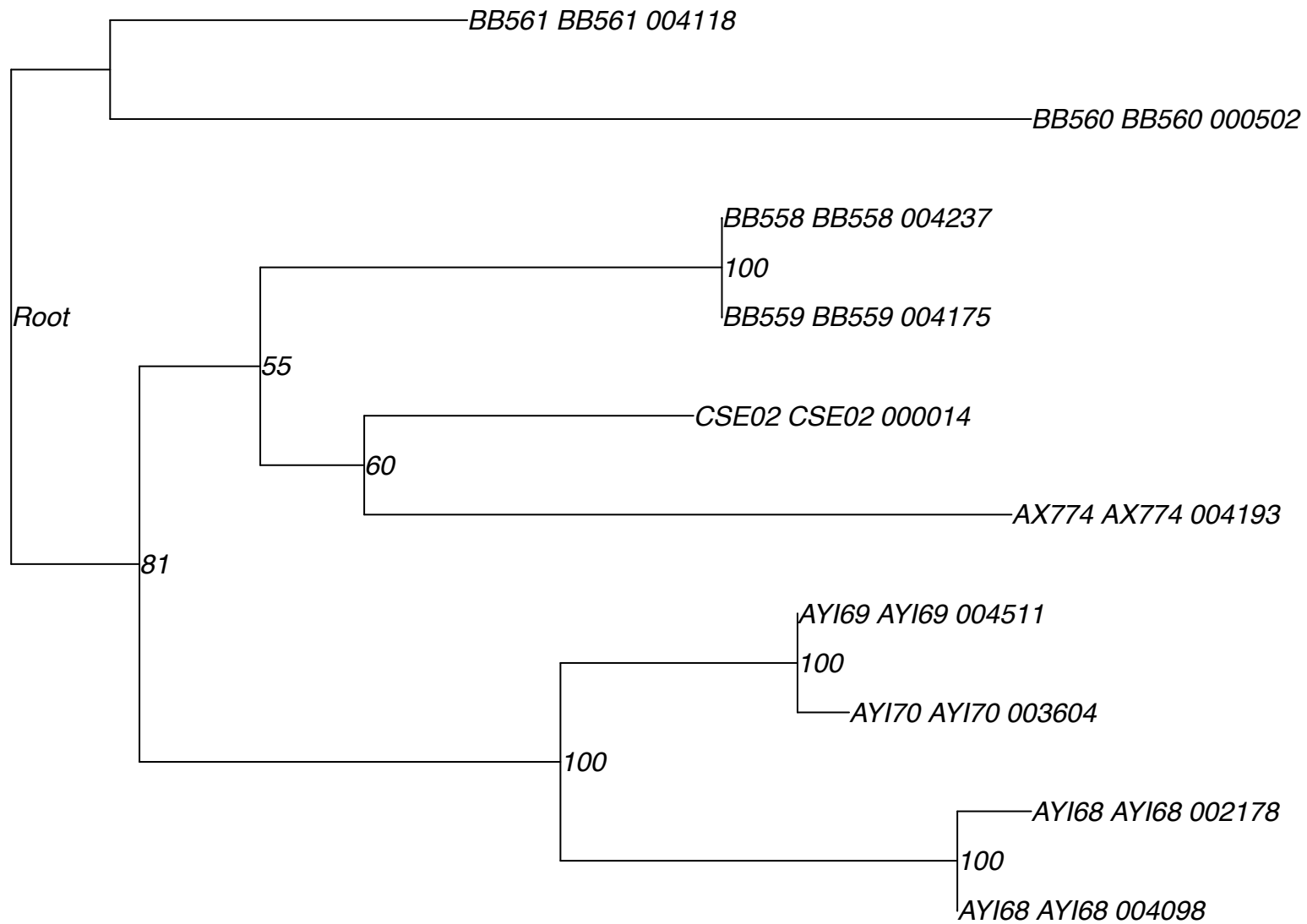

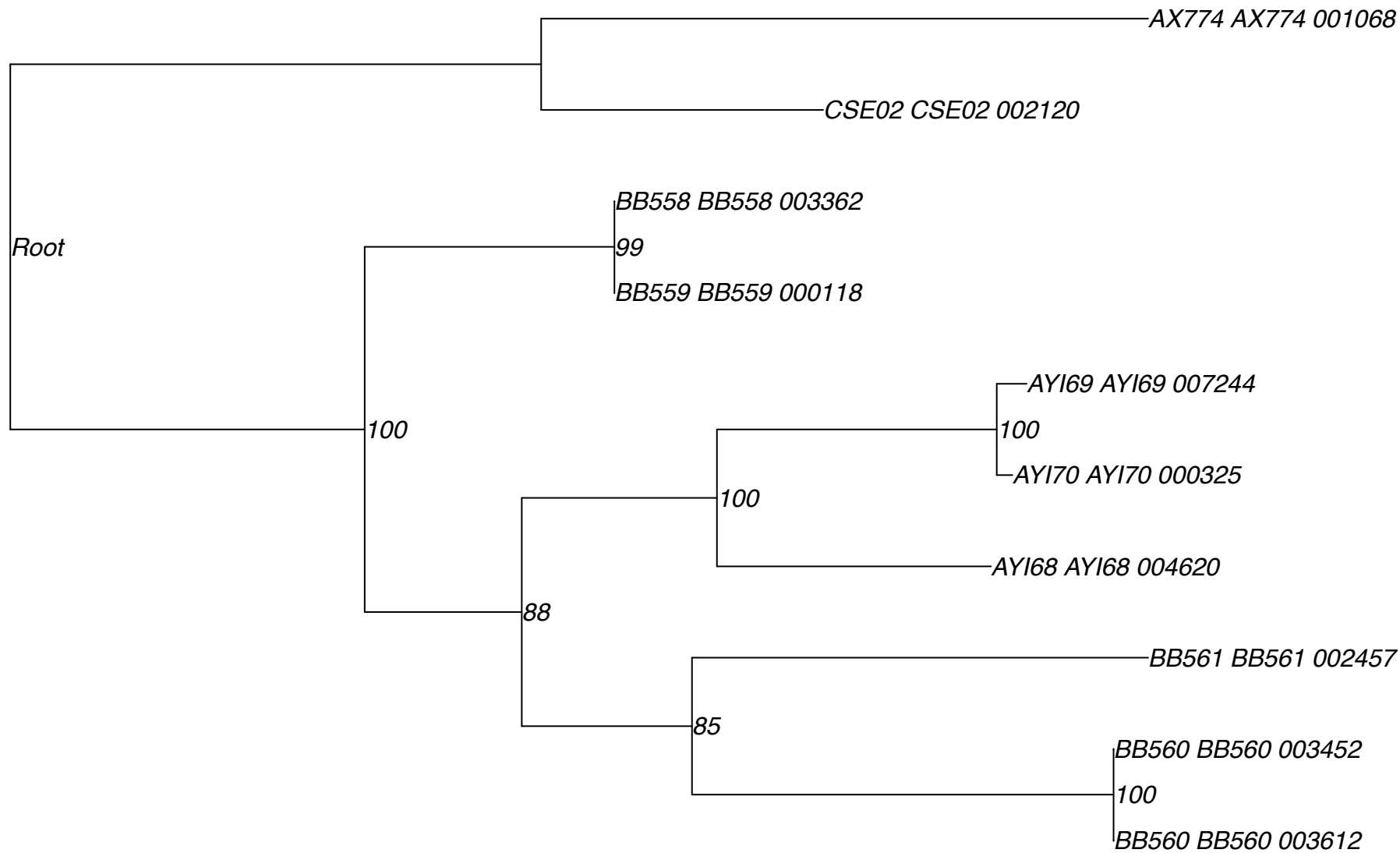

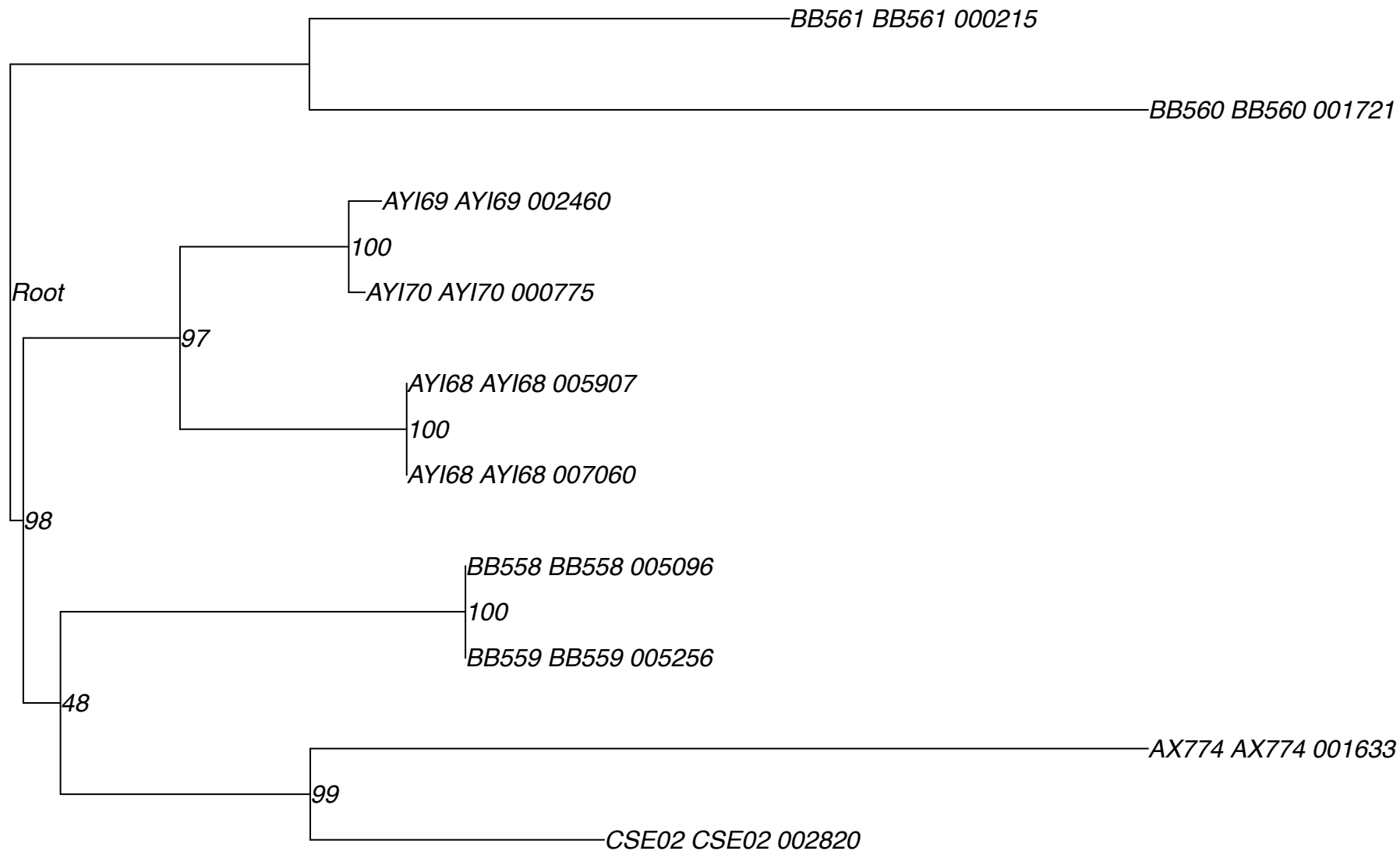

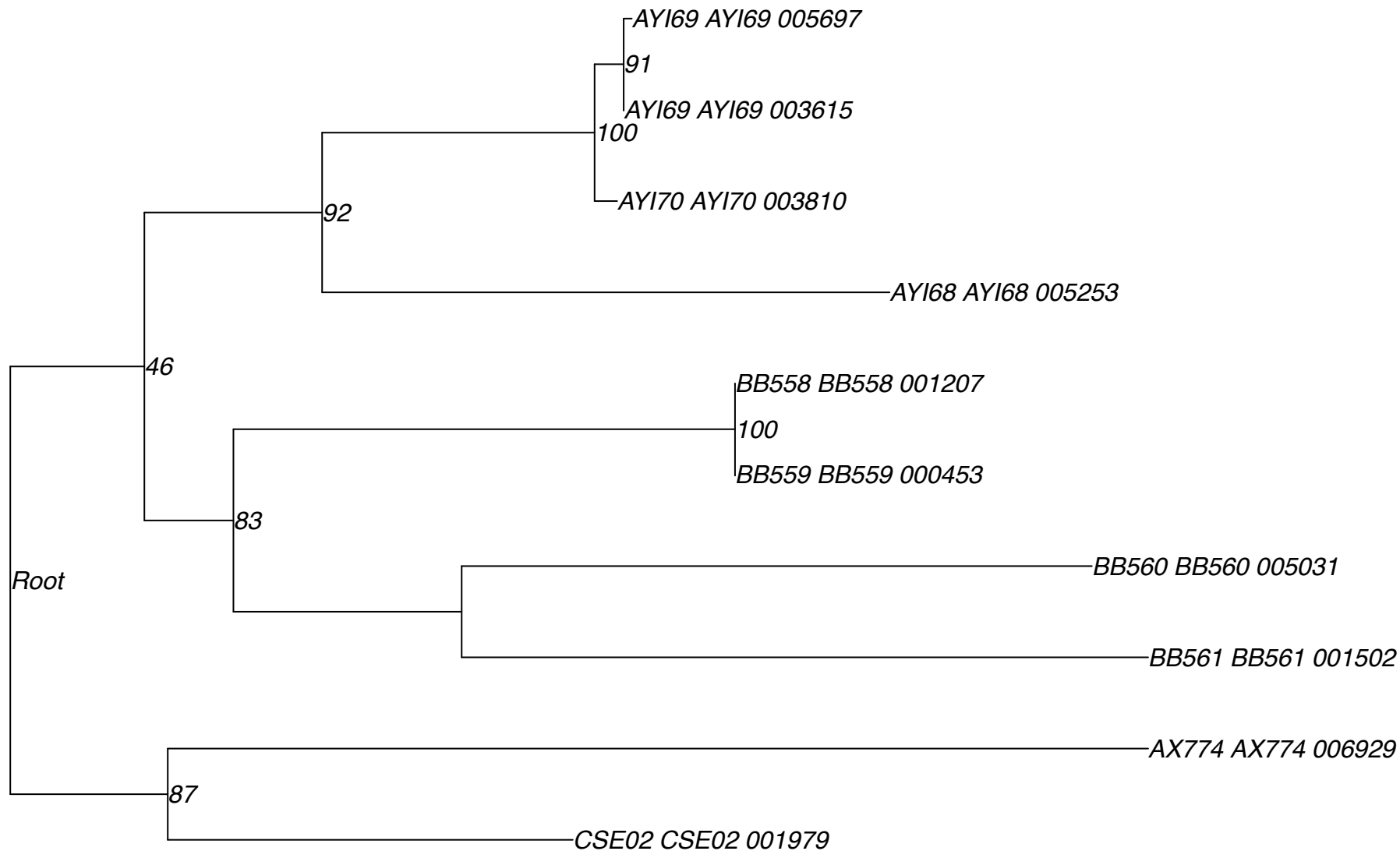

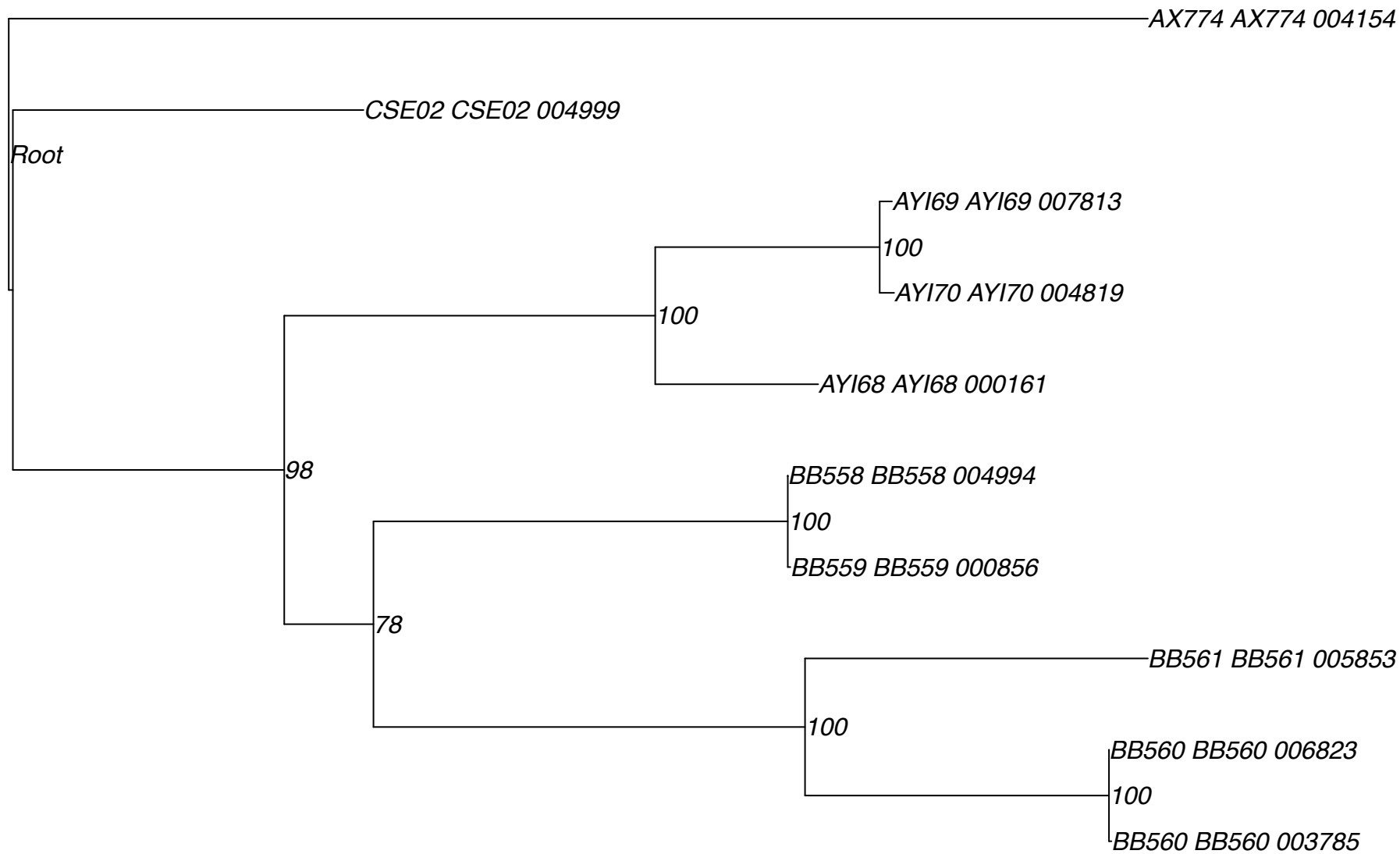

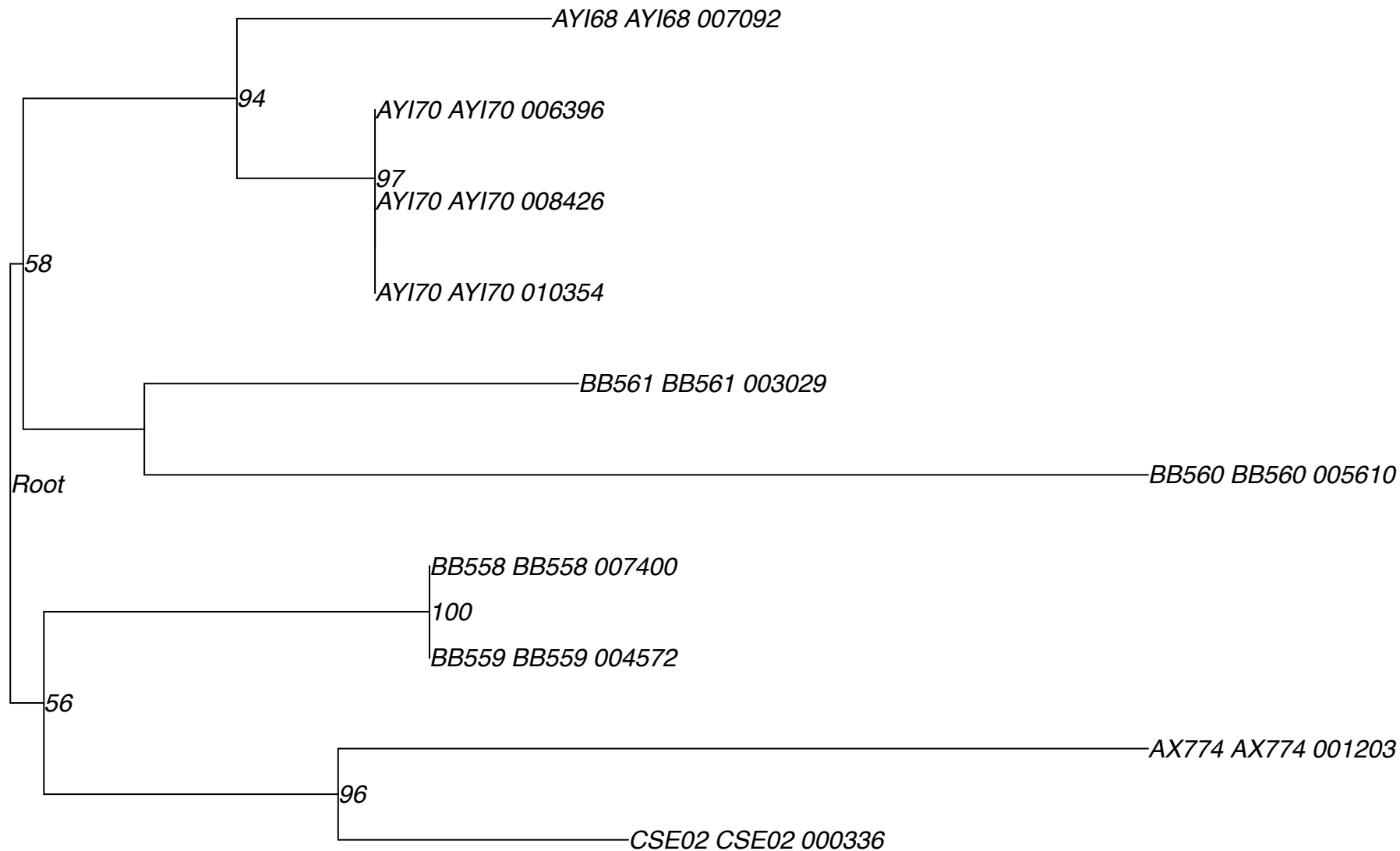

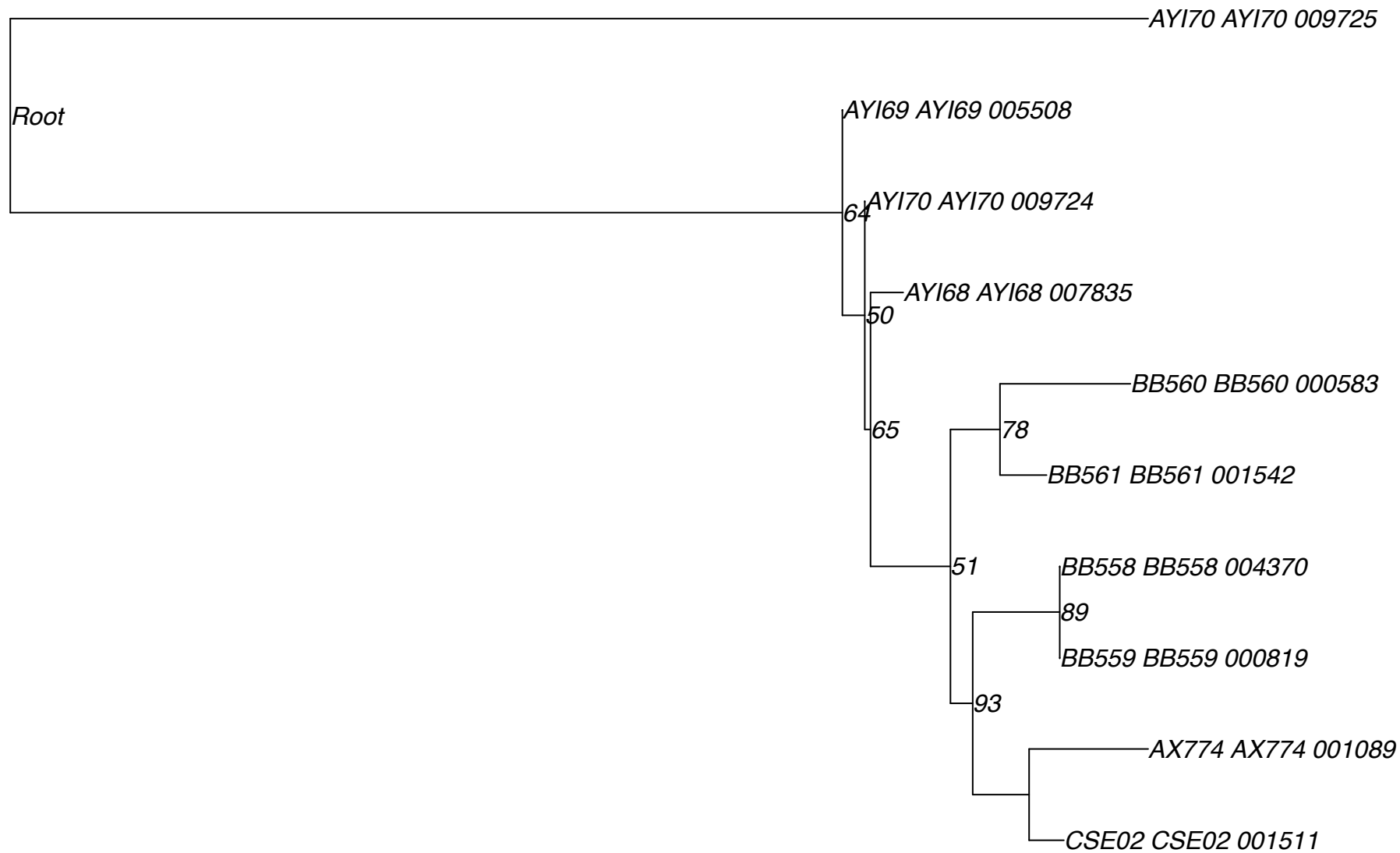

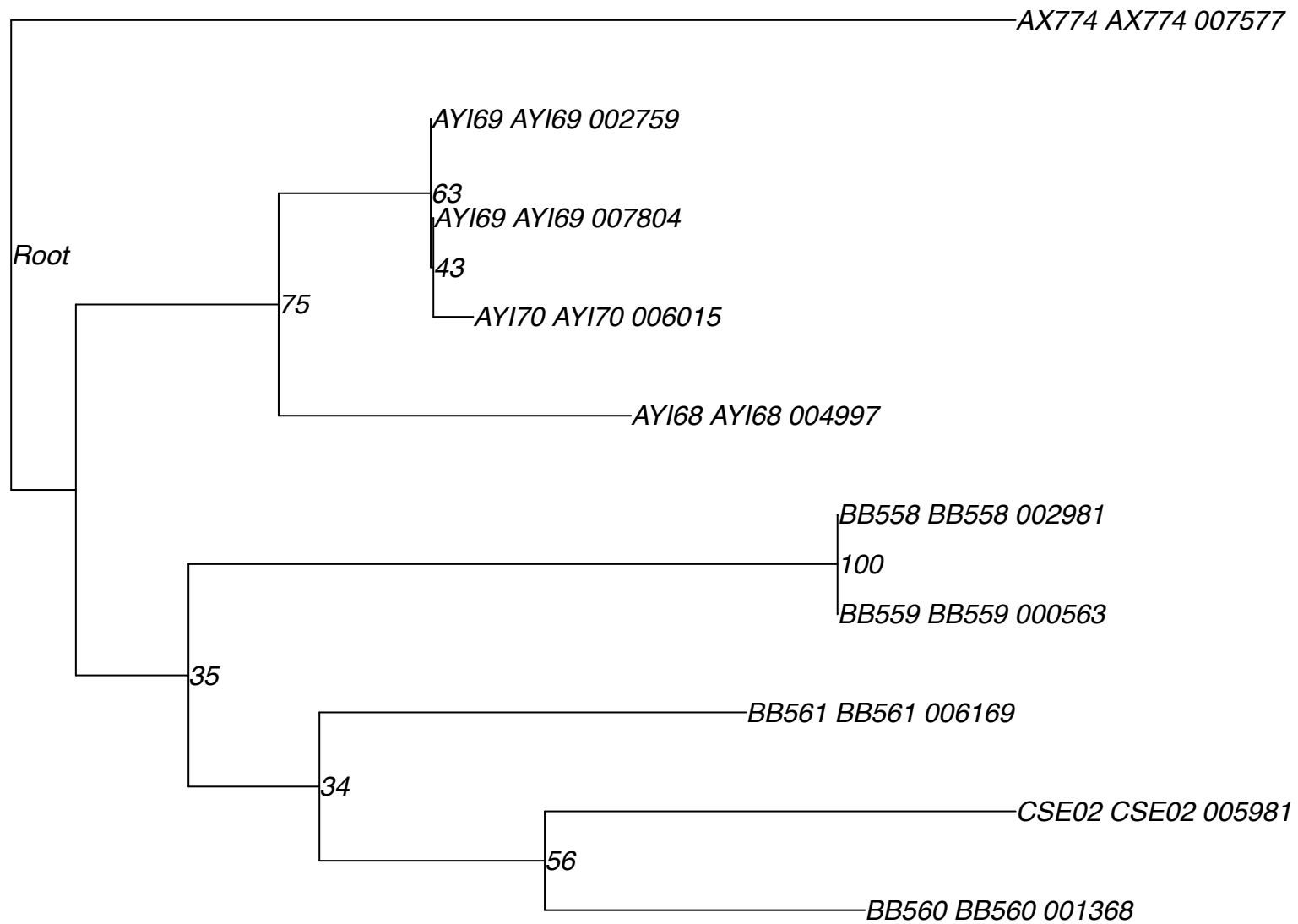

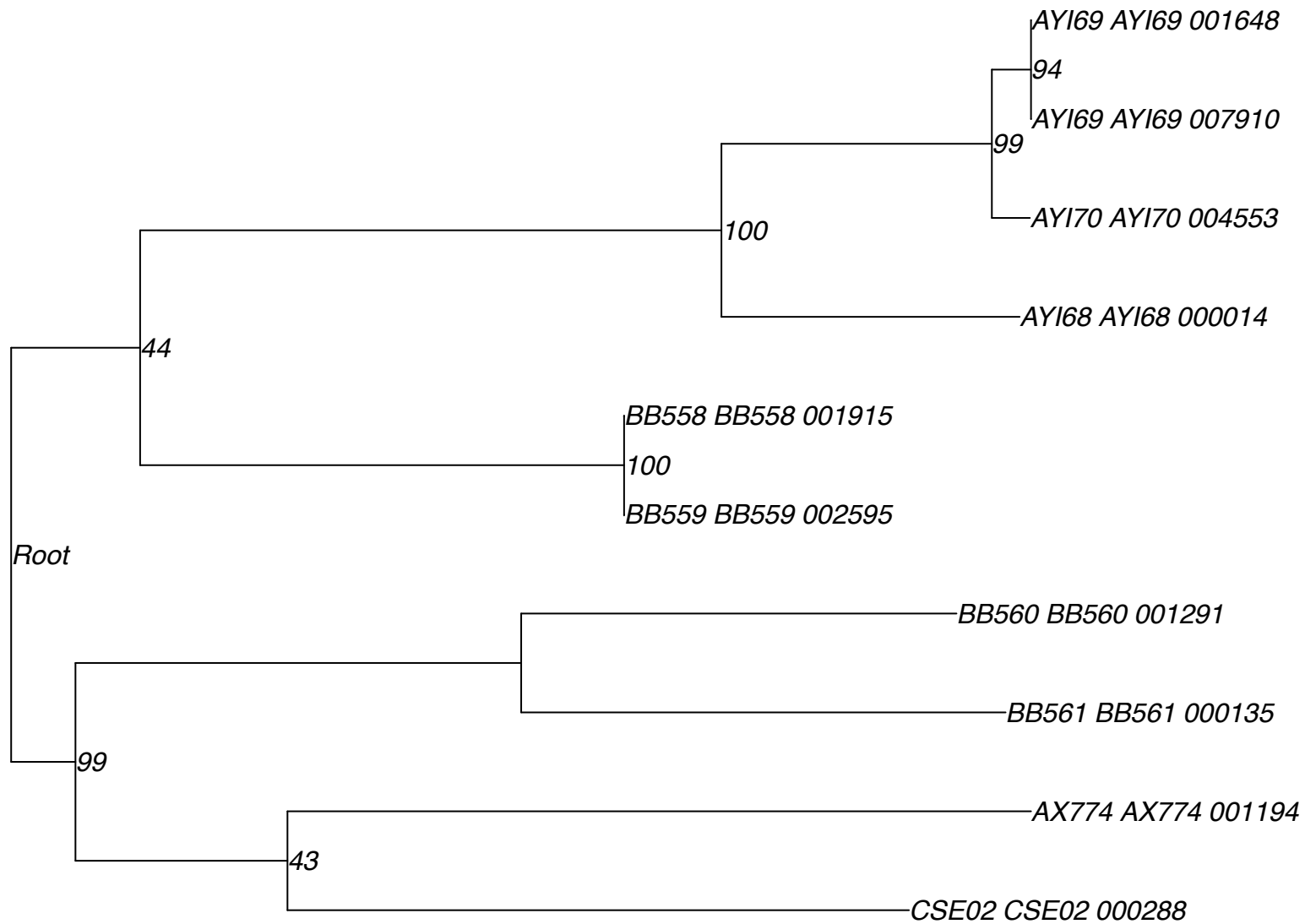

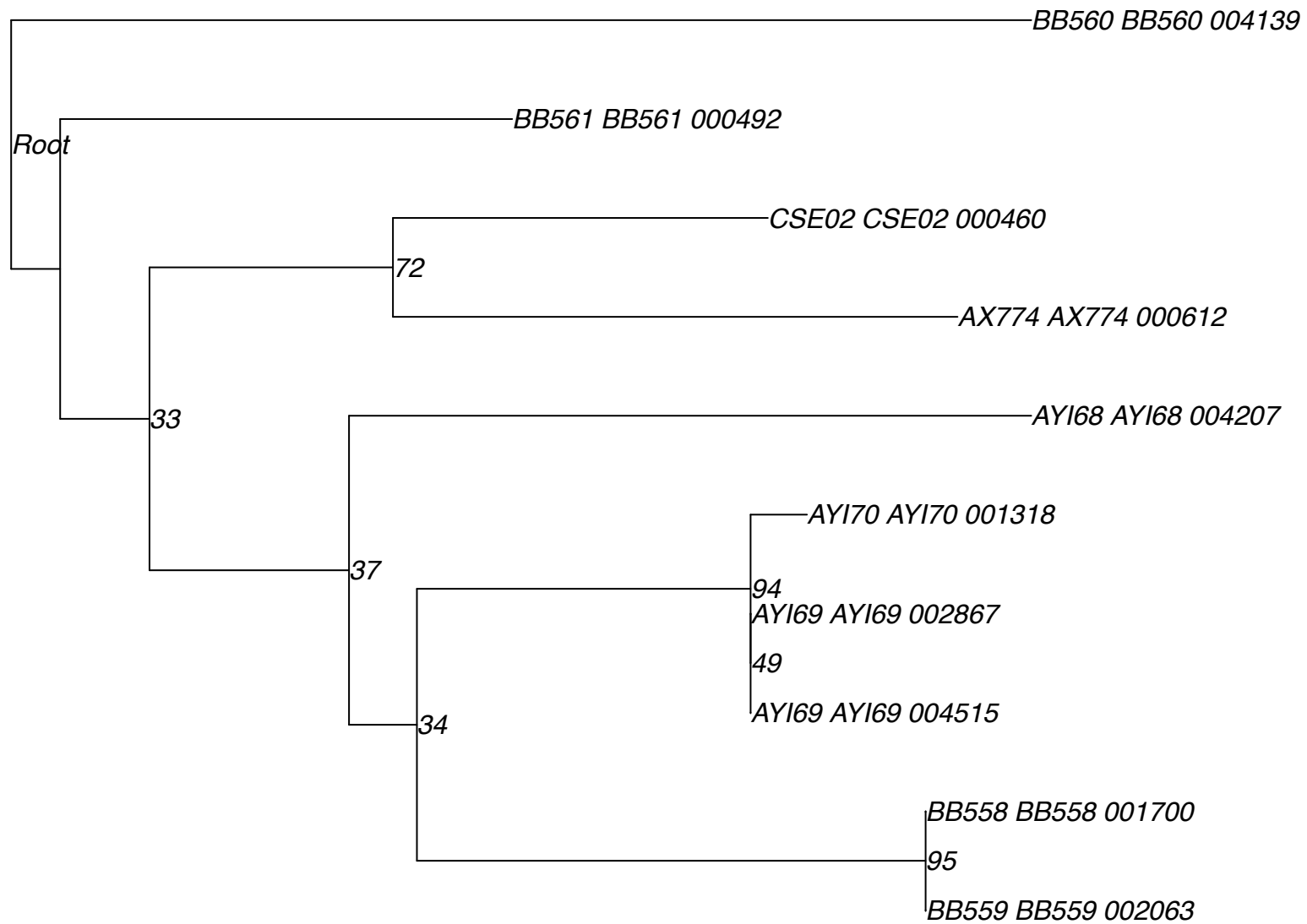

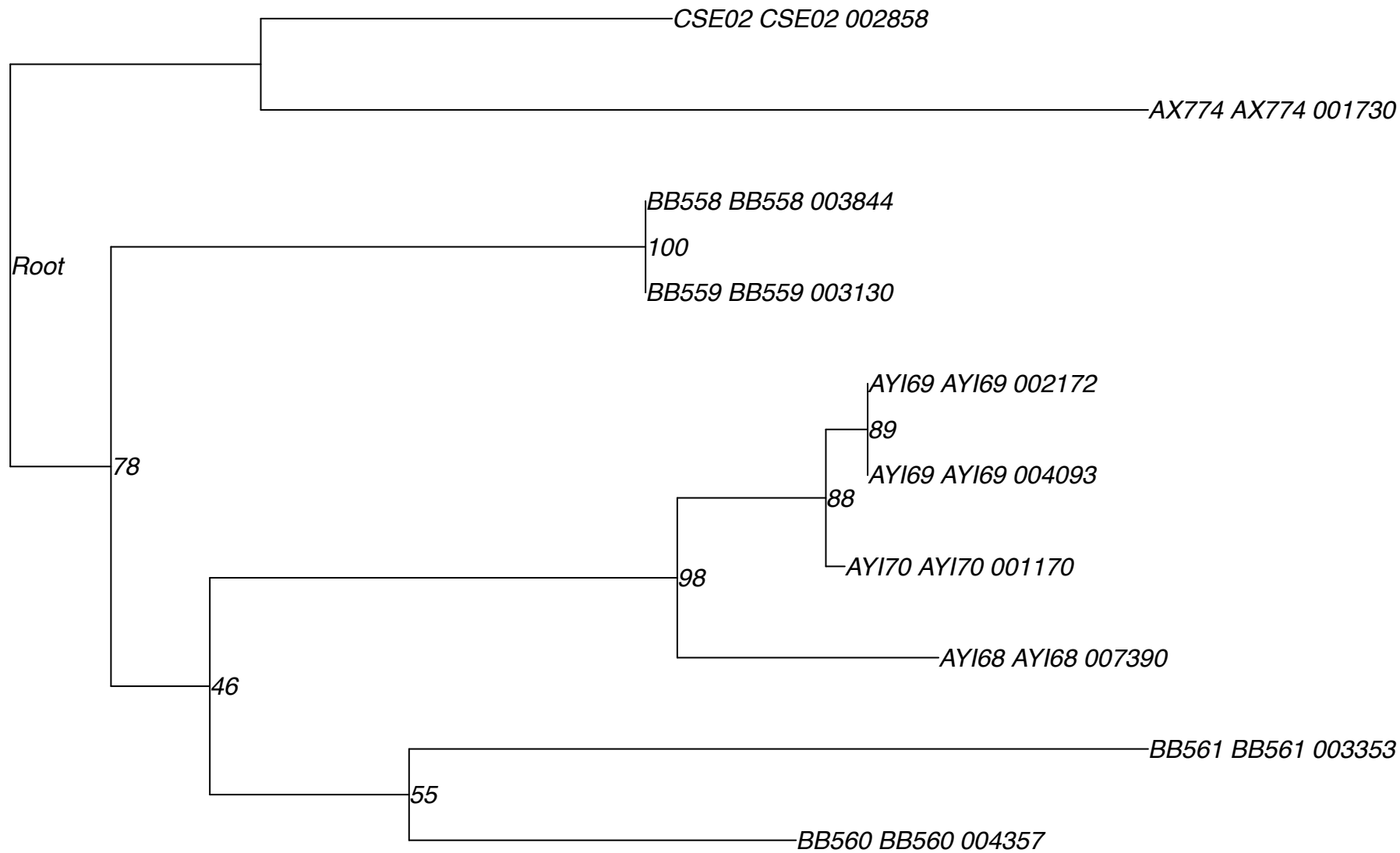

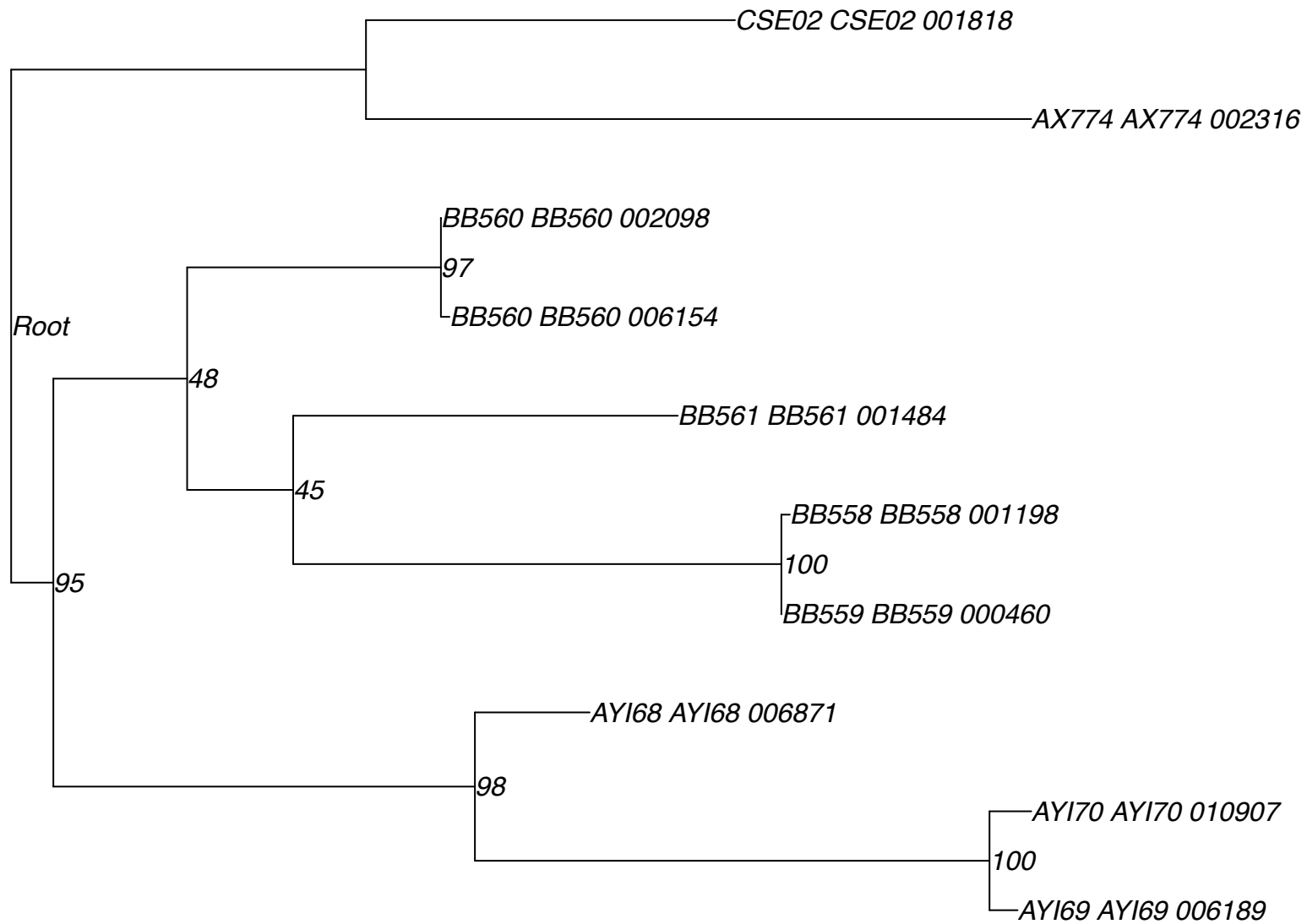

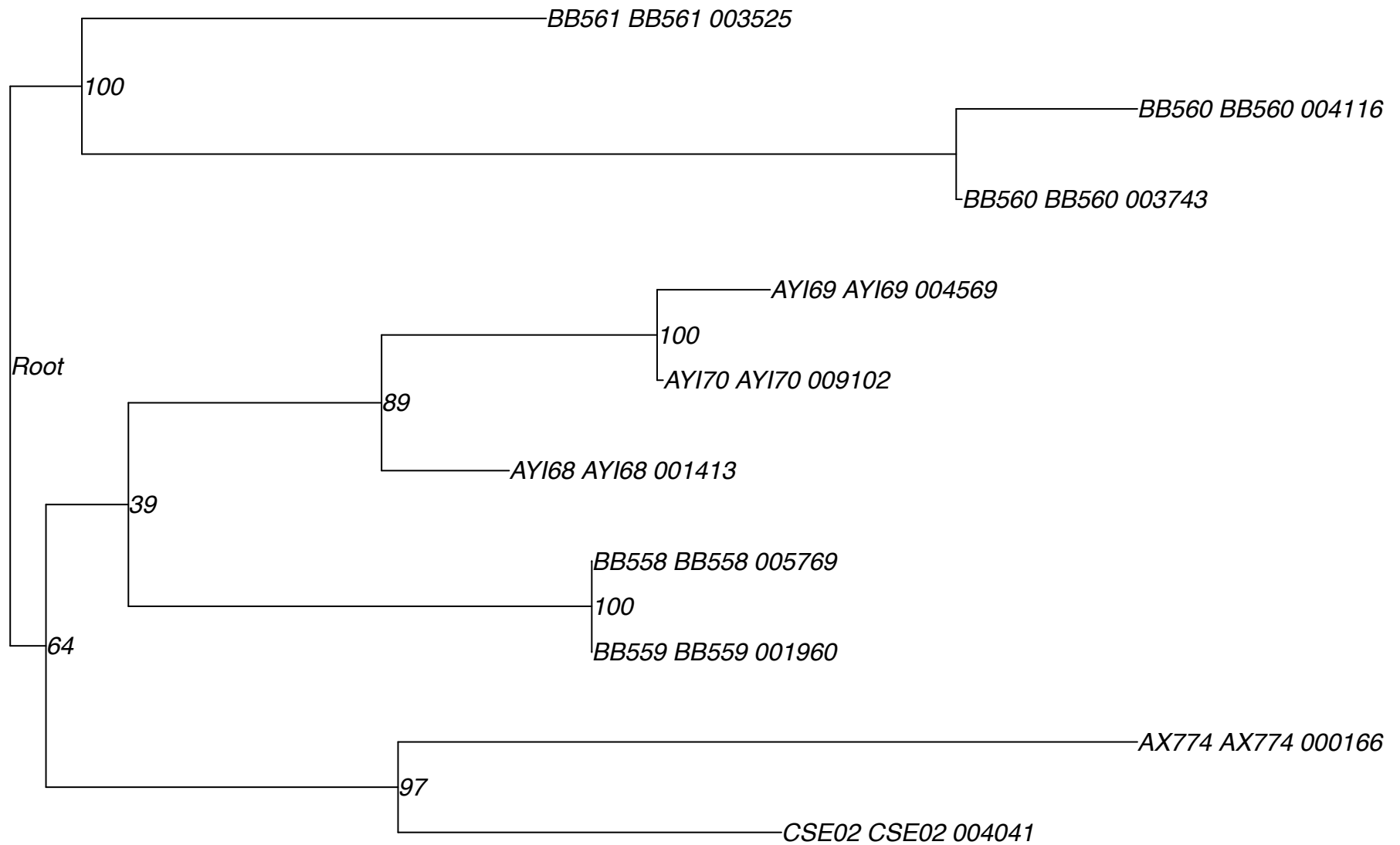

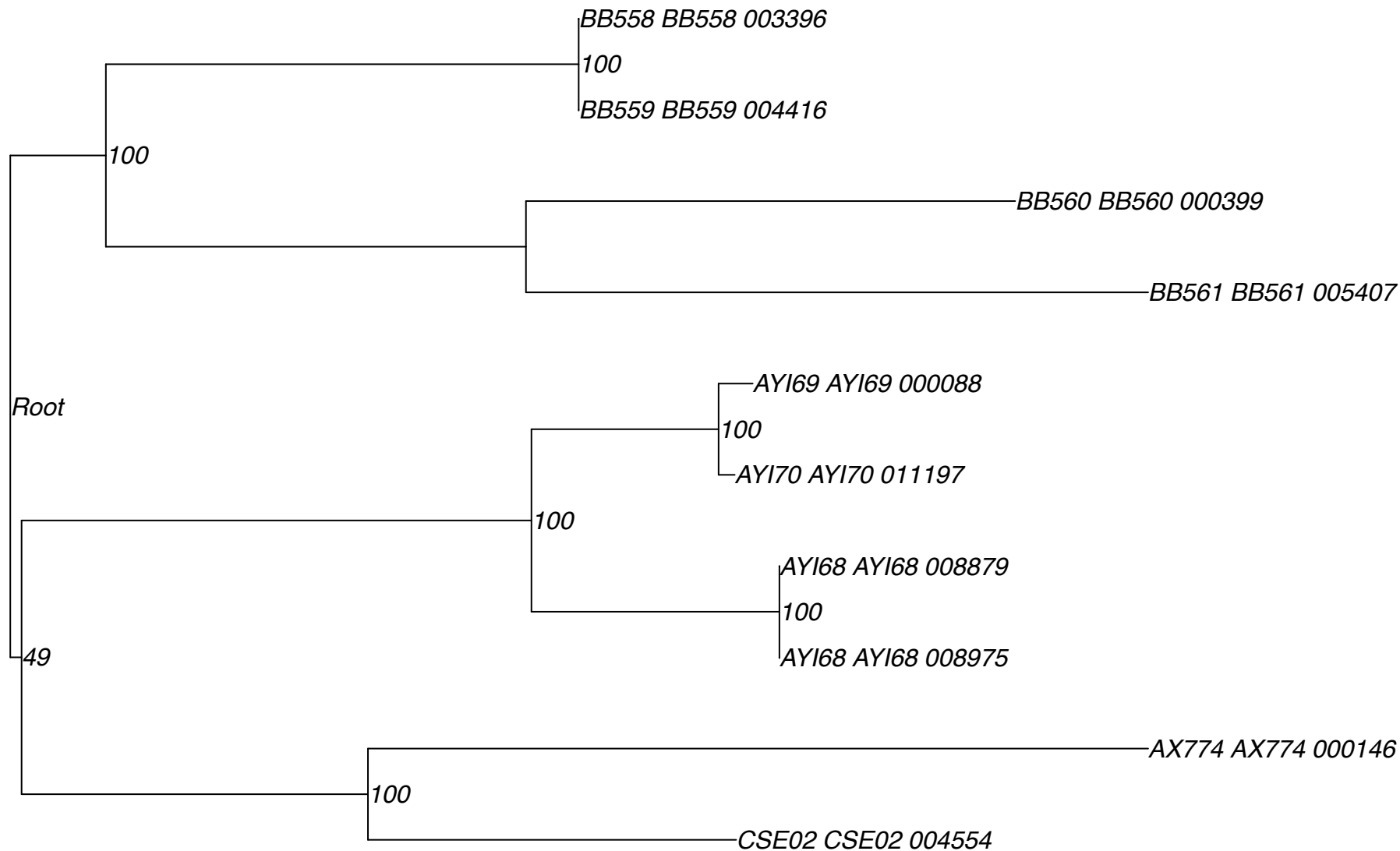

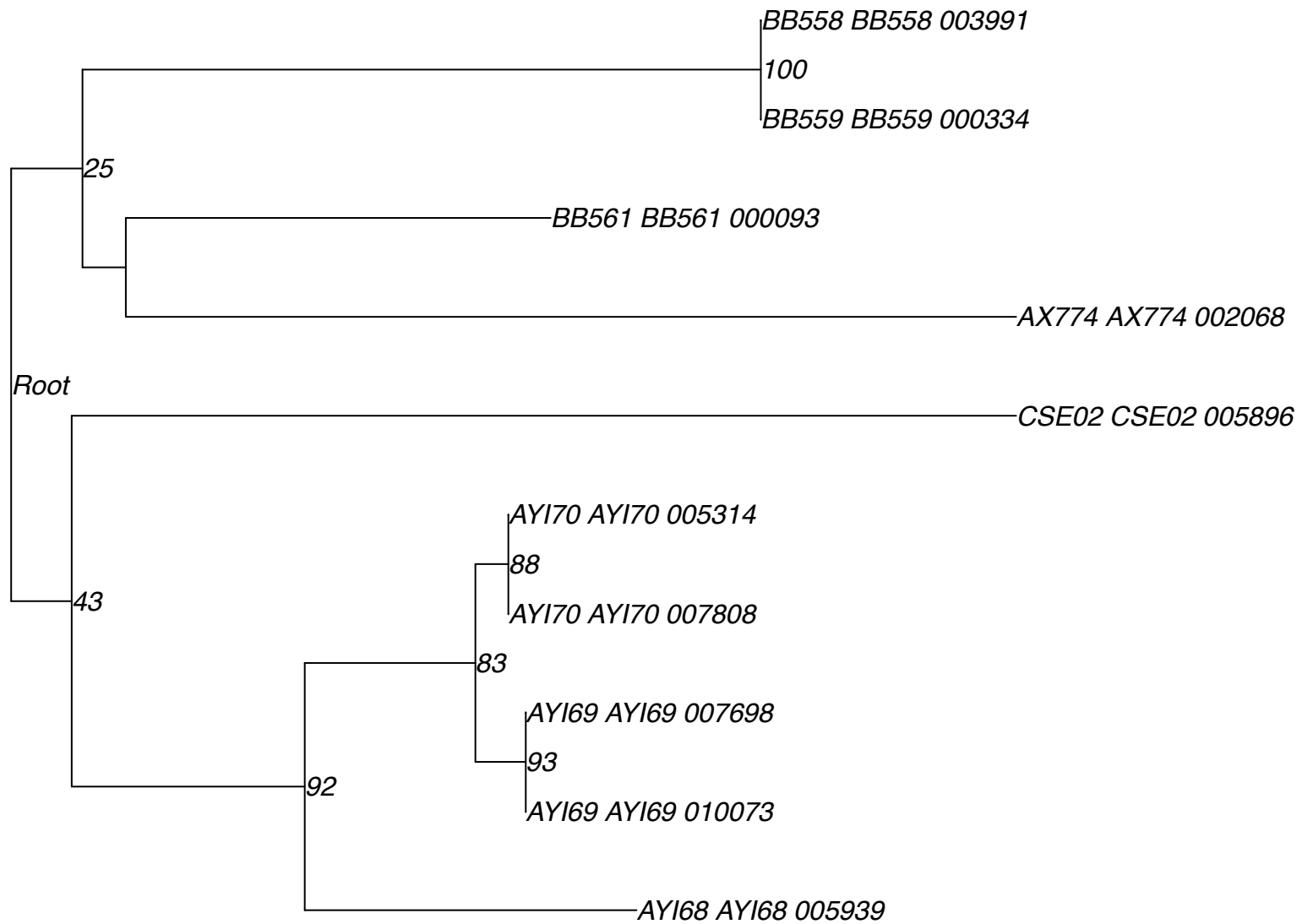

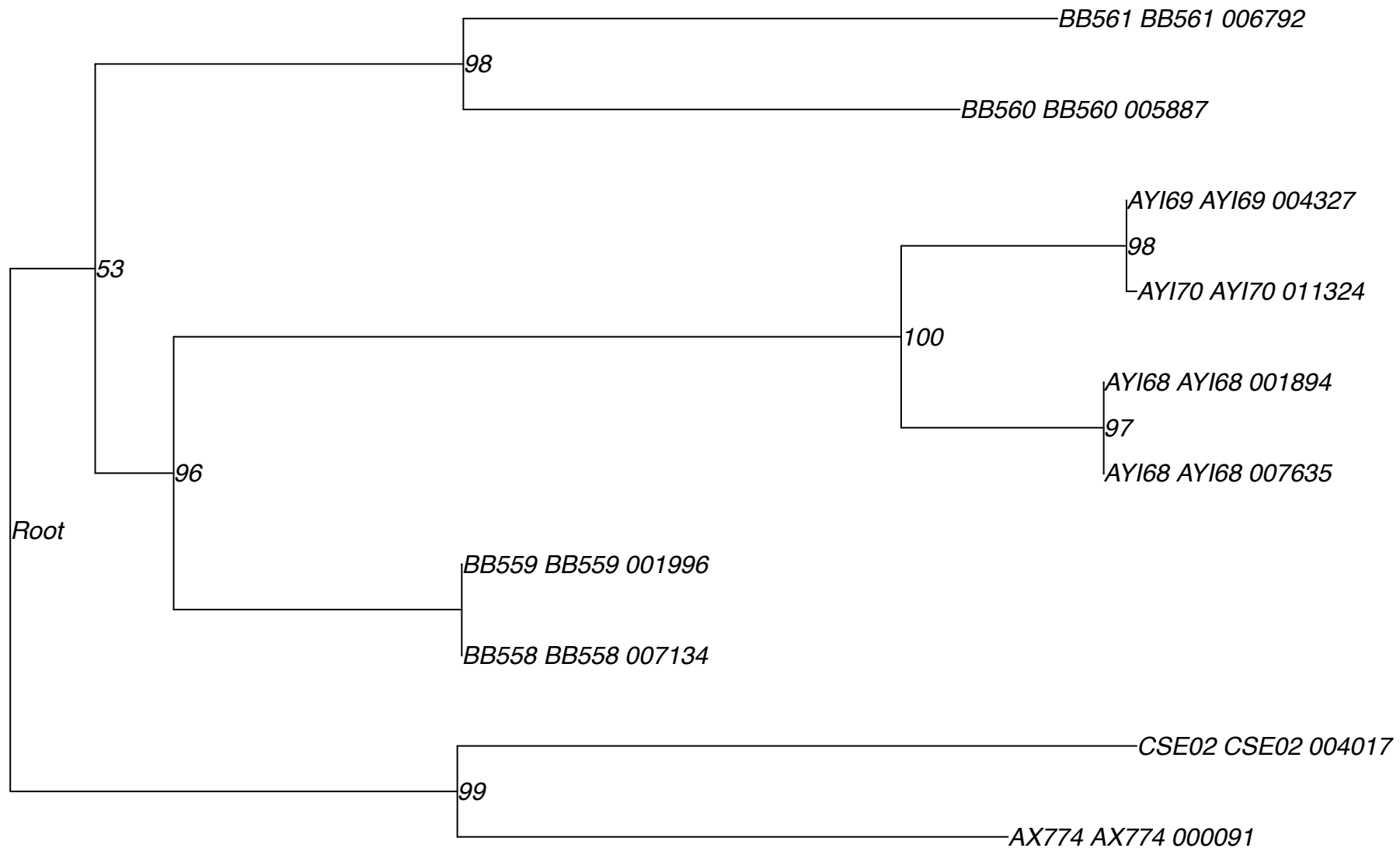

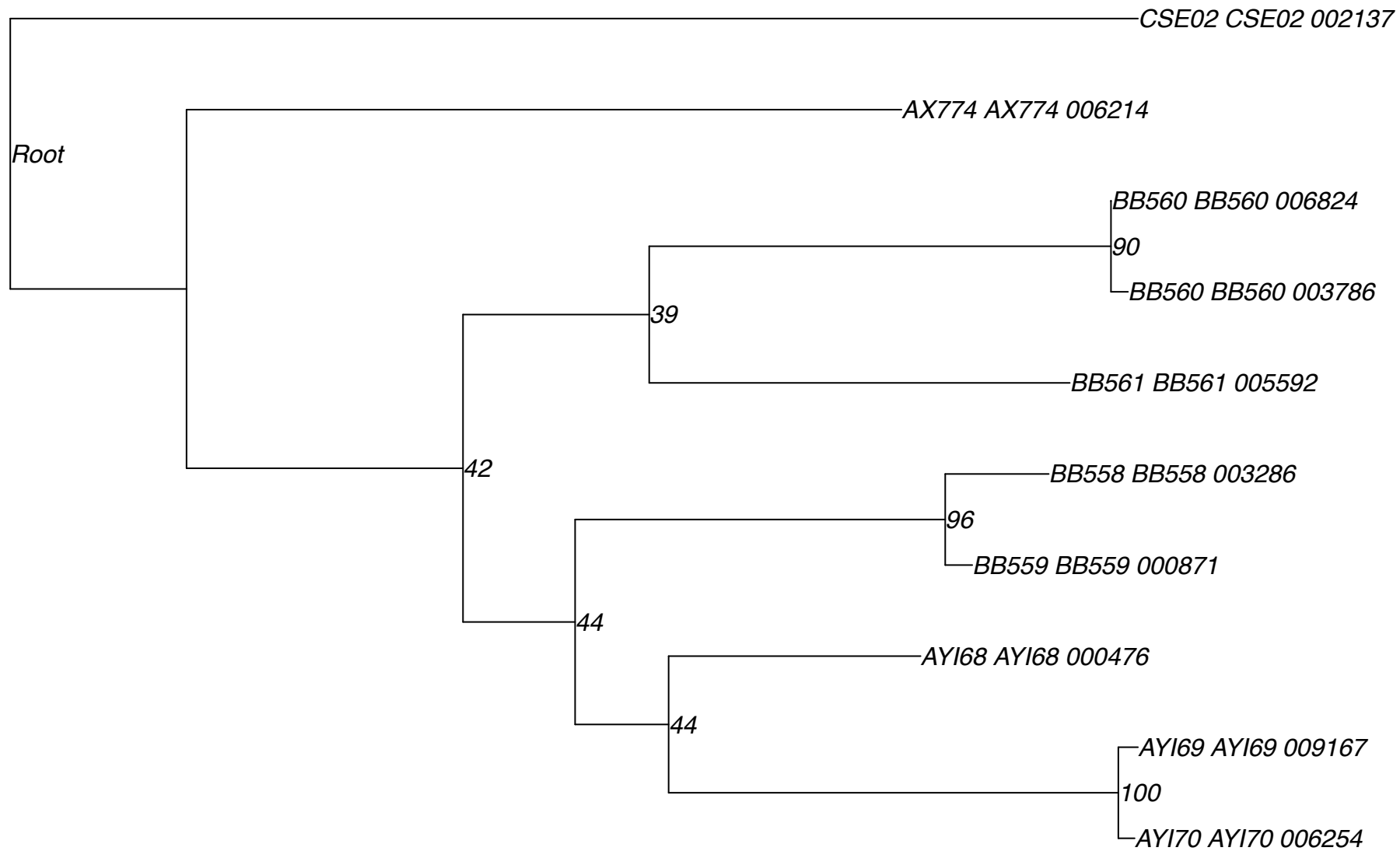

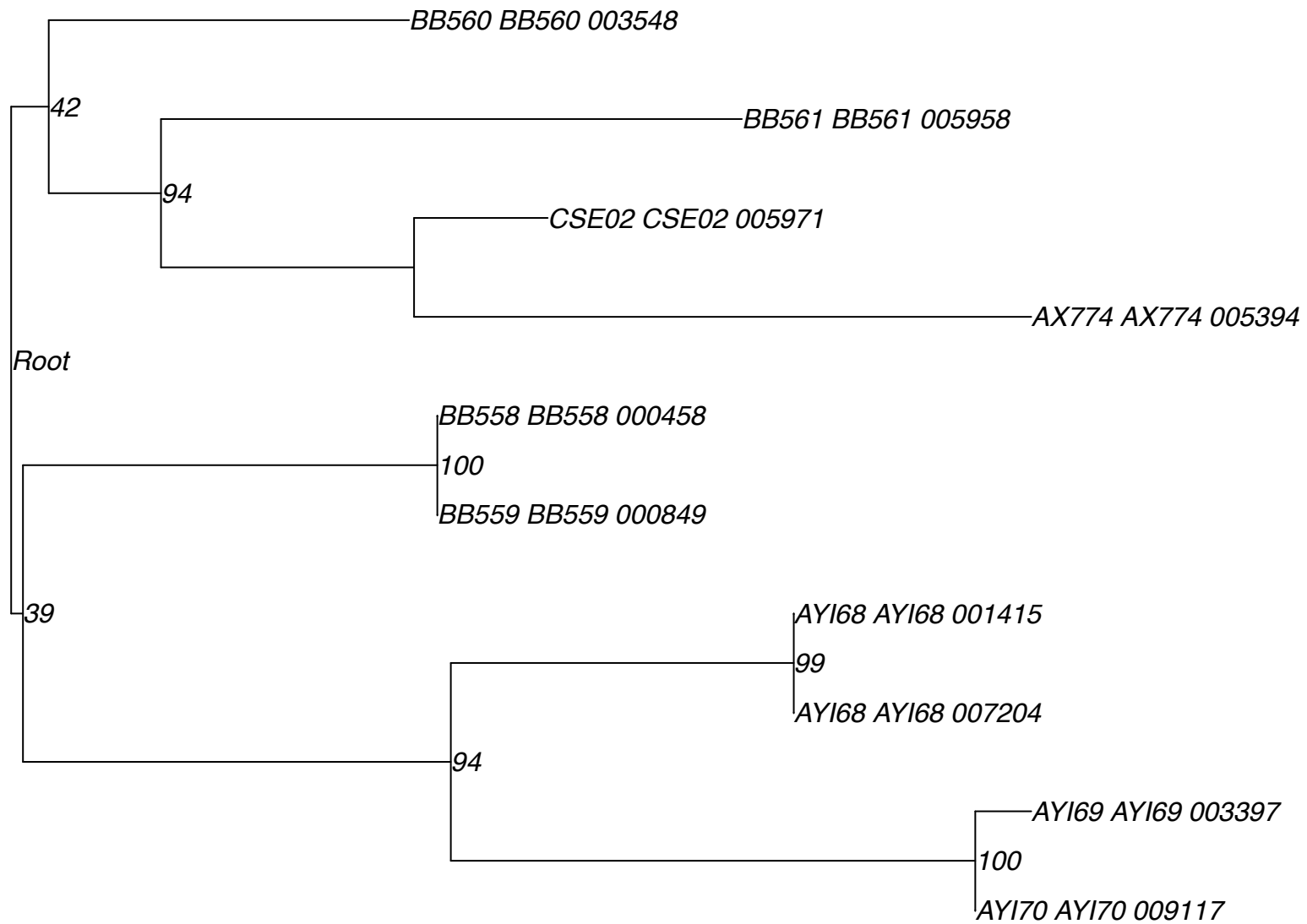

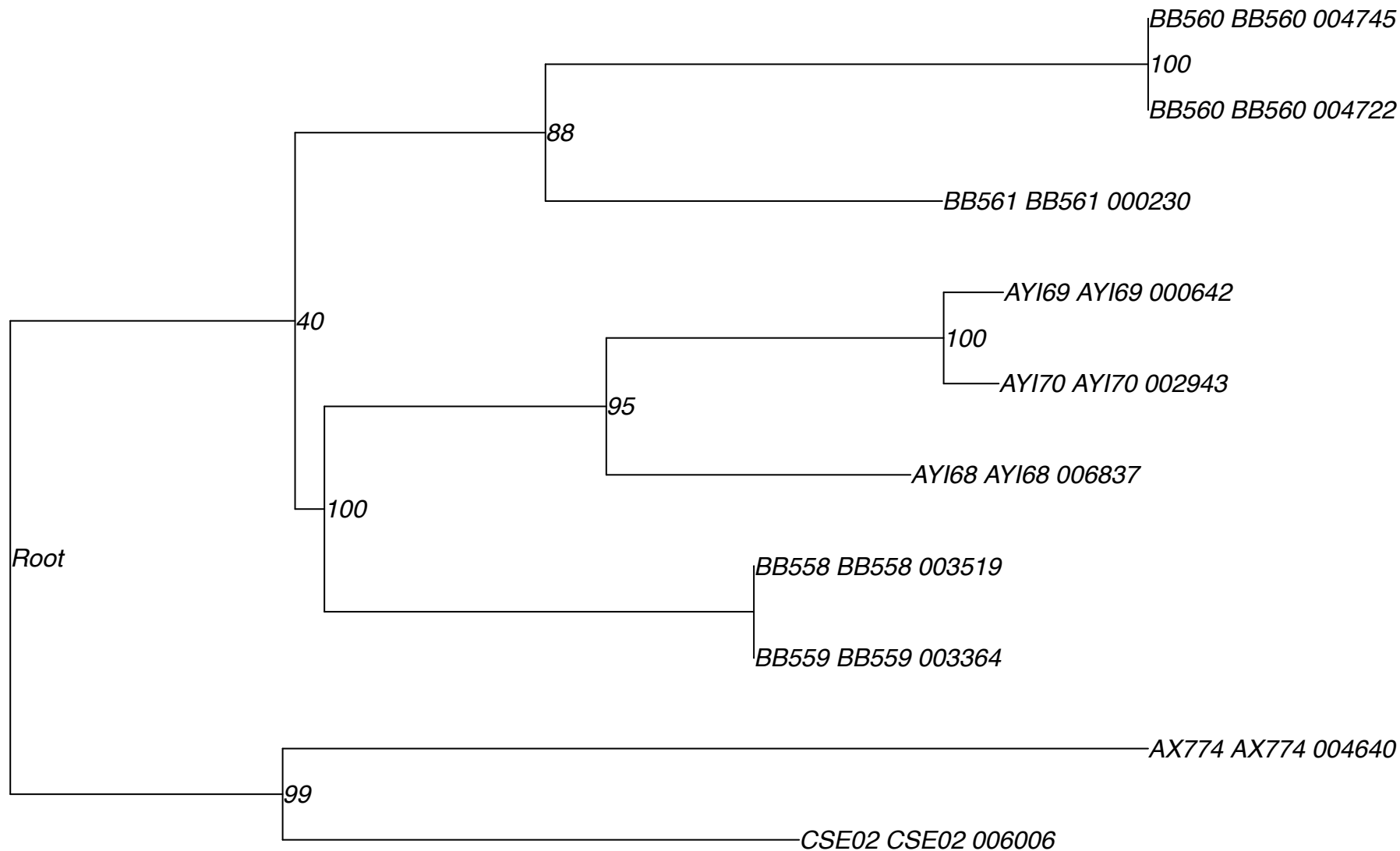

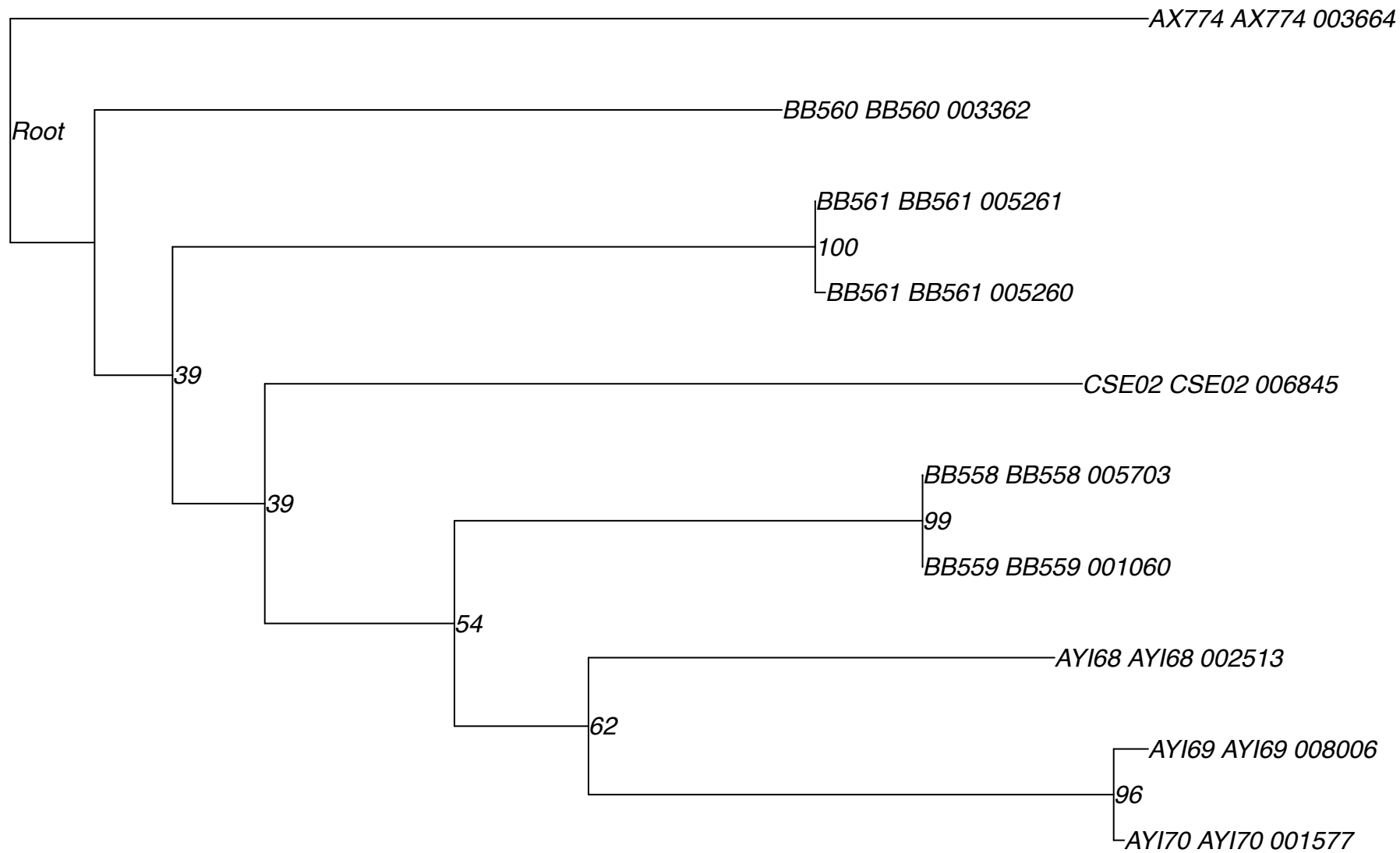

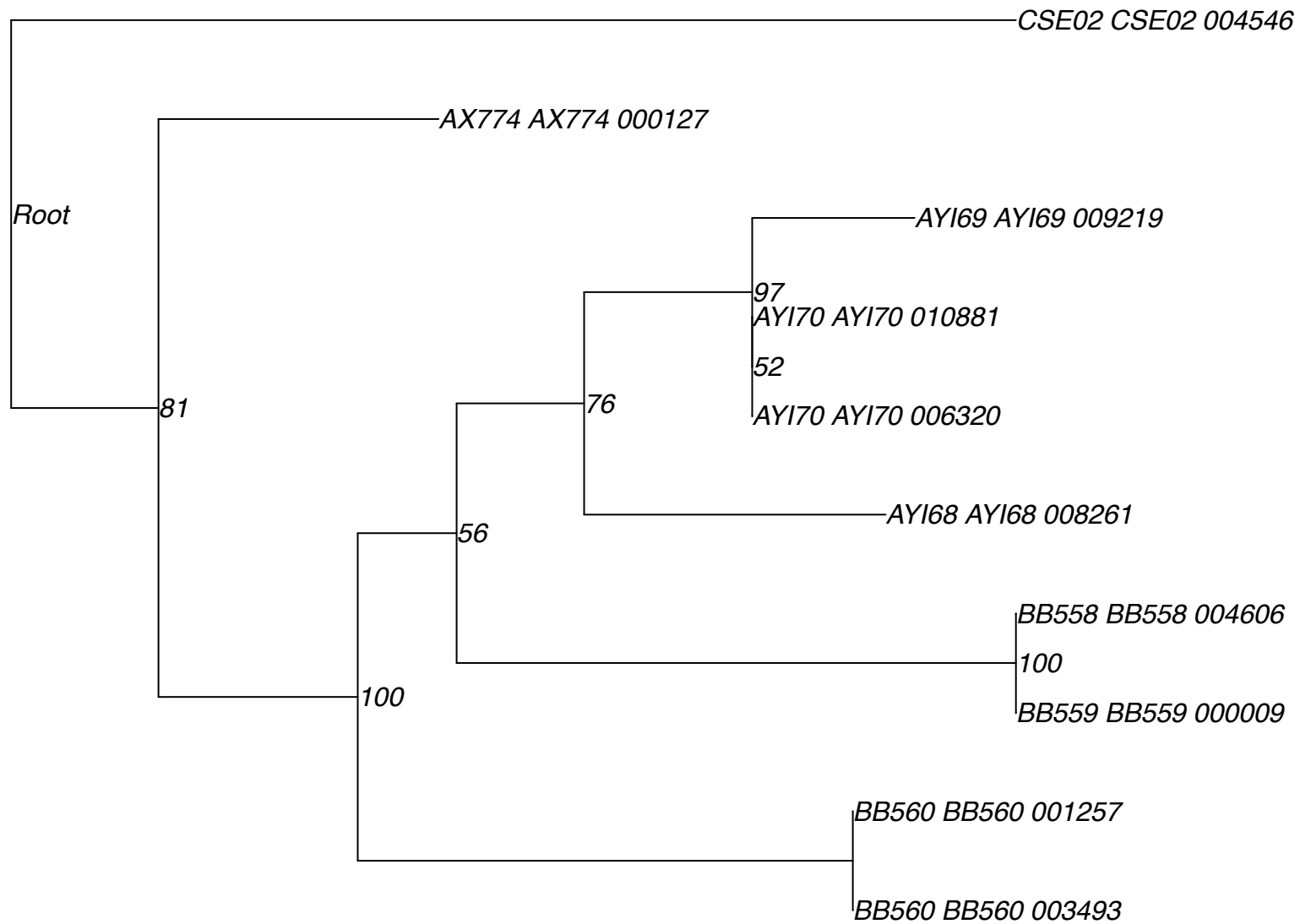

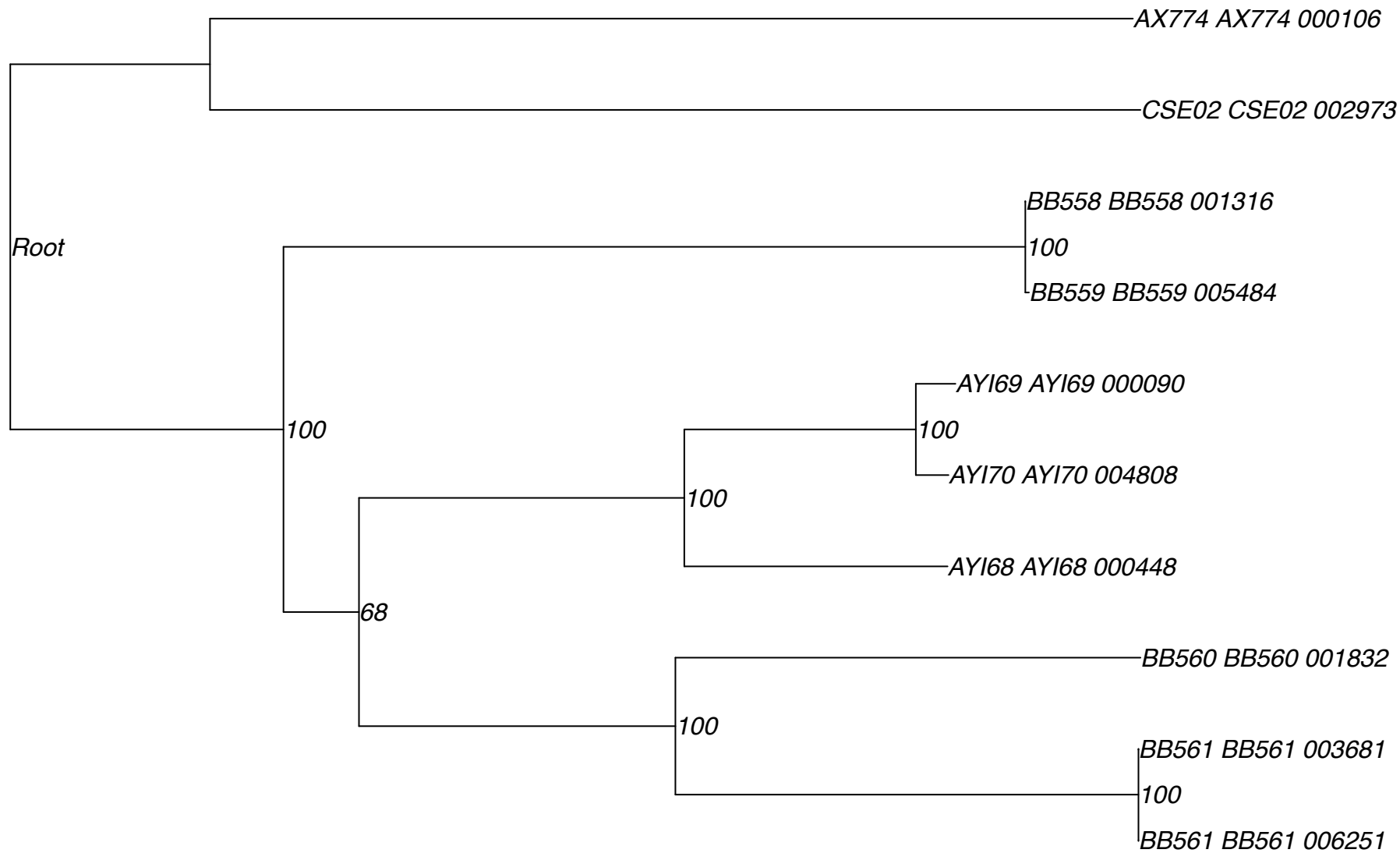

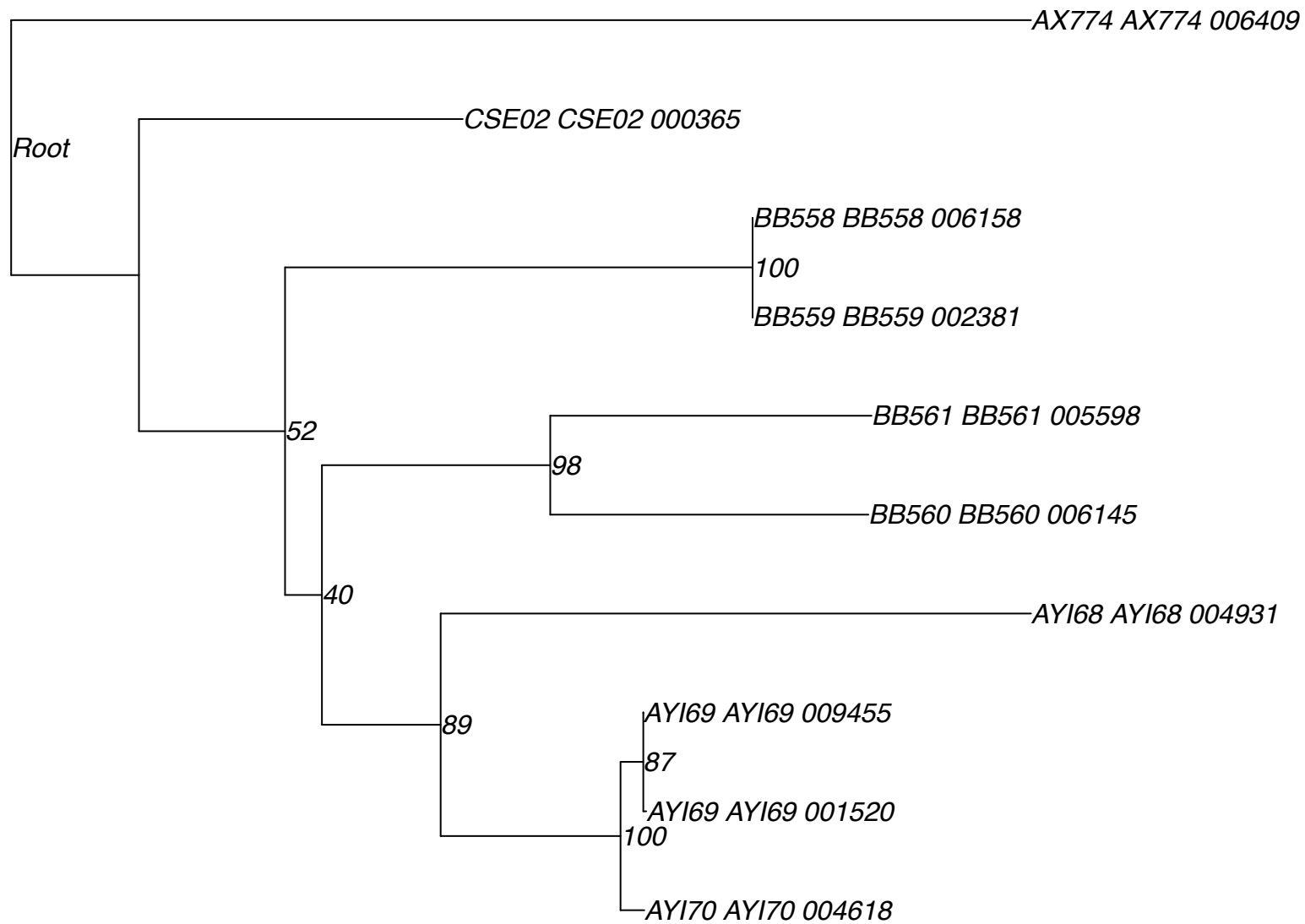

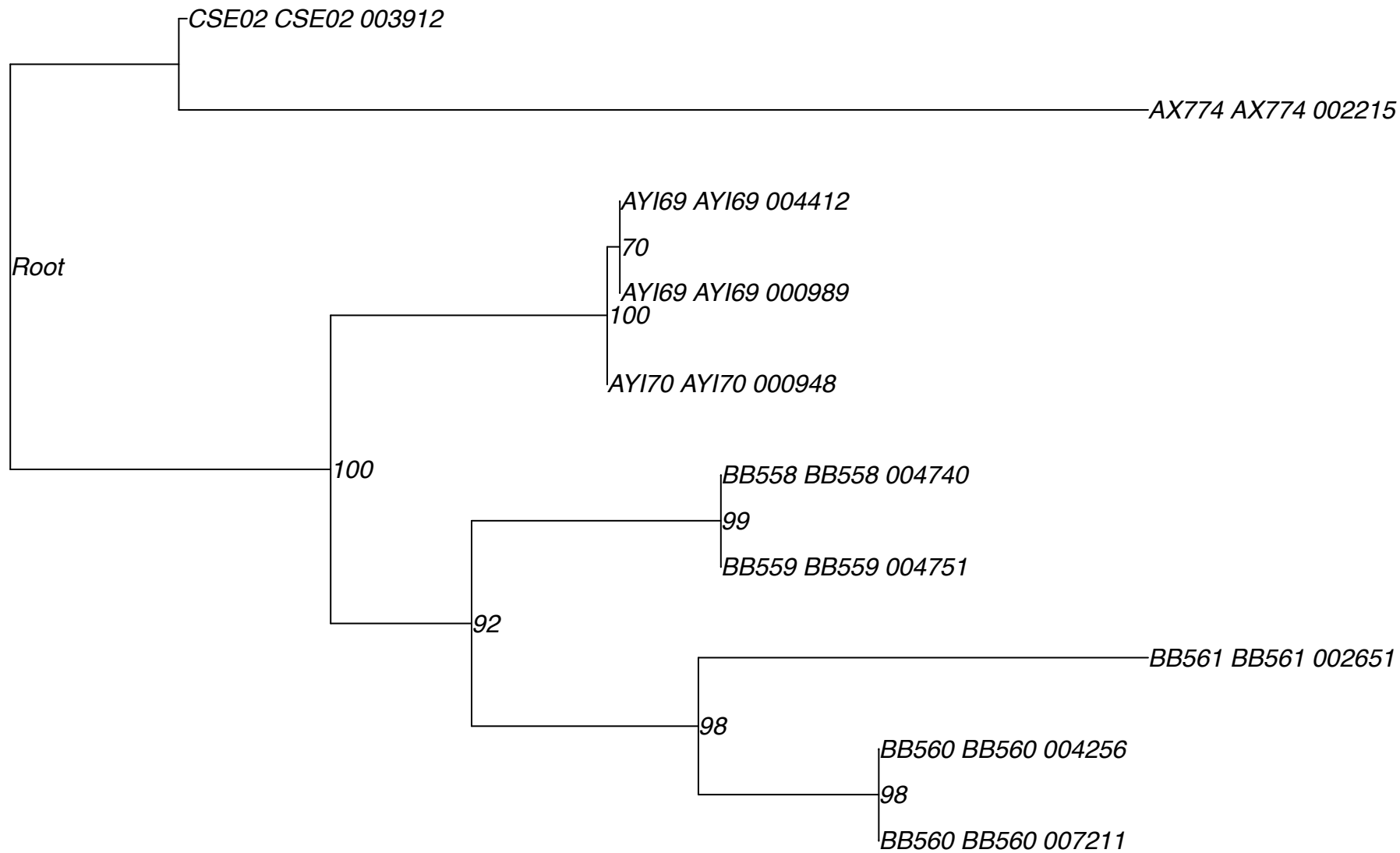

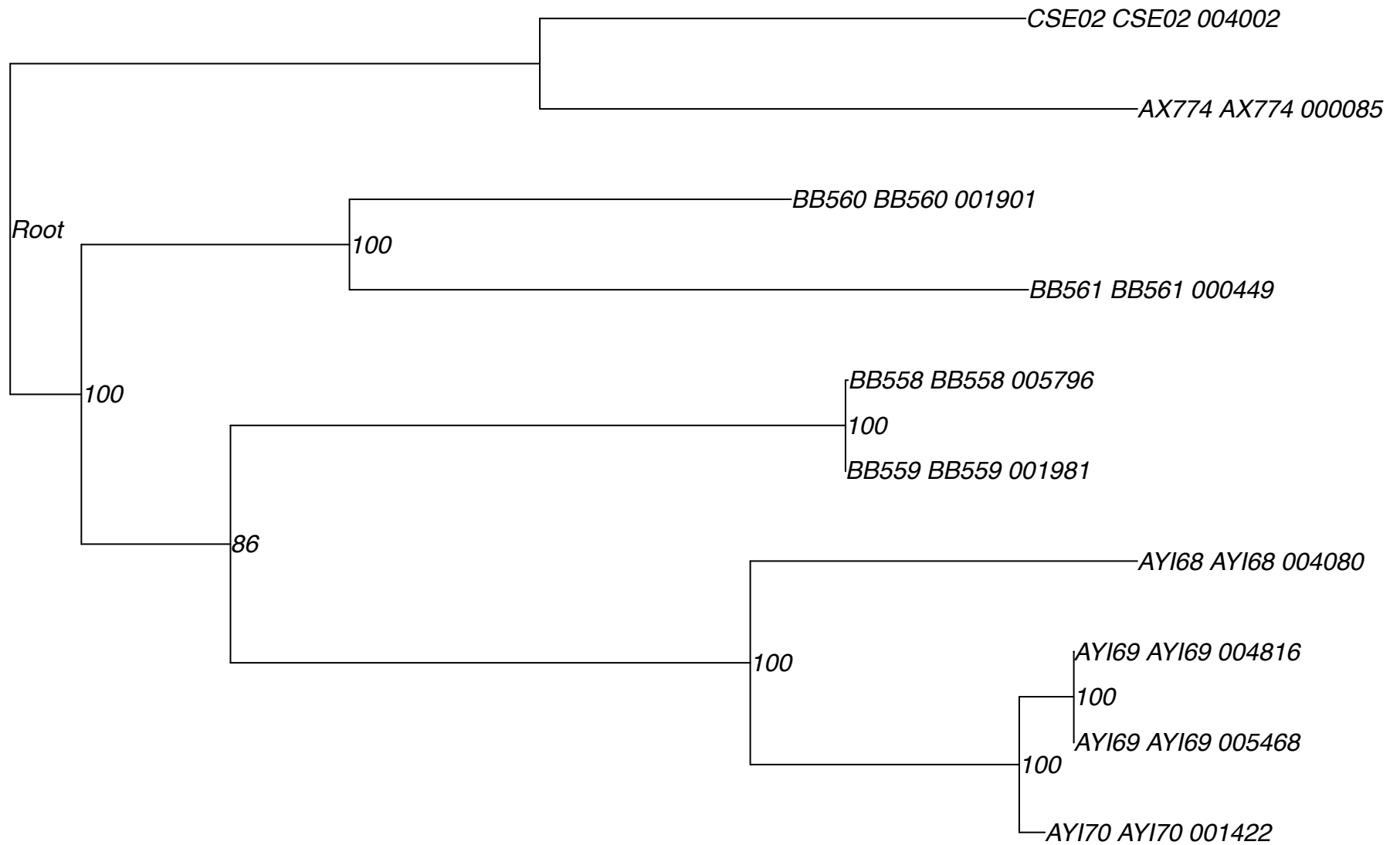

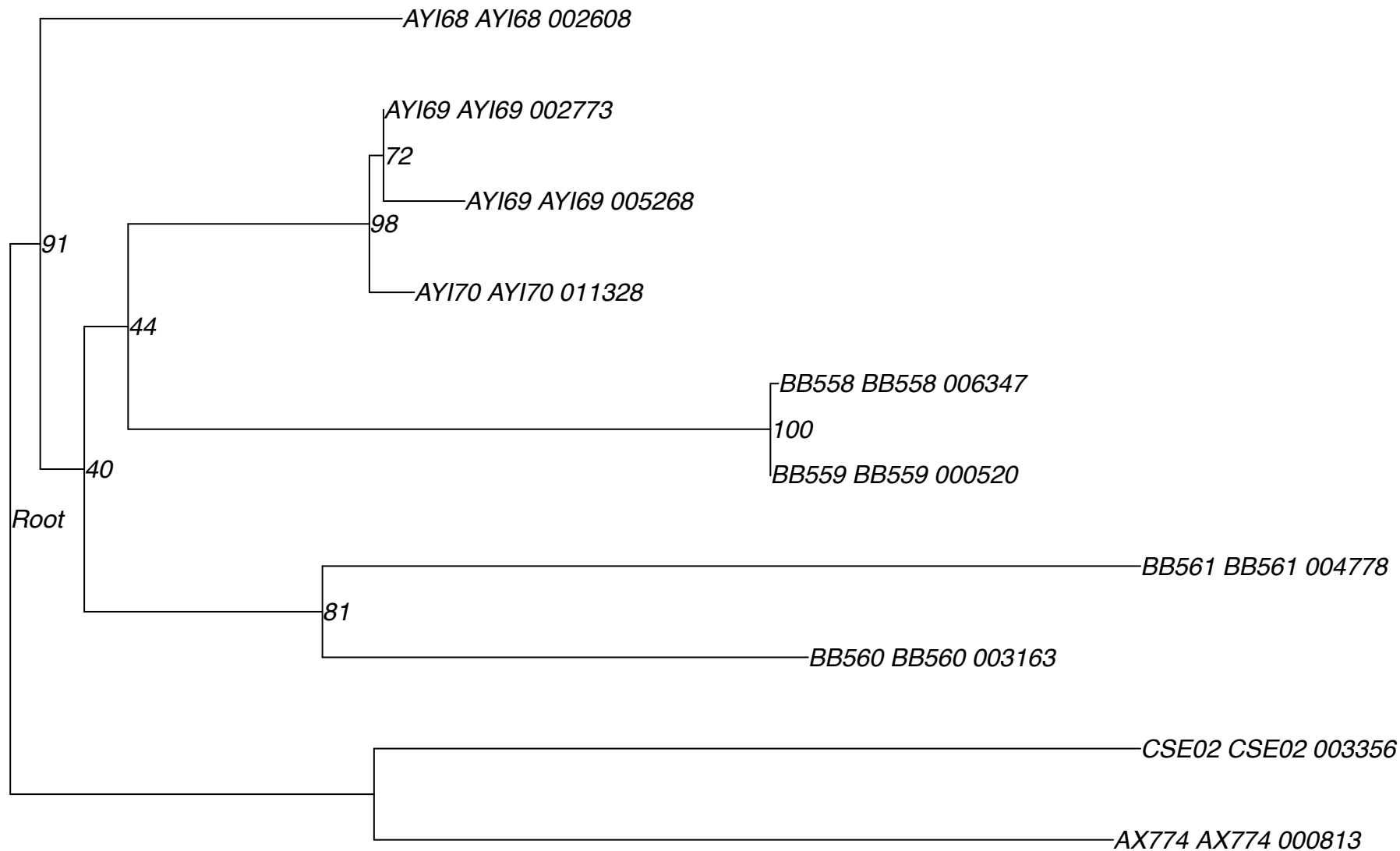

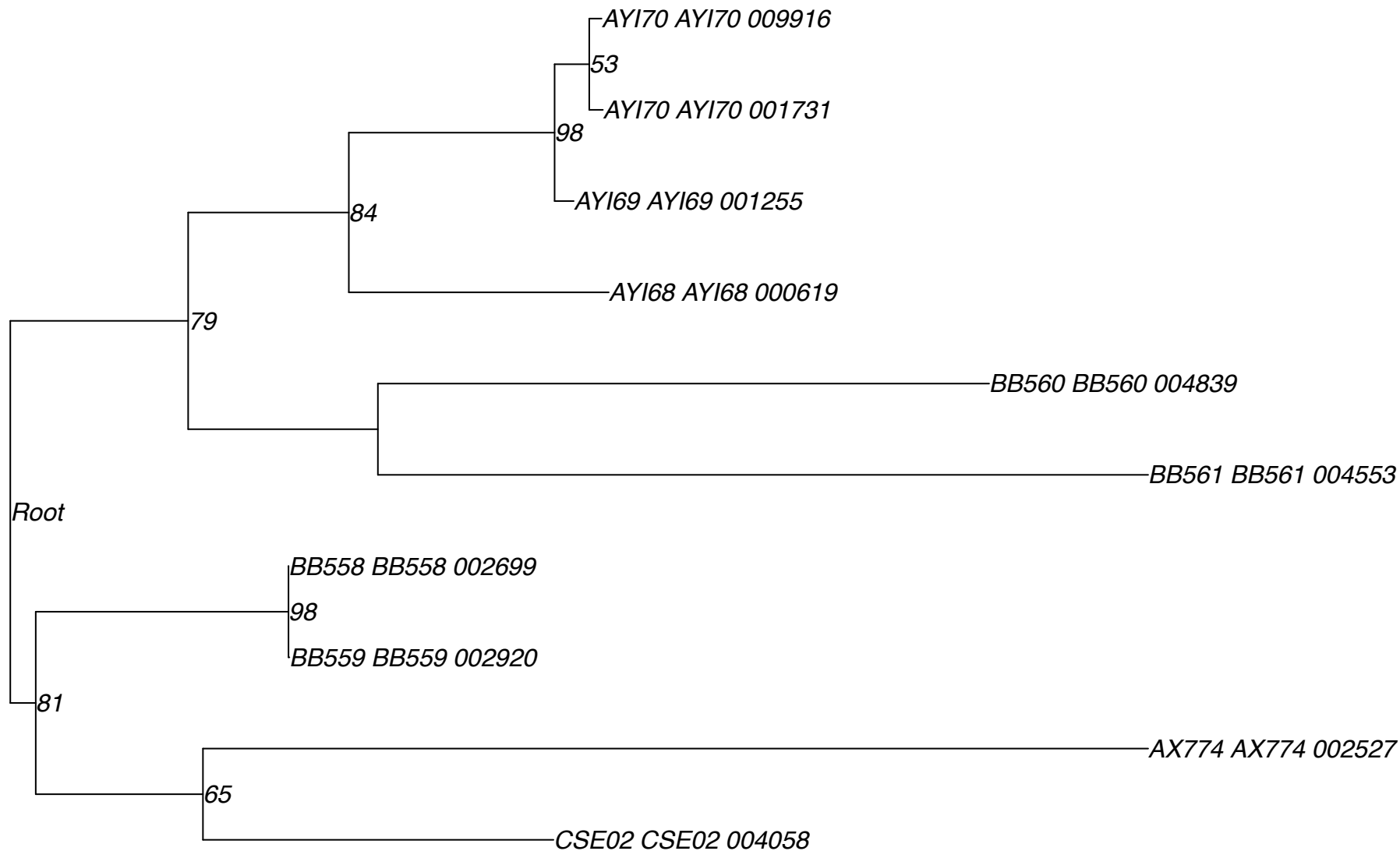

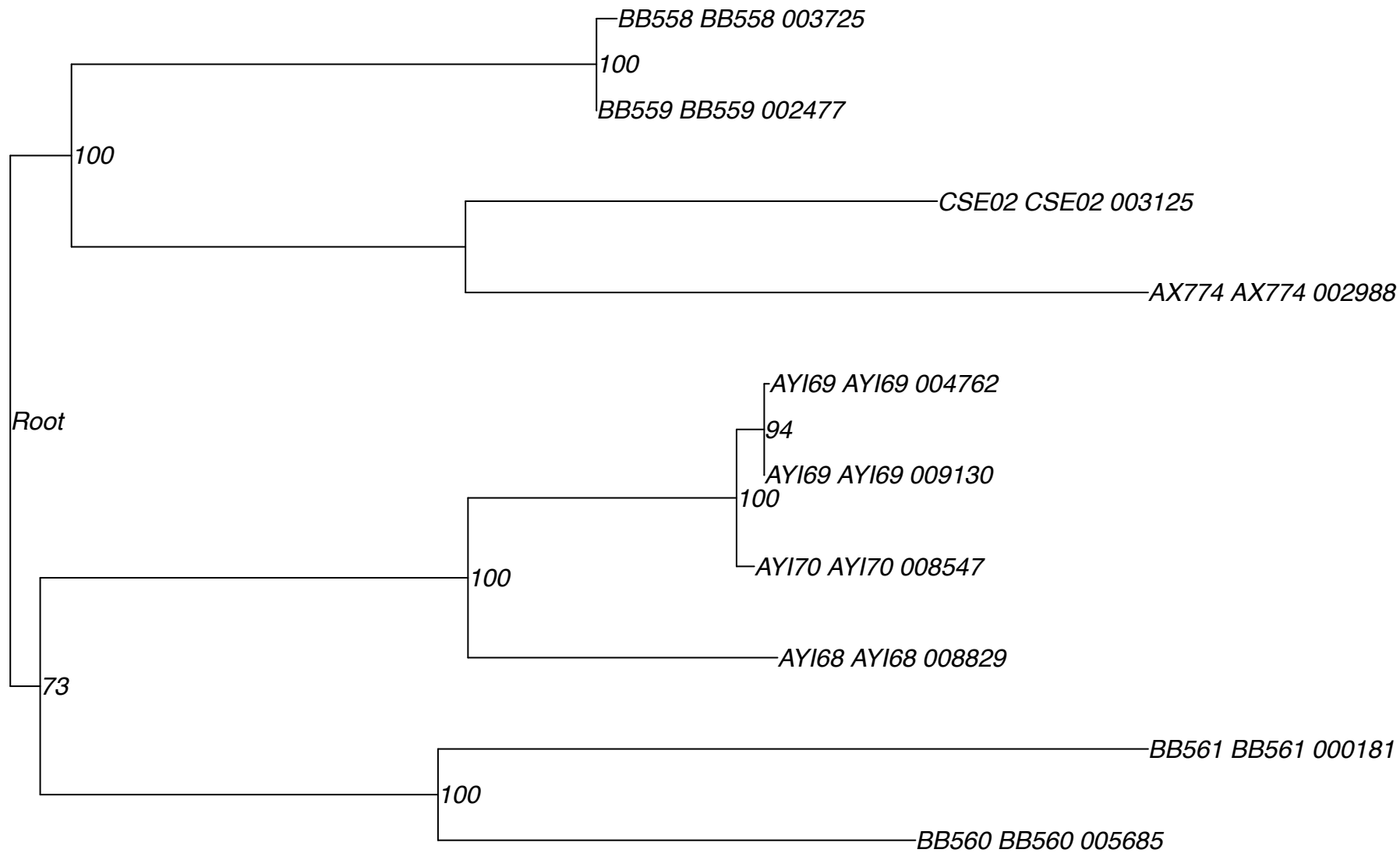

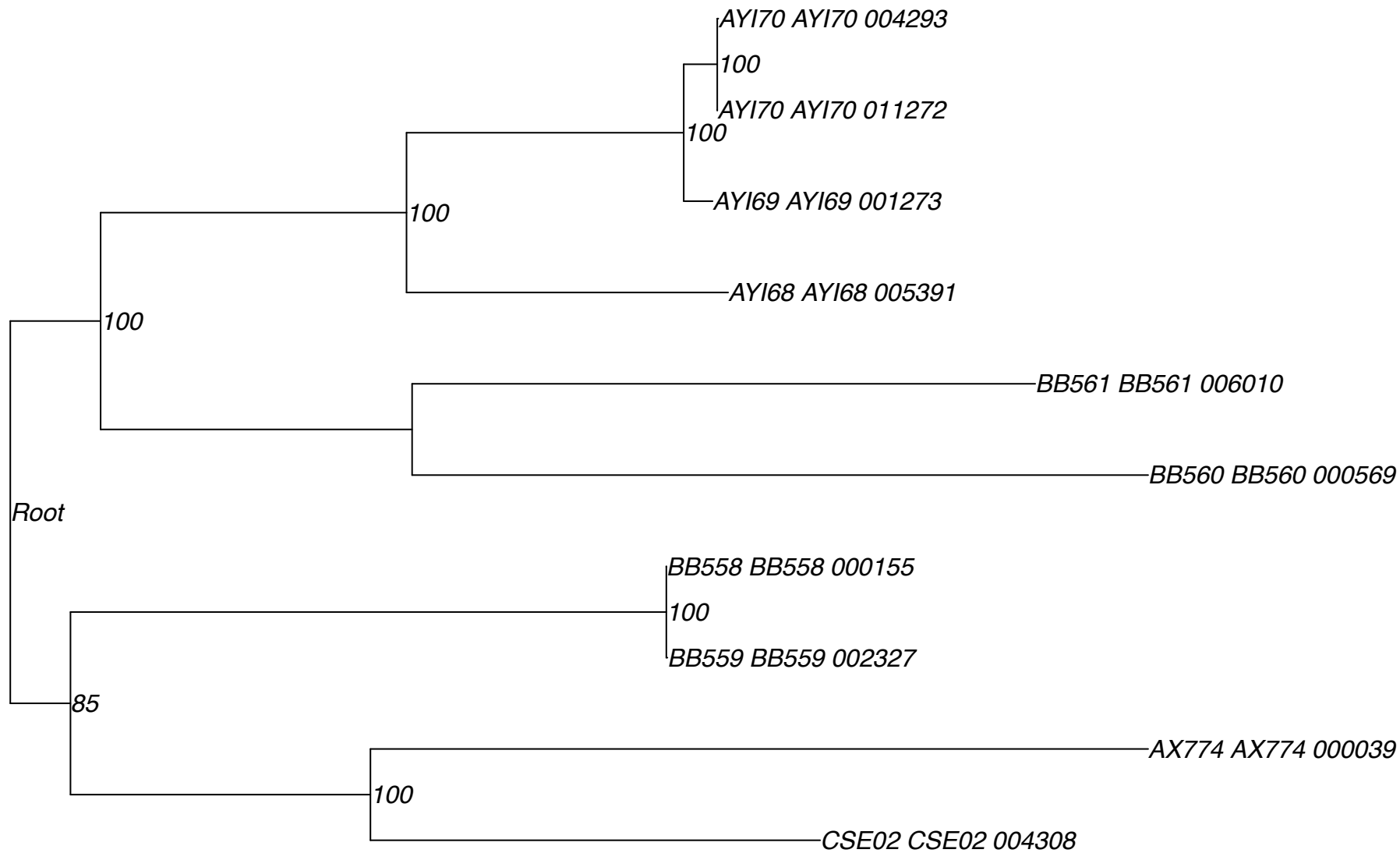

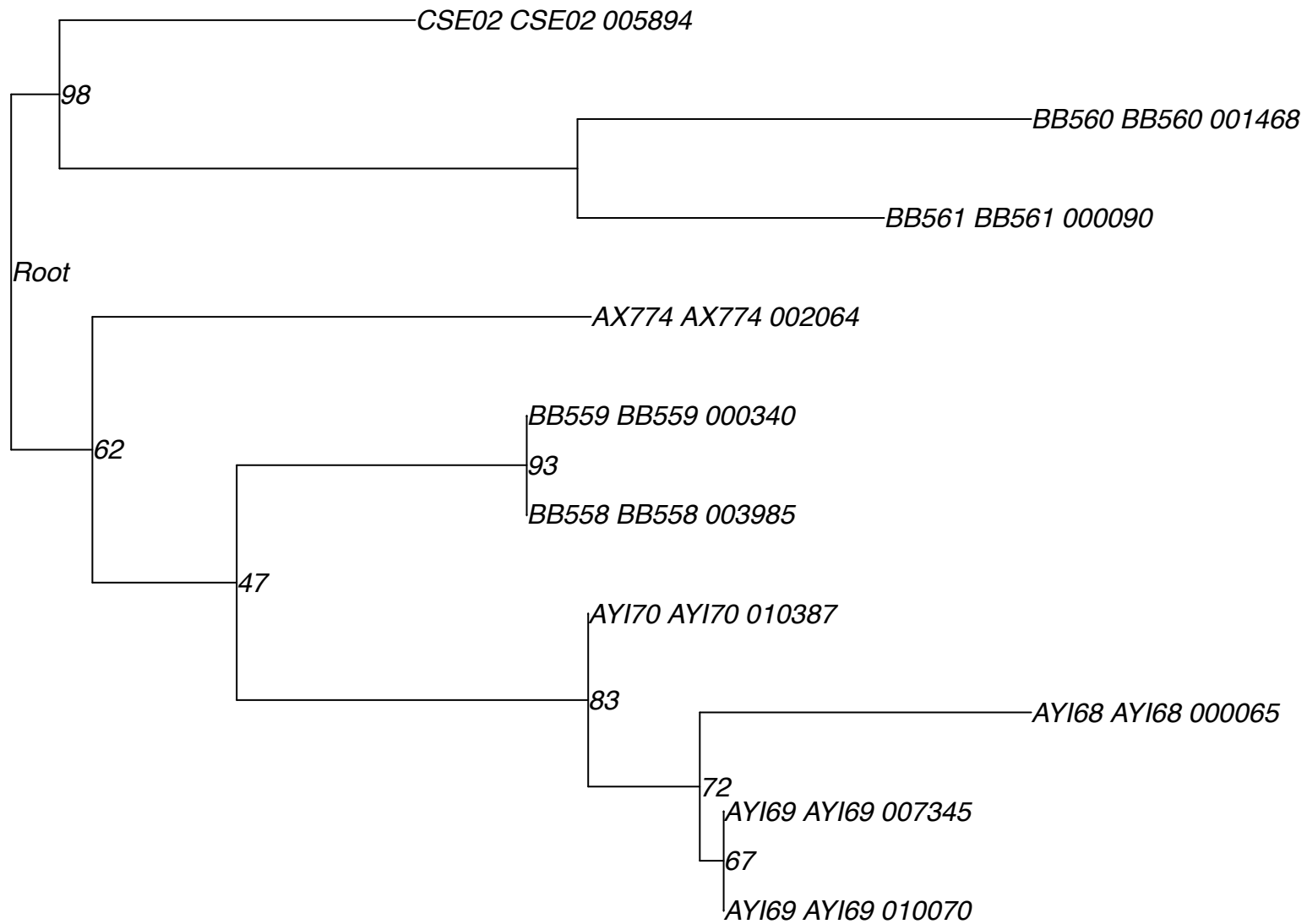

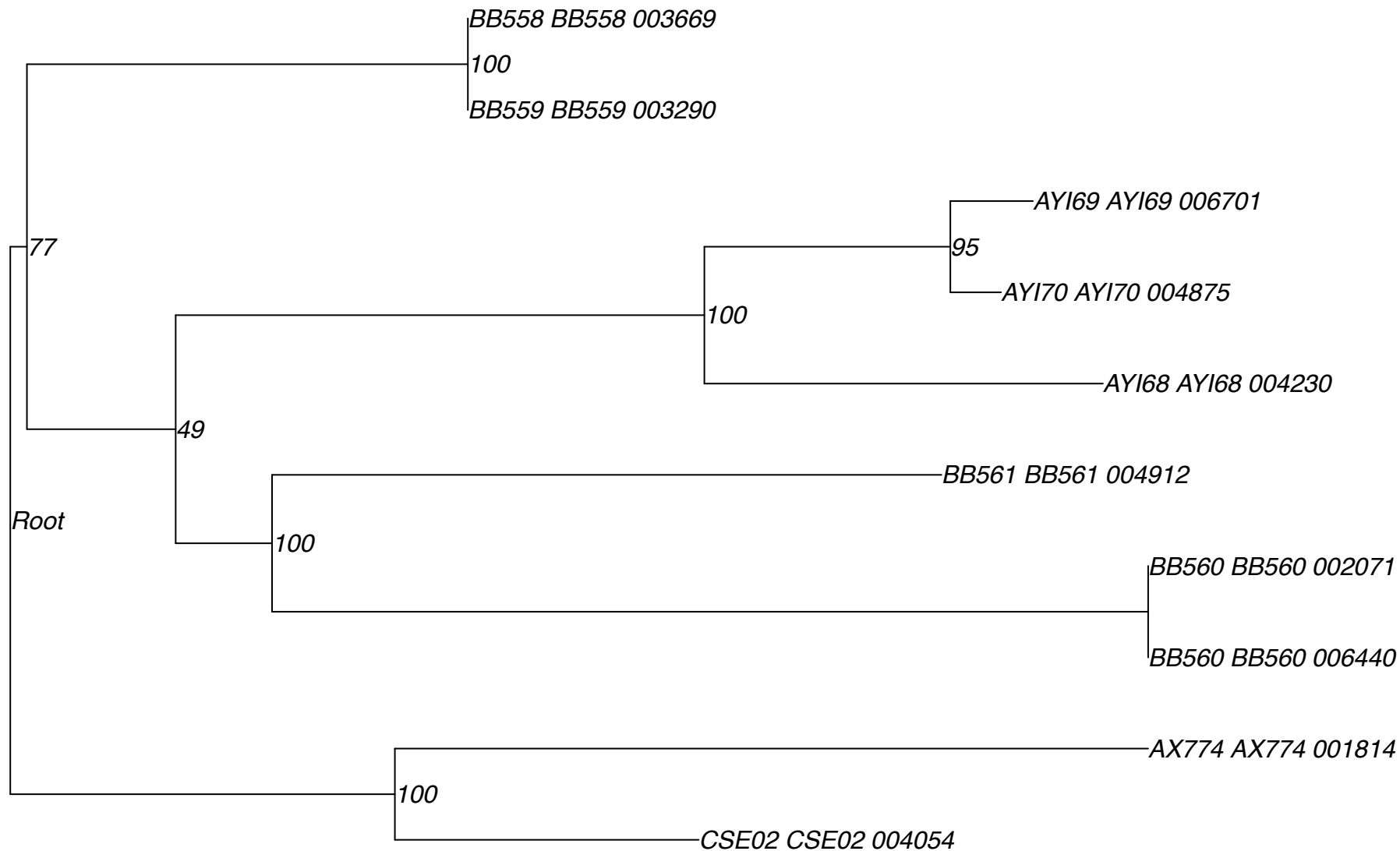

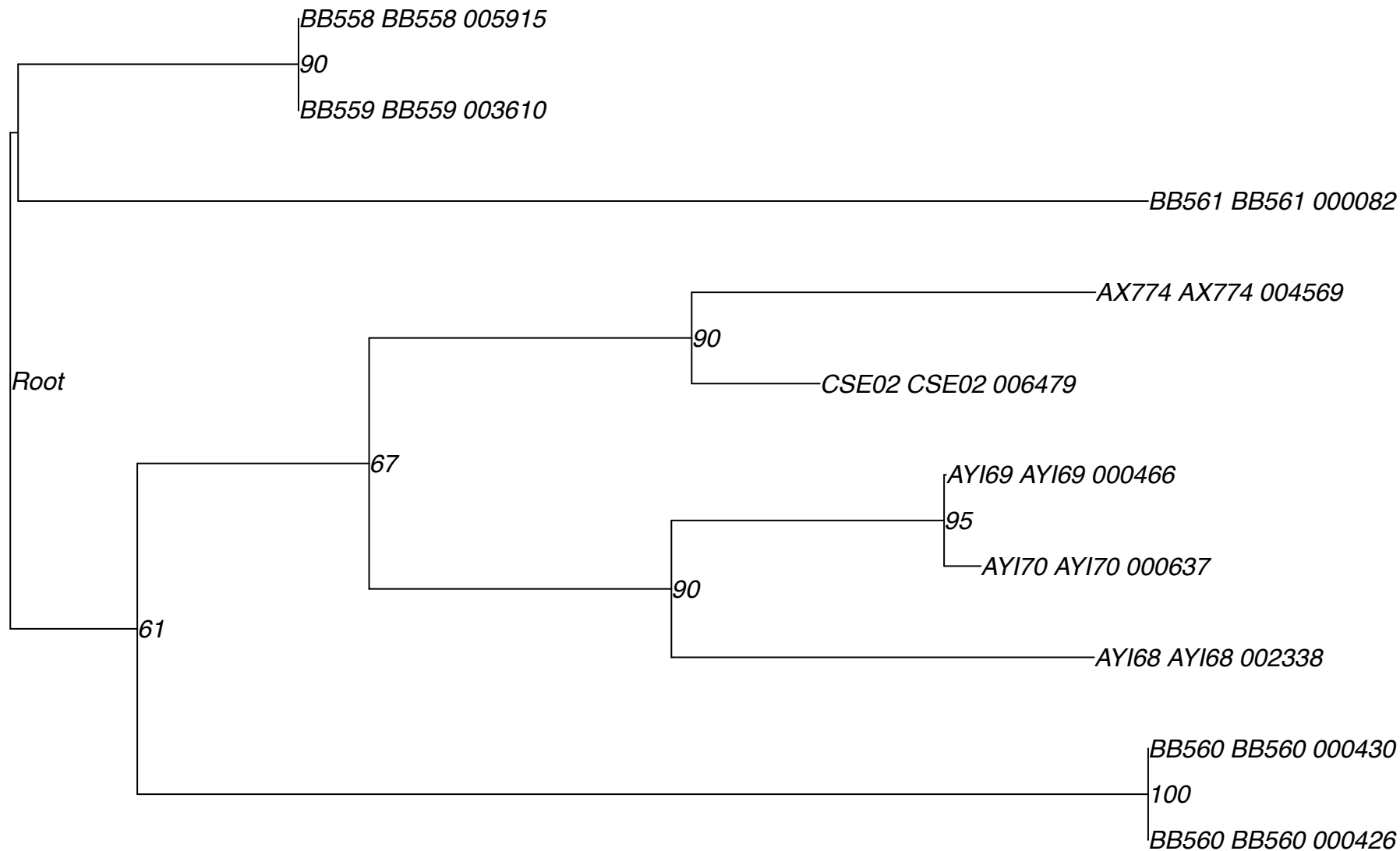

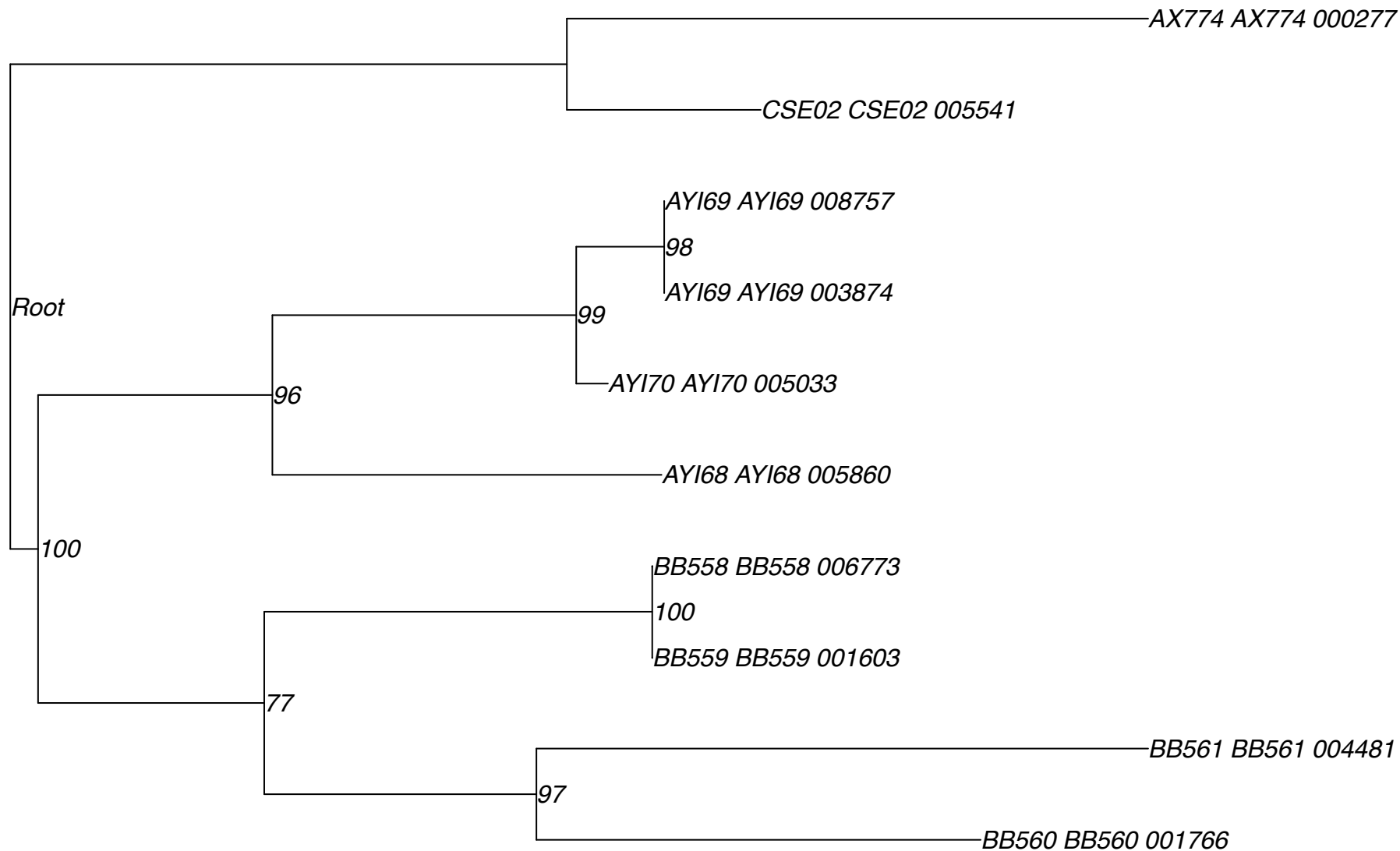

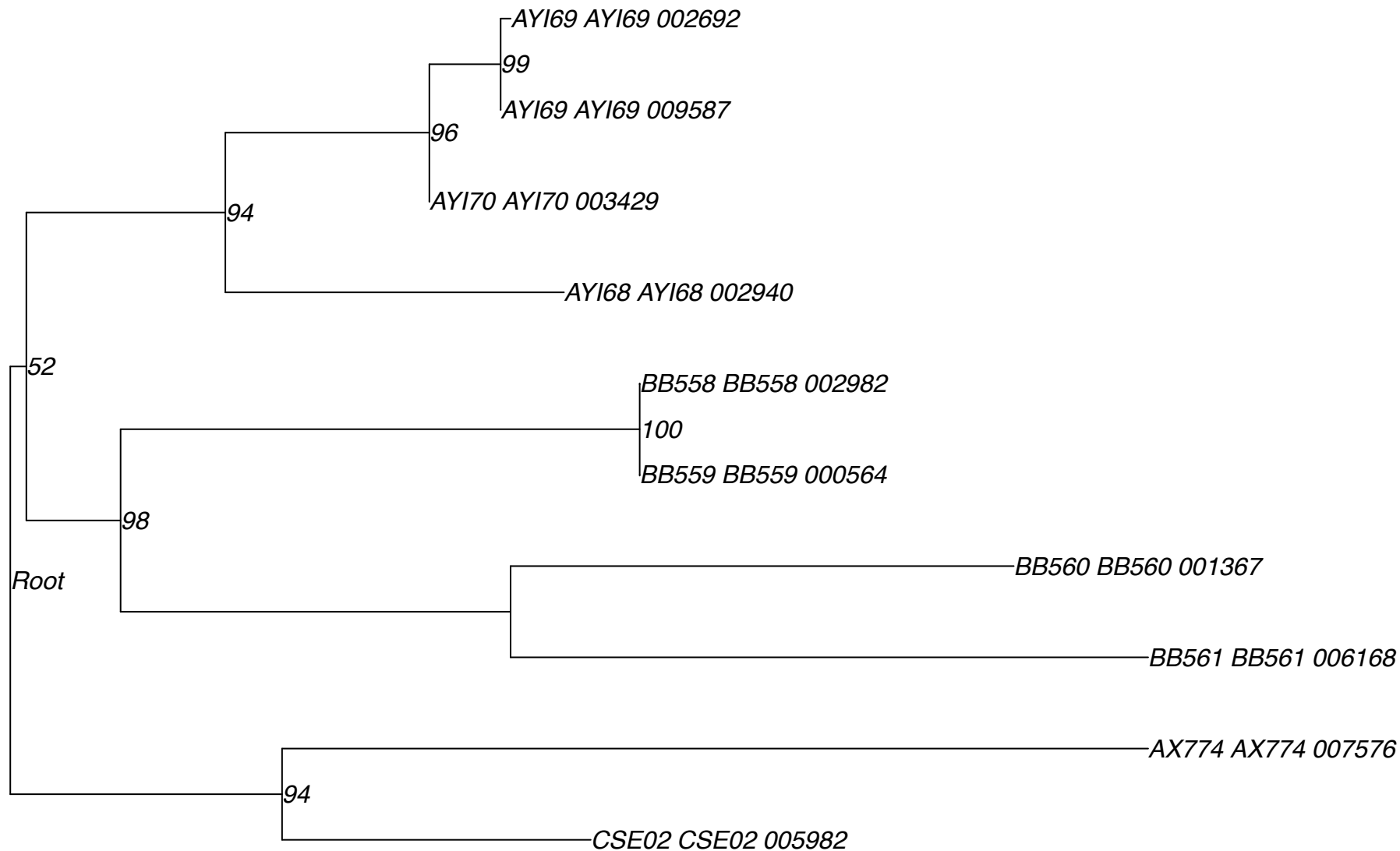

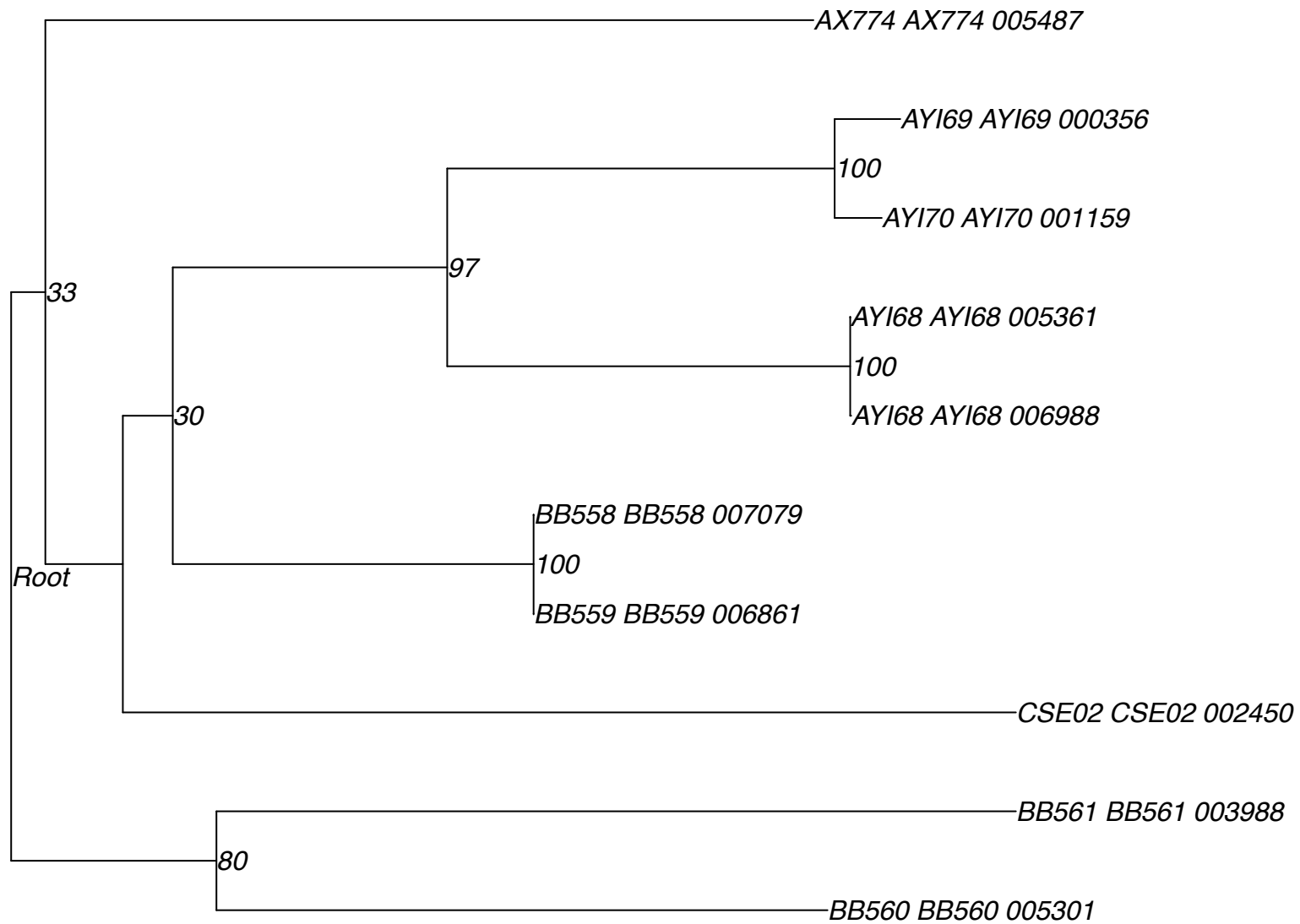

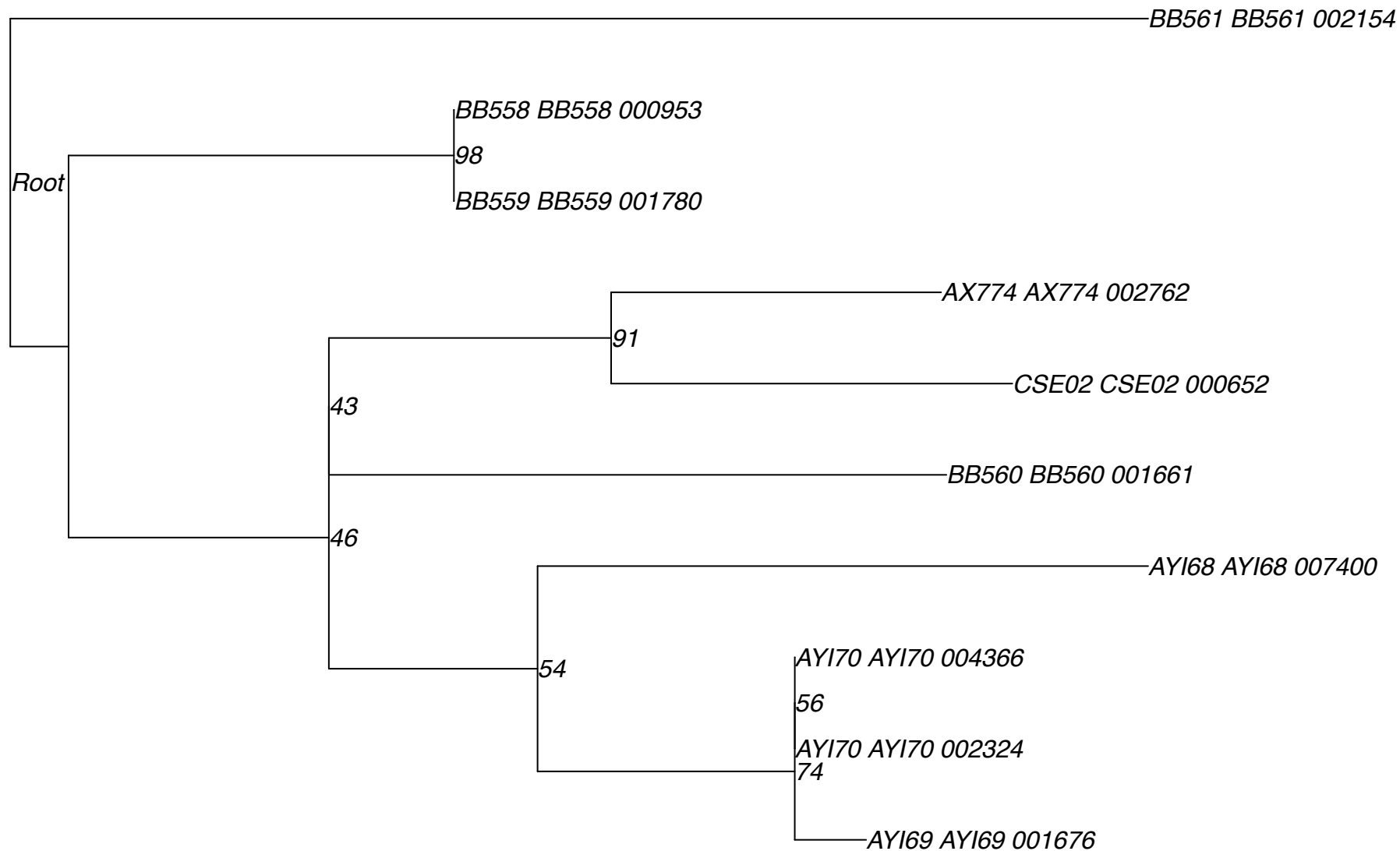

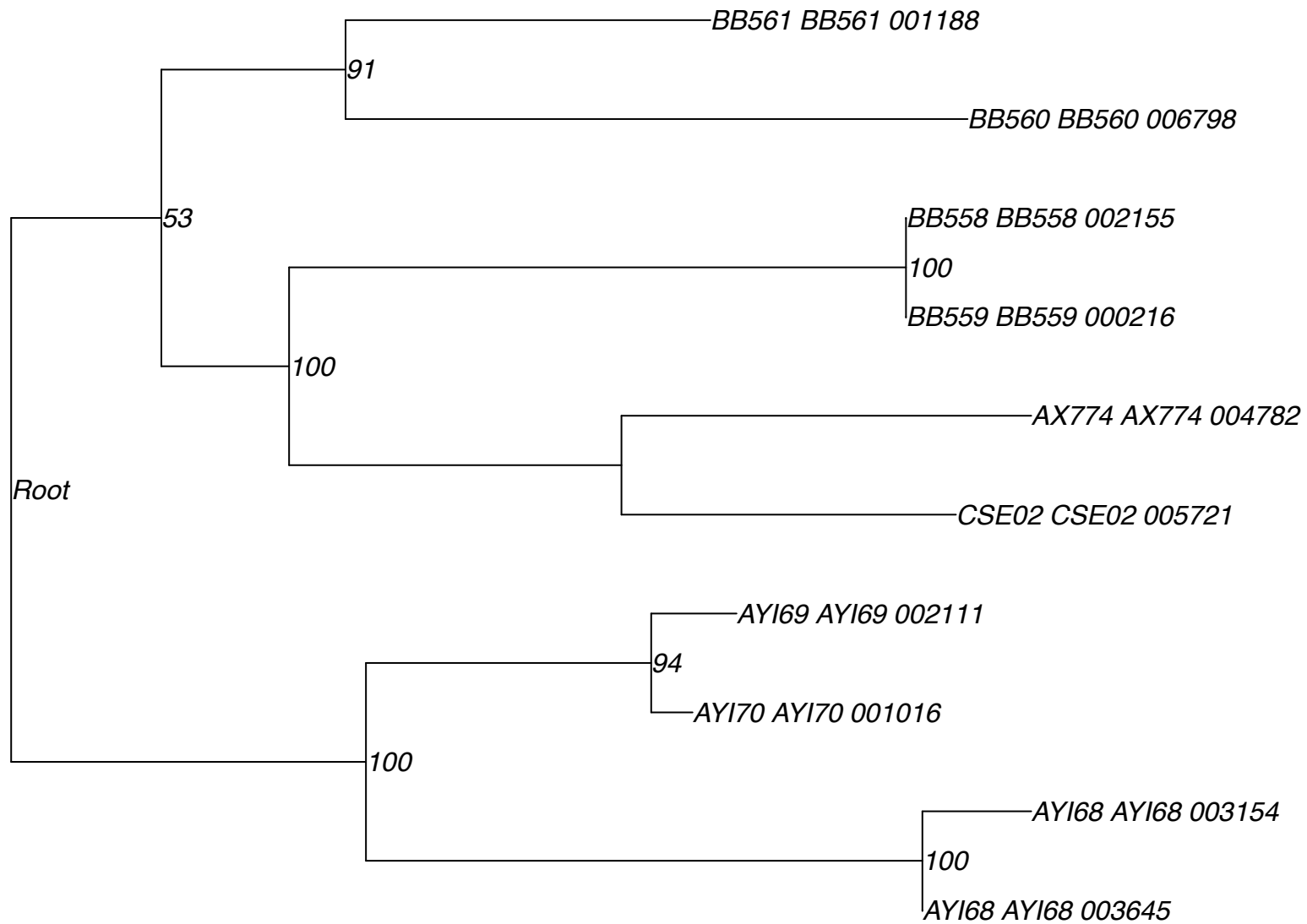

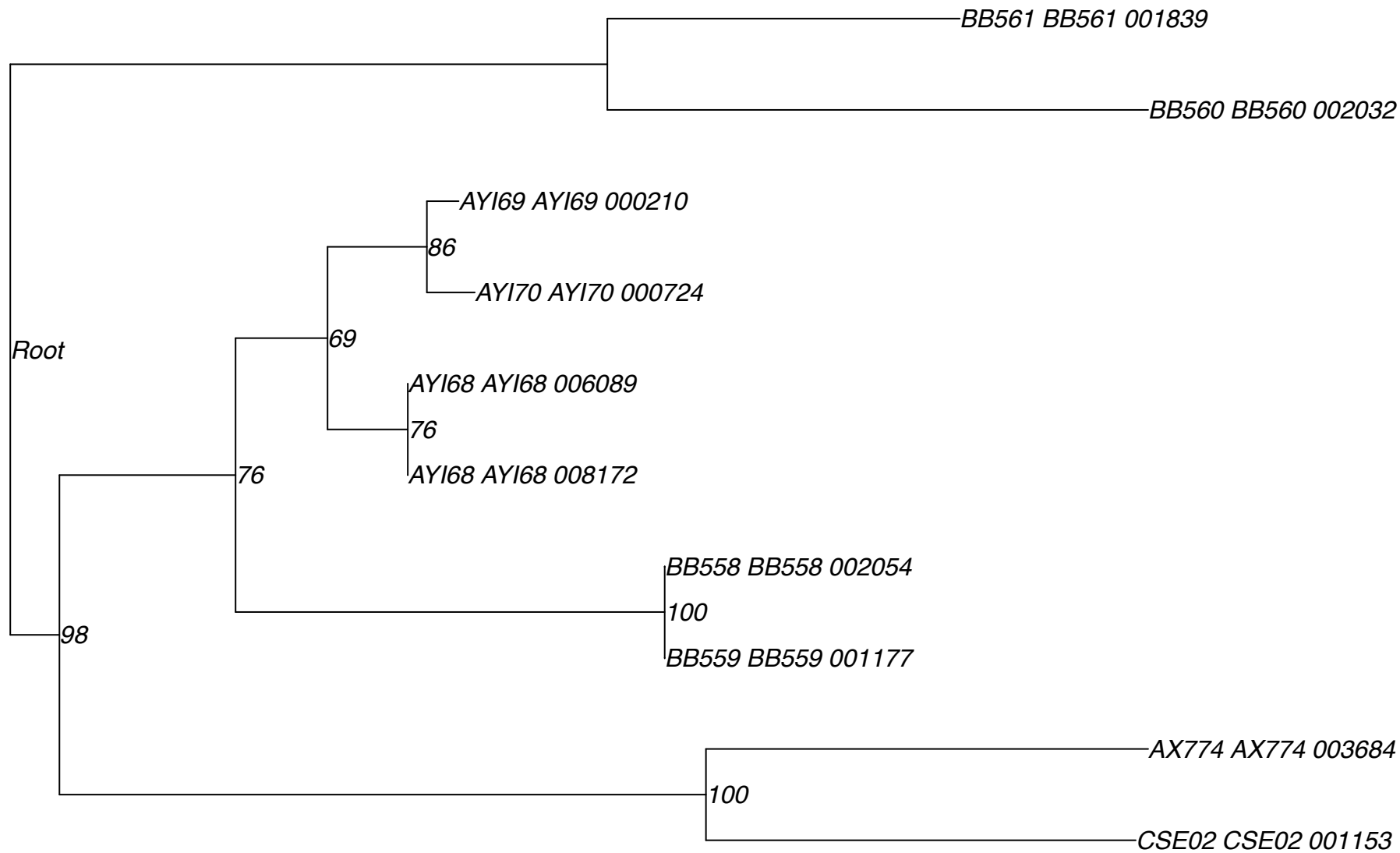

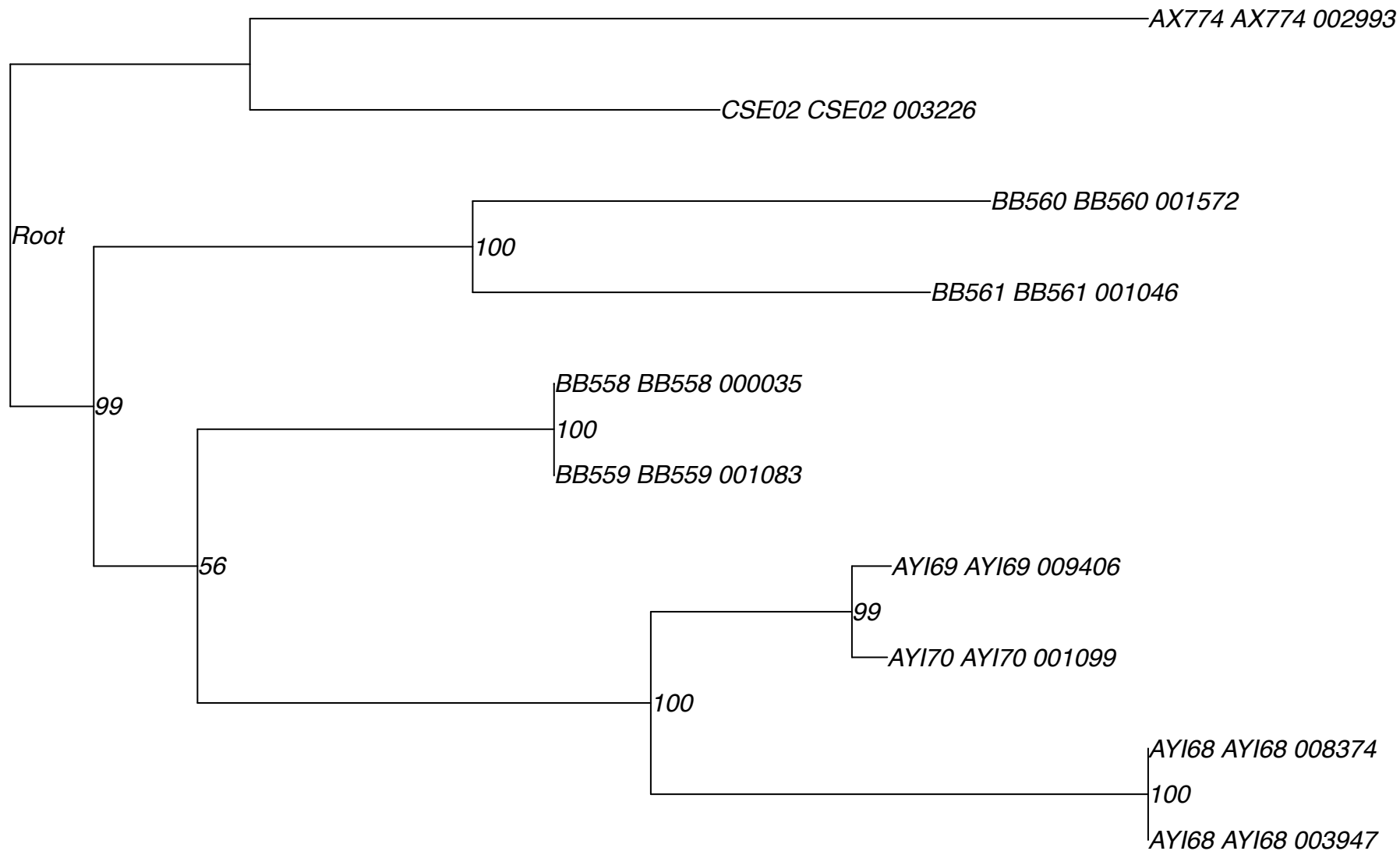

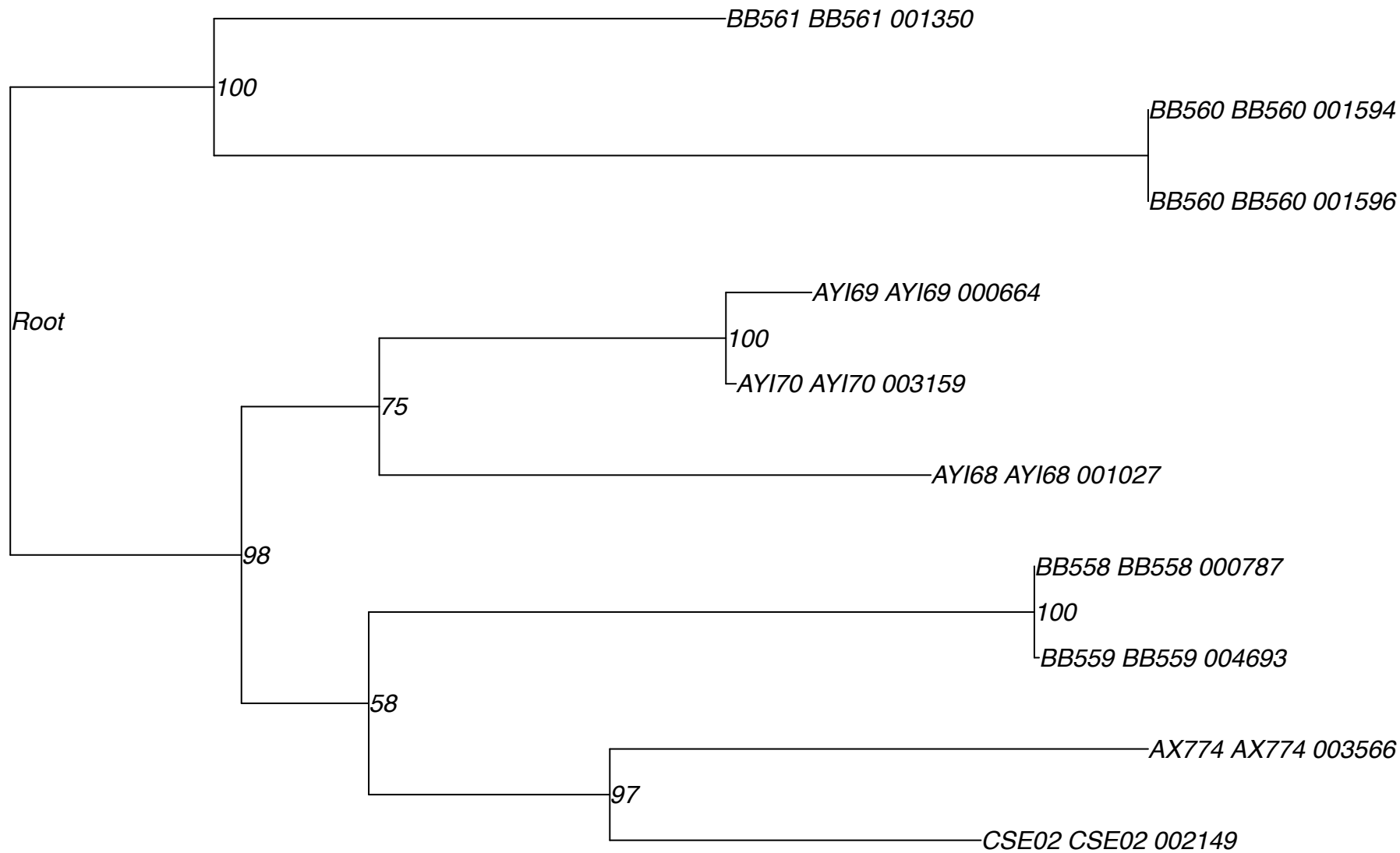

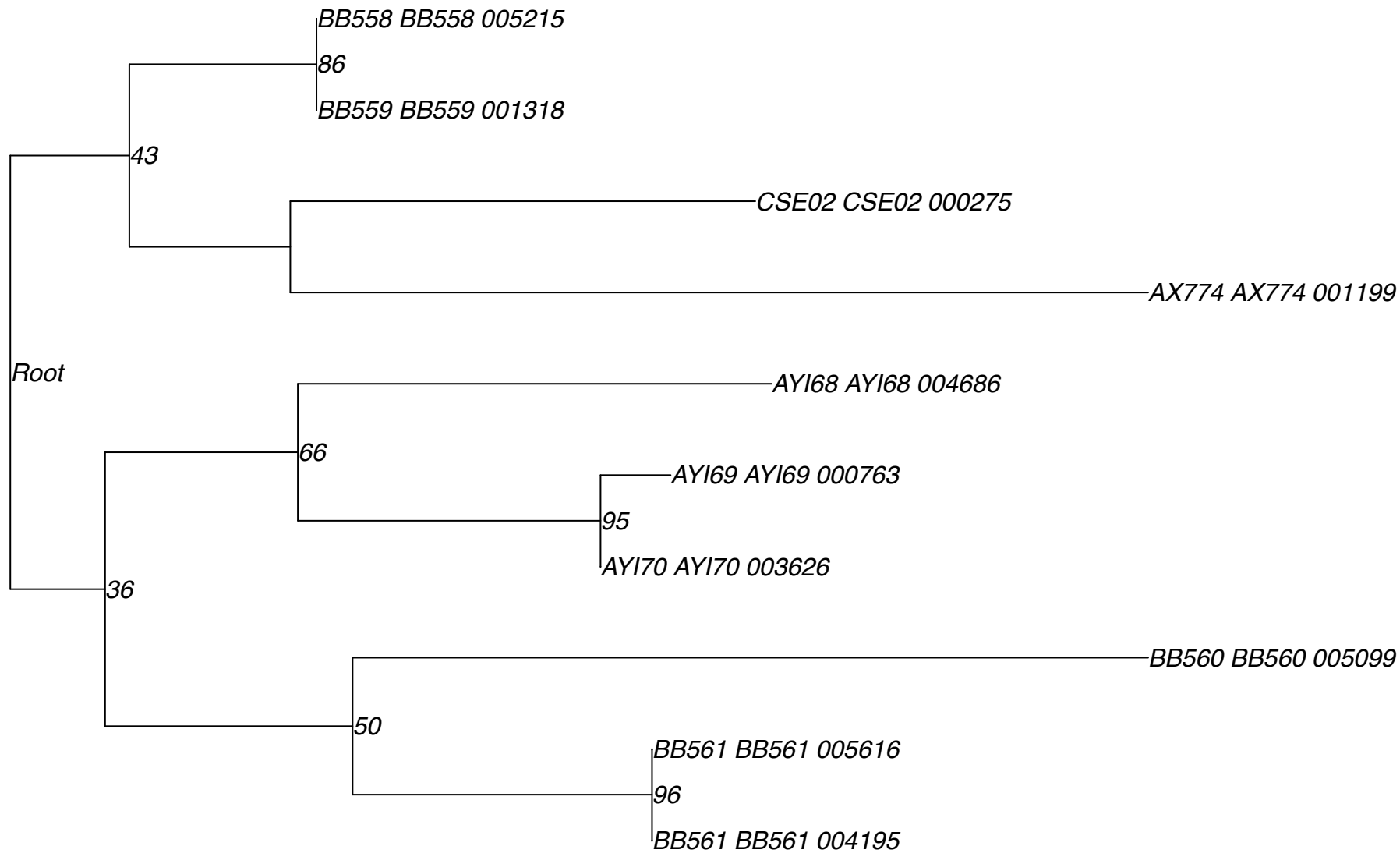

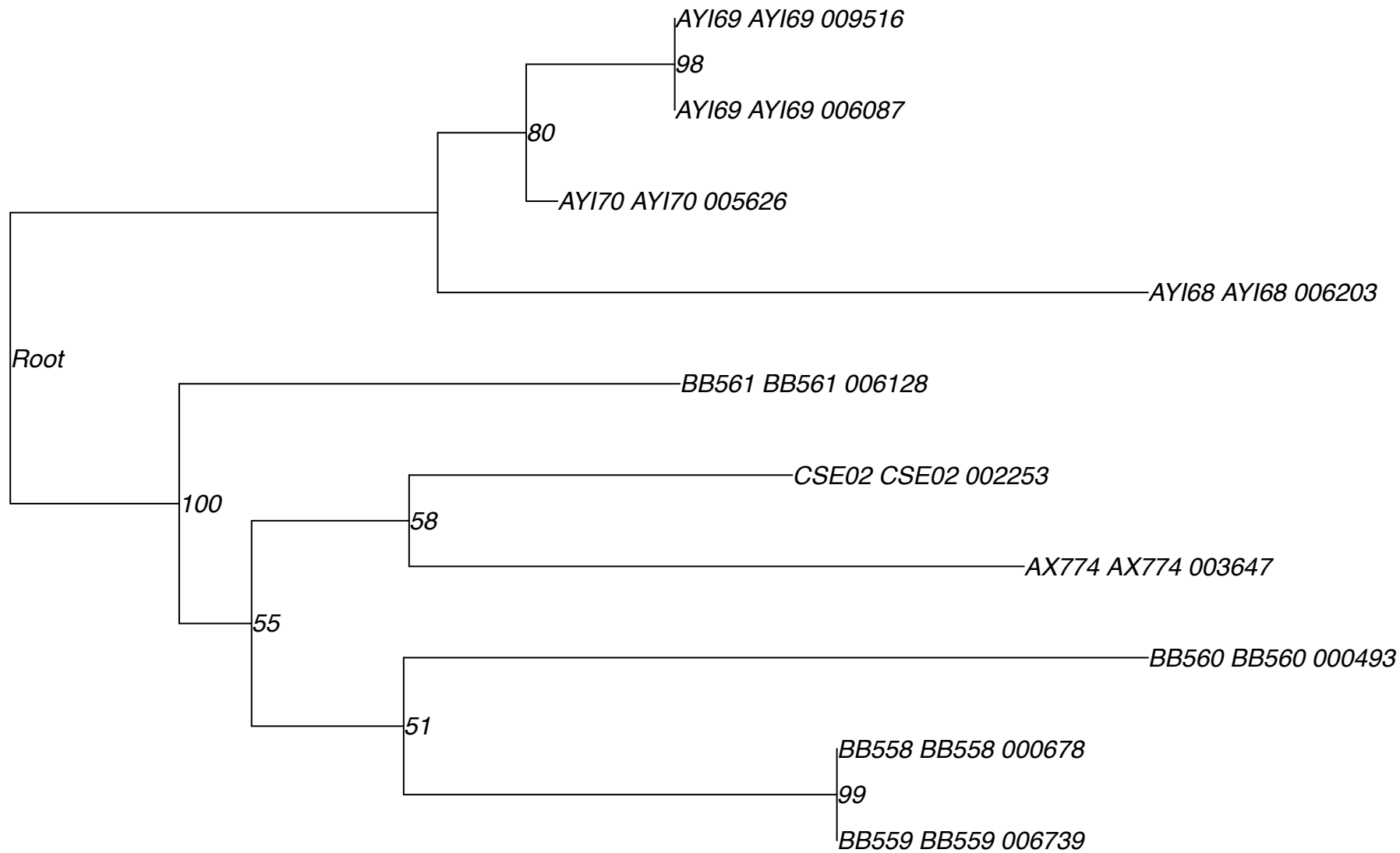

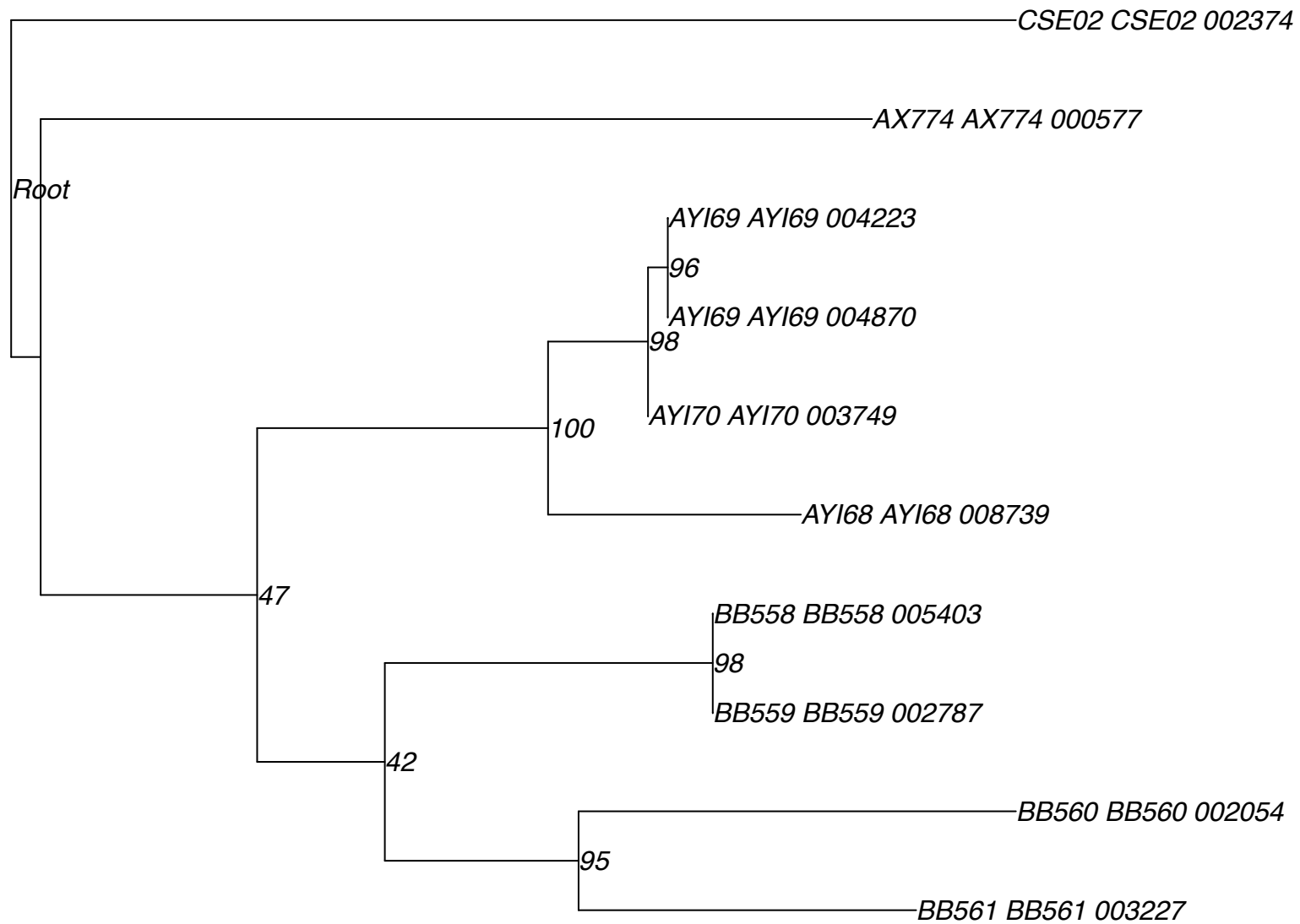

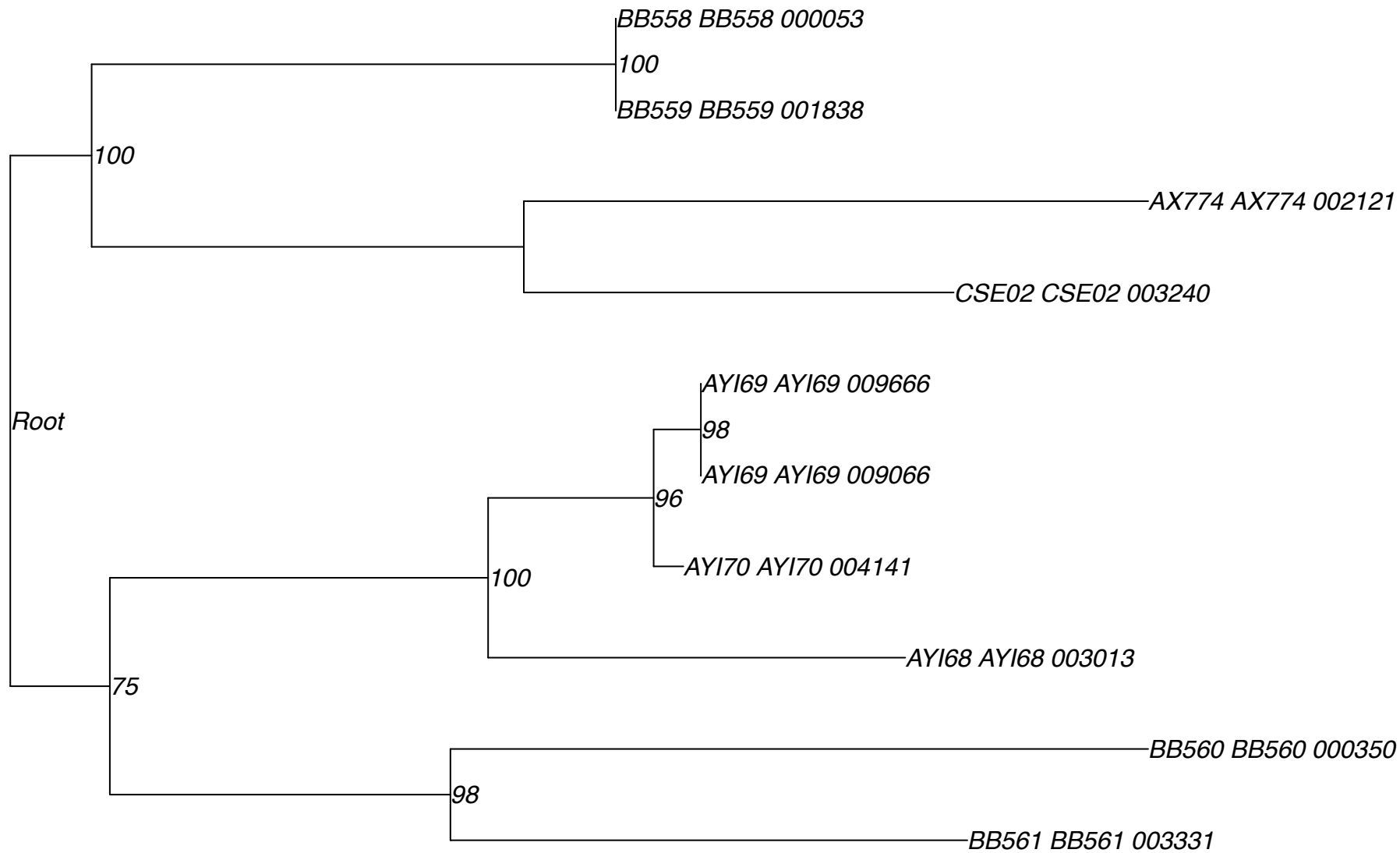

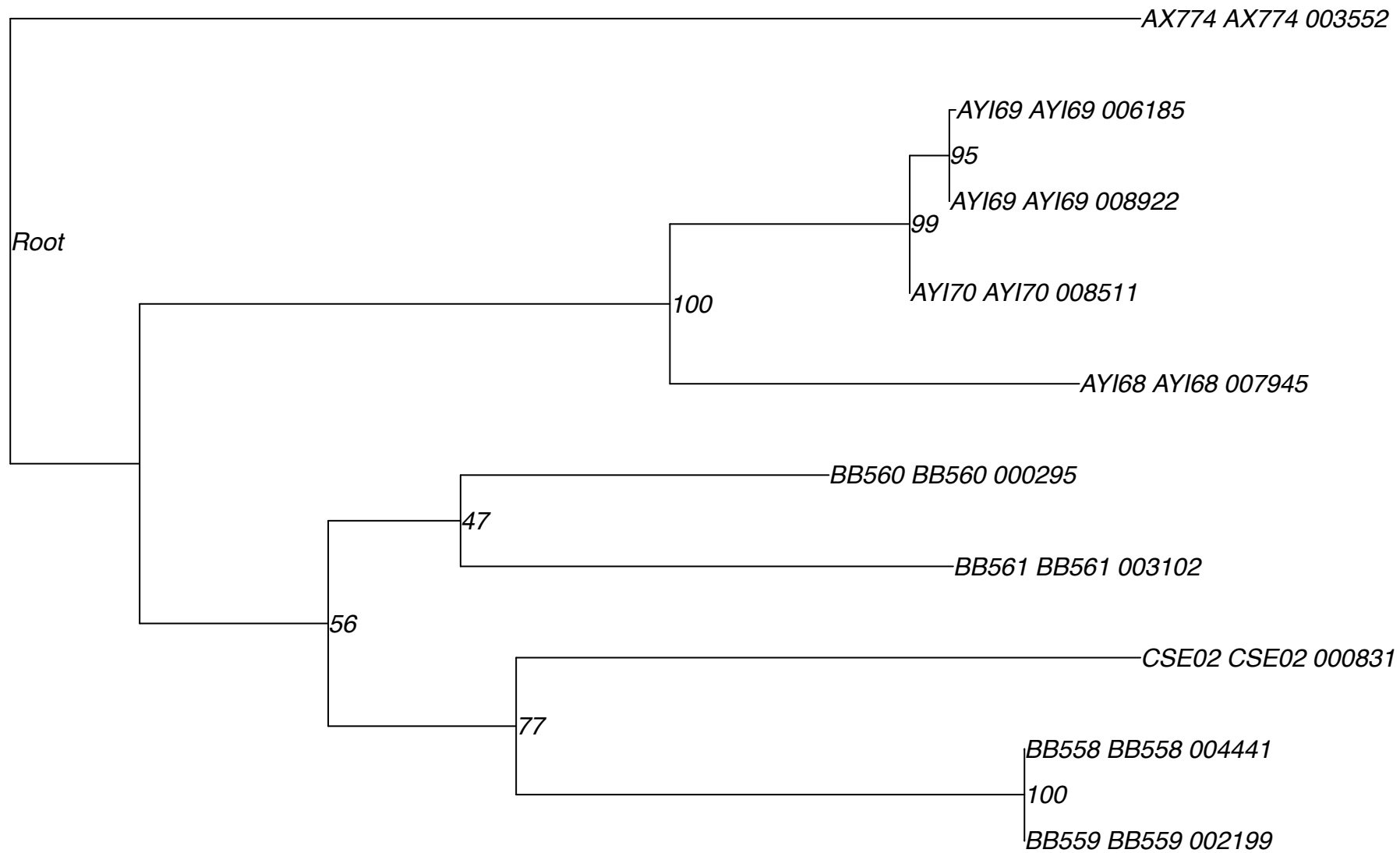

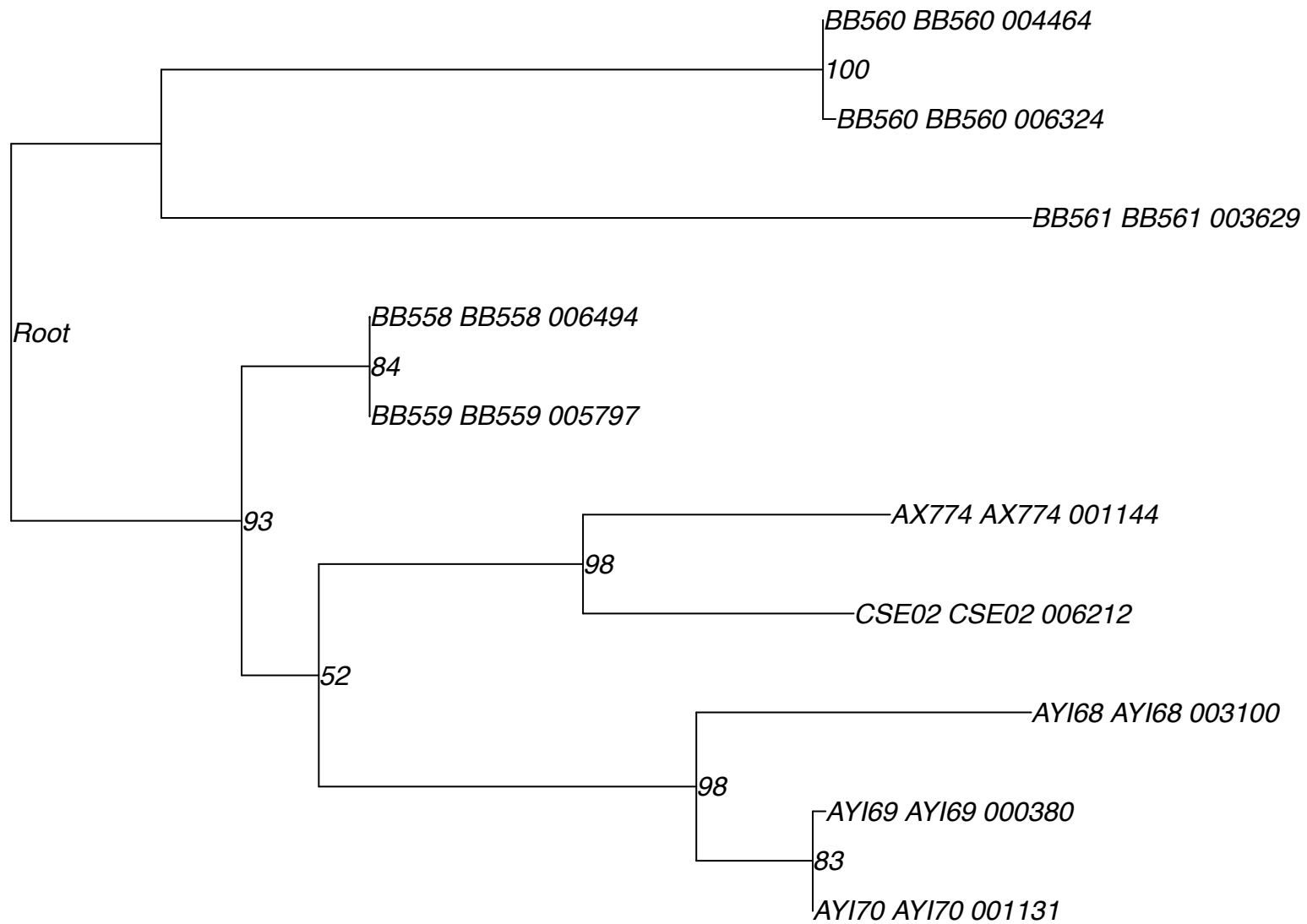

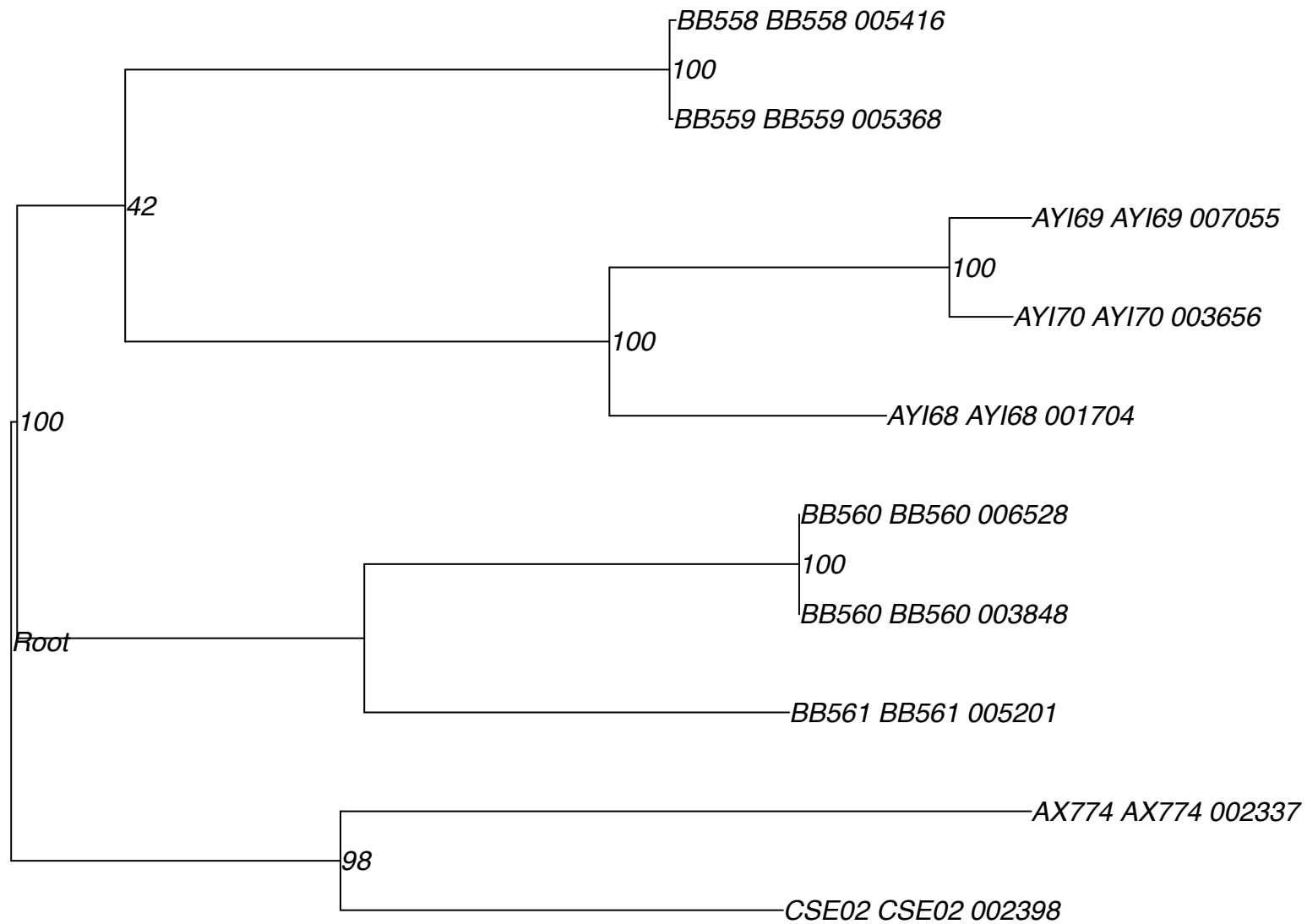

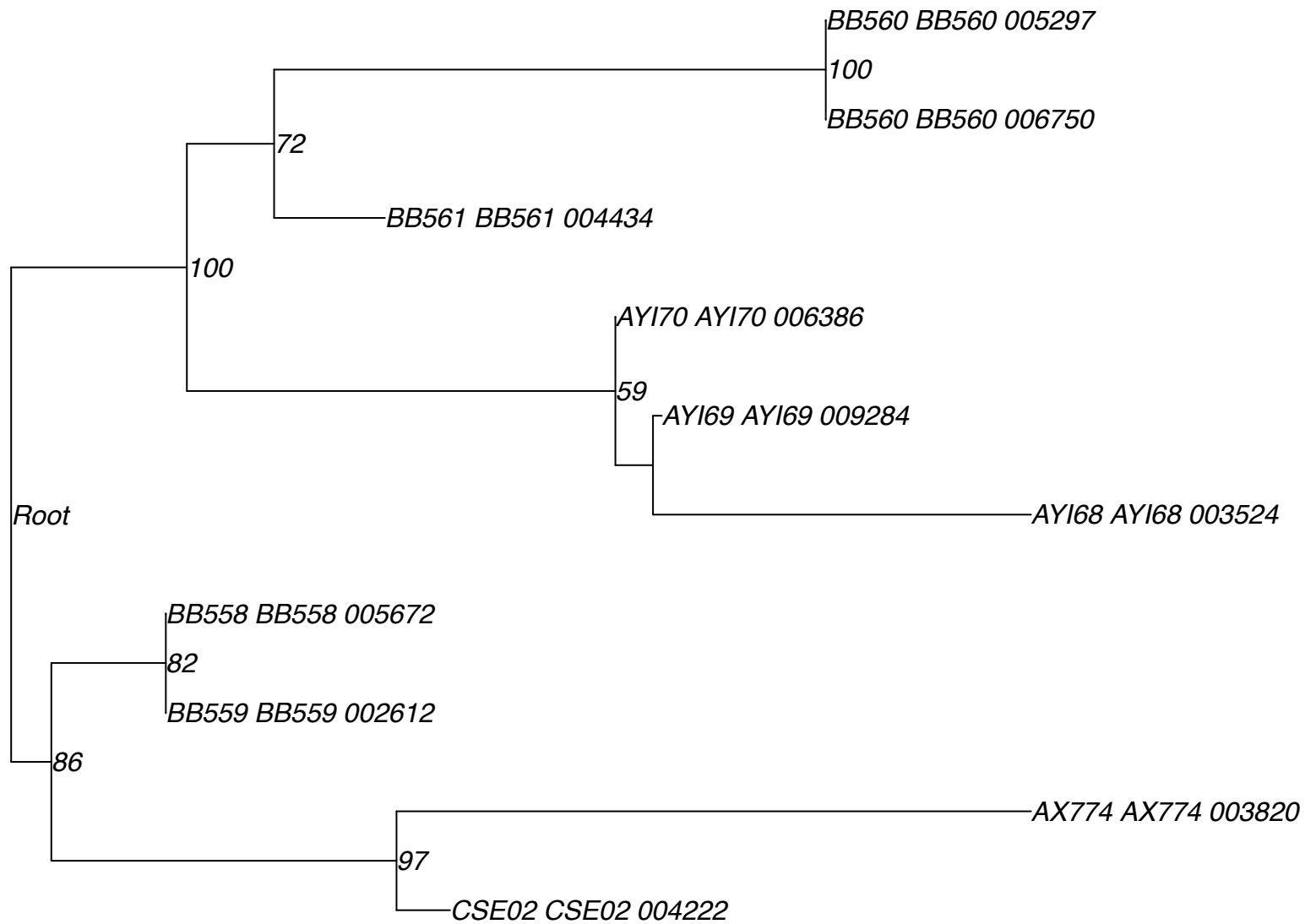

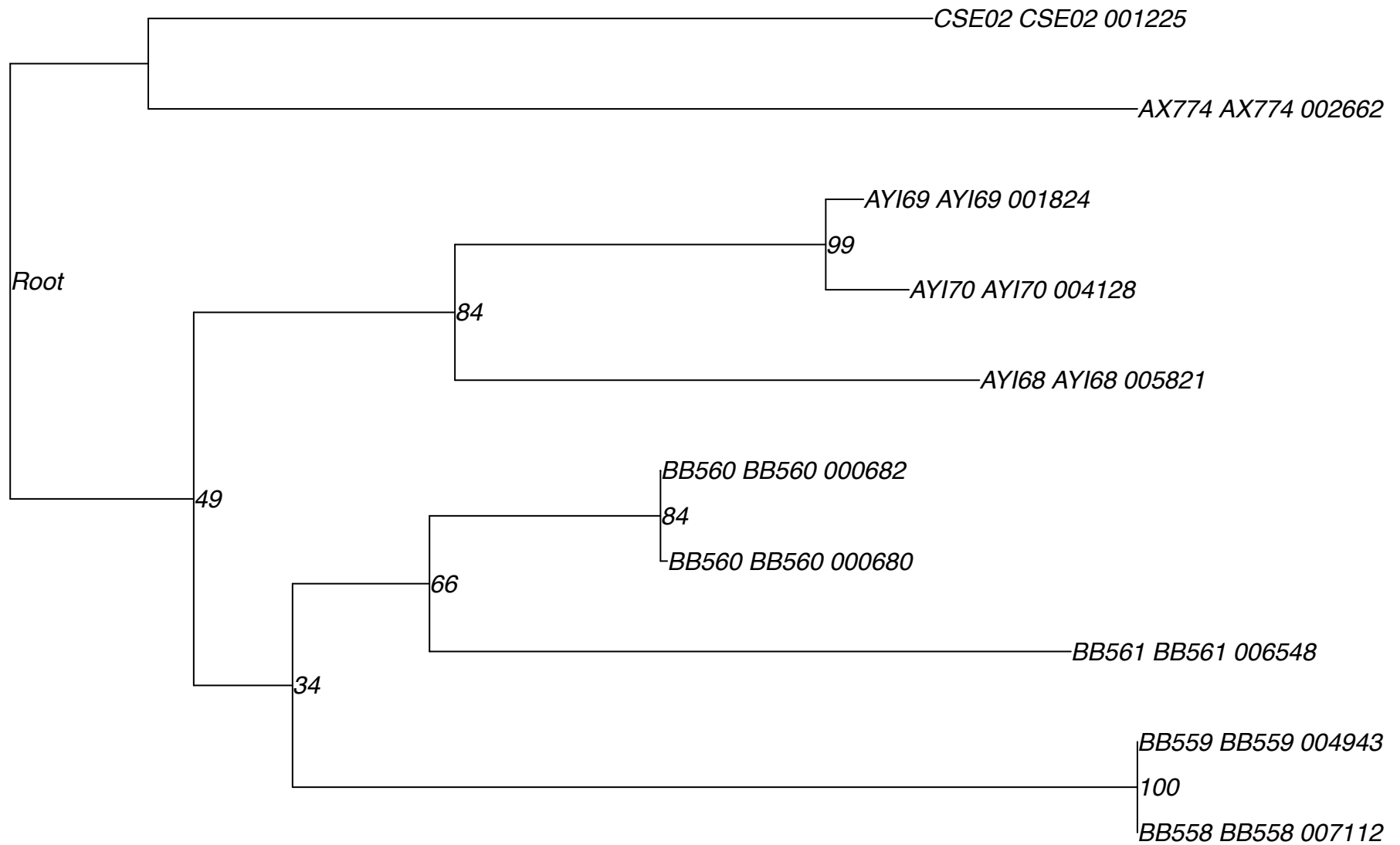

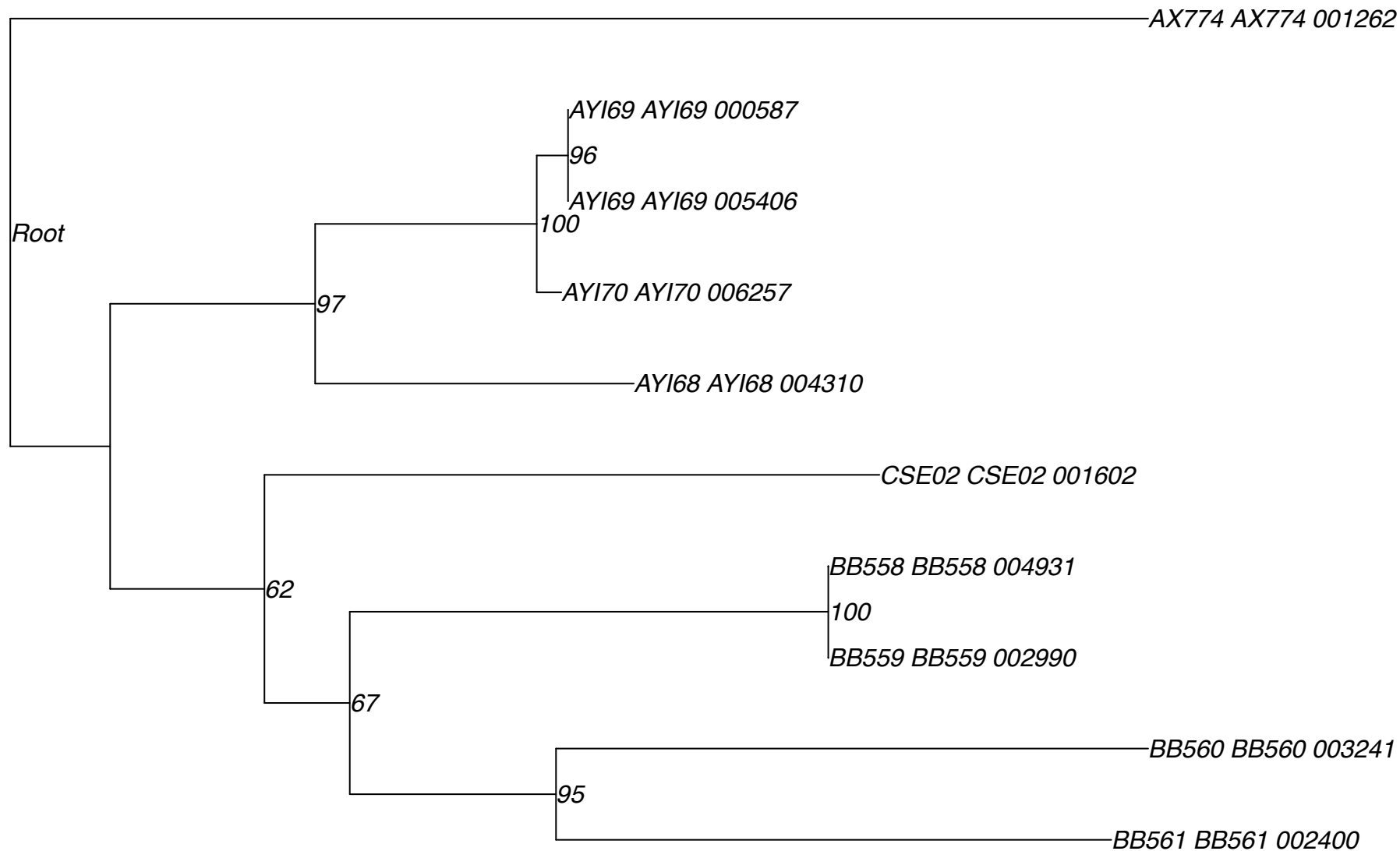

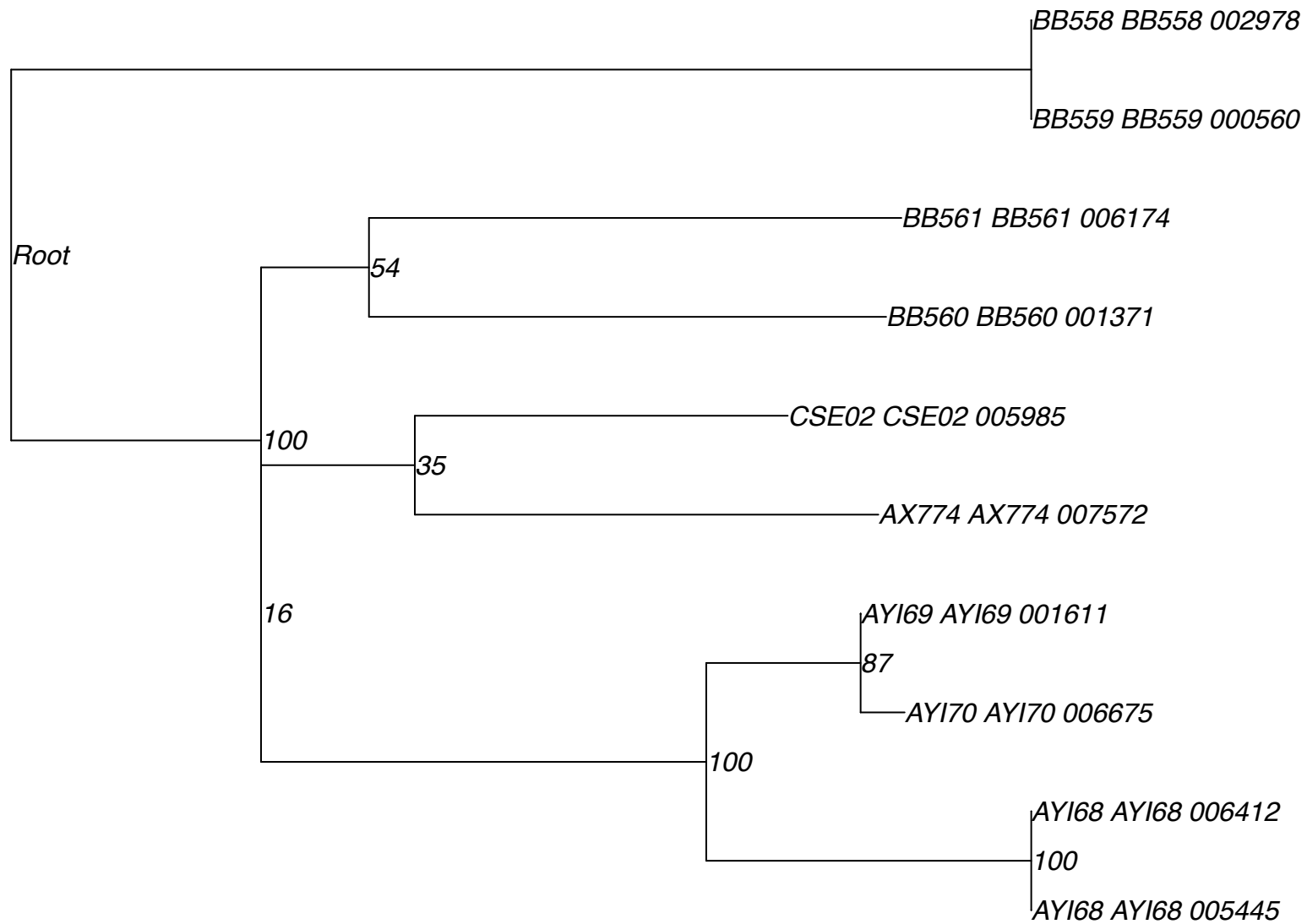

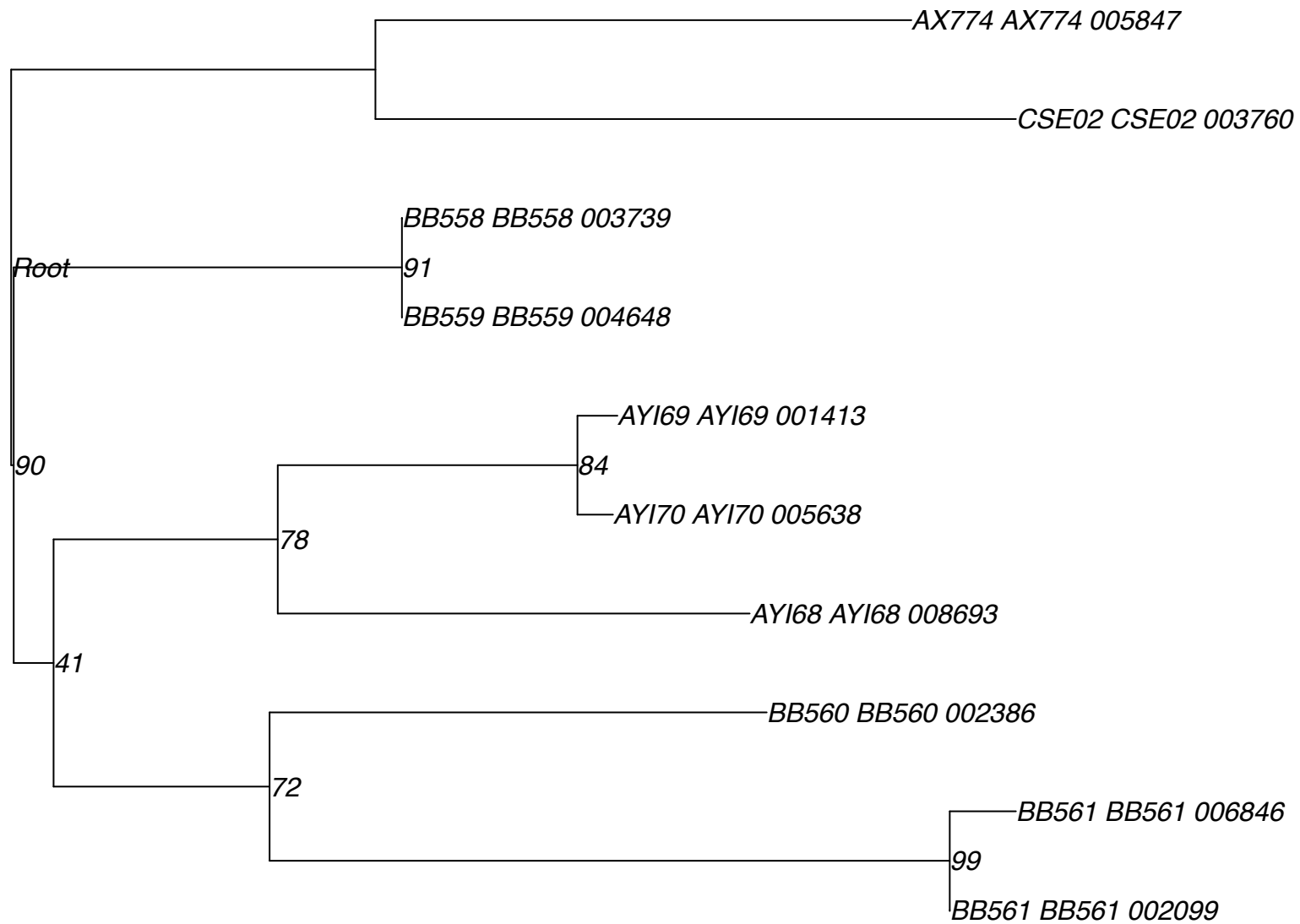

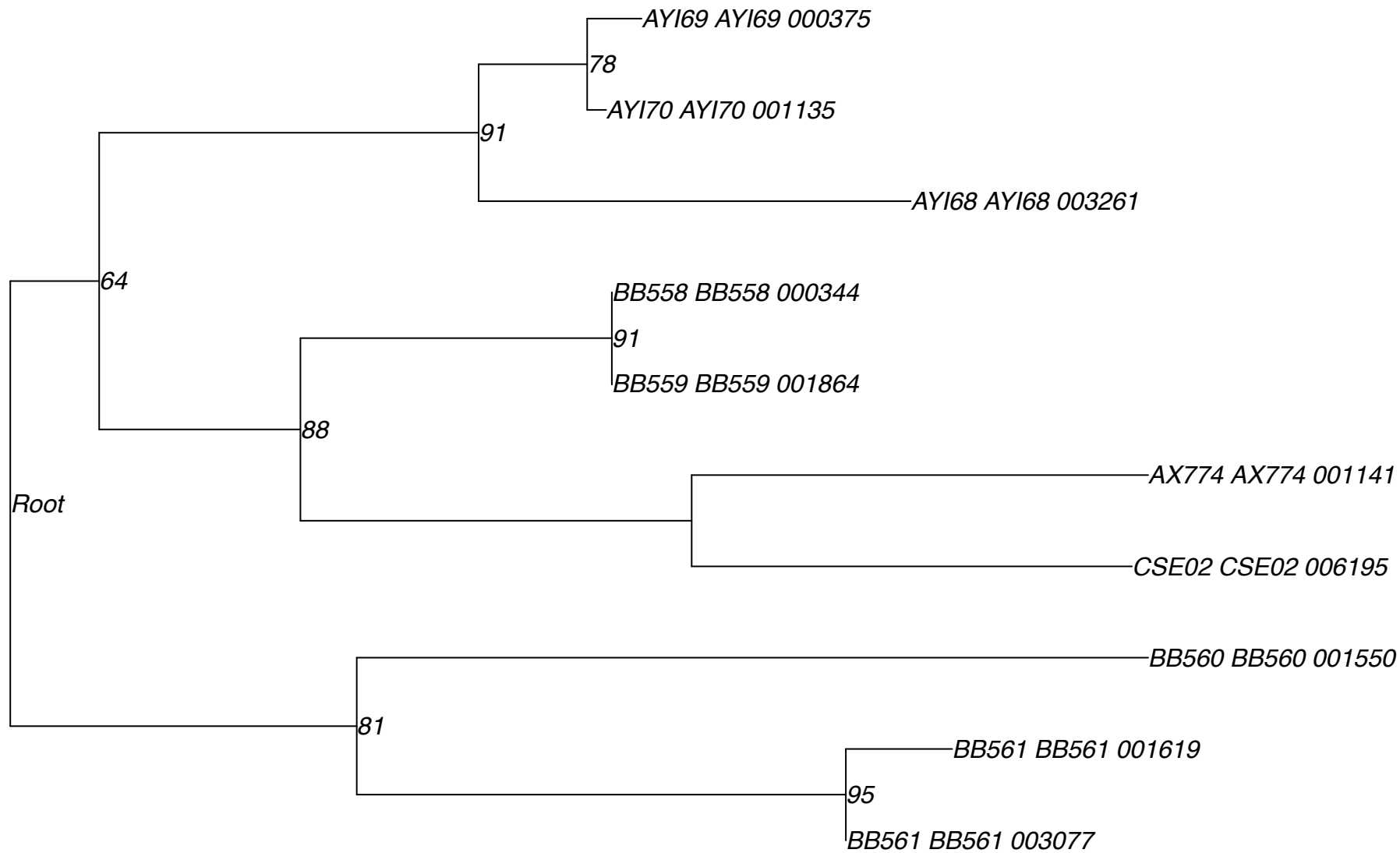

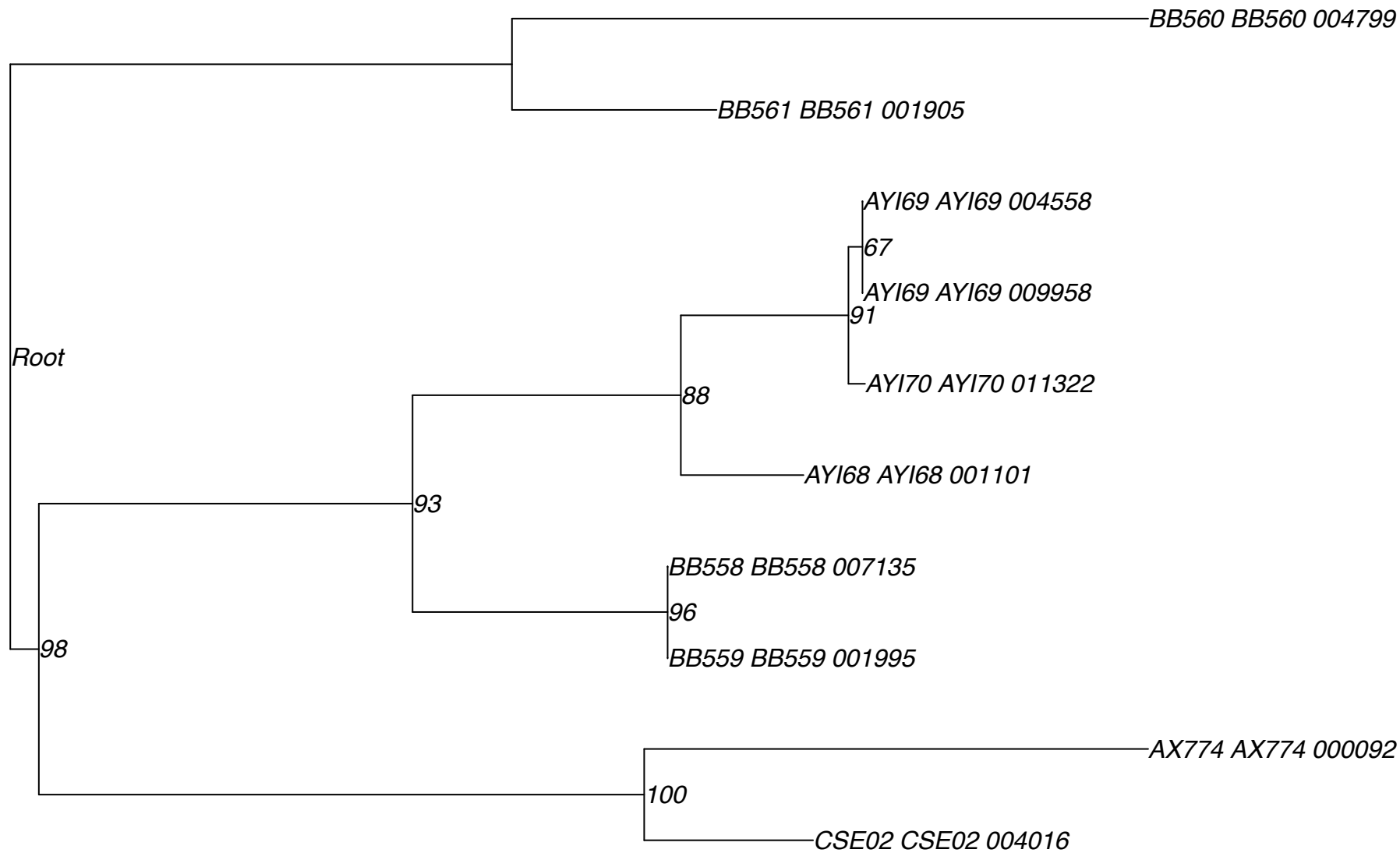

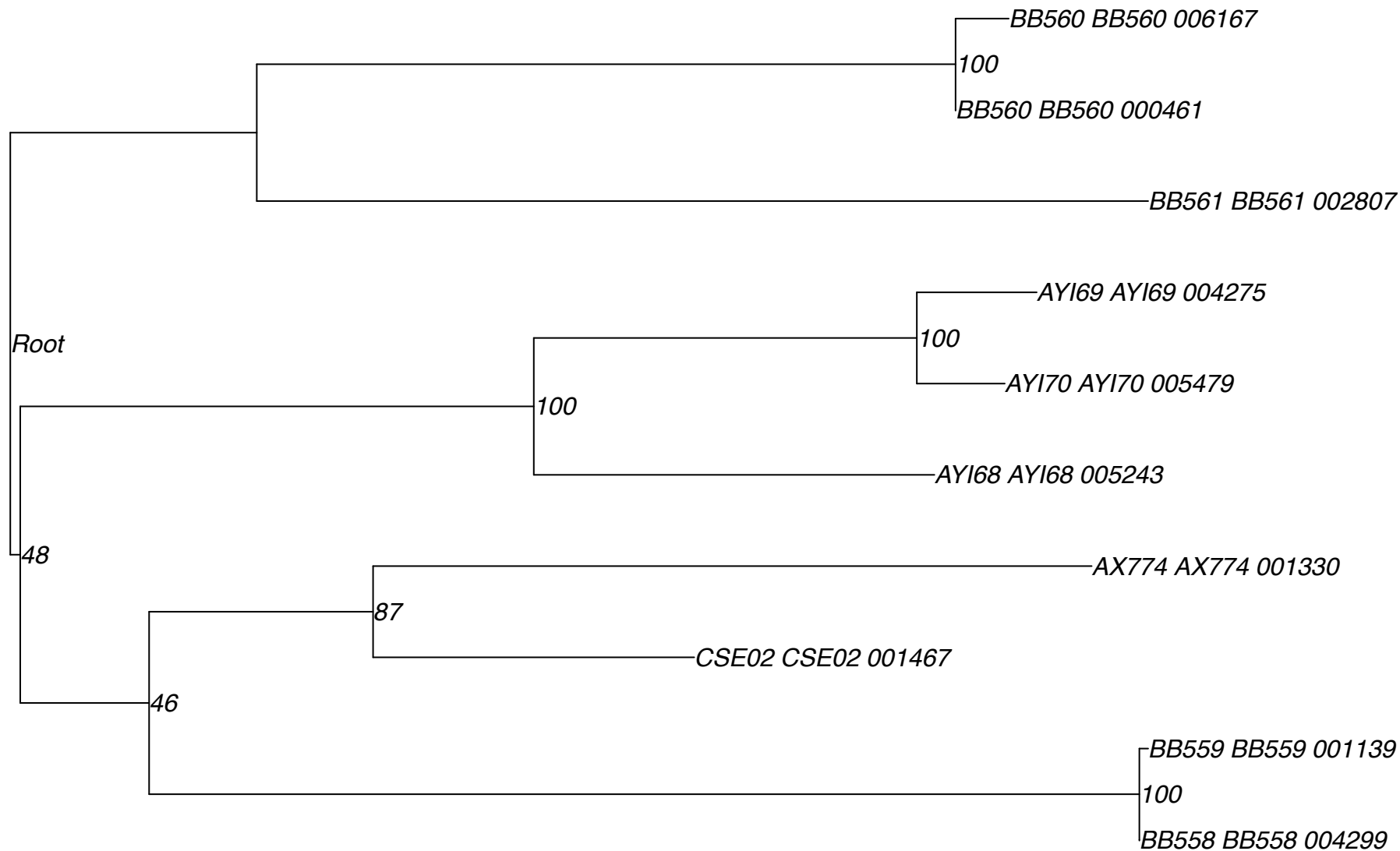

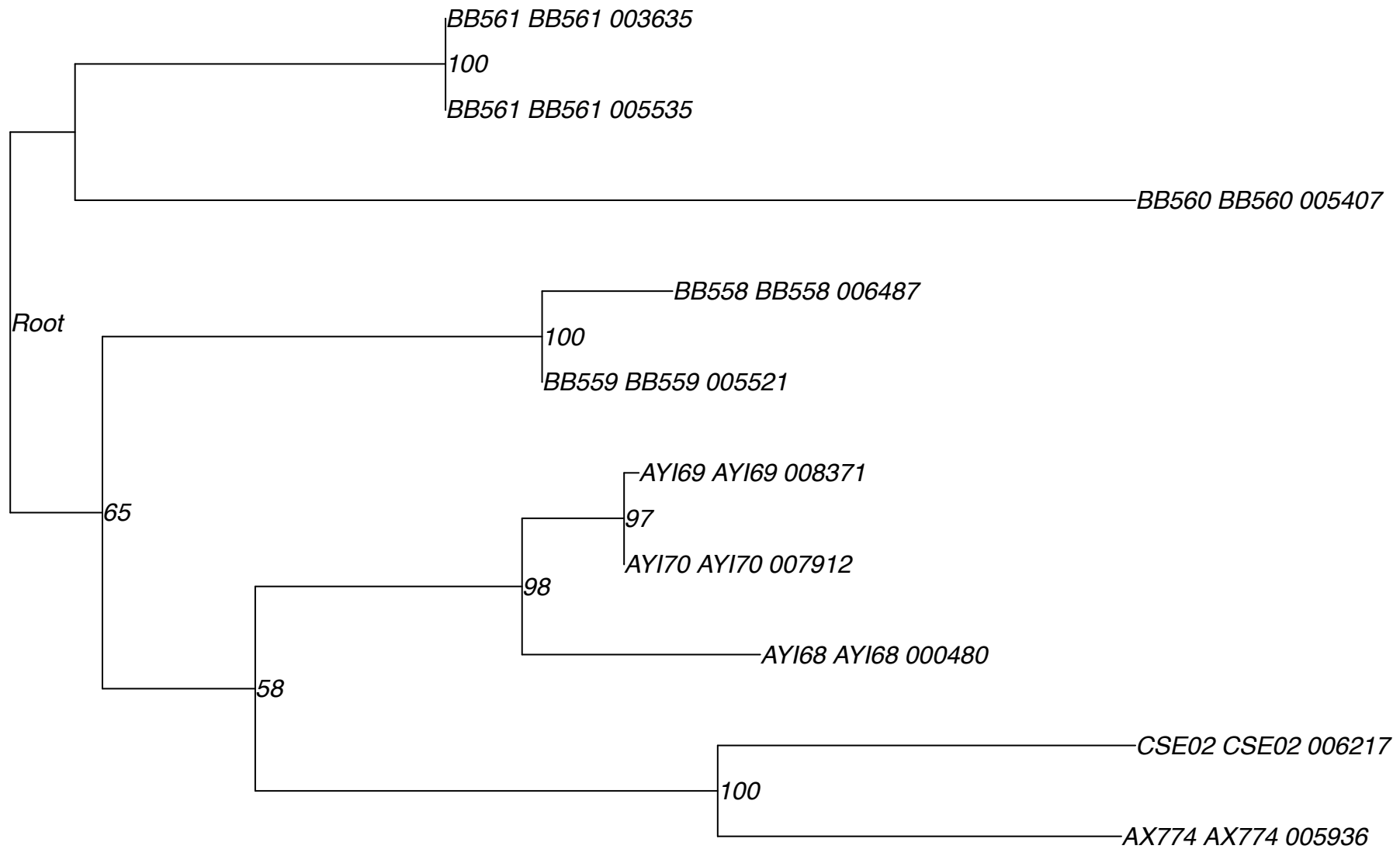

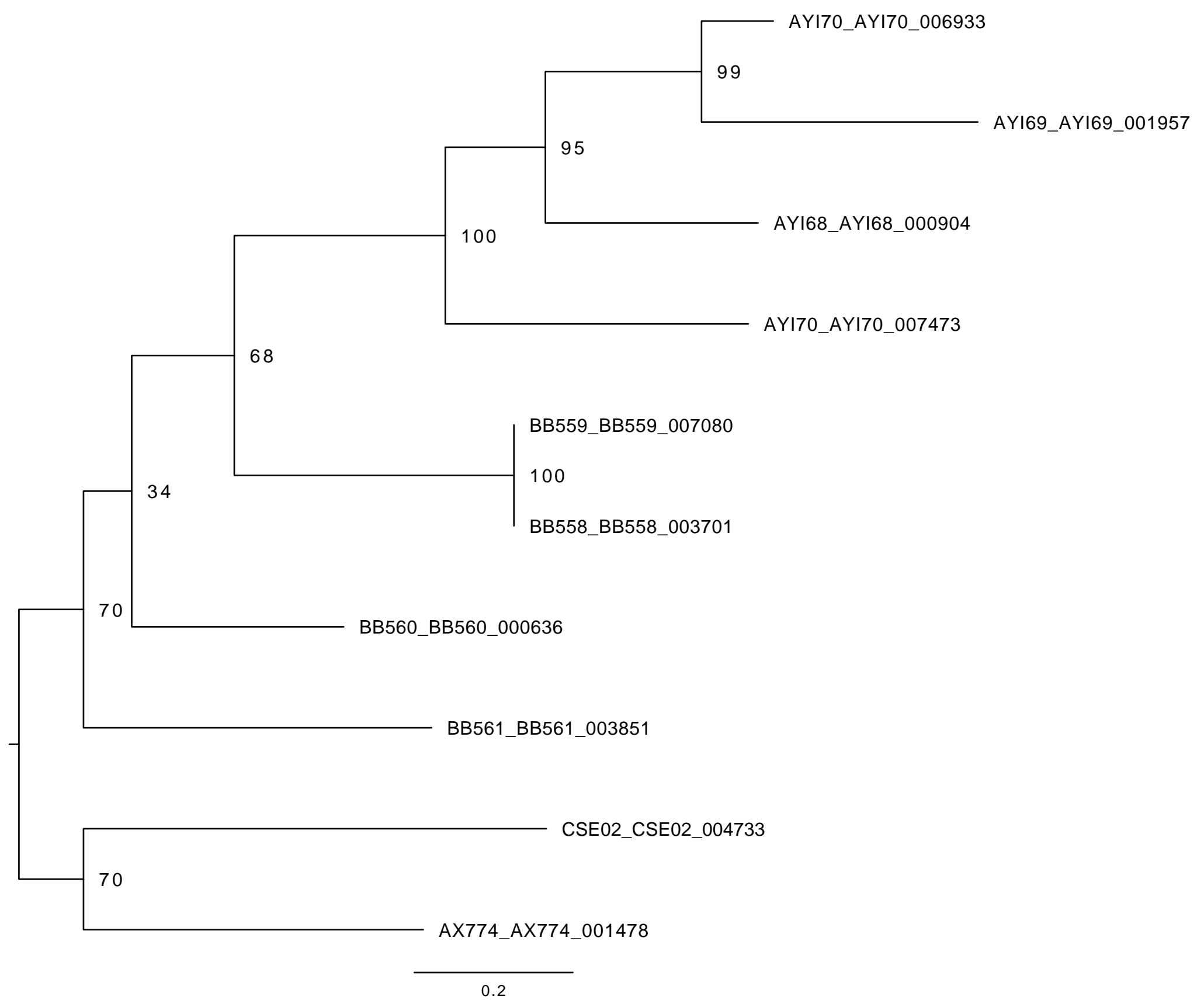

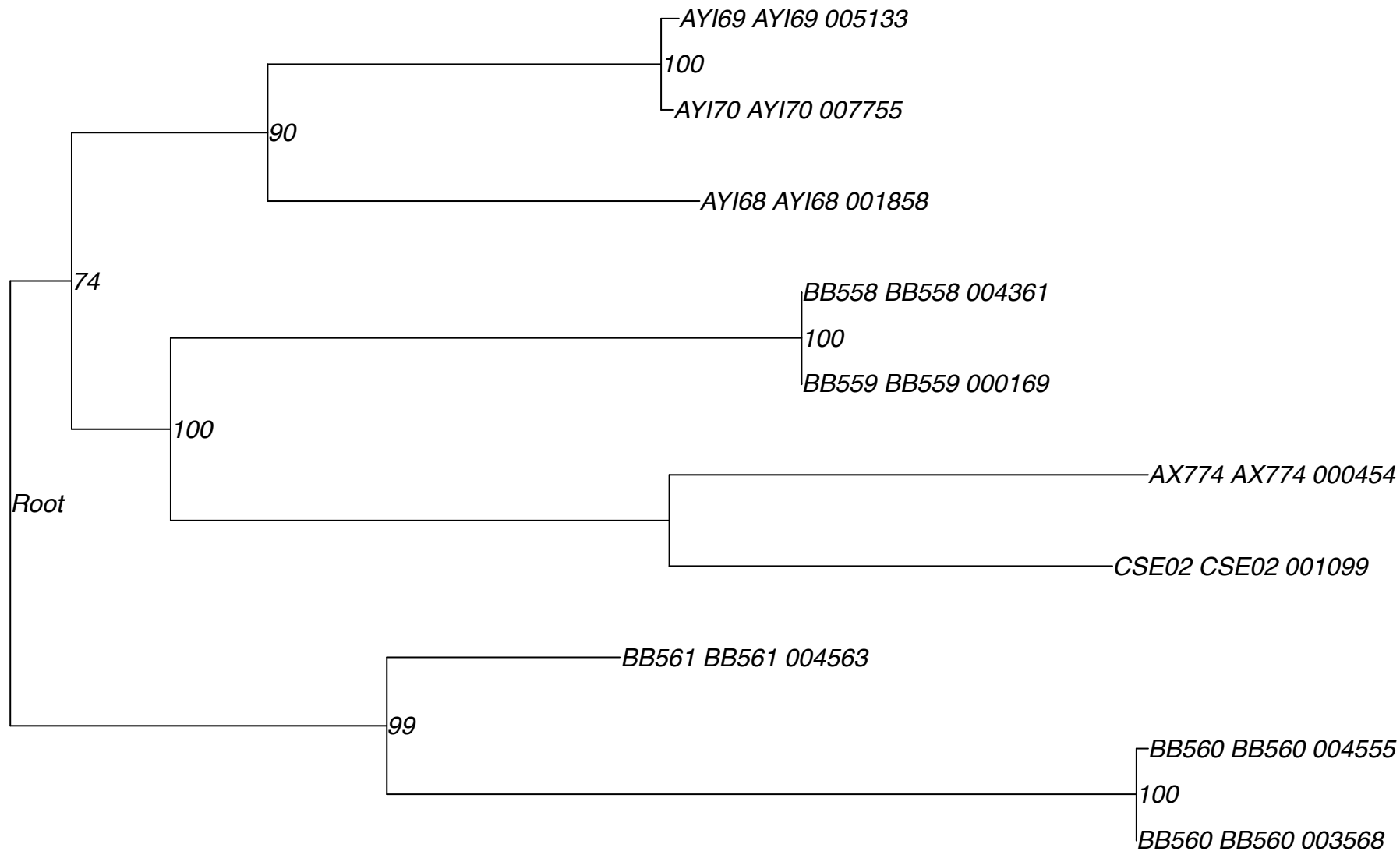

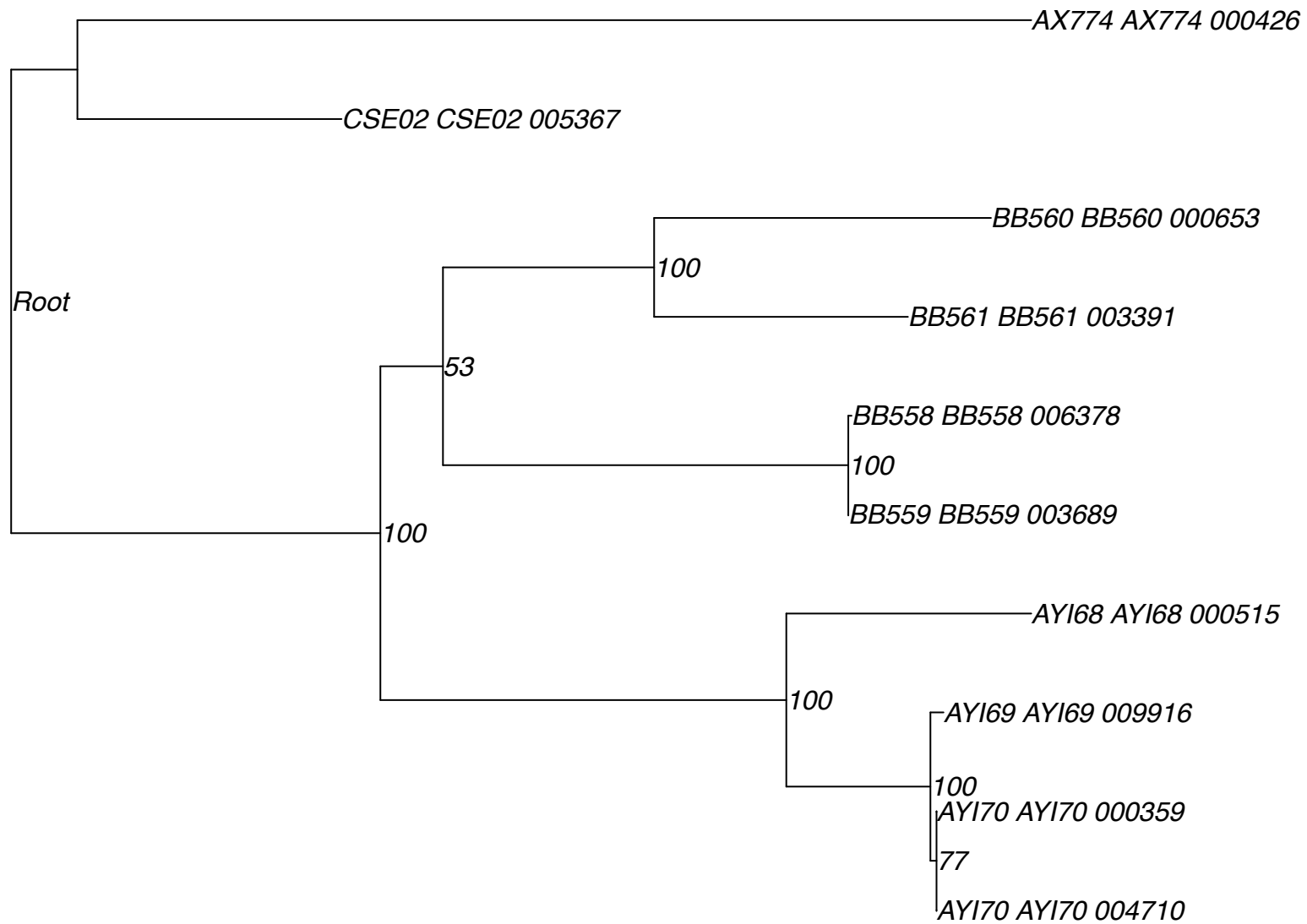

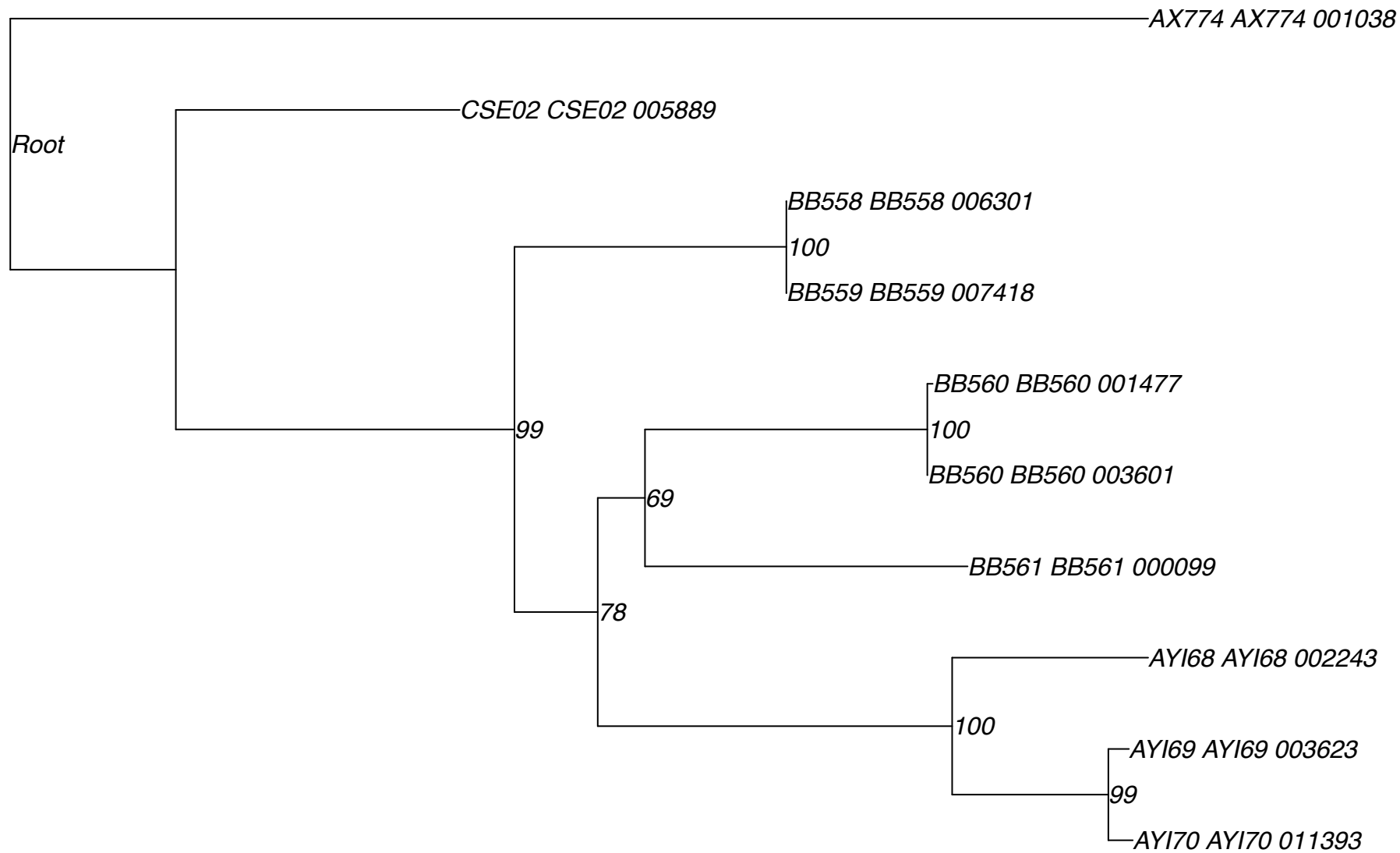

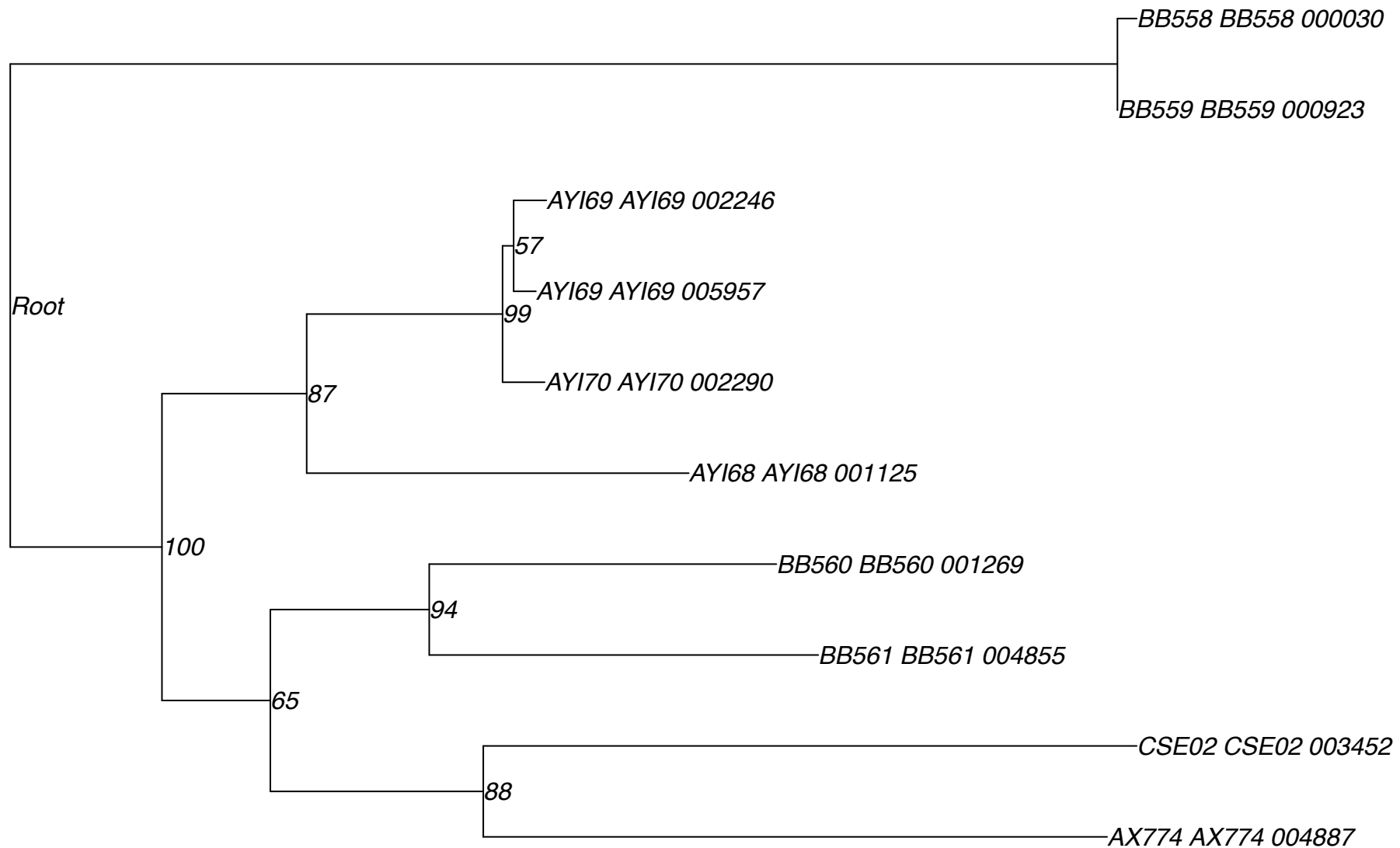

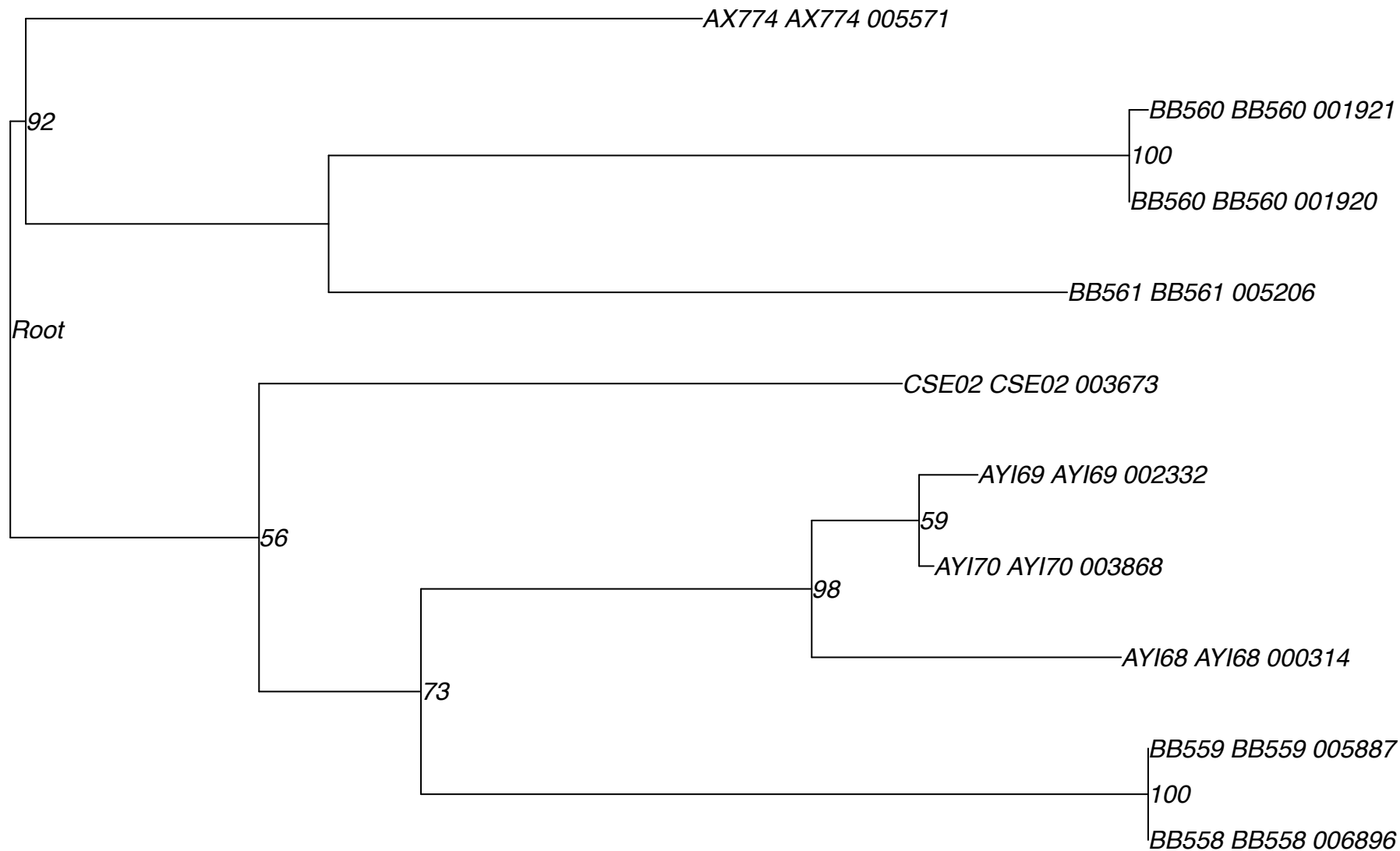

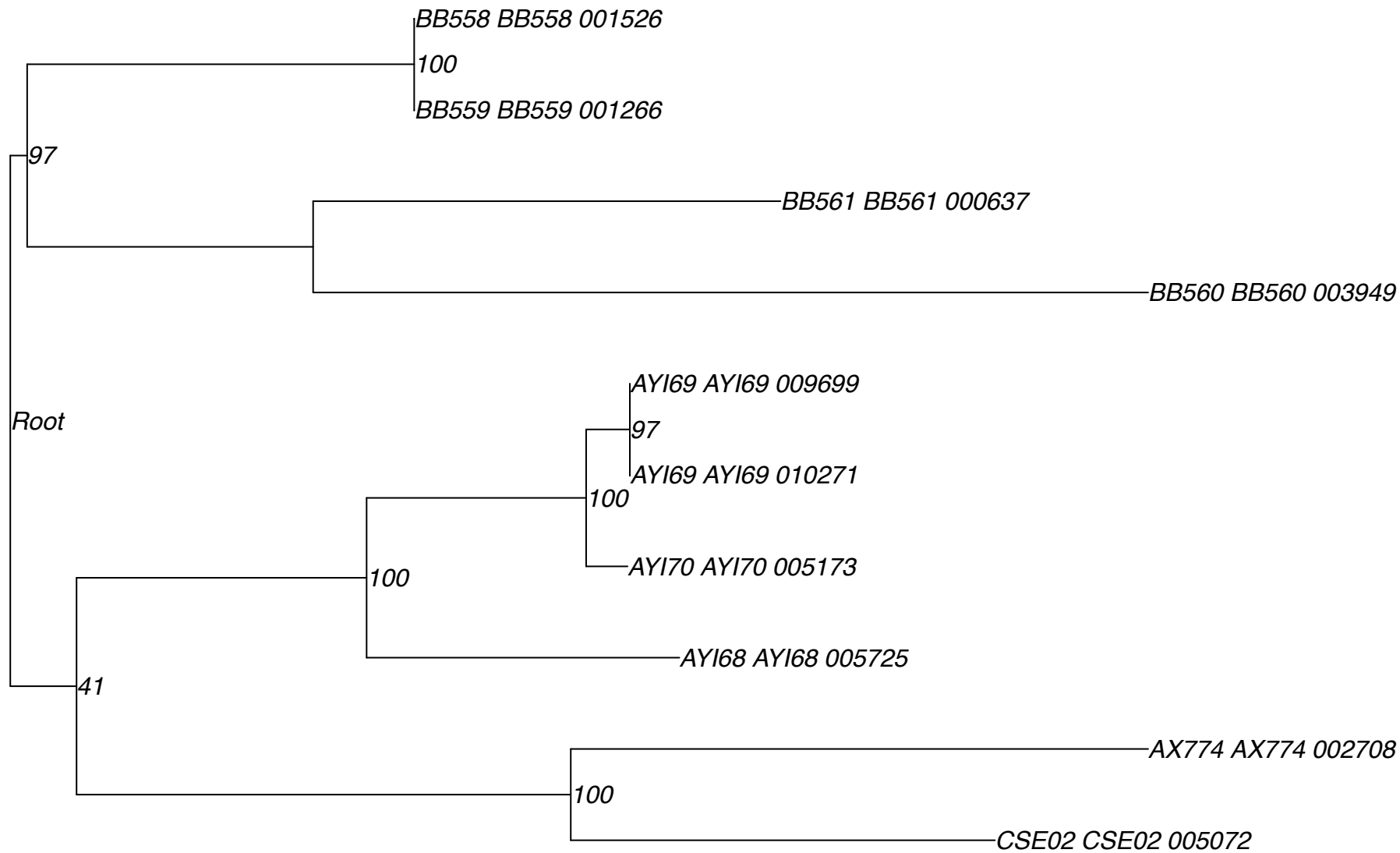

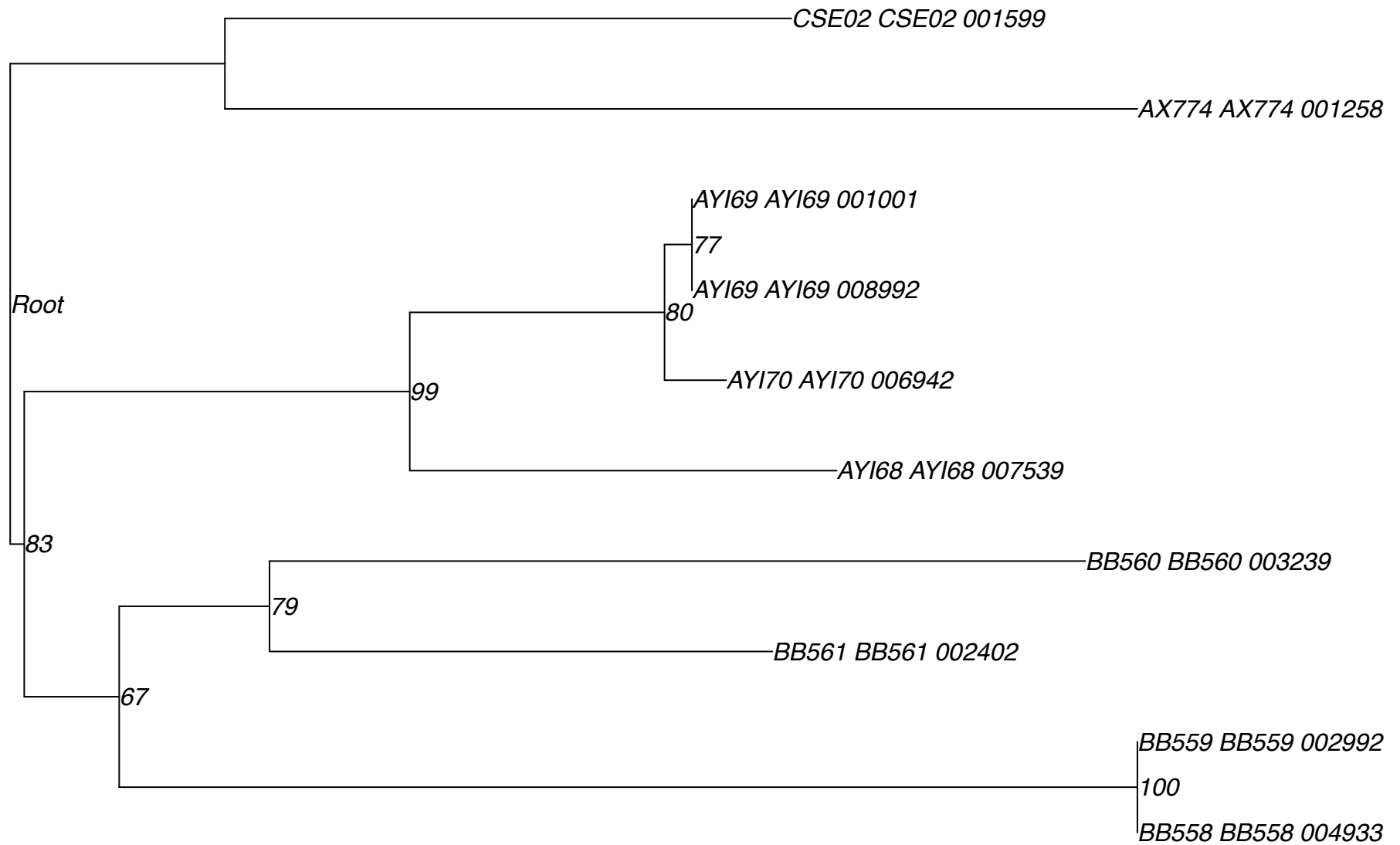

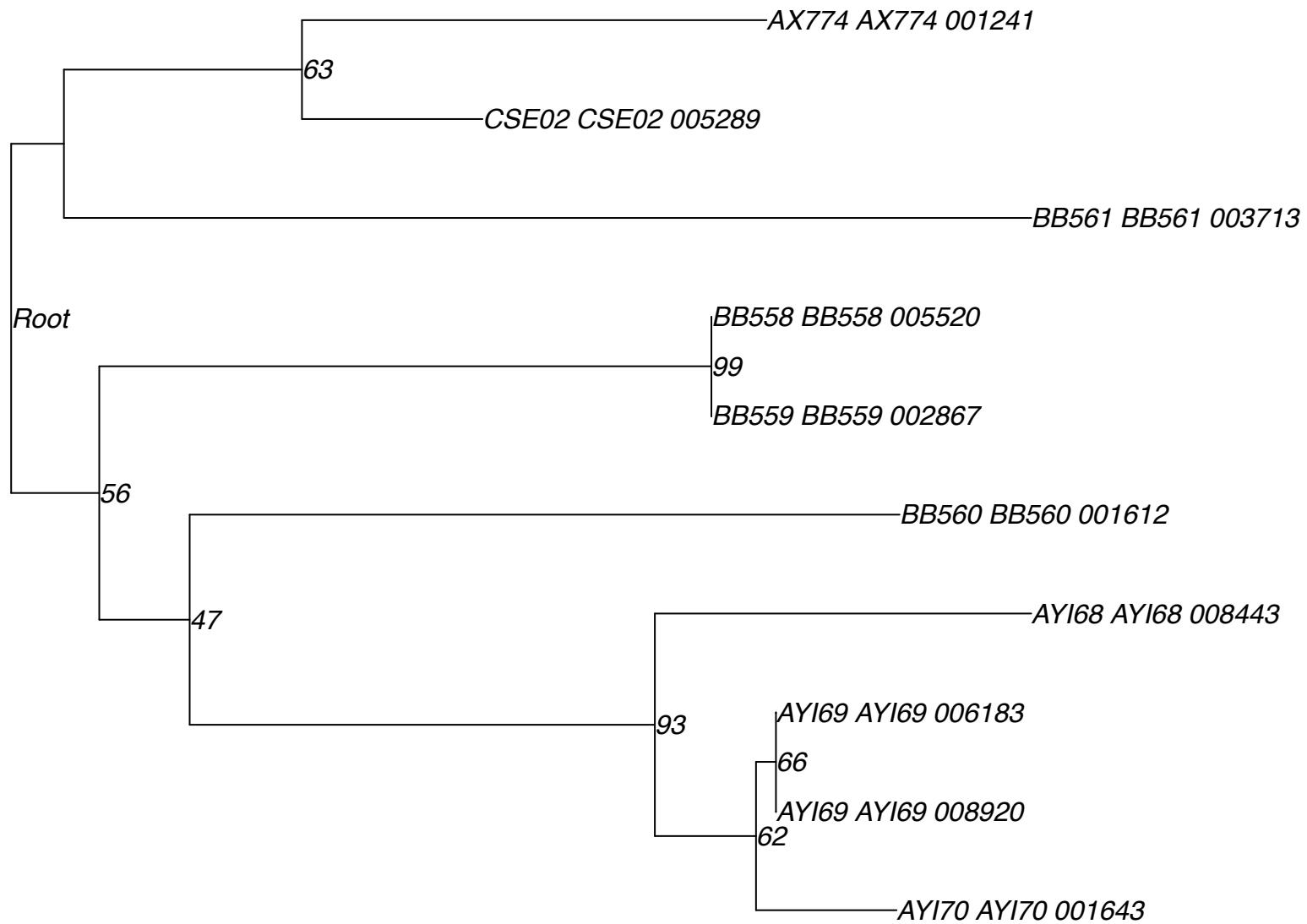

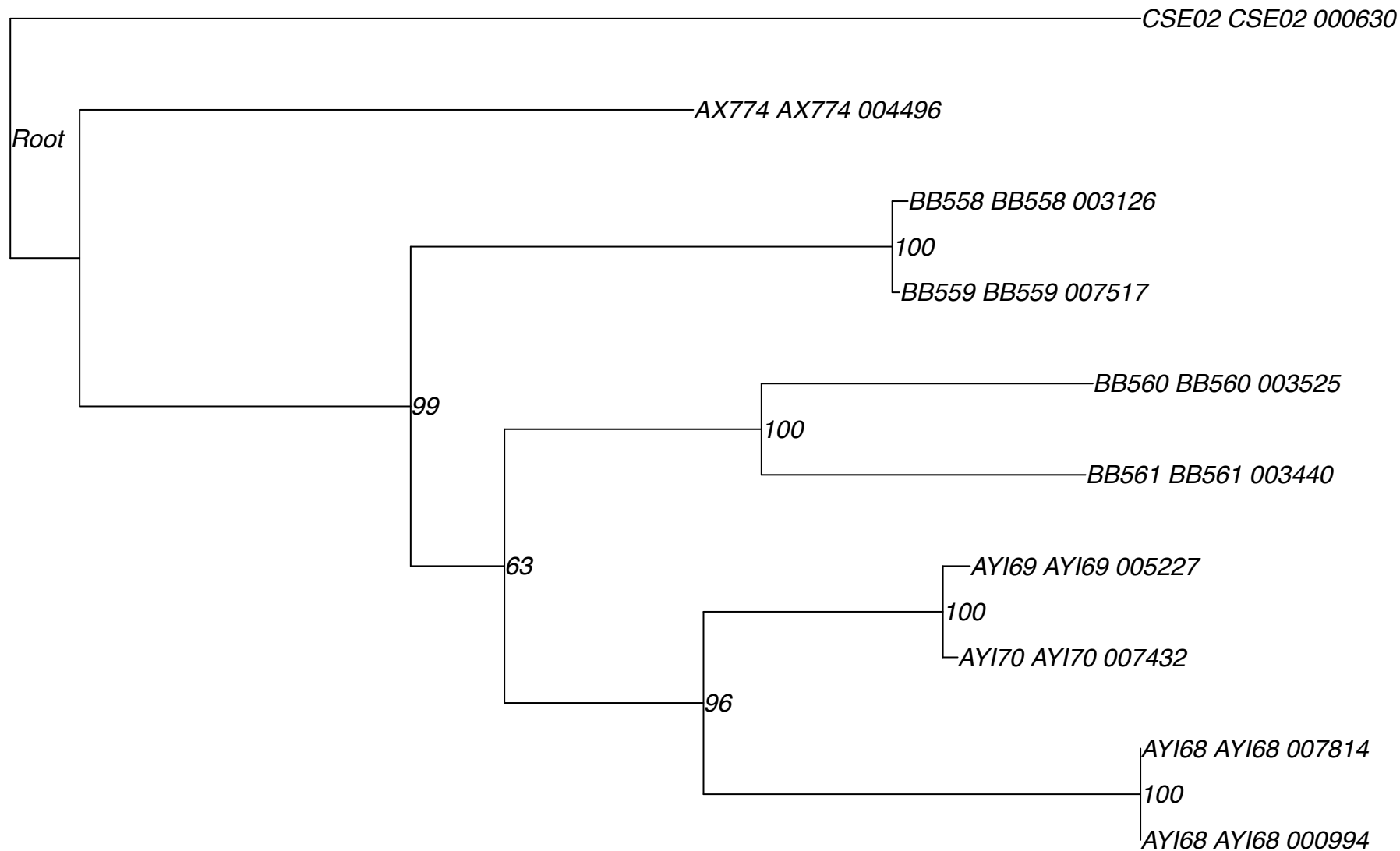

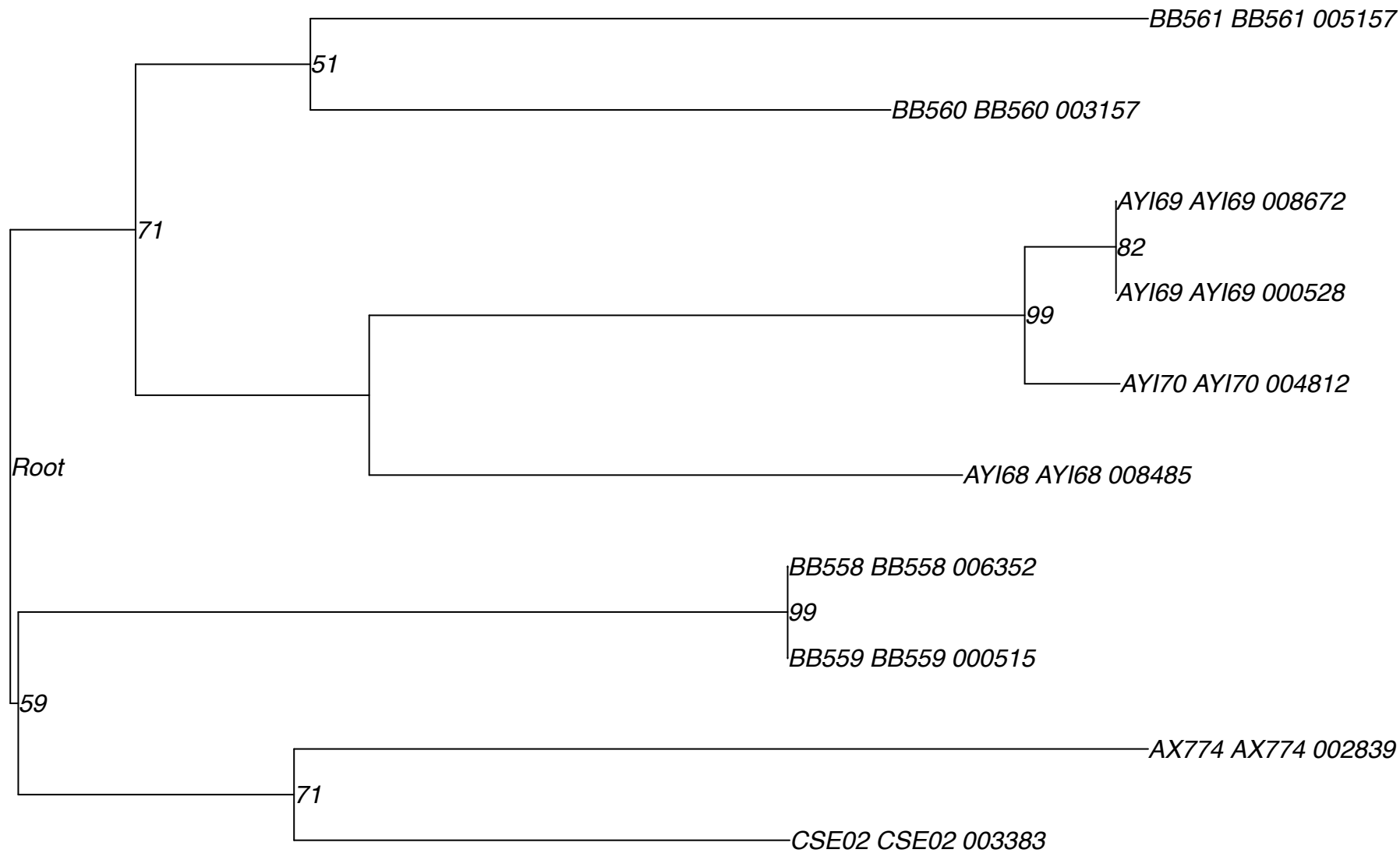

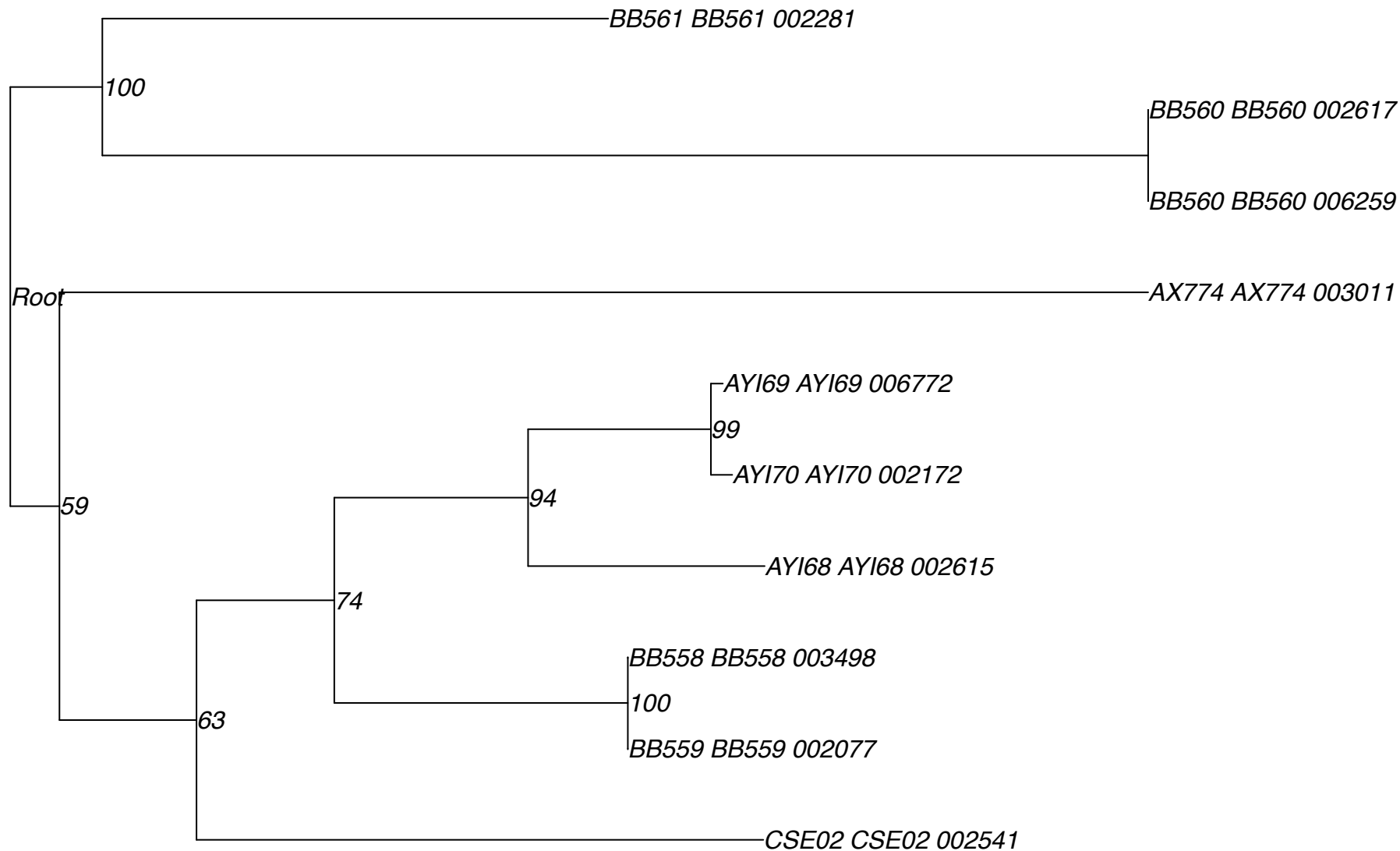

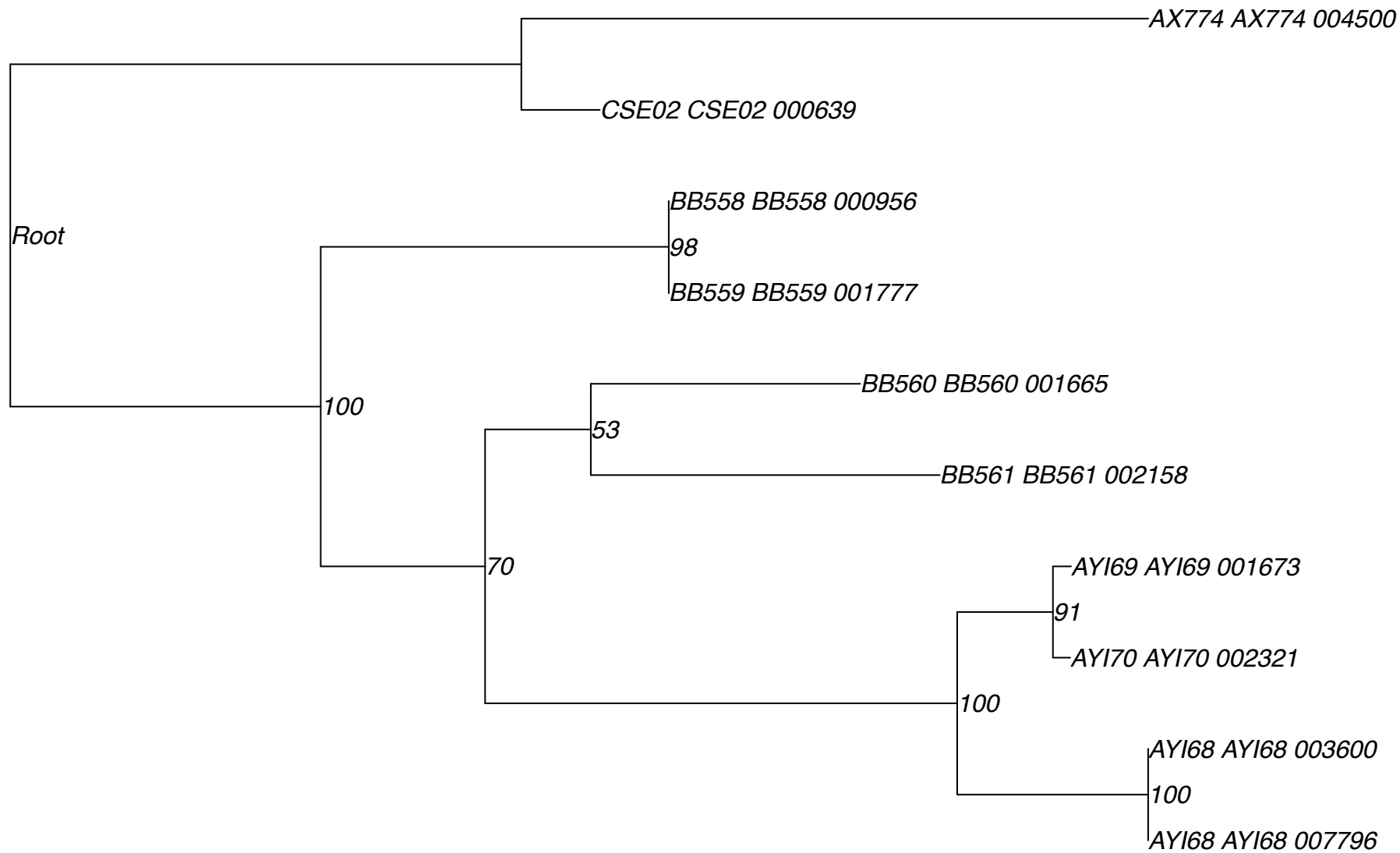

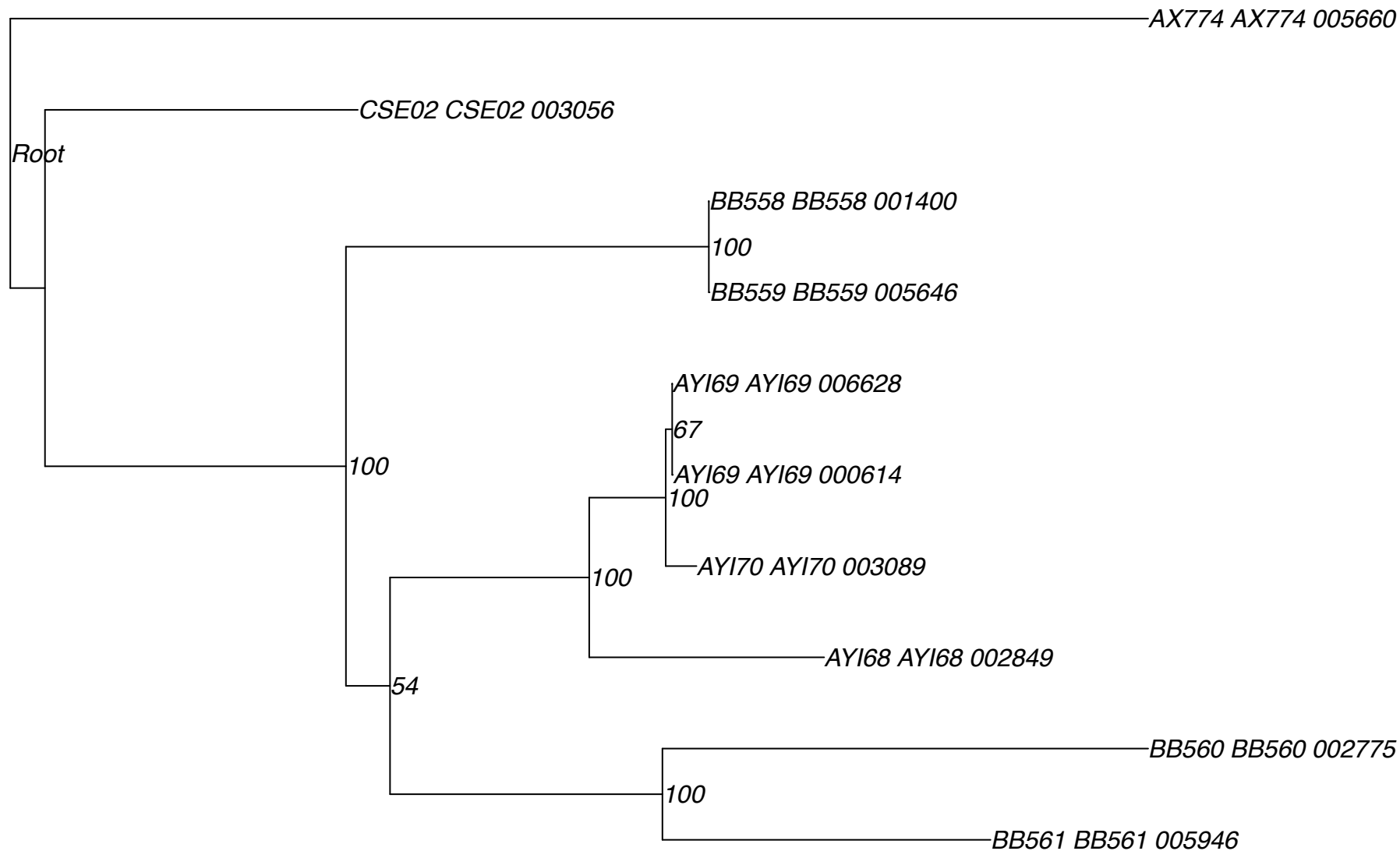

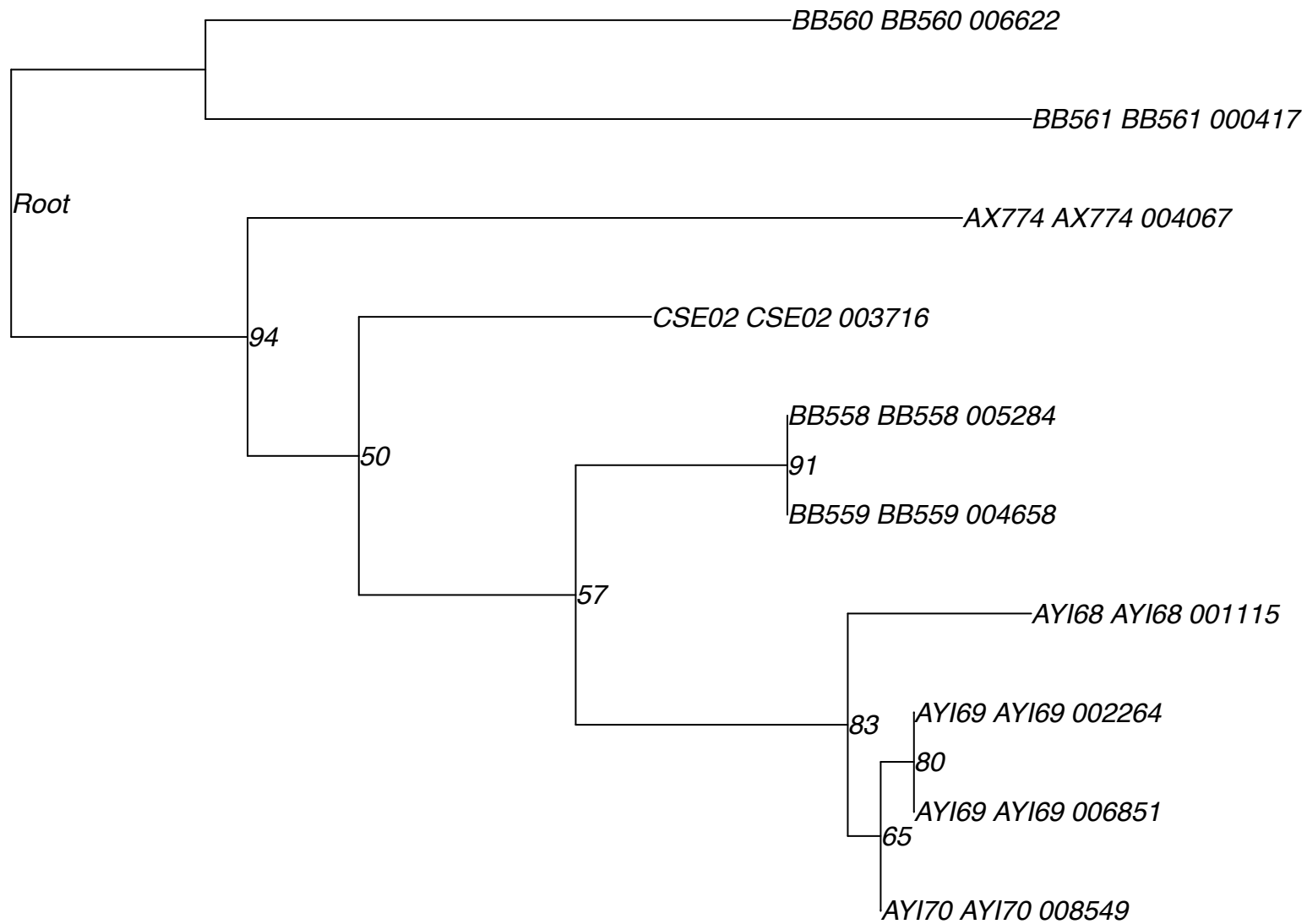

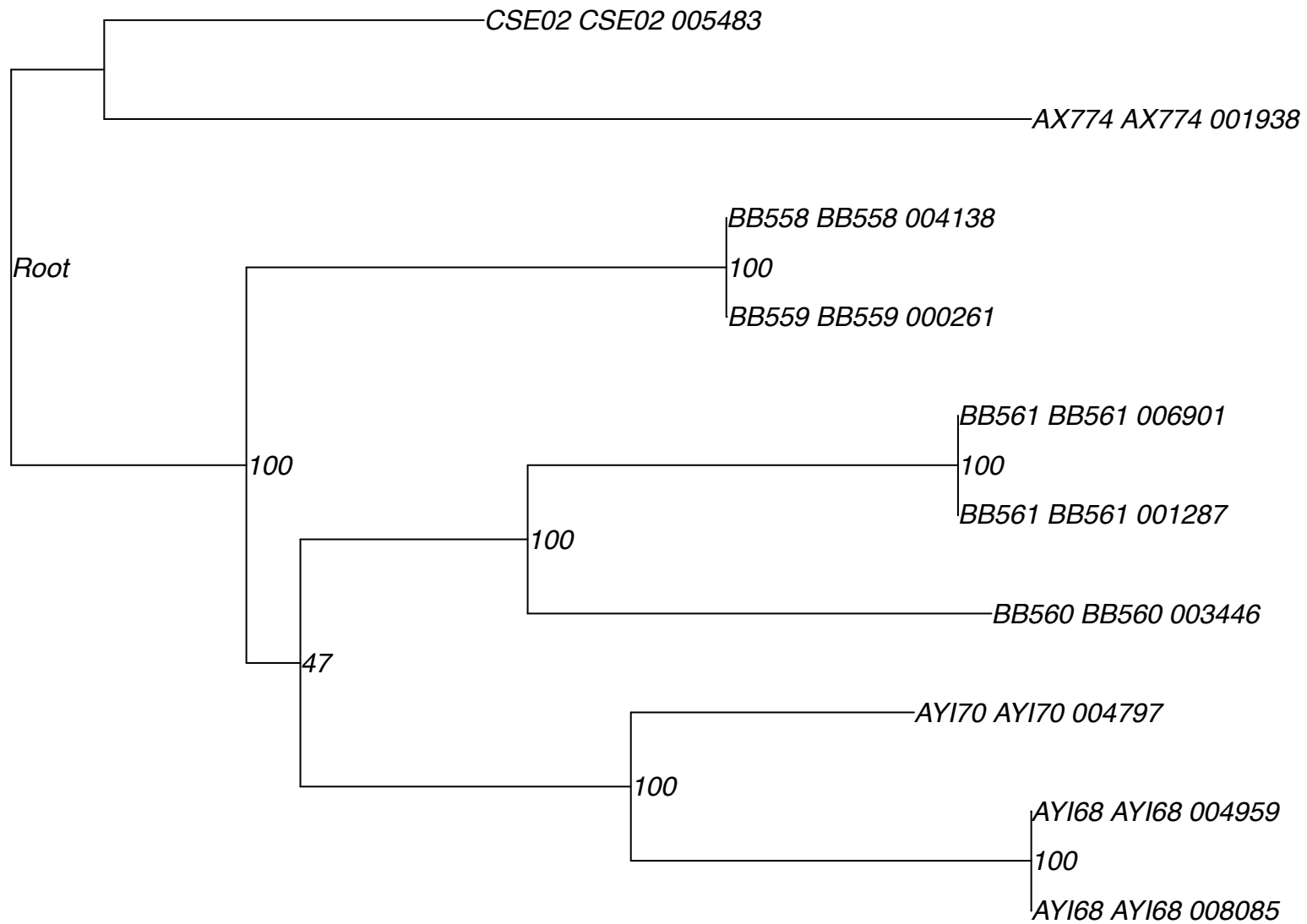

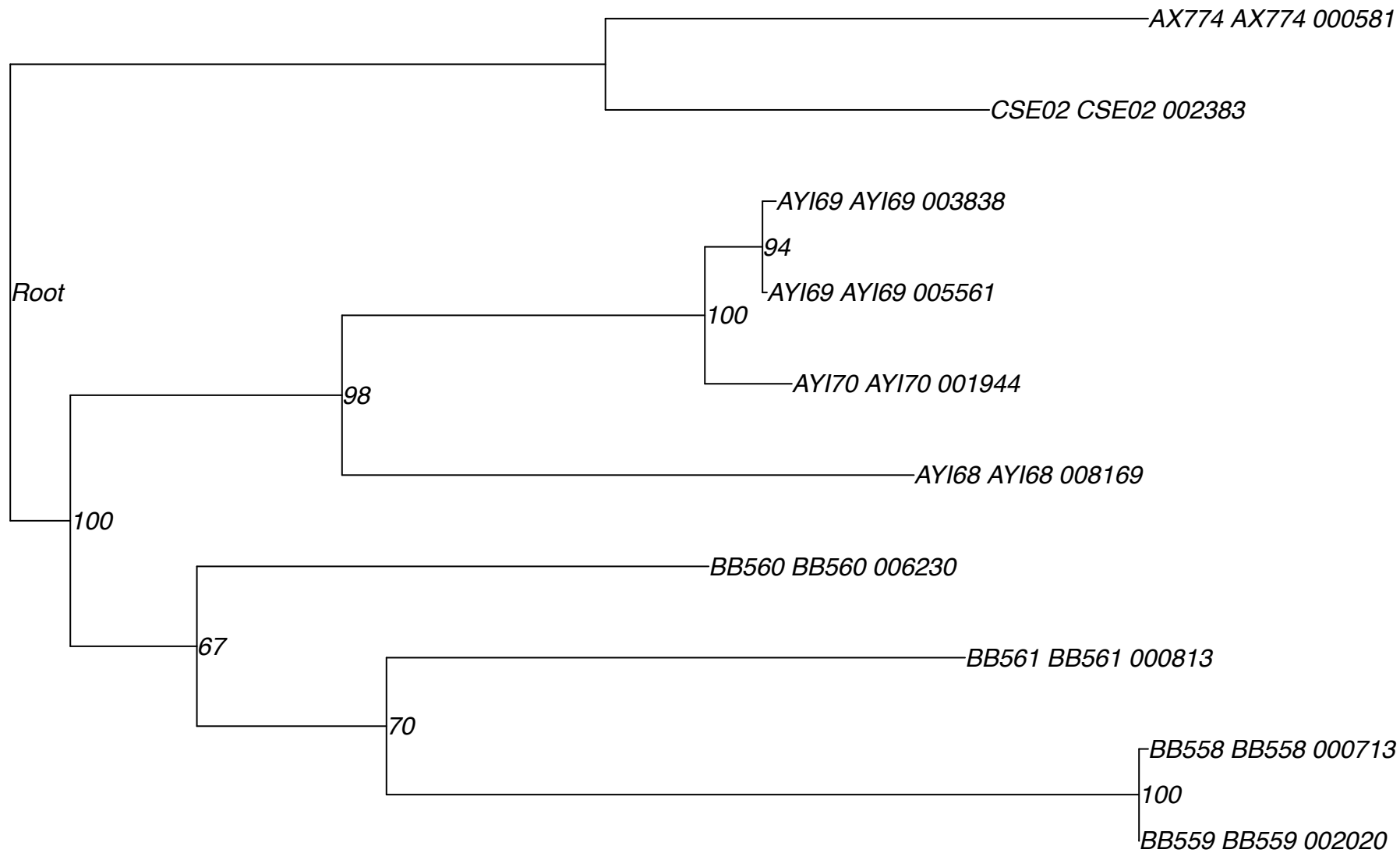

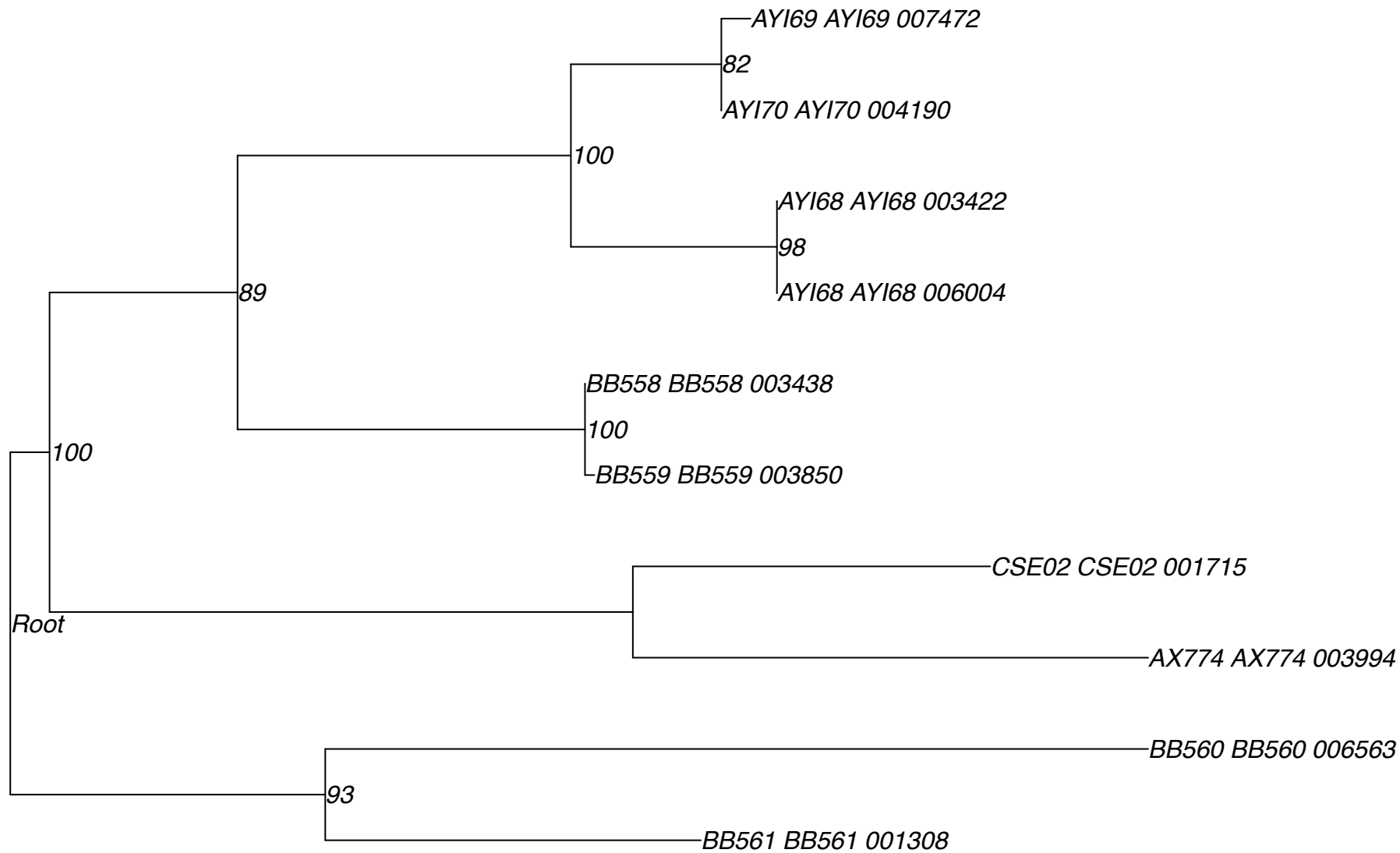

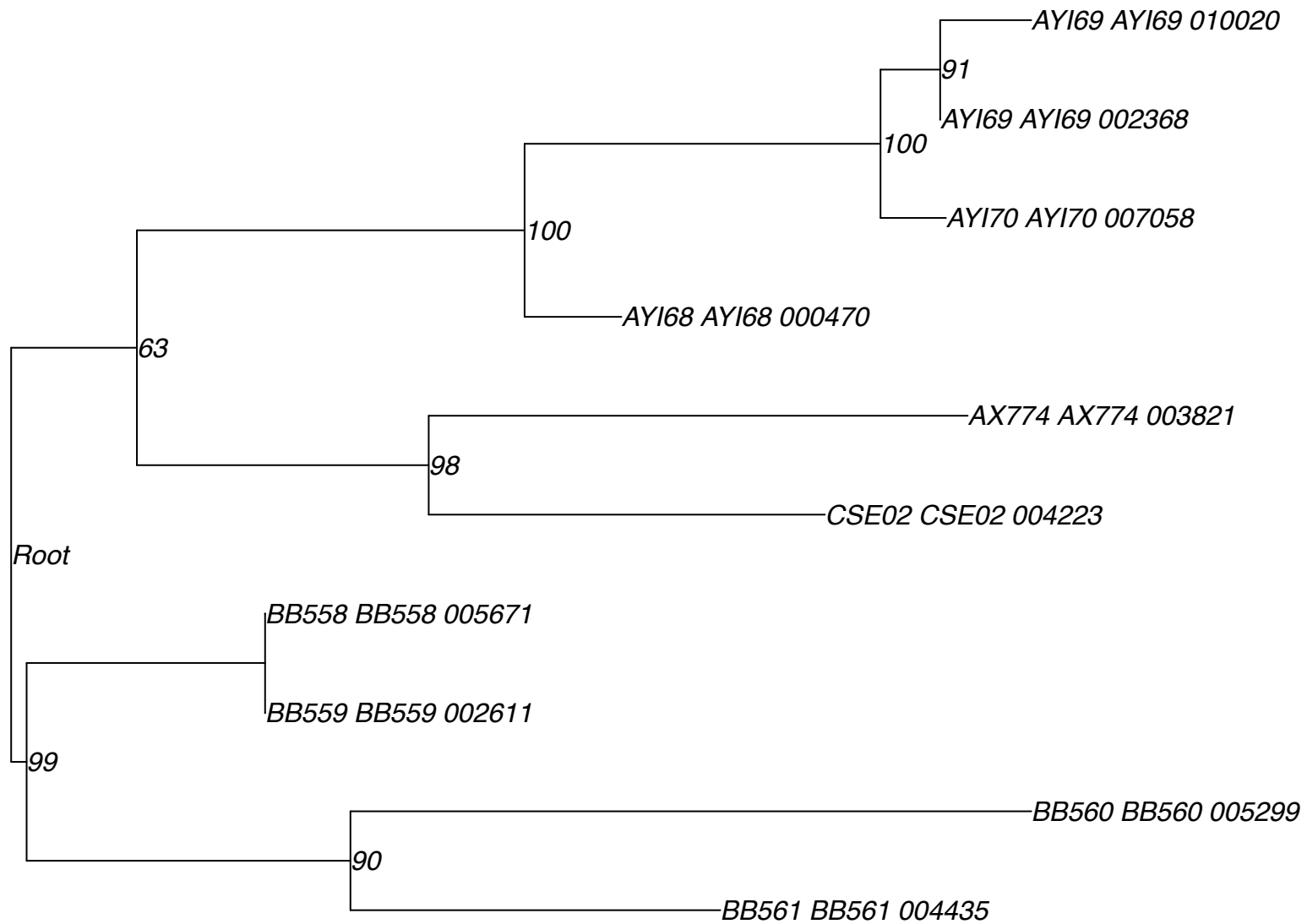

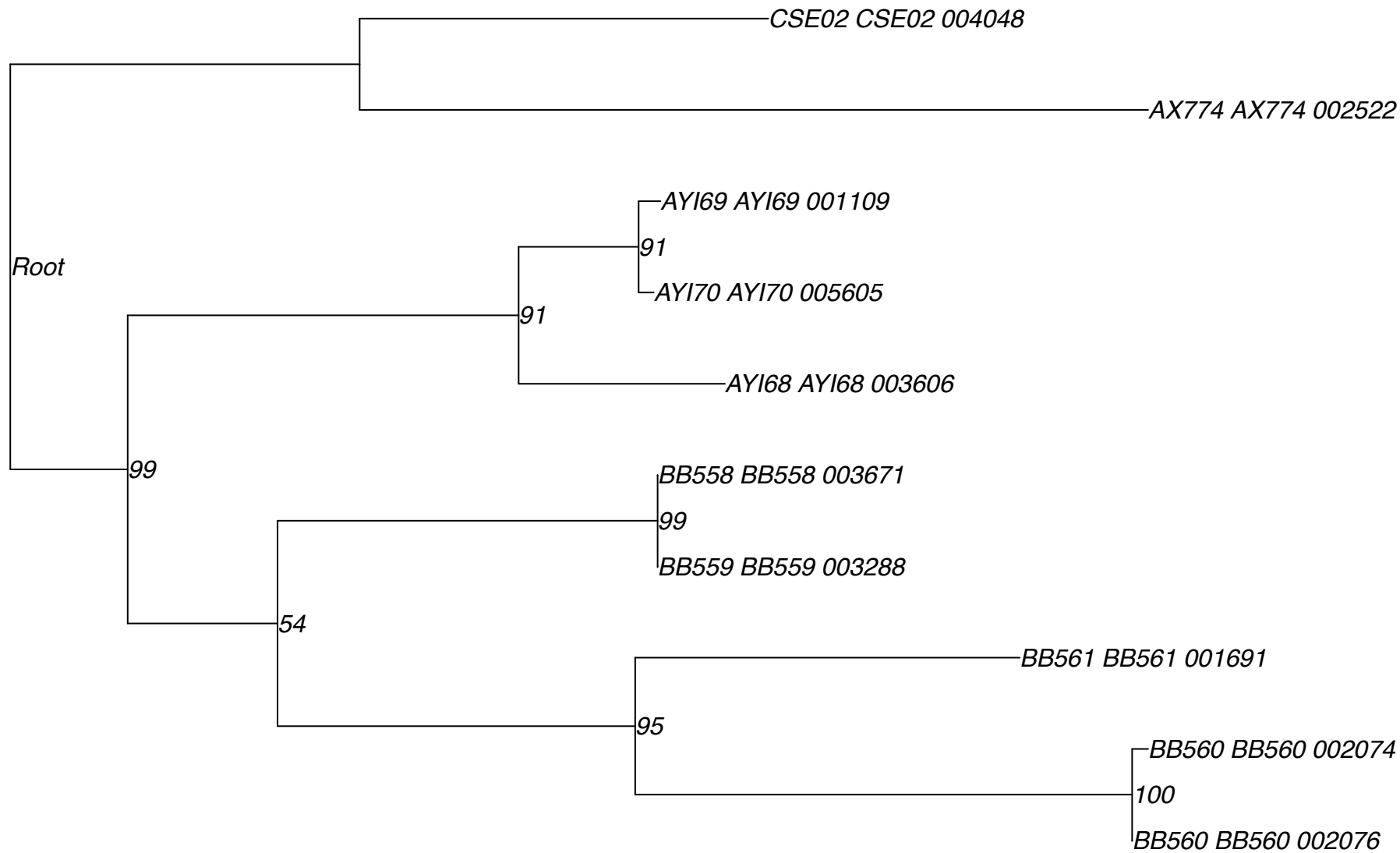

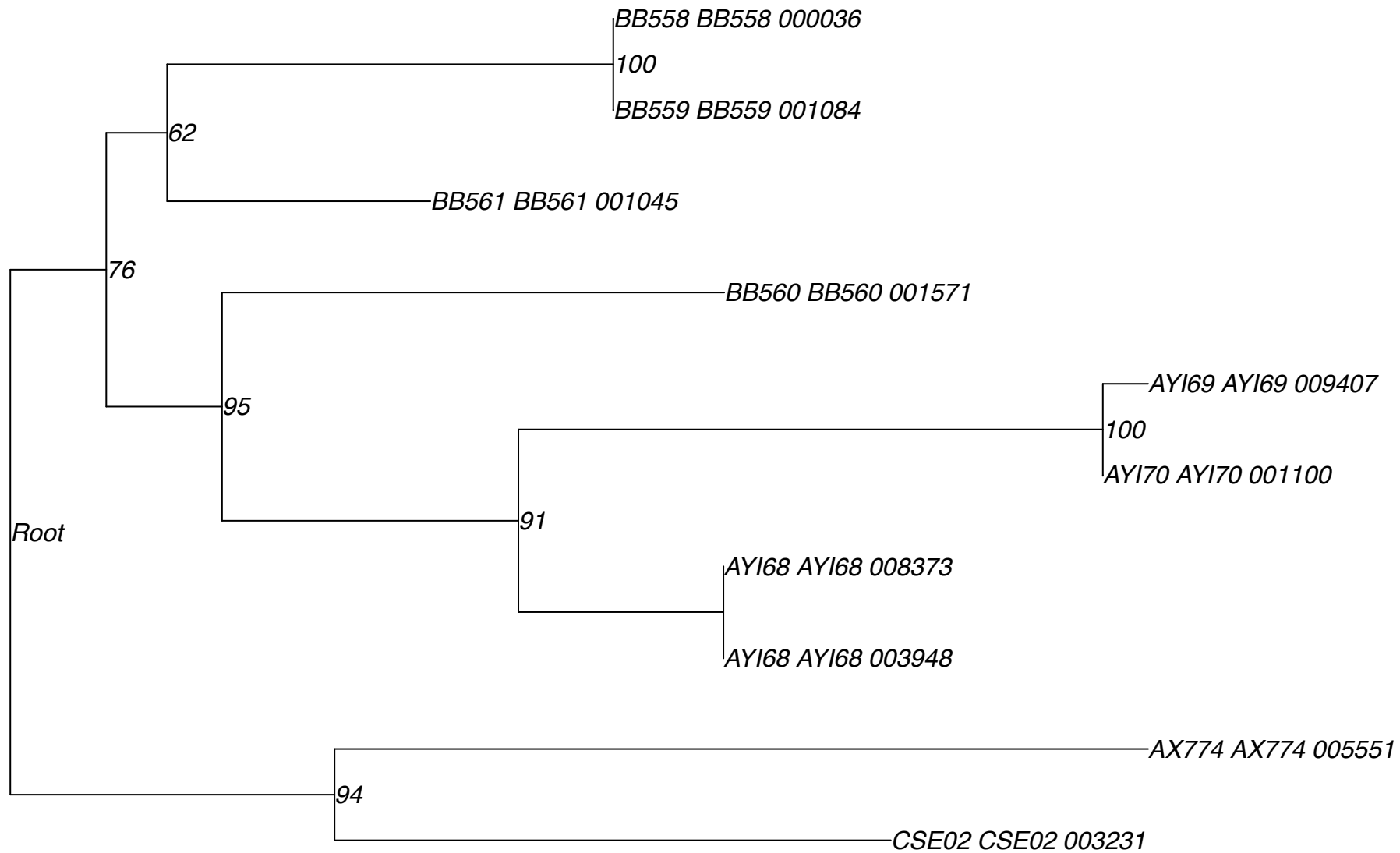

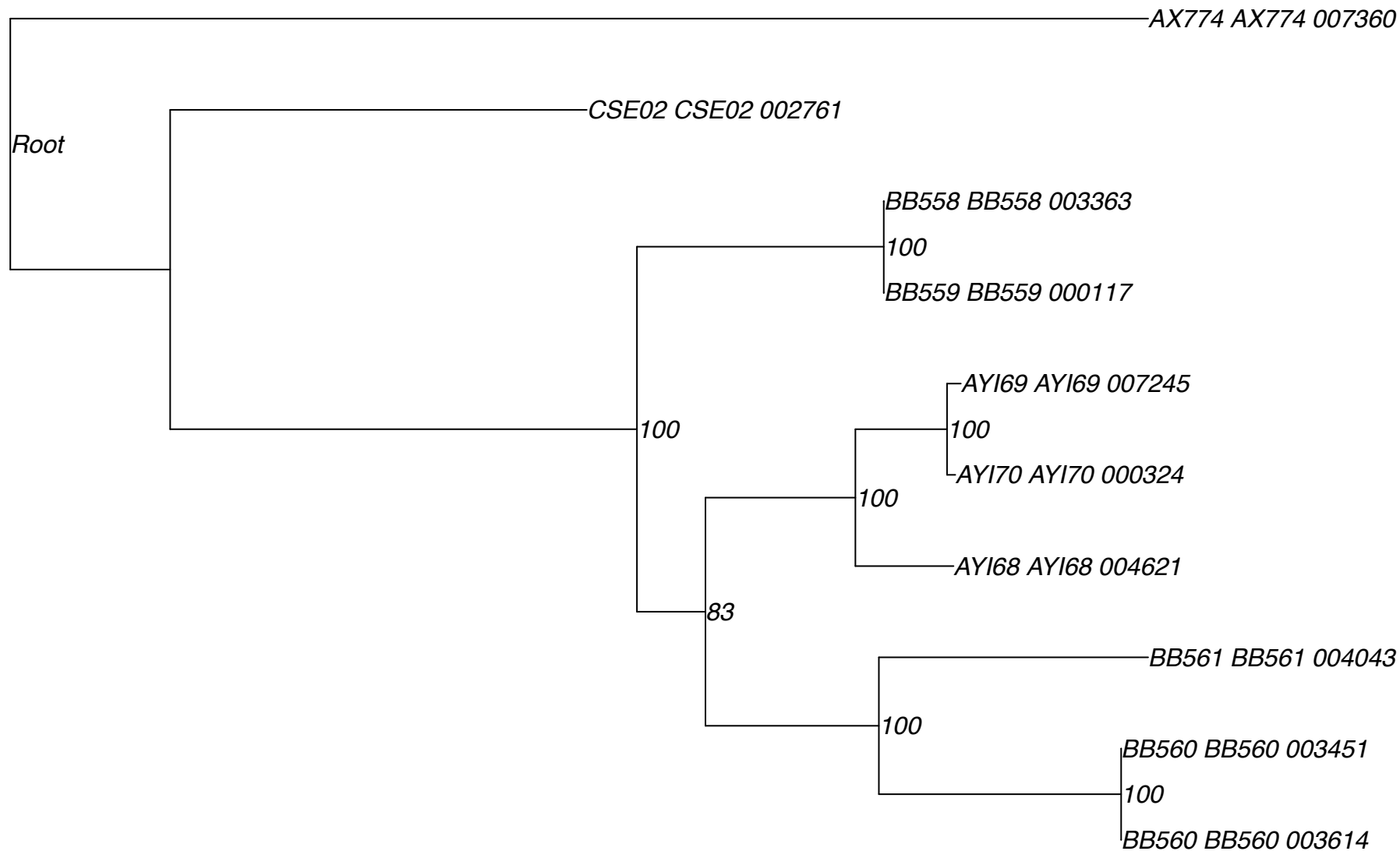

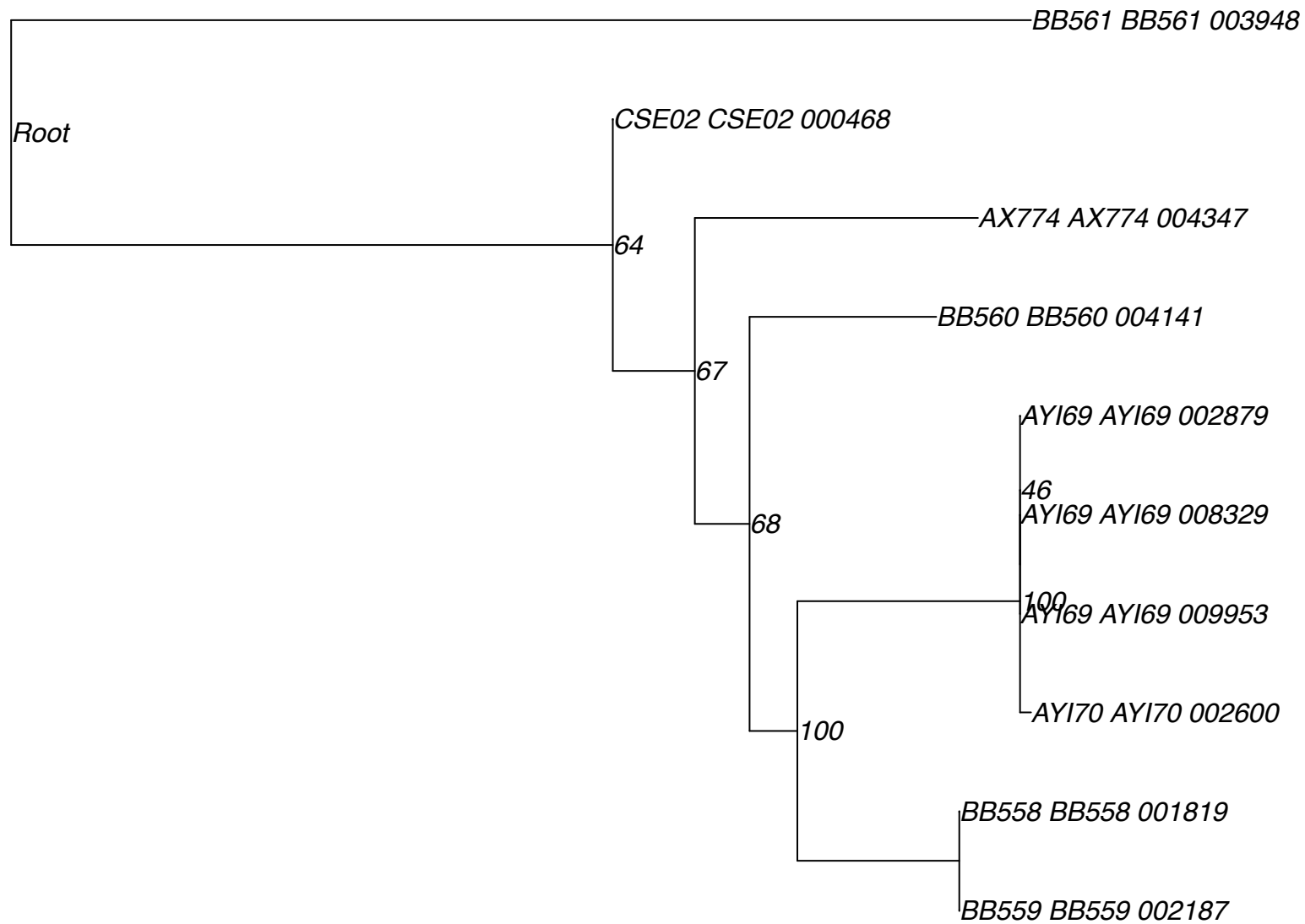

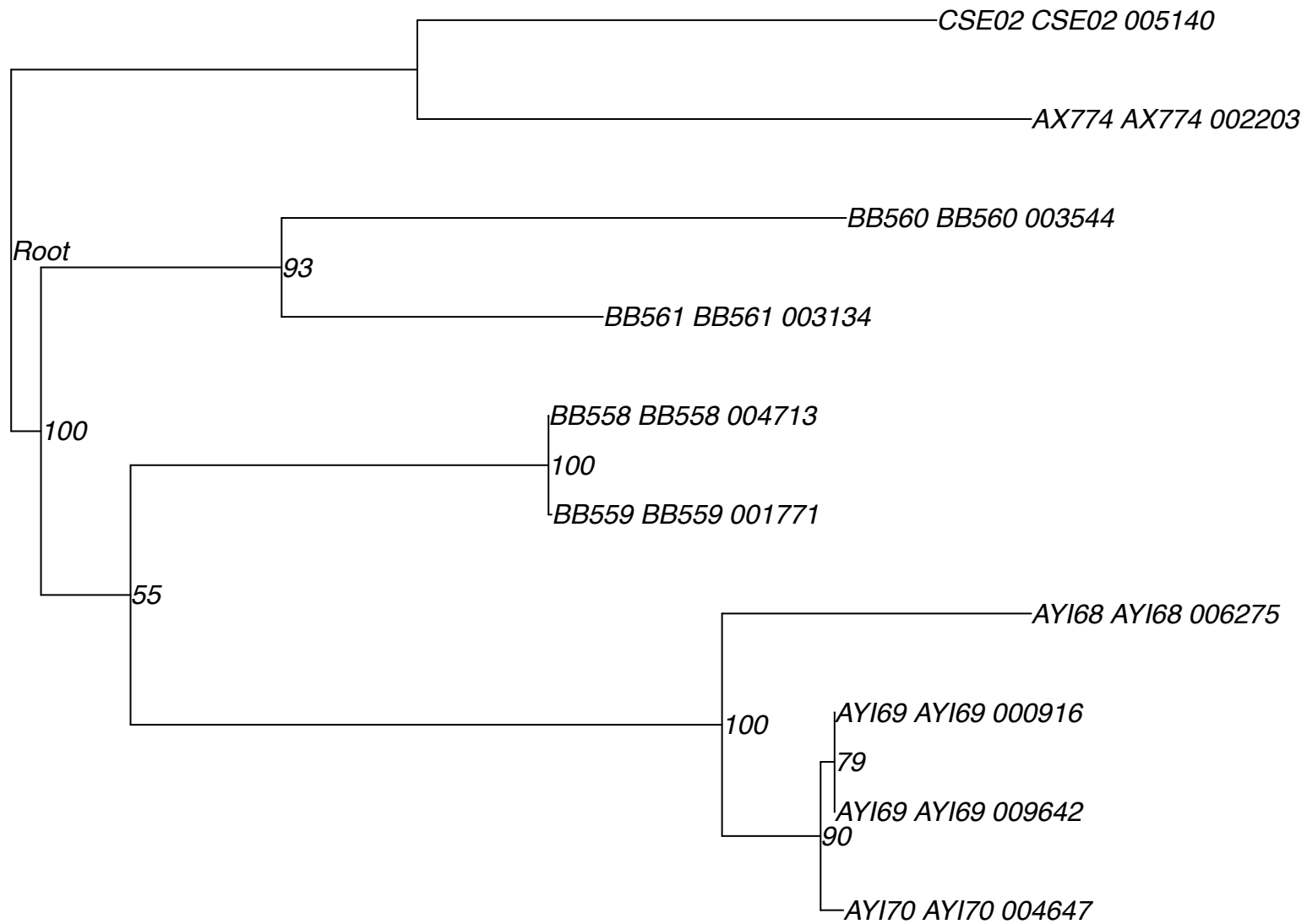

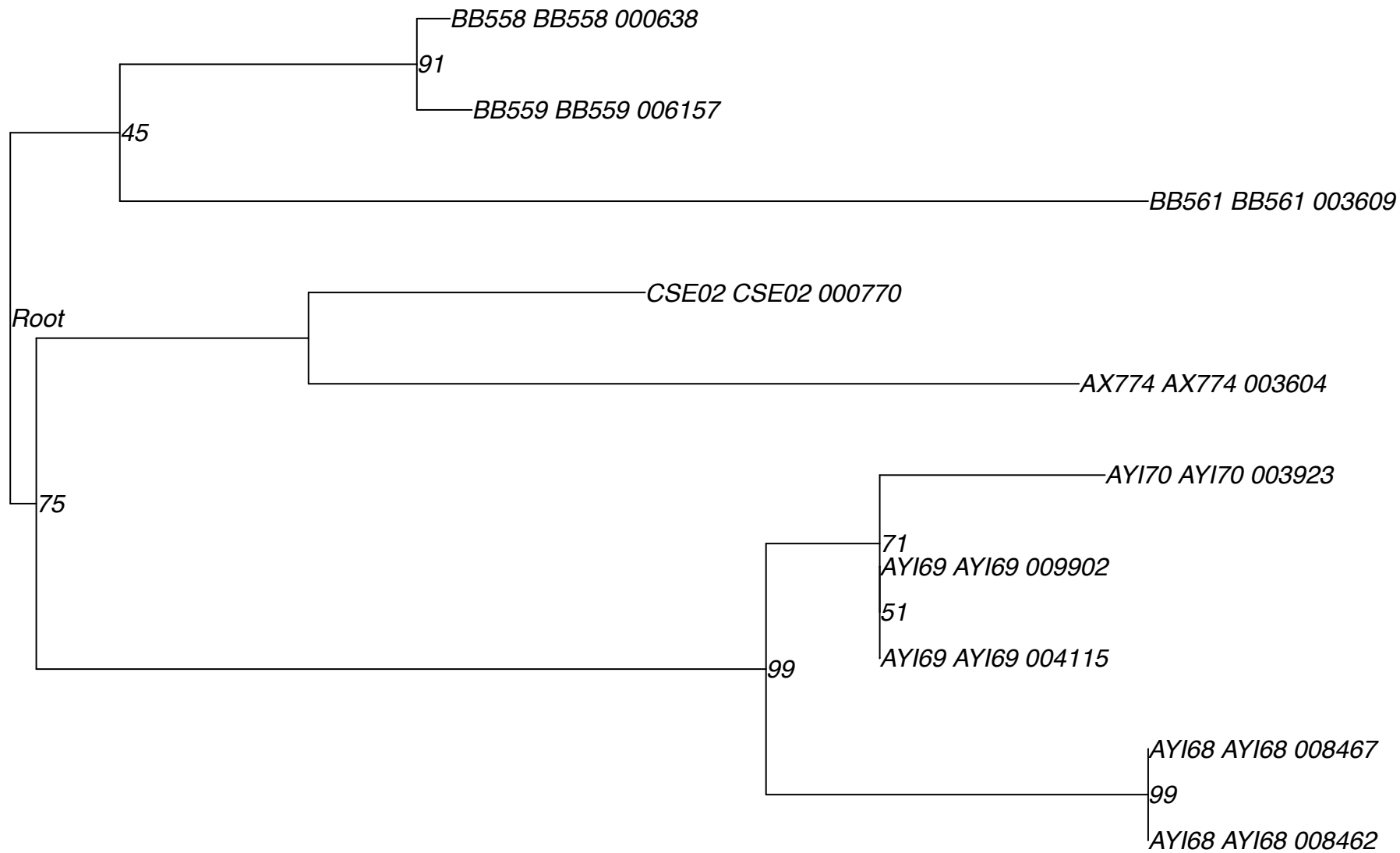

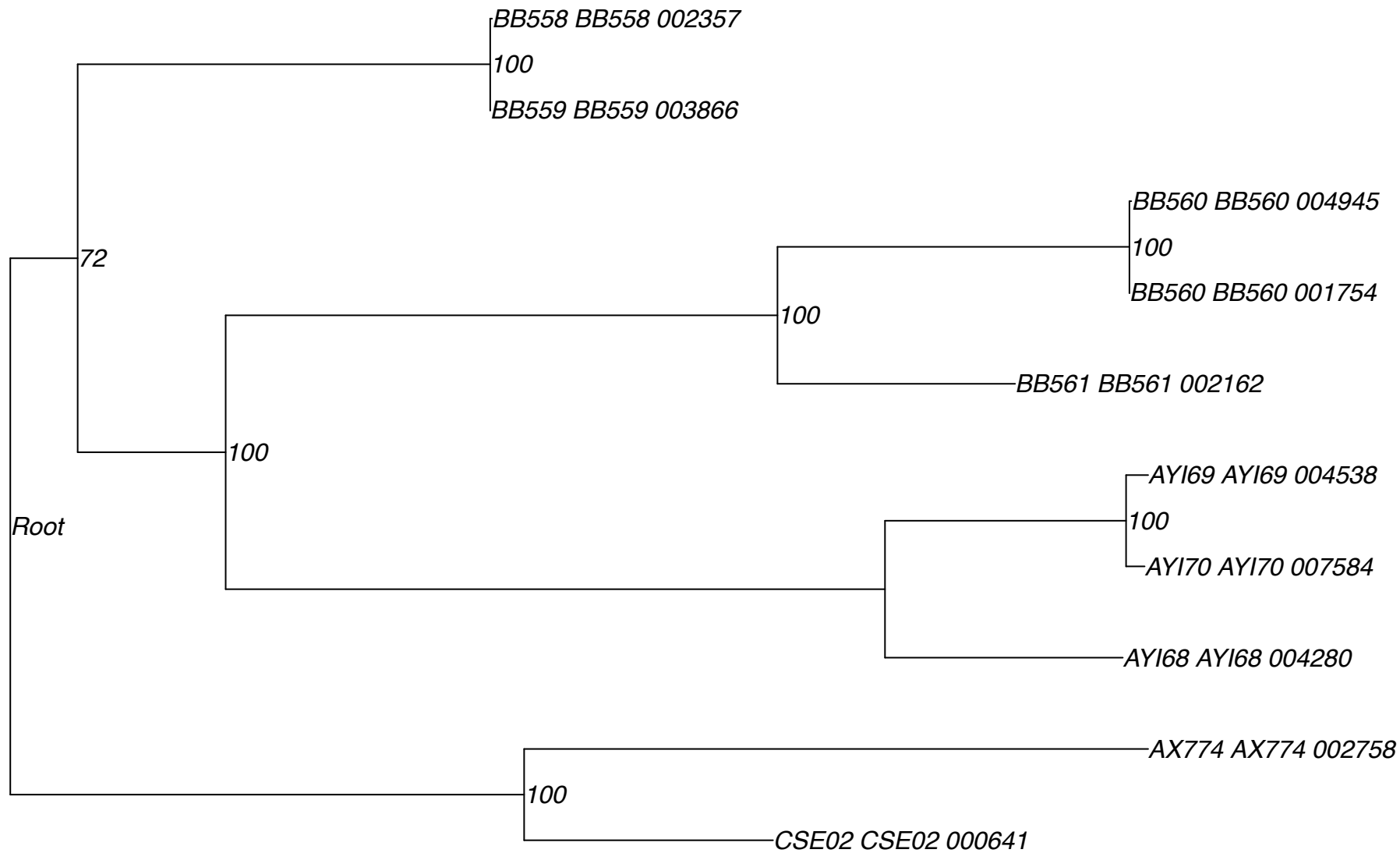

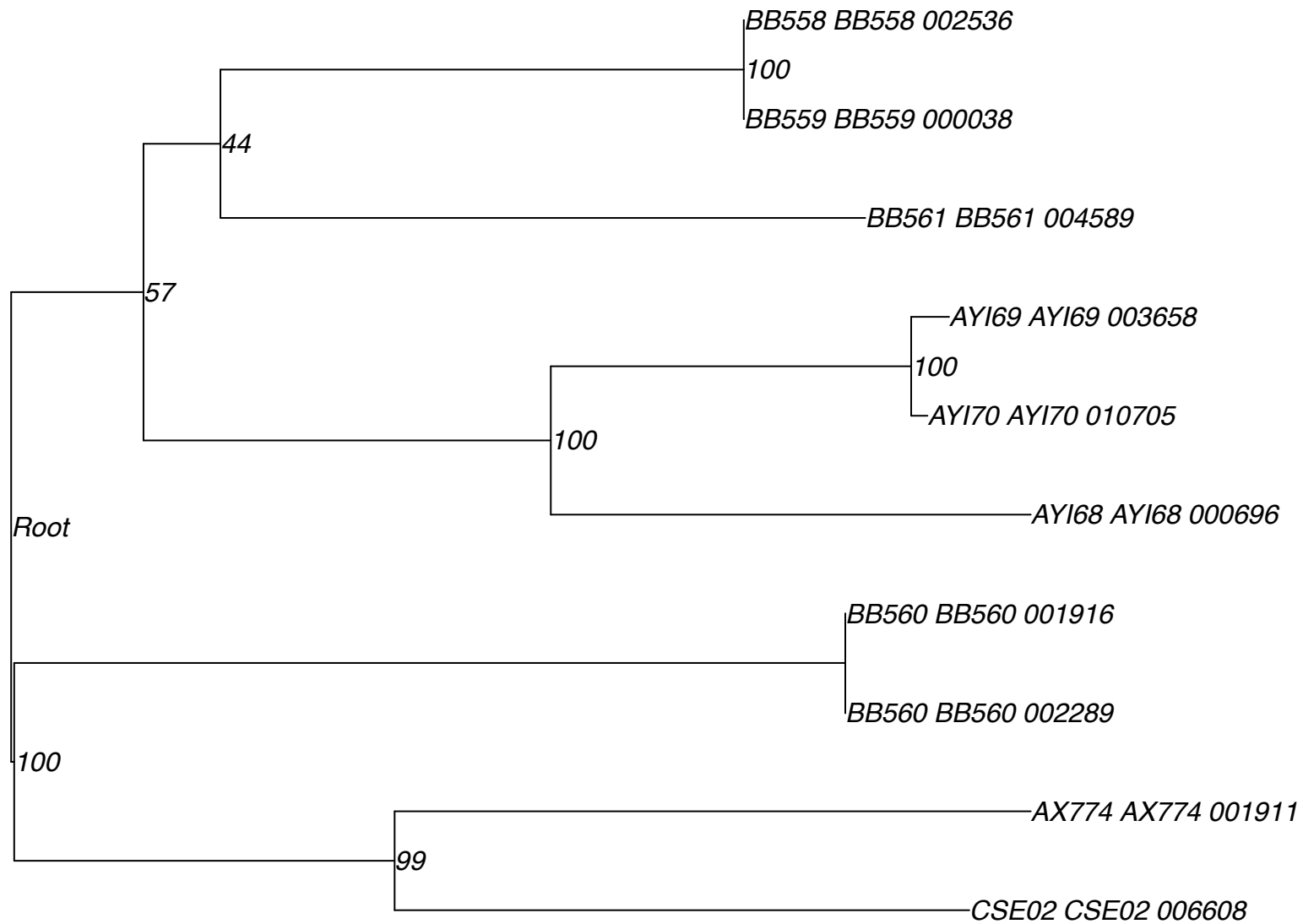

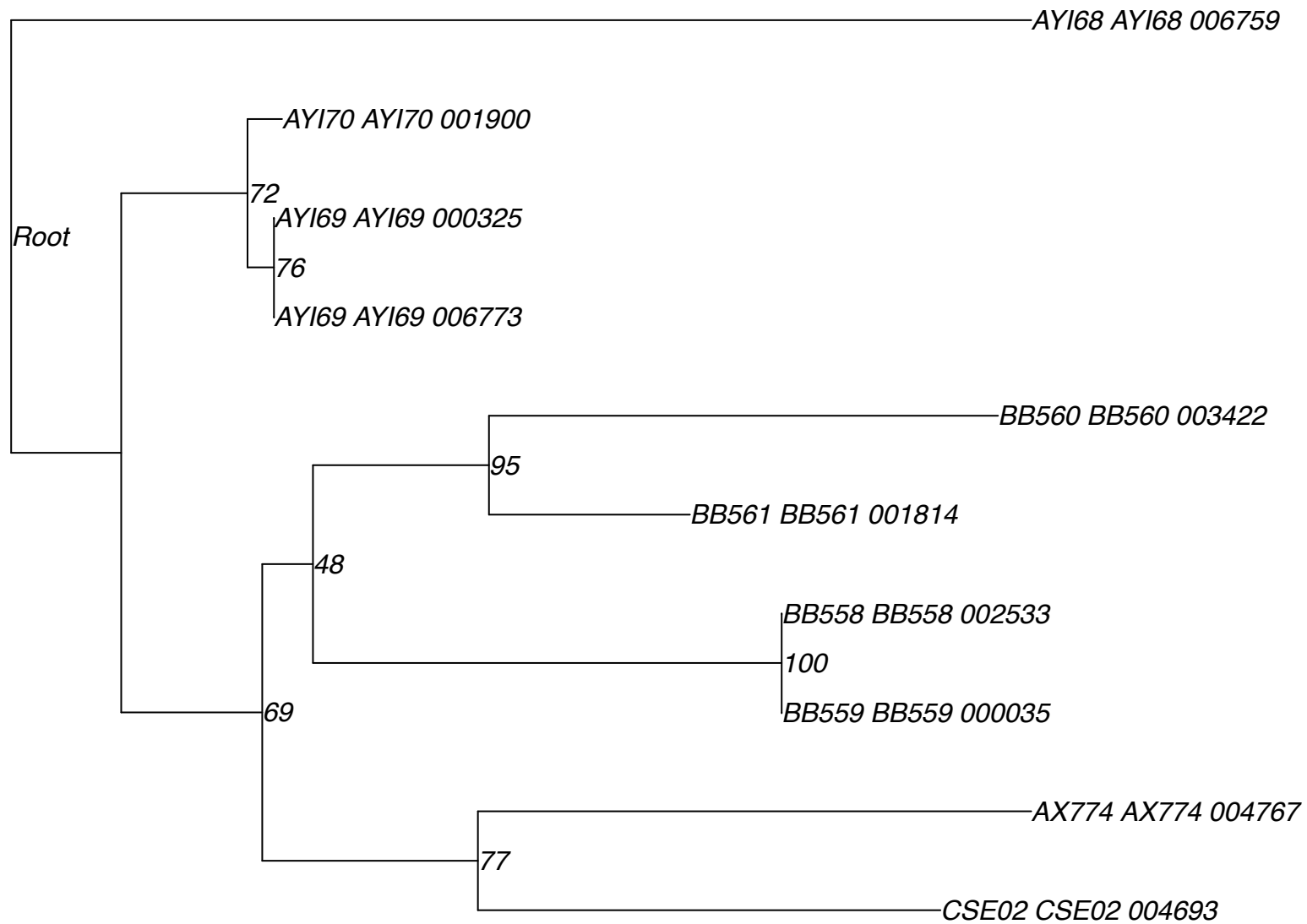

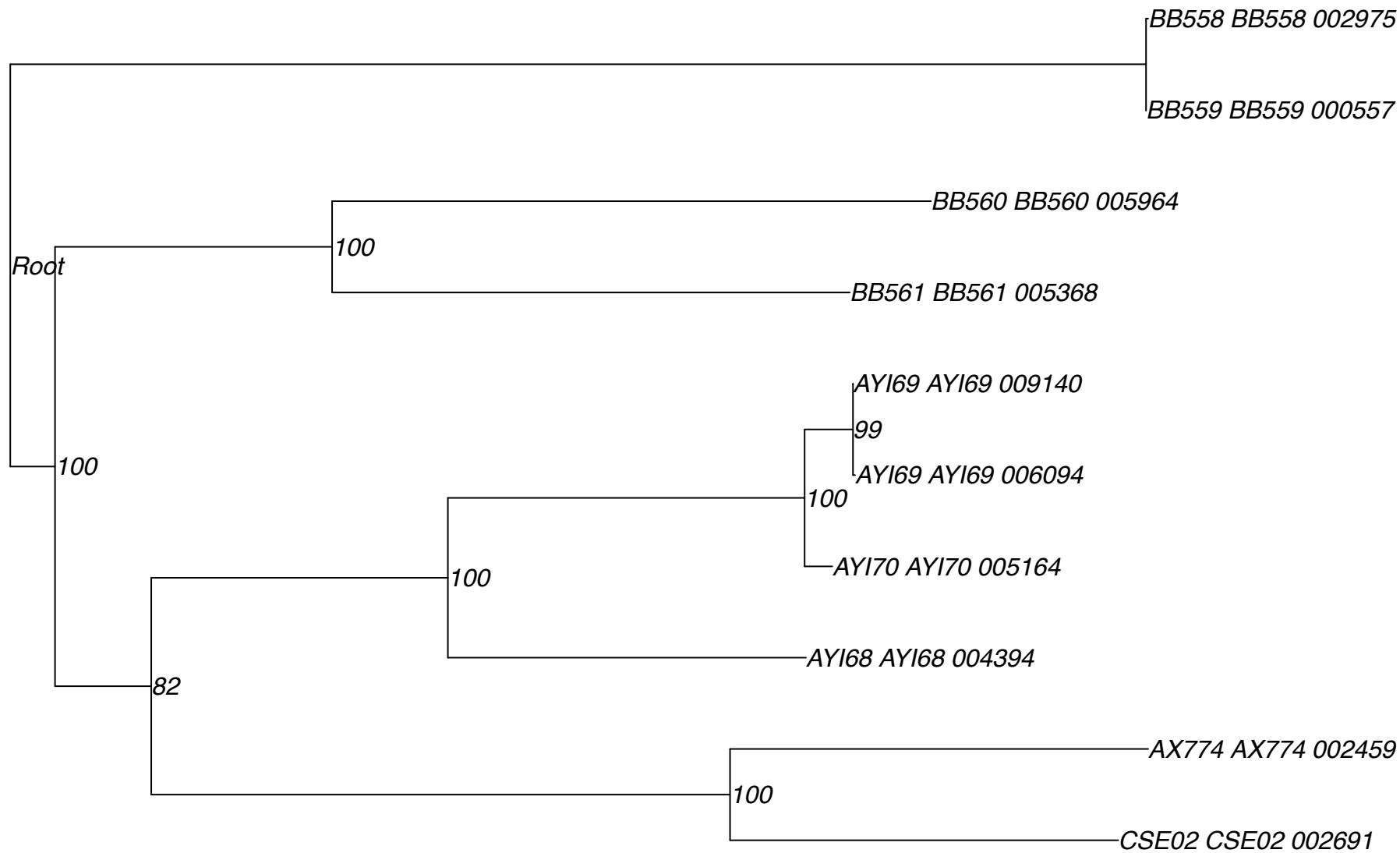

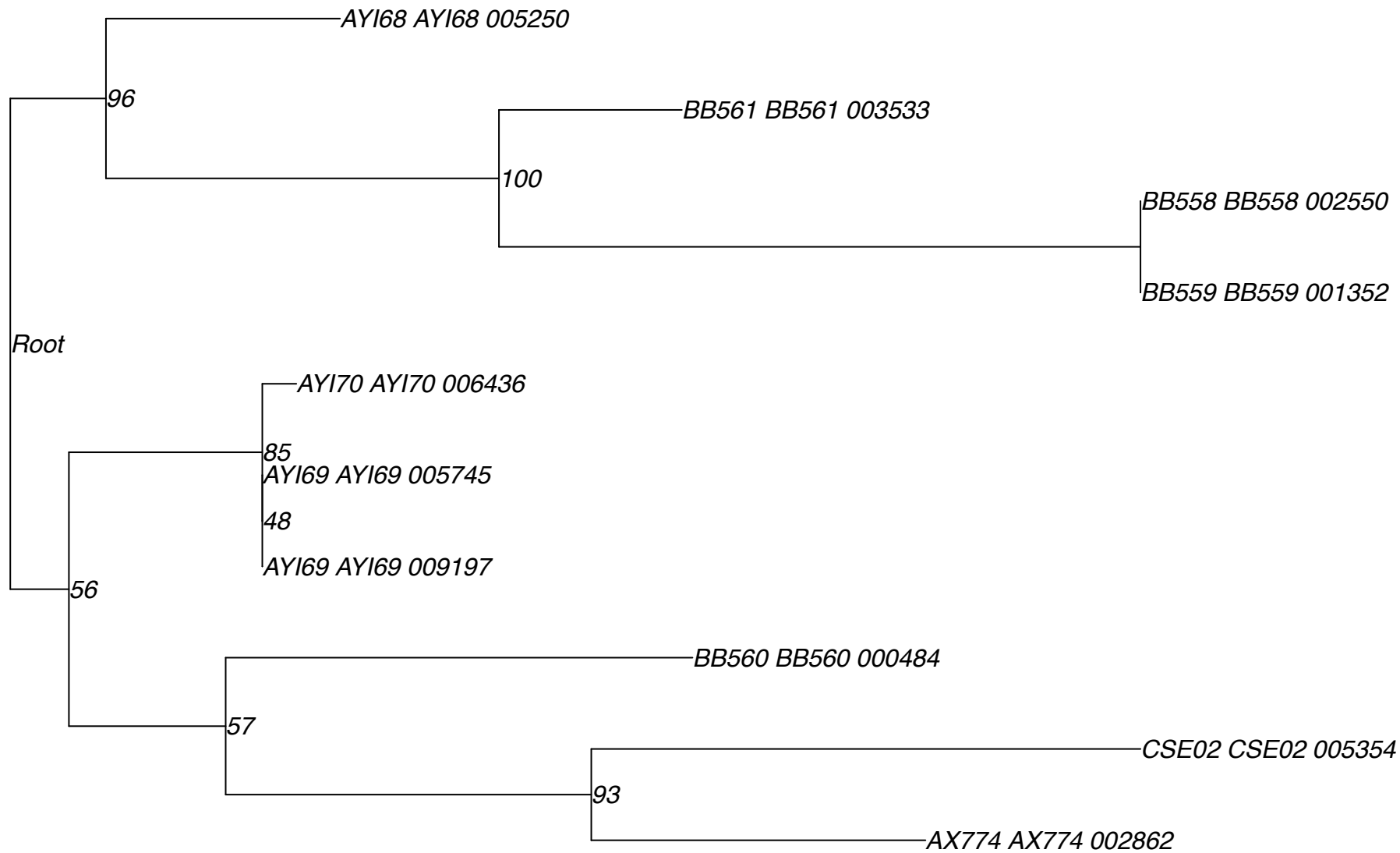

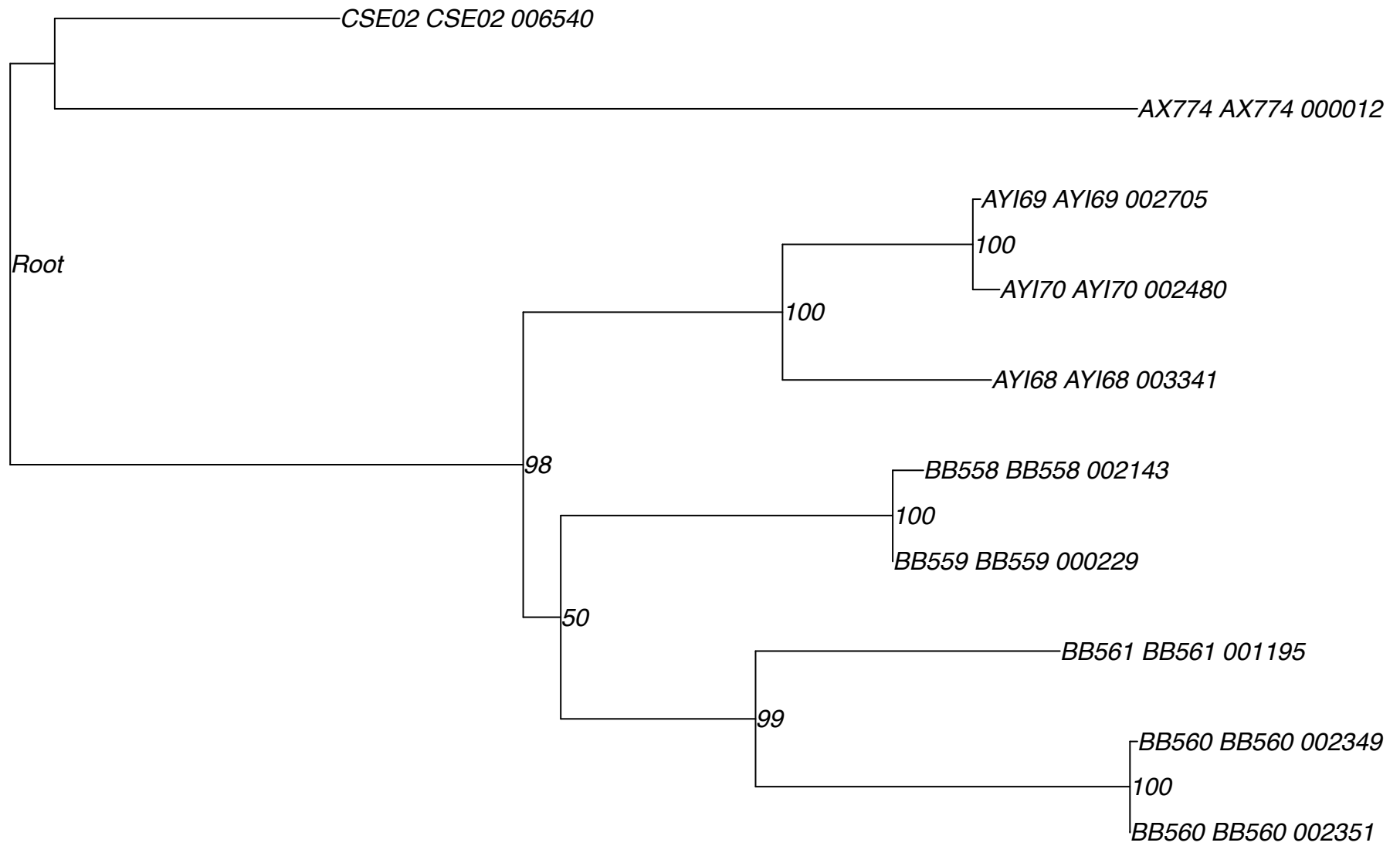

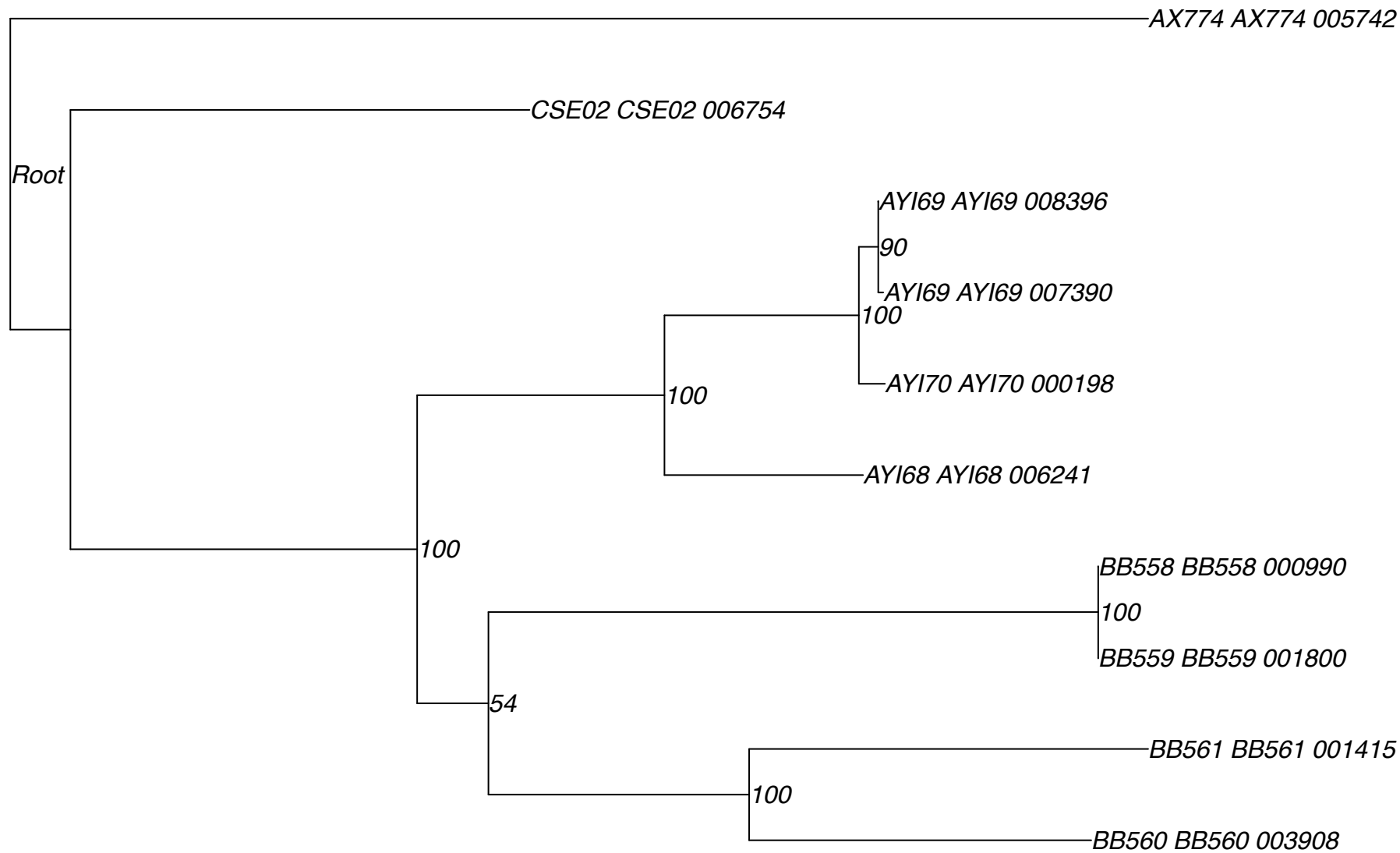

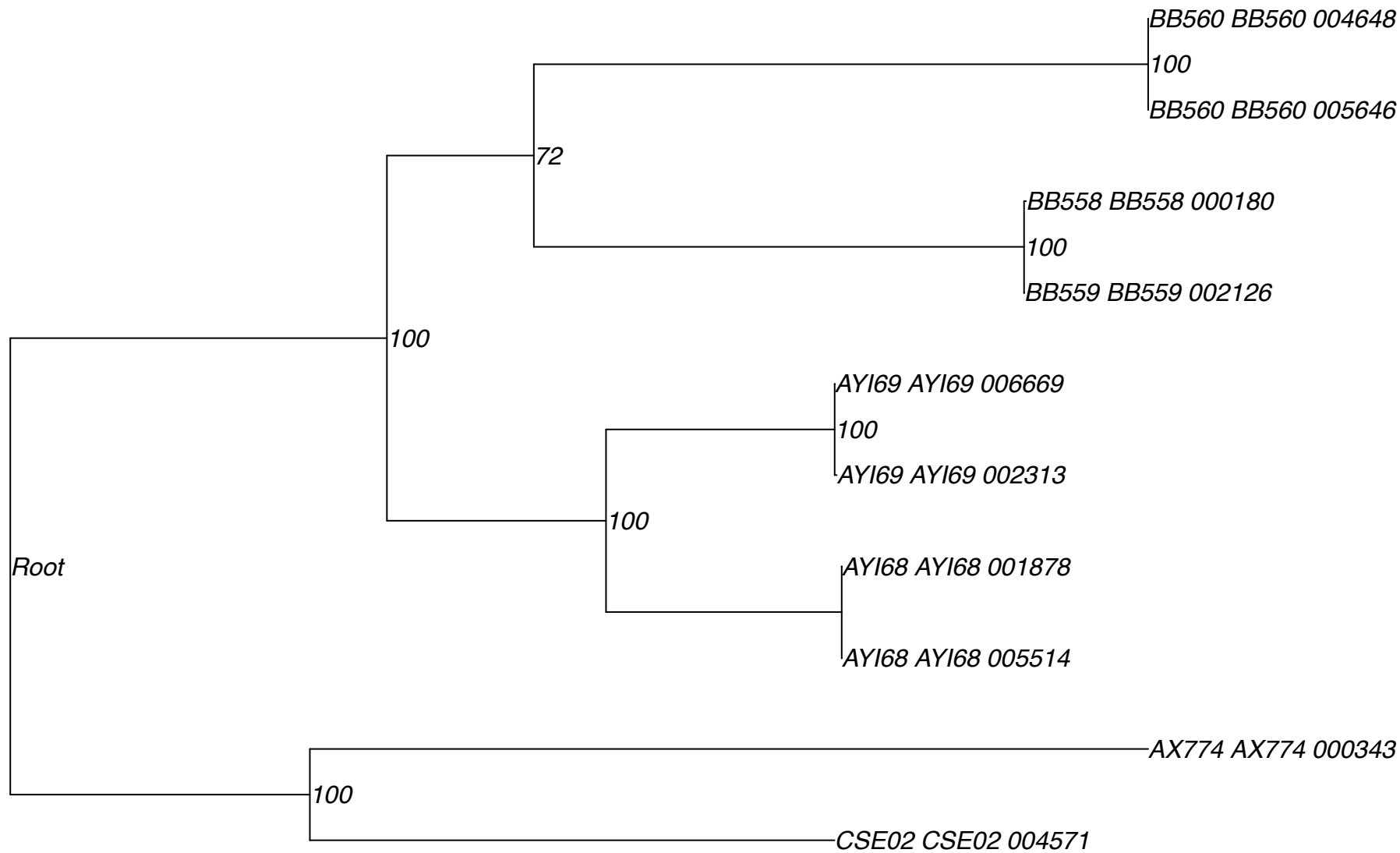

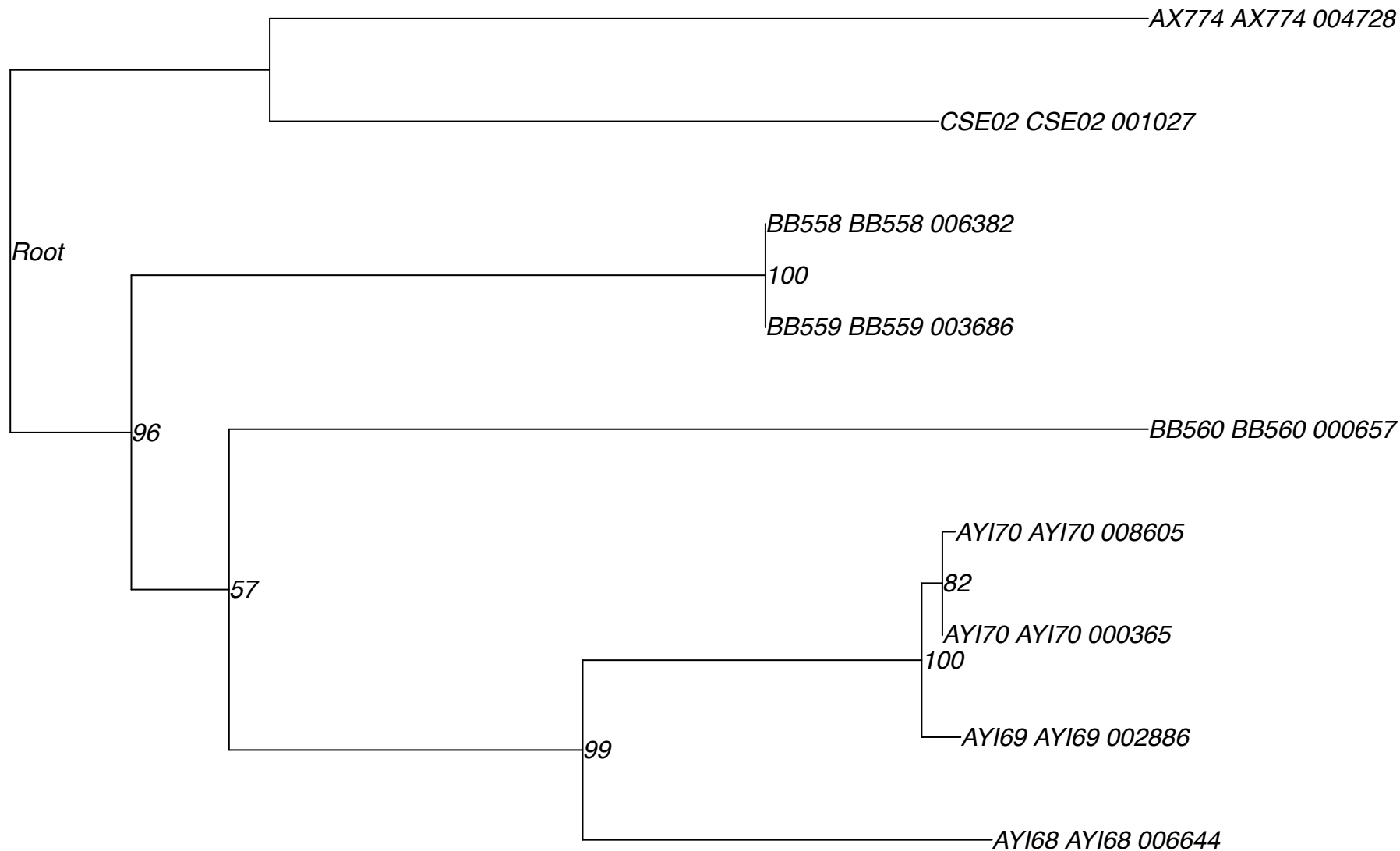

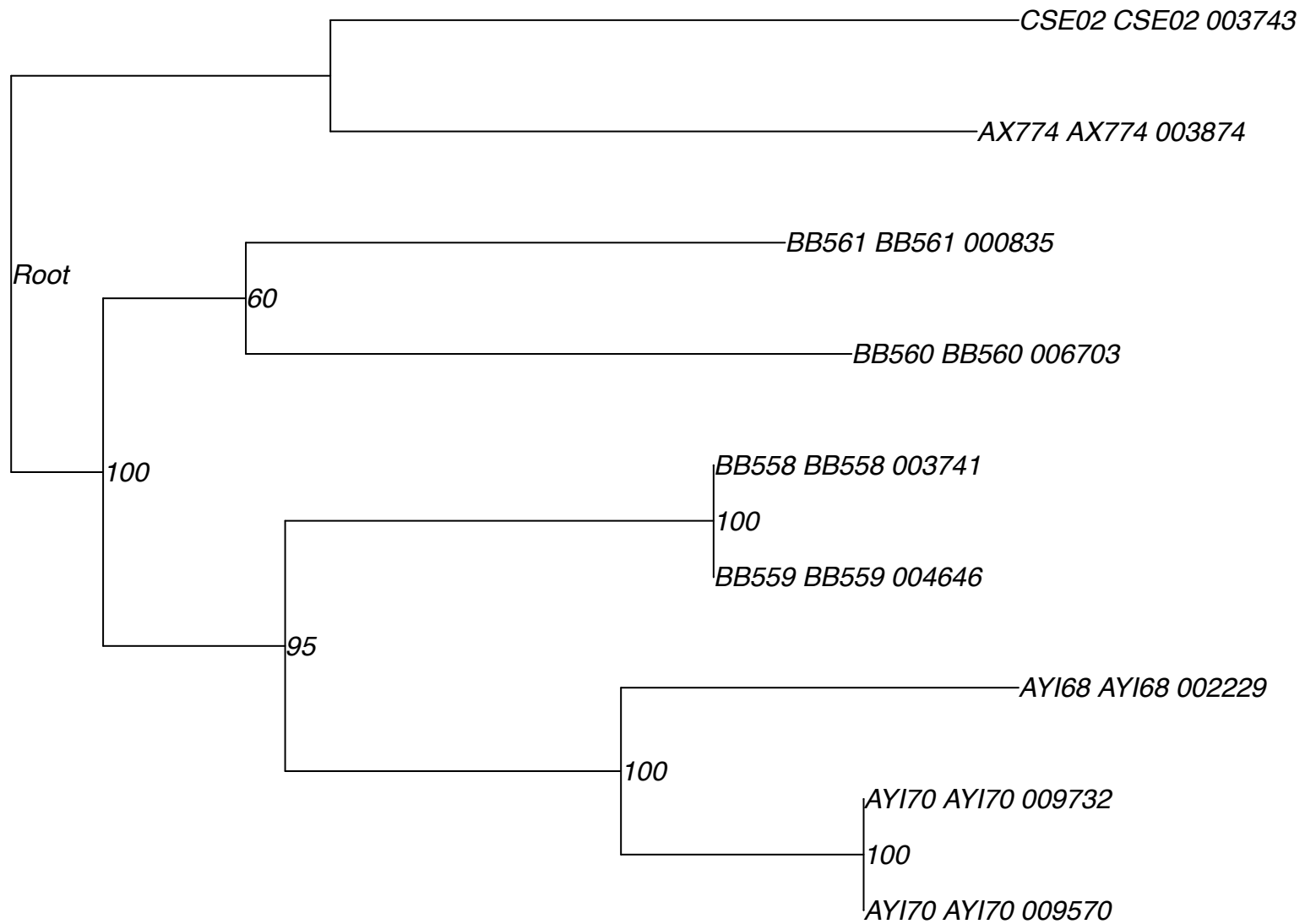

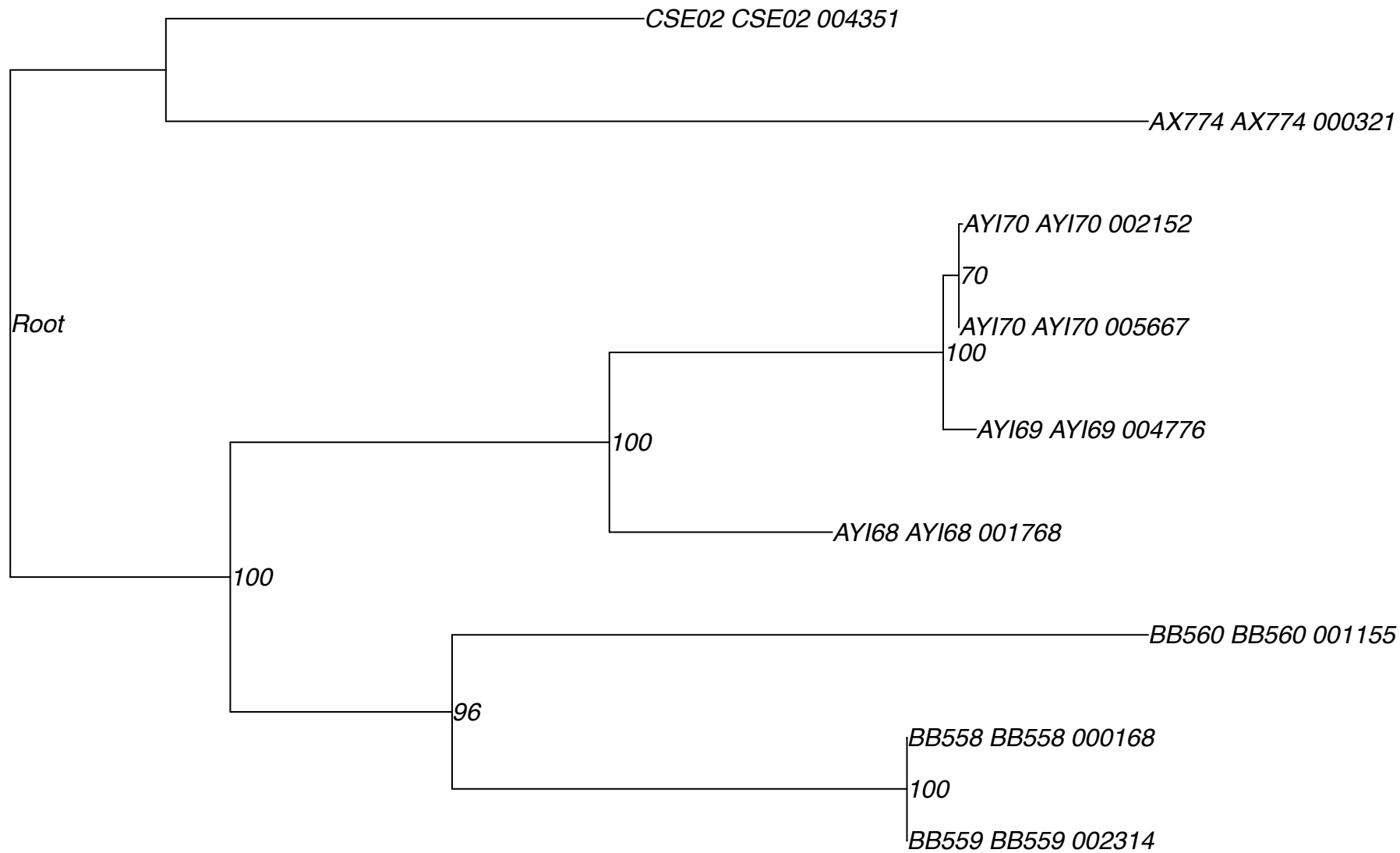

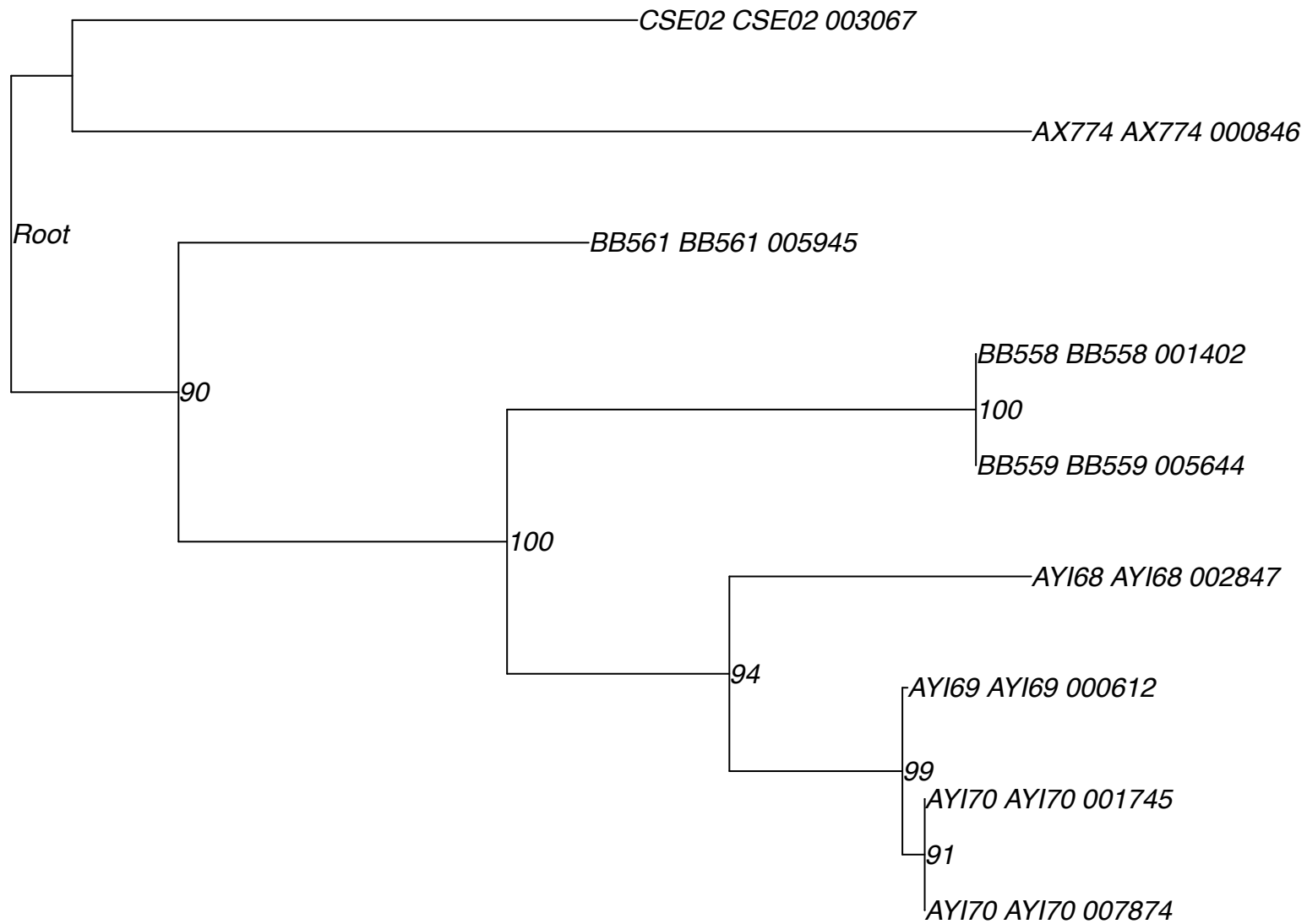

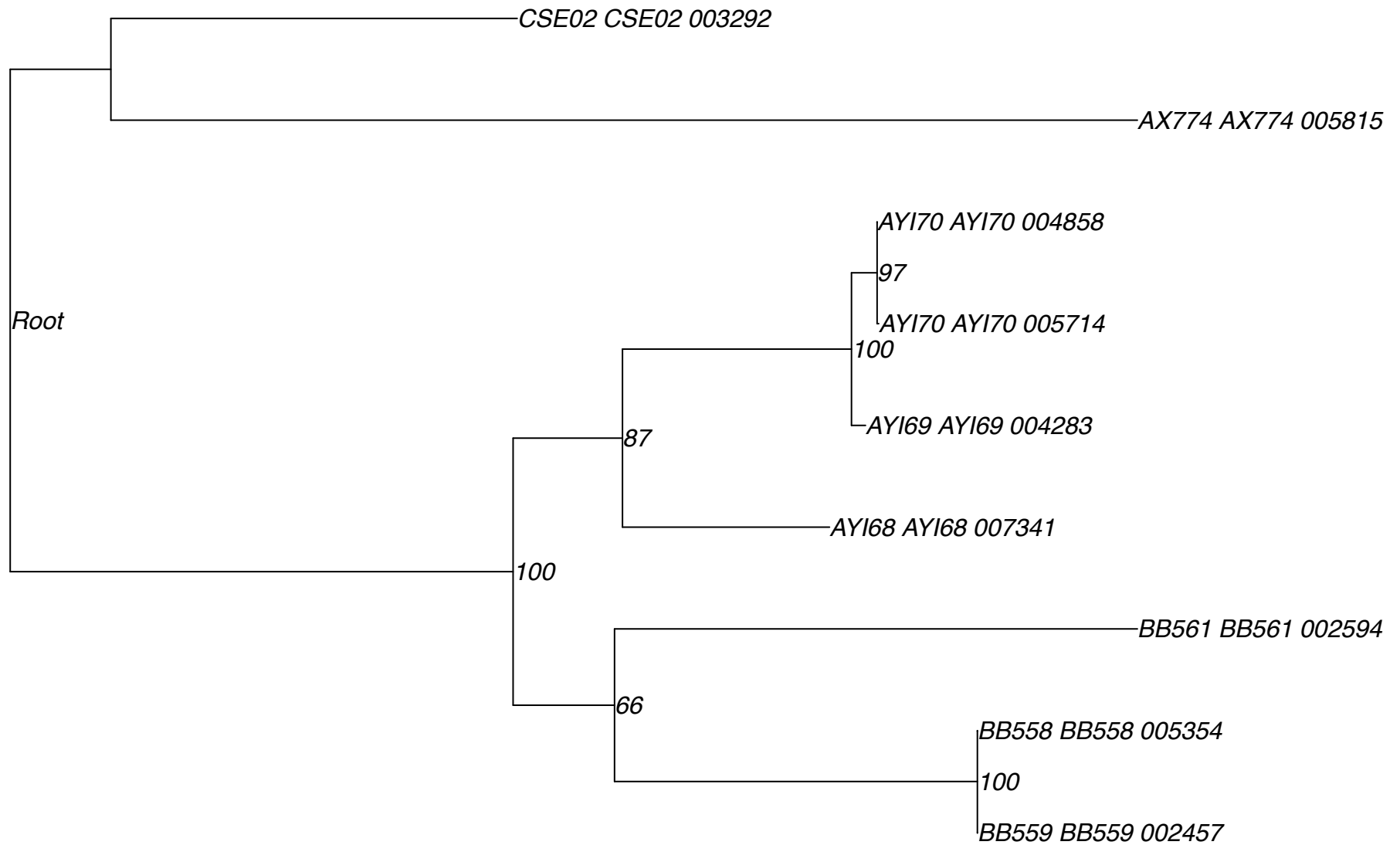

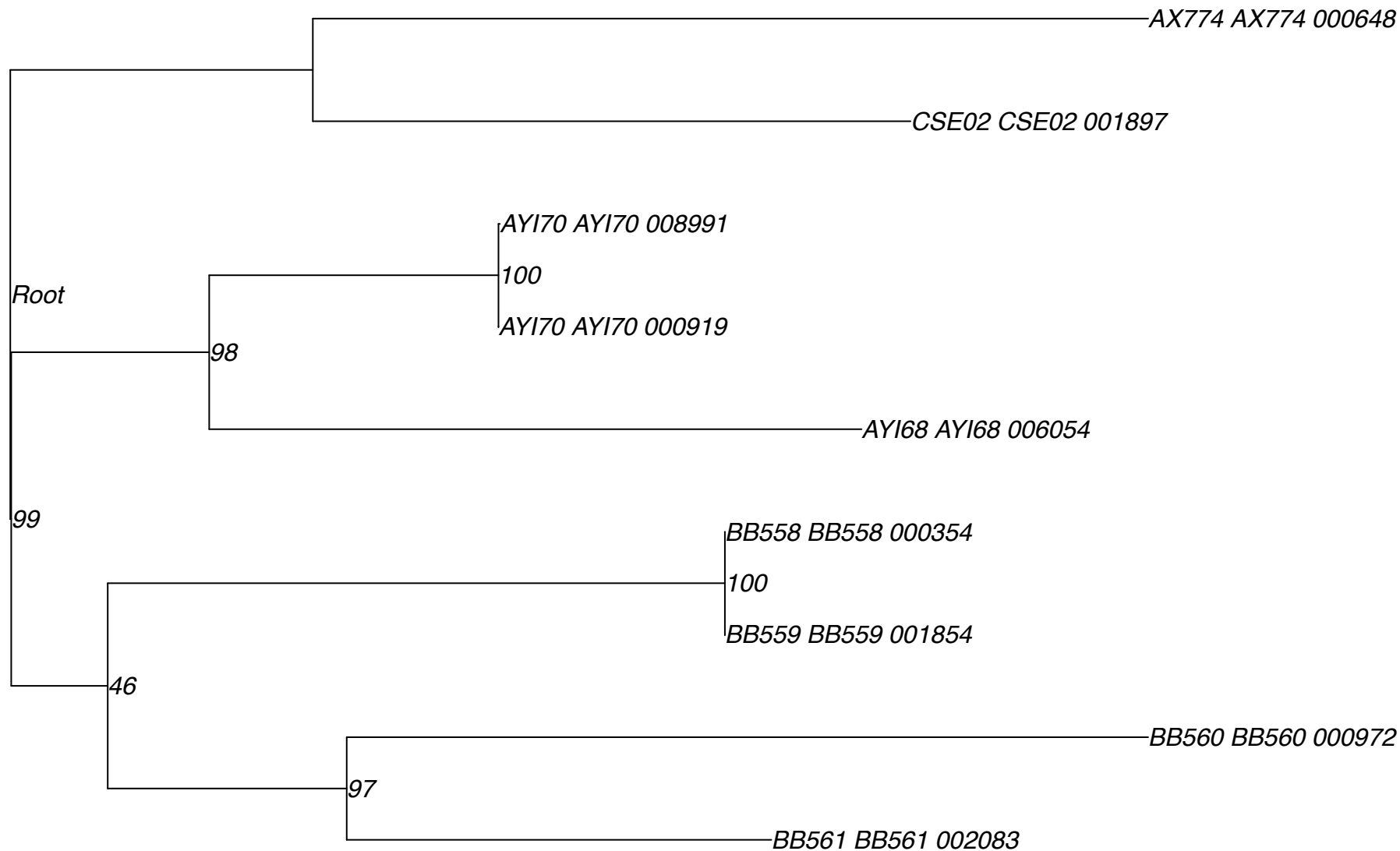

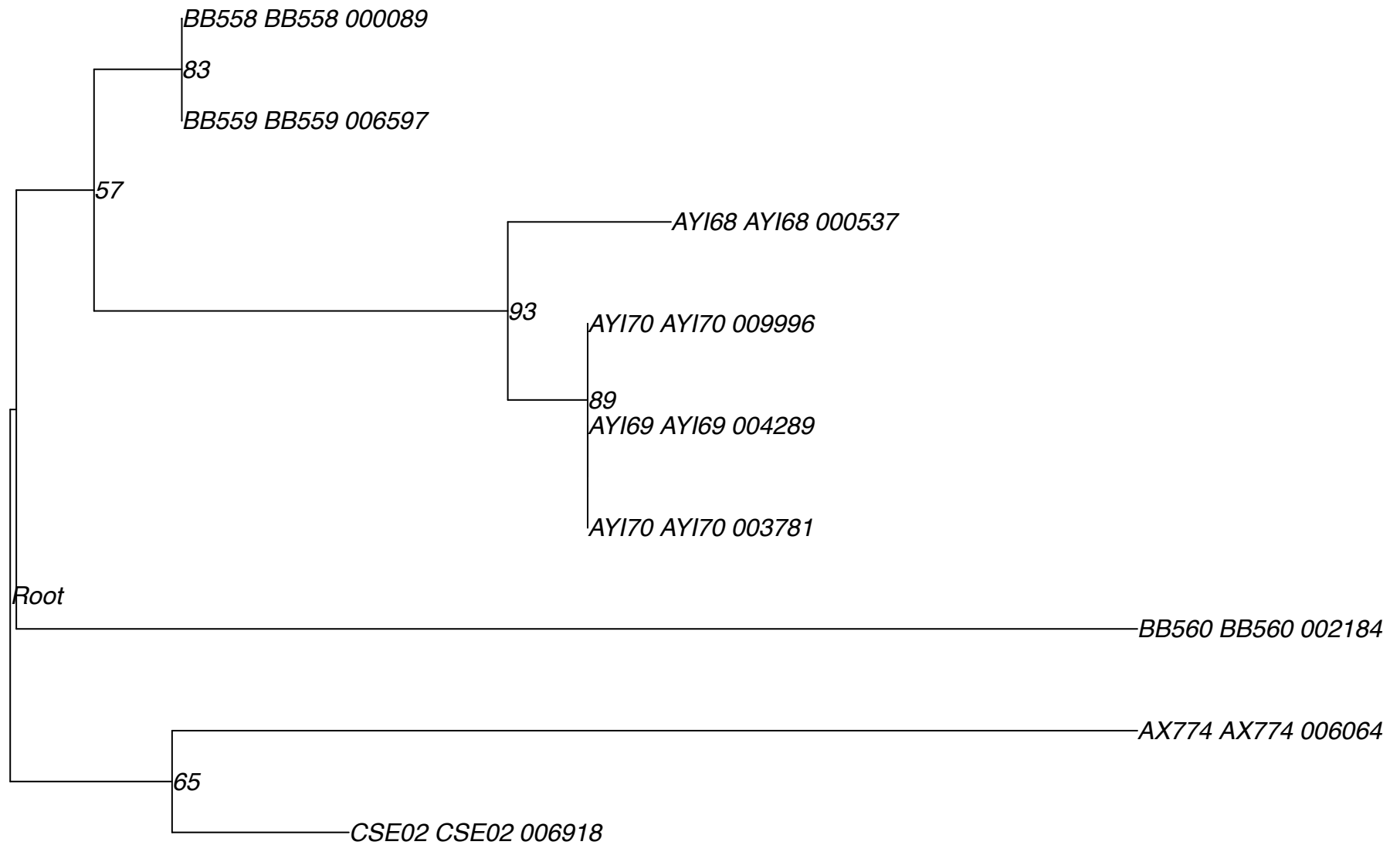

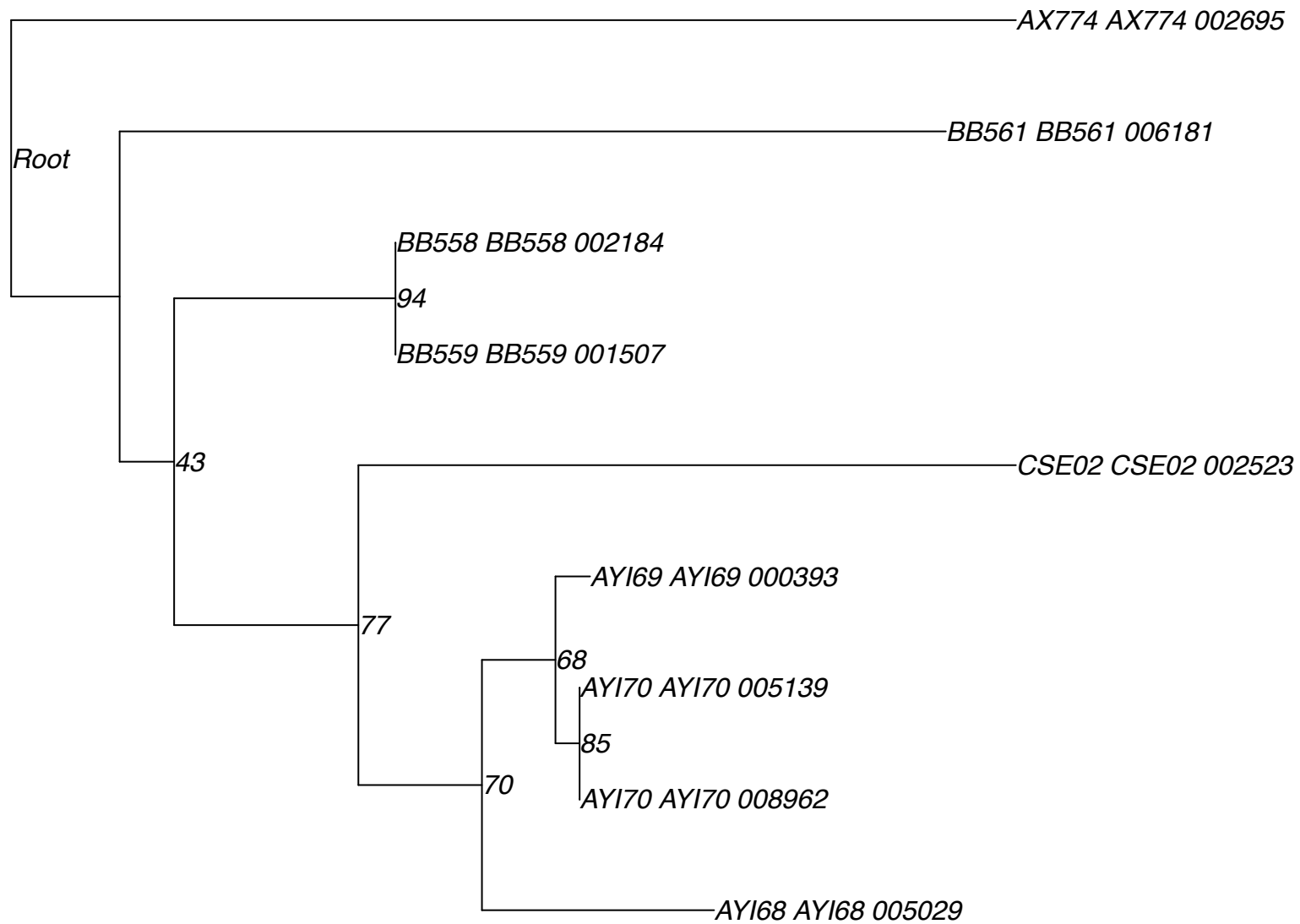

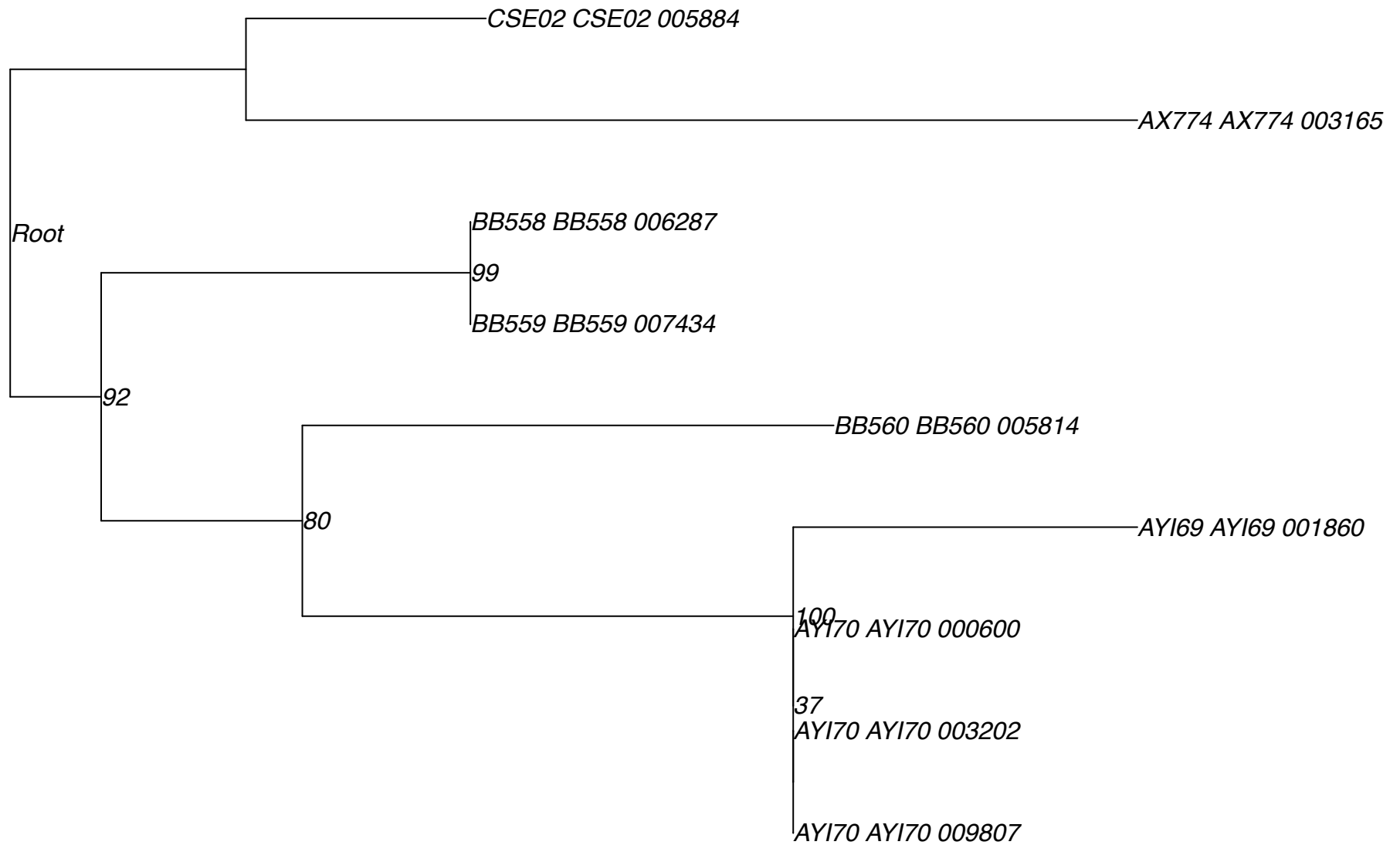

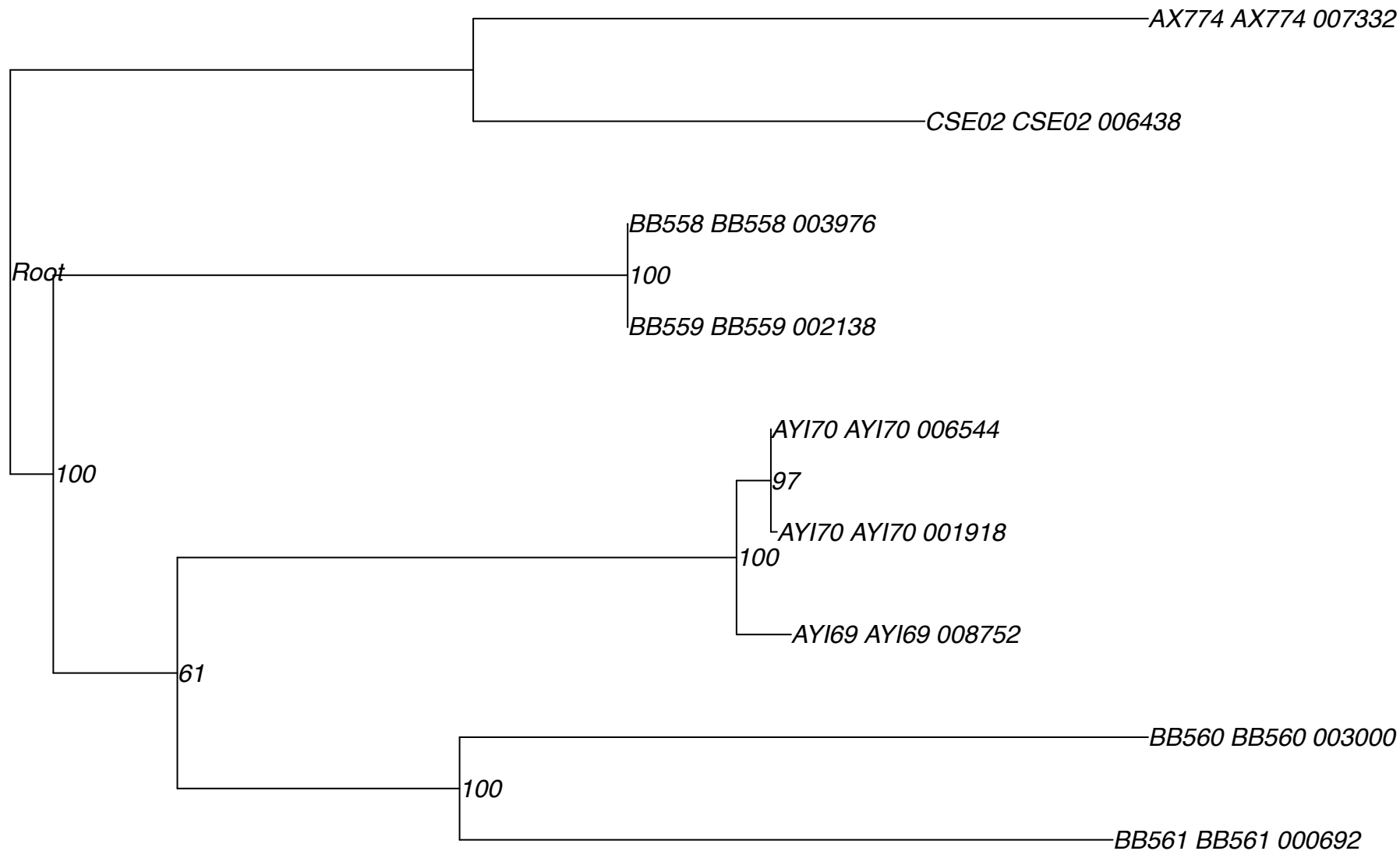

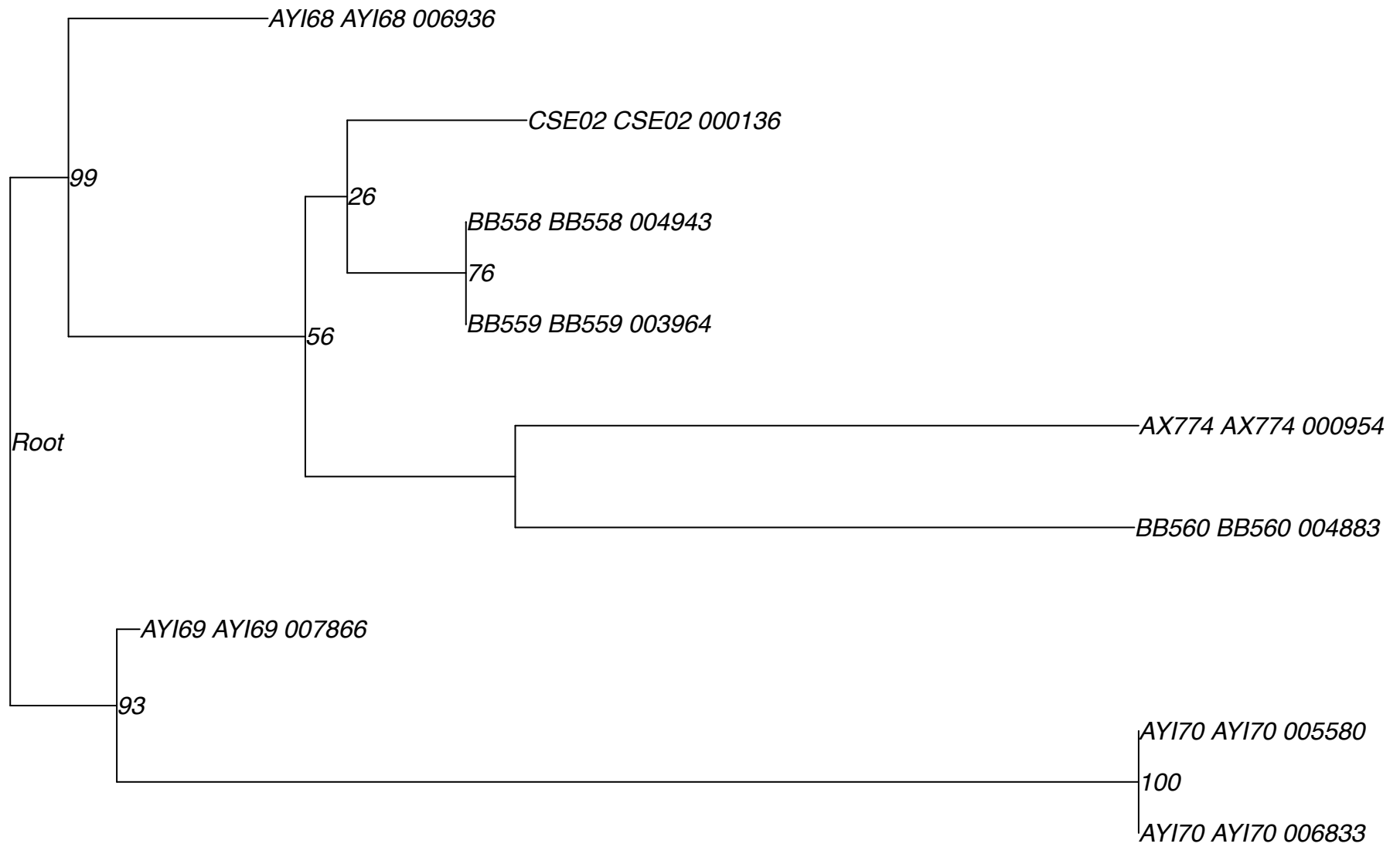

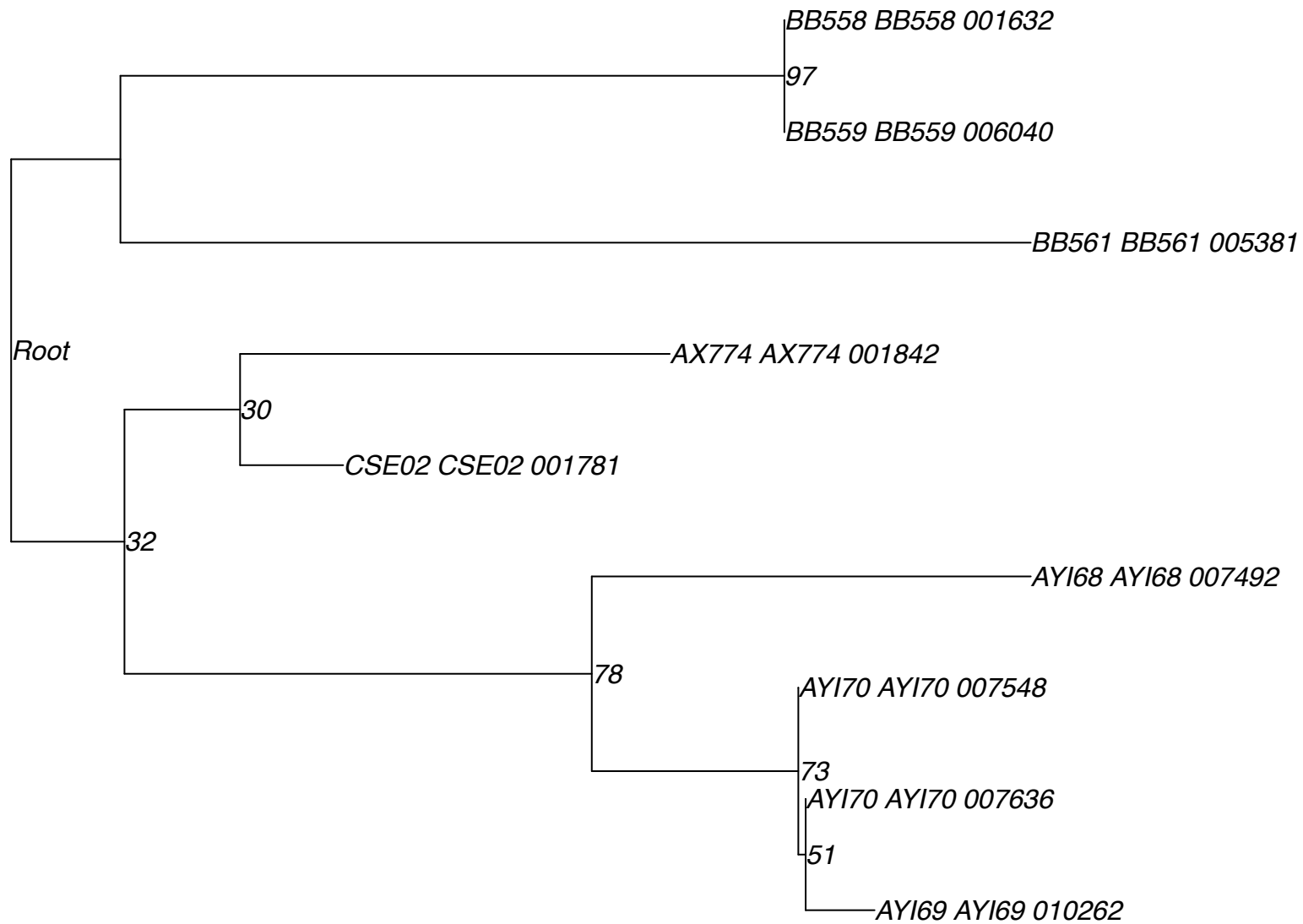

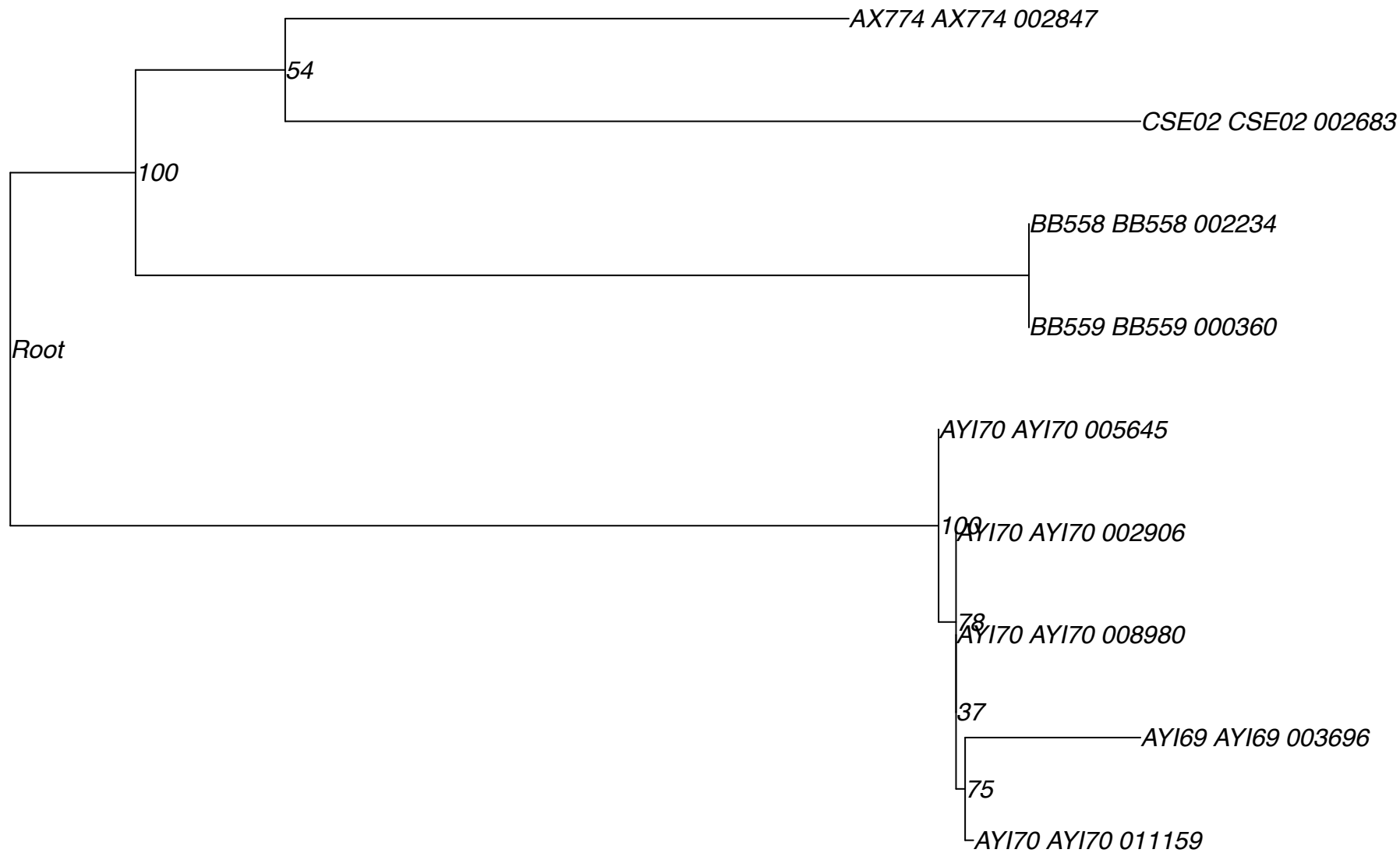

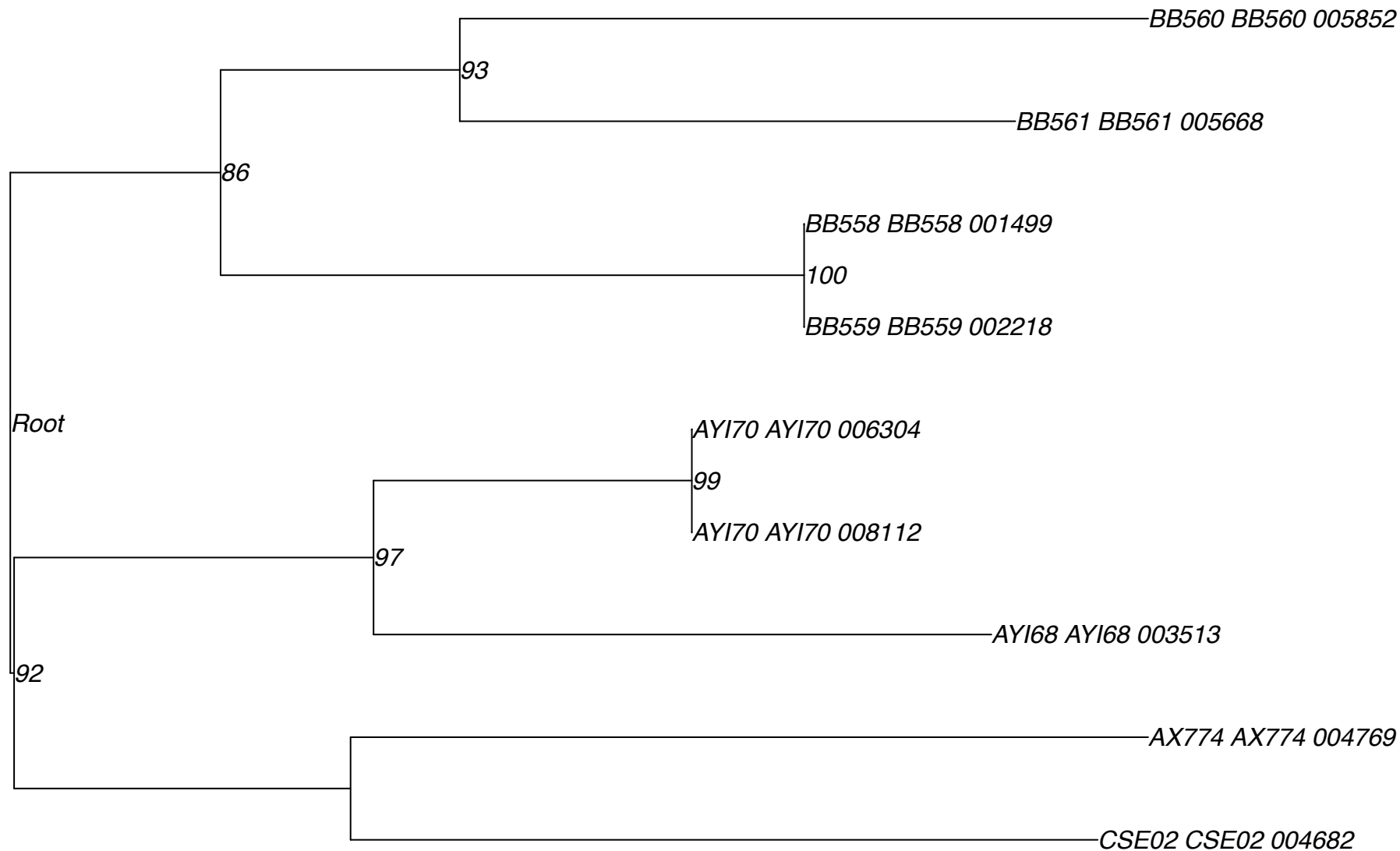

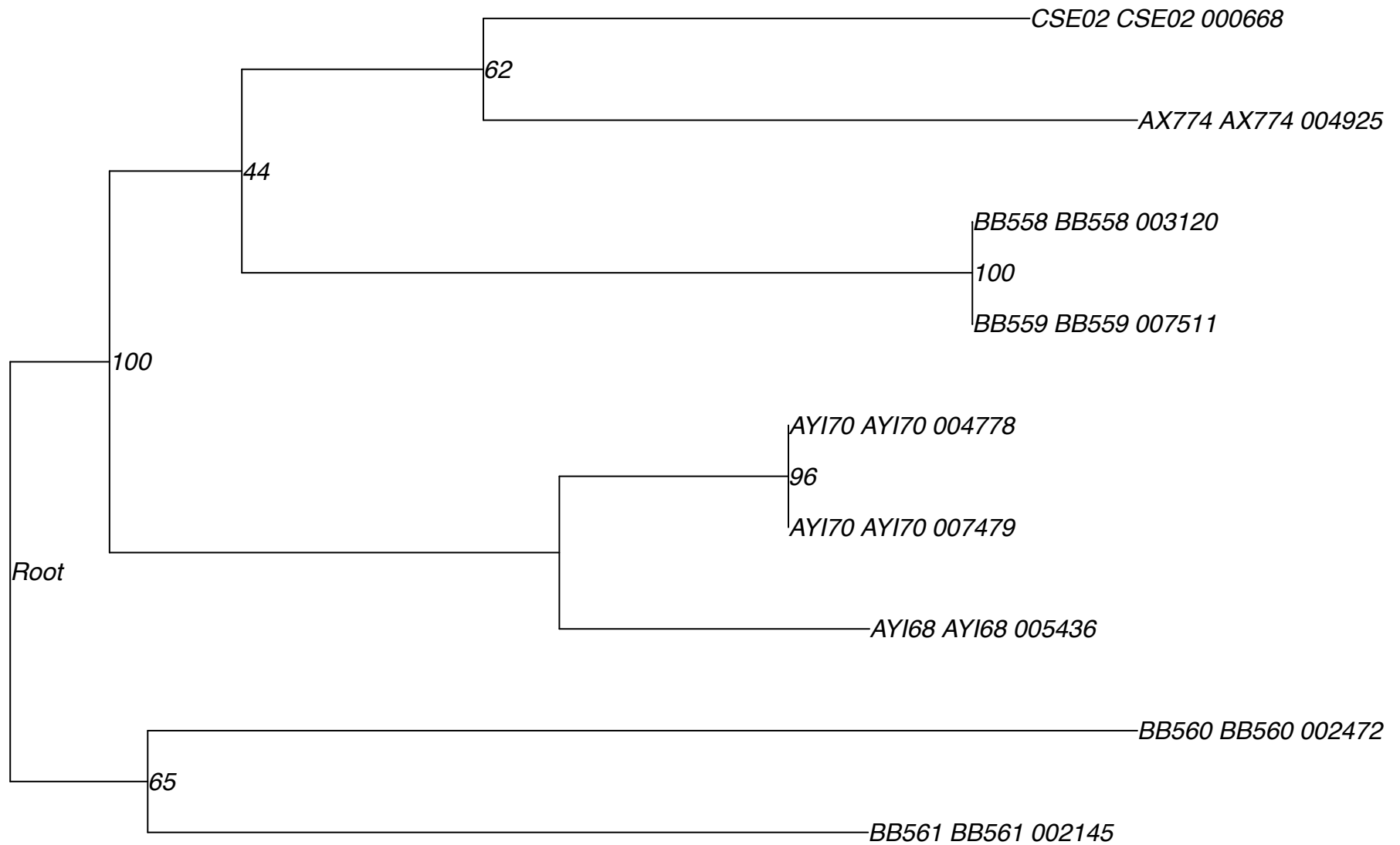

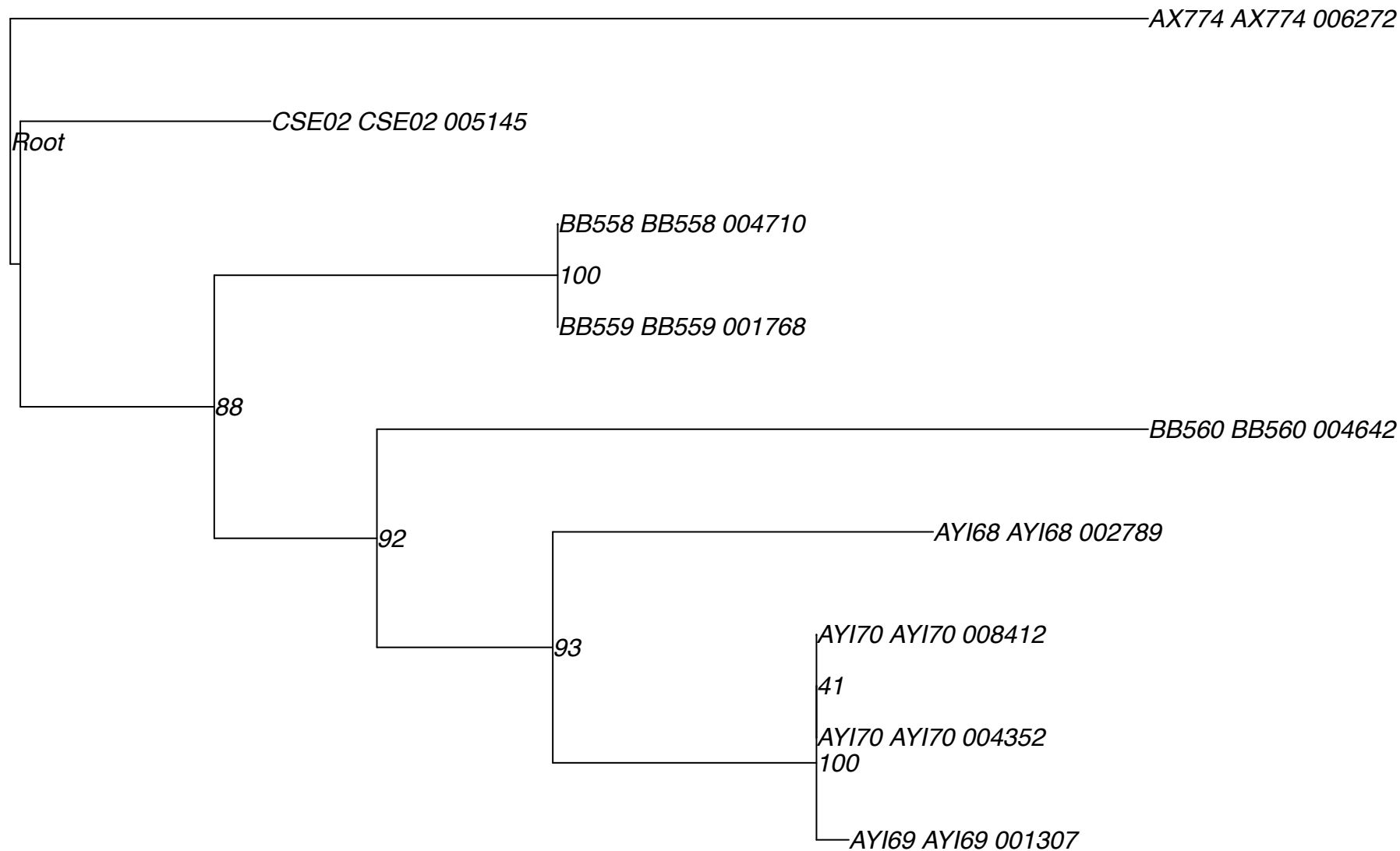

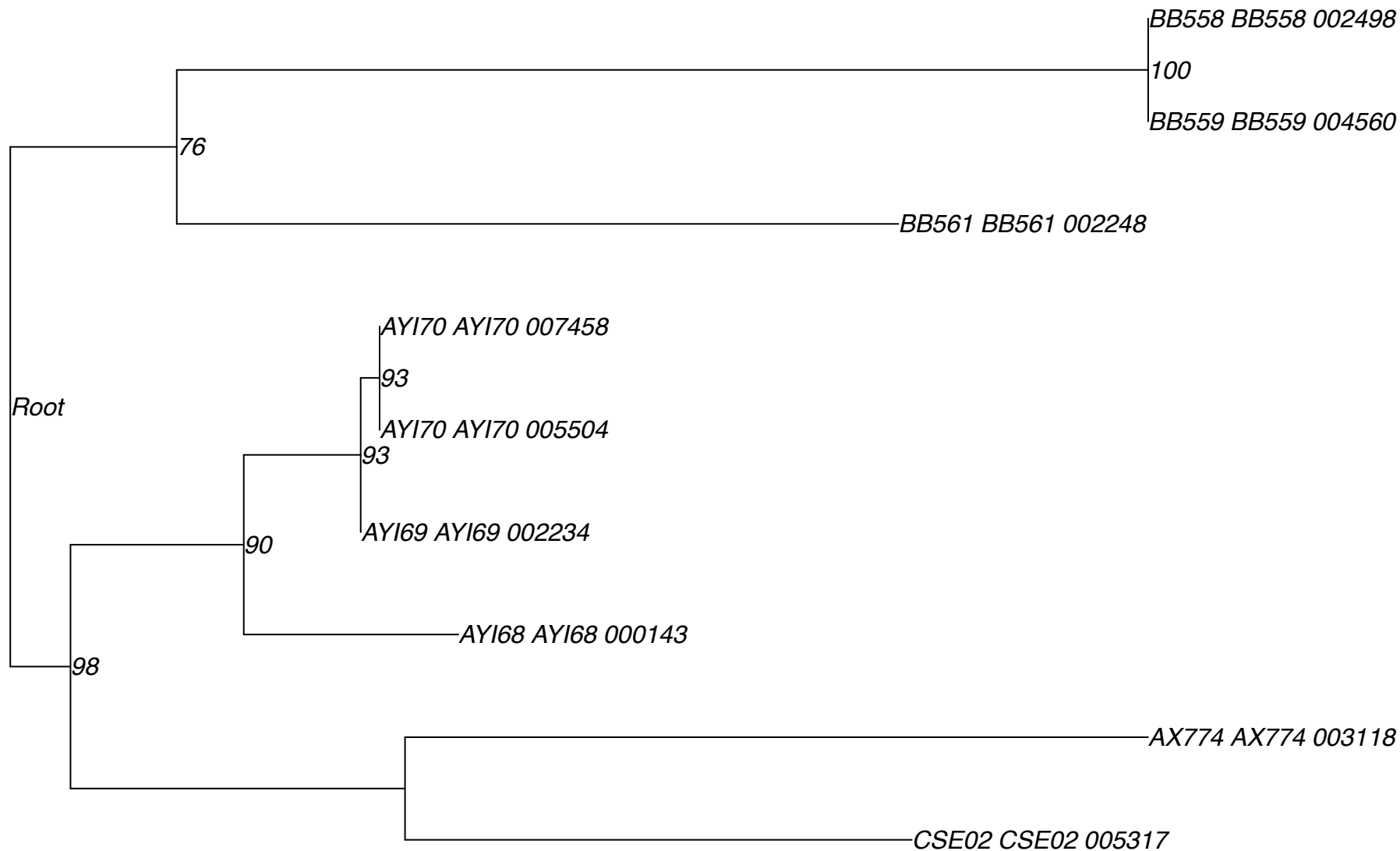

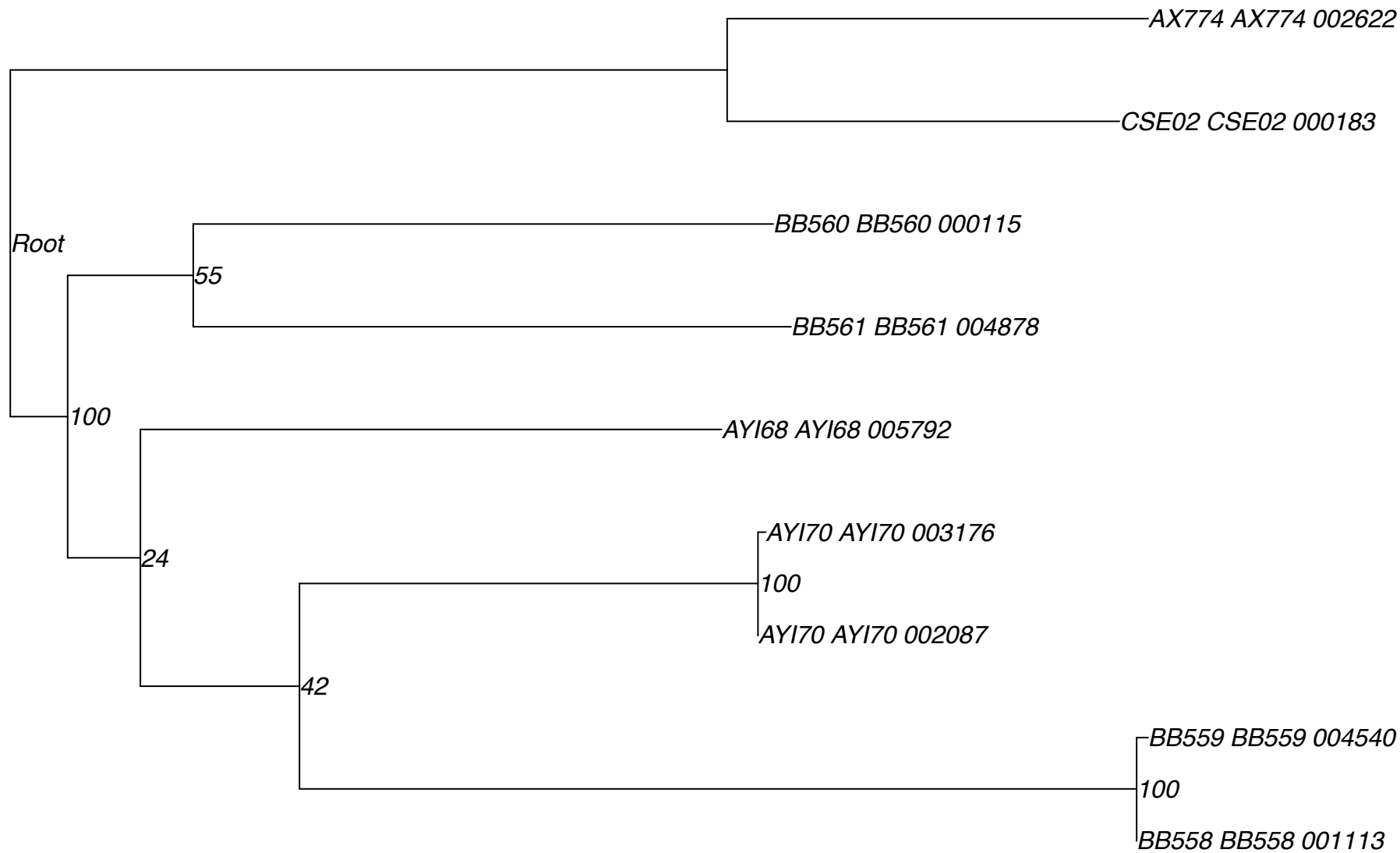

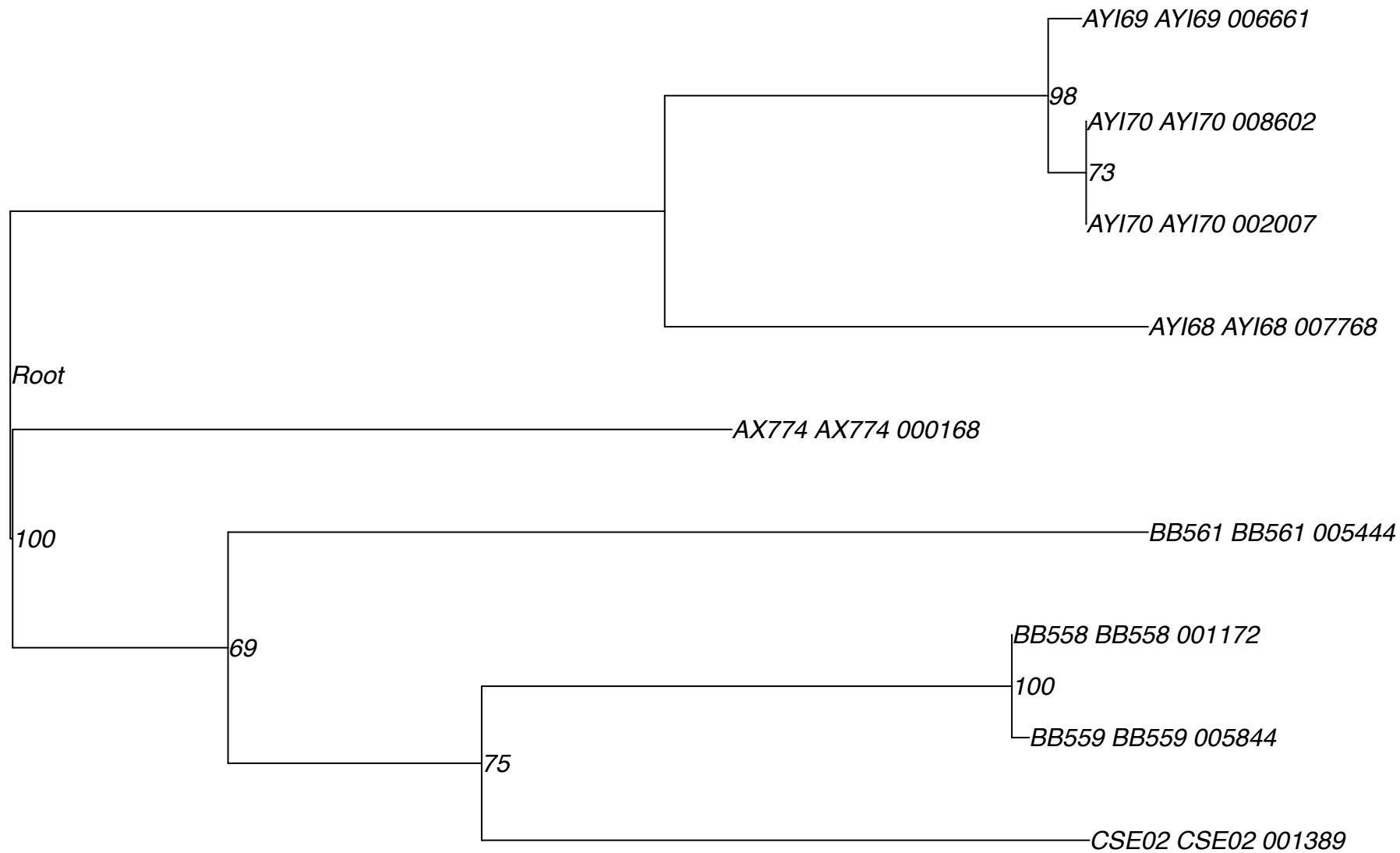

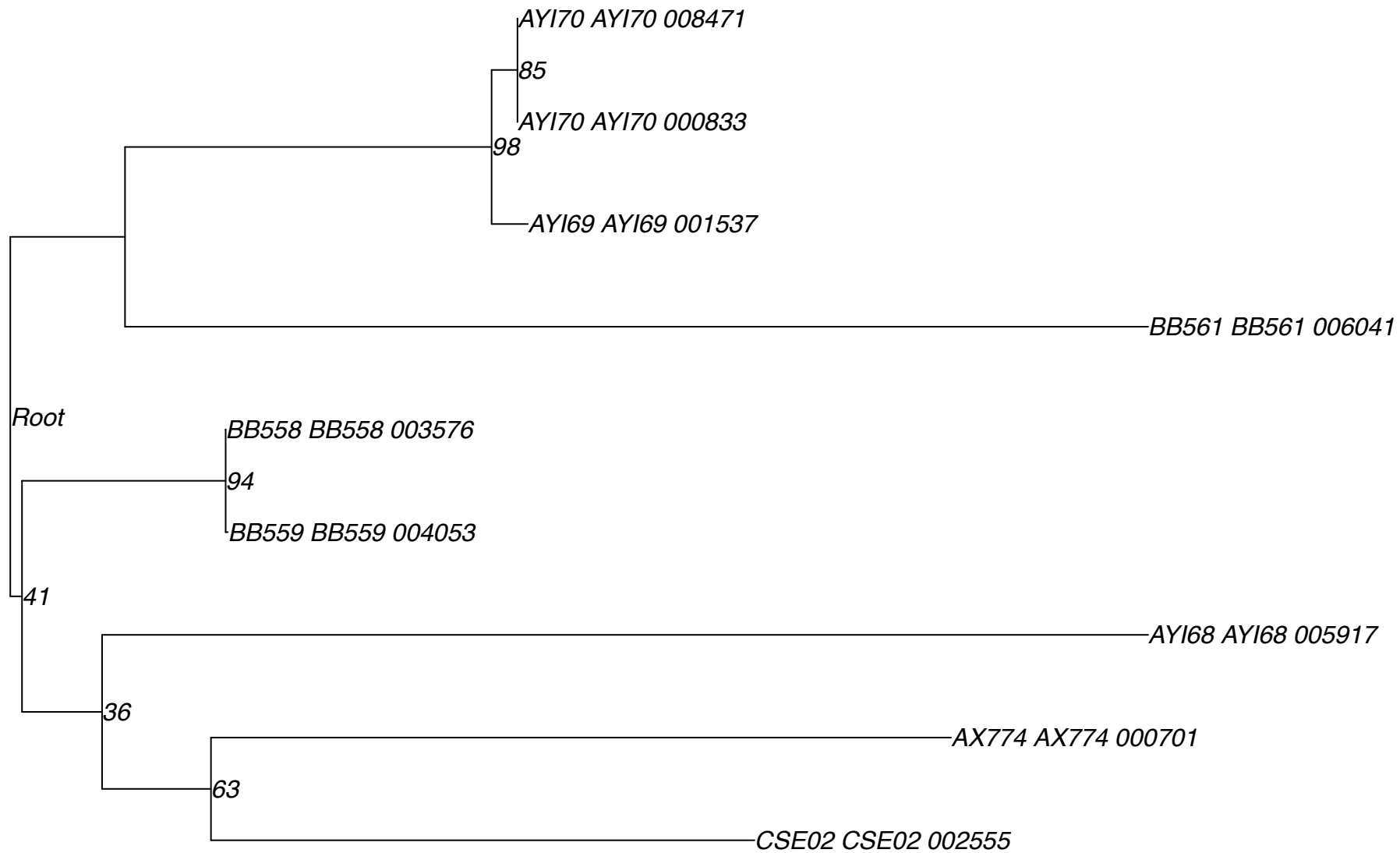

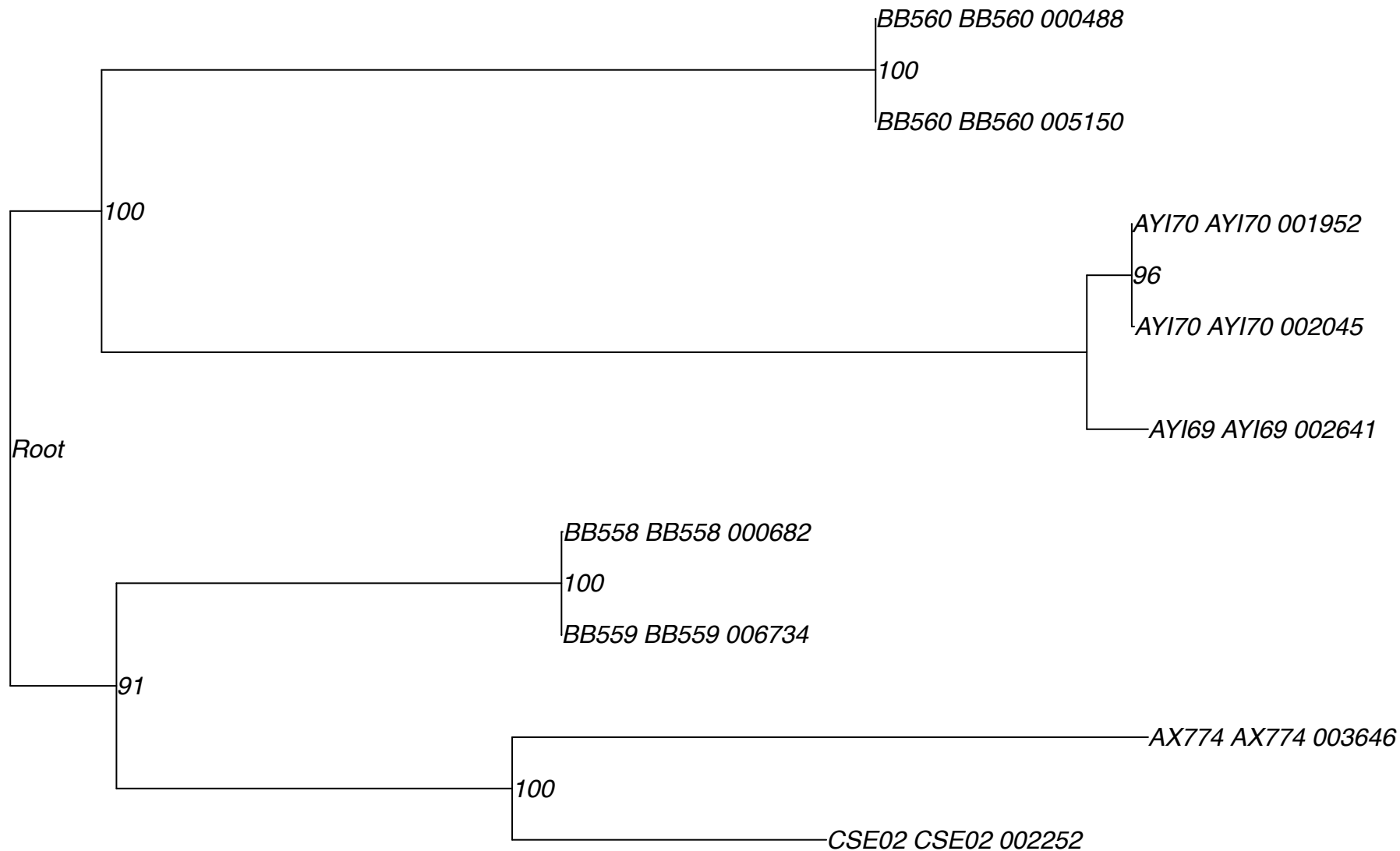

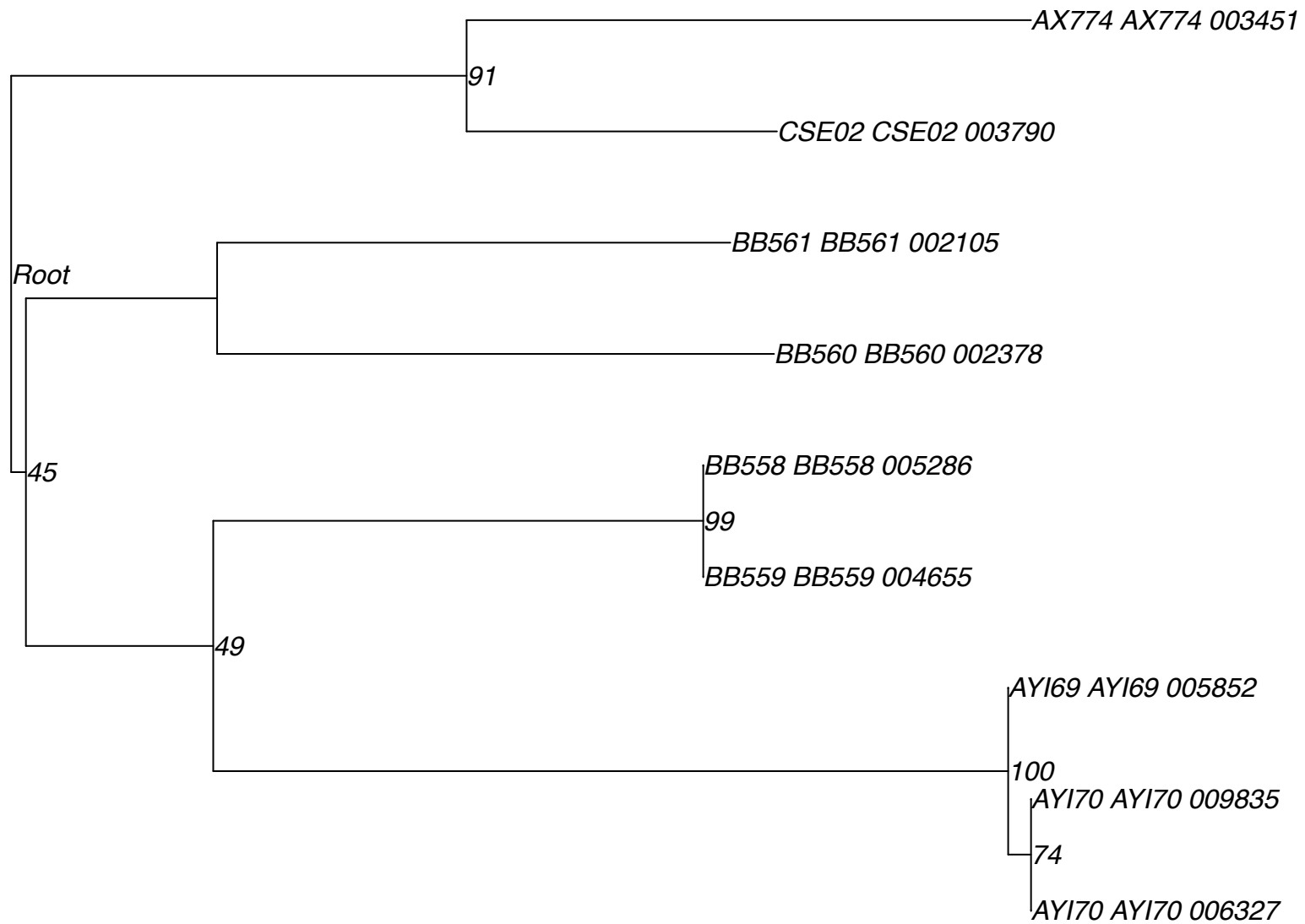

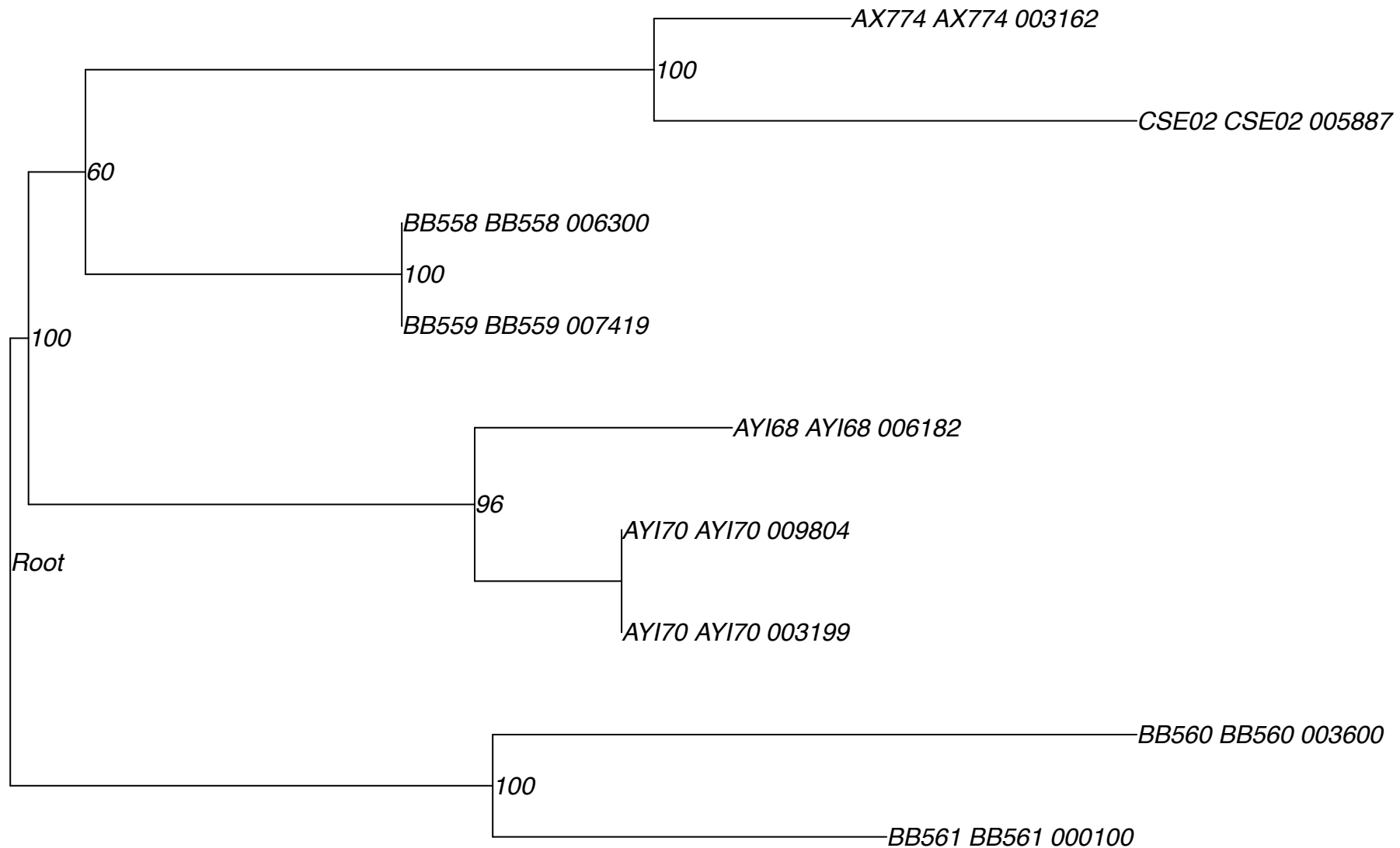

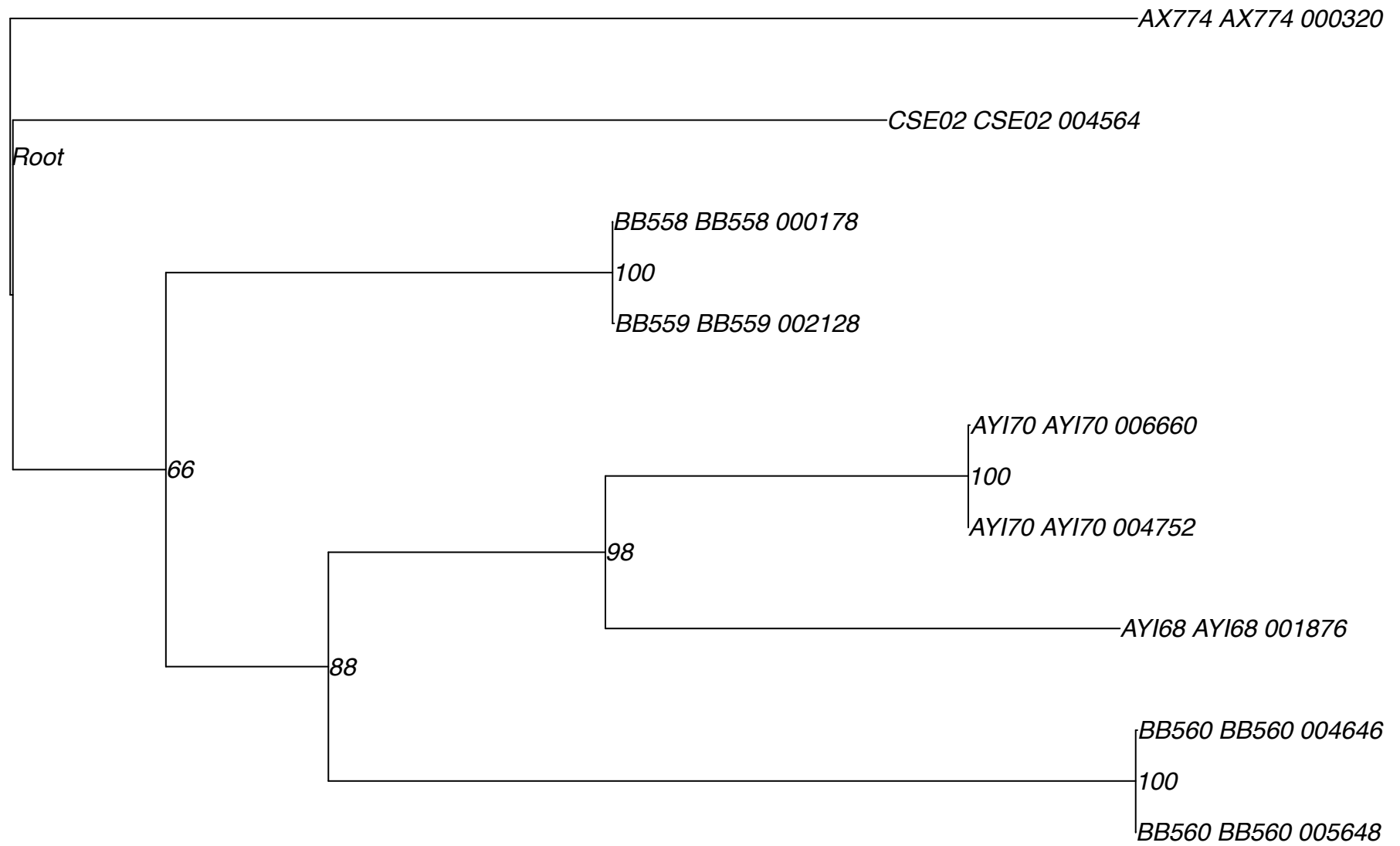

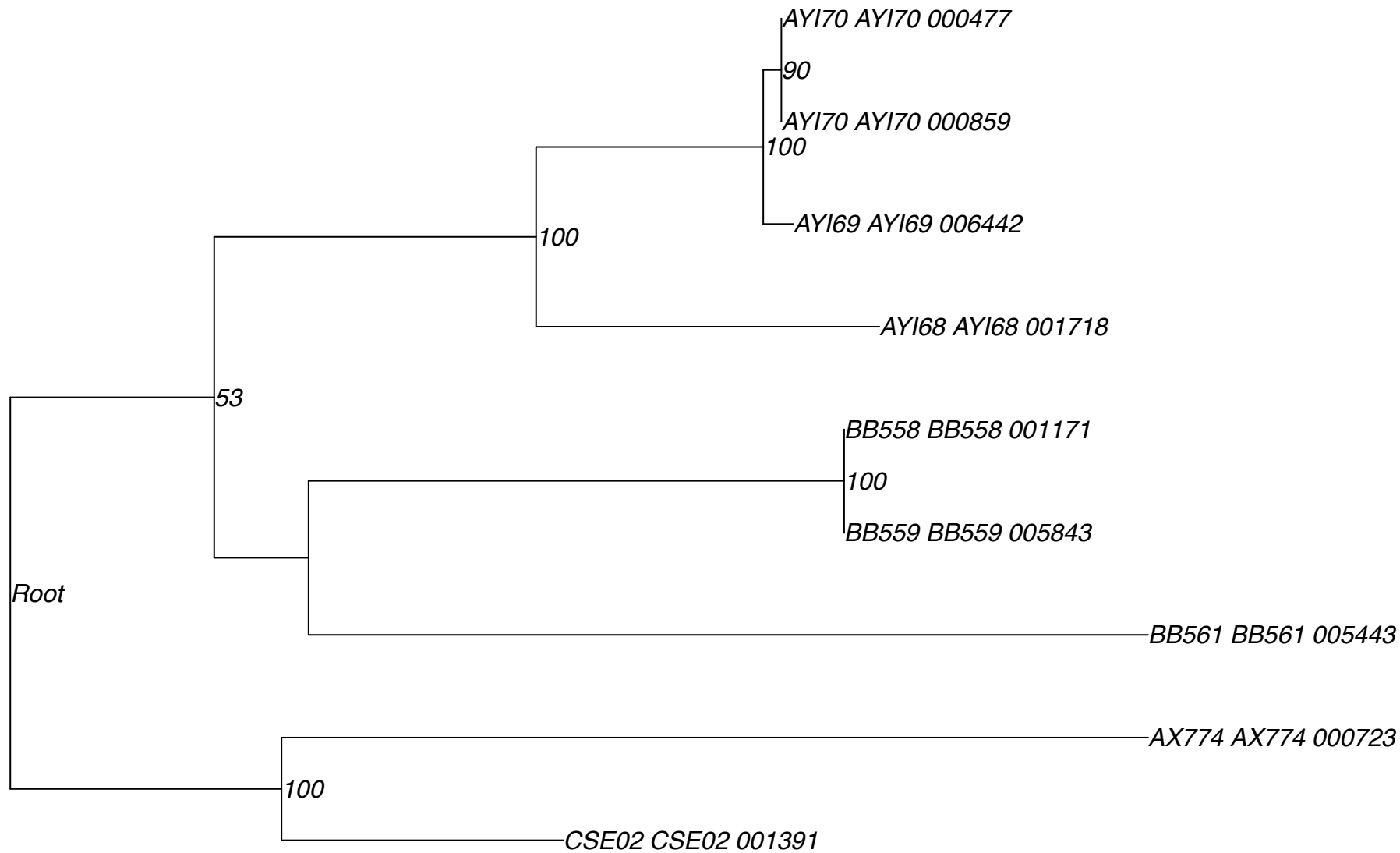

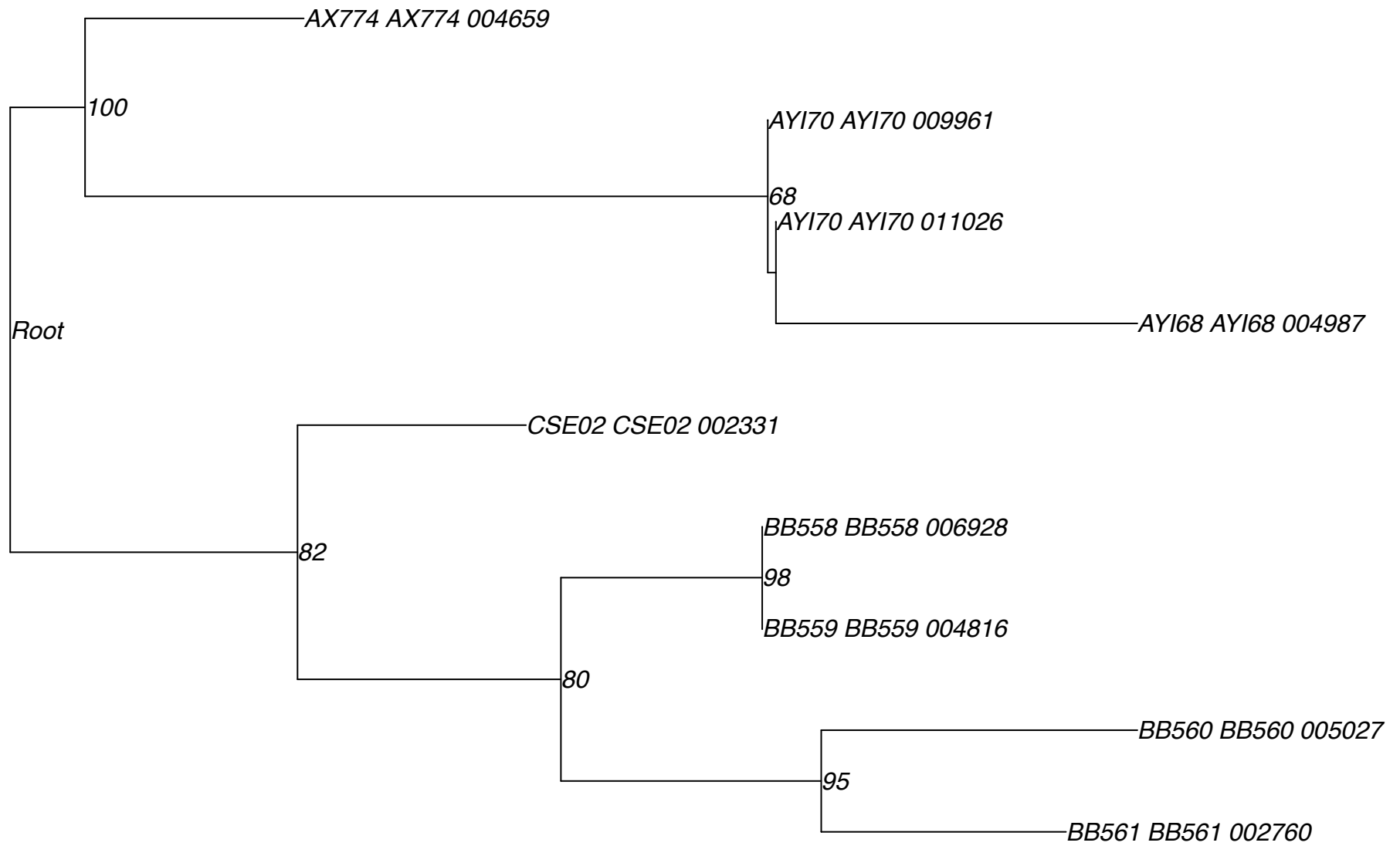

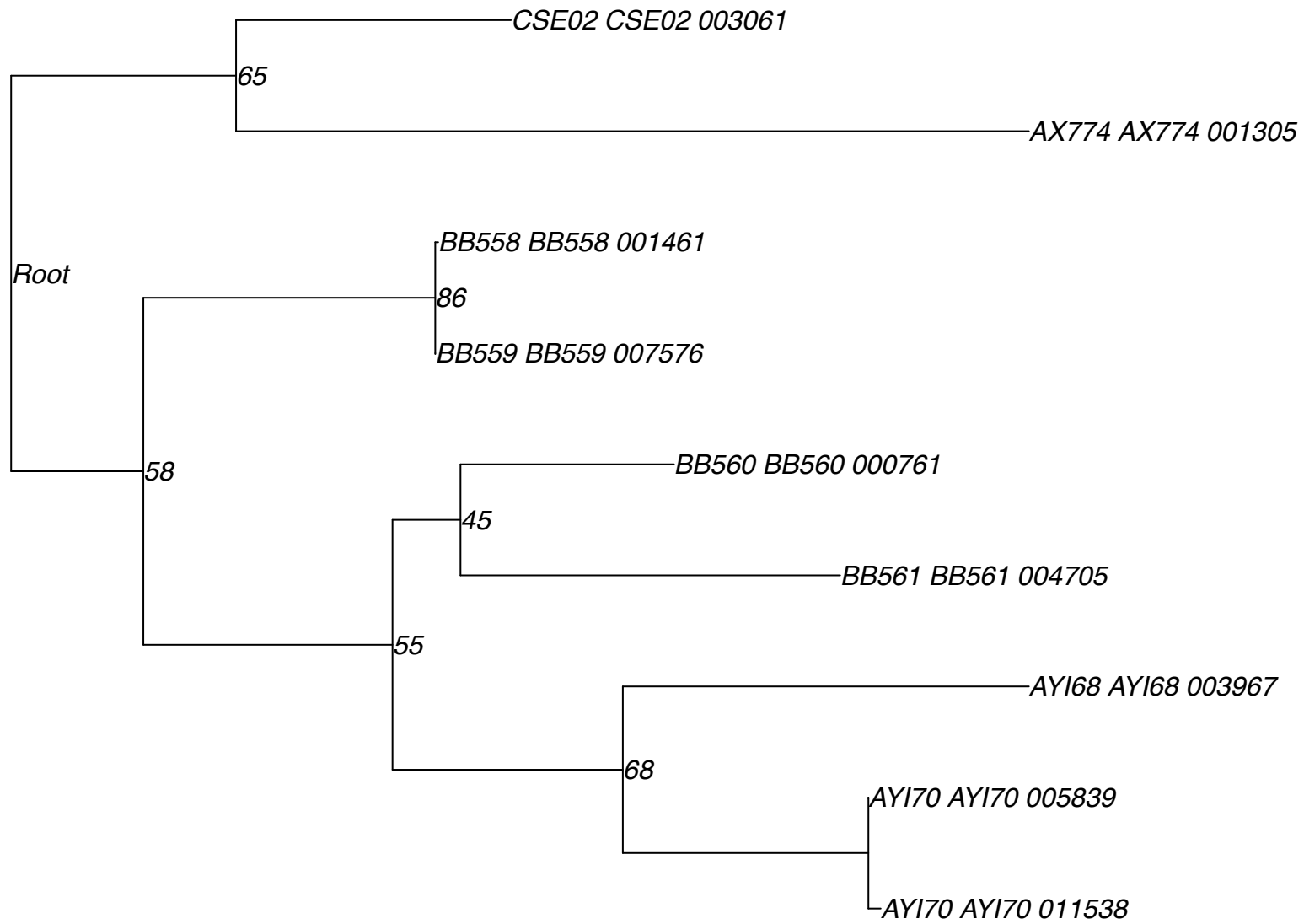

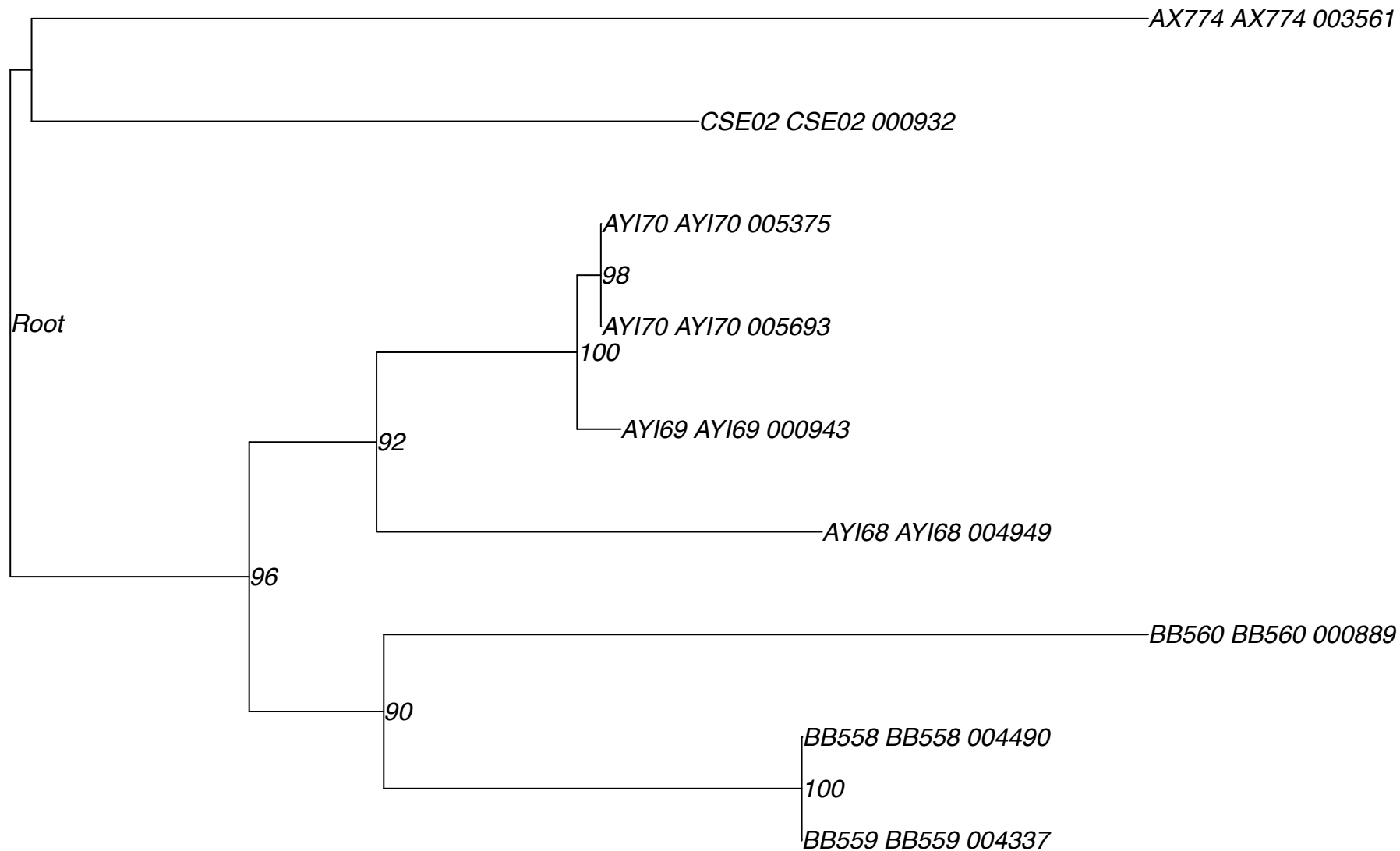

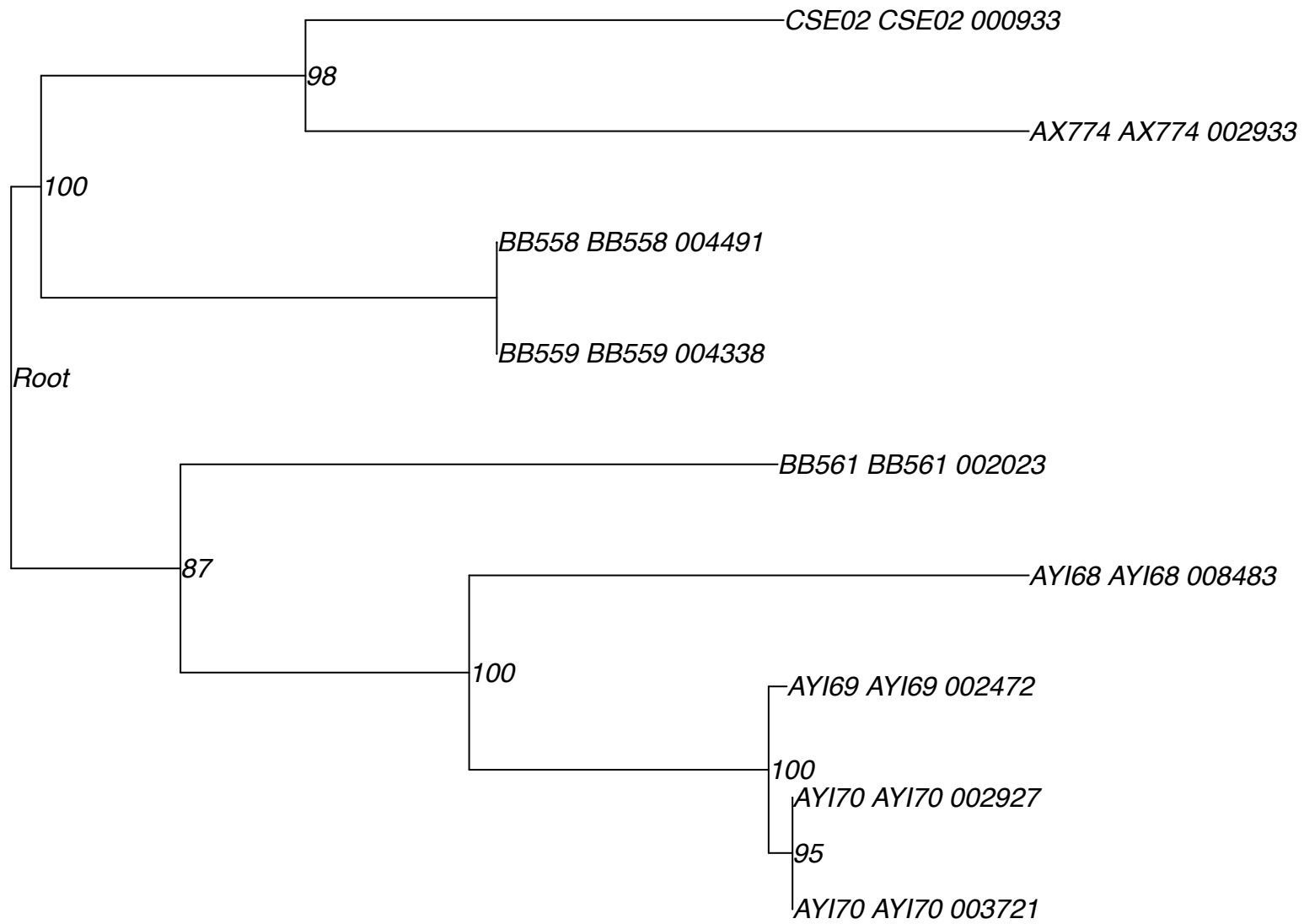

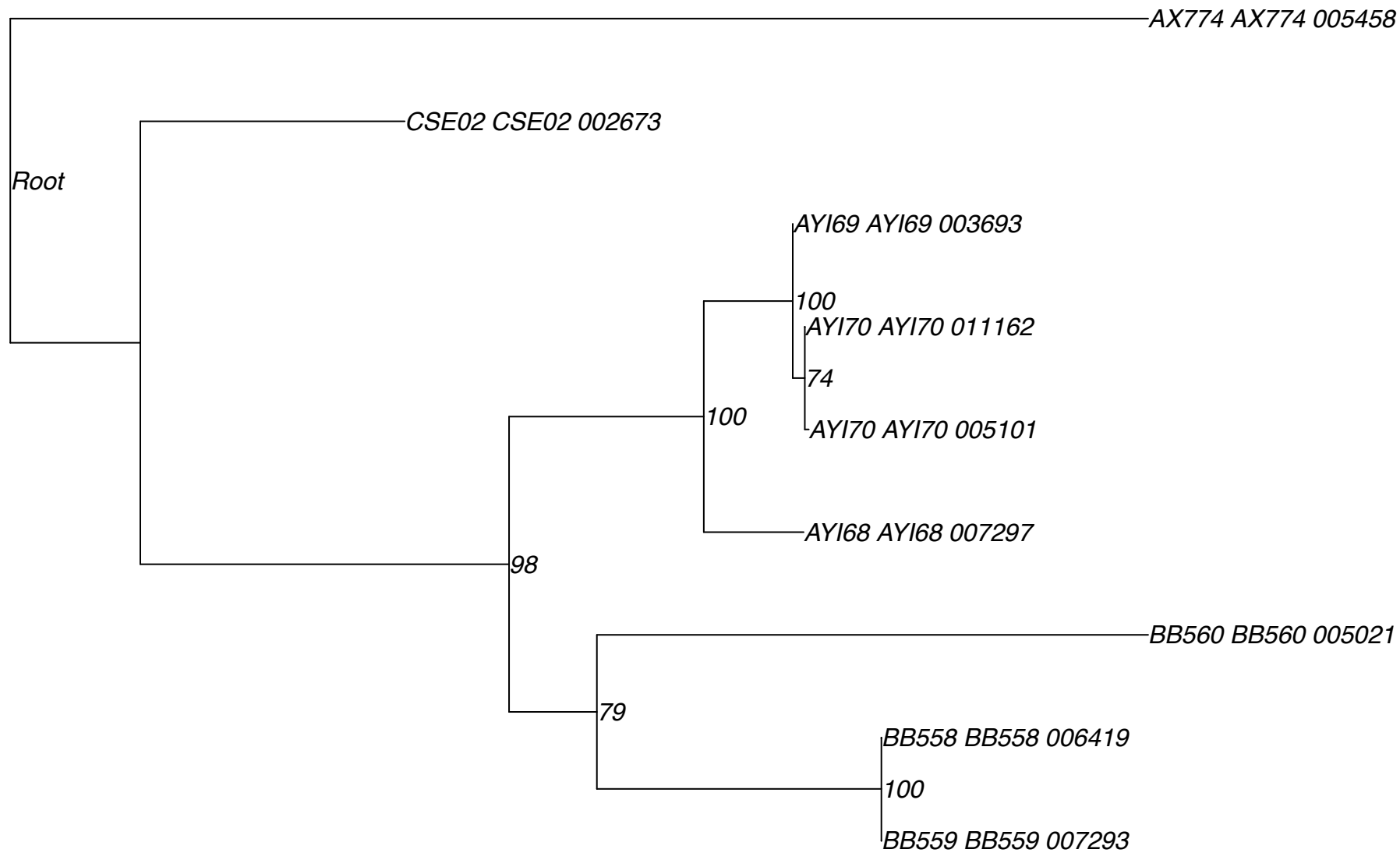

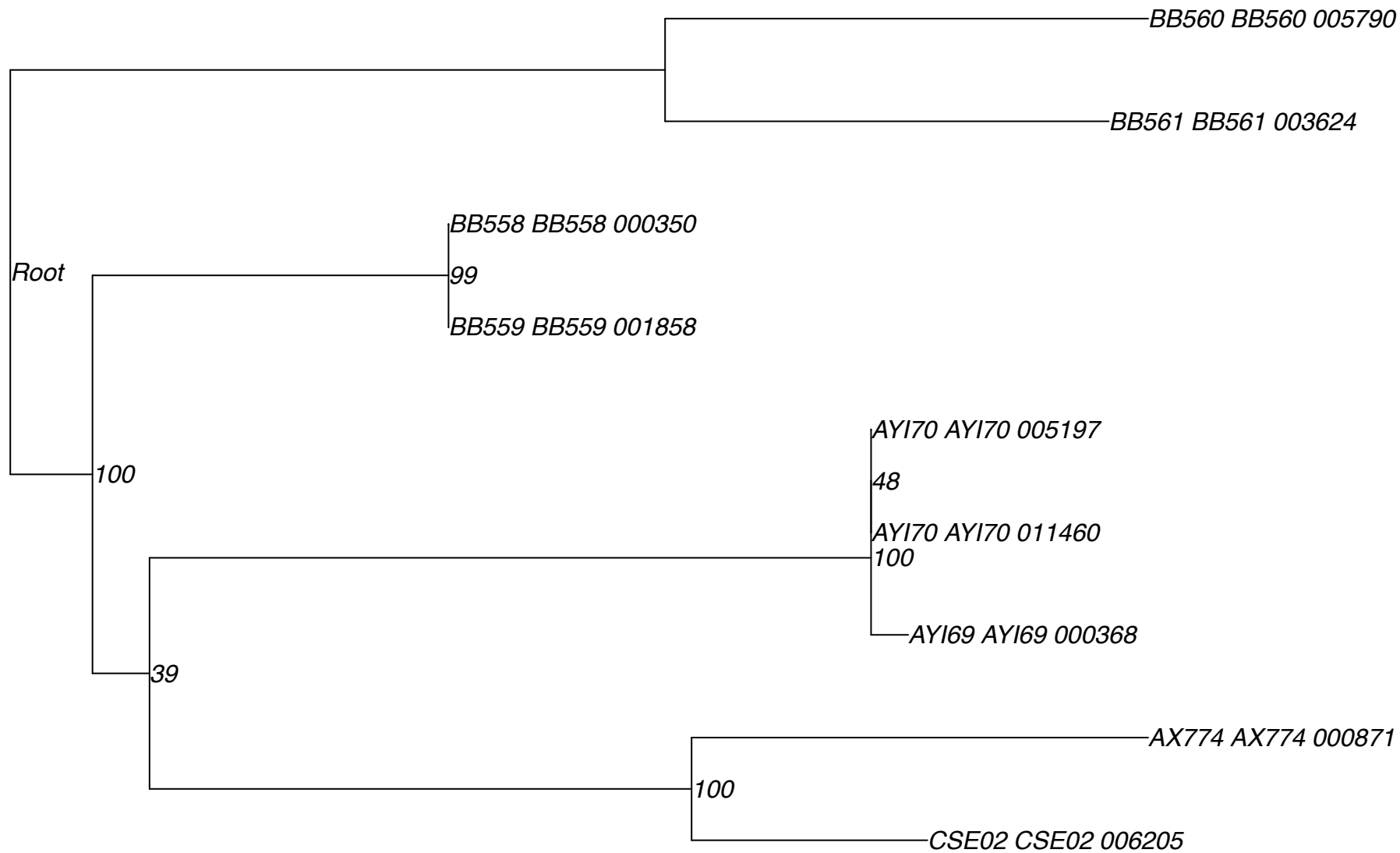

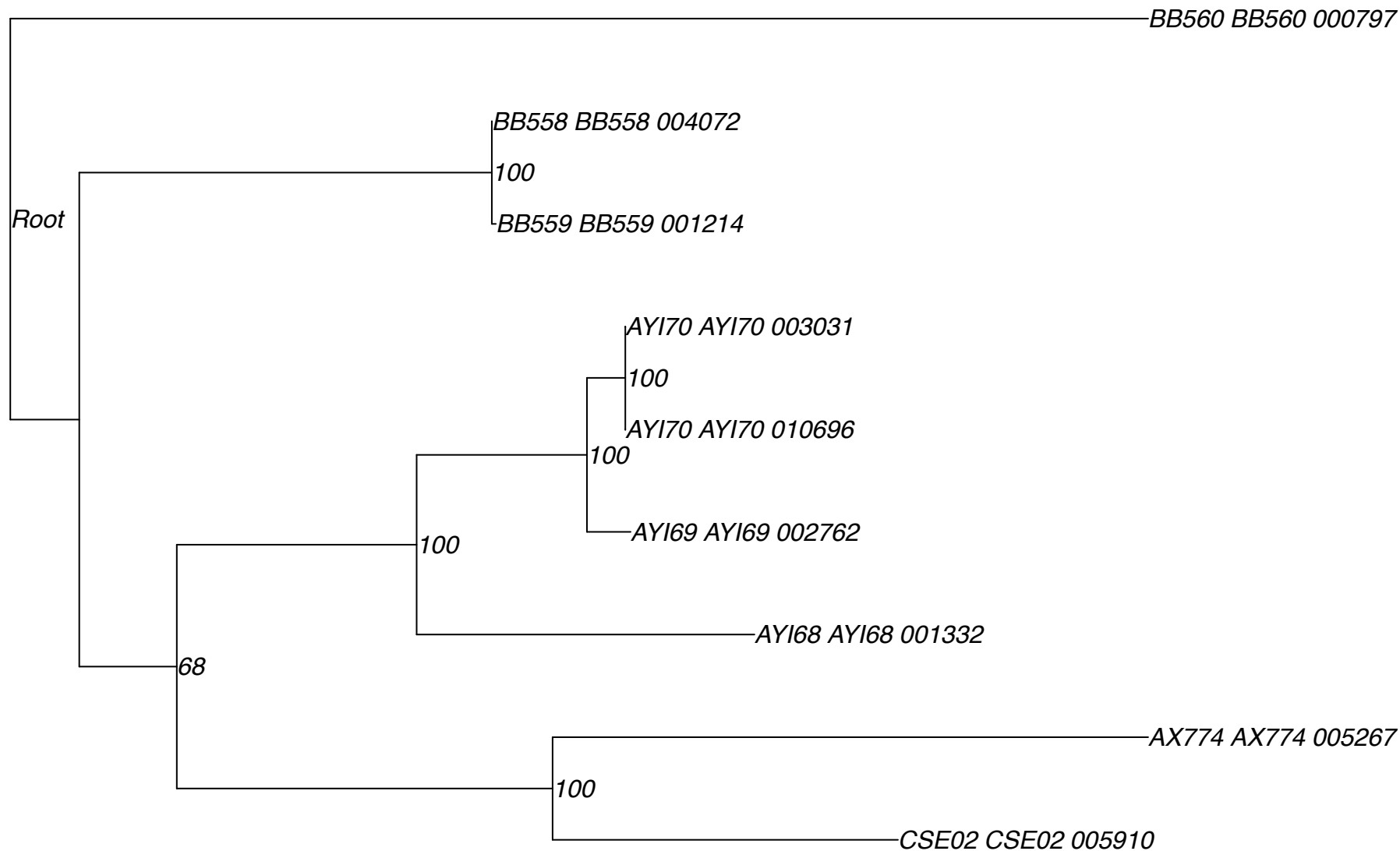

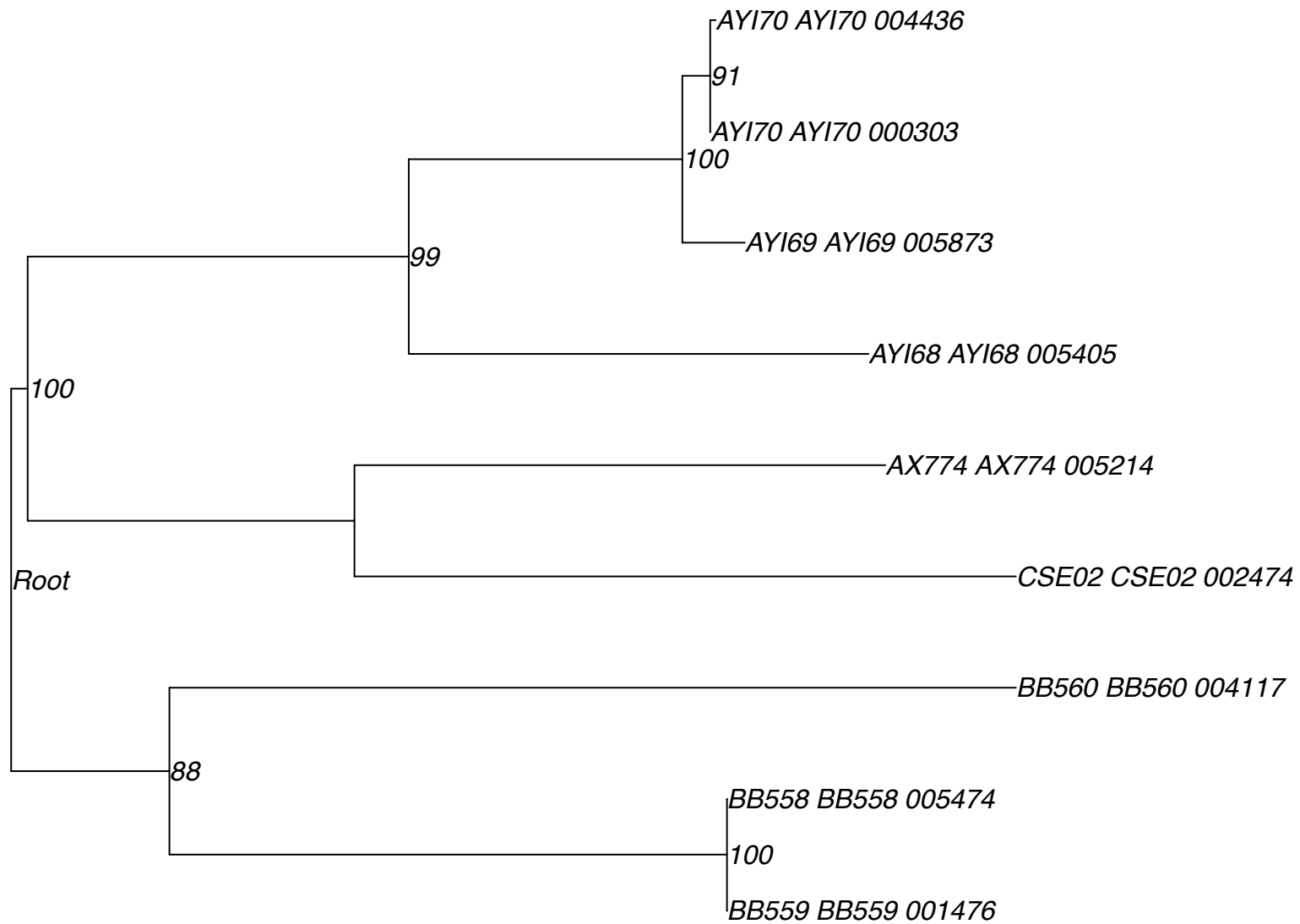

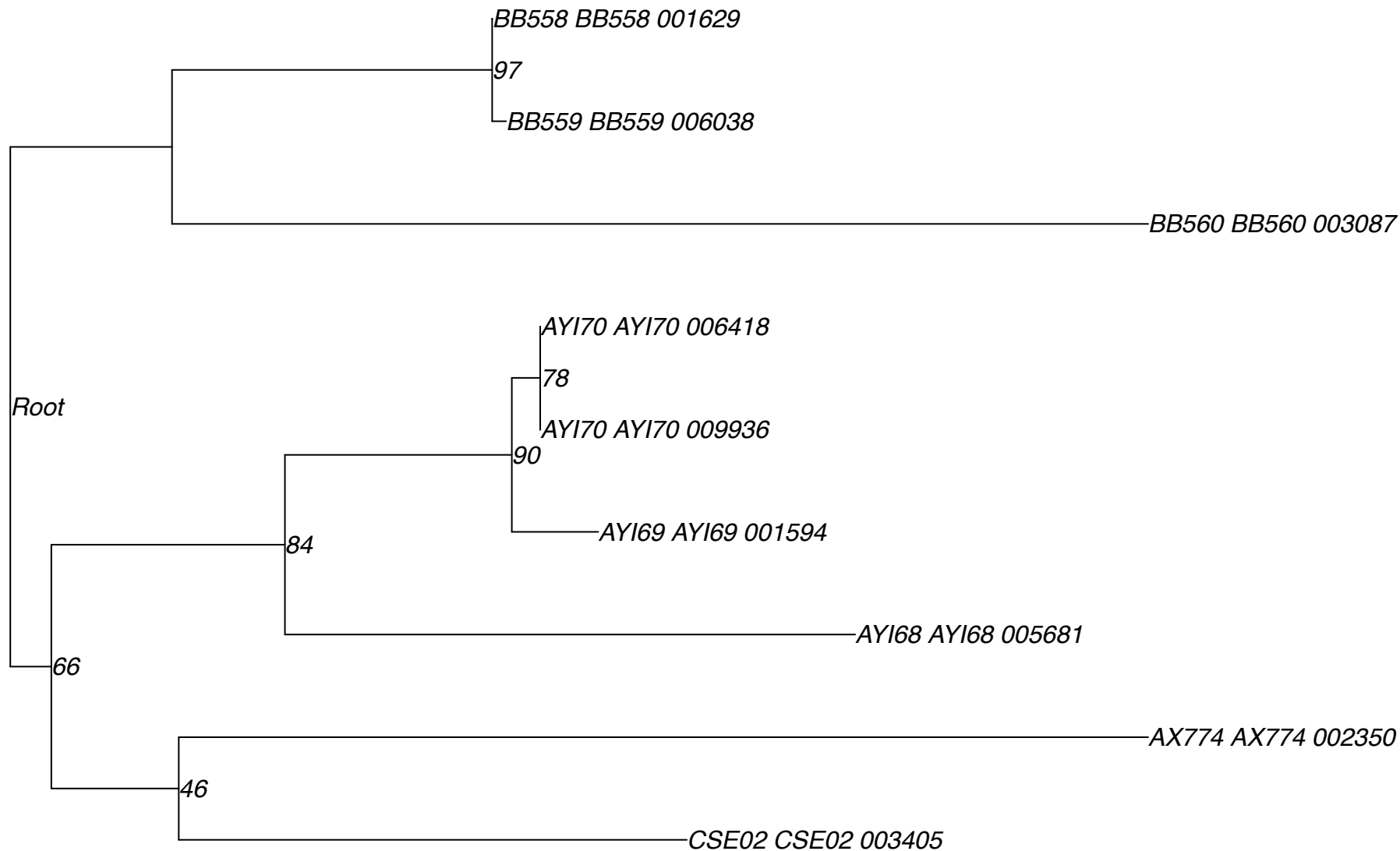

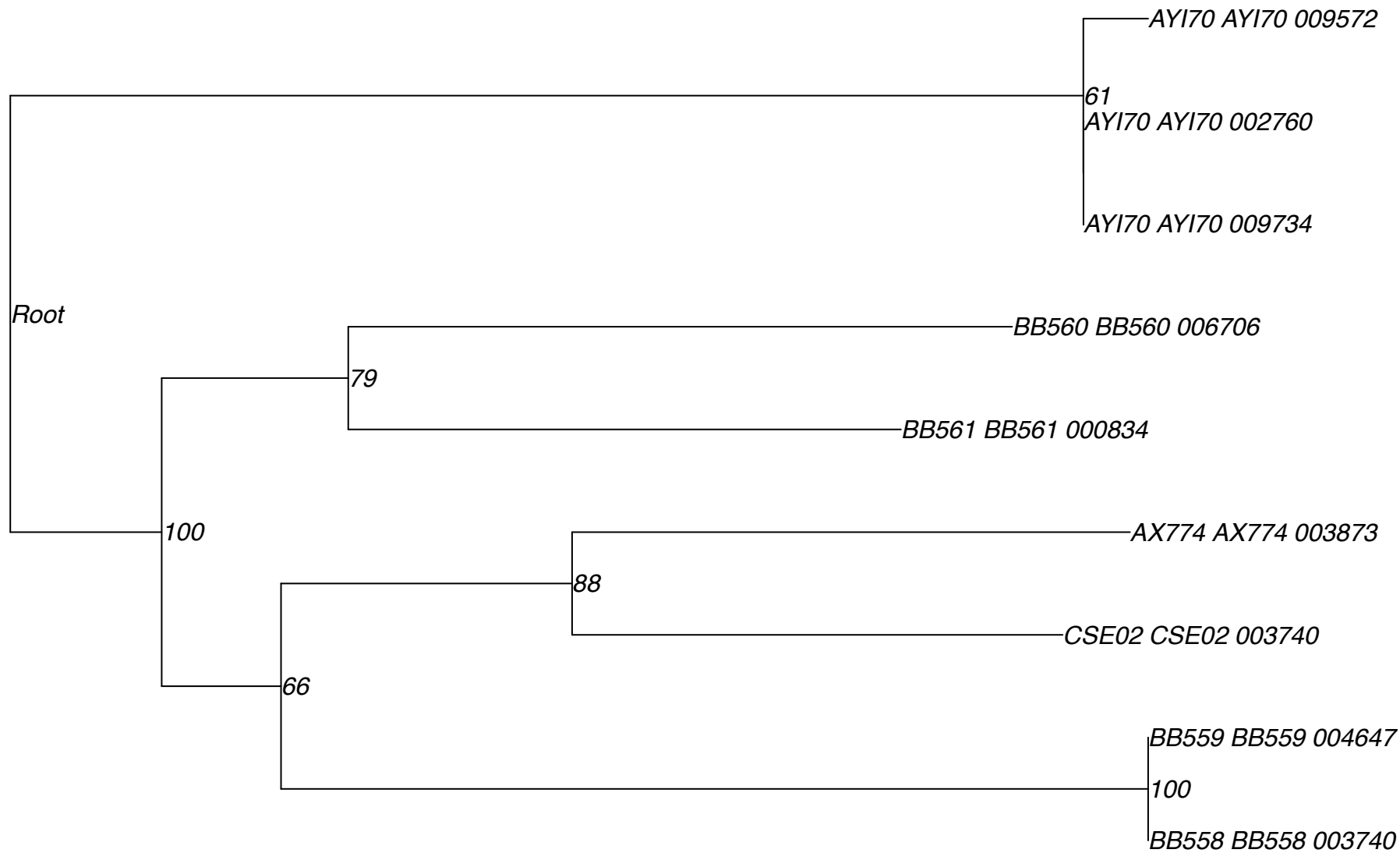

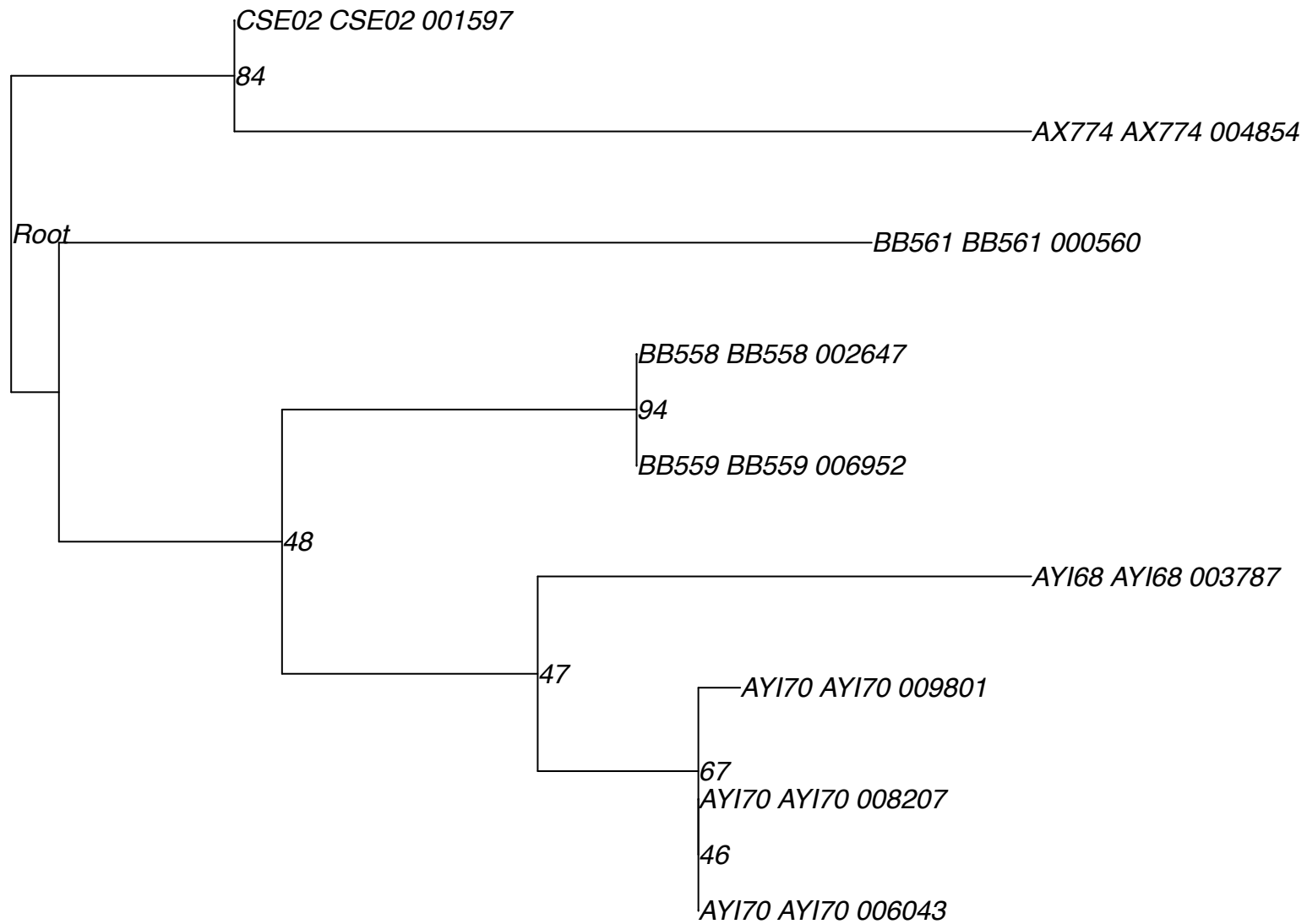

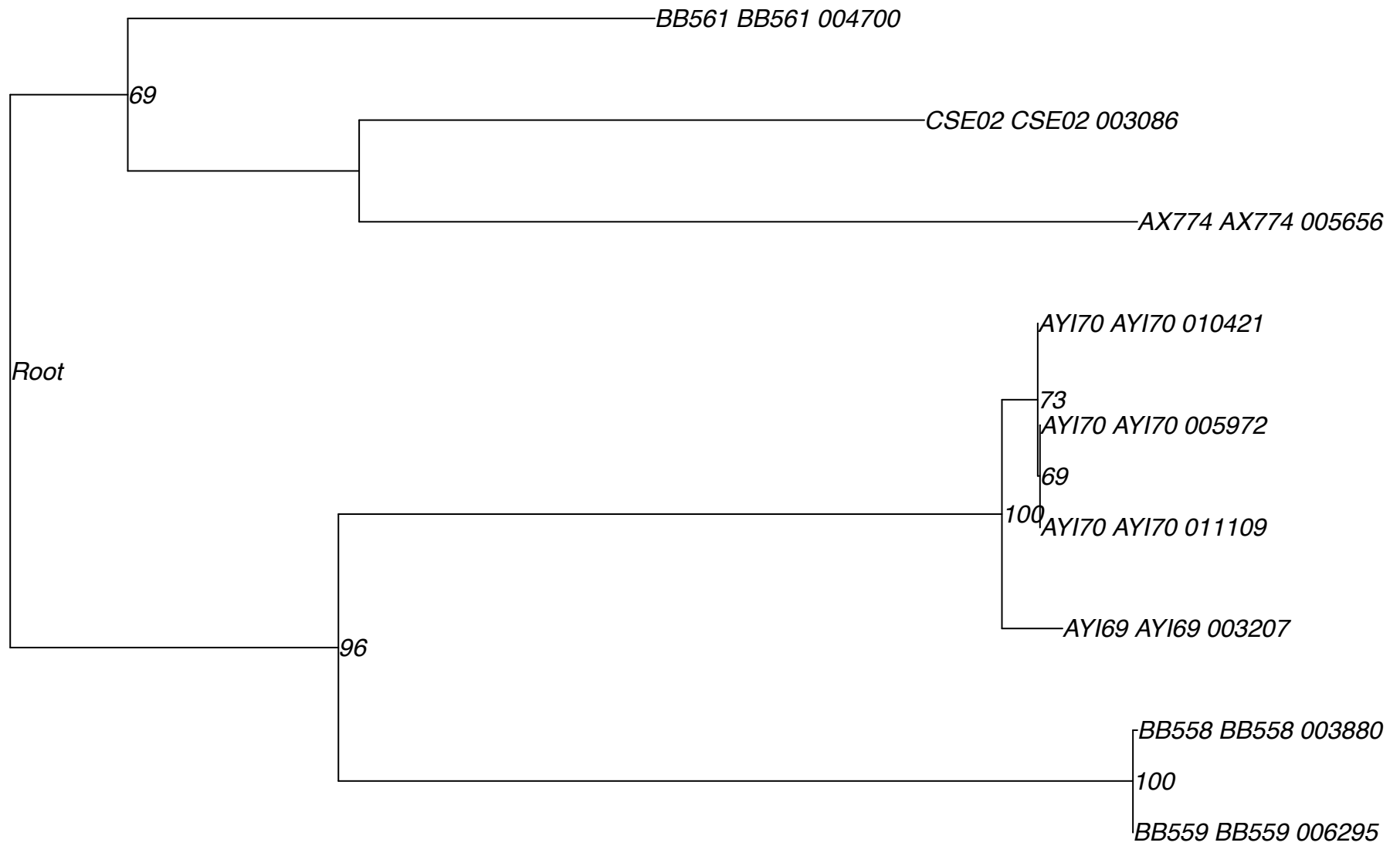

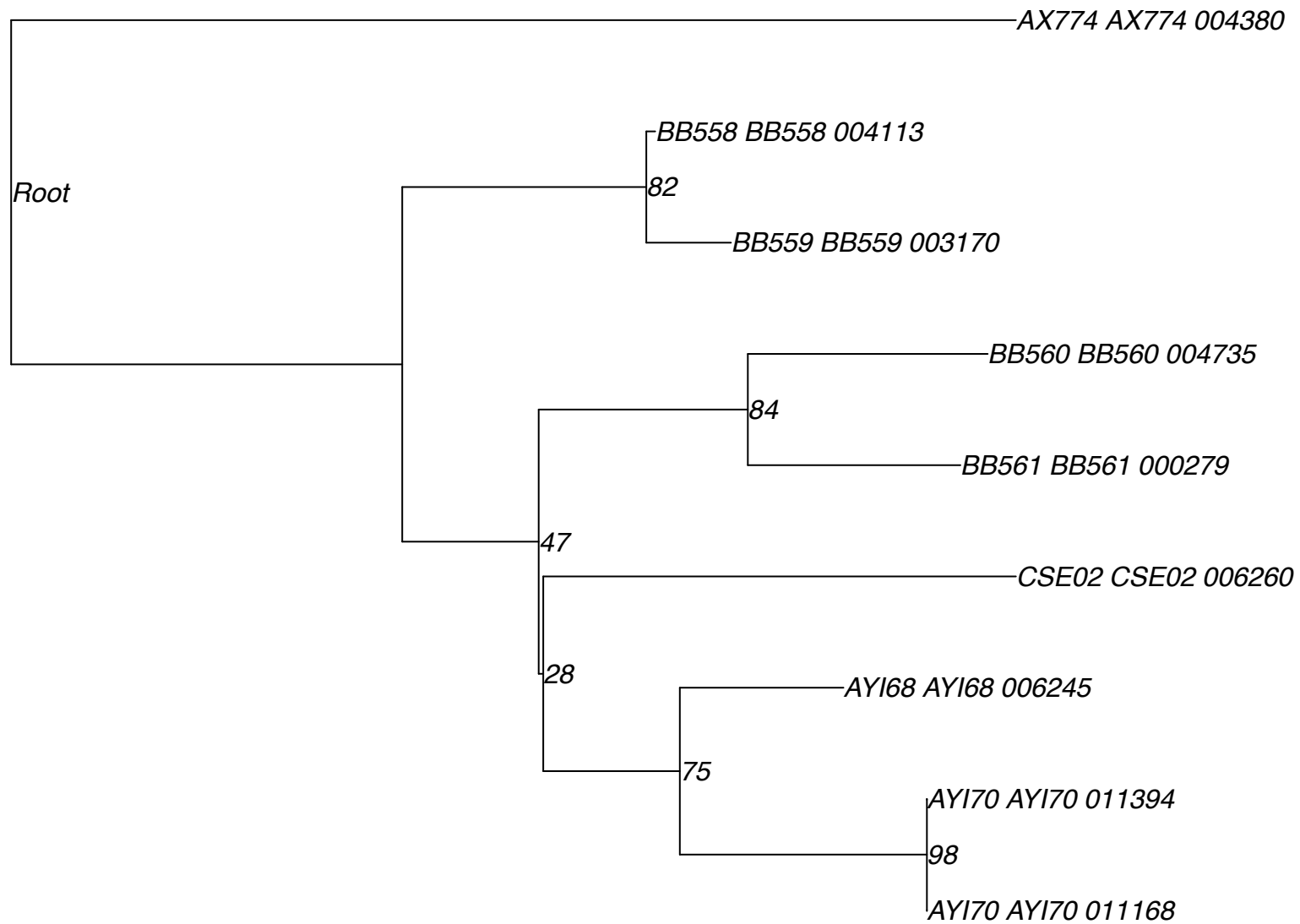

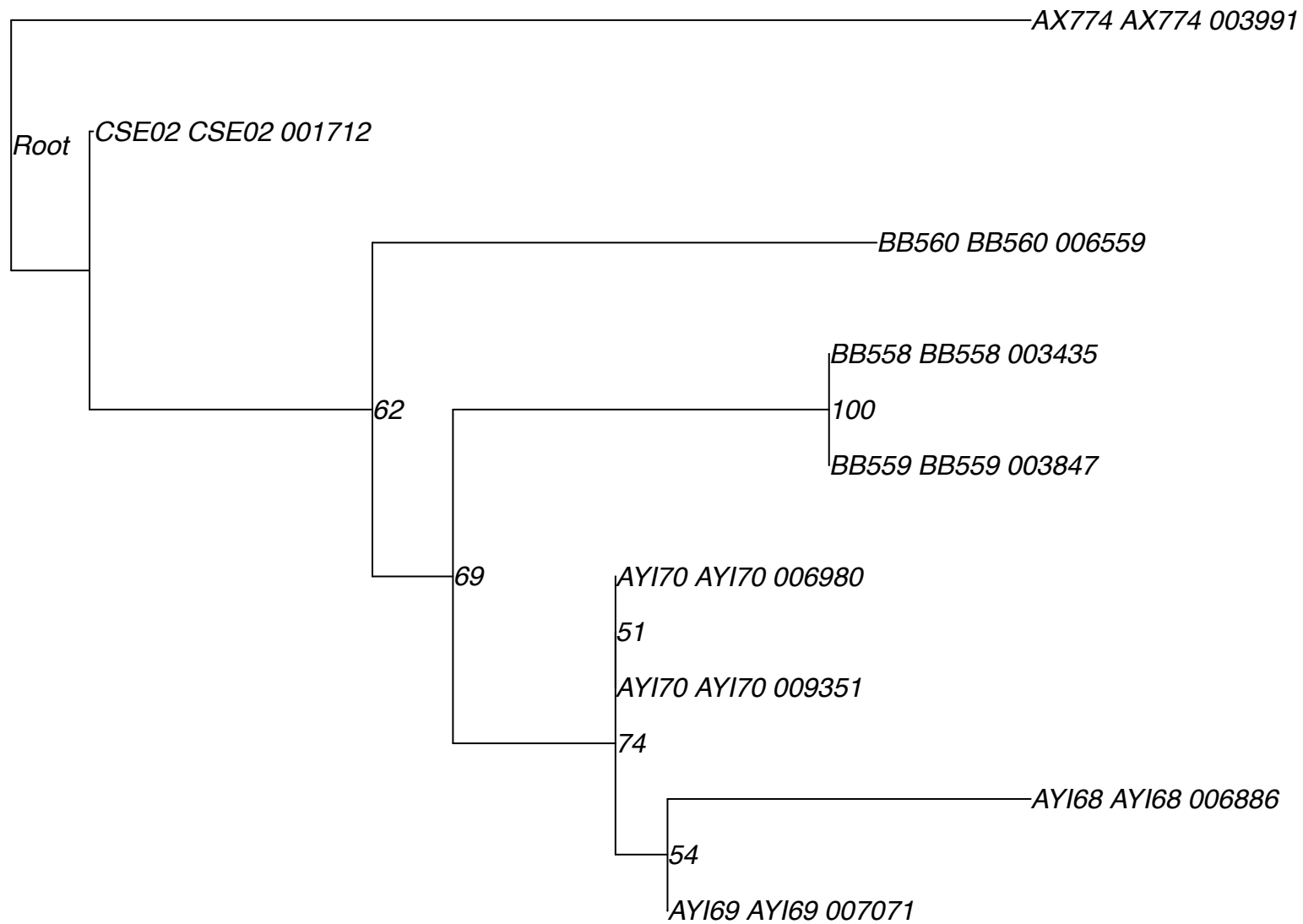

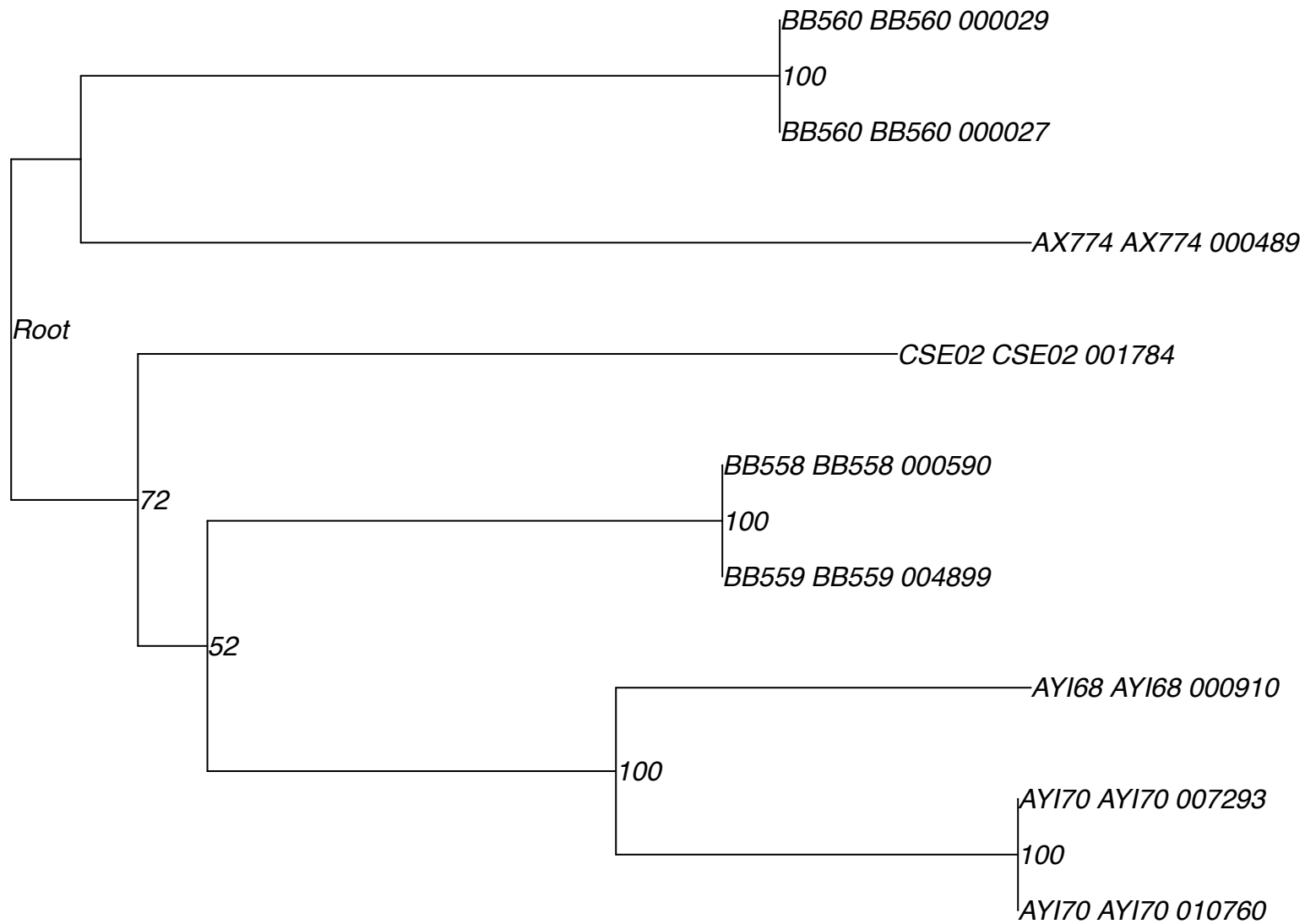

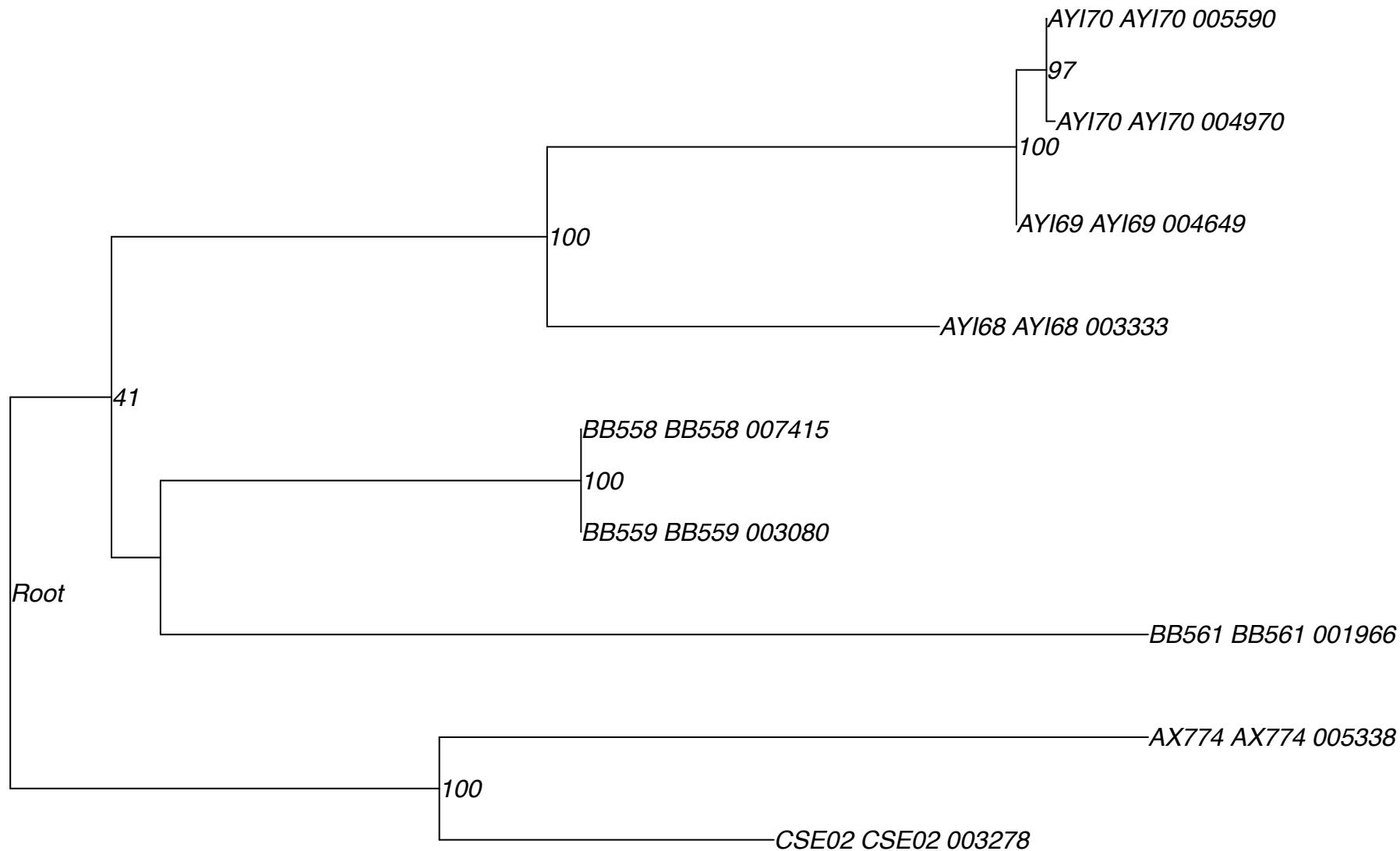

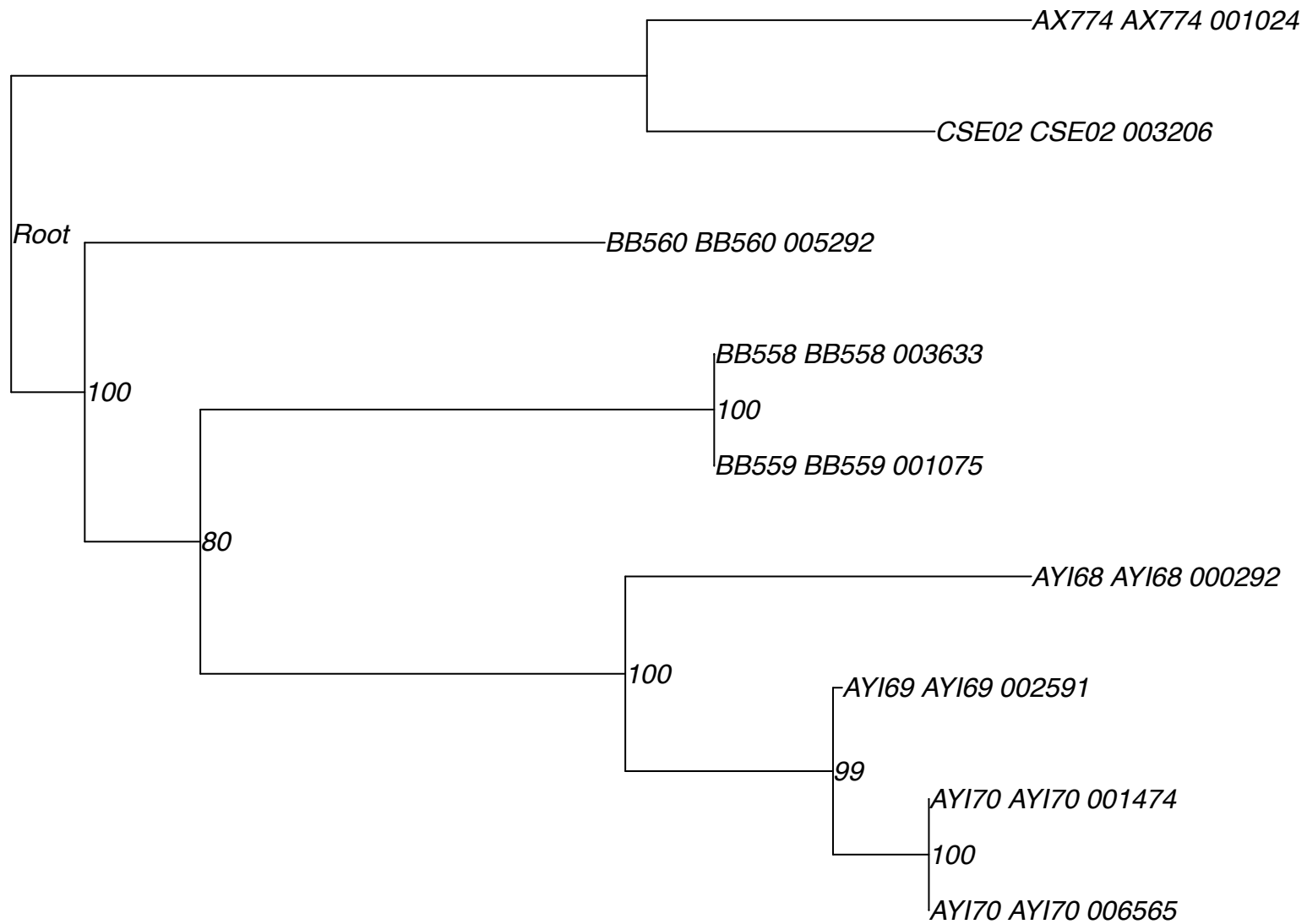

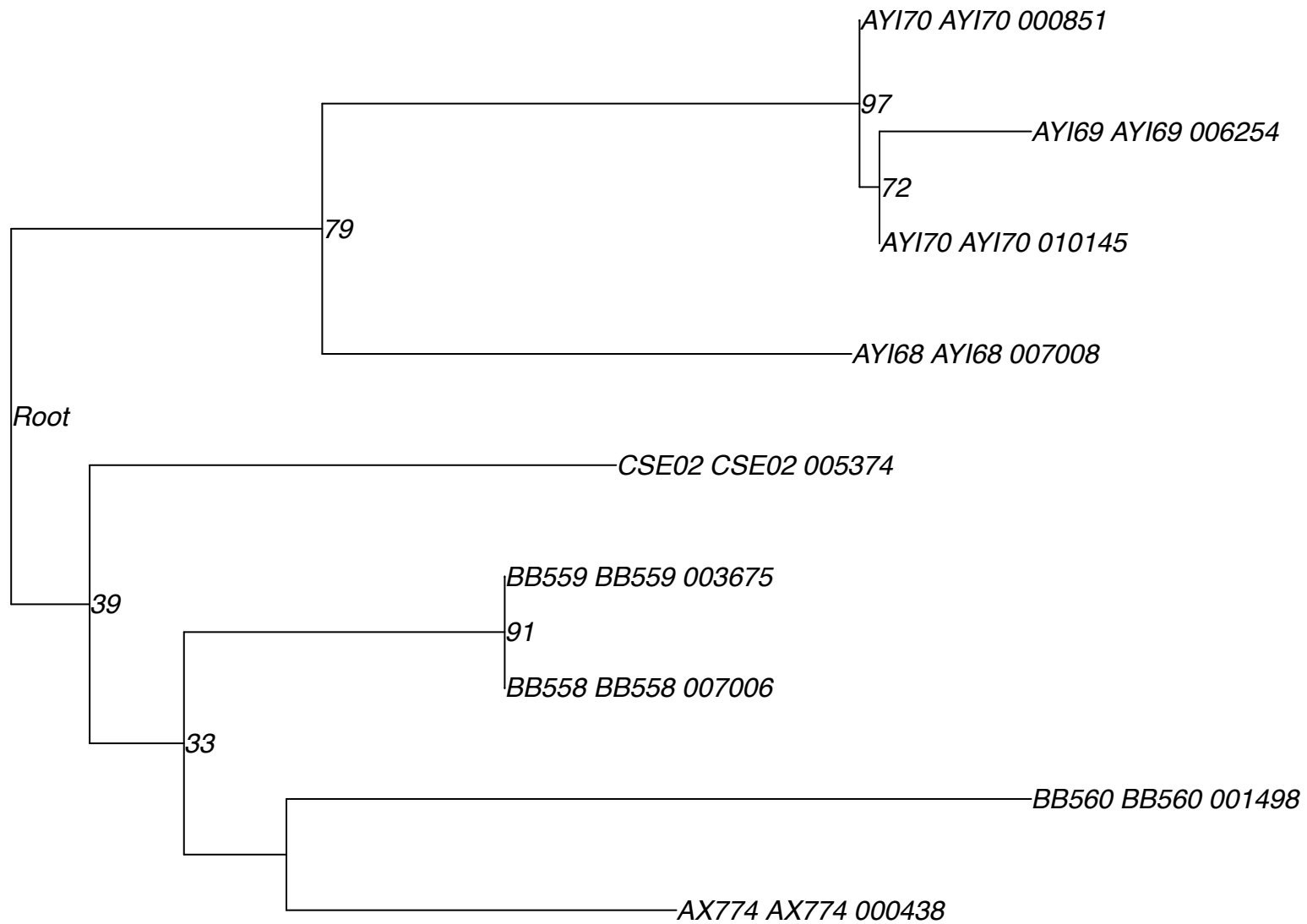

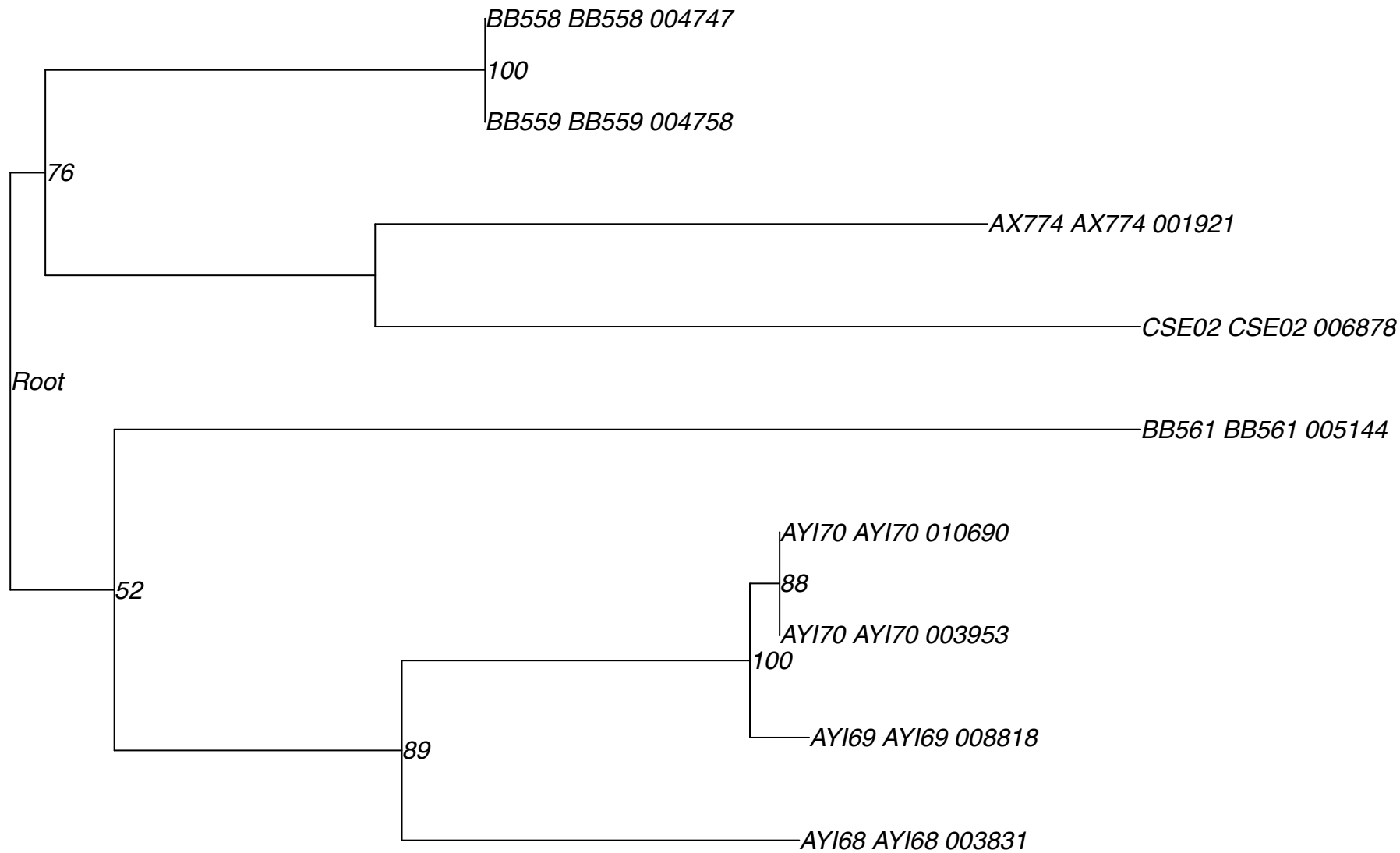

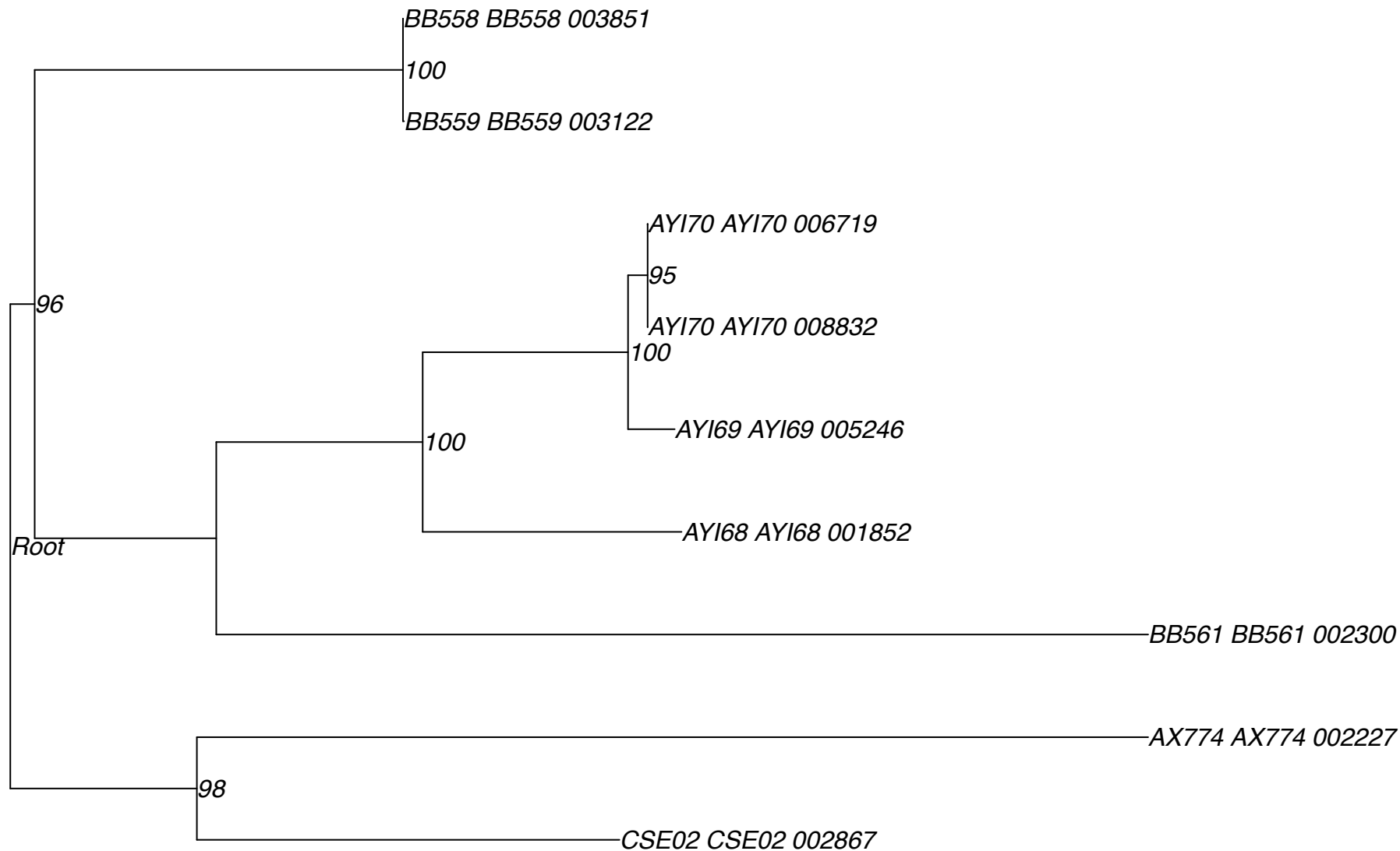

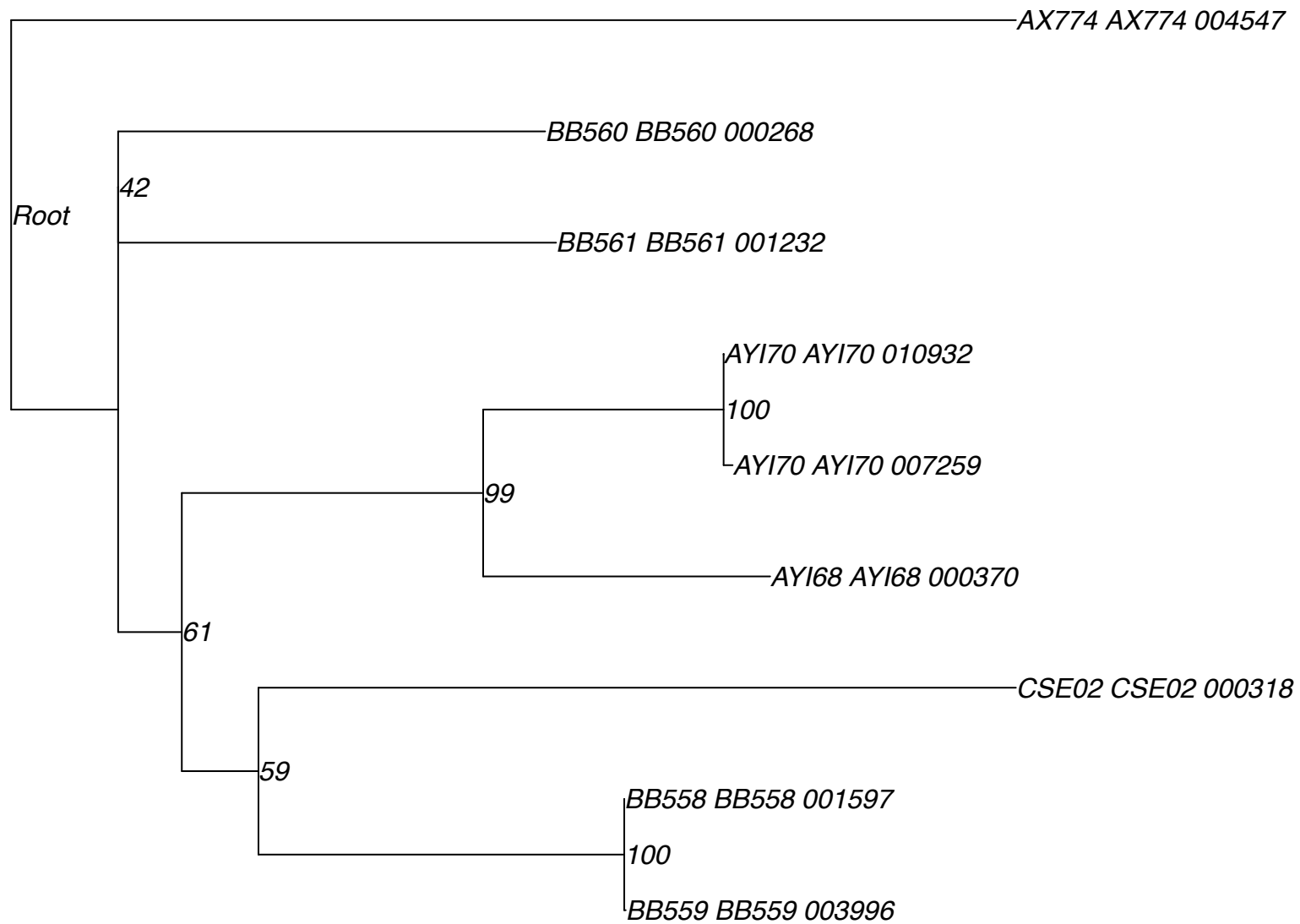

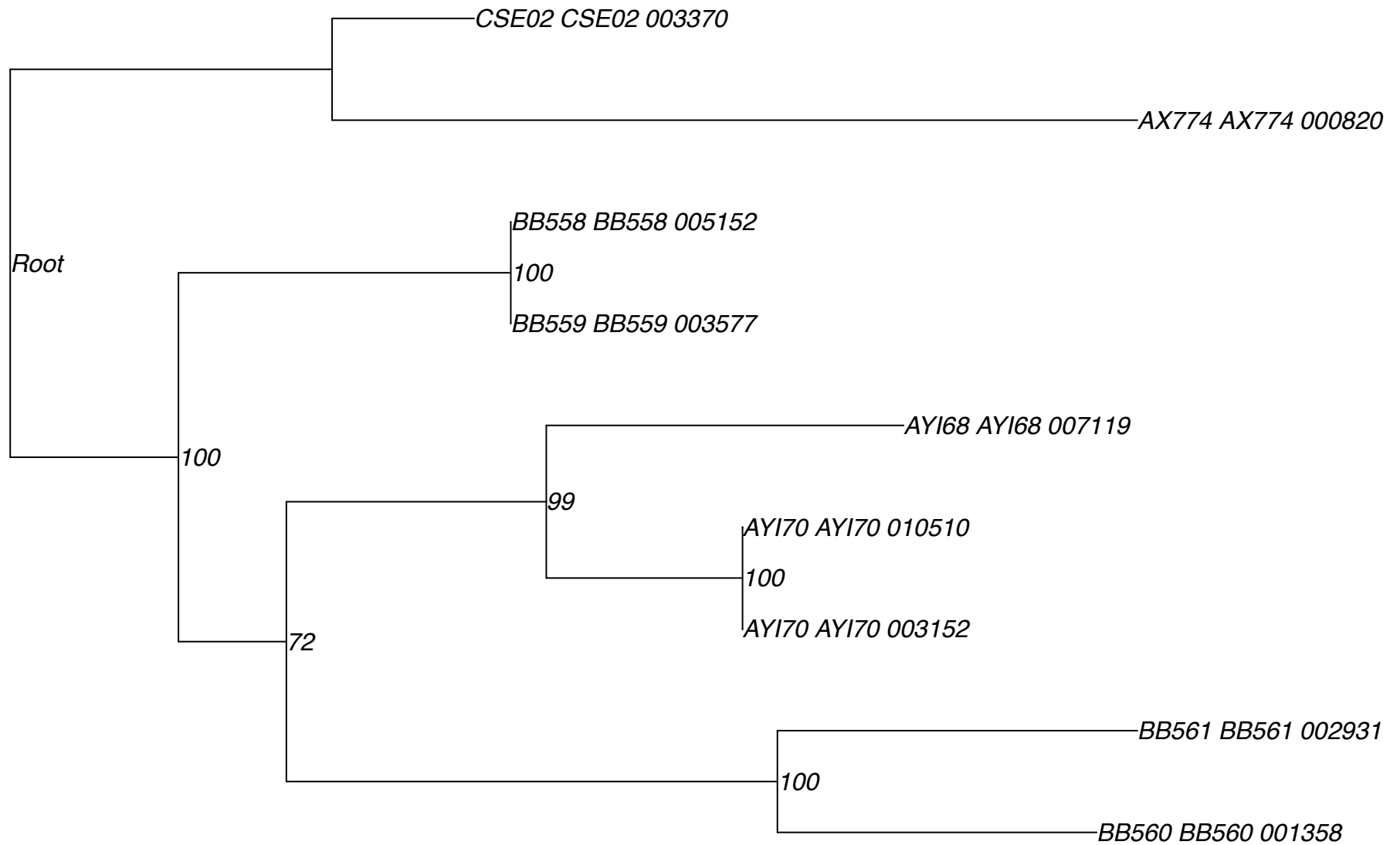

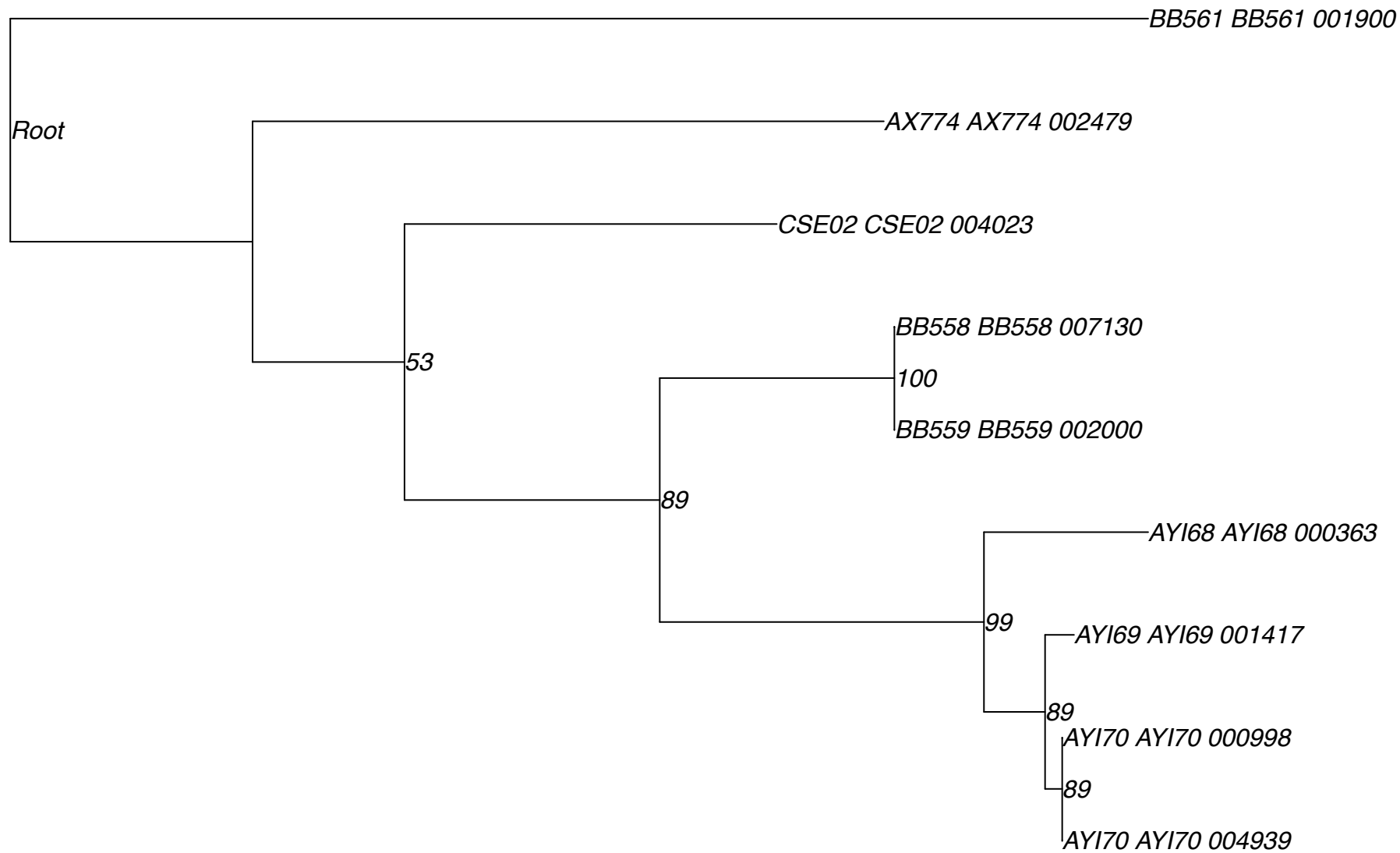

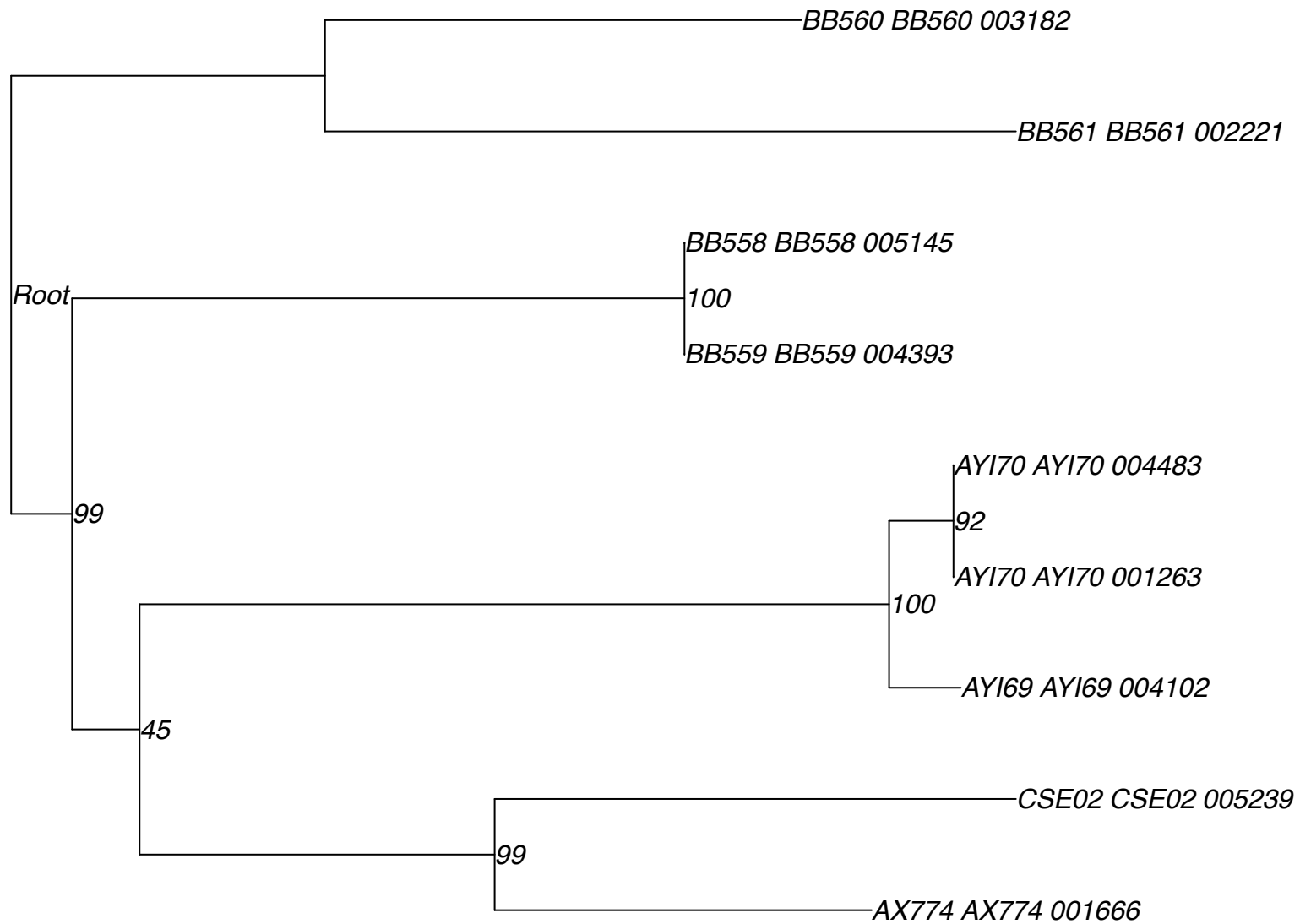

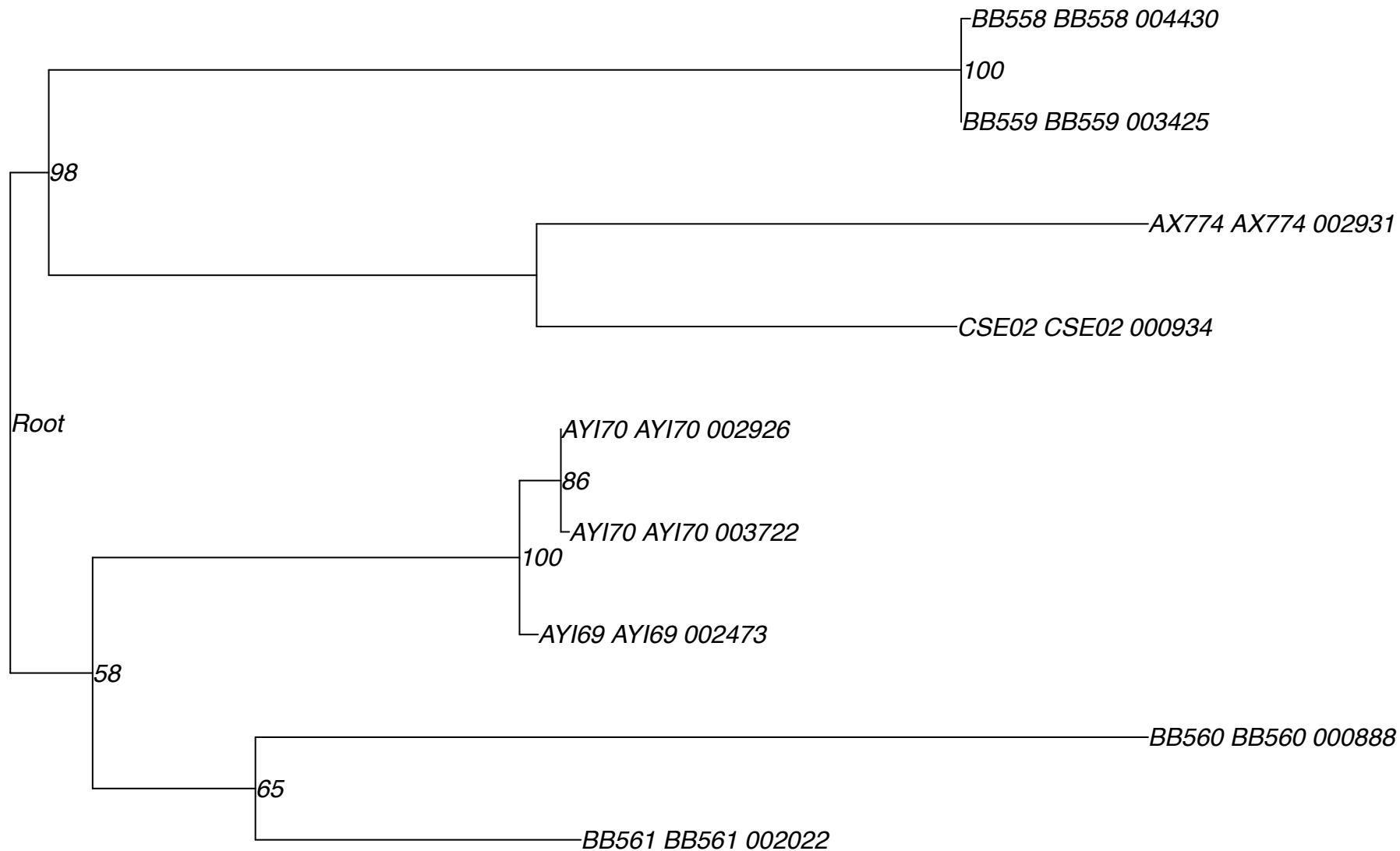

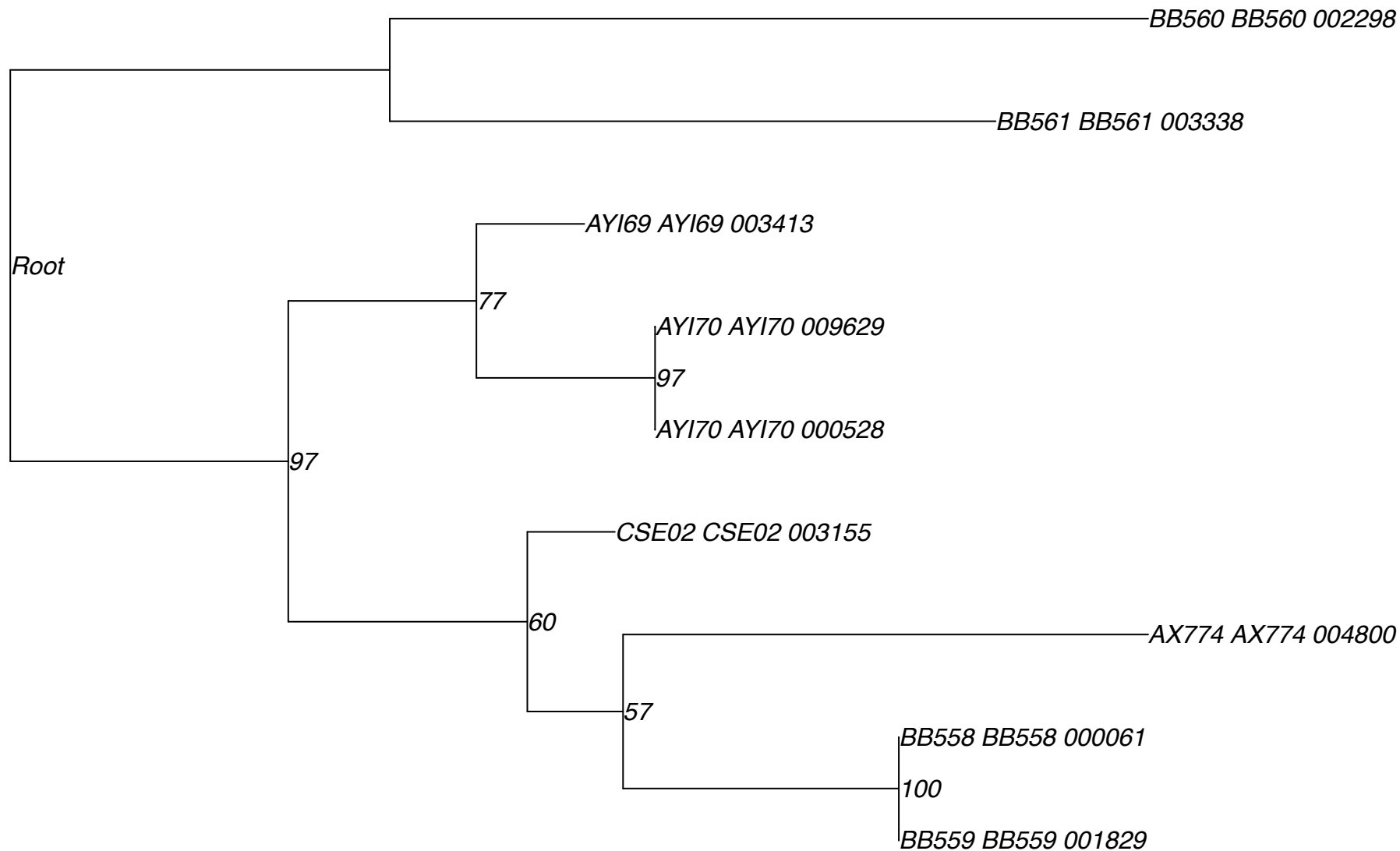

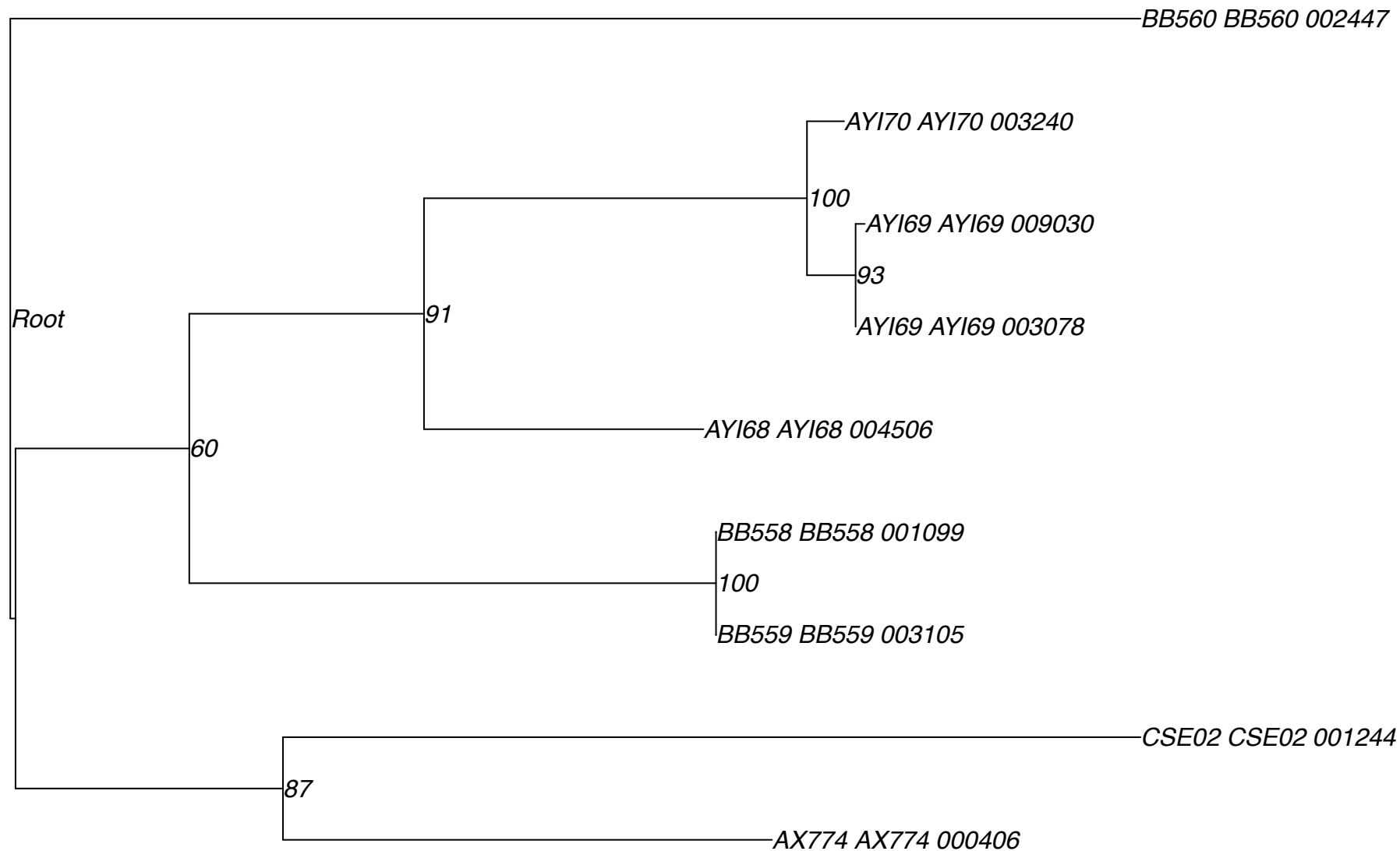

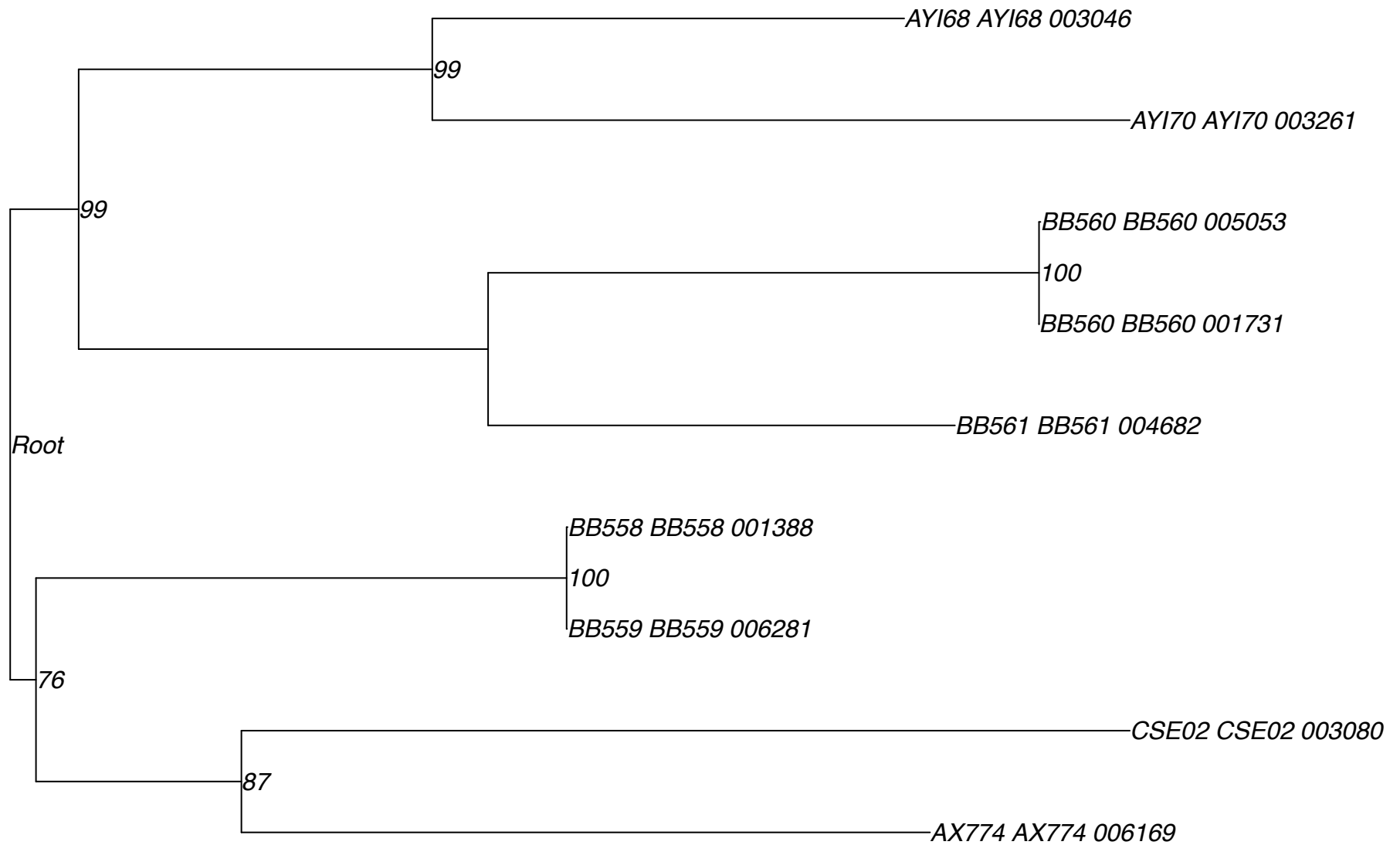

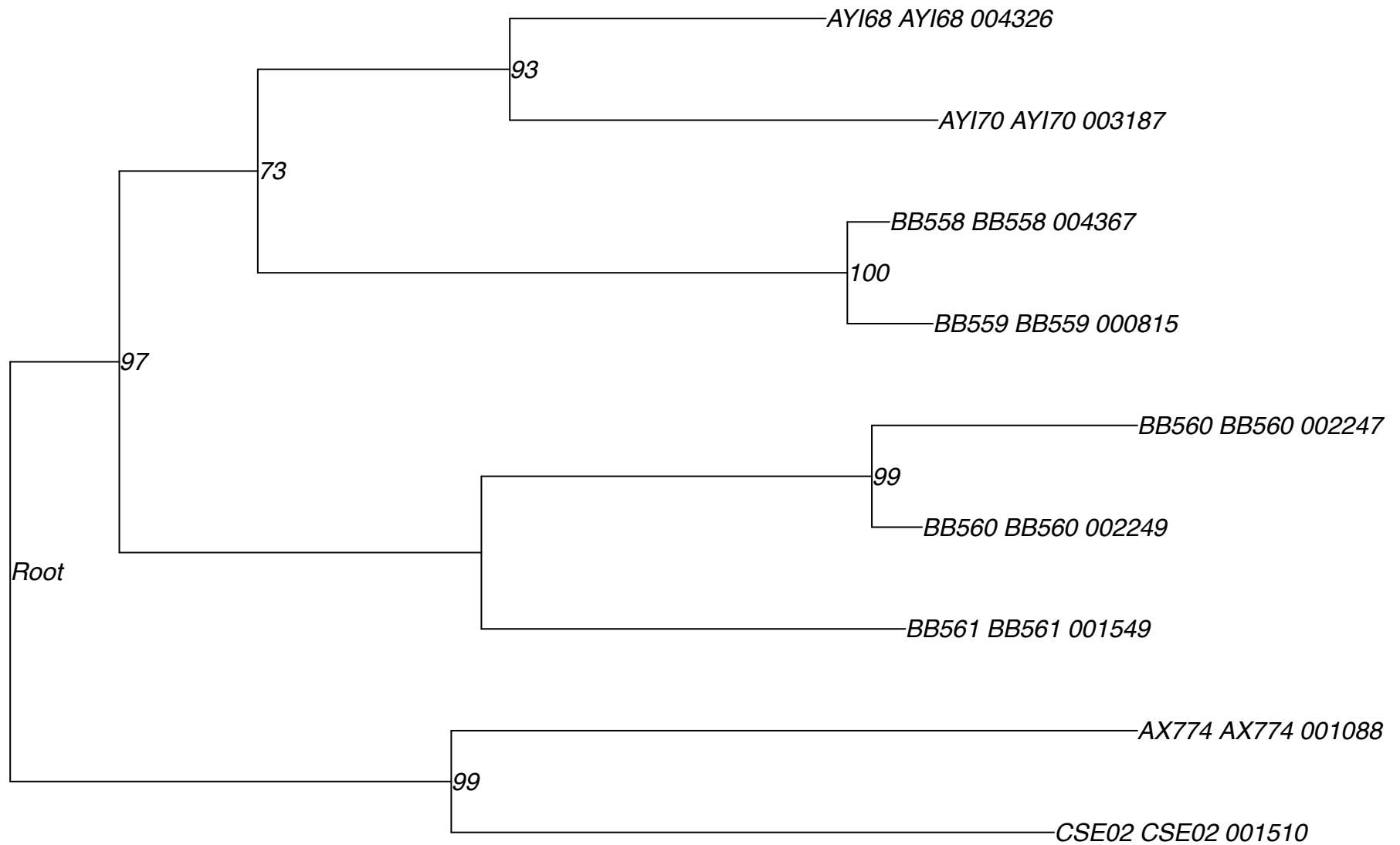

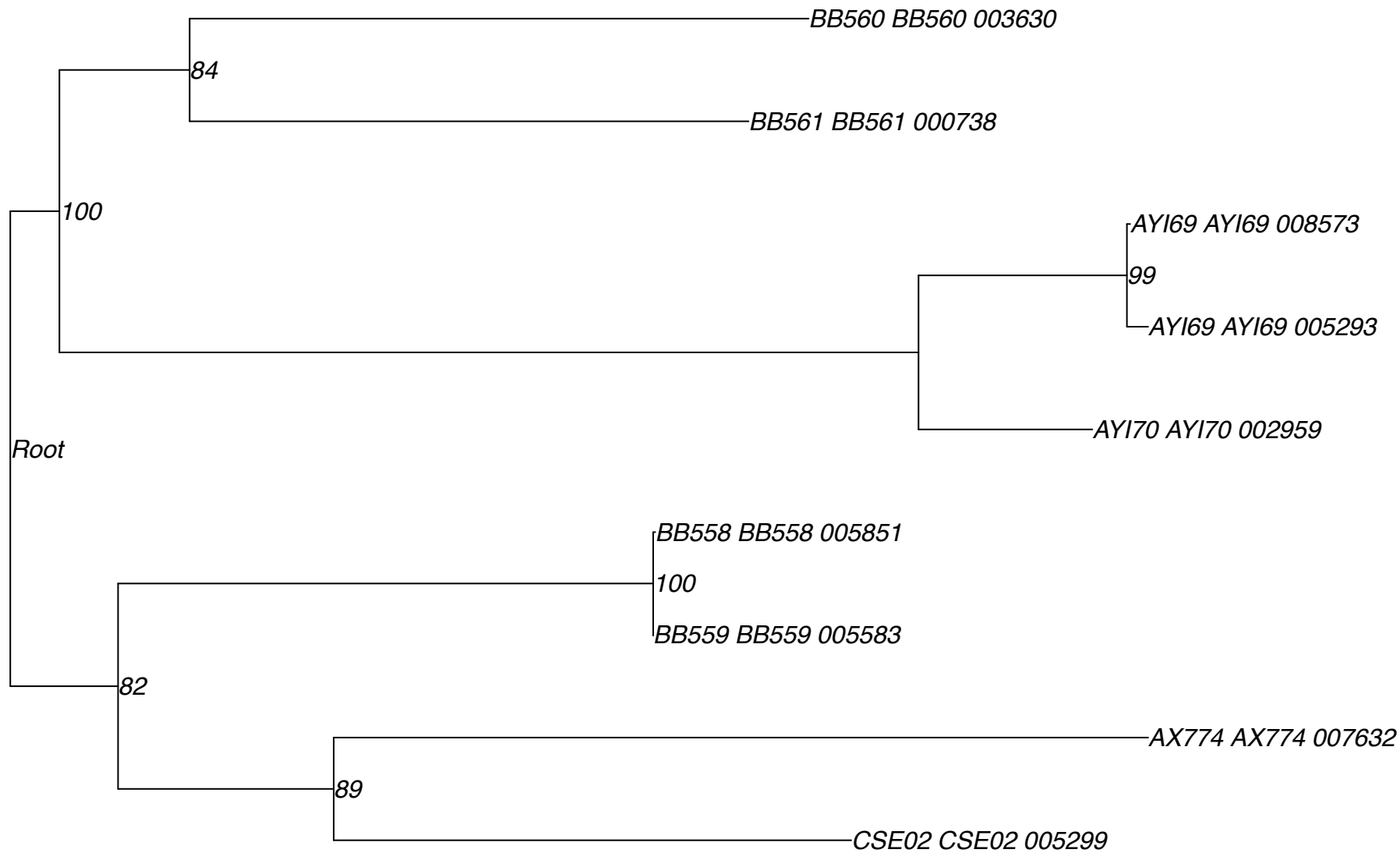

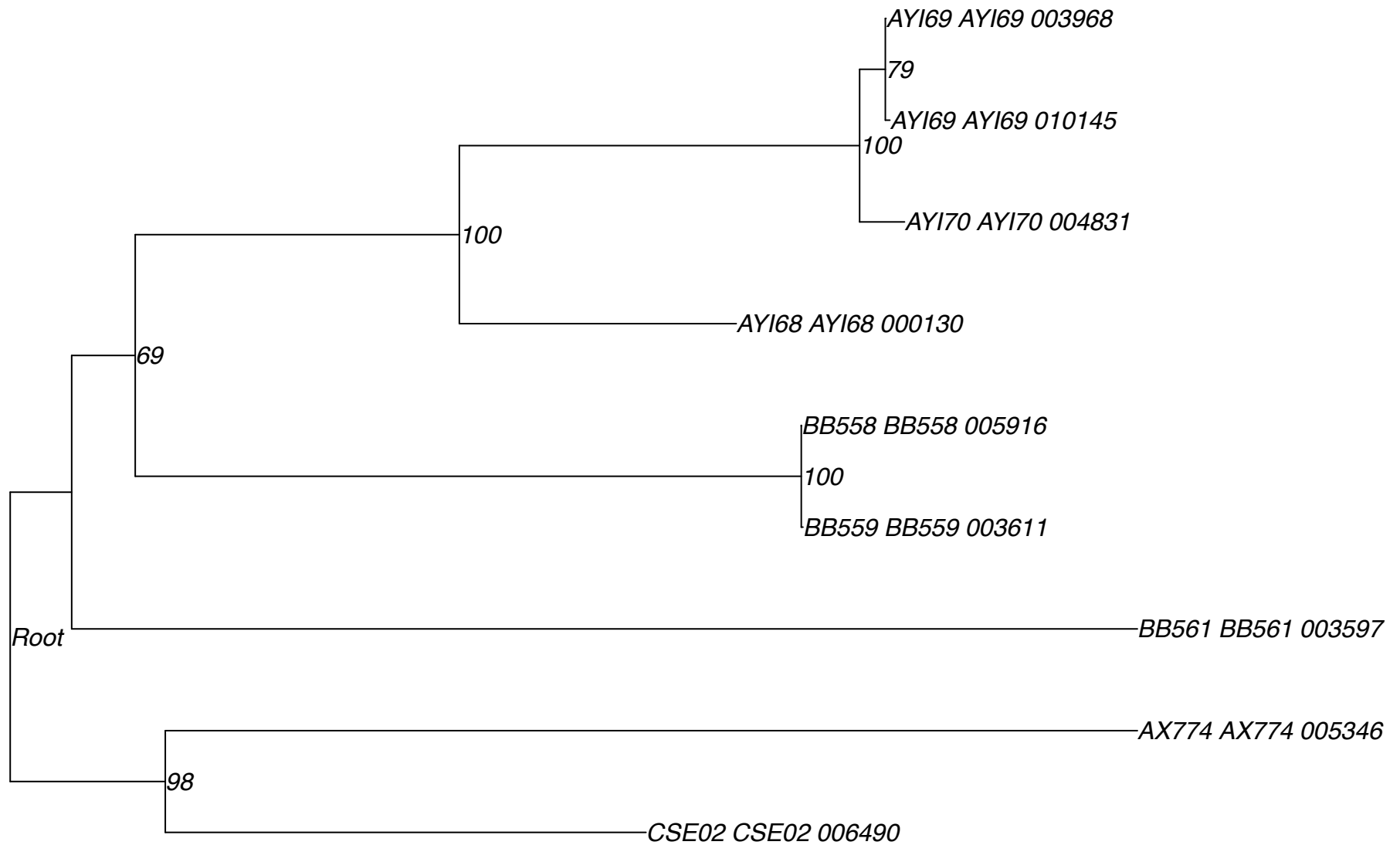

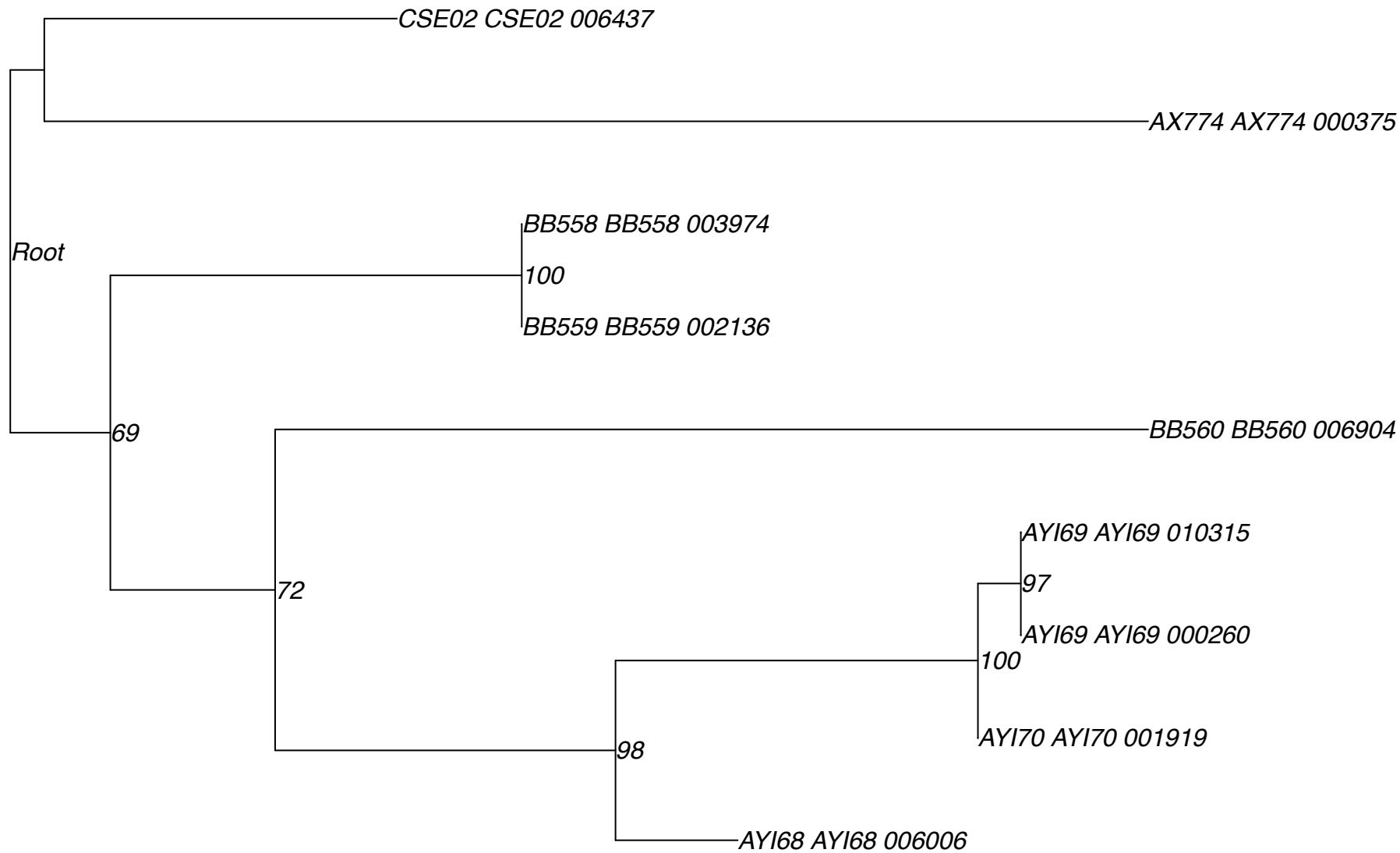

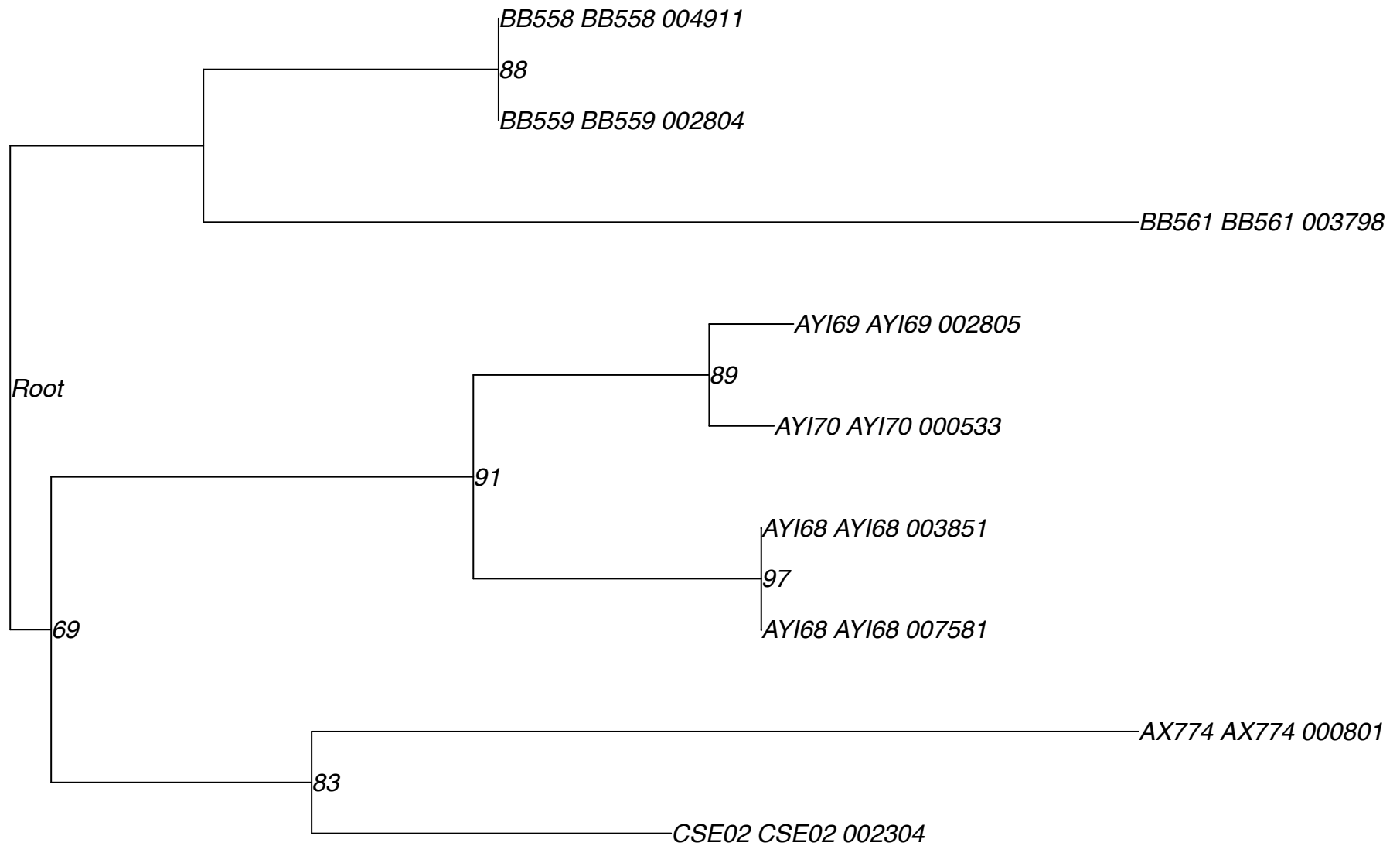

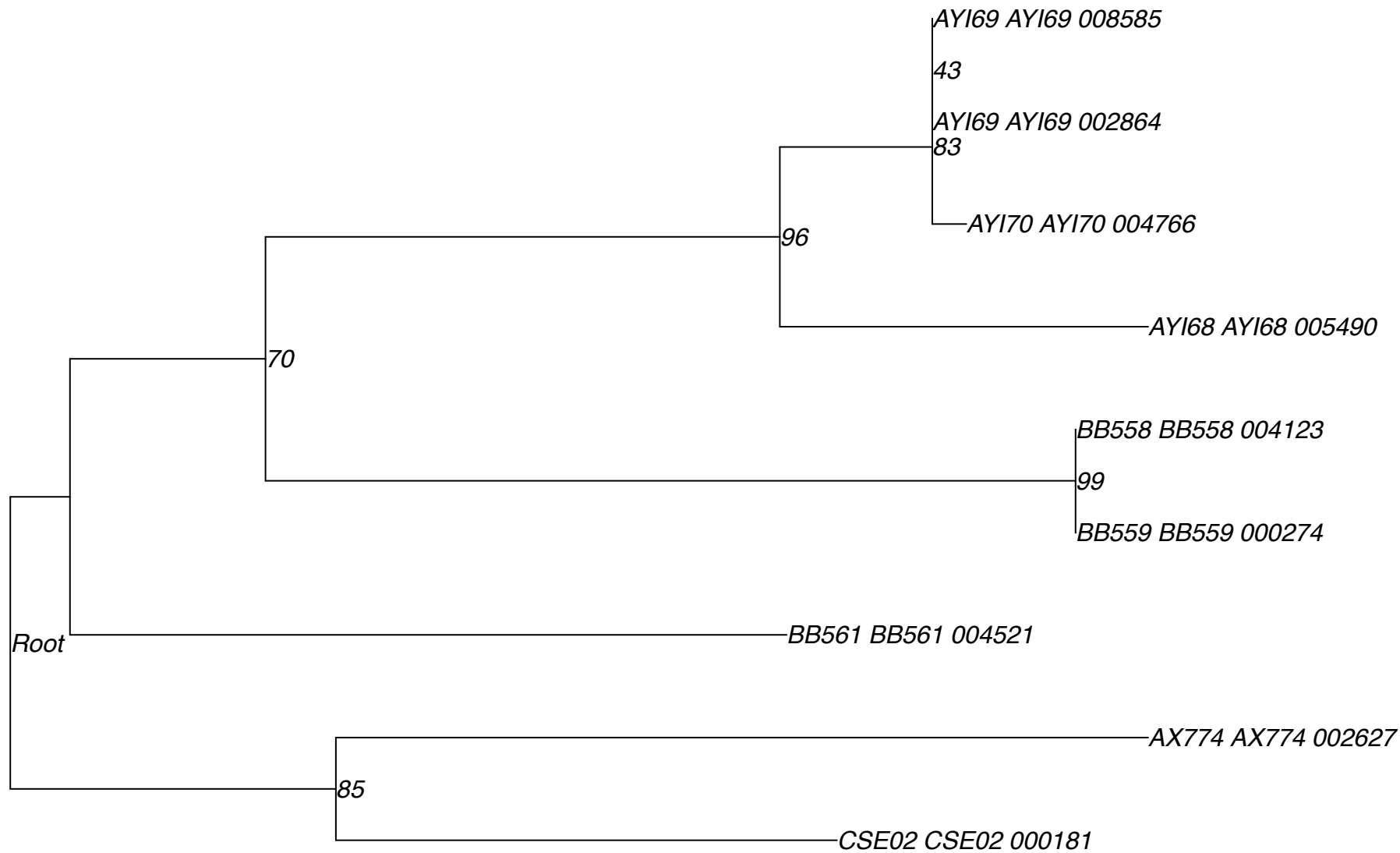

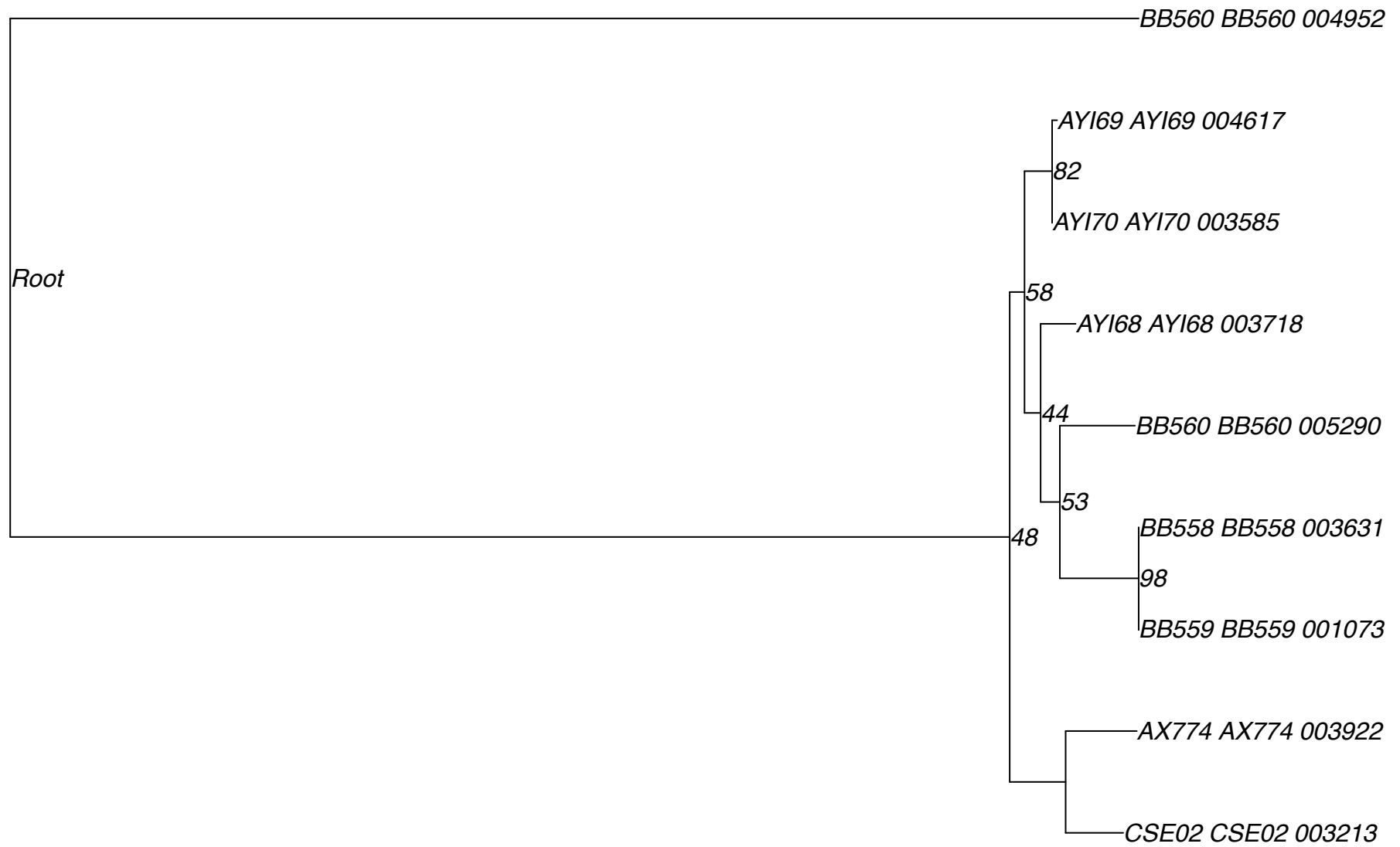

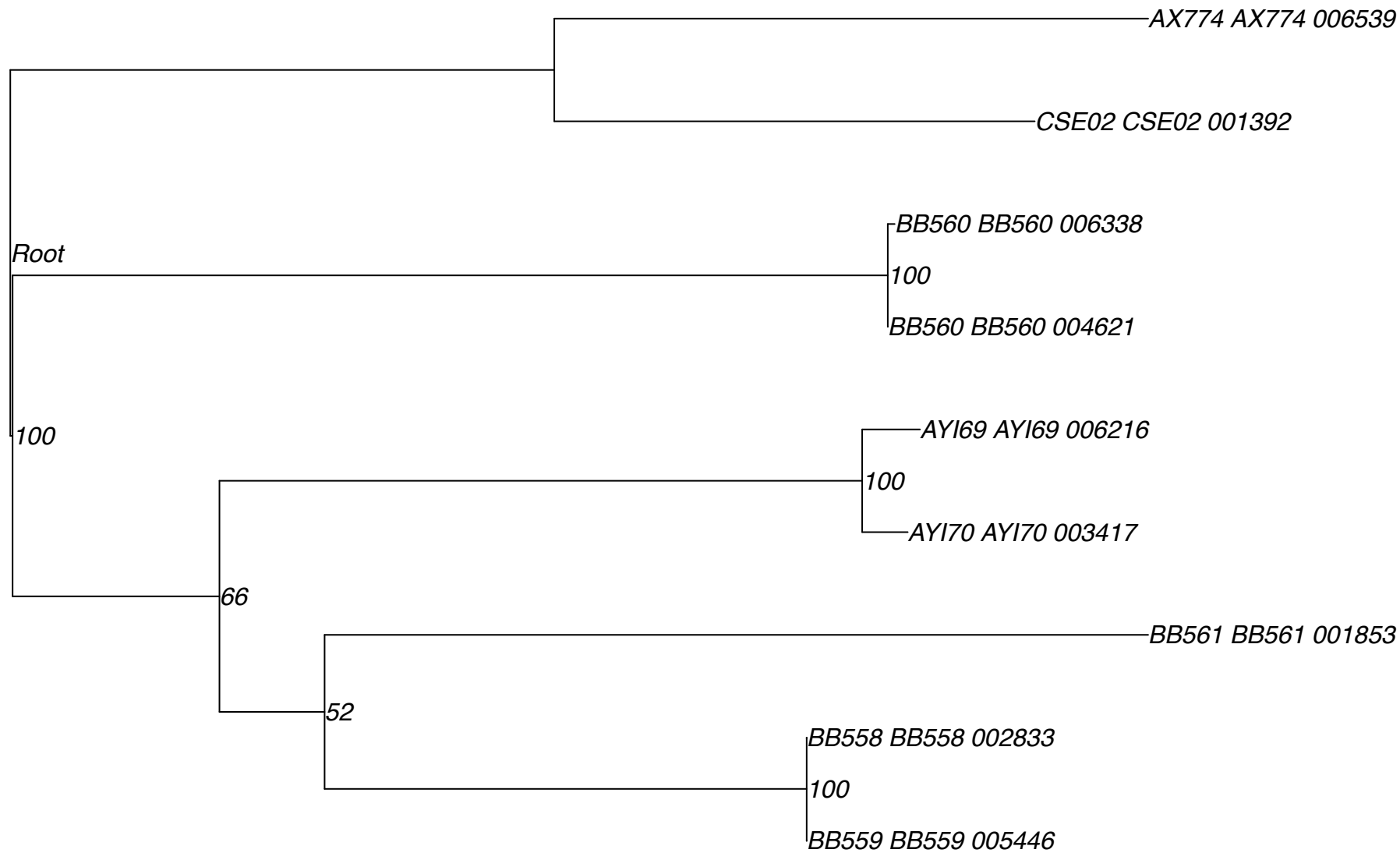

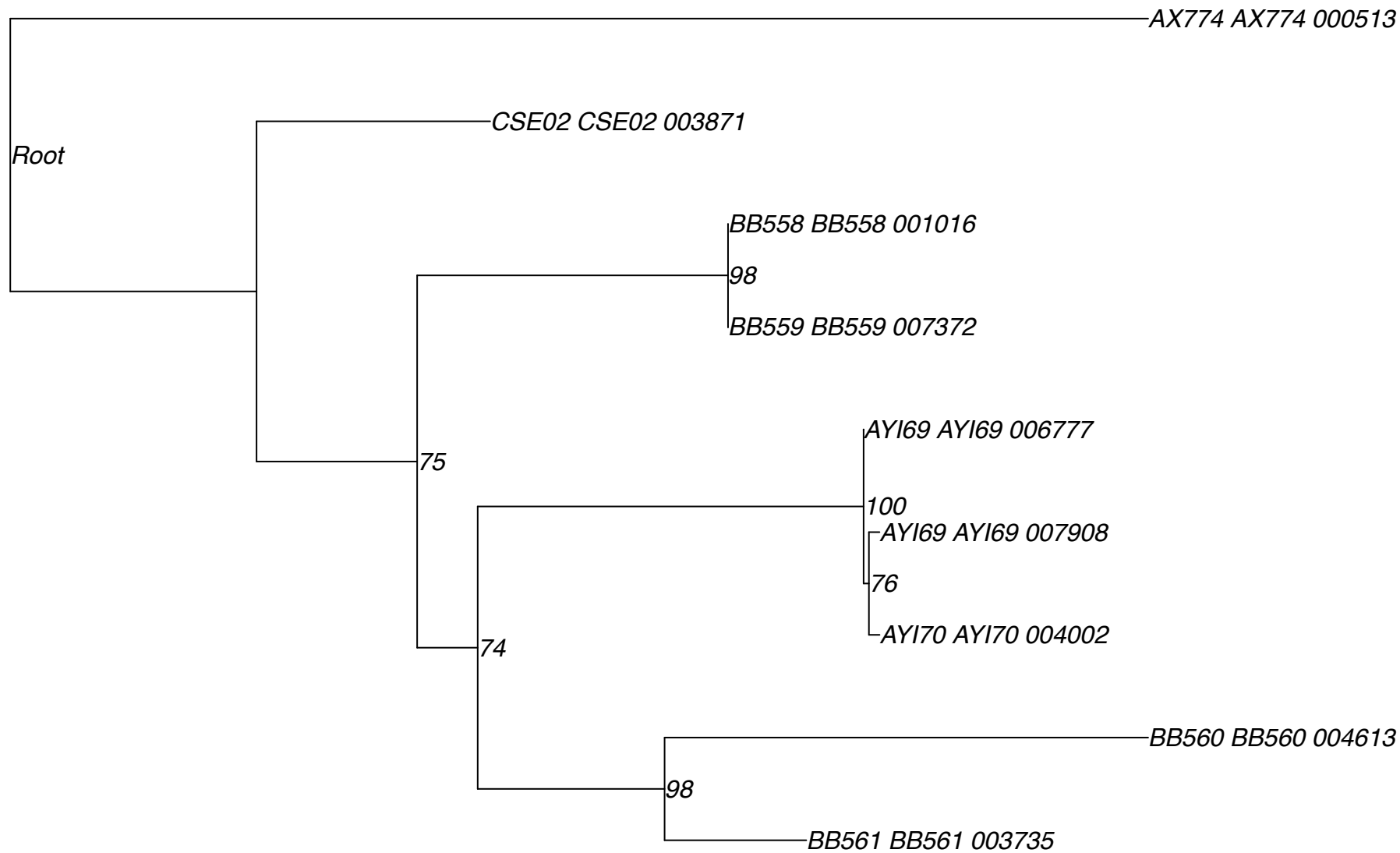

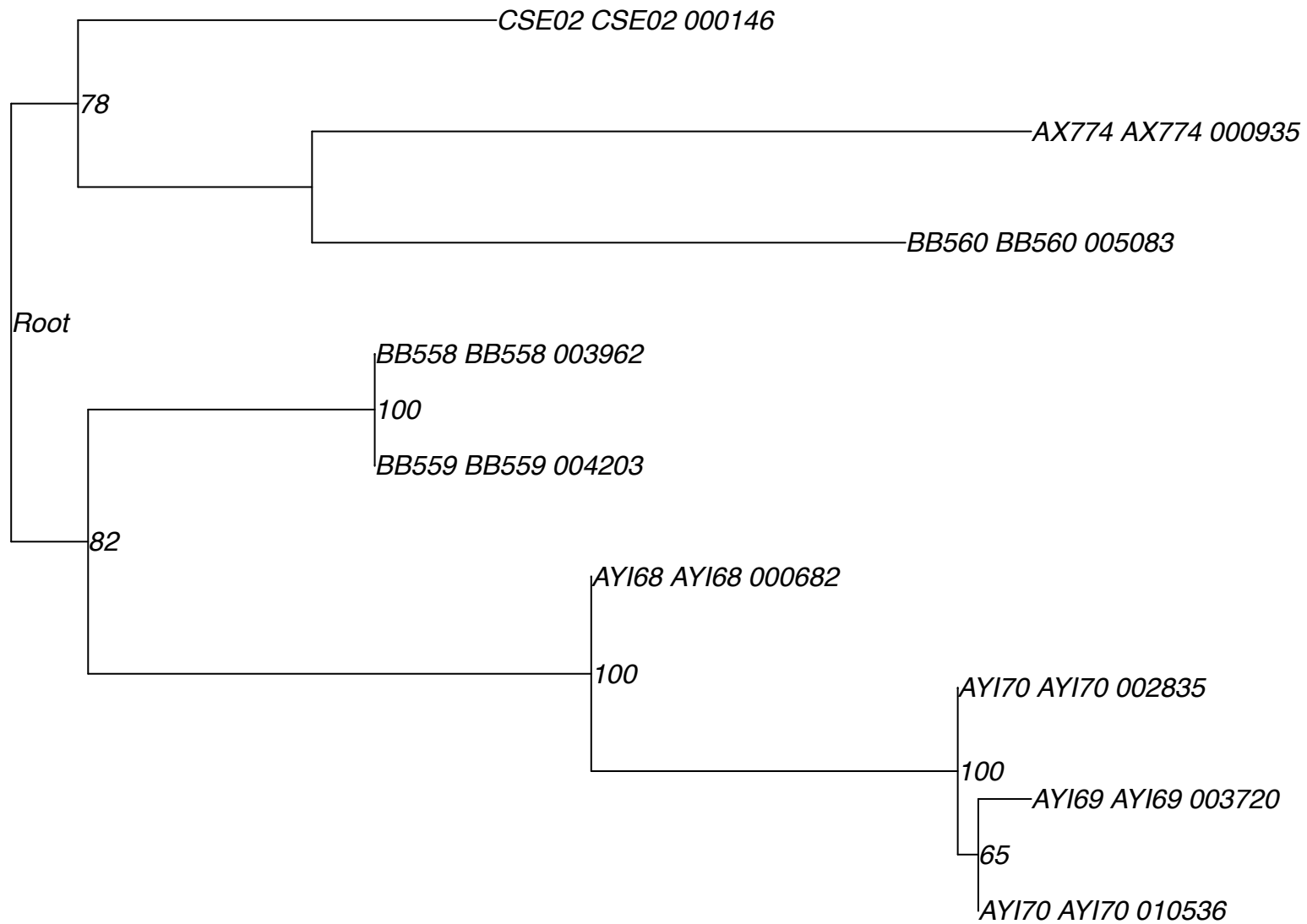

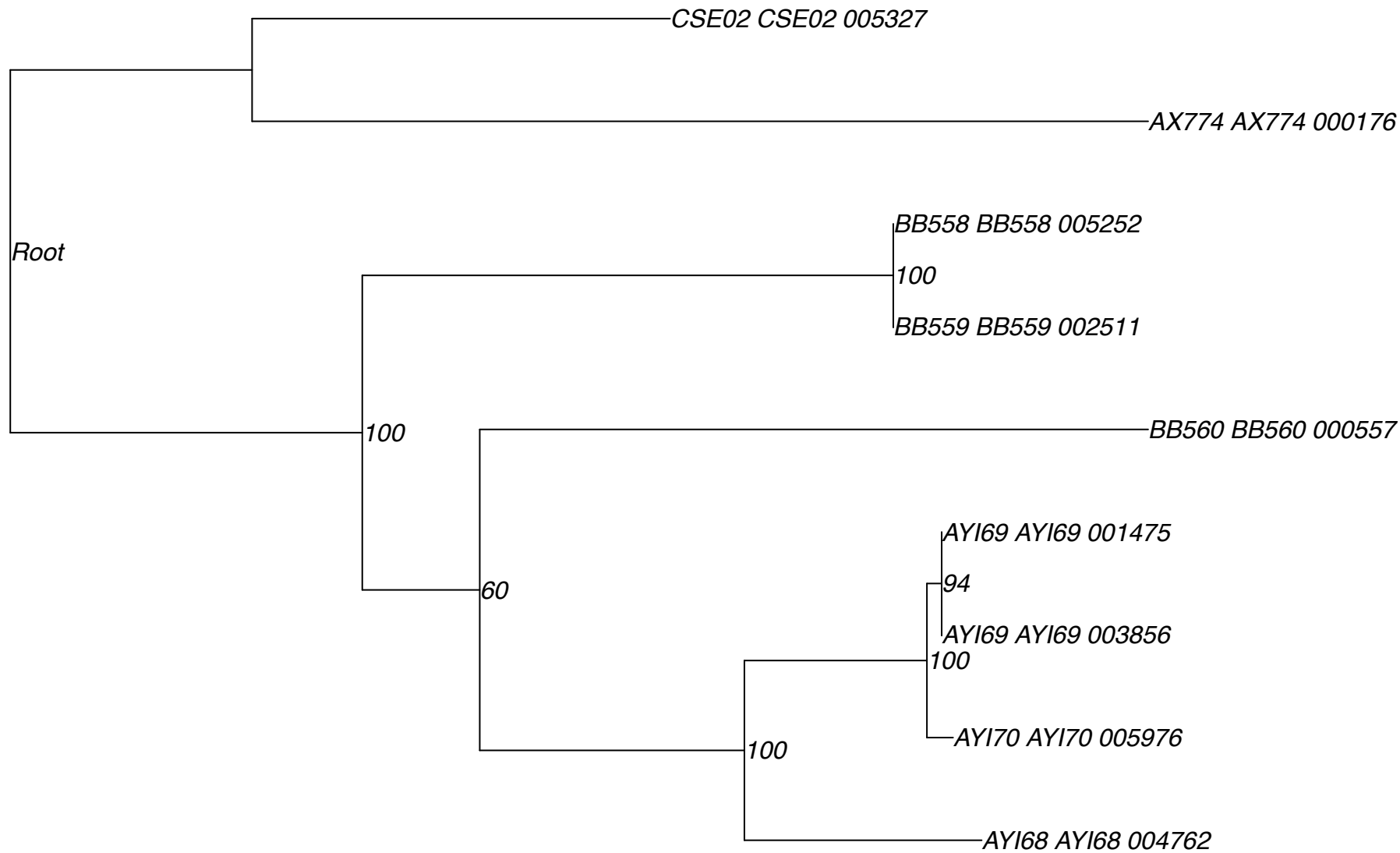

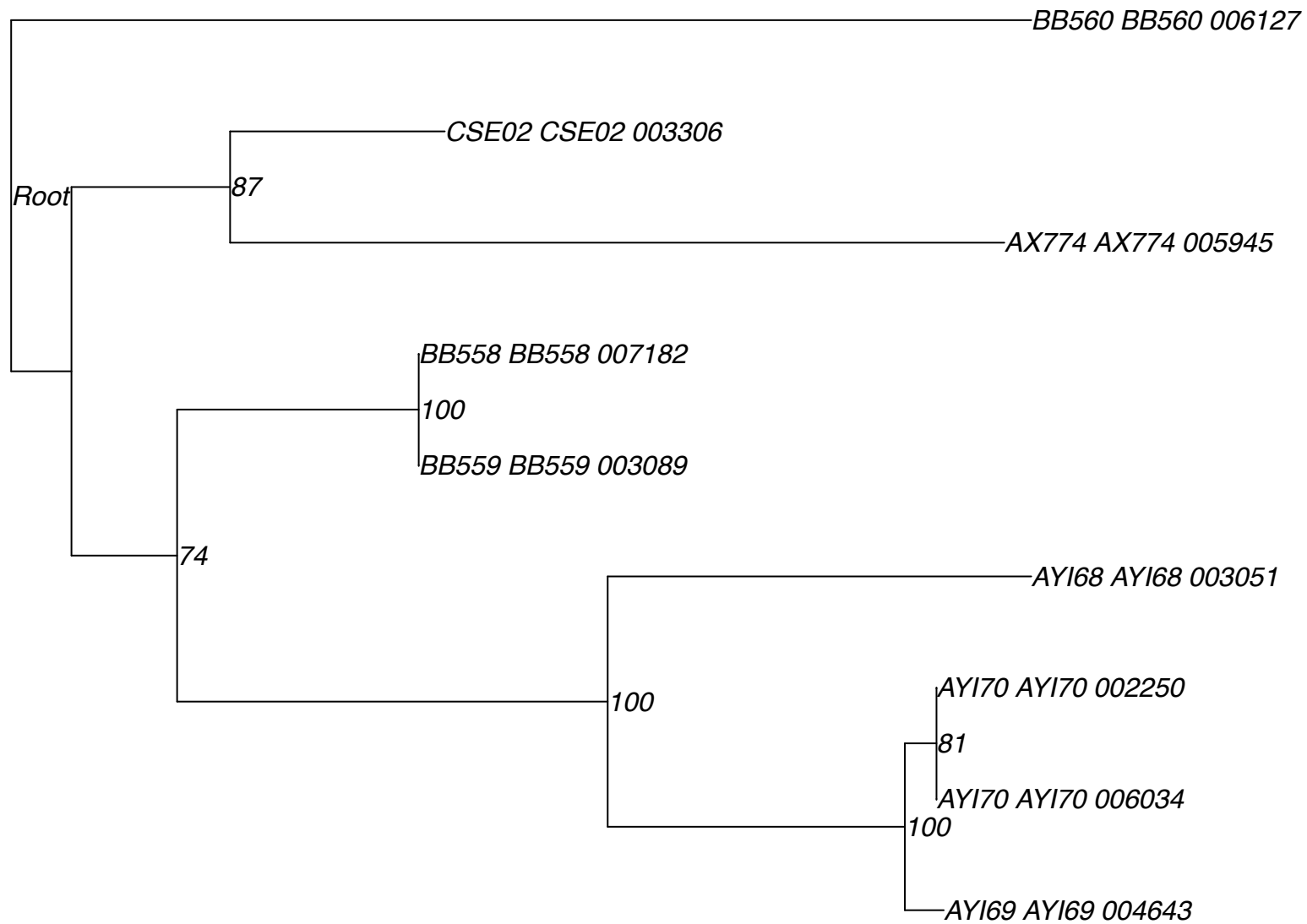

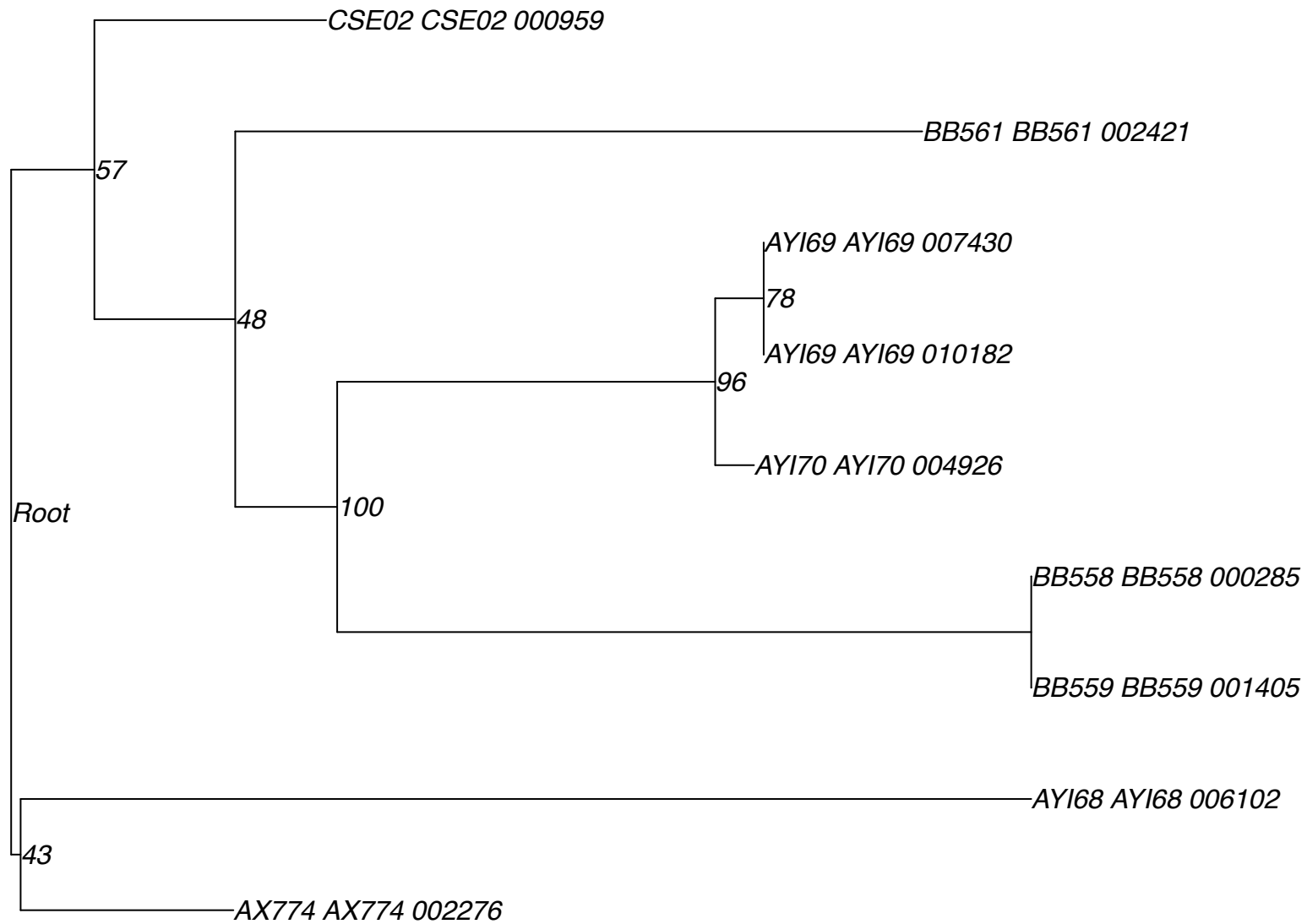

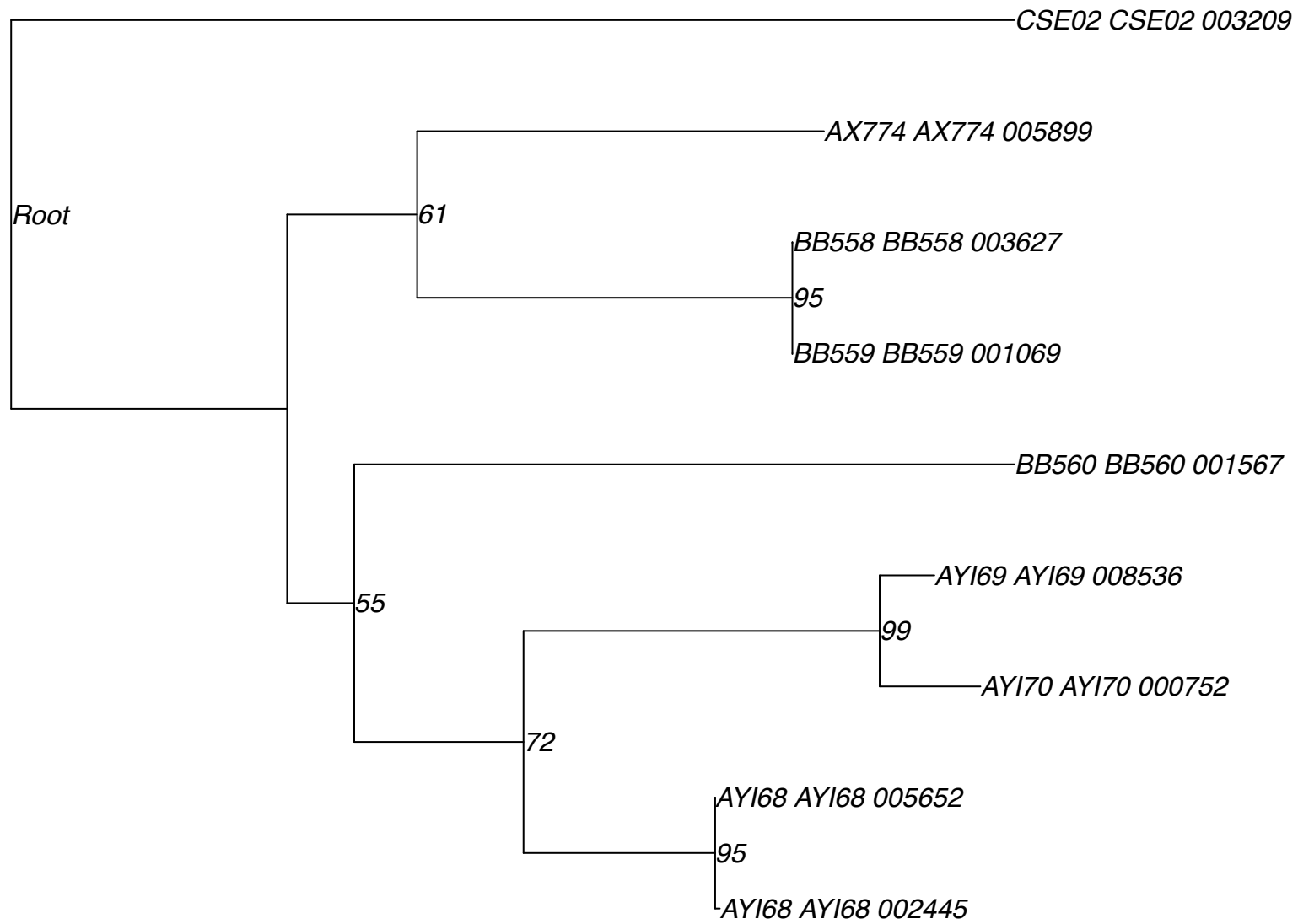

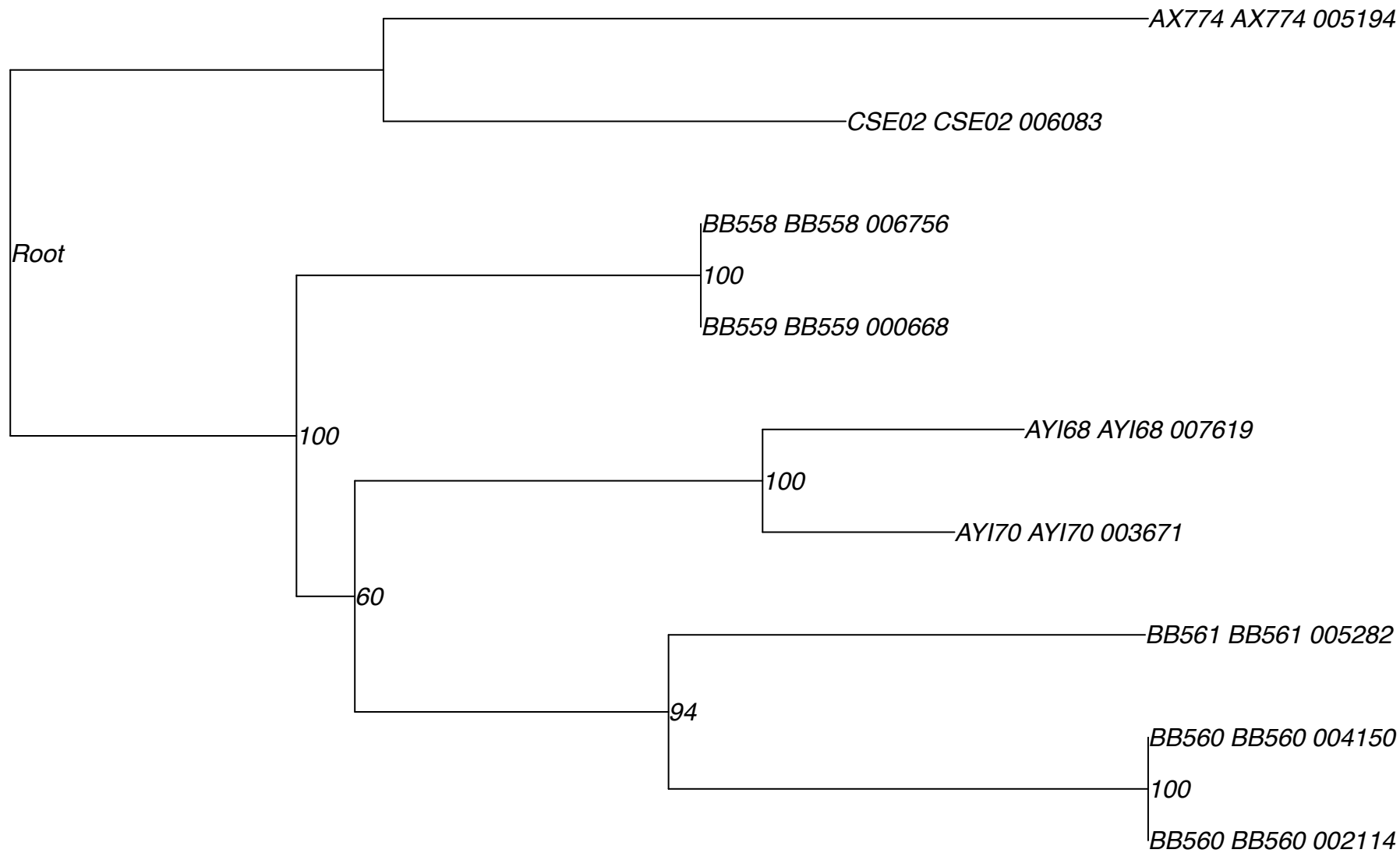

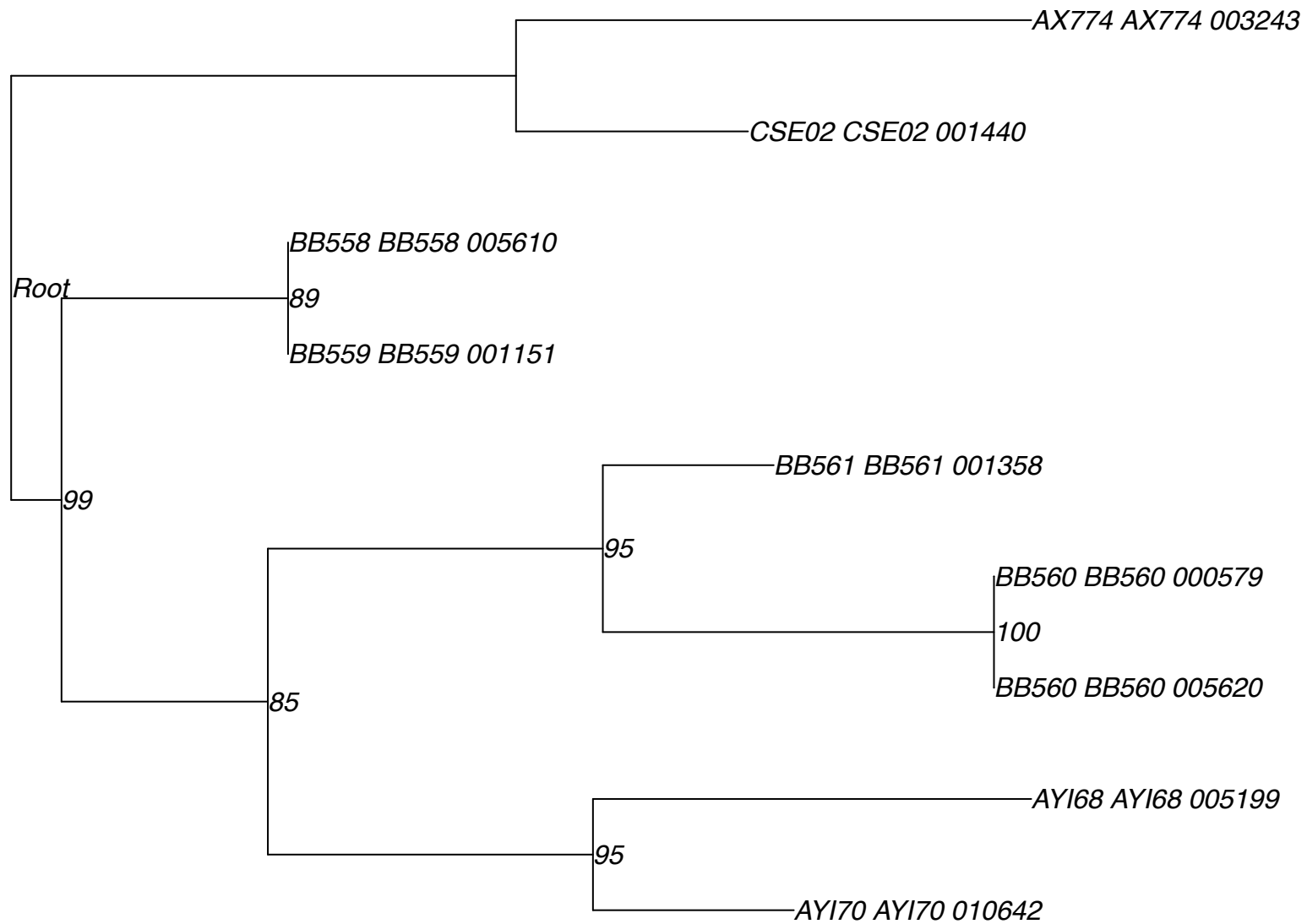

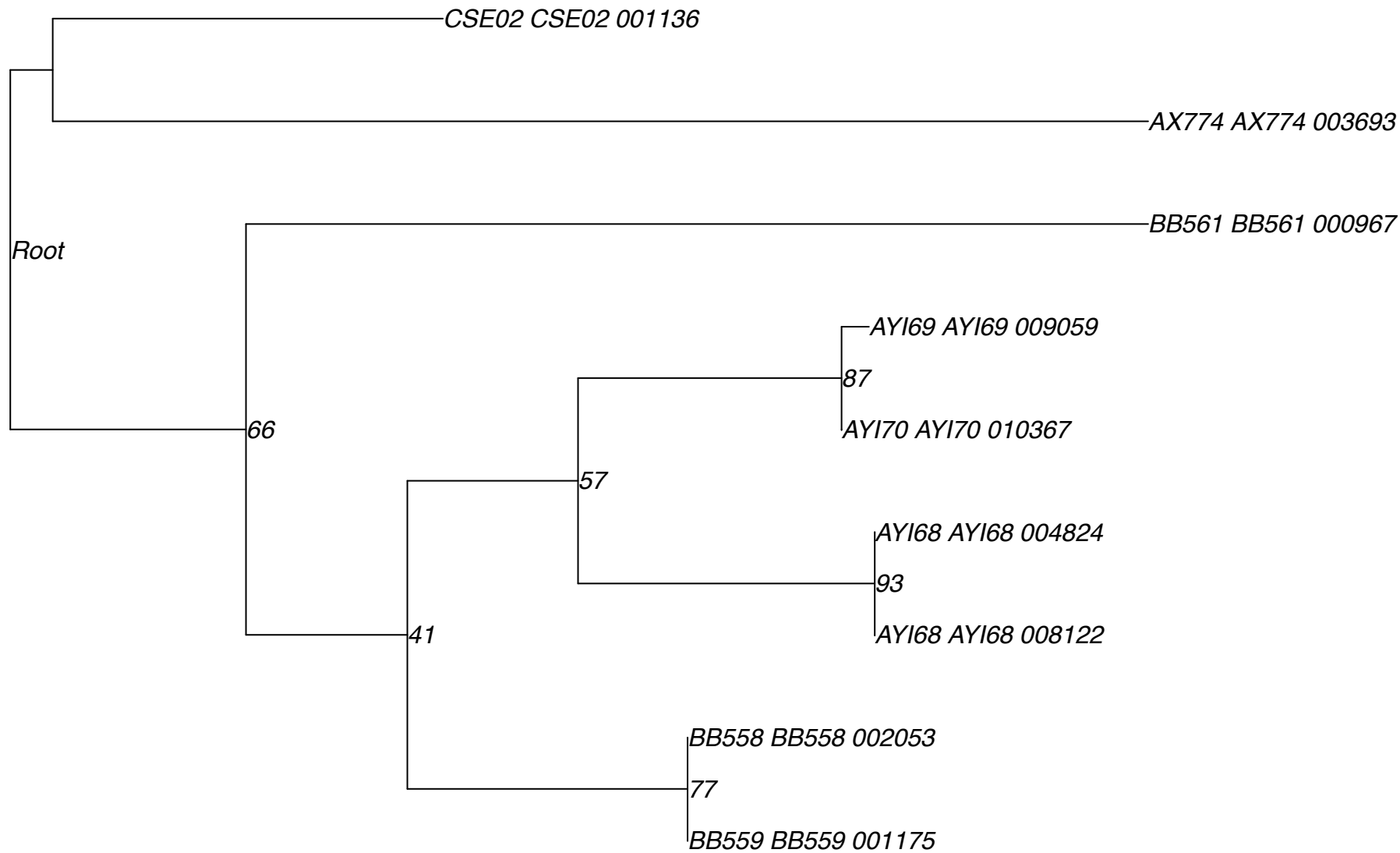

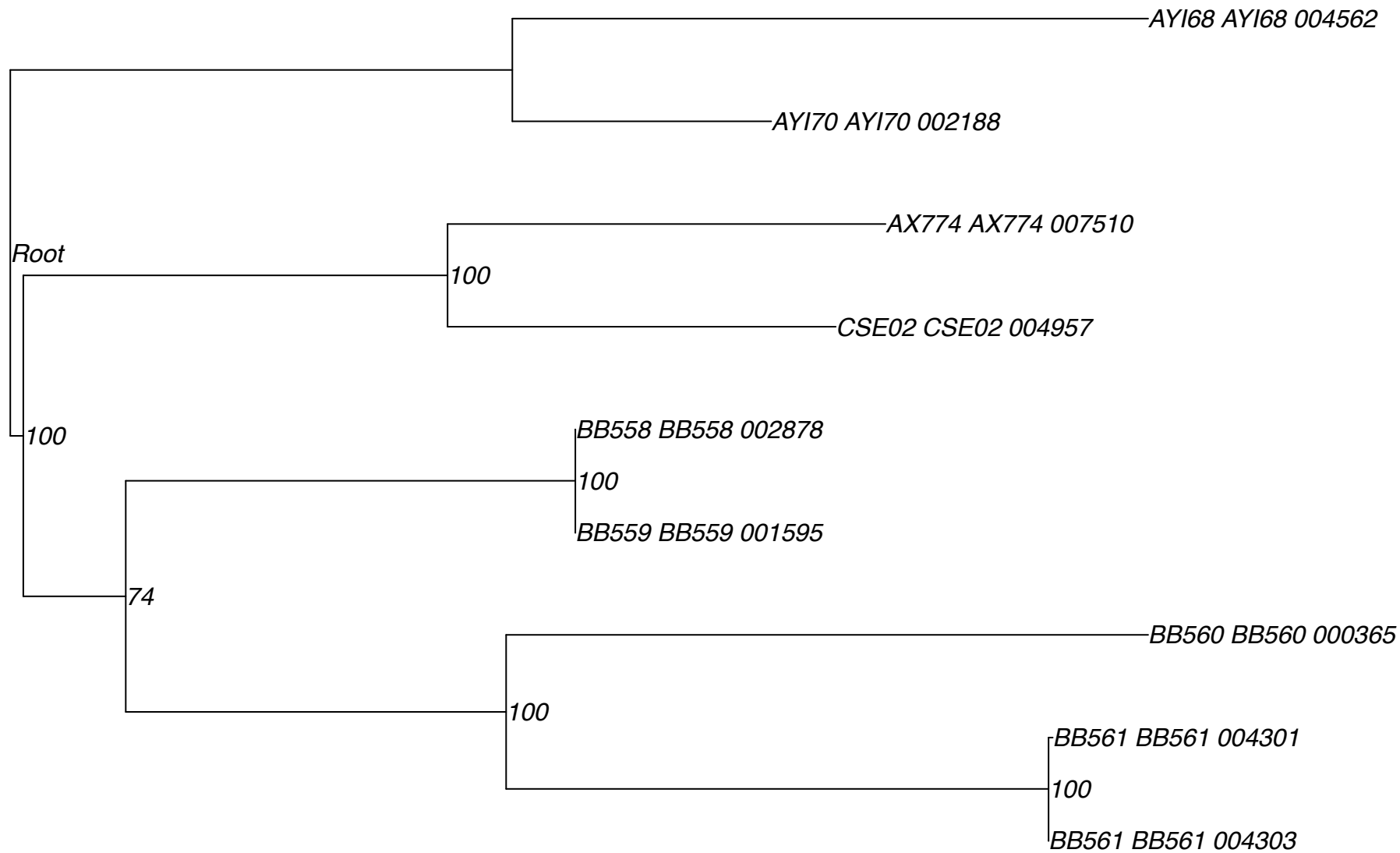

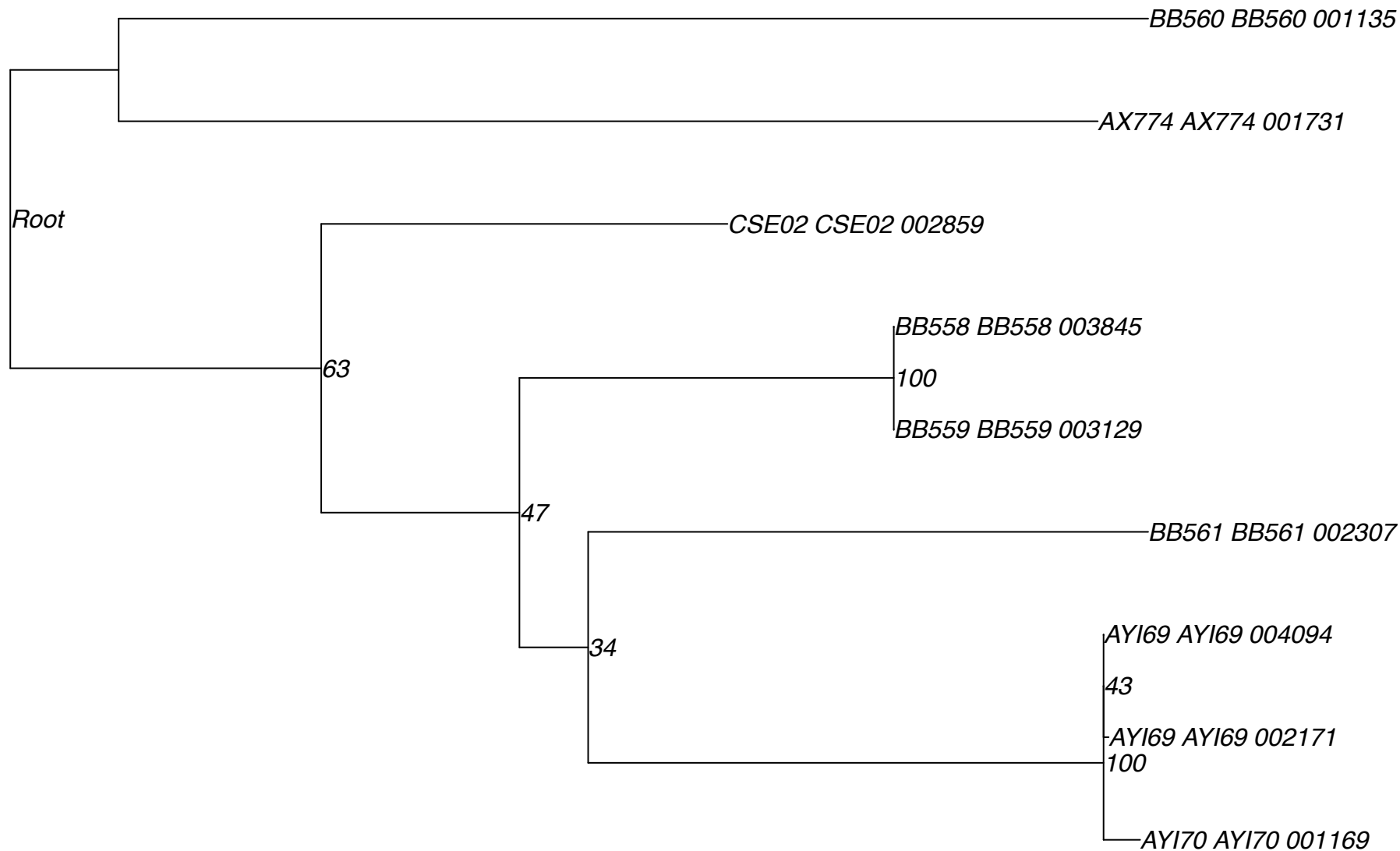

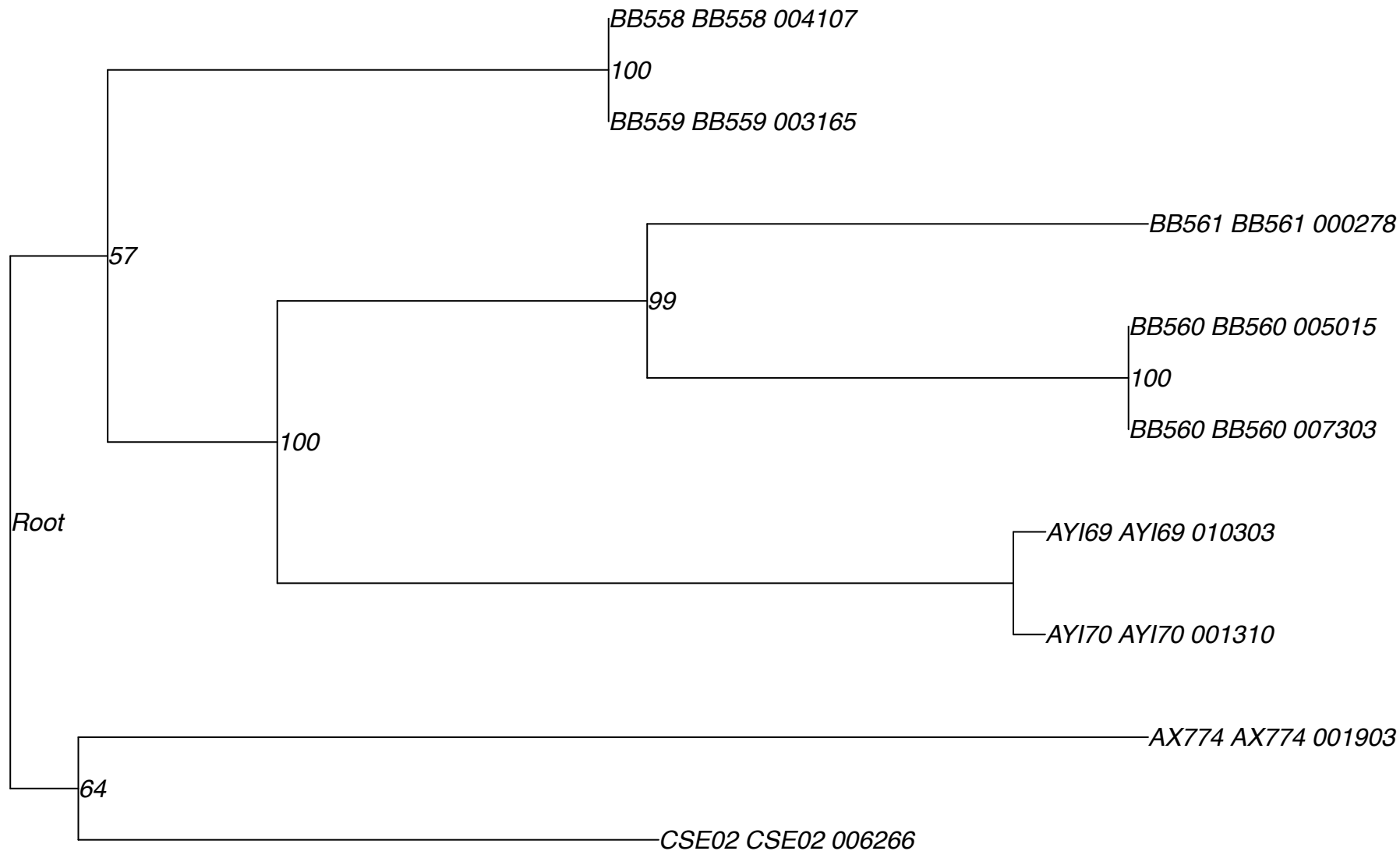

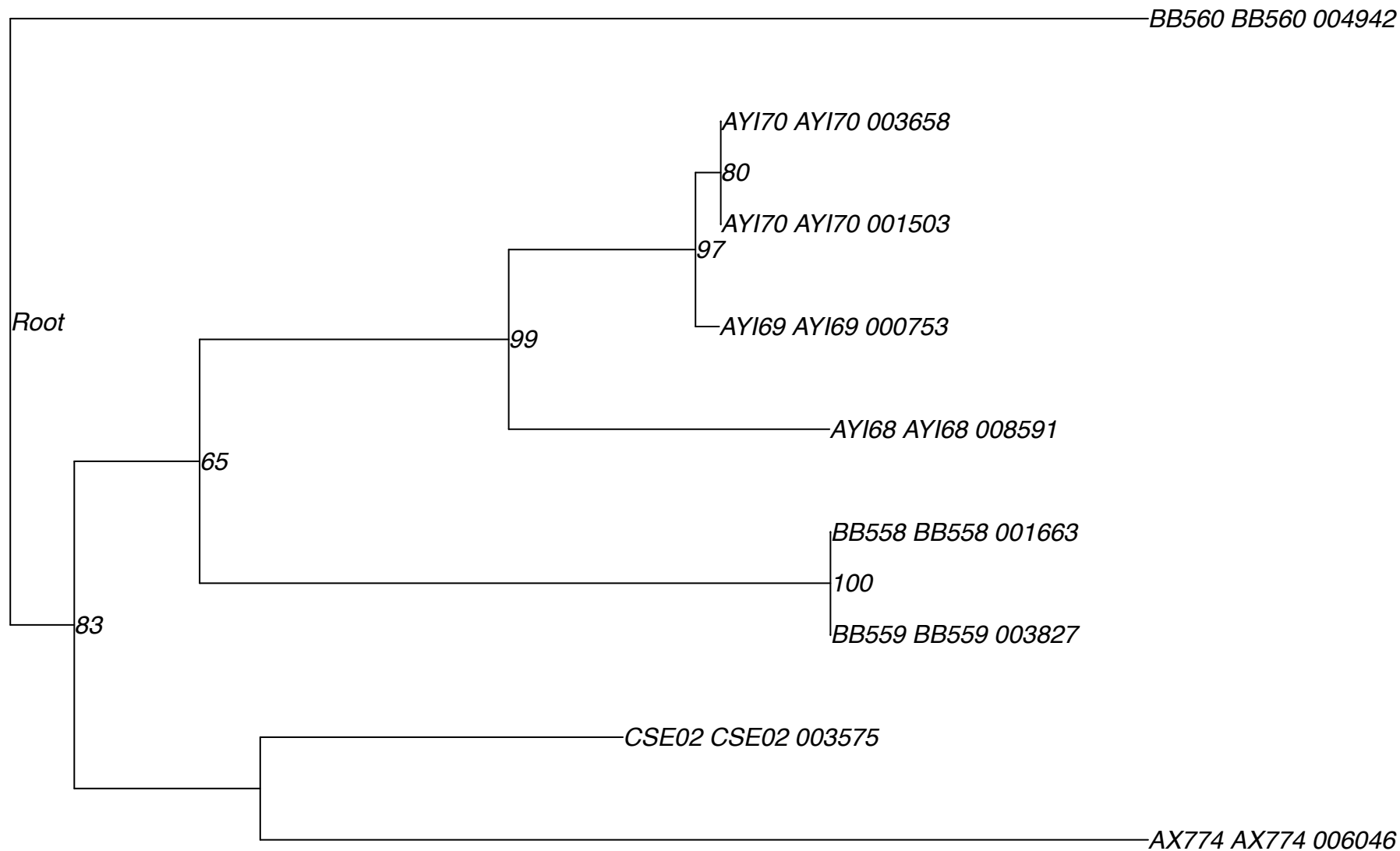

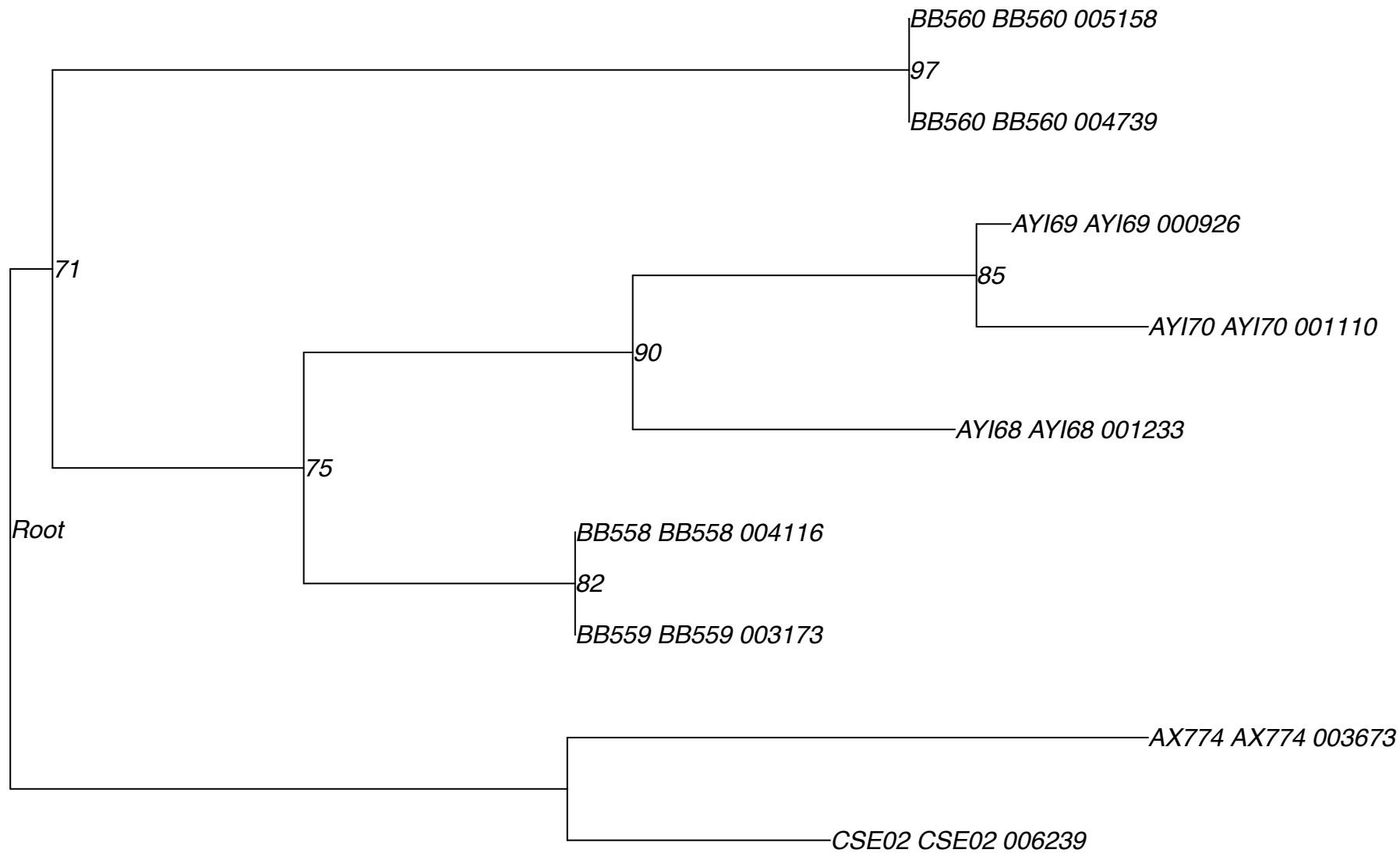

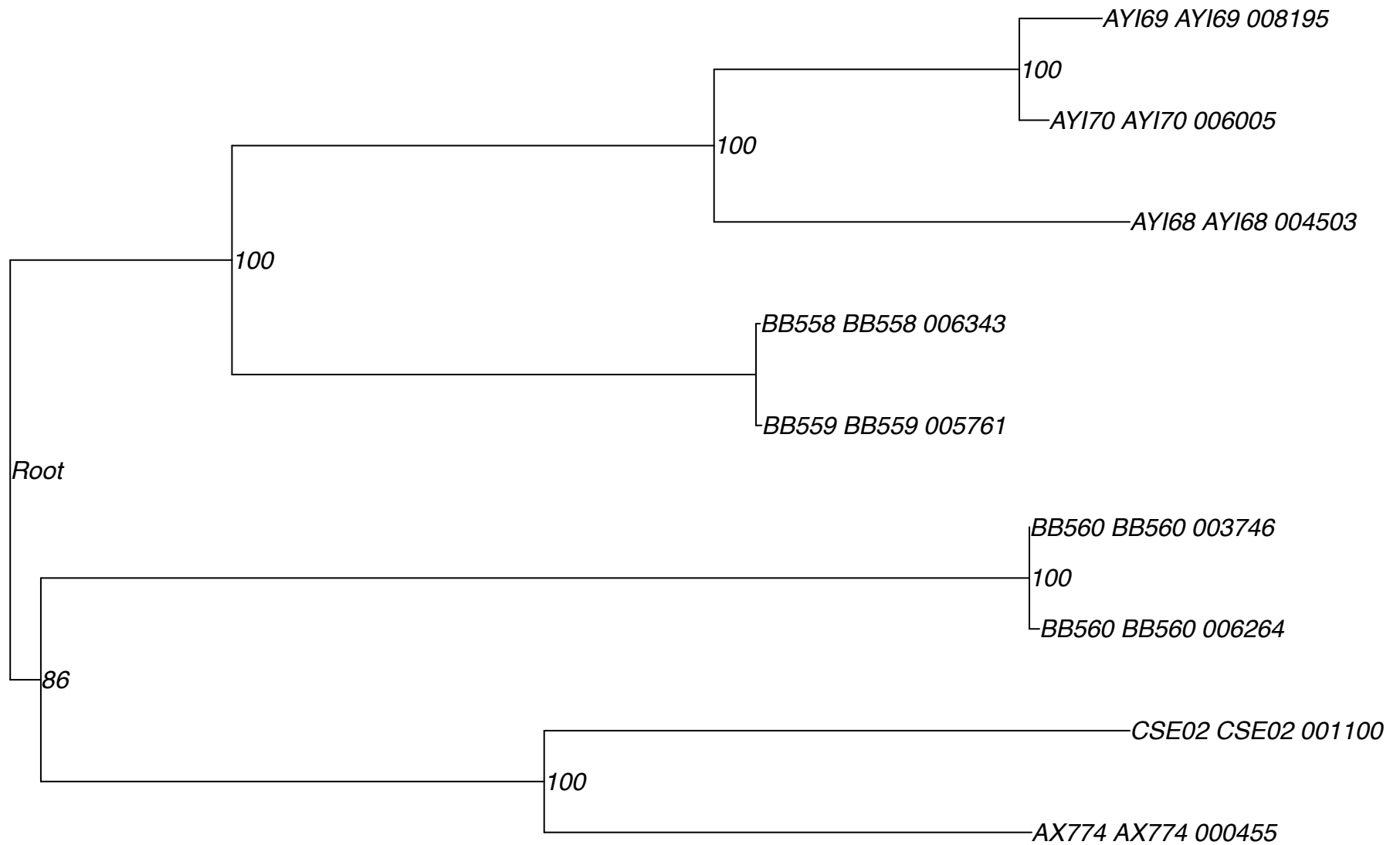

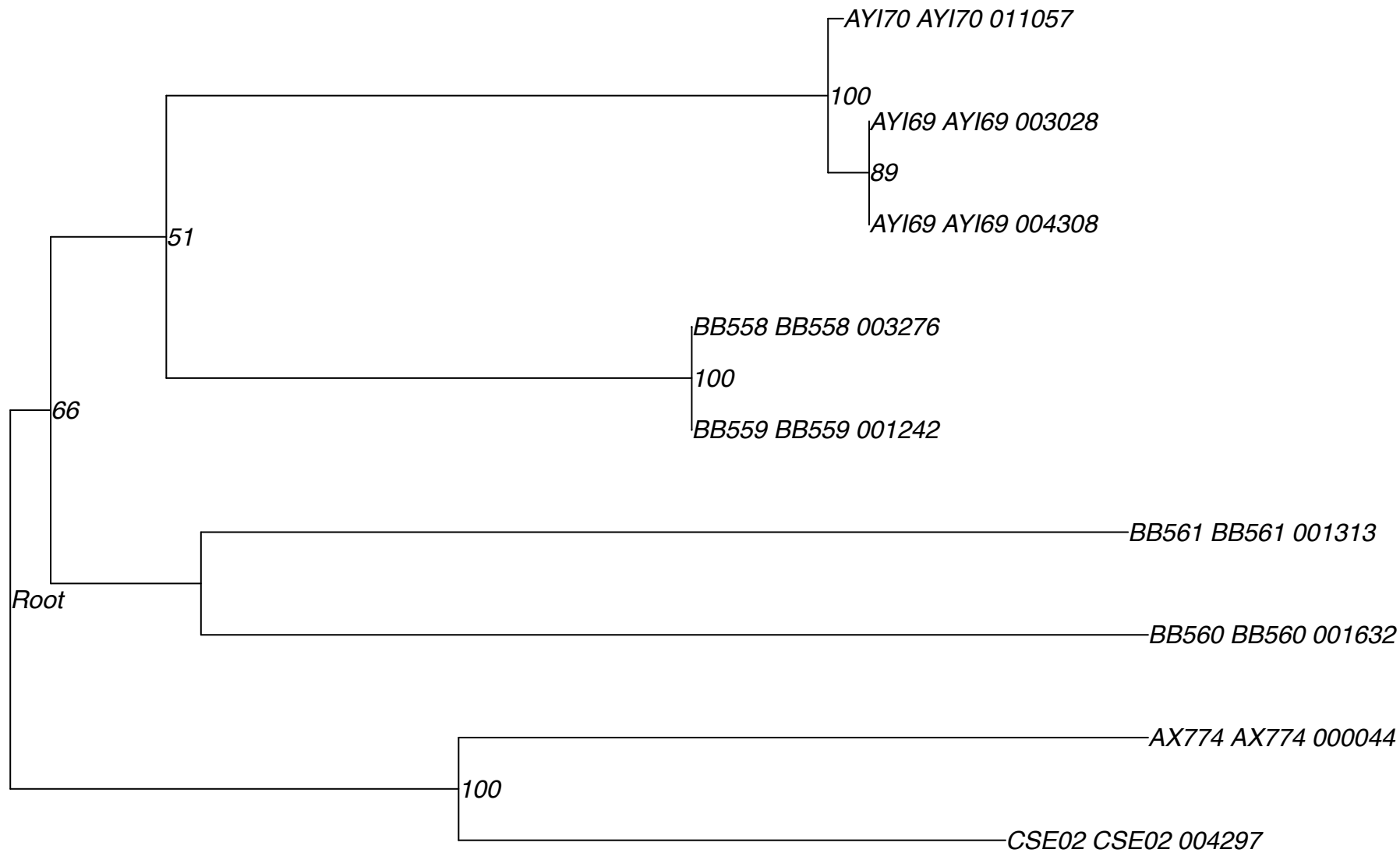

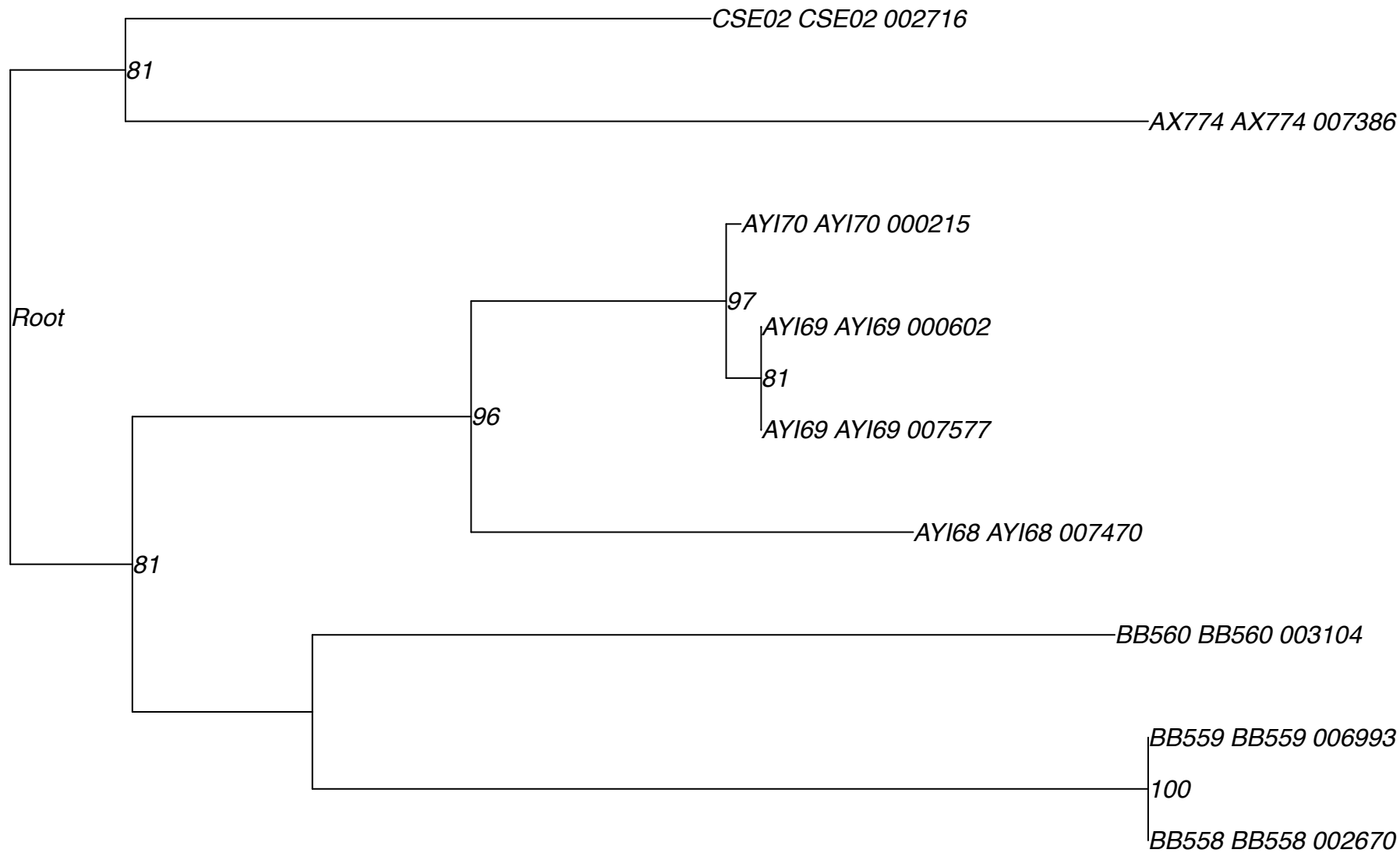

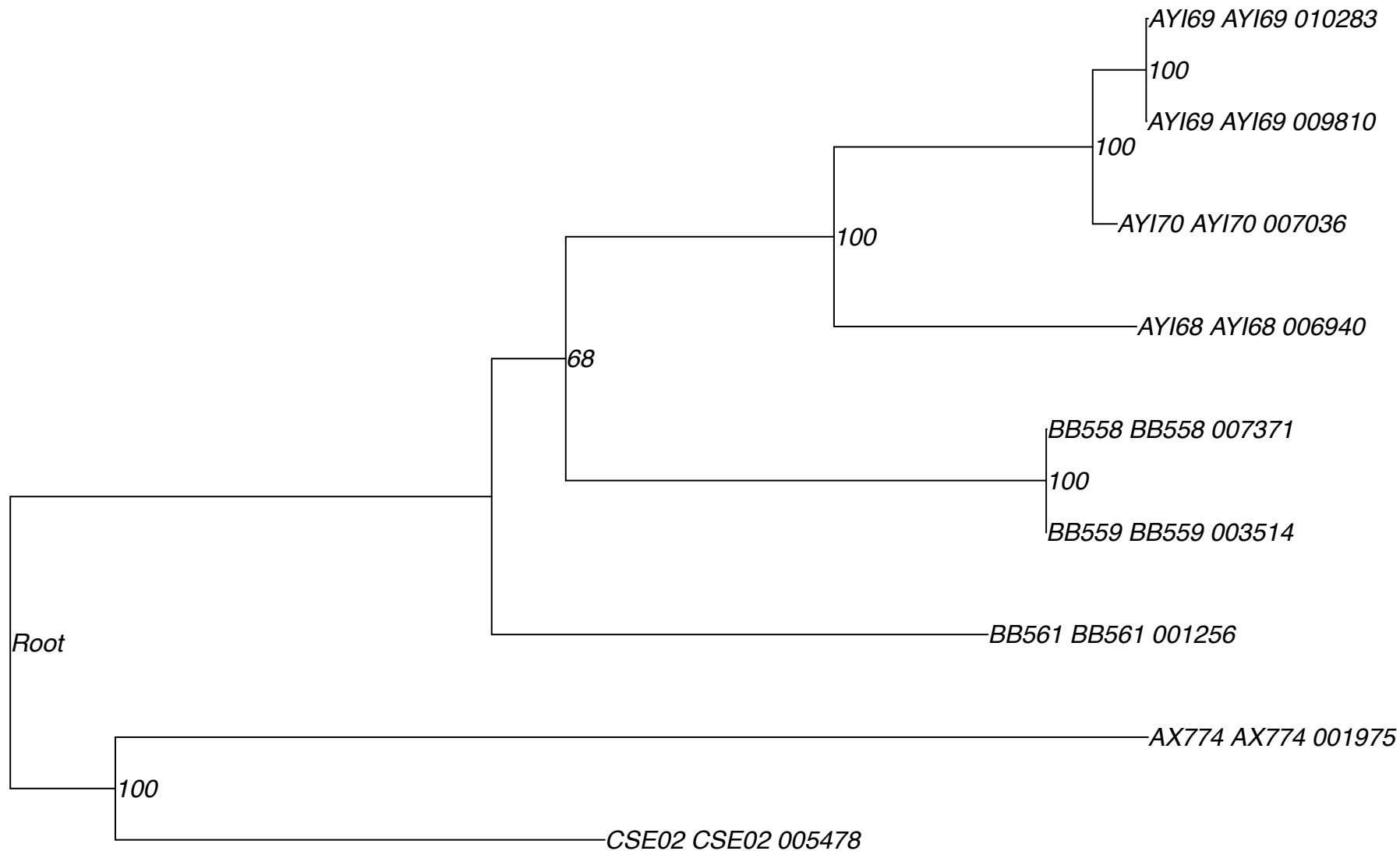

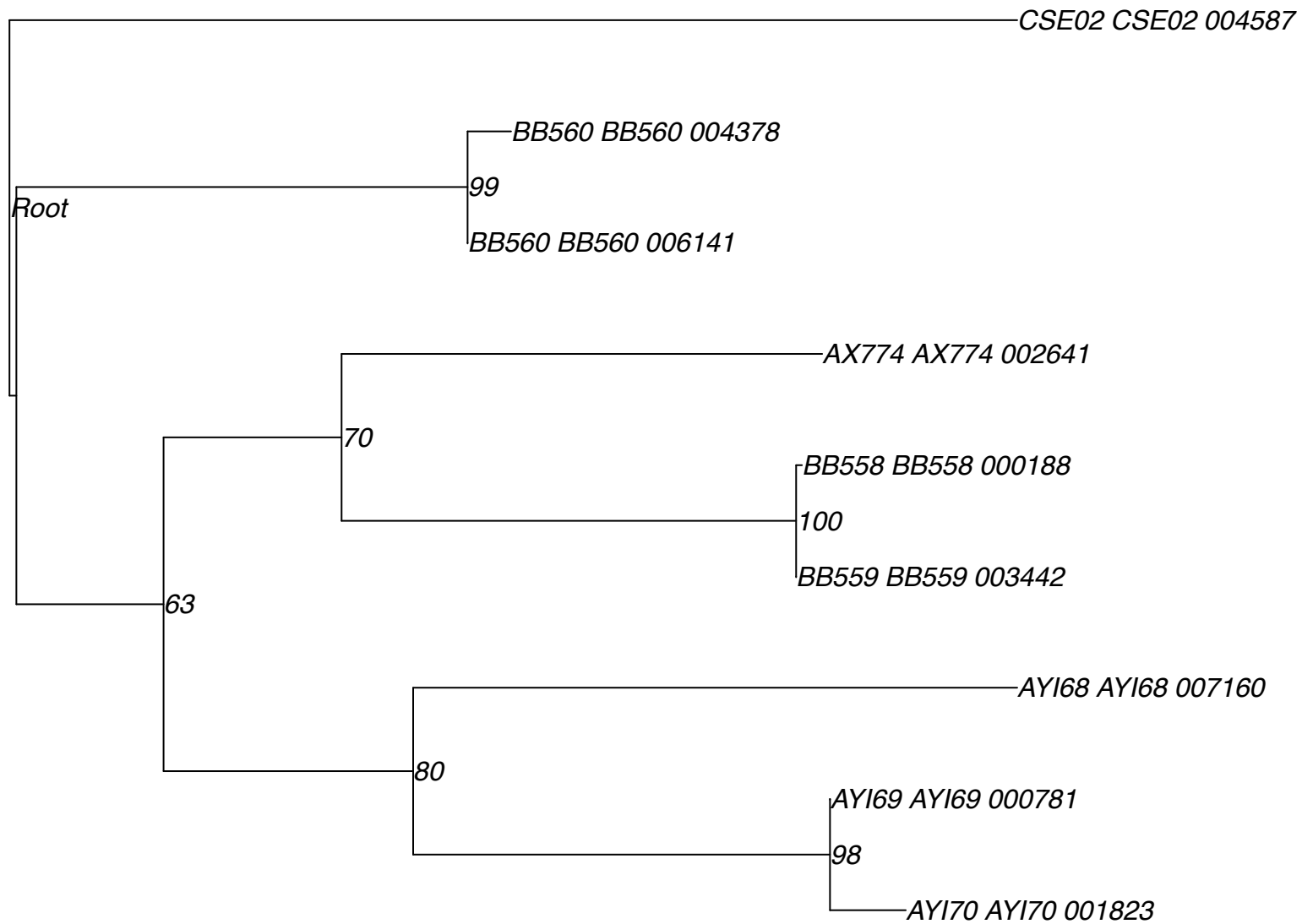

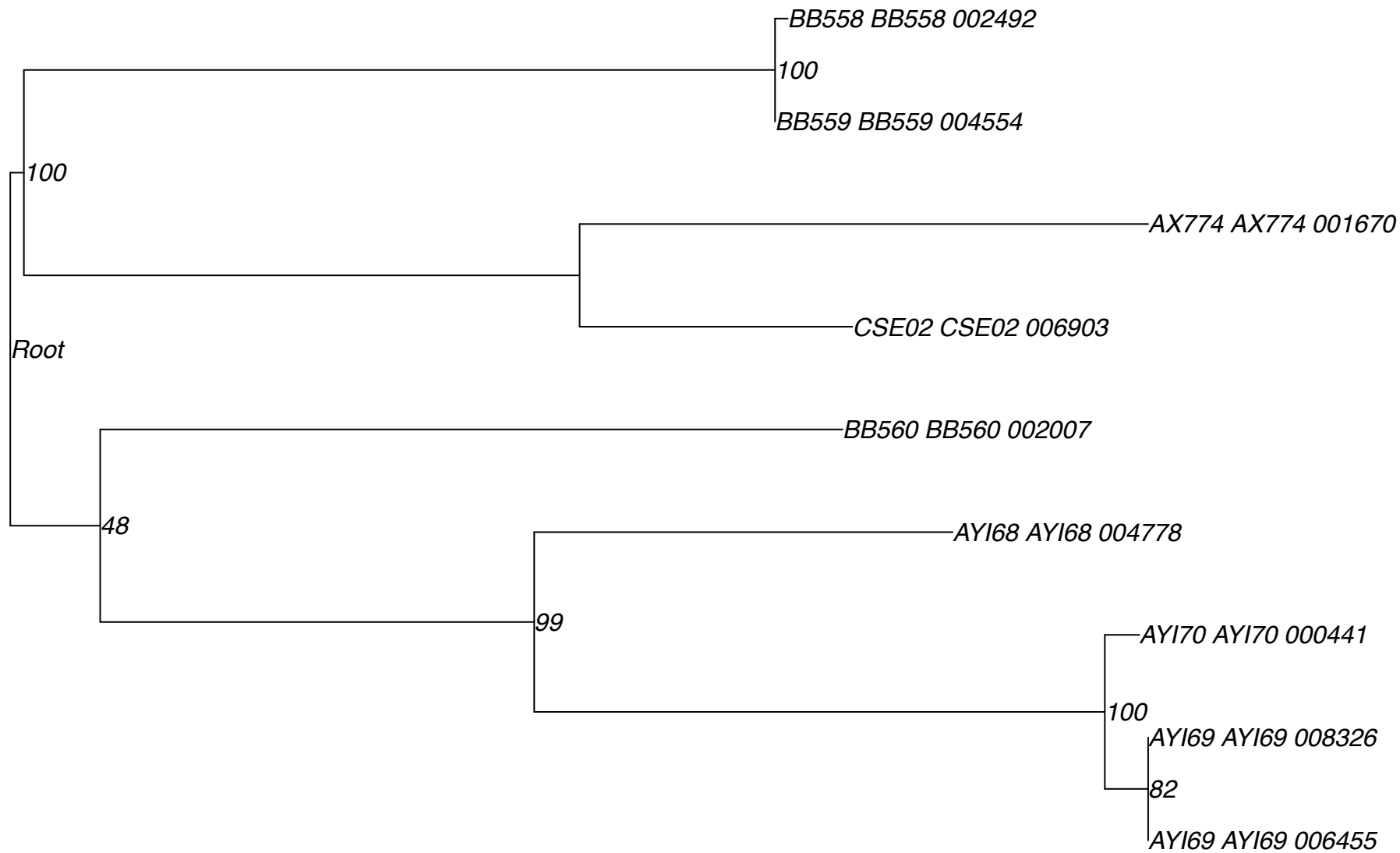

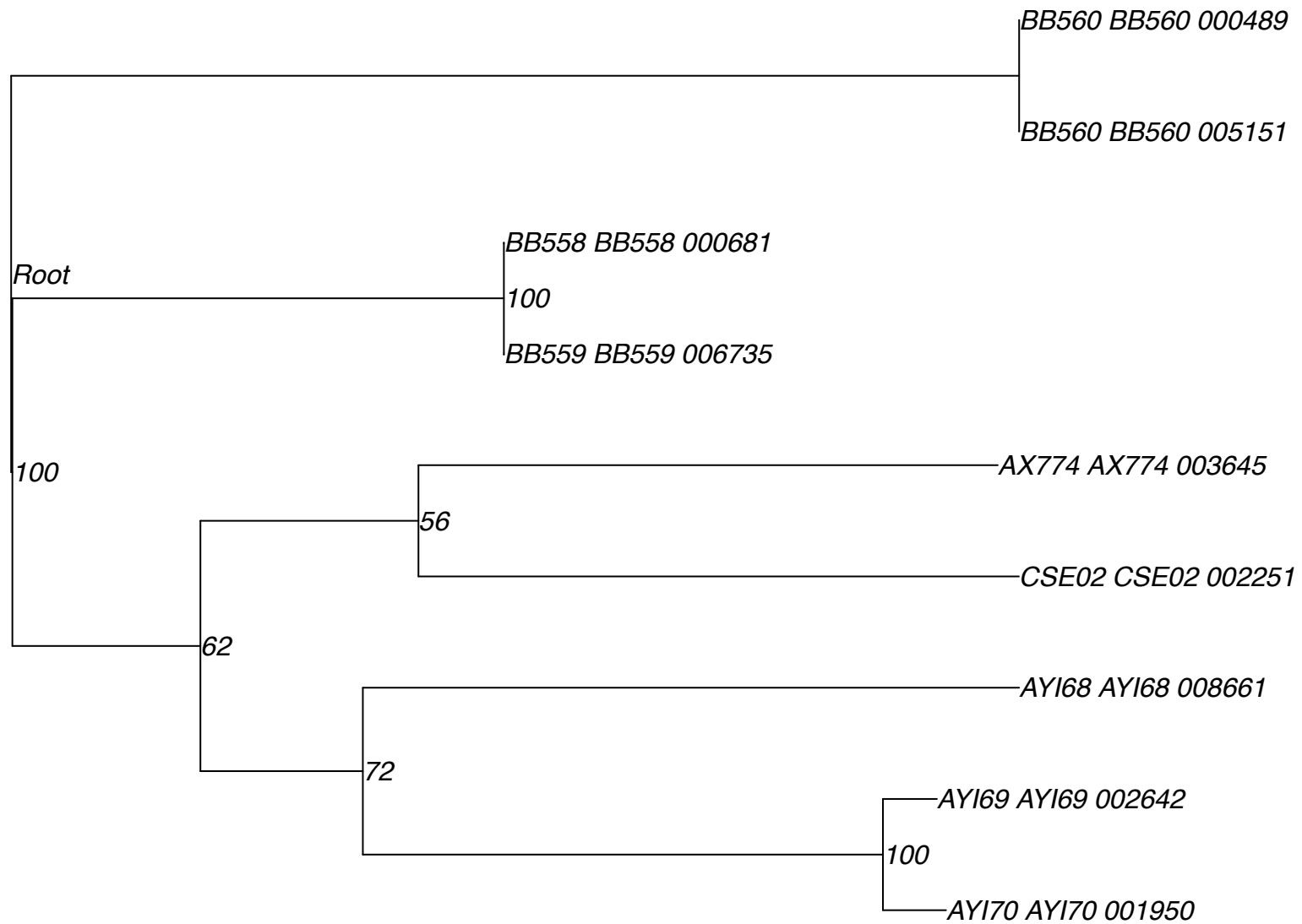

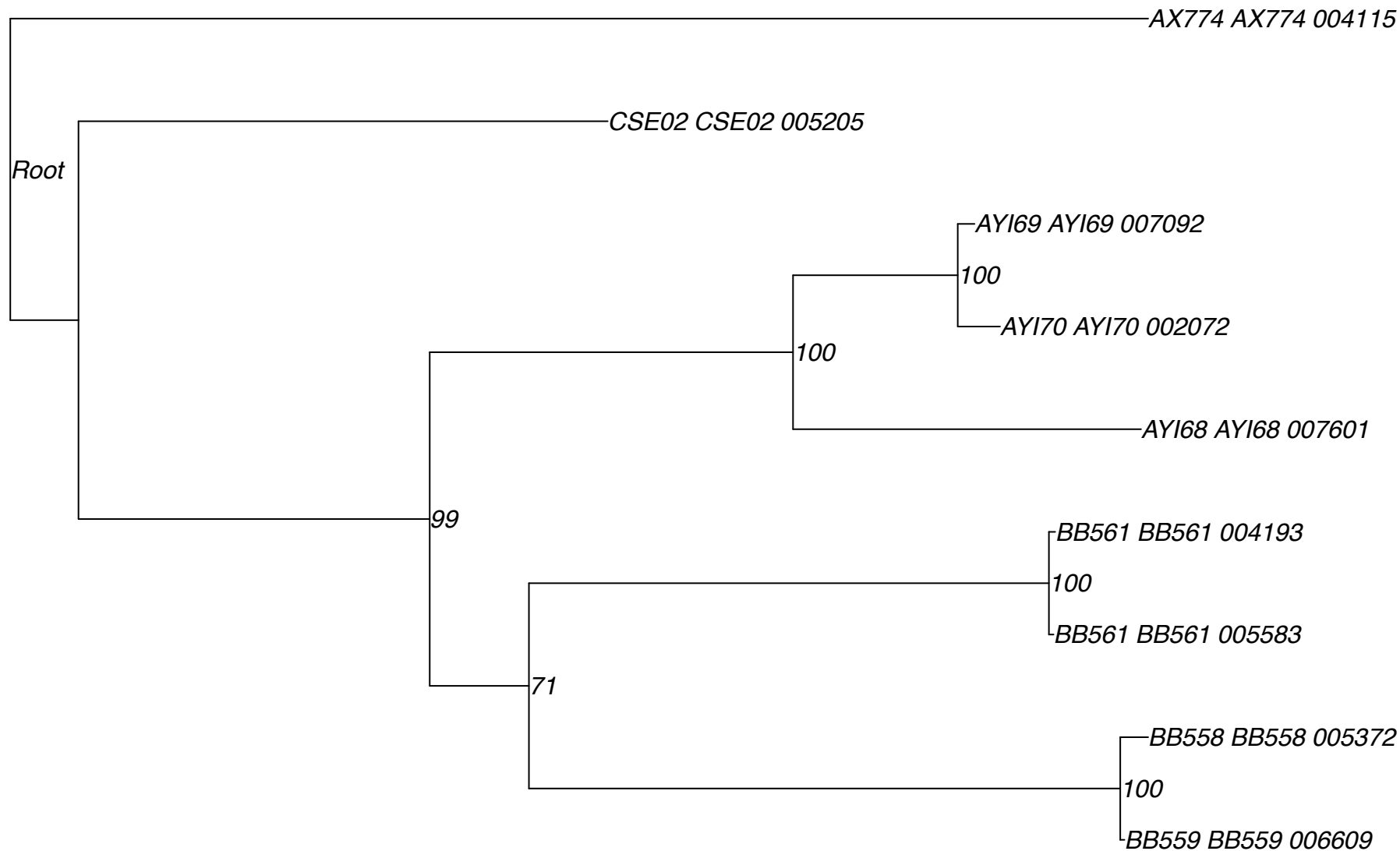

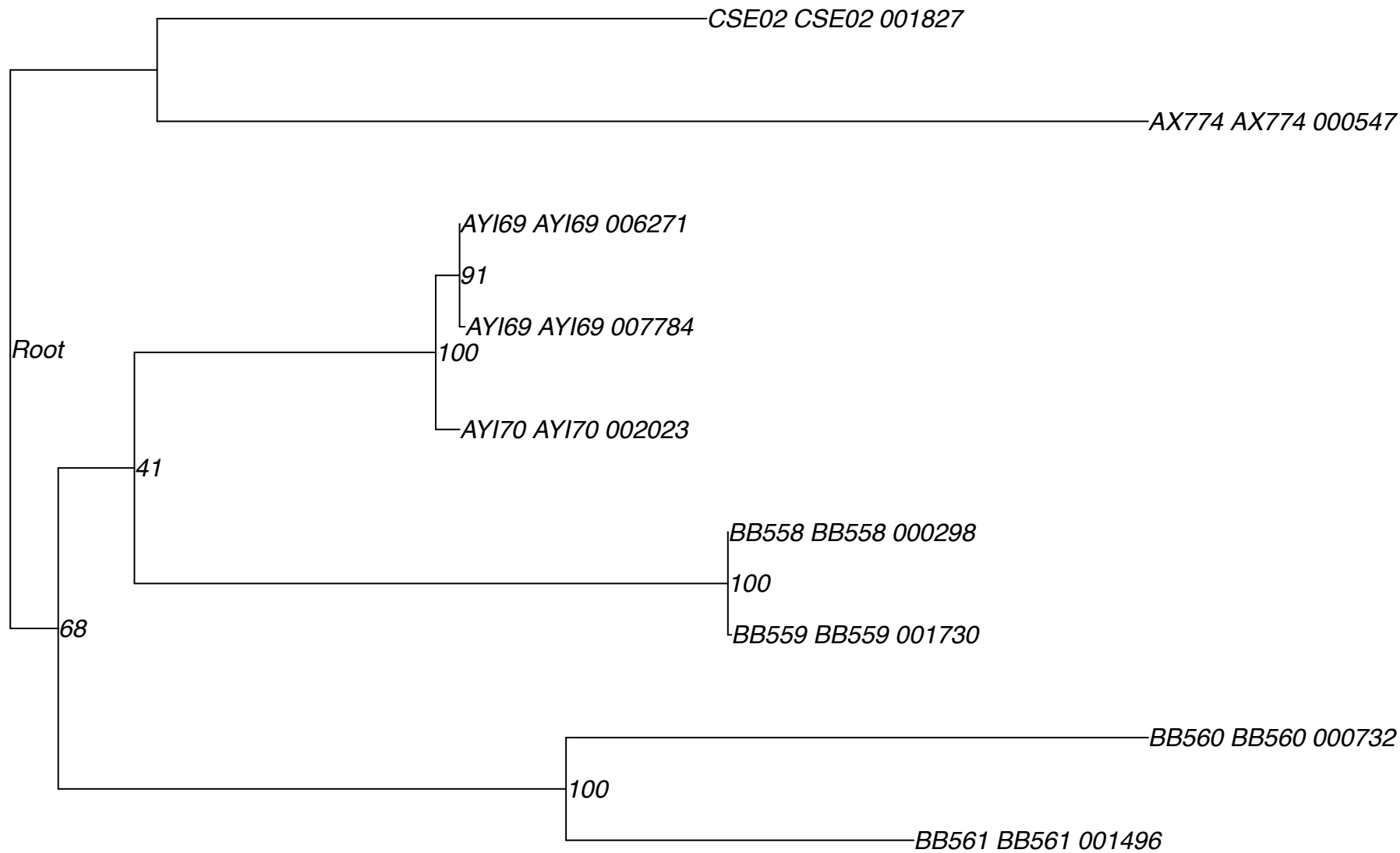

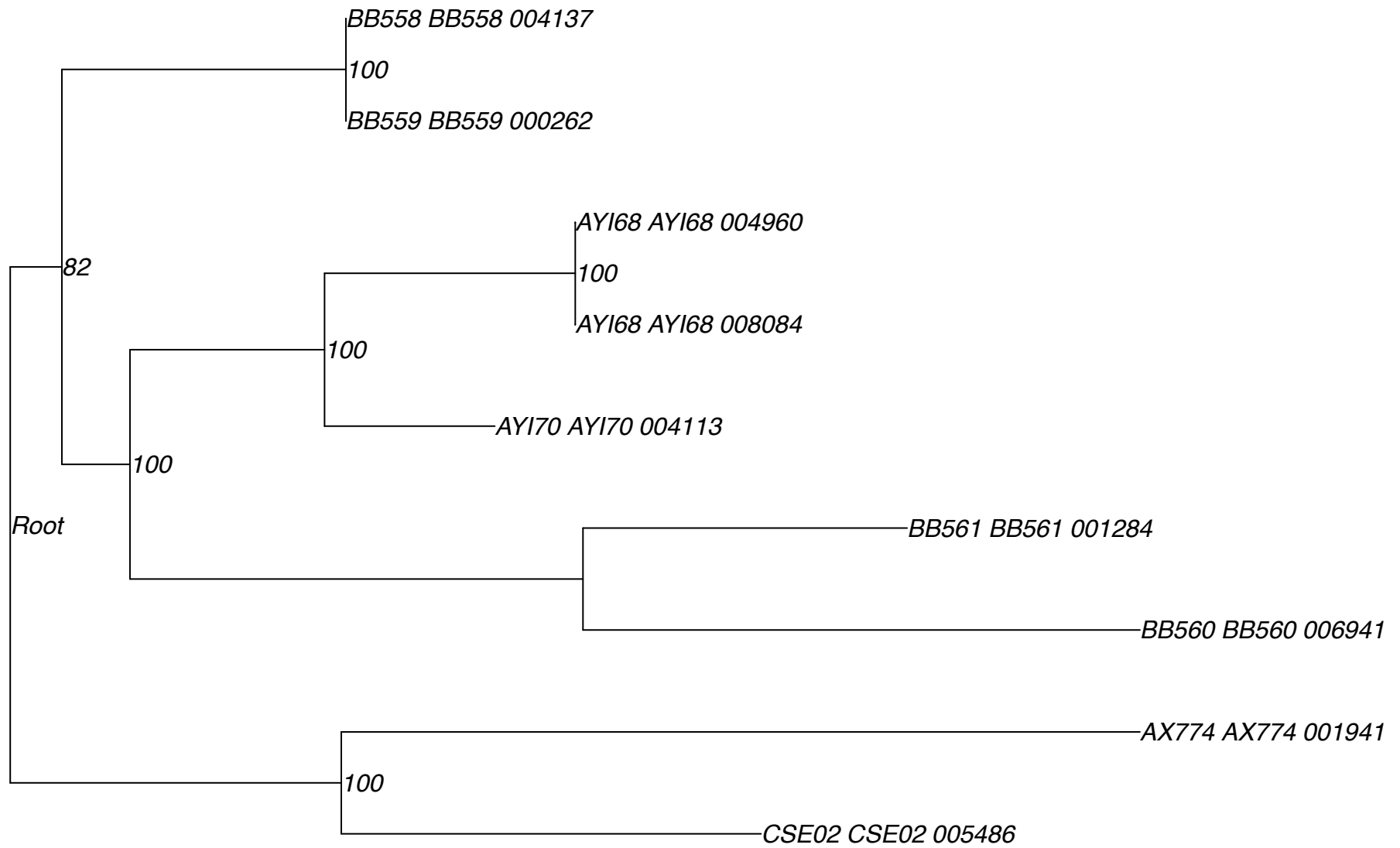

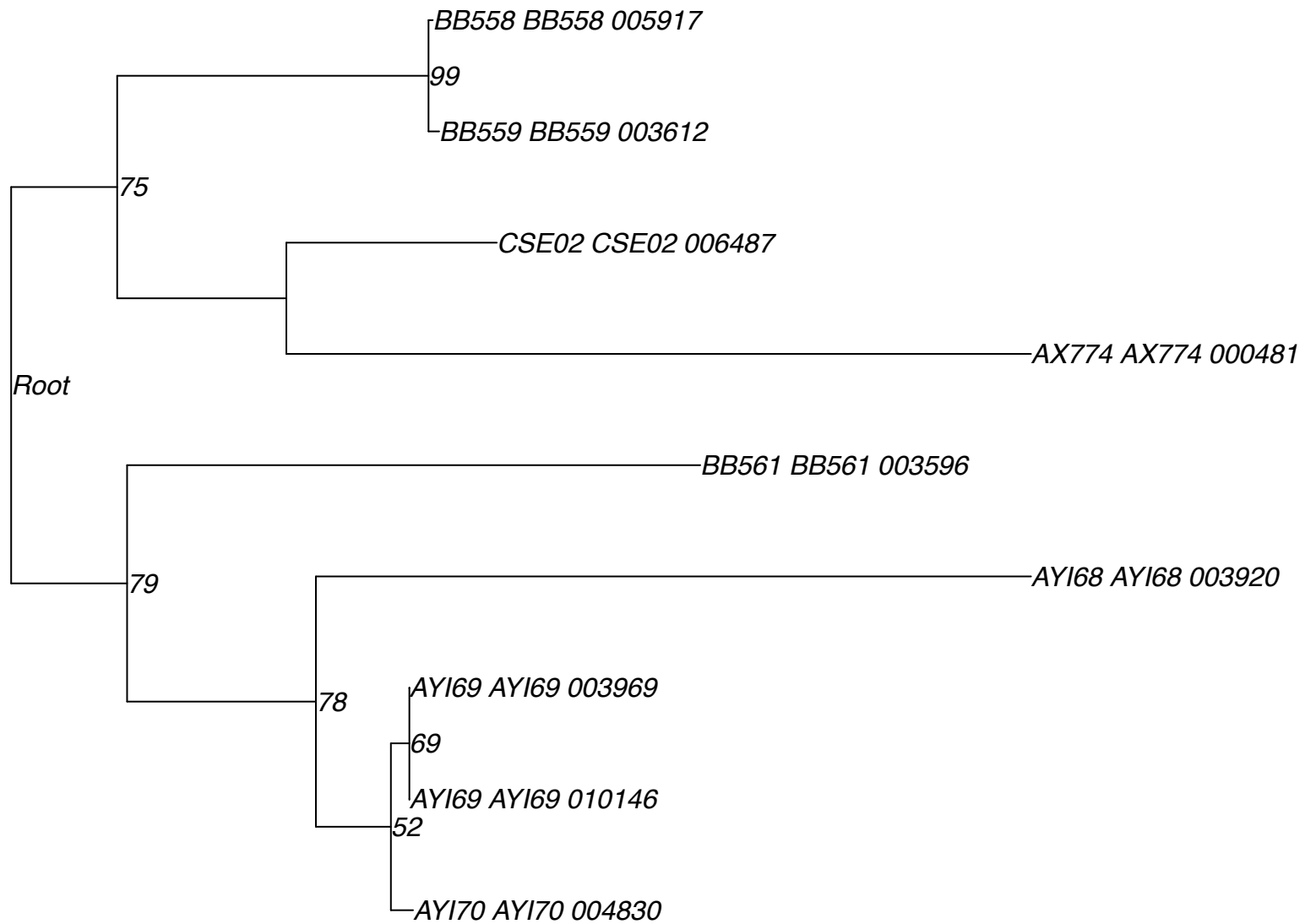

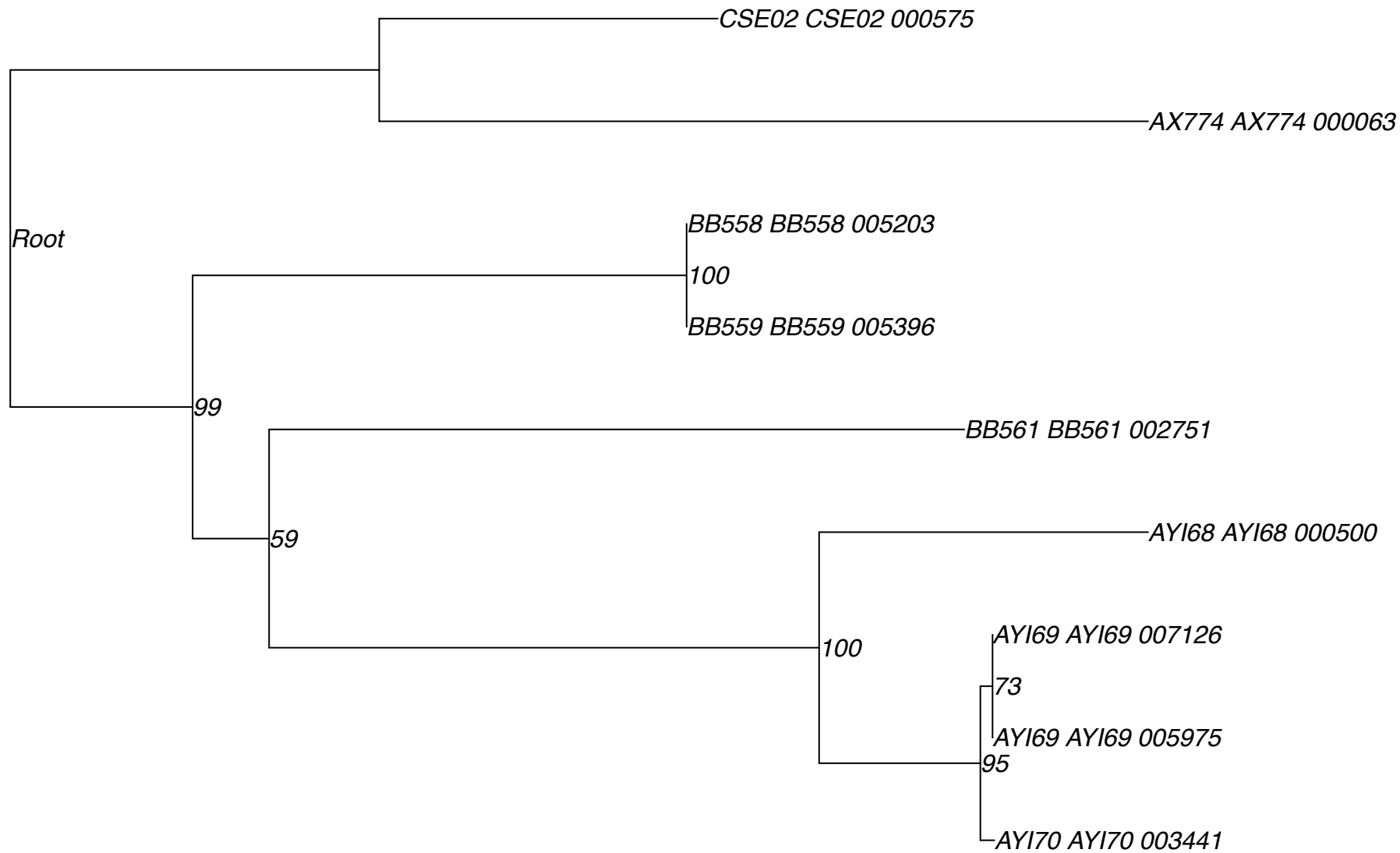

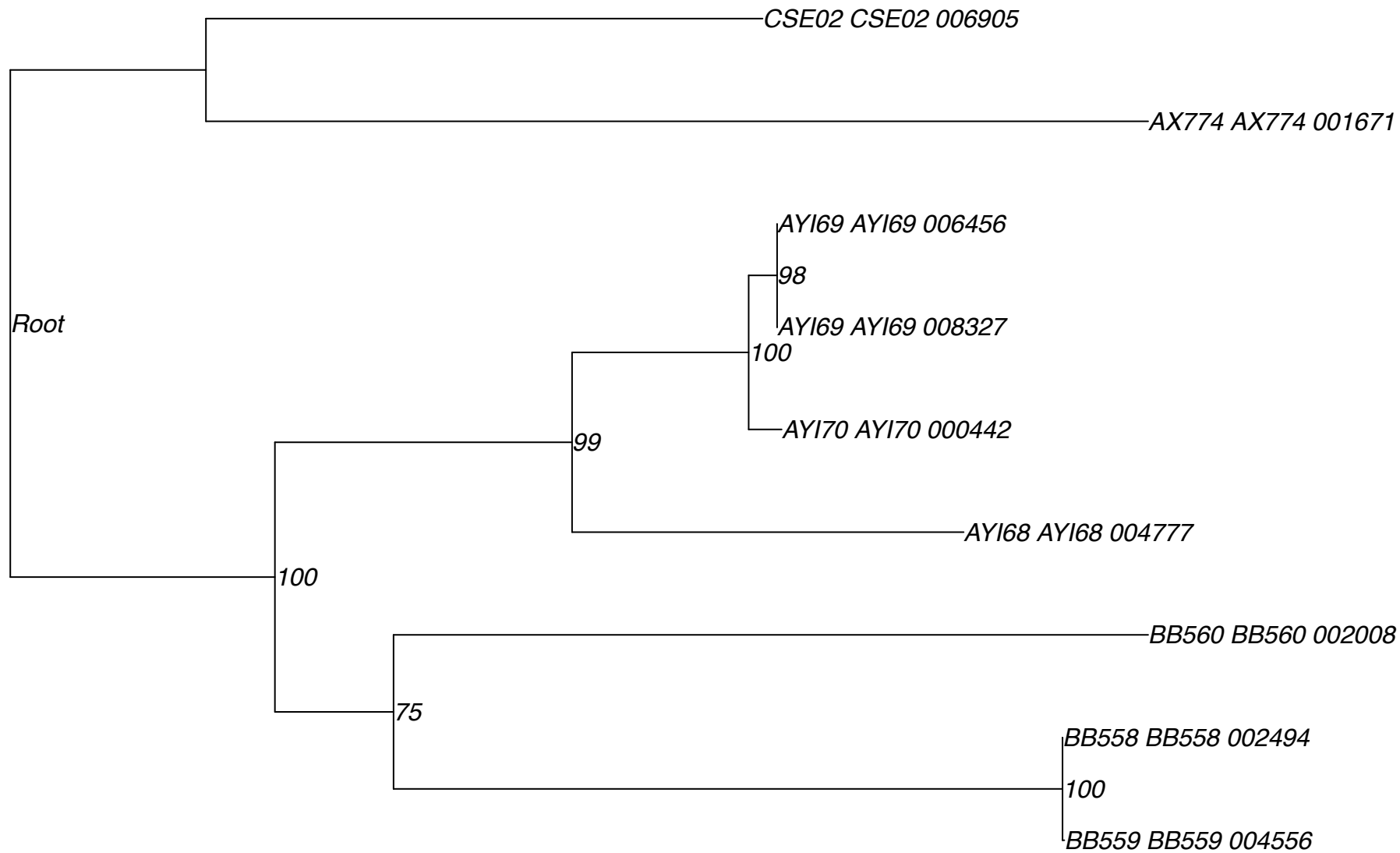

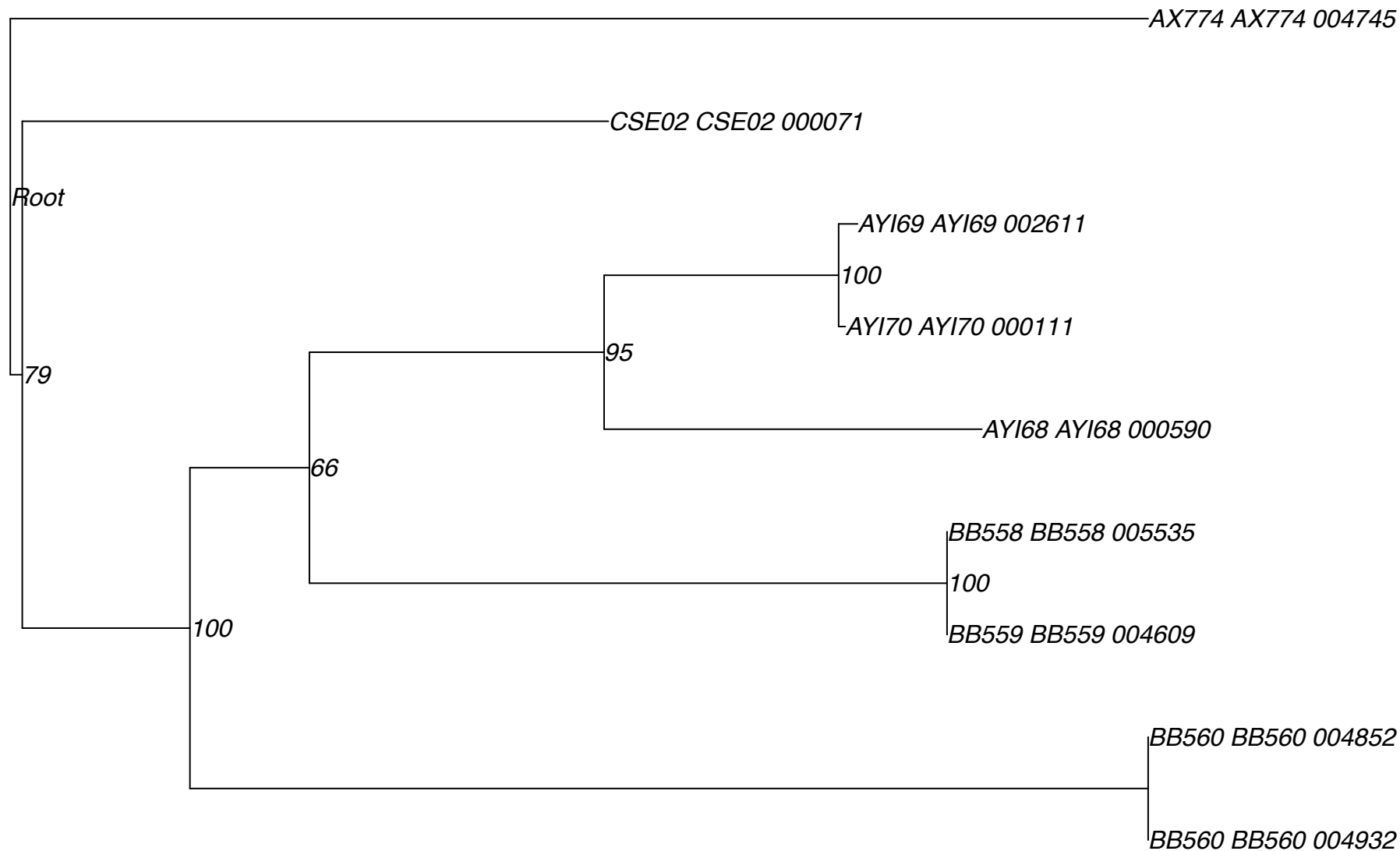

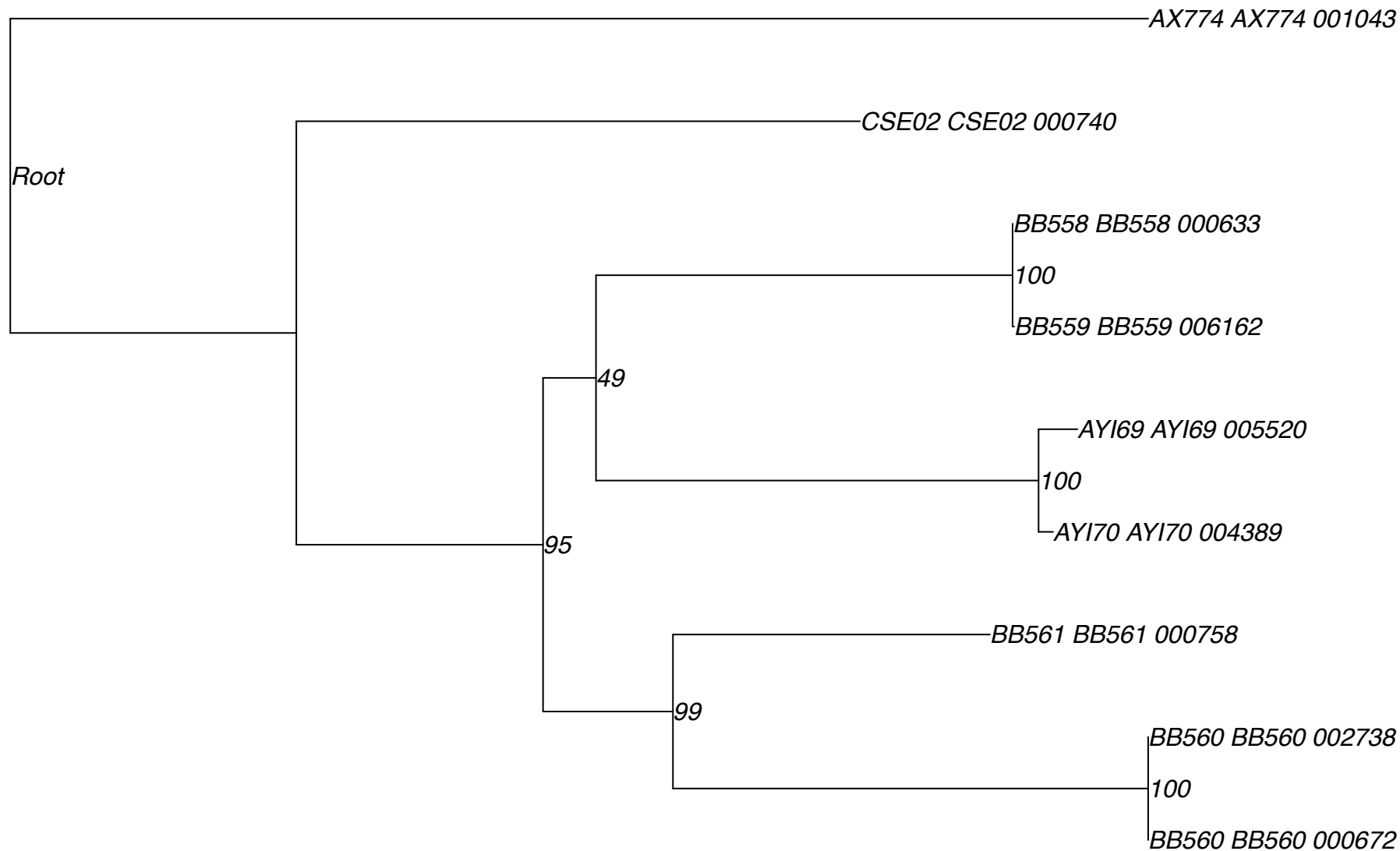

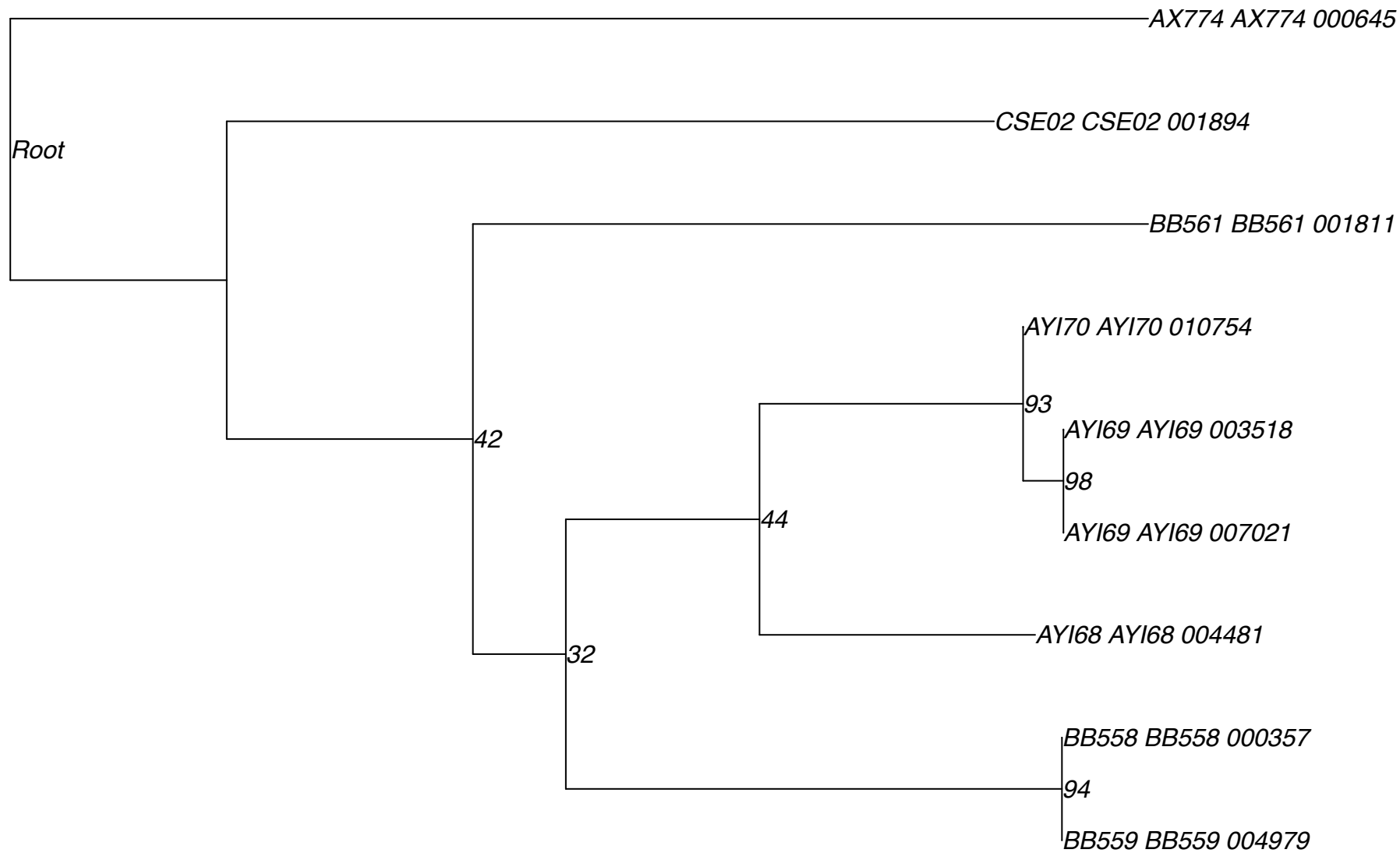

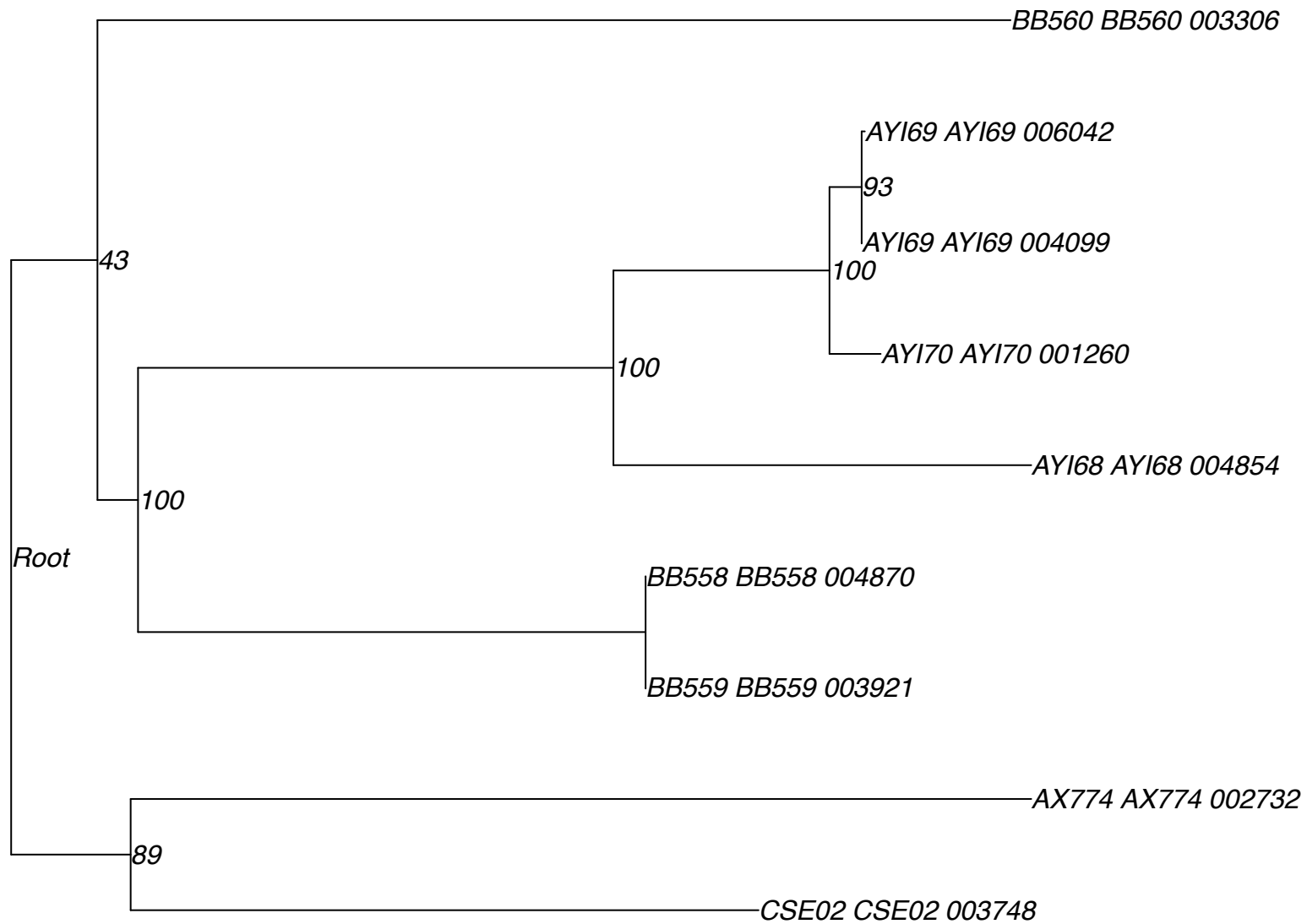

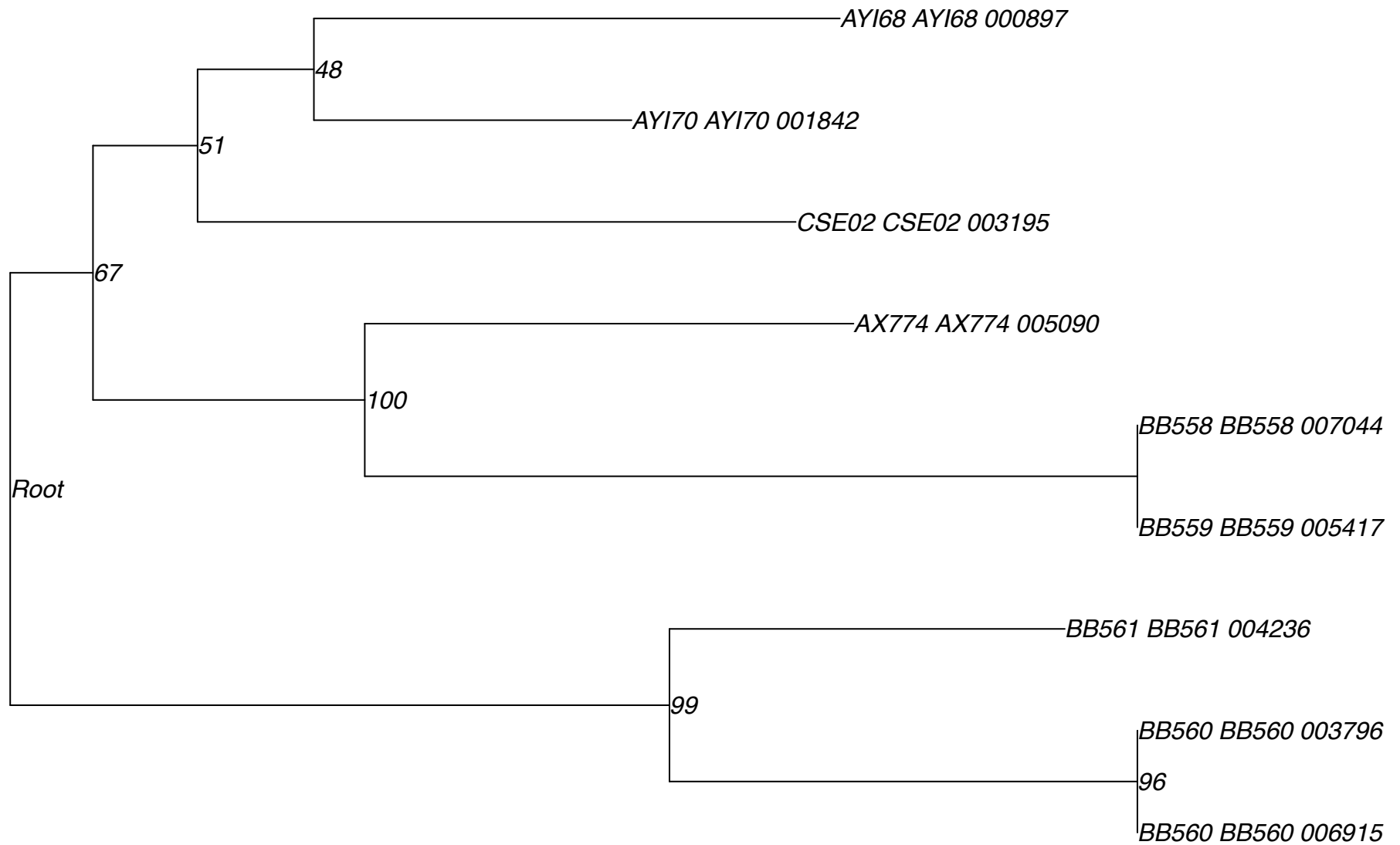

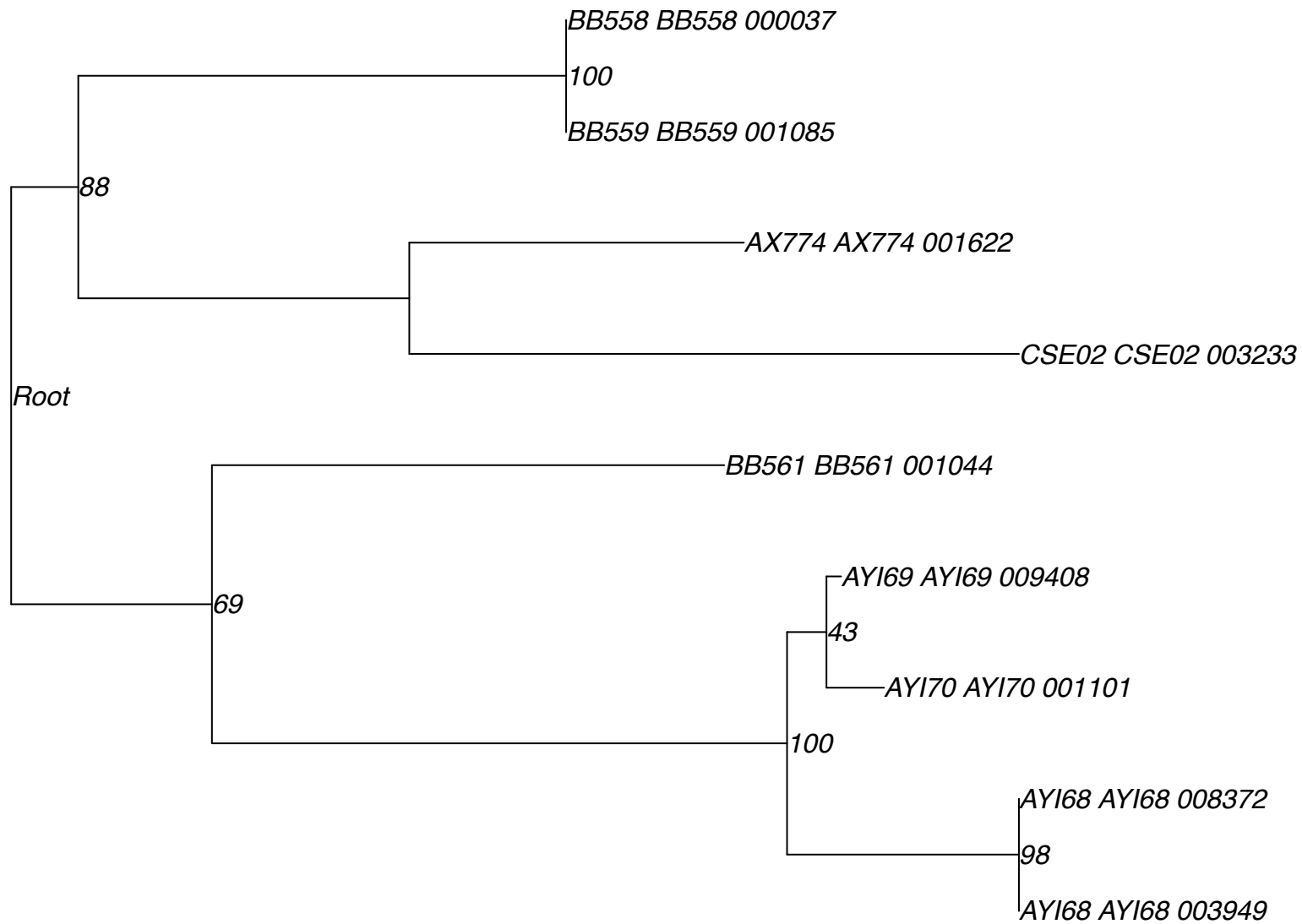

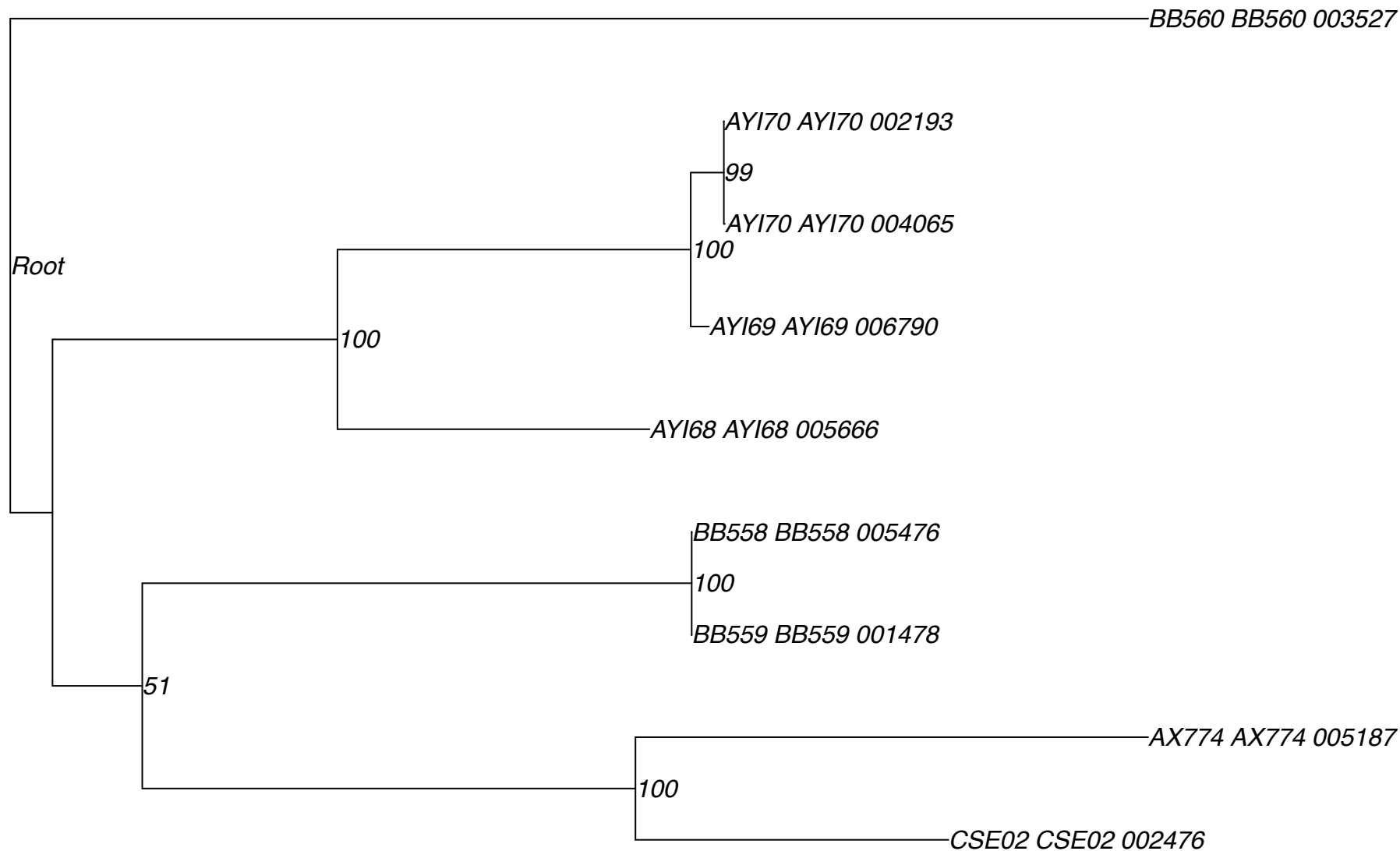

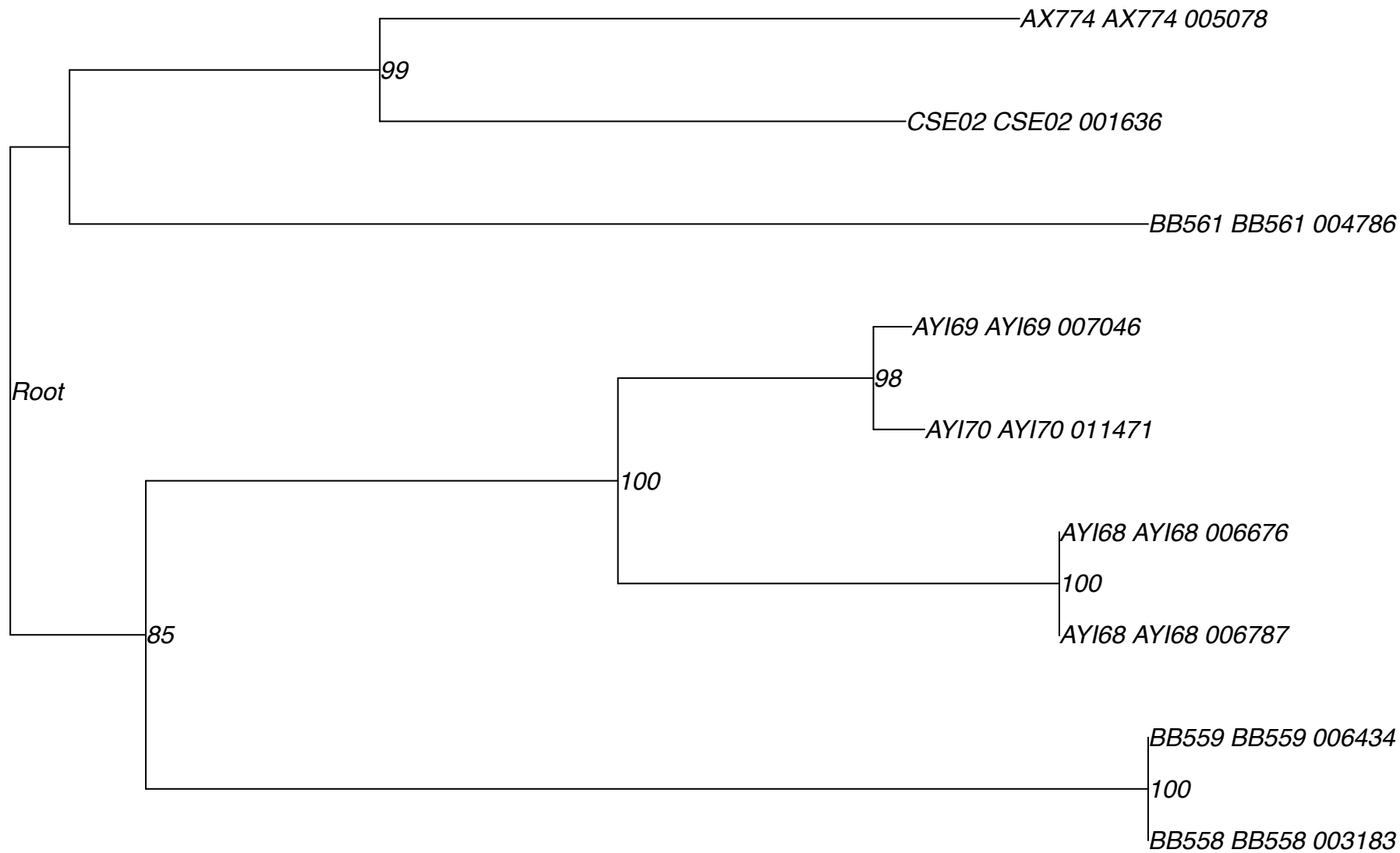

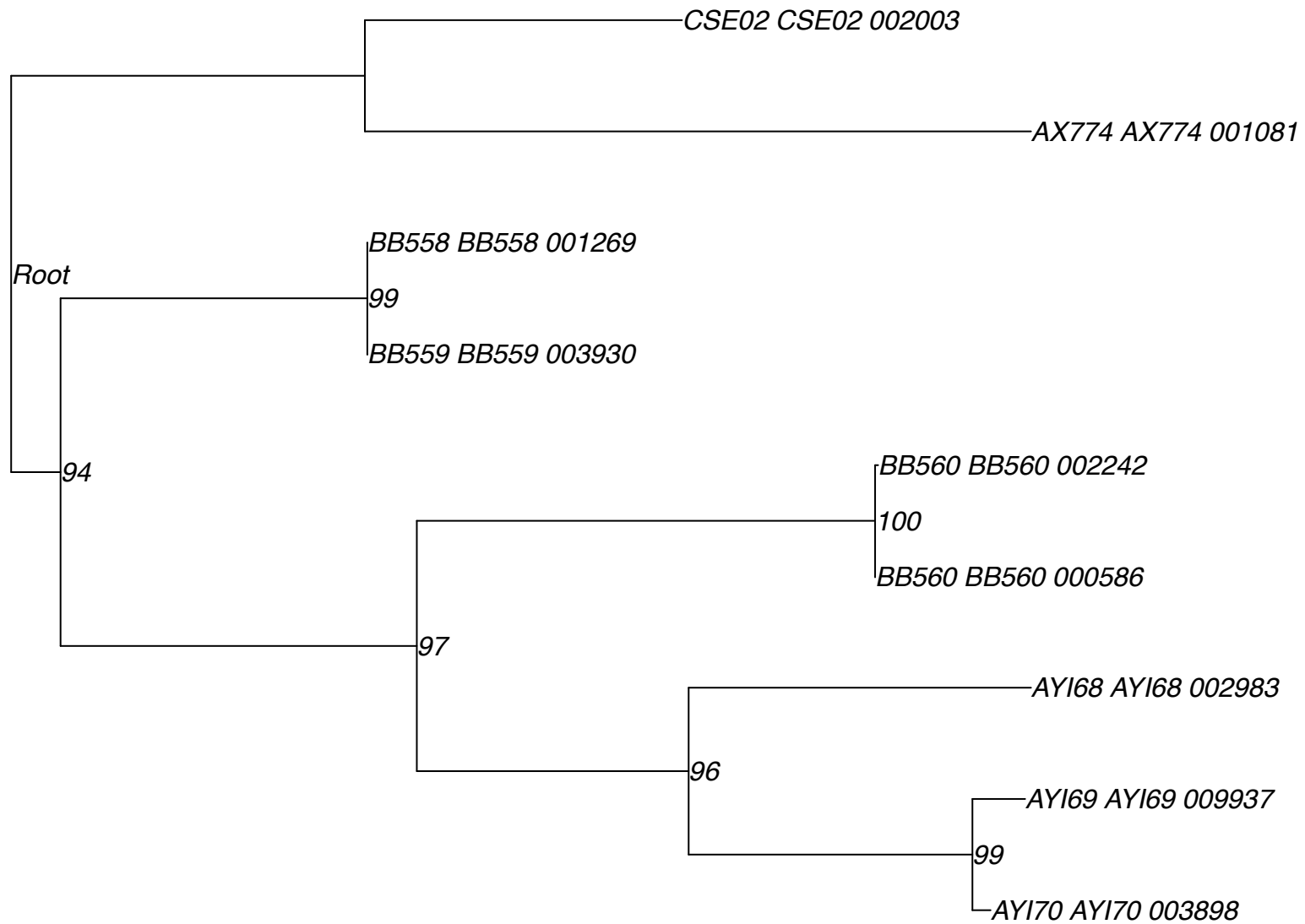

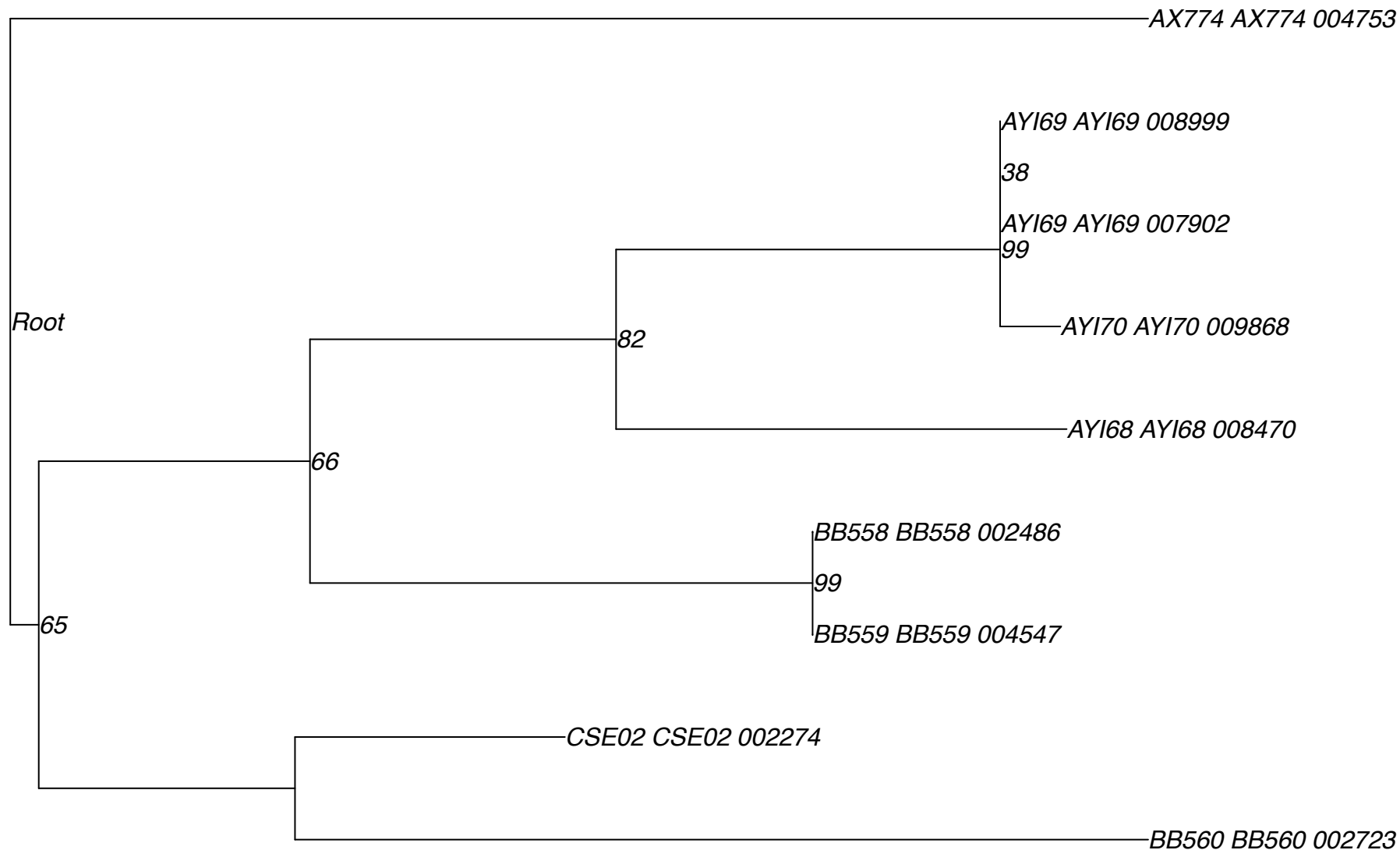

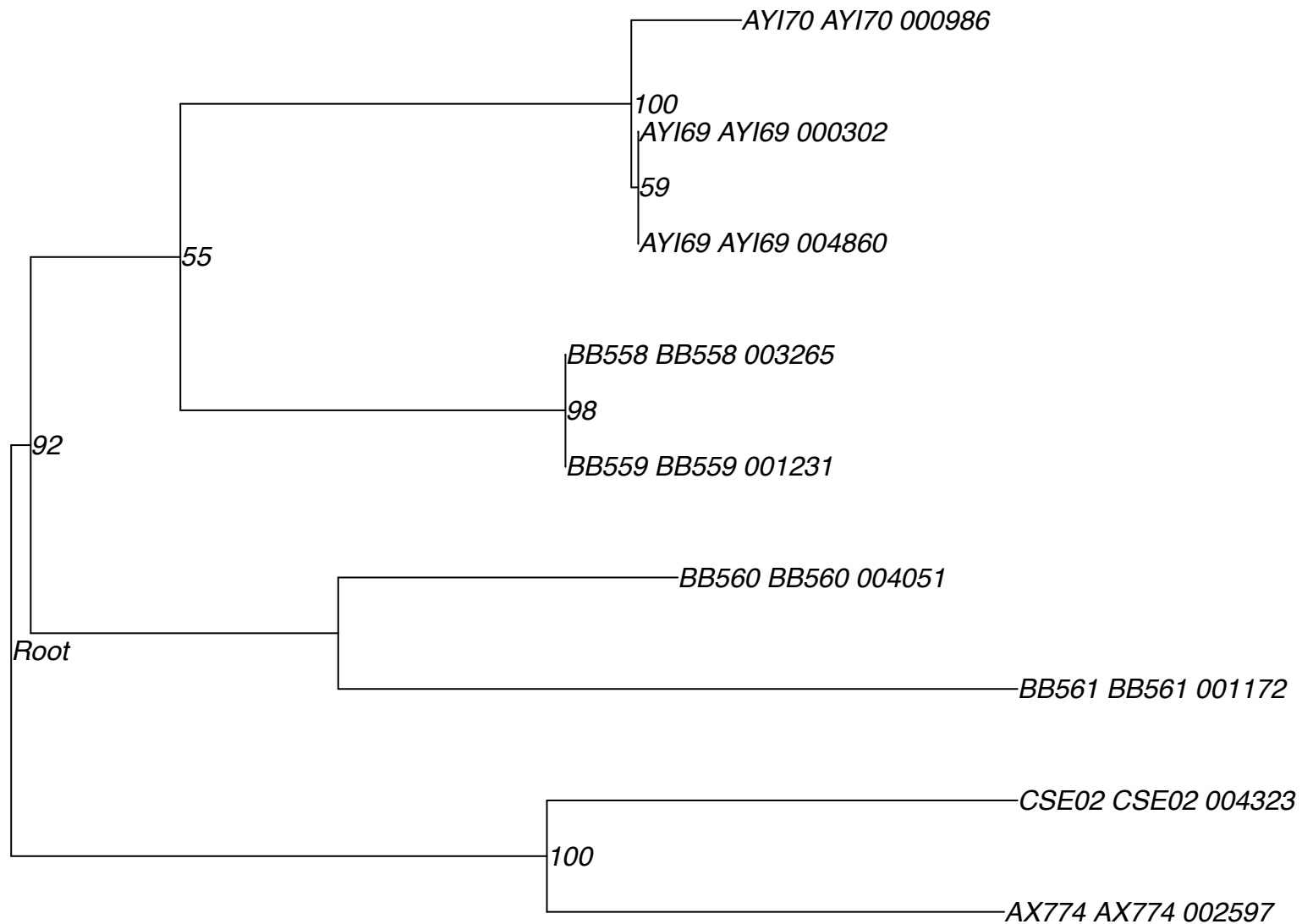

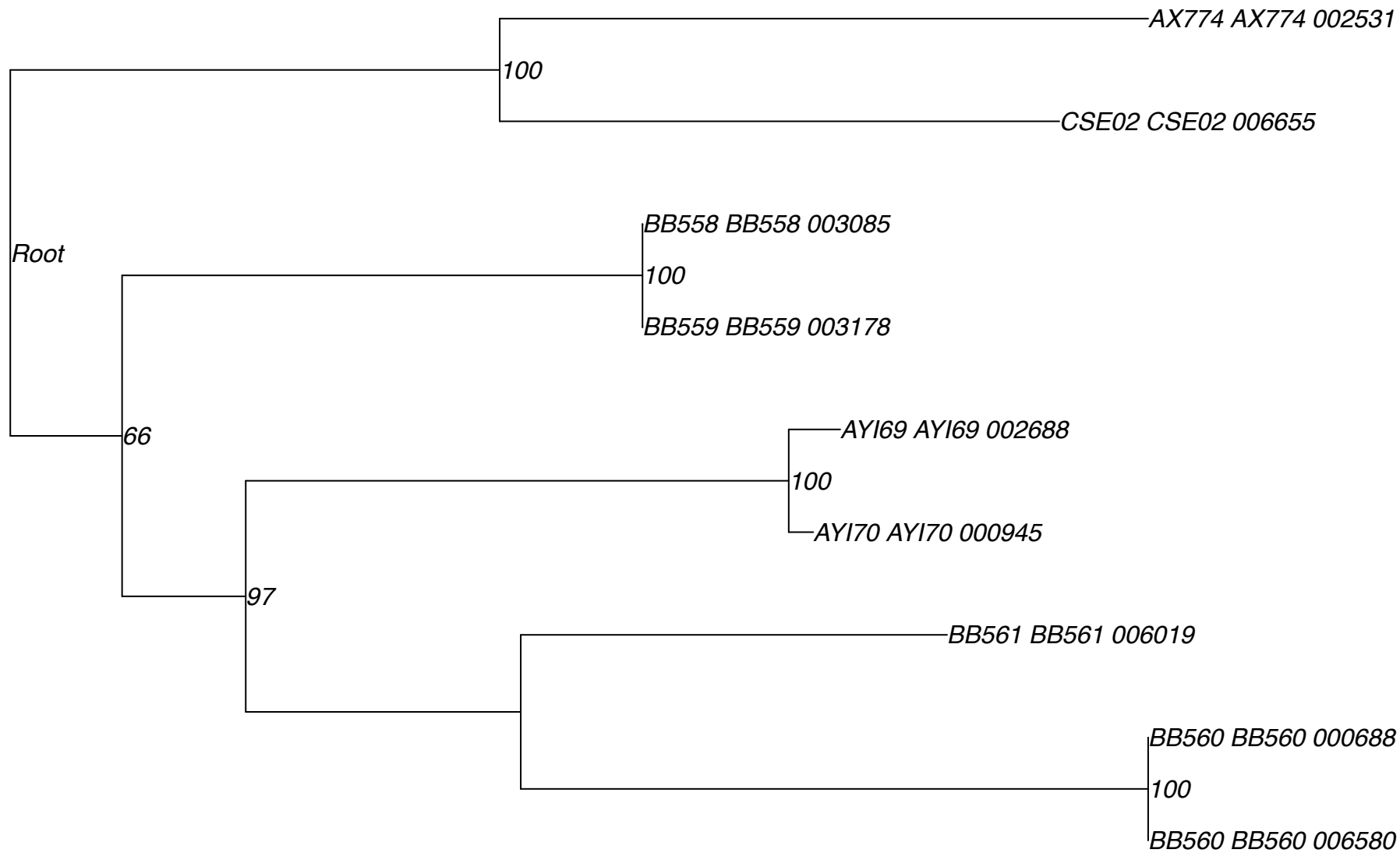

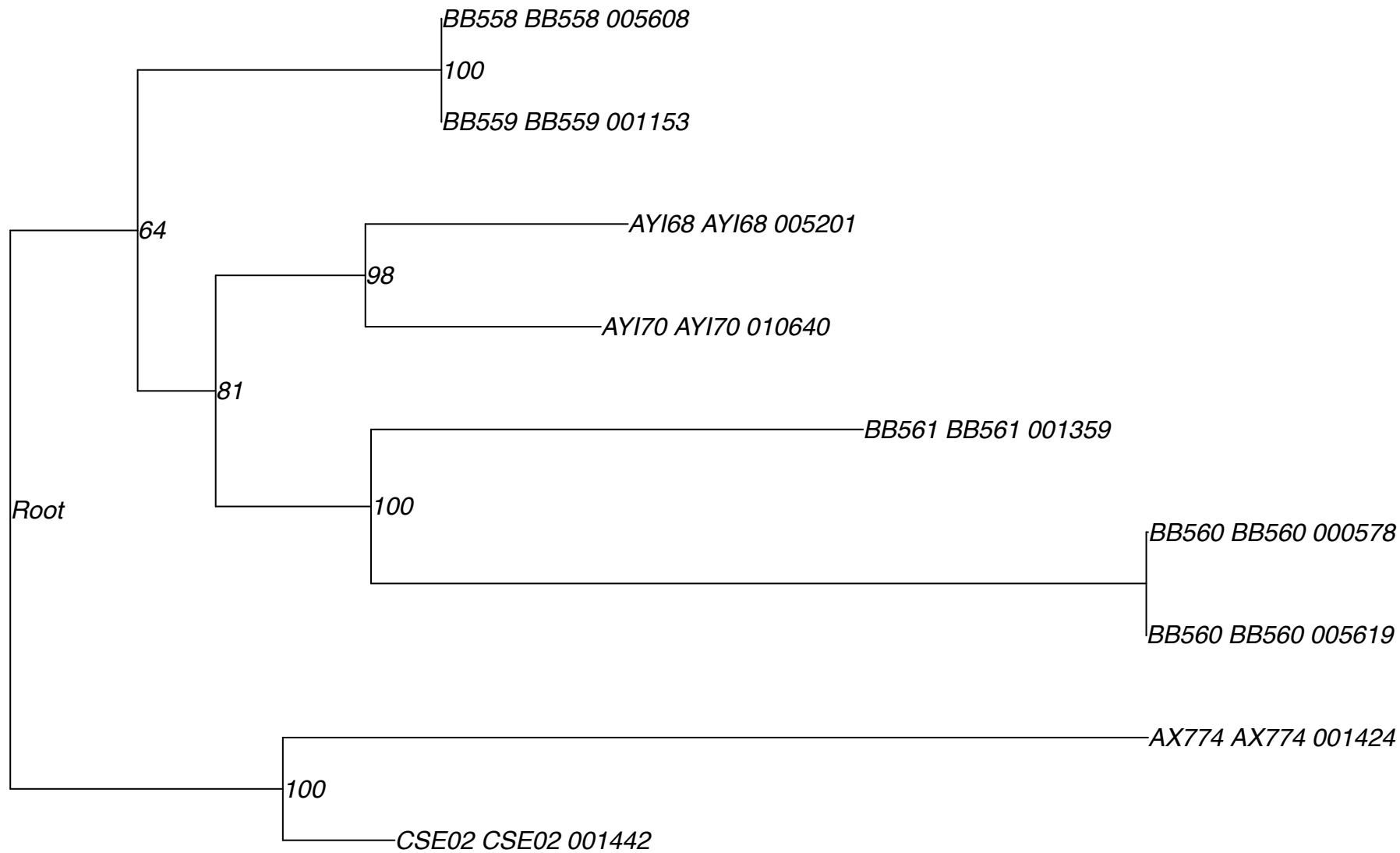

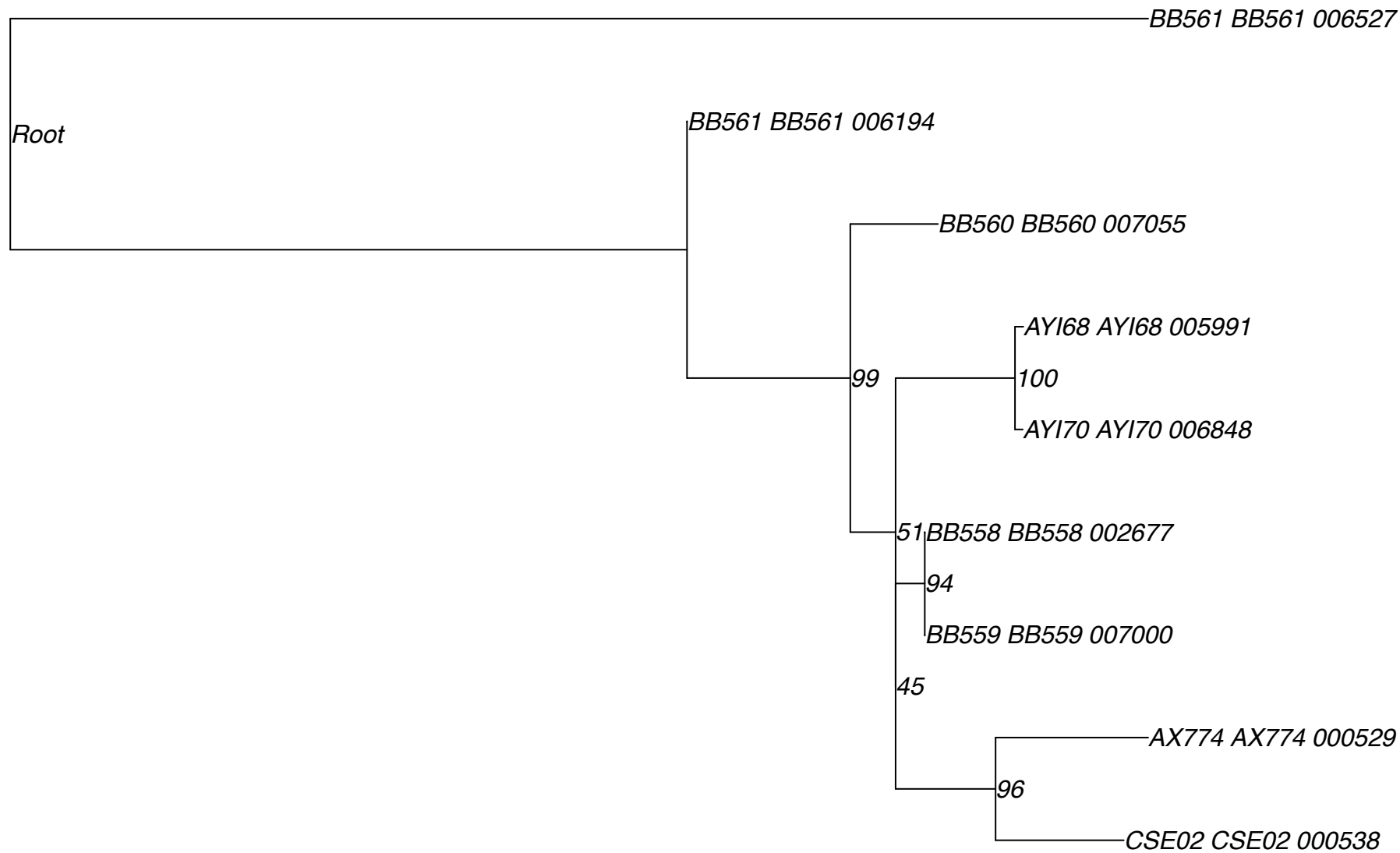

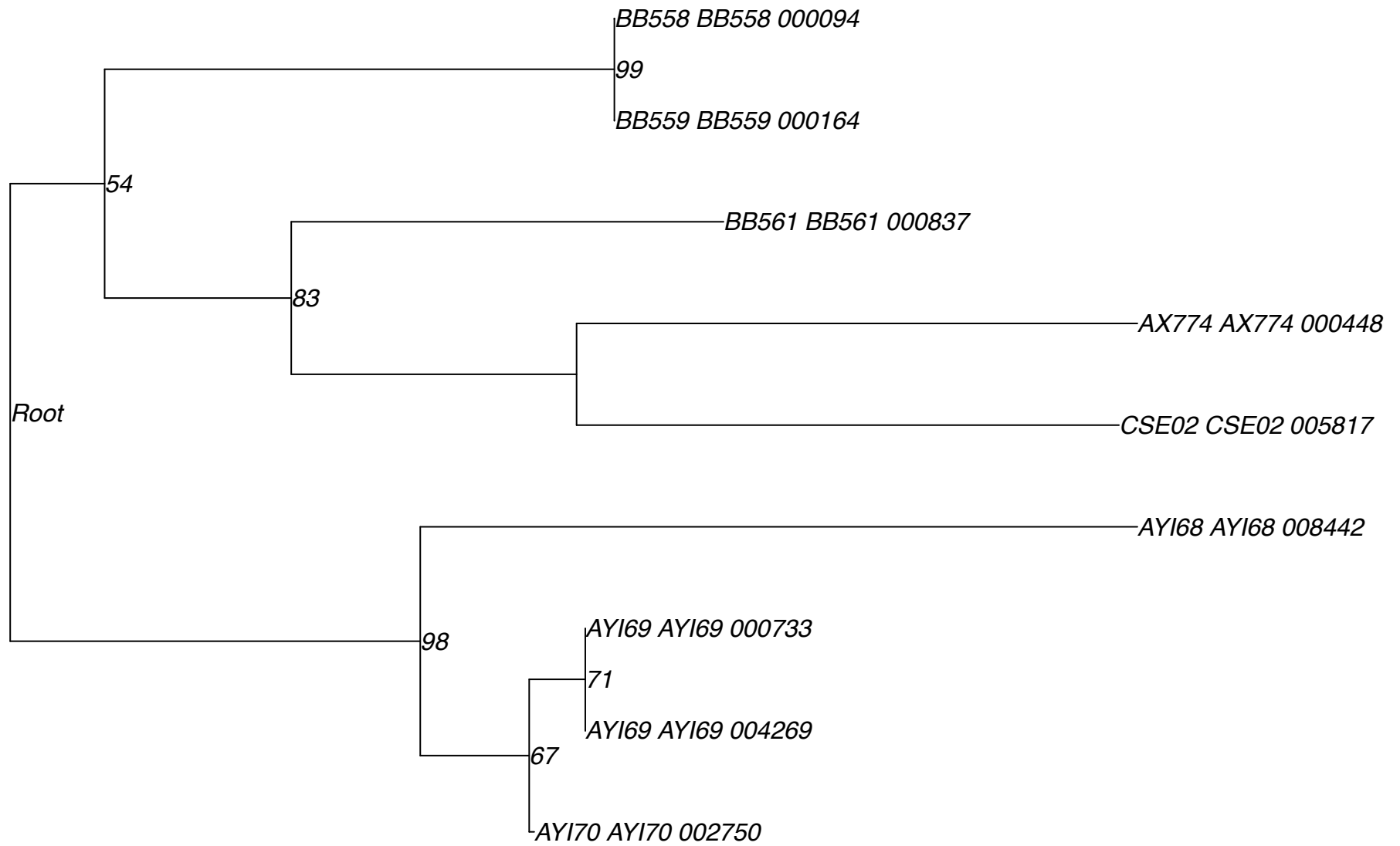

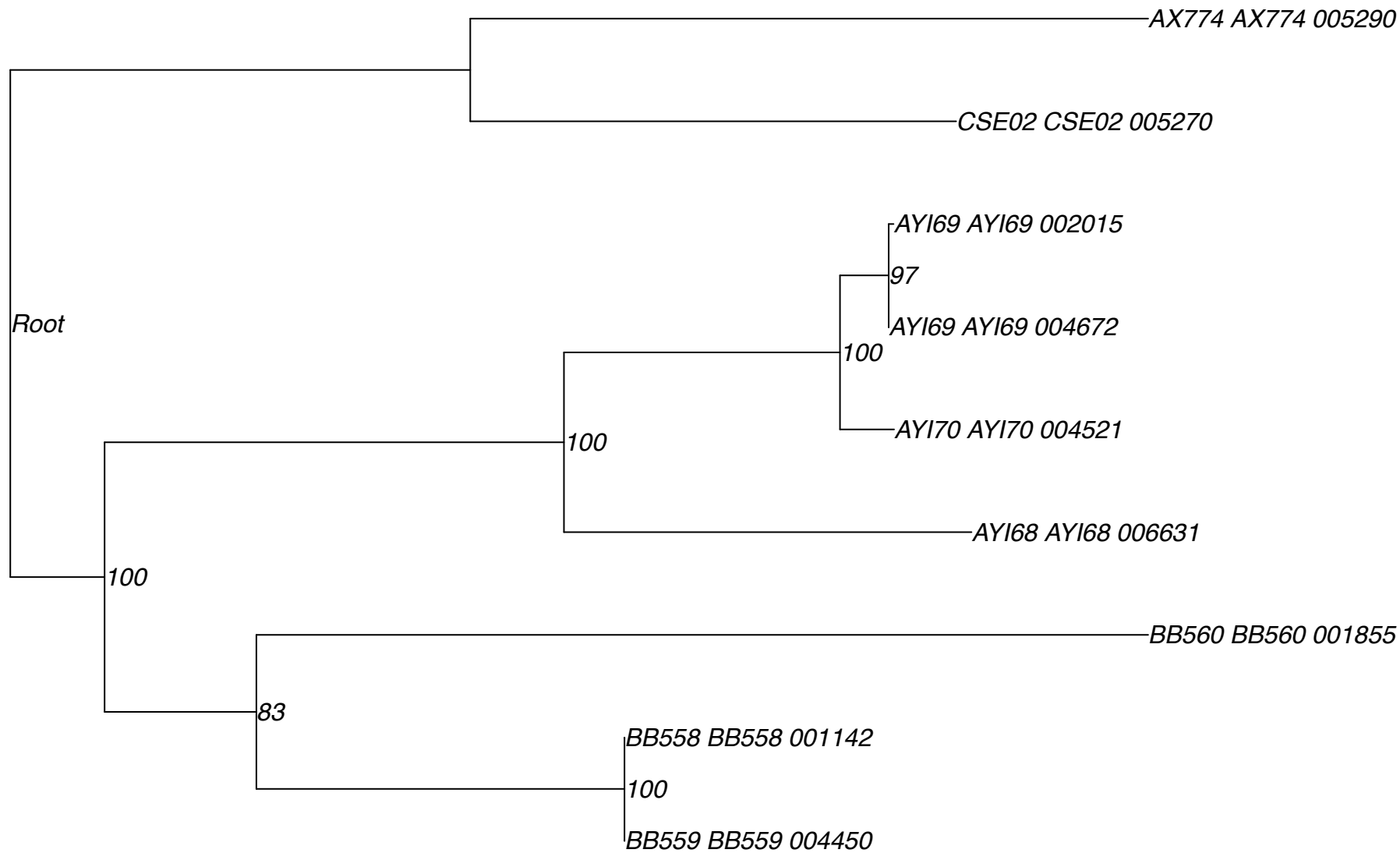

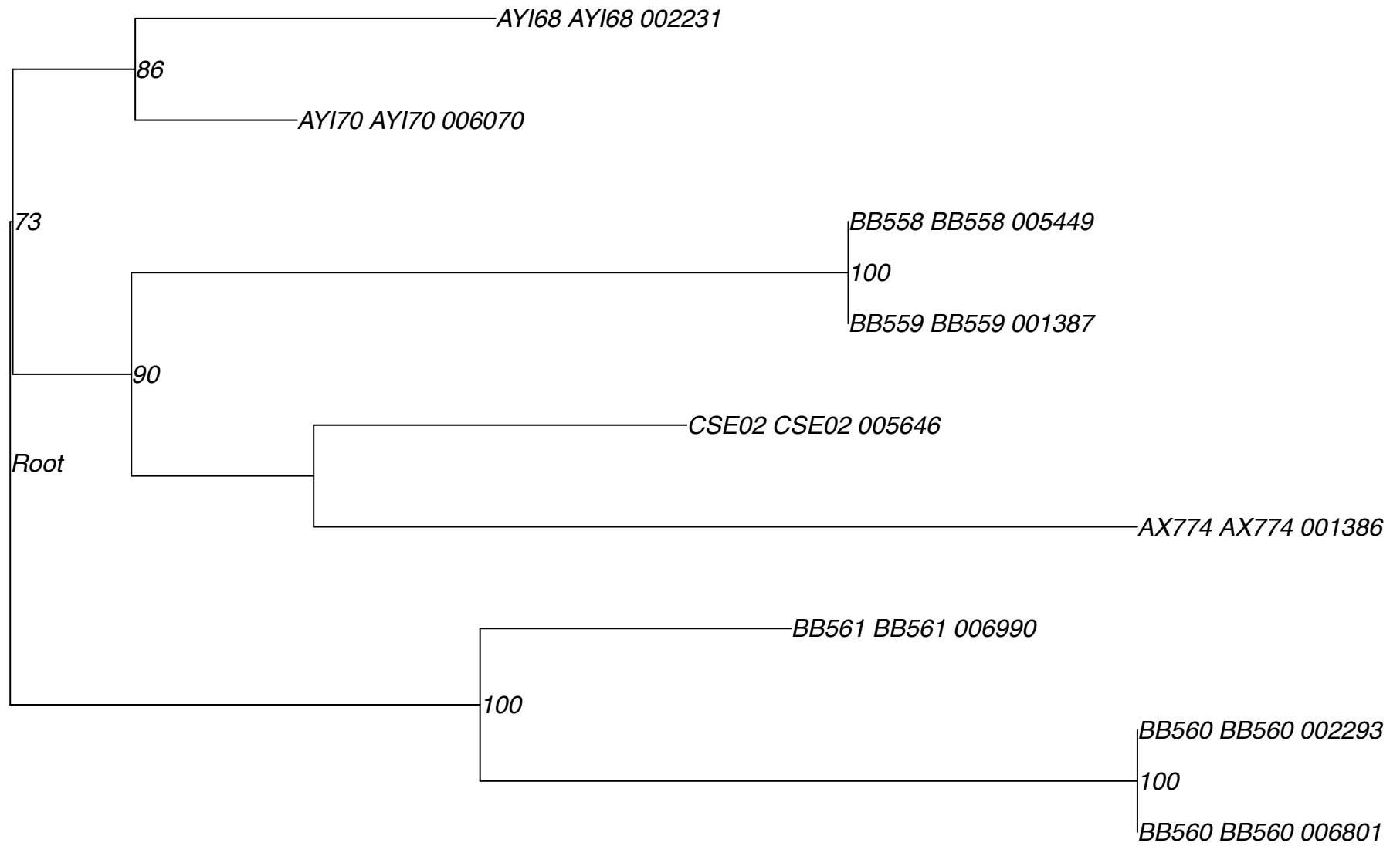

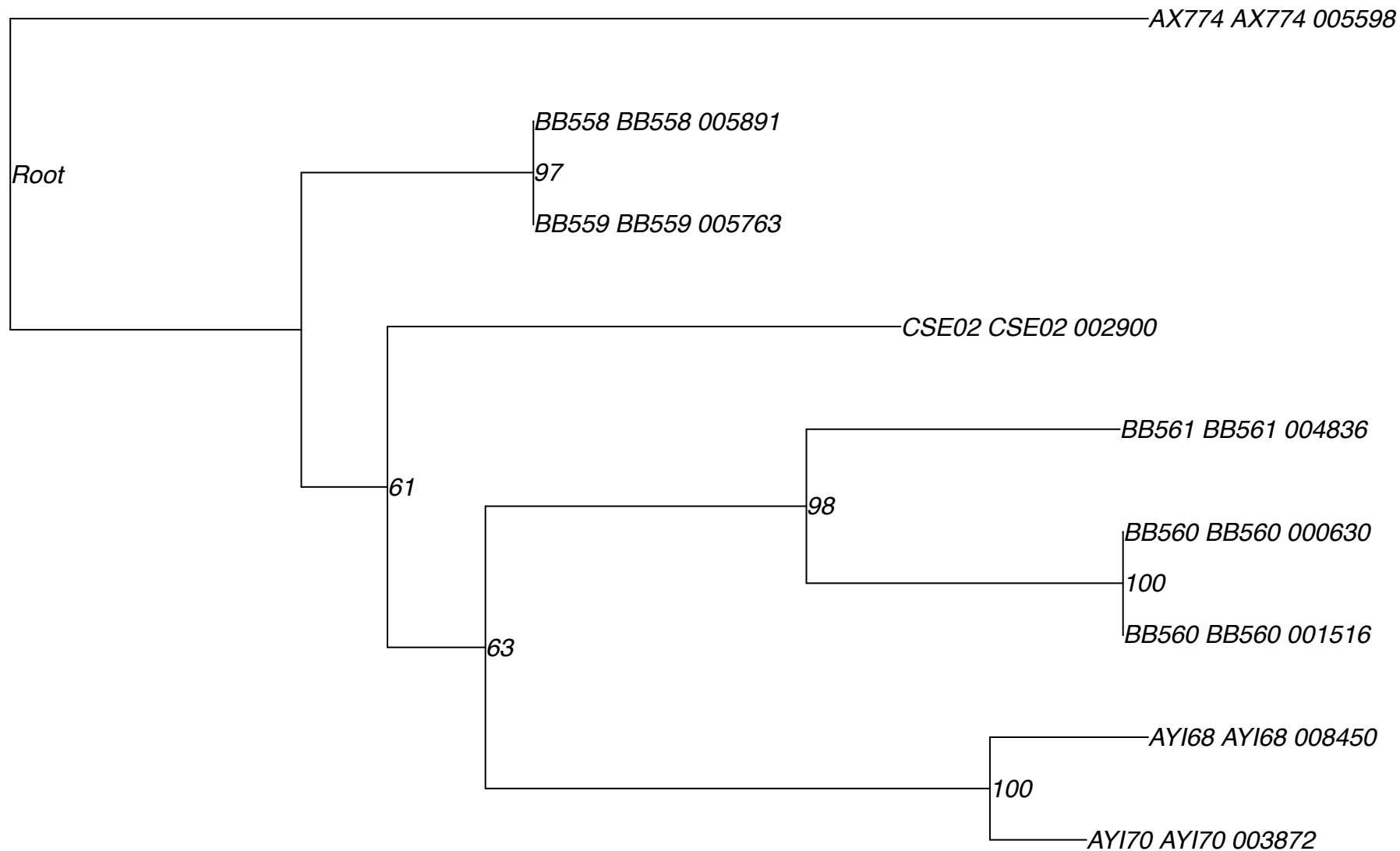

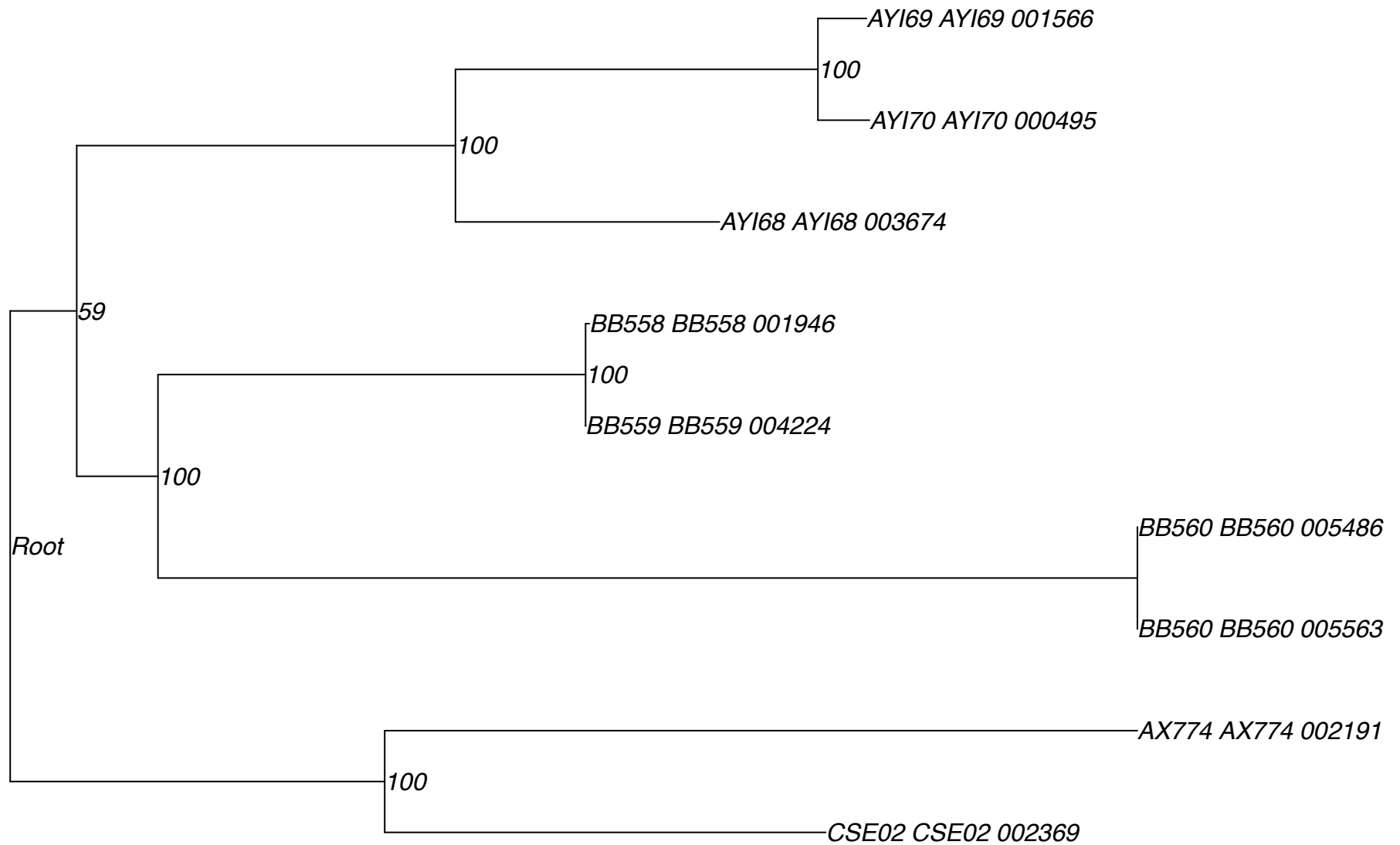

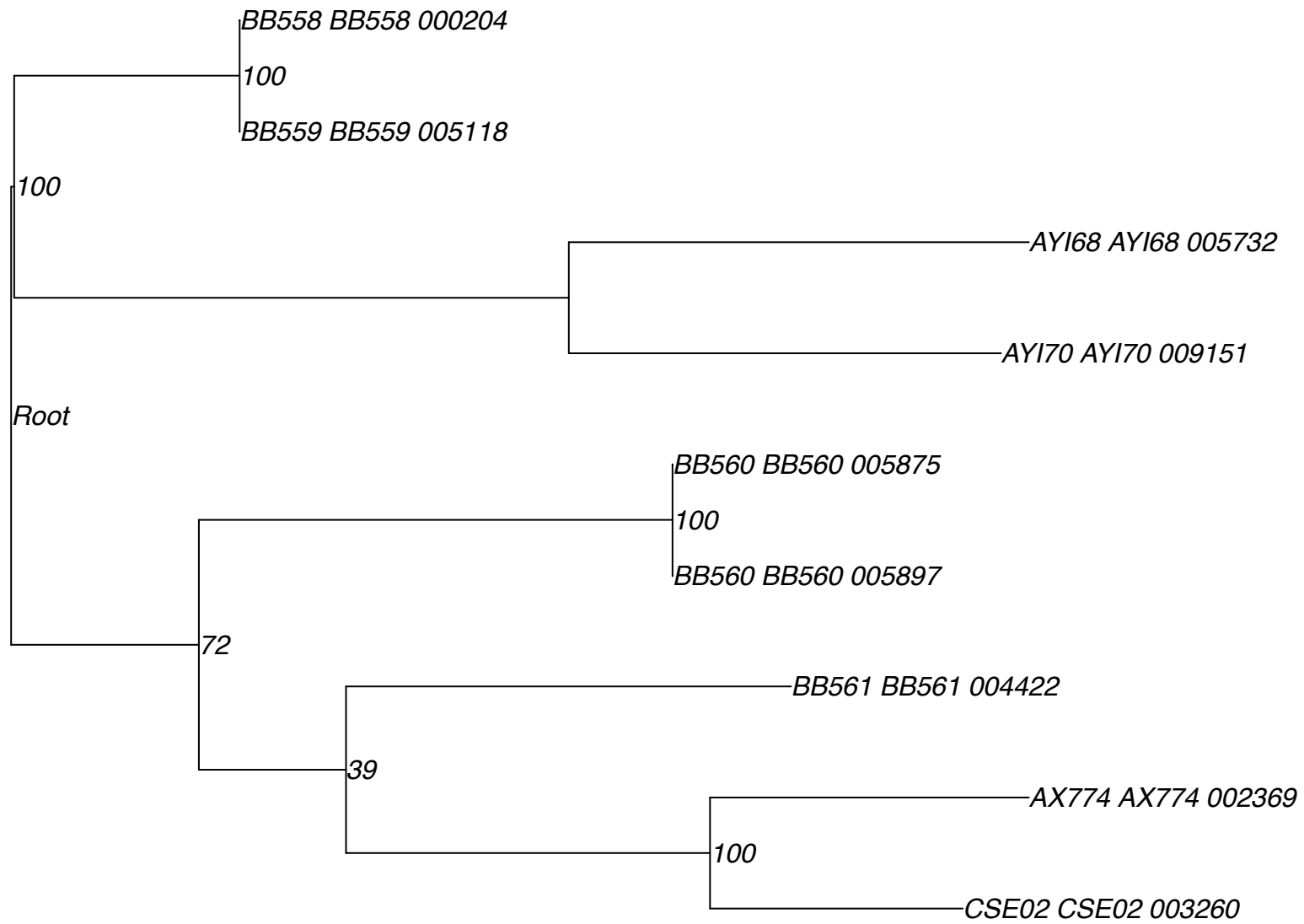

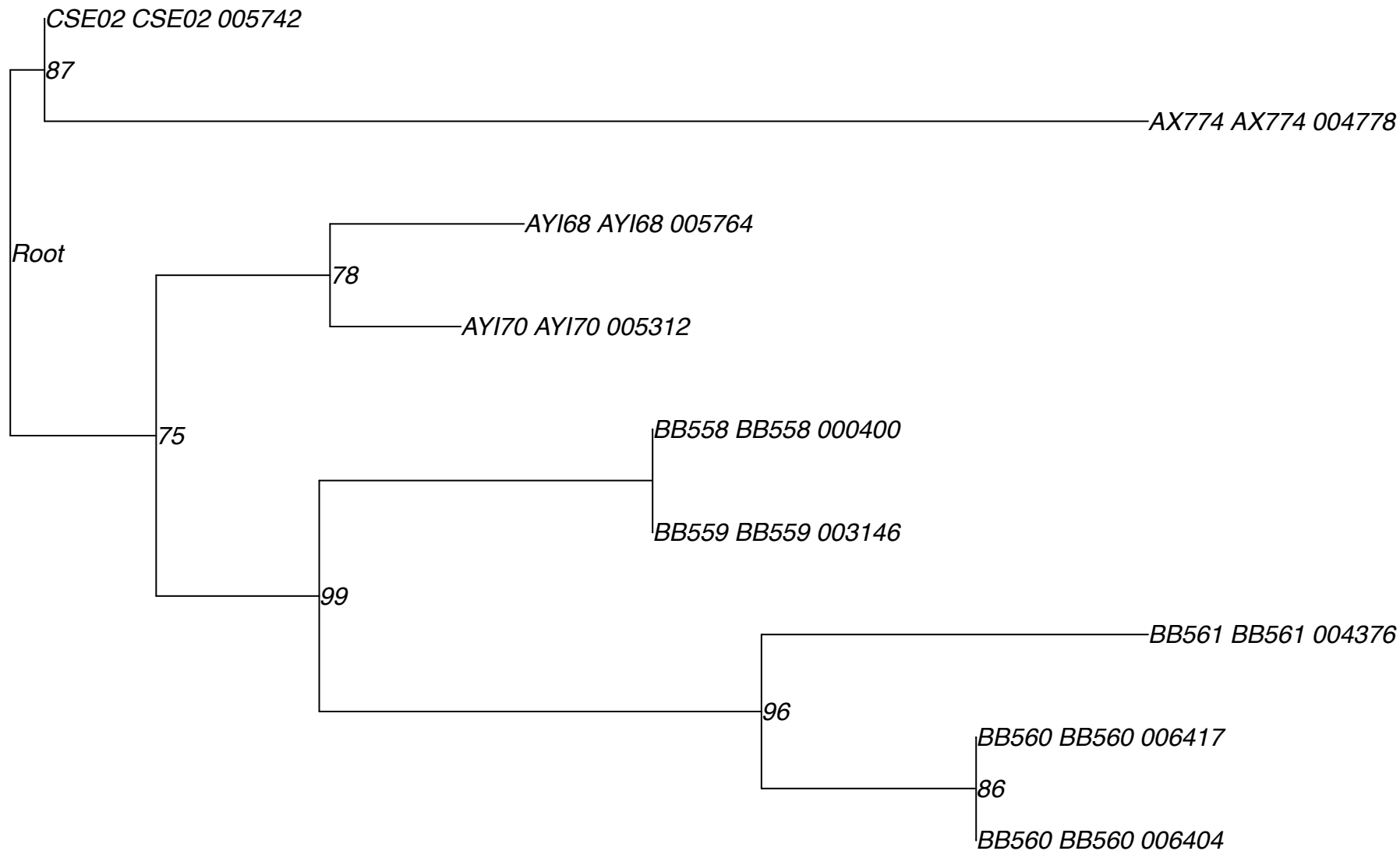

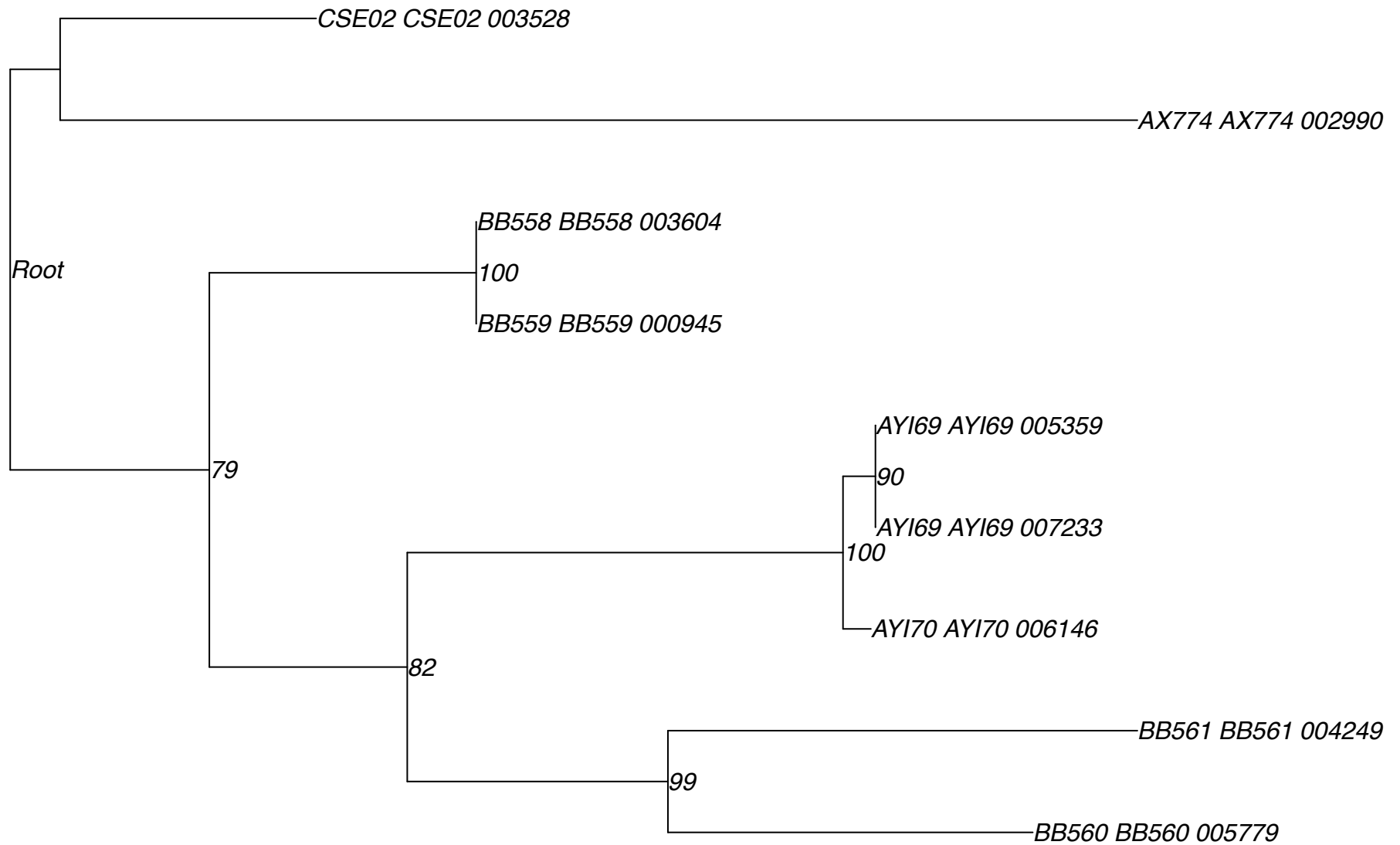

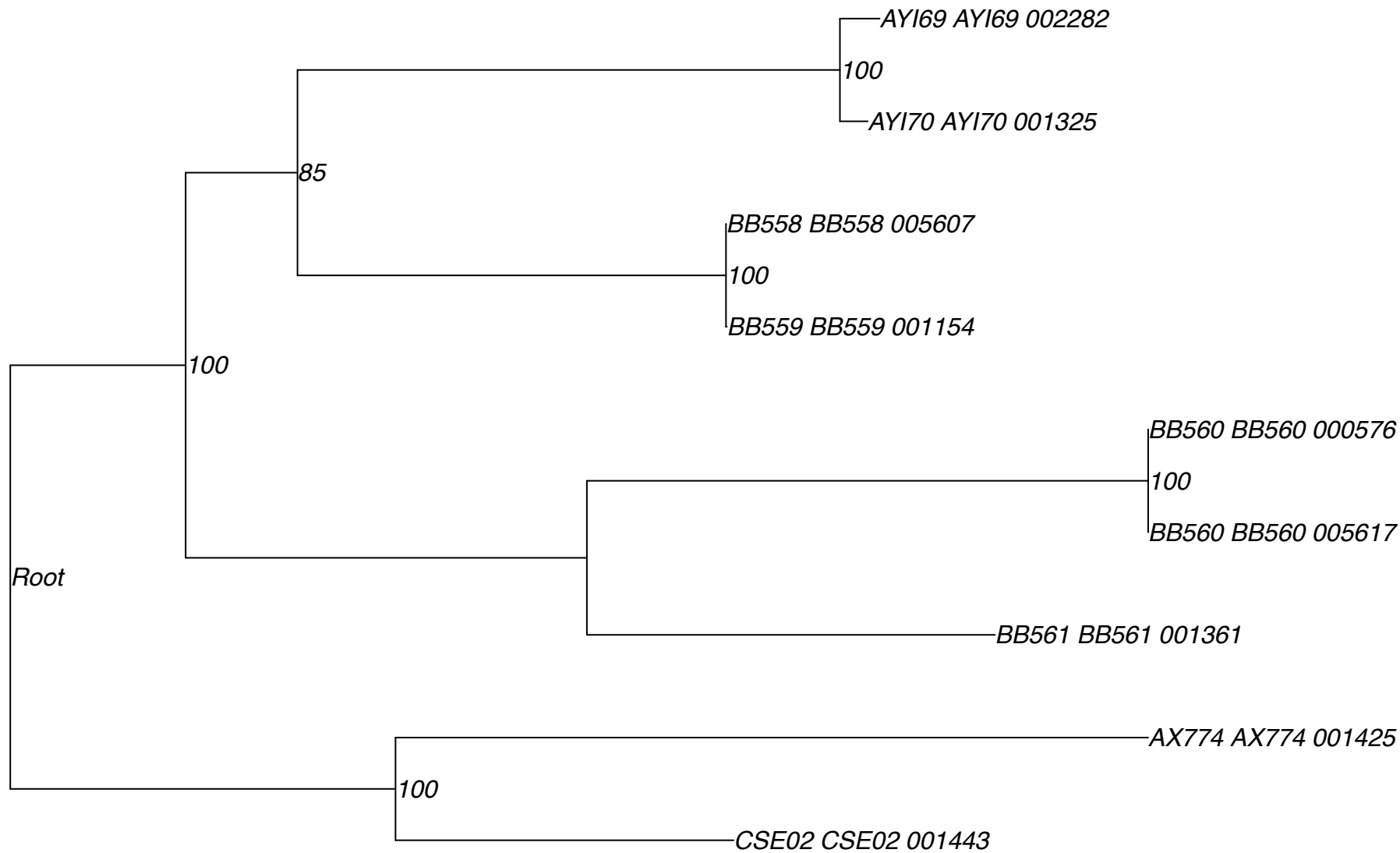

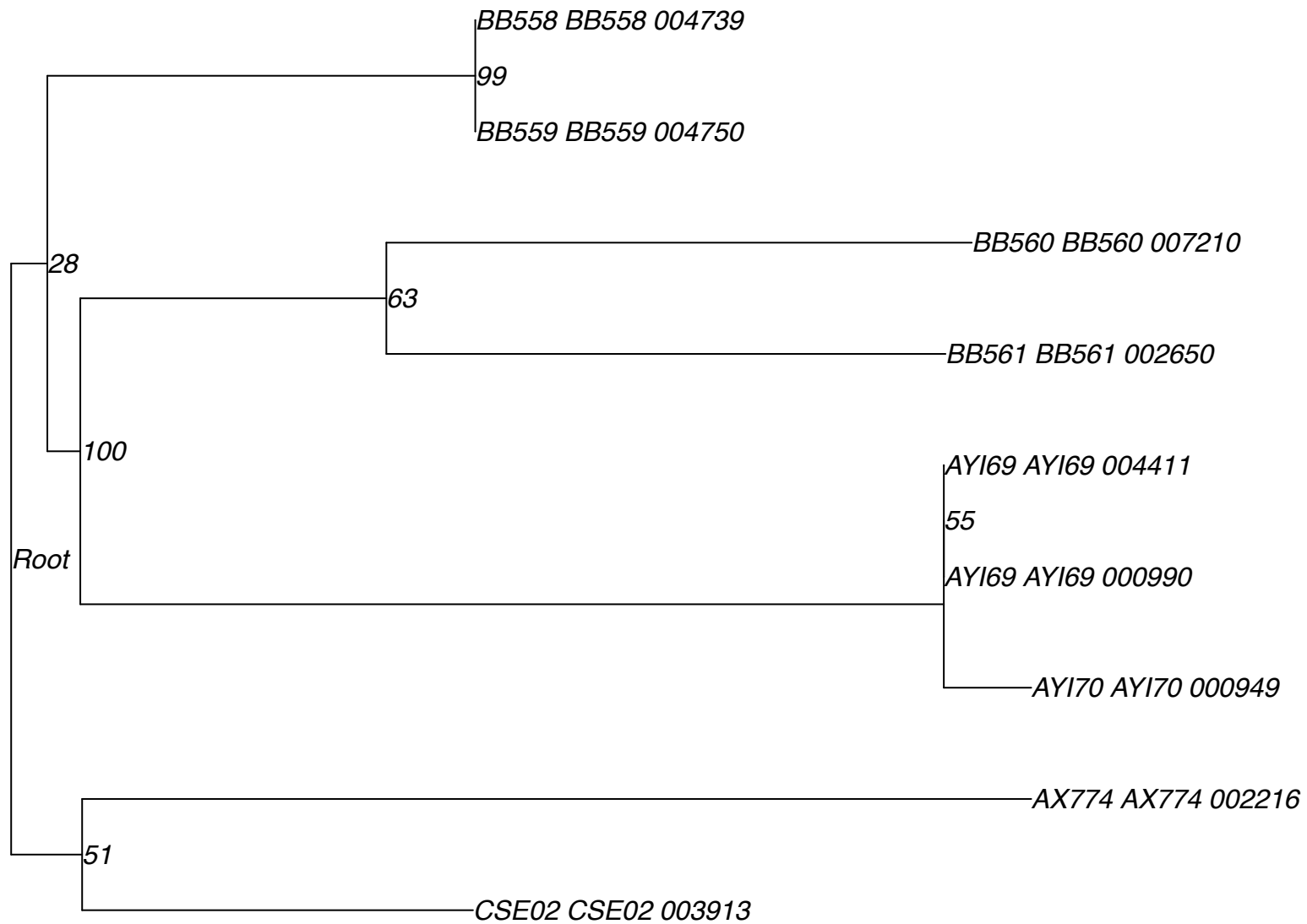

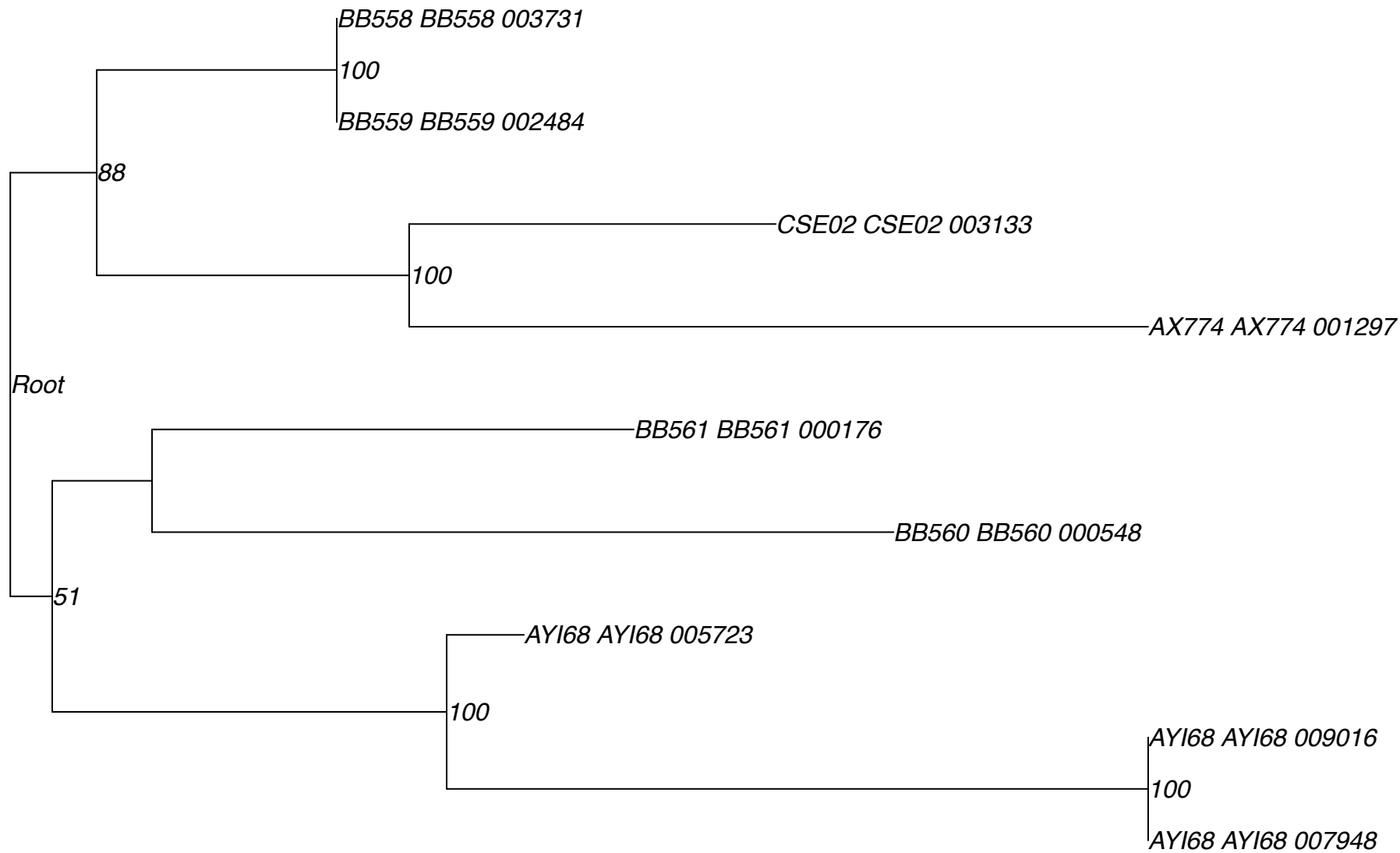

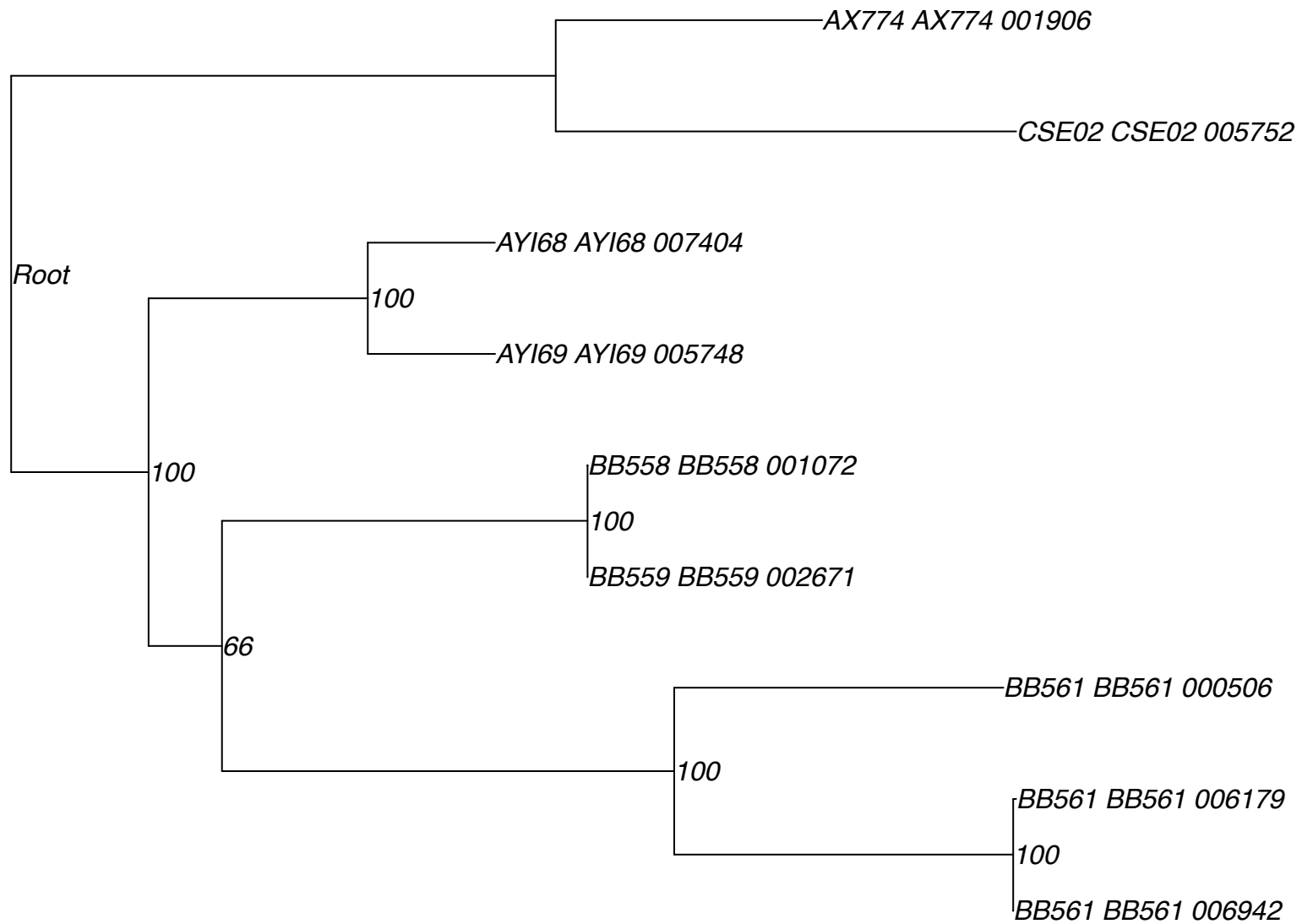

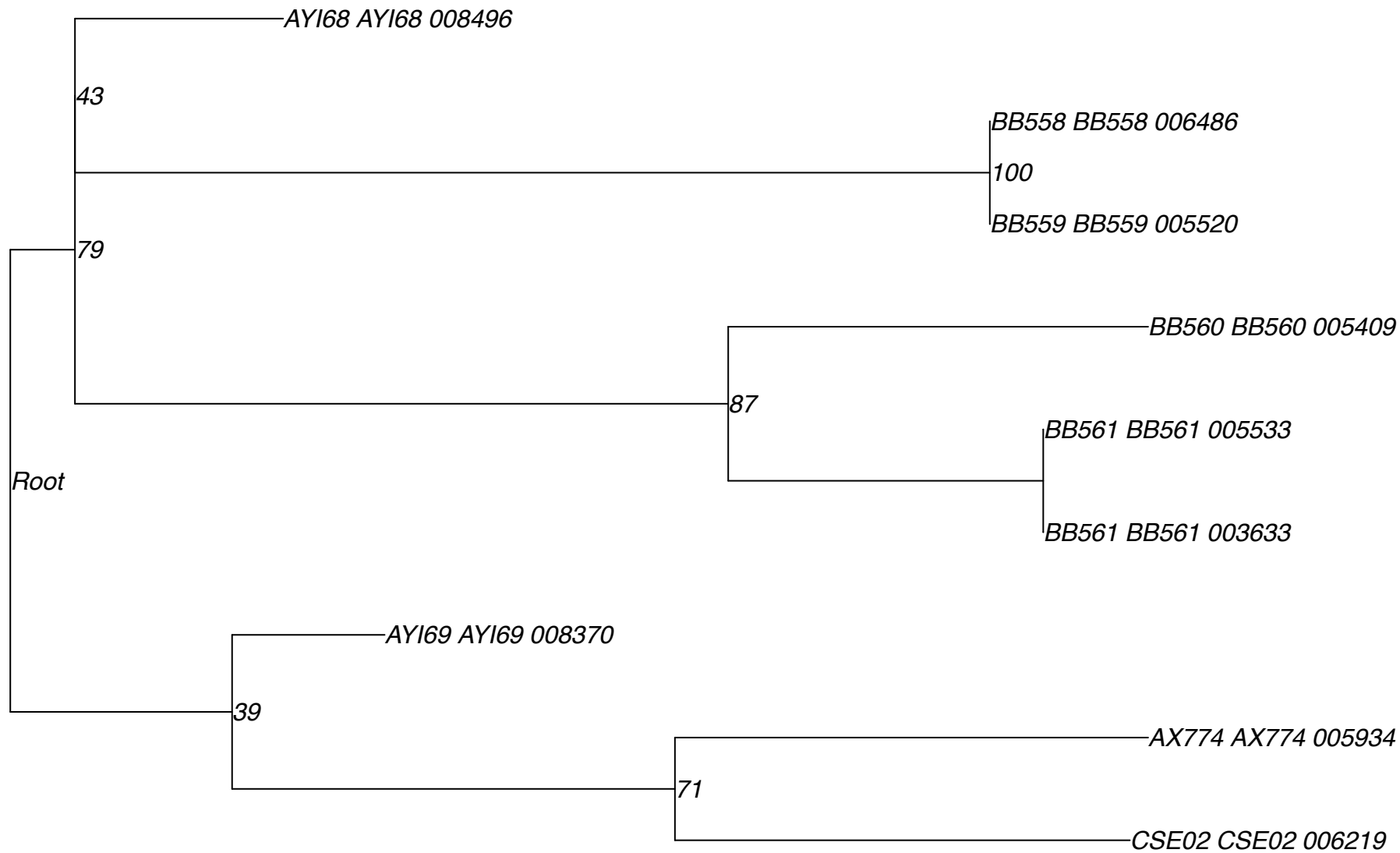

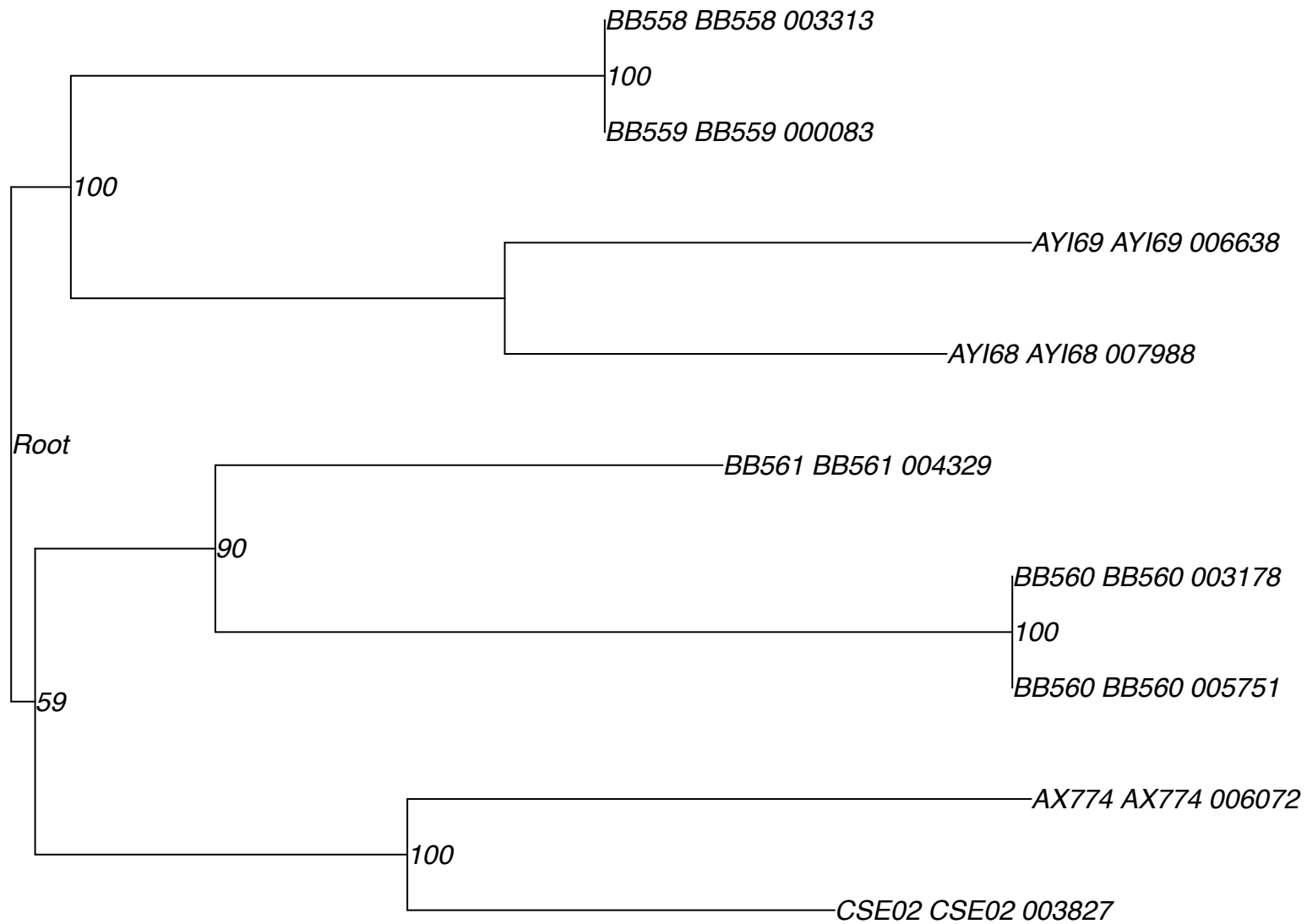

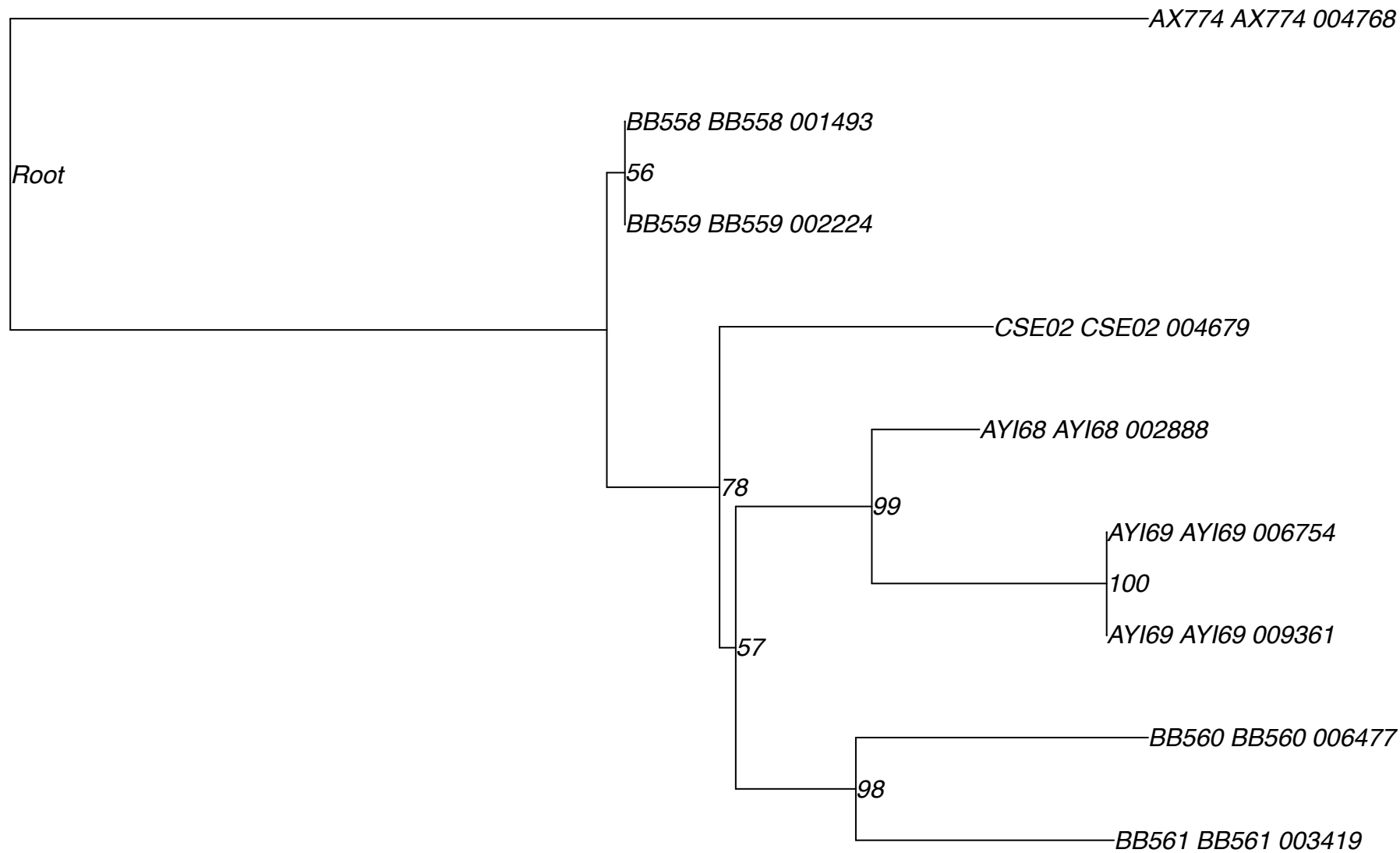

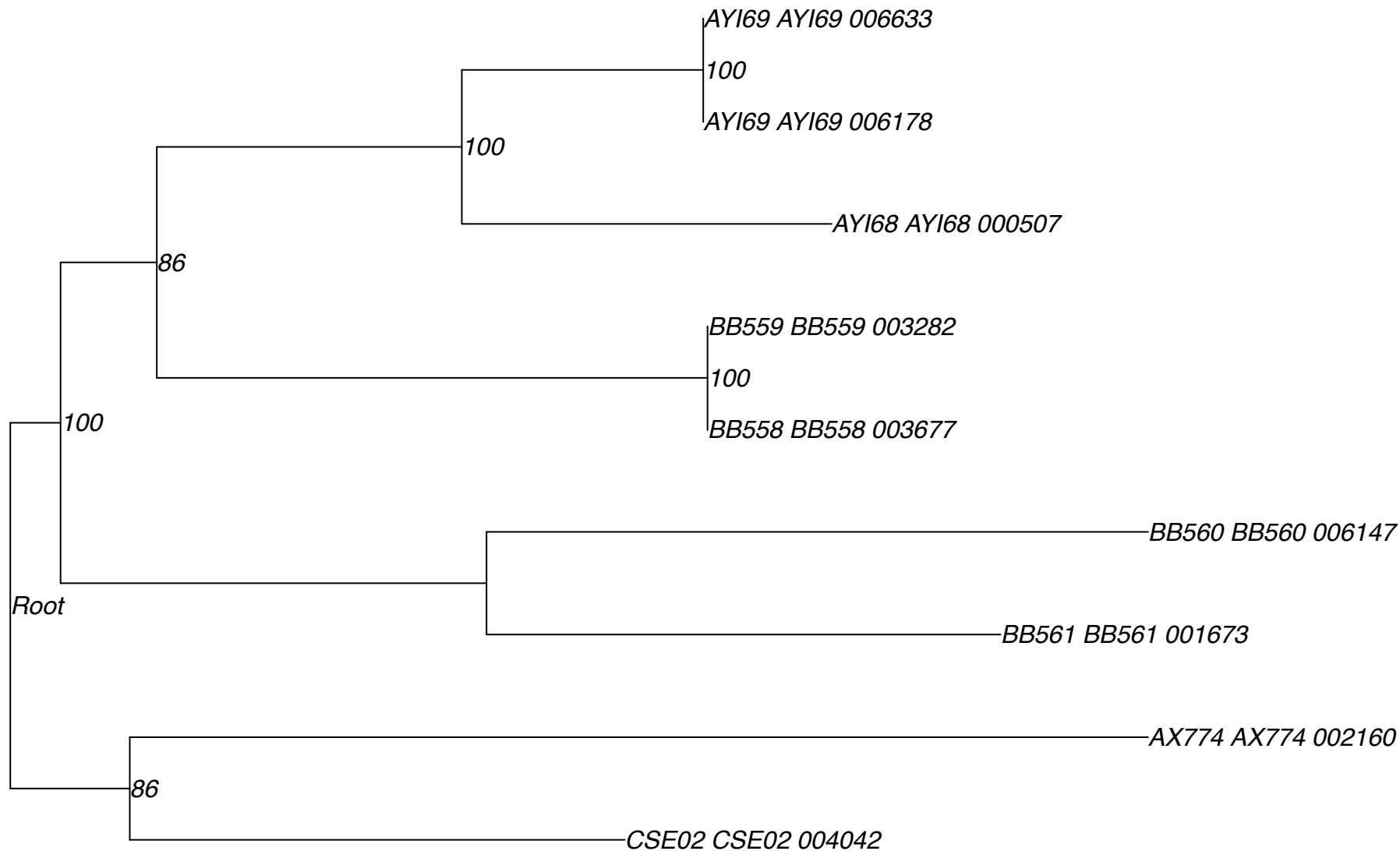

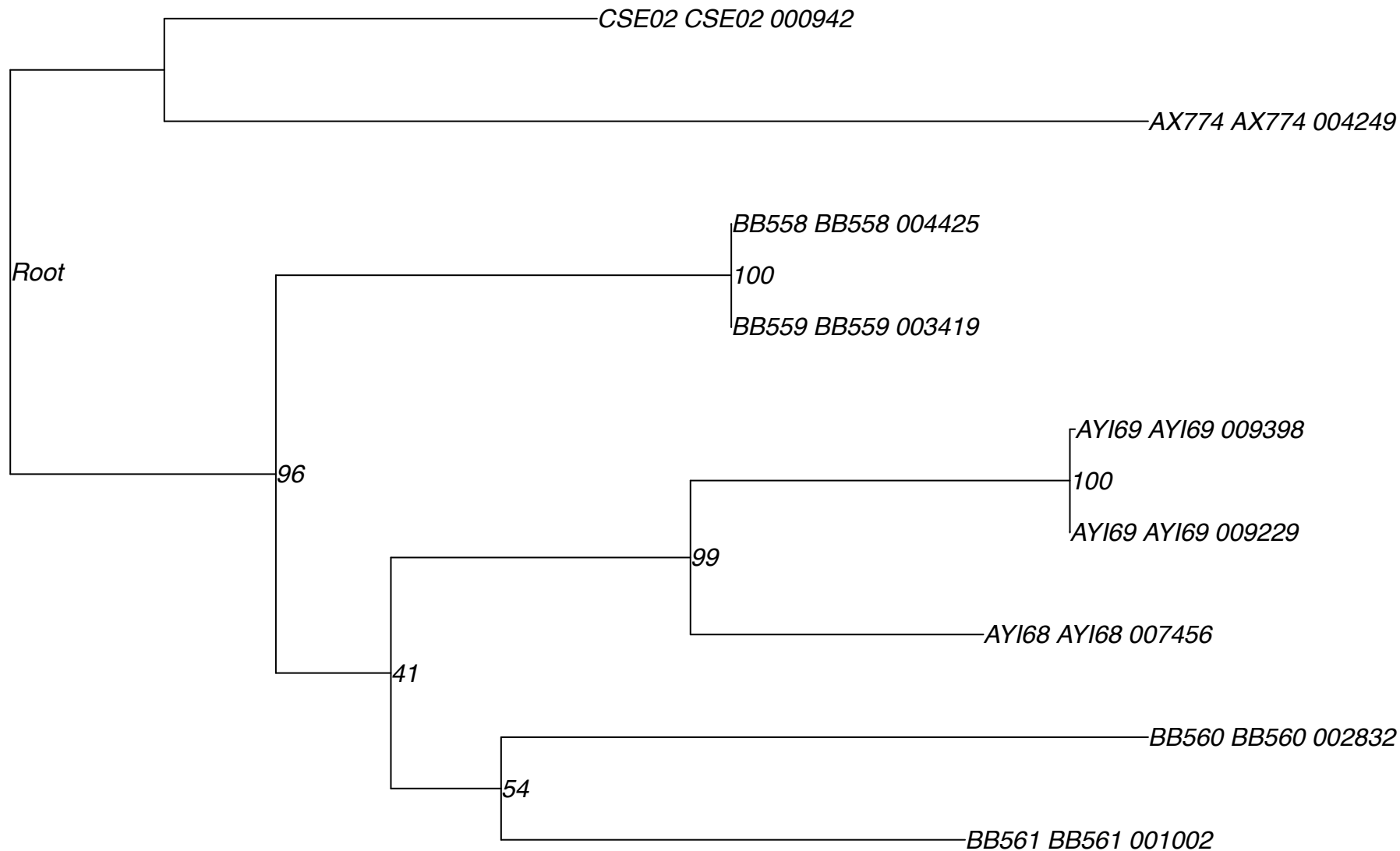

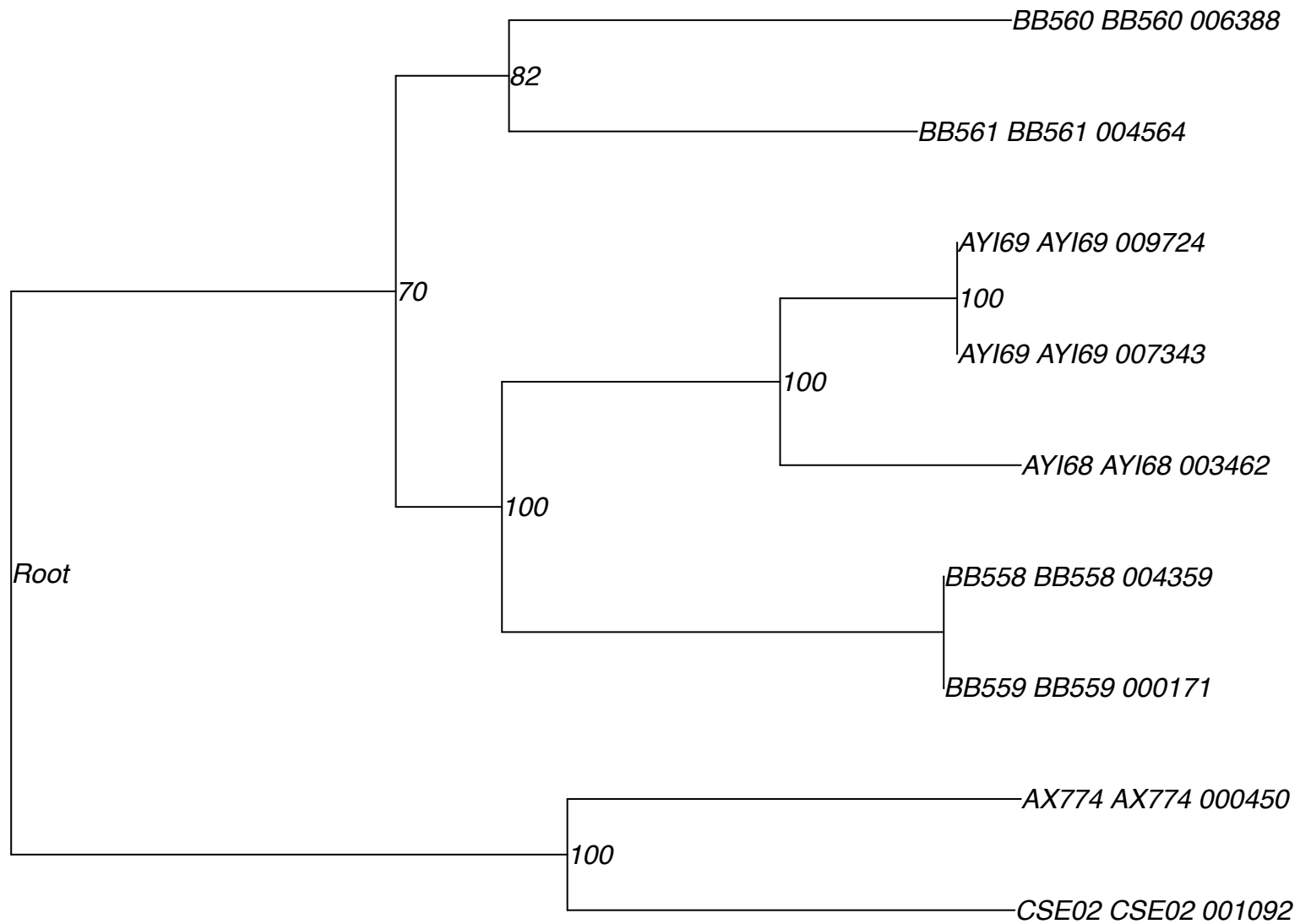

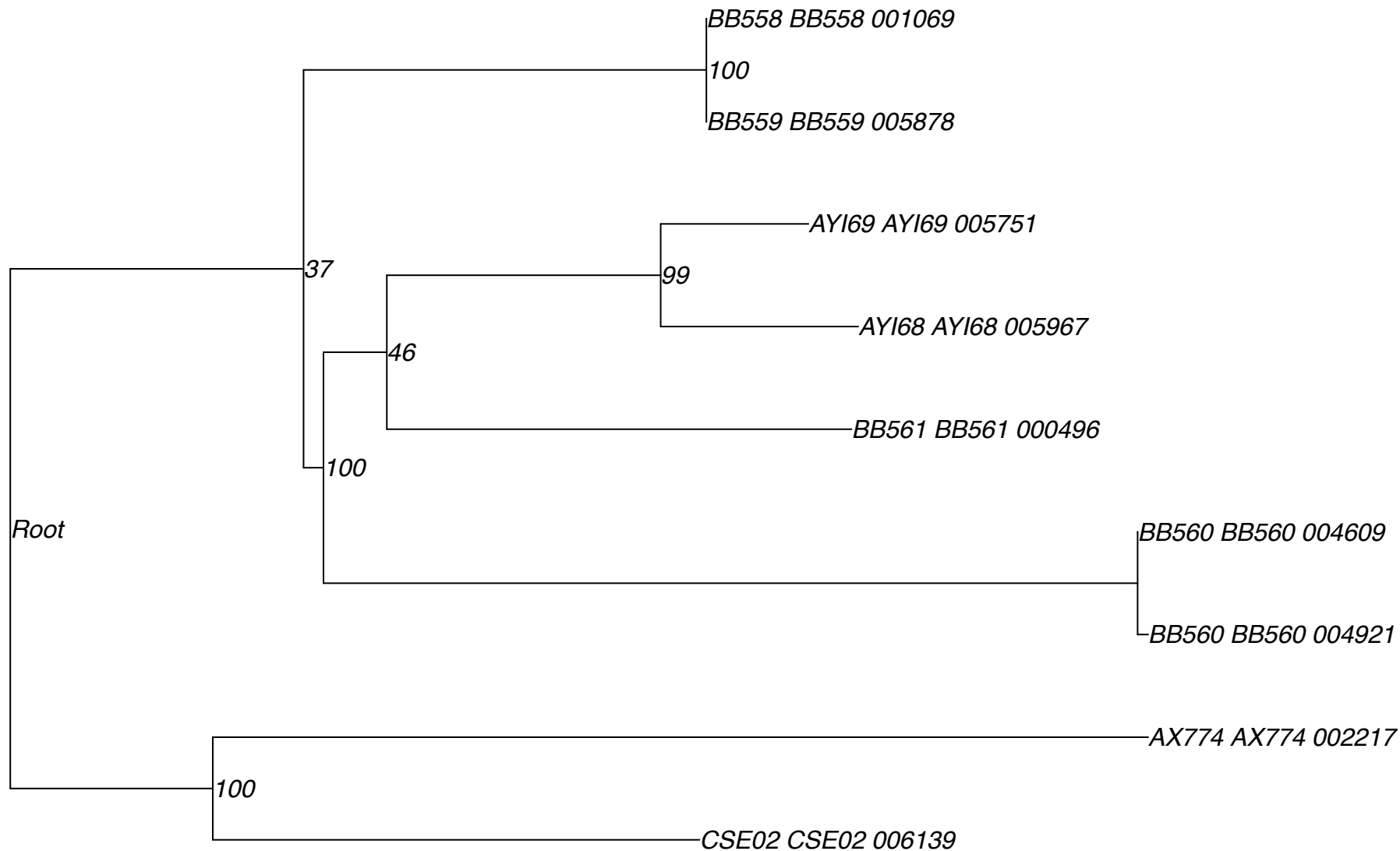

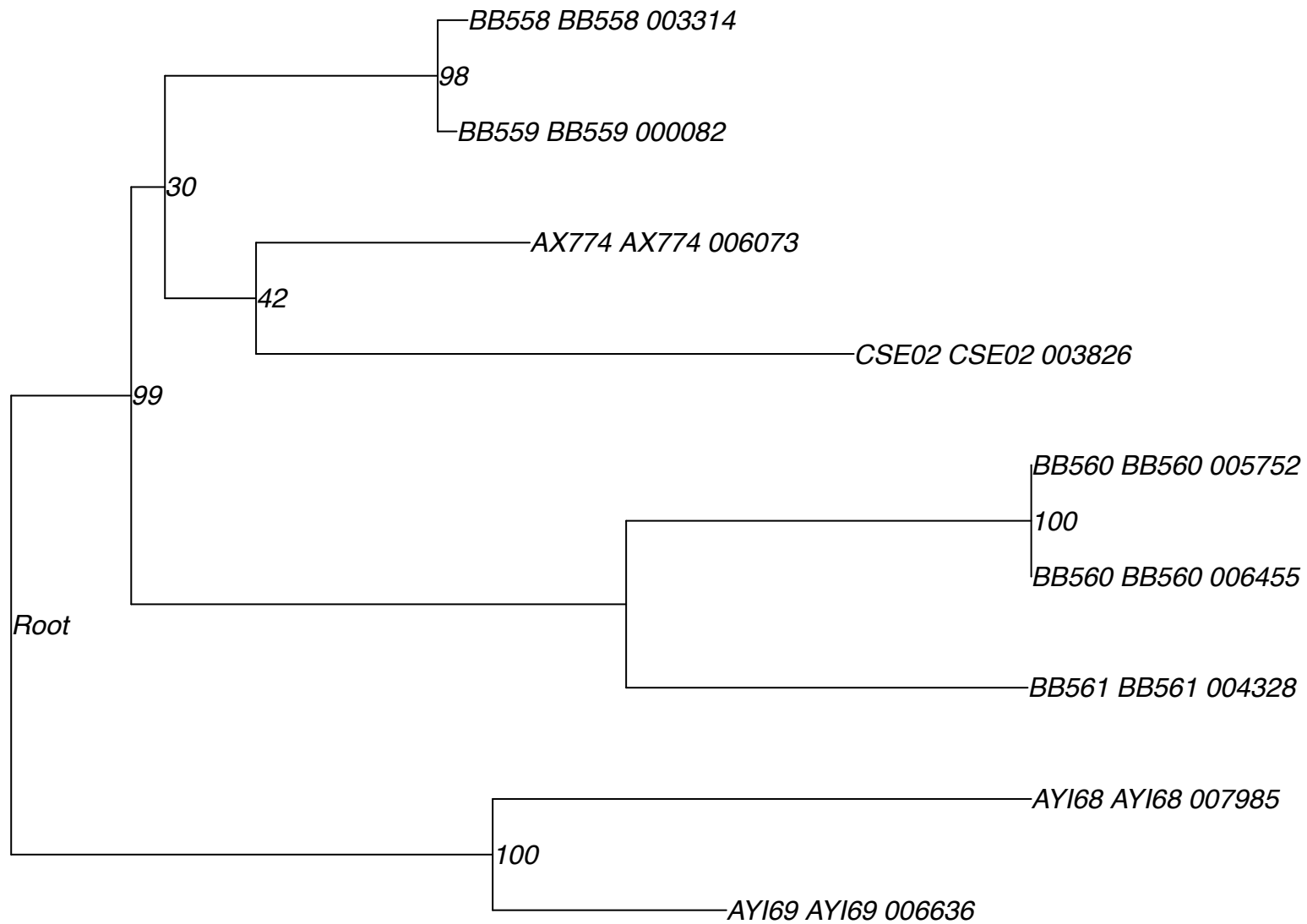

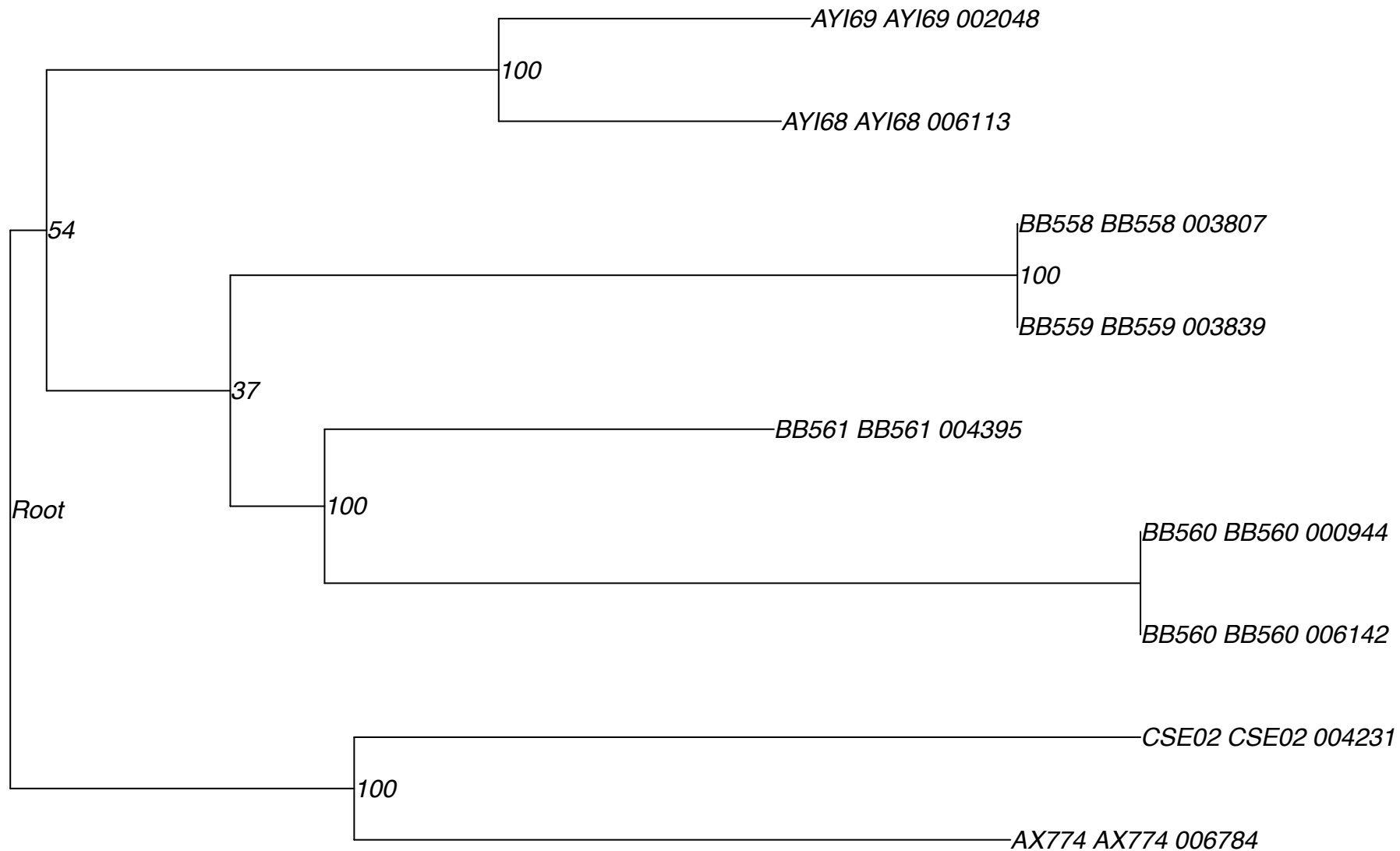

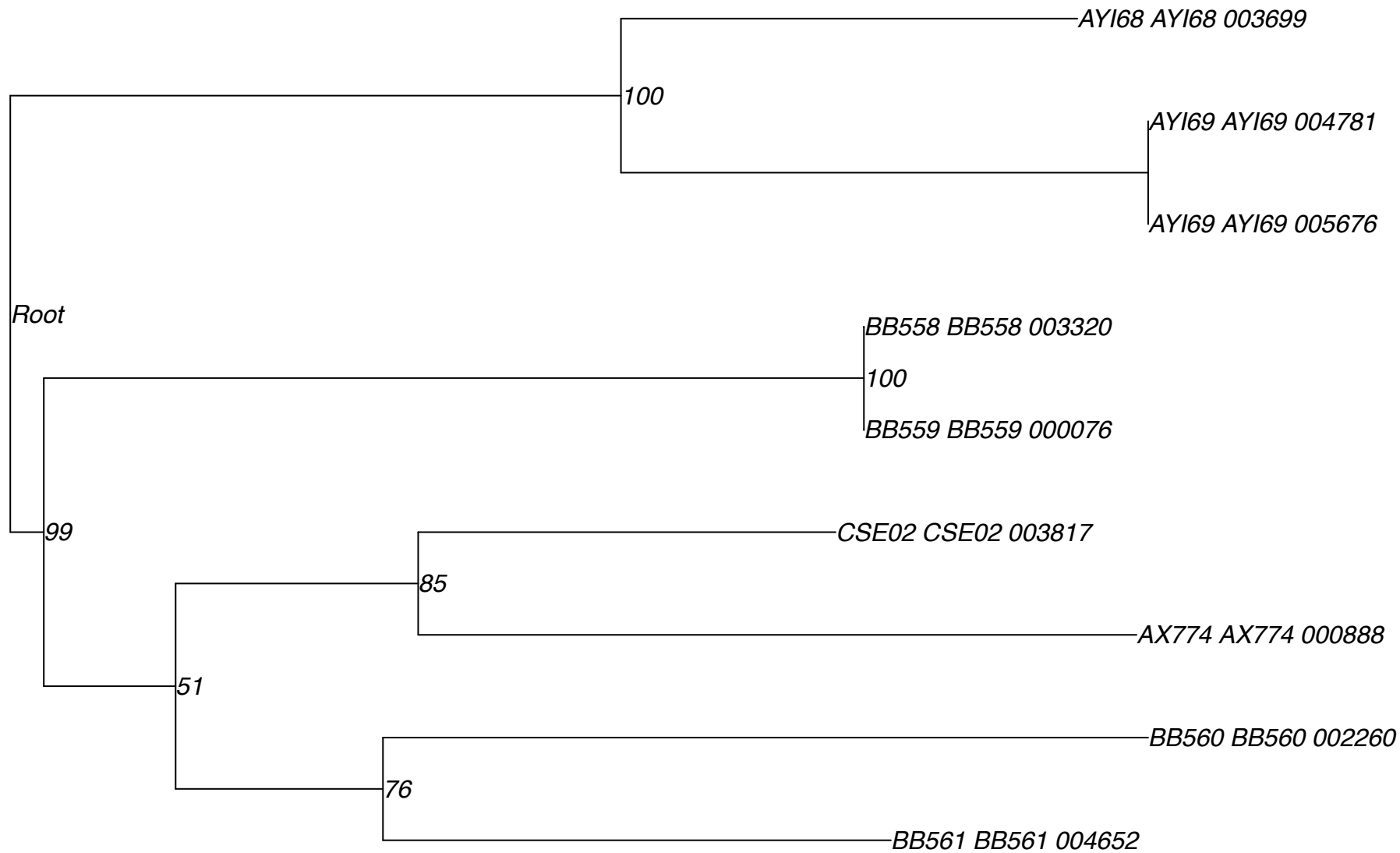

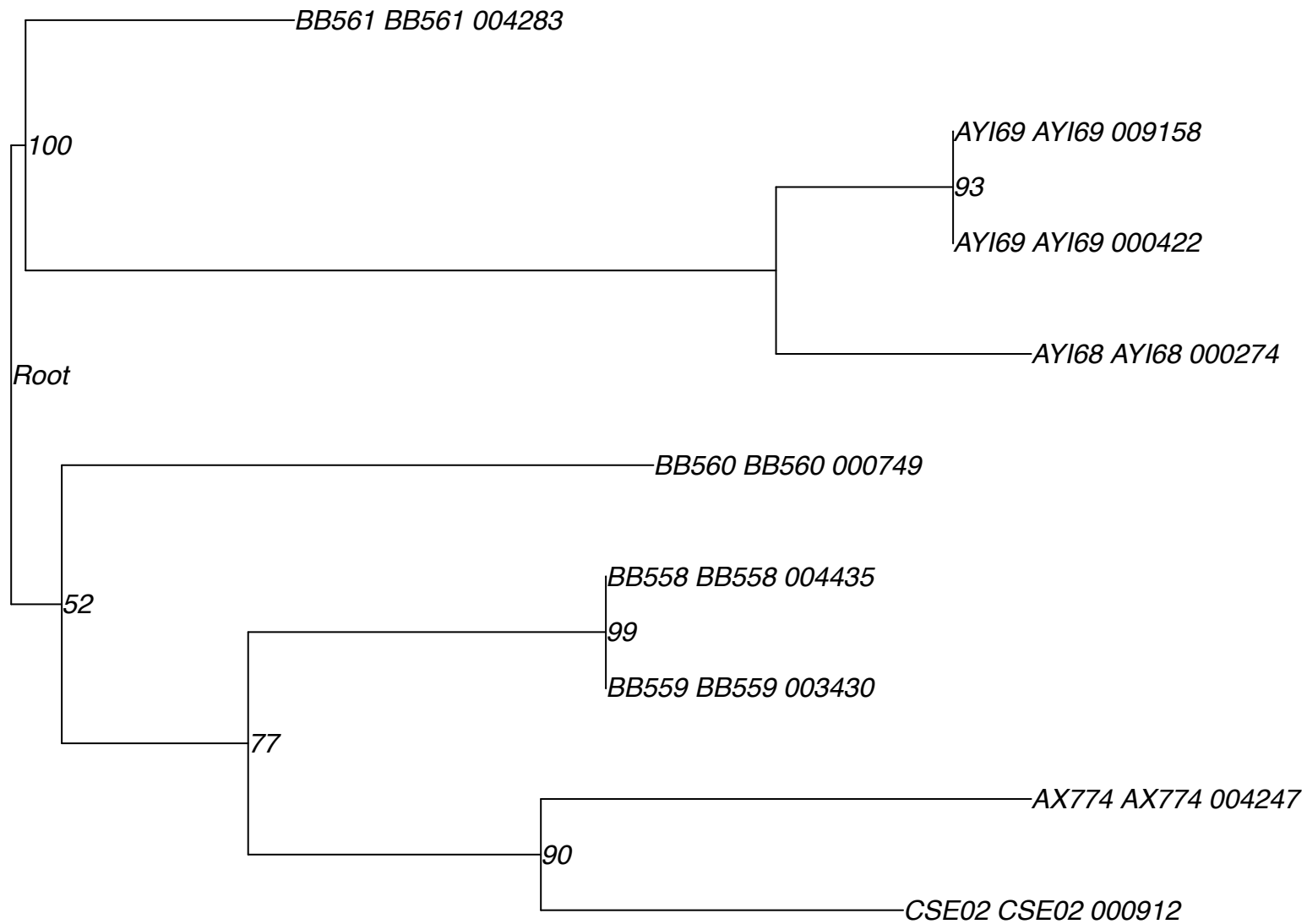

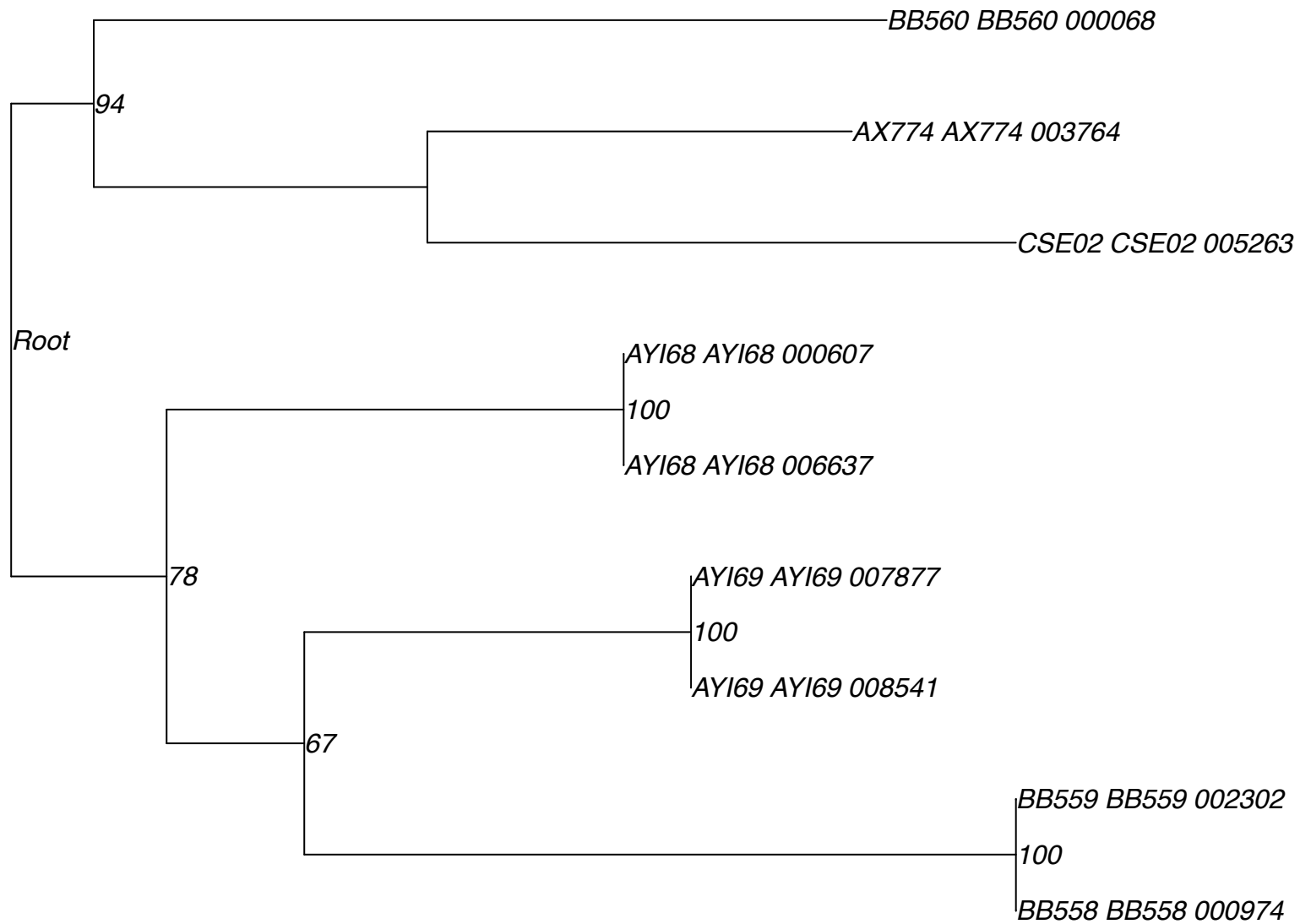

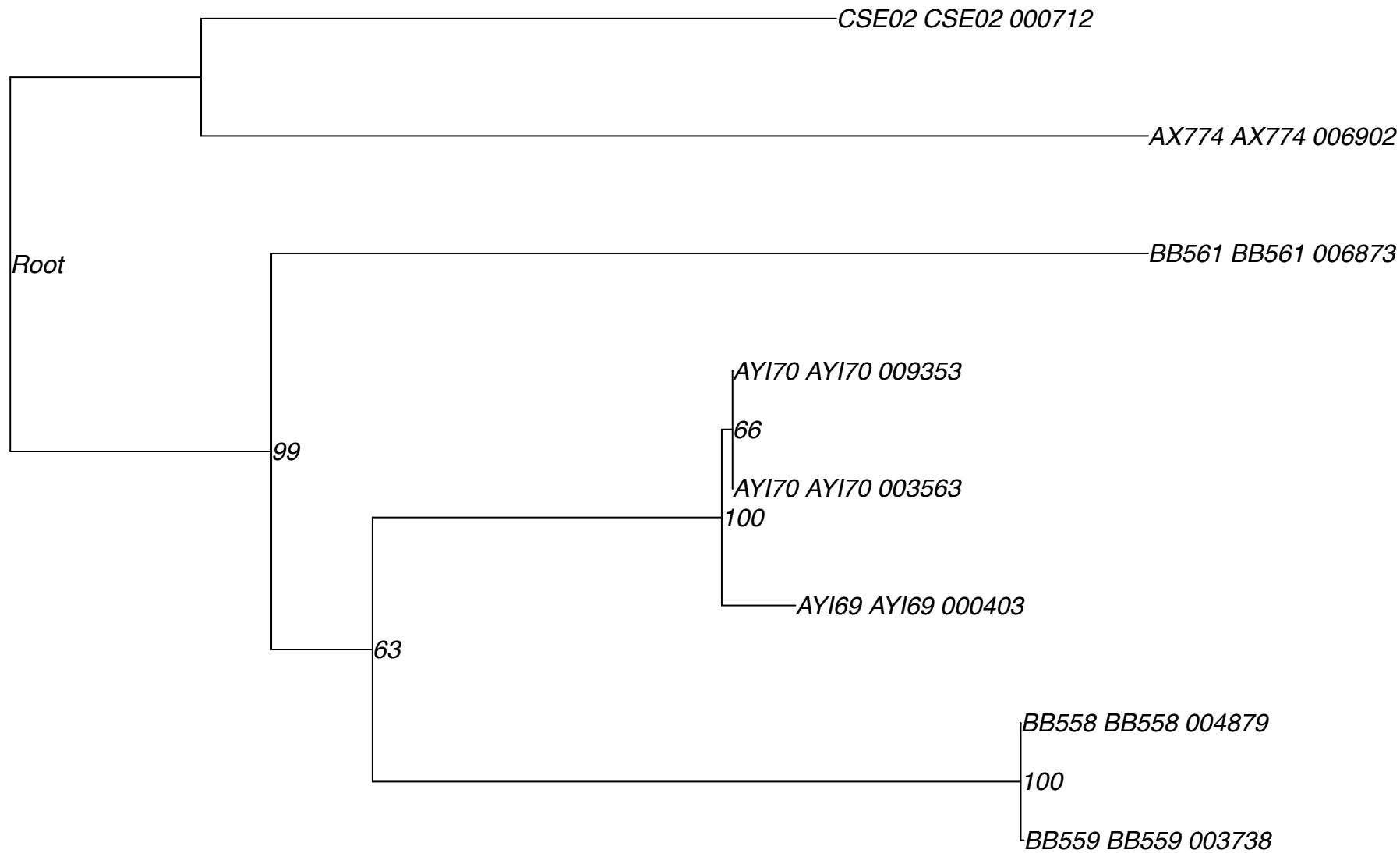

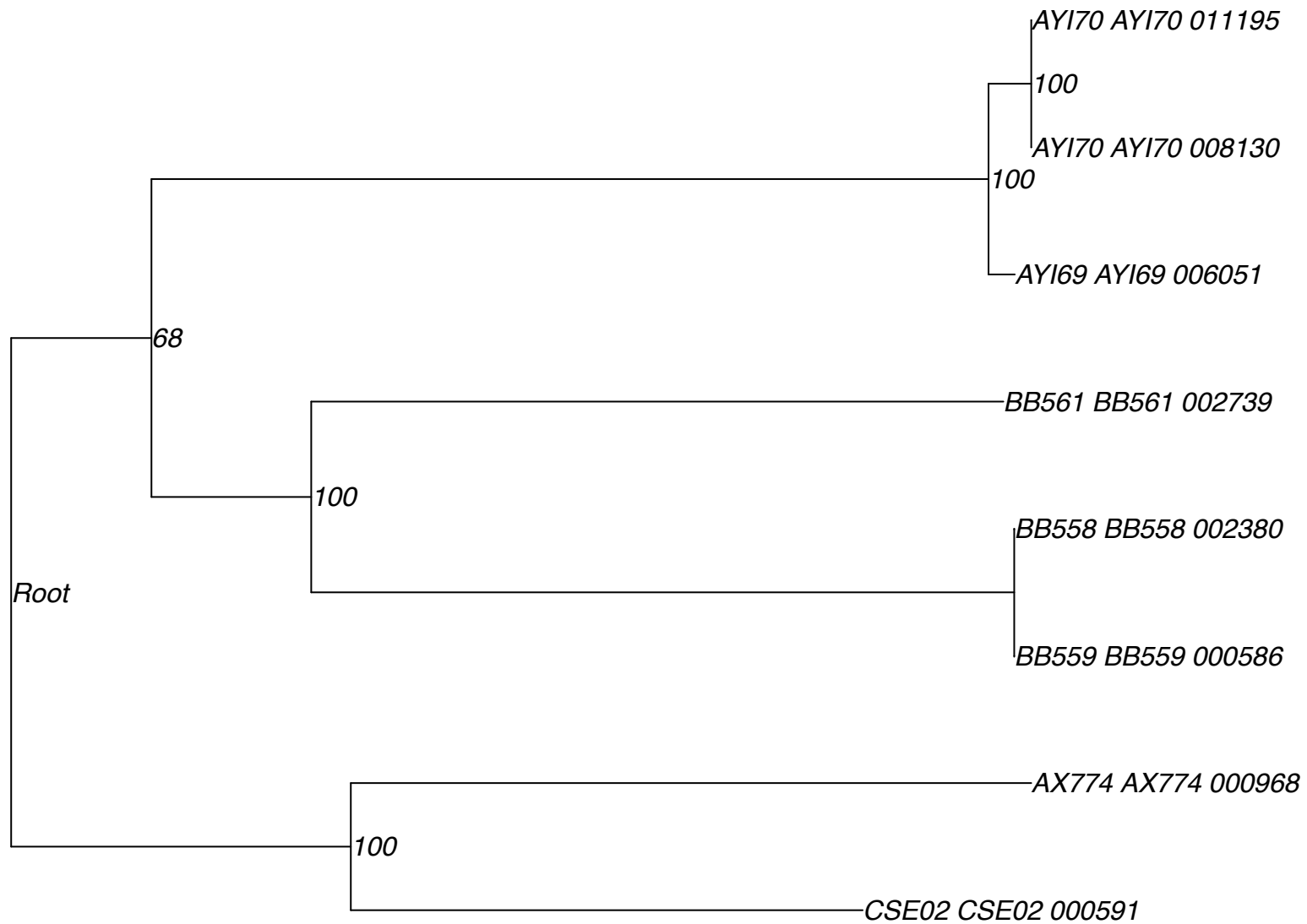

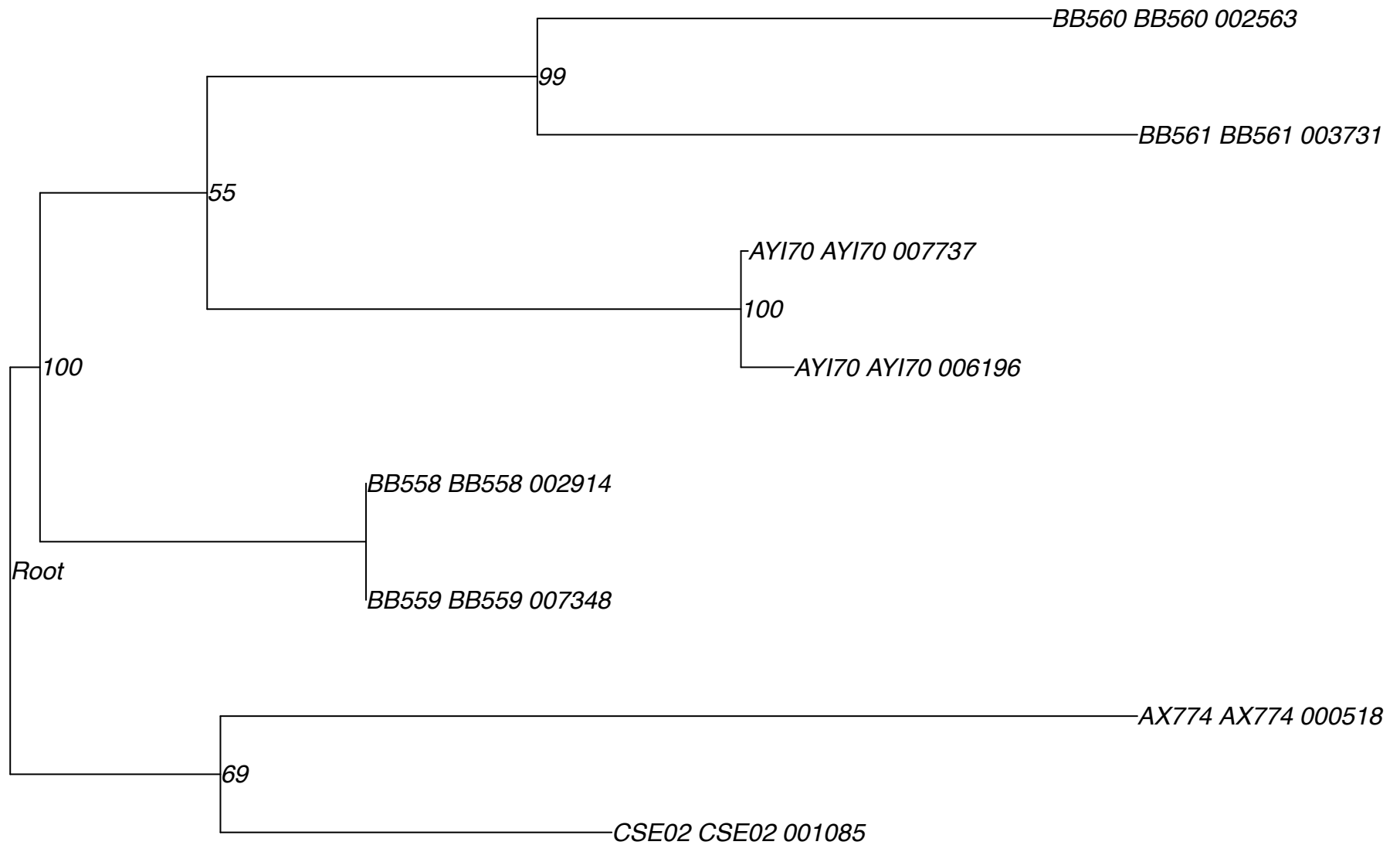

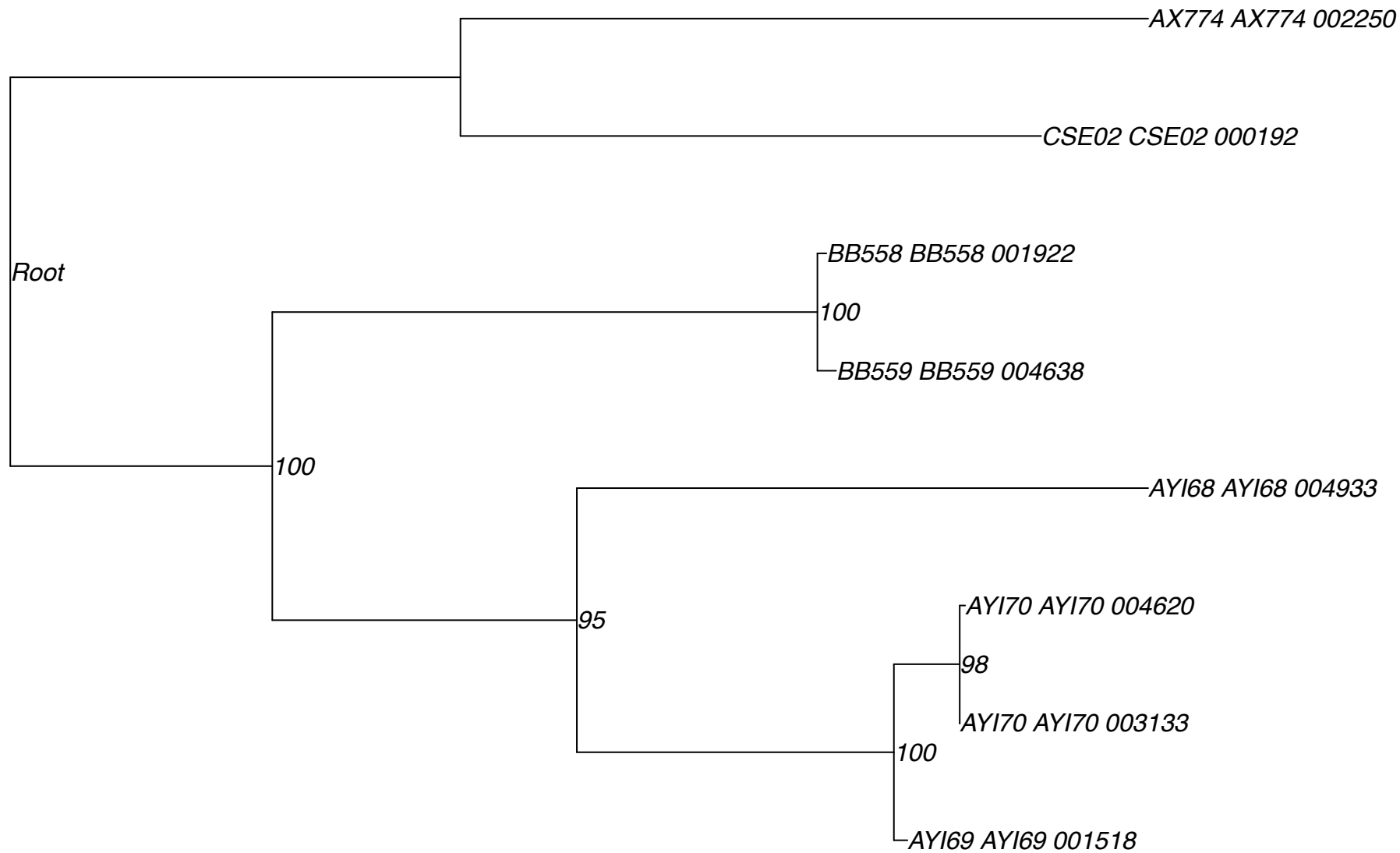

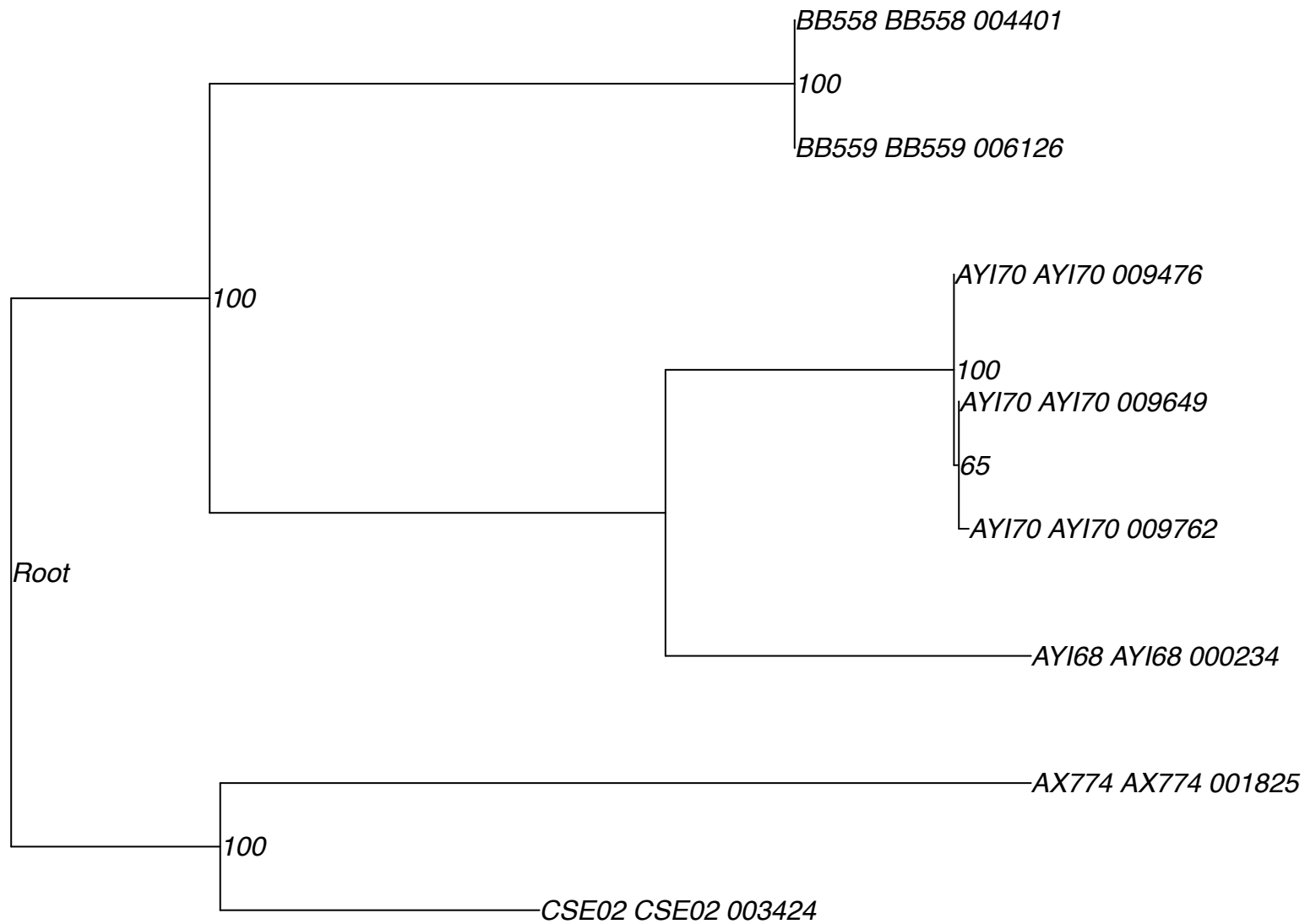

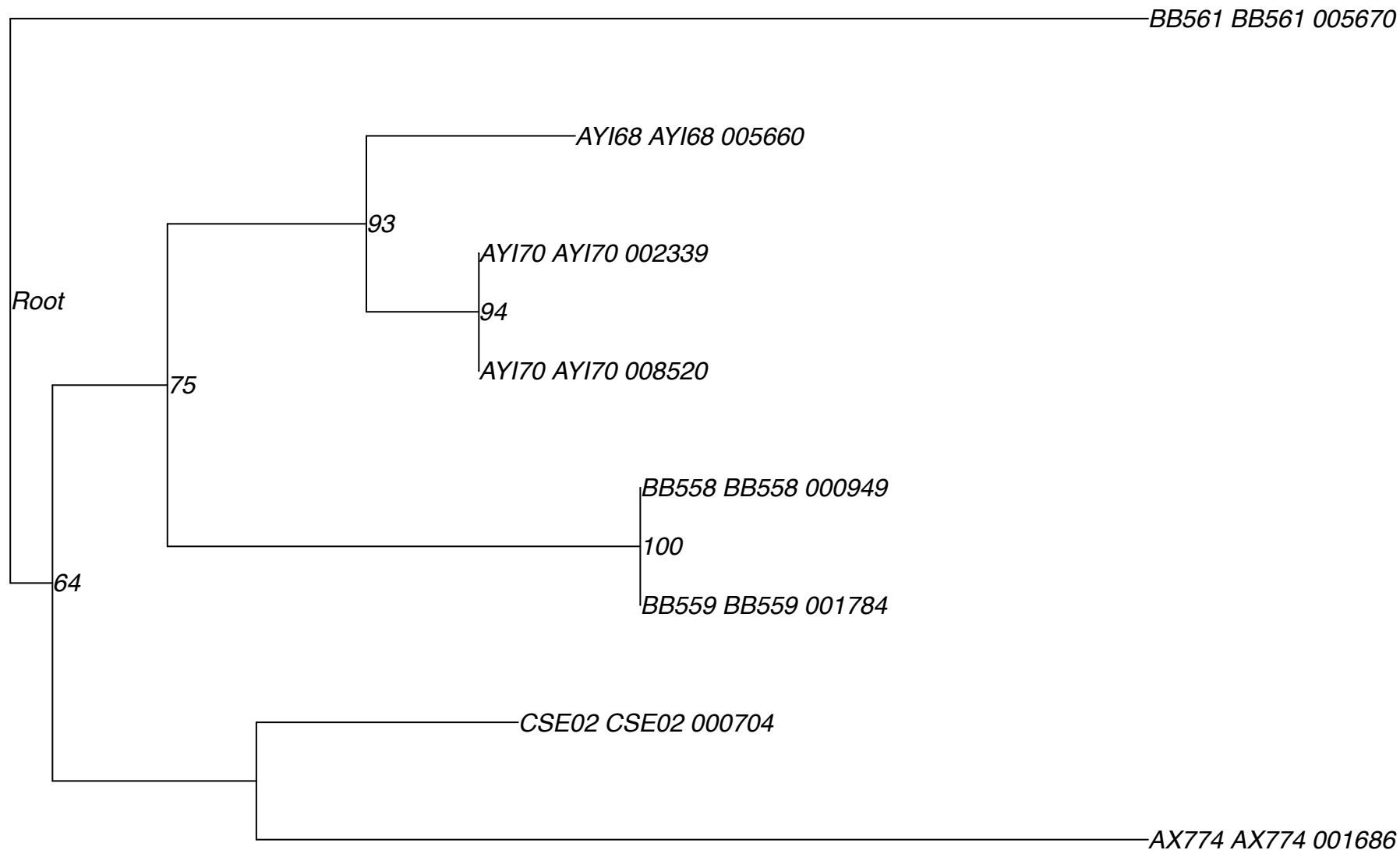

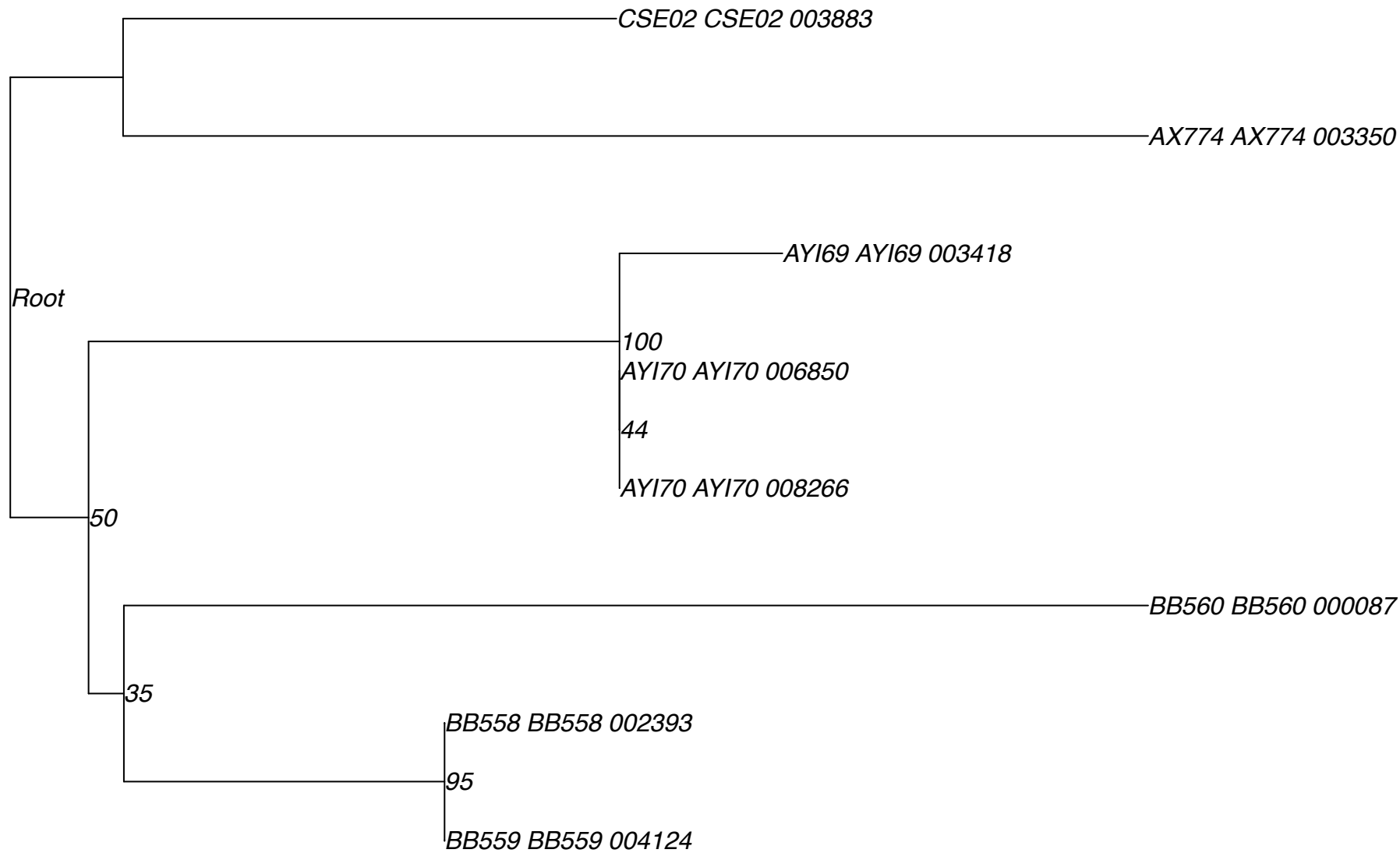

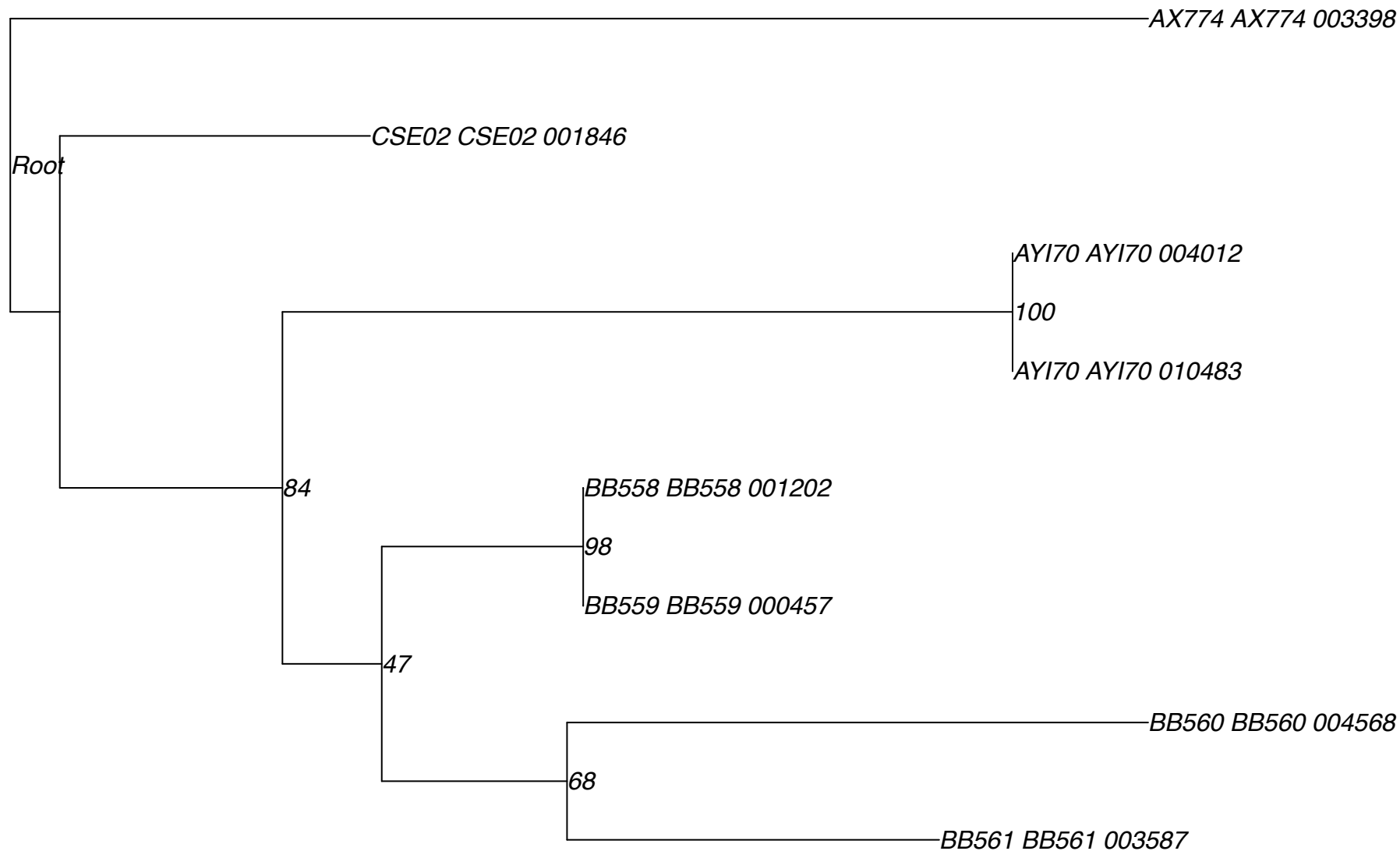

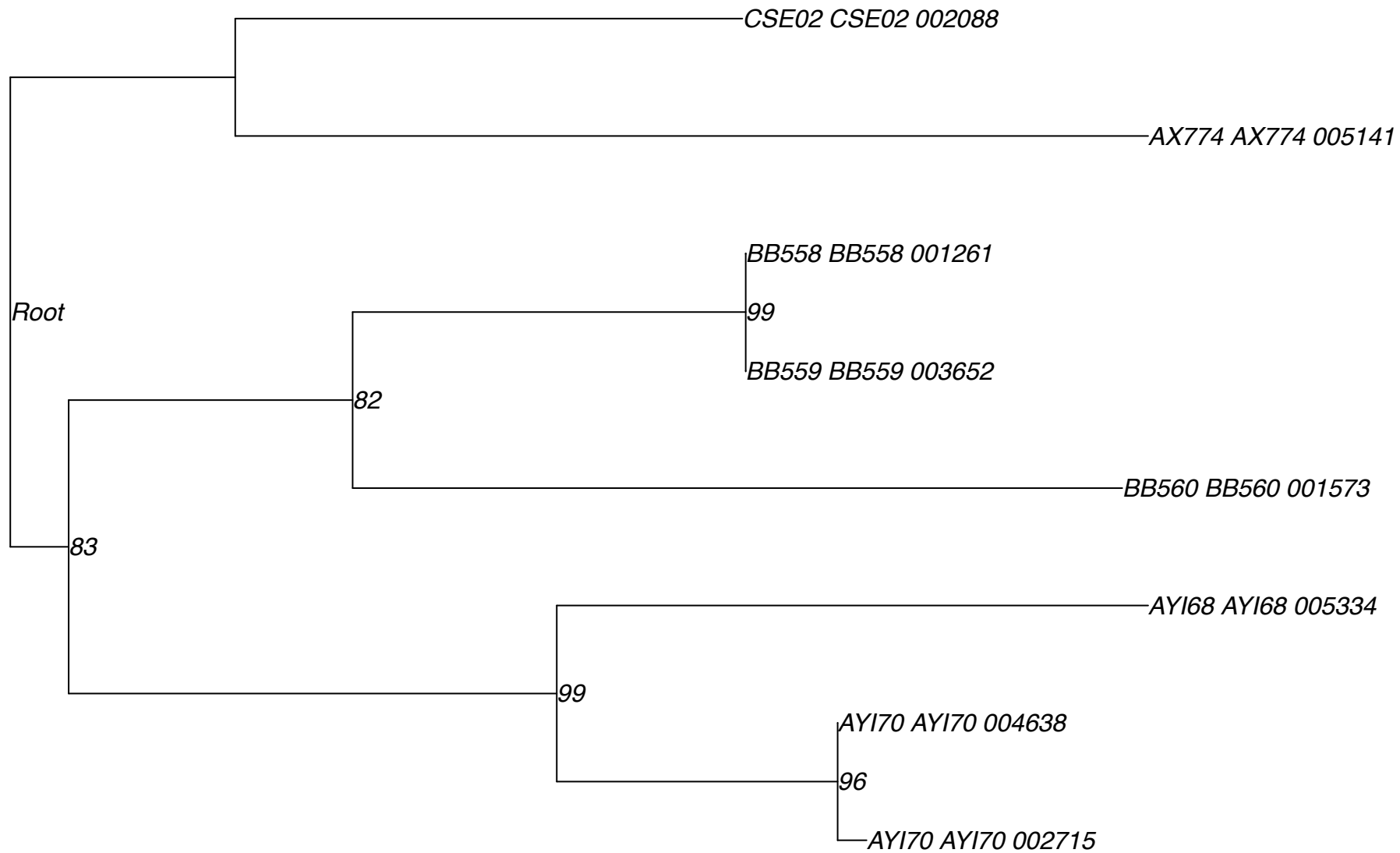

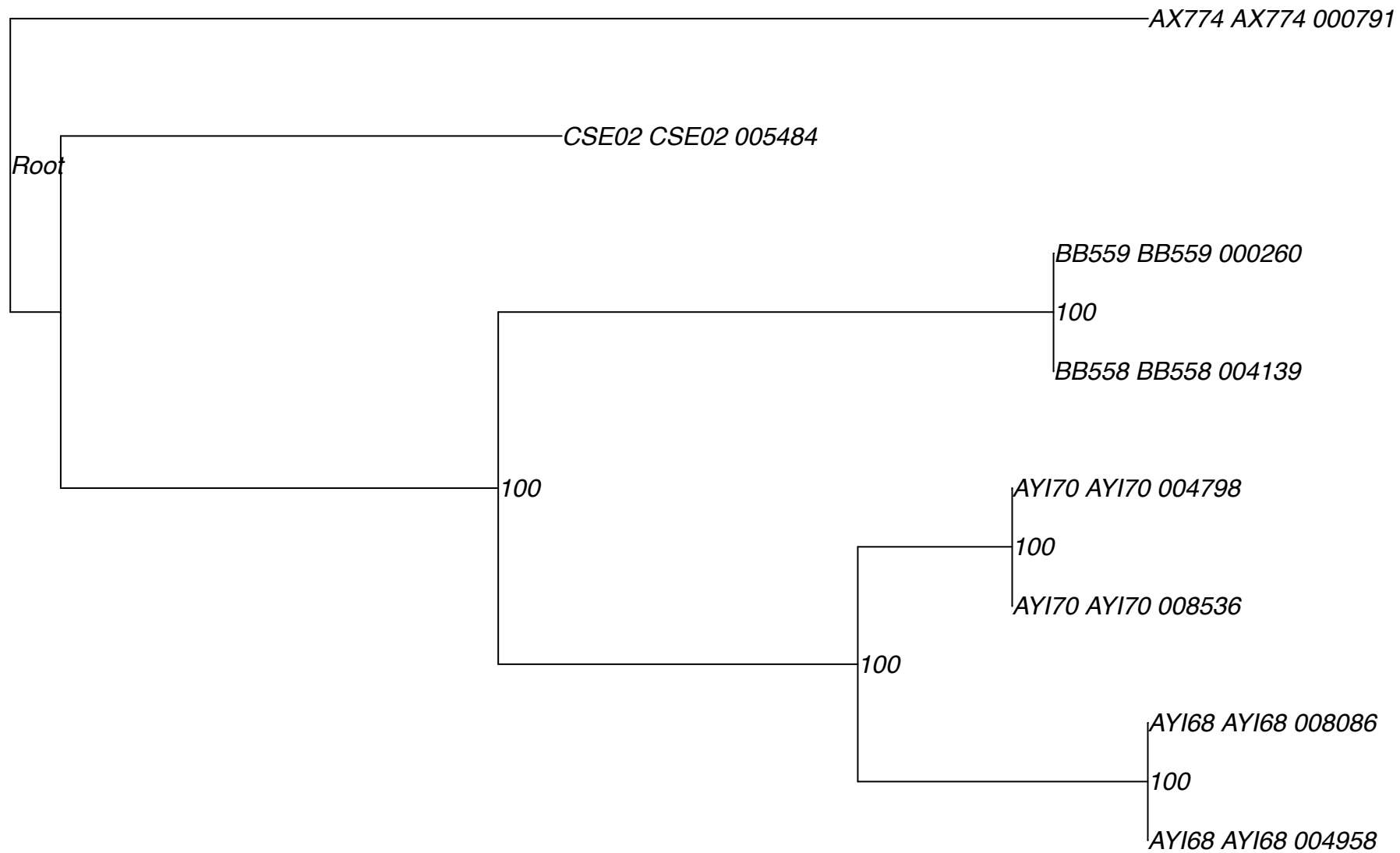

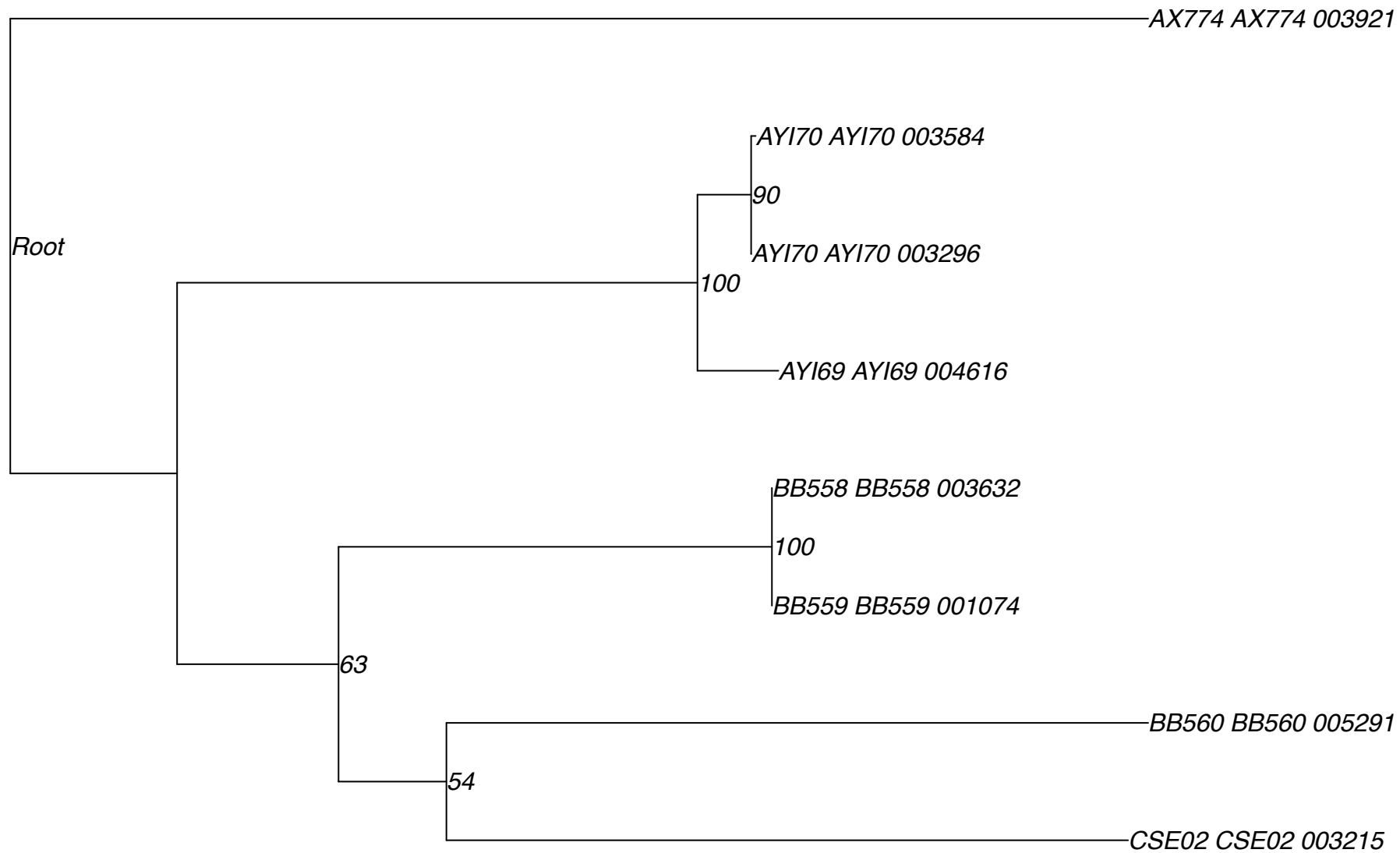

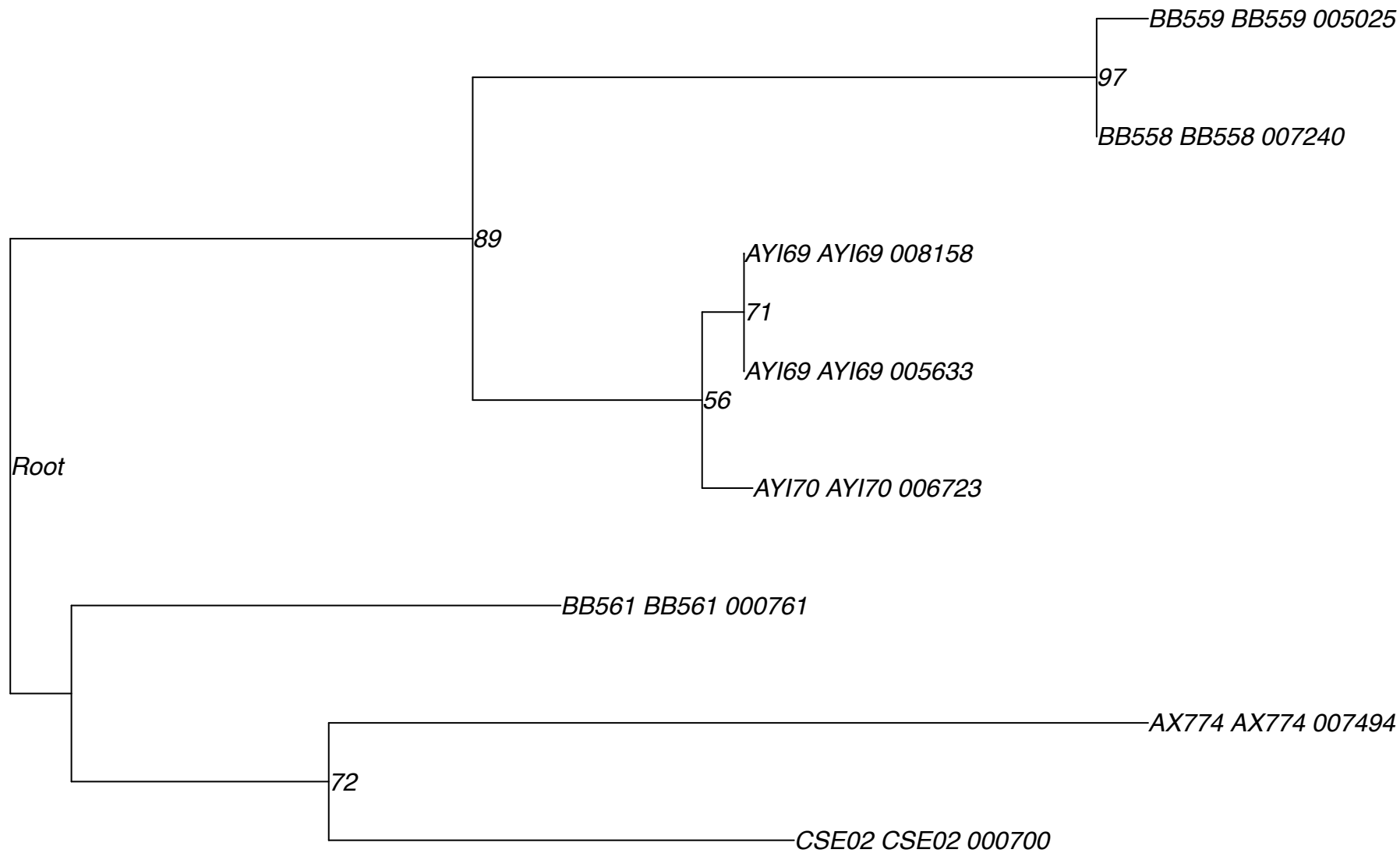

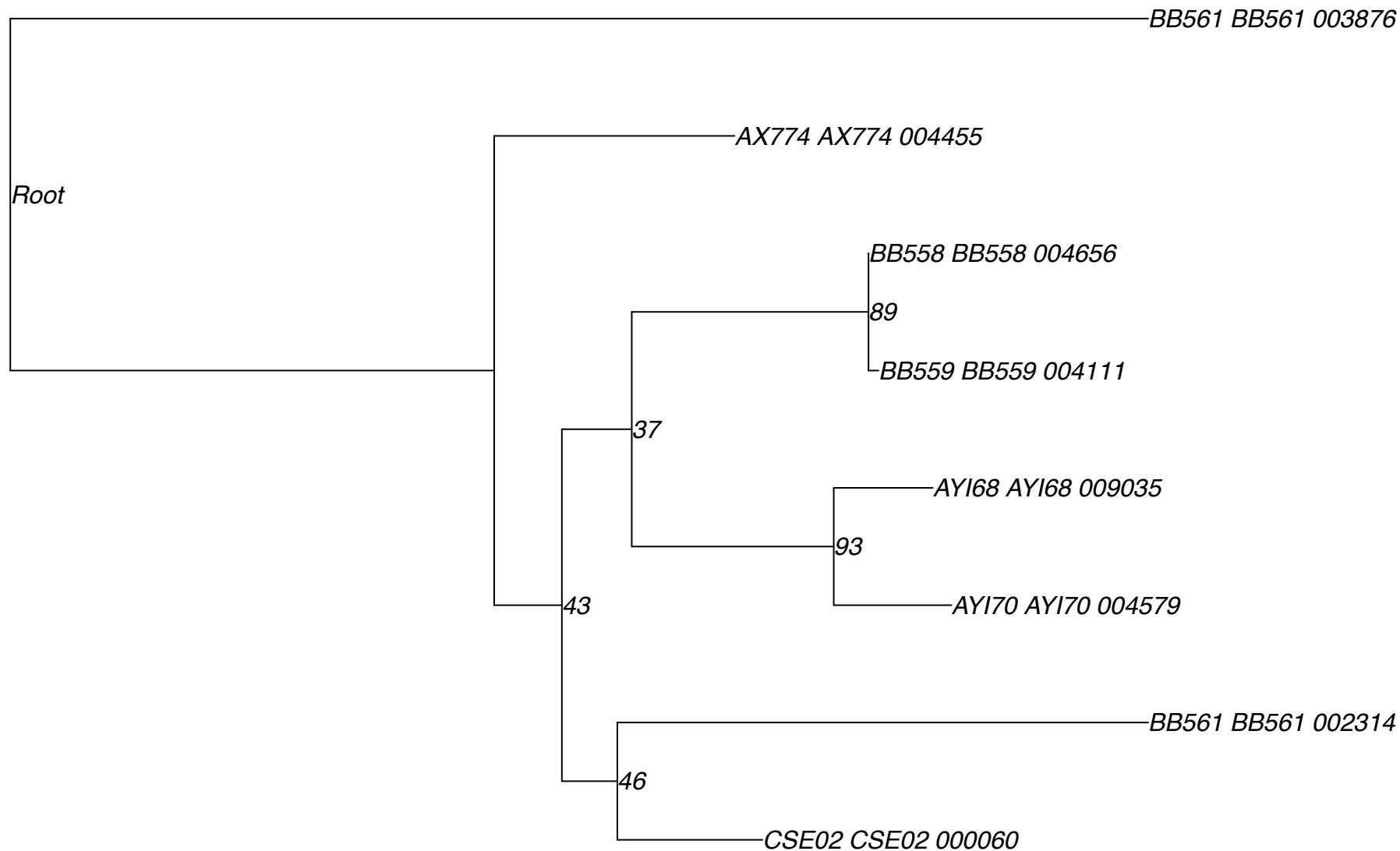

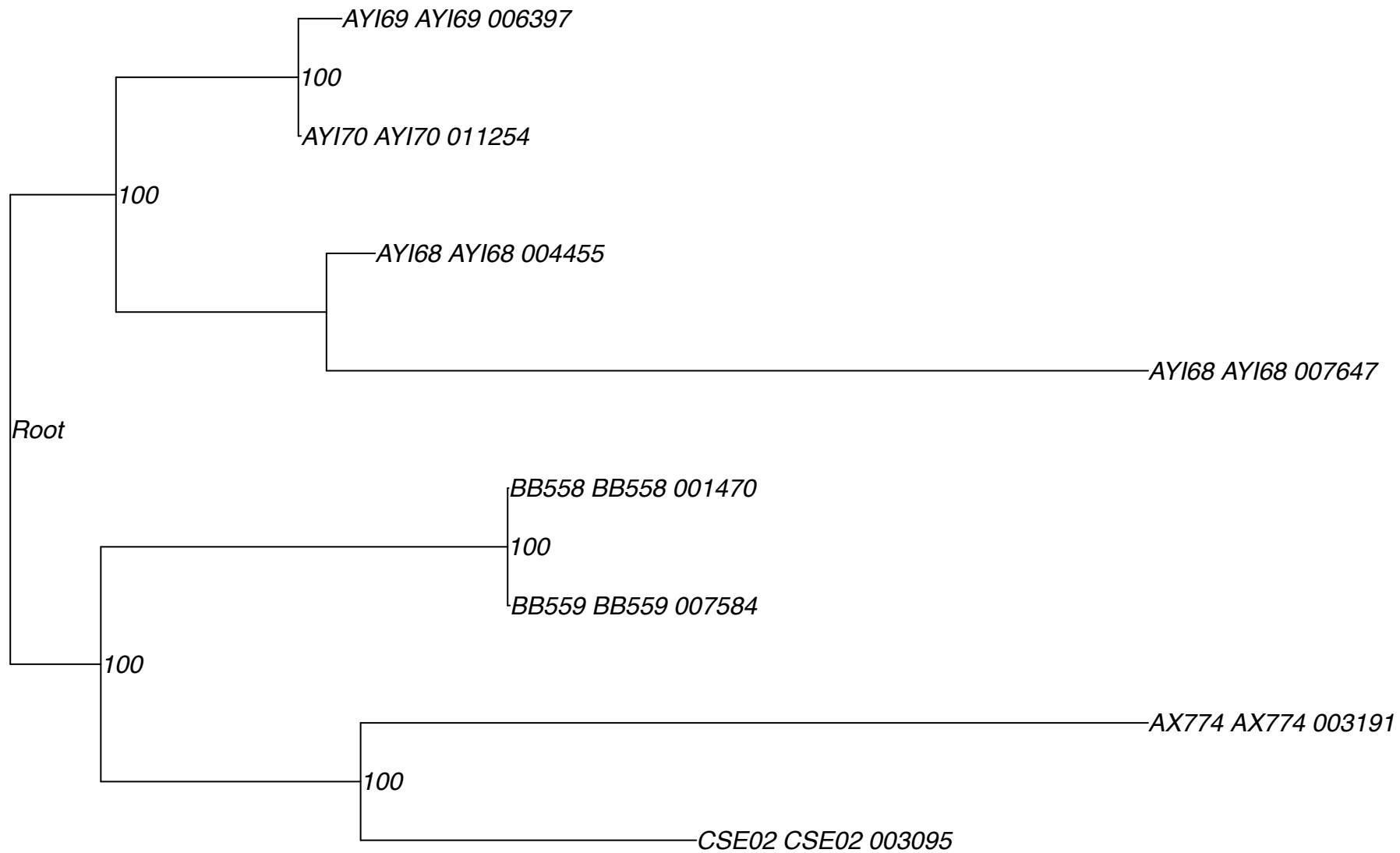

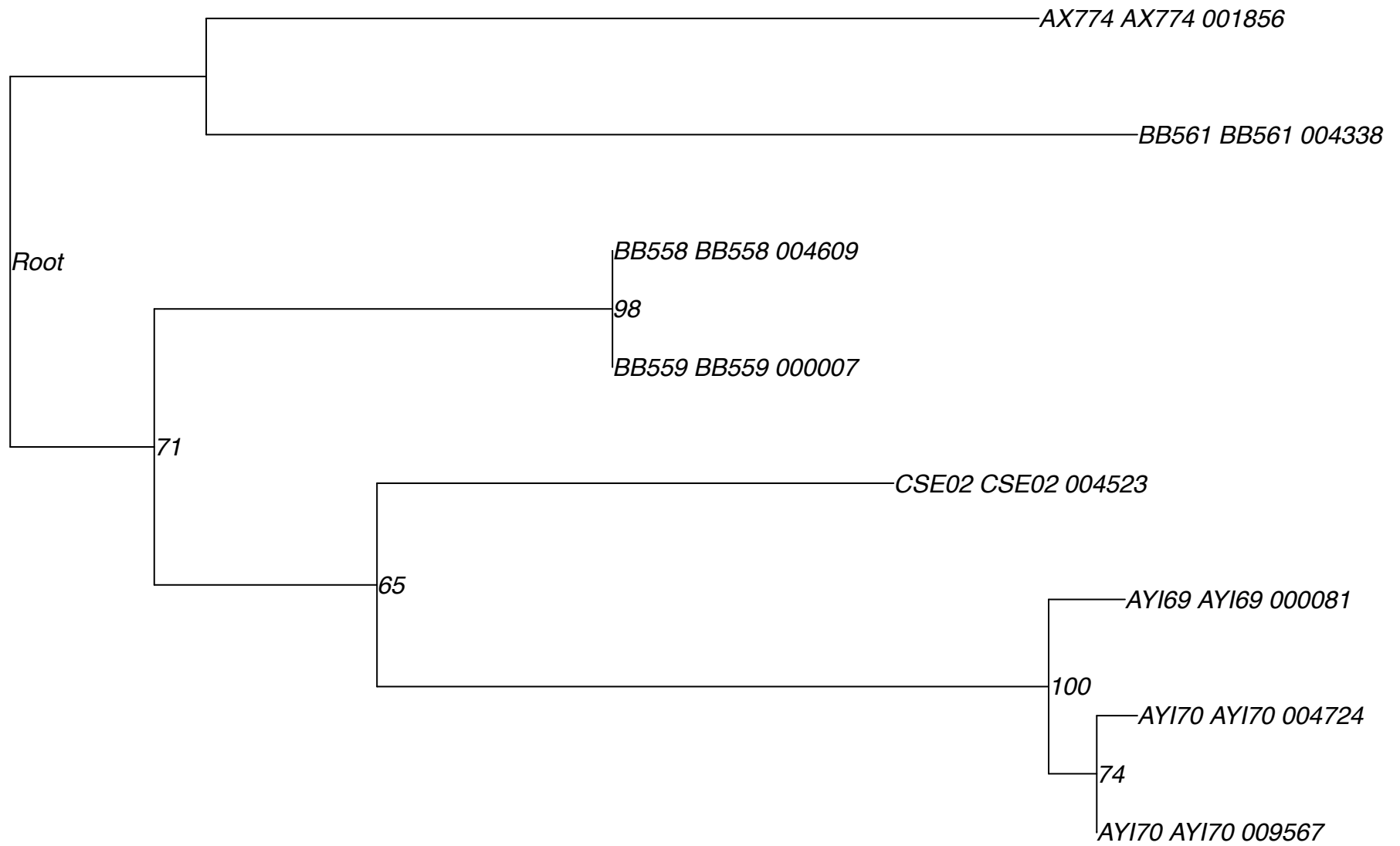

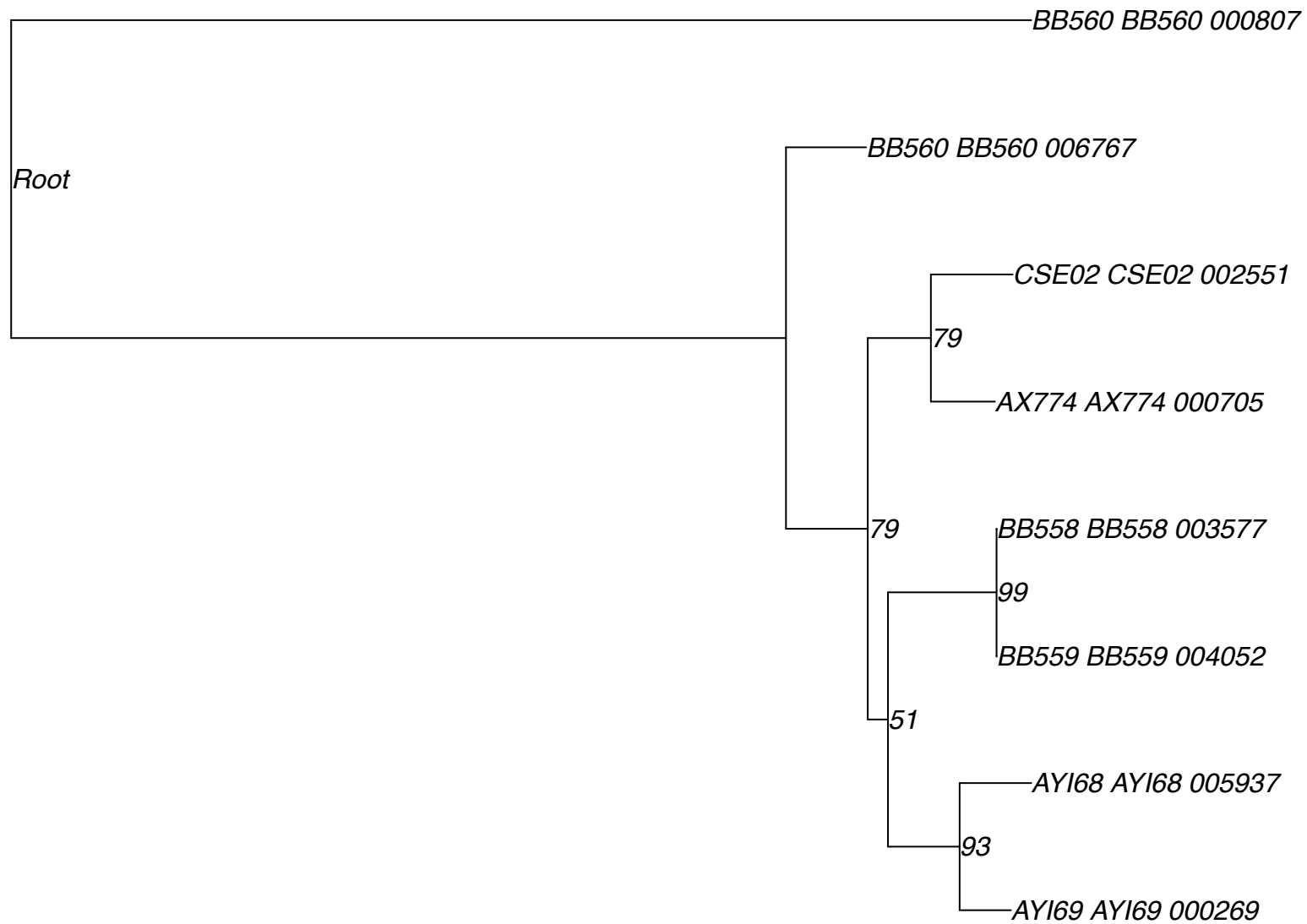

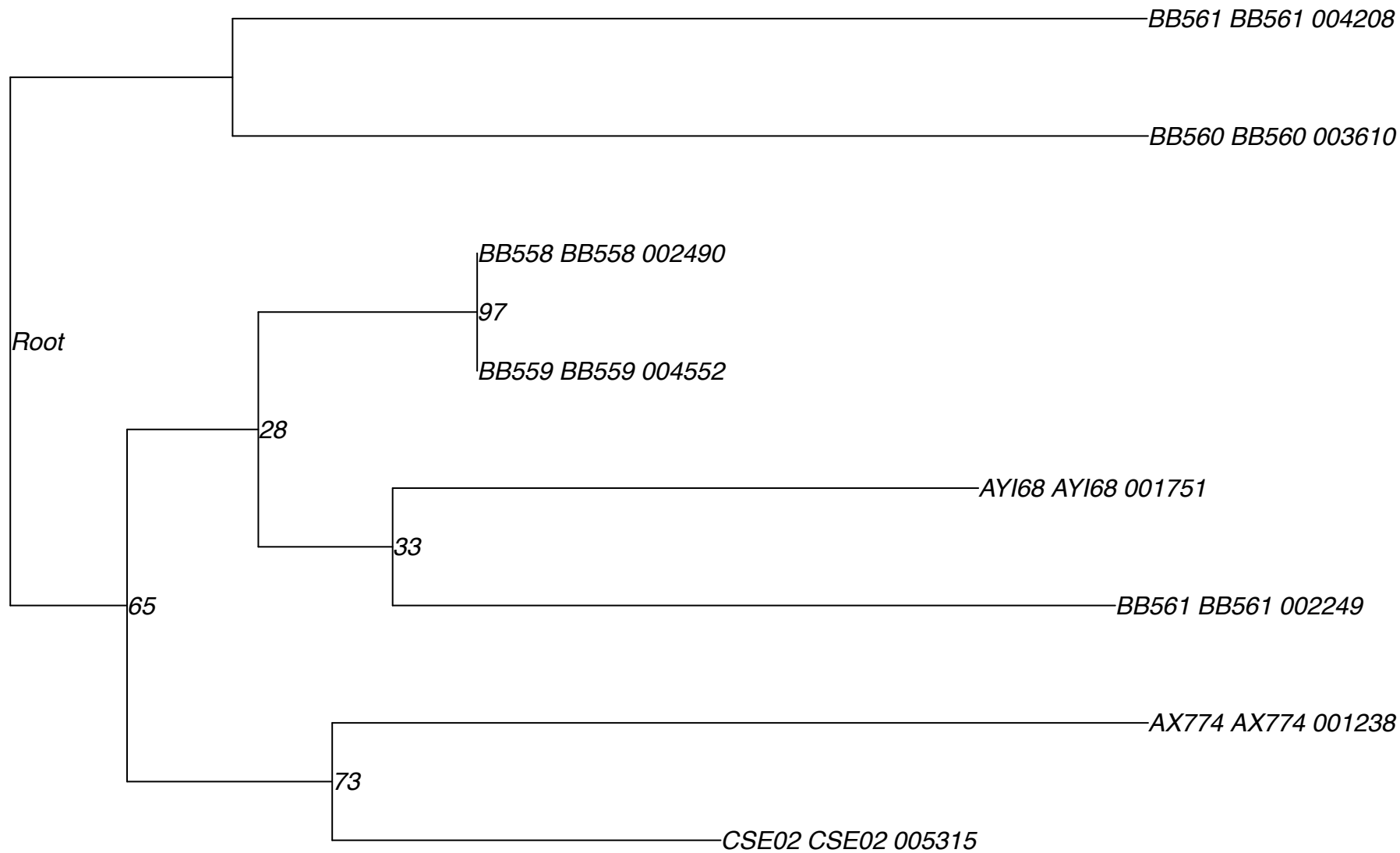

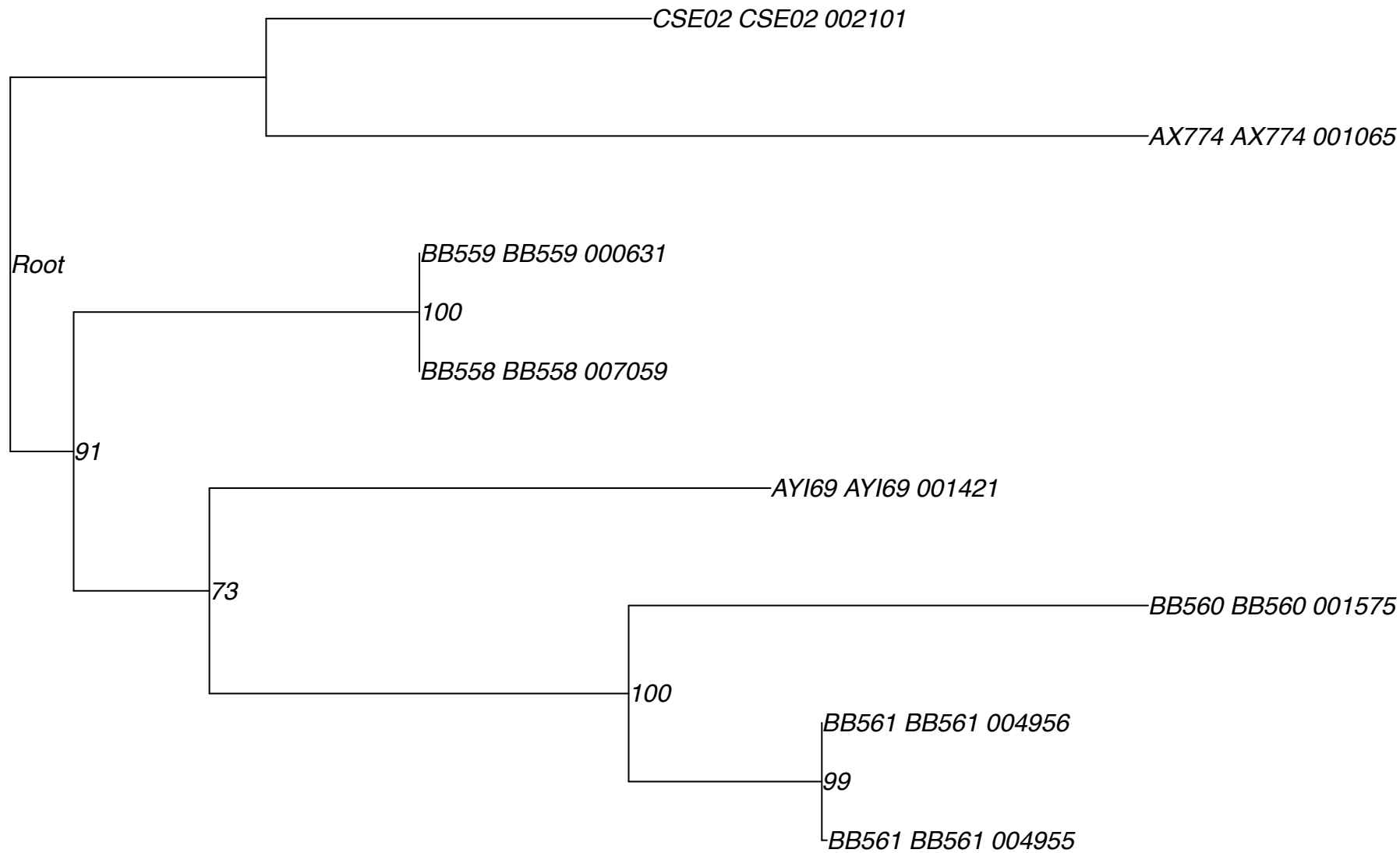

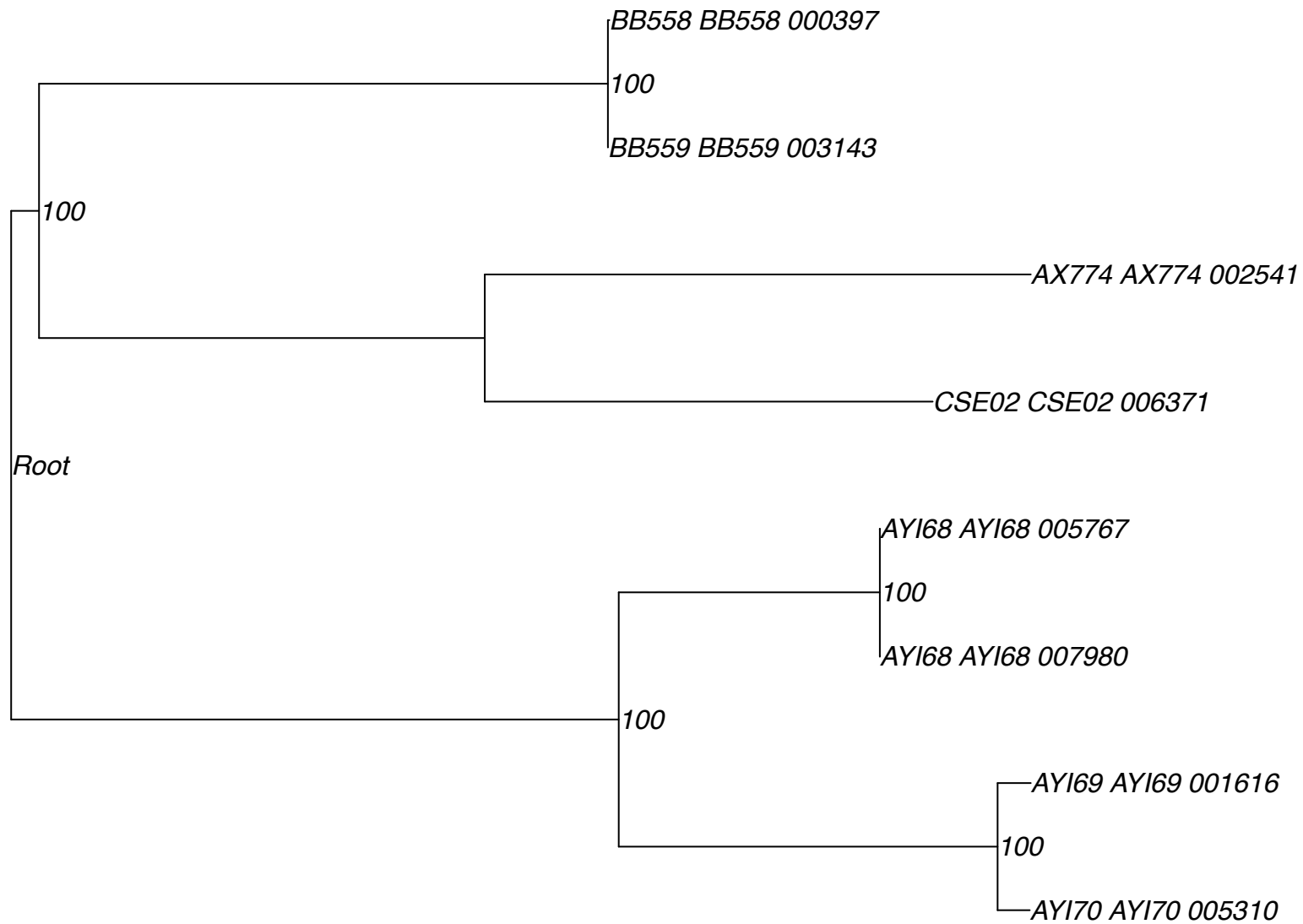

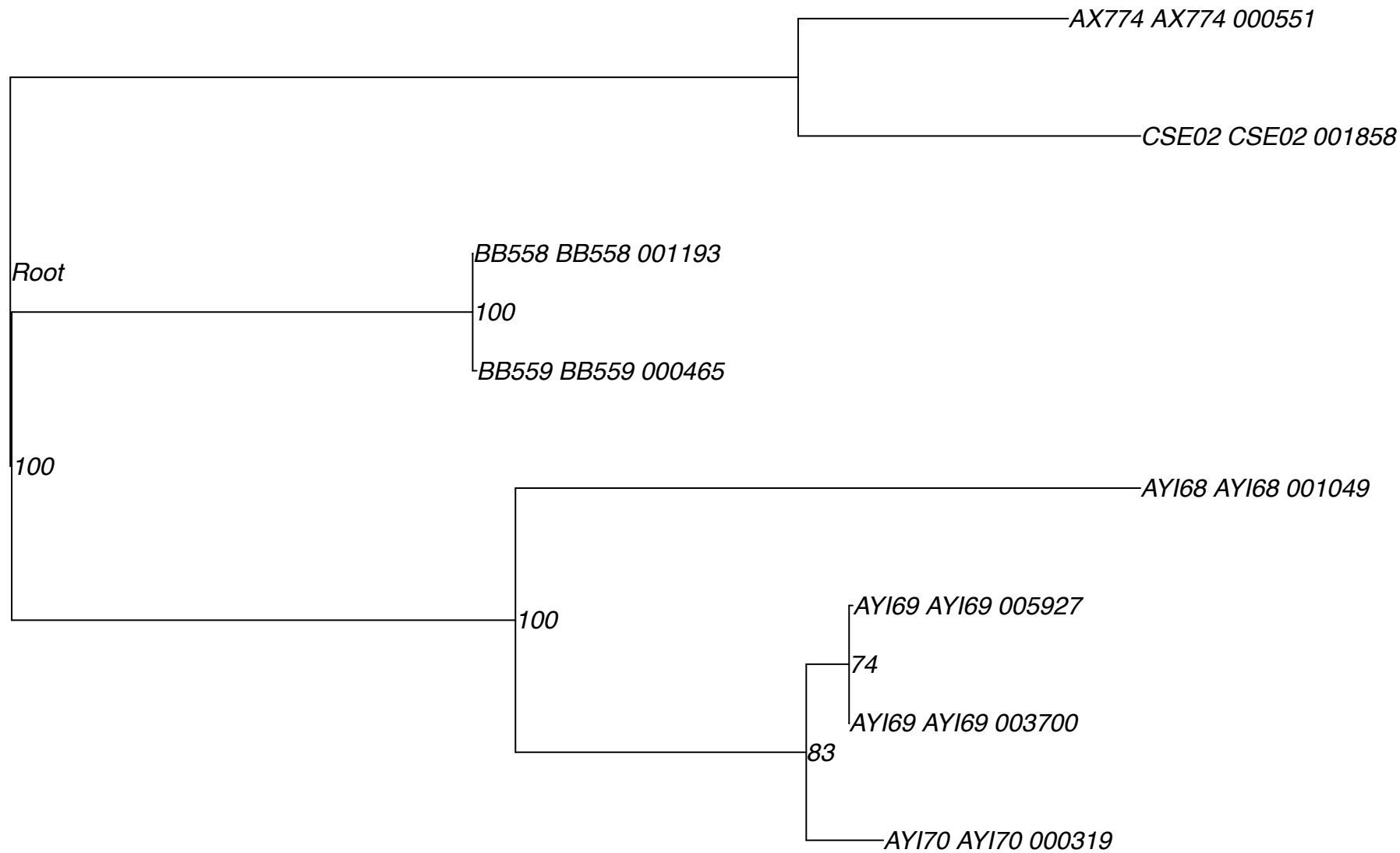

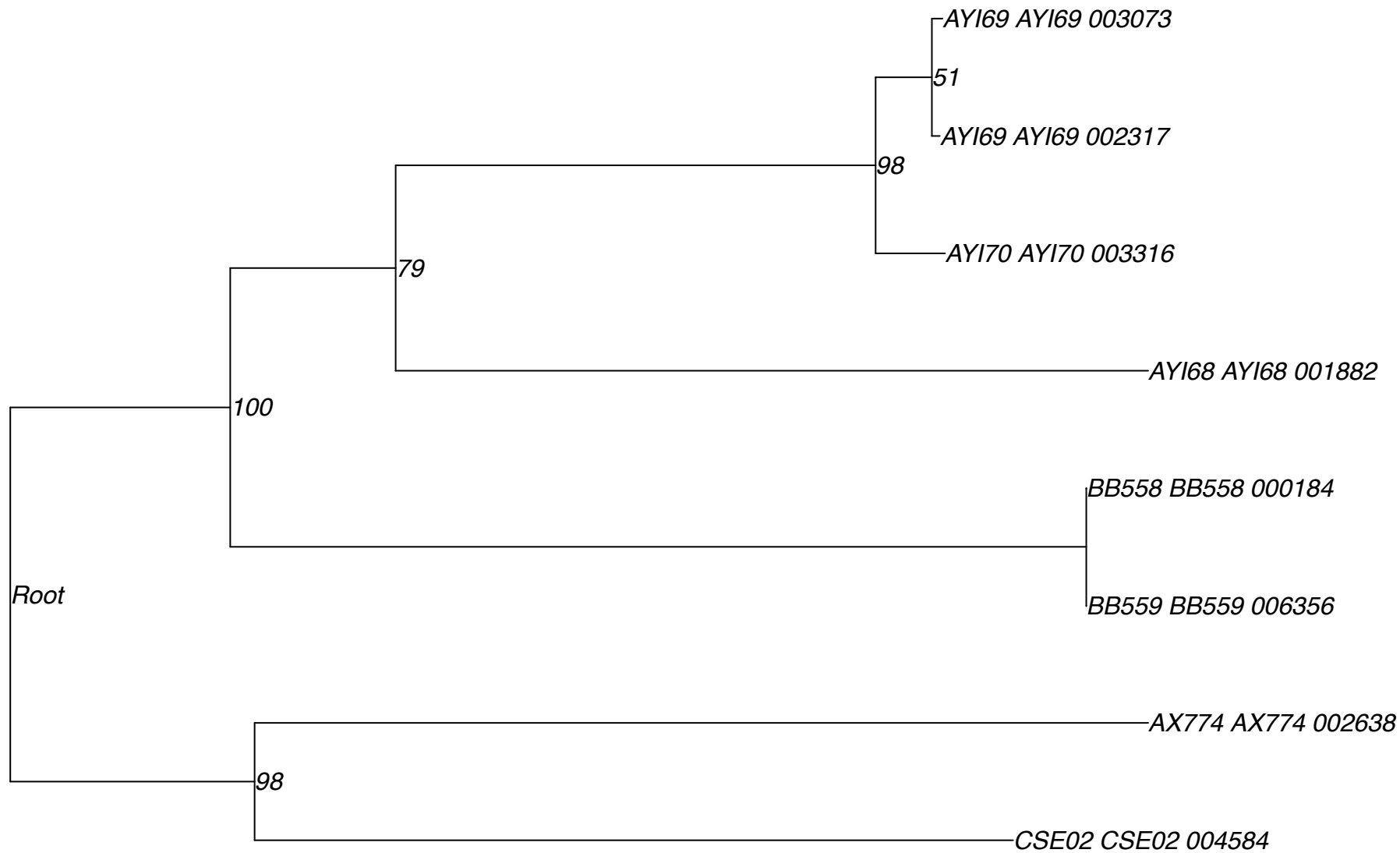

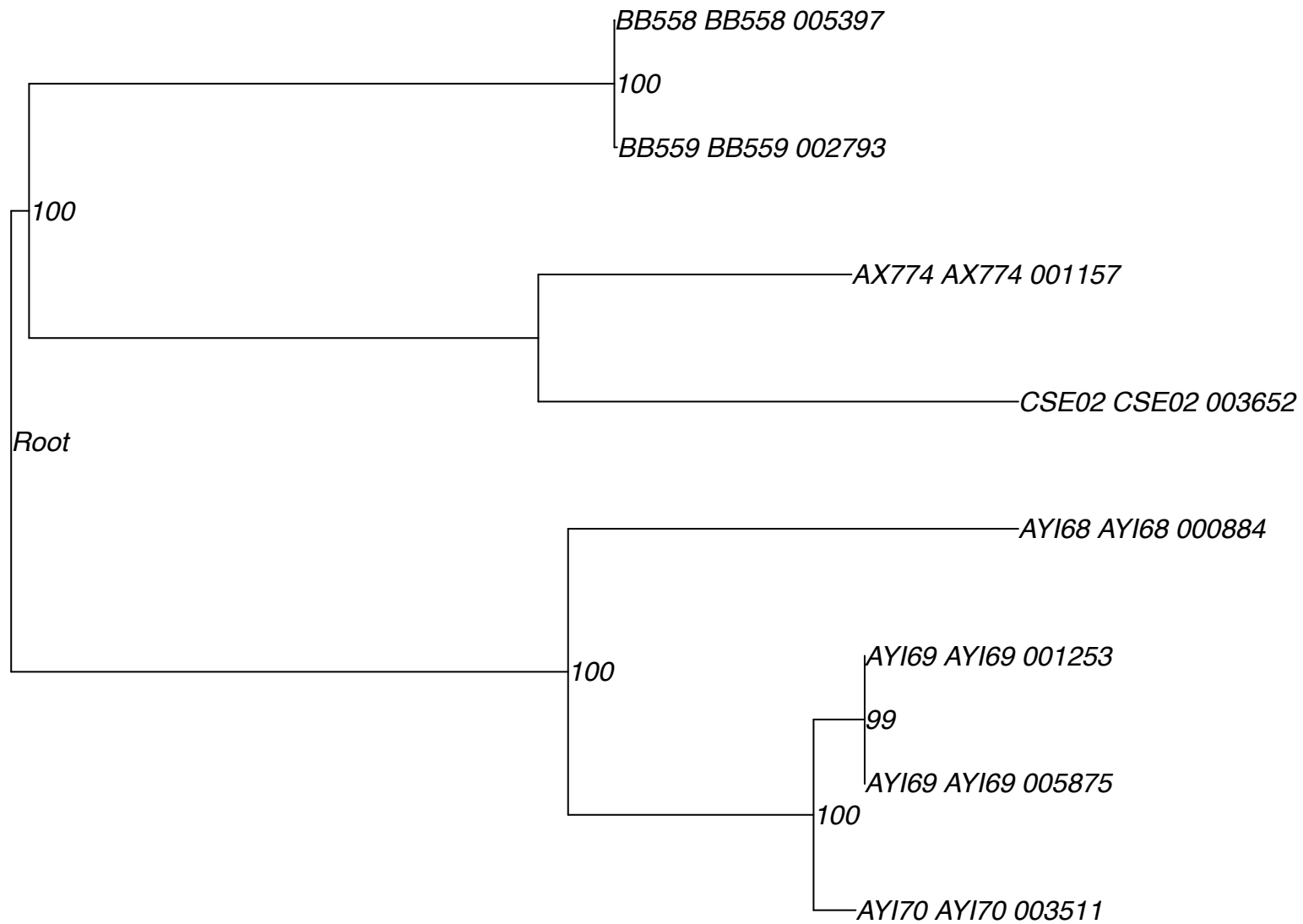

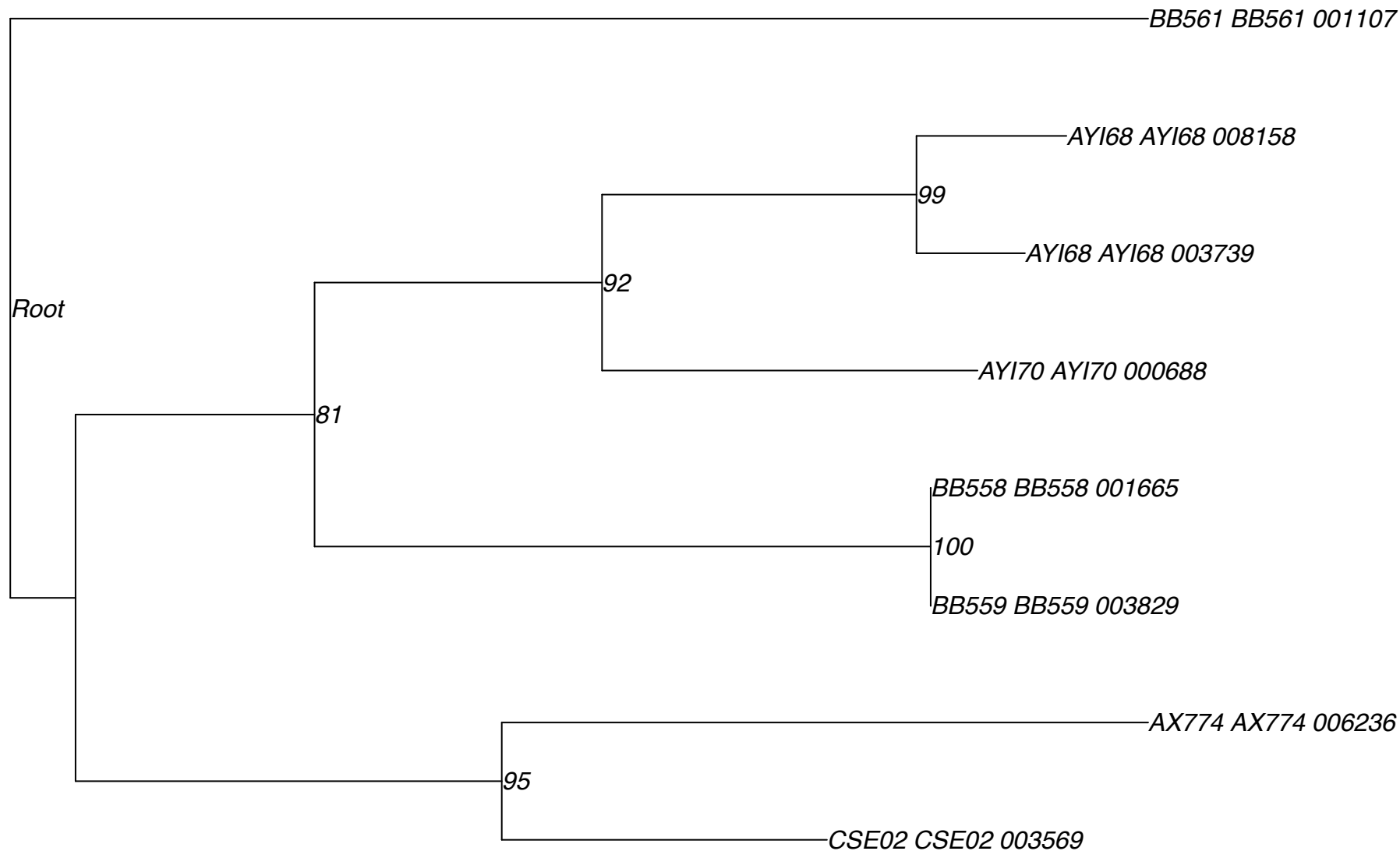

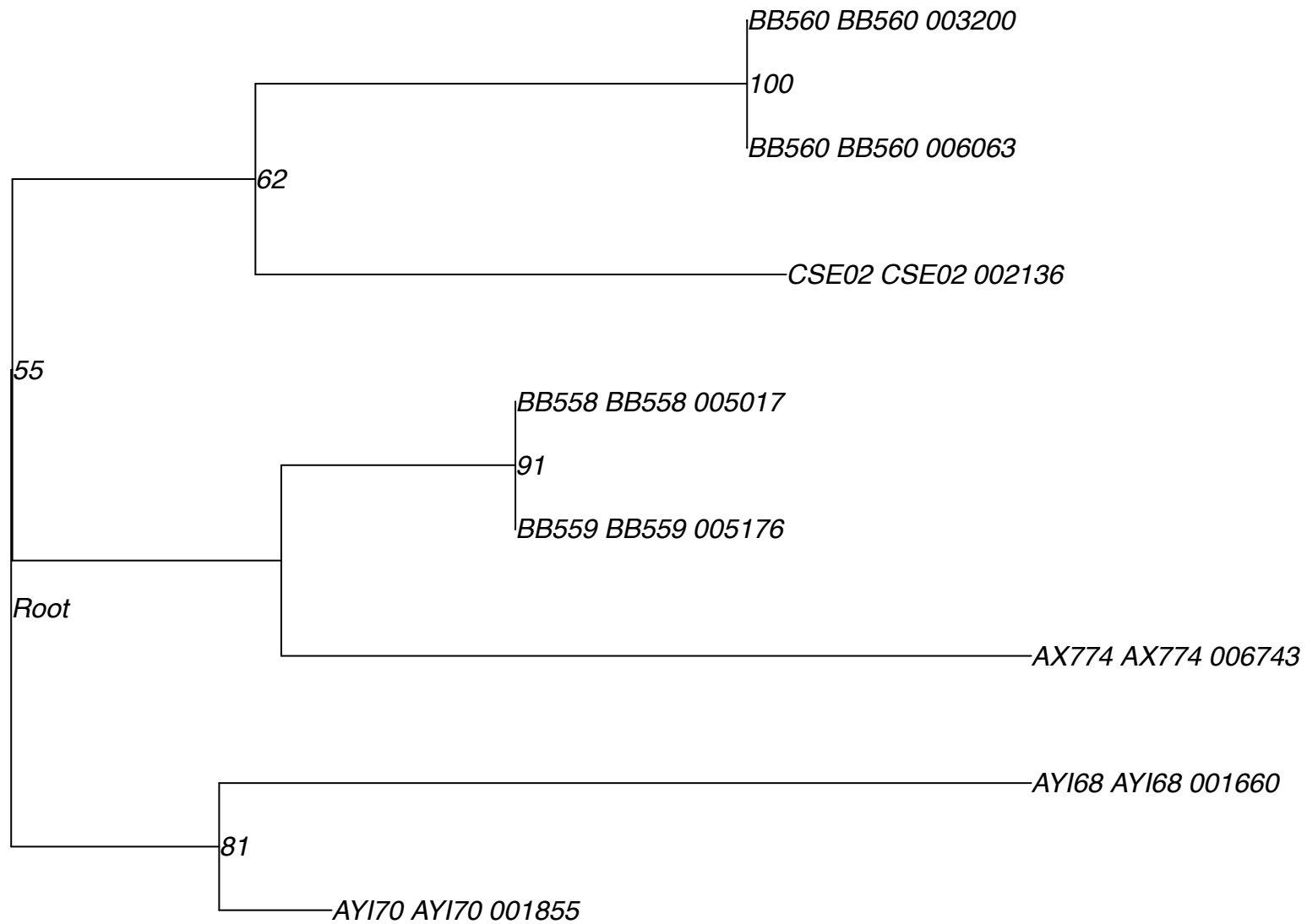

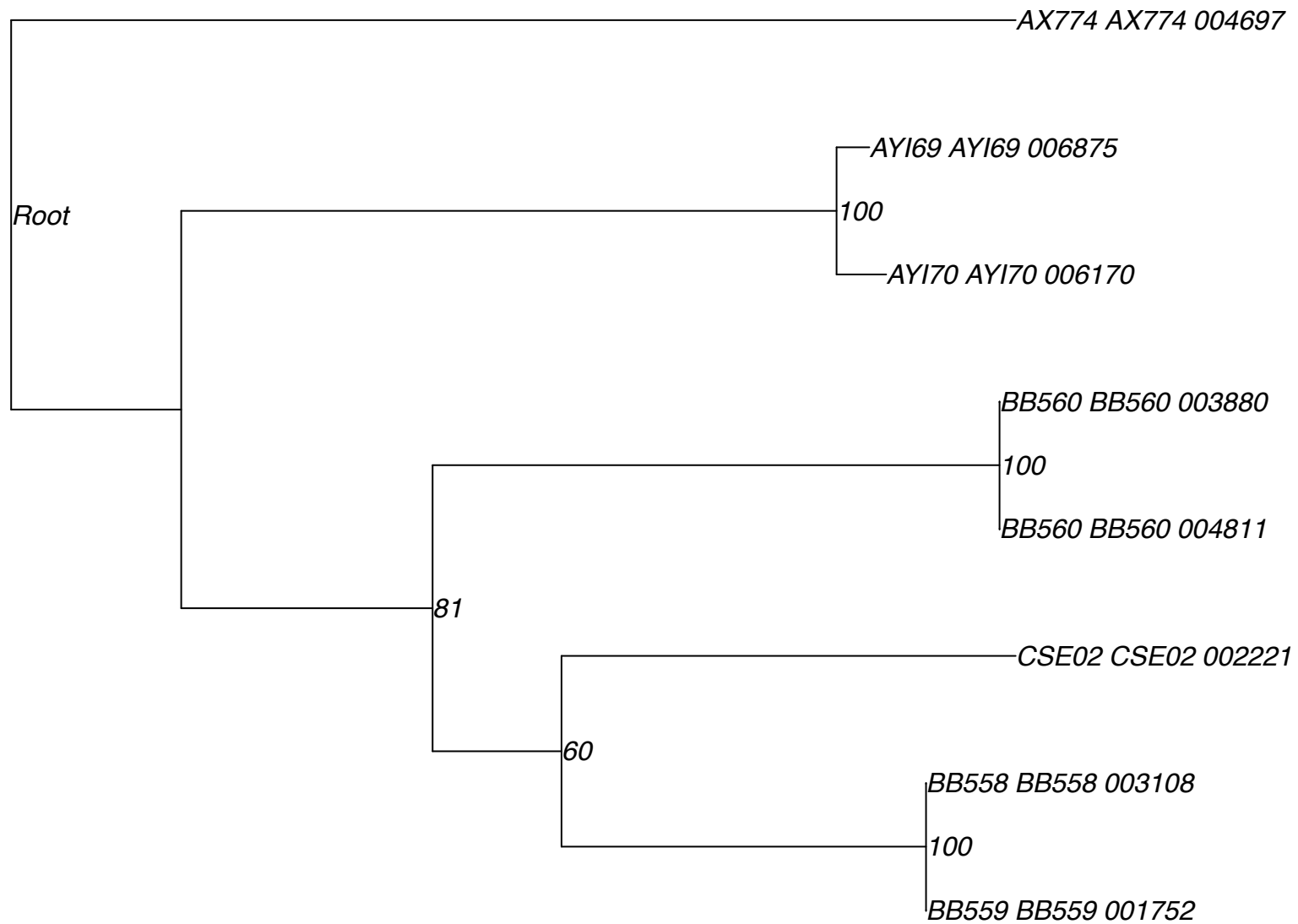

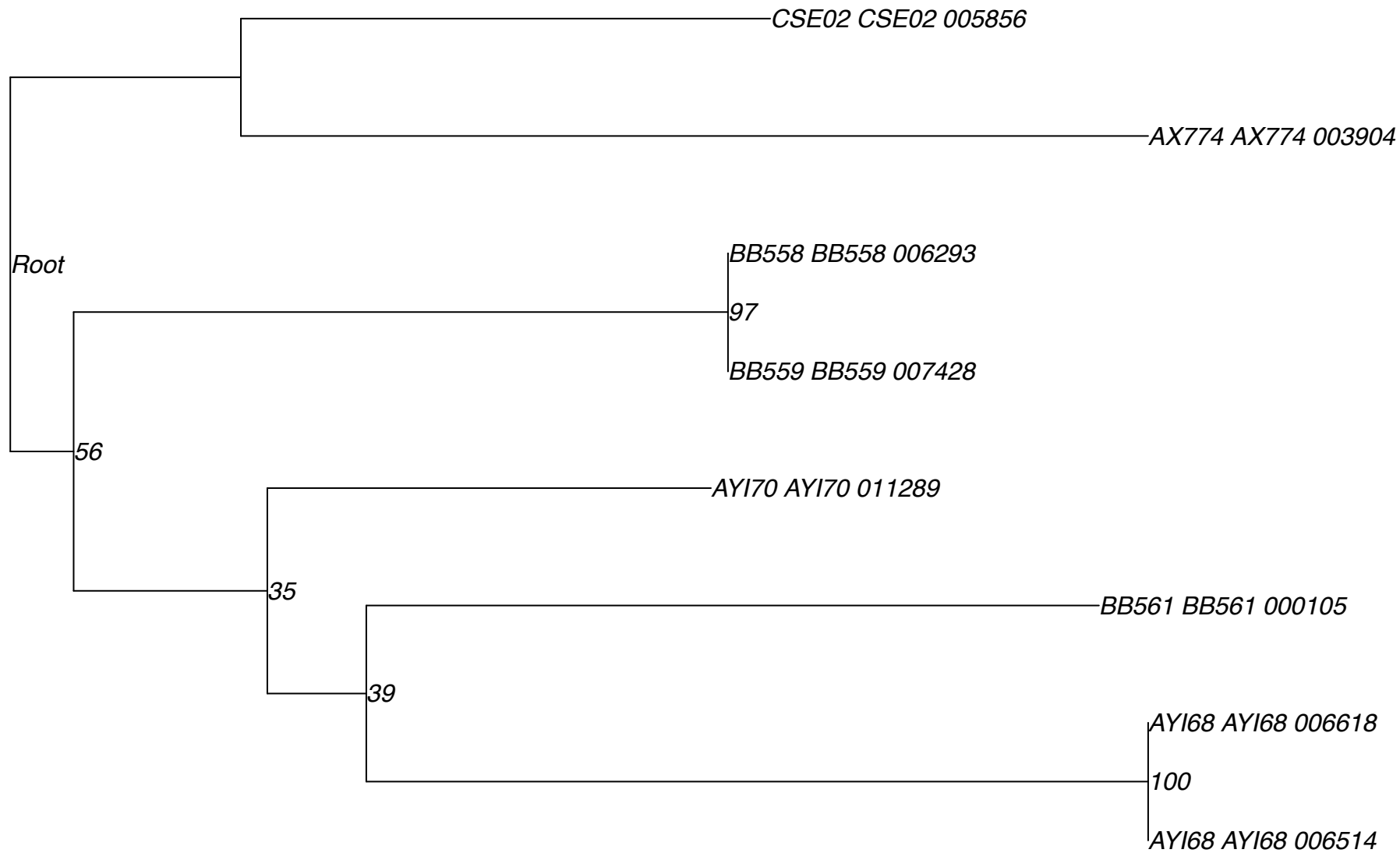

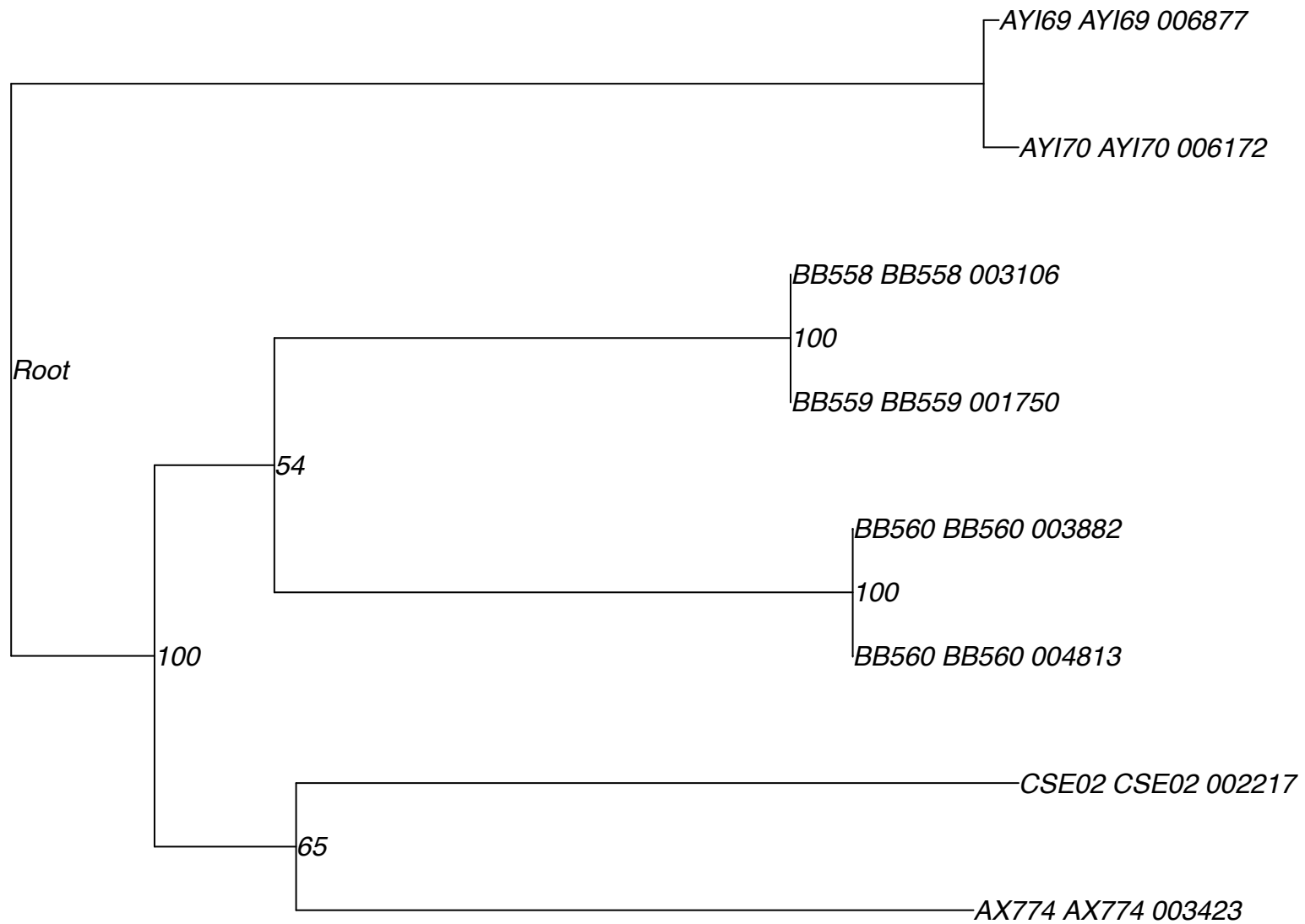

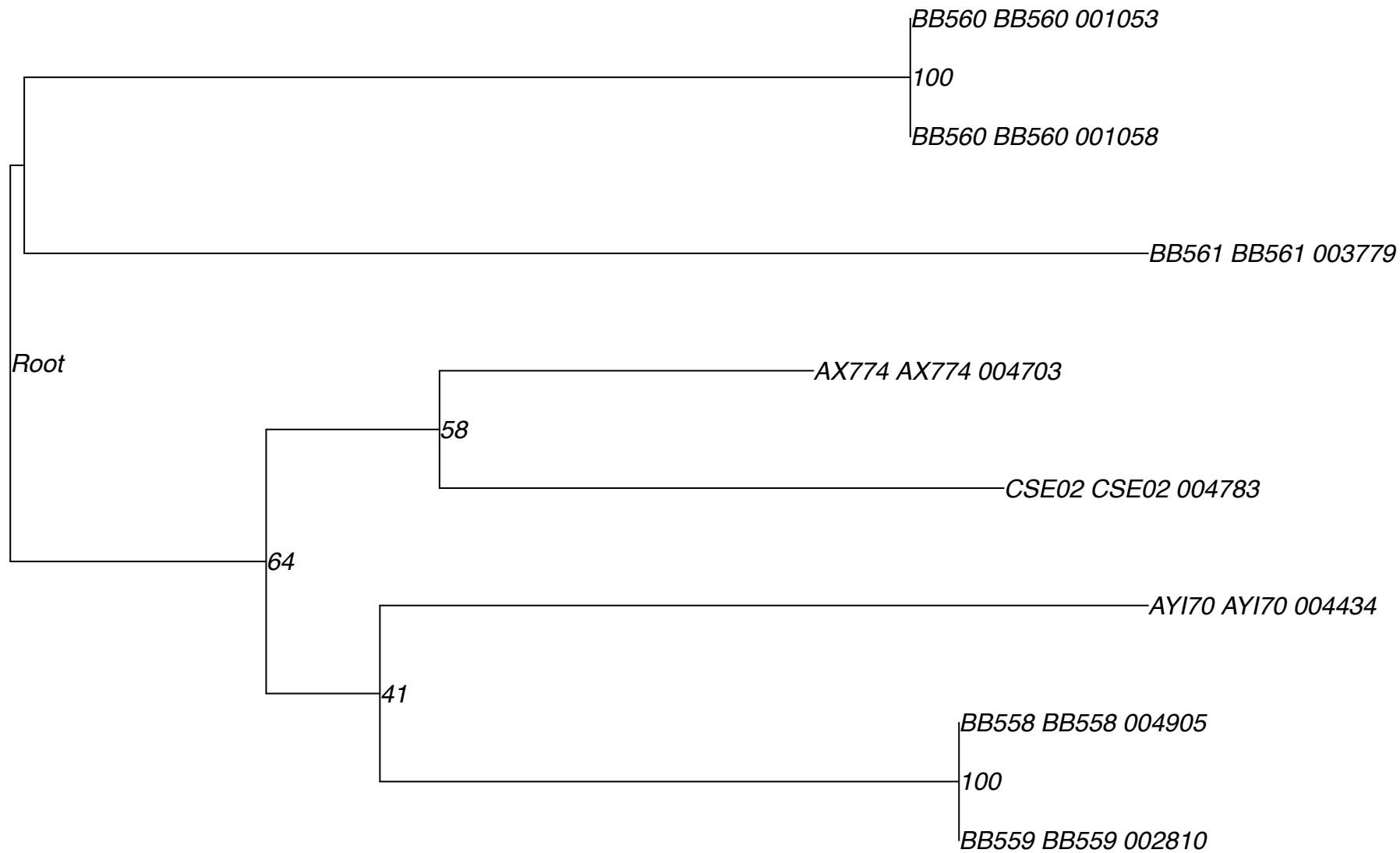

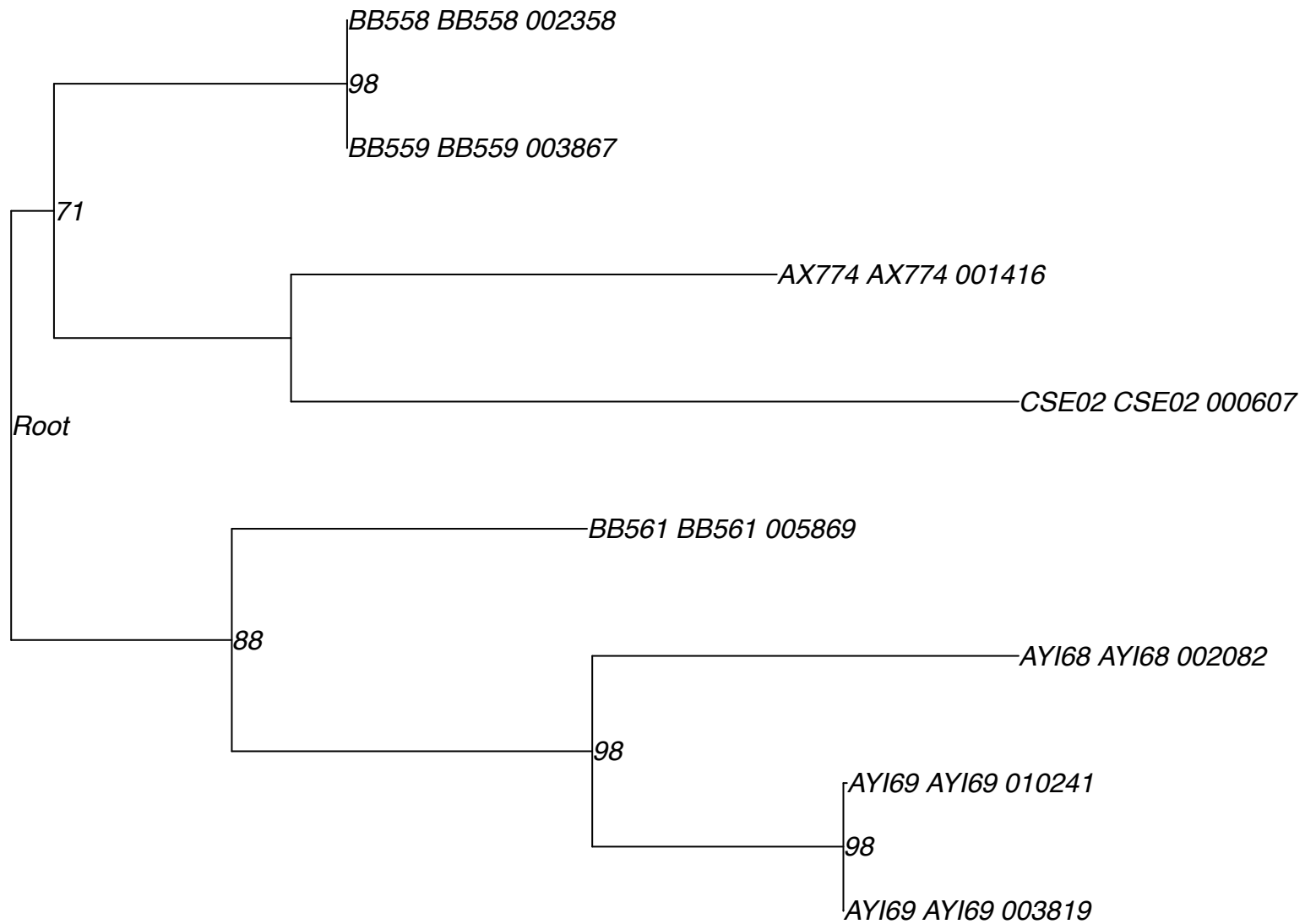

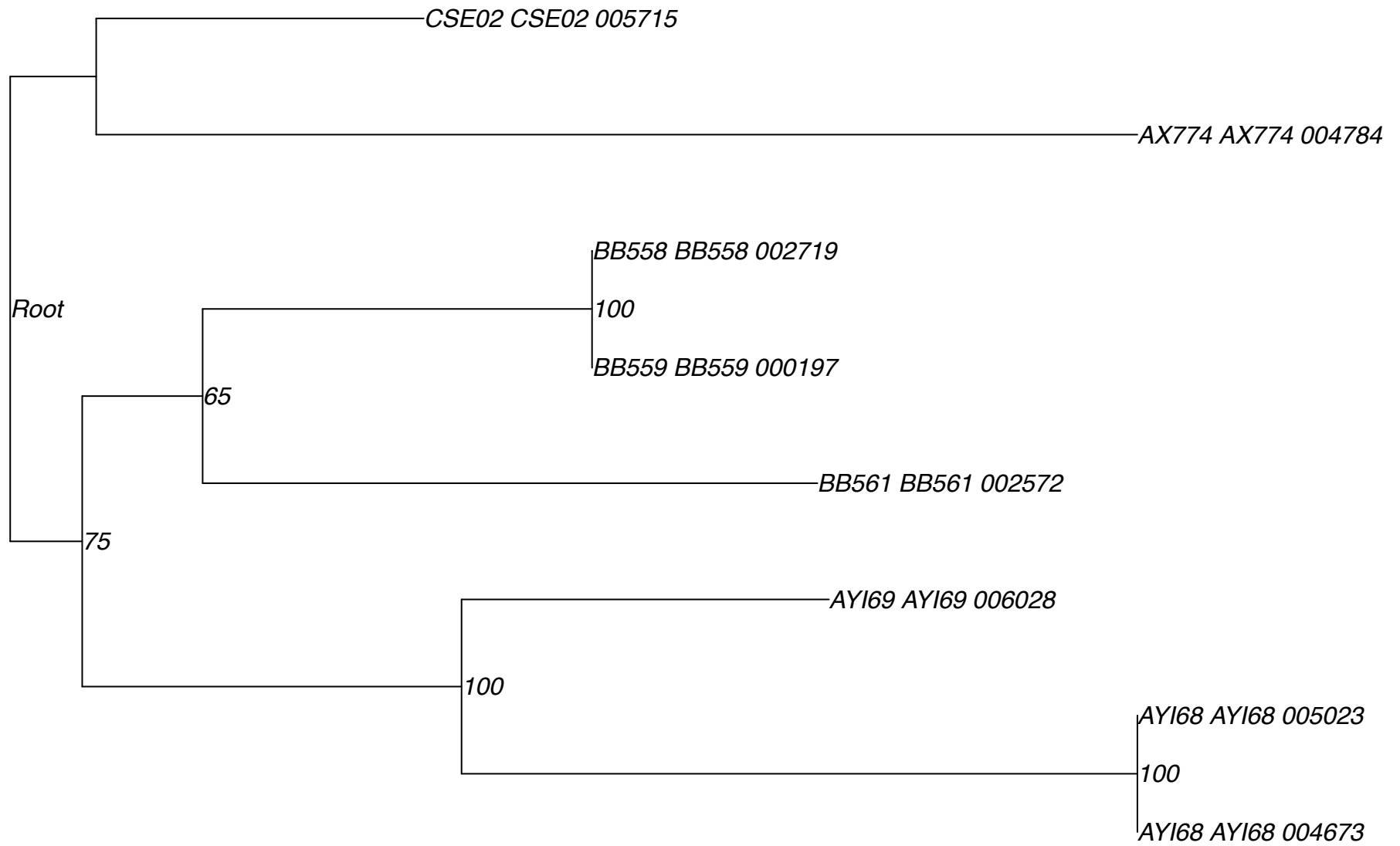

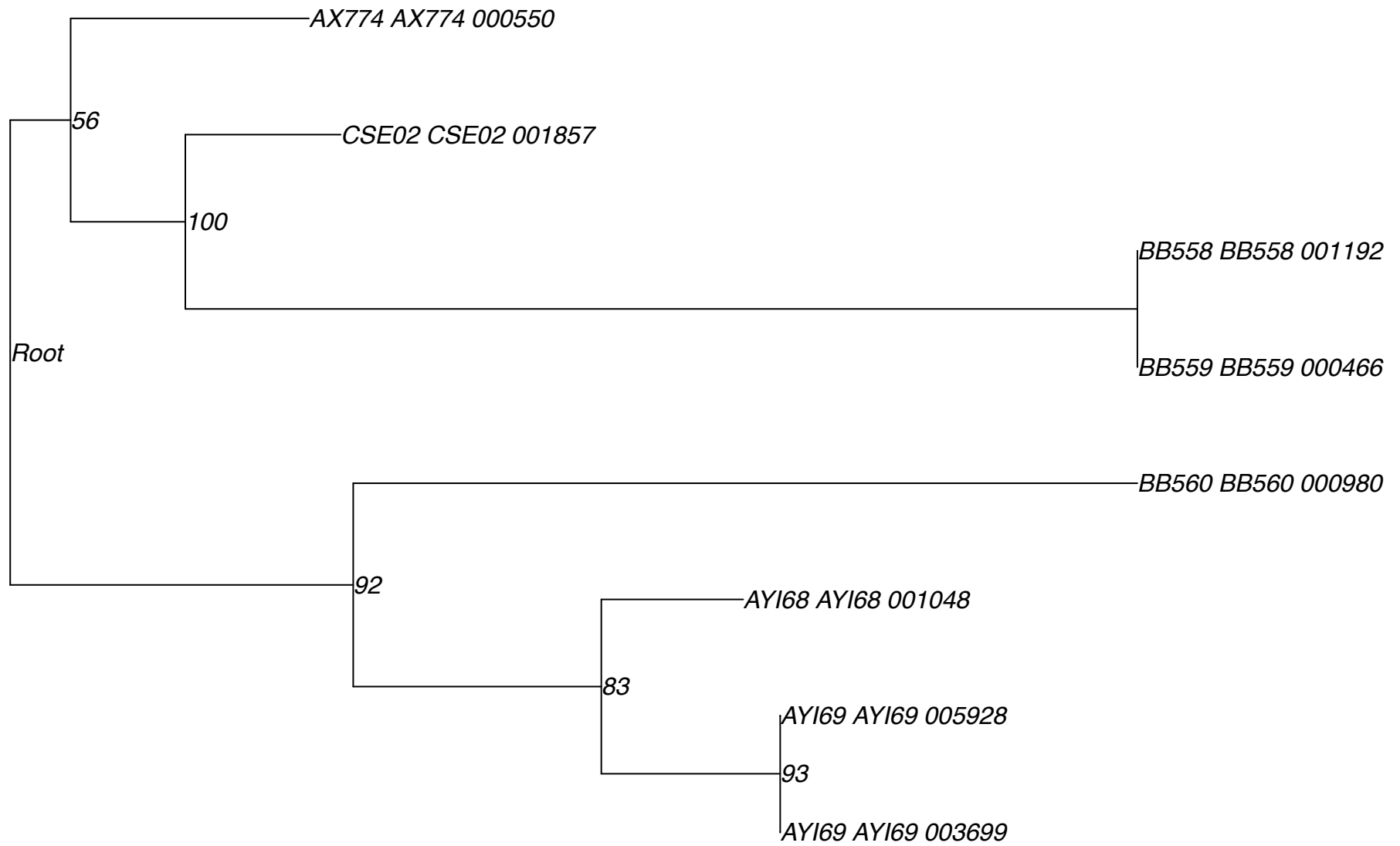

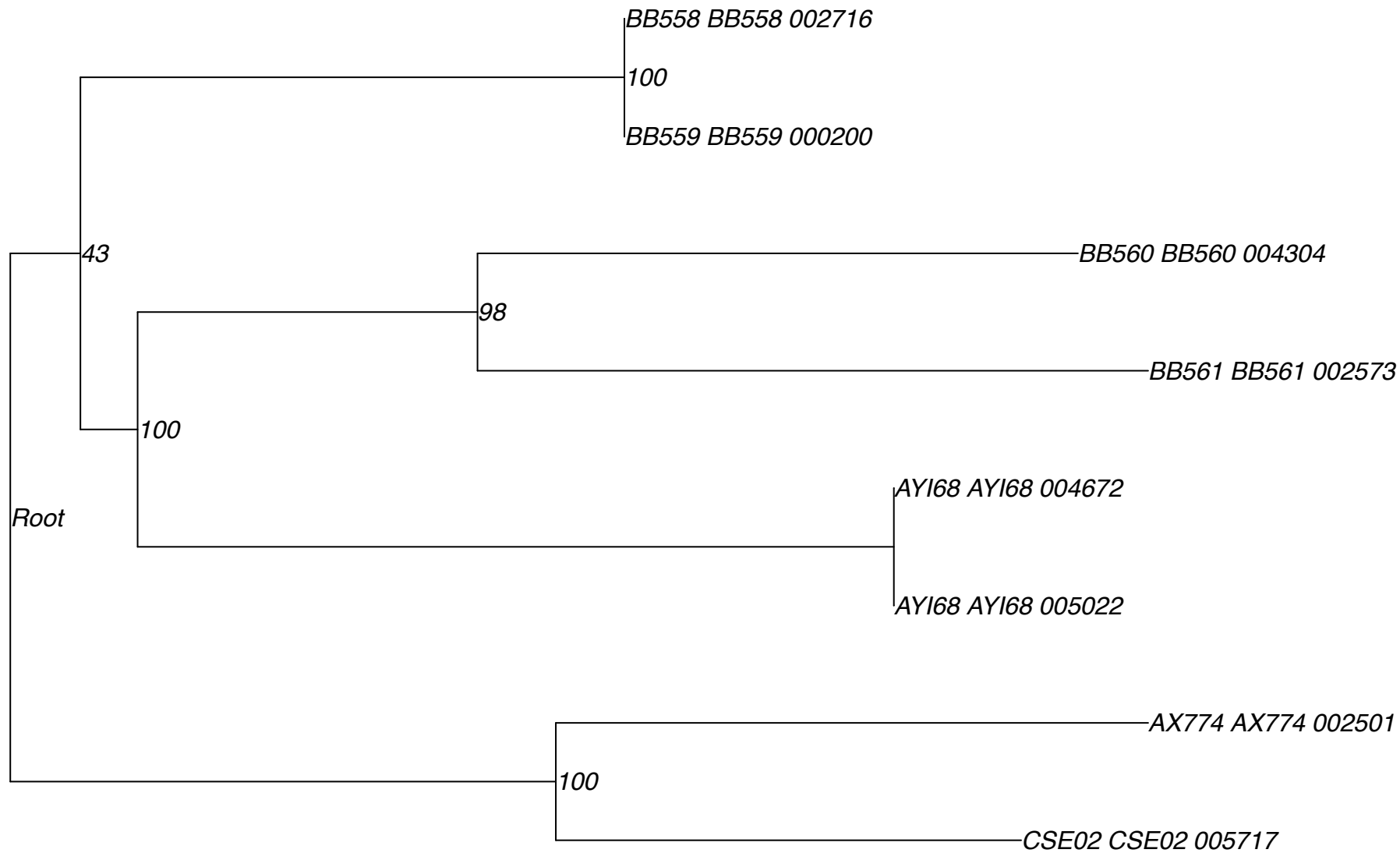

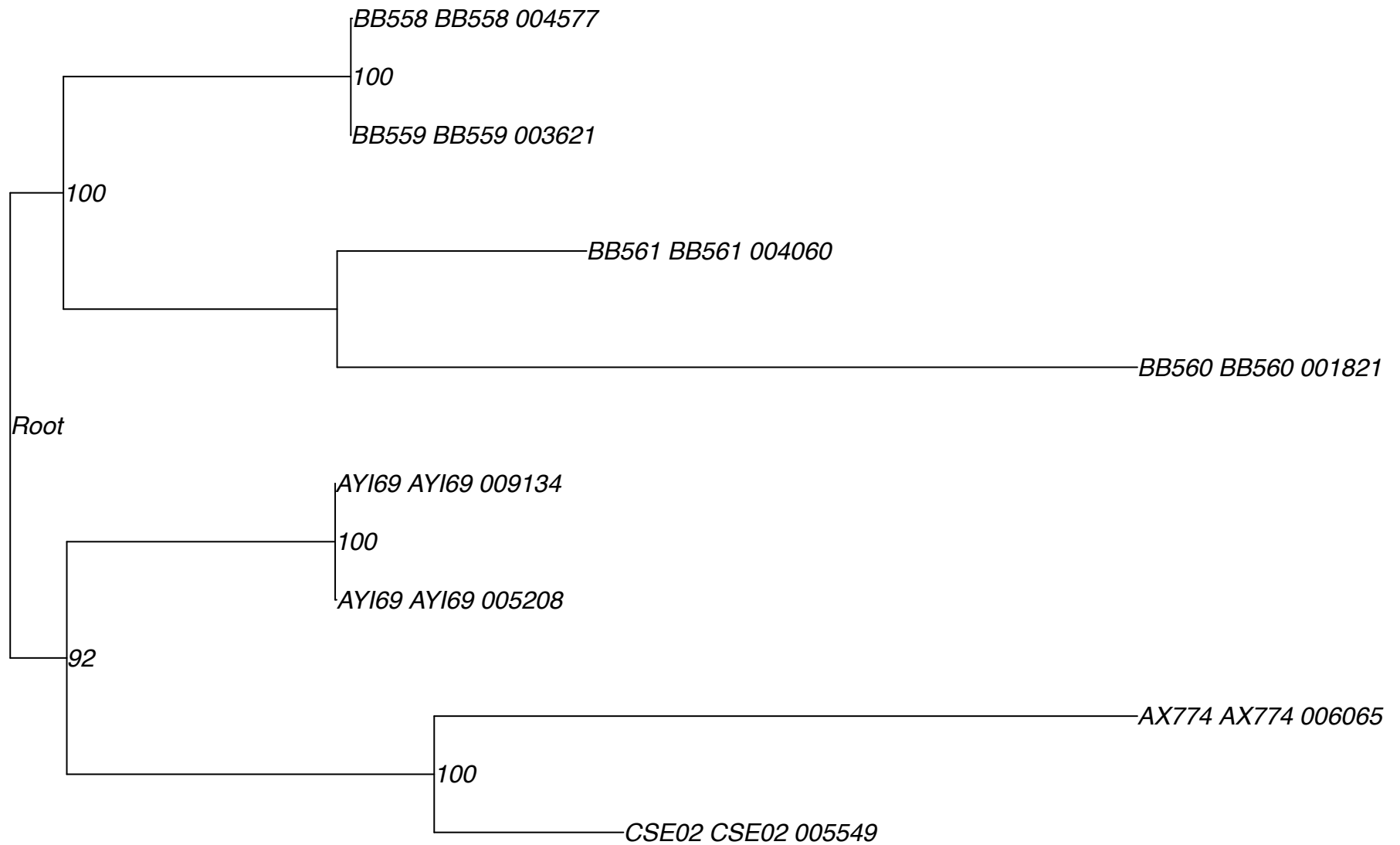

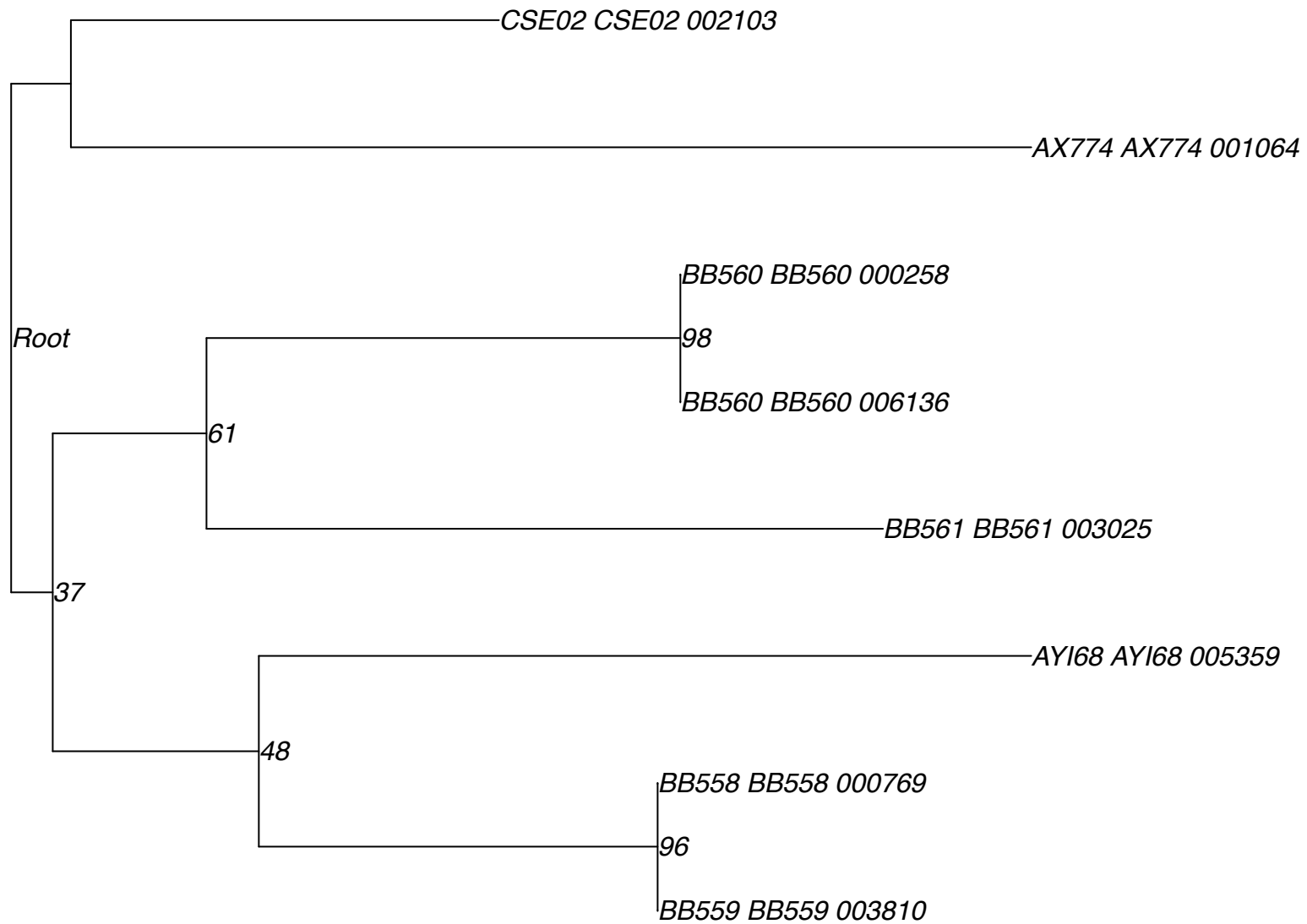

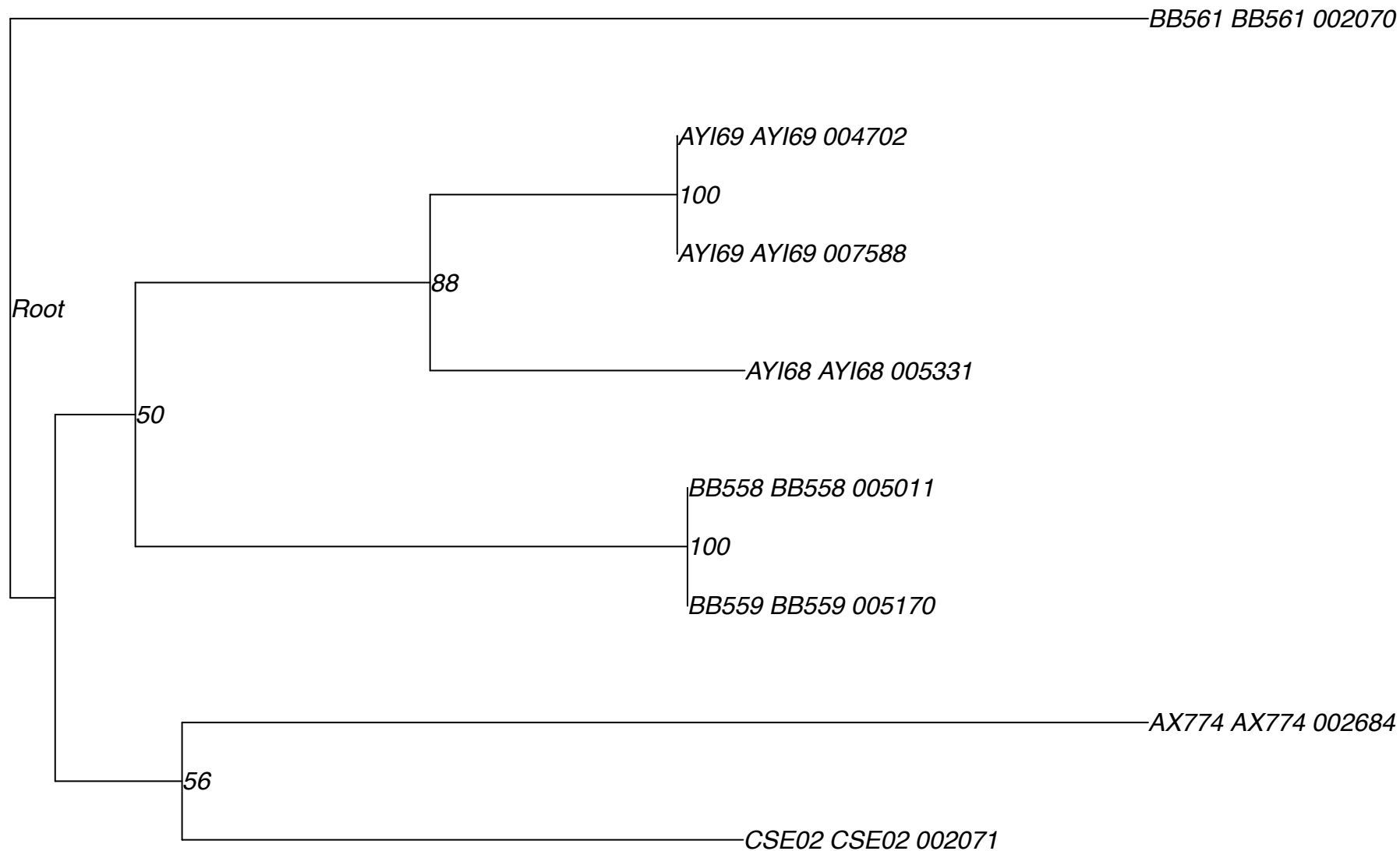

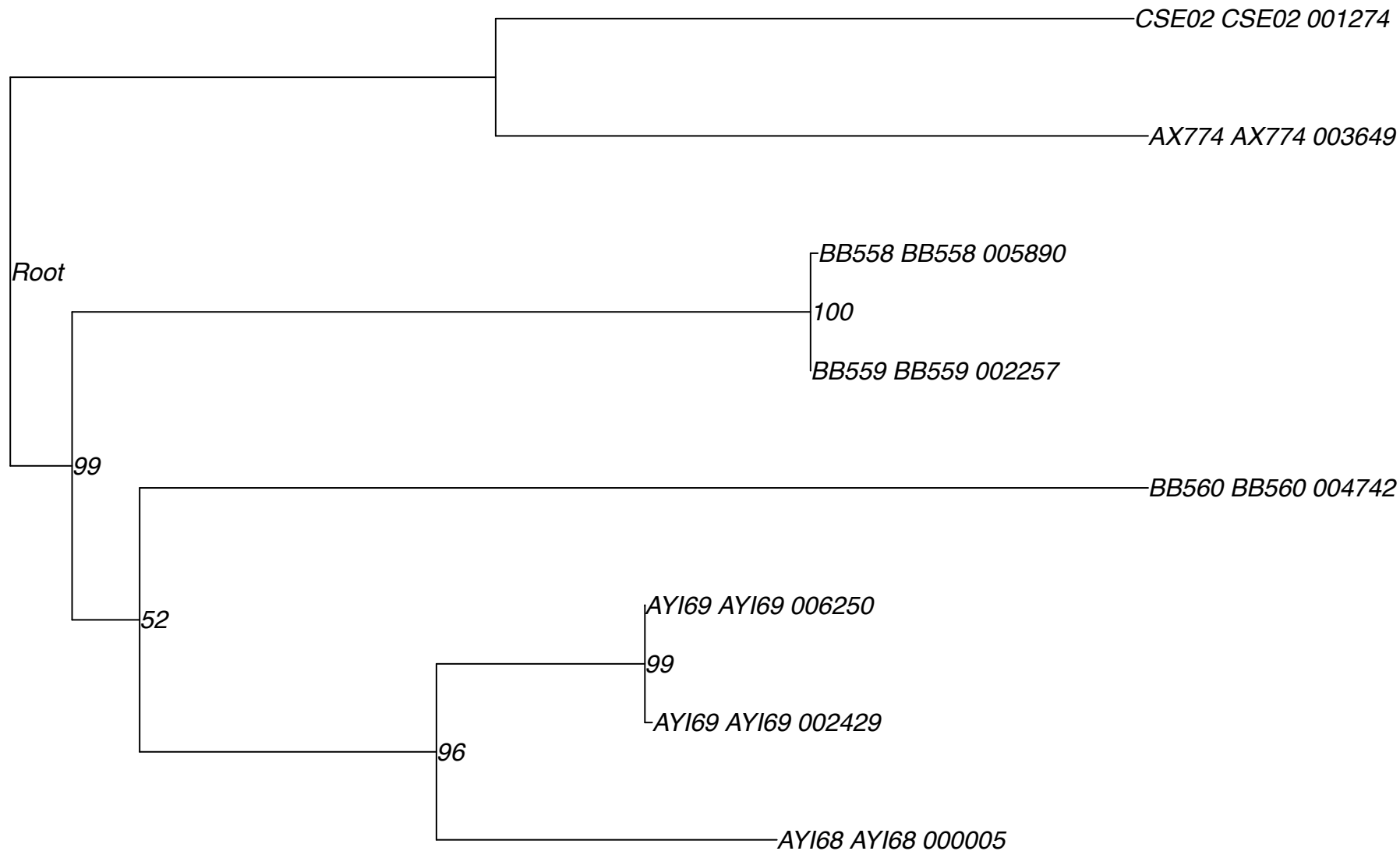

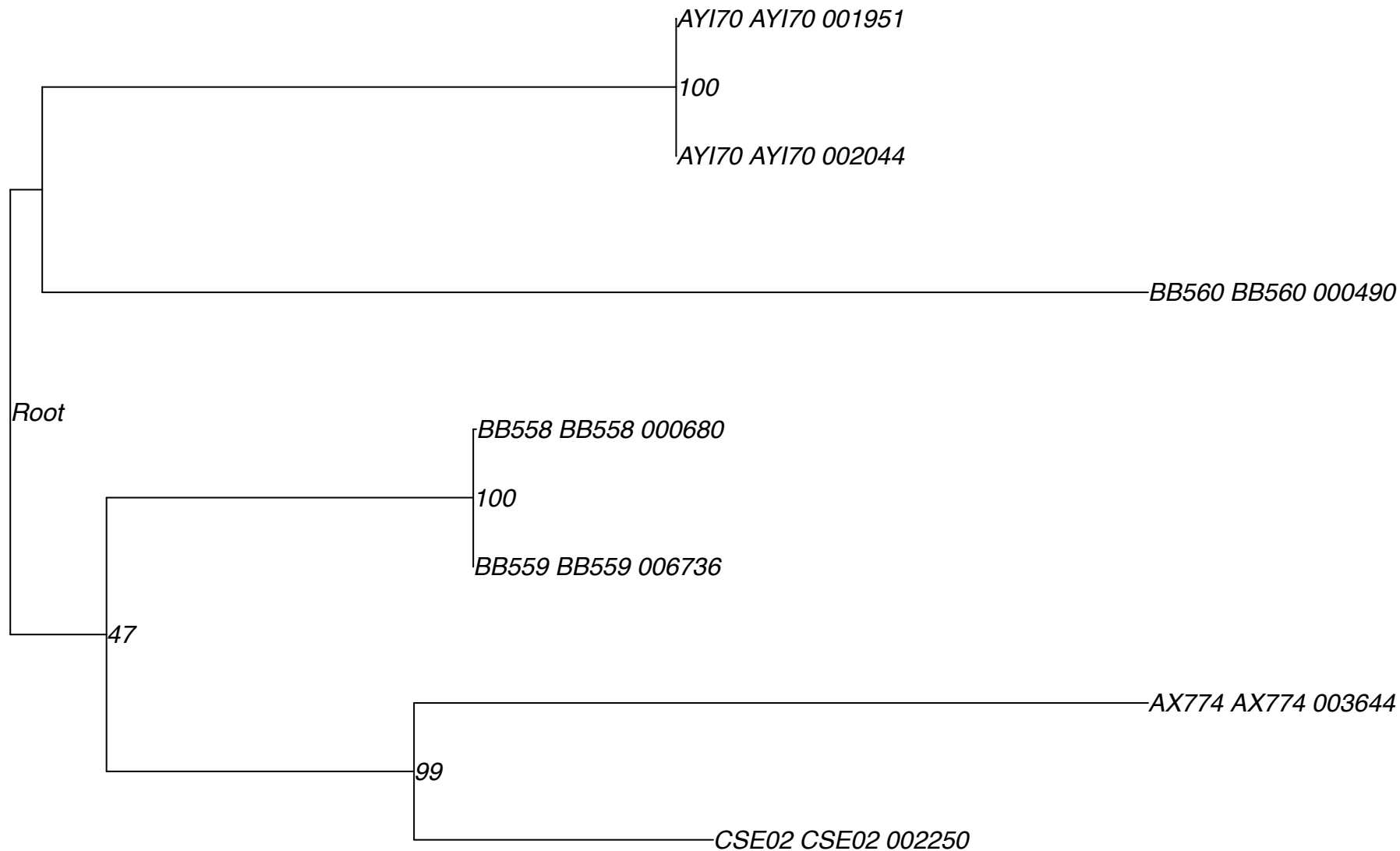

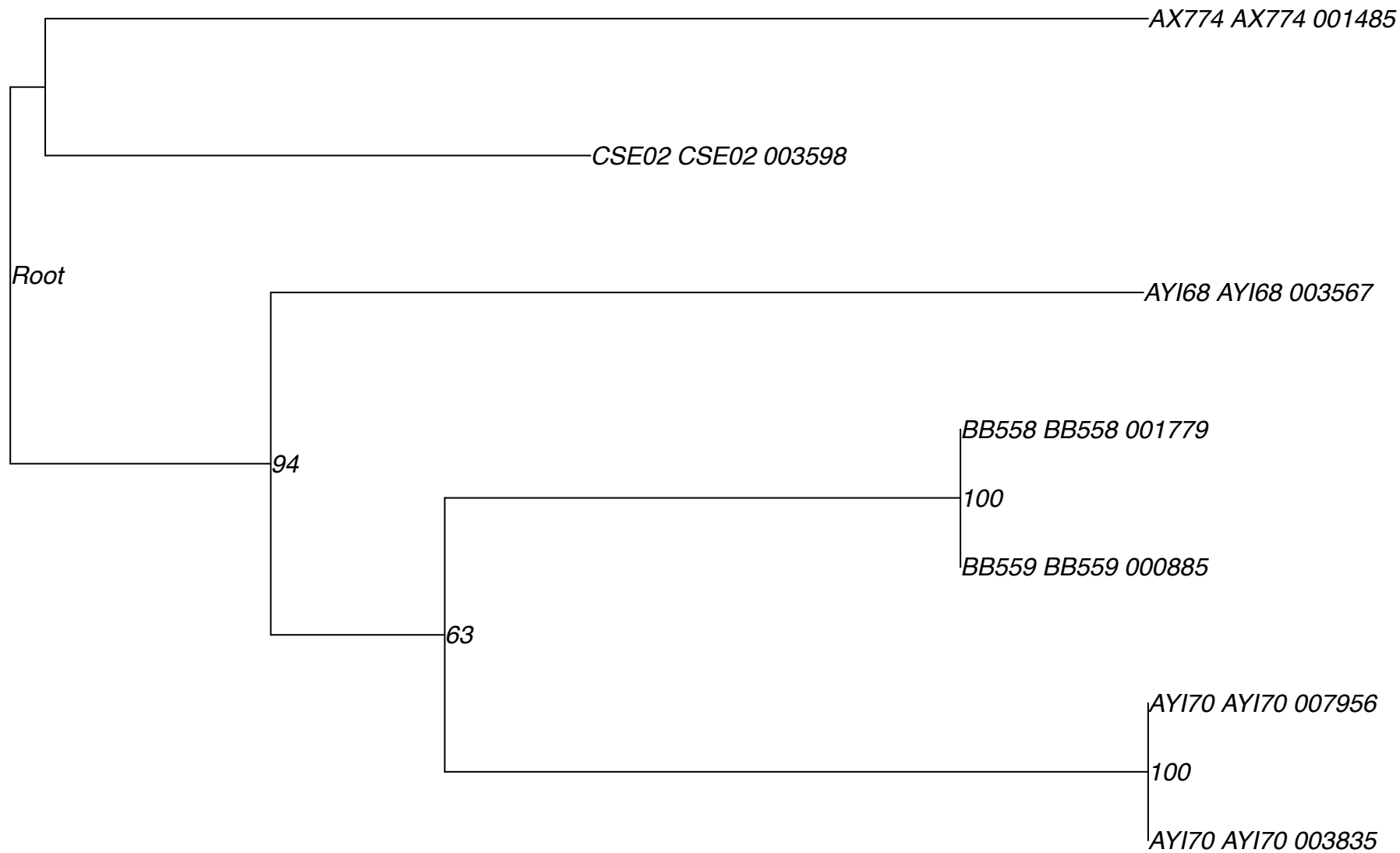

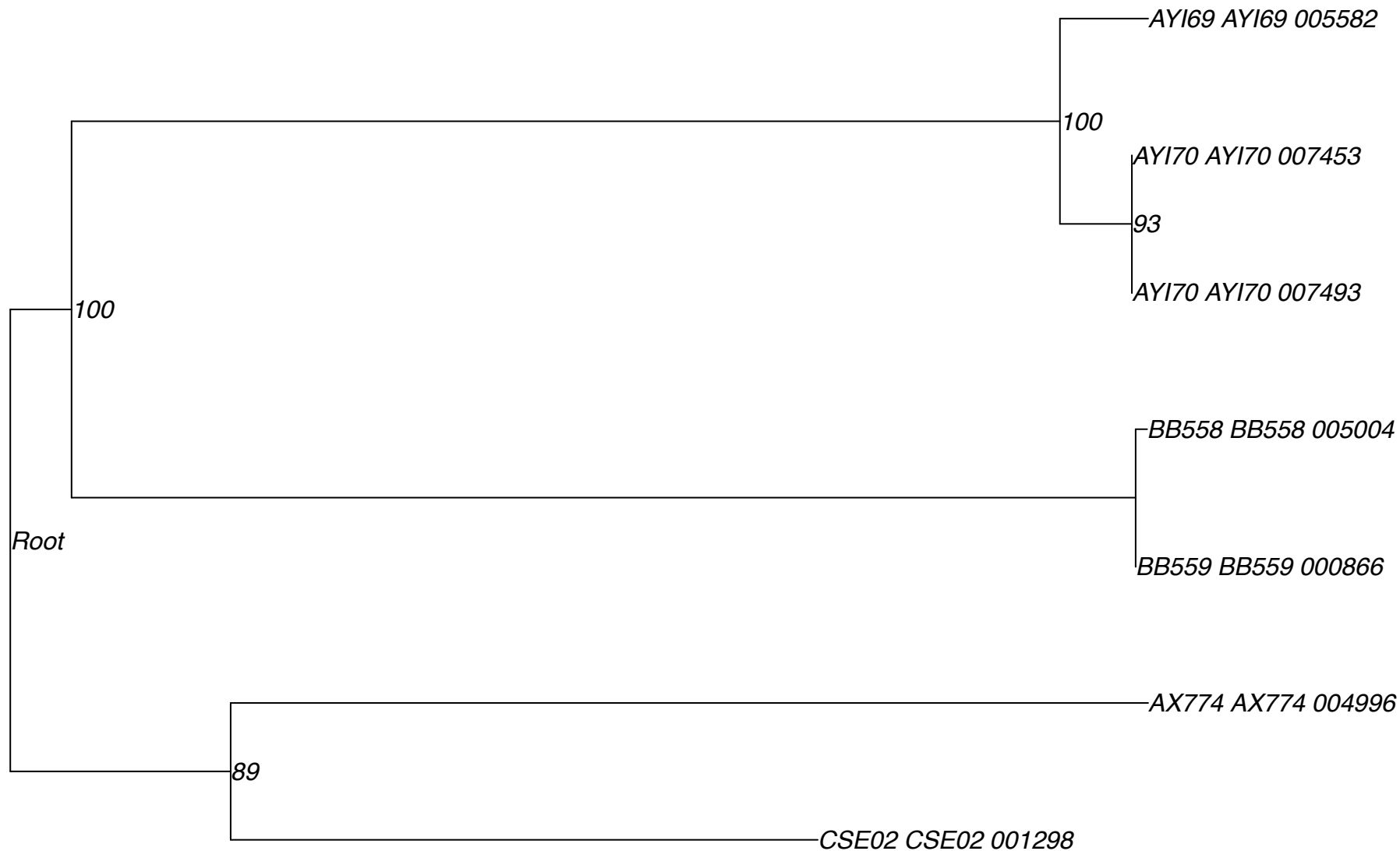

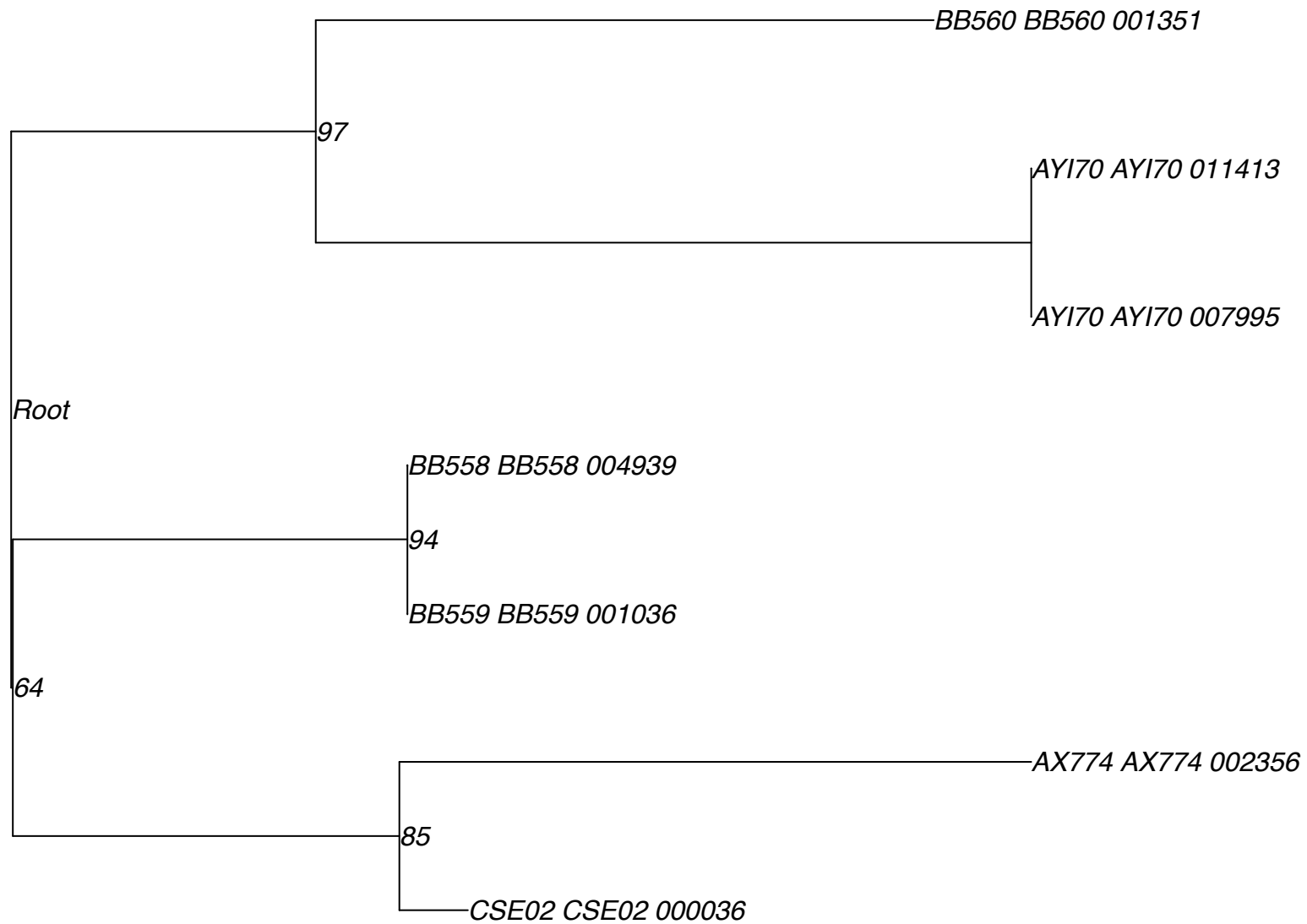

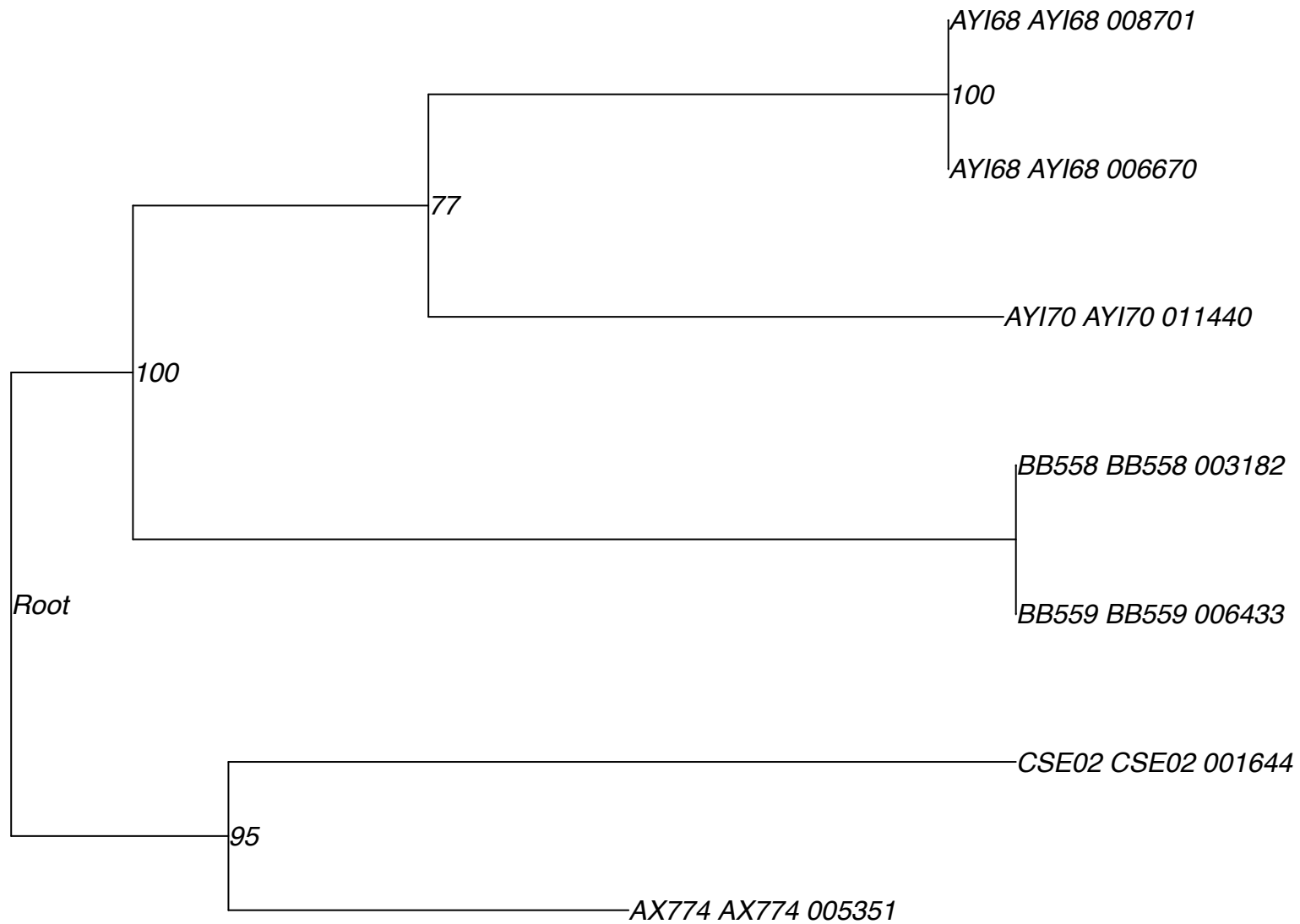

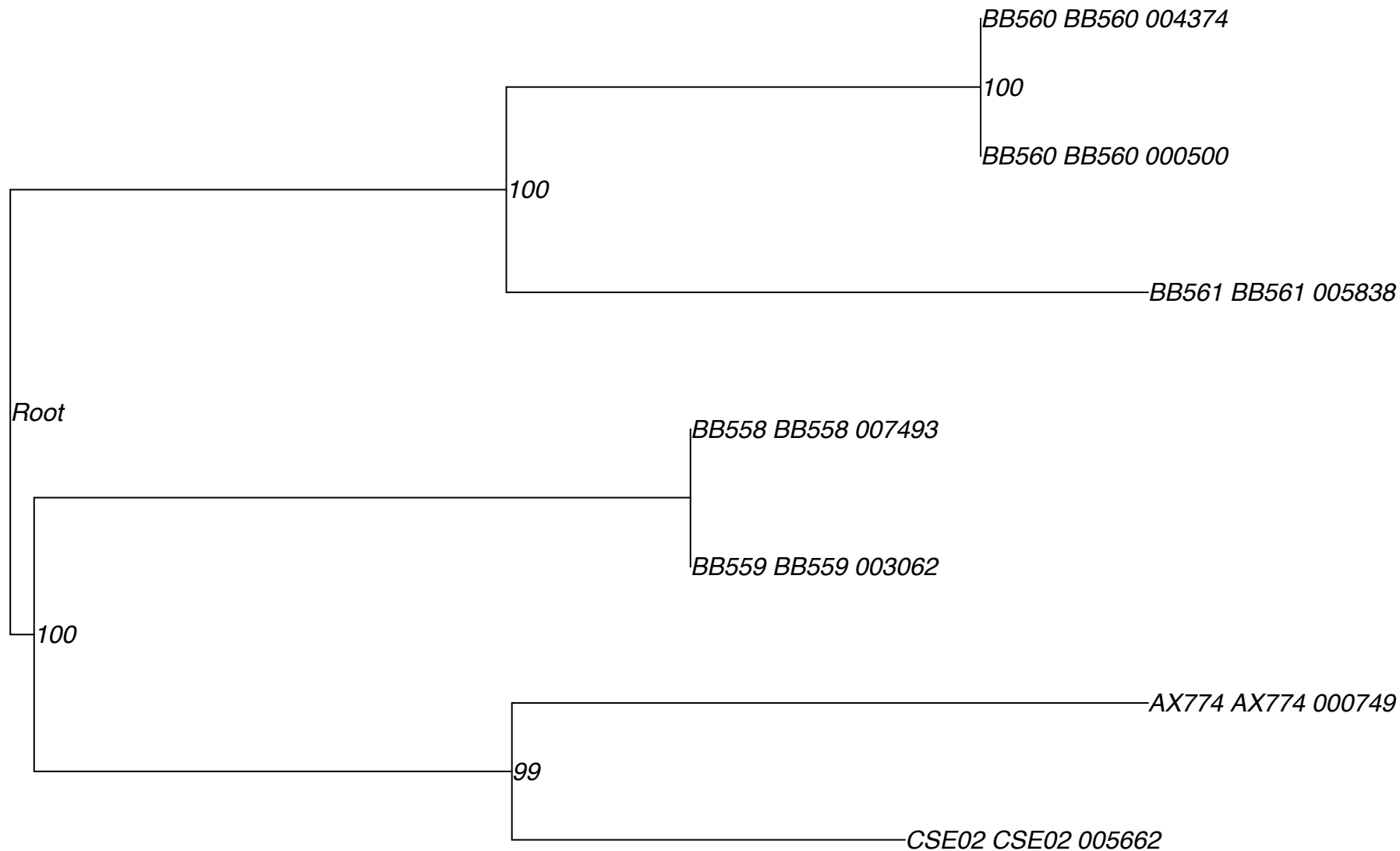

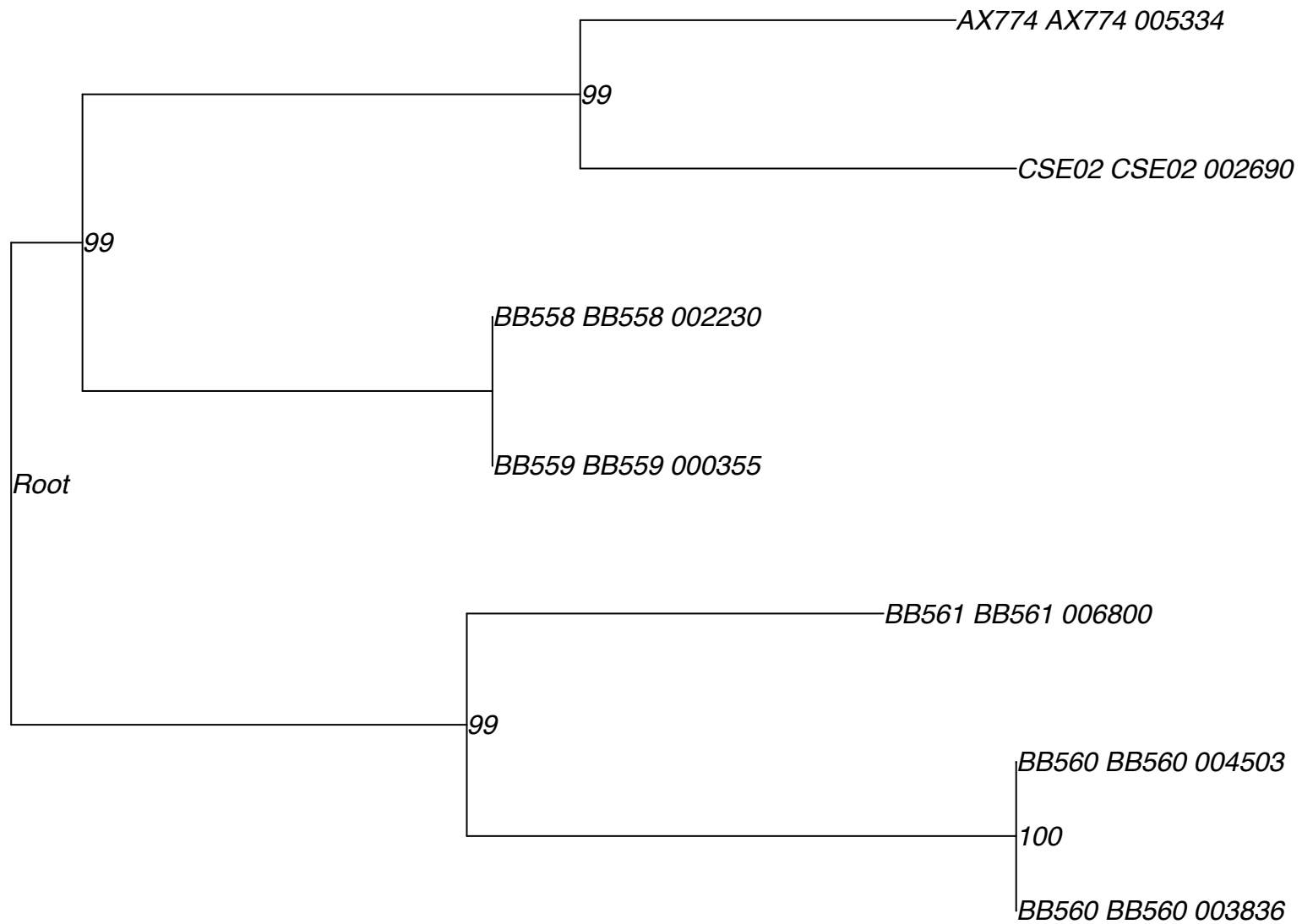

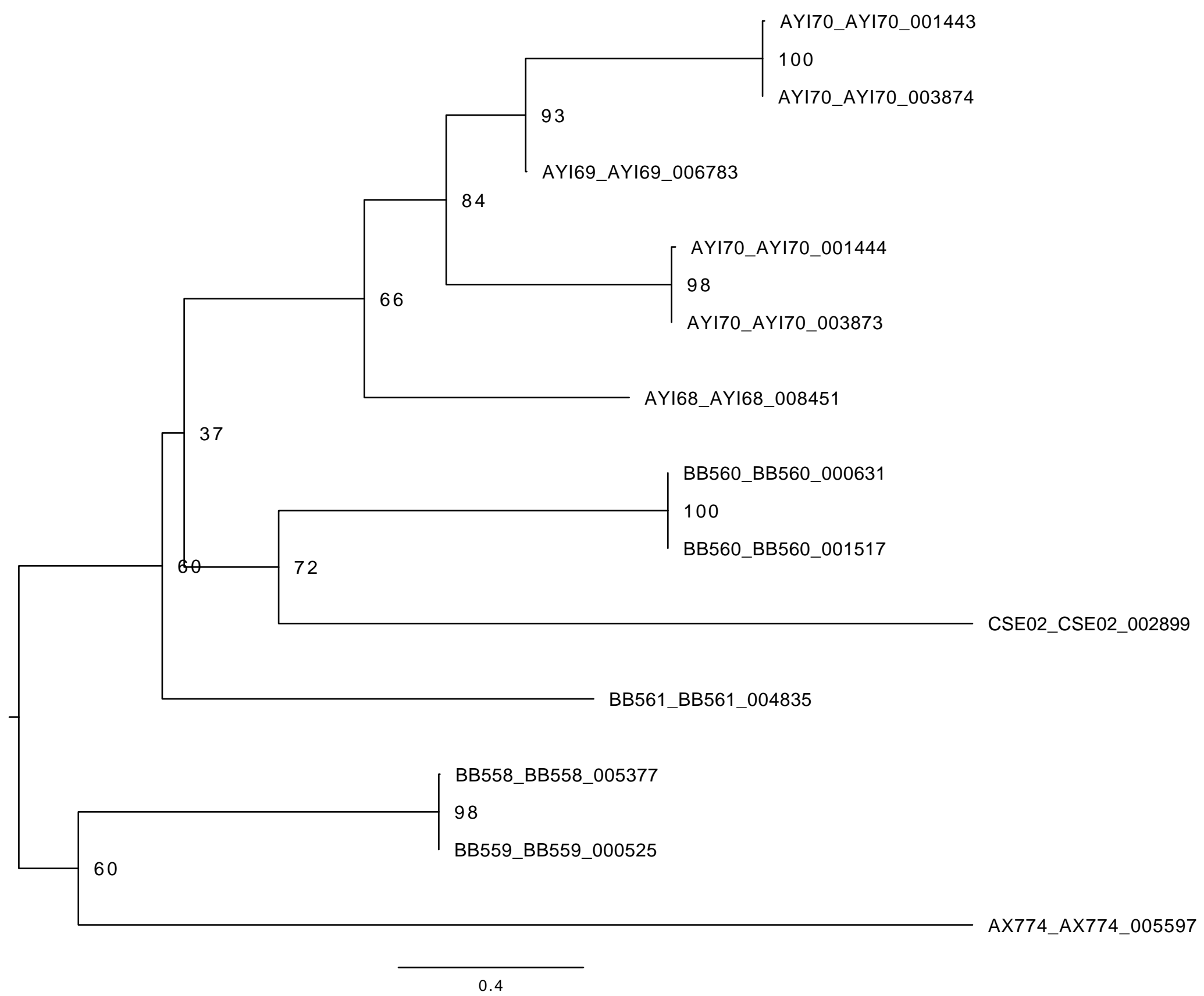

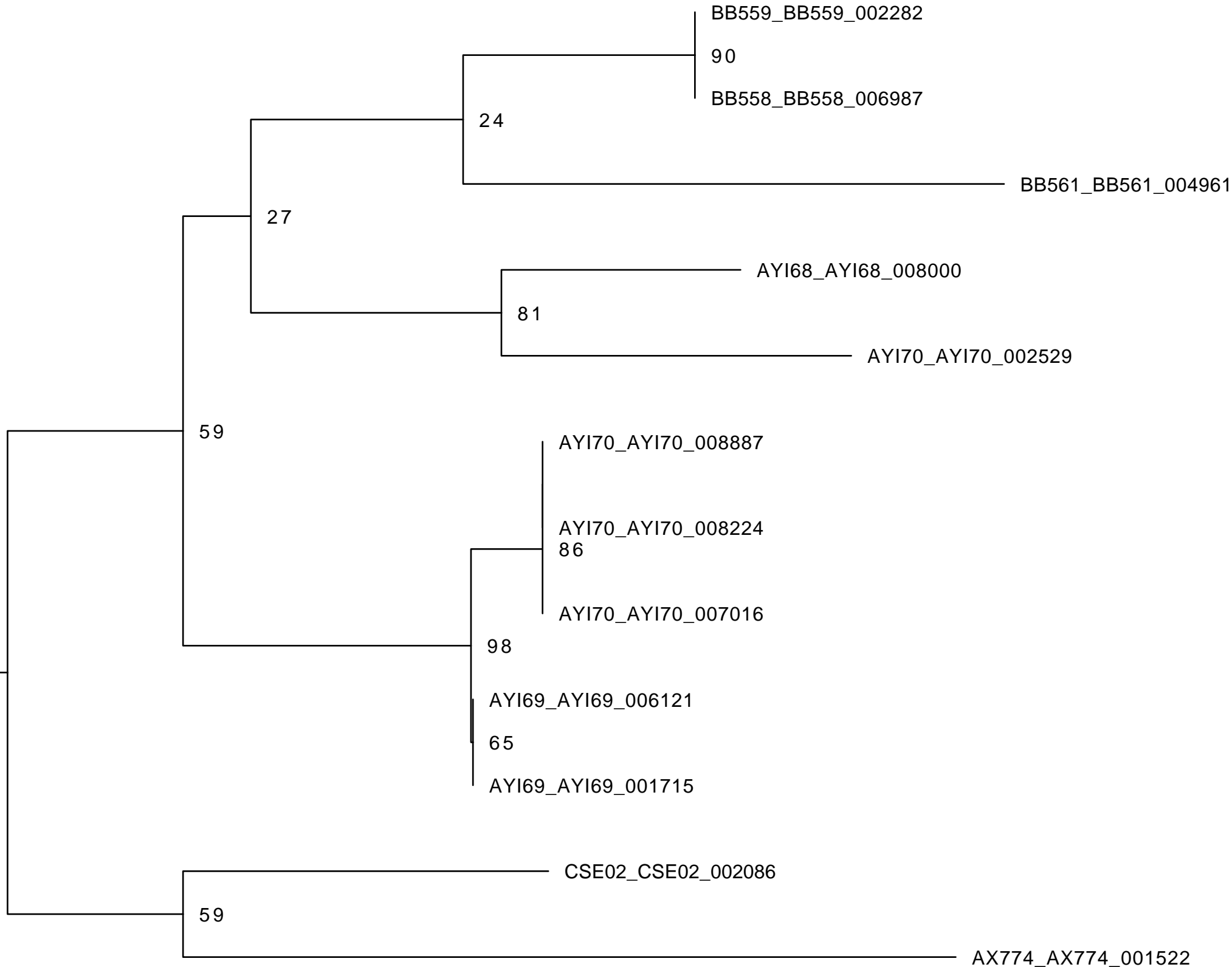

0.06

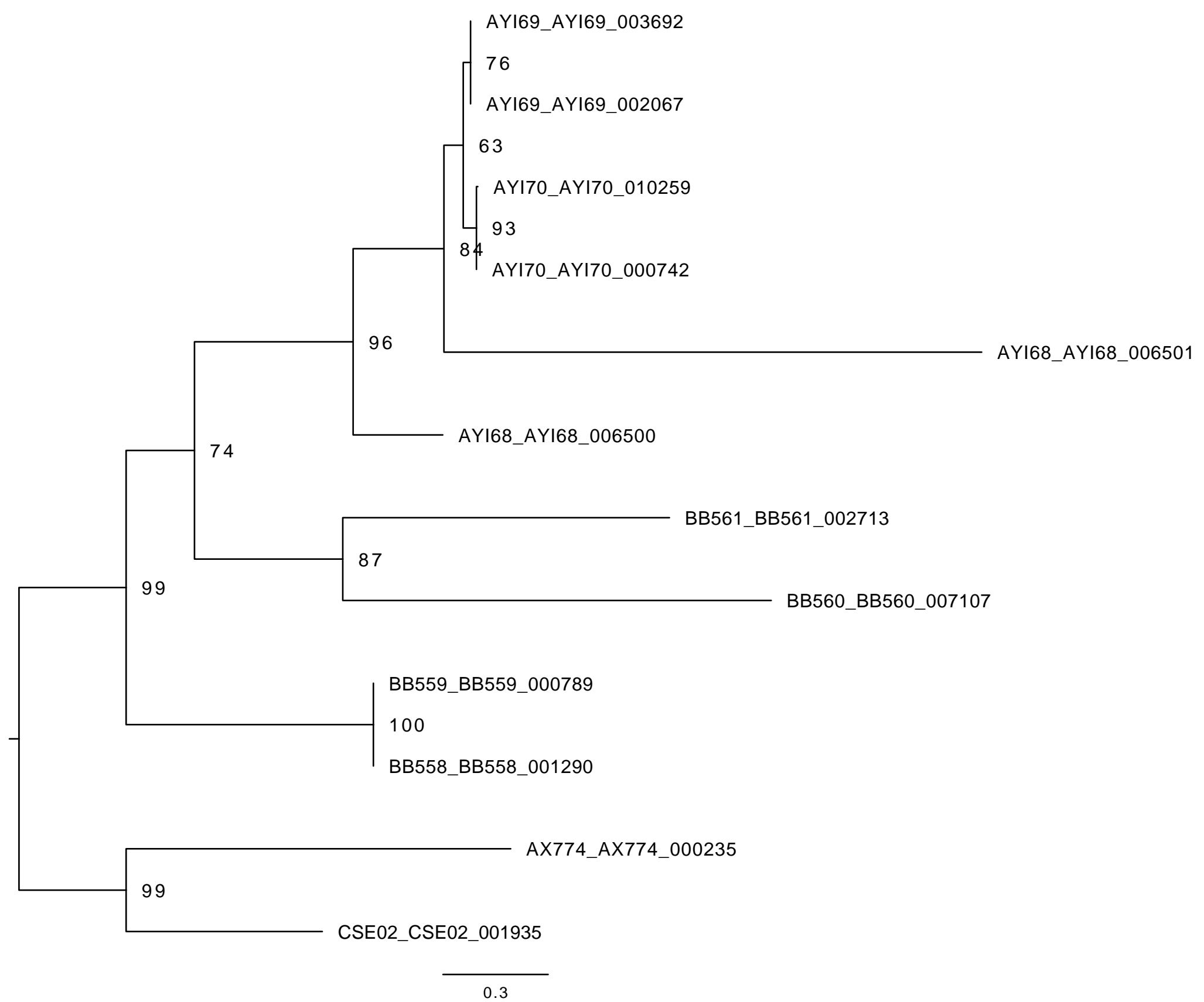

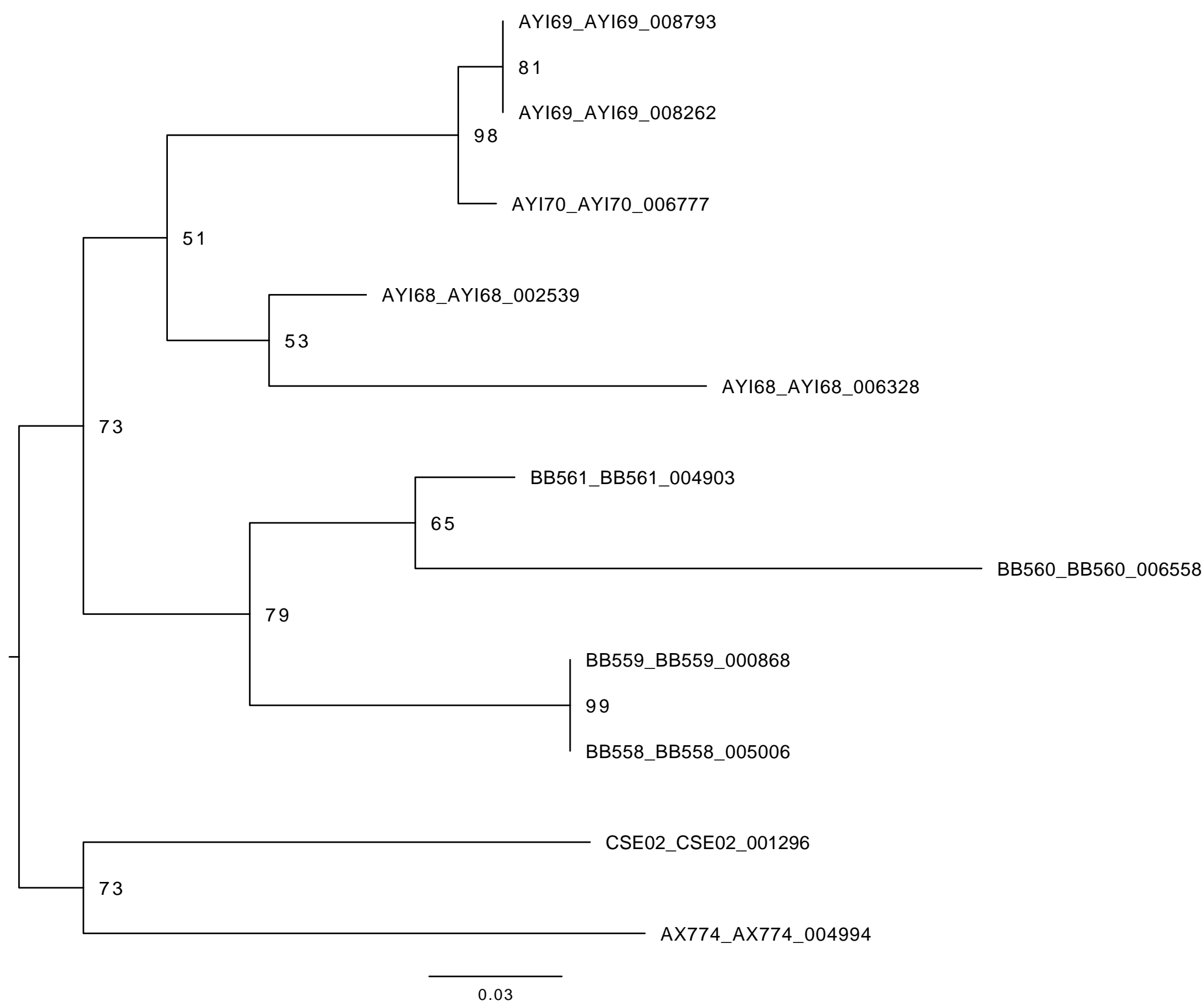

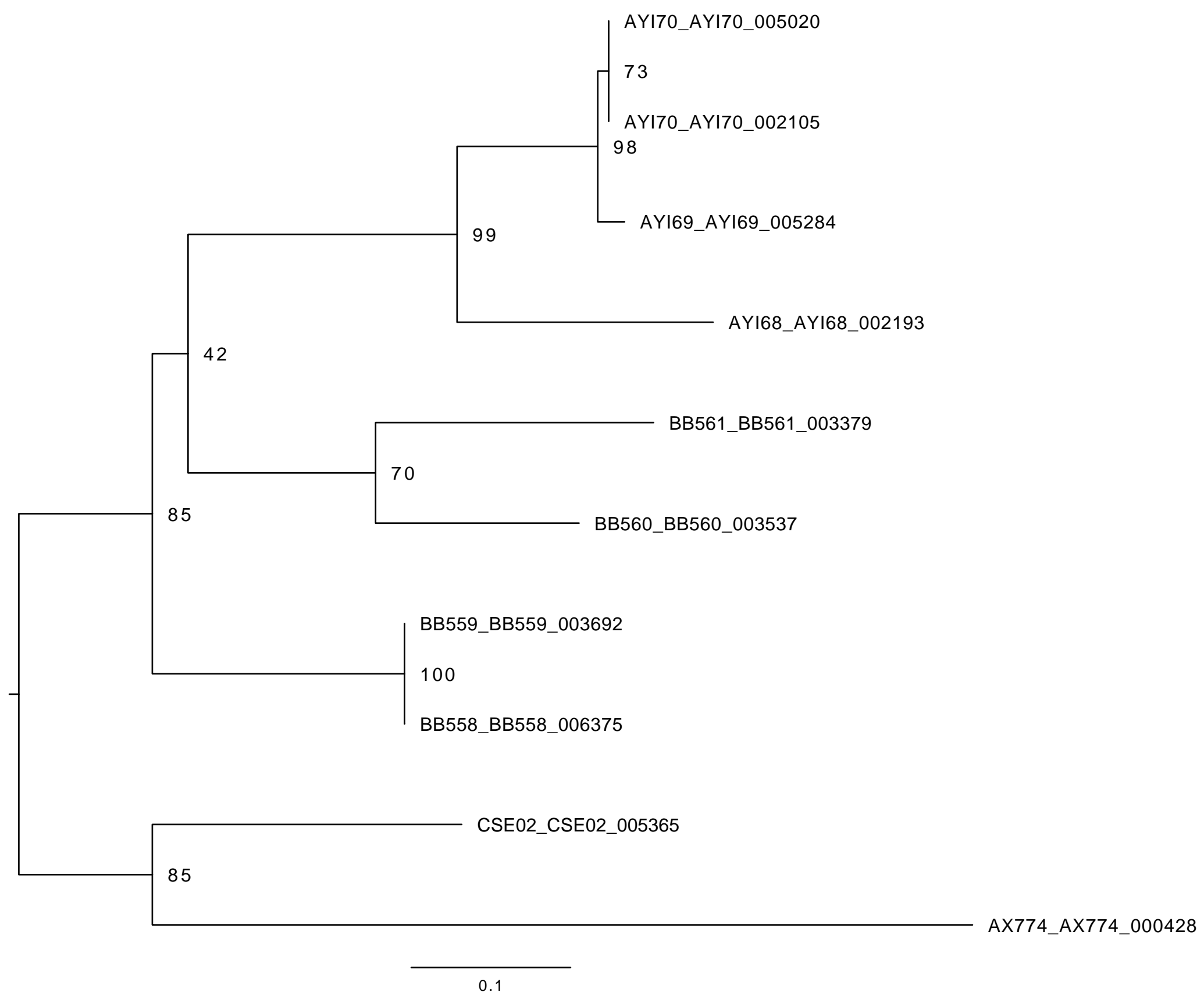

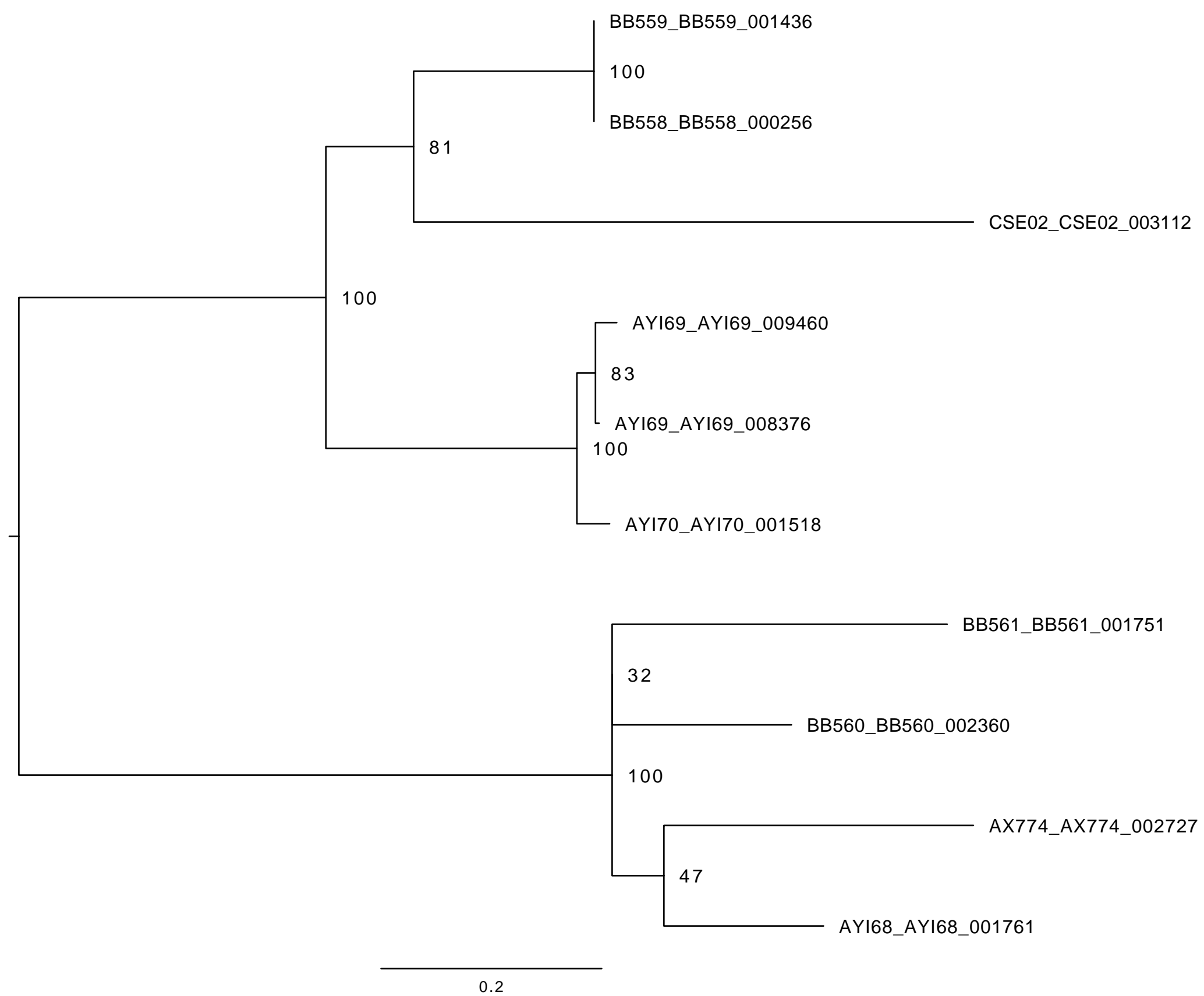

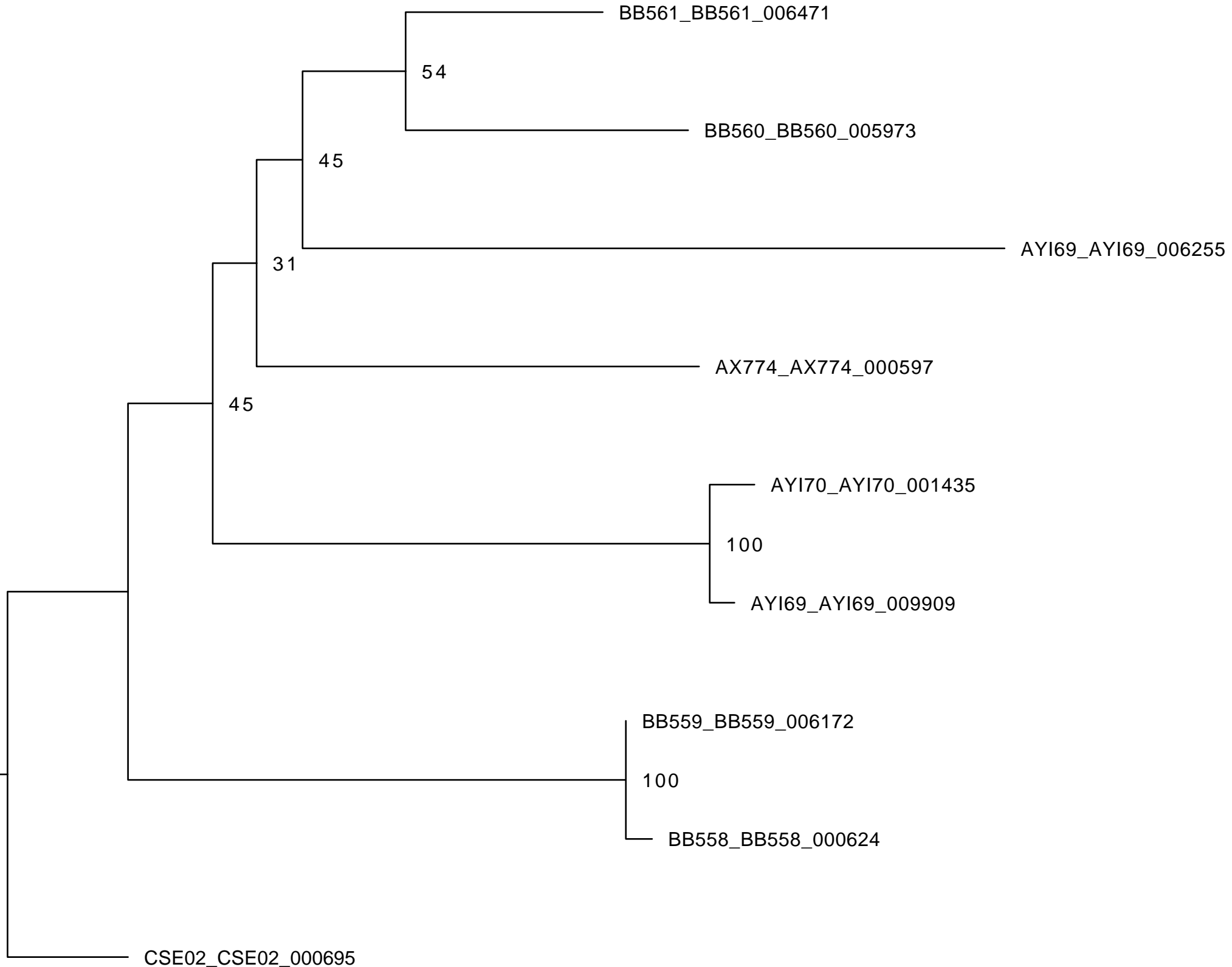

0.09

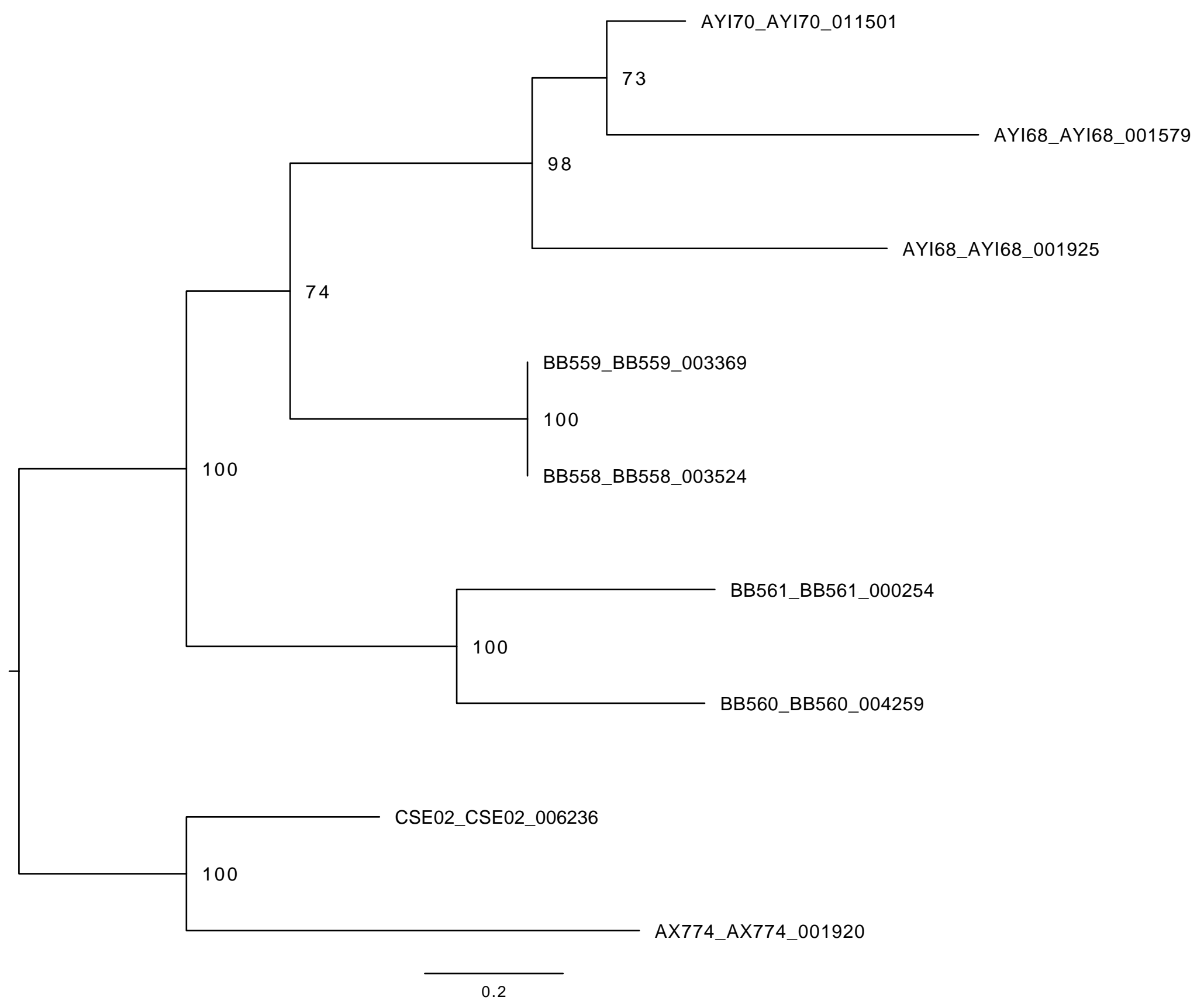

Supplement: FIG S1 [file mbo003183874sf1.pdf]

*Basidiobolus meristosporus*

*Conidiobolus coronatus*

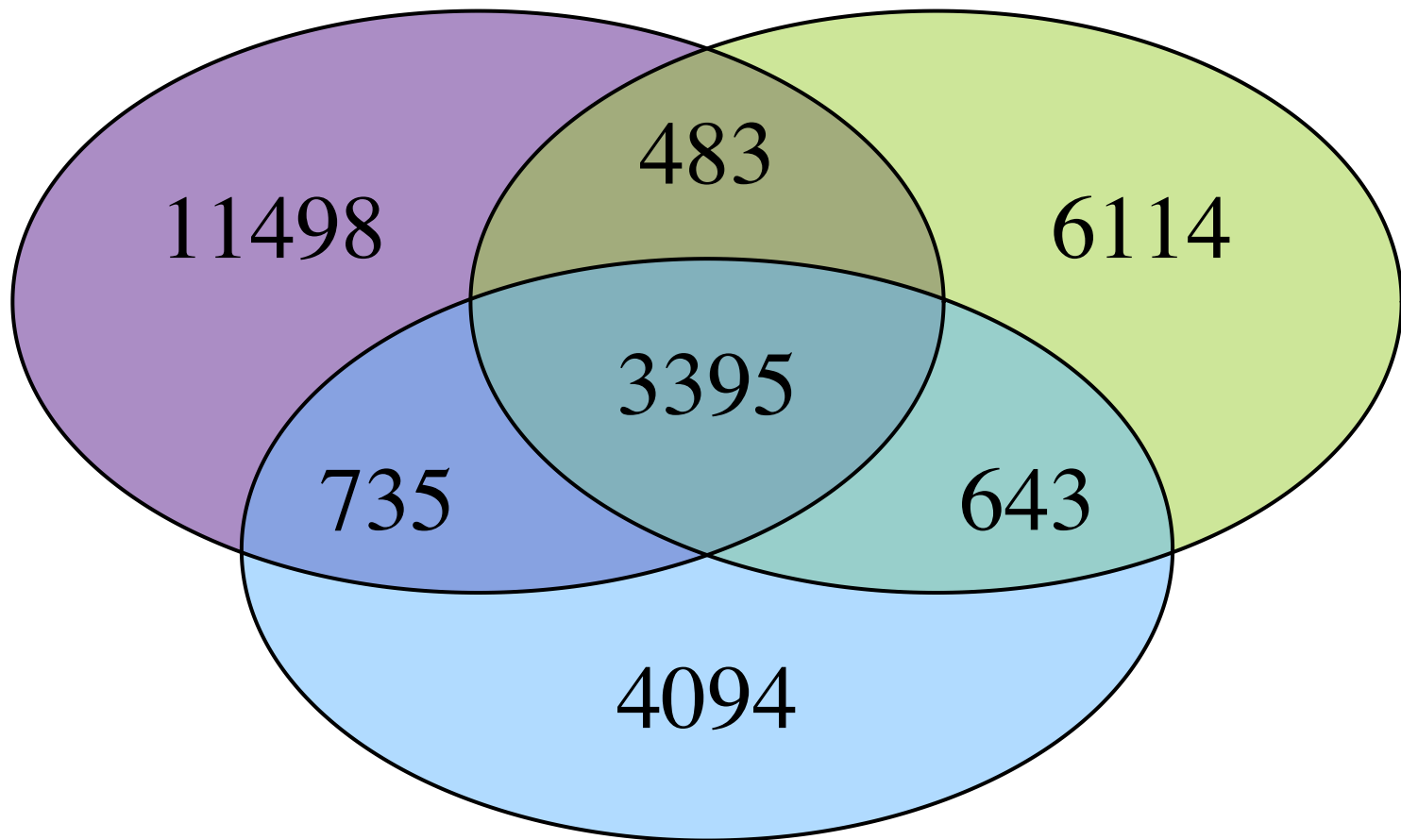

*Conidiobolus thromboides*

Supplement: FIG S2 [file mbo003183874sf2.pdf]
