# Supplementary material for: Comparative Genomics Reveals the Core Gene Toolbox for the Fungus-Insect Symbiosis
Source: mBio. 2018 May 15;9(3):e00636-18. doi: 10.1128/mBio.00636-18 (PMC5954228; doi:10.1128/mBio.00636-18)
Supplement: TABLE S1 [file mbo003183874st1.pdf]

Supplementary Table S1. 1,071 single-copy genes identified from the four Harpellales taxa with the smallest genomes (*Capniomyces stellatus, Furculomyces boomerangus, Smittium angustum,* and *Zancudomyces culisetae*; 25-28 Mb). In total, 582 of the 1,071 orthologous groups (ranked at the top) were selected for further phylogenetic analyses (at least one of the remaining five taxa have minimum of two copies). Each taxon is labeled with the locus name individually: *Capniomyces stellatus* (CSE02)*, Furculomyces boomerangus* (BB559)*, Smittium angustum* (BB558)*, S. megazygosporum* (BB560)*, S. mucronatum* (AYI68)*, S. simulii* (BB561)*, S. culicis* strains GSMNP (AYI70) and ID206W2 (AYI69), and *Zancudomyces culisetae* (AX774).

ORTHOMCL	AYI68	AYI69	AYI70	BB560	BB561	BB558	BB559	AX774	CSE02
ORTHO_00203	4	1	2	3	4	1	1	1	1
ORTHO_00314	3	2	3	1	2	1	1	1	1
ORTHO_00360	2	2	3	2	1	1	1	1	1
ORTHO_00365	2	2	2	3	1	1	1	1	1
ORTHO_00364	1	2	3	2	2	1	1	1	1
ORTHO_00480	1	2	2	2	2	1	1	1	1
ORTHO_00359	3	3	2	1	1	1	1	1	1
ORTHO_00509	2	3	2	1	1	1	1	1	1
ORTHO_00625	2	2	3	1	0	1	1	1	1
ORTHO_00615	2	2	2	1	1	1	1	1	1
ORTHO_00723	2	2	2	1	1	1	1	1	1
ORTHO_00730	2	2	2	1	1	1	1	1	1
ORTHO_00736	2	2	2	1	1	1	1	1	1
ORTHO_01050	2	2	2	1	0	1	1	1	1
ORTHO_01085	2	2	2	0	1	1	1	1	1
ORTHO_00738	2	2	1	2	1	1	1	1	1
ORTHO_00803	2	2	1	2	1	1	1	1	1
ORTHO_02253	2	2	0	2	0	1	1	1	1
ORTHO_00370	2	1	4	2	1	1	1	1	1
ORTHO_00472	2	1	2	0	4	1	1	1	1
ORTHO_00986	2	0	2	2	1	1	1	1	1
ORTHO_00399	1	3	2	3	1	1	1	1	1
ORTHO_00498	1	3	2	1	2	1	1	1	1
ORTHO_00435	1	2	3	2	1	1	1	1	1
ORTHO_00459	1	2	3	2	1	1	1	1	1

ORTHO_00506	1	2	3	1	2	1	1	1	1
ORTHO_00473	1	2	2	3	1	1	1	1	1
ORTHO_00672	1	2	2	2	1	1	1	1	1
ORTHO_00801	1	2	2	2	1	1	1	1	1
ORTHO_00960	1	2	2	2	0	1	1	1	1
ORTHO_00637	1	1	2	2	2	1	1	1	1
ORTHO_00689	0	2	2	3	1	1	1	1	1
ORTHO_00983	3	1	2	1	0	1	1	1	1
ORTHO_01229	2	2	1	1	1	1	1	1	1
ORTHO_01287	2	2	1	1	1	1	1	1	1
ORTHO_01312	2	2	1	1	1	1	1	1	1
ORTHO_01329	2	2	1	1	1	1	1	1	1
ORTHO_01334	2	2	1	1	1	1	1	1	1
ORTHO_01339	2	2	1	1	1	1	1	1	1
ORTHO_01389	2	2	1	1	1	1	1	1	1
ORTHO_01391	2	2	1	1	1	1	1	1	1
ORTHO_01393	2	2	1	1	1	1	1	1	1
ORTHO_02191	2	2	1	0	1	1	1	1	1
ORTHO_03178	2	2	0	1	0	1	1	1	1
ORTHO_00436	2	1	4	1	1	1	1	1	1
ORTHO_00600	2	1	3	1	1	1	1	1	1
ORTHO_00609	2	1	3	1	1	1	1	1	1
ORTHO_00611	2	1	3	1	1	1	1	1	1
ORTHO_01049	2	1	3	1	0	1	1	1	1
ORTHO_00944	2	1	2	1	1	1	1	1	1
ORTHO_00950	2	1	2	1	1	1	1	1	1
ORTHO_00962	2	1	2	1	1	1	1	1	1
ORTHO_01008	2	1	2	1	1	1	1	1	1
ORTHO_01034	2	1	2	1	1	1	1	1	1
ORTHO_01119	2	1	2	1	1	1	1	1	1
ORTHO_01203	2	1	2	1	1	1	1	1	1
ORTHO_01137	2	1	1	2	1	1	1	1	1

ORTHO_01154	2	1	1	2	1	1	1	1	1
ORTHO_01174	2	1	1	2	1	1	1	1	1
ORTHO_01315	2	1	1	2	1	1	1	1	1
ORTHO_03298	2	0	2	0	0	1	1	1	1
ORTHO_02132	2	0	1	1	2	1	1	1	1
ORTHO_00554	1	4	2	1	1	1	1	1	1
ORTHO_00490	1	3	3	1	1	1	1	1	1
ORTHO_00654	1	3	2	1	1	1	1	1	1
ORTHO_00659	1	3	2	1	1	1	1	1	1
ORTHO_00714	1	3	2	1	1	1	1	1	1
ORTHO_00911	1	3	2	0	1	1	1	1	1
ORTHO_01039	1	3	2	0	1	1	1	1	1
ORTHO_00807	1	3	1	2	1	1	1	1	1
ORTHO_00661	1	2	4	0	1	1	1	1	1
ORTHO_00707	1	2	4	0	1	1	1	1	1
ORTHO_00602	1	2	3	1	1	1	1	1	1
ORTHO_00618	1	2	3	1	1	1	1	1	1
ORTHO_00629	1	2	3	1	1	1	1	1	1
ORTHO_00646	1	2	3	1	1	1	1	1	1
ORTHO_00660	1	2	3	1	1	1	1	1	1
ORTHO_01014	1	2	3	1	0	1	1	1	1
ORTHO_00921	1	2	3	0	1	1	1	1	1
ORTHO_00939	1	2	2	1	1	1	1	1	1
ORTHO_00951	1	2	2	1	1	1	1	1	1
ORTHO_00952	1	2	2	1	1	1	1	1	1
ORTHO_00977	1	2	2	1	1	1	1	1	1
ORTHO_00979	1	2	2	1	1	1	1	1	1
ORTHO_00981	1	2	2	1	1	1	1	1	1
ORTHO_00996	1	2	2	1	1	1	1	1	1
ORTHO_01009	1	2	2	1	1	1	1	1	1
ORTHO_01015	1	2	2	1	1	1	1	1	1
ORTHO_01037	1	2	2	1	1	1	1	1	1

ORTHO_01045	1	2	2	1	1	1	1	1	1
ORTHO_01053	1	2	2	1	1	1	1	1	1
ORTHO_01055	1	2	2	1	1	1	1	1	1
ORTHO_01062	1	2	2	1	1	1	1	1	1
ORTHO_01075	1	2	2	1	1	1	1	1	1
ORTHO_01080	1	2	2	1	1	1	1	1	1
ORTHO_01083	1	2	2	1	1	1	1	1	1
ORTHO_01086	1	2	2	1	1	1	1	1	1
ORTHO_01105	1	2	2	1	1	1	1	1	1
ORTHO_01113	1	2	2	1	1	1	1	1	1
ORTHO_01146	1	2	2	1	1	1	1	1	1
ORTHO_01160	1	2	2	1	1	1	1	1	1
ORTHO_01177	1	2	2	1	1	1	1	1	1
ORTHO_01181	1	2	2	1	1	1	1	1	1
ORTHO_01243	1	2	2	1	1	1	1	1	1
ORTHO_01246	1	2	2	1	1	1	1	1	1
ORTHO_01383	1	2	2	1	1	1	1	1	1
ORTHO_01507	1	2	2	1	0	1	1	1	1
ORTHO_01628	1	2	2	1	0	1	1	1	1
ORTHO_01637	1	2	2	1	0	1	1	1	1
ORTHO_01469	1	2	2	0	1	1	1	1	1
ORTHO_01470	1	2	2	0	1	1	1	1	1
ORTHO_01506	1	2	2	0	1	1	1	1	1
ORTHO_01554	1	2	2	0	1	1	1	1	1
ORTHO_01816	1	2	2	0	1	1	1	1	1
ORTHO_00799	1	2	1	3	1	1	1	1	1
ORTHO_00811	1	2	1	3	1	1	1	1	1
ORTHO_00860	1	2	1	3	1	1	1	1	1
ORTHO_01231	1	2	1	2	1	1	1	1	1
ORTHO_01241	1	2	1	2	1	1	1	1	1
ORTHO_01299	1	2	1	2	1	1	1	1	1
ORTHO_01313	1	2	1	2	1	1	1	1	1

ORTHO_01317	1	2	1	2	1	1	1	1	1
ORTHO_01377	1	2	1	2	1	1	1	1	1
ORTHO_01126	1	2	0	3	1	1	1	1	1
ORTHO_00500	1	1	4	2	1	1	1	1	1
ORTHO_00589	1	1	3	2	1	1	1	1	1
ORTHO_00621	1	1	3	2	1	1	1	1	1
ORTHO_00630	1	1	3	2	1	1	1	1	1
ORTHO_01017	1	1	3	2	0	1	1	1	1
ORTHO_01038	1	1	3	2	0	1	1	1	1
ORTHO_00482	1	1	2	4	1	1	1	1	1
ORTHO_00612	1	1	2	3	1	1	1	1	1
ORTHO_00663	1	1	2	3	1	1	1	1	1
ORTHO_00680	1	1	2	3	1	1	1	1	1
ORTHO_00693	1	1	2	3	1	1	1	1	1
ORTHO_00712	1	1	2	3	1	1	1	1	1
ORTHO_00731	1	1	2	3	1	1	1	1	1
ORTHO_00749	1	1	2	3	1	1	1	1	1
ORTHO_00914	1	1	2	2	1	1	1	1	1
ORTHO_00920	1	1	2	2	1	1	1	1	1
ORTHO_00943	1	1	2	2	1	1	1	1	1
ORTHO_00984	1	1	2	2	1	1	1	1	1
ORTHO_00990	1	1	2	2	1	1	1	1	1
ORTHO_01000	1	1	2	2	1	1	1	1	1
ORTHO_01010	1	1	2	2	1	1	1	1	1
ORTHO_01047	1	1	2	2	1	1	1	1	1
ORTHO_01057	1	1	2	2	1	1	1	1	1
ORTHO_01070	1	1	2	2	1	1	1	1	1
ORTHO_01092	1	1	2	2	1	1	1	1	1
ORTHO_01093	1	1	2	2	1	1	1	1	1
ORTHO_01102	1	1	2	2	1	1	1	1	1
ORTHO_01107	1	1	2	2	1	1	1	1	1
ORTHO_01116	1	1	2	2	1	1	1	1	1

ORTHO_01708	1	1	2	2	0	1	1	1	1
ORTHO_01843	1	1	2	2	0	1	1	1	1
ORTHO_00604	1	1	2	1	3	1	1	1	1
ORTHO_00955	1	1	2	1	2	1	1	1	1
ORTHO_00991	1	1	2	1	2	1	1	1	1
ORTHO_01089	1	1	2	1	2	1	1	1	1
ORTHO_01581	1	1	2	0	2	1	1	1	1
ORTHO_00553	1	1	1	4	2	1	1	1	1
ORTHO_01255	1	1	1	2	2	1	1	1	1
ORTHO_01358	1	1	1	2	2	1	1	1	1
ORTHO_00889	1	1	0	4	2	1	1	1	1
ORTHO_01111	1	0	2	3	1	1	1	1	1
ORTHO_01485	1	0	2	2	1	1	1	1	1
ORTHO_02364	1	0	2	2	0	1	1	1	1
ORTHO_02418	1	0	2	2	0	1	1	1	1
ORTHO_00718	0	4	2	1	1	1	1	1	1
ORTHO_00926	0	3	2	1	1	1	1	1	1
ORTHO_01328	0	3	1	1	2	1	1	1	1
ORTHO_00633	0	2	4	1	1	1	1	1	1
ORTHO_00900	0	2	3	1	1	1	1	1	1
ORTHO_01456	0	2	2	1	1	1	1	1	1
ORTHO_01494	0	2	2	1	1	1	1	1	1
ORTHO_01568	0	2	2	1	1	1	1	1	1
ORTHO_01598	0	2	2	1	1	1	1	1	1
ORTHO_01620	0	2	2	1	1	1	1	1	1
ORTHO_01625	0	2	2	1	1	1	1	1	1
ORTHO_01690	0	2	2	1	1	1	1	1	1
ORTHO_02472	0	2	2	1	0	1	1	1	1
ORTHO_01212	0	2	1	4	0	1	1	1	1
ORTHO_01244	0	2	1	3	1	1	1	1	1
ORTHO_01760	0	2	1	2	1	1	1	1	1
ORTHO_01850	0	2	1	2	1	1	1	1	1

ORTHO_00666	0	1	3	3	1	1	1	1	1
ORTHO_01007	0	1	3	2	1	1	1	1	1
ORTHO_01025	0	1	2	3	1	1	1	1	1
ORTHO_01596	0	1	2	2	1	1	1	1	1
ORTHO_01630	0	1	2	2	1	1	1	1	1
ORTHO_01683	0	1	2	2	1	1	1	1	1
ORTHO_02352	0	1	2	2	0	1	1	1	1
ORTHO_02367	0	1	2	2	0	1	1	1	1
ORTHO_01508	0	0	2	4	0	1	1	1	1
ORTHO_03106	3	0	0	1	1	1	1	1	1
ORTHO_01753	2	1	1	1	1	1	1	1	1
ORTHO_01758	2	1	1	1	1	1	1	1	1
ORTHO_01765	2	1	1	1	1	1	1	1	1
ORTHO_01769	2	1	1	1	1	1	1	1	1
ORTHO_01811	2	1	1	1	1	1	1	1	1
ORTHO_01819	2	1	1	1	1	1	1	1	1
ORTHO_01831	2	1	1	1	1	1	1	1	1
ORTHO_01900	2	1	1	1	1	1	1	1	1
ORTHO_01924	2	1	1	1	1	1	1	1	1
ORTHO_01925	2	1	1	1	1	1	1	1	1
ORTHO_01927	2	1	1	1	1	1	1	1	1
ORTHO_02007	2	1	1	1	1	1	1	1	1
ORTHO_02100	2	1	1	1	1	1	1	1	1
ORTHO_02121	2	1	1	1	1	1	1	1	1
ORTHO_02167	2	1	1	1	1	1	1	1	1
ORTHO_02181	2	1	1	1	1	1	1	1	1
ORTHO_02637	2	1	1	1	0	1	1	1	1
ORTHO_02553	2	1	1	0	1	1	1	1	1
ORTHO_02685	2	1	1	0	1	1	1	1	1
ORTHO_02947	2	1	1	0	1	1	1	1	1
ORTHO_02953	2	1	1	0	1	1	1	1	1
ORTHO_03462	2	1	1	0	0	1	1	1	1

ORTHO_03725	2	1	1	0	0	1	1	1	1
ORTHO_03914	2	1	0	1	0	1	1	1	1
ORTHO_03927	2	1	0	0	1	1	1	1	1
ORTHO_02840	2	0	1	1	1	1	1	1	1
ORTHO_02849	2	0	1	1	1	1	1	1	1
ORTHO_02971	2	0	1	1	1	1	1	1	1
ORTHO_03792	2	0	1	0	1	1	1	1	1
ORTHO_03821	2	0	1	0	1	1	1	1	1
ORTHO_04273	2	0	1	0	0	1	1	1	1
ORTHO_03961	2	0	0	1	1	1	1	1	1
ORTHO_01220	1	3	1	1	1	1	1	1	1
ORTHO_01755	1	3	1	1	0	1	1	1	1
ORTHO_01721	1	2	1	1	1	1	1	1	1
ORTHO_01730	1	2	1	1	1	1	1	1	1
ORTHO_01754	1	2	1	1	1	1	1	1	1
ORTHO_01775	1	2	1	1	1	1	1	1	1
ORTHO_01795	1	2	1	1	1	1	1	1	1
ORTHO_01798	1	2	1	1	1	1	1	1	1
ORTHO_01800	1	2	1	1	1	1	1	1	1
ORTHO_01807	1	2	1	1	1	1	1	1	1
ORTHO_01849	1	2	1	1	1	1	1	1	1
ORTHO_01856	1	2	1	1	1	1	1	1	1
ORTHO_01858	1	2	1	1	1	1	1	1	1
ORTHO_01875	1	2	1	1	1	1	1	1	1
ORTHO_01882	1	2	1	1	1	1	1	1	1
ORTHO_01894	1	2	1	1	1	1	1	1	1
ORTHO_01899	1	2	1	1	1	1	1	1	1
ORTHO_01941	1	2	1	1	1	1	1	1	1
ORTHO_01960	1	2	1	1	1	1	1	1	1
ORTHO_01965	1	2	1	1	1	1	1	1	1
ORTHO_01974	1	2	1	1	1	1	1	1	1
ORTHO_02006	1	2	1	1	1	1	1	1	1

ORTHO_02028	1	2	1	1	1	1	1	1	1
ORTHO_02077	1	2	1	1	1	1	1	1	1
ORTHO_02089	1	2	1	1	1	1	1	1	1
ORTHO_02092	1	2	1	1	1	1	1	1	1
ORTHO_02096	1	2	1	1	1	1	1	1	1
ORTHO_02113	1	2	1	1	1	1	1	1	1
ORTHO_02127	1	2	1	1	1	1	1	1	1
ORTHO_02131	1	2	1	1	1	1	1	1	1
ORTHO_02156	1	2	1	1	1	1	1	1	1
ORTHO_02157	1	2	1	1	1	1	1	1	1
ORTHO_02170	1	2	1	1	1	1	1	1	1
ORTHO_02186	1	2	1	1	1	1	1	1	1
ORTHO_02189	1	2	1	1	1	1	1	1	1
ORTHO_02207	1	2	1	1	1	1	1	1	1
ORTHO_02208	1	2	1	1	1	1	1	1	1
ORTHO_02211	1	2	1	1	1	1	1	1	1
ORTHO_02221	1	2	1	1	1	1	1	1	1
ORTHO_02479	1	2	1	1	0	1	1	1	1
ORTHO_02552	1	2	1	1	0	1	1	1	1
ORTHO_02612	1	2	1	1	0	1	1	1	1
ORTHO_02764	1	2	1	1	0	1	1	1	1
ORTHO_02783	1	2	1	1	0	1	1	1	1
ORTHO_02861	1	2	1	1	0	1	1	1	1
ORTHO_02898	1	2	1	1	0	1	1	1	1
ORTHO_02963	1	2	1	1	0	1	1	1	1
ORTHO_03007	1	2	1	1	0	1	1	1	1
ORTHO_02549	1	2	1	0	1	1	1	1	1
ORTHO_02568	1	2	1	0	1	1	1	1	1
ORTHO_02634	1	2	1	0	1	1	1	1	1
ORTHO_02778	1	2	1	0	1	1	1	1	1
ORTHO_02855	1	2	1	0	1	1	1	1	1
ORTHO_02858	1	2	1	0	1	1	1	1	1

ORTHO_02889	1	2	1	0	1	1	1	1	1
ORTHO_03004	1	2	1	0	1	1	1	1	1
ORTHO_03749	1	2	1	0	0	1	1	1	1
ORTHO_03775	1	2	1	0	0	1	1	1	1
ORTHO_03777	1	2	1	0	0	1	1	1	1
ORTHO_02847	1	2	0	1	1	1	1	1	1
ORTHO_03126	1	2	0	1	1	1	1	1	1
ORTHO_03130	1	2	0	1	1	1	1	1	1
ORTHO_03134	1	2	0	1	1	1	1	1	1
ORTHO_03143	1	2	0	1	1	1	1	1	1
ORTHO_03166	1	2	0	1	1	1	1	1	1
ORTHO_03172	1	2	0	1	1	1	1	1	1
ORTHO_03939	1	2	0	1	0	1	1	1	1
ORTHO_03979	1	2	0	1	0	1	1	1	1
ORTHO_03909	1	2	0	0	1	1	1	1	1
ORTHO_03971	1	2	0	0	1	1	1	1	1
ORTHO_00610	1	1	4	1	1	1	1	1	1
ORTHO_00657	1	1	4	1	1	1	1	1	1
ORTHO_00727	1	1	4	1	1	1	1	1	1
ORTHO_00913	1	1	3	1	1	1	1	1	1
ORTHO_00929	1	1	3	1	1	1	1	1	1
ORTHO_00930	1	1	3	1	1	1	1	1	1
ORTHO_00932	1	1	3	1	1	1	1	1	1
ORTHO_00938	1	1	3	1	1	1	1	1	1
ORTHO_00942	1	1	3	1	1	1	1	1	1
ORTHO_00976	1	1	3	1	1	1	1	1	1
ORTHO_00992	1	1	3	1	1	1	1	1	1
ORTHO_00999	1	1	3	1	1	1	1	1	1
ORTHO_01023	1	1	3	1	1	1	1	1	1
ORTHO_01033	1	1	3	1	1	1	1	1	1
ORTHO_01036	1	1	3	1	1	1	1	1	1
ORTHO_01073	1	1	3	1	1	1	1	1	1

ORTHO_01100	1	1	3	1	1	1	1	1	1
ORTHO_01109	1	1	3	1	1	1	1	1	1
ORTHO_01588	1	1	3	1	0	1	1	1	1
ORTHO_01643	1	1	3	1	0	1	1	1	1
ORTHO_01504	1	1	3	0	1	1	1	1	1
ORTHO_01567	1	1	3	0	1	1	1	1	1
ORTHO_01692	1	1	3	0	1	1	1	1	1
ORTHO_01435	1	1	2	1	1	1	1	1	1
ORTHO_01436	1	1	2	1	1	1	1	1	1
ORTHO_01438	1	1	2	1	1	1	1	1	1
ORTHO_01447	1	1	2	1	1	1	1	1	1
ORTHO_01448	1	1	2	1	1	1	1	1	1
ORTHO_01450	1	1	2	1	1	1	1	1	1
ORTHO_01452	1	1	2	1	1	1	1	1	1
ORTHO_01453	1	1	2	1	1	1	1	1	1
ORTHO_01458	1	1	2	1	1	1	1	1	1
ORTHO_01461	1	1	2	1	1	1	1	1	1
ORTHO_01463	1	1	2	1	1	1	1	1	1
ORTHO_01467	1	1	2	1	1	1	1	1	1
ORTHO_01472	1	1	2	1	1	1	1	1	1
ORTHO_01474	1	1	2	1	1	1	1	1	1
ORTHO_01479	1	1	2	1	1	1	1	1	1
ORTHO_01482	1	1	2	1	1	1	1	1	1
ORTHO_01484	1	1	2	1	1	1	1	1	1
ORTHO_01486	1	1	2	1	1	1	1	1	1
ORTHO_01487	1	1	2	1	1	1	1	1	1
ORTHO_01489	1	1	2	1	1	1	1	1	1
ORTHO_01498	1	1	2	1	1	1	1	1	1
ORTHO_01505	1	1	2	1	1	1	1	1	1
ORTHO_01518	1	1	2	1	1	1	1	1	1
ORTHO_01522	1	1	2	1	1	1	1	1	1
ORTHO_01526	1	1	2	1	1	1	1	1	1

ORTHO_01529	1	1	2	1	1	1	1	1	1
ORTHO_01535	1	1	2	1	1	1	1	1	1
ORTHO_01537	1	1	2	1	1	1	1	1	1
ORTHO_01538	1	1	2	1	1	1	1	1	1
ORTHO_01543	1	1	2	1	1	1	1	1	1
ORTHO_01546	1	1	2	1	1	1	1	1	1
ORTHO_01548	1	1	2	1	1	1	1	1	1
ORTHO_01553	1	1	2	1	1	1	1	1	1
ORTHO_01559	1	1	2	1	1	1	1	1	1
ORTHO_01571	1	1	2	1	1	1	1	1	1
ORTHO_01573	1	1	2	1	1	1	1	1	1
ORTHO_01574	1	1	2	1	1	1	1	1	1
ORTHO_01576	1	1	2	1	1	1	1	1	1
ORTHO_01579	1	1	2	1	1	1	1	1	1
ORTHO_01580	1	1	2	1	1	1	1	1	1
ORTHO_01582	1	1	2	1	1	1	1	1	1
ORTHO_01585	1	1	2	1	1	1	1	1	1
ORTHO_01586	1	1	2	1	1	1	1	1	1
ORTHO_01587	1	1	2	1	1	1	1	1	1
ORTHO_01589	1	1	2	1	1	1	1	1	1
ORTHO_01599	1	1	2	1	1	1	1	1	1
ORTHO_01603	1	1	2	1	1	1	1	1	1
ORTHO_01608	1	1	2	1	1	1	1	1	1
ORTHO_01624	1	1	2	1	1	1	1	1	1
ORTHO_01631	1	1	2	1	1	1	1	1	1
ORTHO_01632	1	1	2	1	1	1	1	1	1
ORTHO_01636	1	1	2	1	1	1	1	1	1
ORTHO_01652	1	1	2	1	1	1	1	1	1
ORTHO_01654	1	1	2	1	1	1	1	1	1
ORTHO_01655	1	1	2	1	1	1	1	1	1
ORTHO_01660	1	1	2	1	1	1	1	1	1
ORTHO_01661	1	1	2	1	1	1	1	1	1

ORTHO_01663	1	1	2	1	1	1	1	1	1
ORTHO_01664	1	1	2	1	1	1	1	1	1
ORTHO_01668	1	1	2	1	1	1	1	1	1
ORTHO_01669	1	1	2	1	1	1	1	1	1
ORTHO_01675	1	1	2	1	1	1	1	1	1
ORTHO_01676	1	1	2	1	1	1	1	1	1
ORTHO_01678	1	1	2	1	1	1	1	1	1
ORTHO_01685	1	1	2	1	1	1	1	1	1
ORTHO_01689	1	1	2	1	1	1	1	1	1
ORTHO_01691	1	1	2	1	1	1	1	1	1
ORTHO_01693	1	1	2	1	1	1	1	1	1
ORTHO_01695	1	1	2	1	1	1	1	1	1
ORTHO_01700	1	1	2	1	1	1	1	1	1
ORTHO_01707	1	1	2	1	1	1	1	1	1
ORTHO_01709	1	1	2	1	1	1	1	1	1
ORTHO_01711	1	1	2	1	1	1	1	1	1
ORTHO_01712	1	1	2	1	1	1	1	1	1
ORTHO_01714	1	1	2	1	1	1	1	1	1
ORTHO_01715	1	1	2	1	1	1	1	1	1
ORTHO_01716	1	1	2	1	1	1	1	1	1
ORTHO_01740	1	1	2	1	1	1	1	1	1
ORTHO_01749	1	1	2	1	1	1	1	1	1
ORTHO_01757	1	1	2	1	1	1	1	1	1
ORTHO_01779	1	1	2	1	1	1	1	1	1
ORTHO_01793	1	1	2	1	1	1	1	1	1
ORTHO_01870	1	1	2	1	1	1	1	1	1
ORTHO_01881	1	1	2	1	1	1	1	1	1
ORTHO_01915	1	1	2	1	1	1	1	1	1
ORTHO_02010	1	1	2	1	1	1	1	1	1
ORTHO_02049	1	1	2	1	1	1	1	1	1
ORTHO_02056	1	1	2	1	1	1	1	1	1
ORTHO_02264	1	1	2	1	0	1	1	1	1

ORTHO_02272	1	1	2	1	0	1	1	1	1
ORTHO_02287	1	1	2	1	0	1	1	1	1
ORTHO_02294	1	1	2	1	0	1	1	1	1
ORTHO_02299	1	1	2	1	0	1	1	1	1
ORTHO_02324	1	1	2	1	0	1	1	1	1
ORTHO_02356	1	1	2	1	0	1	1	1	1
ORTHO_02370	1	1	2	1	0	1	1	1	1
ORTHO_02377	1	1	2	1	0	1	1	1	1
ORTHO_02388	1	1	2	1	0	1	1	1	1
ORTHO_02390	1	1	2	1	0	1	1	1	1
ORTHO_02393	1	1	2	1	0	1	1	1	1
ORTHO_02410	1	1	2	1	0	1	1	1	1
ORTHO_02424	1	1	2	1	0	1	1	1	1
ORTHO_02437	1	1	2	1	0	1	1	1	1
ORTHO_02606	1	1	2	1	0	1	1	1	1
ORTHO_02623	1	1	2	1	0	1	1	1	1
ORTHO_02711	1	1	2	1	0	1	1	1	1
ORTHO_02948	1	1	2	1	0	1	1	1	1
ORTHO_02277	1	1	2	0	1	1	1	1	1
ORTHO_02278	1	1	2	0	1	1	1	1	1
ORTHO_02290	1	1	2	0	1	1	1	1	1
ORTHO_02306	1	1	2	0	1	1	1	1	1
ORTHO_02331	1	1	2	0	1	1	1	1	1
ORTHO_02348	1	1	2	0	1	1	1	1	1
ORTHO_02351	1	1	2	0	1	1	1	1	1
ORTHO_02365	1	1	2	0	1	1	1	1	1
ORTHO_02372	1	1	2	0	1	1	1	1	1
ORTHO_02422	1	1	2	0	1	1	1	1	1
ORTHO_02443	1	1	2	0	1	1	1	1	1
ORTHO_02453	1	1	2	0	1	1	1	1	1
ORTHO_02464	1	1	2	0	1	1	1	1	1
ORTHO_03229	1	1	2	0	0	1	1	1	1

ORTHO_00823	1	1	1	4	1	1	1	1	1
ORTHO_01194	1	1	1	3	1	1	1	1	1
ORTHO_01293	1	1	1	3	1	1	1	1	1
ORTHO_01748	1	1	1	2	1	1	1	1	1
ORTHO_01756	1	1	1	2	1	1	1	1	1
ORTHO_01764	1	1	1	2	1	1	1	1	1
ORTHO_01768	1	1	1	2	1	1	1	1	1
ORTHO_01776	1	1	1	2	1	1	1	1	1
ORTHO_01809	1	1	1	2	1	1	1	1	1
ORTHO_01810	1	1	1	2	1	1	1	1	1
ORTHO_01825	1	1	1	2	1	1	1	1	1
ORTHO_01832	1	1	1	2	1	1	1	1	1
ORTHO_01861	1	1	1	2	1	1	1	1	1
ORTHO_01883	1	1	1	2	1	1	1	1	1
ORTHO_01888	1	1	1	2	1	1	1	1	1
ORTHO_01937	1	1	1	2	1	1	1	1	1
ORTHO_01981	1	1	1	2	1	1	1	1	1
ORTHO_01983	1	1	1	2	1	1	1	1	1
ORTHO_01995	1	1	1	2	1	1	1	1	1
ORTHO_02000	1	1	1	2	1	1	1	1	1
ORTHO_02041	1	1	1	2	1	1	1	1	1
ORTHO_02054	1	1	1	2	1	1	1	1	1
ORTHO_02066	1	1	1	2	1	1	1	1	1
ORTHO_02085	1	1	1	2	1	1	1	1	1
ORTHO_02116	1	1	1	2	1	1	1	1	1
ORTHO_02176	1	1	1	2	1	1	1	1	1
ORTHO_02182	1	1	1	2	1	1	1	1	1
ORTHO_02201	1	1	1	2	1	1	1	1	1
ORTHO_02203	1	1	1	2	1	1	1	1	1
ORTHO_02214	1	1	1	2	1	1	1	1	1
ORTHO_02572	1	1	1	2	0	1	1	1	1
ORTHO_02715	1	1	1	2	0	1	1	1	1

ORTHO_02727	1	1	1	2	0	1	1	1	1
ORTHO_02779	1	1	1	2	0	1	1	1	1
ORTHO_02802	1	1	1	2	0	1	1	1	1
ORTHO_02842	1	1	1	2	0	1	1	1	1
ORTHO_02866	1	1	1	2	0	1	1	1	1
ORTHO_02955	1	1	1	2	0	1	1	1	1
ORTHO_03026	1	1	1	2	0	1	1	1	1
ORTHO_01362	1	1	1	1	3	1	1	1	1
ORTHO_01836	1	1	1	1	2	1	1	1	1
ORTHO_01848	1	1	1	1	2	1	1	1	1
ORTHO_01939	1	1	1	1	2	1	1	1	1
ORTHO_02014	1	1	1	1	2	1	1	1	1
ORTHO_02020	1	1	1	1	2	1	1	1	1
ORTHO_02045	1	1	1	1	2	1	1	1	1
ORTHO_02820	1	1	1	0	2	1	1	1	1
ORTHO_02237	1	1	0	3	1	1	1	1	1
ORTHO_03122	1	1	0	2	1	1	1	1	1
ORTHO_03150	1	1	0	2	1	1	1	1	1
ORTHO_03160	1	1	0	2	1	1	1	1	1
ORTHO_03161	1	1	0	2	1	1	1	1	1
ORTHO_03503	1	1	0	2	0	1	1	1	1
ORTHO_03117	1	1	0	1	2	1	1	1	1
ORTHO_03115	1	1	0	0	3	1	1	1	1
ORTHO_00997	1	0	4	1	1	1	1	1	1
ORTHO_01462	1	0	3	1	1	1	1	1	1
ORTHO_01578	1	0	3	1	1	1	1	1	1
ORTHO_01670	1	0	3	1	1	1	1	1	1
ORTHO_01791	1	0	3	1	1	1	1	1	1
ORTHO_02403	1	0	3	0	1	1	1	1	1
ORTHO_03230	1	0	3	0	0	1	1	1	1
ORTHO_02266	1	0	2	1	1	1	1	1	1
ORTHO_02280	1	0	2	1	1	1	1	1	1

ORTHO_02308	1	0	2	1	1	1	1	1	1
ORTHO_02317	1	0	2	1	1	1	1	1	1
ORTHO_02347	1	0	2	1	1	1	1	1	1
ORTHO_02358	1	0	2	1	1	1	1	1	1
ORTHO_02366	1	0	2	1	1	1	1	1	1
ORTHO_02368	1	0	2	1	1	1	1	1	1
ORTHO_02406	1	0	2	1	1	1	1	1	1
ORTHO_02456	1	0	2	1	1	1	1	1	1
ORTHO_02460	1	0	2	1	1	1	1	1	1
ORTHO_03297	1	0	2	1	0	1	1	1	1
ORTHO_03269	1	0	2	0	1	1	1	1	1
ORTHO_04006	1	0	2	0	0	1	1	1	1
ORTHO_02484	1	0	1	2	1	1	1	1	1
ORTHO_02486	1	0	1	2	1	1	1	1	1
ORTHO_02664	1	0	1	2	1	1	1	1	1
ORTHO_02677	1	0	1	2	1	1	1	1	1
ORTHO_02914	1	0	1	2	1	1	1	1	1
ORTHO_02996	1	0	1	2	1	1	1	1	1
ORTHO_03017	1	0	1	2	1	1	1	1	1
ORTHO_03021	1	0	1	2	1	1	1	1	1
ORTHO_03053	1	0	1	2	1	1	1	1	1
ORTHO_03056	1	0	1	2	1	1	1	1	1
ORTHO_03817	1	0	1	2	0	1	1	1	1
ORTHO_02695	1	0	1	1	2	1	1	1	1
ORTHO_02997	1	0	1	1	2	1	1	1	1
ORTHO_03103	1	0	1	1	2	1	1	1	1
ORTHO_03395	1	0	1	0	2	1	1	1	1
ORTHO_03835	1	0	1	0	2	1	1	1	1
ORTHO_03969	1	0	0	2	1	1	1	1	1
ORTHO_03617	1	0	0	1	2	1	1	1	1
ORTHO_02183	0	3	1	1	1	1	1	1	1
ORTHO_02527	0	2	1	1	1	1	1	1	1

ORTHO_02540	0	2	1	1	1	1	1	1	1
ORTHO_02595	0	2	1	1	1	1	1	1	1
ORTHO_02697	0	2	1	1	1	1	1	1	1
ORTHO_02760	0	2	1	1	1	1	1	1	1
ORTHO_02833	0	2	1	1	1	1	1	1	1
ORTHO_02973	0	2	1	1	1	1	1	1	1
ORTHO_03040	0	2	1	1	1	1	1	1	1
ORTHO_03076	0	2	1	1	1	1	1	1	1
ORTHO_03089	0	2	1	1	1	1	1	1	1
ORTHO_03342	0	2	1	0	1	1	1	1	1
ORTHO_03963	0	2	0	1	1	1	1	1	1
ORTHO_00947	0	1	4	1	1	1	1	1	1
ORTHO_02307	0	1	4	0	0	1	1	1	1
ORTHO_02296	0	1	3	1	0	1	1	1	1
ORTHO_02404	0	1	3	0	1	1	1	1	1
ORTHO_02297	0	1	2	1	1	1	1	1	1
ORTHO_02354	0	1	2	1	1	1	1	1	1
ORTHO_02381	0	1	2	1	1	1	1	1	1
ORTHO_02465	0	1	2	1	1	1	1	1	1
ORTHO_02468	0	1	2	1	1	1	1	1	1
ORTHO_02475	0	1	2	1	1	1	1	1	1
ORTHO_03281	0	1	2	1	0	1	1	1	1
ORTHO_03302	0	1	2	1	0	1	1	1	1
ORTHO_03192	0	1	2	0	1	1	1	1	1
ORTHO_03214	0	1	2	0	1	1	1	1	1
ORTHO_03488	0	1	2	0	1	1	1	1	1
ORTHO_04011	0	1	2	0	0	1	1	1	1
ORTHO_02589	0	1	1	2	1	1	1	1	1
ORTHO_02669	0	1	1	2	1	1	1	1	1
ORTHO_02700	0	1	1	2	1	1	1	1	1
ORTHO_02885	0	1	1	2	1	1	1	1	1
ORTHO_02987	0	1	1	2	1	1	1	1	1

ORTHO_03086	0	1	1	2	1	1	1	1	1
ORTHO_03818	0	1	1	2	0	1	1	1	1
ORTHO_03841	0	1	1	2	0	1	1	1	1
ORTHO_03720	0	1	0	1	2	1	1	1	1
ORTHO_02400	0	0	3	1	1	1	1	1	1
ORTHO_03221	0	0	2	1	1	1	1	1	1
ORTHO_03283	0	0	2	1	1	1	1	1	1
ORTHO_04000	0	0	2	1	0	1	1	1	1
ORTHO_04062	0	0	2	1	0	1	1	1	1
ORTHO_03603	0	0	1	2	1	1	1	1	1
ORTHO_03882	0	0	1	2	1	1	1	1	1
ORTHO_04421	0	0	0	2	1	1	1	1	1
ORTHO_04462	0	0	0	2	1	1	1	1	1
ORTHO_02274	1	1	1	1	1	1	1	1	1
ORTHO_02480	1	1	1	1	1	1	1	1	1
ORTHO_02492	1	1	1	1	1	1	1	1	1
ORTHO_02498	1	1	1	1	1	1	1	1	1
ORTHO_02506	1	1	1	1	1	1	1	1	1
ORTHO_02509	1	1	1	1	1	1	1	1	1
ORTHO_02512	1	1	1	1	1	1	1	1	1
ORTHO_02513	1	1	1	1	1	1	1	1	1
ORTHO_02516	1	1	1	1	1	1	1	1	1
ORTHO_02526	1	1	1	1	1	1	1	1	1
ORTHO_02528	1	1	1	1	1	1	1	1	1
ORTHO_02529	1	1	1	1	1	1	1	1	1
ORTHO_02532	1	1	1	1	1	1	1	1	1
ORTHO_02534	1	1	1	1	1	1	1	1	1
ORTHO_02539	1	1	1	1	1	1	1	1	1
ORTHO_02543	1	1	1	1	1	1	1	1	1
ORTHO_02544	1	1	1	1	1	1	1	1	1
ORTHO_02546	1	1	1	1	1	1	1	1	1
ORTHO_02550	1	1	1	1	1	1	1	1	1

ORTHO_02560	1	1	1	1	1	1	1	1	1
ORTHO_02563	1	1	1	1	1	1	1	1	1
ORTHO_02567	1	1	1	1	1	1	1	1	1
ORTHO_02577	1	1	1	1	1	1	1	1	1
ORTHO_02580	1	1	1	1	1	1	1	1	1
ORTHO_02583	1	1	1	1	1	1	1	1	1
ORTHO_02593	1	1	1	1	1	1	1	1	1
ORTHO_02604	1	1	1	1	1	1	1	1	1
ORTHO_02605	1	1	1	1	1	1	1	1	1
ORTHO_02610	1	1	1	1	1	1	1	1	1
ORTHO_02616	1	1	1	1	1	1	1	1	1
ORTHO_02620	1	1	1	1	1	1	1	1	1
ORTHO_02624	1	1	1	1	1	1	1	1	1
ORTHO_02626	1	1	1	1	1	1	1	1	1
ORTHO_02627	1	1	1	1	1	1	1	1	1
ORTHO_02628	1	1	1	1	1	1	1	1	1
ORTHO_02630	1	1	1	1	1	1	1	1	1
ORTHO_02631	1	1	1	1	1	1	1	1	1
ORTHO_02636	1	1	1	1	1	1	1	1	1
ORTHO_02640	1	1	1	1	1	1	1	1	1
ORTHO_02641	1	1	1	1	1	1	1	1	1
ORTHO_02644	1	1	1	1	1	1	1	1	1
ORTHO_02650	1	1	1	1	1	1	1	1	1
ORTHO_02655	1	1	1	1	1	1	1	1	1
ORTHO_02657	1	1	1	1	1	1	1	1	1
ORTHO_02659	1	1	1	1	1	1	1	1	1
ORTHO_02663	1	1	1	1	1	1	1	1	1
ORTHO_02665	1	1	1	1	1	1	1	1	1
ORTHO_02668	1	1	1	1	1	1	1	1	1
ORTHO_02672	1	1	1	1	1	1	1	1	1
ORTHO_02679	1	1	1	1	1	1	1	1	1
ORTHO_02682	1	1	1	1	1	1	1	1	1

ORTHO_02684	1	1	1	1	1	1	1	1	1
ORTHO_02687	1	1	1	1	1	1	1	1	1
ORTHO_02688	1	1	1	1	1	1	1	1	1
ORTHO_02690	1	1	1	1	1	1	1	1	1
ORTHO_02692	1	1	1	1	1	1	1	1	1
ORTHO_02696	1	1	1	1	1	1	1	1	1
ORTHO_02698	1	1	1	1	1	1	1	1	1
ORTHO_02699	1	1	1	1	1	1	1	1	1
ORTHO_02701	1	1	1	1	1	1	1	1	1
ORTHO_02704	1	1	1	1	1	1	1	1	1
ORTHO_02712	1	1	1	1	1	1	1	1	1
ORTHO_02719	1	1	1	1	1	1	1	1	1
ORTHO_02725	1	1	1	1	1	1	1	1	1
ORTHO_02729	1	1	1	1	1	1	1	1	1
ORTHO_02733	1	1	1	1	1	1	1	1	1
ORTHO_02735	1	1	1	1	1	1	1	1	1
ORTHO_02736	1	1	1	1	1	1	1	1	1
ORTHO_02738	1	1	1	1	1	1	1	1	1
ORTHO_02743	1	1	1	1	1	1	1	1	1
ORTHO_02745	1	1	1	1	1	1	1	1	1
ORTHO_02753	1	1	1	1	1	1	1	1	1
ORTHO_02767	1	1	1	1	1	1	1	1	1
ORTHO_02768	1	1	1	1	1	1	1	1	1
ORTHO_02770	1	1	1	1	1	1	1	1	1
ORTHO_02773	1	1	1	1	1	1	1	1	1
ORTHO_02775	1	1	1	1	1	1	1	1	1
ORTHO_02777	1	1	1	1	1	1	1	1	1
ORTHO_02782	1	1	1	1	1	1	1	1	1
ORTHO_02788	1	1	1	1	1	1	1	1	1
ORTHO_02796	1	1	1	1	1	1	1	1	1
ORTHO_02797	1	1	1	1	1	1	1	1	1
ORTHO_02798	1	1	1	1	1	1	1	1	1

ORTHO_02799	1	1	1	1	1	1	1	1	1
ORTHO_02800	1	1	1	1	1	1	1	1	1
ORTHO_02805	1	1	1	1	1	1	1	1	1
ORTHO_02812	1	1	1	1	1	1	1	1	1
ORTHO_02816	1	1	1	1	1	1	1	1	1
ORTHO_02817	1	1	1	1	1	1	1	1	1
ORTHO_02819	1	1	1	1	1	1	1	1	1
ORTHO_02828	1	1	1	1	1	1	1	1	1
ORTHO_02834	1	1	1	1	1	1	1	1	1
ORTHO_02835	1	1	1	1	1	1	1	1	1
ORTHO_02837	1	1	1	1	1	1	1	1	1
ORTHO_02838	1	1	1	1	1	1	1	1	1
ORTHO_02841	1	1	1	1	1	1	1	1	1
ORTHO_02846	1	1	1	1	1	1	1	1	1
ORTHO_02850	1	1	1	1	1	1	1	1	1
ORTHO_02851	1	1	1	1	1	1	1	1	1
ORTHO_02852	1	1	1	1	1	1	1	1	1
ORTHO_02854	1	1	1	1	1	1	1	1	1
ORTHO_02857	1	1	1	1	1	1	1	1	1
ORTHO_02859	1	1	1	1	1	1	1	1	1
ORTHO_02867	1	1	1	1	1	1	1	1	1
ORTHO_02875	1	1	1	1	1	1	1	1	1
ORTHO_02880	1	1	1	1	1	1	1	1	1
ORTHO_02890	1	1	1	1	1	1	1	1	1
ORTHO_02899	1	1	1	1	1	1	1	1	1
ORTHO_02900	1	1	1	1	1	1	1	1	1
ORTHO_02901	1	1	1	1	1	1	1	1	1
ORTHO_02903	1	1	1	1	1	1	1	1	1
ORTHO_02904	1	1	1	1	1	1	1	1	1
ORTHO_02906	1	1	1	1	1	1	1	1	1
ORTHO_02908	1	1	1	1	1	1	1	1	1
ORTHO_02911	1	1	1	1	1	1	1	1	1

ORTHO_02915	1	1	1	1	1	1	1	1	1
ORTHO_02923	1	1	1	1	1	1	1	1	1
ORTHO_02924	1	1	1	1	1	1	1	1	1
ORTHO_02929	1	1	1	1	1	1	1	1	1
ORTHO_02930	1	1	1	1	1	1	1	1	1
ORTHO_02935	1	1	1	1	1	1	1	1	1
ORTHO_02936	1	1	1	1	1	1	1	1	1
ORTHO_02937	1	1	1	1	1	1	1	1	1
ORTHO_02938	1	1	1	1	1	1	1	1	1
ORTHO_02940	1	1	1	1	1	1	1	1	1
ORTHO_02941	1	1	1	1	1	1	1	1	1
ORTHO_02942	1	1	1	1	1	1	1	1	1
ORTHO_02949	1	1	1	1	1	1	1	1	1
ORTHO_02950	1	1	1	1	1	1	1	1	1
ORTHO_02951	1	1	1	1	1	1	1	1	1
ORTHO_02952	1	1	1	1	1	1	1	1	1
ORTHO_02954	1	1	1	1	1	1	1	1	1
ORTHO_02957	1	1	1	1	1	1	1	1	1
ORTHO_02959	1	1	1	1	1	1	1	1	1
ORTHO_02960	1	1	1	1	1	1	1	1	1
ORTHO_02964	1	1	1	1	1	1	1	1	1
ORTHO_02965	1	1	1	1	1	1	1	1	1
ORTHO_02967	1	1	1	1	1	1	1	1	1
ORTHO_02968	1	1	1	1	1	1	1	1	1
ORTHO_02969	1	1	1	1	1	1	1	1	1
ORTHO_02972	1	1	1	1	1	1	1	1	1
ORTHO_02975	1	1	1	1	1	1	1	1	1
ORTHO_02976	1	1	1	1	1	1	1	1	1
ORTHO_02977	1	1	1	1	1	1	1	1	1
ORTHO_02980	1	1	1	1	1	1	1	1	1
ORTHO_02983	1	1	1	1	1	1	1	1	1
ORTHO_02985	1	1	1	1	1	1	1	1	1

ORTHO_02986	1	1	1	1	1	1	1	1	1
ORTHO_02994	1	1	1	1	1	1	1	1	1
ORTHO_02999	1	1	1	1	1	1	1	1	1
ORTHO_03001	1	1	1	1	1	1	1	1	1
ORTHO_03008	1	1	1	1	1	1	1	1	1
ORTHO_03013	1	1	1	1	1	1	1	1	1
ORTHO_03016	1	1	1	1	1	1	1	1	1
ORTHO_03019	1	1	1	1	1	1	1	1	1
ORTHO_03024	1	1	1	1	1	1	1	1	1
ORTHO_03025	1	1	1	1	1	1	1	1	1
ORTHO_03027	1	1	1	1	1	1	1	1	1
ORTHO_03031	1	1	1	1	1	1	1	1	1
ORTHO_03034	1	1	1	1	1	1	1	1	1
ORTHO_03035	1	1	1	1	1	1	1	1	1
ORTHO_03037	1	1	1	1	1	1	1	1	1
ORTHO_03039	1	1	1	1	1	1	1	1	1
ORTHO_03041	1	1	1	1	1	1	1	1	1
ORTHO_03044	1	1	1	1	1	1	1	1	1
ORTHO_03045	1	1	1	1	1	1	1	1	1
ORTHO_03047	1	1	1	1	1	1	1	1	1
ORTHO_03048	1	1	1	1	1	1	1	1	1
ORTHO_03049	1	1	1	1	1	1	1	1	1
ORTHO_03059	1	1	1	1	1	1	1	1	1
ORTHO_03060	1	1	1	1	1	1	1	1	1
ORTHO_03064	1	1	1	1	1	1	1	1	1
ORTHO_03068	1	1	1	1	1	1	1	1	1
ORTHO_03069	1	1	1	1	1	1	1	1	1
ORTHO_03071	1	1	1	1	1	1	1	1	1
ORTHO_03072	1	1	1	1	1	1	1	1	1
ORTHO_03074	1	1	1	1	1	1	1	1	1
ORTHO_03077	1	1	1	1	1	1	1	1	1
ORTHO_03078	1	1	1	1	1	1	1	1	1

ORTHO_03079	1	1	1	1	1	1	1	1	1
ORTHO_03082	1	1	1	1	1	1	1	1	1
ORTHO_03090	1	1	1	1	1	1	1	1	1
ORTHO_03091	1	1	1	1	1	1	1	1	1
ORTHO_03094	1	1	1	1	1	1	1	1	1
ORTHO_03097	1	1	1	1	1	1	1	1	1
ORTHO_03100	1	1	1	1	1	1	1	1	1
ORTHO_03107	1	1	1	1	1	1	1	1	1
ORTHO_03330	1	1	1	1	0	1	1	1	1
ORTHO_03332	1	1	1	1	0	1	1	1	1
ORTHO_03333	1	1	1	1	0	1	1	1	1
ORTHO_03335	1	1	1	1	0	1	1	1	1
ORTHO_03383	1	1	1	1	0	1	1	1	1
ORTHO_03417	1	1	1	1	0	1	1	1	1
ORTHO_03423	1	1	1	1	0	1	1	1	1
ORTHO_03425	1	1	1	1	0	1	1	1	1
ORTHO_03427	1	1	1	1	0	1	1	1	1
ORTHO_03453	1	1	1	1	0	1	1	1	1
ORTHO_03458	1	1	1	1	0	1	1	1	1
ORTHO_03465	1	1	1	1	0	1	1	1	1
ORTHO_03470	1	1	1	1	0	1	1	1	1
ORTHO_03492	1	1	1	1	0	1	1	1	1
ORTHO_03504	1	1	1	1	0	1	1	1	1
ORTHO_03519	1	1	1	1	0	1	1	1	1
ORTHO_03528	1	1	1	1	0	1	1	1	1
ORTHO_03532	1	1	1	1	0	1	1	1	1
ORTHO_03556	1	1	1	1	0	1	1	1	1
ORTHO_03573	1	1	1	1	0	1	1	1	1
ORTHO_03598	1	1	1	1	0	1	1	1	1
ORTHO_03609	1	1	1	1	0	1	1	1	1
ORTHO_03627	1	1	1	1	0	1	1	1	1
ORTHO_03628	1	1	1	1	0	1	1	1	1

ORTHO_03669	1	1	1	1	0	1	1	1	1
ORTHO_03722	1	1	1	1	0	1	1	1	1
ORTHO_03724	1	1	1	1	0	1	1	1	1
ORTHO_03733	1	1	1	1	0	1	1	1	1
ORTHO_03739	1	1	1	1	0	1	1	1	1
ORTHO_03760	1	1	1	1	0	1	1	1	1
ORTHO_03779	1	1	1	1	0	1	1	1	1
ORTHO_03796	1	1	1	1	0	1	1	1	1
ORTHO_03798	1	1	1	1	0	1	1	1	1
ORTHO_03812	1	1	1	1	0	1	1	1	1
ORTHO_03834	1	1	1	1	0	1	1	1	1
ORTHO_03868	1	1	1	1	0	1	1	1	1
ORTHO_03872	1	1	1	1	0	1	1	1	1
ORTHO_03878	1	1	1	1	0	1	1	1	1
ORTHO_03881	1	1	1	1	0	1	1	1	1
ORTHO_03889	1	1	1	1	0	1	1	1	1
ORTHO_03320	1	1	1	0	1	1	1	1	1
ORTHO_03350	1	1	1	0	1	1	1	1	1
ORTHO_03361	1	1	1	0	1	1	1	1	1
ORTHO_03367	1	1	1	0	1	1	1	1	1
ORTHO_03368	1	1	1	0	1	1	1	1	1
ORTHO_03378	1	1	1	0	1	1	1	1	1
ORTHO_03396	1	1	1	0	1	1	1	1	1
ORTHO_03398	1	1	1	0	1	1	1	1	1
ORTHO_03442	1	1	1	0	1	1	1	1	1
ORTHO_03449	1	1	1	0	1	1	1	1	1
ORTHO_03484	1	1	1	0	1	1	1	1	1
ORTHO_03500	1	1	1	0	1	1	1	1	1
ORTHO_03505	1	1	1	0	1	1	1	1	1
ORTHO_03517	1	1	1	0	1	1	1	1	1
ORTHO_03522	1	1	1	0	1	1	1	1	1
ORTHO_03525	1	1	1	0	1	1	1	1	1

ORTHO_03546	1	1	1	0	1	1	1	1	1
ORTHO_03563	1	1	1	0	1	1	1	1	1
ORTHO_03572	1	1	1	0	1	1	1	1	1
ORTHO_03574	1	1	1	0	1	1	1	1	1
ORTHO_03577	1	1	1	0	1	1	1	1	1
ORTHO_03608	1	1	1	0	1	1	1	1	1
ORTHO_03610	1	1	1	0	1	1	1	1	1
ORTHO_03641	1	1	1	0	1	1	1	1	1
ORTHO_03650	1	1	1	0	1	1	1	1	1
ORTHO_03677	1	1	1	0	1	1	1	1	1
ORTHO_03699	1	1	1	0	1	1	1	1	1
ORTHO_03700	1	1	1	0	1	1	1	1	1
ORTHO_03707	1	1	1	0	1	1	1	1	1
ORTHO_03737	1	1	1	0	1	1	1	1	1
ORTHO_03743	1	1	1	0	1	1	1	1	1
ORTHO_03767	1	1	1	0	1	1	1	1	1
ORTHO_03809	1	1	1	0	1	1	1	1	1
ORTHO_03811	1	1	1	0	1	1	1	1	1
ORTHO_03815	1	1	1	0	1	1	1	1	1
ORTHO_03825	1	1	1	0	1	1	1	1	1
ORTHO_03836	1	1	1	0	1	1	1	1	1
ORTHO_03837	1	1	1	0	1	1	1	1	1
ORTHO_03853	1	1	1	0	1	1	1	1	1
ORTHO_03862	1	1	1	0	1	1	1	1	1
ORTHO_03895	1	1	1	0	1	1	1	1	1
ORTHO_04104	1	1	1	0	0	1	1	1	1
ORTHO_04135	1	1	1	0	0	1	1	1	1
ORTHO_04144	1	1	1	0	0	1	1	1	1
ORTHO_04213	1	1	1	0	0	1	1	1	1
ORTHO_04223	1	1	1	0	0	1	1	1	1
ORTHO_04236	1	1	1	0	0	1	1	1	1
ORTHO_04242	1	1	1	0	0	1	1	1	1

ORTHO_04290	1	1	1	0	0	1	1	1	1
ORTHO_04298	1	1	1	0	0	1	1	1	1
ORTHO_04304	1	1	1	0	0	1	1	1	1
ORTHO_04339	1	1	1	0	0	1	1	1	1
ORTHO_04369	1	1	1	0	0	1	1	1	1
ORTHO_03360	1	1	0	1	1	1	1	1	1
ORTHO_03364	1	1	0	1	1	1	1	1	1
ORTHO_03438	1	1	0	1	1	1	1	1	1
ORTHO_03499	1	1	0	1	1	1	1	1	1
ORTHO_03547	1	1	0	1	1	1	1	1	1
ORTHO_03569	1	1	0	1	1	1	1	1	1
ORTHO_03584	1	1	0	1	1	1	1	1	1
ORTHO_03611	1	1	0	1	1	1	1	1	1
ORTHO_03636	1	1	0	1	1	1	1	1	1
ORTHO_03649	1	1	0	1	1	1	1	1	1
ORTHO_03708	1	1	0	1	1	1	1	1	1
ORTHO_03771	1	1	0	1	1	1	1	1	1
ORTHO_03903	1	1	0	1	1	1	1	1	1
ORTHO_03907	1	1	0	1	1	1	1	1	1
ORTHO_03908	1	1	0	1	1	1	1	1	1
ORTHO_03911	1	1	0	1	1	1	1	1	1
ORTHO_03913	1	1	0	1	1	1	1	1	1
ORTHO_03919	1	1	0	1	1	1	1	1	1
ORTHO_03925	1	1	0	1	1	1	1	1	1
ORTHO_03926	1	1	0	1	1	1	1	1	1
ORTHO_03932	1	1	0	1	1	1	1	1	1
ORTHO_03934	1	1	0	1	1	1	1	1	1
ORTHO_03938	1	1	0	1	1	1	1	1	1
ORTHO_03947	1	1	0	1	1	1	1	1	1
ORTHO_03951	1	1	0	1	1	1	1	1	1
ORTHO_03960	1	1	0	1	1	1	1	1	1
ORTHO_03964	1	1	0	1	1	1	1	1	1

ORTHO_03968	1	1	0	1	1	1	1	1	1
ORTHO_03972	1	1	0	1	1	1	1	1	1
ORTHO_03974	1	1	0	1	1	1	1	1	1
ORTHO_03977	1	1	0	1	1	1	1	1	1
ORTHO_04468	1	1	0	1	0	1	1	1	1
ORTHO_04475	1	1	0	1	0	1	1	1	1
ORTHO_04478	1	1	0	1	0	1	1	1	1
ORTHO_04479	1	1	0	1	0	1	1	1	1
ORTHO_04480	1	1	0	1	0	1	1	1	1
ORTHO_04418	1	1	0	0	1	1	1	1	1
ORTHO_04438	1	1	0	0	1	1	1	1	1
ORTHO_04447	1	1	0	0	1	1	1	1	1
ORTHO_04460	1	1	0	0	1	1	1	1	1
ORTHO_04845	1	1	0	0	0	1	1	1	1
ORTHO_03319	1	0	1	1	1	1	1	1	1
ORTHO_03363	1	0	1	1	1	1	1	1	1
ORTHO_03365	1	0	1	1	1	1	1	1	1
ORTHO_03369	1	0	1	1	1	1	1	1	1
ORTHO_03388	1	0	1	1	1	1	1	1	1
ORTHO_03407	1	0	1	1	1	1	1	1	1
ORTHO_03422	1	0	1	1	1	1	1	1	1
ORTHO_03426	1	0	1	1	1	1	1	1	1
ORTHO_03428	1	0	1	1	1	1	1	1	1
ORTHO_03472	1	0	1	1	1	1	1	1	1
ORTHO_03493	1	0	1	1	1	1	1	1	1
ORTHO_03516	1	0	1	1	1	1	1	1	1
ORTHO_03523	1	0	1	1	1	1	1	1	1
ORTHO_03534	1	0	1	1	1	1	1	1	1
ORTHO_03580	1	0	1	1	1	1	1	1	1
ORTHO_03581	1	0	1	1	1	1	1	1	1
ORTHO_03590	1	0	1	1	1	1	1	1	1
ORTHO_03615	1	0	1	1	1	1	1	1	1

ORTHO_03616	1	0	1	1	1	1	1	1	1
ORTHO_03633	1	0	1	1	1	1	1	1	1
ORTHO_03645	1	0	1	1	1	1	1	1	1
ORTHO_03679	1	0	1	1	1	1	1	1	1
ORTHO_03686	1	0	1	1	1	1	1	1	1
ORTHO_03696	1	0	1	1	1	1	1	1	1
ORTHO_03726	1	0	1	1	1	1	1	1	1
ORTHO_03745	1	0	1	1	1	1	1	1	1
ORTHO_03753	1	0	1	1	1	1	1	1	1
ORTHO_03762	1	0	1	1	1	1	1	1	1
ORTHO_03785	1	0	1	1	1	1	1	1	1
ORTHO_03813	1	0	1	1	1	1	1	1	1
ORTHO_03833	1	0	1	1	1	1	1	1	1
ORTHO_03843	1	0	1	1	1	1	1	1	1
ORTHO_03866	1	0	1	1	1	1	1	1	1
ORTHO_04114	1	0	1	1	0	1	1	1	1
ORTHO_04212	1	0	1	1	0	1	1	1	1
ORTHO_04220	1	0	1	1	0	1	1	1	1
ORTHO_04356	1	0	1	1	0	1	1	1	1
ORTHO_04100	1	0	1	0	1	1	1	1	1
ORTHO_04313	1	0	1	0	1	1	1	1	1
ORTHO_04380	1	0	1	0	1	1	1	1	1
ORTHO_04405	1	0	1	0	1	1	1	1	1
ORTHO_04575	1	0	1	0	0	1	1	1	1
ORTHO_04584	1	0	1	0	0	1	1	1	1
ORTHO_04587	1	0	1	0	0	1	1	1	1
ORTHO_04623	1	0	1	0	0	1	1	1	1
ORTHO_04698	1	0	1	0	0	1	1	1	1
ORTHO_04122	1	0	0	1	1	1	1	1	1
ORTHO_04149	1	0	0	1	1	1	1	1	1
ORTHO_04302	1	0	0	1	1	1	1	1	1
ORTHO_04393	1	0	0	1	1	1	1	1	1

ORTHO_04417	1	0	0	1	1	1	1	1	1
ORTHO_04429	1	0	0	1	1	1	1	1	1
ORTHO_04436	1	0	0	1	1	1	1	1	1
ORTHO_04448	1	0	0	1	1	1	1	1	1
ORTHO_04452	1	0	0	1	1	1	1	1	1
ORTHO_04839	1	0	0	1	0	1	1	1	1
ORTHO_04846	1	0	0	1	0	1	1	1	1
ORTHO_04856	1	0	0	1	0	1	1	1	1
ORTHO_04805	1	0	0	0	1	1	1	1	1
ORTHO_04811	1	0	0	0	1	1	1	1	1
ORTHO_05034	1	0	0	0	0	1	1	1	1
ORTHO_03346	0	1	1	1	1	1	1	1	1
ORTHO_03375	0	1	1	1	1	1	1	1	1
ORTHO_03379	0	1	1	1	1	1	1	1	1
ORTHO_03382	0	1	1	1	1	1	1	1	1
ORTHO_03393	0	1	1	1	1	1	1	1	1
ORTHO_03408	0	1	1	1	1	1	1	1	1
ORTHO_03415	0	1	1	1	1	1	1	1	1
ORTHO_03424	0	1	1	1	1	1	1	1	1
ORTHO_03429	0	1	1	1	1	1	1	1	1
ORTHO_03436	0	1	1	1	1	1	1	1	1
ORTHO_03437	0	1	1	1	1	1	1	1	1
ORTHO_03459	0	1	1	1	1	1	1	1	1
ORTHO_03471	0	1	1	1	1	1	1	1	1
ORTHO_03477	0	1	1	1	1	1	1	1	1
ORTHO_03512	0	1	1	1	1	1	1	1	1
ORTHO_03544	0	1	1	1	1	1	1	1	1
ORTHO_03555	0	1	1	1	1	1	1	1	1
ORTHO_03561	0	1	1	1	1	1	1	1	1
ORTHO_03562	0	1	1	1	1	1	1	1	1
ORTHO_03579	0	1	1	1	1	1	1	1	1
ORTHO_03583	0	1	1	1	1	1	1	1	1

ORTHO_03586	0	1	1	1	1	1	1	1	1
ORTHO_03599	0	1	1	1	1	1	1	1	1
ORTHO_03612	0	1	1	1	1	1	1	1	1
ORTHO_03643	0	1	1	1	1	1	1	1	1
ORTHO_03660	0	1	1	1	1	1	1	1	1
ORTHO_03674	0	1	1	1	1	1	1	1	1
ORTHO_03691	0	1	1	1	1	1	1	1	1
ORTHO_03731	0	1	1	1	1	1	1	1	1
ORTHO_03732	0	1	1	1	1	1	1	1	1
ORTHO_03748	0	1	1	1	1	1	1	1	1
ORTHO_03752	0	1	1	1	1	1	1	1	1
ORTHO_03754	0	1	1	1	1	1	1	1	1
ORTHO_03766	0	1	1	1	1	1	1	1	1
ORTHO_03772	0	1	1	1	1	1	1	1	1
ORTHO_03774	0	1	1	1	1	1	1	1	1
ORTHO_03780	0	1	1	1	1	1	1	1	1
ORTHO_03795	0	1	1	1	1	1	1	1	1
ORTHO_03814	0	1	1	1	1	1	1	1	1
ORTHO_03827	0	1	1	1	1	1	1	1	1
ORTHO_03861	0	1	1	1	1	1	1	1	1
ORTHO_03863	0	1	1	1	1	1	1	1	1
ORTHO_03869	0	1	1	1	1	1	1	1	1
ORTHO_03873	0	1	1	1	1	1	1	1	1
ORTHO_03879	0	1	1	1	1	1	1	1	1
ORTHO_03883	0	1	1	1	1	1	1	1	1
ORTHO_04093	0	1	1	1	0	1	1	1	1
ORTHO_04113	0	1	1	1	0	1	1	1	1
ORTHO_04131	0	1	1	1	0	1	1	1	1
ORTHO_04143	0	1	1	1	0	1	1	1	1
ORTHO_04272	0	1	1	1	0	1	1	1	1
ORTHO_04324	0	1	1	1	0	1	1	1	1
ORTHO_04409	0	1	1	1	0	1	1	1	1

ORTHO_04081	0	1	1	0	1	1	1	1	1
ORTHO_04112	0	1	1	0	1	1	1	1	1
ORTHO_04162	0	1	1	0	1	1	1	1	1
ORTHO_04186	0	1	1	0	1	1	1	1	1
ORTHO_04192	0	1	1	0	1	1	1	1	1
ORTHO_04250	0	1	1	0	1	1	1	1	1
ORTHO_04259	0	1	1	0	1	1	1	1	1
ORTHO_04297	0	1	1	0	1	1	1	1	1
ORTHO_04325	0	1	1	0	1	1	1	1	1
ORTHO_04673	0	1	1	0	0	1	1	1	1
ORTHO_04742	0	1	1	0	0	1	1	1	1
ORTHO_04756	0	1	1	0	0	1	1	1	1
ORTHO_04260	0	1	0	1	1	1	1	1	1
ORTHO_04431	0	1	0	1	1	1	1	1	1
ORTHO_04457	0	1	0	1	1	1	1	1	1
ORTHO_04847	0	1	0	1	0	1	1	1	1
ORTHO_04803	0	1	0	0	1	1	1	1	1
ORTHO_04176	0	0	1	1	1	1	1	1	1
ORTHO_04222	0	0	1	1	1	1	1	1	1
ORTHO_04228	0	0	1	1	1	1	1	1	1
ORTHO_04294	0	0	1	1	1	1	1	1	1
ORTHO_04320	0	0	1	1	1	1	1	1	1
ORTHO_04322	0	0	1	1	1	1	1	1	1
ORTHO_04580	0	0	1	1	0	1	1	1	1
ORTHO_04624	0	0	1	1	0	1	1	1	1
ORTHO_04737	0	0	1	0	1	1	1	1	1
ORTHO_05030	0	0	1	0	0	1	1	1	1
ORTHO_04608	0	0	0	1	1	1	1	1	1
ORTHO_04678	0	0	0	1	1	1	1	1	1
ORTHO_04751	0	0	0	1	1	1	1	1	1
ORTHO_04788	0	0	0	1	1	1	1	1	1
ORTHO_04801	0	0	0	1	1	1	1	1	1

ORTHO_04802	0	0	0	1	1	1	1	1	1
ORTHO_04806	0	0	0	1	1	1	1	1	1
ORTHO_04835	0	0	0	1	1	1	1	1	1
ORTHO_04836	0	0	0	1	1	1	1	1	1
ORTHO_05152	0	0	0	1	0	1	1	1	1
ORTHO_05174	0	0	0	1	0	1	1	1	1
ORTHO_05108	0	0	0	0	1	1	1	1	1
ORTHO_05162	0	0	0	0	1	1	1	1	1
ORTHO_05169	0	0	0	0	1	1	1	1	1
ORTHO_05400	0	0	0	0	0	1	1	1	1
ORTHO_05414	0	0	0	0	0	1	1	1	1
ORTHO_05441	0	0	0	0	0	1	1	1	1
ORTHO_05567	0	0	0	0	0	1	1	1	1
ORTHO_05583	0	0	0	0	0	1	1	1	1
ORTHO_05591	0	0	0	0	0	1	1	1	1
ORTHO_05592	0	0	0	0	0	1	1	1	1
ORTHO_05595	0	0	0	0	0	1	1	1	1
ORTHO_05598	0	0	0	0	0	1	1	1	1
ORTHO_05599	0	0	0	0	0	1	1	1	1
ORTHO_05610	0	0	0	0	0	1	1	1	1
ORTHO_05611	0	0	0	0	0	1	1	1	1
ORTHO_05614	0	0	0	0	0	1	1	1	1
