# Supplementary material for: Comparative Genomics Reveals the Core Gene Toolbox for the Fungus-Insect Symbiosis
Source: mBio. 2018 May 15;9(3):e00636-18. doi: 10.1128/mBio.00636-18 (PMC5954228; doi:10.1128/mBio.00636-18)
Supplement: TABLE S2 [file mbo003183874st2.pdf]

**Supplementary Table S2**. Genome wide comparisons among 18 insect-associated fungi.

| Strains | Host range | Genome size (Mb) | Predicted gene models | Signal peptide | Transmembrane helices | PHI genes | Source | NCBI accession |
|---|---|---|---|---|---|---|---|---|
| *Metarhizium robertsii* | Generalist: Insecta | 39.0 | 11,688 | 1,707 | 11,022 | 3,858 | Hu et al. 2014 (46) | ADNJ00000000/JGI |
| *Metarhizium acridum* | Specialist: Locust | 38.1 | 9,849 | 1,212 | 9,136 | 3,268 | Gao et al. 2011 (29) | ADNI00000000 |
| *Ophiocordyceps unilateralis* | Specialist: Formicidae | 26.1 | 7,821 | 1,209 | 6,456 | 2,411 | de Bekker et al. 2015 (26) | LAZP01000000 |
| *Ophiocordyceps sinensis* | Specialist: Hepialidae | 120 | 6,972 | 895 | 5,183 | 2,094 | Hu et al. 2013 (47) | ANOV00000000 |
| *Beauvaria bassiana* | Generalist: Insecta | 33.7 | 10,364 | 1,486 | 10,568 | 3,371 | Xiao et al. 2012 (27) | ADAH00000000 |
| *Cordyceps militaris* | Generalist: Insecta | 32.2 | 9,651 | 1,323 | 10,250 | 3,114 | Zheng et al. 2011 (45) | AEVU00000000 |
| *Conidiobolus coronatus* | Generalist: Insecta | 39.9 | 10,635 | 1,438 | 9,313 | 2,709 | Chang et al. 2015 (48) | JXYT00000000 |
| *Conidiobolus thromboides* | Generalist: Insecta | 24.6 | 8,867 | 1,103 | 7,786 | 2,474 | Arnesen et al. BioRxiv (50) | JGI |
| *Basidiobolus meristosporus* | Generalist: Insecta/Amphibia | 89.5 | 16,111 | 2,409 | 15,443 | 5,041 | Mondo et al. 2017 (49) | JGI |
| *Smittium culicis* ID-206-W2 | Generalist: Diptera | 71.0 | 10,024 | 753 | 5,035 | 1,965 | Wang et al. 2016 (38) | LSSM00000000 |
| *Smittium culicis* GSMNP | Generalist: Diptera | 77.1 | 11,209 | 952 | 6,200 | 2,313 | Wang et al. 2016 (38) | LSSN00000000 |
| *Smittium mucronatum* | Specialist: *Psectrocladius* | 102.4 | 8,712 | 700 | 4,550 | 1,725 | Wang et al. 2016 (38) | LSSL00000000 |
| *Furculomyces boomerangus* | Generalist: Diptera | 28.1 | 7,338 | 729 | 5,525 | 1,990 | Present study | MBFT00000000 |
| *Smittium angustum* | Generalist: Diptera | 28.1 | 7,821 | 716 | 5,232 | 2,018 | Present study | MBFU00000000 |
| *Smittium simulii* | Generalist: Diptera | 43.9 | 6,519 | 673 | 3,599 | 1,471 | Present study | MBFR00000000 |
| *Smittium megazygosporum* | Generalist: Diptera | 43.6 | 7,132 | 593 | 4,383 | 1,774 | Present study | MBFS00000000 |
| *Capniomyces stellatus* | Specialist: *Allocapnia* | 24.8 | 6,649 | 792 | 4,440 | 1,763 | Wang et al. 2016 (39) | LUVW00000000 |
| *Zancudomyces culisetae* | Generalist: Diptera | 28.7 | 7,387 | 693 | 4,565 | 1,905 | Wang et al. 2016 (38) | LSSK00000000 |
