# Supplementary material for: Comparative Genomics Reveals the Core Gene Toolbox for the Fungus-Insect Symbiosis
Source: mBio. 2018 May 15;9(3):e00636-18. doi: 10.1128/mBio.00636-18 (PMC5954228; doi:10.1128/mBio.00636-18)
Supplement: TABLE S3 [file mbo003183874st3.pdf]

Supplementary Table S3. Information of the Fungus-Insect Symbiotic Core Domains.

FISCoDomains	Information
Amidohydro_2	Amidohydrolases
AZUL	Amino-terminal Zinc-binding domain of ubiquitin ligase E3A
CDT1_C	DNA replication factor CDT1: The protein encoded by this gene is a key licensing factor which, along with the protein Cdc6, functions to license DNA by forming the pre-replication complex (pre-RC). Its activity during the cell cycle is tightly regulated by its association with the protein geminin, which both inhibits Cdt1 activity during S phase in order to prevent re-replication of DNA and prevents it from ubiquitination and subsequent proteolysis.
CGI-121	Kinase binding protein CGI-121
CTD_bind	RNA polymerase II-binding domain
MMtag	Kinase phosphorylation protein
NMO	Nitronate monooxygenase
NUDIX_2	Nucleotide hydrolase that hydrolyse a wide range of organic pyrophosphates, including nucleoside di- and triphosphates, di-nucleoside and diphospho-inositol polyphosphates, nucleotide sugars and RNA caps, with varying degrees of substrate specificity
PAF-AH_p_II	Platelet-activating factor acetylhydrolase, isoform II; responsible for inactivation of platelet-activating factor through cleavage of an acetyl group
Pept_tRNA_hydro	Peptidyl-tRNA hydrolase
Pinin_SDK_memA	pinin/SDK/memA/ protein conserved region, A common feature of the members of this family is that they may all participate in regulating protein-protein interactions.
RPT	A repeated domain in UCH-protein
RRM_2	RNA recognition motif 2
RXT2_N	RXT2 has been demonstrated to be involved in conjugation with cellular fusion (mating) and invasive growth
SBF	Sodium Bile acid symporter family
